# A repertoire of protease inhibitor families in *Amblyomma americanum* and other tick species: inter-species comparative analyses

**DOI:** 10.1186/s13071-017-2080-1

**Published:** 2017-03-22

**Authors:** Lindsay M. Porter, Željko M. Radulović, Albert Mulenga

**Affiliations:** Department of Veterinary Pathobiology, Texas A&M University College of Veterinary Medicine and Biomedical Sciences, 4647 TAMU, College Station, TX 77843 USA

**Keywords:** Protease inhibitors in ticks, *Amblyomma americanum*, Hard ticks

## Abstract

**Background:**

Protease inhibitors (PIs) are important regulators of physiology and represent anti-parasitic druggable and vaccine targets. We conducted bioinformatic analyses of genome and transcriptome data to determine the protease inhibitor (PI) repertoire in *Amblyomma americanum* and in 25 other ixodid tick species. For *A. americanum*, we compared the PI repertoires in fed and unfed, male and female *A. americanum* ticks. We also analyzed PI repertoires of female 48, 96 and 120 h-fed midgut (MG) and salivary gland (SG) tissues.

**Results:**

We found 1,595 putative non-redundant PI sequences across 26 ixodid tick species. Ticks express PIs from at least 18 different families: I1, I2, I4, I8, I21, I25, I29, I31, I32, I35, I39, I43, I51, I53, I63, I68, I72 and I74 (MEROPS)*.* The largest PI families were I2, I4 and I8 and lowest in I21, I31, I32, I35 and I68. The majority (75%) of tick PIs putatively inhibit serine proteases, with ~11 and 9% putatively regulating cysteine or metalloprotease-mediated pathways, respectively, and ~4% putatively regulating multiple/mixed protease types. In *A. americanum*, we found 370 PIs in female and 354 in male ticks. In *A. americanum* we found 231 and 442 in unfed and fed ticks, respectively. In females, we found 206 and 164 PIs in SG and MG, respectively. The majority of highly cross-tick species conserved PIs were in families I1, I2, I8, I21, I25, I29, I39 and I43.

**Conclusions:**

Ticks appear to express large and diverse repertoires of PIs that primarily target serine protease-mediated pathways. We speculate that PI families with the highest repertoires may contain functionally redundant members while those with the lowest repertoires are functionally non-redundant PIs. We found some highly conserved PIs in the latter category, which we propose as potential candidates for broad-spectrum anti-tick vaccine candidates or druggable targets in tick control.

**Electronic supplementary material:**

The online version of this article (doi:10.1186/s13071-017-2080-1) contains supplementary material, which is available to authorized users.

## Background

Ticks are a significant source of morbidity and mortality in both public health and veterinary medicine. Ticks may transmit a wide diversity of pathogenic organisms to their hosts including viruses, bacteria, protozoa and helminthes [1]. In the United States alone, there are at least 15 human diseases for which the associated pathogens are transmitted by ticks [2]. Between 1982 and 2001, the list of human diseases caused by tick-vectored pathogens grew substantially with the addition of 15 new bacterial pathogens [3]. Of veterinary concern, the major diseases caused by tick-transmitted pathogens are Lyme, babesiosis, theileriosis, heartwater, anaplasmosis, cytauxzoonosis and hepatozoonosis [4, 5]. In livestock production, an estimated 80% of the world’s cattle populations are affected by disease-causing tick-borne pathogens [6]. Aside from transmitting disease agents, ticks themselves can lead to a variety of indirect veterinary and medical morbidity including toxicosis, paralysis, anemia, wounds susceptible to bacterial or screwworm fly infections, damages to hides and even death [4]. Limitations to acaricide use are the resistance to these chemicals, environmental contamination, cost of development for new chemicals and also food contamination [7–11]. Immunization of hosts against tick feeding is a validated alternative to acaricide-based tick control [9, 12]. However, the major bottleneck to the development of anti-tick vaccines is the discovery of effective target antigens. Protease inhibitors are among some of the most attractive potential anti-tick vaccine antigens.

The balance between proteases and protease inhibitors is essential for the regulation of normal homeostasis across life: from microbes, to plants, to animals. Throughout taxa, essential pathways are regulated by proteases [13–18]. Left uncontrolled, however, protease activity can result in diseases such as cancer, emphysema and blood coagulation disorders [19–23]. Thus, to avoid aberrant protease activity, protease inhibitors tightly and precisely control protease activity. In parasites including ticks, protease inhibitor function is viewed from two perspectives: one being their significance in regulating the homeostasis of the tick itself, and the other being their significance in regulating the parasite-host interaction.

Ticks accomplish feeding by disrupting host tissue and then imbibing blood that bleeds into the wounded area. Tick feeding provokes the host’s protease-mediated defense pathways including inflammation, blood clotting and platelet aggregation. Thus, ticks were proposed to secrete protease inhibitors to evade these defense mechanisms, making them prime candidates for anti-tick vaccine studies [24, 25]. Prior to the advent of DNA technology, efforts were focused on purifying proteases and their inhibitors from crude tick protein extracts for medicinal applications [26–28]. However, many tick cDNAs encoding putative protease inhibitors have now been cloned from multiple tick species [24, 29–35]. Additionally, with the recent sequencing of the *I. scapularis* genome [36], and of several tick transcriptomes [37–49], the focus of tick protease inhibitor research can shift from discovery to characterization. Data mining of sequences from these studies, as available in public databases, has revealed that tick genomes, like many other organisms, encode for high numbers of both proteases and protease inhibitors. Although proteases and their inhibitors are attractive anti-tick vaccine antigens, high numbers of sequences in certain groups of PI families suggests possible functional redundancy. Redundant systems are a potential problem in that targeting one member may result in ticks switching to a functionally equivalent substitute. Therefore, a more detailed bioinformatic investigation into these sequences might reveal a better prioritization plan for vaccine candidate selection. To this end, the first step and the goal of this study was to organize and prioritize the protease inhibitors, into redundant and least or non-redundant systems.

In this study we used *Amblyomma americanum* in-house transcriptome data, as well as putative protease inhibitor sequences for ixodid tick species that have been deposited in the MEROPS [50], and/or GenBank databases to compile a reference of all reported putative protease inhibitors in ixodid ticks. We found and analyzed 1,595 non-redundant putative PI sequences across 26 ixodid tick species. In *A. americanum* we found evidence to support previous findings of a time-dependent PI expression in salivary glands [49, 51] coined as “sialome switch” [52], however our analyses provide evidence of time-dependent PI expression throughout tick tissues. Additionally, our global analysis differentiated tick PI families that are likely redundant or non-redundant, as well as PIs that are conserved across tick species and that may regulate pathways essential in all ticks. This study serves as the first step in prioritizing tick PIs as anti-tick vaccine antigen candidates.

## Methods

### Identification of putative tick protease inhibitors (PI)

Putative tick PI sequences were identified from two sources. A previously assembled *A. americanum* transcriptome (BioProject accession number PRJNA226980) was annotated by local batch blasting against the NCBI protein database and the Conserved Domain Database (CDD) using the CLC Genomics Workbench software vers 8.0.1 (Qiagen, Hilden, Germany) as previously described [53]. Tick PI sequences were also downloaded from the NCBI GenBank database using keyword searches of the database. For PIs not uniquely characterized by a specific domain, annotations were based on > 95% identity to annotated sequences in databases. PI sequences were also acquired from the MEROPS version 9.4 database (http://merops.sanger.ac.uk/) [50], and noted according to PI family.

Annotated putative PI sequences from each tick species were sorted according to family and compiled into a single file. All files were then subjected to multiple sequence alignments, against themselves, to identify redundant sequences in each file. An identity value of 95% or greater was deemed as evidence of redundancy, and only one sequence was retained for further analysis. In this way, we determined a non-redundant count for each PI family for each tick species. Sequences where family membership could not be verified on the basis of known domains or similarity to characterized members of the protease inhibitor family were also eliminated from the study.

### Identification of putative expression patterns and homologs in other ticks

To determine apparent expression patterns, putative *A. americanum* tick PI sequences were further screened for presence or absence in different transcriptomes: male and female, unfed and fed whole *A. americanum* ticks, as well as dissected salivary gland (SG) and midgut (MG) of 48, 96 and 120 h fed female ticks. Identification of highly conserved tick serine protease inhibitors (serpins) in family I4 was previously accomplished [53]. In this study, we determined PIs homologs for all other PI families by BLASTX screening of *A. americanum* PIs against other tick PIs of the same family. PI sequences in other tick species where identity values were ≥ 50% were included in further analyses.

## Results and discussion

### Tick genomes likely encode hundreds of protease inhibitors

**Fig. 1:**
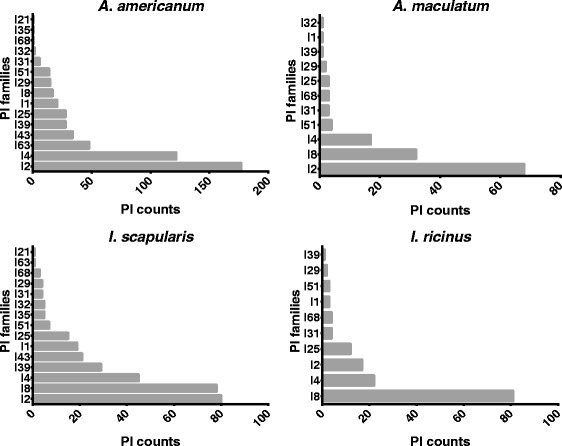
PI repertoire for four major medically important tick species. The count of protease inhibitors (PIs) in each PI family for *Amblyomma americanum*, *Amblyomma maculatum*, *Ixodes scapularis* and *Ixodes ricinus* are shown. PI families where total count was less than ten were excluded from the graphs

Additional file 1: Table S1 (please note the different tabs) lists a total of 1,595 putative non-redundant PI sequences across 26 ixodid tick species. Our analysis of our *A. americanum* transcriptome data revealed 515 putative PIs for this species (Table 1). We also found more than 100 PI sequences for three additional tick species: *Ixodes scapularis* (239), *Ixodes ricinus* (149), *Amblyomma maculatum* (135) and *Amblyomma cajennense* (106) (Fig. 1). Between ten and 100 sequences were found for eight tick species: *Amblyomma triste* (83), *Rhipicephalus pulchellus* (76), *Rhipicephalus microplus* (57), *Rhipicephalus appendiculatus* (35), *Amblyomma parvum* (31), *Haemaphysalis longicornis* (20), *Amblyomma variegatum* (20) and *Rhipicephalus sanguineus* (13) (Table 1). The remaining 13 tick species currently have less than ten reported non-redundant PI sequences: *Dermacentor variabilis* (8), *Dermacentor andersoni* (8), *Rhipicephalus haemaphysaloides* (7), *Ixodes pacificus* (7), *Ixodes persulcatus* (2), *Haemaphysalis bispinosa* (2), *Hyalomma anatolicum anatolicum* (2), and one each for *Amblyomma hebraeum*, *Hyalomma marginatum rufipes*, *Dermacentor silvarum*, *Ixodes ovatus*, *Rhipicephalus bursa* and *Rhipicephalus annulatus*. Since five well-studied tick species have more than 100 reported PI sequences, we predict that this will also be the case for other ixodid tick species, once further sequence data becomes available. Interestingly, we found that the large PI counts for *A. americanum*, *A. maculatum*, *A. cajennense*, *I. scapularis* and *I. ricinus* are consistent with current counts in other well-studied arthropods, such as *Anopheles gambiae* (131) *Apis mellifera* (224), *Bombyx mori* (269), *Culex quinquefasciatus* (99), *Drosophila melanogaster* (166) and *Tribolium castaneum* (184) [50]. We would like to advise the reader that data presented in this study need further validation using quantitative (q) RT-PCR. Despite this limitation, these data provide important information on protease inhibitors expressed in tick genomes.

**Table 1:** *Amblyomma americanum* protease inhibitor counts

Inhibitor family	Sex related^a^			Feeding related^b^			Tissue distribution^c^			Total
	F	M	F&M	FD	UF	FD&UF	SG	MG	SG&MG	
I1	2	8	11	8	1	12	3	2	6	21
I2	69	35	73	102	30	45	60	14	27	177
I4^d^	33	57	31	87	12	21	12	10	26	122
I8	6	2	9	6	2	9	5	0	5	17
I21	0	0	1	0	0	1	0	0	1	1
I25	6	10	12	17	0	11	3	3	9	28
I29	7	7	1	12	1	2	1	4	1	15
I31	2	0	4	3	1	2	0	1	4	6
I32	0	0	2	0	0	2	0	0	2	2
I35	1	0	0	0	0	1	0	0	1	1
I39	13	2	13	14	4	10	8	5	9	28
I43	17	10	7	14	11	9	8	7	3	34
I51	0	9	5	9	0	5	1	0	4	14
I63	8	8	32	9	9	30	0	12	7	48
I68^e^	1	–	–	–	–	–	1	–	–	1
Total	164	148	202	282	71	160	101	59	105	515

^a^Count of protease inhibitors found present in female (F) or male (M) ticks, and number found present in both females and males (F&M)

^b^Count of protease inhibitors found present in fed (FD) or unfed (UF) ticks, and number found present in both fed and unfed (FD&UF)

^c^Count of protease inhibitors found present in salivary glands (SG) or midgut (MG), and number found present in both of these tissues (SG&MG)

^d^Data reported here are from our previously published analysis of family I4 [53]

^e^Data reported here are from [52] which analyzed female salivary gland data only

### *Amblyomma americanum* PI repertories may vary by sex, tissue and feeding time point

**Fig. 2:**
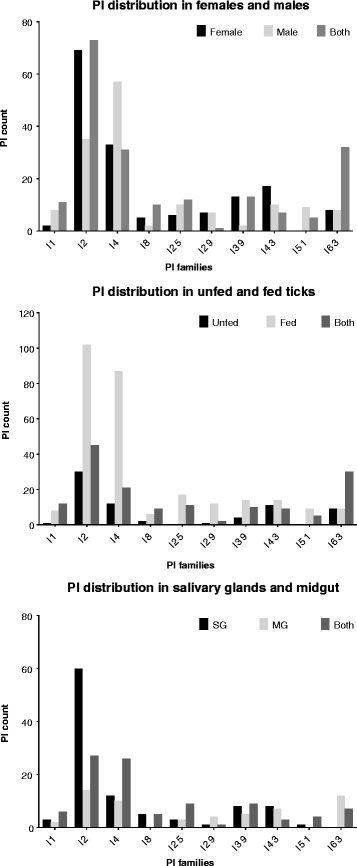
*Amblyomma americanum* PI repertoire according to sex, unfed/fed and SG/MG tissues. The count of protease inhibitors (PIs) for each PI family that were found exclusively in males, exclusively in females or in both; found exclusively in unfed ticks, exclusively in fed ticks or found in both; and found exclusively in salivary glands (SG), exclusively in midgut (MG) or found in both are shown. PI families where total count was less than 10 were excluded from the graphs

Evidence in other *in silico* transcriptome analyses show evidence of sex-based differences in transcript levels during feeding in *R. appendiculatus* [51], and temporal and sex-based differences in transcript repertoires across feeding time points in *R. pulchellus* [49]. Additionally, studies using qRT-PCR analysis have validated spatio-temporal variance of PI expression in ticks [54–56] as well as semi-quantitative RT-PCR analyses showing spatio-temporal differences in PI expression patterns [57–59]. Although our study is entirely *in silico*, our data support the hypothesis that at least some tick PIs are differentially expressed in tissues, time points and possibly between the sexes. In our transcriptome data we found that of 515 *A. americanum* PI sequences, 164 (~32%) and 148 (~29%) transcripts were found exclusively in females and males, respectively, while 202 (~39%) were found in both sexes (Table 1, Fig. 2). The next step will be to further explore the hypothesis that *A. americanum* ticks express sex-specific PIs using qRT-PCR.

**Fig. 3:**
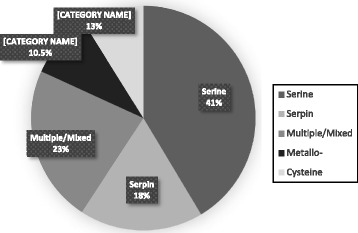
Proportions of tick protease inhibitors (PIs) which are predicted inhibit each protease catalytic type. Tick PIs with putative activity against serine or multiple/mixed activity represent the vast majority of tick PIs. Serpins are shown separately from other PI families showing activity against multiple catalytic types to illustrate that most tick PIs likely do inhibit proteases of the serine catalytic type

In *A. americanum* SG and MG tissues at 48, 96 and 120 h of feeding, we found a total of 265 PIs across 14 families (Table 1, Figs. 2, 3). It is interesting to note that we found more PI transcripts in SG (205) than in MG (162). Also interesting is that of the 515 total *A. americanum* PIs, we could not find 250 in either SG or MG tissues. While technical errors could explain the lack of PIs in these tissues, the data suggests the possibility that at least some PIs are not expressed in these tissues, and that they might be expressed in other tissues that are not primarily exposed to the host components. Although data here need further validation using qRT-PCR, the 250 sequences not found in both SG and MG may not be priority candidates for anti-tick vaccine development. Data for individual families are outlined in the following sections. We would like to note that data here are qualitative and not quantitative; therefore, it is possible that distribution patterns for *A. americanum* PIs may change after further analyses.

### Majority of tick PIs putatively inhibit serine protease-mediated pathways

Figure 3 shows that the majority (~72.5%, 1,159/1,595) of tick PI sequences in this study belong to families inhibiting serine proteases and are therefore putative inhibitors of serine proteases: 527 in I2 (Kunitz-like serine protease inhibitors), 287 in I4 (serine protease inhibitors, [serpin]), 237 in I8 (TIL domain elastase inhibitors), 61 in I1 (Kazal), 31 in I51 (PEBP, phosphatidylethanolamine-binding protein), 10 in I53 (madanin), three in family I21 (secretogranin), two in I72 (chimadanin) and one in I74 (variegin, thrombin inhibitor) (Additional file 2: Table S2 and Fig. 1). Of the remaining 436 PI sequences, 164 (~13%) belong to families inhibiting cysteine proteases: 102 in I25 (cystatin), 53 in I31 (thyropin), 39 in I29 (CTLA, Cytotoxic T-lymphocyte antigens) and 12 in I32 (survivin); and 134 (~10.5%) belong to families of metalloprotease inhibitors: 75 in I43 (oprins), 62 in I63 (pro-eosinophil major basic protein), 24 in I68 (tick carboxypeptidase inhibitor) and 6 in I35 (tissue inhibitor of metalloproteinases). The remaining 63 PIs (~4%) are in family I39, alpha-2-macroglobulins, which are non-specific PIs.

The relative PI family distribution we observed at the global level was mirrored at the individual tick species level with most PIs belonging to families inhibiting serine proteases. We would like to note that some tick serpins may inhibit both cysteine and serine proteases, which would change the relative proportions of proteases inhibited ([60]; unpublished observations by the authors). Nevertheless, such a high number of putative inhibitors of serine proteases suggest that inhibitors of serine proteases play an important and extensive role in regulating tick physiology.

In combination with our observations that ticks express high numbers of PIs for some families, these data suggest a potential for highly functionally redundant systems, whereby several proteins can accomplish the same physiological goal. Evidence of functional redundancy could signal that those PIs regulate tick physiological pathways that must be functional without fail, and thus finding ways to block such PIs could prove to be highly effective in tick control. On the other hand, PI families with fewer sequences could be interpreted as less likely to have extensive functional redundancy and these proteins could be the most attractive targets for anti-tick vaccine development.

### Families of PIs encoded in *A. americanum* and other tick species

#### Family I1: Kazal

In *A. americanum* we found 21 non-redundant Kazal protease inhibitor (KPI) sequences (Table 1)*.* In our transcriptome analysis we found the majority of these (19/21) in male ticks, while we detected a little more than half (13/21) in female ticks (Table 1). It is interesting to note that eight KPI transcripts were found only in males, therefore future experiments should aim to validate whether or not these PIs are differentially expressed in the sexes. Strikingly, in our data we found almost all KPIs (20/21) in fed ticks, strongly supporting the hypothesis that many KPIs function in feeding physiology.

In 11 other ixodid tick species we found 33 putatively non-redundant KPI sequences (Additional file 1: Table S1): 24 for *I. scapularis*, four for *I. ricinus*, three for *A. variegatum*, two for *A. cajennense* and only one KPI sequence for each of *A. maculatum*, *A. triste*, *A. parvum*, *R. microplus*, *R. sanguineus*, *R. pulchellus* and *H. longicornis*. Our interspecific BLAST analyses showed 13 *A. americanum* KPIs to be highly conserved in other ixodid tick species, primarily *I. scapularis* (Additional file 2: Table S2). Amino acid identities exceeded 65% for six *A. americanum* Kazal PIs with four of these ranging between 81 and 98%. Interestingly, we found these four very highly conserved PIs to be expressed either in all or almost all tissues at all of the time points examined in this study (Additional file 2: Table S2). Six of the seven less-conserved KPIs (identities of 50–66%) had a more limited tissue/time point/sex distribution. Notably, three of these six were found only in fed males.

The only functional data currently available for tick KPIs is from *H. longicornis* (ABB76182.1) and is a follistatin-related protein (FRP, homologous to human FRP), binding activin A and BMP-2 [61]. Activin has been implicated in cell growth and differentiation, wound repair and acute inflammation in the innate immune response [62, 63]. We found one ABB76182.1 homolog in *A. americanum* with 84% identity. Several inhibitory KPIs from other hematophagous organisms have been described. Bdellins from the medicinal leech *Hirudo medicinalis* are Kazal-type inhibitors of trypsin and plasmin [64]. Additionally, three Kazal-type thrombin inhibitors from hematophagous triatoma bugs have been described: dipetalogastin [65], infestin [66] and rhodniin, [67]. In ticks, several proteins containing Kazal domains have been reported including an organic anion transporting polypeptide (Oatp), from *A. americanum* [68] and from *I. scapularis* [69], as well as insulin growth factor binding proteins from *R. appendiculatus*, *R. microplus* and *A. variegatum* [47]. Inhibitory properties for tick KPIs however have not been reported. Given the important role KPIs play in facilitating hematophagy in other organisms, it will be interesting to investigate if tick KPIs function the same.

#### Family I2: Kunitz

In *A. americanum* we found 177 non-redundant Kunitz sequences. In our transcriptome data we found many Kunitz sequences only in female ticks (69) and many only in male ticks (35), while 73 were confirmed to be expressed in both males and females, again suggesting there may be differential expression of this PI family between the sexes. Also noteworthy is the 60 Kunitz PIs found only in SG suggesting there may be at least some Kunitz PIs in *A. americanum* that are only expressed in SG. The 87 Kunitz sequences found in SG shows the complexity of tick salivary gland physiology. Notably, similar numbers of Kunitz sequences were found only in unfed females (30) as were found common to both fed and unfed females (45), while more than double those numbers were found only in fed female ticks (102). These preliminary data support a hypothesis that at least some Kunitz sequences may be expressed only in fed ticks and this hypothesis should be confirmed with qRT-PCR analysis. Such a marked diversity of Kunitz sequences during feeding demonstrates their importance in tick feeding physiology. In 17 other ixodid tick species we found 350 putatively non-redundant Kunitz sequences (Additional file 1: Table S1): 117 for *I. scapularis*, 72 for *A. maculatum*, 36 for *A. triste*, 32 for *A. cajennense*, 22 for *R. pulchellus*, 17 for *I. ricinus*, nine for *R. microplus*, seven each for *I. pacificus*, *A. variegatum* and *A. parvum*, six each for *H. longicornis* and *R. sanguineus*, four for *D. andersoni*, three for *D. variabilis*, two each for *R. appendiculatus* and *R. haemaphysaloides*, and one for *A. hebraeum*. Our BLASTX analyses revealed 12 *A. americanum* Kunitz PIs homologs in other tick species with identities at ≥ 60% (Additional file 2: Table S2). Interestingly, all except one of these 12 were found in fed ticks, supporting the hypothesis that conserved *A. americanum* PIs play an important role during feeding. However, it is worth mentioning that four of 12 were confirmed by our transcriptome data to be expressed in all or almost all tissues and time points examined in this study.

Kunitz is a large family found in ticks and other hematophagous species [50, 70–73]. Accordingly, we found that 18 of 26 tick species in this study had Kunitz sequences deposited in GenBank (Additional file 1: Table S1). Kunitz proteins are serine protease inhibitors of the S1 protease family [50]. Two Kunitz inhibitors from *I. scapularis* have been functionally characterized: Ixolaris (AAK83022) [74] and penthalaris (AAM93636) [75]. Ixolaris and penthalaris are tissue factor pathway inhibitors, inhibiting blood clotting *via* FVIIa/TF binding [74, 75]. Many Kunitz-type inhibitors from other tick species have also been described, and are now known to inhibit a range of proteases including thrombin, trypsin, plasmin, kallikrein, neutrophil elastase and FXa [70]. Some well-described examples of tick Kunitz inhibitors include the thrombin-induced platelet aggregation inhibitors savignygrin (*Ornithodoros savignyi*, AAM54048), [76], disagregin (*O. moubata*, AAB30092), [77, 78] and the thrombin inhibitors savignin (*O. savignyi*, AAL37210), [79], boophilin (*R. microplus*, CAC82583), [80], amblin (*Amblyomma hebraeum*, AAR97367), [81] and hemalin (*H. longicornis*, BAH02683), [82]. Additionally, a trypsin-inhibiting Kunitz protein from *D. andersoni* shows bacteriostatic properties and is upregulated during infection with *Rickettsia* [83] demonstrating a role for Kunitz inhibitors aside from facilitating hematophagy. The widespread nature of this family across tick species and its apparent role in feeding and defense indicate the likelihood that all ticks express Kunitz inhibitors. However, the large number of Kunitz inhibitors suggests that this family is likely to contain functionally redundant members.

#### 3.12 Family I4: Serpins

A previous thorough analysis of serine protease inhibitors (serpins) in *A. americanum* shows 122 unique serpin sequences for this species [53]. In ten other tick species we found 165 putatively non-redundant serpin sequences: *I. scapularis* (45), *R. appendiculatus* (29), *R. microplus* (27), *I. ricinus* (22), *A. maculatum* (17), *R. pulchellus* (16), *A. variegatum* (4), *R. haemaphysaloides* (2), *I. persulcatus* (2) and *H. longicornis* (1) (Additional file 1: Table S1). Serpins were originally identified as inhibitors of serine proteases [84]. While other PIs inhibit serine proteases, this family is differentiated from other PIs by the RCL of the serpin domain. Further functional studies show certain serpins have cross-class inhibitory functions against cysteine proteases [85–87]. For additional reading, we refer the reader to Porter et al. [53].

#### Family I8: Chymotrypsin/Elastase Inhibitor

In *A. americanum* we found 17 non-redundant I8 inhibitor sequences. Our transcriptome data confirmed the expression of more than half of these (nine of 17) in both male and female ticks, while six were found only in females and two were found only in males. Our data also confirmed the expression of more than half (nine of 17) of I8 PIs in both fed and unfed ticks, while six were found only in fed ticks and two only in unfed ticks. We also found 10 sequences in SG, five of which were only in SG while the other five were also in MG. Based on these data it will be interesting to explore the hypothesis of differential I8 PI expression using qRT-PCR data (Fig. 2, Table 1).

In 10 other ixodid tick species we found 220 non-redundant family I8 sequences (Additional file 1: Table S1): 81 in *I. ricinus*, 78 in *I. scapularis*, 43 in *A. cajennense*, 35 in *A. triste*, 32 in *A. maculatum*, 21 in *A. parvum*, 5 in *R. microplus*, 1 in *H. marginatum rufipes*, 1 in *R. pulchellus* and 1 in *A. variegatum* (Additional file 1: Table S1). Based on the currently available data, family I8 appears to make up a much larger proportion of the *Ixodes* PI repertoire than the *Amblyomma* repertoire (Fig. 1). In *A. americanum* we found seven I8 sequences conserved in other tick species, with amino acid identities ranging from 50 to 72% (Additional file 2: Table S2). Of note is the *A. americanum* I8 sequence with 72% identity to *R. microplus* ixodidin, which is an antimicrobial peptide found in tick hemocytes [88].

There are currently few functional studies for family I8 members in ticks, however in *R. microplus*, inhibitory properties have been verified for some I8 members. These include ixodidin, a single TIL domain chymotrypsin/elastase inhibitor with antimicrobial properties [88], and BmSI-7, another single TIL domain protein shown to inhibit the bacterial protease subtisilin A and the fungal protease Pr1 [89]. Also, a recent study reported a TIL domain protein from *I. scapularis* nymphs as being upregulated and secreted at 48 h post-attachment [90]. Additionally, an I8 family member from *I. ricinus*, also upregulated after feeding, shows similarity to von Willebrand Factor [91] and thus may play a role in platelet aggregation inhibition [92]. In the *A. maculatum* sialotranscriptome 85 CDS (57 complete) that contain TIL domains (or without a TIL domain but having similarity to such sequences) were reported [45] however, redundancy analysis data were not provided.

#### Family I21: Secretogranin

In *A. americanum* we found only one non-redundant I21 sequence. Strikingly, our transcriptome data confirmed the expression of this sequence in every tissue and time point, with the exception of SG at 48 and 120 h. Since this sequence is expressed in males, females, unfed ticks and fed ticks in multiple tissues, we speculate that this protein may serve an important role. We also found only one non-redundant I21 sequence in *I. scapularis* and *D. variabilis* (Additional file 1: Table S1). Interestingly, the *A. americanum* sequence shows 92% identity to the *D. variabilis* sequence (ACJ12615.1), and 69% identity to the *I. scapularis* sequence (Additional file 2: Table S2). The *I. scapularis* and *D. variabilis* sequences share 75% identity.

Family I21 members are inhibitors of serine endopeptidases [50] and have a secretogranin_V domain (GenBank). The type sequence for this family is a neuroendocrine protein from humans, named 7B2, which inhibits the prohormone convertase PC2 [93]. Accordingly, the I21 sequence in *D. variabilis* was found in the synganglion (NCBI), and bioinformatic annotations of these sequences are as neuroendocrine proteins. However, functional studies of tick secretogranins have not yet been reported. The apparent tissue and time point ubiquity and lack of family expansion for *A. americanum* secretogranins makes this family very intriguing, particularly since the pattern of a single secretogranin sequence seems to extend to other tick species. Single- or low-member PI families are interesting from the perspective of their being targeted for control strategies, since it indicates a potential lack of functional redundancy for these proteins.

#### Family I25: Cystatins

In *A. americanum* we found 28 non-redundant cystatin sequences*.* Our transcriptome data confirmed the expression of 15 cystatins in SG and MG (Table 1). We did not find transcripts for all 28 cystatins in all tissues and time points or in both sexes. We found only 18 in females and only 22 in males. It will be interesting to use qRT-PCR data to explore the possibility of differential expression of cystatins in *A. americanum*. It is interesting to note however, that all cystatins we found to be expressed in unfed male and in unfed female ticks were also found in fed ticks. These data support the hypothesis that ticks likely do not express any cystatins only in the unfed stage.

In 12 other ixodid tick species we found 74 cystatins: *I. scapularis* (15), *I. ricinus* (14), *A. cajennense* (12), *R. pulchellus* (9), *R. microplus* (7), *H. longicornis* (5), *A. maculatum* (3), *R. sanguineus* (3), *D. variabilis* (2), and one each for *A. variegatum*, *R. haemaphysaloides*, *I. ovatus* and *D. silvarium* (Additional file 1: Table S1). Inter-species BLASTX analyses showed eight *A. americanum* cystatins have homologs across multiple tick species with amino acid identities ranging from 50 to 82% (Additional file 2: Table S2). This makes them interesting targets for anti-tick vaccines that would be capable of protecting against many species of ticks.

Cystatins are papain-like cysteine protease inhibitors [50]. Functional data show that silencing of *A. americanum* cystatin RNA results in reduced tick engorgement weights and failure to feed [94]. In *H. longicornis*, the cystatin Hlcyst-2 is expressed highest in MG and hemocytes, shows increased expression at feeding, and has been implicated in tick immunity due to inhibitory effects on the growth of *Babesia bovis* [95]. In *I. scapularis*, the cystatin sialostatin-1 is a cathepsin L inhibitor involved in anti-inflammation and inhibition of cytotoxic T-lymphocyte proliferation [96].

#### Family I29: Cytotoxic T-Lymphocyte Antigen (CTLA)

Family I29 is also known as cytotoxic T-lymphocyte antigen-2 alpha, (CTLA-2). In *A. americanum* we found 15 non-redundant CTLA sequences (Table 1). Interestingly, eight CTLAs in males were not found in females and eight in females were not found in males. Additionally, 14 of 15 CTLAs were found in fed ticks, with only two of those 14 found in unfed ticks. Similarly, we found only six of 15 CTLA sequences in SG and MG tissues. Taken together, these data suggest the potential of differential PI expression across tissues and time points that should be further investigated. Most striking in our transcriptome data was that unlike most other PI families, we found almost all CTLA sequences in only a single tissue and time point. While this could be explained by a technical rather than biological reason, it is nonetheless worth noting and worth following up with qRT-PCR expression analysis.

In 12 other ixodid tick species we found 24 putatively non-redundant CTLA sequences: four each for *I. scapularis* and *R. appendiculatus*, two each for *I. ricinus*, *R. haemaphysaloides*, *A. maculatum*, *A. variegatum*, *D. variabilis* and *H. anatolicum anatolicum*, and one each for *R. microplus*, *R. annulatus*, *R. pulchellus* and *H. longicornis* (Additional file 1: Table S1). Inter-specific BLAST analyses revealed eight *A. americanum* CTLAs with homologs in other tick species (Additional file 2: Table S2). CTLAs are unique cysteine proteases inhibitors, where inhibition of the peptidase is via the peptidase propeptide [50]. Therefore, these proteins have a domain profile that includes the I29 inhibitor domain, followed by a C1A peptidase domain. Strikingly, we found a high number of tick CTLAs with 60% or more identity to *A. americanum* CTLAs, and in every case, the region conserved between sequences included both the I29 inhibitor domain and the protease domain regions. The MEROPS database reports that the *I. scapularis* genome lacks family I29 homologs, however, we found four non-redundant *I. scapularis* sequences showing I29 inhibitor domains in the NCBI database (Table 1). While two of the most conserved *A. americanum* CTLAs were not confirmed in our data to be expressed in MG tissues, their homologs in other ticks (AAO60045, −46, −48, ACF35530 and XP_002403652) are characterized as midgut cysteine proteinases. Further investigation is required to determine whether or not *A. americanum* expresses these CTLAs in MG.

In mice, *Drosophila* and *Bombyx mori* CTLAs are inhibitors of cathepsin-L cysteine proteases [97–99]. Our BLAST analyses revealed that one *A. americanum* CTLA and 12 CTLAs from 10 other tick species show 40–62% identity (Additional file 2: Table S2) to the I29 inhibitor and cathepsin L protease regions of a CTLA in *Sarcophaga peregrina* (flesh fly, BAA76272) involved in clearing foreign proteins in this insect [100]. The role of CTLAs in tick physiology remains unknown, however it will be interesting to investigate the potential for tick CTLAs to be involved in eliminating the excess proteins in blood meals or unwanted proteins expressed by tick-borne disease agents.

#### Family I31: Thyropins

Family I31 is known as equistatin inhibitory unit 1 and members are called thyropins due to their thyropin (TY) domains [50, 101]. Thyropins are inhibitors of papain-like cysteine proteases and cathepsin-D [50, 101, 102]. In *A. americanum* we found six non-redundant thyropin sequences (Table 1). Unlike most of the other 13 families we analyzed in this study, our data showed that transcripts expressed in males were also expressed in females. Similarly, our data showed that transcripts expressed in SG were also expressed in MG, with the exception of one transcript that was found in neither tissue. Since our data do not support an hypothesis that thyropins play a role in SG-specific physiology, these PIs may not be recommendable as anti-tick vaccine candidates.

In nine other ixodid tick species we found 47 putatively non-redundant thyropins: 16 for *A. cajennense*, seven for *A. triste*, six for *I. ricinus*, five for *A. maculatum*, four each for *I. scapularis* and *R. pulchellus*, three for *R. microplus*, and one each for *A. variegatum* and *R. sanguineus* in public databases (Additional file 1: Table S1). Our BLASTX searches show four of six *A. americanum* thyropin have homologs in other tick species (Additional file 2: Table S2). One interesting observation is that the two most highly conserved *A. americanum* thyropins, homologs of an *A. variegatum* thyropin (DAA34697) at 73 and 85% identity values, were found only in fed males and in SG and MG of female ticks. It will be interesting to further investigate with qRT-PCR if these thyropins are only expressed in SG and MG tissues in males and females.

Thyropin domains are found in many types of proteins including in saxiphilin, which binds the neurotoxin saxitoxin [103], in testican, which is a cathepsin-L inhibitor and regulates some matrix metalloproteases (MMPs) [104] and in nidogen which functions in basement membrane formation [105] and neutrophil chemotaxis [106]. The functional role of thyropins in ticks is not yet known.

#### Family I32: Inhibitor apoptosis (IAP)

In *A. americanum*, we found two non-redundant IAP sequences (Table 1). Interestingly, our transcriptome data confirm expression of both IAPs in fed and unfed male and female ticks, and in both SG and MG tissues. The wide distribution of these IAPs implies their importance in *A. americanum* physiology. In five other ixodid tick species we found 10 non-redundant IAPs: five for *I. scapularis*; two for *A. triste*; and one each for *A. maculatum*, *R. microplus* and *R. pulchellus* (Additional file 1: Table S1).

IAPs are also referred to as BIRC or BIR (baculovirus inhibitor repeat containing) proteins. Since there are many sequence annotations in GenBank that include the terms “apoptosis inhibitor,” but do not have BIR domains and are not protease inhibitors, we counted only sequences showing a BIR domain in CDD searches. Our two *A. americanum* IAPs each had a homolog in another tick species (Additional file 2: Table S2). Notably, one sequence showed 70% identity to *R. microplus* AIT40207 while the other sequence had 54% identity to *I. scapularis* survivin (XP_002413809.1). IAPs inhibit caspases and cysteine endopeptidases [50, 107]. According to CDD search analyses in this study, some tick IAPs also have a C-terminus really interesting new gene (RING) domain also found in other IAP proteins [108, 109]. It has been shown that RING domains in IAPs allow them to ubiquinate caspases for degradation and removal [102]. Based on our analysis, tick IAPs with RING domains are found in *I. scapularis*, *A. triste*, *R. microplus* and *R. pulchellus*. However, the function of IAPs in tick biology remains to be investigated.

#### Family I35: Tissue inhibitor of metalloproteinases (TIMPs)

Family I35 is referred to as TIMP (tissue inhibitor of metalloproteinases) [50]. In *A. americanum* we found only one TIMP sequence (Table 1). In our transcriptome data we found just four redundant TIMP contigs, all of which were found only in females in our transcriptome data. The *A. americanum* TIMP was found in both SG and MG, and in both fed and unfed females. It will be interesting to confirm the lack of TIMP expression in male ticks using qRT-PCR data. We found only five TIMPs for other ixodid tick species, all in *I. scapularis* (Additional file 1: Table S1). BLASTX analyses show one of these three sequences to have 68% to the *A. americanum* TIMP (Additional file 2: Table S2). These data suggest ticks have a small TIMP inhibitor repertoire that may not be present at all in some species.

TIMPs are metalloendopeptidase inhibitors [50, 110]. In humans TIMPs are inhibitors of matrixins, which function in tissue remodeling, inflammation and cancer pathogenesis [111, 112]. Additionally, TIMPs are apoptosis and angiogenesis regulators [113]. From the perspective of tick feeding, which occurs over a period of several days, blocking tissue remodeling and repair is essential to maintain the feeding site. Therefore, tick TIMPs as wound-repair inhibitors is an interesting avenue for future research. Furthermore, since our analyses show ticks appear to have few TIMP sequences, these PIs might be involved in low-redundancy or no-redundancy pathways. However, the role of TIMPs in the biology of ticks or of any hematophagous organism remains to be explored.

#### Family I39: Alpha2-macroglobulins, (α-2 M)

In *A. americanum* we found 28 non-redundant alpha-2macroglobulins (*α-*2 M) sequences (Table 1)*.* Our transcriptome data confirmed the expression of the majority of these 28 (26/28) in female ticks, while we found only a little more than half (15/28) in male ticks. Similarly, in SG and MG tissues we found 18 of 28 *α-*2Ms, and confirmed expression of nine in both tissues. As our data support that these inhibitors are expressed in many different tick tissues, this is an indication that they may serve diverse functions in tick physiology.

In seven other ixodid tick species we found 35 *α-*2Ms: *I. scapularis* (17), *R. pulchellus* (11), *R. microplus* (3), and one each for *I. ricinus*, *A. maculatum*, *A. triste* and *A. parvum* (Additional file 1: Table S1, Fig. 2). Our interspecific BLAST analyses showed four *A. americanum α-*2Ms are highly conserved in *I. scapularis* (Additional file 2: Table S2), with identities ranging from 76 to 83%. These PIs appear to be widely distributed in males and females in many tissue and time points which could indicate they may not be involved in inhibiting tick host proteases. Notably however, three of these four were not found in MG tissues. Interestingly, we found one *A. americanum α-*2 M with 68% identity to an *α-*2 M in an argasid tick (*Ornithodorous moubata*, AAN10129). Such high identity values of metastriate *α-*2Ms with prostriate and argasid tick *α-*2Ms indicates that at least some *α-*2Ms likely control fundamental pathways in tick physiology. Accordingly, these PIs warrant further consideration as potential anti-tick vaccine or druggable targets. Most *A. americanum α-*2Ms however, did not show identity with any other tick *α-*2 M, suggesting significant divergence for some members of this family.

In both vertebrates and invertebrates, *α-*2Ms are considered components of the innate immune system involved in clearance of rogue endogenous and exogenous proteases [114, 115]. Emerging functional data show this is also the case in ticks. Indeed, one *α*2M in *D. variabilis* (BQ426156) is differentially upregulated when this species is infected with *Rickettsia montana* [116]. In other studies, RNAi-mediated silencing of *I. ricinus α*2M mRNA shows reduced phagocytosis of pathogens by tick cells, validating the significance of this protein family in the tick immune response [117]. The *α*2M-like protein from the ixodid tick *I. ricinus* has been characterized as functioning in pathogen phagocytosis by hemocytes in the hemolymph [117]. Similarly, the *α*2M TAM (tick *α*-macroglobulin) from *Ornithodoros moubata* is a trypsin inhibitor expressed by tick hemocytes and SG tissues [118, 119]. Collectively, this evidence suggests we may expect to find *α*2Ms in all tick species.

#### Family I43: Opossum proteinase inhibitor (oprin)

In *A. americanum* we found 34 non-redundant oprin sequences (Table 1). Of these 34, our transcriptome data confirm the expression of 11 in SG and 10 in MG tissues and nine found in both tissues, while almost half were not detected in these tissues. It will be interesting to use qRT-PCR methods to validate the lack of expression of some oprin sequences in *A. americanum* SG and MG tissues. We also found 20 oprin sequences in unfed and 23 in fed ticks. These data imply that oprins serve diverse functional roles in tick physiology. Interestingly, we found seven sequences present only in SG and five only in MG, but that were not in males or in whole tick females. Based on these data it will be interesting to explore in future studies the hypothesis that these seven oprins are exclusively expressed in female SG or MG. As with CTLA sequences, we detected most oprin sequences in only one tissue at single feeding time point, which will be interesting to validated using qPCR analysis.

In other ixodid ticks we found 41 putatively non-redundant oprins and only in *I. scapularis*. While the MEROPS database indicates 22 oprins for *I. scapularis* (Additional file 1: Table S1), we found two sequences (MEROPS ID: MER218533 and MER218534) to be identical; therefore, MER218534 was eliminated from further analysis. Another MEROPS entry (MEROPS ID: MER160224) is concurrently listed in both the Kazal and oprin families. The domain profile of this protein contains a single Kazal domain and two Ig domain regions, and therefore may belong to two inhibitor families, or may be a mis-annotation. Inter-specific BLASTX searches of *A. americanum* oprin sequences revealed six homologs in *I. scapularis* with > 70% identity and eight with 50–67% identity (Additional file 2: Table S2). We found most of these highly conserved *A. americanum* oprins only in limited tissues and time points. For example, the top three conserved oprins at 83, 82 and 80% identity with *I. scapularis* sequences were found only in MG at 48- and 120 h, only in SG at 48 h, and only in unfed males, respectively, however, these data should be further validated using qRT-PCR analysis.

Oprins are inhibitors of metalloendopeptidases and have varying numbers of immunoglobulin domains [120], but no defining domain profile. While several oprins function as snake venom metalloprotease inhibitors [120, 121], one oprin has been characterized as an immunoglobulin alpha FC receptor (FCalphaRI), which is the IgA receptor found on myeloid cells [122]. In this study, BLASTP scanning of tick sequences using the oprin type-entry from *D. virginiana* (MEROPS ID: MER019033), the alpha1B glycoprotein entry from humans (MEROPS ID: MER018491) and the Ig alpha FC receptor from humans (MEROPS ID: MER033169), revealed no tick sequences of significant similarity, indicating I43 proteins may serve different functional roles in ticks.

#### Family I51: Phosphatidylethanolamine-binding proteins (PEBPs)

In *A. americanum* we found 14 non-redundant phosphatidylethanolamine-binding protein (PEBP) sequences (Table 1). Our transcriptome data confirm expression of all 14 PEBPs in males, however nine were not found in females. The five PEBPs found in female ticks were also found in male ticks, which will be interesting to validate using qRT-PCR data since this would indicate females do not express any unique PEBPs. Since this is one of the smaller PI families, which could indicate low or no functional redundancy of PEBPs in ticks, it will be interesting to further investigate the function of the five PEPBs found in SG and MG tissues in this study.

In six other ixodid tick species we found 17 non-redundant PEBPs: seven for *I. scapularis*, four for *A. maculatum*, three for *I. ricinus*, and one each for *A. triste*, *A. parvum* and *A. cajennense* (Additional file 1: Table S1). Seven CDS have been reported in *A. maculatum* [45], and six can be found in GenBank, however only four of those six are non-redundant, therefore an exact count for *A. maculatum* remains to be determined. Data are less often collected for male ticks than for female ticks and this might explain why the number of known PEBPs in other tick species is less than in *A. americanum*. We found homologs to only three of 14 *A. americium* PEBPs in other ticks and only in *I. scapularis* (Additional file 2: Table S2). However, the identities for these sequences ranged from 56 to 69%. Interestingly, two of these three PEBPs were confirmed by our transcriptome data to be expressed in all tissues and feeding time points. Given the evolutionary distance between *Amblyomma* and *Ixodes*, we speculate homologs in metastriate tick species will be found when more data become available.

PEBPs are widespread among all types of organisms, however the function of PEBPs in various organisms is still being investigated. Some PEBPs have shown inhibitory function against thrombin, chymotrypsin and neuropsin [123]. Additionally, inhibition of the non-protease proteins Raf-1 kinase and G-protein-coupled receptor kinase 2 has been proposed [124]. The role of PEBPs in tick physiology has not yet been investigated beyond comparative transcriptomics. In *D. variabilis*, one putative PEBP was downregulated in the synganglion of replete-fed females, as compared to partially fed females [42]. More recently, PEBP transcripts were found in the salivary glands of three *Amblyomma* species: *A. parvum*, *A. cajennense* and *A. triste* [44]. These findings suggest PEBPs may play several different roles in tick physiology.

#### Family I53: Madanins

We found no madanin sequences in our *A. americanum* libraries. Family I53 members, known as madanins, were first discovered as blood coagulation inhibitors from *H. longicornis* [125], found in the salivary glands of feeding ticks [34]. This family has since been reported from the salivary proteome of *H. marginatum rufipes*, where a total of four sequences were annotated as madanin 1–4 [38], however none of these sequences show an I53 domain in a CDD search and these sequences show no identity to other madanin sequences and were therefore excluded them from our protease inhibitor counts. In *D. andersoni* four sequences have been identified as putative salivary madanin proteins [126] which we included in our counts, however sequences were not found in GenBank or UniProt for domain verification. In *H. bispinosa* two I53 domain sequences named haemathrin −1 and −2 have been deposited in GenBank (Additional file 1: Table S1).

Madanins are between 78 and 80 amino acids in length and share between 30 and 96% sequence identity (data not shown). Madanins have two unique features. One is that these sequences lack cysteine residues, which is relatively uncommon among thrombin inhibitors of blood feeding arthropods [127]. The other feature is two clusters of acidic residues in the N-terminus of the sequence (first 2/3 s of the sequence) [127]. Crystallography studies show madanins bind with low affinity to thrombin and bind to thrombin’s active site [127]. Madanins found in other tick species have not yet been characterized.

#### Family I63: Pro-eosinophilic major basic protein (pro-MBP)

In *A. americanum* we found 47 non-redundant pro-MBPs (Table 1). Our transcriptome data confirm expression of most (32/47) of these PIs in both male and female ticks, and most (30/47) in both fed and unfed ticks. Interestingly, we found eight pro-MBPs only in female ticks and eight only in male ticks. We also found nine pro-MBPs exclusively in each of unfed and fed ticks. Therefore, while it appears that most pro-MBPs in *A. americanum* are not sex- or time point-specific, it will be interesting to verify by qRT-PCR if there are some that are differentially expressed. While we found 48 total pro-MBP sequences in *A. americanum*, only 19 were found in SG and MG tissues, and it will be interesting to determine with qRT-PCR analysis if the other 27 are only expressed elsewhere in the tick.

We found one pro-MBP sequence for *I. scapularis* in MEROPS, but could not find pro-MBPs in any other tick species. Additional file 2: Table S2 outlines results from BLASTX scanning of *A. americanum* pro-MBPs against other tick sequences in GenBank. Eighteen of 47 *A. americanum* sequences had homologs in *I. scapularis*, which led us to increase the pro-MBP count for *I. scapularis* to 14 putatively non-redundant sequences. What is striking is the very high similarity between *I. scapularis* and *A. americanum* pro-MBP sequences: four sequences had 63–66% identity, four sequences had 75–79% identity, five sequences had 80–87% identity and two sequences had 94–95% identity to *I. scapularis* sequences. For ten of the 18 conserved *A. americanum* pro-MBPs, our transcriptome data could confirm expression in only a single tissue and time point, and six of these 10 were in unfed males only in our data. It will be interesting to further investigate the hypothesis of tissue and time point-specific expression of conserved *A. americanum* pro-MBPs.

In humans, pro-MBP has been shown to inhibit pregnancy-associated plasma protein-A (PAPP-A), which is a metalloprotease found both in a variety of normal tissues and in injured vasculature and skin, and is responsible for promoting cell growth and repair [128, 129]. Another I63 member in snake venom, bothrojaracin (*Bothrops jararaca*), has been characterized as a thrombin-induced platelet aggregation inhibitor, interacting with thrombin’s exosite 1 to inhibit factor V activation [130, 131]. Interestingly, it has been noted that pro-MBP is capable of inhibiting MBP [132, 133], which is toxic to the schistosomulum of *Schistosoma mansoni* [132, 133], however, there have been no studies investigating the role of the pro-MBP protein in any tick species or other parasites.

#### Family I68: Tick Carboxypeptidase Inhibitor (TCI)

Family I68 sequences are known as tick carboxypeptidase inhibitors (tick CPI, or TCI). In *A. americanum* we found a single non-redundant TCI in GenBank (JAG91585) (Additional file 2: Table S2)*.* In eight other ixodid tick species we found 23 putatively non-redundant TCI sequences: 10 for *R. pulchellus*, four for *I. ricinus* [134], three each for *A. maculatum* [45] and *I. scapularis* (MEROPS), and one each for *R. bursa* [135], *R. sanguineus* [39] and *H. longicornis* [136]; and (Additional file 1: Table S1). It is interesting to note the low family membership for TCIs, with most species having just one or a few non-redundant sequences. This could indicate that TCIs are functionally non-redundant and could be interesting targets for tick control. *Rhipicephalus pulchellus* is a notable exception, with 10 non-redundant TCIs. However, of the 10 non-redundant sequences our CDD searchers showed I68 domains in only eight. In *D. andersoni* six potential orthologs have been reported [137] but are not available in public databases for further analysis. The only TCI found for *A. americanum* showed 61% identity to the carboxypeptidase inhibitor in *H. longicornis* (ABO93460).

As the name suggests, TCIs are found exclusively in ticks and inhibit carboxypeptidases [50]. Functional studies show that the TCI in *R. bursa* inhibits proteases using its C-terminus at the protease active site, and its N-terminus at the carboxypeptidase exosite, and show a role for this protein in fibrinolysis acceleration by inhibiting thrombin-activatable fibrinolysis inhibitor (TAFI) [135]. TAFI also functions in wound healing, tissue remodeling and inflammation [135, 138]. Additionally, TCIs from both *R. bursa* and *H. longicornis* have been demonstrated to inhibit carboxypeptidases A and B, which function in mast cell-related inflammation, and in peptide digestion in pancreas and mast cells [136, 139, 140]. This is interesting because mast cells are suggested to be important in host defense against parasitism. Indeed, inhibitors of mast cell carboxypeptidase A from *Ascaris suum* have been concluded as a significant for the nematode’s survival within its host [140, 141]. More data are needed to further characterize the role of TCIs in tick physiology.

#### 3.35 Family I72: Chimadanins

We found no chimadanins in *A. americanum*. Family I72 was established with the discovery of a blood coagulation inhibitor from *H. longicornis* [142]. This protein was named chimadanin and remains the sole member for this family, not having been discovered in any other organism (Additional file 1: Table S1). Chimadanin is a thrombin inhibitor of only 93 amino acids [142]. Expression of this protein is notable in the salivary glands of ticks during feeding [142]. This protein shows no domains in a CDD search. A recent study of the *R. sanguineus* sialotranscriptome resulted in a sequence bioinformatically annotated as chimadanin anti-thrombin like (ACX53883) [39], however a CDD search of this sequence showed an I53 domain. Thus, the placement of this sequence in family I72 is likely incorrect, leaving the chimadanin from *H. longicornis* as the sole I72 sequence. Due to the lack of a specific domain characterizing this family it may be difficult to assign new members to this family. Future assignment may be limited to sequences showing very high sequence conservation, followed by functional characterization studies.

#### Family I74: Variegin

Family I74, known as variegin, is comprised of a single sequence from one tick species, *A. variegatum* (Additional file 1: Table S1). As was the case in our *A. americanum* transcriptome analysis and in the *A. triste*, *A. parvum* and *A. cajennense* transcriptome analyses, studies may fail to find this protein because of its unusually small size of only 32 amino acids [44]. Like madanins, this sequence lacks cysteine residues [143]. Functional characterization of this protein shows that it is a thrombin inhibitor [144]. A prolonged inhibition of thrombin is interesting from the perspective of tick feeding which requires an interruption of blood coagulation for the many days over which tick feeding occurs. It will be interesting to see if further tick transcriptome analyses reveal this protein in other tick species.

## Conclusions

Studies characterizing the function of tick PIs show a role in host protease regulation [50, 60, 145, 146], and in the regulation of tick proteases [55, 83]. This study shows that there are at least 1,595 known putative non-redundant PIs in ixodid tick species. Our *A. americanum* transcriptome analyses reveal that just six PI families: I2, I4, I25, I39, I43 and I63 represent the vast majority (80%) of the *A. americanum* PI repertoire. Based on these results, we predict many more tick PIs are as yet undiscovered, primarily from one of the big three families: I2, I4 and I8. Though limited by the fact that transcripts in this study may not have been found due to technical and not biological reasons, our analysis of *A. americium* transcriptomes showed all PI families were always present, (with the exception of I35), but also supported previous *in silico*, semi-quantitiative and quantitative RT-PCR data showing differential expression of specific PIs within families [49, 51–59], between the sexes, tissues, and throughout feeding. It is not possible to study all 1,595 tick PIs in a reasonable time frame, therefore this study provides a prioritization template for selecting suitable anti-tick vaccine and/or pharmacologically relevant targets. The discovery of tick PIs that are conserved across different tick species [53] suggests some PIs likely regulate pathways that important to all ticks, and suggests these could be targeted for development of universal anti-tick vaccines. Conversely, PI families found neither in the *I. scapularis* genome, nor in our extensive transcriptome data for *A. americanum*, suggest we will find more PIs unique to certain tick species. These proteins are not attractive candidates for a broad-spectrum anti-tick vaccine. We propose that tick PI families with low PI numbers, suggesting non-redundancy in function, and that are highly conserved across species be among the priority proteins to investigate in future studies.

## Additional files

Additional file 1: Table S1. Counts of protease inhibitors for ixodid tick species and GenBank accession numbers for accessed tick PI sequences by PI family. (XLSX 97 kb)

Additional file 2: Table S2. Results of *Amblyomma americanum* BLASTX search against tick sequences in the GenBank database. (XLSX 83 kb)

Additional file 3: FASTA sequences for *Amblyomma americanum* contigs from Illumina sequencing, by PI family. (ZIP 638 kb)
