# Supplementary material for: A repertoire of protease inhibitor families in Amblyomma americanum and other tick species: inter-species comparative analyses
Source: Parasit Vectors. 2017 Mar 22;10:152. doi: 10.1186/s13071-017-2080-1 (PMC5361777; doi:10.1186/s13071-017-2080-1)
Supplement: Supplementary file 3 — FASTA sequences for Amblyomma americanum contigs from Illumina sequencing, by PI family. (ZIP 638 kb) [file 13071_2017_2080_MOESM3_ESM.zip › A. americanum I1.docx]

>AAUF916

CCACTAGTCCACGCTAGAGCCTGTTGCGGCCTTGCCCTCCTCTTTGAAACGGATACGCGAAGGCATGGTTTCCGCTGCGGGATCGTGCGATCGTATTTCCGAGCCAAGCTCAATTTGATTACTGCCGTTCGCGTGAGCGGCGTCACTCGCGTCGGAAGCACTTGGCAACACCTACAATGAAGTTCTTGAGTGCGTGGTTGCTTTTCGCTGCTGGTTGGGCAAGCGTCACATCGGTGCCGGTAAAAGATGAGATCACAGATAGTCAGCTGGACATCTGCGCCGCCGTTATATGTCGGCCCGGAAGGGTGTGTCGGATCTTGGACAATGGAGTGGCGTCGTGCCAGTGTGTTCAGCACTGCCCATCCCACCACAAGCCTGTATGCGGTACTAATGGAGTGACTTACGACAACCACTGTATGCTTCACAAAGACGCCTGCCTGGGCCAAAAGCACATATCCATCAAGCACAAGGGACACTGCAAAAAGCCCAAGGTCAAGTTGCCACATCACAAGTATCACCAAAAGCCAGTTGTTTGCTTCCAACATGAGAGGGATCGCATTCGAAGGCACTTTGTGGAGCTTCTCGAATCTAAGGTCTCTCACGACAGGCATTACCCAAGAGTGGTTGACGATGTGTTTGATGACTGTGACCATGACGGTGACGATTTCCTCAGCACTCATGAGCTTTCAAAGTGTGTCCGGAGGAACAACACCATGTTCCACACATGGCTGAAACATAGAAGTGAACTGGCAGCTACACTGTGTATCGATGCCATGGTGGAGGTTGCTGACAGAAATTCAGATTGGCTACTCTCAAGGCGGGAGTTCCAAAATTTCATGAGGCCATCGTACCGTCCACCTATCAAGAGCTGCCCTTTGGAGGACCGGTACTATAAAGATGGTGATGAAATAATGATCGACTGCAGTAACTGTGTCTGTGCTGGTGGGAACTGGTCTTGTGCCAGCTTTCCATGTGAACTCAAGGCACCAAAGCACAAGAAAGGGCATCACCACAAGAAGAACAACAATGTTTCAGACTAACTAAATACTATACGTATGCAATGTGTCGAACAGTTGCCAGTGATGGTTTTCCCAGTCAAACCATATAATATTATGCGTTGTGATAAATGTATCTATTGAGTGCTATATACACGTTTTAAGTTTCCCCCACCTGCTTGTACAACATTTATCTTGTTGAATAAATACTCTTAGAGCAGTAATCTAAAGTATGTGCTCAATTTTCATATCAGATGTTACTATTTACAGACTACTAGTAGTTCCATTGTTAATGGACTGTTGCTATCACAACCACAACTTTCAGTGTAAACTTATTCATACTGAACAGCATTAGAGTAAGCAGTTTTTGGACAGTTCACCAGCAGCATAATGTTTTGTAGGAAACTGCCAGGCAAGATGTTGATTTCAAGCATACATTAATTGTAGCAGATCTGAAGGCTGGCTTTTGTTATACCAGACTAGACATGTGGGCATGCATGTTCCTTGTTATACACATTGATGCATTTGTGTGATGCATGATAAGCACCTGTCATGGCAGGATTCTTTACCCATTCTTCTCGAGTACACAACCAATTACAAAACAAAGCAATCTTCATTCTGTGTGTTTGTGTCACATTACTGTCCAGCTTGAGAAACAAGCCAATGTGCTTAGTGTGCTTCCACAGGATAGATTGAAGAGCAGTATATACAAAGGGCTGTGAAAGAAAGTTTTTTGTGGCTGGTGTTTTCTTATATAAAATNNNNNNNNNNNNNCATGCACCTACTCCTCTTTATGGCCTGGGGGG

>AAUF3985

GCATTCCCAAACGCTAGAGCGCTCGCTAGCTTTTTGCAGGAACAGCAAAATAAGTGTTGAAAGAATTTTTTTTTTCGAAAAAGTTTATTTGAATCTCGTAAAATGGCAACCAAACAAAAATGAAAACAATGAGGACAAGGTGACGATCACGAGCAGAAGCTTTTTGGCATGACGACGATATTCCGGCCGCAGTTTCTCTGTTGCATGCGGCAGTGGTTTAGGTAGCGTTGTCCGTCCGATCCGCACACGGGGTCCAGGATGGGCAGGCATCGCTTGGGGCACTTGGGAGGCTCCAGGCACCGGTTCAGCGGCACCAGCTGAACGTGCTCCTTGCTCCCGCAGGTCTCCTTCTTGAAGAAGCACTCGTTCAGGTACACCTCGCCGTTGGACCCGCACACGGGCTCGTACAGCTCCGAGCAGTCCTGGGGACACGAGTCCAGCTTGCGGGCCTCTCTGGCGCTGGCTATGCAGACAGCCAGTGTTTGCGTTCCGACGACCTTCCCGCAATTTCGTCGCTGCATGATGCACAGGTTGGGGTAGAAGCGGCCGTCCTTTCCGCACACCGGGTCGTAGATGTCGAGGCACTTGTCCGGGCAGAGCGGGTGGCGGCCGCGGCAGCGCTCCCACTCCACCACCCGAACGGGCTCCAGGGGACCCCCGCGCTGCCGCCGCTGTTTCCGGCAGTTGGCGTTCCTCAGCGAGCATTCGCTGTTGTACACCCGGCCGTTGGACCCGCACACCGGAACCGGAAACGATTCATTGCAGGGCATTCCGCAGGCAGAGACGCCGGGACTGCCATCATCAATTCGTGGTTGATCCACAGGTAACAATGGCTGCTCTGTAGCTGTGCCCGCGCTGCTGGCTGCGTTGGAACCTGGCTGCTGGCTGTCTCCTTCTACCGTCGGTGGCAGCGCGGTCGTAGGCACAGCTTTCGCTGCATGACTGATGAGCAGAAGGAG

>AAUF4897

TAACTCCATCAGCATCTTTTTTTTCGGAAGTTCTGTTTGCCAAATGTATTGACTTCAGCACAAAACAAAATTTTTGTTGCTTGTGAAGAACTTGCACTTGATTTTTTGTTGCTGCAAATATTTTTCAGACAGTAGGTGCCTGAAATATAGTACACTTTGGTGTGGTTATGCTTCGATATATTGCGGATCTCGGTGCCCATTTAAGGCAACATCTGTACAAGTATCGACGCTAGATGCATTCATTCCAGAAGATGCAACCACCCTGGGATTCGCAGGCGACTTGGGCACTCAGTGTACTCATTATCTTTTAATGAGCCACTGCTTTCCCTAATTTTCCTTTTGTTAAAAAACCCACATCACTGTTCTCTCACTTTTCTTGAAGCTGTCCTGGTGTTCGAAGGCAGTTGCATTTGGCAGCCTGTCTGTCAATGCGGAGCACATCCAGACCAGGGCAGACCGTATATATACAGTACCTTCACTTCGTTCTAAAACCTTTCGGTTGGAGCTCAATTTCTGTCCATTGTCATTGAATCCAAAAGGGGATTTTTGTTAAGCTGCTCTAGAATTCAAAACCTGCTGGCCTCTGGCCGTACGTTGGCCTTTTGGATGCTCTCAACCGAGAGCGCCTCAGTGATGGTTGTCATGCTGTTGTACTTGTCTTGCTCCTCAGTGCTCTCCTCAGCGAGGTCGTCCTCATAGAAGCGCTTTATGCGTCCGCTCAGCAGGAACTGGCCGATGTAGCAGACACCAGCAATGACTGCAAAGATGACCGTGATGAGGTGCAACGAGTAGCGTAGTTTGTCCGCGTCGTAAACCCAGCAGTTGCCACGCCGGCCACATTTCTCCTCCCACACAATACACGAGTTGTCCAGGATAGCGCCATACACCAAAGGGTATGGGATGAAAGCAAACATGTTCAAGAGAGAGCCTGTGGTTCCCAAGGCCATGCTTTTATCATTGGGGTCGACGCACCTGAGGGAGATCAACAGAGCGCCTACGCGGCCCGTGGAGCCCAGGAATTGGCCAGCAATCATAATGCCCAGAAAGAGGCCCAGCTGCTGGCACGAAGATCCACAGAGCCCCGAGGTCACATAGGAGTCGTCCACTCCTTGGTTTCCTTGCGCGACGCAGCTGCAACCTTTGTATATCGTCATGTTGGACGATGTCATTCCCGTGCTTCTGCAGCCGGCATGGCAAGGTGAGAAGTAGCTGATCATCCTATTGCTCGAGCACACCGGCTCGTAGATATGCGTGTTGCAGTTGCAGTCCACGTTGCATGTGTCCAGCAGGGACCTCGAGATGACATCTTTTCCCGGTGTGACTCCTGCGATCACGGGGCTTTCGCAGCCGATGAACATCATGGCTACGAATCCCGCCGTCATGGCGAGCTCGACGAATGCGGAGTAGCCGGCCACGATGCGTGGCCGTGGCCGGAATCGGTGCAACACGACGCCGGAGATTAAGAAGCCGACCATTGTAGTCGCCACCTTGGCCACCCCTGTGTAGTAGTTTGCCTTGGACGCCGACTGCCTGAATTGGAACTCGACGTACTTGTTGAACGATGTGCCATAGCCGGCTGTGGCAAGCAGGATGAAGGTCAGGCCAATTGAGTGCAACATGAAGATCGGGTTGCCTGCCAGCCGCTTTAGAATCACCNNNNNNNNNNNNNNNNNNNNNNNNNNNNNNNNNNNCTTTAGAATCACCATTATGTCTCTGACCATGGCAATGCTGAATCCGTGCTTTCCCGACCCTTCGTGTCCGTCCTCGCCTTTGGCGCCCTTCTGGGACTGGAGCGCGGAGAGCTTCTTCCGCTGGTTCACCTGGTAGTTCTTGCCGGCAGGCAGAATGCGTGGGAACAGCATCATGGGCAGGGCGATGATGCTCAGTCCAATGCCGAAAAAAATGTATCCCACCCACCAGGCGCCCACCCAACGCGGGTCGCCGGGTGTGATGCCTGGGTCGCGGAAAGGATCTTCGTAAAAGCGCAAGCATGCCCCAGCGGTCACGAATCCCAGTACAGGACCTAGGAGCCTGAAAGCAATCCCCATGCTGAAGTAGAGGGGCGAGTTCTTCTTGCGGATGTTGTCGTCCATGTAACTGCTGCCTATGATGAAGAAGG

>AAUF13189

GGTAACACTGAAGTCAGGACACATTTAATAGAAACCACTTCTCCAGGCTAGCATTGTACAGGCAGTCCTTCAAGAGCCTGTTTCGTTGCCACTCATTGTTTTTCATAAAACGAAACCGCCAGAGAAACCATTTTCAGGGCATGCTCGTGACGCACCAGAAACTATGGTTCCTCAAGGTTGATCCTATTGCAGCTTACTGATTCAGATTTTAGATTGTCTCTCAAAAAAATCACCCTTCCATCTATCAAACACCAACGAGATAAAAAAAAATGCACTGAGAAATTGCCAGCTCCTACGCAGAGCAGTCTGCAAGCAGTTCTTTCCCGCGTCAATCAACCAAGAGCACAGCAGAAACTCTCTTTCTTGCCGAGTGTTCTCGGAGGATACACCCCTCGGGGTGTTAATTTCACTCCTCGTCGGGGTCGTTTGTGGGCACCATGTGGAATATCTCGTTCCTCGTCAGTTTCCTGAAGGAGCCCTTCGATGACCTCCTGGACGAAGAGTGGAGGTCCGCCTCTCCCGGCGGCGGTACACGGCTCCTGTCGGCCAAGAAGCGCTTCTCTCGGCCGTAGAGGTTGCCCAGGCGACCGCTCAGCAGAAAGACTACGAAATTCAGTGCCACGCCAACCATGAGCAGGACGAAGGTGGTGCCGTGCAGCGAGTAGTTGAAGGTGCGCTGGTCGTACACCCAGCAGTTTCCCCGCTGGCCGCAGCTTTCCTCCCACACCACGCACGTCGAGTCCACCAATGAGCCGTACACCAACGGGTAGGGCAGGAACGAGAACAGTGCAAGGACTCCTTCAGCGAATCCGAGTGCAAATCCCTTGTCCTGCTGATCAACACATCTGAGTGGAATGACAATCTGTGCCGCCTTCGTGCTGAAGGAGAACCCTAAGGCGATGCACAAAAGCATAATGTAGAAGGTCAGGAAAGGACAGTCGCTTTCGCAGAACCCGTCTTTAGCCCTCCGAGCCGGACCGTCATTGTCAAAGGCGTCCACGCACTTGCAGTTGTCGTATGTCCCTCTCTCTGCTGTGCCGTTCGGTCCTTTGGTGGCGCATCCGGCAAAGCAAGGAGAAAAATACGTGGTCTTTCCCTCGCATATGGGATGGAACACTTCGGTAGCGCAGGAGCAGTTTTGGTTGCAGCTGTTGACCAGGTCTAATCTTGATCGGAGTTCGCCTTTTTCTCTCAGAGGAGGCTGTGGGCAGCTGAAAGCCATCAGTGAGATAAATATTAGAGCTGTGATAAGCTCCGTAGCGAAGATGATGGCTGTTAGTATGCGCGCGGAAGGCCGAAACGTCTTCATCAAGAACGCTCCACCCAGTGTGGCTACCATGGCACACCCGATCGGGAACGTTCCACTTAGGACTGAAGCAGCACTGGGTGACTGTTGGAAATGCTCTTGCACGTATTTAGGCACGTACGTACAAAAACCCAGGACACCGAACCACTTGAAGACGTGAGCGCCCATCTGTGCGACTATTATGGGGTTCTTGATAAGTCTCTTGGACGCTTCGAAGGCTTCTCTTAGGCTCATGTCTTGCTCTTCCTCAGTCGGCTGGTCGGAATTTTCTTCAGCCAGACGCCTTGGGAAGAAGAAGAGCGGCATGGAGAATAGCAGCTGCAGGAAGCCGAGAATGAAGAAGCCTAGCCACCAGGCTCCTATCCAACGCGGGTCTTCGTCCGCGAAGCCTGGGTCGTAATATGGGTCTTCGTAGATTGCGAGAATGAGGGCTGAGAAGATGAATCCCAGGCACGGTCCACATCCACGAACAACGGTCATGATCGCCATGTAGACGGCTGAGTTCTTCTTGCGGATGAGGTCGTCCAAGTAGGCTGTGCCTGCCGAGTAGTAGACGGCAGTGCCGATGCCCGTGAGGAAGTTGCAGCTGACCAGCAGCAGCAGCGCCGGAAGCGTCTCGGTCTTGCGCGACTGGGCGCAGTTGAGCTCACGGTAGCGCCGGTCCTCTTCCTGGTCGTGGCAGAAGTGGTTCAGGTCCGACTCGACCTCGGGGTCCGTCATGCTGAACGCCTCGTCCGCCTTGCCGAACAGCCAGTAGGGCAGCGAGGCCAGAAGGCACGAGGCCCCCATGAGCACAGCTGCGCCTCCCACCCATCTTGGCCGGTGCCTCTTTGGACTGTAGGTGCCGATGAAGACACCCAGAAGAATAGGACCGAAGTTGTCGGCGAAAAAGACGAAACCTGCCGTGCGGCTCCCGTAGGCGAACCGCCTTTCCAGCGTCGTGTTGACGCCGATGAGGTAGGAGTAGTAGCAGCCTTGCAGGATACCCAGGGCAGAGAAACAGATCATGTAGCACACCGGTTTAGTGAAGAAGGCCAGGAAGGCTCGCAGTGCCCCCTTCTTCTCCGCCTCCTTAGAGCTGCCGCCACGCTCCGCGGTTGGAGCCATGACTCAGCAGTTGCGAAGAACGCTGTTAACAGCACCTTTCCCTTTTCTCCAAAATTTGTGCTCAGCTGGCTCTTTCGGCGCATGGGCAATGACGGCGGTTTATGGCGACGTCAGGCCGGCCCATTCGCAGCAGTCAAGGCGTCCCGACAGGGTGCTGCCGATTTCCACACCGATTATCAGGGAACGAGATACCA

>AAUF14964

CGTCGGTGTTCAACGTCACGAGCTTCCTGTTGTCGGGCGTGCTGATCCACCGACTGAGGCCGCCGCCCAAGGTGCTGGCCTGGTACAACGTGGTCGTCACCTTCGTCGTCTCCTGCGGATTCGTCGTGGCGATGCTCGTCAAGTGCGACTACGGAACCATGCCCGGGGTATCGGTCGTCCAAGGGAACCTTGACCTGAACAACAAGTGCAACGAAGAATGTTACTGCACCCTTCAGAGTTATCAGCCCGTGTGCGAACCCGTCGGAGGCACCGTATACTTCTCTCCTTGCTTTGCTGGATGCCAAAAGCCCGAGGGAGAGGTGGGAGCTAACTTGACGAAGCTGACCAACTGCAACTGCCTGAAGACGTTCGACGACAGCGACTTCTTCTCAGGAAACGCCGTGGTTGGGTTCTGCAAAGGCACCTGCTCCATGTTCGTGCAGTTCATCATCATAGTCTCGCTGGTGCAGTTCATGGGCATGTCCACGTCCGTGAGCCACACGCTCTTCATGCTTCGGAGCATAAGCCCAAGTGACAAGACCATCGCCCTTGGACTGGCCAATGCTCTGGCGAATTTGCTGTCGTACATTCCTTACCCGCTCATCTACGGCGCCGTGATCGACAGCTCTTGCCAGGTGTGGGAGAGCGCCTGCGGGACGAGCGGCAACTGCTGGCTGTACGACCTAACGCGATTGCGCCACTCGTACCTGGGCACGTCGGCCGGCTTCCTGGCCGTGTCGGGCATCTTCTCGATCGCCGTCGCCCTGGTGGCGGGTGACCTGAAGGACTTCTACGGGGACGCCTACGTACAGGTGTCCCACTTGGGTGGCGCTGACTTCGAAGCACGGAACATCGGCGTCGAGAAACGCAATAAGACTGTCGCCAAACAGGAGAGTAACGGTCACCGCCCCAAGTGACCGCCGGGAGGCCCGACGGTGTAGCACGTTGTCGGAGGCGTGTTACTGTTATTCCATTTATGGACGCTAACCAAAACTTTTTCGAGGTTCTTTAAAAGTCTAGTGCTCGTAGTGCTAGCAGCCGCTGGAGCCAATCAGCTTCTGTGGCTTCTTGCAGCGGAAAGGTTCCCGCGGCGGTCGCATTTCAGTTGAGGAAGGACGCAACGTCTATG

>AAUF18077

TCTGCCTAAGTACTTGGAGAGCCAGTTTCGGCTCACCGAGACCAAGGCCAACATGATTACAGGGTTTGCTGGCATTCTCGTCATGGGTGTGGGCATTTTTGCCAGTGGCACATTCATGCGCAAGTACAAGCCCAACGCCCGCTTTGTTGCCAAATGGATTGCATTCACTGCCCTGGCCTACTCCGTCGGCATGGTCATCCTCATGTGGGTGGGCTGTCCCCTCGGAGACTACGTTGGCCTCAATTCAGAACACAAAAGCCTAGACTACCTACCTCCAGCCTGCAACAGCACTTCTTGCGAGTGCAAGTCTGGGCTGTTCTCTCCAGTGTGCCATGATAGTGTGACCTACTTGTCGCCATGCCTGGCTGGCTGTTCACAAGTGTCTGGCAGTGATCAGCAGCCGAACTTCAGTGACTGCCATTGCACAGGAACAAATGAAACCATAACAAATGGGTTCTGCTCCCTTGAGTGCAACAACCTGACCTGGTACATCGTCATCTTCTCTGTTTTTGTACTCATCCACTCAACATCTGAAGTAGGGTCTATGCTACTGACCCTCAGATGTGTTGAATCCCATGACAAGGCCCTTGCATTGGGACTCATCCAGTTTGCCATTGGATTGTTTGGGAACGTGCCTTGTCCCATCATCTATGGTGCAGTCGTTGACTCTGCCTGCCTCTTCTGGGAAGACAACTGCGGAGAACCGGGCGCATGCCGCGTCTACGACCCGGCCAAGTTTCGTATGGTGTTCCATGGAGTCACAGCTGTCATCATGTTTGTGGCGTTCCTTGTGGATGCTGTTGTTTGGTACAAGGCGAGCTCCATCCACATTCACGAAGAGGAAGAGACCAGGGACACTGCGGTGGCAGCGGCAGCTCACGACGTGTCCTCTTCGACTCCACCAGTCCTGACGGCACATGCGGAAACAGAATCGTGCGTGTGAGCAGAGGTGGCTTTCCTCACGGCCGAGTGCTCTGGGTTGAGAAAAAAG

>AAUF18894

GAGGACCAAGTTGAGGATCGCTGCAGTGAAGGACGGAAGGCCGCGGGAGACGACTGCAGAGCAGGGACCGCTTCACGCTTTGTGTGGCGCACAGCGTTCATTGTTTAGCAGGGTGCGCACATCGCAAAACTAAACCTTCATTTCGGCCACCGCCACCACTGCCAAACGCGCTGAGTCTCTAGCCCGACCAGCCAAGCAAGCACTGAGCCATGACGCTCATCAGCGGAGACAGACAGCGGTTCCCGCGAGACGACAGCGTCTCCAGCCTCGGTACCATGACCAACTCGCAAAGCAACGAGCCCCTGGTGGTCGGCAAGCCTCCTGTCATCCCAGACCCCGTGGGTATACCCGACACCGCAGTGCATCCTAGGGGAATGGTCGCCGACTATGACGAAGACGTGGGCGGGACCTGCGGTTGGGGTTGCCTGCGACCCAGCTGGCTCCAGCGTTTCCGCACGCCTCCGTGGGTGCTGTTCTTCCTGTGCTGGGCGGGGTTCCTGCAGGGACTCATCGTGAACGGCTTCGTCAACGTGGTCATCACCACCATCGAGCGGCGCTTCCAGCTGCGCAGCTCCGAGAGCGGCCTTGTGGCCAGCGGGTACGACATCGCGTCCTTCCTGCTCCTGGCGCCCATCAGCTACTTCGGCGGCACCCGGAGCAAGCCGCTGTTCGTGGGCGTCGGCTGCCTCGTGCTGGGCCTCGGTGCCTTCGTCTTCTCGCTGCCGCACTTCCTGGCCGGTACGTACGCCTTCAGCACCGAGGACGACCGTGAGAGCCTCTGCCGCCTGGCGGCCAACGCCACCGAGGGCTTCTGCGGGAGCAGGGAGGTGGGCTCCCTGAACCAGTACAAGTACATGTTCCTCGCGGGACAGATGCTGCATGGCGCCGGGGCGACACCCTTCTTCACTCTCGGCTGTACTTACCTGGACGAGATCGTGTCTACGAAGATGTCCTCTGTGTACATTGGTATTTTCTATACCATGGCGATCATCGGCCCTGCACTTGGCTATATTCTAGGCGGCCAATTTCTGAAGATATACACCGACCTCTCCGTAGATGCTTCAGCATTAGGCTTGACGCCCTCCAGTGGTGTCTGGGTGGGCGCCTGGTGGATTGGTTTCGTCGTGTCCTCCGTAACGGCTCTTCTTGCTGCCGTCCCCATTAGTGCCTTCCCCAAGGTCCTCCCAGGTTCATTAAAGCTGCAGGCTCAGAAGAAATCCGAGATGCACCAGAAGCTTCAGAAGAGCGAAGCGGTGCAGAGCGGCTTCGGGGCCCGCGCCAAGGATCTCCCGGCATCCTTCAAGATACTCATCACCAACCCGACCTTTGTGTTCCTAAGCTTGGCCGGAGCCACCGAAGGTATGCTGGTATCTGGCCTGGCGACATTCCTTCCTAAAGTCATTGAGTTCCAGTTCAGCATAGCGGCCAGTTTAGCGGCCCTAATAATGGGTGCGGTGACAGTTCCGGGCGCTGGTGGTGGCACATTCCTAGGCGGCTACTTCGTGAAGAAGTTCAACCTGCGCTGCGCCGGCATCATCAAGATGTGCGTCCTCTGCTCTCTCGTTCCACTCGTCACCATCTTCGCATTCTTCTTCAGCTGTCCGAACGTCAGATTCGCGGGCGTCAATTACAAGACGAACAACGTCACCGATGGAATAGACCGCTTCCTGTTAGACTGCAATAATCAGTGTCACTGCCAAATGGAAGACTTTGACCCCATCTGCGGAACGGACCACGTCATGTACTATTCCGCATGCTTTGCAGGTTGCCAAGAGGTGCACCACTATGGATCAACAAAAGTGTACGAGGACTGCCGCTGCATTGATCATCCGGGCAGAAACGTAACAATTAATGGGAAGCAAGTGACCATCCAGGCGGAGAGAGACAAATGTCCAAGCGAATGCAACTTCCTCGTCTTCTTCCTTATCGCCATGTTCGTCTGCATGGTCTTCACATTCCTGGTCAGCATGCCTTCGCTGGCCGCCACACTCAGGTGTGTTGCAGCTAGCCAGAAGTCTTTCGGTCTCGGCATACAGTGGATTGCTGTGCGGCTACTAGGGACCATACCGGCGCCCATCATGTTCGGCTACCTGATCGACCGCAGCTGCGTGCTGTGGCCCGGCTCGTGCGATGACTCCGGCGCCTGCGCCGTCTACGAGAATGGCCAGATGGCGCGCAACCTGCTCGCGCTGCTTGCCACCGTCAAGTTCCTCTCCTGTCTCTTCTTCTTCCTCTCGTGGCTGCTCTACAAGGCGCCCGAGGGCGAGGACGAAGAGGACGACGGCAACCAGGCTGCGGCGCCTGAGAAGTCTGGCGTCGCCACCATCAAGCAGGAGACTGTTGTGACTGGTGCTCCGTTTAAGACGGCCACGAACGGTGCAGCCGCCGGACAGCAGCAGAATCACAGCAGTGACAACAACAGCACGTGGTTCTGAACAGTGCAAAGAAACAGAGTGCAAGTTGTATTTG

>AAUF19120

CCTTCTTCATCATAGGCAGCAGCTACATGGACGACAACATCCGCAAGAAGAACTCGCCCCTCTACTTCGGAATGGGGCTTGCCTTCAGACTCCTGGGTCCTGTTCTGGGATTTGTGACTGCCGGGGTTTGCCTGCGTTTTTACGAAGACCCCTTCCACGACCCGGGCATCACACCAAGGGACCCACGCTGGGTGGGTGCATGGTGGATGGGCTACATTCTGTTCGGCATCGGGCTGAGCATCGTCGCCCTGCCCATGATGCTGTTCCCGCGCATTCTGCCCGCCGGCAAGAACTACAAGGTGAACCGCCTGCAGAAGCTGTCCGCACTCAAGTCCCAGAAGGGCGCCAAAGGCGAGGAAGGACAGGAAGGGTCGGGAAAGCACGGATTCAGCATCGCCATGGTGAAAGACATAATGGTGATTCTNNNNNNNNNNNNNNNNNNNNNNNNNNNNNNNNNNNNNNNNNNNNNNNNNNNNNNNNNNNNNNNNNNNNNAGCAACCCGATCTTCATGTTTCACTCAATTGGCCTGATATTCGTCCTGCTTGCCACGGCCGGCTACGGCACCTCGTTCAGCAAGTACGTCGAGTTCCAGTTCAGGCAGTCGGCGTCCAAGGCTAACTACTACACAGGGGCAGCGAAAGTGGTGACTACCCTGATCGGCATCGTCACCGGCGGCGTCGTGGTGCACCGGTTCCGACCACGGCCGCGCATCGTGGCAGGCTACTCAGCGTTCGTCGAGATCGCCATGATGGCGGGCTTCGTGGTCATGATGTTCATCGGCTGCGAAAGTCCCGTCATCGCGGGAGTCACTCCTGGTGGCAACGTCACCGCGAGTTCGCTGCTGGACACATGCAACGTGGACTGTAACTGCAACACGCAGATCTACGAGCCGGTGTGCTCGAGCAATAGGATGATCAGCTACTTCTCACCTTGCCATGCCGGCTGCAAAAGCGTGGGAACGACATCGTCCAACATGACGATATACAAAGACTGCAGCTGCGTCGC

>AAUM38395

CCTGCTCGCTGCGTTTGCTGCGGCCGCGCTCGCCCTCGCTGCTGCAGCTGCGGGCCCGACGCTGACTCTCCAGAATGAAGGATGTCCACCGTCAACAAAGGCATGCACCGGAGGAGAGCCTGTCTGTGGCTCGGATGGGCGCACATATGCCACACGGTGCGACTTTGAGCGTGCCCAGTGTGAGGAGCACTCTTTGGAATTGAGCCATGTGGGACCGTGCTCTGAGCCGACGCCAGCCGCAACTGCAGCACGATGCCTGCTCCAGCGCAAGCAGCAGCAGTCCGACTTGTTTGTGCCAGAATGCGCACCGGATGGAGGCTTCAAGCGTGTCCAATGTCATCACATGACGGGATACTGCTGGTGTGTGGATGACCAGGGCCGGCCCCTGGCGGGAACCTCCATGCACCTCCAGCAGCCCAACTGTACCACGCGGCGACAAGGCTCGTTCCAGCGCAGGGCCTCTCACCAGGGTCAGGCCAAGATAGGTTGCTCAAGCG

>AAUM5263

ATCTATCAAACACCAACGAGATAAAAAAAAATGCACTGAGAAATTGTCAGCTCCTACGCAGAGCAGTCTGCAAGCAGTTCTTTCCCGCGTCAATCAACCAAGAGCACAGCAGAAACTCTCTTTCTTGCCGAGTGTTCNNNNNNNNNNNNNNNNNNNNNNNNNNNNNNNNNNNNNNNNNNNNNNNNNNNNNNNNNNNNNNNNNNNNNNNNCTCGTCAGTTTCCTGAAGGAGCCCTTCGATGACCTCCTGGACGAAGAGTGGAGGTCCGCCTCTCCCGGCGGCGGTACACGGCTCCTGTCGGCCAAGAAGCGCTTCTCTCGGCCGTAGAGGTTGCCCAGGCGACCGCTCAGCAGAAAGACTACGAAATTCAGTGCCACGCCAACCATGAGCAGGACGAAGGTGGTGCCGTGCAGCGAGTAGTTGAAGGTGCGCTGGTCGTACACCCAGCAGTTTCCCCGCTGGCCGCAGCTTTCCTCCCACACCACGCACGTCGAGTCCACCAGTGAGCCGTACACCAACGGGTAGGGCAGGAACGAGAACAGTGCAAGGACTCCTTCAGCGAATCCGAGTGCAAATGCCTTGTCCTGCTGATCAACGCATCTGAGTGGAATGACAATCTGTGCCGCCTTCGTGCTGAAGGAGAACCCTAAGGCGATGCACAAAAGCATAATGTAGAAGGTCAGGAAAGGACAGTCGCTTTCGCAGAACCCGTCTTTAGCCCTCCGAGCCGGACCGTCATTGTCAAAGGCGTCCACGCACTTGCAGTTGTCGTATGTCCCTCTCTCTGCTGTGCCGTTCGGTCCTTTGGTGGCGCATCCGGCAAAGCAAGGAGAAAAATACGTAGTCTTTCCCTCGCATATGGGATGGAACACTTCGGTAGCGCAGGAGCAGTTTTGGTTGCAGCTGTTGACCAGGTCTAATCTTGATCGGAGTTCGCCTTTTTCTCTCAGAGGAGGCTGTGGGCAGCTGAAAGCCATCAGTGAGATAAATATTAGAGCTGTGATAAGCTCCGTAGCGAAGATGATGGCTGTTAGTATGCGCGCGGAAGGCCGAAACGTCTTCATCAAGAACGCTCCACCCAGTGTGGCTACCATGGCACACCCGATCGGGAACGTTCCACTTAGGACTGAAGCAGCACTGGGTGACTGTTGGAAATGCTCTTGCACGTATTTAGGCACGTACGTACAAAAACCCAGGACACCGAACCACTTGAAGACGTGAGCGCCCATCTGTGCGACTATTATGGGGTTCTTGATAAGTCTCTTGGACGCTTCGAAGGCTTCTCTTAGGCTCATGTCTTGCTCTTCCTCAGTCGGCTGGTCAGAATTTTCTTCAGCCAGACGCCTTGGGAAGAAGAAGAGCGGCAGGGAGAATAGCAGCTGCAGGAAGCCGAGAATGAAGAAGCCTAGCCACCAGGCTCCTATCCAACGCGGGTCTTCGTCCGCGAAGCCTGGGTCGTAATATGGGTCTTCGTAGATTGCGAGAATGAGGGCTGAGAAGATGAATCCCAGGCACGGTCCACATCCACGAACAACTGTCATGATCGCCATGTAGACGGCTGAGTTCTTCTTGCGGATGAGGTCGTCCAAGTAGGCTGTGCCTGCCGAGTAGTAGACGGCAGTGCCGATGCCCGTGAGGAAGTTGCAGCTGACCAGCAGCAGCAGCGCCGGAAGCGTCTCGGTCTTGCGCGACTGGGCGCAGTTGAGCTCACGGTAGCGCCGGTCCTCTTCCTGGTCGTGGCAGAAGTGGTTCAGGTCCGACTCGACCTCGGGGTCCGTCATGCTGAACGCCTCGTCCGCCTTGCCGAACAGCCAGTAGGGCAGCGAGGCCAGAAGGCACGAGGCCCCCATGAGCACAGCTGCGCCTCCCACCCATCTTGGCCGGTGCCTCTTTGGACTGTAGGTGCCGATGAAGACACCCAGAAGAATAGGACCGAAGTTGTCGGCGAAAAAGACGAAACCTGCTGTGCGGCTCCCGTAGGCGAACCGCCTTTCCAGCGTCGTGTTGACGCCGATGAGGTAGGAGTAGTAGCAGCCTTGCAGGATACCCAGGGCAGAGAAACAGATCATGTAGCACACCGGTTTAGTGAAGAAG

>AAUM13175

GCGACCACGCAGAGAGAAAGAGAGGGCATCCCGACAGAGAAGATGAGGCGCTCTCCTGGTGACACAATGGCGCGGGGCAGCTGTGTGTGCACAGGTTGCGTTCGAAGCGGAAGCCCCTCCGCCACCAGTGCGTAGGCACACTTTGGCCGCTGCCGCTGCCCGCCTGTGGTCAGCCCCTTTGCGACTGCTGGAACCTTCCACACACGAGATACCTTCGGCGGCACGAGGAGTGACTGCGCCTTCGAACAGCGGGGAACCAGCCGGCCTGGAGACGTCGCGCAACAAGATGAACCAAAATGGCACCCAGAAGCCGGCATGGACCGTCGAGCGGACGACGGCGGAACAGGAGCGACCCGACGCGAAGTCGAAGCCGGACGCCGTGGCGAACGGTGGATGCGCCGACGGCGCCGTTCAGGAGGAGCACGACTACATGTGCGGCCTGGGAAGCTGCTACCCGCGGTTCCTGCAGCGGTTTGCCAATCCTAGGATGTTTTTCATCGTCTACAGCCTCATGGGCATCCTCCAGGGCGCGTACAAGACGTACTTCGTGGGCACGCTGTCGACCATCGAGCGGCGCTTCGCCATGTCCAGCCAGACGACGGGCATCATCCTGATCGCGGACAACCTGAGCCCCATCATCATCAACCTGCTGGCCGGCTACTACGCGAACCGCATCAGCAGGCCCAAGATCATGGGCATCGGCGCGCTCATCGTGGTCGTCAGCTGCTTCATCAGCATCGTGCCATACATGGTGTATGGCCCGGGACTGCACATACTCACCAACACGGCGCACGCCCTGGGTGCGCGGCGGAGGAGTCCGGACAGCCTTCTCTGCAACTCGGCGGCGGCGGGCCACCGCGAGTGCGCCGGCTCCTCGCTCGCGGACAGCTACACGCCGTTCGCGCTCTTCTTCGCGGCCAACTTCCTGAACGGCTTCGGTGGGTCCACCTTCTTCATCGCCGGCAGCAGCTACATGGACGACAACATCCGCAAGAAGAACTCGCCCCTCTACTTCGGAATGGGGCTTGCCTTCAGACTCCTGGGTCCTGTTCTGGGATTTGTGACTGCCGGGGTTTGCCTGCGTTTTTACGAAGACCCCTTCCACGACCCGGGCATCACTCCCAGGGACCCACGCTGGGTGGGTGCATGGTGGATGGGCTACATTCTGTTCGGCATCGGGCTGAGCATCGTCGCCCTGCCCATGATGCTGTTCCCGCGCATCCTGCCCGCCGGCAAGAACTACAAGGTGAACCGCCTGCAGAAGCTGTCCGCACTCAAGTCCCAGAAGGGCGCCAAGGGCGAGGAAGGACAGGAAGGGTCGGGAAAGCACGGATTCAGCATCGCCATGGTGAAAGACATAATGGTGATTCTAAAGCGACTGGCGAGCAACCCGATCTTCATGTTTCACTCAATTGGCCTGATCTTCGTCCTGCTTGCCACGGCCGGCTACGGCACCTCGTTCAGCAAGTACGTCGAGTTCCAGTTCAGGCAGTCGGCGTCCAAGGCTAACTACTACACAGGGGCAGCGAAAGTGGTGACTACCCTGATCGGCATCGTCACCGGCGGCGTCGTGGTGCACCGGTTCCGACCACGGCCGCGCATCGTGGCAGGCTACTCAGCGTTCGTCGAGATCGCCATGATGGCGGGCTTCGTGGTCATGATGTTCATCGGCTGCGAAAGTCCCGTCATCGCGGGAGTCACTCCTGGTGGAAACGTCACCGCGAGTTCGCTGCTGGACACATGCAACGTGGACTGCAACTGCAACACGCATATCTACGAGCCGGTGTGCTCGAGCAATAGGATGATCAGCTACTTCTCACCTTGCCATGCCGGCTGCAAAAGCGTGGGAACGACATCGTCCAACATGACGATATACAAAGACTGCAGCTGCGTCGCGCAAGGAAACCAAGGAGGGGACGATTCCTACGTGACCTCGGGGCTCTGTGGATCCTCGTGCCAGCAGCTGGGCCTCTTTCTGGGCATTGTGATCGCTGGCCAGTTCCTGGGCTCCACGGGCCG

>AAUM19226

CGGGCGGGACCTGCGGTTGGGGTTGCCTGCGACCCAGCTGGCTCCAGCGGTTCCGCACGCCTCCGTGGGTGCTGTTCTTCCTGTGCTGGGCGGGGTTCCTGCAGGGACTCATCGTGAACGGCTTCGTCAACGTGGTCATCACCACCATCGAGCGGCGCTTCCAGCTGCGCAGCTCCGAGAGCGGCCTTGTGGCCAGCGGGTACGACATCGCGTCCTTCCTGCTCCTGGCGCCCATCAGCTACTTCGGCGGCACCCGGAGCAAGCCGCTGTTCGTGGGCGTCGGCTGCCTCGTGCTGGGCCTCGGTGCCTTCGTCTTCTCGCTGCCGCACTTCCTGGCCGGTACGTACGCCTTCAGCACCGAGGACGACCGTGAGAGCCTCTGCCGCCTGGCGGCCAACGCCACCGAGGGCTTCTGCGGGAGCAGGGAGGTGGGCTCCCTGAACCAGTACAAGTACATGTTCCTCGCGGGACAGATGCTGCACGGCGCCGGGGCGACACCCTTCTTCACGCTCGGCTGTACTTACCTGGACGAGATCGTGTCTACGAAGATGTCCTCTGTGTACATTGGTATTTTCTATACCATGGCGATCATCGGCCCTGCACTTGGCTATATTCTAGGCGGCCAATTTCTGAAGATATACACCGACCTCTCCGTAGATGCTTCAGCATTAGGCTTGACGCCCTCCAGTGGTGTCTGGGTGGGCGCCTGGTGGATTGGTTTCGTCGTGTCCTCCGTAACGGCTCTTCTTGCTGCCGTCCCCATTAGTGCCTTCCCCAAGGTCCTCCCAGGTTCATTAAAGCTGCAGGCTCAGAAGAAATCCGAGATGCACCAGAAGCTTCAGAAGAGCGAAGCGGTGCAGAGCGGCTTCGGGGCCCGCGCCAAGGATCTCCCGGCATCCTTCAAGATACTCATCACCAACCCGACCTTTGTGTTCCTAAGCTTGGCCGGAGCCACCGAAGGTATGCTGGTATCTGGCCTGGCGACATTCCTTCCTAAAGTCATTGAGTTCCAGTTCAGCATAGCGGCCAGTTTAGCGGCCCTAATAATGGGTGCGGTGACAGTTCCGGGCGCTGGTGGTGGCACATTCCTAGGCGGCTACTTCGTGAAGAAGTTCAACCTGCGCTGCGCCGGCATCATCAAGATGTGCGTCCTCTGCTCTCTCGTTCCACTCGTCACCATCTTCGCATTCTTCTTCAGCTGTCCGAACGTCAGATTCGCGGGCGTCAATTACAAGACGAACAACGTCACCGATGGAATAGACCGCTTCCTGTTAGACTGCAATAATCAGTGTCACTGCCAAATGGAAGACTTTGACCCCATCTGCGGAACGGACCACGTCATGTACTATTCCGCATGCTTTGCAGGTTGCCAAGAGGTGCACCACTATGGATCAACAAAGGTGTACGAGGACTGCCGCTGCATTGATCATCCGGGCAGAAACGTAACAATTAATGGGAAGCAAGTGACCATCCAGGCGGAGAGAGACAAATGTCCAAGCGAATGCAACTTCCTCGTCTTCTTCCTTATCGCCATGTTCGTCTGCATGGTCTTCACATTCCTGGTCAGCATGCCTTCGCTGGCCGCCACACTCAGGTGTGTTGCAGCTAGCCAGAAGTCTTTCGGTCTCGGCATACAGTGGATTGCTGTGCGGCTACTAGGGACCATACCGGCGCCCATCATGTTCGGCTACCTGATCGACCGCAGCTGCGTGCTGTGGCCCGGCTCGTGCGATGACTCCGGCGCCTGCGCCGTCTACGAGAATGGCCAGATGGCGCGCAACCTGCTCGCGCTGCTTGCCACCGTCAAGTTCCTCTCCTGTCTCTTCTTCTTCCTCTCGTGGCTGCTCTACAAGGCGCCCGAGGGCGAGGACGAAGAGGACGACGGCAACCAGGCTGCGGCGCCTGAGAAGTCTGGCGTCGCCACCATCAAGCAGGAGACTGTTGTGACTGGTGCTCCGTTTAAGACGGCCACGAACGGTGCAGCCGCCGGACAGCAGCAGAATCATAGCAGCGACAACAACAGCACGTGGTTCTGAACAGTGCAAAGAAACAGAGTGCAAGTTGTATTTGGGGTCGTGTGCATCGGGGCCCAGTGATGCTCGCCGTGCTGGTGTGCAAAGAGTGGCGTAGTTCTTCGCGGTTCCACGGTTCTTGGCTGTTCGCAGCTGTGAGAGGCATCATGAACCTCTGT

>AAUM22645

GAATATTCTTGGGTGGCTACGTTCTGAAGAAGTTCCAGATGAGACCAAAAGGGGCCATCCAGTTTGTGCTCTGCTTCAACTTGCTATGCATGGGCCTCTACACTCTCCTGTACTTCTTAGGCTGTGATAACATTCGAATGGCAGGTGCCACACTTCCCTACTTTAACAACAGCAGCCTGCTGGAGACGTTTCAGGTGAACCTGACTGCACTCTGCAACATGGGCTGCCGTTGTTCACCCAACGACATCGAGCCTGTCTGTGGTCGCAATGGCATCACTTACTTTTCATTNNNNNNNGCGGGCTGTCGGCCTGGAGCTGGTCACGGATCACATCTCAACTACACGTCATGTGCCTGCATCGTGCCTAATGTGACCGTGAGCCCCGAAGTGACAGCTGTGCCGCTAGCAACAAGCGGGCCATGCCCACAGCCATGCCTGGCCTTCATCCCATTCATGGTGCTCCTGTTCGCCATGACACTGGTGGTCTCCATCACGCAGATGCCACTGCTTATGATAATACTGCGATCTGTTGGAGAAGAAGAGAGGTCGTTTGCATTGGGAATGCAGTTTGTAATATTTAGGCTATTTGGTTACATACCAGCTCCAATAATGTTTGGAAACGTGATAGACTCGTCATGCATCCTCAGGAAGGCTCACTGTGGGAAGCCTGGAGGATTCTGCTTGGTCTATGACATTGAGCAGTTCAGACTAAGGTATATTGCCGTGTGCTCTGGTCTCAAAGTGGCTGCGGGGCTGTTGTTTTTCCTTGACTGGCTCCTCATCACGTGGCGTCACAAACGGGAGCTTAAGGAAGCTCCACCCATGACGGTTGGGGAGATCGTCTCCTCAATCATTTCACTGGACCGACTATCCGCCCTTGGCTGGGGCGACG

>AAUM26802

TGCCTGCTGCTGCTGGCGAGCGTTCCCGTGCTGCTCTTCCCCAAGGTGATGCCGGCCCGGCTGCCGGACCCGCCGCTGGTCTTCAAGAAGCGAGCCGACTCCAGCCACATCTCGGAGATCAGCAAATCGTTGACCCGGCTGCTCCGGAAGCGAGTGTACGTGCTGCAGTTGTTCGTGTCCATGCTGATGTACAGCGGCCTGCAAGGATACACCATGTTCTCGGCCAAGTACATGGAGGTCGAGTTTCGCAACTCGGCGGCCAGGGCCAGCGCGTTCGCTGGGCTGATCTCGTCGGTGTTCAACGTCACGAGCTTCCTGTTGTCGGGCGTGGTGATCCACCGACTGAGGCCGCCGCCCAAGGTGCTGGCCTGGTACAACGTGGTCGTCACCTTCGTCGTCTCCTGCGGATTCGTCGTGGCGATGCTCGTCAAGTGCGACTACGGAACCATGCCCGGGGTATCGGTCGTCCAAGGGAACCTTGACCTGAACAACAAGTGCAACGAAGAATGTTACTGCACCCTTCAGAGTTATCAGCCCGTGTGCGAACCCGTCGGAGGCACCGTATACTTCTCTCCGTGCTTCGCTGGATGCCAAAAGCCCGAGGGAGAGGTGGGAGCTAACTTGACGAAGCTGACCAACTGCAACTGCCTGAAGACGTTCGACGACAGCGACTTCTTCTCAGGAAACGCCGTGGTTGGGTTCTGCAAGGGCACCTGCTCCATGTTTGTGCAGTTCATCATCATAGTCTCGCTGGTGCAGTTCATGGGCATGTCCACGTCCGTGAGCCACACGCTCTTCATGCTTAGAAGCATAAGCCCAAGCGACAAGACCATCGCCCTTGGACTGGCCAATGCTCTGGCGAATTTGCTGTCGTACATCCCTTACCCGCTCATCTACGGCGCCGTGATCGACAGCTCCTGCCAGGTGTGGGAGAGCGCCTGCGGGACGAGCGGCAACTGCTGGCTGTACGACCTAACGCGATTGCGCCACTCGTACCTGGGCACGTCGGCCGGCTTCCTGGCCGTGTCGGGCATCTTCTCGATCGCCGTCGCCCTGGTGGCGGGTGACCTGAAGGACTTCTACGGGGACGCCTACGTGCAGGTGTCCCACTTGGGTGGCGCTGACTTCGAAGCACGGAACATCGGCGTCGAGAAACGCAATAAGACTGTCGCCAAACAGGAGAGTAACGGTCACCGCCCCAAGTGACTGCCGGGAGACCCGACGGTGTAGCACGTTGTCGGATACGTGTTACTGTTATTCCATTTATGGACGCTAACCAAAACTTTTTCGAGG

>AAUM37647

CTGCCCCAAGCACCAGCTCCAGCAGAAGATGCCGCTCATCAGTTCACCGCCTGCTTCGCCGTCGGGAGCCAAGAAGGCCCTGAAAGACAAGCCCACACTCAGAGATTTCCCCACAGCCATTCGGCGGCTGTTGAAGAACGAGATCCTGCTGTACCGGACGGCCAGCAGTGTCCTGCACATCTTGCCAATCGCTGGCCTCTACACTTTTCTGCCTAAGTACTTGGAGTGCCAGTTTCGGCTCACGGAGACCAAGGCCAACATGATTACAGGGTTTGCTGGCATTCTCGTCATGGGTGTGGGCATTTTTGCCAGTGGCACATTCATGCGCAAGTACAAGCCCAACGCCCGCTTTGTTGCCAAATGGATTGCATTCACTGCCCTGGCCTACTCCGTCGGCATGGTCATCCTCATGTGGGTGGGCTGTCCCCTCGGAGACTACGTTGGCCTCAATTCAGAACACAAAAGCCTAGACTATCTACCTCCAGCCTGCAACAGCACTTCTTGCGAGTGCAAGTCTGGGCTGTTCTCTCCAGTGTGCCATGATAGTGTGACCTACTTGTCGCCATGCCTGGCTGGCTGTTCACAAGTGTCTGGCAGTGATCAGCAGCCGAACTTCAGTGACTGCCATTGCACAGGAACAAACGAAACCATAACAAATGGGTTCTGCTCCCTTGAGTGCAACAACCTGACCTGGTACATCGTCATCTTCTCTGTTTTTGTACTCATCCACTCAACATCCGAAGTAGGGTCTATGCTACTGACCCTCAGATGTGTTGAATCCCATGACAAGGCCCTTGCATTGGGACTCATCCAGTTTGCCATTGGATTGTTTGGGAACGTGCCTTGTCCCATCATCTATGGTGCAGTCGTTGACTCTGCCTGCCTCTTCTGGGAAGACAACTGCGGAGAACCGGGCGCATGCCGCGTCTACGACCCGGCCAAGTTTCGTATGGTGTTCCATGGAGTCACA

>AAUM56409

CCGGCCCCGGCTGGTCACCTTGTACACGACGCTGTGTGACGTGCTCAGGCTGGCTTGCCTCGTGGCGTGCGCCTTTATCGGTTGCGAAGCCGTACGACTCGCGGGCACCGTCACCACCGTCGCTGGCCACGGGTCGTCAATACAGCTGGCTTGCAGTGATAGCTGCAACTGCACCACCAGGCACTTCCAGCCGCTTTGCGACCCAGTCAACCGTACTGTTTTCTTCTCTCCATGTCATGCGGGCTGCACCAAGTTTTCCGCTACAGATTCTGGGGAAGTGTCCTTCGACGAGTGCTCGTGCTTGCCCAACCTGCCCAGCCAACTACAGCGGGGCCAGCAAAAGGAGCAGCTTGTGCAAGCCGACCAAGGGGCTCCGTACTTAGGGCTTTGCAGCGGGGACACGTGCAGCAACGTGCTGGCATTCATCGCGCTGTCGTCGCTGGTCGGCTTCATCGCGCGAACCACAACTGTGGGACACACTATCGTCGGGCTCAGGTGCGTGTCCCGCGAGGAGAAGGCAATGGCACTTGGCGTCCAGGAAG

>AAUM5795

GCTCTCGTTTCTTCGAATCTACGGCCGGCTAGACCGTAGGCGAACACACTTGCAGCGGCCAGTCCGCAGTCGCCACTCGTAGCAGGCAGATCCCAGAGCGATCCACCGCCATGAGAGCCGTCCACTGCACCCCGTTGCTGCTGCTCGCGCTGTGCGGCCTGCTGGCGCCGGCGTCGTCGCGCAAGGAGTGCGGGCCTTGCGACCTGGACCGGTGCGAGCCTCCGAGCGGCCAGTGTCTGGCCGGCATCGTTCAAGACGCGTGCGGCTGCTGCATGGTGTGCGGTCAGCGCGAGGGCCGGCGCTGCTACCACCGGTCGGTGAAGGGCAGCCTCGTGGACGGGCCGTGCGGCGAGGACCTGGAGTGCCGGCTGCGCCGCGACCTGGCCCCCGGAGACCCCGCCGAAGCGCTGTGCGTGTGCTCGCGCCAGGAGCCCGTGTGCGGCACCGACGGCGTCACCTACGACAACGTGTGCCAGCTGACCGAGGCCCGGTACCGGCTGCGCAACGGGCTCGAGGCGGCCTCCAGGGGACCCTGCTACTCCGCGCCCCGAGTGGTGACCGCTCCGGAGAACACGCGCAACCGGACGGGTGGCCGCGCCGCCATGACCTGCGAGGTGTCCGGCTTCCCGGTGCCCACCATCGAGTGGCGCGTCGACCGCGGGGACGGACCGCTCAAGTCGCTGCCCACGGACAGCTCCCGCATCAACGTGCAGAGCCGCGGCGGACCGGACAGCTTCGAGGTGACCAGCTGGCTGCAGCTGCTCGACCTGAGGCCAGAGGACACGGCCACCTACTGGTGCGTGGGCGCCAACGAGAACGGAGAGGCCTCGGCCGCCGCCAAGCTCAACGTGCTGCCCTAGGCCGCCCCTCGTCCTTGTTGTTGACTCCTCCTCCTTGTTGTTGAACGCCATCCATACTTGCATTACACACCCGCGCGTCGTCGTCTCGTTTAATGTGTTC

>AAUM1016

GACAGGTGCTTATCATGCATCACACAAATGCATCAATGTGTATAACAAGGAACATGCATGCCCACATGTCTAGTCTGGTATAACAAAAGCCAGCCTTCAGATCTGCTACAATTAATGTATGCTTGAAATCAACATCTTGCCTGGCAGTTTCCTACAAAACATTATGCTGCTGGTGAACTGTCCAAAAACTGCTTACTCTAATGCTGTTCAGTATGAATAAGTTTACACTGAAAGTTGTGGTTGTGATAGCAACAGTTCATTAACAATGGAACTACTAGTAGTCTGTAAATAGTAACGTCTGATATGAAAATTGAGCACATACTTTAGATTACTGCTCTAAGAGTATTTATTCAACAAGATAAATGTTGTACAAGCAGGTGGGGGAAACTTAAAACGTGTATATAGCACTCAATAGATACATTTATCACAACGCATAATATTATATGGTTTGACTGGGAAAACCATCACTGGCAACTGTTCGACACATTGCATACGTATAGTATTTAGTTAGTCTGAAACATTGTTGTTCTTCTTGTGGTGATGCCCTTTCTTGTGCTTTGGTGCCTTGAGTTCGCATGGAAAGCTGGCACAGGACCAGTTCCCACCAGCACAGACACAGTTACTGCAGTCGATCATTATTTCATCACCATCTTTATAGTACCGGTCCTCCAAAGGGCAGCTCTTGATAGGTGGACGGTACGATGGCCTCATGAAATTTTGGAACTCCCGCCTTGAGAGTAGCCAATCTGAATTTCTGTCAGCAACCTCCACCATGGCATCGATACACAGTGTAGCTGCCAGTTCACTTCTATGTTTCAGCCATGTGTGGAACATGGTGTTGTTCCTCCGGACACACTTTGAAAGCTCATGAGTGCTGAGGAAATCGTCACCGTCATGGTCACAGTCATCAAACACATCGTCAACCACTCTTGGGTAATGCCTGTCATGAGAGACCTTAGATTCGAGAAGCTCCACAAAGTGCCTTCGAATGCGATCCCTCTCATGTTGGAAGCAAACAACTGGCTTTTGGTGATACTTGTGATGTGGCAACTTGACCTTGGGCTTTTTGCAGTGTCCCTTGTGCTTGATGGATATGTGCTTTTGGCCCAGGCAGGCGTCTTTGTGAAGCATACAGTGGTTGTCGTAAGTCACTCCATTAGTACCGCATACAGGCTTGTGGTGGGATGGGCAGTGCTGAACACACTGGCACGACGCCACTCCATTGTCCAAGATCCGACACACCCTTCCGGGCCGACATATAACGGCGGCGCAGATGTCCAGCTGACTATCTGTGATCTCATCTTTTACCGGCACCGATGTGACGCTTGCCCAACCAGCAGCGAAAAGCAACCACGCACTCAAGAACTTCATTGTAGGTGTTGCCAAGTGCTTCCGACGCGAGTGACGCCGCTCACGCGAACGGCAGTAATCAAATTGAGCTTGGCTCGGAAATACGATCGCACGATCCCGCAGCGGAAACCATGCCTTCGCGTATCCGTTTCAA

>AAUM21551

TTTTTTTCGAAAAAGTTTATTTGAATCTCGTAAAATGGCAACCAAACAAAAATGAAAACAATGAGGACAAGGTGACGATCACGAGCAGAAGCTTTTTGGCATGACGACGATATTCCGGCCGCAGTTTCTCTGTTGCATGCGGCAGTGGTTTAGGTAGCGTTGTCCGTCCGATCCGCACACGGGGTCCAGGATGGGCAGGCATCGCTTGGGGCACTTGGGAGGCTCCAGGCACCGGTTCAGCGGCACCAGCTGAACGTGCTCCTTGCTCCCGCAGGTCTCCTTCTTGAAGAAGCACTCGTTCAGGTACACCTCGCCGTTGGACCCGCACACGGGCTCGTACAGCTCCGAGCAGTCCTGGGGACACGAGTCCAGCTTGCGGGCCTCTCTGGCGCTGGCTATGCAGACAGCCAGTGTTTGCGTTCCGACGACCTTCCCGCAATTTCGTCGCTGCATGATGCACAGGTTGGGGTAGAAGCGGCCGTCCTTTCCGCACACCGGGTCGTAGATGTCGAGGCACTTGTCCGGGCAGAGCGGGTGGCGGCCGCGGCAGCGCTCCCACTCCACCACCCGAACGGGCTCCAGGGGACCCCCGCGCTGCCGCCGCTGTTTCCGGCAGTTGGCGTTCCTCAGCGAGCATTCGCTGTTGTACACCCGGCCGTTGGACCCGCACACCGGAACCGGAAACGATTCATTGCAGGGCATTCCGCAGGCAGAGACGCCGGGACT

>AAUM39948

CGTACAAGAGGCGACTGCACGAGCGAGATCTGCCGTGCGAGGCCAAGTACTGCCCCTGGAACGGGGACTGCAGCGAGGACCGGCTGGGCGGCGCCCGCTGCATCTGCCCGTCTTCGTGCGGCTTCGAGTCGGCGCCCGTGTGCGCCCTGGACGGGGTCACGTACTCCAACCGCTGCAAGATGCGCCTCGACTCATGCCGCAAGCAGAAGAGGCTGTGGAGCCGCCACGAGGGCGCCTGCCAGGCCACCCCTCCGTACGGGTCGCCCTACTGACCGCCAGGCGGCCGGTAAAGGTCGCGT

>AAFM1692

AAGTGAAGGAACACATTAAACGAGACGACGACGCGCGGGTGTGTAATGCAAGTATGGATGGCGTTCAACAACAAGGAGGAGGAGTCAACAACAAGGACGAGGGGCGGCCTAGGGCAGCACGTTGAGCTTGGCGGCGGCCGAGGCCTCTCCGTTCTCGTTGGCGCCCACGCACCAGTAGGTGGCCGTGTCCTCTGGCCTCAGGTCGAGCAGCTGCANNNNNNNNNNNNGGCCTCAGGTCGAGCAGCTGCAGCCAGCTGGTCACCTCGAAGCTGTCCGGTCCGCCGCGGCTCTGCACGTTGATGCGGGAGCTGTCCGTGGGCAGCGACTTGAGCGGTCCGTCCCCGCGGTCGACGCGCCACTCGATGGTGGGCACCGGGAAGCCGGACACCTCGCAGGTCATGGCGGCGCGGCCACCCGTCCGGTTGCGCGTGTTCTCCGGAGCGGTNNNNNNNNNNNNNNNNNTGCGCGTGTTCTCCGGAGCGGTCACCACTCGGGGCGCGGAGTAGCAGGGTCCCCTGGAGGCCGCCTCGAGCCCGTTGCGCAGCCGGTACCGGGCCTCGGTCAGCTGGCACACGTTGTCGTAGGTGACGCCGTCGGTGCCGCACACGGGCTCCTGGCGCGAGCACACGCACAGCGCTTCGGCGGGGTCTCCGGGGGCCAGGTCGCGGCGCAGCCGGCACTCCAGGTCCTCGCCGCACGGCCCGTCCACGAGGCTGCCCTTCACCGACCGGTGGTAGCAGCGCCGGCCCTCGCGCTGACCGCACACCATGCAGCAGCCGCACGCGTCTTGAACGATGCCGGCCAG

>AAFM3034

CTTACCCTCGGCGTATTCTTTTTTCTCTCTCAATCTCGGCACGGGCAGCCCGACTGCCGCTACAGGAGGAGAGAGCCGTTTGCCGGCCGTCTGCCGGAGTACGTCGTCGTCCGTATCGGAAGGAACATGTCTACCGGTCCGAATCCTGTCTGCCGCCGCTGCTCCAGCATCGCGCTCCTTCTGCTCATCAGTCATGCAGCGAAAGCTGTGCCTACGACCGCGCTGCCACCGACGGTAGAAGGAGACAGCCAGCAGCCAGGTTCCAACGCAGTCAGCAGCGCGGGCACAGCTACAGAGCAGCCATTGTTACCTGTGGATCAACCACGAATTGATGGCAGTCCCGGCGTCTCTGCCTGCGGAATGCCCTGCAATGAATCGTTTCCGGTTCCGGTGTGCGGGTCCAACGGCCGGGTGTACAACAGCGAATGCTCGCTGAGGAACGCCAACTGCCGGAAACAGCGGCGGCAGCGCGGGGGTCCCCTGGAGCCCGTTCGGGTGGTGGAGTGGGAGCGCTGCCGCGGCCGCCACCCGCTCTGCCCGGACAAGTGCCTCGACATCTACGACCCGGTGTGCGGAAAGGACGGCCGCTTCTACCCCAACCTGTGCATCATGCAGCGACGAAATTGCGGGAAGGTCGTCGGAACGCAAACACTGGCTGTCTGCATAGCCAGCGCCAGAGAGGCCCGCAAGCTGGACTCGTGCCCCCAGGACTGCTCGGAGCTGTACGAGCCCGTGTGCGGGTCCAACGGCGAGGTGTACCTGAACGAGTGCTTCTTCAAGAAGGAGACCTGCGGGAGCAAGGAGCACGTTCAGATGGTGCCGCTGAACCGGTGCCTGGAGCCTCCCAAGTGCCCCAAGCGATGCCTGCCCATCCTGGACCCCGTGTGCGGATCGGACGGACAACGCTACCTAAACCACTGCCGCATGCAACAGAGAAACTGCGGCCGGAATATCGTCGTCATGCCAAAAAGCTTCTGCTCGTGATCGTCACCTTGTCCTCATTGTTTTCATTTTTGTTTGGTTGCCATTTTACGAGATTCAAATAAACTTTTTCGAAAAAAA

>AAFM5095

ACAATGGAACTACTAGTAGTCTGTAAATAGTAACGTCTGATATGAAAATTGAGCACATACTTTAGATTACTGCTCTAAGAGTATTTATTCAACAAGATAAATGTTGTACAAGCAGGTGGGGAAACTTAAAACGTGTATATAGCACTCAATAGATGCATTTATCACAACGCATAATATTATATGGTTTGACTGGGAAAACCATCACTGGCAACTGTTCGACACATTGCATACGTATAGTATTTAGTTAGTCTGAAACATTGTTGTTCTTCTTGTGGTGATGCCCTTTCTTGTGCTTTGGTGCCTTGAGTTCACATGGAAAGCTGGCACAAGACCAGTTCCCACCAGCACAGACACAGTTACTGCAGTCGATCATTATTTCATCACCATCTTTATAGTACCGGTCCTCCAAAGGGCAGCTCTTGATAGGTGGACGGTACGATGGCCTCATGAAATTTTGGAACTCCCGCCTTGAGAGTAGCCAATCTGAATTTCTGTCAGCAACCTCCACCATGGCATCGATACACAGTGTAGCTGCCAGTTCACTTCTATGTTTCAGCCATGTGTGGAACATGGTGTTGTTCCTCCGGACACACTTTGAAAGCTCATGAGTGCTGAGGAAATCGTCACCGTCATGGTCACAGTCATCAAACACATCGTCAACCACTCTTGGGTAATGCCTGTCGTGAGAGACCTTAGATTCGAGAAGCTCCACAAAGTGCCTTCGAATGCGATCCCTCTCATGTTGGAAGCAAACAACTGGCTTTTGGTGATACTTGTGATGTGGCAACTTGACCTTGGGCTTTTTGCAGTGTCCCTTGTGCTTGATGGATATGTGCTTTTGGCCCAGGCAGGCGTCTTTGTGAAGCATACAGTGGTTGTCGTAAGTCACTCCATTAGTACCGCATACAGGCTTGTGGTGGGATGGGCAGTGCTGAACACACTGGCACGACGCCACTCCATTGTCCAAGATCCGACACACCCTTCCGGGCCGACATATAACGGCGGCGCAGATGTCCAGCTGACTATCTGTGATCTCATCTTTTACCGGCACCGATGTGACGCTTGCCCAACCAGCAGCGAAAAGCAACCACGCACTCAAGAACTTCATTGTAGGTGTTGCCAAGTGCCTCCGACGCGAGTGACGCCGCTCACGCGAACGGCAGTAATCAAATTGAGCTTGGCTCGGAAATACGATCGCACGATCCCGCAGCGGAAACCATGCCTTCGCGTATCCGTTTCAAAG

>AAFM8150

TCAGCGGCGCCGGTATGGTGCCTGCAAGGACCACGAACTCCAAAAATGGAGGCACCCATCTCAGTCACGCAGCGACCCAAGAGGCGGATCATGGCCCACTGTATTCCCAGGGCAAAGGATTTCTGTTTGGCCGTGACGCACCTAATGGTGACGCTTATGCCGGGTGCGCTGATGTAGAAGGTGCTGAATAGGCAGATGAAGGTGCCAAGCGTGTAGAAGAGGTAGGCGGAGCATTCCTGTGTGCAGCGCACCCTCTCGGCCGCCACCACAAACTCGGTCGAGCTGTTGTCGCTTGACTCTGTCGGGATGCAATCACAGTTGCCGTACACCGTGACGGCCTCCTCATTTTGGGCGGCGTTTCGGCAGCCGGCCAAACACGGAGACATGTACATCTTGAAGTCCCGGCCGCAGATGGGGTTGAAGGTGAGCGGGCAGCCGCACTCGCTGGTGCACTTGGGTGTCAGCACAAGCTTCTTCGTGATGTCGATGCCCTCGTAGTGCAGGTTGGGGCAGGAGACGAGCAGCATGAAGAAGACGAACCACGTGACCACGGAGACGACGATGCACATGCGTATGATGTTGGCGCAGCTCAGGTCGAGCTTGGAGACCACGTAGCCGCCCAGGAAGCAGCCCAGGCTGGCACTCGGGATAGCGATCATGCCCAGCAGCAGGGCTGCCGAGGCCGCGGTCATGCCGAACTGGTTCTCGAACAGCTTGGTGGCGAAGTTGGAGTAGCCAGAGCCAATCATCGTTTCGACGGTGGCGGCCAGGCTGATGAAGACGAACGTGTTGTTCCTGAGCAGGTGGTAGACAGCCTTGGGCATGTCGGAGAGCCGCAGCCGGACCGCGTCGTGCTCGGCTTCCAAGGCGCGCGTGTCCACCTCGACGCGCTTCTGGGCCAACATCTCAGCGGTGCCGGGCAGCGTCTTGGGGAAGCCGGAGATGGGCACGCTGACAAGGATCGCCGAGCAGCCGGTGATGAGGAAGCCGATCCACCAAGCACCGATCCACACGTTGCTCGAAGGCGACAGGCCGAGGCTCCTGGGGTCAACGGTGATGTCGGTGTAGACGTTGAGCAGCATGCCGCCCAAGATGAAGCCCACGGCTGGCCCGATGATGGCTGCTGTGTAGTAGATGCCGATGTACCAGCTGGACTTTCGAGCCGTCAGGTTATCGTCCAGGTACGCAATGGCGAGCGTGTAGAAGGGCACGGTGGAGCAGCCGTGGACCAGGTTGGCCACCATGAAGACGAAGCGGTAATTTCGGAGCGCGCGGCTGCCCTCGGGGTTGATGATGGTCGCCTTGTCTTCCTCGAAAGGGCACAGGTCCTTCACCTCCTGGCTGTACTTGTACTGGGGCGCCACGAAGTGCGGCAGGGTGAAAAGGAAGGAGCCGACGCCCATGAAGAAGACGCCGTTGGCCATGATGCGCGGCTTGTGCATCTCGCTCCCGAAGAAGGTGACCGGCGTCATGAGCATCAGCGAGCCCACGTTGTACATGGACACGATGAGGCCGGCCTCGACGCTCCGCAGGTGGAAGCGCCGCTCGAGCGTGGGCAGCGACACGTTGATGAAGCCGCTGATGACCATGCCCTGCACGAAGCCGGCCACGCAGAAGCACACCAGCACCCACTCGGCCGACGCGAACCTCTGCAGTGGGGCCGGATGCCAACCCCACCAGCCGCAGTGCGTGTCCGCTTCGCTCCCCTCCCGCGCCTGCCTACTCCGCAGCATGGCGTCGGACGTCCCACAGTGCTGCTTCGACCGGCCTTCCTCCTCT

>AAFM18135

GAGGTCACTTTGCTGGCAAGTTTAGCATTCCTCTTCCTTTCGGCACGCTTGCGGGCGTTCCGGCGGTAGACGATGAGCGACGAGTAGAAGAGGACCATGCTCGCCGCAAGCATGACGGCCAGAGCGTAGAACATCCCCCGCGCCATGCCCTCGTTCTGGTAGGCGATGCAGTTGCCACTCGAGCCGCAGGTCCGGTGCCACATCAGGCATGTCTGGTCCACGATGCCGCCCAATATCACCGGCGCCGGAATGGATCCCAGCAGGCGAATGATGATTGTGTTGATTCCAAGTGCCAGGGACCGCTCTGCGGGCTTGACGCACCTCATGGCGGCCGCGGTGGCAGGTGCCGCGTTGAGGAAGGCCGAGCTGAGCGACAGGAAGACGCCGACCAGGTACGGGAAGAGCAGCGCGCAGTCGACGCTGCAGCGAGACCGGGTCGCCTGCACGGCCTCCAGGAGGACCTGCTCGCTACTCACGCCCGGCACCTCGCTCAGCGTGCCGTTCACGCACGAGCAGTCGCTGTACATCTTGACGTCCTTGACCTGGAACTCTCTGCGGCAGCCCGCTAGGCATGGCGAGAGGTAAACTACGTCGTCAGCACCGCAGATCGGGTTAAGTATACTCGACGTGCAGTTGCAGTCACTGTTGCACTGGAAATCGAAAGTGGTGGGCCTCGGGGCCCCCACAAATCCCTCTGGTGTGGCATAGGTGTTCTCTTGGCAGTTGACCAGGAAGGTGAGCACGCCGAACCAGGAAAAGCAGCAGTTGTACAAGCAGAGTCGCATGATGCCCGCGTAGTCCAGGTTCCAGCGGCTCACCAGGGCGCCTCCAATTACGGCTCCGAAGCCACCGCCCACGAGCACGATAGGTCCGGTAAGGTAGGCTGACCTGGCGGACGGCATTCCCAACTGCGACTGGAAGAACTTCGTCAGCATGGACGTGATGCCTATGGCGAACATCTGGTTGAAGGACATGGCGAAGCAGAGCAGCATGAATGGCACGTTTGTGAGCAGCCGCCGGATGGCCTTGGGCAAGTCGGTGAGGCGGCGACCGAAGTCGCTCGTGGCCANNNNNNNNNNNNNNNNNNNNNNNNNNNNNNNNNNNNNNNNNNNNNNNNNNNNNNNNNNNNNNNNNNNNNNNNNNNNNTAGCCGGGCAGGTGCTTCGGGAAGCAGGCGGTGAGCAGGCCGAGAGTGAATGCCATGATTGCTGACACCAGGAAGCCCATCCACCAGGCACCAACCCAGGCGGGGCTTTTGGATGTCAAGCCGAGGGCAGAGACGTCGGTGGTGATGTCGACGTAGTAGGTGAGCGTGTAGCCAGCGA

>AAFM21772

CTGGCTACTTGCCTGATGTCAGTAGCCGCCAGACACAACAGTACATGTCTCTTTGGAGCGCGCTCCATGTTTGAACACGAAGAAAAGCGACCATGGCGAATCGTATTTTTTTTCTTTTCTTTCCGCCGCCTAATCCGGCACGGCACCGCACACACAAGCACGCACACTGTACAGGTTCGCCATGGAAGGAGCAGTCGTCAGTCAGGCAGCGCGGCGACGGAAACGGGCGGCGGCCGCGGCTCGCGGAGGAAGAAGAGCGCCAGGCCGAAGCAGCAGAGCGAGGCCGACTTCAGCGCCAAGATGAGGAAGTACACGTTGCGGTTGAGCAGTCGGTTGTCGTAGATGGCGCAGGCGCCCTGCGACGAGCCGCACGGCTCCTCCCACAGCACGCAGCTGGCGTCGATCGTGTGGCCGAACGCCGCGCCCGCCGGGATCAGCCCGAATGCCCGCACGATTGTCCATTGAATTCCGAGGCCGACTGACTTGTGTTGCTCAGGGATGCACCTGAGCGTGGCCGCCACGGCGGGCGTGCTGAGGAAGAAGGTGGCGAACACGATGGCAGCCAGGATGGGTATGAAGACGTTGACGAGGCCGCACGGATTGGGGCACTTGTTCCGCACCGCCTCGATCTCGACCATGGTGCCCGGCACGGTGCCGAACGGGCTGACGGAGGAGGATCCCGCTGCTGCTGTTGCGCCACTGCCTCCGGCCGTGTGGTTCACGCACAGGCACTGCGAGTACGCCCTGCTGTCGGCGGTGGCGTACTCGCGCAGGCACCCGGCGAAACAGGGCGAGTAGTAGGTGTACCCGTCGCGGCCGCATATGGGATTGAAGCGCGTCGTGGTGCAGCCGCAGCGGCTGTTGCACGCCTCGGTCAGCGACACGTTCACGCTAGACACGCTGGTGAGGCTGGTGTCGTACTGGACGTTCCGGTAGGGTATGTTGCCACAGTGGAAAAGCAGAACCGTGACCAGCGTGATGGGCGCAAGCAGCACGAGCGCGCACATGCGCACGATTCCCCTGGCGCCCAGCTCGTAGCGAGAGATGACTACGCCGCCCAGCATGACACCCACCATGGCTGTCGGTATGGTCACTGAGCCCATAATCATTGAAGCTGTCGACGCTGTCATGCTAAACTGCGCCTGCAACATCTTCACCAGGAATGAAGAC

>AAFM26894

GCCATACCGAGCGCCTGTGGTGGGACCCTCCTGGGGGGCTACCTCATTGACAAGCTTGACCTGACCTGCGCCAACATCATTCGTATGTCTGTCTTCGCCAGCATGTTCACCTGGCTCGTGATGGGCTTCGTCCTTTTCCACTGTCCGAATTCGTCCTTTGCGGGGATGTCCTTCACCAAAGGGTATCACTTCGACCCCAACGCAGCCTGCAACGCTCATTGCAAGTGCAGCGAAATACTCTACAGTCCAGTTTGCGGCGCGGACAACGTCACGTACTATTCTCCCTGCTTCGCGGGCTGCCGACGGGAGAACATCGTTCAAGGCACTGTGGTGTATTCCGACTGTAGCTGCATCGCCACCGTGCGCGAGAAGGACGTCCTGTCGGTGCAGGCCGAGCTGACGCGCTGCCAGACGACGTGCAAGGCGCTGGTGCCCTTCTCGGTGGGCCTCTTCTTGGCGCTCTTCGGCACCTTCCTCAACAGCGCCCCAGGCATGAGCGCCACCATTCGGACCGTCGGGGAGCCGACCAAGCCGGTGGCGTTGGGTCTGCAGTGGGTCTCGGTGCGGCTCTTCGGCACCATCTTGGCGCCCATCTTGTTTGGCTCCATCATCGACCGCAGCTGCCTGACGTGGCAGGGCCTGTGCGGCGGCCAGCGTGGCACCTGCATCATATACGAAAACAGCAGCATGAGCTACAACCTCTTCGGCTTCCTCATTTTTCTCAAGACGCTGTCGGNNNNCTTCTTCTTCGCCGCCTGGGTCACCATCCGCCACTGAACGCTGCGCATTATTGTA

>AAFM27624

CTTGCCACGGCCGGCTACGGCACCTCGTTCAGCAAGTACGTCGAGTTCCAGTTCAGGCAGTCGGCGTCCAAGGCTAACTACTACACAGGGGCAGCGAAAGTGGTGACNNNNCTGATCGGCATCGTCACCGGCGGCGTCGTGGTGCACCGGTTCCGACCACGGCCGCGCATCGTGGCAGGCTACTCAGCGTTCGTCGAGATCGCCATGATGGCGGGCTTCGTGGTCATGATGTTCATCGGCTGCGAAAGTCCCGTCATCGCGGGAGTCACTCCTGGTGGAAACGTCACCGCGAGTTCGCTGCTGGACACATGCAACGTGGACTGTAACTGCAACACGCAGATCTACGAGCCGGTGTGCTCAAGCAATAGGATGATCAGCTACTTCTCACCTTGCCATGCCGGCTGCAAAAGCGTGGGAACGACATCGTCCAACATGACGATATACAAAGACTGCAGCTGCGTCGCGCAAGGAAACCAAGGAGTGGACGACTCCTACGTGACCTCGGGGCTCTGTGGATCCTCGTGCCAGCAGCTGGGCCTCTTTCTGGGCATTGTGATCGCTGGCCAGTTCCTGGGCTCCACGGGCCGTGTAGGCGGTCTGCTGATCTTCCTGAGGTGCGTCGATCCTATTGATAAAAGCATGGCCCTGGGGACCACAGGTTCTCTGCTGAATATGCTTGCTTTCATCCCGTACCCTCTGGTGTATGGCGCCATCCTGGACAACTCGTGCATTGTGTGGGAGGAGAAATGTGGCCGGCGCGGCAACTGCTGGGTTTACGATGCGGACAAGCTGCGCTATTCGCTGCATCTCATCACGGTCATCTTCTTAGCCATAGCTGGTGCCTGCTACATTGGCCAGTTCCTGCTGAGCGGGCGCATAAAGCGCTTCTACGAGGACGACCTTGCTGAGGACAGCACCGATGATCAGGACAAGTACGAGAGCAAGACTACCATCGCCGAGGCGCTCTCGGTTGAGAGCATCCAAAAGGCCGACGAGCTGCGAGAGGCCACCAGATTTTGAATCCCAGTGCAGCTTAACATAAATGCTCTGGCGAATTCAATGACGACAGAAAGTGAGCTCCAACCGCGAAGTTTCAGGGCGAAGTGAAGACACTCTATACACTAGAGACGGAAAGTTTCCACAGATTCCAACGACAATGTCTTCGTCCTGGTCTGGATGTGCTCCGCGCTGACAGACAGGTTGCCAGATGCAACGTGCTTTTGAACGCCAGGATGGGTTCAAGGAGAGACAGAACACTGATGTGGGTTTCTCGACAACGAAAGAAAAAGTAGGGGAAGCAGTGGCTTATTACGAGACAGTGAGTACGAAGAGTGTCAGAGGTCCCCTGCGAGCTACAGGGACGTTGAATTTATCCTTTAGTGAATGTGTTTCGCGTAGACATCCTGATCTTGTCAGCAT

>AAFM39139

GTTCAACGTCACGAGCTTCCTGTTGTCGGGCGTGGTGATCCACCGACTGAGGCCGCCGCCCAAGGTGCTCGCCTGGTACAATGTGGTCGTCACCTTCGTCGTCTCCTGCGGATTCGTCGTGGCGATGCTCGTCAAGTGCGACTACGGAACCATGCCCGGGGTATCGGTCGTCCAAGGGAACCTTGACCTGAACAACAAGTGCAACGAAGAGTGTTACTGCACCCTTCAGAGTTATCAGCCCGTGTGCGAACCCGTCGGAGGCACCGTATACTTCTCTCCGTGCTTTGCTGGATGCCAAAAGCCCGAGGGAGAGGTGGGAGCTAACTTGACGAAGCTGACCAACTGCAACTGCCTGAAGACGTTCGACGACAGCGACTTCTTCTCAGGAAACGCCGTGGTTGGGTTCTGCAAGGGCACCTGCTCCATGTTCGTGCAGTTCATCATCATAGTCTCGCTGGTGCAGTTCATGGGCATGTCCACGTCCGTGAGCCACACGCTCTTCATGCTTCGGAGCATAAGCCCAAGTGACAAGACCATCGCCCTTGGACTGGCCAATGCTCTGGCGAATTTGCTGTCGTACATCCCTTACCCGCTCATCTACGGCGCCGTGATCGACAGCTCTTGCCAGGTGTGGGAGAGCGCCTGCGGAACGAGCGGCAACTGCTGGCTGTACGACCTAACGCGATTGCGCCACTCGTACCTGGGCACGTCGGCCGGCTTCCTGGCCGTGTCGGGCATCTTCTCGATCGCCGTCGCCCTGGTGGCGGGTGACCTGAAGGACTTCTACGGGGACGCCTACGTACAGGTGTCCCACTTGGGTGGCGCTGACTTCGAAGCACGGAACATCGGCGTCGAGAAACGCAATAAGACTGTCGCCAAACAGGAGAGTAACGGTCACCTCCCCAAGTGACTGCCGGGAGGCCCGACGGTGTAGCACGTTGTCGGATACGTGTTACTGTTATTCCATTTATGGACGTTAACCAAAACTTTTTCGAGGTTCTTCGACAGTCTAGTGCTAGCAGCCGCTGGAGCCAATCAGCTTCTGTGGCGTCTTGCAGCGGAAAGCTTCCCGCGGC

>AAFM41940

CGAGATCGTGTCTACGAAGATGTCCTCTGTGTACATTGGTATTTTCTATACCATGGCGATCATCGGCCCTGCACTTGGCTATATTCTAGGCGGCCAATTTCTGAAGATATACACCGACCTCTCCGTAGATGCTTCAGCATTAGGCTTGACGCCCTCCAGTGGTGTCTGGGTGGGCGCCTGGTGGATANNNNNNNNNNNNTCCTCCGTAACGGCTCTTCTTGCTGCCGTCCCCATTAGTGCCTTCCCCAAGGTCCTCCCAGGTTCATTAAAGCTGCAGGCTCAGAAGAAATCCGAGATGCACCAGAAGCTTCAGAAGAGCGAAGCGGTGCAGAGCGGCTTCGGGGCCCGCGCCAAGGATCTCCCGGCATCCTTCAAGATACTCATCACCAACCCGACCTTTGTGTTCCTAAGCTTGGCCGGAGCCACCGAAGGTATGCTGGTATCTGGCCTGGCGACATTCCTTCCTAAAGTCATTGAGTTCCAGTTCAGCATCGCAGCCAGTTTAGCGGCCCTAATAATGGGTGCGGTGACAGTTCCGGGCGCTGGTGGTGGCACATTCCTAGGCGGCTACTTCGTGAAGAAGTTCAACCTGCGCTGCGCCGGCATCATCAAGATGTGCGTCCTCTGCTCTCTTGTTCCACTCGTCACCATCTTCGCATTCTTCTTCAGCTGTCCCAACGTCAGATTCGCGGGCGTCAATTACAAGACGAACAACGTCACCGATGGAATAGACCGCTTCCTGTTAGACTGCAATAATCAGTGTCACTGCCAAATGGAAGACTTTGACCCCATCTGCGGAACGGACCACGTCATGTACTATTCCGCATGCTTTGCAGGTTGCCAAGAGGTGCACCACTATGGATCAACAAAAGTGTACGAGGACTGCCGCTGCATTGATCATCCGGGCAGAAACGTAACAATTAATGGGAAGCAAGTGACCATCCAGGCGGAGAGAGACAAATGTCCAAGCGAATGCAACTTCCTCGTCTTCTTCCTTATCGCCATGTTCGTCTGCATGGTCTTCACATTCCTGGTCAGCATGCCTTCGCTGGCCGCCACGCTCAGGTGTGTTGCAGCTAGCCAGAAGTCTTTCGGTCTCGGCATACAGTGGATTGCTGTGCGGCTACTAGGGACCATACCGGCGCCCATCATGTTCGGCT

>AAFM58501

TCCGCCTGCACCTTCTCCGGGAGGCCCCACAGCACCATCGTCTCCACGCCCGGGTGCGAGATGCACGGGCAGTCGGTGTACACCTTGAAGCTTCCGTAGATGTAGTCCCTCTTGCAGCCGGCGAAGCAGGGCGAGTAGTAGGTGAGCATGTCCTTGCCGCACACCGGGTCGTAGTTCTCGATGGGGCACGAGCAGTGCGAGTTGCAGGGCTGGTCGAACTGGTTGGACACGTTGGACACGTAGAAGCGGCTCGACGTGTGGTTCATGCCGAAGAAGGCCGGGCTCGGGCAGCTGTAGAGCAGCACGAAGGTGCTCAGCCACGGCACCACCGAGCACATGAGGCACATGCGCACGATGCCGTCCACGTCCAGGTTCAGCTTGTGCACGCAGTAGCCGCCCAGCAGGATGCCGCCGCACGCGCCCGGAACGGCCACGCTGCCCAGTATGTGAGCGATGTTGGAGGAGGTCAAGCCGAGCTGGGACTCGAAGAGCTTCGTCATGAACGTCGCCAGGCCGGTCATTATCATGGTCTCCGCTGTGCCGGCAAGGGTGAGGAACATGTACGTAGGATTATTAAGCAAGGCGCCGACATTGGCTGGGATGTCCTTGAAGCCGTCGTGCACCTCCTTGGTCTTCTGGCCTTTCTTGGCGACGGCGTCGCATTTCTC

>AAFM22140

CCGATGCCGGTAGATTTGACGTCCTCGTCGATGGACCTCAACAGCGCCGTCAGTGCGGGCACGATGAGCAGGAAGGTGAAGAAGAGAGCTATGAAGATAGCACCCGCATACACGAGCACGAGGTTGCAGTCAGGGGAGCAAGGATCCCGCTTAGCCATGTAGTAGAAAAGGGTGTCGTTGTCGAGCGGCGTGCTCAGTGTAGGCCCGTTCACGCAGGTGCACTGCGTATAGAGCTTGGTCTTCTTCGATCGTTTCTCGTCAGCGCAGCCGGCGATGCAGGGCGAGTAGTA

>AAFM39753

CACCGTCGCTGGCCACGGGTCGTCAATACAGCTGGCTTGCAGTGATAGCTGCAACTGCACCACCAGGCACTTCCAGCCGCTTTGCGACCCAGTCAACCGTACTGTTTTCTTCTCTCCATGCCATGCGGGCTGCACCAAGTTTTCCGCTACAGATTCTGGGGAAGTGTCCTTCGACGAGTGCTCGTGCTTGCCCAACCTGCCCAGCCAACTACAGCGGGGCCAGCAAAAGGAGCAGCTTGTGCAAGCCGACCAAGGGGCTCCGTACTTAGGGCTTTGCAGC

>AAFM49846

CTGGAAAATTGCAACCAAGTTTTGGTTGAATCGGCTGCGGTCAACGCTTGAGCAACCTATCTTGGCCTGACCCTGGTGAGAGGCCCTGCGCTGGAACGAGCCTTGTCGCCGCGTGGTACAGTTGGGCTGCTGGAGGTGCATGGAGGTTCCCGCCAGGGGCCGGCCCTGGTCATCCACACACCAGCAGTATCCCGTCATGTGATGACATTGGACACGCTTGAAGCCTCCATCCGGTGCGCATTCTGGCACAAACAAGTCGGACTGCTGCTGCTTGCGCTGGAGCAGGCATCGTGCTGCAGTTGCGGCTGGCGTCGGCTCAGAGCACGGTCCCACATGGCTCAATTCCAAAGAGTGCTCCTCACACTGGGCACGCTCAAAGTCGCACCGTGTGGCATATGTGCGCCCATCCGAGCCACAGACAGGCTCTCCTCCGGTGCATGCCTTTGTTGACGGTGGACATCCTTCATTTTCACTGGTCGAGTGGTCGCTCCTCCCCTTCGTTCTTCACGATCGTACATTAAACTACAGGAAAGGG

>AAFM43046

CCGCTGTGCGCGGAAAAAAGGGGTTCTCGTCCGCTGCATCTAAGCCCTTTTTTCTTTTCTTTCGCAGCCTTTTTTTCTCGGCCGAATCGGTACGTGCCACTGGCCAGTCCAAACTACTGCGCGTAGCTGAGGCGACAGCGAGATTTGGAGAACCGCGCCGACGAGCACCTATGTGAAGGGTAGCGAGTCGGTGCGCTCAGCTCGTCGCATCCGCCGTTTTGATCCGGTGTTGGTGAGGTTGCGAAAGCTTTAGGCCTAAACTTGCAGCCGATCGGCGCTAGGAAGGAGCAATGCATACTGACAAGAAGGCGTGGCTCTGGGTAGTTGTTTTGGCGTTCCTTCTTTGCACGGCGTATTCAGCTACCATCAAGGACAAATCTAAAGACTCCGAGGAGTGGGNNNNNNNNNNCGAAGAGCACTCTCGAGATGAGTCTTCCNNNNNNNNNNNNNNNNNAAAAGATCTCAAGGCGACGTATAATGCTTGGTCCTTCGCCGATGCCGACCTTTGCCGCACTGTCCAATGCAAGCCGACCCAGGAGTGCCTGATTCAAGATGAAAACACAGCAATCTGTGTCAGTGCAAAGAAGGTCAAGAAGATGAAGAAGGCATCTCTGTCTGGCAGCTGGGGCAGCTACGATAGACAGCAGAGCACAGCTATGCCACTTACCACGACTGCAGCTGGCACTGATAGTGCTGCTGCAGTGAGCAGCACAGCCAAGCCGTGGAGCAGTGCAGAGGCAGAAGACGATGACGATGATGACCAGTACCTGGAAGATGACAGCAGCCAGGAAATGGAACGGTGCCCACCGTGCCCCGTGTTCAAGGCGGACTATGTGTGCGGCACGGACAACCAGACTTATTCTTCTGAGTGTCGGCTGCAGTACCACAACTGCCGCCAGCGTACCTCAGTGCAGGTCTCCTGCAAGGGATTCTGCCCGTGCAAAGCTGTGAAAAGCCTAGAAGAACAGCAGCGTGAGAGCCGTCAGAAAGCACG

>AAFF6116

GGCACCATGTGGAATATCTCGTTCCTCGTCAGTTTCCTGAAGGAGCCCTTCGATGACCTCCTGGACGAAGAGTGGAGGTCCGCCTCTCCCGGCGGCGGTACACGGCTCCTGTCGGCCAAGAAGCGCTTCTCTCGGCCGTAGAGGTTGCCCAGCCGACCGCTCAGCAGAAAGACTACGAAATTCAGTGCCACGCCAACCATGAGCAGGACGAAGGTGGTGCCGTGCAGCGAGTAGTTGAAGGTGCGCTGGTCGTACACCCAGCAGTTTCCCCGCTGGCCGCAGCTTTCCTCCCACACCACGCACGTCGAGTCCACCAGTGAGCCGTACACCAACGGGTAGGGCAGGAACGAGAACAGTGCAAGGACTCCTTCAGCGAATCCGAGTGCAAATGCCTTGTCCTGCTGATCAACGCATCTGAGTGGAATGACAATCTGTGCCGCCTTCGTGCTGAAGGAGAACCCTAAGGCGATGCACAAAAGCATAATGTAGAAGGTCAGGAAAGGACAGTCGCTTTCGCAGAACCCGTCTTTAGCCCTCCGAGCCGGACCGTCATTGTCAAAGGCGTCCACGCACTTGCAGTTGTCGTATGTCCCTCTCTCTGCTGTGCCGTTCGGTCCTTTGGTGGCGCATCCGGCAAAGCAAGGAGAAAAATACGTAGTCTTTCCCTCGCATATGGGATGGAACACCTCGGTAGCGCAGGAGCAGTTTTGGTTGCAGCTGTTGACCAGGTCTAATCTTGATCGGAGTTCGCCTTTTTCTCTCAGGGGAGGCTGTGGGCAGCTGAAAGCCATCAGTGAGATAAATATTAGAGCTGTGATAAGCTCCGTAGCGAAGATGATGGCTGTTAGTATGCGCGCGGAAGGCCGAAACGTCTTCATCAAGAACGCTCCACCCAGTGTGGCTACCATGGCACACCCGATCGGGAACGTTCCACTTAGGACTGAAGCAGCACTGGGTGACTGTTGGAAATGCTCTTGCACGTATTTAGGCACGTACGTACAAAAACCCAGGACACCGAACCACTTGAAGACGTGAGCGCCCATCTGAGCGACTATTATGGGGTTCTTGATAAGTCTCTTGGACGCTTCGAACGCTTCTCTTAGGCTCATGTCTTGCTCTTCCTCAGTCGGCTGGTC

>AAFF21957

CCCTCCAGTGGTGTCTGGGTGGGCGCCTGGTGGATTGGTTTCGTCGTGTCCTCCGTAACGGCTCTTCTTGCTGCCGTCCCCATTAGTGCCTTCCCCAAGGTCCTCCCAGGTTCATTAAAGCTGCAGGCTCAGAAGAAATCCGAGATGCACCAGAAGCTTCAGAAGAGCGAAGCGGTGCAAAGCGGCTTCGGGGCCCGCGCCAAGGATCTCCCGGCATCCTTCAAGATACTCATCACCAACCCGACCTTTGTGTTCCTAAGCTTGGCCGGAGCCACCGAAGGTATGCTGGTATCTGGCCTGGCGACATTCCTTCCTAAAGTCATTGAGTTCCAGTTCAGCATCGCGGCCAGTTTAGCGGCCCTAATAATGGGTGCGGTGACAGTTCCGGGCGCTGGTGGTGGCACATTCCTAGGCGGCTACTTCGTGAAGAAGTTCAACCTGCGCTGCGCCGGCATCATCAAGATGTGCGTCCTCTGCTCTCTCGTTCCACTCGTCACCATCTTCGCATTCTTCTTCAGCTGTCCCAACGTCAGATTCGCGGGCGTCAATTACAAGACGAACAACGTCACCGATGGAATAGACCGCTTCCTGTTAGACTGCAATAATCAGTGTCACTGCCAAATGGAAGACTTTGACCCCATCTGCGGAACGGACCACGTCATGTACTATTCCGCATGCTTTGCAGGTTGCCAAGAGGTGCACCACTATGGATCAACAAAAGTGTACGAGGACTGCCGCTGCATTGATCATCCGGGCAGAAACGTAACAATTAATGGGAAGCAAGTGACCATCCAGGCGGAGAGAGACAAATGTCCAAGCGAATGCAACTTCCTCGTCTTCTTCCTTATCGCCATGTTCGTCTGCATGGTCTTCACATTCCTGGTCAGCATGCCTTCGCTGGCCGCCACGCTCAGGTGTGTTGCAGCTAGCCAGAAGTCTTTCGGTCTCGGCATACAGTGGATTGCTGTGCGGCTACTAGGGACCATACCAGCGCCCATCATGTTCGGCTACCTGATCGACCGGAGCTGCGTGCTGTGGCCCGGCTCGTGCGATGACTCCGGCGCCTGCGCCGTCTACGAGAATGGCCAGATGGCGCGCAACCTGCTCGCGCTGCTTGCCACCGTCAAGTTCCTCTCCTGTCTCTTCTTCTTCCTCTCGTGGCTGCTCTACAAGGCGCCCGAGGGCGAGGACGAAGAGGACGACGGCAACCAGGCTGCGGCGCCTGAGAAGTCTGGCGTCGCCACCATCAAGCAGGAGACTGTTGTGACTGGTGCTCCGTTTAAGACGGCCACGAACGGTGCAGCCGCCGGACAGCAGCAGAATCACAGCAGTGACAACAACAGCACGTGGTTCTGAACAGTGCAAAGAAACAGAGTGCAAGTTGTATTTGAGGTCATTTGCATCGGGGCCCAGCGATGCTCGCCGTGCTGGTGTGCAAGGAGTGGTGTAGTTCTTCGCGGTTCCACGGTTCTTGGCTGTTCGCAGCTGCGAGA

>AAFF38633

CCACCAGGCACTCCCAGCCGCTTTGCGACCCAGTCAACCGTACTGTTTTCTTCTCTCCGTGCCATGCGGGCTGCACCAAGTTTTCCGCTACAGATTCTGGGGAAGTGTCCTTCGACGAGTGCTCGTGCTTGCCCAACCTGCCCAGCCAACTACAGCGGGGCCAGCAAAAGGAGCAGCTTGTGCAAGCCGACCAAGGGGCTCCGTACTTAGGCCTTTGCAGCGGGGACACGTGCAGCAACGTGCTGGCATTCATCGCGCTGTCGTCGCTGGTCGGCTTCATCGCGCGAACCACAACTGTGGGACACACTATCGTCGGGCTCAGGTGCGTGTCCCACGAGGAGAAAGCCATGGCACTTGGCGTCCA

>AAFF42567

TCGATAAGAGAGGAAGTCAAAGATGCCGTTCGAGTTTTGAGGAGACTCACAAGAAACCCGATCTACGTATTCAGGACGCTGGGAAACATAGCAGTGTATATGGCCCTGACTGGTTACTACGTCTCCTTCCCAAAATACACGCAGCACCAGTTCCATCAGACTGCTTCAAGGGCCAGTCTGTTCACAGGGCCCGTCGTGATTGTGTCGAACATGGTGGGAACCCTGGCGGGGGCTGTGTTCGTTCACCGTTGGCAGCCTCGGCCACGGATCATCGCCTGGCATAACGTACTCGTCACACTCCTGGCCGTGCTGGGCATCTCGGCGCTCATGGCTGTAGACTGCGGCACCCTCCGCTACCCAATCGCAGCACACCAGGAAACCGGAAGCAGTACCGTAGAAAACGCTTGCACGCGCGAATGCTCGTGCTCTCCGGACGTGCACCGCCCCGTGTGCGACGAGTCAACGGGGATGCAGTACTTCTCCCCGTGCTTTGCCGGGTGCAGCTCACAATTGGGAAACGAGAGCGAATTTAAAGACTGCCGCTGTTTGGTGAATGAAAGGGAGACAGATAACTTCTACAGCGGGGTCGTCGCGTCCGGCAAATGCGAGCAGGACTGCTCAAGCGCCCTGACGGCGTTCTCGGTGGTTGTGTTTGTCATCCA

>AAFF44878

TCCAAACATTATTGGAGCTGGTATGTAACCAAATAGCCTAAATATTACAAACTGCATTCCCAATGCAAATGACCTCTCTTCTTCTCCAACAGATCGCAGTATTATCATAAGCAGTGGCATCTGCGTGATGGAGACCACCAGTGTCATGGCGAACAGGAGCACCATGAATGGGATGAAGGCCAGGCATGGCTGTGGGCATGGCCCGCTTGTTGCTAGCGGCACAGCTGTCACTTCGGGGCTCACGGTCACATTAGGCACGATGCAGGCACACGACGTGTAGTTGAGATGTGATCCGTGACCAGCTCCAGGCCGACAGCCCGCATGGCACGGTGAAAAGTAAGTGATGCCATTGCGACCACAGACAGGCTCGATGTCGTTGGGTGAACAACGG

>AAFF1927

CGAGGACCTGGAGTGCCGGCTGCGCCGCGACCTGGCCCCCGGAGACCCCGCCGAAGCGCTGTGCGTGTGCTCGCGCCAGGAGCCCGTGTGCGGCACCGACGGCGTCACCTACGACAACGTGTGCCAGCTGACCGAGGCCCGGTACCGGCTGCGCAACGGGCTCGAGGCGGCCTCCAGGGGACCCTGCTACTCCGCGCCCCGAGTGGTGACCGCTCCGGAGAACACGCGCAACCGGACGGGTGGCCGCGCCGCCATGACCTGCGAGGTGTCCGGCTTCCCGGTGCCCACCATCGAGTGGCGCGTCGACCGCGGGGACGGACCGCTCAAGTCGCTGCCCACGGACAGCTCCCGCATCAACGTGCAGAGCCGCGGCGGACCGGACAGCTTCGAGGTGACCAGCTGGCTGCAGCTGCTCGACCTGAGGCCCGAGGACACGGCCACCTATTGGTGCGTGGGCGCCAACGAGAACGGAGAGGCCTCGGCCGCCGCCAAGCTCAACGTGCTGCCCTAGGCCGCCCCTCGTCCTTGTTGTTGACTCCTCCTCCTTGTTGTTGAACGCCATCCATACTTGCATTACACACCCGCGCGTCGTCGTCTCGTTTAATGTGTTCCTTCACTTGGTTTTATATTCTTTTTTTCGTCGTCG

>AAFF2152

CACATACTTTAGATTACTGCTCTAAGAGTATTTATTCAACAAGATAAATGTTGTACAAGCAGGTGGGGAAACTTAAAACGTGTATATAGCACTCAATAGATGCATTTATCACAACGCATAATATTATATGGTTTGACTGGGAAAACCATCACTGGCAACTGTTCGACACATTGCATACGTATAGTATTTAGTTAGTCTGAAACATTGTTGTTCTTCTTGTGGTGATGCCCTTTCTTGTGCTTTGGTGCCTTGAGTTCACATGGAAAGCTGGCACAAGACCAGTTCCCACCAGCACAGACACAGTTACTGCAGTCGATCATTATTTCATCACCATCTTTATAGTACCGGTCCTCCAAAGGGCAGCTCTTGATAGGTGGACGGTACGATGGCCTCATGAAATTTTGGAACTCCCGCCTTGAGAGTAGCCAATCTGAATTTCTGTCAGCAACCTCCACCATGGCATCGATACACAGTGTAGCTGCCAGTTCACTTCTATGTTTCAGCCATGTGTGGAACATGGTGTTGTTCCTCCGGACACACTTTGAAAGCTCATGAGTGCTGAGGAAATCGTCACCGTCATGGTCACAGTCATCAAACACATCGTCAACCACTCTTGGGTAATGCCTGTCGTGAGAGACCTTAGATTCGAGAAGCTCCACAAAGTGCCTTCGAATGCGATCCCTCTCATGTTGGAAGCAAACAACTGGCTTTTGGTGATACTTGTGATGTGGCAACTTGACCTTGGGCTTTTTGCAGTGTCCCTTGTGCTTGATGGATATGTGCTTTTGGCCCAGGCAGGCGTCTTTGTGAAGCATACAGTGGTTGTCGTAAGTCACTCCATTAGTACCGCATACAGGCTTGTGGTGGGATGGGCAGTGCTGAACACACTGGCACGACGCCACTCCATTGTCCAAGATCCGACACACCCTTCCGGGCCGACATATAACGGCGGCGCAGATGTCCAGCTGACTATCTGTGATCTCATCTTTTACCGGCACCGATGTGACGCTTGCCCAACCAGCAGCGAAAAGCAACCACGCACTCAAGAACTTCATTGTAGGTGTTGCCAAGTGCCTCCGACGCGAGTGACGCCGCTCACGCGAACGGCAGTAATCAAATTGAGCTTGGCTCGGAAATACGATCGCACGATCCCGCAGCGGAAACCATGCCTTCGCGTATCCGTTTCAAA

>AAFF17370

TTTTTTCGAAAAAGTTTATTTGAATCTCGTAAAATGGCAACCAAACAAAAATGAAAACAATGAGGACAAGGTGACGATCACGAGCAGAAGCTTTTTGGCATGACGACGATATTCCGGCCGCAGTTTCTCTGTTGCATGCGGCAGTGGTTTAGGTAGCGTTGTCCGTCCGATCCGCACACGGGGTCCAGGATGGGCAGGCATCGCTTGGGGCACTTGGGAGGCTCCAGGCACCGGTTCAGCGGCACCATCTGAACGTGCTCCTTGCTCCCGCAGGTCTCCTTCTTGAAGAAGCACTCGTTCAGGTACACCTCGCCGTTGGACCCGCACACGGGCTCGTACAGCTCCGAGCAGTCCTGGGGACACGAGTCCAGCTTGCGGGCCTCTCTGGCGCTGGCTATGCAGACAGCCAGTGTTTGCGTTCCGACGACCTTCCCGCAATTTCGTCGCTGCATGATGCACAGGTTGGGGTAGAAGCGGCCGTCCTTTCCGCACACCGGGTCGTAGATGTCGAGGCACTTGTCCGGGCAGAGCGGGTGGCGGCCGCGGCAGCGCTCCCACTCCACCACCCGAACGGGCTCCAGGGGACCCCCGCGCTGCCGCCGCTGTTTCCGGCAGTTGGCGTTCCTCAGCGAGCATTCGCTGTTGTACACCCGGCCGTTGGACCCGCACACCGGAACCGGAAACGATTCATTGCAGGGCATTCCGCAGGCAGAGACGCCGGGACTGCCATCAATTCGTGGTTGATCCACAGGTAACAATGGCTGCTCTGTAGCTGTGCCCGCGCTGCTGACTGCGTTGGAACCTGGCTGCTGGCTGTCTCCTTCTACCGTCGGTGGCAGCGCGGTCGTAGGCACAGCTTTCGCTGCATGACTGATGAGCAGAAGGAGCGCGATGCTGGAGCAGCGGCAGCAGACAGGATTCGGACCGGTAGACATGTTCCTTCCGATACGGACGACGACGTACTCCGGCAGACGGCCGGCAAACGGCTCTCTCCTCCTGTAGCGGCAGTCGGGCTGCCCGTGCCGAGATTGAGAGAGAAAAAAA

>SG12025463

TGCCAGGGGACCCCCGCGCTGCCGCCGCTGTTTCCGGCAGTTGGCGTTCCTCAGCGAGCATTCGCTGTTGTACACCCGGCCGTTGGACCCGCACACCGGAACCGGAAACGATTCATTGCAGGGCATTCCGCAGGCAGAGACGCCGGGACTGCCATCAATTCGTGGTTGATCCACAGATAACAATGGCTGCTCTGTAGCTGTGCCCGCGCTGCTGACTGCGTTGGAACCTGGCTGCTGGCTGTCTCCTTCTACCGTCGGTGGCAGCGCGGTCGTAGGCACAGCTTTCGCTGCATGACTGATGAGCAGAAGGAGCGCGATGCTGGAGCAGCGGCGGCAGACAGGATTCGGACCGGTAGACATGTTCCTTCCGATACGGACGACGACGTACTCCGGCAGACGGCCGGCAAACGGC

>SG12041235

GGCTTTCCCGTGGCATCTTTATTGGCTTTCTTCTCCTTGTCAATAGTGTTTTGTAGCTTGGCCATATAGCGGTCCCAGCGTGCTTTCTGACGGCTCTCACGCTGCTGTTCTTCTAGGCTTTTCACAGCTTTGCACGGGCAGAATCCCTTGCAGGAGACCTGCACTGAGGTACGCTGGCGGCAGTTGTGGTACTGCAGCCGACACTCAGAAGAATAAGTCTGGTTGTCCGTGCCGCACACATAGTCCGCCTTGAACACGGGGCGCGGTGGGCACCG

>SG1204501

ATGTCAACTTTCCATTTGAGACATCCAACCTGTCACCAGGCAACCCGCACTGGGTGGGTGCTTGGTGGCTGGGCGTGTTCATTGTGGGTGCGGCACTCATCGTTACGGCACTGCCCATGATGGCGTTCCCGCGCAACCTGCCTCAGCGGCGGTCCTCGCAACGAGTGCCGCGGCCTGCCTTGGACCGCAATGGCGCCATCTGCCCCAAGCACCAGCTCCAGCAGAAGATGCCGCTCATCAGTTCACCGCCTGCTTCGCCGTCGGGAGCCAAGAAGGCCCTGAAAGACAAGCCCACACTCAGAGNNNNNNNNNNNNNNNNNNNNAGATTTCCCCACAGCCATTCGGCGGCTGTTGAAGAACGAGATCCTGCTGTACCGGACGGCCAGCAGTGTCCTGCACATCTTGCCAATCGCTGGCCTCTACACTTTTCTGCCCAAGTACTTGGAGAGCCAGTTTCGGCTCACCGAGACCAAGGCCAACATGATTACAGGGTTTGCTGGCATTCTCGTCATGGGTGTGGGCATTTTTGCCAGTGGCACATTCATGCGCAAGTACAAGCCCAACGCCCGCTTTGTTGCCAAATGGATTGCATTCACTGCCCTGGCCTACTCCGTCGGCATGGTCATCCTCATGTGGGTGGGCTGTCCCCTCGGAGACTACGTTGGCCTCAATTCAGAACACAAAAGCCTAGACTATCTACCTCCAGCCTGCAACAGCACTTCTTGCGAGTGCAAGTCTGGGCTGTTCTCTCCAGTGTGCCATGATAGTGTGACCTACTTGTCGCCATGCCTGGCTGGCTGTTCACAAGTGTCTGGCAGTGATCAGCAGCCGAACTTCAGTGACTGCCATTGCACAGGAACAAACGAAACCATAACAAATGGGTTCTGCTCCCTTGAGTGCAACAACCTGACCTGGTACATCGTCATCTTCTCTGTTTTTGTACTCATCCACTCAACATCCGAAGTAGGGTCTATGCTACTGACCCTCAGATGTGTTGAATCCCATGACAAGGCCCTTGCATTGGGACTCATCCAGTTTGCCATTGGATTGTTTGGGAACGTGCCTTGTCCCATCATCTATGGTGCAGTCGTTGACTCTGCCTGCCTCTTCTGGGAAGACAACTGCGGAGAACCGGGCGCATGCCGCGTCTACGACCCGGCCAAGTTTCGTATGGTGTTCCATGGAGTCACAGCTGTCATCATGTTTGTGGCGTTCCTTGTGGATGCTGTTGTTTGGTACAAGGCGAGCTCCATCCACATTCACGAAGAGGAAGAGACCAGGGACACTGCGGTGGCAGCGGCAGCTCACGACGTGTCCTCTTCGACTCCACCAGTCCTGACGGCACATGCGGAAACAGAATCGTGCGTGTGAGCAGAGGTGGCTTTCCTCACGGCCGAGTGCTCTGGGTTGAGAAAAAAGGACGCCATGGTGGTCAGTGTTGGGCCTCTTCGATTTGTCTGTACTGGCAGAACCAGTTCGGTGGCAGAGCATTTGAAGCAGTTCAGTTTTCAGGGGCACCTGATTGTTCCTTTTTCACTACTCTTTGCCACCTACACAGCAAGCTTGCTAAGCTCTGTAGCCCATACTGGCAGTTTCACCGGAGATGATCGCCGTATGTGGTTCATCAGGGAGAGAGAATGCACTCACTGCCATGTGTCAGTGTGTGGCTTCACAACAGCTGTCTTCAAATGTCACACATCTTACTTGTCCCAGAGAAGTTGGCTGCAGTAGTTTTCAATTTCTACC

>SG1209978

GTTCCGACCGTGGCGACACTTACGGCGCGGACGCGCAACTTGCAACATGCCACAGCACTGGCTTAGGTGCCGTCGGGTTCATCGTGATATCGCACTGTCGCTTCAGCTTTTTCTTAACCGACGCTCCTGGGAACTGCGAAAGCCGAGCTGAAACGTTCGCTGTGAATAACCAGTGTGTTGATGCAGCATAAAACGCCAGGATGGCGCAGAATATCTCGACAGTTTTATTCCGCGGTTCGGTCACGAATCCACCGCTTGCTTCCACTTCCGTAAGCACGCGAGCCATTTTCGCCAAATTATTTCTGAGACAGAGCGTATTTCCCTCCGCGAACCGTCCTTCTACGCGGAGGCTTCCCTGCCAGTGGCTTGCTGTGCTGGCAAGAGTGGCGTCGCGCTAGGCGCCGAGTCCGCGTCGCTCGTCGCTGAGTCCTCGTAGAAATTCGTCACCCGGTCGCTGTAGTGGACCACGGCACCCTGGAAGAAGCAACCGACGACAAGCAAAGCCGCGGTGACTCCGTGGAGCAGGGAGCGGAACTTGGGCAGGTCGTAGAGCCAGCAGGCGCCCCGCTCGCCACATCGGTCCTCCCACACCAGGCAGCTGGCGTCCGCCACGGCTCCGTAGATGAGCGGGTACGGTATGAACGCAAACATATTCATAATGGCGGACAATATAGACAGGGCTGCGCTTTTGTCATTCACGTTCAGAGACCTGATCAAAAGTATCGTTGAACCAACCAGGGTGGTTGAGTAGCAGACCTGGATGACAAACACAACCACCGAGAACGCCGTCAGGGCGCTTGAGCAGTCCTGCTCGCATTTGCCGGACGCGACGACCCCGCTGTAGAAAGTCTCTTTCTCCCCTTCATTCACCAAACAGCGGCAGTCTTTAAATTCGCTCTCGTTTCCCAATTGTGAGCTGCACCCGGCGAAGCACGGAGAGAAGTACTGCATCCCCGTTGACTCGTCGCACACGGGGCGGTGCACGTCCGGAGAGCACGAGCATTCGCGCGTGCAAGCGTTTTCCACGGTACTGCTTCCGGTTTCGTGGTGCGCTGCGATCGGGTAGCGGAGTGTGCCGCAGTCTACAGCCATGAGCGCCGAGATGCCCAGCACGGCCAGGAGGGTGACGAGTACGTTATGCCAGGCGATGATCCGTGGCCGCGGCTGCCAACGGTGAACGAACACGGCCCCCGCCAGGGTGCCCACCATGTTCGACACAATCACGACGGGCCCTGTGAACAGACTGGCCCTTGAAGCAGTCTGATGGAACTGGTGCTGCGTGTATTTTGGGAAGGAGACGTAGTAACCAGTCAGGGCTATATACACTGCTATGTTTCCCAGCGTCCTGAATACGTAGATCGGGTTTCTTGTGAGTCTCCTCAAAACTCGAACGGCATCTTTGACTTCCTCTCTTATCGAGGATGAGCCAGCCGAACAGGAAACCACCGGATCGACGAGTGAGGCATCGTCTCCGGCCATCTTCGATGAGCGCCGCAGTGTCCGTGGGAAGAGGGCTACTGGGAAGGTGCTCAGTAGGAGGCATAGGCCGACCACCAGGTAGCCCATCCACCAGGCCCCAATCCAGCGAGGGTCCCCGGGGCTGATGCCCGGCTCCGCCCACGGATCCTCGTAGTGGCTGAGGCAGAAGGAACCCAAAGTCAGCCCGACGACGGGCCCTATGAAGCGGCATATGTAGATGCTTCCGAAATATAGGGCCGAGTTCTTTTTCTTGACGTTGTCGTCCATGTACGTAGTGCCCACGACGTAGCAGGCAACGCCGCCCAGGCCATTCAAGAAGTTCCCCGCGAACAGAAGCGTCACGGCACCCACCGCAGACCAATCCGATCGATGGCTTTCGCAGTGGCCTGAACGATTGAAGACACCGCCGTTACCGCCGTCCTCACTGCAAAACTGGAGGGGCGGGGCCGTCGAGTTTTCCGGAGCGCTGGCCTGCTCCAGAAGGTGCGTCCCTGCGCCGAATATAGCGTATGGCAGGTAGCTGACCAGTGCGCCCACGAGCGACAGGAGCATGCCTCCCGATATCCAGTTGGGCATGCTTGTCCGACGCAGGAACAGCAGGAACACGGCGCTGGCCAGAAGAGGACTCAGGTCGTCGGCGATGAGGATGATGCTCGAAACGCGGCTCGATATGGAGAAGCGTTTCTCGACGCTAGATAGAGTCCCGACCATGTAGGAGCGATAGGCTCCCTGGCTGACGCCGAGCAGTCCGAAGACGATTGCGTAGACACGTGGCGTGGCGAACCTTTGCAGCCAGTTCGGGCGGAAAGGTCCACACCCGCAGCGGTAGCGACGGCCATCAACGCCAGCATCCGAAGACTCCTTCCTCTCGGACTGCGAACTGCTATCCGCACTGTCTTGGCAGACG

>SG12014426

CCTGCTCCTGGCGCCCATCAGCTACTTCGGCGGCACCCGGAGCAAGCCGCTGTTCGTGGGCGTCGGCTGCCTCGTGCTGGGCCTCGGTGCCTTCGTCTTCTCGCTGCCGCACTTCCTGGCCGGTACGTACGCCTTCAGCACCGAGGACGACCGTGAGAGCCTCTGCCGCCTGGCGGCCAACGCCACCGAGGGTTTCTGCGGGAGCAGGGAGGTGGGCTCCCTGAACCAGTACAAGTACATGTTCCTCGCGGGACAGATGCTGCATGGCGCCGGGGCGACACCCTTCTTCACACTCGGCTGTACTTACCTGGACGAGATCGTGTCTACGAAGATGTCCTCTGTGTACATTGGTATTTTCTATACCATGGCGATCATCGGCCCTGCACTTGGCTATATTCTAGGCGGCCAATTTCTGAAGATATACACCGACCTCTCCGTAGATGCTTCAGCATTAGGCTTGACGCCCTCCAGTGGTGTCTGGGTGGGCGCCTGGTGGATTGGTTTCGTCGTGTCCTCCGTAACGGCTCTCCTTGCTGCCGTCCCCATTAGTGCCTTCCCCAAGGTCCTCCCAGGCTCATTAAAGCTGCAGGCTCAGAAGAAATCCGAGATGCACCAGAAGCTTCAGAAGAGCGAAGCGGTGCAGAGCGGCTTCGGGGCCCGCGCCAAGGATCTCCCGGCATCCTTCAAGATACTCATCACCAACCCGACCTTTGTGTTCCTAAGCTTGGCCGGAGCCACCGAAGGTATGCTGGTATCTGGCCTGGCGACATTCCTTCCTAAAGTCATTGAGTTCCAGTTCAGCATAGCGGCCAGTTTAGCGGCCCTAATAATGGGTGCGGTGACAGTTCCGGGCGCTGGTGGTGGCACATTCCTAGGCGGCTACTTCGTGAAGAAGTTCAACCTGCGCTGCGCCGGCATCATCAAGATGTGCGTCCTCTGCTCTCTCGTTCCACTCGTCACCATCTTCGCATTCTTCTTCAGCTGTCCCAACGTCAGATTCGCGGGCGTCAATTACAAGACGAACAACGTCACCGATGGAATAGACCGCTTCCTGTTAGACTGCAATAATCAGTGTCACTGCCAAATGGAAGACTTTGACCCCATCTGCGGAACGGACCACGTCATGTACTATTCCGCATGCTTTGCAGGTTGCCAAGAGGTGCACCACTATGGATCAACAAAAGTGTACGAGGACTGCCGCTGCATTGATCATCCGGGCAGAAACGTAACAATTAATGGGAAGCAAGTGACCATCCAGGCGGAGAGAGACAAATGTCCAAGCGAATGCAACTTCCTCGTCTTCTTCCTTATCGCCATGTTCGTCTGCATGGTCTTCACATTCCTGGTCAGCATGCCTTCGCTGGCCGCCACACTCAGGTGTGTTGCAGCTAGCCAGAAGTCTTTCGGTCTCGGCATACAGTGGATTGCTGTGCGGCTACTAGGGACCATACCGGCGCCCATCATGTTCGGCTACCTGATCGACCGCAGCTG

>SG12020992

GCAGGCGACTTGGGCACTCAGTCTACTCATTATCTTTTAATGAGCCACTGCTTTCCCTAATTTTCCTTTTGTTAAAAAACCCACATCACTGTTCTCTCACTTTTCTTGAAGCTGTCCTGGTGTTCGAAAGCAGTTGCATTTGGCAGCCTGTCTGTCAATGCGGAGCACATCCAGACCAGGGCAGACCGTATATATATACAGTACATTCACTTCGTTCTAAAACCTTTCGGTTGGAGCTCAATTTCTGTCCATTGTCATTGAATCCAAAAGGGGATTTTTGTTAAGCTGCTCTAGAATTCAAAACCTGCTGGCCTCTGGCCGTACGTTGGCCTTTTGGATGCTCTCAACCGAGAGCGCCTCAGTGATAGTTGTCATGCTGTTGTACTTGTCTTGCTCCTCAGTGCTCTCCTCAGCGAGATCGTCCTCATAGAAGCGCTTTATGCGTCCGCTCAGCAGGAACTGGCCGATGTAGCAGACACCAGCAATGGTTGCAAAGATGACCGTGATGAGGTGCAACGAGTAGCGTAGTTTGTCCGCGTCGTAAACCCAGCAGTTGCCACGCCGGCCACATTTCTCCTCCCACACAATGCACGAGTTGTCCAGGATGGCGCCATACACCAGAGGGTACGGGATGAAAGCAAGCATATTCAGCAGAGAACCTGTGGTCCCCAGGGCCATGCTTTTATCGATAGGATCGACGCACCTCAGGAAGATCAGCAGGCCGCCTACACGGCCCGTGGAGCCCAGGAACTGGCCAGCGATCACAATGCCCAGAAAGAGGCCCAGCTGCTGGCACGAGGATCCACAGAGCCCCGAGGTCACGTAGGAGTCGTCCCCTCCTTGGTTTCCTTGCGCGACGCAGCTGCAGTCTTTGTATATCGTCATGTTGGACGATGTCGTCCCCACGCTTTTGCAGCCGGCATGGCAAGGTGAGAAGTAGCTGATCATCCTATTGCTCGAGCACACCGGCTCGTAGATCTGCGTGTTGCAGTTACAGTCCACGTTGCATGTGTCCAGCAGCGAACTCGCGGTGACGTTTCCACCAGGAGTGACTCCCGCGATGACGGGACTTTCGCAGCCGATGAACATCATGACTACGAAGCCCGCCATCATGGCGATCTCGACGAACGCTGAGTAGCCTGCCACGATGCGCGGCCGTGGTCGGAACCGATGCACCACGACGCCGCCGGTGACGATGCCGATCAGGGTGGTCACCACTTTCGCTGCCCCTGTGTAGTAGTTAGCCTTGGACGCCGACTGCCTGAACTGGAACTCGACGTACTTGCTGAACGAGGTGCCGTAGCCGGCCGTGGCAAGCAGGACGAAGATGAGGCCAATTGAGTGAAACATGAAGATCGGGTTGCTCGCCAGTCGCTTTAGAATCACCATTATGTCTTTCACCATGGCGATGCTGAATCCGTGCTTTCCCGACCCTTCCTGTCCTTCCTCGCCCTTGGCGCCCTTCTGGGACTTGAGTGCGGACAGCTTCTGCAGGCGGTTCACCTTGTAGTTCTTGCCGGCGGGCAGGATGCGCGGGAACAGCATCATGGGCAGGGCGACGATGCTCAGCCCGATGCCGAACAGAATGTAGCCCATCCACCAGGCACCCACCCAGCGTGGGTCCCTTGGTGTGATGCCCGGGTCGTGGAAGGGGTCTTCGTAAAAACGCAGGCAAACCCCGGCAGTCACAAATCCCAGAACAGGACCCAGGAGCCTGAAG

>SG12020993

AATGCCCAGAAAGAGGCCCAGCTGCTGGCACGAAGATCCACAGAGCCCCGAGGTCACATAGGAGTCGTCCACTCCTTGGTTTCCTTGTGCGACGCAGCTGCAACCTTTGTATATCGTCATGTTGGACGATGTCATTCCCGTGCTTCTGCAGCCGGCATGGCAAGGTGAGAAGTAGCTGATCATCCTATTGCTCGAGCACACCGGCTCGTAGATATGCGTGTTGCAGTTGCAGTCCACGTTGCATGTGTCCAGCAG

>SG1201973

GAACACATTAAACGAGACGACGACGCGCGGGTGTGTAATGCAAGTATGGATGGCGTTCAACAACAAGGAGGAGGAGTCAACAACAAGGACGAGGGGCGGCCTAGGGCAGCACGTTGAGCTTGGCGGCGGCCGAGGCCTCTCCGTTCTCGTTGGCGCCCACGCACCAGTAGGTGGCCGTGTCCTCTGGCCTCAGGTCGAGCAGCTGCAGCCAGCTGGTCACCTCGAAGCTGTCCGGTCCGCCGCGGCTCTGCACGTTGATGCGGGAGCTGTCCGTGGGCAGCGACTTGAGCGGTCCGTCCCCGCGGTCGACGCGCCACTCGATGGTGGGCACCGGGAAGCCGGACACCTCGCAGGTCATGGCGGCGCGGCCACCCGTCCGGTTGCGCGTGTTCTCCGGAGCGGTCACCACTCGGGGCGCGGAGTAGCAGGGTCCCCTGGAGGCCGCCTCGAGCCCGTTGCGCAGCCGGTACCGGGCCTCGGTCAGCTGGCACACGTTGTCGTAGGTGACGCCGTCGGTGCCGCACACGGGCTCCTGGCGCGAGCACACGCACAGCGCTTCGGCGGGGTCTCCGGG

>SG1205921

CTTTAGATTACTGCTCTAAGAGTATTTATTCAACAAGATAAATGTTGTACAAGCAGGTGGGGAAACTTAAAACGTGTATATAGCACTCAATAGATGCATTTATCACAACACATAATATTATATGGTTTGACTGGGAAAACCATCACTGGCAACTGTTCGACACATTGCATACGTATAGTATTTAGTTAGTCTGAAACATTGTTGTTCTTCTTGTGGTGATGCCCTTTCTTGTGCTTTGGTGCCTTGAGTTCACATGGAAAGCTGGCACAAGACCAGTTCCCACCAGCACAGACACAGTTACTGCAGTCGATCATTATTTCATCACCATCTTTATAGTACCGGTCCTCCAAAGGGCAGCTCTTGATAGGTGGACGGTACGATGGCCTCATGAAATTTTGGAACTCCCGCCTTGAGAGTAGCCAATCTGAATTTCTGTCAGCAACCTCCACCATGGCATCGATACACAGTGTAGCTGCCAGTTCACTTCTATGTTTCAGCCATGTGTGGAACATGGTGTTGTTCCTCCGGACACACTTTGAAAGCTCATGAGTGCTGAGGAAATCGTCACCGTCATGGTCACAGTCATCAAACACATCGTCAACCACTCTTGGGTAATGCCTGTCGTGAGAGACCTTAGATTCGAGAAGCTCCACAAAGTGCCTTCGAATGCGATCCCTCTCATGTTGGAAGCAAACAACTGGCTTTTGGTGATACTTGTGATGTGGCAACTTGACCTTGGGCTTTTTGCAGTGTCCCTTGTGCTTGATGGATATGTGCTTTTGGCCCAGGCAGGCGTCTTTGTGAAGCATACAGTGGTTGTCGTAAGTCACTCCATTAGTACCGCATACAGGCTTGTGGTGGGATGGGCAGTGCTGAACACACTGGCACGACGCCACTCCATTGTCCAAGATCCGACACACCCTTCCGGGCCGACATATAACGGCGGCGCAGATGTCCAGCTGACTATCTGTGATCTCATCTTTTACCGGCACCGATGTGACGCTTGCCCAACCAGCAGCGAAAAGCAACCACGCACTCAAGAACTTCATTGTAGGTGTTGCCAAGTGCCTCCGACGCGAGTGACGCCGCTCACGCGAACGGCAGTAATCAAATTGAGCTTGGCTCGGAAATACGATCGCACGATCCCGCAGCGGAAACCATGCCTTCGCG

>SG12020404

CGAAAAAGTTTATTTGAATCTCGTAAAATGGCAACCAAACAAAAATGAAAACAATGAGGACAAGGTGACGATCACGAGCAGAAGCTTTTTGGCATGACGACGATATTCCGGCCGCAGTTTCTCTGTTGCATGCGGCAGTGGTTTAGGTAGCGTTGTCCGTCCGATCCGCACACGGGGTCCAGGATGGGCAGGCATCGCTTGGGGCACTTGGGAGGCTCCAGGCACCGGTTCAGCGGCACCAGCTGAACGTGCTCCTTGCTCCCGCAGGTCTCCTTCTTGAAGAAGCACTCGTTCAGGTACACCTCGCCGTTGGACCCGCACACGGGCTCGTACAGCTCCGAGCAGTCCTGGGGGCACGAGTCCAGCTTGCGGGCCTCTCTGGCGCTGGCTATGCAGACAGCCAGTGTTTGCGTTCCGACGACCTTCCCGCAATTTCGTCGCTGCATGATGCACAGGTTGGGGTAGAAGCGGCCGTCCTTTCCGCACACCGGGTCGTAGATGTCGAGGCACTTGTCCGGGCAGAGCGGGTGGCGGCCGCGGCAGCGCTCCCACTCC

>SG9613158

ACGATGATGACCAGTACCTGGAAGATGACAGCAGCCAGGAAATGGAACGGTGCCCACCGTGCCCCGTGTTCAAGGCGGACTATGTGTGCGGCACGGACAACCAGACTTATTCTTCTGAGTGTCGGCTGCAGTACCACAACTGCCGCCAGCGTACCTCAGTGCAGGTCTCCTGCAAGGGATTCTGCCCGTGCAAAGCTGTGAAAAGCCTAGAAGAACAGCAGCGTGAGAGCCGTCAGAAAGCACGCTGGGACCGCTATATGGCCAAGCTACAAAACACTATTGACAAGGAGAAGAAAGCCAATAAAGATGCCACGGGAAAGCCTGCTGCCTTCAACAACAGACTGAAGTCAAAGTTCAGTTCCAAGGACTCGTGGGCCAGCAAGGATGGTCATAATTCAGTCATGACGAACAAGGTTTCCTCCCCTGTTGTCAGCAAGGATAAAGGGTGCACTAATGATGAACTGCAGGCCATGGGTGACCGACTGCTTGACTGGTTTTCTGTGGTCATGAGTGACCATCAACTAAGCACTCAAGTATCCACCAGACGGAGGACCCATAGGGTGTCCGATTACCATTTGCCAGAGTGCAAGCCTGAGGTAGGCTGGATGTTCCACCACCTTGACTCGG

>SG96815

GCGCCGTGTTCCTGCTGTTCCTGCGTCGGACAAGCATGCCCAACTGGATACCGGGAGGCATGCTCCTGTCGCTCGTGGGCGCACTGGTCAGCTACCTGCCATACGCTATATTCGGCGCAGGGACGCACCTTCTGGAGCAGGCCAGCGCTCCGGAAAACTCGACGGCCCCGCCCCTCCAGTTTTGCAGTGAGGACGGCGGTAACGGCGGTGTCTTCAATCGTTCAGGCCACTGCGAAAGCCATCGATCGGATTGGTCTGCGGTGGGTGCCGTGACGCTTCTGTTCGCGGGGAACTTCTTGAATGGCCTGGGCGGCGTTGCCTGCTACGTCGTGGGCACTACGTACNNNNNNNNNNNNNNNNNNNNNNNNNNNNNNNNNNNNNNNNNNNNNNNNNNNNNNNNNNNNNNNNNNNNNNNNNNNNNNNNNNNNTTGGGTTCCTTCTGCCTCAGCCACTACGAGGATCCGTGGGCGGAGCCGGGCATCAGCCCCGGGGACCCTCGCTGGATTGGGGCCTGGTGGATGGGCTACCTGGTGGTCGGCCTATGCCTCCTACTGAGCACCTTCCCAGTAGCCCTCTTCCCACGGACACTGCGGCGCTCATCGAAGATGGCCGGAGACGATGCCTCACTCGTCGATCCGGTGGTTTCCTGTTCGGCTGGCTCATCCTCGATAAGAGAGGAAGTCAAAGATGCCGTTCGAGTTTTGAGGAGACTCACAAGAAACCCGATCTACGTATTCAGGACGCTGGGAAACATAGCAGTGTATATAGCCCTGACTGGTTACTACGTCTCCTTCCCAAAATACACGCAGCACCAGTTCCATCAGACTGCTTCAAGGGCCAGTCTGTTCACAGGGCCCGTCGTGATTGTGTCGAACATGGTGGGAACCCTGGCGGGGGCCGTGTTCGTTCACCGTTGGCAGCCGCGGCCACGGATCATCGCCTGGCATAACGTACTCGTCACCCTCCTGGCCGTGCTGGGCATCTCGGCGCTCATGGCTGTAGACTGCGGCACACTCCGCTACCCGATCGCAGCGCACCACGAAACCGGAAGCAGTACCGTGGAAAACGCTTGCACGCGCGAATGCTCGTGCTCTCCGGACGTGCACCGCCCCGTGTGCGACGAGTCAACGGGGATGCAGTACTTCTCCCCGTGCTTCGCCGGGTGCAGCTCACAATTGGGAAACGAGAGCGAATTTAAAGACTGCCGCTGTTTGGTGAATGAAGGGGTGAAAGAGAACTTCTACAGCGGGGTCGTCGCGTCCGGCAAATGCGAGCAGGACTGCTCAAGCGCCCTGACGGCGTTCTCGGTGGTTGTGTTTGTCATCCAGGTCTGCTACTCAACCACCCTGGTTGGTTCAACGATACTTTTGATCAGGTCTCTGAACGTGAATGACAAAAGCGCAGCCCTGTCCATATTGTCCGCCATTATGAATATGTTTGCGTTCATACCGTACCCGCTCATCTACGGAGCCGTGGCGGACGCCAGCTGCCTGGTGTGGGAGGACCGATGCGGCGAGCGGGGCGCCTGCTGGCTCTACGACCTGCCCAAGTTCCGCTCCCTGCTGCACGGAGTCACCGCGGCTTTGCTTGTCGTCGGTTGCTTCTTCCAGGGTGCCGTGGTCCACTACAGCGACCGGGTGACGAATTTCTACGAGGACTCAGCGACGAGCGACGCGGACTCGGCGCCTAGCGCGACGCCACTCTTGCCAGCACAGCAAGCCACTGGCAGGGAAGCCTCCGCGTAGAAGGACGGTTCGCGGAGGGAAATACGCTCTGTCTCAGAAATAATTTGGCGAAAATGGCTCGCGTGCTTACAGAAGTGGAAGCAAGCGGTGGATTCGTGACCGAACCGCGGAATAAAACTGTCGAGATATTCTGCGCCATCCTGGCGTTTTATGCTGCATCAACACACTGGTTATTCACAGCGAACGTTTCAGCTCGGCTTTCGCAGCTCTCAGGAGCGTCGGTTAAGAAGAAGCTGAAGCGACAGTGCGATATCACGATGAACCCAACGGCACCTTCNNNNNNNNNNNNNNNNNNNNGAAGAGATAAATACCGCAGCAATGGCACGTTCGAGGCGAACCGTGTAAGCCGATGATAGCGCGAGCACGTGGGAAGAGAAGAAACCTAAACAGATGAGAG

>SG965925

AAATGTGTCACTAAATTTCAAACCAATAATTTAGAAAGGCAAGTTCGTCATCCCAATAAACAGTGAAAATCTTATAGTACAACTTCAGGTTGAATTCACTGTACGTAAGTTAAGGCAAGTGCAAGTGCTGGATTGAAGCACATGAACATTAGTCTTGATTCCAACGAGAGTACCACAATTTAACAGTGCAGGCTTTGTATGCACATTTCGTATATGCACTAAGGAGAGGCACTCGAAAGACAGCATTAAAATGTCTGTTCAGATTTAACCATTAACCTAAGATCAGAACCATATCTAACAATGAAAATTTTGAGATATTTTCAACAGGCATAACAGACTCATCCAAGCAAGATTGTGCGAAATTCAGGATGCTGCTTTGCCATCTGCTGGGTACGCCAGCAGCAAGAGCCTTGATAAATTATGAGTGAAAATTTTTCTTCTTGGTCTAAAGTCACAAGCTTATGAACAGCATAGCATGAAAAAGTCGCATAGCTTTCGCACCAAACAAAGGAACACTACGCAAAGAAATGATCTCATATATTACTAAAACTCAAGGAAAACAAAAAAGCAAATATCGTTTTCACAAGGGTAAAAGAATAGAGAAAAACAAGCTCTCCTGTAATTGTGCGTTTTCTCCTTTCTCGAACACAGAGCTTTCATGTCGCGAATGACTGTCCTTTTCTCGACAGCTTGCAGTTCAAATCTTGCCAACACATTTTACCCCATCAAAATCATTTCACACAGTTCGCTCACTTCTAGCCTGGCAGCTCAACCTCTCTAGAAAAAAAAAAAATAAAGCACTCTCCACACCAAGAGTCCGTGCACACATTTCAGAAGCTGCCTTTCTCACACTGCACCAGCAACAAAAGCACCCAGTGGGCACAAGGGCAATCACCACCACACAACAAAGCAACATGCGAAATGAGTCATTTCTGTACAAAAACAGATGCACTCGATCGTCCAGCGATGCAAACAATATGTGGGAGGGTCTCTTCGATTTTGCTCAAACAGGACAGACCAGTACTGCAATAGTTTCACACTCTGGCAGCCCTACATTACCTCACAAAAAGCCTGCAACACTTGCAACACGAAATGGAGCAGGTGCTAACAACTGTACTCAAGCGAACACAGCACGTGGCCTAGTTAACCTCCCTGTCTTTCCATTCTTTCTAGCTCGCATCACTGCCCTTAGCAGTTGTAACGCTCCTACTCAAAAATGTCAAGCCCTAAACTGCGACCTGCTCGGTCCATTCTAACAAACATTGGTAGAAATTGAAAACTACTGCAGCCAACTTCTCTGGGACAAGTAAGATGTGTGACATTTGAAGACAGCTGTTGTGAAGCCACACACTGACACATGGCAGTGAGTGCATTCTCTCTCCCTGATGAACCACATACGGCGATCATCTCCGGTGAAACTGCCAGTATGGGCTACAGAGCTTAGCAAGCTTGCTGTGTAGGTGGCAAAGAGTAGTGAAAAAGGAACAATCAGGTGCCCCTGATAACCGAACTGCTTCAAATGCTCTGCCACCGAACTGGTTCTGCCAGTACAGACAAATCGAAGAGGCCCAACACTGACCACCATGGCGTCCTTTTTTCTCAACCCAGAGCACTCGGCCGTGAGGAAAGCCACCTCTGCTCACACGCACGATTCTGTTTCCGCATGTGCCGTCAGGACTGGTGGAGTCGAAGAGGACACGTCGTGAGCTGCCGCTGCCACCGCAGTGTCCCTGGTCTCTTCCTCTTCGTGAATGTGGATGGAGCTCGCCTTGTACCAAACAACAGCATCCACAAGGAACGCCACAAACATGATGACAGCTGTGACTCCATGGAACACCATACGAAACTTGGCCGGGTCGTAGACGCGACATGCGCCCGGTTCTCCGCAGTTGTCTTCCCAGAAGAGGCAGGCAGAGTCAACGACTGCACCATAGATGATGGGACAAGGCACGTTCCCAAACAATCCAATGGCAAACTGGATGAGTCCCAATGCAAGGGCCTTGTCATGGGATTCAACACATCTGAGGGTCAGTAGCATAGACCCTACTTCGGATGTTGAGTGGATGAGTACAAAAACAGAGAAGATGACGATGTACCAGGTCAGGTTGTTGCACTCAAGGGAGCAGAACCCATTTGTTATGGTTTCATTTGTTCCTGTGCAATGGCAGTCACTGAAGTTCGGCTGCTGATCACTGCCAGACACTTGTGAACAGCCAGCCAGGCATGGCGACAAGTAGGTCACACTATCATGGCACACTGGAGAGAACAGCCCAGACTTGCACTCGCAAGAAGTGCTGTTGCAGGCTGGAGGTAGGTAGTCTAGGCTTTTGTGTTCTGAATTGAGGCCAACGTAGTCTCCGAGGGGACAGCCCACCCACATGAGGATGACCATGCCGACGGAGTAGGCCAGGGCAGTGAATGCAATCCATTTGGCAACAAAGCGGGCGTTGGGCTTGTACTTGCGCATGAATGTGCCACTGGCAAAAATGCCCACACCCAT

>SG9635692

CGATCATCGGCCCTGCACTTGGCTATATTCTAGGCGGCCAATTTCTGAAGATATACACCGACCTCTCCGTAGATTCCTCAGCATTAGGCTTGACGCCCTCCAGTGGTGTCTGGGTGGGCGCCTGGTGGATTGGTTTCGTCGTGTCCTCCGTAACGGCTCTTCTTGCTGCCGTCCCCATTAGTGCCTTCCCCAAGGTCCTCCCAGGCTCATTAAAGCGGCAGGCTCAGAAGAAATCTGAAATGCACCAGAAGCTTCAGAAGAGCGAAGCGGTGCAAAGCGGCTTCGGGGCCCGCGCCAAGGATCTCCCGGCATCCTTCAAGATACTCATCACCAACCCGACCTTTGTGTTCCTAAGCTTGGCCGGAGCCACCGAAGGTATGCTGGTATCTGGCCTGGCGACATTCCTTCCTAAAGTCATTGAGTTCCAGTTCAGCATAGCGGCCAGTTTAGCGGCCCTAATAATGGGTGCGGTGACAGTTCCGGGCGCTGGTGGTGGCACATTCCTAGGCGGCTACTTCGTGAAGAAGTTCAACCTGCGCTGCGCCGGCATCATCAAGATGTGCGTCCTCTGCTCTCTCGTTCCACTCGTCACCATCTTCGCATTCTTCTTCAGCTGTCCCAACGTCAGATTCGCGGGCGTCAATTACAAGACGAACAACGTCACCGATGGAATAGACCGCTTCCTGTTAGACTGCAATAATCAGTGTCACTGCCAAATGGAAGACTTTGACCCCATCTGCGGAACGGACCACGTCATGTACTATTCCGCATGCTTTGCAGGTTGCCAAGAGGTGCACCACTATGGATCAACAAAAGTGTACGAG

>SG9639427

CGGGCTTCGTAGTCATGATGTTCATCGGCTGCGAAAGTCCCGTCATCGCGGGAGTCACTCCTGGTGGAAACGTCACCGCGAGTTCGCTGCTGGACACATGCAACGTAGACTGCAACTGCAACACGCAGATCTACGAGCCGGTGTGCTCGAGCAATAGGATGATCAGCTACTTCTCACCTTGCCATGCCGGCTGCAAAAGCGTGGGGACGACATCGTCCAACATGACGATATACAAAGACTGCAGCTGCGTCGCGCAAGGAAACCAAGGAGGGGACG

>SG964256

CGGAGACCCCGCCGAAGCGCTGTGCGTGTGCTCGCGCCAGGAGCCCGTGTGCGGCACCGACGGCGTCACCTACGACAACGTGTGCCAGCTGACCGAGGCCCGGTACCGGCTGCGCAACGGGCTCGAGGCGGCCTCCAGGGGACCCTGCTACTCCGCGCCCCGAGTGGTGACCGCTCCGGAGAACACGCGCAACCGGACGGGTGGCCGCGCCGCCATGACCTGCGAGGTGTCCGGCTTCCCGGTGCCCACCATCGAGTGGCGCGTCGACCGCGGGGACGGACCGCTCAAGTCGCTGCCCACGGACAGCTCCCGCATCAACGTGCAGAGCCGCGGCGGACCGGACAGCTTCGAGGTGACCAGCTGGCTGCAGCTGCTCGACCTGAGGCCAGAGGACACGGCCACCTACTGGTGCGTGGGCGCCAACGAGAACGGAGAGGCCTCGGCCGCCGCCAAGCTCAACGTGCTGCCCTAGGCCGCCCCTCGTCCTTGTTGTTGACTCCTCCTCCTTGTTGTTGAACGCCATCCATACTTGCATTACACACCCGCGCGTCGTCGTCTCGTTTAATGTGTTC

>SG967826

TATTTCCGAGCCAAGCTCAATTTGATTACTGCCGTTCGCGTGAGCGGCGTCACTCGCGTCGGAGGCACTTGGCAACACCTACAATGAAGTTCTTGAGTGCGTGGTTGCTTTTCGCTGCTGGTTGGGCAAGCGTCACATCGGTGCCGGTAAAAGATGAGATCACAGATAGTCAGCTGGACATCTGCGCCGCCGTTATATGTCGGCCCGGAAGGGTGTGTCGGATCTTGGACAATGGAGTGGCGTCGTGCCAGTGTGTTCAGCACTGCCCATCCCACCACAAGCCTGTATGCGGTACTAATGGAGTGACTTACGACAACCACTGTATGCTTCACAAAGATGCCTGCCTGGGCCAAAAACACATATCCATCAAGCACAAGGGACACTGCAAAAAGCCCAAGGTCAAGTTGCCACATCACAAGTATCACCAAAAGCCAGTTGTTTGCTTCCAACATGAGAGGGATCGCATTCGAAGGCACTTTGTGGAGCTTCTCGAATCTAAGGTCTCTCACGACAGGCATTACCCAAGAGTGGTTGACGATGTGTTTGATGACTGTGACCATGACGGTGACGATTTCCTCAGCACTCATGAGCTTTCAAAGTGTGTCCGGAGGAACAACACCATGTTCCACACATGGCTGAAACATAGAAGTGAACTGGCAGCCACACTGTGTATCGATGCCATGGTGGAGGTTGCTGACAGAAATTCAGATTGGCTACTCTCAAGGCGGGAGTTCCAAAATTTCATGAGGCCATCGTACCGTCCACCTATCAAGAGCTGCCCTTTGGAGGACCGGTACTATAAAGATGGTGATGAAATAATGATCGACTGCAGTAACTGTGTCTGTGCTGGTGGGAACTGGTCTTGTGCCAGCTTTCCATGTGAACTCAAGGCACCAAAGCACAAGAAAGGGCATCACCACAAGAAGAACAACAATGTTTCAGACTAACTAAATACTATACGTATGCAATGTGTCGAACAGTTGCCAGTGATGGTTTTCCCAGTCAAACCATATAATATTATGTGTTGTGATAAATGCATCTATTGAGTGCTATATACACGTTTTAAGTTTCCCCACCTGCTTGTACAACATTTATCTTGTTGAATAAATACTCTTAGAGCAG

>SG9625792

AAAAAGTTTATTGAATCTCGTAAAATGGCAACCAAACAAAAATGAAAACAATGAGGACAAGGTGACGATCACGAGCAGAAGCTTTTTGGCATGACGACGATATTCCGGCCGCAGTTTCTCTGCTGCATGCGGCAATGGTTTAGGTAGCGTTGTCCGTCCGATCCGCACACGGGGTCCAGGATGGGCAGGCATCGCTTGGGGCACTTGGGAGGCTCCAGGCACCGGTTCAGCGGCACCATCTGAACGTGCTCCTTGCTCCCGCAGGTCTCCTTCTTGAAAAAGCACTCGTTCAGGTACACCTCGCCGTTGGACCCGCACACGGGCTCGTACAGCTCCGAGCAGTCCTGGGGGCACGAGTCCAGCTTGCGGGCCTCTCTGGCGCTGGCTATGCAGACAGCCAGTGTTTGCGTTCCGACGACCTTCCCGCAATTTCGTCGCTGCATGATGCACAGGTTGGGGTAGAAGCGGCCGTCCTTTCCGCACACCGGGTCGTAGATGTCGAGGCACTTGTCCGGGCAGAGCGGGTGGCGGCCGCGGCAGCGCTCCCACTCCACCACCCGAACGGGCTCCAGGGGACCCCCGCGCTTCCGCCGCTGTTTCCGGCAGTTGGCGTTCCTCAGCGAGCATTCGCTGTTGTACACCCGGCCGTTGGACCCGCACACCGGAACCGGAAACGATTCATTGCAGGGCATTCCGCAGGCAGAGACGCCGGGACTGCCATCAATTCGTGGTTGATCCACAGGTACCAATGGCTGTTCTGTAGCTGTGCCCGCGCTGCTGACTGCGTTGGAACCTGGCTGCTGGCTGTCTCCTTCTACCGTCGGTGGCAGCGCGGTCGTAGGCACAGCTTTCGCTGCATGACTGATGAGCAGAAGGAGCGCGATGCTGGAGCAGCGGCGGCAGACAGGATTCGGACCGGTAGACATGTTCCTTCCGATACGGACGACGACGTACTCCGGCAGACGGCCGGCAAACGGCTCTCTCCTCCTG

>SG483932

CAGGCAACCCGCACTGGGTGGGTGCTTGGTGGCTGGGCGTGTTCATTGTGGGTGCGGCACTCATCGTTACGGCACTGCCCATGATGGCGTTCCCGCGCAACCTGCNNNAGCGACGGTCCTCGCAACGAGTGCCGCGGCCTGCCTTGGACCGCAATGGCGCCATCTGCCCCAAGCACCAGCTCCAGCAGAAGATGCCGCTCATCAGTTCACCGCCTGCTTCGCCGTCGGGAGCCAAGAAGGCCCTGAAAGACAAGCCCACACTCAGAGATTTCCCCACAGCCATTCGGCGGCTGTTGAAGAACGAGATCCTGCTGTACCGGACGGCCAGCAGTGTCCTGCACATCTTGCCAATCGCTGGCCTCTACACTTTTCTGCCTAAGTACTTGGAGAGCCAATTTCGGCTCACGGAGACCAAGGCCAACATGATTACAGGGTTTGCTGGCATTCTCGTCATGGGTGTGGGCATTTTTGCCAGTGGCACATTCATGCGCAAGTACAAGCCCAACGCCCGCTTTGTTGCCAAATGGATTGCATTCACTGCCCTGGCCTACTCCGTCGGCATGGTCATCCTCATGTGGGTGGGCTGTCCCCTCGGAGACTACGTTGGCCTCAATTCAGAACACAAAAGCCTAGACTATCTACCTCCAGCCTGCAACAGCACTTCTTGCGAGTGCAAGTCTGGGCTGTTCTCTCCAGTGTGCCATGATAGTGTGACCTACTTGTCGCCATGCCTGGCTGGCTGTTCACAAGTGTCTGGCAGTGATCAGCAGCCGAACTTCAGTGACTGCCATTGCACAGGAACAAACGAAACCATAACAAATGGGTTCTGCTCCCTTGAGTGCAACAACCTGACCTGGTACATCGTCATCTTCTCTGTTTTTGTACTCATCCACTCAACATCCGAAGTAGGGTCTATGCTACTGACCCTCAGATGTGTTGAATCCCATGACAAGGCCCTTGCATTGGGACTCATCCAGTTTGCCATTGGATTGTTTGGGAACGTGCCTTGTCCCATCATCTATGGTGCAGTCGTTGACTCTGCCTGCCTCTTCTGGGAAGACAACTGCGGAGAACCGGGCGCATGCCGCGTCTACGACCCGGCCAAGTTTCGTATGGTGTTCCATGGAGTCACAGCTGTCATCATGTTTGTGGCGTTCCTTGTGGATGCTGTTGTTTGGTACAAGGCGAGCTCCATCCACATTCACGAAGAGGAAGAGACCAGGGACACTGCGGTGGCAGCGGCAGCTCACGACGTGTCCTCTTCGACTCCACCAGTCCTGACGGCACATGCGGAAACAGAATCGTGCGTGTGAGCAGAGGTGGCTTTCCTCACGGCCGAGTGCTCTGGGTTGAGAAAAAAGGACGCCATGGTGGTCAGTGTTGGGCCTCTTCGATTTGTCTGTACTGGCAGAACCAGTTCGGTGGCAGAGCATTTGAAGCAGTTCAGTTTTCAGGGGCACCTGATTGTTCCTTTTTCACTACTCTTTGCCACCTACACAGCAAGCTTGCTAAGCTCTGTAGCCCATACTGGCAGTTTCACCGGAGATGATCGCCGTATGTGGTTCATCAGGGAGAGAGAATGCACTCACTGCCATGTGTCAGTGTGTGGCTTCACAACAGCTGTCTTCAAATGTCACACATCTTACTTGTCCCAGAGAAGTTGGCTGCAGTAGTTTTCAATTTCTACCAATGTTTGTTAGAATGGACCGAGCAGGTCGCAGTTTAGGGCTTGACATTTTTGAGTAGGAGCGTTACAACTGCTAAGGGCAGACATGCGAGCTAGAAAGAATGGAAAGACAGGGAGGTTAACTAGGCCACGTGCTGTGTTCGCTTGAGTACAGTTGTTAGCACCTGCTCCATTTCGTGTTGCAAGTGTTGCAGGCTTTTTGTGAGGTAATGTAGGGCTGCCAGAGTGTGAAACTATTGCAGTACTGGTCTGTCCTGTTTGAGCAAAATCGAAGAGACCCTCCCACATATTGTTTGCATCGCTGGACGATCGAGTGCATCTGTTTTTGTACAGAAATGACTCATTTCGCATGTTGCTTTGTTGTGTGGTGGTGATTGCCCTTGTGCCCACTGGGTGCTTTTGTTGCTGGTGCAGTGTGAGAAAGGCAGCTTCTGAAATGTGTGCACGGACTCTTGGTGTGGAGAGTGCTTTATTTTTTTTTTTTCTAGAGAGGTTGAGCTGCCAGGCTAGAAGTGAGCGAACTGTGTGAAATGATTTTGATGGGGTAAAATGTGTTGGCAAGATTTGAACTGCAAGCTGTCGAGAAAAGGACAGTCATTCGCGACATGAAAGCTCTGTGTTCGAGAAAGGAGAAAACGCACAATTACAGGAGAGCTTGTTTTTCTCTATTCTTTTACCCTTGTGAAAACGATATTTGCTTTTTTGTTTTCCTTGAGTTTTAGTAATATATGAGATCATTTCTTTGCGTAGTGTTCCTTTGTTTGGTGCGAAAGCTATGCGACTTTTTCATGCTATGCTGTTCATAAGCTTGTGACTTTAGACCAAGAAGAAAAATTTTCACTCATAATTTATCAAGGCTCTTGCTGCTGGCGTACCCAGCAGATGGCAAAGCAGCATCCTGAATTTCGCACAATCTTGCTTGGATGAGTTTGTTATGCCTGTTGAAAATATCTCAAAATTTTCATTGTTAGATATGGTTCTGATCTTAGGTTAATGGTTAAATCTGAACAGACATTTTGATGCTGTCTTTCGAGTGCCTCTCCTTAGTGCATATACGAAATGTGCATACAAAGCCTGCACTGTTAAATTGTGGTACTCTCGATGGAATCAAGACTAATGTTCATGTGCTTCAATCCAGCACTTGCACTTGCCTTAACTTACGTACAGTGAATTCAATCTGAAGTTGTACTATAAGATTTTCAGTGTTCATTGGGATGACGAACTTGCCTTTCTAAATTATTGATGGGAGTTTAGTGGCACATTTTATAAGAAATTCGGCTATTGCTGCCTGCTTGTTTGCTTGTGGCACTGGAATGGGGTAAACTGTGACATCTTAATGTAGCAAAATGAGATGCAGCTGTTATTATATCAAACGTGTCTGCTGTCTGTGTGAAGA

>SG486673

CTCGCCCCTCTACTTCGGAATGGGGCTTGCGTTCAGGCTCCTGGGTCCTGTTCTGGGATTTGTGACTGCCGGGGTTTGCCTGCGTTTTTACGAAGACCCCTTCCACGACCCGGGCATCACACCAAGGGACCCACGCTGGGTGGGTGCATGGTGGATGGGCTACATTCTGTTCGGCATCGGGCTGAGCATCGTCGCCCTGCCCATGATGCTGTTCCCGCGCATCCTGCCCGCCGGCAAGAACTACAAGGTGAACCGCCTGCAGAAGCTGTCCGCACTCAAGTCCCAGAAGGGCGCCAAGGGCGAGGAAGGACAGGAAGGGTCGGGAAAGCACGGATTCAGCATCGCCATGGTGAAAGACATAATGGTGATTCTAAAGCGGCTGGCAAGCAACCCGATCTTCATGTTTCACTCAATTGGCCTCATCTTCGTCCTGCTTGCCACGGCCGGCTACGGCACCTCGTTCAGCAAGTACGTCGAGTTCCAGTTCAGGCAGTCGGCGTCCAAGGCTAACTACTACACAGGGGCAGCGAAAGTGGTGACTACCCTCATCGGCATCGTCACCGGCGGCGTCGTGGTGCACCGGTTCCGACCACGGCCGCGCATCGTGGCAGGCTACTCAGCGTTCGTCGAGATCGCCATGATGGCGGGCTTCGTAGTCATGATGTTCATCGGCTGCGAAAGTCCCGTCATCGCGGGAGTCACTCCTGGTGGAAACGTCACCGCGAGTTCGCTGCTGGACACATGCAACGTGGACTGTAACTGCAACACGCAGATCTACGAGCCGGTGTGCTCGAGCAATAGGATGATCAGCTACTTCTCACCTTGCCATGCCGGCTGCAAAAGCGTGGGGACGACATCGTCCAACATGACGATATACAAAGACTGCAGCTGCGTCGCGCAAGGAAACCAAGGAGGGGACGACTCCTACGTGACCTCGGGGCTCTGTGGATCCTCGTGCCAGCAGCTGGGCCTCTTTCTGGGCATTGTGATCGCTGGCCAGTTCCTGGGCTCCACGGGCCGTGTAGGCGGCCTGCTGATCTTCCTGAGGTGCGTCGATCCTATCGATAAAAGCATGGCCCTGGGGACCACAGGTTCTCTGCTGAATATGCTTGCTTTCATCCCGTACCCTCTNNNNNNNNNNNNNNNNNNNNNNNNNNNNNNNNNNNNNNNNNNNNNNNNNNNNNNNNNNNNNNNNNNNNNNNNNNNNNNNNNNNTACGACGCGGACAAGCTGCGCTATTCGCTGCATCTCATCACGGTCATCTTCTTAGCCATAGCTGGTGCCTGCTACATTGGCCAGTTCCTGCTGAGCGGACGCATAAAGCGCTTCTACGAGGACGACCTTGCTGAGGACAGCACCGATGATCAGGACAAGTACGAGAGCAAGACTACCATCGCCGAGGCGCTCTCGGTTGAGAGCATCCAAAAGGCCGACGAGCTGCGAGAGGCCACCAGATTTTGAATCCCAGTGCAGCTTAACAGAAATGTTCTGGCGAATTCAATGGCGACAGAAAGTGAGCTCCAACCGCGAAGTTTCAGAGCGAAGTGAAGTCACTCTATGCACTAGAGACGGAAAGTTTCCACAGATTCCAACGACAANNNNNNNNNNNNNNNNNNNNNNNNNNNNNNNNNNNNNNNNNNNNNCCAGGATGGGTTCAAGGAGAGACAGAACACTGATGTGGGTTTCTCGACAACGAAAGAAAAACTGGAGGAAGCAGTGGCTTATTACAAGACAGTGAGTACGAAGAGTGTCAGAAGTCCGCTGCGAGCTACAGGGGCGTTGAATTTATCCTTTAATGAATGTGTTTCGCGTAGACATCCTGATCTTGTCAGCATGGGCACCGAAATGAACGATATCAAGGCACAGCACCTCCACCAGGGTGTGATTGCTTCACGCACCTGCTGTTTCAAGAACCTTTGTAGCGGCAAAAAATCAAGCGCAAGTTCTTCACACACACAANNNNNNNNNNNNNNNNNNNNNNNNNNNNNNNNNNNNNNNNNNNNNNNNNNNNNNNNNNNNNNNNNNNNNNNNNNNNNNNNNNNNNNNNNNNNNNNNNNNNNNTTTCAGTAGAACAAAAACAGAGCAGCTAAGGATAAGAGATGGTGAACAGTCATTGATTATGAAATATGTGCTGGGAATGGAGCACCCGATCAGCCTACACAGCGTATTCAAGAAAACATTACCAACATATGGTGTGAAAAAGTTCTGAACAAGCACCAG

>SG4813020

AGCAGAAGCAGGGCGTCGCCTGTGTCGTGCCAAGTGGAGACTCATCGCAGAGCCTGAATCACTTCGCGTGCTTTGACAGAGTTAAAACCACCAAAGCAATGGAAACCGGCGTCTGCCAAGACAGTGCGGATAGCAGTTCGCAGTCCGAGAGGAAGGAGTCTTCGGATGCTGGCGTTGACGGCCGTCGCTACCGCTGCGGGTGTGGACCTTTCCGCCCGAACTGGCTGCAAAGGTTCGCCACGCCACGTGTCTACGCAGTCGTCTTCGGACTGCTCGGCGTCAGCCAGGGAGCCTATCGCTCCTACATGGTCGGGACTCTGTCTAGCGTCGAGAAACGCTTCTCCATATCGAGCCGCGTTTCGAGCATCATCCTCATCGCCGACGACCTGAGTCCTCTTCTGGCCAGCGCCGTGTTCCTGCTGTTCCTGCGTCGGACAAGCATGCCCAACTGGATATCGGGAGGCATGCTCCTGTCGCTCGTAGGCGCACTGGTCAGCTACCTGCCATACGCTATATTCGGCGCAGGGAAGCACCTTCTGGAGCAGGCCAGCGCTCCGGAAAACTCGACGGCCCCGCCCCTCCAGTTTTGCAGTGAGGACGGCGGTAACGGCGGTGTCTTCAATCGTTCAGGCCACTGCGAAAGCCATCGATCGGATTGGTCTGCGGTGGGTGCCGTGACGCTTCTGTTCGCGGGGAACTTCTTGAATGGCCTGGGCGGCGTTGCCTGCTACGTCGTGGGCACTACGTACATGGACGACAACGTCAAGAAAAAGAACTCGGCCCTATATTTCGGAAGCATCTACATATGCCGCTTCATAGGGCCCGTCGTCGGGCTGACTTTGGGTTCCTTCTGCCTCAGCCACTACGAGGATCCTTGGGCGGAGCCGGGCATCAGCCCCGGGGACCCTCGCTGGATTGGGGCCTGGTGGATGGGCTACCTGGTGGTCGGCCTATGCCTCCTACTGAGCACCTTCCCAGTAGCCCTCTTCCCACGGACACTGCGGCGCTCATCGAAGATGGCCGGAGACGATGCCTCACTCGTCGATCCGGTGGTTTCCTGTTCGGCTGGCTCATCCTCGATAAGAGAGGAAGTCAAAGATGCCGTTCGAGTTTTGAGGAGACTCACAAGAAACCCGATCTACGTATTCAGGACGCTGGGAAACATAGCAGTGTATATAGCCCTGACTGGTTACTACGTCTCCTTCCCAAAATACACGCAGCACCAGTTCCATCAGACTGCTTCAAGGGCCAGTCTGTTCACAGGGCCCGTCGTGATTGTGTCGAACATGGTGGGAACCCTGGCGGGGGCCGTGTTCGTTCACCGTTGGCAGCCGCGGCCACGGATCATCGCCTGGCATAACGTACTCGTCACCCTCCTGGCCGTGCTGGGCATCTCGGCGCTCATGGCTGTAGACTGCGGCACACTCCGCTACCCGATCGCAGCGCACCACGAAACCGGAAGCAGTACCGTGGAAAACGCTTGCACGCGCGAATGCTCGTGCTCTCCAGACGTGCACCGGCCCGTGTGCGACGAGTCAACAGGGATGCAGTACTTCTCGCCGTGCTTCGCCGGGTGCAGCTCACAATTGGGAAACGAGAGCGAATTTAAAGACTGCCGCTGTTTGGTGAATGAAGGGGTGAAAGAGAACTTCTACAGCGGGGTCGTCGCGTCCGGCAAATGCGAGCAGGACTGCTCAAGCGCCCTGACGGCGTTCTCGGTGGTTGTGTTTGTCATCCAGGTCTGCTACTCAACCACCCTGGTTGGTTCAACGATACTTTTGATCAGGTCTCTGAACGTGAATGACAAAAGCGCAGCCCTGTCTATATTGTCCGCCATTATGAATATGTTTGCGTTCATACCGTACCCGCTCATCTACGGAGCCGTGGCGGACGCCAGCTGCCTGGTGTGGGAGGACCGATGCGGCGAGCGGGGCGCCTGCTGGCTCTACGACCTGCCAAAGTTCCGCTCCCTGCTGCACGGAGTCACCGCGGCTTTGCTTGTCGTCGGTTGCTTCTTCCAGGGTGCCGTGGTCCACTACAGCGACCGGGTGACGAATTTCTACGAGGACTCAGCGACGAGCGACGCGGACTCGGCGCCCAGCGCAACGCCACTCTTGCCAGCACAGCAAGCCACTGGCAGGGAAGCCTCCGCGTAGAAGGACGGCTCGCGGAGGGAAATACGCTCTGTCTCAGAAATAATTTGGCGAAAATGGCTCGCGTGCTTACAGAAGTGGAAGCAAGAGGTGGATTCGTGACCGAACCGCGGAATAAAACTGTCGAGATATTCTGCGCCATCCTGGCGTTTTATGCTGCATCAACACACTGGTTATTCACAGCGAACGTTTCAGCTCGGCTTTCGCAGTTCCCAGGAGCGTCGGTTAAGAAAAAGCTGAAGCGACAGTGCGATATCACGATGAACCCGACGGCACCTAAGCCAGTGCTGTGGCATGTTGCAAGTTGCGCGTCCGCGCCGTAAGTGTCGCCACGGTCGGAACGATTGCAGCGATCCTGGTATCTGGCGGCTCATGTGTTCTAGGTTTCCA

>SG4818037

GTGGCGGCCAGCGAAGGCATGCTGACCAGGAATGTGAAGACCATGCAGACGAACATGGCGATAAGGAAGAAGACGAGGAAGTTGCATTCGCTTGGACATTTGTCTCTCTCCGCCTGGATGGTCACTTGCTTCCCATTAATTGTTACGTTTCTGCCCGGATGATCAATGCAGCGGCAGTCCTCGTACACTTTTGTTGATCCATAGTGGTGCACCTCTTGGCAACCTGCAAAGCATGCGGAATAGTACATGACGTGGTCCGTTCCGCAGATGGGGTCAAAGTCTTCCATTTGGCAGTGGCACTGATTATTGCAGTCTAACAGGAAGCGGTCTATTCCATCGGTGACGTTGTTCGTCTTGTAATTGACGCCCGCGAATCTGACGTTGGGACAGCTGAAGAAGAATGCGAAGATGGTGACGAGTGGAACGAGAGAGCAGAGGACGCACATCTTGATGATGCCGGCGCAGCGCAGGTTGAACTTCTTCACGAAGTAGCCGCCTAGGAATGTGCCACCACCAGCGCCCGGAACTGTCACCGCACCCATTATTAGGGCCGCTAAACTGGCCGCGATGCTGAACTGGAACTCAATGACTTTAGGAAGGAATGTCGCCAGGCCAGATACCAGCATACCTTCGGTGGCTCCGGCCAAGCTTAGGAACACAAAGGTCGGGTTGGTGATGAGTATCTTGAAGGATGCCGGGAGATCCTTGGCGCGGGCCCCGAAGCCGCTTTGCACCGCTTCGCTCTTCTGAAGCTTCTGGTGCATCTCGGATTTCTTCTGAGCCTGCAGCTTTAATGAGCCTGGGAGGACCTTGGGGAAGGCACTAATGGGGACGGCAGCAAGGAGAGCCGTTACGGACGACACGACGAA

>SG4811655

GGAATGCCCTGCAATGAATCGTTTCCGGTTCCGGTGTGCGGGTCCAACGGCCGGGTGTACAACAGCGAATGCTCGCTGAGGAACGCCAACTGCCGGAAACAGCGGCGGCAGCGCGGGGGTCCCCTGGAGCCCGTTCGGGTGGTGGAGTGGGAGCGCTGCCGCGGCCGCCACCCGCTCTGCCCGGACAAGTGCCTCGACATCTACGACCCGGTGTGCGGAAAGGACGGCCGCTTCTACCCCAACCTGTGCATCATGCAGCGACGAAATTGCGGGAAGGTCGTCGGAACGCAAACACTGGCTGTCTGCATAGCCAGCGCCAGAGAGGCCCGCAAGCTGGACTCGTGCCCCCAGGACTGCTCGGAGCTGTACGAGCCCGTGTGCGGGTCCAACGGCGAGGTGTACCTGAACGAGTGCTTCTTCAAGAAGGAGACCTGCGGGAGCAAGGAGCACGTTCAGCTGGTGCCGCTGAACCGGTGCCTGGAGCCTCCCAAGTGCCCCAAGCGATGCCTGCCCATCCTGGACCCCGTGTGCGGATCGGACGGACAACGCTACCTAAACCATTGCCGCATGCAGCAGAGAAACTGCGGCCGGAATATCGTCGTCATGCCAAAAAGCTTCTGCTCGTGATCGTCACCTTGTCCTCATTGTTTTCATTTTTG

>SG4812339

TTTTTTTACTGCTCTAAGAGTATTTATTCAACAAGATAAATGTTGTACAAGCAGGTGGGGAAACTTAAAACGTGTATATAGCACTCAATAGATGCATTTATCACAACACATAATATTATATGGTTTGACTGGGAAAACCATCACTGGCAACTGTTCGACACATTGCATACGTATAGTATTTAGTTAGTCTGAAACATTGTTGTTCTTCTTGTGGTGATGCCCTTTCTTGTGCTTTGGTGCCTTGAGTTCACATGGAAAGCTGGCACAAGACCAGTTCCCACCAGCACAGACACAGTTACTGCAGTCGATCATTATTTCATCACCATCTTTATAGTACCGGTCCTCCAAAGGGCAGCTCTTGATAGGTGGACGGTACGATGGCCTCATGAAATTTTGGAACTCCCGCCTTGAGAGTAGCCAATCTGAATTTCTGTCAGCAACCTCCACCATGGCATCGATACACAGTGTAGCTGCCAGTTCACTTCTATGTTTCAGCCATGTGTGGAACATGGTGTTGTTCCTCCGGACACACTTTGAAAGCTCATGAGTGCTGAGGAAATCGTCACCGTCATGGTCACAGTCATCAAACACATCGTCAACCACTCTTGGGTAATGCCTGTCGTGAGAGACCTTAGATTCGAGAAGCTCCACAAAGTGCCTTCGAATGCGATCCCTCTCATGTTGGAAGCAAACAACTGGCTTTTGGTGATACTTGTGATGTGGCAACTTGACCTTGGGCTTTTTGCAGTGTCCCTTGTGCTTGATGGATATGTGCTTTTGGCCCAGGCAGGCGTCTTTGTGAAGCATACAGTGGTTGTCGTAAGTCACTCCATTAGTACCGCATACAGGCTTGTGGTGGGATGGGCAGTGCTGAACACACTGGCACGACGCCACTCCATTGTCCAAGATCCGACACACCCTTCCGGGCCGACATATAACGGCGGCGCAGATGTCCAGCTGACTATCTGTGATCTCATCTTTTACCGGCACCGATGTGACGCTTGCCCAACCAGCAGCGAAAAGCAACCACGCACTCAAGAACTTCATTGTAGGTGTTGCCAAGTGCCTCCGACGCGAGTGACGCCGCTCACGCGAACGGCAGTAATCAAATTGAGCTTGGCTCGGAAATATGATCGCACGATCCCGCAGCGGAAACCATGCCTTCGCGTATCCG

>SG4825334

CGCCGAAGCGCTGTGCGTGTGCTCGCGCCAGGAGCCCGTGTGCGGCACCGACGGCGTCACCTACGACAACGTGTGCCAGCTGACCGAGGCCCGGTACCGGCTGCGCAACGGGCTCGAGGCGGCCTCCAGGGGACCCTGCTACTCCGCGCCCCGAGTGGTGACCGCTCCGGAGAACACGCGCAACCGGACGGGTGGCCGCGCCGCCATGACCTGCGAGGTGTCCGGCTTCCCGGTGCCCACCATCGAGTGGCGCGTCGACCGCGGGGACGGACCGCTCAAGTCGCTGCCCACGGACAGCTCCCGCATCAACGTGCAGAGCCGCGGCGGACCGGACAGCTTCGAGGTGACCAGCTGGCTGCAGCTGCTCGACCTGAGGCC

>MG12024025

CCCCGCGCTGCCGCCGCTGTTTCCGGCAGTTGGCGTTCCTCAGCGAGCATTCGCTGTTGTACACCCGGCCGTTGGACCCGCACACCGGAACCGGAAACGATTCATTGCAGGGCATTCCGCAGGCAGAGACGCCGGGACTGCCATCAATTCGTGGTTGATCCACAGGTAACAATGGCTGCTCTGTAGCTGTGCCCGCGCTGCTGACTGCGTTGGAACCTGGCTGCTGGCTGTCTCCTTCTACCGTCGGTGGCAGCGCGGTCGTAGGCACAGCTTTCGCTGCATGACTGATGAGCAGAAGGAGCGCGATGCAGGAGCAGCGGCGGCAGACAGGATTCGGACCGGTAGACATGTTCCTTCCGATACGGACGACGACGTACTCCGGCAGACGGCCGGCAAACGGCTCTCTCCTCCTGTAGCGGCAGTCG

>MG120329

TATGCATGTATGCCACTTGGACTGCAAACTGTAATTCAGGACATTGATTCATTATTAAGTTCAAGAAACCAACATGCACGAATTCTCTCACCTTTTCAATATCCTATTTAGCACGAAAACATTCAACAAGCTGTGGTACAAGTAAATTTAACACGCATGCACTGAAACATGCTGCCTAAAGAAACCTCTGCTATGGAGTGCGTCAGCACACCTTATTGACAGCGTACTTTACTGGATTTTGGTTGCAACACTACATCAGTAACTTTGAGTTGGAGTCAACTGGAGGAGGAGACCAGAGTTAAAGCACAATACCTTGCAGGGAACACATGGGCCATGGTTATGTACAAACATAAAATTCAAACTGAACATAAACGATAATGCCAGAAATATGACATCCAGAAATATAATTTGCAACTGTGCAACCATGATACAAGTAAGTAGTGCTTAGTCAGTAGTATGCTGCGTTTTATTGTTTTGTGTTCACAATTCTGACAGCATATGATCTGCCTCACAGTTTATCACTATGACCTTAAAGTATTTGGTATGCAGGCTGCTGTTACCAGGTAGGAGAAAAGAAACAACAATAACCAACATGCTGACAACGACTTTTTCAGAAAAACTTCATTGAAAACTATTAAACAAGCTGGCGCTGAATATCACAGACACCAGACGGGCATACTAAAAGAAACTTAAAACAAAGCACCATGACAGCTGTCACTCGAATAAACAATGTGACTGTATTCTGCCCTGTTCACGGGAAATGTGGAACATTGCGTGATAAAAACACAACTCCGCAAGATTTCACAGTGAATGTTTCCCCATCGCATGGCACATACTGACACTCATGCCGACGGTTTTCAAGTTCATTTAAGAAACTACTTAGTGTCATGTGACTTTCAACAATCAAATATATCCTTCCATATTGTCGATCTATATACTTTTTGTATCATATTTTTTACAAAAATAAGGTCTCCTAAAGACCAGCAAACAACTTGGGGGATCACATCATGTATACCATGATCAGAAAAATGTATGTGTGTATGCAGGGTCTAACGATGGAAGAAGAAAGCACTGCAAATGTGAAACAGTACATAAACCAAAATGCGGCAAAGCATGCACCAAGAGGGCACCGAGATCAGACACTTCAAAAAAGCAGGTGCACTGGCTATTGTGTTCAAAATCATTTCCCCATAGTGCTTTGTGCATCCTTGTAGAGCCTGTGTATAAGGCGACAATTCTGCTAATTCACATAATATTTATCTCTTCATCTCTATCTTTCTCTGCCAAATGCTGGTCAGCAACTATAAAACAACACATTTTACACTTTTTAAAACAGCAACAGCAAATGTGCCTGTCTTTAAAAGGCAGGAAATGCAGAAAAATTACGTCGAATCTCAAGCACGTCCAAAAGAAAGATGTAATGACATGGCCTTCAGTGGTAAGCATGCTGGTTAAATACCATGAAAACATATGCCTAAAAGCAATTCCGCTAGGCAGGAACCAAAACAAAAAAAGAGCTTTTCGCAATTCAAAGCAAGACATGATTCATATTTTGCAAGCATGTGTGTTTCTTTGGTGAAAAATGTTTCAACAGGCTTACCAAAAAAATGCTTAATTTCTTTCTTCCGTAGGTGTCTCTTAAGCCACATGTTATAAAACTCTCTATAACACATCTCCAGCAACAACTAACATTCATACACACATACACATTTACAAACACACACACATATTTACAAAACACTTTTGATTTCGTAACATGAATTCATCAACAGCACCTTTAGTAAGTTTTTTTTTTTTGTGAGGAAGGGAATGCTTACACCGGAGCACATCTAGCAATGCATACCCACATGCTGCTATTCACATGCGCTTATTTCACATTCATGCACTTCAGATATGTGCATGCAAATACTGCCTGTGCACACAATACACACACAAGATAACTTCACTCCAAGATGCATTTCCATGCTTGTATGTTATGGATGTGTACTGTCAAAATTACAATGCAGCCTTTAGTAAACATCACCTAGCTAGGTTGCCTTAAAATCAAAATCAGCAGCCCCACCAATTTTGCCTAATTAGTCGTGGTATAGATTCAAATCTTGATCTAGAAAAGTTTCAGTGAAGGTGAGGAAATCAAGAACTTTGTTTAACTAAAAAGCATTTGAGTCTATCTTGATCTCATGGAGCTTAATTATTTCTTCCAAAAATTAAAAGACAATTTAAATTAGTTGCACAAACTGGCATTAAATGCACCCTCTGCACAGGGTCAGTATTAGCAAATGAGATGTTTTCACAAAAATATAAGAAAGCTACAGATGCCAGGAACATTAAAGATACTGCAAATGCAACTAATAAGAACAGTACACAAACAAAACCACATTCATGCCACAGGCACCTGCCGAAGTGAATAACTAGTACAGTGCAGTTGTGTCCTGCATTCTTCACACAGACAGCAGACACGTTTGATATAATAACAGCTGCATCTCATTTTGCTACATTAAGATGTCACAGTTTACCCCATTCCAGTGCCACAAGCAAANNNNNNNNNNNNNNNNTGAAAATCTTATAGTACAACTTCAGATTGAATTCACTGTACGTAAGTTAAGGCAAGTGCAAGTGCTGGATTGAAGCACATGAACATTAGTCTTGATTCCATCGAGAGTACCACAATTTAACAGTGCAGGCTTTGTATGCACATTTCGTATATGCACTAAGGAGAGGCACTCGAAAGACAGCATCAAAATGTCTGTTCAGATTTAACCATTAACCTAAGATCAGAACCATATCTAACAATGAAAATTTTGAGATATTTTCAACAGGCATAACAAACTCATCCAAGCAAGATTGTGCGAAATTCAGGATGCTGCTTTGCCATCTGCTGGGTACGCCAGCAGCAAGAGCCTTGATAAATTATGAGTGAAAATTTTTCTTCTTGGTCTAAAGTCACAAGCTTATGAACAGCATAGCATGAAAAAGTCGCATAGCTTTCGCACCAAACAAAGGAACACTACGCAAAGAAATGATCTCATATATTACTAAAACTCAAGGAAAACAAAAAAGCAAATATCGTTTTCACAAGGGTAAAAGAATAGAGAAAAACAAGCTCTCCTGTAATTGTGCGTTTTCTCCTTTCTCGAACACAGAGCTTTCATGTCGCGAATGACTGTCCTTTTCTCGACAGCTTGCAGTTCAAATCTTGCCAACACATTTTACCCCATCAAAATCATTTCACACAGTTCGCTCACTTCTAGCCTGGCAGCTCAACCTCTCTAGAAAAAAAAAAAATAAAGCACTCTCCACACCAAGAGTCCGTGCACACATTTCAGAAGCTGCCTTTCTCACACTGCACCAGCAACAAAAGCACCCAGTGGGCACAAGGGCAATCACCACCACACAACAAAGCAACATGCGAAATGAGTCATTTCTGTACAAAAACAGATGCACTCGATCGTCCAGCGATGCAAACAATATGTGGGAGGGTCTCTTCGATTTTGCTCAAACAGGACAGACCAGTACTGCAATAGTTTCACACTCTGGCAGCCCTACATTACCTCACAAAAAGCCTGCAACACTTGCAACACGAAATGGAGCAGGTGCTAACAACTGTACTCAAGCGAACACAGCACGTGGCCTAGTTAACCTCCCTGTCTTTCCATTCTTTCTAGCTCGCATCACTGCCCTTAGCAGTTGTAACGCTCCTACTCAAAAATGTCAAGCCCTAAACTGCGACCTGCTCGGTCCATTCTAACAAACATTGGTAGAAATTGAAAACTACTGCAGCCAACTTCTCTGGGACAAGTAAGATGTGTGACATTTGAAGACAGCTGTTGTGAAGCCACACACTGACACATGGCAGTGAGTGCATTCTCTCTCCCTGATGAACCACATACGGCGATCATCTCCGGTGAAACTGCCAGTATGGGCTACAGAGCTTAGCAAGCTTGCTGTGTAGGTGGCAAAGAGTAGTGAAAAAGGAACAATCAGGTGCCCCTGAAAACTGAACTGCTTCAAATGCTCTGCCACCGAACTGGTTCTGCCAGTACAGACAAATCGAAGAGGCCCAACACTGACCACCATGGCGTCCTTTTTTCTCAACCCAGAGCACTCGGCCGTGAGGAAAGCCACCTCTGCTCACACGCACGATTCTGTTTCCGCATGTGCCGTCAGGACTGGTGGAGTCGAAGAGGACACGTCGTGAGCTGCCGCTGCCACCGCAGTGTCCCTGGTCTCTTCCTCTTCGTGAATGTGGATGGAGCTCGCCTTGTACCAAACAACAGCATCCACAAGGAACGCCACAAACATGATGACAGCTGTGACTCCATGGAACACCATACGAAACTTGGCCGGGTCGTAGACGCGGCATGCGCCCGGTTCTCCGCAGTTGTCTTCCCAGAAGAGGCAGGCAGAGTCAACGACTGCACCATAGATGATGGGACAAGGCACGTTCCCAAACAATCCAATGGCAAACTGGATGAGTCCCAATGCAAGGGCCTTGTCATGGGATTCAACACATCTGAGGGTCAGTAGCATAGACCCTACTTCGGATGTTGAGTGGATGAGTACAAAAACAGAGAAGATGACGATGTACCAGGTCAGGTTGTTGCACTCAAGGGAGCAGAACCCATTTGTTATGGTTTCGTTTGTTCCTGTGCAATGGCAGTCACTGAAGTTCGGCTGCTGATCACTGCCAGACACTTGTGAACAGCCAGCCAGGCATGGCGACAAGTAGGTCACACTATCATGGCACACTGGAGAGAACAGCCCAGACTTGCACTCGCAAGAAGTGCTGTTGCAGGCTGGAGGTAGGTAGTCTAGGCTTTTGTGTTCTGAATTGAGGCCAACGTAGTCTCCGAGGGGACAGCCCACCCACATGAGGATGACCATGCCGACGGAGTAGGCCAGGGCAGTGAATGCAATCCATTTGGCAACAAAGCGGGCGTTGGGCTTGTACTTGCGCATGAATGTGCCACTGGCAAAAATGCCCACACCCATGACGAGAATGCCAGCAAACCCTGTAATCATGTTGGCCTTGGTCTCGGTGAGCCGAAACTGGCTCTCCAAGTACTTGGGCAGAAAAGTGTAGAGGCCAGCGATTGGCAAGATGTGCAGGACACTGCTGGCCGTCCGGTACAGCAGGATCTCGTTCTTCAACAGCCGCCGAATGGCTGTGGGGAAATCTNNNNNNNNNNNNNNNNNNNNNNNNNNNCTCTGAGTGTGGGCTTGTCTTTCAGGGCCTTCTTGGCTCCCGACGGCGAAGCAGGCGGTGAACTGATGAGCGGCATCTTCTGCTGGAGCTGGTGCTTGGGGCAGATGGCGCCATTGCGGTCCAAGGCAGGCCGCGGCACTCGTTGCGAGGACCGTCGCTGAGGCAGGTTGCGCGGGAACGCCATCATGGGCAGTGCCGTAACGATGAGTGCCGCACCCACAATGAACACGCCCAGCCACCAAGCACCCACCCAGTGCGGGTTGCCTGGTGACAGGTTGGATGTCTCAAATGGAAAGTTGACATAGACGCTAGTGCAGTAGGAGCCGAGAAGGAAGCCAAATACTGGACCAAATATCCTAACACCTATTGTGATACCGAAGTACAACGGTGACTCCCGGTTGGCAACGTTGTCGTCGATGTAGGGTATGCCAAGCGTGTAGACCGCTGTGGCACCGATGCCGATGAAGAGCAGGCTGATAAAGAACACCGAGAGCACCACGTGTGTGATGCGGTTCTGACTGAGCGGTCGCGCCGACGCATCGCAGATGGCTGTCTGCCGCAGCGCGTCGGCC

>MG1206673

GACTTGTCCTTCAAAAAGAGCACAATCTACAAAAAAGAACGTCTAGAAAGTTTCACAATGCTCGTAGCTAGAATGCAGTTTTTTCTTTCTGTGATCGGAATGGTGAGGTGCCTAAAAAAAGAAAAAGAAATGCCCCTTCGTTCAATATCGGCAATGCAGTGGACATGGTATCCGTCAGCAGCATCGACACATTTGCCAGAAGCCCATGATTCGCGCGCCATCATGCAACCCCACTCAAACTTAGAAGTCCTGAATATCACACCGCGAGTACAGTCCGCAGGCGTTGTAAAGCACGCCGGCGCTGTCATCACTTCATTCACGCTTGGATGATCACCAGACGAAGTCGGACAGGGTCCGAATTGCCGAGTTTTTGATGAACCAGTGTCTGCACAACTTTTGCTTGGAGGCGTCATGCTTGCATGCACCAGAATAAGAACAAAGTCCTTTTCGTTGCCTGCTTATCACGAGCCCCTACTTGCGCTGACGCCTGTAGAGAACTCGAGAGAACTTTACGGATTATGCTCGGAAAAAGTCGAACCACCGTGCCATTTGGACGATTTCATTGCGAAATTCACTGTCCTTGAGGTTCTGATTTATTACCCCATGAGCTGTGGGAGCGCATACAAGTCACAGTACTGAGGAGACAGATAGCTGCAAGAAAAGCCACGGTCGCAACCTACAGATGACCCTTTCGCATAATGTTCTTGCACCATACCCACAAGGTCGCACACACAGAGGTTCATGATGCCTCTCGCAGCTGCGAACAGCCAAGAACCGTGGAACCGCGAAGAACTACACCACTCCTTGCACACCAGCACGGCGAGCATCGCTGGGCCCCGATGCAAATGACCCCAAATACAACTTGCACTCTGTTTCTTTGCACTGTTCAGAACCACGTGCTGTTGTTGTCACTGCTGTGATTCTGCTGCTGTCCGGCGGCTGCACCGTTCGTGGCCGTCTTAAACGGAGCACCAGTCACCACATTCTCCTGCTTGATGGTGGCGACGCCAGACTTCTCAGGCGCCGCAGCCTGGTTGCCGTCGTCCTCTTCGTCCTCGCCCTCAGGTGCCTTGTAGAGTAGCCACGAGAGGAAGAAGAAGAGACAGGAGAGGAACTTGACGGTGGCAAGCAGCGCGAGCAGGTTGCGCGCCATCTGGCCATTCTCGTAGACGGCGCAGGCGCCGGAGTCATCGCACGAGCCGGGCCACAGGACGCAGCTCCGGTCGATCAGGTAGCCGAACATGATGGGCGCCGGTATGGTCCCTAGTAGCCGCACAGCAATCCACTGTATGCCGAGACCGAAAGACTTCTGGCTAGCTGCAACACACCTGAGTGTGGCGGCCAGCGAAGGCATGCTGACCAGGAATGTGAAGACCATGCAGACGAACATGGCGATAAGGAAGAAGACGAGGAAGTTGCATTCGCTTGGACATTTGTCTCTCTCCGCCTGGATGGTCACTTGCTTCCCATTAATTGTTACGTTTCTGCCCGGATGATCAATGCAGCGGCAGTCCTCGTACACTTTTGTTGATCCATAGTGGTGCACCTCTTGGCAACCTGCAAAGCATGCGGAATAGTACATGACGTGGTCCGTTCCGCAGATGGGGTCAAAGTCTTCCATTTGGCAGTGACACTGATTATTGCAGTCTAACAGGAAGCGGTCTATTCCATCGGTGACGTTGTTCGTCTTGTAATTGACGCCCGCGAATCTGACGTTCGGACAGCTGAAGAAGAATGCGAAGATGGTGACGAGTGGAACGAGAGAGCAGAGGACGCACATCTTGATGATGCCGGCGCAGCGCAGGTTGAACTTCTTCACGAAGTAGCCGCCTAGGAATGTGCCACCACCAGCGCCCGGAACTGTCACCGCACCCATTATTAGGGCCGCTAAACTGGCCGCGATGCTGAACTGGAACTCAATGACTTTAGGAAGGAATGTCGCCAGGCCAGATACCAGCATACCTTCGGTGGCTCCGGCCAAGCTTAGGAACACAAAGGTCGGGTTGGTGATGAGTATCTTGAAGGATGCCGGGAGATCCTTGGCGCGGGCCCCGAAGCCGCTCTGCACCGCTTCGCTCTTCTGAAGCTTCTGGTGCATCTCGGATTTCTTCTGAGCCTGCAGCTTTAATGAGCCTGGGAGGACCTTGGGGAAGGCACTAATGGGGACGGCAGCAAGAAGAGCCGTTACGGAGGACACGACGAAACCAATCCACCAGGCGCCCACCCAGACACCACTGGAGGGCGTCAAGCCTAATGCTGAAGCATCTACGGAGAGGTCGGTGTATATCTTCAGAAATTGGCCGCCTAGAATATAGCCAAGTGCAGGGCCGATGATCGCCATGGTATAGAAAATACCAATGTACACAGAGGACATCTTCGTAGACACGATCTCGTCCAGGTAAGTACAGCCGAGTGTGAAGAAGGGTGTCGCCCCGGCGCCATGCAGCATCTGTCCCGCGAGGAACATGTACTTGTACTGGTTCAGGGAGCCCACCTCCCTGCTCCCGCAGAAGCCCTCGGTGGCGTTGGCCGCCAGGCGGCAGAGGCTCTCCCGGTCGTCATCGGTGCTGAAGGCGTACGTACCGGCCAGGAAGTGCGGCAGCGAGAAGACGAAGGCACCGAGGCCCAGCACGAGGCAGCCGACGCCCACGAACAGCGGCTTGCTCCGGGTGCCGCCGAAGTAGCTGATGGGCGCCAGGAGCAGGAAGGACGCGATGTCGTACCCGCTGGCCACAAGGCCGCTCTCGGAGCTGCGCAGCTGGAAGCGCCGCTCGATGGTGGTGATGACCACGTTGACGAAGCCGTTCACGATGAGTCCCTGCAGGAACCCCGCCCAGCACAGGAAGAACAGCACCCACGGAGGCGTGCGGAACCGCTGGAGCCAGCT

>MG12014555

TTTCTTTAGCAACCCGTCTGTACGGGAATGAAATAAAGAACTGAAGAATTTAAAATTGCACTGGTATGTGGGAACGCTAGACAAGCTGAAAAACNNNNNNNNNNNNNNNNNNNNNNNNNNATTTTAATAATGCACCACATGAAATGCACGTAACATATCTCATACGCACGGACTAGGCATTCGTAAGGAACCGAAGAAAATATGAAACACAGTTTCCTGTTTCCGTCAGTTCAGAATCTGGTAGAAGGAACGATTTCGTGAGCATGGCTGGTGCAATACAGTCCAGCGTGGTTTTCAGTGGTAACACTGAAGTCAGGACACATTTAATAGAAACCACTTCTGCAGGCTAGCATTGTACAGGCAGTCCTGTTTCGTTGCCACTTATTGTTTTTCATGAAACGAAACCGCCAGAGAAACCATTTTCAGGGCATGCTCGTGACGCACCAGAAACTATGGTTCCTCAAGGTTGATCCTATTGCAGCTTACTGATTCAGATTTTAGATTGTCTCTCAAAGAAATCACCCTTCCATCTATCAAACACCAACGAGATAAAAAAAAAATGCACTGAGAAATTGTCAGCTCCTACGCAGAGCAGTCTGCAAGCAGTTCTTTCCCGCGTCAATCAACCAAGAGCACAGCAGAAACTCTCTTTCTTGCCGAGTGTTCTCAGAGGATACACCCCTCGGGGTGTTAATTTCACTCCTCGTCGGGGTCGTTTGTTGGCACCATGTGGAATATCTCGTTTCTCGTCAGTTTCCTGAAGGAGCCCTTCGATGACCTCCTGGACGAAGAGTGGAGGTCCGCCTCTCCCGGCGGCGGTACACGGCTCCTGTCTGCCAAGAAGCGCTTCTCTCGGCCGTAGAGGTTGCCCAGGCGACCGCTCAGCAGAAAGACTACGAAATTCAGTGCCACGCCAACCATGAGCAGGACGAAGGTGGTGCCGTGCAGCGAGTAGTTGAAGGTGCGCTGGTCGTACACCCAGCAGTTTCCCCGCTGGCCGCAGCTTTCCTCCCACACCACGCACGTCGAGTCCACCAATGAGCCGTACACCAACGGGTAGGGCAGGAACGAGAACAGTGCAAGGACTCCTTCAGCAAATCCGAGTGCAAATCCCTTGTCCTGCTGATCAACACATCTGAGTGGAATGACAATCTGCGCCGCCTTCGTGCTGAAGGAGAACCCTAAGGCGATGCACAAAAGCATAATGTAGAAGGTCAGGAAAGGACAGTCGCTTTCGCAGAACCCGTCTTTAGCCCTCCGAGCCGGACCGTCATTGTCAAAGGCGTCCACGCACTTGCAGTTGTCGTATGTCCCTCTCTCTGCTGTGCCGTTCGGTCCTTTGGTGGCGCATCCGGCAAAGCAAGGAGAAAAATACGTAGTCTTTCCCTCGCATATGGGATGGAACACTTCGGTAGCGCAGGAGCAGTTTTGGTTGCAGCTGTTGACCAGGTCTAATCTTGATCGGAGTTCGCCTTTTTCTCTCAGAGGAGGCTGTGGGCAGCTGAAAGCCATCAGTGAGATAAATATTAGAGCTGTGATAAGCTCCGTAGCGAAGATGATGGCTGTTAGTATGCGCGCGGAAGGCCGAAACGTCTTCATCAAGAACGCTCCACCCAGTGTGGCTACCATGGCACACCCGATCGGGAACGTTCCACTTAGGACTGAAGCAGCACTGGGTGACTGTTGGAAATGCTCTTGCACGTATTTAGGCACGTACGTACAAAAACCCAGGACACCGAACCACTTGAAGACGTGAGCGCCCATCTGAGCGACTATTATGGGGTTCTTGATAAGTCTCTTGGACGCTTCGAACGCTTCTCTTAGGCTCATGTCTTGCTCTTCCTCAGTCGGCTGGTCGGAATTTTCTTCAGCCAGACGCCTTGGGAAGAAGAAGAGGGGCAGGGAGAATAGCAGCTGCAGGAAGCCGAGAATGAAGAAGCCTAGCCACCAGGCTCCTATCCAACGCGGGTCTTCGTCCGCGAAGCCTGGGTCGTAATATGGGTCTTCGTAGATTGCGAGAATGAGGGCTGAGAAGATGAATCCCAGGCACGGTCCACATCCACGAACAACTGTCATGATCGCCATGTAGACGGCTGAGTTCTTCTTGCGGATGAGGTCGTCCAAGTAGGCTGTGCCTGCCGAGTAGTAAACGGCAGTGCCGATGCCCGTGAGGAAGTTGCAGCTGACCAGCAGCAGCAGCGCCGGAAGCGTCTCAGTCTTGCGCGACTGGGCGCAGTTGAGCTCTCGGTAGCGCCAGTCCTCTTCCTGGTCGTGGCAGAAGTGGTTCAGGTCCGACTCTACCTCGGGGTCCGTCATGCTGAACGCCTCGTCCGCCTTGCCGAACAGCCAGTAGGGCAGCGAGGCCAGAAGGCACGAGGCCCCCATGAGCACAGCTGCGCCTCCCACCCATCTTGGCCGGTGCCTCTTTGGACTGTAGGTGCCGATGAAGACACCCAGAAGAATAGGACCGAAGTTGTCGGCGAAGAAGACGAAACCTGCTGTGCGGCTCCCGTAGGCGAACCGCCTTTCCAGCGTCGTGTTGACGCCGATGAGGTAGGAGTAGTAGCAGCCTTGCAGGATACCCAGGGCAGAGAAACAGATCATGTAGCACACCGGTTTAGTGAAGAAGGCCAGGAAGGCTCGCAGTGCCCCCTTCTTCTCCGCCTCCTTAGAGCTGCCNNNNNNNNNNNNNNNNNNNNNNNNNNNNNNNNNNNNNNNNNNNNNNNNNNNNNNNNNNNNNNNNNNNNNNNNNNNNNNNNNNNNNNNNNNNNNNNNNCTTTTCTCCAAAATTTGTGCTCAGCTGGCTCTTTCGGCGCATGGGCAATGACGGCGGTTTATGGCGACGTCAGGCCGGCCCATTCGCAGCAGTCAAGGCGTCCCGACAGGGTGCTGCCGATTTCCACACCGATTAT

>MG12034117

GCGGCGCCGGAGACGGTGGCCCTGCGGAAATCAGTCGCGACTGCGCTAGCCAGGCGACGGAGAGCTATCTGCCCTTTATGATTCTCTTCGTGGCGTCTTTCCTGAACGGCTTCGGCCAGAGCGTTCTGCAAGTCGCCGGCAGTAGCTACATCGACGACAGCGTCAAGAAGAAGAGCTCGCCGTTGTTTTTTGGGGCAAGCTTCTCCGTACGGTCCATTGGTCCAGCACTGGGATTCCTTGCTGCCAGCGTCTGCCTCTCACTCTACGAGGATCCCTTCCTGAGTCCGGACATTCCGACCACGGACCCGCGATGGGTTGGCTGCTGGTGNNNNNGCTACGTGTTCTGGGGCTGCCTGCTGCTGCTGGCGAGCGTTCCCGNNNNNNNNNNNNNNNNNNCGATGCCGGCCCGTCTGCCGGATCCGCCGCTGGTCTTCAAGAAGCGAGCCGACTCCAGCCACATCTCGGAGATCAGCAAATCGTTGACCCGGCTGCTCCGGAAGCGAGTGTACGTGCTGCAGTTGTTCGTGTCCATGCTGATGTACAGCGGCCTGCAAGGATACACCATGTTCTCGGCAAAGTACATGGAGGTCGAGTTTCGCAACTCTGCGGCCAGGGCCAGCGCGTTCGCTGGGCTGATCTCGTCGGTGTTCAACGTCACGAGCTTCCTGTTGTCGGGCGTGGTGATCCACCGACTGAGGCCGCCGCCCAAGGTGCTCGCCTGGTACAATGTGGTCGTCACCTTCGTCGTCTCCTGCGGATTCGTCGTGGCGATGCTCGTCAAGTGCGACTACGGAACCATGCCCGGGGTATCGGTCGTCCAAGGGAACCTTGACCTGAACAACAAGTGCAACGAAGAATGTTACTGCACCCTTCAGAGTTATCAGCCCGTGTGCGAACCCGTCGGAGGCACCGTATACTTCTCTCCGTGCTTTGCTGGATGCCAAAAGCCCGAGGGAGAGGTGGGAGCTAACTTGACGAAGCTGACCAACTGCAACTGCCTGAAGACGTTCGACGACAGCGACTTCTTCTCAGGAAACGCCGTGGTTGGGTTCTGCAAGGGCACCTGCTCCATGTTCGTGCAGTTCATCATCATAGTCTCGCTCGTGCAGTTCATGGGCATGTCCACGTCCGTGAGCCACACGCTCTTCATGCTTCGGAGCATAAGCCCAAGTGACAAGACCATCGCCCTTGGACTGGCCAATGCTCTGGCGAATTTGCTGTCGTACATTCCTTACCCGCTCATCTACGGCGCCGTGATCGACAGCTCTTGCCAGGTGTGGGAGAGCGCCTGCGGAACGAGCGGCAACTGCTGGCTGTACGACCTAACGCGATTGCGCCACTCGTACCTGGGCACGTCGGCCGGCTTCCTGGCCGTGTCGGGCATCTTCTCGATCGCCGTCGCCCTGGTGGCGGGTGACCTGAAGGACTTCTACGGGGACGCCTACGTGCAGGTGTCCCACTTGGGTGGCGCTGACTTCGAAGCACGGAACATCGGCGTCGAGAA

>MG1206792

GCCGGAGACCCCGCCGAAGCGCTGTGCGTGTGCTCGCGCCAGGAGCCCGTGTGCGGCACCGACGGCGTCACCTACGACAACGTGTGCCAGCTGACCGAGGCCCGGTACCGGCTGCGCAACGGGCTCGAGGCGGCCTCCAGGGGACCCTGCTACTCCGCGCCCCGAGTGGTGACCGCTCCGGAGAACACGCGCAACCGGACGGGTGGCCGCGCCGCCATGACCTGCGAGGTGTCCGGCTTCCCGGTGCCCACCATCGAGTGGCGCGTCGACCGCGGGGACGGACCGCTCAAGTCGCTGCCCACGGACAGCTCCCGCATCAACGTGCAGAGCCGCGGCGGACCGGACAGCTTCGAGGTGACCAGCTGGCTGCAGCTGCTCGACCTGAGGCCAGAGGACACGGCCACCTACTGGTGCGTGGGCGCCAACGAGAACGGAGAGGCCTCGGCCGCCGCCAAGCTCAACGTGCTGCCCTAGGCCGCCCCTCGTCCTTGTTGTTGACTCCTCCTCCTTGTTGTTGAACGCCATCCATACTTGCATTACACACCCGCGCGTCGTCGTCTCGTTTAATGTGTTC

>MG1202584

CTCTTTGAAACGGATACGCGAAGGCATGGTTTCCGCTGCGGGATCGTGCGATCATATTTCCGAGCCAAGCTCAATTTGATTACTGCCGTTCGCGTGAGCGGCGTCACTCGCGTCGGAGGCACTTGGCAACACCTACAATGAAGTTCTTGAGTGCGTGGTTGCTTTTCGCTGCTGGTTGGGCAAGCGTCACATCGGTGCCGGTAAAAGATGAGATCACAGATAGTCAGCTGGACATCTGCGCCGCCGTTATATGTCGGCCCGGAAGGGTGTGTCGGATCTTGGACAATGGAGTGGCGTCGTGCCAGTGTGTTCAGCACTGCCCATCCCACCACAAGCCTGTATGCGGTACTAATGGAGTGACTTACGACAACCACTGTATGCTTCACAAAGACGCCTGCCTGGGCCAAAAGCACATATCCATCAAGCACAAGGGACACTGCAAAAAGCCCAAGGTCAAGTTGCCACATCACAAGTATCACCAAAAGCCAGTTGTTTGCTTCCAACATGAGAGGGATCGCATTCGAAGGCACTTTGTGGAGCTTCTCGAATCTAAGGTCTCTCACGACAGGCATTACCCAAGAGTGGTTGACGATGTGTTTGATGACTGTGACCATGACGGTGACGATTTCCTCAGCACTCATGAGCTTTCAAAGTGTGTCCGGAGGAACAACACCATGTTCCACACATGGCTGAAACATAGAAGTGAACTGGCAGCTACACTGTGTATCGATGCCATGGTGGAGGTTGCTGACAGAAATTCAGATTGGCTACTCTCAAGGCGGGAGTTCCAAAATTTCATGAGGCCATCGTACCGTCCACCTATCAAGAGCTGCCCTTTGGAGGACCGGTACTATAAAGATGGTGATGAAATAATGATCGACTGCAGTAACTGTGTCTGTGCTGGTGGGAACTGGTCTTGTGCCAGCTTTCCATGTGAACTCAAGGCACCAAAGCACAAGAAAGGGCATCACCACAAGAAGAACAACAATGTTTCAGACTAACTAAATACTATACGTATGCAATGTGTCGAACAGTTGCCAGTGATGGTTTTCCCAGTCAAACCATATAATATTATGTGTTGTGATAAATGCATCTATTGAGTGCTATATACACGTTTTAAGTTTCCCCACCTGCTTGTACAACATTTATCTTGTTGAATAAATACTCTTAGAGCAGTAATCTAAAGTATGTGCTCAATTTTCATATCAGACGTTAGTATTTACAGACTACTAGTAGTTCCATTGTTAAT

>MG12022812

GTCCCCTGGAGCCCGTTCGGGTGGTGGAGTGGGAGCGCTGCCGCGGCCGCCACCCGCTCTGCCCGGACAAGTGCCTCGACATCTACGACCCGGTGTGCGGAAAGGACGGCCGCTTCTACCCCAACCTGTGCATCATGCAGCGACGAAATTGCGGGAAGGTCGTCGGAACGCAAACACTGGCTGTCTGCATAGCCAGCGCCAGAGAGGCCCGCAAGCTGGACTCGTGCCCCCAGGACTGCTCGGAGCTGTACGAGCCCGTGTGCGGGTCCAACGGCGAGGTGTACCTGAACGAGTGCTTCTTCAAGAAGGAGACCTGCGGGAGCAAGGAGCACGTTCAGCTGGTGCCGCTGAACCGGTGCCTGGAGCCTCCCAAGTGCCCCAAGCGATGCCTGCCCATCCTGGACCCCGTGTGCGGATCGGACGGACAACGCTACCTAAACCACTGCCGCATGCAACAGAGAAACTGCGGCCGGAATATCGTCGTCATGCCAAAAAGCTTCTGCTCGTGATCGTCACCTTGTCCTCATTGTTTTCATTTTTGTTTGGTTGCCATTTTACGAG

>MG961043

CCGGTGGCCGACGCGCTGCGGCAGACAGCCATCTGCGATGCGTCGGCGCGACCGCTCAGTCAGAACCGCATCACACACGTGGTGCTCTCGGTGTTCTTTATCAGCCTGCTCTTCATCGGCATCGGTGCCACAGCGGTCTACACGCTTGGCATACCCTACATCGACGACAACGTTGCCAACCGGGAGTCACCGTTGTACTTCGGTATCACAATAGGTGTTAGGATATTTGGTCCAGTATTTGGCTTCCTTCTCGGCTCCTACTGCACTAGCGTCTATGTCAACTTTCCGTTTGAGACATCCAACCTGTCACCAGGCAACCCGCACTGGGTGGGTGCTTGGTGGCTGGGCGTGTTCATTGTGGGTGCGGCACTCATCGTTACGGCACTGCCCATGATGGCGTTCCCGCGCAACCTGCCTCAGCGGCGGTCCTCGCAACGAGTGCCGCGGCCTGCCTTGGACCGCAATGGCGCCATCTGCCCCAAGCACCAGCTCCAGCAGAAGATGCCGCTCATCAGTTCACCGCCTGCTTCGCCGTCGGGAGCCAAGAAGGCCCTGAAAGACAAGCCCACACTCAGAGATTTCCCCACAGCCATTCGGCGGCTGTTGAAGAACGAGATCCTGCTGTACCGGACGGCCAGCAGTGTCCTGCACATCTTGCCAATCGCTGGCCTCTACACTTTTCTGCCTAAGTACTTGGAGAGCCAGTTTCGGCTCACCGAGACCAAGGCCAACATGATTACAGGGTTTGCTGGCATTCTCGTCATGGGTGTGGGCATTTTTGCCAGTGGCACATTCATGCGCAAGTACAAGCCCAACGCCCGCTTTGTTGCCAAATGGATTGCATTCACTGCCCTGGCCTACTCCGTCGGCATGGTCATCCTCATGTGGGTGGGCTGTCCCCTCGGAGACTACGTTGGCCTCAATTCAGAACACAAAAGCCTAGACTACCTACCTCCAGCCTGCAACAGCACTTCTTGCGAGTGCAAGTCTGGGCTGTTCTCTCCAGTGTGCCATGATAGTGTGACCTACTTGTCGCCATGCCTGGCTGGCTGTTCACAAGTGTCTGGCAGTGATCAGCAGCCGAACTTCAGTGACTGCCATTGCACAGGAACAAATGAAACCATAACAAATGGGTTCTGCTCCCTTGAGTGCAACAACCTGACCTGGTACATCGTCATCTTCTCTGTTTTTGTACTCATCCACTCAACATCCGAAGTAGGGTCTATGCTACTGACCCTCAGATGTGTTGAATCCCATGACAAGGCCCTTGCATTGGGACTCATCCAGTTTGCCATTGGATTGTTTGGGAACGTGCCTTGTCCCATCATCTATGGTGCAGTCGTTGACTCTGCCTGCCTCTTCTGGGAAGACAACTGCGGAGAACCGGGCGCATGTCGCGTCTACGACCCGGCCAAGTTTCGTATGGTGTTCCATGGAGTCACAGCTGTCATCATGTTTGTGGCGTTCCTTGTGGATGCTGTTGTTTGGTACAAGGCGAGCTCCATCCACATTCACGAAGAGGAAGAGACCAGGGACACTGCGGTGGCAGCGGCAGCTCACGACGTGTCCTCTTCGACTCCACCAGTCCTGACGGCACATGCGGAAACAGAATCGTGCGTGTGAGCAGAGGTGGCTTTCCTCACGGCCGAGTGCTCTGGGTTGAGAAAAAAGGACGCCATGGTGGTCAGTGTTGGGCCTCTTCGATTTGTCTGTACTGGCAGAACCAGTTCGGTGGCAGAGCATTTGAAGCAGTTCAGTTTTCAGGGGCACCTGATTGTTCCTTTTTCACTACTCTTTGCCACCTACACAGCAAGCTTGCTAAGCTCTGTAGCCCATACTGGCAGTTTCACCGGAGATGATCGCCGTATGTGGTTCATCAGGGAGAGAGAATGCACTCACTGCCATGTGTCAGTGTGTGGCTTCACAACAGCTGTCTTCAAATGTCACACATCTTACTTGTCCCAGAGAAGTTGGCTGCAGTAGTTTTCAATTTCTACCAATGTTTGTTAGAATGGACCGAGCAGGTCGCAGTTTAGGGCTTGACATTTTTGAGTAGGAGCGTTACAACTGCTAAGGGCAGTGATGCGAGCTAGAAAGAATGGAAAGACAGGGAGGTTAACTAGGCCACGTGCTGTGTTCGCTTGAGTACAGTTGTTAGCACCTGCTCCATTTCGTGTTGCAAGTGTTGCAGGCTTTTTGTGAGGTAATGTAGGGCTGCCAGAGTGTGAAACTATTGCAGTACTGGTCTGTCCTGTTTGAGCAAAATCGAAGAGACCCTCCCACATATTGTTTGCATCGCTGGACGATCGAGTGCATCTGTTTTTGTACAGAAATGACTCATTTCGCATGTTGCTTTGTTGTGTGGTGGTGATTGCCCTTGTGCCCACTGGGTGCTTTTGTTGCTGGTGCAGTGTGAGAAAGGCAGCTTCTGAAATGTGTGCACGGACTCTTGGTGTGGAGAGTGCTTTATTTTTTTTTTTTCTAGAGAGGTTGAGCTGCCAGGCTAGAAGTGAGCGAACTGTGTGAAATGATTTTGATGGGGTAAAATGTGTTGGCAAGATTTGAACTGCAAGCTGTCGAGAAAAGGACAGTCATTCGCGACATGAAAGCTCTGTGTTCGAGAAAGGAGAAAACGCACAATTACAGGAGAGCTTGTTTTTCTCTATTCTTTTACCCTTGTGAAAACGATATTTGCTTTTTTGTTTTCCTTGAGTTTTAGTAATATATGAGATCATTTCTTTGCGTAGTGTTCCTTTGTTTGGTGCGAAAGCTATGCGACTTTTTCATGCTATGCTGTTCATAAGCTTGTGACTTTAGACCAAGAAGAAAAATTTTCACTCATAATTTATCAAGGCTCTTGCTGCTGGGGTACCCAGCAGATGGCAAAGCAGCATCCTGAATTTCGCACAATCTTGCTTGGATGAGTTTGTTATGCCTGTTGAAAATATCTCAAAATTTTCATTGTTAGATATGGTTCTGATCTTAGGTTAATGGTTAAATCTGAACAGACATTTT

>MG962187

CGGGCCGTGCGGCGAGGACCTGGAGTGCCGGCTGCGCCGCGACCTGGCCCCCGGAGACCCCGCCGAAGCGCTGTGCGTGTGCTCGCGCCAGGAGCCCGTGTGCGGCACCGACGGCGTCACCTACGACAACGTGTGCCAGCTGACCGAGGCCCGGTACCGGCTGCGCAACGGGCTCGAGGCGGCCTCCAGGGGACCCTGCTACTCCGCGCCCCGAGTGGTGACCGCTCCGGAGAACACGCGCAACCGGACGGGTGGCCGCGCCGCCATGACCTGCGAGGTGTCCGGCTTCCCGGTGCCCACCATCGAGTGGCGCGTCGACCGCGGGGACGGACCGCTCAAGTCGCTGCCCACGGACAGCTCCCGCATCAACGTGCAGAGCCGCGGCGGACCGGACAGCTTCGAGGTGACCAGCTGGCTGCAGCTGCTCGACCTGAGGCCAGAGGACACGGCCACCTACTGGTGCGTGGGCGCCAACGAGAACGGAGAGGCCTCGGCCGCCGCCAAGCTCAACGTGCTGCCCTAGGCCGCCCCTCGTCCTTGTTGTTGACTCCTCCTCCTTGTTGTTGAACGCCATCCATACTTGCATTACACACCCGCGCGTCGTCGTCTCGTTTAATGTGTTCGTTCACTTCGTTTTATATTCTTTTTTTTCGTCGTCGTCATACGTGCTAGTGGTTCTTCTTCCGTTTTTCTTCTCGAGAACCGTTCGTGTGCATCGTTTCAACGGCAGCTGATCACGGCTCCTGATGGCCTGCAGAGCCCAGACCAAAGCTCATTAGACAATTTTGTGCACCATTCGTCTCTGTACCTAAACGAGTGCGTCAAGCGATCGCTTTTGCAACTCGCGGTCTATTTTGCCTATTTTGCGCTCCGTGAACAGTTTGAGGTCGAGAGGAAGTAAACGAAAAAACAACAAAACCATGCAGGGCTATCAAAATACGTGTTTATATATCACGGATGTACTTAGTAACAATGTATTCTATGCATCCTGGCGGAAGCCATTGAAACTGGGACCGAATAGTGAACAAATAGTCGAAGCACTGGACCAAAAGCAAGAAAAAAAAAAGGAATATGACGAGCATGCCTGCGTATCTGTCGAGGAGCAATTCTAACGCGAAGACGCCGTTTTATCCACAACCATATAACTGCATTTACCAGCGCCAGCGACAAACGTACCAAAAACGTCGCAGCTGAGATTCTAGTCGCGTCCCACTTGACACTGCCAACATGCCTCGCACCATTCCTGCGGGGACGGGGAGGCCGCTACACACGTAGTCTTCTCGAGTCTTCGGTAGATCGCGTGCAAACTCAAAATACGCCTTCCAGGGCAGCCGCATGCTCTGGTGTCAACTGGTGTTCCACTGTTGGCGTGTGCGTTTCTGCACCTAAGTGCTGCCATTTTGCTTGTTTATTTGTTGGAATTGGTGGTCGCTTGCGCGGTGTACTTTCGCTTTGTTTTAGAAAAAAATACTGCAATAAAGTGACGTTTACAAACTTAAAAAAAAAAATACTGCAATAAAG

>MG962416

GTTGTTCGTGGATGTGGACCGTGCCTGGGATTCATCTTCTCAGCCCTCATTCTCGCAATCTACGAAGACCCATATTACGACCCAGGCTTCGCGGACGAAGACCCGCGTTGGATAGGAGCCTGGTGGCTAGGCTTCTTCATTCTCGGCTTCCTGCAGCTGCTATTCTCCGTGCCGCTCTTCTTCTTCCCAAGGCGTCTGGCTGAAGAAAATTCTGACCAGCCGACTGAGGAAGAGCAAGACATGAGCCTAAGAGAAGCCTTCGAAGCTTCCAAGAGACTTATCAAGAACCCCATAATAGTCGCTCAGATGGGCGCTCACGTCTTCAAGTGGTTCGGTGTCCTGGGTTTTTGTACGTACGTGCCTAAATACGTGCAAGAGCATTTCCAACAGTCACCCAGTGCTGCTTCAGTCCTAAGTGGAACGTTCCCGATCGGGTGTGCCATGGTAGCCACACTGGGTGGAGCGTTCTTGATGAAGACGTTTCGGCCTTCCGCGCGCATACTAACAGCCATCATCTTCGCTACGGAGCTTATCACAGCTCTAATATTTATCTCACTGATGGCTTTCAGCTGCCCACAGCCTCCCCTGAGAGAAAAAGGCGAACTCCGATCAAGATTAGACCTGGTCAACAGCTGCAACCAAAACTGCTCCTGCGCTACCGAAGTGTTCCATCCCATATGCGAGGGAAAGACTACGTATTTTTCTCCTTGCTTTGCCGGATGCGCCACCAAAGGACCGAACGGCACAGCAGAGAGAGGGACATACGACAACTGCAAGTGCGTGGACGCCTTTGACAATGACGGTCCGGCTCGGAGGGCTAAAGACGGGTTCTGCGAAAGCGACTGTCCTTTCCTGACCTTCTACATTATGCTTTTGTGCATCGCCTTAGGGTTCTCCTTCAGCACGAAGGCGGCACAGATTGTCATTCCACTCAGATGCGTTGATCAGCAGGACAAGGCATTTGCACTCGGATTCGCTGAAGGAGTCCTTGCACTGTTCTCGTTCCTGCCCTACCCGTTGGTGTACGGCTCATTGGTGGACTCGACGTGCGTGGTGTGGGAGGAAAGCTGCGGCCAGCGGGGAAACTGCTGGGTGTACGACCAGCGCACCTTCAACTACTCGCTGCACGGCACCACCTTCGTCCTGCTCATGGTTGGCGTGGCACTGAATTTCGTAGTCTTTCTGCTGAGCGGTCGCCTGGGCAACCTCTACGGCCGAGAGAAGCGCTTCTTGGCGGACAGGAGCCGTGTACCGCCGCCGGGAGAGGCGGACCTCCACTCTTCGTCCAGGAGGTCATCGAAGGGCTCCTTCAGGAAACTGACGAGAAACGAGATATTCCACATGGTGCCCACAAACGACCCCGACGAGGAGTGAAATTAACACCCCGAGGGGTGTATCCTCTGGGAACACTCGGCAAGAAAGAGAGTTTCTGCTGTGCTCTTGGTTGATTGACGCGGGAAAGAACTGCTTGCAGACTGCTCTGCGTAGGAGCTGACAATTTCTCAGTGCATTTTTTTTATCTCGTTGGTGTTTGATAGATGGAAGGGTGATTTTTTTGAGAGACAATCTAAAATCTGAATCAGTAAGCTGCAATAGGATCAACCTTGAGGAACCATAGTTTCTGGTGCGTCACGAGCATGCCCTGAAAATGGTTTCTCTGGCGGTTTCGTTTCATGAAAAACAATGAGTGGCAACGAAACAGGCTCTTGAAGGACTGCCTGTACAATGCTAGCCTGCAGAAGTGGTTTCTATTAAATGTGTCCTGACTTCAGTGTTACCACTGAAAACCACGCTGGACTGTATTGCACCAGCCATGCTCACGAAATCGTTCCTTCTACCAGATTCTGAACTGACGGAAACAGGAAACTGTGTTTCATATTTTCTTCGGTTCCTTACGAATGCCTAGTCCGTGCGTATGAGGTATGTTACGTGCATTTCATGTGGTGCATTATTAAAATAACTTACGTCTCTTGAAAAAAAA

>MG962599

TGTAGAGTAGCCACGAGAGGAAGAAGAAGAGACAGGAGAGGAACTTGACGGTGGCAAGCAGCGCGAGCAGGTTGCGCGCCATCTGGCCATTCTCGTAGACGGCGCAGGCGCCGGAGTCATCGCACGAGCCGGGCCACAGCACGCAGCTGCGGTCGATCAGGTAGCCGAACATGATGGGCGCCGGTATGGTCCCTAGTAGCCGCACAGCAATCCACTGTATGCCGAGACCGAAAGACTTCTGGCTAGCTGCAACACACCTGAGTGTGGCGGCCAGCGAAGGCATGCTGACCAGGAATGTGAAGACCATGCAGACGAACATGGCGATAAGGAAGAAGACGAGGAAGTTGCATTCGCTTGGACATTTGTCTCTCTCCGCCTGGATGGTCACTTGCTTCCCATTAATTGTTACGTTTCTGCCTGGATGATCAATGCAGCGGCAGTCCTCGTACACTTTTGTTGACCCGTAGTGGTGCACCTCTTGGCAACCTGCAAAGCATGCGGAATAGTACATGACGTGGTCCGTTCCGCAGATGGGGTCAAAGTCTTCCATTTGGCAGTGACACTGATTATTGCAGTCTAACAGGAAGCGGTCTATTCCATCGGTGACGTTGTTCGTCTTGTAATTGACGCCCGCGAATCTGACGTTCGGACAGCTGAAGAAGAATGCGAAGATGGTGACGAGTGGAACGAGAGAGCAGAGGACGCACATCTTGATGATGCCGGCGCAGCGCAGGTTGAACTTCTTCACGAAGTAGCCGCCTAGGAATGTGCCACCACCAGCGCCCGGAACTGTCACCGCACCCATTATTAGGGCCGCTAAACTGGCCGCTATGCTGAACTGGAACTCAATGACTTTAGGAAGGAATGTCGCCAGGCCAGATACCAGCATACCTTCGGTGGCTCCGGCCAAGCTTAGGAACACAAAGGTCGGGTTGGTGATGAGTATCTTGAAGGATGCCGGGAGATCCTTGGCGCGGGCCCCGAAGCCGCTTTGCACCGCTTCGCTCTTCTGAAGCTTCTGGTGCATCTCGGATTTCTTCTGAGCCTGCAGCTTTAATGAACCTGGGAGGACCTTGGGGAAGGCACTAATGGGGACGGCAGCAAGAAGAGCCGTTACGGAGGACACGACGAAACCAATCCACCAGGCGCCCACCCAGACACCACTGGAGGGCGTCAAGCCTAATGCTGAAGCATCTACGGAGAGGTCGGTGTATATCTTCAGAAATTGGCCGCCTAGAATATAGCCAAGTGCAGGGCCGATGATCGCCATGGTATAGAAAATACCAATGTACACAGAGGACATCTTCGTAGACACGATCTCGTCCAGGTAAGTACAGCCGAGTGTGAAGAAGGGTGTCGCCCCGGCGCCATGCAGCATCTG

>MG963068

TCTTTTTTTTTACTGCTCTAAGAGTATTTATTCAACAAGATAAATGTTGTACAAGCAGGTGGGGAAACTTAAAACGTGTATATAGCACTCAATAGATGCATTTATCACAACACATAATATTATATGGTTTGACTGGGAAAACCATCACTGGCAACTGTTCGACACATTGCATACGTATAGTATTTAGTTAGTCTGAAACATTGTTGTTCTTCTTGTGGTGATGCCCTTTCTTGTGCTTTGGTGCCTTGAGTTCACATGGAAAGCTGGCACAAGACCAGTTCCCACCAGCACAGACACAGTTACTGCAGTCGATCATTATTTCATCACCATCTTTATAGTACCGGTCCTCCAAAGGGCAGCTCTTGATAGGTGGACGGTACGATGGCCTCATGAAATTTTGGAACTCCCGCCTTGAGAGTAGCCAATCTGAATTTCTGTCAGCAACCTCCACCATGGCATCGATACACAGTGTGGCTGCCAGTTCACTTCTATGTTTCAGCCATGTGTGGAACATGGTGTTGTTCCTCCGGACACACTTTGAAAGCTCATGAGTGCTGAGGAAATCGTCACCGTCATGGTCACAGTCATCAAACACATCGTCAACCACTCTTGGGTAATGCCTGTCGTGAGAGACCTTAGATTCGAGAAGCTCCACAAAGTGCCTTCGAATGCGATCCCTCTCATGTTGGAAGCAAACAACTGGCTTTTGGTGATACTTGTGATGTGGCAACTTGACCTTGGGCTTTTTGCAGTGTCCCTTGTGCTTGATGGATATGTGTTTTTGGCCCAGGCAGGCATCTTTGTGAAGCATACAGTGGTTGTCGTAAGTCACTCCATTAGTACCGCATACAGGCTTGTGGTGGGATGGGCAGTGCTGAACACACTGGCACGACGCCACTCCATTGTCCAAGATCCGACACACCCTTCCGGGCCGACATATAACGGCGGCGCAGATGTCCAGCTGACTATCTGTGATCTCATCTTTTACCGGCACCGATGTGACGCTTGCCCAACCAGCAGCGAAAAGCAACCACGCACTCAAGAACTTCATTGTAGGTGTTGCCAAGTGCCTCCGACGCGAGTGACGCCGCTCACGCGAACGGCAGTAATCAAATTGAGCTTGGCTCGGAAATATGATCGCACGATCCCGCAGCGGAAACCATGCCTTCGCG

>MG9610759

GTCGAGTTTCGCAACTCTGCGGCCAGGGCCAGCGCGTTCGCTGGGCTGATCTCGTCGGTGTTCAACGTCACGAGCTTCCTGTTGTCGGGCGTGGTGATCCACCGACTGAGGCCGCCGCCCAAGGTGCTGGCCTGGTACAACGTGGTCGTCACCTTCGTCGTCTCCTGCGGATTCGTCGTGGCGATGCTCGTCAAGTGCGACTACGGAACCATGCCCGGGGTATCGGTCGTCCAAGGGAACCTTGACCTGAACAACAAGTGCAACGAAGAATGTTACTGCACCCTTCAGAGTTATCAGCCCGTGTGCGAACCCGTCGGAGGCACCGTATACTTCTCTCCTTGCTTTGCTGGATGCCAAAAGCCCGAGGGAGAGGTGGGAGCTAACTTGACGAAGCTGACCAACTGCAACTGCCTGAAGACGTTCGACGACAGCGACTTCTTCTCAGGAAACGCCGTGGTTGGGTTCTGCAAGGGCACCTGCTCCATGTTCGTGCAGTTCATCATCATAGTCTCGCTGGTGCAGTTCATGGGCATGTCCACGTCCGTGAGCCACACGCTCTTCATGCTTCGGAGCATAAGCCCAAGTGACAAGACCATCGCCCTTGGACTGGCCAATGCTCTGGCGAATTTGCTGTCGTACATTCCTTACCCGCTCATCTACGGCGCCGTGATCGACAGCTCTTGCCAGGTGTGGGAGAGCGCCTGCGGAACGAGCGGCAACTGCTGGCTGTACGACCTAACGCGATTGCGCCACTCGTACCTGGGCACGTCGGCCGGCTTCCTGGCCGTGTCGGGCATCTTCTCGATCGCCGTCGCCCTGGTGGCGGGTGACCTGAAGGACTTCTACGGGGACGCCTACGTACAGGTGTCCCACTTGGGTGGCGCTGACTTCGAAGCACGGAACATCGGCGTCGAGAAACGCAATAAGACTGTCGCCAAACAGGAGAGCAACGGTCACCGCCCCAAGTGACCGCCGGGAGACCCGACGGTGTAGCACGTTGTCGGAGACGTGTTACTGTTATTCCATTTATGGACGCTAACCAAAGCTTTTTCGAGGTTCTTCGACAGTCTAGTGCTAGCAGCCGCTGGAGCCAATCAACTTCTGTGGCTTCTTGCAGCGGAAAGGTTCCTGCGGCGGTCGCATTTCAGTTGAGGAAGGACGCAACGTCTATG

>MG9612755

CCCGGACAAGTGCCTCGACATCTACGACCCGGTGTGCGGAAAGGACGGCCGCTTCTACCCCAACCTGTGCATCATGCAGCGACGAAATTGCGGGAAGGTCGTCGGAACGCAAACACTGGCTGTCTGCATAGCCAGCGCCAGAGAGGCCCGCAAGCTGGACTCGTGCCCCCAGGACTGCTCGGAGCTGTACGAGCCCGTGTGCGGGTCCAACGGCGAGGTGTACCTGAACGAGTGCTTTTTCAAGAAGGAGACCTGCGGGAGCAAGGAGCACGTTCAGATGGTGCCGCTGAACCGGTGCCTGGAGCCTCCCAAGTGCCCCAAGCGATGCCTGCCCATCCTGGACCCCGTGTGCGGATCGGACGGACAACGCTACCTAAACCATTGCCGCATGCAGCAGAGAAACTGCGGCCGGAATATCGTCGTCATGCCAAAAAGCTTCTGCTCGTGATCGTCACCTTGTCCTCATTGTTTTCATTTTTGTTGGTTGCCATTTTACGAGATTCAATAAACTTTTTCGAAAAAAAAAAATTTTTCTCAAAAAAAA

>MG9618659

GTCAAGCAGTCGGTCACCCATGGCCTGCAGGTCATCATTAGTGCACCCTTTATCCTTGCTGACAACAGGGGAGGAAACCTTGTTCGTCATGACTGAATTATGACCATCCTTGCTGGCCCACGAGTCCTTGGAACTGAACTTTGACTTCAGTCTGTTGTTGAAGGCAGCAGGCTTTCCCGTGGCATCTTTATTGGCTTTCTTCTCCTTGTCAATAGTGTTTTGTAGCTTGGCCATATAGCGGTCCCAGCGTGCTTTCTGACGGCTCTCACGCTGCTGTTCTTCTAGGCTTTTCACAGCTTTGCATGGACAGAATCCCTTGCAGGAGACCTGCACTGAGGTACGCTGGCGGCAGTTGTGGTACTGCAGCCGACACTCAGAAGAATAAGTCTGGTTGTCCGTGCCGCACACATAGTCCGCCTTGAACACGGGGCACGGTGGGCACCGTTCCATTTCCTGGCTGCTGTCATCTTCCAGGTACTGGTCATCATCGTCATCGTCTTCTGCCTCTGCACTGCTCCACGGCTTGGCTGTGCTGCTCACTGCAGCAGCACTATCAGTGCCAGCTGCAGTCGTGGTAAGTGGCATAGCTGTGCTCTGCTGTCTA

>MG9632964

CCGACTGCCGCTACAGGAGGAGAGAGCCGTTTGCCGGCCGTCTGCCGGAGTACGTCGTCGTCCGTATCGGAAGGAACATGTCTACCGGTCCGAATCCTGTCTGCTGCCGCTGCTCCAGCATCGCGCTCCTTCTGCTCATCAGTCATGCAGCGAAAGCTGTGCCTACGACCGCGCTGCCACCGACGGTAGAAGGAGACAGCCAGCAGCCAGGTTCCAACGCAGTCAGCAGCGCGGGCACAGCTACAGAACAGCCATTGGTACCTGTGGATCAACCACGAATTGATGGCAGTCCCGGCGTCTCTGCCTGCGGAATGCCCTGCAATGAATCGTTTCCGGTTCCGGTGTGCGGGTCCAACGGCCGGGTGTACAACAGCGAATGCTCGCTGAGGAACGCCAACTGCCGGAAACAGCGGCGGCAGCGCGGGG

>MG48225

TTGCCTTCCTCTTTGAAACGGATACGCGAAGGCATGGTTTCCGCTGCGGGATCGTGCGATCGTATTTCCGAGCCAAGCTCAATTTGATTACTGCCGTTCGCGTGAGCGGCGTCACTCGCGTCGGAGGCACTTGGCAACACCTACAATGAAGTTCTTGAGTGCGTGGTTGCTTTTCGCTGCTGGTTGGGCAAGCGTCACATCGGTGCCGGTAAAAGATGAGATCACAGATAGTCAGCTGGACATCTGCGCCGCCGTTATATGTCGGCCCGGAAGGGTGTGTCGGATCTTGGACAATGGAGTGGCGTCGTGCCAGTGTGTTCAGCACTGCCCATCCCACCACAAGCCTGTATGCGGTACTAATGGAGTGACTTACGACAACCACTGTATGCTTCACAAAGACGCCTGCCTGGGCCAAAAGCACATATCCATCAAGCACAAGGGACACTGCAAAAAGCCCAAGGTCAAGTTGCCACATCACAAGTATCACCAAAAGCCAGTTGTTTGCTTCCAACATGAGAGGGATCGCATTCGAAGGCACTTTGTGGAGCTTCTCGAATCTAAGGTCTCTCACGACAGGCATTACCCAAGAGTGGTTGACGATGTGTTTGATGACTGTGACCATGACGGTGACGATTTCCTCAGCACTCATGAGCTTTCAAAGTGTGTCCGGAGGAACAACACCATGTTCCACACATGGCTGAAACATAGAAGTGAACTGGCAGCTACACTGTGTATCGATGCCATGGTGGAGGTTGCTGACAGAAATTCAGATTGGCTACTCTCAAGGCGGGAGTTCCAAAATTTCATGAGGCCATCGTACCGTCCACCTATCAAGAGCTGCCCTTTGGAGGACCGGTACTATAAAGATGGTGATGAAATAATGATCGACTGCAGTAACTGTGTCTGTGCTGGTGGGAACTGGTCTTGTGCCAGCTTTCCATGTGAACTCAAGGCACCAAAGCACAAGAAAGGGCATCACCACAAGAAGAACAACAATGTTTCAGACTAACTAAATACTATACGTATGCAATGTGTCGAACAGTTGCCAGTGATGGTTTTCCCAGTCAAACCATATAATATTATGTGTTGTGATAAATGCATCTATTGAGTGCTATATACACGTTTTAAGTTTCCCCACCTGCTTGTACAACATTTATCTTGTTGAATAAATACTCTTAGAGCAGTAAAAAAAA

>MG481981

CCGATGCAAATGACCCCAAATACAACTTGCACTCTGTTTCTTTGCACTGTTCAGAACCACGTGCTGTTGTTGTCACTGCTGTGATTCTGCTGCTGTCCGGCGGCTGCACCGTTCGTGGCCGTCTTAAACGGAGCACCAGTCACAACAGTCTCCTGCTTGATGGTGGCGACGCCAGACTTCTCAGGCACCGCAGCCTGGTTGCCGTCGTCCTCTTCGTCCTCGCCCTCGGGCGCCTTGTAGAGCAGCCACGAGAGGAAGAAGAAGAGACAGGAGAGGAACTTGACGGTGGCAAGCAGCGCGAGCAGGTTGCGCGCCATCTGGCCATTCTCGTAGACGGCGCAGGCGCCGGAGTCATCGCACGAGCCGGGCCACAGCACGCAGCTGCGGTCGATCAGGTAGCCGAACATGATGGGCGCCGGTATGGTCCCTAGTAGCCGCACAGCAATCCACTGTATGCCGAGACCGAAAGACTTCTGGCTAGCTGCAACACACCTGAGTGTGGCGGCCAGCGAAGGCATGCTGACCAGGAATGTGAAGACCATGCAGACGAACATGGCGATAAGGAAGAAGACGAGGAAGTTGCATTCGCTTGGACATTTGTCTCTCTCCGCCTGGATGGTCACTTGCTTCCCATTAATTGTTACGTTTCTGCCCGGATGATCAATGCAGCGGCAGTCCTCGTACACTTTTGTTGATCCATAGTGGTGCACCTCTTGGCAACCTGCAAAGCATGCGGAATAGTACATGACGTGGTCCGTTCCGCAGATGGGGTCAAAGTCTTCCATTTGGCAGTGACACTGATTATTGCAGTCTAACAGGAAGCGGTCTATTCCATCGGTGACGTTGTTCGTCTTGTAATTGACGCCCGCGAATCTGACGTTGGGACAGCTGAAGAAGAATGCGAAGATGGTGACGAGTGGAACGAGAGAGCAGAGGACGCACATCTTGATGATGCCGGCGCAGCGCAGGTTGAACTTCTTCACGAAGTAGCCGCCTAGGAATGTGCCACCACCAGCGCCCGGAACTGTCACCGCACCCATTATTAGGGCCGCTAAACTGGCCGCGATGCTGAACTGGAACTCAATGACTTTAGGAAGGAATGTCGCCAGGCCTGATACCAGCATACCTTCGGTGGCTCCGGCCAAGCTTAGGAACACAAAGGTCGGGTTGGTGATGAGTATCTTGAAGGATGCCGGGAGATCCTTGGCGCGGGCCCCGAAGCCGCTTTGCACCGCTTCGCTCTTCTGAAGCTTCTGGTGCATCTCGGATTTCTTCTGGGCCTGCAGCTTTAATGAACCTGGGAGGACCTTGGGGAAGGCACTAATGGGGACGGCAGCAAGAAGAGCCGTTACGGAGGACACGACGAAACCAATCCACCAGGCGCCCACCCAGACACCACTGGAGGGCGTCAAGCCTAATGCTGAAGCATCTACGGAGAGGTCGGTGTATATCTTCAGAAATTGGCCGCCTAGAATATAGCCAAGTGCAGGGCCGATGATCGCCATGGTATAGAAAATACCAATGTACACAGAGGACATCTTCGTAGACACGATCTCGTCCAGGTAAGTACAGCCGAGTGTGAAGAAGGGTGTCGCCCCTGCACCGTGCAGCATCTGTCCCGCGAGGAACATGTACTTGTACTGGTTCAGGGAGCCCACCTCCCTGCTCCCGCAGAAACCCTCGGTGGCGTTGGCCGCCAGGCGGCAGAGGCTCTCACGGTCGTCCTCGGTGCTGAAGGCGTACGTACCGGCCAGGAAGTGCGGCAGCGAGAAGACGAAGGCACCGAGGCCCAGCACGAGGCAGCCGACGCCCACGAACAGCGGCTTGCTCCGGGTGCCGCCGAAGTAGCTGATGGGCGCCAGGAGCAGGAAGGACGCGATGTCGTACCCGCTGGCCACAAGGCCGCTCTCGGAGCTGCGCAGCTGGAAGCGCCGCTCGATGGTGGTGATGACCACGTTGACGAAGCCGTTCACGATGAGTCCCTGCAGGAACCCCGCCCAGCACAGGAAGAACAGCACCCACGGAGGCGTGCGGAAACGCTGGAGCCAGCTGGGTCGCAGGCAACCCCAACCGCAGGTCCCGCCCACGTCTTCGTCATAGTCGGCGACGATTCCCCTAGGA

>MG482463

ACAGCACCCGCATGTGGCATGACGGTCTTCGCCATTGAGCGGGTCTTCGTCTTCCTGTCCTCCTCCATCTTCTGTTTTCTCGCATCCCGCGGGCCTCTCAGTCGCGCTGCCATCAACTAGGCAAACGGCCTCCGAGTCACCCGCTGCTGCTTCGGCAGTGATTAGCTTGTCCTTCTGCGAAGCCTTCGTTGTCGTGTCGGAGTAGAAGTCGGCCAAGCGGCCGCTGTGGTAGACGAGCCCCACCTCAAAGAGTATTGCGGAGAGCAAAATGGCGACAGACAAGCCGTGGTAGGTGTAGCGCAGCCGGTCCGCGTCGTAAATCCAGCAAACGCCCGGTCGGCCACATTTATCCTCCCAGACGAGGCATGATGAGTCGAAGATAGCGCCATAGAGTATCGGGTACGGAATGTACGTGAATATGCTGCTCAATCCTTCCTGGACGCCAAGTGCCATGGCCTTCTCCTCGTGGGACACGCACCTGAGTCCGACGATAGTGTGTCCCACAGTTGTGGTTCGCGCGATGAAGCCGACCAGCGACGACAGCGCGATGAATGCCAGCACGTTGCTGCACGTGTCCCCGCTGCAAAGCCCTAAGTACGGAGCCCCTTGGTCGGCTTGCACAAGCTGCTCCTTTAGCTGGCCCCGCTGTAGTTGGCTGGGCAGGTTGGGCAAGCACGAGCACTCGTCGAAGGACACTTCCCCAGAATCTGTAGCGGAAAACTTGGTGCAGCCCGCATGGCACGGAGAGAAGAAAACAGTACGGTTGACTGGGTCGCAAAGCGGCTGGAAGTGCCTGGTGGTGCAGTTGCAGCTATCACTGCAAGCCAGCTGTATTGACGACCCGTGGCCAGCGACGGTGTTGACGGTGCCCGCGAGTCGTACGGCTTCGCAACCGATAAAGGCGCACGCCACGAGGCAAGCCAGCCTGAGCACGTCACACAGCGTCGTGTACAAGGTGACCAGCCGGGGCCGGGGCCTGAAAGCGTGCAGCGCCAGGCCGCCCACACCCAGGCCAAGCACGTTGGTGATCATAAGCACAGGACCGATAAGGAAGCTGGCCTGAGATGCGGACGTCCTGAACTGGACCTCGACGTACTTGGAGCTCATCATGGCGTGCCCAAGGTTAGCATTGCTCGCCAGCAGCCAGTACACGAGTCGGAACACGTACGCCGGGTTCCGGAACAGAGAGCGCACGGAGCACCAGAAGCCAACCTTTTTTCCAGCCTCATCGCCCTTC

>MG483557

AGGGGCGGCCTAGGGCAGCACGTTGAGCTTGGCGGCGGCCGAGGCCTCTCCGTTCTCGTTGGCGCCCACGCACCAGTAGGTGGCCGTGTCCTCTGGCCTCAGGTCGAGCAGCTGCAGCCAGCTGGTCACCTCGAAGCTGTCCGGTCCGCCGCGGCTCTGCACGTTGATGCGGGAGCTGTCCGTGGGCAGCGACTTGAGCGGTCCGTCCCCGCGGTCGACGCGCCACTCGATGGTGGGCACCGGGAAGCCGGACACCTCGCAGGTCATGGCGGCGCGGCCACCCGTCCGGTTGCGCGTGTTCTCCGGAGCGGTCACCACTCGGGGCGCGGAGTAGCAGGGTCCCCTGGAGGCCGCCTCGAGCCCGTTGCGCAGCCGGTACCGGGCCTCGGTCAGCTGGCACACGTTGTCGTAGGTGACGCCGTCGGTGCCGCACACGGGCTCCTGGCGCGAGCACACGCACAGCGCTTCGGCGGGGTCTCCGGGGGCCAGGTCGCGG

>MG487234

ATTTTAATAATGCACCACATGAAATGCACGTAACATACCTCATACGCACGGACTAGGCATTCGTAAGGAACCGAAGAAAATATGAAACACAGTTTCCTGTTTCCGTCAGTTCAGAATCTGGTAGAAGGAACGATTTCGTGAGCATGGCTGGTGCAATACAGTCCAGCGTGGTTTTCAGTGCTAACACTGAAGTCAGGACACTATTAACAGAAACCACTTCTGCAGGCTAGCATTGTACAGGCAGTCCTTCAAGAGCCTGTTTCGTTGCCACTCATTGTTTTTCATGAAACGAAACCGCCAGAGAAACCATTTTCAGGGCATGCTCGTGACGCACCAGAAACTATGGTTCCTCAAGGTTGATCCTATTGCAGCTTACTGATTCAGATTTTAGATTGTCTCTCAAAAAAATCACCCTTCCATCTATCAAACACCAACGAGATAAAAAAAATGCACTGAGAAATTGTCAGCTCCTACGCAGAGCAGTCTGCAAGCAGTTCTTTCCCGCGTCAATCAACCAAGAGCACAGCAGAAACTCTCTTTCTTGCCGAGTGTTCNNNNNNNNNNNNNNNNNNNNNNNNNNNNNNNNNNNNNNNNNNNNNNNNNNNNNNNNNNNNNNNNNNNNNNGGCACCATGTGGAATATCTCGTTCCTCGTCAGTTTCCTGAAGGAGCCCTTCGATGACCTCCTGGACGAAGAGTGGAGGTCCGCCTCTCCCGGCGGCGGTACACGGCTCCTGTCGGCCAAGAAGCGCTTCTCTCGGCCGTAGAGGTTGCCCAGGCGACCGCTCAGCAGAAAGACTACGAAATTCAGTGCCACGCCAACCATGAGCAGGACGAAGGTGGTGCCGTGCAGCGAGTAGTTGAAGGTGCGCTGGTCGTACACCCAGCAGTTTCCCCGCTGGCCGCAGCTTTCCTCCCACACCACGCACGTCGAGTCCACCAACGAGCCGTACACCAACGGGTAGGGCAGGAACGAGAAAAGTGCAAGGACTCCTTCAGCGAATCCGAGTGCAAATGCCTTGTCCTGCTGATCAACGCATCTGAGTGGAATGACAATCTGTGCCGCCTTCGTGCTGAAGGAGAACCCTAAGGCGATGCACAAAAGCATAATGTAGAAGGTCAGGAAAGGACAGTCGCTTTCGCAGAACCCGTCTTTAGCCCTCCGAGCCGGACCGTCATTGTCAAAGGCGTCCACGCACTTGCAGTTGTCGTATGTCCCTCTCTCTGCTGTGCCGTTCGGTCCTTTGGTGGCGCATCCGGCAAAGCAAGGAGAAAAATACGTAGTCTTTCCCTCGCATATGGGATGGAACACTTCGGTAGCGCAGGAGCAGTTTTGGTTGCAGCTGTTGACCAGGTCTAATCTTGATCGGAGTTCGCCTTTTTCTCTCAGAGGAGGCTGTGGGCAGCTGAAAGCCATCAGTGAGATAAATATTAGAGCTGTGATAAGCTCCGTAGCGAAGATGATGGCTGTTAGTATGCGCGCGGAAGGCCGAAACGTCTTCATCAAGAACGCTCCACCCAGTGTGGCTACCATGGCACACCCGATCGGGAACGTTCCACTTAGGACTGAAGCAGCACTGGGTGACTGTTGGAAATGCTCTTGCACGTATTTAGGCACGTACGTACAAAAACCCAGGACACCGAACCACTTGAAGACGTGAGCGCCCATCTGAGCGACTATTATGGGGTTCTTGATAAGTCTCTTGGAAGCTTCGAAGGCTTCTCTTAGGCTCATGTCTTGCTCTTCCTCAGTCGGCTGGTCAGAATTTTCTTCAGCCAGACGCCTTGGGAAGAAGAAGAGCGGCACGGAGAATAGCAGCTGCAGGAAGCCGAGAATGAAGAAGCCTAGCCACCAGGCTCCTATCCAACGCGGGTCTTCGTCCGCGAAGCCTGGGTCGTAATATGGGTCTTCGTAGATTGCGAGAATGAGGGCTGAGAAGATGAATCCCAGGCACGGTCCACATCCACGAACAACTGTCATGATCGCCATGTAGACGGCTGAGTTCTTCTTGCGGATGAGGTCGTCCAAGTAGGCTGTGCCTGCCGAGTAGTAGACGGCAGTGCCGATGCCCGTGAGGAAGTTGCAGCTGACCAGCAGCAGCAGCGCCGGAAGCGTCTCGGTCTTGCGCGACTGGGCGCAGTTGAGCTCTCGGTAGCGCCGGTCCTCTTCCTGGTCGTGGCAGAAGTGGTTCAGGTCCGACTCGACCTCGGGGTCCGTCATGCTGAACGCCTCGTCCGCCTTGCCGAACAGCCAGTAGGGCAGCGAGGCCAGAAGGCACGAGGCCCCCATGAGCACAGCTGCGCCTCCCACCCATCTTGGCCGGTGCCTCTTTGGACTGTAGGTGCCGATGAAGACACCCAGAAGAATAGGACCGAAGTTGTCGGCGAAGAAGACGAAACCTGCTGTACGGCTTCCGTAGGCGAACCGCCTTTCCAGCGTCGTGTTGACGCCGATGAGGTAGGAGTAGTAGCAGCCTTGCAGGATACCCAGGGCAGAGAAACAGATCATGTAGCACACCGGTTTAGTGAAGAAGGCCAGGAAGGCTCGCAGTGCCCCCTTCTTCTCCGCCTCCTTAGAGCTGCCACCGCGCTCCGCGGTTGGAGCCATGACTCAGCAGTTGCGAAGAACGCTGTTAACAGCACCTTTCCCTTTTCTCCAAAATTTGTGCTCAGCTGGCTCTTTCGGCGCATGGGCAATGACGGCGGTTTATGGCGACGTCAGGCCGGCCCATTCGCAGCAGTCAAGGCGTCCCGACAGGGTGCTGCCGATTTCCACACCGATTATCAGGGAACGAGATACCACATTGTGCCGTGTCTCCGGC

>MG4811798

TCTGGCGCTGGCTATGCAGACAGCCAGTGTTTGCGTTCCGACGACCTTCCCGCAATTTCGTCGCTGCATGATGCACAGGTTGGGGTAGAAGCGGCCGTCCTTTCCGCACACCGGGTCGTAGATGTCGAGGCACTTGTCCGGGCAGAGCGGGTGGCGGCCGCGGCAGCGCTCCCACTCCACCACACGAACGGGCTCCAGGGGACCCCCGCGCTGCCGCCGCTGTTTCCGGCAGTTGGCGTTCCTCAGCGAGCATTCGCTGTTGTACACCCGGCCGTTGGACCCGCACACCGGAACCGGAAACGATTCATTGCAGGGCATTCCGCAGGCAGAGACGCCGGGACTGCCATCAATTCGTGGTTGATCCACAGGTACCAATGGCTGTTCTGTAGCTGTGCCCGCGCTGCTGACTGCGTTGGAACCTGGCTGCTGGCTGTCTCCTTCTACCGTCGGTGGCAGCGCGGTCGTAGGCACAGCTTTCGCTGCATGACTGATGAGCAGAAGGAGCGCGATGCTGGAGCAGCGGCGGCAGACAGGATTCGGACCGGTAGACATGTTCCTTCCGATACGGACGACGACGTACTCCGGCAGACGGCCGGCAAACGGCTCTCTCCTCCTGTAGCGGCAGTCGGGC

>MG4815040

CTGATCTCGTCGGTGTTCAACGTGACGAGCTTCCTGTTGTCGGGCGTGGTGATCCACCGACTGAGGCCGCCGCCCAAGGTGCTGGCCTGGTACAACGTGGTCGTCACCTTCGTCGTCTCCTGCGGATTCGTCGTGGCGATGCTCGTCAAGTGCGACTACGGAACCATGCCCGGGGTATCGGTCGTCCAAGGGAACCTTGACCTGAACAACAAGTGCAACGAAGAATGTTACTGCACCCTTCAGAGTTATCAGCCCGTGTGCGAACCCGTCGGAGGCACTGTATACTTCTCTCCTTGCTTTGCTGGATGCCAAAAGCCCGAGGGAGAGGTGGGAGCTAACTTGACGAAGCTGACCAACTGCAACTGCCTGAAGACGTTCGACGACAGCGACTTCTTCTCAGGAAACGCCGTGGTTGGGTTCTGCAAGGGCACCTGCTCCATGTTCGTGCAGTTCATCATCATAGTCTCGCTCGTGCAGTTCATGGGCATGTCCACGTCCGTGAGCCACACGCTCTTCATGCTTCGGAGCATAAGCCCAAGTGACAAGACCATCGCCCTTGGACTGGCCAATGCTCTGGCGAATTTGCTGTCGTACATTCCTTACCCGCTCATCTACGGCGCCGTGATCGACAGCTCTTGCCAGGTGTGGGAGAGCGCCTGCGGAACGAGCGGCAACTGCTGGCTGTACGACCTAACGCGATTGCGCCACTCGTACCTGGGCACGTCGGCCGGCTTCCTGGCCGTGTCGGGCATCTTCTCGATCGCCGTCGCCCTGGTGGCGGGTGACCTGAAGGACTTCTACGGGGACGCCTACGTGCAGGTGTCCCACTTGGGTGGCGCTGACTTCGAAGCACGGAACATCGGCGTCGAGAAGCGCAATAAGACTGTAGCCAAACAGGACAGTAACGGTCACCGCCCCAAGTGATTGCCGGGAGACCCGACGGTGTAGCACGTTGTCGGAGACGTGTTACTGTTATTCCATTTATGGACGCTAACCAAAGCTTTTTCGAGGTTCTTCGACAGTCTAGTGCTAGCAGCCGCTGGAGCCAATCAGCTTCTGTGGCGTCTTGCAGCGGAAAGCTTCCCGCGGCGATCGCATTTCAGTTGAGGAAGGACGCAACGTCTATGCGCCTTGAGTTTCTTGAAAGGCAAATCAACCCCAACCGTAGGGATTAACCCGAAAACTTTCAGTGCAGCGTCCAATGTATCATTAGACCACCCTTAATCGTTAAACTACGTTCTTCTTCTTGAAGGCTGTACTGCTGTCTGGGGTGACGGTGTTAGCGCTGGAGGTGCAATGCAGGCATCAGCTAACACTGCGTTAGAAAACCCCGTCAGAACCGTGTAATATACGGCTCCACGGTGACGCCTTCAACATAATAACAAAAAACTGCGCTTACAACTAAACGGCTTTAACAGGTAACGGCTAACGGATTAACAGGCAACAGCTAATGGCTTCGGCTTAACAACTAAAATCTGTGGTCTAGCAACTAAAAGCTGATGCACAGATGAGTATCAACGATTCAACGATGGATGCATAGCACTCCTAAGTGACC

>MG4816553

GATCACGAGCAGAAGCTTTTTGGCATGACGACGATATTCCGGCCGCAGTTTCTCTGCTGCATGCGGCAATGGTTTAGGTAGCGTTGTCCGTCCGATCCGCACACGGGGTCCAGGATGGGCAGGCATCGCTTGGGGCACTTGGGAGGCTCCAGGCACCGGTTCAGCGGCACCAGCTGAACGTGCTCCTTGCTCCCGCAGGTCTCCTTCTTGAAAAAGCACTCGTTCAGGTACACCTCGCCGTTGGACCCGCACACGGGCTCGTACAGCTCCGAGCAGTCCTGGGGGCACGAGTCCAGCTTGCGGGCCTCTCTGGCGCTGGCTATGCAGACAGCCAGTGTTTGCGTTCCGACGACCTTCCCGCAATTTCGTCGCTGCATGATGCACAGGTTGGGGTAGAAGCGGC

>MG4817124

TTCGGCTCACCGAGACCAAGGCCAACATGATTACAGGGTTTGCTGGCATTCTCGTCATGGGTGTGGGCATTTTTGCCAGTGGCACATTCATGCGCAAGTACAAGCCCAACGCCCGCTTTGTTGCCAAATGGATTGCATTCACTGCCCTGGCCTACTCCGTCGGCATGGTCATCCTCATGTGGGTGGGCTGTCCCCTCGGAGACTACGTTGGCCTCAATTCAGAACACAAAAGCCTAGACTACCTACCTCCAGCCTGCAACAGCACTTCTTGCGAGTGCAAGTCTGGGCTGTTCTCTCCAGTGTGCCATGATAGTGTGACCTACTTGTCGCCATGCCTGGCTGGCTGTTCACAAGTGTCTGGCAGTGATCAGCAGCCGAACTTCAGTGACTGCCATTGCACAGGAACAAACGAAACCATAACAAATGGGTTCTGCTCCCTTGAGTGCAACAACCTGACCTGGTACATCGTCATCTTCTCTGTTTTTGTACTCATCCACTCAACATCCGAAGTAGGGTCTATGCTACTGACCCTCAGATGTGTTGAATCCCATGACAAGGCCCTTGCATTGGGACTCATCCAGTTTGCCATTGGATTGTTTGGGAACGTGCCTTGTCCCATCATCTATGGTGCAGTCGTTGACTCTGCCTGCCTCTTCTGGGAAGACAACTGCGGAGAACCGGGCGCATGCCGCGTCTACGACCCGGCCAAGTTTCGTATGGTGTTCCATGGAGTCACAGCTGTCATCATGTTTGTGGCGTTCCTTGTGGATGCTGTTGTTTGGTACAAGGCGAGCTCCATCCACATTCACGAAGAGGAAGAGACCAG

>MG4833042

CAAGTTCCCATCCGAGTCAAGGTGGTGGAACATCCAGCCTACCTCAGGCTTGCACTCTGGCAAATGGTAATCGGACACCCTATGGGTCCTCCGTCTGGTGGATACTTGAGTGCTTAGTTGATGGTCACTCATGACCACAGAAAACCAGTCAAGCAGTCGGTCACCCATGGCCTGCAGGTCATCATTAGTGCACCCTTTATCCTTGCTGACAACAGGGGAGGAAACCTTGTTCGTCATGACTGAATTATGACCATCCTTGCTGGCCCACGAGTCCTTGGAACTGAACTTTGACTTCAGTCTGTTGTTGAAGGCAGCAGGCTTTCCCGTGGCATCTTTATTGGCTTTCTTCTCCTTGTCAATAGTGTTTTGTAGCTTGGCCATATAGCGGTCCCAGCGTGCTTTCTGACGGCTCTCACGCTGCTGTTCTTCTAGGCTTTTCACAGCTTTGCACGGGCAGAATCCCTTGCAGGAGACCTGCACTGAGGTACGCTGGCGGCAGTTGTGGTACTGCAGCCGACACTCAGAAGAATAAGTCTGGTTGTCCGTGCCGCACACATAGTCCGCCTTGAACACGGGGCACGGTGGGCACCGTTCCATTTCCTGGCTGCTGTCATCTTCCAGGTACTGGTCATC
