# Supplementary material for: A repertoire of protease inhibitor families in Amblyomma americanum and other tick species: inter-species comparative analyses
Source: Parasit Vectors. 2017 Mar 22;10:152. doi: 10.1186/s13071-017-2080-1 (PMC5361777; doi:10.1186/s13071-017-2080-1)
Supplement: Supplementary file 3 — FASTA sequences for Amblyomma americanum contigs from Illumina sequencing, by PI family. (ZIP 638 kb) [file 13071_2017_2080_MOESM3_ESM.zip › A. americanum I2.docx]

| >SG12068  ACAGCTGCTGCTCATCCAAATTTATTTTAGGATACGTTTTGCACAGATTAACAGCTGCTGCTCATCCAAATTACCTCTAATTTACTGTGTGCGGTTGTTGTGGTTACCAAGGTAATTGCTTTCCCAGGTCATGTTTAATCGTAAGTACGTGTAATTGCCACATGCTTTCATGCATTCCTCTTTGGTATCGAAGTTATTTAAGTCGTCCCCACAGCCTCCGTAAGTGAATTCCTGGCATGATACTCCATTAAAGAACCAAGCAGGAATCAGGGCTCTGCAAGGACTGTCCTCGGGCATCTTTCTGCATAATTCTTTGCATTCCTTTTGTTTGTTAGGATCAGAACATATTTTCGCACCAGGGCTTTCATCGTAGAACCAGGCCGCACACGCCGTGAAGAGAAGAGCAGC |
| --- |
| >SG12069  TTAACAGCTGCTGCTCATCCAAGTCACCTCTAATTTACTGTGTGCGGTTGTTGTGGTTATCAGGGTAATCGTTTTCCCAGGTCATGTTTAATCGTGGGTACGTGTAATTGCCACATGCTTCCATGCATTCTTCTTCGGAATCGAAGTTATTGTAGTCGTCCCCGCAGCCTCCGTATGTGAATTTCTGGCATGATATTCCATTAAAAAACCAAGCAGGAATCAGGGCTCTGCAAGGTCCTTCATCGGGCATCTTACTGCATAATTCTCTGCATTCCTCTTTTTTTTCAGGATCAGCACATATTTTCACGCCAGGGTTTTCATAAAAAAAGGCCGCACACGCCGTGAAGAGAAGAGCAGCGACTAGCAGTACGCGCATTTCGTCCAGAAGAGTATGCGGCGACGGTGCTACTCTGCACGGGGCGTACTCTTCTGGACGAAATGCGC |
| >SG120134  TAGGTGAAACACCCTGTACAGTGTTGCTTGTGATTATGGTGTCCGTCCAGGAGAAGAAAGTGCCGGGTGGTTTTGCCTGGAATTCGTGTGGAATGGCCAATATCACAAGACACAACAATAAGCTTCGGCACCATGGCCCCTGATGATGGTCCTGCGTCCGCCATGCCCATGGCGGTGGTTGCTCGTTTACATCCTAAACTGAATTCGCTTTGGTAGCACAGAATGTCGCTCCGAAATGAAACGAAATATAAATGAAGAAATACAAAATACATCTCCTGTTCGTACCTAAACCATGCGAACAGATCAAAGCGTGGGATTCCGTGTTCCCTCTTCGCCAGCAAGTGGCACTGGCTTCCTCTAGCAGGAAAAAGGTTGTTGAAGATGTAGGTGCGCCTATACGCACCGGAGTCATCCTTTATTCTCCGGAGCCAGGGGCAGAACTAGAATCCNNNNNNNNNNNNNNNNNNNNNNNNNNNNNNNNNNNNNNNNNNNNNNNNNNNNNNNNNNNNNNNNNNNNNNNNNNNNNNNNNNNNNNNNNNNNNNNNNNNNNNNNNNNNNNNNNNNNNNAGTATTTCTTGTTGGCTTTTTCCGTCAACTGTTGGCAATACTNNNTGTCTTTCTTAGTAAATTTTTGAGCACACTTTTTCATGCATGCATTGCAGCTGTCAAATCTGTTCCGAGTTCCTCGACATCCGCCGTAGATAAANNNNNNNNNNNAGCCGCTTTTTGTGTTATATGACCAGTTGAGGAAGAAAGCTTTGCANNNNNNNNNTTTCCGCCCAGGTTCTCTGCATATTGGGTTTGGTGGACGATACCCGCTTACGAA |
| >SG120159  TTGACGAAAACGTTTATTTCTTGTTACCGAGAATAAGAAAAATGCGTAGTAGCAAAATATTTTCAAATTTCCTCGCTACTCTGCAGCACTGTATGTCTTCATATCAATTGAATCCCTCTTGACGAAAACGTTTACTTCTTATTTTCCTGCTTTGCAAGTGCGCCGACATTCGCCTAAGGTTGGAAAGTTGTTTCCTGTGTAGCTGCAGCCCATATCGCTGTGACACCTGCCGGTTTTAGGACTAAAATAGTAAACTGGAAATATCCTCCCGCGGCAAAGGGCAGACGGTCTCGGAGCAGTGCAGACGGCAACACTTCGACAGGTAGAATCGCATTCTAGTTTGGTTTCAAAAGGAGCTCGGCCACATGTCCATGTGCACTGCTTGGTAGAACGGCTGAATGCGTAGCTGCGGTGACGGCAATCATTTTGCAGTGGCGGGCGGCTAGATTTGCACCGCGGATCGTGTGGGTTTGGCCGCCTGGCACTACAAAGTGCCAATAGAGCAAGCAACAGAACCGCCTTCCAGATGGCCA |
| >SG120203  TCTTTCTTAGTAAATTTTTTAGCACAAATGTTCATGCATTCATAGCAGCTGTCAAATCTGTTCCGAGTTCCTCGACATCCGCCGTAGATAAANNNNNNNNNNNAGCCGCTTTTTGTGTTATATGACCACTTGAGGAAGAAAGCTTTGCANNNNNNNNTTTTCCGCCCAGGTTCTCTGCAGATTGGGTTTGGGGGACGATACCCGCTTACGAAG |
| >SG120280  CATGGCCATCTGGAAGGCGGTTATGTTGCTTGCTCTATTGGCACTTTGTAGTGCCAGGCGGCCAAACCCACACGATTCGCGATGCAGATCTAGCCGCCCGGCGGTGCAAAATGATTGCCAGCACCGCAGCTACGCATTCAACCGTAGGACCAAGCAGTGCACATGGACATGCGGCCGAGCTCCTTTTGAAACCCAAAGAGAATGCGATTCTACCTGTCGAAGTGTTGCCGTCTGCACTGCTCCGAGACCGTATACCCTTTGCCGCGGGAAGACATTTCCAGTTTTCTATTTTAGTCCTTCGACCGGCATGTGCCACAGCGATATGGGCTGCAGCTACGCAGGAAACAACTTTCCAACCTTAGGCGAATGTCGGCGCACTTGCAAAGCAGGATAATAAGAAATAATCGTTCTTGTCCAGGGGATTAAACTGAAAAGAAGACAAACAGTGCTACAGAGCAGAGAGGAAATGTGAAAATATTTGGCTCCTACGCATTTCTCTTATCCTCGGGAACAAGGAATAAACTATTGAAAAGAAGACAAACAGTGCT |
| >SG120338  GCTGGACTCCTAATTCCTGGCTAAACAAGCGACTGAAAATGAAGTTCCTTCTGTTTACTCTTCTTCTTTCTCTAGGAGATGTGGATCCTGGTCGCGGCAGAGCAATCGCGGCAGCGGTGACGCCGAACTCTTTGGATGCAAGTTTGGGACCATTGGCGAGAAATTTATCTCCAGGCGCAGGTGTAGCCAGCAGAGAGGACGCAGTTGATTGGGCTGAAATATGCAGTGGGGATAAGAAGAACGAAACGTGCGAAGATGTATGCAGCGAGCACCCAGTATTAAGATCTTATTGTAGAGCTCTTATCCCTCGCTGGTGGTACAACGGACAATGGTGCGAAGAATTTTACTACGGCGGCTGTTACGGTGGCCGAAACAACTTCGAATCACGGGAACAATGCCAGAAGGCATGTGGCAAGTTCAAATTCCCGCAGCTGGTGTTATTAGACGATTGAGCGTAACTCAAGCAAGAAAATACATACCATGGACAATAATCTCCTGATGACTGGAAATGGAAGGTTCACTTATAACAACCAAATTGGGAAAGCATACGCAACGAATAAATTGCGGCCTGAGTTGTTTATTAATTTCTTTTTTTATACAGCAGCTTCATTCAGAGGTATAGCAGGAGTGGCAGGTGTGCACAAAAAAAATTGGTGGAGACGCTTAAGCTCCACCTTGAAGCTGTGACGCGATTGCGTAATGAGTTGATTGTCATATATGTAGAATAGACAGGAAGTTTTAGTAATGGAACACGTGATAAAGAAATGTTTT |
| >SG120395  GCCAGCGCCCTCGAGGACCGGGAGAGCGAAGCAGGCAGCGGCTGAGACGCCATGGCCTCTGCGGCTGCTTTGTCGTGGTTCCTTGTCACCTCGCTGACGGGCCTTGGAATACTGCCCGCGCCGACGACACCGGCAGAGGCCCTGGTGTGTGTCCCCAAGCCGGCCAGCGCCCTCGAGGACCTCGATTACAAGAAGTTCTCGGGAGACTGGCGAGAGGTGTTGAAGAGCAAGCACGCTCCGGTTCTCCTGCAGAAAGGCTCTTGGCGCTTTGACTTCGACACCAACACGTTCGCCTTTAGCGCAGCACTTGCAGGCAATGAGACATGCCTGCCGGCCATTGTCGGCAAAGTCCACAAAGTGCTGCCCGTCGGACAATTTCAGCTCGAGTACGACTTTTTTGGGCAGCCTGTACAAGAGACGCTGGCCGTGGTAGCCACGGACTACGGGAGCTACGCCGTGCTGCACCGGTGCGCGCATGAGCAGCCCCACGCGTCGGTGTGTGCCCCGGATGCGACACACGTCAGCGTCCTTGCGAGGCACGTGCTCGAACGAGACACCTCTCTGGAGATCACGCGCCACTTGGAGAACGTCTGCGTTGCTGTCGCACAGCTGGAACCGCACGAGTTTGACGAAGCATGCACTCTACCGTCCGATGAAGATCCCACCTTGGCCGAAATACCACCAGGCACGGGAATATGCTTCCTGGAACGAAAGGAAGGCTTCTGTGCGAATAAGCAGACAGTCTTCTACTACGACCGAGAGCAACGCAGCTGCCTGAACTTCACTTTCACCGGTTGTGGAGCCAACGAAAACCACTTCCTTACTCGCCAAGAATGTGTTGAAAGATGCGAGAAGCCACTGGAGCAAGCTCGAGCTCCACCTGTGGAATCCGAATCAGCCCAGTGTCCCCAGCGTGCAGCATGCGGCCTAGCCTGCGCTCACTGCTGCCAGCGGCGAGAGAAAGACAGCTGTATTGCGTGCAATTGCCTCGCGTCCGAAACAGAGCCCCTTCTCAAGAAAGCTGACTGCCTCGCGGCTCCCGAGCTGTGCCCGGCTGGTTGTCAGAGTAGGGAGGTGTCTGCGTCGTGCTACGCCTGCGAATGTGCTGCTGCAGACGGCACTGTTGAATATGCACTGGAGTACGTGGATGCCCAAATCGCAGTGCCCTGCCCAGTGACTTGCGTGAAGCGAATTTCGAGCAGCGGCGCCCAGAGCTGTCACTGTCCGTCTGATGGTGCGTGCAAGTTGCCGGTGAAAGAAGGCAACTGCAATGAAAGGATTCCCCGCTACTTCTTCAACGTCACTTCCGGCGTTTGCGACGTGTTCTACTACACCGGCTGTGGTGGGAACGAGAACAGCTTCGCCTCCCATGAAGAGTGCATTGCCCAGTGTGAAGATCCCTGCCGCCTGCCAATGGATCCTGGCAGCTGCAACGACACGCAGGAGCGCTACTACTTCAACTCACAGACGGGATTGTGTGAGACCTTCGAGTACGGCGGCTGCGAAGGGAACAAGAACAACTTTGCAGACCTCGACGCGTGCAAGACGCTTTGCGAAGATGTTTGCTCGCAGCCCCAAGACCCGGGCCCATGCTACGCGTACTTCCGGCGCTTCTACTACAATAAGCAGGAGGATCGCTGCCTGCCTTTCATCTTCGGTGGCTGCATGGGCAACGGAAACAACTTTTACACCAGCACCCAGTGCAACGGACGCTGCCGATTGCATGTTGCTGAGGACGGCGCAGCCGGAAAGGACAGGGTGCATGTTGCTGAGGACGGCGTAGCCGGAAAGGACAGGGAAATGTGTCACCAGCCGGTGGACGAAGGCCACTGCGACACCGACAGCAGTGGCCCAGGGGCCACTACACCAGAGATCCGCTTCTACTACGACGTGCAGAAAGAACTGTGCGAGCGGTTCAACTACCAGGGATGTGGTGGAAACGACAACAACTTCCGTACCGTAGACGGATGCAACATGACCTGCTTTGGTGTGCGCATCAACTTGGCCAGGACGGCTGCGAGCTGCCCGGAGTCCATGCAGTGCAGCTGCGGTGATGAGCAGGAGGCCAAGTGCGGGTCATGCGAGTGCGGGAACGCAGCCCCGCGTAGCAACGGGGCCGCCCTCTACGCTCTAGTGTTCCTCACTGGTGCATCGGTGACAGCGTCGCTCTGCACTCTCTGAAGAAGCGACGAGGAGGACGGAAATATTTCTTTTTTGTTCCCAGATTCTCGATTAGCGCATGCGTGATCACAGGCGCACGCGGTGTGTAGTGAAATCTGCGCGCACTGGCAGTAACTGCCAAGGACGTCCACGAATGCAATTTGTGTCCCTGGCGATATGAACTTTCTGTCGCATTCCATCGGTGCGCCCAATCTCTAAAATATGCATTGTCTTTGTGGCAGTCAACGGAATTTGCTTACAAGCAGCGTCTTTTTCAGCAACACTGGATAACTGCAGCCAGGCAATCTTGCCCTCTGCACAAAATTTTCTAACGCAACAAATGCCTCTGGCGGCGTGGAGACTATCGGCAAGTTCAACCACGGCGCATCTGCCTCGACGCGCTCCTCCACTTAGCGTGAGCCTGCCACAGATAGAGCCGAGCCGACGGGCAGCGCGGCACAGCAATTGTAAGGATGTGCGCGATTGTGTGCGGTGCCGCTCGTAGTCTTGGCACATGCGCCGCAACTAACGATAGGGACGCAGTGGCGTTAGGCATAGTGCGCGCGAAAAGAATTTGGAACAACCGAGGTTCCATGGTGGCACGGGGAAGGAGCAACACGAGGTTTTTAGGTCGCACAAGCAAATCGGTGACGTGAATGCCTGGTTGCTGTCATTACCCTAGCACGGGGAATGTCGGTCGACGACAGCCGTTCGCCAAGAGCGCATACTATCGCCTACTAGTCCAAGGTTCGACAGCCCGTTTGGCCAGGAAAAGACATTGCAGGAGGAGAAATCGACGAGTGCCAAACAGAAGGGTAAAATACAAAGGCGGGGGCTTTTCGAGACCCTTACAAGCGCAATGTTCACATGTAAAAGGAATGACTGGACAGCCGCCTTGATATGCGCGATCAGCGCCACAGTGATCATGAGTATGTTTCGGGCAAGGGCTCACGTGTCTGTGCGCGATTGGTTGTGTGGATATGAAATGACATCAGGTGCAGACATGATAAGAATGGCTTTTCGATAGCCATCATTGTAATTCTGTCCCAGACAATAGTGTGTCTGGCGCATTCGTGGTGTTCTACCAGGCCATTCAATGCCTTTTACTTTTAGTGACACAAAACGAAGAAATTATCTCTGTGGTTTCAGTAAGCACTTTCAAAAATTCTTAATCTTATGGTTAAAGAGGAAAAATCTCCGTATAAATACATTCGCACCCTCGGCGGACGTCAGTTACTAAACGATTCGCACTAGTAAGGCATATGACAAAGTACGCTTTAAGATTAGCGTTTTGGTTAAAACGGTGTGGTGACTAGTGCTAATTGAGAGCTGGAAGCGAAGGGAAAGACGATGCCTTATCAGTTTCTGAAAATGTTCTGTAAAACCGTGCAGTGCGAGTAAATGCCAGGCTCTTTTTCGCACAATACCGAAACTGAGTGCATGAGGTGTGCAGGGAGCCCAGTGCAAACACAACGACCGCTGGTGACACGGTGCAGGGCCCACAGTCTCAACACAAATACACCGCTATTGCAGTGAAAATGAAGTAATCTGTCCTAAAGTGCACTCCCTTTCACAAAAAAAGTGCGCTAGAGGAAAGCGTACACCTGTTTTATTCTTTCTGCGTTTAGAGTGTACGGCGCCCAATTCTAACTGCCCGCACATTTTGCACCACGGCATACACTTTTGTTTGCTTTGCCACTTGGTCCAAAAAAATTATAGTCGTAGCCACCCGCGTATCTGTTATTTTTTAGAGGGGCGAAGCAAAATGAAGTGCATGCCGTGGTCCAATGAAGCTTATATTTAACAGCGTTTTTCGGCGAGCCCTTCCAAAATTGCTCAAAATAGGGTGGTCTCAAAAGAGACTGCTATCCGCGGCAGTTCTGAATTATTTTGAAAATATGCTTTTTTCTTGCTAAATAATTAGCTCTCACTACTGGCAGCCCCGTAGTTTACGATGAGTATTCATTTTCTTGCACATTGCGACGAAGCTTAAGAGCACTTTCCGTGGTGTATTAGCAAAAGCAGTTCGAAGTACCGTGTCTATGGTTCCTAGGATTATCTTGCCAGCATTGGTCTGTTTTCATGTTACATTTTTGTTATGCTAACGACCAGACTCTGTTCATTCCAAAATAATTTATTAAATCTCATTAAAGTAGCGATTTGATTACTATAGTTGTAAAGTCACTCACCTCCACTGCGGCCGATTGTGTGTTCCTGAACAGTTTTCCTCTGTGTTTGAAACACCCTATAATTAGGAATGCTTGAAAGTGCTGGTGACATACCGGGCAAACGAGCACATATCAAAAACCTCCGCATTCAAGAAGTGAATTGCTATGTCACACGTGAGCTGGCATCGTGTGAGGGTAGCAGCTCCTCAAAGTAAATTTTTTAACTCTCTTACAATACCCTCCTTCCCCCTATAATGATAGGTGCGAACGGTCCAGCCAGCCTCATCATCAGTGACTACGGCACCAGTTAAAACTCGATTTAACGAAAGCCGATTTTGTGAAGTTCTCGATCTAACGAAAAAAATTTTATACCCCGGCAAGTATTCATAGGGTTCAATGTTCGAATAACCTGAAATAACGAAAGAAACGATCACTAAACCCTATTTAATGAAGTTTTTCCGGGAATAAACGACGATGTCTCGCAGCTTGTTGGAAGCGTGCCAGCAGTACATCTCCGCGCCCAACTCACGACCCTGGTTTTCTTCTGACGGGGCTGCATGCTGCGCTGCTGAACGGAACATAGGACTCGGCAGTCGGCGGTGCTTTCCACCTTGGCCTCGAATCCGGTTTGTCATATGGTGCTTTTATGCACGAGCGATTCGGGCCGCTTTCGCGGACTTTTCATACGCGAAGGCGTGGGCTGTCGGCAAATGCGACGCCTCGGTAGCTTTTCAGTGCCGCTACGTTAGAATTTAAGATCTCAAAGGCCGTAAAAATGCCGTCTTCGATTTTACGAACTTCCCGATTTAACGAAATTTTTAACGGCTGCTTCTCGCTTAGTTGAATCGAGTTTTAACTGCATTTATTTTGATTCTGGTTTACAGGTGCGAAAGAGCCCGTGACTACAGCTTGTCCGCCTACATGAGCAGTTGTTGGCGCGTGCCGTGCGTTAAACTACGACGTTGTCATTTGCTGTGTTCGAGGTTAACTCGCGGATCACGAAGGCACAATTTTCAAGCAGAACTTAGCGGAGAAAGAAAGTACCACATTAGCAAGGACATTTCTGTGCAAGAGTTGCGCATTATGGTCAGCAATGAATGGCAATTTCATAACAGGTGGCATCTTTGCCATCGTTTTGCGAGAAGCTGCCGTCCCCACTGCCGGCTTTGAAGGGGGCGTGCAGCTGCTTCATCGCCGACTGAGCGTGCCTACCATGGTCATTCTGCTCTTTGTTAGGGAAGGTCCACCAATAATTCTTGTTGGGCATTTGGTCAAAATAGCAAACCACACCAAAAAGCTTCCATGAGATGGCATCACAGGTGGTGTACCTTGTTGGTGCATAGCATAAATATTAATTCCTACAAAGTCCATACTTATCTCACTTTCCTGCCCAGTCATTCACGAAGATTATATCAACGCATTAACGGGAAGTGCTTGAGCATGCAACAAACTTTTATGGCTTTTAATGCTGTTCGTCGGAACGGGTACAAAAAGCATATGATACGGGGCGAGCTATAAAGAAGTGGAATAGATTTTTAAACAAGAACAACTGAAAAGAAACCTATATCAACCATCTAAAATCATCGGCAGTAAAATATTTGCATTGCATGCCGCGCGGTAAAGAGCTGAAAACTCAAGTGTGGGTGTGTGCGTTTTTAAGGTTCATGTTTACTTTTATTTCTTTTTTCGAGAGCGAGTTGATGGGATAGTTTGTGCTCCATGGTGCAAACGCAGCGCAAAGGACAGAGACATTCAATTCAACTCAATTCAGTTCAGTTTATTTTCCAGAAAAAAGAACATGTCGCGGACAGATTTAAAGGCTAAAAGTTGTTAGGAACAGCTTGACTGCTCTCAAGTACCATTAAATGACATTCAACGACGGAAGGCACAAAAACTGGTTGAAAGCAAATAGCACAAGGGCATTTAAAACAAAACGAAAATAATCAGGGGGGACATTGTTTCTAGGAAGTCCGAGGAATTCAAGACGGGAGCCCCCATAGTAAATCGACATATCAACCCTATTGGTGATTGGTGACGTCATGAGTATACCGATCTGGTGATTGATTGGTGACGTCACATATTTATCCAATTAGTGATTGATCGGTAACGTCACTTATAGAACCAAGATGTCAAATTCGTATTTCCATTGGTGTACCTCACTTTAACCTCATTGATATCAATTCTAGCAAACCAAACAGCTAACGCTCGTTACTTCAACGTCTTCCAGACCGTGGCGGCATCTTTTTTTGCACGCTTTTTATTATTTTGTTGTATATTTTTCTGGTGATAGACGCGCATGAATGTAGTTTTGCGTGAAAAAAAAATCATTGTGAGCTGTTTTTGTTTCGTGCTTTTGATTTCTTCCCCGTACAATCGTGGTACAGTTGAAAGAATGATTAAAGAATACATAGCCAGGGTTTATATTGTTTAACCAGGAACTCGGCTCGCAAGATATATCTGTGACATGCTCCAAATGTGCGCACCGAGTGCATGATGCGGGCAGCGAAATGCTACGCGCGCAGTGAGGGTTCGAAGAGTTCTGGAGCTGCTATGCTGAGGCGCGCTTATCCAAGAGAACGTTTCCTTGTTGACGCCGAGGCTGATTGAGATGGAATGCTGCAGACCAGGAGGGAAGGAAGGTAGTCACCTGCAGAGAAGCATTCACCAGGAACGATTGACATCAGCGTCGCTTGGTGCAATTCGTTTTATTTTATATGCTGTACCTGTGAAAGGAAAGAGTTTATTTTGACCGTGTTATTTGTGTCCATTTCCTTAAGCGCGCTACTCCTCCTTGAGGATCTGTCCTGAACTGCAAGCTGAAGCAAGTTGAAGTTCCAAGTAAACGCTGAAGGCATAATGAAATGAACACAAATAAAACGCTCCTCGAGTAGCACTGGCTTTATTTCACTAGTTCACTTCCTTATGGACGAATAGAGAAGGTGTGACTCATAATATTCTTTGTGTAA |
| >SG120538  AGTCGTTACATGCGGTGCTACATGAATATCTACCAAGCTTTAGGCTTCTTCTTTCTTCTACCAATGTGCTTGGCCATGCATAGCAGCATAAGCCCGAAAGACCCTGCCGTGTGCTATGCTCCACGCCCTGCCAGCTACTGCGATAGACCTGAAAACGTCTTTCCCGTCTTTTTTTATTATCCTGGGAGTCAATCATGTTTTCAAGATGTTGGTTGTACTCTGATGGGTAATAACTTTGCAAGCCTAGAAGAATGCGAAAGAGTCTGCTTAAGAGGAAGAGCGCAACCACCGGCACAACCGATGGCGTTGCATTTTCCTTTGGTTCACTTCTAAAGTTTGTCATCAACCACTGCAGCGACTGAGGGAGAAGTTTGGATTTGAAGAATTTCGCCTTGAAGATTAAACCCCACTGGGCAATGGGTACTGATGAAGCAATGGTTGTAATTTCGGCGCATGCCTTCTGTGGAAATCCTCTAAAATAAAAAAGTTTATATTCTAAGCATTTAAAAAAAAAAAAAAAA |
| >SG120844  GCTCTGTACAGGTGAAGTTTATCCCCATTTTTTCTATCTGCAATAGCCATATGCAATCAAAGCACCGCAGTGAGCTTATAAGAGAACGAAGAGGCAAGAACATTTCCCAAGCAAGCTTCCACTTCGCTGAAAAGACATGGAACTGTACCCGGCGTTGGCATTTTTTCTTTATTTAGGGCTCTGCGCAGCAACAAGCCGATCCACGAGAGATCCAAGATGCATCTCAAACAAACCAATCGTTAATACAAGACGATGCTCAACACCTTCATGGCAGTTCAACTTAATTGAAAAGAAGTGTAATGAGACGTGCAACAAGGACGGCCCGTTCGACAGCAAGCTTGCTTGCGATGGATACTGTCGTAGTGTTGACGTGTGCACGGCTCCACGTGCTGTGTCTTCCTGCGCCGGCGATGTACACCCTGTTTTCTACTATGATCCCTCGACACGCAGCTGCCTCAAGGACATGGGGTGCATATACTATGGAAATAACTTTCCCACCATTAAGGAATGCCAGGAAACGTGTATGAGACGTCGACCGAAGCCAACAA |
| >SG1201268  TACAAAATATTGACGTCAGCAGCCTCGGGGAATGCGTTTGTGGGGAAAGCAGACTTCCAGGCATTCTTCCTTCGAGTCAAACAAGTTTGGTCCGCCGTTGCAGCCGCCGTATAAGAACTGAAAGCAATTCTTGTGGTAGTGGTCGTAGTACCACTTGAAAGCAAATGCGTAACATGGTCCACGGTTTGGCCAGTGACGACAGTCCTCCGGGGATCTTCGTCGTTCGCAGACCTTGCGGCACTCCGGCATGCTGGGGAAGTTGTTACCGCGATTGTGACAGCCCGATCTTTTCTCGCACAATTTCGTGTCCTGGTTGTAGGAGTACACGCTGCGCTCCTTCTCGGAGCAAGAATGGGGTAGGGGGGGTTGGCTGCACACATCTCGGCTCCTGCACACTTCACTGCACAGCTGCCTTGAGGAAAAGGAGTTTGCCCGCGCAAGGTGACAGCCAACACCCAGGCGGCATTCCCTGGTGTGTCTGTCGTAGTACCACATGTGCCTTGACGGCGCGTAGTTGGCACAAGCAGGGTCCTCGGTTGGTCTGGGAAAGTGGCAGAAGGACTCTGACGGGCATGTCTTTTCGCAGGTGCTCGTCTTCACGAAGCTGTTGCCAGCCTTAGGGCAGACGAACCGGCGTTCGCACCTCTTGGCGTCCAAATTGTAGACCCACCTCTCCATAAGGCGTCCACAGCCGTGGGTTGGCGGGAAGGAGTAGCACACGGGACTCGGCATTGGTACCGGCGAGACTCGTCTTACGCAGACCCTGCGGCATTCTTGTAGGCTTGGGAAGTTATTCCCCCGGTTATGGCAGTTAACGTCTTTCAGGCACTGGCCGGAGGTTGGGTCGTAGTACCACACGGTCCTCTGTCCGCTGTCGCACAGTTCCAAAGGCCTCGGTGCATCACACACGTCGACTGCTCTGCACATCGAGACGCATTCTGATCTCGTGACGAAGGCATTGGCTCCGATCACACAATTGGCCGATTTTTTGCACCGATGAGTCTTTGCGTCGAAGAACCATTGGCTTTGTGCGGTGGTATTGGAGCAGTGTTGATCAACTTTCGGGAAGAGGCAGAAAGAATACGCCGGACAAGCGTACTCGCAGTCCTCCCTGCTGTGGAAGTTGTTTCGATCCTCGGCGCAGGTCTGGCTTCTTAGGCACGACTTGGTGAGCATGTCGAAGTGCCACCTCTGTCCGGCATTTGCCACCCGGCACGGGTGGTAGCCCACGGCGTCATAGCACTTCGACTGAGGGTTCTTTGGTCCCCAAAACGCATCCTCGTACTCGCGTTGGCCGCTCGCGAGTCCCGCGAGCAGAAGGCACAGCATCCAGGGCACGGCGGCGTTCTTCATGTTCTTCCCTCTCGTGCGGTGGTCCCGCCCCGCGCACTCAACGCCGTCTGGCCGATGGGAACGCTCTTCTCCGACCGCCAACACGCGGCACGCACGCAGCGTAGTAAAGCGCCTATCTTGCGCTTCCTCCGGGCAACCAACCTCTTGCTTTCTTCTTGCGCGCCTTCTCTCCTTTCTTCTCACCCCGACGCTTGCCGCCGCCGCCGCTGG |
| >SG1201628  TGCATCCATAATGAAGTCCTGGGTCTTCATACTGCTGGCTTGTTTCACACTACTTGAGTGGTCAACAACGATAAGTATCCCTGAAGAAGGGCATGAAGACGTATGCAGCAAGCCTCCCGAAAAAGGCGAGAGCTGCAGTAGTGGGTTCCAGGGCACAAAGTGGTACTTCAATGCAACAACAAGAACGTGCAGTACATTCATATACCTGGGATGCGGAGGAAATGAAAACAGGTTTCCTGACAACGAAACCTGCATACTAACATGCGACCCTCCTACTTACAGCTACGAGGAGTATGAAAAGCGACTTCAAGAGAGCAACAAGAAAGGAAAACAAACGAAAGGAAAACAAAGAAAAGGAACAGCAAATCCTGAAAAGTCGCAAGACTAAAAAATAATAATTCTGAATAAAGGCTGATCACCAGCCATGCATTCATTCAAGGAAAACAAACGAAAGGAAAACAAAGAAA |
| >SG1201629  GCCATGCCAGTACCCAGAAAGCAAAAACAACAAAATGGTGGAAGTTTTTCATCCTGGAAGCTAAATTTTATTCTATTTTTATGCCATGCTGTATCAGGACGTAGATTATACAGTGCTTTACCGCTTTTCCTCCTCTTGGTCATAGTACCAGGTCAGAGGGTCGCATGTTTCCTTGCACATGCCACTATCAGGAAACCTATTGGCATTTCCTCCGCATCCCAGGTATTCGAATGTACTGCACGTTTTTGTTGTTGCATTGAAGTTCCACATTTCACCGCTGCGCCCACCGACGCAAGGCGTGCCTTTGTCAGGAGCCTTGCCGCATTCGTCTTCAGCTTTTATTTCAGGGACATTCGTCGTAGTTGACCACTCAAGTAGTGTGAAAAAAGCCAGCAGTATGAAGACCCAGGACTTCATTATGGATGCAACGAAGCAAGATACACCAATACACCTCGCTCCGCCCTCCAGAAGGCCTCGCCCAATAGAACCCTTTCAACAGCACTGTCGTTCACTGGTGCGCAGGGGAACCCAAATTATTGTACGGTGTCGCTAGTGCACGTGCCAGCAGTGTGTTCAACAGCGCAGCGGTGCCTAGCGACACCGTACAACCAGCCCATTTTTTTTCAATTTTTTGAAATTTTAATTTT |
| >SG1201758  GCTTTTTCTTCATGTGTAACGTATGCACTGTGACAACAAGGGGTAGCCATCCATCCTACTCCTCAGATGAAACATCGTCGCTCGATAACCGCGGAGGGATACATGCTCGAGGAAGCAAAGGGGAATAACAGATGTACATTGATACGTTAATTATTTTTCATAGAAATATTGCACAAAAGAATCCTACTAGGTACAAAAAAACTCAACGAGGGAATGCTTGGAAATAGAAAGCCTCGAAAGAAGTGCAAAGGCGTGGCTGCGGGCGGCAGTTCATGATGGGGTCTCCAGCGTGCGCCGGACTCTTCGGGGAACGTTATCGCTGGGTGCCTGTCGCGAAGTGACTACCACTCGCACCACTTCCGGAAACCAGAAGACTCTCTCAGCTGCTGTGTCGAAGTCCAAGAATCCGTCTTGGTGGCACTACCGGAGCTGCTTCCGCTTCCGCGACGAAAGAGGTAATGACCCTTCGGCCAGCTGGCCACGCATGCCACAAATGTCATGGTTGGTCCTTAAAGTCACAAAGGAGTTACAGGTGTTTTATAACATCTGTGGCCCATTTCTTAGCATTACTTGAATGGTTTTTATAGACAATTTTTATGCATAATTACATCATTCATTACGAGACTAAAATTAACATGCCTGAGTTGTGTGTAATTTAACCAGATCAACGCTCCAGACACAGGCACAGTTTATAGATACAGCTACCAGTTACAGGGAATGATACCTTCAAATACAACTTTTTTTCCAAGGGCGACTAAAGATTGGAACAAACTACCAGCTCCAGTTGCAATATCTTTATCTGTCAGTGCTTTTTCTGCTGGTTTAGAGGCTCTCATCTTCCCTGATATGAATGCGTCTTAGTATGTGAATTATGTTTCTGATGAGCATAATACGAACTTAATTTTGCCTTTGTTCACTGTATATCACTTTTTATAAATACTTCCACTGTTTTAGACATGTTATCTTCTCTTTGTTGTATTTTTTTCCAGCTCCTGTAAAGGCCCTTACTTGGGTTGACAGTATGAATAAACAAACAACAAAGCAAATATGAATTTTTGCATGAATTTTTTCTTAATTCAAGCGTGTCTACTTTCGGACAATGGCACAAACACTTATCAAAACAGAAATTGTTTCCGATATCAATTATGAAGACAACAGATATTGACCTTAATTGCTGACTATTGATCATAAATCATAATGCAGACTCTCAGTTCCGCTGGCAAATTCGCATGTGTCTTTCACATCTAGCTAAAAATTGGAAACGCAACCATGACTTCAACTTAAAAGAAGAATATTCAAAACGGGACATGACAGCATGCAGTTGATATATTTATTGTTGACATTTGAAAAATTATCTGGCGTCGTCATTTTACCAGTTACAGCCAGTGTCCACGTTCCTCGGCTTGCGCGGCCTTCGAAATAGAGAAAATGCTGCCTTTGCGACCTTTAAGGTAATTAAACGAACAAGAAAGAATGCCGCCATTCAAGGCTGTATGTCGCAGCGACCTCTCGTTCATATGCCTCGAAATGGACGCATAGTATGATTGCTTTTATCGCCTCGGACAGGCACGTACAGCGACTTCTCGCACCACGTGACACAGAAATTCAGCTGCTGAGATTTCATATCATCGCGACGCTCGGAACGCATCCCTCAGATCAAAGCCATAAGTTTTTTTATTATTATTAGGCAATTTCCCACAGCGAGACACGATAACTCTCCTTCGAAGCCATCGTCTGCTAGCAGGGCTCACGTTTGCTCTTGGACTGCGGAGTAGCCATCGATGCCACCGTCGCGCTAGAGCCATCACCTAATTCCTCCGATAAAAATGCAGTTAGAGGAGGAAACTTTGAAGCCCTGTATAAAGAAAAACATTGTGCTGGCAAGCTGCTGCTTGATGATGCACGCAAGAATTAAATTACGCGAAAGTCTGAACATTGCCAACGGAAAAGCGGTTGTCGCTGTGCTTCGTAAACCAAGAAAAGTCCTATATTTAATGTCAGTTCCTCGATCGCTTGTGACAGAGGACGTGAACTCACGCTATCTAAGCACAGTGCTTCATCGCTTTTGTCGATGAAATTTCATCGTCTGAATTACTGATATCGTGAACCTTGATTCTCTTGATTCCAACTTATCCTAAAAATGGATTCCCAATCTTTTCACCACAGTCACCAAAATCTTTTCAACTATACATGACCAACGCTCAAAATGCCATTAGAAATACATTAGGCTTCTCAAAATTATAGCTTTTTTATAGAAAACTCATCCTACTGAAGCACGTTGTTTTTCAAGTTATTCATTAGTGTTTTTGAATTTACTCCTTAGTGTTGTGCAAATCAGTTTTCAAGTCGTTGCTTAGATGCGCTACTGGTCTGCAATACTTGACAGATATTCTTCGCAGGTGTTTCGCGGGTCTCGGTTATTAGCGCATGTCTGCATGCAGCTCTCGCAGCTCGAGAACACGTTCCCCCTAGCGTTGCATCCATTGTAGCTGAACAGAAAACAAACTTCGTAGGTTCGATTGTAGTACCACCTTTGAACATTGCTATTGAAGCGTTTACAAGACTGGCGAGGTTCTTTATTGAAGCAGTTGCGGGGATTCTTGTTAGGTTTTCTCTCCAAGCCATAGAT |
| >SG1201970  TAGTTTTTCTAGCACAATCTCCGCTCCGTCCTTCATCAGATATGCTGTTACCTGATCCGCACCAGGTGCTTTTCCCCTTTGCATTGCTCGTATGGCTTTCTTTACTTCCACTCTGCCATAAACGTCCTGATTAGTTTGGCTACAGTATATATTTGTGCAGAACTATTTGGTTACTTTAACTGTTTTATCCATAATGCTGATGACACTGCCCTCTCTGTCTCTTAGCGCATACATCTAATGTTTACCTCTACCAAATTTCCTCTTTACTGTTTTTAAGCTACCTCCGTTCTTTAGAGCATGCGTGATTTCATCCATATTATAGTTCTTTATGCCTGCTACCTTGCTCTTATTTATTAACTTCCATAGGTCTGCCATTTCTATTCTGTCGGTAGTGTTAGGAACTTTCATGCTTTGACATTTCTTAATCAGATTTTTTTTTGTCTCCTGAGATAGCTTGCCGCTATCCTGTCGAATCACCCTGCCACTTACTTCTAATTCGCACTCTGTAATGATAGCTGTGAGATTGTTATCATTATGTGAGCACTCAGACCACCTTCCTCAGTTAGAACCGAATATATGTTCTGCAGCGATATCCTGACTTCATCTACTCTTCCTCTTACCGCTAACTCGTTAATGGACTTCCGCTTTATTAGGGTCATCCATTCCCTCTTCAAGTCCAAGCTAATTCTTGAACTCACCATTCTGTGGTCGCTACAACGAACTTTTTCGAGGGCGGCCACATCTTGCACGATACCAGGGTGAGCGAATATTATAAAGTCTATTTCATTTTTAGTATCACCATTGGGGCTGTTCCACGTCCATTTCCTATTCTTTCGTTTCCGGAAGACGGTATTCATGATCCGTGAACTTTTTTCTCCTTGCGAACTCTACTAATAACTCCCCCCTGCTATTCCTGGAGCCTATGCCATAGTCGCCTACCACCTGGTCTCCGGCGTGCTTATTGCCTGCCACCGCATTGAAGTCACTCATTATAGAGTATACTTTGACTATACTTTGTTCAATCGACCTCAAGGTCAAGCAGAACACAAACATGTGCGCCGGTCTGACCTCGTGAAGTAGTGCTTTCGCACAAAAAAAGGTCAGGCCAACTAATATTTGAACACTCATTTTGAACAGTCACATATGCAGATACATACACGTACGTTATAAGACTCCGTACGCACACACGCCGGCACTCTAATAACATCTTGTATTTTTATTTGTAGCACTCAGACTGGCCTAAGTAAATGTTAGTGATCTTCGGAATCAATTATGGAAACGTTACTAACGCACATGCGATCGTTCGGCGTAATAAAATTGCTCCACTATGTCACATAATCTCAGATATCCACTTTAGCCAATAACCTTTTTGGCGTATTTTTTTTCTTCTTTCCCCAGTCACAGAATTTTAGGGAGTTTTCTCCGAAGCAGCTTTCAGATTAACACATCTGGCCATTTTGTGCAGGTGCACACTGCATCACACCTCCTGATGATCCGAATTTTTGCGGGAAACGCCTGCAAGTTTATTCCTACGATAAAAAGCAGGGTTCCTGCAGAATCCAGAACAATTGTGATCGTGGCGGCAGCAACTTCAATAGTTTGCAAGATTGTAAACGGACATGCGAATTGAAGAAAAGGAAGTAATGTGTCCTCGCTGTGGCCTTACGCACTGTGCTCTACAATGAAGGAAGAAAGGAGATACGCTTCCTAAATGCAAAATGCCTACGAGCATTACGCGAAGAGAACAACCACCGTCTGTTACCTTGCGAATGGCTCTATGGAAGAGCATCGAATGCTTCATAAATTTATCACAATATGTATGTAGTCCTTAGCATCTCAATTTCTTTTTTTGATATGGACACAAAAGGGCACAAAAGGGTTGGTGCCTGATATTGGCACCGGCTACTGCTTTTAGAAAGTGTAATCTTTAATCGCAGTAAACGGCACAGAATACAAAATACAATAAATGAAAGAGCAACGATCACAATTAATAATAACGGTAAGAGCATAATAGATGAGACACATGATATTGATACATAAAGATGATTGACATCCTCGGAAGCCCACGATTATGTAATATGAAAGAAACACTCGCCGGGACAAAAAATCACGCATTCCACCCAAGCAACATATCTAGTAACTCCCTTGGAATCCGCTACCCTTGAGAAGACTCCAGCGCCACAGTTTCCCAGAGGCTTTTTATCAAAGGCGGCCAAGTCCACCCTCAGTCGTGAGCTAAAATTGTCATGCTCTGTGCAATGCAATAAACGATTATTTCCGAGATCGGA |
| >SG1202347  GTTGCGTATGCTTTCCCAATTTGGTTTTTATAAGTGAACCTTCCATTTCGAATCATCAGGAGATTATTGTCCACGGTATGTATATTCTTGCTTGAGTTACGCTCAATCGTCCAATAACACCAGCAGCGGGAAACTGAAGTTGCCACATGCATTCTGGCATTGCTCCGGGGATTCGAAGTTGTTCCGGCCACCGCTGCAGCCGCCGTACGTAAACTTCTCGCAAAATTGCCCGTTGTACCACCAACGAGAGATAAGAGCTCTACAAGGTCCTTCGACGGGGCCCTCCCTGCATACATCTTCGCACGTTT |
| >SG1202876  CGGGGCGTTAAATCGCATAACTTTTATTTGCATATTCTTTTAGCAGACGCAAAGGGCTTCTTGTGATTCCGATGTGGTTACATTATGTGGGCTTTGTGGGAGCTCGCAAAATGCACGTCCGAAGGCACTCTTTCTCAGTTTTAAAGTTGGCGCCTGCTCGGTTGCATCCATAAGAGCGCTTTTTGCAGGTACCCGTGGTTTGGTCGTAGAAGTACGTGGTAAGTTTTTTTTCACAGACCTCGCTGTCAAGTGGAGGGCTTATGCACTGCTGTCCTTTGCAAGTCACTTGACATTCTCGCAATGAAGAAAATCTGTTGCCGTTTCCATCGCATCCTTTGTACAAAAACAATATGCAATTGCAACTTTTGTTGTCATAATAGTACATCAACTGAGGGGTTCCACATTCGCTTCCGGCTTTCATCGGCATTAAGCAGTCCGTCCCTGCTCGACAGCTCCAGTTGCACATTTCCTCTGTAGAGAACAGATAGACACCATAATGAGCAGTCCGTGTCGTTTTGATACATTCATGTTGTGACTCATTATAATAGTAGCCGGTGATGAGGTTGGACCATTGTTTCCAGTTCGAATTGCATTTATCTTCTGCTGCAGACCCCACAGTGGCGAAGAACCCTGTTAGAGCAATCACGAACCCCGAAAACGCAGTCATTATGCTTTTCCAACTGGTCACGTCTGTGAAATGCTTTAAAATCTTGCTATGAAGGTGCCTTGGAATTCGTTAAGGTTAAAATGAAA |
| >SG1203191  AAACATACAGCCGCTGACTCGCGGTTCAGCCAAGCCTATAAAAGAAGAAGGCTGACACACGGGGTTGTATTGGCTGCATACGTTTCATGACAATTTTCTCGGTGTTTTCACTGCAAGTTGAGTGATGAATTCCTACGCCGCGAGCTTGTTACTGGTGTCAGCTGTCGCAGTTTTTACAGTAAAGTGTGAAACTCTGCAGTGCGAAAGCAAAGAAGGCAAGACAAAAGAGTCGAATTATTGGATGTGCAACGATAAAAGCACAAGCTGTTTGCGACGCGATTTCTATTATGACAATAAAACCAACAGTTGCCAATTCCTTGGCTTTATGGGATGTGACGGCAACGATAATAATTTTCTCTCGCTGCCAGAATGCGTCTCTCATTGCAGATCGACATCAAAACTATCGGACTATGTTTTAAATTACTTCAAAAAGCGCTTCCCCAACTGCACTATGAAATCTGACCCAAGCTCAGACAACGGTGGCATTCGGCGATTCTACTACAATTCTACCTCGAACAAGTGCCTGCCGGTGGATGTCAAGAACGGCGATAAATACTTCCCAGATATGAATATCTGCGTAAACTTGTGCAGTGCAGATAGAACACCCCTTCCGCGCTGCAATCAGCAGATGGACACCGGAAAGGAGCCCAAAAACTGGAAATGTCATGCTGATAAAAATACTCGTTACACAACATGCAACAAAACAGTTGCAGCTAAACAATAAAAACAGTTCTATCGAAGAAAAAAA |
| >SG1203203  GCGTTCATAGACAATAAATAAGCTGTGTTCTATTTATTTCGTCATTAGAAATTTGAACACGTTTCGTAGCATTTCTCTCTTGATGGGAAATTGTTGCAGTTTCCACCATTAGTCGACCACGCGAACGATTCGCATTGTTTCTTCTTTTTGTTGTAGAACCAGCGCTCTTCGCGTACGAAGCCGGGAATACCTAAATTCTTCGGGCAGTAACATCCTTTTCTTTTAAGCCTTGCACACTTCTTGTGTTCGGGTGGAAGGCTGCATACCGGTTTAGGTTTCTTCTTCTCGCCGTCTACGAGGAGCACGGCGCACAAAAATACAATCAGCAACACCAAGAAAGTAAGGCTGCGCATAGTGGTGGTTAGATAGGCTGGAGGTGGCTGGATATCTGT |
| >SG1203491  TGTGTCTTCCTGCGCCGGCGATGTACACCCCGTTTTCTACTACGATCCCAGGACAGGCAACTGTCACAATGACACTGGGTGCATATACAATGGAAATAACTTTCCCACTATTACAGAGTGCAAGGAAACTTGTATGAGACGTCGACTGAAGCCAACAAAACCTTGGCAGTGTTTTGTGCTTCCGAGTAAAGGACACCCTTGTCCCTGGCGTTACAGCTCAAAGCGTTTTTACTACGATCCTTACACTGGCCAGTGCATACGTTTTCGGTACTACGGCTGCGGAGGGAGCGCAAATAATTTCCCCTCCCGCAATTACTGCATCAAATACTGCGCAAAGCACTAGAGGATGCTTTAAGACCTTGATTCTTCCCAGTGAAACATGGGTGACATGCCGGAGCAGCTAATTCAAGCAAGATAAAGAACTTATACCATTGGTCACGTAACATAAAACGGCATTATGATTACGGCTAAGCATCAGTTAGCTATTTATGCTGTGGATTTACTGAAAAATAAAACCTTC |
| >SG1203492  TCAATAAATCCGCTGCATAAACCTCTAACTGATGCTTATCCGTGATCGAAAGACCTTGTGATTTGGCGTAACCAATGCGATAAGTTCTTTATCTCTCCGGCATGTCTCTTGCGCAGTGCTTGATGCAGTAGTTGCGGGAGGGGAAACTATTTGCGCTCCCTCCGCAGCCCCAGTACCAAAAAGGTATGCACGTGCCGGTGCGAGGTTTGTAGTAGAAACGTATTGAGACGAAACCCCATCGGCAAGGGTACCCATGAGTCGGAGACACGAAACACTCTCGAGGTATTGTTGGCTTCGGTCGACGTC |
| >SG1204774  AACCGCGTTCCGTTAAGGCAGCGGTTAGTTTTTTTTTTCCCTTGGGTGATTAGTTTGGTCGTTTGTTGCAACTTTAGCTAACAAAAAAAAAATATCGGTTTAGTACGCATCCGAGCAAGTGGAATGCAGGAAGTGTTCTTGAGAAAGAAGTGTCGAGAAAAGTCTGGTTCAGGCTGGTGCCGTATCTCCACCACACGTTCCAGCGCACGGGGAGCACGCGTTGAATGACTCCTTGCGTGCGTTACGCTTCCGGAGGAAGACATCTTGGTTGGTGCTTAGCTTCGTTTTTCGAGACTTGCGCCGTGGCACTCCTTGTGACAGGTCTCGTGCGAGGGGAAGTTGTTCCTGTTCCCCTTGTGTCCGCAGAAGGTGAACGTCTTGCAGGCATCCTCGACCACGTTGTAGTACCAGCGCTGGGAACAGGGCCAGCGACTGCCGCGGTTCGGCTTCAGCGTGCACAGGTCCGGCTTGCGGTGAGGGCTGTGCTTGGGCCAGGGAGCCAAGGCGATGTGCACCGCGTGGGGCTGCGTGACGCCAAACTCCAACGTCGCCTCGTTGGCGCCCAGTGGCTCGACCGGCGATGCAGC |
| >SG1205578  CCTAGCGCGAGTGCTCCGTGCCTCCAAATTTCTTTGTGCTGTCTTATGAAAACGTGTTTGCCACTGTTGAGGTTTTTTATTCATTTTTTTGCAGTAGCTTGCTTGACGCAGATTGTGTACCGGGCTTCATTATCCACGGTACACTCCCAACCTTGCGGCGCCTTGCCAACGATCTTTTCTGCCCTACAGCGTGGAAGCTCTGTACGTTTTTTCGGTTTGCAGAACTTTACGCAGCTGTTAATATCGGGGAAATACGCATCGCCGTCGCCACTCTTAACGAGCACTGGCCGGCATCGGTCCGAGGTAGAATTGTAGAGGAATCGCCGAATGCCACCCTGCATTGGGCGTTCAACAGGTTTCATGGCGCAGTCCGGGAAAAGCTTCTTGAAACGCTCGACAGCATGAGGCGATAGTGTCGTAGCCATTCTACAGTGAGCGGCACAATCGAAAAGCGACGGAAAATTATTCTTGTTGCCGTTGCATCCCATAAAGCCAAGGAAATTGCACGTTTTGCTGGGTGGGTCATAGTAGTAGTCACGCCGGCGGCAGGTTGTGGCTGTTGTATTTCGTTCACAAATCCAAAGATCGGACATCCTCTTGTCATTGGGATTTGTGCTGTTTTTACATTCCGGTTTTTCAATGTTTTCTTGCTGACCGCCTAAAACCGTAATCGCTGATGCCAGGAGTAAAGAGCCAGTGTAGAAATTCATTGCCACACTTGCTTCTTATGCCACAAAAAAGTGTAACCAAACTTTCAGAATGCGATCTTAGACCTAGTGTAAGCACCCGCTTTATACGCTTGACCTAGGAGTTTTATGCCTGACGATATTATTCGTTGTAAGTTTCCTCCGGAGAACAGGGGCGGCA |
| >SG1206766  AGACGTCGACCGAAGCCAACAAAACCTTGGAAGTGTTTAGTGTTTCCGACTCAAGGGTACCCTTGTCGATGGGGTTCCGGCTCAGTACGTTTCTACTACAAACCTCGCACCGGCCAGTGCATACCTTTTTGGTACTGGGGCTGCGGAGGGACCGCAAATAATTTCTCGTCCTACCGACACTGCATGAAACATTGTGCAAAGCACTAGTGTCTGCTTGAAGTATTTGATTCTTCCCACTGAAACATGGGAGACATGCCGGAGAGTTAAAGAACTTATCTCATTGGTTACGTCACATCACAGGGTCTTTGGATGACTTCTAAGCAGCAGTTAGCTGTTTATGCAGCGGATTTATTGA |
| >SG1206769  ATCAGCGCCAAGCCTATAAAACACTGGCTCACACAAAGGCTGAGCTTGCTTTCATAAAGTTTAACGAAAGTTTTTTCTGGTGATATAGGACGCAAGTGCCGTCATGAAGCGTTATGCCATGGCTGTGTTGCTGGCATCAGCGGTTGCGGTTTTAAGAGGTGGAACAGTTGGCAATGAAGCCCCGCAATGTCAAAGCAAGAATAGCGCCAACGCCAACACAAAATATTCGTACTGGAGGCAATATGGCGAAGAGTGGTATCGGCGCGACTACTACTACGACAACGTTTCCGACACGTGCAAATTCCTCGGCTTTAAGGGATGTGACGGTAACAGAAATAACTTCCCGTCGGTTGATGACTGCATCTCTCATTGCAGGAAGGGCGTCAACTTAAACCATCCTGCGAATTCTGAACTTGCGTCAACTTAAACCATCCTGCTTCAAAACCGATTCTGGATCGCTTAAAGTACTGCAACATGACGTTTGACGCAAAGACAGACAGTGGAAACATTCGCCGTTTCTATTATAATTCTACCTCGCAACAATGCCAGCCGGTGGATGTAAAGAAAGGCGATCTATACTTTCCCGGTATGCATTTCTGCGTGAAGATATGCAATGCAACGAATTCTGAACTTCCACGCTGCAAAGACGAAAAGAACAATGGAAGTTTTCCGGTTGGATGGAAATGCAAAGTGGATAAAAAATACAGCAACACAGTGTGTTCTAAGCAGCTTCCTGCTTCTAAATGATTAAAATCAACTTTGACAATGGGAG |
| >SG1207314  CTCAGTTCCTGACAGCCTGCAGATAATGGCTGCTTATTTTTCTTTGAATAGTTTATTCCATCTGTGTGAAGATTTTCAGAAATTCAGAGAGAAAAATTTGCTTCCAGCGGGTGGTAAAAACTAGATGACGCTGGTTACTTGGCTCTTCTTGCATTGCTTGGCTTGTTAGTATTCCAATTTCCACCATTCTGCTTCTCCAGCTTCTTGCAAACGAACTTTGCTTTTCGCAGGTTCAGTCCTCCACATTTCCTCATGCAATCCTCGCAGTGTTCAAAATTATTCGCATTTCCTTGGCAGCCGCCGTAGATAAACAAAAAACAGGAATTTTTACTTTTGTTATACCACCACTTGGGCATTGATGCTTTGCAGGGCCCAACGTCTTTCGGAAGGGAGCAGACTTTTTTTCTGTTGAAACGGGCCTGTGAAAGAGCCAGGCCGAGGAGGAGCAGAAAGAGACACGCCGGTAACCTGGCCATCG |
| >SG1208491  TAGTGCAAGGGTACAAACCCCCGAAATATTGCAATGCACGACTAATCGTTGGACAATGCGGGCACGAGCGGCCTTCAACTCCAAGATGGTACTTTGATGCGAGATACGGCTACTGCGGTCGCTTCTTGTGGGGCGGATGCGGCGGGAATAAGAACAACTTTCCCAACTGTACATCCTGCATGACTACTTGTTCGACTCACCCAGATCCCGCAGGCGCCTGTCGCCACATTCTTCACTCCCCATGATTGAGGAAGAACAGCTTAGCCAGAAAATTCCCGGTCCTGCTCT |
| >SG1208580  TTTTTTGAACAGCTCCGCCAATATTTATTTGTTAGTATGCATTCGCAATTGTGTTGTTACACGAGAGCCTTCTATTTCGTATCTTCGGAAGATGATTGTACGTAGTATCAATATTGTGCCTTCTCTTCTGATGAATCGCCCAATAACATCAGCCGTGTGAAATTGAACCTGCCACATGCCTTCTGGCATTGATCCCGGGATTCAAAGTTGTTTAGGTTGCCGCCGCAGCCGCCGTAGAAAAATCCCTCGCACCATTGCCCGTTGTACCACCAACGAGGGATAAGAGCTCTACAAGGTCCTGGGACCGGGTCCTCCCTGCATATATCTGCGCACGTTTCGTTGTCCCGACCTTGACTGCATATTTCCGCCCACTTTTCTGCTTCGTCAGCGAAGGCCGCACCTCCTAGAGAAAGAAGAAGAGT |
| >SG1208842  AGAGCACAACAGCGTCGACTATGATTATAGCTCATTTCCTTATTCTTCTGGAGTTTCTGGTGTCCGCGCATGCGGCTAGTTATCGGACACCACAAAGTTGTTTGCAGTTACCTGCAGTTGGCCACTGCAAAGCAAAGTTCCCGAGATGGTATTATGATGTCTCAACAAAGCACTGCAAAGTATTTATATACGGCGGATGTGGTGGCAATTCCAACAGGTTTGAAAGTGAGGTCGAATGCCAAAAAACCTGCCTGCCTGGAGCTCCCGTGAAACGAGTTTGTAGCCTTAGGCTACCAAACAGATCTTGCAAGACGGGAGTTCATACATGGGCATATGACTCGGATGCTGGCCGCTGTCGCTTTTTCCTGCACGGCCACTGCAACCGAAACGCCAACAGGTTTTCAAACTGTCTGGATTGCATGAAAAGATGCAGCGGTGCAAAACCCAAAAATGCATGGAGAATCTGCCAAAAACTGACTACTGAAGTGATCGAAAAATATGGCAATGCTCTGCGCCCCCGCGTGGGAGGACCAGAGTAGGTTCGTCTATGCATGCCTTCCAGAAATGATAAGGCGATGTTTAGTTACGCAATAAAAGAAGCTTTTTTAATCAAATATCTGAAATGCA |
| >SG1209028  TTTTTTTTGATTTTTTAGGTAATTTATTACTTTTTCCCAATCATAAGAAAGATAACGAAGGCAAGGTAGTCAGGTGTCCTCAGATTTTCTCCTTCCTCTAAAGCTTTTTTTTGACTTTCTTAAAAAAAAAATTCCGAGGACGAATAAAAGTCCTTCAATATTTTCCTGCTTTGCACGTGCGCTGGCATTCGCCTAAGGTTGGAAAGTTGTTTCCTCTGTAGCTGCAGCCCATATCTAAGTGGCACTTGCCGGTCATATTATTCAGATAGTATACAGGAAATGCCTTGCCCTGGCATTGAGTAAATGGTCTGGGTGCAGCGCAGACGGCCGCACTTCGACAGACTGAATCGCACTCTATTTTGGTTACAAACGGGCCTCGCCCGCATGTCCAAGAACACTGTCTGGTAGAACGGTCGAATACGTAGCTGCGGTGATAACAATCATTTTGTAGTGCTGGGCGGCTTGATCTGCATCGTGAATCGTATGGTGTTGGCCTCCGGGCGCTGCATAGGGCCAATAGACCAAGCAACAGAAACGGCTTCCAGATGGCCATGATCGAAC |
| >SG1209350  TTTTGCTTTTATCGCTAATATTTTTATTTTTGATAGCAATGCCATCGCGTTTTTCCCCAGGCATTCACAGTCCAAGCGTCCAGAGTCGACTTCCAAAGCGTAGTGTAGCGTTGCTGGCTTCGCGTGCGTGTTCCTGTTCGCATCAGTCTTCCCCCCTCTCGTGGGCCTCCTTGCCCAGGCACTGCATGCGGCACTCGTCGCTGGATATGAAGTTGTTGCGGTTCCCTCCGAAGCCCAGGAAGACGAACGGGCGGCACGACTGCTTCTTCGAGTCGAAGTAGTAATGCCACACGCGCGAAGGGCCGTAGCCACGATTAGGGGGCAGCTTGCAGATTCTCGACGTAATAACGCTTCCGCATCGTCGTAGGCACTGCTTGCGGCTTTTGAAGTTGTTGTCGTTGCCTTCGCACCCGCCGTAGACGAAGAGGCGGCAAGTCCCTCGCATGAAGTTGAAGTAGTAGCGGTACACTGTGCCTTTGCAGGGTCCCCGATCGGGATATTCCGTGCACTGCATGTAGGCTTTGTCTTCCTCGAAACACACGCATCCTGTTAACGACACCAGAAATACGGTCGCTGCAAGCGCAATCCAAGTCATATTTT |
| >SG12010210  TTTGCAAGTGCGCCGACATTCGTTTAGGGTTGGAAAGTTGTTTCCTGAGTAGCTGCAGCCCATATCGAATTCGCACTTGCCGGTCCAACGATTAAAATAGTAGACTAGAAATGTCCTCCCGCTGCAAAGGGCATACGGTCTGGGAGCAGTGCAGACGGCAACACNNNNNNNNNNNNNNNNNNNACGGCAACACTTCGACAGGTAACATCGCATTGTATTTGGGTTTCAAAAGGAGCGCGGCCGCAGGTCCATGTGCACTGCTTGGTATTACGGTTGAATGCGTAGCTTCGGTGATTACAATTACCTTTCAGCACCGGGCGACTTGATCTGCATCGTGAATCGTGTGGGTTTGGCCGCC |
| >SG12012101  CGAGTGGAAAGTTTGGCCAGTACTGAGCAGTGGCTACTACTACAACAAGACAGAACATCAATGCTTGCGTATTAAAAACACAAATGATTTCCGTGGCCTTATCTTCAGTACTGTGGAGGAATGCACTTGGAAGTGCACAGCAGTTTTGGACTGCCCTCGCAAGCCTCAAGACCTTTCTGCTACTTGTGCGGAAACAGGAAAGTTAAAGTACTATTTCGATAGTGGAAAAAAAATATGCACGCTTTTTCTCGATACTGGCTGTGGAGACAGCGGAAACAAGTTCGAGACGCAGCGCGATTGTCAAGTTAGTTGCCTGG |
| >SG12012153  TTCGCATTCAAAGCACGTAAGCTTTTATTTTTGCTTCTGCTCCCTCTTCAGCACAGCAGACGACAGTTGTTTTCTCTCTTATGTTCATGGCACACTATACCGTTATTTTATGAGGTCTCCTTTCTCGCTTTGCTTGGTTTTGGAAGTAAGGTCCCATGGTGACCGCATCCTACCGCTTCTCCAGTTCACATTTCTGGCGACAATCACCGATGTTTTTAAAGTTGCGGCCGCCACGATCACAATCATTGCGCTCTACGCAGATGCCTCGTTCTTTGTTGTACGAATAAGTTTTTTTCTTTTCCTTGCAGTAGTTGGGATCATCTGGAGGTTCAATGCAGGTTGAGCCTTGACAGGTAACTTCACACTGTCGTAATGTCTTGAACTTATTTCCGTTGTCTCCACAGCCAGTATTCAGGAAAAGTGCACACGAATTTCTTGAACCATCAAAGTAGTACATTAACCGGGGTTGTTCACCGCATTTGCGAGTAAGATCTTCAGGCTTGCGAGGACAGTCAATAACTGCCTGACATGTCCAAGCGCACAATTCCATGGTTTGGAAGATGTGACCGT |
| >SG12012650  TCGGCAACGCTGTTGACACGATTCTTGCTCTGCACTGCCGCATCAAGACAAGCAAGATTTCATCCTAAGCGCCCCGCCCTGCGGCACGTGCCAGCAGCTACAGAAAAAGTGGTCATCTGCAAGCCCGTTATCTGCTGTTGGGACGTCAGCATCGAAGGACAACGGCTATAAAAGAACACAACGAGCACCAGCGCACTTCACTTGGCAGCGGCGACGACCTACTTTGCACATCATCTAAAGAACGCTTCTGCTTTTCGCTGCAGATAGACATGAAGACCTTCGTCATCGTGGCTTTTGTTGCAGCTGTCTTTCTGGATGNNNNNNNNNNNNNNNNNNGTGTCTGCTGATTTGCTGCCTCTAGAAGAGACACATGAGGCTGCGTGCAACTTGCCACCTAAAATAGGAAAGAACTGTGGACCAGACCAGTCTGGCGTGACGAACAAGAAAGGGCAGATGTGGTACTTCAATGCTACGACAAATGAATGCCACACTTTCCTATACGAAGGATGCGGCGGCAATGACAATCGGTTTCAAACCGAAGAAATGTGTTACACTATATGCAATCCACCGACCTATGACGAGACAGAGTATTTGCGTAACCTCAACCAAGGTACACGTACTGCTGAAGAATTCGAGCCAAACCAAAAATAAAAATTCACAAGCCAATTTTTGCAACGATTTCATCACTTCCTTCATTTCTCAAAGCGCAATGTTTAGATGGTGAGAGGAATAAACCTCAGGTAGACTGCAAAAAAA |
| >SG12012929  CAGAATGCTTTAGTTGAGCAGAATGCGTGCACTTGTGTGCGGTGCAGTAACTTTCTTATTTCTCTGTGCCTCTCTAGCACGTTCTGAAAAGTTTCCATGTGATTCAGACTGGAGACATTCGTCGGGGCTCAACGAAGGTTACTACTACAACAAGACCGAACATCTGTGTGTGCATAAGACGGCGGCTAATCCTCCCGAAGTCTACCTATTTCGCACGGAAGAGTTGTGCAACTGGGAATGTCGATCACATACAGACTGTTTGCAACGGCCCGTCAACCCTGAAAACGATTGCGGCACACATGAGCCTATGTACTATTATGACAGCACAACTTGCCAGTGTGTACTCTTTCTATATAAGGGATGCAAAGATAATGGTAACAAGTTTTCGACAGTTCGAGAATGTCAAGTGACCTGTCAAGGATTGAACTGCATCACCCCGCCAAAGGATGATGATGATATATGTCAAGGGACGTATAAGACGTATTATTATGACGAAAATAAAGGCGCCTGCCGAGAACAGCCCAGGAAATGTCACCGAGGTGGCGCTTCCTTTAAAACAAATAGAGATTGCGAGCGCTCATGTCTTTTGAGGAGTCCAAGTAAAAATGCAAAACCCCAATAAACTGTTTCACTCCCGCAAAGGAGAGAGTCACAGCAGGACAACATGGCAAGTTTAGTGCCAGTTAATCGCAACGGAAAAAGCGGCAATATGGCCGTTGCCCTTTTGAAATGAATGAATACGCTGAATAAAATTGTGTACTCG |
| >SG12014572  CTAAGAATGGTTTCGAGCCGCCCTTCGGACTGCCCCATATTACCGTCGGTAAAATGAGAGCTCAAGCTATATACTCAGCATGTTTGGTTTACTTCGCTTTTGTGCACGCCGCAAGTTTCCGCATATGGCCACGTTGTTGGCGAGGAAAGGCTGTTGGAAGCTGCGGGAAGAAGATTCCGTCGTGGTATTATGACTTTTGGTCCGGGAAATGTAAAGGCTTTCTCTACAGCGGTTGTGGCGGGAATCC |
| >SG12016366  GCGGCCCTCCAGCAGTGCTCCACAAAACTGAGGGGTTCTCGGCGCAGTCGGAAACGCTGTTGACACGGTTATGCTTCTCACACATAGATATGAAGAACTTCGTCATCATGGCTTCTCTTGCAGCTGGCTTTCTGGATGCAGTGTCTGCTCATTGGCTGCCCCCAGAAGAGGAACAAGAGGCTGTCTGCAACCTGCCACCTAGAAAAGGAAAGAAGTGTAGACAAGACAAGCCTGGCATTAAGAACCCGGAAGGGGAGATGTGGTACTTCAACTCTGCCACAAATGAATGCCGCCCCTTCAATTATAGAGGATGCGGCGGCAATGACAATCGGTTTCAAACCGAACAAATGTGTTATGATATATGCGATCCACCGACCTATGACATGGAAGAGTATTTGCACAACCTCAACCAAGGTGCACCTACTGCTGAGGAATTCGAGCCACCACAGGAA |
| >SG12018391  GGCGGCTCGTTCGAACAAACGGGGGACACGTATTGTGACACCTTCATAGTACAGTAGCAGCGCAGATAACTTATCCAGCAGATTTCGAAAAAAAGAAGGCTGGACGAAAAGGTAACGGGTTAGTGGCATAGCATTAAAGCCTAGTTTTCCGAACATTTCAGATATGAGCGCCACGATCAGAGTACAAGCGTGCCTCTTTCTGCTTCTCTTTTGCCTGGCTTTCACAGAGGCGGCTCGTTCGAACAAACGGATATGCAAGTTTCCAAAAGAAAAAGGACGCTGCAGAGCATCTATTCCATCTTGGTGGTTCAACATTCGCAAAGGACGCTGTGAAGAATTTCTGTACGGAGGATGCGGAGGAAATAAGAATAGATTTGACAGTTGCCCTCTGTGCTTGAACGCGTGCCGAAAAGGAGGAATATCAGGGGACAGGACAAGGCTTTGCATCCAGCTCCGTAGAAACACTGGGAGAACTCGCTGACTGTAGCACTCTCTGTGAAGAGCGGAAAACCAGGCGTAATGGGGACTGCAAGGCCTGTTCGCAAGATATGCCTTCGTTCTGTCATGATGCGAAGCAACTGTGACAGATAAATAAAGTATTTTGGGCACG |
| >SG12021443  CTGTCTTTTTATTTTGCTTGATATGGCAAAAACAAACAGTGCCGCAATGTTCAAGAGCCGGACCGGGAATTCTCTTGCTACGGTGTTCTTGCCTGATCAATGGGGGTTGATAATATGGCGGCAGGCGCCTACAGGATCTGGGTGAGTTGAACATCTATTCATGCAGGTTTTACAGTCATCAAAGTTGTTCTTATTTCCGCCACATCCGCCCCACAAGAAGCGACCGCAGAGGTTGTATCTCGCATCATAGTACCATCTTGGAGTTGAAGGCCGGTCGTGCCCGCATTGTCCAACCTTTGGTGGGGCTTTGCAATATCTTGGGGGGGTGTACCCTTGCACTAATACTA |
| >SG12021646  CGAAGGCGTCTGCCGGGCAAGCATTCCGCGGTTCTACTTCAACCCAGCTGAAGGAAAATGCTCCTTCTTTATCTACGGTGGTTGTGAAGGAAACGAAAACAACTTCGAAACTATCGAGGAATGCGAAAAAACATGTGGCGAGCCAGAGAGGTCCAGTGACTTCGAGGGAGCTGACTTCGAGACTGGTTGCGCGCCAAAACCGCAACGCGGCTTTTGCAAGGGCTTCTTAGACCACTGGTTCTTCAACGTGACCTCGGGCGAGTGCGAGGCGTTCCTCTACAGTGGCTGCGGTGGAAACGACAACAACTACGAGTCGAAGGAAGAGTGCGAGATTGCCTGCAAGCTTACGTAGCCCGCTGGACAAATGAAATGA |
| >SG12022502  CTTGACTGGAATAGCATTCGGAGAAAGGAGCCTGAAGATGGACCCTCGAACCTTCTTCTTTCTATTTGCTGTCCTGACTGTGGTGTCGGCCAAGTCGTTTTCACTCCCTCCAGAGTGCCTTGAAAAAGCCGACATCGGTCGGTGCAACAGCTTTGAACCTAAGTGGTTCTTTGATGCGGATGCAGGGCACTGCAAGCCATTTCAGTATGGCGGATGCGGGCAAAACAAAAACGTTTTTTCTAATTGCACTGCCTGCATGAGTCGATGCACTGCCCATGATGATCCCAATAGAGCATGCGAATCATATCTTGAAGTAATGTATGGCCAGCAAGGATGAAGGCAGCAAGCACCACGGCGGGGATTTTCAGCATATCTTGGTTTACACATGTGAAGATTTGGTCGGACT |
| >SG12025137  GTTGTGTTGTCTGAAAAAAAGAGAATTATGATGTGTTCTACCAAAGCGTGGGCAAAACCTTGTCCTAAACTGATGCGGAACGGTACCTATGACTCGTTCAAGTTAGGATTCGTCTCCAAGGTGCAAAAAGCGTACAAGTTCAGTTAGCTTCCGCTTCCGCTCAAAAATGGGTGTTCAGTTCCGGTTTCGGGTTTAGATGCGGGTCTCTGTCGACACCCTGGGTTCGAGGAAGTATTTAGTGAAAAAATTATGATCCTGATCACTTCATGCTCAATACTTCTCTAAGCTACATCCTGGCTTCAATTGCGCTCCTTTTTCTTTTTCANNNNNNNNNNNNNNNNNNNCAATCTGCAGAGAACTTGGGCGAAAAACGAGTCTGCAAAGCCTACTTTCTCATGTGGTAATATAACAATAAAAATGGCGTGTGCGAACCCTTCATCTACGGCGGATGTCAAGGAACTCGAAACAGATATGACAACTGCCATAAATGCATGAAAACGTCAGCTCGTAAGGCATGCAAAACTATCCGATGCGCTGCATCCGAC |
| >SG12026527  GTCCCTGGTCTGCCTGCTCCTCATCTCCCTTTCACTCGTGTGCCATGCGGCGCACAAGAAAGCTTGCAACCTGAAGAAGGACCCCGGCAACTGCGAGGACGCCTCCACCAAATGGTACTACGACAGCAAGACCAACGCGTGCAAGCTGTTCGTGTACGGCGGATGCGACGGCAACGACAACCGCTTCGACACAGAGGCCAAGTGCAAGGCGGAATGCGTGCATCCCCGCAAGACGACCAGCGGATAGGGGCGGCAGCAGCAGAGCCAGCCTCACTGACGGCAGCGGCGATCCGGTTCGACACATTAAAG |
| >SG12027605  CAGCGTGGCTGCTTCTGCTTCTCGTCGGCGTAGCTTTTTCACATGCCTGGTTTAACAGAAAAAAAGTCTGCACCCAGCGAAAAGAAGTTGGACCCTGCAGAGCATCGATACCTATGTGGTGGTATAACGCAAAAAGACAATACTGCAATTTGTTTATCTACGGTGGCTGTCAAGGAAATCAGAATAAATTTCAACACTGCGACGAGTGTATGAAGAAATGCGGTGGAATGGGCTGGTGGGAAGCGAAGAAGTTTTGCAGGAAGCTGGAGAAAGTTGGACCCTGCAGAGCATCGATACCTATGTGGTGGTATAACGCAAAAAGACAATACTGCAATTTGTT |
| >SG12027928  GGGACAGCACGTCTCACTTAGGTGTTTCAGTCAGGAACATAAGGTACGTAGCGTAATGCGATGCTAGCGCTGTCATTGGTACTTCCGATCCAGAAGTGATGTCAGCCGCAGACGCGGAGGCATTCCTCATATGAAACAAAGCGATTCTGGTTTCCTTGGCATCCGCCGTAAACGAACTCTCGGCACGTCCCGGATGAAATGTCGTAATAGAACGAGCGGAAGTAGGCGAAGCAGACTCCGGGACTAGGCGGCAGGCTGCAAAATCTGGGCCTTCTCTGAGCATTGGCACCATTGAACGAGATGGCCAAAAGCACAGCCGCCAGCACAGTCAGTCGAAGCATCGTCGACACCTGAGATGGTCGCGGAC |
| >SG12028319  GCTGGACACAATTAGTGATCTCGCGCCAAGAAACCAAGATGCATCTCCTAACTTCCATCCTCACCGTATGCGCTATCGTGGCTTCGGTTCTCTCCGAGCAAAACAAAACACGATGCGTGATGCCAAATATTGTAAGCGGGCCAAACTGTCGCCAAATCACCTTGAGGTACTTTTACAATACAACGTCCGAAATGTGCGAACACTTCCGGTGGAATGGCTGTGGCACAAAAGGCTTGTTTGGATCTCGCTACGATTGTGTCTCCACATGCAATGAAAACCAAGGCGCTCCATTCTGCGCTGATTCTCCACCAAGTCCTTGCGAGGAGGAGAAAGCTAAAAAAGGGAGAAAAAGGTATTACTACAACATAACGACACAAACCTGTGAGGAGTACATATTCTGCGGAGGACGGCAGAGCTTGCTCGACAATAATTATTTCATCGCAAGAGGATACTGCGAAAAGCAATGCGGAGGGTTCAACGAAGAAACAGCAAAGGCCAAGGATGAGTCCGTGGTTCTCGAATGACCTGGAGTGGACGTTATCCACTTTTCGCGGCTCGAAGATGAACCACATGAAATAAAAAAGCTTTCGC |
| >SG12030101  CGATGGCCAGGTTACCGGCGTGCCTCTTTCTGCTCCTCCTCGGCCTGGCTCTTTCACAGGCCCGTTTCAACAGAAAAAAAGTCTGCTCCCTTCCAAAAGTCGTTGGGCCCTGCAAAGCATCCATGCCCAAATGGTGGTATAACATCAACACAGGTTCCTGTGTTTTCTTTATCTACGGCGGCTGCCAGGGAAATGATAACAGTTTTGATCACTGCGAGGACTGCATGACGAAATGTGGCGGACTGAGATGGCGAAAAGCAAAGATCGCTTGCAAAAGGCTGCAGAAGCAAAACAGTGGAAATAGGTATTTGCACAAGCC |
| >SG12030779  GGGAACTGCATAATTTACTAACAGCATGTATCTTTATACAGTAATCACATTAGACGAATTTTGCAATAATTACACCTAAAAAGTCCCAAGGCCTATATTAGTACTCTCACGAAAATAACCAAATGAAAATAGTATGCATAAGTGCAACCACACATGTGATGGCTTTCCAGAAACACGTTAGACCGTGGAAGTAAANAGATAAAGAAAACCTTCACTTCATGACGCAACACTACAACACTGCAGTTAAAGAGGTTTTTAAGAGACTGGAGTCATCTTTATTTGTTGGAGACGCTAGTAAGCTAGTTTTCTTATTTTGTACTATTGTATGCACACATTTTTTGAGGCGGAACAATGTGATACAGCTGTCATTCTTGCTGCTGTTTTCCCAGCAAGATTGGGTATATGCACGTCAAGCGCTTTCTTGTCCAATCGGAAGATCCCAAGCTATTTTGGCTCGTCATCCCTTGTGCCTCCGGCGTCTTCGCAGACACGCCTGCACTCGCGAAGTGTCCGAAATCGGTTCGCATTTCCCCCGCACCCTCCGTAGACGAACCTTCTGCACCGACCGAACAGGCCGTCGTAGAAGTAGAGCACGTAGTGGCCGAAGCAGTTCCCGGAACTTGCGGGCAGCGAGCACAGCCTTCGCCCGCTTTGCGCACCGGCAACTGCACGAGGAGGGATCGGAGCTCGTGTTATATGCTTTTGAAAAGAAGGTGCCAGATTTTCNNNNNNNNNCCAAAATGAACTAGCGGCAGCGACAGCAGTATCATTCCTCTTTCAGCGGGAGCGCGCTCCAAGGGCCACAATGTTCAAGTCCAGTTCGATCATAATAGCGAACAGGCCACATTAGTGAGAGAAATTAAACTTCGTTAAAAAGATTAGCATTCAAATGTGGTCTTCTTATACCAGGATATATCGCTGGATGCTGCGTTACGTGCACGGTGGACGCGCGTGCTTTTAGGTTACGACAAAAGTATAGGGCCAAATACTTTTAATTTGGTTTAAG |
| >SG12030970  CCGGCTTGAAAGATTATCTTTATTAACGGAAGCAAAAGCAGCAAAGCAGCAGCGCAATCTTATAATTAAGATGTTGTTGTTGTTAGCGTATCAAAGGATGGGCTAACAACAACAATTAAGAAGTACGGAATCACTACGTTACATTAGATTCTTTTTCTTCCCTGTTCCCTTTAGCGCTACTCGCAAAAATACATATAGTGTAAAGAATGGCACGACGAATTATTCTTCGATCTCTTCTTAGCGCGGCCGAAGACCTCCGCGCCGTCGACCTCCGCAAGCCCTCATGCAATCACGTTGTGCTTTAAAGTTGTTGCCGTTGCTGCGGCAGCCGCCGTAGGTGAACTGGCGGCAAGTATTGCTGCTGGCGTCGTAGTAGAATCGGAGGAAGCTCGCCTTGCAAGGCCCAGTGTATGGCGGCTCCATGCATACGCCTACGCCGGATCCGGGAAGCCGCCCCTTCTTGTCATCGAAGTTGGCCTTCTTGTTGCTGAATGTGGTGAGAGGCGTTTCGTTCAGTGTTTTCGGAGCGCATGTCTTTTCGCAATCCTCCTTGTAGAGGTATCTGTTCTGGTT |
| >SG12031253  CCACTGCCCACTGAGCACCCTGTCGACCATCCCACGCCACTTGATGTCTGTGCACAGCCTAAGCAGCCGGGACCTTGCTACGGTGTGCTGATGATGTGGTACTATGACACCACGACCCGCGAGTGCCGCAACTTCACCTACGGTGGCTGTGAGGGCAACGACAATCGCTTCGAGTCTCGGGAGCTCTGCGAACAGCGCTGCGGCCGTGCTGTACCACCAGTCGTAGTGCCACCTCCTCGGCGTCCCACTAAGCCTAGGCGCATTAGCAAAGGCATTTGCAAGAAAATGGCTGATGCGGGAACCTGCAGTGAAATGCATGCTAAAT |
| >SG12031969  CTTCACGGAGATTCCTCTTCCCTTGCCAATAAAAAAAGCAAACAGATTTCTCTTCCTTTTGGTCTAGAAAAGCGCATTATTCTTGATGACGTCGCTTTAATATTATTACATCCTTCTATAATTGGGCATGCATTCCTCTTGGCACTTTTCCGCAGTCTCGAATCTGTTGTCCTCAGGCCAGTATGCTATCCGCTTGAAGGGCGAGACCGCCCATGCGGGCTTGCACAAGTTAGTATCAGCGTCATAAATGTACGTCTTCGTACCGATAAGCCGGACACCCGTACCTTTCTTCAACAGGCAAAGCGAACGATGAAAGCATGTTTCCAGGCATTCTTTTCTTGATGCGAAGTTGTTTTCGTTTCCCCCTTGGCAACTGTATGAGATGAACGTCTCGCACTTCTTTGTTAGGTGATTGAACCCGTAACGCGTTCCCCAGCCCGATGGACAGGCGCCTCCTGTAGGAATAATGGGCAGTTCGCATCGACGATCGTTGAGGGC |
| >SG12032250  GGACACTAAAGCGGAAAAGATTTAAAGCATGAGAGTTTGCGTGTTGATTATATTTACTCTCCTGTCACTGTTTGACGCAGGGACAGCGGTGAAGAAAACTCGTCGCAGGGAACCGAAGGCTGCCGATTGCAATTACGAGCTAAAATTAGGAGAAACTTGTGATGAAAGGAAGCCTCGCGGGAAGTGGTTCTTTAACAAAACTGGAAAAACATGCGAACGTTTTACATATAAAGGATGCAAAGGGAATCCTAACAGGTTTCGCACCCAGGACGAATGCAAGCAAACTTGCACCGCAGACGCGACTAATACTGCGCCATTAGAGATTCCTGTCCGTCCTGAATGCAAGAAAAGCGAG |
| >SG12037225  TAACGAAGCTCTCAGAGTCTTTGTTGCGTTTGGCTTCTGGGCAGCCATTATTCCCGGCCACGCACTTCTCAGCGACGATATCTGCAATCGTCCACGCGCAGTGCCCACCTGTCTGGACACCGCATTTGAAGTTTACTTTTATTCACCAGACACCGGAATGTGCCACAGCGATGTGAGCTGTACTCTCGAAGGCAACAACTTTCGCACGCTTCAAGAGTGCATGAGTGCTTGTGGAAGTAGTTCGCCGATGTCGACTGCACACACAGGGATGACGATAGCTGAGCCCTTGTACGCCATCTTTTATTGAAAATGAATTCCGGTTTGTGGTGTGCTTTTGACCTCGTTGGGCGTCGATGGAGCTCTTTCAGCGATATGAGGAAACTTCAC |
| >SG12038947  CGCGCTCAGCCTGTTTTTTGGTCAAAACACGCGCGTCGCCTGCGGCAGTGCATTTCGAGCTGGCGCTTCTTTGAGCGTACGAGCCCTAATATCGGCCGCAGACTCTGCGGCATTGTCCAACAGAGTTGAATCTGTTCGCGTTGCCACCGCATCCGCCGTAGGTGAACGAGAGGCATCTTCCCGATCTACTATCGAAGTAGAAGGCTGGGAAGGCGCCGCGGCAGGGTCCCTGGCTAGGAGGAAGGAAGCACTGGCCTTGAGCATTGGTGCCTGCTACGACAATAGCCAGCAGCAAGGCGACGACCACAAGCGAACGAGGCATCTTGCGAACGGGCGGTCCCGGTGGGTGCGTTT |
| >SG12039305  ATGAGCTCGATGATCAGACTTCAAGTTTGCGTTTTTTTACTTCTCATCTGCCTGGCTGTCTCACAGGCGGCTCGTCAGAGAAGACGAATCTGCAGAGAACNNNNNNNAGTGAAAGGACCATGCGATGCCTCCAAGATTAGGTGGTCATTTAATCCGAAAACCGGAAGATGCAGCCCATTTATCTACGGCGGATGTGAAGGAACTCGGAACAGGTTCCCGACCTGTTGCGAATGCTTTAGAAGATGCACTGGATCCTACACCAGGAAGAACAGCAGGACTTGCCGGAGGTTGCTTGAAACCCA |
| >SG12040348  TTACGATGCTAGTGGTGTAGTTAGCTTTGCATGTCGCCTCGCAGTCATCCTTGGTGTTGAACCTGTTCTTCTTGCTTGAGAACGGAGAGTATCGCGAAGTCTTGCAGCTGTCGCTCTCGACGTCGTAGTAGAATTTTTTCACAAGTCCAAACCTATTCTTGGGACCAGGCTCTACGCACTTGCTGCCAGAGTCCCGGCCACAAGTGTTCCAGCACTCCCTTGCGGAGTCGAACCTGTTGGGATAGCCACCGCAGCCGTAGTAGAC |
| >SG12040979  ACCTCTGTGAACAGACCTGCCTCCATCAGGAGACACTGGACCCATGTGAACAGTCGGTTGCACCTGGATCGTGTAGGGGCTCATATACGCGGTTTTACTATGACCGGCAGGAAAATCGGTGCAAGCAGTTCACTTATGGTGGCTGTCAGGGTAATGCCAACAACTTTGCCACTGAAGAAGAATGCTCCGAACGATGTGTCTCTCTCGGTGCAAAGGATATCTGCATCCTGTCCAAGGAGGTAGGCCAGTGCCTGTCGGAAGACCGCAAGTGGTACTACGACTATGTGGAGGGGCG |
| >SG12041725  AAGTGCAGCCTAGAGCCTGACATAGGGAGCTCTTGTGAAGGCAGGAGCCCTGACATTAAGTGGTACTTCAACTCAATCACAAAAATCTGCAATCCGTTCTACTACTGTGGGTGTGGAGGCAACATAAACAACTTTCCGGAAGAGGAGGTGTGCTTACAGACGTGTGATCCCGTTACGTATTTCGAAGATGACTACTAAAACAGCATTGAAAACCCTGCACAGAAATGACACGCAACTGTCAAGAGCTCGTTTCGAAATGAAAGCTTTGTAGTTATAATCTGTGTGTGTTTTCTGGACGTCTTCAGAAGCAGAG |
| >SG12042517  GTAGTAGACGAACCTTTCACACCTTTTGTTCTTGTTGTTGTATCCAAATGCTACCTTACGTGAACCATTTGGGCACTCCCTCCCGCGATGTATTGGGTAACTGCAATTGCTTTCGACGTATTTGCCGCATGTTTTCCAGCATTCGGATTCGTAGAGAAAATTGTTTCCATTTCCTCCGCAGCCTTCGTAGTCAAACTTTTCGCAGGCTTTTGTTTTACTGTTGAACCAGAAGTTCTGCATGCGGCTCGACCTGCTGCAGTTGCGACCCGCGTGCTTTGGAAGGAGGCATGAATCTTGGGCGAATTTTCCACATTGCGCCTGGCACTCCTTTTCAGTCTGGAAATGGTTCTCATTGTGCTCGCAGTAGATGTAACGCACGCATCTTTGCTTGAACGGATTGAACCGGTACCCCTTATATCCAGCTCGGCAAATTTGTTTGG |

| >SG9671  CCATGCCCATGGCGGTGGTTGCTCGTTTACATCCTAAACTGAATTCGCTTTGGTAGCACAGAATGTCGCTCCGAAATGAAACGAAATATAAATGAAGAAATACAAAATACATCTCCTGTTCGTACCTAAACCATGCGAACAGATCAAAGCGTGGGATTCCGTGTTCCCTCTTCGCCAGCAAGTGGCACTGGCTTCCTCTAGCAGGAAAAAGGTTGTTGAAGATGTAGGTGCGCCTATACGCACCGGAGTCATCCTTTATTCTCCGGAGCCAGGGGCAGAACTAGAATCCAAAATATGCGTGCAGCACTTTCTGGCTTTCGCAAGCATTAGACAGTTTAATGAACTCGCTGAAGTACTTTTCGAGAAAGGAGATTGTCGCGTATTTTGACAGCCTTAATTAAGCTTGGCGGTTCCGGTTATTTCGGCATGGCCGTCGGGAAGTATTTCTTGTTGGCTTTTTCCGTCAACTGTTGGCAATACTNNCTGTCTTTCTTAGTAAATTTTTGAGCACACTTTTTCATGCATGCATTGCAGCTGTCAAATCTGTTCCGAGTTCCTCGACATCCGCCGTAGATAAATGCTTCGCACAAGCCGCTTTTTGTGTTATATGACCAGTTGAGGAAGAAAGCTTTGCANNNNNNNNTTTTCCGCCCAGGTTCTCTGCATATTGGGTTTGGTGGACGATACCCGCTTACGAAG |
| --- |
| >SG9684  TAGTTAGCGCTTCTTAGCTGTATTCTGATGTACCACACCACTGACAGTGCTGCGCCCAAAAAAGCGTTCTTCTAATCACAGAAGCTCATTTTTAAAAATTCTGCAAATTTATAGGTGAAACACCCTGTACAGTGTTGCTTGTGATTATGGTGTCCGTCCAGGAGAAGAAAGTGCCGGGTGGTTTTGCCTGGAATTCGTGTGGAATGGCCAATATCACAAGACACAACAATAAGCTTCGGCACCATGGCCCCTGATGATGGTCCTGCGTCCGCCATGCCCATGGCGGTGGTTGCACGTTTACATCCTAAACTGAATTCGCTTTCGTAGCACAGAATGTCGCTCCGAAATGAAACGAAATATAAATGAAGAAATACAAAATACGTCTTCTGTTCGTACCTAAACCATGCGAACAGATCAAAGCGTGGGATTCCGGGTTCCCTCTTCGCCAACAAGTGGCACTGGCTTCCTCTAGCAGGAAAAAGGTTGGTGAAGATGTAGGTGCGCCTATACGCACCGGAGTCATCCTTTATTTTCCGGTGCCAGGGGCAGAACTAGAATCCNNNNNNNNNNNNNNNNNNNNNNNNNNNNNNNNNNNNNNNNNNNNNNNNNNNNNNNNNNNNNNNNNNNNNNNNNNNNNNNNNNNNNNNNNNNNNNNNNNNNNNNNNNNNNNNNNNNNNNATTTCTTGTTGGCTTTTTCAGTCAACTGTTGGCAATACTNNNNNTCTTTCTTAGTAAATTTTTTAGCACAAATGTTCATGCATTCATAGCAGCTGTCAAATCTGTTCCGAGTTCCTCGACATCCGCCGTAGATAAATGCTTCGCACAAGCNNCTTTTTGTGTTATATGACCACTTGAGGAAGAAAGCTTTGCANNNNNNNNTTTTCCGCCCAGGTTCTCTGCAGATTGGGTTTGGGGGACGATACCCGCTTACGAAGGTCGCAGCGAGCAGCAGCACGCTGAGTATGGCTATGAGCTTCATAGTCGATTTCGCTCCTTTTTTCGTAGAAATCGGGAACGCAGCTGAAAACGGTCAATCTGTGCGGCTTTATATATGGATGGCGTTGTTCCGACAGATATTATCTGGTTGTTTTTTAGTGTGCAGTTTAGACATCTCCTTTTTAATGAAAGAAAATGC |
| >SG96255  TCCTAATTCCTGGCTAAACGAGCGACTGAAAATGAAGTTCCTTCTGTTTACTCTTCTTCTTTCTCTAGGAGATGTGGATCCTGGTCGCGGCAGAGCAATCGCGGCAGCGGTGACGCCGAACTCTTTGGATGCAAGTTTGGGACCATTGGCGAGGAATTTATCTCCAGGCGCAGGTGTAGCCAGCAGAGAGGACGCAGTTGATTGGGCTGAAATATGCAGTGGGGATAAGAAGAACGAAACGTGCGAAGATGTATGCAGGGAGGGCCCCGTCGAAGGACCTTGTAGAGCTCTTATCTCTCGTTGGTGGTACAACGGGCAATTTTGCGAGAAGTTTACGTACGGCGGCTGCAGCGGTGGCCGGAACAACTTCGAATCCCCGGAGCAATGCCAGAATGCATGTGGCAACTTCAGTTTCCCGCTGCTGGTGTTATTGGACGATTGAGCGTAACTCAAGCAAGAATATACATACCGTGGACAATAATCTCCTGATGATTCGAAATGGAAGGTTCACTTATAAAAACCAAATTGGGAAAGCATACGCAACAAATAAATTGTGGCCCGAGTTGTTTATTTAAAAAAAA |
| >SG96256  TTCTTGCTTGAGTTACGCTCAATCGTCTAATAACACCAGCTGCGGGAATTTGAACTTGCCACATGCCTTCTGGCATTGTTCCCGTGATTCGAAGTTGTTTCGGCCACCGTAACAGCCGCCGTAGTAAAATTCTTCGCACCATTGTCCGTTGTACCACCAGCGAGGGATAAGAGCTCTACAATAAGATCTTAATACTGGGTGCTCGCTGCATACATCTTCGCACGTTTCGTTC |
| >SG96260  TTTTTTTTTTTGCTTTTTCAATACTTTATTCCTTGTTCCCGAGGATAAGAGAAATGCGTAGGAGCCAAATATTTTCACATTTCCTCTCTGCTCTGTAGCACTGTTTGTCTTCTTTTCAGTTGAATCCCCTGGACAAGAACGATTATTTCTTATTATCCTGCTTTGCAAGTGCGCCGACATTCGCCTAAGGTTGGAAAGTTGTTTCCTGCGTAGCTGCAGCCCATATCGCTGTGGCACATGCCGGTCGAAGGACTAAAATAGAAAACTGGAAATGTCTTCCCGCGGCAAAGGGTATACGGTCTCGGAGCAGTGCANNCGGCAACACTTCGACAGGTAGCATCGCATTCTAATTGGGTTTCAAAAGGAGCTCGGCCGCATGTCCATGTGCACTGCTTGGTATTACGGTTGAATGCGTAGCTGCGGTGAGGGCAATTACTTTTCAGCACCGGGAGGCTTGATCTGCATCGTGAATCGTGTGGGTTTGGCCGCCTGGCACTACAAAGTGCCAATAGAGCAAGCAACAGAACCGCCTTCCAGATGGCCATGGTCCCAATTCACGATGCAGATCAAGCCGCCCAGCACTACAAAATGATTGTTATCACCGCAGCTACGTATTCGACCGTTCTACCAGACAGTGTTCTTGGACATGCGGGCGAGGCCCGTTTGTAACCAAAATAGAGTGCGATTCAGTCTGTCGAAGTGCGGCCGTCTGCGCTGCACCCAGACCATTTACTCAATGCCAGGGCAAGGCATTTCCTGTATACTATCTGAATAATATGACCGGCAAGTGCCACTTAGATATGGGCTGCAGCTACAGAGGAAACAACTTTCCAACCTTNNNNNNNNNNNNNNNNNNNNNNNNNNNNNNNNNNNNNNNNNNNNNNNNNNNACTTTCCAACCTTAGGCGAATGCCAGCGCACGTGCAAAGCAGGAAAATATTGAAGGACTTTTATTCGTCCTCGGAATTTTTTTTTTAAGAAAGTCAAAAAAAAGCTTTAGAGGAAGGAGAAAATCTGAGGACACCTGACTACCTTGCCTTCGTTATCTTTCTTATGATTGGGAAAAAGTAATAAATTAC |
| >SG96343  CGGCCACGCACTTCTCAGCGACGATATCTGCAATCGTCCACGCGCAGTGCCCACCTGTCTGGACACCGCATTTGAAGTTTACTTTTATTCACCAGACACCGGAATGTGCCACAGCGATGTGAGCTGTACTCTCGAAGGCAACAACTTTCGCACGCTTCAAGAGTGCATGAGTGCTTGTGGAAGTAGTTCGCCGATGTCGACTGCACACACAGGGATGACGATAGCTGAGCCCTTGTACGCCATCTTTTATTGAAAATGAATTCCGGTTTGTGGTGTGCTTTTGACCTCGTTGGGCGTCGATGGAGCTCTTTCAGCGATATGAGGAAACTTCACCTAAACGACCTCATAATAAAATGAGTTCTTTTGCAGTCCTCAAGGTTGTGCAGTTTGTGAAACTTTTTACCTTGCTGGTCTGTCTCCTGGTCGGGCCAATCTCCTTTCCGTCTCGTAAATAAATGATCTCAAAACATAAAAAA |
| >SG96391  TGGACGGGCCACGACTGCGAGTGTCTAACATGAATATCTACCAAGTTTTAGGCTTCTTCTTTCTTCTGCCAGTGTGCATGGCCGTGCATCACAGCAGCAGCTTGAAAGACCCAGCCGTGTGCACTGCCCCACGCCCTGCCAGCTATTGCTATGGTGGAGCCTTTGAGGTCTTTTATTTTGAACCCTTGAGTAACTCATGTTTAAAAGAATTAGGTTGTACTCTGCACGGCAATAACTTTAAAAGCAGAGAAGAATGTGAAAGACTCTGCTTAAGAGGAACCGCGCAACCACAGCCACGCCCGATGGCCTTATTTTATCCTTGGGTTTGGCTCTAAGGTTTATCATCACCCAGTGCAGCGACAAAGGGAGCACTCTTGATTTGAAGAATTTCACCTTGAAGAATAAAACCCACCGGGCAATGGGCATTGATGAATCAGTGGTTATACTTTTGGTGCACGTCTTCTGTGGAAATCCTCAAAAATAAAAAAAGGCTAACATTATAAAAATTCCACTGACGCAGATATCTTTTAAATCGAGTCAAAATGCACCAATCTGGGTCGAGAACAGCGGGCTCTGCCATGGTTGCAGTTTAAATGTAAAGGAGAGAAACTTACCACTTCATTTTAGCCATATATAGCTGCTCAAAATTTGCCTGACAAAAAATTTTAGTAACATTATTTAGAAAGTCTTTAGACAAGTGTTAATAGAATACATTGACGTTGAATTAAAATCTTT |
| >SG96397  ACGTGCTGTGTCTTCCTGCGCCGGCGATGTACACCCTGTTTTCTACTATGATCCCTCGACAAGCAGCTGCCTCAAGGACATGGGGTGCATATACTATGGAAATAACTTTCCCACGATTAAGGAATGCCAGAAAACGTGTATGAGACGTAGACCGAAGCCAACAAAACCTTGGAAGTGTTTAGTGTTTCCGACTCAAGGGTACCCTTGTCGATGGGGTTCCGGCTCAGTACGTTTCTACTACAAACCTCGCACCGGCCAGTGCATACCTTTTTGGTACTGGGGCTGCGGAGGGACCGCAAATAATTTCTCGTCCTACCGACACTGCATGAAACATTGTGCAAAGCACTAGTGTCTGCTTGAAGTATTTGATTCTTCCCACTGAAACATG |
| >SG96398  TTTTTTTTGCTTTGTGTGCAGCAATATTTTATTTTTCAATAAATCCGCTGCATAAACAGCTAACTGCTGCTTAGAAGTCATCCAAAGACCCTGTGATGTGACGTAACCAATGAGATAAGTTCTTTAACTCTCCGGCATGTCTCCCATGTTTCGGTGGGAAGAATAAAATACTTCAAGCAGACACTAGNNNNNNNNNNNNNNNNNNNNNNNNNNNNNNNNNNNNNNNNNNNNNNNNNCGGTGCGAGGTTTGTAGTAGAATCGTACTGAGCCGGAACCCCATCGACAAGGGTACCCTTGAGTCGGAAACACTAAACACTTCCAAGGTTTTGTTGGCTTCGGTCGACGTCTCATACACGTTTCCTGGCATTCCTCAATGGTGGGAAAGTTATTTCCATAGTATATGCACCCCATGTCCCTGAGGCAGCTGCGTGTCGTGGGATCATAGTAGAAAACAGGGTGTATATCGCTGGCGCAGGAGGACACAGCACGTGGAGCCGTGC |
| >SG96486  CTCAGACAGAATAGTGCGCGTCGTTCCAGGTTTTTATTAAAGTGCTGAGAACATTTTGTTTTAATATGCTTTGTTCACATACTTAGTTCTTTAAGAACGGAATATTTAATATGAAAGGCCACTTGGATGTTTTCCGGTTTGGTTGAAGCCCTAAGTTGTATCTTTGGCGAAATGCTTCGGTCCTCTTCTTGCAGATTTTCCAAGCGTGTTTCTTCCCGCTGCACCGCTTCATGCACTTCATGCAGCTGTCAAACCTGTTGGCGTTCCCGTAGCATCCCCCATAAATGAAACCACGACACAGATCCACATCGGCGTCGTAGTACCATGACGGAGAAAAGCCCGTGCAGTTTCCAGTTTTCGGCTTCAGGCTGCAAACTAGTTTTCGCTTAGATTTACGTGTGCAGGCTTTCTGGCATTCTTGTTCAGATGAAAACCTGTTCGAGTTCTCACCACATCCGCGGTAGAGGAACCCTTTGCAACTCAAGGTCCACAAATCGTAGTACCAAGAAGGAATCTTAGCTGTACAATTTC |
| >SG96487  GAAATTGTACAGCTAAGATTCCGTCTTGGTACTACGATTTGTGGACCTTAAGTTGCAAAGGGTTCCTCTACAGTGGATGTGGTGAGAACTCAAACAGGTTTACGTCCGAACAAGGATGCCAGAAAGCCTGCACACGTAAAGCTAAGCGAAAACCAGTTTGCAGCCTGAAGCCGAAAACTGGAAATTGCACGGGCTTTTCTCCGTCATGGTACTACGACCCCGATGTAGACCTGTGTCGTGGTTTCATTTACGGGGGCTGCTACGGAAACGCCAACAGGTTTGACAGCTGCACGAAGTGCATGAAGCGGTGCAGTGGGAAGAACCACGCTTGGAAAATCTGCAAGAAGAGGACCGAAGCATTTCGCAAGAGATACAACTTAGGGCTTCAGCCAAAGCGCAAAACATCCAAGTGGCCTTTCATATTAAGCATTCCATTCATAAAGAACTAAGTATGTGTACAAAGCATATTAAACCAAAATGTTCTTAGCGATTAAATAAAAAACTTGAACGACGCG |
| >SG96909  CAATGCCTCAAACCGTCAAATAGAAGGCATCAGCGCCAAGCCTATAAAACACTGGCTCACACAAAGGCTGAGCTTGCTTTCATAAAGTTTAACGAAAGTTTTTTCTGGTGATATAGGACGCAAGTGCCGTCATGAAGCGTTATGCCATGGCTGTGTTGCTGGCATCAGCGGTTGCGGTTTTAAGAGGTGGAACAGTTGGCAATGAAGCCCCGCAATGTCAAAGCAAGAATAGCGCCAACGCCAACACAAAATATTCGTACTGGAGGCAATATGGCGAAGAGTGGTATCGGCGCGACTACTACTACGACAACGTTTCCGACACGTGCAAATTCCTCGGCTTTAAGGGATGTGACGGTAACAGAAATAACTTCCCGTCGGTTGATGACTGCATCTCTCATTGCAGGAAGGGCGTCAACTTAAACCATCCTGCTTCAAAACCGATTCTGGATCGCTTAAAGTACTGCAACATGACGTTTGACGCAAAGACAGACAGTGGAAACATTCGCCGTTTCTATTATAATTCTACCTCGCAACAATGCCAGCCGGTGGATGTAAAGAAAGGCGATCTATACTTTCCCGGTATGCATTTCTGCGTGAAGATATGCAATGCAACGAATTCTGAACTTCCACGCTG |
| >SG961002  CACAATGCTCGACCATTAGTACTTCAATATAAAAGTGGCGGCTTCCAGATTATGGCACGTATTGCTACTGAGAGCACAACAGCGTCGACTATGATTATAGCTCATTTCCTTATTCTTCTGGAGTTTCTGGTGTCCGCGCATGCGGCTAGTTATCGGACACCACAAAGTTGTTTGCAGTTACCTGCAGTTGGCCACTGCAAAGCAAAGTTCCCGAGATGGTATTATGATGTCTCAACAAAGCACTGCAAAGTATTTATATACGGCGGATGTGGTGGCAATTCCAACAGGTTTGAAAGTGAGGTCGAATGCCAAAAAACCTGCCTGCCTGGAGCTCCCGTGAAACGAGTTTGTAGCCTTAGGCTACCAAACAGATCTTGCAAGACGGGAGTTCATACATGGGCATATGACTCGGATGCTGGCCGCTGTCGCTTTTTCCTGCACGGCCACTGCAACCGAAACGCCAACAGGTTTTCAAACTGTCTGGATTGCATGAAAAGATGCAGCGGTGCAAAACCCAAAAATGCATGGAGAATCTGCCAAAAACTGACTACTGAAGTGATCGAAAAATATGGCAATGCTCTGCGCCCCCGCGTGGGAGGACCAGAGTAGGTTCGTCTATGCATGCCTTCCAGAAATGATAAGGCGATGTTTAGTTACGCAATAAAAGAAGCTTTTTTAATCAAATATCTGAAATGCAC |
| >SG961179  GCCAGCCACCTCCAGCCTATCTAACCACCACTATGCGCAGCCTTACTTTCTTGGTGTTGCTGATTGTATTTTTGTGCGCCGTGCTCCTCGTAGACGGCGAGAAGAAGAAACCTAAACCGGTATGCAGCCTTCCACCCGAACACAAGAAGTGTGCAAGGCTTAAAAGAAAAGGATGTTACTGCCCGAAGAATTTAGGTATTCCCGGCTTCGTACGCGAAGAGCGCTGGTTCTACAACAAAAAGAAGAAACAATGCGAATCGTTCGCGTGGTCGACTAATGGTGGAAACTGCAACAATTTCCCATCAAGAGAGAAATGCTACGAAACGTGTTCAAATTTCTAATGACGAAATAAATAGAACACAGCTTATTTATTGTCTAAA |
| >SG961452  TTAAAAAGGTCTTTTTATTGCATGAAAAAGCATCGCCTTATCATTTCCAGAAGGCATGCATAGACAAACCTACTCTGGTCCTCCAACGATGGGGCGCAGACGATTGCCATGTTTTTTAATAACTTCAGCAGTTAGTTTCAAGCAGAGTTTCTGTGCCTTTCCGGGTTGCATTCCGCTGCATCTGTCCATGCACTCGAGACAGCTTCGAAAGCTGTTGGCGTTTCGTTTGCATTCACCGTGCAAAAAAAAGCCACAGCGACCAGCATTCGAGTCAAACGCCCATGAATAAACTCGCCTCCCGCATTTTCCTTTTGGTGGCTTAAGGCTACAAACTGGTCTCACAGGAGCTCCAGGCAAGCAGGCTTCTTGGCATTTAACCTCGGTGTGAAACC |
| >SG961709  TGCTCCGAGAAGGAGCGCAGCGTGTACTCCTACAACCAGGACACGAAATTGTGCGAGAAAAGATCGGGCTGTCACAATCGCGGTAACAACTTCCCCAGCATGCCGGAGTGCCGCAAGGTCTGCGAACGACGAAGATCCCCGGAGGACTGTCGTCACTGGCCAAACCGTGGACCATGTTACGCATTTGCTTTCAAGTGGTACTACGACCACTACCACAAGAACTGCTTTCAGTTCTTATACGGCGGCTGCAACGGCGGACCAAACTTGTTTGACTCGAAGGAAGAATGCCTGGAAGTCTGCTTTCCCCACAAACGCATTCCCCGAGGCTGCTGACGTCAATATTTTGTATGATGGAAACGTGGACACAA |
| >SG962425  GCCAGTGCTACTCGAGGAGCGTTTTATTTGTGTTCATTTCATTATGCCTTCAGCGTTTACTTGGAACTTCAACTTGCTTCAGCTTGCAGTTCAGGACAGATCCTCAAGGAGGAGTAGCGCGCTTAAGGAAATGGACACAAATAACACGGTCAAAATAAACTCTTTCCTTTCACAGGTACAGCATATAAAATAAAACGAATTGCACCAAGCGACGCTGATGTCAATCGTTCCTGGTGAATGCTTCTCTGCAGGTGACTACCTTCCTTCCCTCCTGGTCTGCAGCATTCCATCTCAATCAGCCTCGGCGTCAACAAGGAAACGTTCTCTTGGATAAGCGCGCCTCAGCATAGCAGCTCCAGAACTCTTCGAACCCTCACTGCGCGCGTAGCATTTCGCTGCCCGCATCATGCACTCGGTGCGCACATTTGGAGCATGTCACAGATATATCTTGCGAGCCGAGTTCCTGGTTAAACAATATAAACCCTGGCTATGTATTCTTTAATCATTCTTTCAACTGTACCACGATTGTACGGGGAAGAAATCAAAAGCACGAAACAAAAACAGCTCACAATGATTTTTTTTTCACGCAAAACTACATTCATGCGCGTCTATCACCAGAAAAATATACAACAAAATAATAAAAAGCGTGCAAAAAAAGATGCCGCCACGGTCTGGAAGACGTTGAAGTAACGAGCGTTAGCTGTTTGGTTTGCTAGAATTGATATCAATGAGGTTAAAGTGAGGTACACCAATGGAAATACGAATTTGACATCTTGGTTCTATAAGTGACGTTACCGATCAATCACTAATTGGATAAATATGTGACGTCACCAATCAATCACCAGATCGGTATACTCATGACGTCACCAATCACCAATAGGGTTGATATGTCGATTTACTATGGGGGCTCCCGTCTTGAATTCCTCGGACTTCCTAGAAACAATGTCCCCCCTGATTATTTTCGTTTTGTTTTAAATGCCCTTGTGCTATTTGCTTTCAACCAGTTTTTGTGCCTTCCGTCGTTGAATGTCATTTAATGGTACTTGAGAGCAGTCAAGCTGTTCCTAACAACTTTTAGCCTTTAAATCTGTCCGCGACATGTTCTTTTTTCTGGAAAATAAACTGAACTGAATTGAGTTGAATTGAATGTCTCTGTCCTTTGCGCTGCGTTTGCACCATGGAGCACAAACTATCCCATCAACTCGCTCTCGAAAAAAGAAATAAAAGTAAACATGAACCTTAAAAACGCACACACCCACACTTGAGTTTTCAGCTCTTTACCGCGCGGCATGCAATGCAAATATTTTACTGCCGATGATTTTAGATGGTTGATATAGGTTTCTTTTCAGTTGTTCTTGTTTAAAAATCTATTCCACTTCTTTATAGCTCGCCCCGTATCATATGCTTTTTGTACCCGTTCCGACGAACAGCATTAAAAGCCATAAAAGTTTGTTGCATGCTCAAGCACTTCCCGTTAATGCGTTGATATAATCTTCGTGAATGACTGGGCAGGAAAGTGAGATAAGTATGGACTTTGTAGGAATTAATATTTATGCTATGCACCAACAAGGTACACCACCTGTGATGCCATCTCATGGAAGCTTTTTGGTGTGGTTTGCTATTTTGACCAAATGCCCAACAAGAATTATTGGTGGACCTTCCCTAACAAAGAGCAGAATGACCATGGTAGGCACGCTCAGTCGGCGATGAAGCAGCTGCACGCCCCCTTCAAAGCCGGCAGTGGGGACGGCAGCTTCTCGCAAAACGATGGCAAAGATGCCACCTGTTATGAAATTGCCATTCATTGCTGACCATAATGCGCAACTCTTGCACAGAAATGTCCTTGCTAATGTGGTACTTTCTTTCTCCGCTAAGTTCTGCTTGAAAATTGTGCCTTCGTGATCCGCGAGTTAACCTCGAACACAGCAAATGACAACGTCGTAGTTTAACGCACGGCACGCGCCAACAACTGCTCATGTAGGCGGACAAGCTGTAGTCACGTGCTCTTTCGCACCTGTAAACCAGAATCAAAATAAATGCAGTTAAAACTCGATTCAACTAAGCGAGAAGCAGCCGTTAAAAATTTCGTTAAATCGGGAAGTTCGTAAAATCGAAGACGGCATTTTTACGGCCTTTGAGATCTTAAATTCTAACGTAGCGGCACTGAAAAGCTACCGAGGCGTCGCATTTGCCGACAGCCCACGCCTTCGCGTATGAAAAGTCCGCGAAAGCGGCCCGAATCGCTCGTGCATAAAAGCACCATATGACAAACCGGATTCGAGGCCAAGGTGGAAAGCACCGCCGACTGCCGAGTCCTATGTTCCGTTCAGCAGCGCAGCATGCAGCCCCGTCAGAAGAAAACCAGGGTCGTGAGTTGGGCGCGGAGATGTACTGCTGGCACGCTTCCAACAAGCTGCGAGACATCGTCGTTTATTCCCGGAAAAACTTCATTAAATAGGGTTTAGTGATCGTTTCTTTCGTTATTTCAGGTTATTCGAACATTGAACCCTATGAATACTTGCCGGGGTATAAAATTTTTTTCGTTAGATCGAGAACTTCACAAAATCGGCTTTCGTTAAATCGAGTTTTAACTGGTGCCGTAGTTACTGATGATGAGGCTGGCTGGACCGTTCGCACCTATCATTATAGGGGGAAGGAGGGTATTGTAAGAGAGTTAAAAAATTTACTTTGAGGAGCTGCTACCCTCACACGATGCCAGCTCACGTGTGACATAGCAATTCACTTCTTGAATGCGGAGGTTTTTGATATGTGTTCGTTTGCCCGGTATGTCACCAGCACTTTCAAGCATTCCTAATTATAGGGTGTTTCAAACACAGAGGAAAACTGTTCAGGAACACACAATCGGCCGCAGTGGAGGTGAGTGACTTTACAACTATAGTAATCAAATCGCTACTTTAATGAGATTTAATAAATTATTTTGGAATGAACAGAGTCTGGTCGTTAGCATAACAAAAATGTAACATGAAAACAGACCAATGCTGGCAAGATAATCCTAGGAACCATAGACACGGTACTTCGAACTGCTTTTGCTAATACACCACGGAAAGTGCTCTTAAGCTTCGTCGCAATGTGCAAGAAAATGAATACTCATCGTAAACTACGGGGCTGCCAGTAGTGAGAGCTAATTATTTAGCAAGAAAAAAGCATATTTTCAAAATAATTCAGAACTGCCGCGGATAGCAGTCTCTTTTGAGACCACCCTATTTTGAGCAATTTTGGAAGGGCTCGCCGAAAAACGCTGTTAAATATAAGCTTCATTGGACCACGGCATGCACTTCATTTTGCTTCGCCCCTCTAAAAAATAACAGATACGCGGGTGGCTACGACTATAATTTTTTTGGACCAAGTGGCAAAGCAAACAAAAGTGTATGCCGTGGTGCAAAATGTGCGGGCAGTTAGAATTGGGCGCCGTACACTCTAAACGCAGAAAGAATAAAACAGGTGTACGCTTTCCTCTAGCGCACTTTTTTTGTGAAAGGGAGTGCACTTTAGGACAGATTACTTCATTTTCACTGCAATAGCGGTGTATTTGTGTTGAGACTGTGGGCCCTGCACCGTGTCACCAGCGGTCGTTGTGTTTGCACTGGGCTCCCTGCACACCTCATGCACTCAGTTTCGGTATTGTGCGAAAAAGAGCCTCGCATTTACTCGCACTGCACGGTTTTACAGAACATTTTCAGAAACTGATAAGGCATCGTCTTTCCCTTCGCTTCCAGCTCTCAATTAGCACTAGTCACCACACCGTTTTAACCAAAACGCTAATCTTAAAGCGTACTTTGTCATATGCCTTACTAGTGCGAATCGTTTAGTAACTGACGTCCGCCGAGGGTGCGAATGTATTTATACGGAGATTTTTCCTCTTTAACCATAAGATTAAGAATTTTTGAAAGTGCTTACTGAAACCACAGAGATAATTTCTTCGTTTTGTGTCACTAAAAGTAAAAGGCATTGAATGGCCTGGTAGAACACCACGAATGCGCCAGACACACTATTGTCTGGGACAGAATTACAATGATGGCTATCGAAAAGCCATTCTTATCATGTCTGCACCTGATGTCATTTCATATCCACACAACCAATCGCGCACAGACACGTGAGCCCTTGCCCGAAACATACTCATGATCACTGTGGCGCTGATCGCGCATATCAAGGCGGCTGTCCAGTCATTCCTTTTACATGTGAACATTGCGCTTGTAAGGGTCTCGAAAAGCCCCCGCCTTTGTATTTTACCCTTCTGTTTGGCACTCGTCGATTTCTCCTCCTGCAATGTCTTTTCCTGGCCAAACGGGCTGTCGAACCTTGGACTAGTAGGCGATAGTATGCGCGCTTGGCGAACGGCTGTCGTCGACCGACATTCCCCGTGCTAGGGTAATGACAGCAACCAGGCATTCACGTCACCGATTTGCTTGTGCGACCTAAAAACCTCGTGTTGCTCCTTCCCCGTGCCACCATGGAACCTCGGTTGTTCCAAATTCTTTTCGCGCGCACTATGCCTAACGCCACTGCGTCCCTATCGTTAGTTGCGGCGCATGTGCCAAGACTACGAGCGGCACCGCACACAATCGCGCACATCCTTACAATTGCTGTGCCGCGCTGCCCGTCGGCTCGGCTCTATCTGTGGCAGGCTCACGCTAAGTGGAGGAGCGCGTCGAGGCAGATGCGCCGTGGTTGAACTTGCCGATAGTCTCCACGCCGCCAGAGGCATTTGTTGCGTTAGAAAATTTTGTGCAGAGGGCAAGATTGCCTGGCTGCAGTTATCCAGTGTTGCTGAAAAAGACGCTGCTTGTAAGCAAATTCCGTTGACTGCCACAAAGACAATGCATATTTTAGAGATTGGGCGCACCGATGGAATGCGACAGAAAGTTCATATCGCCAGGGACACAAATTGCATTCGTGGACGTCCTTGGCAGTTACTGCCAGTGCGCGCAGATTTCACTACACACCGCGTGCGCCTGTGATCACGCATGCGCTAATCGAGAATCTGGGAACAAAAAAGAAATATTTCCGTCCTCCTCGTCGCTTCTTCAGAGAGTGCAGAGCGACGCTGTCACCGATGCACCAGTGAGGAACACTAGAGCGTAGAGGGCGGCCCCGTTGCTACGCGGGGCTGCGTTCCCGCACTCGCATGACCCGCACTTGGCCTCCTGCTCATCACCGCAGCTGCACTGCATGGACTCCGGGCAGCTCGCAGCCGTCCTGGCCAAGTTGATGCGCACACCAAAGCAGGTCATGTTGCATCCGTCTACGGTACGGAAGTTGTTGTCGTTTCCACCACATCCCTGGTAGTTGAACCGCTCGCACAGTTCTTTCTGCACGTCGTAGTAGAAGCGGATCTCTGGTGTAGTGGCCCCTGGGCCACTGCTGTCGGTGTCGCAGTGGCCTTCGTCCACCGGCTGGTGACACATTTCCCTGTCCTTTCCGGCTACGCCGTCCTCAGCAACATGCACCCTGTCCTTTCCGGCTGCGCCGTCCTCAGCAACATGCAATCGGCAGCGTCCGTTGCACTGGGTGCTGGTGTAAAAGTTGTTTCCGTTGCCCATGCAGCCACCGAAGATGAAAGGCAGGCAGCGATCCTCCTGCTTATTGTAGTAGAAGCGCCGGAAGTACGCGTAGCATGGGCCCGGGTCTTGGGGCTGCGAGCAAACATCTTCGCAAAGCGTCTTGCACGCGTCGAGGTCTGCAAAGTTGTTCTTGTTCCCTTCGCAGCCGCCGTACTCGAAGGTCTCACACAATCCCGTCTGTGAGTTGAAGTAGTAGCGCTCCTGCGTGTCGTTGCAGCTGCCAGGATCCATTGGCAGGCGGCAGGGATCTTCACACTGGGCAATGCACTCTTCATGGGAGGCGAAGCTGTTCTCGTTCCCACCACAGCCGGTGTAGTAGAACACGTCGCAAACGCCGGAAGTGACGTTGAAGAAGTAGCGGGGAATCCTTTCATTGCAGTTGCCTTCTTTCACCGGCAACTTGCACGCACCATCAGACGGACAGTGACAGCTCTGGGCGCCGCTGCTCGAAATTCGCTTCACGCAAGTCACTGGGCAGGGCACTGCGATTTGGGCATCCACGTACTCCAGTGCATATTCAACAGTGCCGTCTGCAGCAGCACATTCGCAGGCGTAGCACGACGCAGACACCTCCCTACTCTGACAACCAGCCGGGCACAGCTCGGGAGCCGCGAGGCAGTCAGCTTTCTTGAGAAGGGGCTCTGTTTCGGACGCGAGGCAATTGCACGCAATACAGCTGTCTTTCTCTCGCCGCTGGCAGCAGTGAGCGCAGGCTAGGCCGCATGCTGCACGCTGGGGACACTGGGCTGATTCGGATTCCACAGGTGGAGCTCGAGCTTGCTCCAGTGGCTTCTCGCATCTTTCAACACATTCTTGGCGAGTAAGGAAGTGGTTTTCGTTGGCTCCACAACCGGTGAAAGTGAAGTTCAGGCAGCTGCGTTGCTCTCGGTCGTAGTAGAAGACTGTCTGCTTATTCGCACAGAAGCCTTCCTTTCGTTCCAGGAAGCATATTCCCGTGCCTGGTGGTATTTCGGCCAAGGTGGGATCTTCATCGGACGGTAGAGTGCATGCTTCGTCAAACTCGTGCGGTTCCAGCTGTGCGACAGCAACGCAGACGTTCTCCAAGTGGCGCGTGATCTCCAGAGAGGTGTCTCGTTCGAGCACGTGCCTCGCAAGGACGCTGACGTGTGTCGCATCCGGGGCACACACCGACGCGTGGGGCTGCTCATGCGCGCACCGGTGCAGCACGGCGTAGCTCCCGTAGTCCGTGGCTACCACGGCCAGCGTCTCTTGTACAGGCTGCCCAAAAAAGTCGTACTCGAGCTGAAATTGTCCGACGGGCAGCACTTTGTGGACTTTGCCGACAATGGCCGGCAGGCATGTCTCATTGCCTGCAAGTGCTGCGCTAAAGGCGAACGTGTTGGTGTCGAAGTCAAAGCGCCAAGAGCCTTTCTGCAGGAGAACCGGAGCGTGCTTGCTCTTCAACACCTCTCGCCAGTCTCCCGAGAACTTCTTGTAATCGAGGTCCTCGAGGGCGCTGGCCGGCTTGGGGACACACACCAGGGCCTCTGCCGGTGTCGTCGGCGCGGGCAGTATTCCAAGGCCCGTCAGCGAGGTGACAAGGAACCACGACAAAGCAGCCGCAGAGGCCATGGCGTCTCAGCCGCTGCCTGCTTCGC |
| >SG963021  CTTTATCTCTCCGGCATGTCTCTTGCGCAGTGCTTGATGCAGTAGTTGCGGGAGGGGAAACTATTTGCGCTCCCTCCGCAGCCCCAGTACCAAAAAGGTATGCACGTGCCGGTGCGAGGTTTGTAGTAGAAACGTATTGAGACGAAACCCCATCGGCAAGGGTACCCATGAGTCGGAGACACGAAACACTCTCGAGGTATTGTTGGCTTCGGTCGACGTCTCATACACGTTTCCTGGCATTCCTTAATGGTGGGAAAGTTATTTCCATAGTATATGCACCCCATGTCCTTGAGGCAGCTGCGTGTCGAGGGATCATAGTAGAAAACAGGGTGTATATCGCTGGCGCAGGAAGACACAGCACGTGGAGCCGTGCACACGTCAACACTACGACAGTATCCATCGCAAGCAAGCTTGCTGTCGAACGGGCCGTCCTTGTTGCACGTCTCATTACACTTCTTTTCAATTAAGTTGAACTGCCATGAAGGTGTTGAGCATCGTCTTGTATTAACGATTGGTTTGTTTGAGATGCATCTTGGATCTCTCGTGGATCGGCTTGTTGCTGCGCAGAGCCCTAAATAAAGAAAAACTGCCAACGCCGGGTACATTTCCATGTCTTTTCAGCCAAGTGGAAGCTTGCTTGGGAAATGTTCTTGCCTCTTCGTTCTCTTATAAGCTCACTGCGGTGCTTTGATTGCATATGG |
| >SG963193  CGCCGACAACCATTTATTGTGCTGGAGAGGCTTTCTTATGCTATTGCTAGACATAATTGATGCTAGCAGTTTATAAATGTTAGAAGGTGGCCGTTATTAGAATCGTCTTGCGTTGGGCGCACCCCACGGTCTGAGGTTCGCACTCGCTAGTTTCTCCAGTTTTCGACAAATCCTCTTGATTCTCTGCCGGGGTTTTCCTGTGCAGACCCGCATGCATTGCTCGCAGGTGTCAAATTTGTTGCCATTTCCCCCACAGCCGCCGTAAATAAAAACCCTACATTCTCCTTTGATCCGGTTAAAATACCACATTGGAAAGTAGGCTCTGCACACTCCAGACTCTTTTTGAAGCCCGCAGACTTTGTTTCGGGCACGTCCTTGGGAGACAGAGAGGCAGAAGAGAAGGATAAGAAGGTACAGCTGCAATCGCGGAATTGATTTCATCTTTCAGATTTGGCGCTGCCCTGGGCTTTCTTGCGATACCGCTAAAAATTGACAGTTCAACGCTGCCTGTTACTTGCGGTAGTCTATAGGTGAATTCCCTGTTATGGGACTGAATGCGCAACTATTCTGGGAGCGTCGCAAGAGGAGAGCTCGGTTGGCACAAGTATCGGCGCGCACGTG |
| >SG963201  GTATAAAAACTCCCTCATTTCCTTATGAATGGAATCCTTAGTGACAGCTGGGGCCACGACGGTTTTTTGGGATGCAGGTTTGATCCCAGGTTGTTTTCTTCGAGAAATTTTTTAGTTTGCTTCTTGCATATTTTTCTTGCGTTGTTATTTCCGCTGCATCGTTTCATGCACTTGAGACAAGTTTCAAACCTATTGGCGTTTCCTTTGCAGCCACCGTAAATGAGGCCACGGCATTCGTCCAATTCGGGGTCGTAGTACCATAATGGAATAGCGGCCTTGCATTTACCAGTTTTCGGCTTCAGGCTGCAAACCTCCTTCCGCTTAGATTTGCGTAAGCAGGATTTCTGGCATTCTTCTTCGGTTGGAAACCTATTCGAATTCCCACCACATCCACTGTAGAGGAACCCCTTGCAAC |
| >SG963202  ACCTATTGGCGTTTCCTTTGCAACCACCGTAAATGAGGCCACGACATTCGTCCAATTCGGGGTCGTAGTACCATAATGGAATAGCGGCCTTGCATTTACCAGTTTTCGGCTTGAGGCTACATACCTCCTTGCGCTTAGATTTGCGTAAGCAGGATTTCTGACATTCTTCTTCGGTTGGAAACCTGTTCGAATTCCCGCCACATCCGCTGTAGAGGAACCCCTTGCAACGGAAGCTCCAAAAATCATAGTACCAGGCCGGAACCTTATTTTGACAGTTCCCCACAGCCTTCGGTCGGAAACAACGCGACCACTTTATACCTGCAGCTTGCCTGAGTACCAAAGTAAAGACGAAAGCACCTATGAAAGCTTGTGGTCTCATATTGCAAGCTGCGGCACTGAGGTAACTGAAGCCAAACAGCAACCGTTGGCCACTTCTTTTGTGTTCATGTCGCGCAGGTGTACATGTGCT |
| >SG963262  CAATATTTATTTGTTAGTATGCATTCGCAATTGTGTTGTTACACGAGAGCCTTCTATTTCGTATCTTCGGAAGATGATTGTACGTAGTATCAATATTGTGCCTTCTCTTCTGATGAATCGCCCAATAACATCAGCCGTGTGAAATTGAACCTGCCACATGCCTTCTGGCATTGATCCCGGGATTCAAAGTTGTTTAGGTTGCCGCCGCAGCCGCCGTAGAAAAATCCCTCGCACCATTGCCCGTTGTACCACCAACG |
| >SG963394  TTTGTGGGAGCTCGCAAAATGCACGTCCGAAGGCACTCTTTCTCAGTTTTAAAGTTGGCGCCTGCTCGGTTGCATCCATAAGAGCGCTTTTTGCAGGTACCCGTGGTTTGGTCGTAGAAGTACGTGGTAAGTTTTTTTTCACAGACCTCGCTGTCAAGTGGAGGGCTTATGCACTGCTGTCCTTTGCAAGTCACTTGACATTCTCGCAATGAAGAAAATCTGTTGCCGTTTCCATCGCATCCTTTGTACAAAAACAATATGCAATTGCAACTTTTGTTGTCATAATAGTACATCAACTGAGGGGTTCCACATTCGCTTCCGGCTTTCATCGGCATTAAGCAGTCCGTCCCTGCTCGACAGCTCCAGTTGCACATTTCCTCTGTAGAGAACAGATAGACACCATAATGAGCAGTCCGTGTCGTTTTGATACATTCATGTTGTGACTCATTATAATAGTAGCCGGTGATGAGGTTGGACCATTGTTTCCAGTTCGAATTGCATTTATCTTCTGCTGCAGACCCCACAGTGGCGAAGAACCCTGTTAGAGCAATCACGAACCCCGAAAACGCAGTCATTATGCTTTTCCAACTGGTCACGTCTGTGAAATGCTTTAAAATCTTGCTATGAAGGTGCCTTGGAATTCGTTAAGGTTAAAAAAAA |
| >SG963940  TTCTTTTATTGCATACCAAAACATCGCTTTATCATTTCCGGAATGCGTGCATAGACAAACCTACTCTTGTCTTCCAACGATGGGGCGGAGAGCCTTGCCATATTTTTCGATCACTTCAGTAGTCAGTTTCTTGCAGAGCCTCCGTGCATTTTCAGGTGTCGTTCCGCTGCATCTTCCCATGCACTGGAGACAGGTTGAAAACCTGTTGGCGTTTCGATTGCAGTCGCCGTGCAGAAAAAAGCGACAGAGGCCAGCTCCGAAGTCATATGCCCATGTATAACCTCCCTTCTGGCAAGTTTGTTTTGGTGGCCTAAGGCTACAAACTGGCATCACTGGAACTCCAGGCAAGCAGGTTTTTTGGCATCCGGCCTCGTATTTAAACCTGTTCTTATTACCTTTGCATCCGCCGTATACAAATGCTTTGCAGTTGTTTGTTGACGGATCATAATACCAACTCGGGACCTTTGCTTTGCAGGGGCCAACTGCAGGTGGGTTCAAACAACGTGGTGGTCTCGCATAACTAGTCGCGTGCGCGGACACCACGAACCCCATAAGAATAAGGAAATTAGCTGTATTCATGGTCGACGCTGTTGTGC |
| >SG964180  AGAAGTCTATAGACTGTCTATAGACCATTTTATAAGGGTCCAGTGAGCTGCCCTCCCTGTCCGCGACCGCGGCGCCCTCTTGCGGTCAGTCCGGGATGCAGACCGCCCGGCATTCCTGGGCGGAGCGGAATCTGTTGAGGTTGCCGCCACAGCCGGAAAACCAAAAAGGAAAGCAGTGGTTCTTTTCTTTGTCGTAGTGATACCGCACCACCCAGGAGCCGCATTGGCCTCGGTCCATTGGAAGCTTGCAGTAGATGTTGTAGTTTTGGAACCAGCAAGTCCTCATGCACATCTGTCTGCTTCTGAAATTGTTTCCGGTGCCCTCGCAACCAGTGTAGACGTAGCGGATGCACCGTCCCACGCCCCAGTGGAAGTAGTAGCGGAACAGCCTCCCGTTGCACCTTCCGGCATTTGGAGGCACGAAACAGTTGGGCTCCGTTCTTTCGTGAGGTCTGGAACGCAGCTCGCCGGTGGTGTCACTTTCGCCATGTGTCTCGTTGGCGCCGACGTTGGCAGTGACGTTGGCACTGACGTTGGCCTGAGCCGTGACGGTGGCGAGACACGCCGATAACACCAGCAATACGGCCAGTAGGTATGTACCGTTCGTCACTCCCAGAAGTTTAAATCGACTCCTAGCGCCATATCTCAGCCACCTTCTTGCCATTACCAATCCGCTCGCGCACAGACCGAGGGAAAACCCGCAAAACACGAAGTCCACAGCAGCTCTTGCTTTGCCAGCGAAGCGATGTCCGCCGCAGCAGCAGGGGGTCGCCCAGCGTTCTTCTCATTGTCCTCTTGTGCCG |
| >SG965850  GTCTCTGCCGACAGTAATACATCCCCAGATCTCACCAAGAAAGCGATGGCAATTAATTACTGCATCTTGCTCACCCTTTTCGCAGCGGCTTTCGCGCAGCGAAACGCCATCTGCCGGCTGCCGCCAGACGAAGGCATCTGCCGGGCAAGCATTCCGCGGTTCTACTTCAACCCAGCTGAAGGAAAATGCTCCTTCTTTATCTACGGTGGTTGTGAAGGAAACGAAAACAACTTCGAAACTATCGAGGAATGCGAAAAAACATGTGGTGAACCAGAGAAGCCCAGTGACTTCGAGGGAGCTGACTTCGAGACTGGTTGCGCGCCAAAACCGCAACGCGGCTTTTGCAAGGGCTTCTTAGACCACTGGTTCTTCAACGTGACCTCGGGTCAGTGCGAGACGTTCCTCTACAGTGGCTGCGGTGGAAACGACAACAACTACGAGTCGAAGGAAGAGTGCGAGATTGCCTGCAAGCTTACGTAGCCCGCTGGACAGATGAAATGAACACTTCGCTCTCTCTTTTGCCCTGCTCATTCCGCACATGCATTGGGCGAACACAAAGCCTGCAAGACATGAGAGAACGGCTGTTGTAATTTTTCAGAGAAATAAAAAGAAAATACCCCAAG |
| >SG965891  TGAGTCGAACAAGTTTTCATGCAGGATGTACAGTTGAGAAAGATATTCCTATTTCCGCCGCATCCGCCCCNNNNNAAGGGACCGCAGTACCCGTATCGGTCATCAAAGTACCATCTTTCATGTGTAGGCCGCTCGTGTCCACATTGTCCAACCTTTGGTGCTGTCTTGCAATATTTTGGGGGTGTGTACCCTTGCACTAATACTACTGTGACACAGAGCAGGGCGTACAGAAAGCAGCTTGGATTCTTCATCGTTAAGAAAGCTAGGAAAAGACTTGTCCTACTGTAGGGTTTCGCGGTTGGGTTTATATACGCAAGCTAAAGCAACATTTCGAGTGACGTTGCCGGTGAAAACGTGCGCAAGCAGCCTTTNNNNNNNNNNGAACGGGAATTATAGCATGATAAATGTCAACATATTCTCGTCAGATTCACGTTATTTAAGAATAGCGTATATCTGCTAGCTTTGCTGCGGCACAATACAACGCCTAGTTTT |
| >SG966046  ACACACGTCACCAGTTCAAAGAGCACAATGCTCGCGAATCTGGGGTGCCTACTCGTTTCCATCGGTTTCTTAGCCGGTGGAGTGTGCGCGGACACAGATGTCCAGTGCGATTCGGACTTGAAAGAGGGGGTTGAAGCAGCCGAAGGCTACTACTACAACAAGACCATACATCGGTGCGTCTATACGAAGGCGACGGGATTCTCTTACGGCCATGTTTTCCCCACAGAAGAAATGTGCAACTGGAAGTGCCGAGCACACACTGACTGTTTACAACCGAAGAACCCACTAGGGGAAAGTGCATGCGGGCAACCTCAACTGATGTATTATTACGACAAGGACTCTTGCCAGTGTATATTGTTCCTAAATAAGGGTTGCCAAGATAATGGGAACAGCTTTCTAACGCTGCGAGAATGCACTGTCACTTGCCTCGGACGGCGCTGTATCGAAGCTCCTCCTGATGACGAGGACATCTGCAGCGGAAAATATGTGGCATTCCACTATGACAAGAATTCAGGCAACTGTGTAGAGCGTCGTTGGCCATGCTACCGGGAAGGTTCTAGATTTAAAACCAAAGGAGAATGTCAACGTTCATGTCTTTTGAAACGGCCGCCTCAAGCACCTAATGCATAGCAATGTATCGCTCAAACCACAATGGCCAGTTTTTGCCAGAGTGTACCGAAGGAGAGTGAATGAATAACGTAGGCTGCCATCGCATCAACAATATTTTTAACAAAGCAAGAATAAGTATTCGTAGTAGTAGTAGTAGTAGT |
| >SG966253  TTTTTTTAAGATAAAAATGACATAGAAAATCATTTATTTTGGCGCGCTCAGCCTGTTTTTTGGTCAAAACACGCGCGTCGCCTGCGGCAGTGCATTTCGAGCTGGCGCTTCTTTGAGCGTACGAGCCCTAATATCGGCCGCAGACTCTGCGGCATTGTCCAACAGAGTTGAATCTGTTCGCGTTGCCACCGCATCCGCCGTAGGTGAACGAGAGGCATCTTCCCGATCTACTATCGAAGTAGAAGGCTGGGAAGGCGCCGCGGCAGGGTCCCTGGCTAGGAGGAAGGAAGCACTGGCCTTGAGCATTGGTGCCTGCTACGACAATAGCCAGCAGCAAGGCGACGACCACAAGCGAACGAGGCATCTTGCGAACGGGCGGTCCGGGTGGGTGCGTTTCTCACGAGTGAGGATGCTGGGCAGCCTTGAGTCGGTCAAATATGCGCGCGGCGGCGTCCTGCCGAGCAGCAACGGCACCTTCGAGATTCCGGTTTCCTGGGCGCGCAGCGGCGCACGTGTGGTCGGTTGG |
| >SG966337  AGTCGCTTATGTTTTTATTTTTGATAACGATGCCATCGCGTTTTTCCCCAGGCATTCACAGTCCAAGCGTCCAGAGTCGACTTCCAAAGCGTAGGGTAGCGTTGCTGGCTTCGCGTGCGTGTTCCGGCTCGCATCAGTCTTCCCCCCTCTCGTGGGCCTCCTTGCCCAGGCACTGCATGCGGCACTCGTCGCTGGATATGAAGTTGTTGCGGTTCCCTCCGAAGCCCAGGAAGACGAATGGGCGGCACGACTGCTTCTTCGAGTCGAAGTAGTAATGCAGCACGCGCGAAGGGCCGTAGCCACGATTAGGGGGCAGCTTGCAGATTCTCGACGTAATAACGCTTGCGCATCGTCGTAGGCACTGCTTGCGGCTTTTGAAGTTGTTGTCGTTGCCTTCGCATCCGCCGTAGACGAAGAGGCGGCAAGTCCCTCGCATGAAGTTGAAGTAGTAGCGGTACACTGTGCCTTTGCAGGGTCCCCGATCGGGGTACTCCGTGCACTGCATGTAGGCTTTGTCTTCCTCGAAACACACGCATCCTGTTAACGACACCAGAAATACGGTCGCTGCAAGCGCAATCCAAGTCATATTTTCCAGAGATGTCGTCCCAAGTCTTTAAGCCGTAGCTTCAGGGAGCGCTGCAGTGTCGGCCCTCACATTGGGCAGGGTAATCGGTCCAACCGTCCAGCTGTCTCGGATCGTCAGGCCTCAAGCAGGTGCA |
| >SG966698  CGACACCACGATTGGTACTGTAAATAAGGTACTATGATGTTGCGCACCCTAATCATGCTCTTTCTCTATGACTTTTCAGCAGGCCAATCATCGGTAGCGAATGATCCCAAATGCACTCTGGAGAGCCCCATCATAACGACACCTGGTTGCGCCTCTCCTGGCTGGCAGTTTGATCAAAAGTCGAGAGAATGCGTATCCACGTGCAATTCCGCTGCGCCGTTTAAAGACAAAACACTCTGCGATGCAACTTGTCGAAGCAGAGAAGTTTGCACAGCTCCCCGAGCTGCTACTGGATGCGTAGCTGGTTCTGAAGTGAATGTCTTCTACTATGAGCCCCATTCGAAAAATTGCCTTCCTGATTTATCCTGCGGCTACACCGGCAATAATTTTCCAACCGCAGCCGAATGCATCAAGACTTGCGGTGGCCAAATTGGGCAACCGGACTACTGCAGACATCCCCCAAATCAAGGATACACCTGCGACAGAGCAATGGGATCATATCGTTACTTCTACGACGTTAGTGCAAGGCAATGCTTATGGTTTCCTTACTTTGGATGCGGTGGCGGACTGAACAATTTCTTGACGTATGAACAGTGCCGTAAGCACTGTATGCAAGGCTGAGAGTGATAAGTTGAATTGTTGGAGAACAGCGAAAAGCGAACGCCGGAAGGTTGCAGCAGCAGTTCTGTGCTGGTTGCGATTAAAAATAAAACAGTCTTGAAGAAATAAAAAAA |
| >SG968216  CTGCGCTGTTGATCCCACTGCTTGCACGTATTTTGCTCCGCTGCATCCATAATGAAGTCCTGGGTCTTCATACTGCTGGCTTGTTTCACACTACTTGAGTGGTCAACAACGATAAGTATCCCTGAAGAAGGGCATGAAGACGTATGCAGCAAGCCTCCCGAAAAAGGCGAGAGCTGCAGTAGTGGGTTCCAGGGCACAAAGTGGTACTTCAATGCAACAACAAGAACGTGCAGTACATTCATATACCTGGGATGCGGAGGAAATGAAAACAGGTTTCCTGACAACGAAACCTGCATACTAACATGCGACCCTCCTACTTACAGCTACGAGGAGTATGAAAAGCGACTTCAAGAGAGCAACAAGAAAGGAAAACAAACGAAAGGAAAACAAAGAAAAGGAACAGCAAATCCTGAAAAGTCGCAAGACTAAAAAATAATAATTCTGAATAAAGGCTGATCACCAGCCATGCATTCATTCAAG |
| >SG9610003  CTCTCTTGCGACGGACGATGTCGTAGCGTTGACGTTTGCACGGCTCCACGTGTTGTGTCTTCCTGCGCCGGCGATGTACACCCCGTTTTCTACTACGATCCCAGGACAGGCAACTGTCACAATGACACTGGGTGCATATACAATGGAAATAACTTTCCCACTATTACAGAGTGCAAGGAAACTTGTATGAGACGTCGACTGAAGCCAACAAAACCTTGGCAGTGTTTTGTGCTTCCGAGTAAAGGACACCCTTGTCCCTGGCGTTACAGCTCAAAGCGTTTTTACTACGATCCTTACACTGGCCAGTGCATACGTTTTCGGTACTACGGCTGCGGAGGGAGCGCAAATAATTTCCCCTCCCGCAATTACTGCATCAAATACTGCGCAAAGCACTAGAGGATGCTTTAAGACCTTGATTCTTCCCAGTGAAACATGGGTGACATGCCGGAGCAGCTAATTCAAGCAAGATAAAGAACTTATACCATTGGTCACGTAACATAAAACGGCATTATGATTACGGCTAAGCATCAGTTAGCTATTTATGCTGTGGATTTACTGAAAAATAAAACCTTCCTGCCCACGTAGC |
| >SG9610843  GCGCCGAAATTACAACCATTGCTTCATCAGTACCCATTGCCCAGTGGGGTTTAATCTTCAAGGCGAAATTCTTCAAATCCAAACTTCTCCCTCAGTCGCTGCAGTGGTTGATGACAAACTTTAGAAGTGAACCAAAGGAAAATGCAACGCCATCGGTTGTGCCGGTGGTTGCGCTCTTCCTCTTAAGCAGACTCTTTCGCATTCTTCTAGGCTTGCAAAGTTATTACCCATCAGAGTACAACCAACATCTTGAAAACATGATTGACTCCCAGGATAATAAAAAAAGACGGGAAAGACGTTTTCAGGTCTATCGCAGTAGCTGGCAGGGCGTGGAGCATAGCACACGGCAGGGTCTTTCGGGCTTATGCTGCTATGCATGGCCA |
| >SG9610983  TGCAGACTAGTTTTTTACTGTGCCATAACTTTGTAAGTGGGACAGCAGGTCTCACTTAGGTGTTTCAGTCAGGAACATAAGGTACGTAGCGCTGTAAATGGTAGTTCCGATCCAGAAGTGATGTCAGCCGCAGACGCGGAGGCATTCCTCATATGAAACAAAGCGATTCTGGTTTCCTTGGCATCCGCCGTAAACGAACTCTCGGCACGTCCCGGATGAAATGTCGTAATAGAACGAGCGGAAGTAGGCGAAGCAGACTCCGGGACTAGGCGGCAGGCTGCAAAATCTGGGCCTTCTCTGAGCATTGGCACCATTGAACGAGATGGCCAACAGGACGGCCGCCAGCACAGTCAATCGAAGCATCGTCGACACCTGAGATGGTCGCGGACGCCGGTGGAGTTCCGG |
| >SG9612675  GCCGACTGTTCCTTCTCAGTATGAAGGCCTACTTGTTCCTTGCTTTCATCGGCGCCGCTTGTGCGGCTACAAATGTTGACAAACAGTGCACTGCAAAAGCGGAAAAGGGACTTTGCAAGGCTAAGCTTCCAAGGTGGTGGTTCAATACGGATTCTGGCAAGTGCGAGCTCTTCTACTACGGAGGCTGCGGTGGCAACCAGAACAGATACCTCTACAAGGAGGACTGCGAAAAGACATGCGCTCCGAAAACACTGAACGAAACGCCTCTCACCACATTCAGCAACAAGAAGGCCAACTTCGATGACAAGAAGGGGCGGCTTCCCGGATCCGGCGTAGGCGTATGCATGGAGCCGCCATACACTGGGCCTTGCAAGGCGAGCTTCCTCCGATTCTACTACGACGCCAGCAGCAATACTTGCCGCCAGTTCACCTACGGCGGCTGCCGCAGCAACGGCAACAACTTTAAAGCACAACGTGATTGCATGAGGGCTTGCGGAGGTCGACGGCGCGGAGGTCTTCGGCCGCGCTAAGAAGAGCTCGAAGAATAATTCGTCGTGCCATTCTTTACACTATATGTATTTTTGCGAGTAGCGCTAAAGGGAACAGGGAAGAAAAAGAATCTAATGTAACGTAGTGATTCCGTACTTCTTAATTGTTGTTGTTAGCCCATCCTTTGATACGC |
| >SG9613017  CCAAAATACTTTATTTATCTGTCACAGTTGCTTCGCATCATGACAGAACGAAGGCATATCTTGCGAACAGGCCTTGCAGTCCCCATTACGCCTGGTTTTCCGCTCTTCACAGAGAGTGCTACAGTCAGCGAGTTCTCCCAGTGTTTCTACGGAGCTGGATGCAAAGCCTTGTCCTGTCCCCTGATATTCCTCCTTTTCGGCACGCGTTCAAGCACAGAGGGCAACTGTCAAATCTATTCTTATTTCCTCCGCATCCTCCGTACAGAAATTCTTCACAGCGTCCTTTGCGAATGTTGAACCACCAAGATGGAATAGATGCTCTGCAGCGTCCTTTTTCTTTTGGAAACTTGCATATCCGTTTGTTCGAACGAGCCGCCTCTGTGAAAGCCAGGCAAAAGAGAAGCAGAAAGAGGCACGCTTGTACTCTGATCGTGGCGCTCATATCTGAAATGTTCGGAAAACTAGGCTTTAATGCTATGCCACTAACCCGTTACCTTTTCGTCCAGCCTTCTTTTTTTCGAAATCTGCTGGATAAGTTATCTGCGCTGCTACTGTACTATGAAGGTGTCACAATACGTGTCCCCCGTAAGCGAAGAGACTG |
| >SG9614464  CAATACTATGGGAAGGACAAAAGGGAACTGCATAATTTACTAACAGCATGTATCTTTATACAGTAATCACATTAGACGAATTTTGCAATAATTACACCTAAAAAGTCCCAAGGCCTATATTAGTACTCTCACGAAAATAACCAAATGAAAATAGTATGCATAAGTGCAACCACACATGTGATGGCTTTCCAGAAACACGTTAGACCGTGGAAGTAAAAAGATAAAGAAAACCTTCACTTCATGACGCAACACTACAACACTGCAGTTAAAGAGGTTTTTAAGAGACTGGAGTCATCTTTATTTGTTGGAGACGCTAGTAAGCTAGTTTTCTTATTTTGTACTATTGTATGCACACATTTTTTGAGGCGGAACAATGTGATACAGCTGTCATTCTTGCTGCTGTTTTCCCAGCAAGATTGGGTATATGCACGTCAAGCGCTTTCTTGTCCAATCGGAAGATCCCAAGCTATTTTGGCTCGTCATCCCTTGTGCCTCCGGCGTCTTCGCAGACACGCCTGCACTCGCGAAGTGTCCGAAATCGGTTCGCATTTCCCCCGCACCCTCCGTAGACGAACCTTCTGCACCGACCGAACAGGCCGTCGTAGAAGTAGAGCACGTAGTGGCCGAAGCAGTTCCCGGAACTTGCGGGCAGCGAGCACAGCCTTCGCCCGCTTTGCGCACCGGCAACTGCACGAGGAGGGATCGGAGCTCGTGTTATATGCTTTTGAAAAGAAGGTGCCAGATTTTCTGCCCTGTCCCAAAATGAACTAGCGGCAGCGACAGCAGTATCATTCCTCTTTCAGCGGGAGCGCGCTCCAAGGGCCACAATGTTCAAGTCCAGTTCGATCATAATAGCGAACAGGCCACATTAGTGAGAGAAATTAAACTTCGTTAAAAAGATTAGCATTCAAATGTGGTCTTCTTATACCAGGATATATCGCTGGATGCTGCGTTACGTGCACGGTGGACGCGCGTGCTTTTAGGTTACGACAAAAGTATAGGGCCAAATACTTTTAATTTGGTTTAAG |
| >SG9615170  CACAAGTCCAAACCTATTCTTGGGACCAGGCTCTACGCACTTGCTGCCAGAGTCCCGGCCACAAGTGTTCCAGCACTCCCTTGCGGAGTCGAACCTGTTGGGGTAGCCACCGCAGCCGTAGTAGACGAACCTTTCACACCTTTTGTTCTTGTTGTTGTATCCAAATGCTACCTTACGTGTACCATTTGGGCACTTCTTTCCGCGATGTATTGGGTAACTGCAATTGCTTTCGACGTATTTGCCGCATGTTTTCCAGCATTCGGATTCGTAGAGAAAATTGTTTCCATTTCCTCCGCAGCCTTCGTAGTCAAACTGTTCGCAGGCTTTTGTTTGACTGTTGAACCAGAAGTTCTGCATGCG |
| >SG9618095  GCAGTAGCTTGCTTGACGCAGATTGTGTACCGGGCTTCATTATCCACGGTACACTCCCAACCTTGCGGCGCCTTGCCAACGATCTTTTCTGCCCTACAGCGTGGAAGCTCTGTACGTTTTTTCGGTTTGCAGAACTTTACGCAGCTGTTAATATCGGGGAAATACGCATCGCCGTCGCCACTCTTAACGAGCACTGGCCGGCATCGGTCCGAGGTAGAATTGTAGAGGAATCGCCGAATGCCACCCTGCATTGGGCGTTCAACAGGTTTCATGGCGCAGTCCGGGAAAAGCTTCTTGAAACGCTCGACAGCATGAGGCGATAGTGTCGTAGCCATTCTACAGTGAGCGGCACAATCGAAAAGCGACGGAAAATTATTCTTGTTGCCGTTGCATCCCATAAAGCCAAGGAAATTGCACGTTTTGCTGGGTGGGTCATAGTAGTAGTCACGCCGGCGGCAGGTTGTGGCTGTTGTATTTCGTTCACAAATCCAAAGATCGGACATCCTCTTGTCATTGGGATTTGTGCTGTTTTTACATTCCGGTTTTTCAATGTTTTCTTGCTGACCGCCTAAAACCGTAATCGCTGATGCCAGGAGTAAAGAGCCAGTGTAGAAATTCATTGCCACACTTGCTTCTTATGCCACAAAAAAGTGTAACCAAACTTTCAGAATGCGATCTTAGACCTAGTGTAAGCACCCGCTTTATACGCTTGACCTAGGAGTTTTATCGCATTCTGAAAGTTTGGTTACACTTTTTTGTGGCATAAGAAGCAAGTG |
| >SG9618446  CGCATGTTTCCTTGCACATGCCACTATCAGGAAACCTATTGGCATTTCCTCCGCATCCCAGGTATTCGAATGTACTGCACGTTTTTGTTGTTGCATTGAAGTTCCACATTTCACCGCTGCGCCCACCGACGCAAGGCGTGCCTTTGTCAGGAGCCTTGCCGCATTCGTCTTCAGCTTTTATTTCAGGGACATTCGTCGTAGTTGACCACTCAAGTAGTGTGA |
| >SG9623885  CACAATTAGTGATCTCGCGCCAAGAAACCAAGATGCATCTCCTAACTTCCATCCTCACCGTATGCGCTATCGTGGCTTCGGTTCTCTCCGAGCAAAACAAAACACGATGCGTGATGCCAAATATTGTAAGCGGGCCAAACTGTCGCCAAATCACCTTGAGGTACTTTTACAATACAACGTCCGAAATGTGCGAACACTTCCGGTGGAATGGCTGTGGCACAAAAGGCTTGTTTGGATCTCGCTACGATTGTGTCTCCACATGCAATGAAAACCAAGGCGCTCCATTCTGCGCTGATTCTCCACCAAGTCCTTGCGAGGAGGAGAAAGCTAAAAAAGGGAGAAAAAGGTATTACTACAACATAACGACACAAACCTGTGAGGAGTACATATTCTGCGGAGGACGGCAGAGCTTGCTCGACAATAATTATTTCATCGCAAGAGGATACTGCGAAAAGCAATGCGGAGGGTTCAACGAAGAAACAGCAAAGGCCAAGGATGAGTCCGTGG |
| >SG9624006  CGGACTGCCCCATAGTACCTTCGGTAAAATGAGAGCTCAAGCTATATACTCAGCATGTTTGGTTTACTTCGCTTTTGTGCACGCCGCAAGTTTCCGCATATGGCCACGTTGTTGGCGAGGAAAGGCTGTTGGAAGCTGCGGGAAGAAGATTCCGTCGTGGTATTATGACTTTTGGTCCGGGAAATGTAAAGGCTTTCTCTACAGCGGTTGTGGCGGGAATCCCAACAGATTTTCGTCTGAAGTCGAATGCCAAAGGTGGTGCATAGGCCGAGCTAGTGCTGCTGAATTCTGCAGCCTGAAACCAATAGTAGGCAACTGCGAATCATTTATCCCATCGTGGTTCTATGACCCGGAACACGATTGCTGTCGTGGTTTCATTTACGGTGGCTGCAAAGGAAAC |
| >SG9627987  CCGGCGTCCGGCGTTCGGAACGATTTCTTCTCTGTACAGTTCAATGTTTTAATGTCGAACACGAAAGAGGTTACCCTTGGAATTAACCCTCTGTGCTTGATAGGAGTCTTCAGGCATAGTGAATCGGGGTTGCACACTCTCATGCACTCCGTGAAATTGTCAAAACTGTTACGAATGTTTGGCTTGCACCTTGAGTGCCAGAAGTGGTCGCACTTTCTAGTTGAAGGGTCATATGTGAATTTCAGTTCAGGCCCTTGGTTCCCG |
| >SG9629093  CTTGCGCCGCCAGCATGAAGTCCCTGGTCTGCCTGCTCCTCATCTCCCTTTCACTCGTGTGCCATGCGGCGCACAAGAAAGCTTGCAACCTGAAGAAGGACCCCGGCAACTGCGAGGACGCCTCCACCAAATGGTACTACGACAGCAAGACCAACGCGTGCAAGCTGTTCGTGTACGGCGGATGCGACGGCAACGACAACCGCTTCGACACAGAGGCCAAGTGCAAGGCGGAATGCGTGCATCCCCGCAAGACGACCAGCGGATAGGGGCGGCAGCAGCAGAGCCAGCCTCACTGACGGCAGCGGCGATCCGGTTCGACACATTAAAGACTGCTTGCACATGG |
| >SG9629562  CTGGTCGGTAGTTGGGTTTCCCGTCGGCACTTCGGTATGCTTCAATCCTGCACAGCTCTTCCTCATGGCACGAGAACGTCTCGCAAGGGTTGCTGCAGCGACANNNNNNNNNNNNNNNNNNNNNNNCAGTCTTATTCTTTCCCAAGGGGCACTGCAGTTCCTCACAGTTTGACGGCAGGCACTCTGGCGCTGGCGTGCGGGGCACAGTTGCCGGTGTCAACGGAATAGGCTCTGGAAGTGGTGTGACTGCCATTGGTGGCTTCACACGTGGGTCCAACTCTTCGCTTGTGTCACTAGGCTCTGGAGGGGAAGGCGGCATCCATTTCCTGTCCGCATCAGGATCGATGATTTCGTCATCAATGCTGTCCATTGTGACCCCAGAGCAGATCTTCCGGCAGATCTCGGCCGACTTGAAGCGGTTCTTATTGCCTCCACAGCCGNNNNNNNNNNNNNNNNNNNNNNNCCCGCTCTTTACGTCGTAGAACCATTTAGCATGCATTTCACTG |
| >SG9630704  GCTTAGAGAAGTGAAGAAAGTGATGAAATCCTTGCAAAGAATTTCATTGTGAATTTTCATTCCTGTGGTGGCTCGAATTCCTCAGCAGTAGGTGCACCTTGGTTGAGGTTGTGCAAATACTCTTCCATGTCATAGGTCGGTGGATCGCATATATCATAACACATTTGTTCGGTTTGAAACCGATTGTCATTGCCGCCGCATCCTCTATAATTGAAGGGGCGGCATTCATTTGTGGCAGAGTTGAAGTACCACATCTCCCCTTCCGGGTTCTTAATGCCAGGCTTGTCTTGTCTACACTTCTTTCCTTTTCTAGGTGGCAGGTTGCAGACAGCCTCTTGTTCCT |
| >SG9633063  ATTTTGGGGGAATTTAAACCCGCCACAATGCATTGTACAAAGCACCATTGAGCTAAAGGAATTGGCCGTCAATTCTTCTCCAATAACTTCATACATTAGGTATCGCTCGCAAGTTCCAGTTTTGGCGTTGTAGAAATATCTGACTCTTTTTCGGGGGTCCTCTTCGTATGTAGAAAAAGGAGGCCAGTGTAAGCAAATGCCATCTTCTTCATCTGGGATACCACACTCCGTTGCGCACTCAAAGCGCGTCTCGAACAGGCCTTCCGAGATACAGCCGTTCCATTTCACATACTGGCACTCTTCAGACACTGGATTGTAGTAATACCTTGGGACTCTCGCAGTGCATTGTGGTTCGTCGTCGTTAATTGGCATACGGCACTTTTTTTTTACGTATCCCACGCT |
| >SG9637142  TGAACCGGTACCCCTTATATCCAGCTCGGCAAATTTGTTTGGGCTCTGGCAGGGCACACACTCCGAGAACAGGGTCTCGACATTTCTTACTGCATTCCGCAAATTTGTCGAACCTGTTGCCGTTTCCATTGCAGCCTCCGTAGTAAAATACCTCGCAACGGTACGACGTCGCGTTGTAAAACCATTTCTTGAACATCCCTCTGCATATTCCGGGATCCGGCTCTTGCATGCAGTCTTCGCTGGGCGGCACAGTTTTACTGGCAGCGCAGATTCCTG |
| >SG9638880  TGAGATGCAAAAATGATTTTTTTATTTCGTTATTCAACGAACAGTAATGCAGCTTGCGATGCTAGCTGAGGATACCCGATCTGCGGTTAAACATTATTCATCATTTCACTTAATTGGGAGAAACTTCAGAAAGATATCTGCATTCTTCTCGTGGGTCAGTCCTGTCAGCGCATGTCTGCATGCAGCTCTGGCAGGTCGAGAACACGTTCCGCCTAGCGTTGCATCCGTTGTAGCTGAACAGAAAACAAACTTCGTAGGTTCGATTGTAGTACCACCTTTGAACATTGCTATTGAAGCGTCTACAATTCTGGCGAGGTGCTTTATTGAAGCAGTTGCGGGGATTCTTGTTAGGCTTTCTCTCCAAGCCATAGATCAGAATAAGCATTCCAAGAAGAAAGACAGTCGTTGT |
| >SG9639080  CCATAATGAAAAAGAGCCTAAACTGCGCTTTACATATAACCCTAGCACTGGAAAGTGCGACCATTTCTGGGATTTAAGCTGCGATGGACAAATTCTCAACAGTTTTGAGAATTTCACGGAATGTATGACAGCGTGCAACCCCGATTCAAAATGCCTGGCGACCCCTGACAAGCATTTTCAGTTTCCACGGAAAACCTCTTTCGTGTTTGACATAAAT |
| >SG9641062  CGCAGTATTAGTCGCGTCTGCGGTGCAAGTTTGCTTGCATTCGTCCTGGGTGCGAAACCTGTTAGGATTCCCTTTGCATCCTTTATATGTAAAACGTTCGCATGTTTTTCCAGTTTTGTTAAAGAACCACTTCCCGCGAGGCTTCCTTTCATCACAAGTTTCTCCTAATTTTAGCTCGTAATTGCAATCGGCAGCCTTCGGTTCCCTGCGACGAGTTTTCTTCACCGCTGTCCCTGCGTCAAACAGTGACAGGAGAGTAAATATAATCAACGCGCAAACTCTCATGCTTTAAATCTTTTCCGCTTTAGTGTCCTGCCAGCAGTGG |
| >SG9641450  CAGGGCCCTTGCAGTGGCAACTTCATACAGTGGTTCTACGATCCCGAGACAGACAGGTGCCATGAGTTCACATACGGTGGTTGCCAGGGCAATGCCAACCGCTTCAATGATCGTGAGGCCTGCGAGGCGCGGTGCCGCAAGGGCGCACCACTGCCCACTGAGCACCCTGTCGACCATCCCACGCCACTTGATGTCTGTGCACAGCCTAAGCAGCCGGGACCTTGCTACGGTGTGCTGATGATGTGGTACTACGACACCACGACCCGCGAGTGCCGCAACTTCACCTACGGTGGCTGTGAGGGCAACGACAATCGCTTCGAGTCTCGGGAGCTCTGCGAACAGCGCTGCGGCCGTGCTGTACCACCAGTCGTAGTGCCACCTCCTCGGCGTCCCACTAAGCCTAGGCGCATTAGCAAAGGCATTTGCAAGAAAATGGCTGATGCGGGAACCTGCAG |

| >SG4844  CGCCAACAACTATTTATTGTGCTGAAGAGGCTTTCTTATGCACATGGCTAGACATAATTGATGCTATCAGTTTGTATATAATGGAAGGTGGCCGTTATTAGAATCGTCTTGCGTTGGGTGTACCCCTCGGTCTGAGGTTCGCACTTGCTTGTTTTTCCAGTCTTTGACAAATCCTCTTTATACTCCGCCAGGGTTTTCCTGAGCACGCCCGCATGCATTTCTCGCAGGTGCCAAATTTGTTCATATTTCCCCCACAGCCGCCGTAGATAAAAACTCTACATCCACCTTTGGACCAGTTAAAATACCACATTGGAAAGTAGGCTCTGCAGACCCCAGTCTCTTTTGGAAGCCTGCAGACTTTGTTTCGTAGACGTCCTTGTGAGACAGAGAGGCAGAAGAGGAGGAGGAGAAGGTACAACTGCAGTCCCGTAAATGATTTCATGTTTCAGATTTGGCGCAGCGCTGGGCTTTCTTGCGATACCGCTAAAAAATTACAGTTCCACACCAACTGTTACTTGTGAAGGTCTATACGTGACTTCCCTGTTATGGGACTGAATGCGCAGTTATGCAGGGAGCGTCACAAGAGGATAGATCTGTTGGCACAAGTATCGG |
| --- |
| >SG48176  AAATGTCCAGCAGCTATGTATGGCTAAAATCAAGAGTAATTTTTCTTTCTCATATGATCGTTTAGTAAAAAAGCTGTAATCTTCTGCGCGACACATGTCTTGAAATCAATATAGAAGATTATGCGCTCTAAATGTCCAGCAGCTATGTATGGCTAAAATCAAGAGTAATTTTTCTTTCGTTTATGTTTAAAATGCAACAGTGGGCAAGTCAAGGCCGCTATTTTCGGGCGAAATTGGAGCATTTTGACTTGATGGAAAAGAGATCTGCATAAATTAAATGCTTAGAATATAAACTTTTTTATTTTAGAGGATTTCCACAGAAGGCATGCGCCGAAATTACAACCATTGCTTCATCAGTACCCATTGCCCAGTGGGGTTTAATCTTCAAGGCGAAATTCTTCAAATCCAAACTTCTCCCTCAGTCGCTGCAGTGGTTGATGACAAACTTTAGAAGTGAACCAAAGGAAAATGCAACGCCATCGGTTGTGCCGGTGGTTGCGCTCTTCCTCTTAAGCAGACTCTTTCGCATTCTTCTAGGCTTGCAAAGTTATTACCCATCAGAGTACAACCAACATCTTGAAAACATGATTGACTCCCAGGATAATAAAAAAAGACGGGAAAGACGTTTTCAGGTCTATCGCAGTAGCTGGCAGGGCGTGGAGCATAGCACACGGCAGGGTCTTTCGGGCTTATGCTGCTATGCATGGCCAAGCACATTGGTAGAAGAAAGAAGAAGCCTAAAGCTTGGTAGATATTCATGTTGCACATTCGTAAGGGGGGCATGAAGGCCTTCGTTGCCG |
| >SG48291  CTCCCGCTCCTTCTATACCTCTGAAGCTGCAGTATACAAAAATAAAAAAAACGAATAAATAAACAACTCGGGCCACAATTTATTTGTTGCGTATGCTTTCCCAATTTGGTTTTTATAAGTGAACCTTCCATTTCGAATCATCAGGAGATTATTGTCCACGGTATGTATATTCTTGCTTGAGTTACGCTCAATCGTCCAATAACACCAGCAGCGGGAAACTGAAGTTGCCACATGCATTCTGGCATTGCTCCGGGGATTCGAAGTTGTTCCGGCCACCGCTGCAGCCGCCGTACGTAAACTTCTCGCAAAATTGCCCGTTGTACCACCAACGAGAGATAAGAGCTCTACAAGGTCCTTCGACGGGGCCCTCCCTGCATACATCTTCGCACGTTTCGTTCTTCTTATCCCCACTGCATATTTCAGCCCAATCAACTGCGTCCTCTCTGCTGGCTACACCTGCGCCTGGAGATAAATTCCTCGCCAATGGTCCCAAACTTGCATCCAAAGAGTTCGGCGTCACCGCTGCCGCGATTGCTCTGCCGCGACCAGGATCCACATCTCCTAGAGAAAGAAGAAGAGTAAACAGAAGGAACTTCATTTTCAGTCGCTTGTTTAGCCAGGAATTAGGAGTCCAGCATTTTTGTAGGCAGAGATGAGCTTATTTATCTACTCAGCCCCTGACATTAATCCAAGTTCTCCATTCAGACCTAAAACGGCCGGGCCTTTCTCGTTCGGAGTTTAACATTAAGAGCAACA |
| >SG48292  ACTCTTCTTCTTTCTCTAGGAGGTGCGGCCTTCGAAGACGAAGCAGAAAAATGGGCGAAAATATGCAGTCAAGGTCTGAACAACACAGCATGCGCAGAAATATGCAGGGATGACCCGGTCGAAGGACCTTGTAGAGCTCNNNNNNNNNNNNNNNNNNNNNNNNNNNNNNNNNCGTTGGTGGTACAACGGGCAATTTTGCGAAAATTTTACGTACGGCGGCTGCAACGGCAACCTAAACAACTTTGAATCCCGGGATCAATGCCAGAATGCATGTGGCAGGTTCAATTTCACACGGTTGATGTTATTGGGCGATTCATCAGAAGAGAAGGCACAATATTGATACTACGTACAATCATCTTCCGAAGATACGAAATAGAAGGCTCTCGTGTAACAACACAATTGCGAATGCATACTAACAAATAAATATTGGCGGAA |
| >SG48298  CGCAGATGTTTTCTCATGCGACGTGCTCCTGCAAGTACATTGACAGGAAAAGGCTTCAAAATAACCTGCTCTGTACAGGTGAAGTTTATCCCCATTTTTTCTATCTGCAATAGCCATATGCAATCAAAGCACCGCAGTGAGCTTATAAGAGAACGAAGAGGCAAGAACATTTCCCAAGCAAGCTTCCACTTCGCTGAAAAGACATGGAACTGTACCCGGCGTTGGCAGTTTTTCTTTATTTAGGGCTCTGCGCAGCAACAAGCCGATCCACGAGAGATCCAAGATGCATCTCAAACAAACCAATCGTTAATACAAGACGATGCTCAACACCTTCATGGCAGTTCAACTTAATTGAAAAGAAGTGTAATGAGACGTGCAACAAGGACGGCCCGTTCGACAGCAAGCTTGCTTGCGATGGATACTGTCGTAGTGTTGACGTGTGCACGGCTCCACGTGCTGTGTCNNNNNNNNNNNNNNNNNNNNNNNNNNNNNNNNNNNNNNNNNNNNNAGGAATGCCAGGAAACGTGTATGAGACGTCGACCGAAGCCAACAAAACCTTGGAAGTGTTTAGTGTTTCCGACTCAAGGGTACCCTTGTCGATGGGGTTCCGGCTCAGTACGTTTCTACTACAAACCTCGCACCGGCCAGTGCATACCTTTTTGGTACTGGGGCTGCGGAGGGACCGCAAATAATTTCTCGTCCTACCGACACTGCATGAAACATTGTGCAAAGCACTAGTGTCTGCTTGAAGTATTTGATTCTTCCCACTGAAACATGGGAGACATGCCGGAGAGTTAAAGAACTTATCTCATTGGTTACGTCACATCACAGGGTCTTTGGATGACTTCTAAGCAGCAGTTAGCTGTTTATGCAGCGGATTTATTGAAAAATAAAATATTGCTGCACACAAAGCAAAAAAAAAAA |
| >SG48340  TGTCTTTCTTAGTAAATTTTTTAGCACAAATGTTCATGCATTCATAGCAGCTGTCAAATCTGTTCCGAGTTCCTCGACATCCGCCGTAGATAAATGCTTCGCACAAGCCNNNNNTTGTGTTATATGACCACTTGAGGAAGAAAGCTTTGCNNNNNNNNNTTTTCCGCCCAGGTTCTCTGCAGATTGGGTTTGGGGGACGATACCCGCTTACGA |
| >SG48543  AATTGGGACCATGGCCATCTGGAAGGCGGTTCTGTTGCTTGCTCTATTGGCACTTTGTAGTGCCAGGCGGCCAAACCCACACGATTCACGATGCAGATCAAGCCTCCCGGTGCTGAAAAGTAATTGCCCTCACCGCAGCTACGCATTCAACCGTAATACCAAGCAGTGCACATGGACATGCGGCCGAGCTCCTTTTGAAACCCAATTAGAATGCGATGCTACCTGTCGAAGTGTTGCCGTCTGCACTGCTCCGAGACCGTATACCCTTTGCCGCGGGAAGACATTTCCAGTTTTCTATTTTAGTCCTTGGACCGGCATGTGCCACAGCGATATGGGCTGCAGCTACGCAGGAAACAACTTTCCAACCTTAGGCGAATGTCGGCGCACTTGCAAAGCAGGATAATAAGAAATAATCGTTCTTGTCCAGGGGATTCAACTGAAAAGAAGACAAACAGTGCTACAGAGCAGAGAGGAAATGTGAAAATATTTGGCTCCTACGCATTTCTCTTATCCTCGGGAACAAGGAATAAAGTATTGAAAAAGCATCAAAAAAAAAAAAA |
| >SG481728  TCACAGTGAATATTTTAATAGGTCAGAAAAAATTTTATTTGTTGTGTCCACGTTTCCATCATACAAAATATTGACGTCAGCAGCCTCGGGGAATGCGTTTGTGGGGAAAGCAGACTTCCAGGCATTCTTCCTTCGAGTCAAACAAGTTTGGTCCGCCGTTGCAGCCGCCGTATAAGAACTGAAAGCAATTCTTGTGGTAGTAGTCGTAGTACCACTTGAAAGCAAATGCGTAACATGGTCCACGGTTTGGCCAGTGACGACAGTCCTCCGGGGATCTTCGTCGTTCGCAGACCTTGCGGCACTCCGGCATGCTGGGGAAGTTGTTACCGCGATTGTGACAGCCNNNNNNNNNNNNNNNNNNNNNNNNNNNNNNNNNNNNNNNNNNNNNNNNNNNNNNNNNNNNNNNNNNNNNNNNNNNNNNNNNNNNNNNNNNNNNNNNNNNNNNNNNNNGGCTCCTGCACACTTCACTGCAGAGCTGCCTTGAGGAAAAGGAGTTTGCCCGCGCAAGGTGACAGCCAACACCCAGGCGGCATTCCCTGGTGTGTCTGTCGTAGTACCACGTGTGCCTTGACGGCGCGTAGTTGGCACAAGCAGGGTCCTC |
| >SG481732  TGGCAACACGAAAAAAACAACGAGCGCACCAGAAACCACTGTTAAATATAGCAGCTTTAGACTGTGGTCATGGAAATCAAAAGATGCGTTTATTTTCTTTTAATTCTGGCCACCGTGTACGGTACACCACTCAACAATTCGTCCATCTGCTTTCTGCCTCAAGTAAAAGGCAGATGCAGAGGGCTGTTTGATATGTGGCACTATAACTCTACCAATGATAGATGCTCAATGTTCATATATGGAGGATGCGGCGGCAACGAAAACCGGTTTGAGAACTGCACGCTGTGCATGGATTCATGTAGCAACAACGAAAATCGAACAGAAATTTGCCGACAGCTTGAACAAGCAGCTCAAGACGAATACTACGCTGAAATGAACGATTATACAGGCGGTAATGGCTACACCGCTCCGCCACGTGA |
| >SG481772  ATCCACTCGGAGAAGATGAAAATCAACGAAGCTCTCAGAGTCTTTGTTGCGTTTGGCTTGTGGGCAGCCATTGTGCCCGGCCACGCACTTCTCAGAGACGATATCTGCAATCGTCCACGCCCAGTGCCCACCTGTCTGGGGCACGTATTTGAAGTTTACTATTATTCACCAGTGACAGGAAAGTGCCACAGCGATTTGACCTGTACTCACGAAGGCAACAACTTTCCCACGTATCACGAGTGCATGAGAACTTGTGGAAGGATGGCGCCGATGTCGACTGCACCCATGGGGATGACGATGGAAGGGCCCTTTTACGCCTTCTTTTTATGAAAATGAATTCCAATTTCTGGTGTGGTTTTGACCTCATTGGGCGTCGATGGAGCTTTGTCAACGATATGAGGAAACCACCTAAACGACCTCAAAATAAAATGAGTTCTTTTGCAG |
| >SG482011  GTTCAATACGGATTCTGGCAGGGTTTAGTGTCGACTGTTCCTTCTCAGTATGAAGGCCTACTTGTTCCTTGCTTTCATCGGCGCCGCTTGTGCGGCTACAAATGTTGACAAACAGTGCACTGCAAAAGCGGAAAAGGGACTTTGCAAGGCTAAGCTTCCAAGGTGGTGGTTCAATACGGATTCTGGCAAGTGCGAGCTCTTCTACTACGGAGGCTGCGGTGGCAACCAGAACAGATACCTCTACAAGGAGGACTGCGAAAAGACATGCGCTCCGAAAACACTGAACGAAACGCCTCTCACCACATTCAGCAACAAGAAGGCCAACTTCGATGACAAGAAGGGGCGGCTTCCCGGATCCGGCGTAGGCGTATGCATGGAGCCGCCATACACTGGGCCTTGCAAGGCGAGCTTCCTCCGATTCTACTACGACGCCAGCAGCAATACTTGCCGCCAGTTCACCTACGGCGGCTGCCGCAGCAACGGCAACAACTTTAAAGCACAACGTGATTGCATGAGGGCTTGCGGAGGTCGACGGCGCGGAGGTCTTCGGCCGCGCTAAGAAGAGATCGAAGAATAATTCGTCGTGCCATTCTTTACACTATATGTATTTTTGCGAGTAGCGCTAAAGGGAACGGGGAAGAAAAAGAATCTAATGTAACGTAGTGATTCCGTACTTCTTAATTGTTGTTGTTAGCCCATCCTTTGATACGCTAACAACAACAACATCTTAATTATAAGATTGCGCTGCTGCTTTGCTGCTTTTGCTTCCGTTAATAAAGATAATCTTTCAAAAAAAA |
| >SG482330  GGTTTAGTGATCGTTTCTTTCGGTATTTCAGGTTATTCGAACATTGAACCCTATGAATACTTGCCGGGGTATAAAATTTTTTTCGTTAGATCGAGAACTTCACAAAATCGGCTTTCGTTAAATCGAGTTTTAACTGGTGCCGTAGTCACTGATGATGAGGCTGGCTGGACCTTTCGCACCTATCATTATAGGGGGAAGGAGGGTATTGTAAGAGAGTTAAAAAATTTACTTTGAGGAGCTGCTACCCTCACACGATGCCAGCTCACGTGTGACATAGCAATTCACTTCTTGAATGCGGAGGTTTTTGATATGTGTTCGTTTGCCCGGTATGTCACCAGCACTTTCAAGCATTCCTAATTATAGGGTGTTTCAAACACAGAGGAAAACTGTTCAGGAACACACAATCGGCCGCAGTGGAGGTGAGTGACTTTACAACTATAGTAATCAAATCGCTACTTTAATGAGATTTAATAAATTATTTTGGAATGAACAGAGTCTGGTCGTTAGCATAACAAAAATGTAACATGAAAACAGACCAATGCTGGCAAGATAATCCTAGGAACCATAGACACGGTACTTCGAACTGCTTTTGCTAATACACCACGGAAAGTGCTCTTAAGCTTCGTCGCAATGTGCAAGAAAATGAATACTCATCGTAAACTACGGGGCTGCCAGTAGTGAGAGCTAATTATTTAGCAAGAAAAAAGCATATTTTCAAAATAATTCAGAACTGCCGCGGATAGCAGTCTCTTTTGAGACCACCCTATTTTGAGCAATTTTGGAAGGGCTCGCCGAAAAACGCTGTTAAATATAAGCTTCATTGGACCACGGCATGCACTTCATTTTGCTTCGCCCCTCTAAAAAATAACAGATACGCGGGTGGCTACGACTATAATTTTTTTGGACCAAGTGGCAAAGCAAACAAAAGTGTATGCCGTGGTGCAAAATGTGCGGGCAGTTAGAATTGGGCGCCGTACACTCTAAACGCAGAAAGAATAAAACAGGTGTACGCTTTCCTCTAGCGCACTTTTTTTGTGAAAGGGAGTGCACTTTAGGACAGATTACTTCATTTTCACTGCAATAGCGGTGTATTTGTGTTGAGACTGTGGGCCCTGCACCGTGTCACCAGCGGTCGTTGTGTTTGCACTGGGCTCCCTGCACACCTCATGCACTCAGTTTCGGTATTGTGCGAAAAAGAGCCTCGCATTTACTCGCACTGCACGGTTTTACAGAACATTTTCAGAAACTGATAAGGCATCGTCTTTCCCTTCGCTTCCAGCTCTCAATTAGCACTAGTCACCACACCGTTTTAACCAAAACGCTAATCTTAAAGCGTACTTTGTCATATGCCTTACTAGTGCGAATCGTTTAGTAACTGACGTCCGCCGAGGGTGCGAATGTATTTATACGGAGATTTTTCCTCTTTAACCATAAGATTAAGAATTTTTGAAAGTGCTTACTGAAACCACAGAGATAATTTCTTCGTTTTGTGTCACTAAAAGTAAAAGGCATTGAATGGCCTGGTAGAACACCACGAATGCGCCAGACACACTATTGTCTGGGACAGAATTACAATGATGGCTATCGAAAAGCCATTCTTATCATGTCTGCACCTGATGTCGTTTCATATCCACACAACCAATCGCGCACAGACACGTGAGCCCTTGCCCGAAACATACTCATGATCACTGTGGCGCTGATCGCGCATATCAAGGCGGCTGTCCAGTCATTCCTTTTACATGTGAACATTGCGCTTGTAAGGGTCTCGAAAAGCCCCCGCCTTTGTATTTTACCCTTCTGTTTGGCACTCGTCGATTTCTCCTCCTGCAATGTCTTTTCCTGGCCAAACGGGCTGTCGAACCTTGGACTAGTAGGCGATAGTATGCGCGCTTGGCGAACGGCTGTCGTCGACCGACATTCCCCGTGCTAGGGTAATGACAGCAACCAGGCATTCACGTCACCGATTTGCTTGTGCGACCTAAAAACCTCGTGTTGCTCCTTCCCCGTGCCACCATGGAACCTCGGTTGTTCCAAATTCTTTTCGCGCGCACTATGCCTAACGCCACTGCGTCCCTATCGTTAGTTGCGGCGCATGTGCCAAGACTACGAGCGGCACCGCACACAATCGCGCACATCCTTACAATTGCTGTGCCGCGCTGCCCGTCGGCTCGGCTCTATCTGTGGCAGGCTCACGCTAAGTGGAGGAGCGCGTCGAGGCAGATGCGCCGTGGTTGAACTTGCCGATAGTCTCCACGCCGCCAGAGGCATTTGTTGCGTTAGAAAATTTTGTGCAGAGGGCAAGATTGCCTGGCTGCAGTTATCCAGTGTTGCTGAAAAAGACGCTGCTTGTAAGCAAATTCCGTTGACTGCCACAAAGACAATGCATATTTTAGAGATTGGGCGCACCGATGGAATGCGACAGAAAGTTCATATCGCCAGGGACACAAATTGCATTCGTGGACGTCCTTGGCAGTTACTGCCAGTGCGCGCAGATTTCACTACACACCGCGTGCGCCTGTGATCACGCATGCGCTAATCGAGAATCTGGGAACAAAAAAGAAATATTTCCGTCCTCCTCGTCGCTTCTTCAGAGAGTGCAGAGCGACGCTGTCACCGATGCACCAGTGAGGAACACTAGAGCGTAGAGGGCGGCCCCGTTGCTACGCGGGGCTGCGTTCCCGCACTCGCATGACCCGCACTTGGCCTCCTGCTCATCACCGCAGCTGCACTGCATGGACTCCGGGCAGCTCGCAGCCGTCCTGGCCAAGTTGATGCGCACACCAAAGCAGGTCATGTTGCATCCGTCTACGGTACGGAAGTTGTTGTCGTTTCCACCACATCCCTGGTAGTTGAACCGCTCGCACAGTTCTTTTTGCACGTCGTAGTAGAAGCGGATCTCTGGTGTAGTGGCCCCTGGGCCACTGCTGTCGGTGTCGCAGTGGCCTTCGTCCACCGGCTGGTGACACATTTCCCTGTCCTTTCCGGCTACGCCGTCCTCAGCAACATGCACCCTGTCCTTTCCGGCTGCGCCGTCCTCAGCAACATGCAATCGGCAGCGTCCGTTGCACTGGGTGCTGGTGTAAAAGTTGTTTCCGTTGCCCATGCAGCCACCGAAGATGAAAGGCAGGCAGCGATCCTCCTGCTTATTGTAGTAGAAGCGCCGGAAGTACGCGTAGCATGGGCCCGGGTCTTGGGGCTGCGAGCAAACATCTTCGCAAAGCGTCTTGCACGCGTCGAGGTCTGCAAAGTTGTTCTTGTTCCCTTCGCAGCCGCCGTACTCGAAGGTCTCACACAATCCCGTCTGTGAGTTGAAGTAGTAGCGCTCCTGCGTGTCGTTGCAGCTGCCAGGATCCATTGGCAGGCGGCAGGGATCTTCACACTGGGCAATGCACTCTTCATGGGAGGCGAAGCTGTTCTCGTTCCCACCACAGCCGGTGTAGTAGAACACGTCGCAAACGCCGGAAGTGACGTTGAAGAAGTAGCGGGGAATCCTTTCATTGCAGTTGCCTTCTTTCACCGGCAACTTGCACGCACCATCAGACGGACAGTGACAGCTCTGGGCGCCGCTGCTCGAAATTCGCTTCACGCAAGTCACTGGGCAGGGCACTGCGATTTGGGCATCCACGTACTCCAGTGCATATTCAACAGTGCCGTCTGCAGCAGCACATTCGCAGGCGTAGCACGACGCAGACACCTCCCTACTCTGACAACCAGCCGGGCACAGCTCGGGAGCCGCGAGGCAGTCAGCTTTCTTGAGAAGGGGCTCTGTTTCGGACGCGAGGCAATTGCACGCAATACAGCTGTCTTTCTCTCGCCGCTGGCAGCAGTGAGCGCAGGCTAGGCCGCATGCTGCACGCTGGGGACACTGGGCTGATTCGGATTCCACAGGTGGAGCTCGAGCTTGCTCCAGTGGCTTCTCGCATCTTTCAACACATTCTTGGCGAGTAAGGAAGTGGTTTTCGTTGGCTCCACAACCGGTGAAAGTGAAGTTCAGGCAGCTGCGTTGCTCTCGGTCGTAGTAGAAGACTGTCTGCTTATTCGCACAGAAGCCTTCCTTTCGTTCCAGGAAGCATATTCCCGTGCCTGGTGGTATTTCGGCCAAGGTGGGATCTTCATCNNNNNNNNNNNNNNNNNNNNNGACGGTAGAGTGCATGCTTCGTCAAACTCGTGCGGTTCCAGCTGTGCGACAGCAACGCAGACGTTCTCCAAGTGGCGCGTGATCTCCAGAGAGGTGTCTCGTTCGAGCACGTGCCTCGCAAGGACGCTGACGTGTGTCGCATCCGGGGCACACACCGACGCGTGGGGCTGCTCATGCGCGCACCGGTGCAGCACGGCGTAGCTCCCGTAGTCCGTGGCTACCACGGCCAGCGTCTCTTGTACAGGCTGCCCAAAAAAGTCGTACTCGAGCTGAAATTGTCCGACGGGCAGCACTTTGTGGACTTTGCCGACAATGGCCGGCAGGCATGTCTCATTGCCTGCAAGTGCTGCGCTAAAGGCGAACGTGTTGGTGTCGAAGTCAAAGCGCCAAGAGCCTTTCTGCAGGAGAACCGGAGCGTGCTTGCTCTTCAACACCTCTCGCCAGTCTCCCGAGAACTTCTTGTAATCGAGGTCCTCGAGGGCGCTGGCCGGCTTGGGGACACACACCAGGGCCTCTGCCGGTGTCGTCGGCGCGGGCAGTATTCCAAGGCCCGTCAGCGAGGTGACAAGGAACCACGACAAAGCAGCCGCAGAGGCCATGGCGTCTCAGCCGCTGCCTG |
| >SG482390  CACTATAACTCTACCAATGATATATGCTCACCCTTCACATATGGAGGATGCGACGGCAACCAAAACCGGTTTGATAACTGCACGCACTGCATGGAGTCATGTAGCACAAACGAAAATCGAACACAAATTTGCCAAAAGCTCGAACAAGAGGCTGACGAAGAGTACTATTCGGGATGGGACGATAATACAGGCGGTGGTGGCTACACCGCTCCGCCACGTGAGAATTACTATGAGTACGACGACGAAGAGTAAACATT |
| >SG482486  AAGAAGCGCTTACGGGGGACACGTATTGTGACACCTTCATAGTACAGTAGCAGCGCAGATAACTTATCCAGCAGATTTCGAAAAAAAGAAGGCTGGACGAAAAGGTAACGGGTTAGTGGCATAGCATTAAAGCCTAGTTTTCCGAACATTTCAGATATGAGCGCCACGATCAGAGTACAAGCGTGCCTCTTTCTGCTTCTCTTTTGCCTGGCTTTCACAGAGGCGGCTCGTTCGAACAAACGGATATGCAAGTTTCCAAAAGAAAAAGGACGCTGCAGAGCATCTATTCCATCTTGGTGGTTCAACATTCGCAAAGGACGCTGTGAAGAATTTCTGTACGGAGGATGCGGAGGAAATAAGAATAGATTTGACAGTTGCCCTCTGTGCTTGAACGCGTGCCGAAAAGGAGGAATATCAGGGGACAGGACAAGGCTTTGCATCCAGCTCCGTAGAAACACTGGGAGAACTCGCTGACTGTAGCACTCTCTGTGAAGAGCGGAAAACCAGGCGTAATGGGGACTGCAAGGCCTGTTCGCAAGATATGCCTTCGTTCTGTCATGATGCGAAGCAACTGTGACAGATAAATAAAGTATTTTGGGCACGAAAA |
| >SG482813  GCTTGCAGCGACCGTATTTCTGGTGTCGTTAACAGGATGCGTGTGTTTCGAGGAAGACAAAGCCTACATGCAGTGCACGGAGTATCCCGATCGGGGACCCTGCAAAGGCACAGTGTACCGCTACTACTTCAACTTCATGCGAGGGACTTGCCGCCTCTTCGTCTACGGCGGATGCGAAGGCAACGACAACAACTTCAAAAGCCGCAAGCAGTGCCTGCGACGATGCGCAAGCGTTATTACGTCGAGAATCTGCAAGCTGCCCCCTAATCGTGGCTACGGCCCTTCGCGCGTGTGGCATTACTACTTCGACTCGAAGAAGCAGTCGTGCCGCCCGTTCGTCTTCCTGGGCTTCGGAGGGAACCGCAACAACTTCATATCCAGCGACGAGTGCCGCATGCAGTGCCTGGGCAAGGAGGCCCACGAGAGGGGGGAAGACTGATGCGAACAGGAACACGCACGCGAAGCCAGCAACGCTACACTACGCTTTGGAAGTCGACTCTGGACGCTTGGACTGTGAATGCCTGGG |
| >SG483340  CACATGTACACCTGCACGACTTGAACACAAAAGAAGTGGCCAACGGTTGCTGTTTGGCTTCAGTTACCTCAGTGCCGCAGCTTGCAATATGAGACCACAAGCTTTCATAGGTGCTTTCGTCTTTACTTTGGTACTCAGGCAAGCTGCAGGTATAAAGTGGTCGCGTTGTTTCCGACCGAAGGCTGTGGGGAACTGTCAAAATAAGGTTCCGGCCTGGTACTATGATTTTTGGAGCTTCCGTTGCAAGGGGTTCCTCTACAG |
| >SG483341  AGGTATTTCGTAAAAATGCCATAGAAATAAACAGAGCTTGCTTTTTTCGTGTTGATGTCAAATATATTTTCTGATACAAGTCAAAATTGTGAGGAAGATTCGAACGCTTTATTTTCCCTCAACAGACGTAATTTTTTTTCCTTGACACCACGCTTCGTATAAAAACTCCCTCATTTCCCTATGAATGGAATCCTTAGTGACAGCTGGGGCCACGACGGTTTTTTGTGATGCTGGTTTGATCCCAGGTTGTTTTCTTCGAGAAATATTTTAGTTTGCTTCTTGCATATTTTTCTTGCGTTGTTATTTCCGCTGCATCGTTTCATGCACTTGAGACAAGTTTCAAACCTATTGGCGTTTCCTTTGCAACCACCGTAAATGAGGCCACGACATTCGTCCAATTCGGGGTCGTAGTACCATAATGGAATAGCGGCCTTGCATTTACCAGTTTTCGGCTTGAGGCTACATACCTCCTTGCGCTTAGATTTGCGTAAGCAGGATTTCTGACATTCTTCTTCGGTTGGAAACCTGTTCGAATTCCCGCCACATCCGCTGTAGAGGAACC |
| >SG483537  TTATTCCATCTGTGTGAGGATTTTCAGAAATTCAGAGAGAAAAATTTGCTTCCAGCGGGTGGTAAAAACTAGATGACGCTGGTTACTTGGCTCTTCTTGCATTGCTTGGCTTGTTAGGATTCCAATTTCCACCATTCTGCTTCTCCAGCTTCTTGCAAACGAACTTTGCTTTTCGCAGGTTCAGTCCTCCACATTTCCTCATGCAATCCTCGCAGTGTTCAAAATTATTCGCATTTCCTTGGCAGCCGCCGTAGATAAACAAAAAACAGGAATTTTTATTTTTGTTATACCACCACTTGGGCATCGATGCTTTGCAGGGCCCAACGTCTTTCGGAAGGGAGCAGACTTTTTTTCTGTTGAAACGGGCATGTGAAAGAGCCAGGCCGAG |
| >SG483538  AGATAGTTTATTCGATCTGTGTGAATTTTCAAAAATGCAGAGAGAAGAATATCTTTCCAGCAGGCAGTAAAATCTAGGTGACGCTGGTTATTCGGCTCTTCTTGCATTGATTGGCTTGTGCAAATACCTATTTCCACTGTTTTGCTTCTGCAGCCTTTTGCAAGCGATCTTTGCTTTTCGCCATCTCAGTCCGCCACATTTCGTCATGCAGTCCTCGCAGTGATCAAAACTGTTATCATTTCCCTGGCAGCCGCCGTAGATAAAGAAAACACAGGAACCTGTGTTGATGTTATACCACCATTTGGGCATGGATGCTTTGCAGGGCCCAACGACTTTTGGAAGGGAGCAGACTTTTTTTCTGTTGAAACGGGCCTGTGAAAGAGCCAGGCCGAGGAGGAGCAGAAAGAGGCACGCCGGTAACCTGGCCATCGTAGTCATTTTTCGAATCTTTGCCAAAAATGCGGCTTTGTCGAGATGAGCGGGATGCAATACTTTGCTCCCTGCCTTT |
| >SG483576  GCAGTGTAAAGAAGCGAAATTGGACTGCGAGTGTCTAACATGAATATCTACCAAGTTTTAGGCTTCTTCTTTCTTCTGCCAGTGTGCATGGCCGTGCATCACAGCAGCAGCTTGAAAGACCCAGCCGTGTGCACTGCCCCACGCCCTGCCAGCTATTGCTATGGTGGAGCCTTTGAGGTCTTTTATTTTGAACCCTTGAGTAACTCATGTTTAAAAGAATTAGGTTGTACTCTGCACGGCAATAACTTTAAAAGCAGAGAAGAATGTGAAAGACTCTGCTTAAGAGGAACCGCGCAACCACAGCCACGCCCGATGGCCTTATTTTATCCTTGGGTTTGGCTCTAAGGTTTATCATCACCCAGTGCAGCGACAAAGGGAGCACTCTTGATTTGAAGAATTTCACCTTGAAGAATAAAACCCACCGGGCAATGGGCATTGATGAATCAGTGGTTATACTTTTGGTGCACGTCTTCTGTGGAAATCCTCAAAAATAAAAAAAGGCTAACATTATAAAAATTCCACTGACGCAGATATCTTTTAAATCGAGTCAAAATGCACCAATCTGGGTCGAGAACAGCGGGCTCTGCCATGGTTGCAGTTTAAATGTAAAGGAGAGAAACTTACCACTTCATTTTAGCCATATATAGCTGCTCAAAATTTGCCTGACAAAAAATTTTAGTAACATTATTTAGAAAGTCTTTAGACAAGTGTTAATAGAATACATTGACGTTGAATTAAAA |
| >SG484335  CCTTTGCAGCCACCGTAAATGAAACCACGACAGCAATCGTGTTCCGGGTCATAGAACCACGATGGAATAAATGATTCACAGTTGCCTACTATTGGTTTCAGGCTGCAGAATTCAGCAGCACTAGCTCGGCCTATGCACCACCTTTGGCATTCGACTTCAGACGAAAATCTGTTGGGATTCCCGCCACAACCGCTGTAGAGAAAGCCTTTACATTTCCCGGACCAAAAGTCATAATACCACGACGGAATCTTCTTCCCGCAGCTTCCAACAGCCTTTCCTCGCCAACAACGTGGCCATATGCGGAAACTTGCGGCGTGCACAAAAGCGAAGTAAACCAAACATGCTGAGTATATAGCTTGAGCTCTCATTTTACCGACGGTAATATGGGGCAGTCCGAAGGGCGGCTCGAAGCCATTCTTAG |
| >SG484535  AAACGTGCGAAGATGTATGCAGCGAGCACCCAGTATTAAGATCTTATTGTAGAGCTCTTATCCCTCGCTGGTGGTACAACGGACAATGGTGCGAAGAATTTTACTACGGCGGCTGTTACGGTGGCCGAAACAACTTCGAATCACGGGAACAATGCCAGAAGGCATGTGGCAAGTTCAAATTCCCGCAGCTGGTGTTATTAGACGATTGAGCGTAACTCAAGCAAGAAAATACATACCATGGACAATAATCTCCTGATGACTGGAAATGGAAGGTTCACTTATAACAACCAAATTGGGAAAGCATACGCAACGAATAAATTGCGGCCTGAGTTGTTTATTAATTTCTTTTTTTATACAGCAGCTTCATTCAGAGGTATAGCAGGAGTGGCAGGTGTGCACAAAAAAAATTGGTGGAGACGCTTAAGCTCCACCTTGAAGCTGTGACGCGATTGCGTAATGAGTTGATTGTCATATATGTAGAATAGAC |
| >SG485205  CCGCTGCAGCCACGATTGACCCAGCGCTCTGAGGCTGGGCGGCCGAGCCCCCTAACCACATAGATACCGGGGCTGGTGGAAAGCCGCTGCAGCCACGATTGACCCAGCGCTCTGAGGCTGGGCGGCCGAGCCCCCTAACCACATAGATACCGGGGCTGGTGGAAAGCCGCTGCAGCCACGATTGACCCAGCGCTCTGAGGCTGGGCGGCCGAGCCTCCTAACCACATAGCTACCGGGGCTGGTGGAAAGGTGTCACGGCGTTCGCAAATAGGCCACAGGGTTCCATGTTTGGATTCTGAGACGACTGGAGTTAATTACGCTGGATACCTGCAAAAAATATTTTAATTGCTGCGATAGCATTACAAGGCCCTGCGCAAGCGAATATGGTCACCGCTGTCTCCGCTGCAGCCACGATTGACCCNNNNNNNNNNNNNNNNNNNNNNNNNNNNNNNNNNNNNNNNNATGCCTTAAAGAACCCGACATCGATCTGTGCACTAGCTATGAAGCAAAGTGGTTTTTTGATGTCGAAAAAGGGCACTGCAGACCATTCCAGTACGGAGGATGCCAAAGAAACGAAAACGTTTATCCTGACTGCACTAACTGCATGCGTCGATGTAGTGG |
| >SG485391  GATAGTTTCTCTTTATTTCTCTGAAAAATTACAACAGCCGTTCTCTCATGTCTTGCAGGCTTTGTGTTCGCCCAATGCATGTGCGGATAGAGCAGGGCAAAAGAGAGAGTGAAGTCTTCATTTCATTTGTCCAGCGGGCTACGTAAGCTTGCAGGCAATCTCGCACTCTTCCTTCGACTCGTAGTTGTTGTCGTTTCCACCGCAGCCACTGTAGAGGAACGCCTCGCACTCGCCTGAGGTCACGTTGAAGAACCAGTGGTCTAAGAAGCCCTTGCAAAAGCCGCGTTGCGGTTTTGGCGCGCAACCAGTCTCGAAGTCAGCTCCCTCGAAGTCACTGGACCTCTCTGGCTCGCCACATGTTTTTTCGCATTCCTCGATAGTTTCGAAGTTGTTTTCGTTTCCTTCACAACCACCGTAGATAAAGAAGGAGCATTTTCCTTCAGCTGGGTTGAAGTAGAACCGCGGAATGCTTGCCCGGCAGA |
| >SG485917  ATCGAAGCTTACCTTATAGAAGACAGGCACAATGAGTTCTGCCGCCTTTATTATTTTTTTGGCGTTTGTCACAATGCTGGTGGACACGCGTGGTGCGCCGGACGGCCCCCCTGCAGACTGTATGTTACAAAAGTTAGTGGGAAACTGCAGGGCGTCCATTCCACGGTTCTATTACGACTCGCAATCTCGCTCCTGCAAGGAATTTATATGGGGTGGATGTGAAAAGAACGCCAACAACTTTGAGACGTTAAAGGAGTGTCTGGACAAATGCGTTAATCACAATTCAAGAGGATAGTAAGCCATAAAGCAAGTGAATAGCGTTCTTAATTGAAGCATGTGCACACCAATCGTGCTGTGATTCAAACGAATGCCGTCCAACGTTTCGAAGAGAGCTTGAAATAA |
| >SG486805  CTTATATAGGCCCTGAAACTGCAAACATCATGCATTTGTAAATGTGAATTTTGTGTATAGACCCAAACCTGCCAACAACATGGAGCTGAATGTACTGTGTTAATACGGATTTTCTATTGTATTGTCCACGACATGATTGAGTATTGAGTCAGGTGACTAGGAATTCAGCGAGCAAATTCGCAAATAAAAGGCTGGAAGGAGTGAATTGGACCACTCGCATATCAACAAAGGCGCCTTCTGAGCAAAGCTTCAAAAAATGACCGCAATGACCAGATTACCAGCGTGGCTGCTTCTGCTTCTCGTCGGCGTAGCTTTTTCACATGNNNNNNNNNNNNNNNNNGTCGGCGTAGCTTTTTCACATGCCTGGTTTAACAGAAAAAAAGTCTGCACCCAGCGAAAAGAAGTTGGACCCTGCAGAGCATCGATACCTATGTGGTGGTATAACGCAAAAAGACAATACTGCAATTTGTTTATCTACGGTGGCTGTCAAGGAAATCAGAATAAATTTCAACACTGCGACGAGTGTATGAAGAAATGCGGTG |
| >SG488036  CTGGCCGAAATGCAGCTCCTCACGATTTTTGCTCTCTTTTGTCTGTTGGGAACCACGCTGTCAGCACGCGTCCAAGATAGACGTTGCAGATGGCGTCCAAAAAGTGCTACGGGCATGTGCCATAATGGAGAAGTGCCTAAACTGCGCTTTGCATATAACCCTAGCACTGGAAAGTGCGACCATTTCCGGGATTTAAGCTGCGGTAGACAAATTCTCAACAGTTTTGAGAATTTCACGGAATGTATGACAGCGTGCAACCCCAATTCACCATGCCTGAAGACTCCTATCAACCACAGAGGGTGGATTCCACGGAAAACCTCTTTCGTGTTCGACATCAATACATTGAACTGTACTGAGAAGAAATCGTTCCGAACACCGGACATCGGCCTTCAATATAACAGGTTCTTTGACAAGAACGAATGCAAAGCGAAATGCGAGCCCGATCTTGAACAAATCATTACAAATTCAGGTTAAGTAACCGAAATAAAATTTTCTTTCAAGGCAAA |
| >SG488561  TCCGATCTCCCATTGTCAAAGTTGATTTTAATCATTTAGAAGCAGGAAGCTGCTTAGAACACACTGTGTTGCTGTATTTTTTATCCACTTTGCATTTCCATCCAACCGGAAAACTTCCATTGTTCTTTTCGTCTTTGCAGCGTGGAAGTTCAGAATTCGTTGCATTGCATATCTTCACGCAGAAATGCATACCGGGAAAGTATAGATCGCCTTTCTTTACATCCACCGGCTGGCATTGTTGCGAGGTAGAATTATAATAGAAACGGCGAATGTTTCCACTGTCTGTCTTTGCGTCAAACGTCATGTTGCAGTACTTTAAGCGATCCAGAATCGGTTTTGAAGCAGGATGGTTTAAGTTGACGCCCTTCCTGCAATGAGAGATGCAGTCATCAACCGACGGGAAGTTATTTCTGTTACCGTCACATCCCTTAAAGCCGAGGAATTTGCACGTGTCGGAAACGTTGTCGTAGTAGTAGTCGCGCCGATACCACTCTTCGCCATATTGCCTCCAGTACGAATATTTTGTGTTGGCGTTGGCGCTATTCTTGCTTTGACATTGCGGGGCTTCATTGCCAACTGTTCCACCTCTTAAAACCGCAACCGCTGATGCCAGCAACACAGCCATGGCATAACGCTTCATGACGGCACTTGCGTCCTATATCACCAGAAAAAACTTTCGTTAAACTTTATGAAAGCAAGCTCAGCCTTTGTGTGAGCCAGTGTTTTATAGGCTTGGCGCTG |
| >SG489513  TGCGTAACGAGGCATGTCTTACAATTTCCGGAAGGCATGCGTAGAGGAACCTACTCTCGTCCTCCCAGGATGGGGCGGAGAACATCGCCATATTTTTTGATCACTTCAACAGTCAGTTTCTCGCAGATTCTCTGTGCATTTTTGGGTTTCGTTCCGCTGCATCTGTTCATGCAGTCGAGACAGGTTTTAAACCTGTTGTCGTTTTTATTGCAGTCGCCGTGCAGAAAAAAGCGGCAGCCGCCAGCTTGCCAGTCAAATGACCAAGTATGAACTCCCGTCTTGCAAGTTCTTTTTGGTGGGTGAAGGCTACAAACTGGTTTCGTGGGAACTCCAGGCAAGCAGGTTTTCAGGCATCCGACCTCGTATTGAAACCTGTTGTTATTACCTTCACATCCGCCATATATAAATAGTTTGCACTTGTTTGCTGACGGATCATAATACCATCTCGGGACCTTCGCTTTGCAGCGTCCAACTGCAGGCGGCTTCAAACAACGTCGTGGTCTCGCATAACTAGTCGCGTGCGCGGACACCACAAACCCCATAAGAATAAGGAAATTAGCTGTA |
| >SG489514  AGTTGGCCCCTGCAAAGCAAAGGTCCCGAGTTGGTATTATGATCCGTCAACAAACAACTGCAAAGCATTTGTATACGGCGGATGCAAAGGTAATAAGAACAGGTTTAAATACGAGGCCGGATGCCAAAAAACCTGCTTGCCTGGAGTTCCAGTGATGCCAGTTTGTAGCCTTAGGCCACCAAAACAAACTTGCCAGAAGGGAGGTTATACATGGGCATATGACTTCGGAGCTGGCCTCTGTCGCTTTTTTCTGCACGGCGACTGCAATCGAAACGCCAACAGGTTTTCAACCTGTCTCCAGTGCATGGGAAGATGCAGCGGAACGACACCTGAAAATGCACGGAGGCTCTGCAAGAAACTGACTACTGAAGTGATCGAAAAATATGGCAAGGCTCTCCGCCCCATCGTTGGAAGACAAGAGTAGGTTTGTCTATGCACGCATTCCGGAAATGATAAAGCGATGTTTTG |
| >SG4810223  CATGGCTGGTGATCAGCCTTTATTCAGAATTAATATTTTTAGTCTAGCGACGTTGCATAATTTCCTGTTACTTTTGTTTTCTTTTTCCTTTGTTTGTTTCCTTCTTTCCCTTCCGTTGTGGTCTTTCGTTTTTATTTTGTTATTTATTTCGACTCGGTTCAAGTTGCCTTTGATACTCCCCGTAGTTGAAGGTAGGAGGATCACATACGTCTTGACATGTTTGGTTGTCCGGGAACTTATTGTTATTTGAGTCACATCCACTGTAGTTGAAAGTAAGGCAATGACGAACTGTCTTATTGTAGTACCACATGGGAACTGCATCCGTGTTTCCAACGCAATCCTTTTTGTCGGCTGGGGACGTACCACATAGATCGTCATTTGGATCGTCACTAAAAATCACCTTGCCCATTCCTACAGAATCTCTTAAGGAAACAAAAAGAAAAACAGTCCAGAAGACCACGTAAACGTGCTTCATCAACATGTTACCTGCACGGAGAACTTCCTCGTGCCAGCAGTGGGTTCAACAGCGCAG |
| >SG4810491  GAAATTGTACAGCTAAGATTCCTTCTTGGTACTACGATTTGTGGACCTTGAGTTGCAAAGGGTTCCTCTACAGCGGATGTGGTGAGAACTCGAACAGGTTTACATCTGAACAAGAATGCCAGAAAGCATGCACACGTAAATCTAAGCGAAAACTAGTTTGCAGTCTGAAGCCGAAAAGTGGAAATTGCACGGGATTTTCTCCGTCATGGTACTACG |
| >SG4810580  TCTTGCGACGCTCCCAGAATAGTTGCGCATTCAGTCCCATAACAGGGAATTCACCTATAGACTACCGCAAGTAACAGGCAGCGTTGAACTGTCAATTTTTAGCGGTATCGCAAGAAAGCCCAGGGCAGCGCCAAATCTGAAAGATGAAATCAATTCCGCGATTGCAGCTGTACCTTCTTATCCTTCTCTTCTGCCTCTCTGTCTCCCAAGGACGTGCCCGAAACAAAGTCTGCGGGCTTCAAAAAGAGTCTGGAGTGTGCAGAGCCTACTTTCCAATGTGGTATTTTAACCGGATCAAAGGAGAATGTAGGGTTTTTATTTACGGCGGCTGTGGGGGAAATGGCAACAAATTTGACACCTGCGAGCAATGCATGCGGGTCTGCACAGGAAAACCCCGGCAGAGAATCAAGAGGATTTGTCGAAAACTGGAGAAACTAGCGAGTGCGAACCTCAGACCGTGGGGTGCGCCCAACGCAAGACGATTCTAATAACGGCCACCTTCTAACATTTATAAACTGCTAGCATCAATTATGTCTAGCAATAGCATAAGAAAGCCTCTCCAGCACAATAAATGGTTGTCGG |
| >SG4813074  AAATCGCATAACTTTTATTTGCATATTCTTTTAGCAGACGCAAAGGGCTTCTTGTGATTCCGATGTGGTTACATTATGTGGGCTTTGTGGGAGCTCGCAAAATGCACGTCCGAAGGCACTCTTTCTCAGTTTTAAAGTTGGCGCCTGCTCGGTTGCATCCATAAGAGCGCTTTTTGCAGGTACCCGTGGTTTGGTCGTAGAAGTACGTGGTAAGTTTTTTTTCACAGACCTCGCTGTCAAGTGGAGGGCTTATGCACTGCTGTCCTTTGCAAGTCACTTGACATTCTCGCAATGAAGAAAATCTGTTGCCGTTTCCATCGCATCCTTTGTACAAAAACAATATGCAATTGCAACTTTTGTTGTCATAATAGTACATCAACTGAGGGGTTCCACATTCGCTTCCGGCTTTCATCGGCATTAAGCAGTCCGTCCCTGCTCGACAGCTCCAGTTGCACATTTCCTCTGTAGAGAACAGATAGACACCATAATGAGCAGTCCGTGTCGTTTTGATACATTCATGTTGTGACTCATTATAATAGTAGCCGGTGATGAGGTTGGACCATTGTTTCCAGTTCGAATTGCATTTATCTTCTGCTGCAGACCCCACAGTGGCGAAGAACCCTGTTAGAGCAATCACGAACCCCGAAAACGCAGTCATTATGCTTTTCCAACTGGTCACGTCTGTGAAATGCTTTAAAATCTTGCTATGAAGGTGC |
| >SG4814548  CGGACAGATATCCAGCCACCTCCAGCCTATCTAACCACCACTATGCGCAGCCTTACTTTCTTGGTGTTGCTGATTGTATTTTTGTGCGCCGTGCTCCTCGTAGACGGCGAGAAGAAGAAACCTAAACCGGTATGCAGCCTTCCACCCGAACACAAGAAGTGTGCAAGGCTTAAAAGAAAAGGATGTTACTGCCCGAAGAATTTAGGTATTCCCGGCTTCGTACGCGAAGAGCGCTGGTTCTACAACAAAAAGAAGAAACAATGCGAATCGTTTGCGTGGTCGACTAATGGTGGAAACTGCAACAATTTCCCATCAAGAGAGAAATGCTACGAAACGTGTTCAAATTTCTAATGACGAAAT |
| >SG4815151  CGCTTATTTATTTCCCTGGGGCGTCGTCTTGCGTTAATTCCGGATAGAAATACGTTGTTTGAATTTGCTCTGGTTTCCAAAGAATGCGCTTATTTAGGTAATCATGTACTACCTGGATTTAGCTTGAGTTTCTTCTCCTCTTTTAGCGTAAGATCATGACAAAGCTTCTTAGTCTTCTTATCACGTTTTCCCATGCACTTAAACATACATTCGGCGCAGGAGTCAAATTTGTTTGCATTTCCCCCACAGCCGCCATATATGAACATCCTGCACCCTCCAGATCCGGAATAATACCATTTTGGGATGGAGGCTCTGCATCTTCCTGGATCCTTTTGAAAGCTGCAATATTTCTTCGGAGATCGTGCATTAGACAGGGCCACTAAGAAGAGCAGCAGAAGAAGGCAGGATTGTGCTTTCATAATCGCGCGCATATCTTGATGATTTGGTGCTAACACGATATATCTGAGCTGCAAATGGCCAGAATATTTGAGTGCACGCTTTTATTGGCGAGCGTTTCTG |
| >SG4816232  TTTTTTTCTTCGATAGAACTGTTTTTATTGTTTAGCTGCAACTGTTTTGTTGCATGTTGTGTAACGAGTATTTTTATCAGCATGACATTTCCAGTTTTTGGGCTCCTTTCCGGTGTCCATCTGCTGATTGCAGCGCGGAAGGGGTGTTCTATCTGCACTGCACAAGTTTACGCAGATATTCATATCTGGGAAGTATTTATCGCCGTTCTTGACATCCACCGGCAGGCACTTGTTCGAGGTAGAATTGTAGTAGAATCGCCGAATGCCACCGTTGTCTGAGCTTGGGTCAGATTTCATAGTGCAGTTGGGGAAGCGCTTTTTGAAGTAATTTAAAACATAGTCCGATAGTTTTGATGTCGATCTGCAATGAGAGACGCATTCTGGCAGCGAGAGAAAATTATTATCGTTGCCGTCACATCCCATAAAGCCAAGGAATTGGCAACTGTTGGTTTTATTGTCATAATAGAAATCGCGTCGCAAACAGCTTGTGCTTTTATCGTTGCACATCCAATAATTCGACTCTTTTGTCTTGCCTTCTTTGCTTTCGCACTGCAGAGTTTCACACTTTACTGTAAAAACTGCGACAGCTGA |
| >SG4817523  GGAAGCTCTGTCCCTCGAAAACTGAAGATATGAATGCGCTGACCAGAGTACAAGCATGCCTTCTTATCTTCCTTGTCTGCTTGACTCTCTCACATGCACGTACAGCACAAAAGAGATTGTGCTTTCTTGGCAGCAAGACAGGGCTTTGCAGAGGATATTTTCCTCGTTGGTCTTACAACCGCTGGTCTGGTGTCTGTGAAGTTTTTATCTTTGGCGGCTGTGGTGGAAATAAAAACAACTTTGAAGACTGCCAAACTTGTATGAAAACATGCACTGTGAACATTAGTTATCAAAAACGGAAAAACATTTGCCACAGGCAAACTGTAAAATACCAGCGCTTGCTAAACCCCACAGGTAGAAGGCCCAAATAACCGACCATGCCTAGATCTGTGACCCAAGGAACTTTCGCTGCAATCAACCTTCTGCAGTATCTTTCGACCAATTGAAATAAAC |
| >SG4818041  CTTTTATTGCGTAACTAAACATCGCCTTATCATTTCTGGAAGGCATGCATAGACGAACCTACTCTGGTCCTCCCACGCGGGGGCGCAGAGCATTGCCATATTTTTCGATCACTTCAGTAGTCAGTTTTTGGCAGATTCTCCATGCATTTTTGGGTTTTGCACCGCTGCATCTTTTCATGCAATCCAGACAGTTTGAAAACCTGTTGGCGTTTCGGTTGCAGTGGCCGTGCAGGAAAAAGCGACAGCGGCCAGCATCCGAGTCATATGCCCATGTATGAACTCCCGTCTTGCAAGATCTGTTTGGTAGCCTAAGGCTACAAACTCGTTTCACGGGAGCTCCAGGCAGGCAGGTTTTTTGGCATTCGACCTCACTTTCAAACCTGTTGGAATTGCCACCACATCCGCCGTATATAAATACTTTGCAGTGCTTTGTTGAGACATCATAATACCATCTCGGGAACTTTGCTTTGCAGTGGCCAACTGCAGGTAACTGCAAACAACTTTGTGGTGTCCGATAACTAGCCGCATGCGCGGACACCAGAAACTCCAGAAGAATAAGGAAATGAGCTATAATCATAGTCGACGTATTGCTACTGAGAGCAC |
| >SG4818353  CTCGCATTCAAAGCACGTAAGCTTTTATTTTTGCTTCTACTCCCTCTTCAGCACAGCAGACGACAGTTGTTTTCTCTCTTATGTTCATGGCACACTATACCGTTATTTTATGAGGTCTCCTTTCTCGCTTTGCTTGGTTTTGGAAGTAAGGTCCCATGGTGACCGCATCCTACCGCTTCTCCAGTTCACATTTCTGGCGACAATCACCGATGTTTTTAAAGTTGCGGCCGCCACGATCACAATCATTGCGCTCTACGCAGATGCCTTGTTCTTTGTTGTACGAATAAGTTTTTTTCTTTTCCTGGCAGTAGTTGGGATCATCTGGAGGTTCAATGCAGGTTGAGCCTTGACAGGTAACTTCACACTGTCGTAATGTGTTGAACTTATTTCCGTTGTCTCCACAGCCAGTATTCAGGAAAAGTGCACACGAATTTTTTGAACCATCAAAGTAGTACATTAACCGGGGTTTTTCACGGCATTTGGAAGTAAGATCTTCAGGCTTGCGAGGACAGTCAATAACTGCCTGACATGTCCAAGCGCACAATTCCATGGTTTGGAAGATGTGACCGTCGATACAATTTTTGTCTGTATACACGCATTCATGCTTTTTGTCATCATAATAGTAACCACGCTTGTGAGGTGAACATTGCTTCCAGCCCGAACGGCATTTGTCAGGTTTGTCCGATAGCGCCGCAAATACCATCACCATCTCGACAGAAAGCAACTGCCACAAGATGATATACATTGTGCCCTGTTCAACAAGACACCTGCCTGTGCTCTG |
| >SG4818456  TTTTGATATAAAAAAGATTCTTTTATTGCATACCAAAGCATCGCTTTATCATTTCCGGAATGTGCGCATTTACGAACCTACTCTAGTCTTCCAAAGATGTGGCGGAGAGCATTGCCATATTCTGCGATCGCATCAGTAGTCAGTTTCTTGCAGAGTCTCCGTGCATTTTCGTGTCTCGTTCCGCTGCATCTGGTCATGCACTGGAGACAGCTTGAAAACCTGTTGGCGTTCCGATTGCATTCGCCGTGCAGAAAAAAGCGACAGCGGCCACTTTCGAAGTCATATGCCCATGTATGAACTCCCTTCTTGCAACTTTTTTTTGGTGGCCTAAGGCTACAAACTGGCATCACGGGAACTCCAGGCAAGCAGGTTTTTTGGCATCCGACCTCGGTTTTAAACCTGTTGTTATTACCATTGCATCCGCCGTATAAAAATACTTTGCACTTGTTTGTTGGCGGATCAAAATACCAACTCGGGACCCTTGCTTTGCAGGGGCCAACTGCAGGTGGCTTCAAACAACGTGGGGGTCTCGCATAACTAGTCGCGTGCGCGGAGACCACAAACCCCACAAGAATAAGGAAATTAGCTGTA |
| >SG4818467  CTTTATTTAAACATTGTGTCTAGACATTCAGCTGAGTCATAAATGTTTTTATGGCAGAAGCAAAAAGAATTCAATTTCTTTGCAGATTTTTTATATACTCGTCAAAATCGTAAGTGTCTGGCTGGCAGGTTTCCTTGCATGTATCTGGATCTGGGAAGTTATTGATGCCAGTTCCGCATCCAAAGTACTGAAACTTCAGGCACTTGTCCACCGTAGCATTGAAATACCACTTTTCAGTAGGGCTGTCGCTATCACAAGAATTTTCTCTTGGAGGTGCATCACGGCACCTATCTAGTTCTGGGCTGTATGGGACATCCATAGCAGTTGTGCCCAGAAATACTGTTAAAGAAACCAGGTAAATAAAGATGCAGGCCTTCATTATTGATCCTCCCGGTAAGTGCGCGTGCCAGCAGTGTGTTCA |
| >SG4818977  CAAAAACAGAACCGCATGCTCCATACGCTTCCGAGTTTCTACTTCTAGAGCGCTAAAATTGTAATGTCCACGTCATAACTGGCCATCCAATCAGTTTGCGGATATTCCGCCCATAAACATCGCGAATAAAAGGCTGGACTGCAGAGTAACTGCTTAATCTTATTGCAAGAAAACGCAGTCCTTCGCCAAGTTTACAGATATGAGCAAGATGATCCGATTCCAAGCGTACTTTGTTCTGCTTACCGCCTGCCTTGCCCTCTCACATGCAGCGCGTGCTCCGAACAAGAAAGTGTGCCACCTTCCAAAAGTGCCTGGACCCTGCAGGGCTTTCTTTGTTAACTGGTATTATGTCCCACAACGACAGGAATGTCAGCCTTTCGTTTACGGAGGCTGTCAGGGAAATGCAAACAACTTTCCTACCTGCGAGAAGTGCATGAAAAAGTGCACAGGAAAATCCCATATGAACGTGAAAAGGCTCTGCAAGAAGCTCAGAAAACTTGCTGACAAATACAAGCCAAATGGAAGACAACCCAAATAGGCGACGTTGTCCGCAGCATTCAAAATGCTGGCAGTATATTTTGCTAAACCTATTTGTTAAAAAAAG |
| >SG4819160  TTTTTCTTCAAGACTGTTTTATTTTTAATCGCAACCAGCACAGAACTGCTGCTGCAACCTTCCGGCGTTCGCTTTTCGCTGTTCTCCAACAATTCAACTTATCACTCTCAGCCTTGCATACAGTGCTTACGGCACTGTTCATACGTCAAGAAATTGTTCAGTCCGCCACCGCATCCAAAGTAAGGAAACCATAAGCATTGCCTTGCACTAACGTCGTAGAAGTAACGATATGATCCCATTGCTCTGTCGCAGGTGTATCCTTGATTTGGGGGATGTCTGCAGTAGTCCGGTTGCCCAATTTGGCCACCGCAAGTCTTGATGCATTCGGCTGCGGTTGGAAAATTATTGCCGGTGTAGCCGCAGGATAAATCAGGAAGGCAATTTTTCGAATGGGGCTCATAGTAGAAGACATTCACTTCAGAACCAGCTACGCATCCAGTAGCAGCTCGGGGAGCTGTGCAAACTTCTCTGCTTCGACAAGTTGCATCGCAGAGTGTTTTGTCTTTAAACGGCGCAGCGGAATTGCACGTGGATACGCATTCTCTCGACTTTTGATCAAACTGCCAGCCAGGAGAGGCGCAACCAGGTGTCGTTATGATGGGGCTCTCCAGAGTGCATTTGGGATCATTCGCTACCGATGATTGGCCTGCTGAAAAGTCATAGAGAAAGAGCATGATTAGGGTGCGCAACATCATAGTACCTT |
| >SG4821337  CATCTTGGTTGGTGCTTAGCTTCGTTTTTCGAGACTTGCGCCGTGGCACTCCTTGTGACAGGTCTCGTGCGAGGGGAAGTTGTTCCTGTTCCCCTTGTGTCCGCAGAAGGTGAACGTCTTGCAGGCATCCTCGACCACGTTGTAGTACCAGCGCTGGGAACAGGGCCAGCGACTGCCGCGGTTCGGCTTCAGCGTGCACAGGTCCGGCTTGCGGTG |
| >SG4821711  ATCTACGGCGGATGTCGAGGAAATCGAAACAGATTTGAGAGCTGCTATGAATGCATGAAAAAGTGTGCTCAATTTCCTCAAAAAAGGCAGTGTAGAAAGTTAACTGAAGAAGCCTTCAAGAAATACTTGCGACGGACACCGAAATAAGTGGAACCACCAAGCCTAATTAGGGTTGGCAAAAGAGGCGACAGTCTGTTTTCAAGAAAAATGCTTCGACCAGTTCATTAAACTGTGTAACGCTTCCGAAAAAAAA |
| >SG4823978  CCATGCTGTATCAGGACGTAGATTATACAGTGCTTTACCGCTTTTCCTCCTCTTGGTCATAGTACCAGGTCAGAGGGTCGCATGTTTCCTTGCACATGCCACTATCAGGAAACCTATTGGCATTTCCTCCGCATCCCAGGTATTCGAATGTACTGCACGTTTTTGTTGTTGCATTGAAGTTCCACATTTCACCGCTGCGCCCACCGACGCAAGGCGTGCCTTTGTCAGGAGCCTTGCCGCATTCGTCTTCAGCTTTTATTTCAGGGACATTCGTCGTAGTTGACCACTCAAGTAGTGTGAAAAAAAGCCAGCAGTATGAAGACCCAGGACTTCATTATGGATGCAACGAAGCAAGATACTTTCAACAGCACTGTCGTTCACTGGTGCGCAGGGGAACCCAAATTATTGTACGGTGTCGCTAGTGCACGTGCCAGCAGTGTGTTCAACAGCGCAGCGGTGCCTGTCGGAACTCCAATGACTCTTCTTATGCCCTTCCTGTGGAAAACTTTGAGACGCTCAAGTTGTGATGCCG |
| >SG4825016  GAGAACGGAGAGTATCGCGAAGTCTTGCAGCTGTCGCTCTCGACGTCGTAGTAGTATTTTTTCACAATTCCAAACCTATTCTTGGGACCCGGTTCTACGCACTTGCTCCCAGAGTTCTGGCCACAAGTGTTCCAGCACTCCCTTGCGGAGTCAAACCTGTTGGGGTAGCCACCGCAGCCGTAGTAGACGAACCTTTCACACCTTTTGTTCTTGTTGTTGTATCCAAATGCTACCTTACGTGTACCATTTGGGCACTTCTTTCCGCGATGTATTGGGTAACTGCAATTGCTTTCGACGTATTTGCCGCATGTTTTCCAGCATTCGGATTCGTAGAGAAAATTGTTTCCATTTCCTCCGCAGCCTTCGTAGTCAAACTGTTCGCAGACTTTTGTTTTACTGTTGAACC |
| >SG4825717  TTTTTTACCACTGTTCTGATTTAATTTCTAGTTTTTGCGTCCAGGTACATATCTTTAAAGCCGTTTTGGTTGTCTATCAGTAGTCTTCAAGCTGGAGGAAATCATCATCGTCTTCATCATTTTCTTCGTTTTTTATCGAAATTGCAGGCAGAGTTTTATTGGCAATATCGTCGAATATCCAATAATCCGTTACTTTGAAACAAACTTGAGCAAGACCATCATTTTCATTTTTCTTGCTTTGTATCGAAATTTCAGTAAGATTCTTCTTGGCAATATAGTCCATTATTATTTTGAAACAAACGTATTTCGGTGATTCGAAGTGAATGTGTGTTTCCTCCTCGCACAGCCTTTGGCAGTCGTCTATCCTGCCGAAATTGTTTTCGTTTCCACCACAGCCGTAGAAGCGGAAAGCTTTGCAAGACATTCCGTCGAAGTACCAGCGAATCTGAGTTGTCTTAGTTGAGTTTTTGCATGATTCGTCTCCCCCATCCATTTTCAGTTTGCAAACTTCCTTGCTTTTCGCTGTCCGTGACTCAACACACGTACCCGTGAAGATTATGAGCGACAGAACGGCAAGTTGGCGCATTTCTGAGCGAAGTGGGTTCCTGTTGACTCCCTTTCGAAAAATGCTGTTCTGGCAAACTGGCACG |
| >SG4827168  AGTTGGCCCCTGCAAAGCAAAGCTCCCGAGATGGTATTATGATCCCTCAAACAAGAAGTGCAAAGCATTTATATACGGTGGATGTGGAGGTAATTCCAACAGGTTTCACACCGAGGTTAAATGCCAAGAAGCCTGCTTGCCTGNNNNNNNNNNNNNNNNNGTTTGTAGCCTTAAGCCACCAAAAGGAAAATGCGGGAGACGAGTTTATTCATGGGCGTTTGACTCGAATGCTGGTCGCTGTGGCTTTTTTTTGCACGGTGAATGCAAACGAAACGCCAACAGCTTTCGAAGCTGTCTCGAGTGCATGGACAGATGCAGCGGAATGCAACCCGGAAAGGCACAGAAACTCTGCTTGAA |
| >SG4828577  ATTTTTCGCGGGTCTTGGCTCCTAGCGCATGTCTGCATGCAGCTCTGGCAGCTCGAGAACACGTTCCACCTAGCGTTGCATCCGTTGTAGCTGAACAGAAAACAAACTTCGTAGGTTCGATTGTAGTACCACCTTTGAACATTGCTATTGAAGCGTCTACAATTCTGGCGAGGTGCTTTATTGAAGCAGTTGCGGGGATTCTTGTTAGGCTTTCTCTCCAAGCCATAGAT |
| >SG4831932  TGTAAATCATTTAGTGCCTTAGTATTGAGCGTTGTTTAATAAAATAAAAAGGACAGTTTCTGTACATACCTCATGCAACTTCAGATATTCCTTCAATTTCCTGTCACAGATGAGAGGATAGTCAAAGCGAATTCGAAAAAAAATAAAAATAGCATTCTTTTAGATAATTAAAAGGTCAGTTAAATTAAAATAGAAGTATTGAAGTCAAAGTGAATTCTACAAGTTATTAGCACACAGACAGCAAATACTATTAGCTGCTGGGGGCGCTAGAAAGGAAAACCCAAGAGCATTTACAAGTAATGTCAGAAGTTTAAAGACTCGGTATAATAAACACTAGTATGTTACCCCGGCATGTGACATGCAGACGCCATAAAATAGCTGTTTATTTATACTCTTATGGTACGAAAACAATACTATGGGAAGGACAAAAGGGAACTGCATAATTTACTAACAGCATGTATCTTTATACAGTAATCACATTAGACGAATTTTGCAATAATTACACCTAAAAAGTCCCAAGGCCTATATTAGTACTCTCACGAAAATAACCAAATGAAAATAGTATGCATAAGTGCAACCACACATGTGATGGCTTTCCAGAAACACGTTAGACCGTGGAAGTAAAAAGATAAAGAAAACCTTCACTTCATGACGCAACACTACAACACTGCAGTTAAAGAGGTTTTTAAGAGACTGGAGTCATCTTTATTTGTTGGAGACGCTAGTAAGCTAGTTTTCTTATTTTGTACTATTGTATGCACACATTTTTTGAGGCGGAACAATGTGATACAGCTGTCATTCTTGCTGCTGTTTTCCCAGCAAGATTGGGTATATGCACGTCAAGCGCTTTCTTGTCCAATCGGAAGATCCCAAGCTATTTTGGCTCGTCATCCCTTGTGCCTCCGGCGTCTTCGCAGACACGCCTGCACTCGCGAAGTGTCCGAAATCGGTTCGCATTTCCCCCGCACCCTCCGTAGACGAACCTTCTGCACCGACCGAACAGGCCGTCGTAGAAGTAGAGCACGTAGTGGCCGAAGCAGTTCCCGGAACTTGCGGGCAGCGAGCACAGCCTTCGCCCGCTTTGCGCACCGGCAACTGCACGAGGAGGGATCGGAGCTCGTGTTATATGCTTTTGAAAAGAAGGTGCCAGATTTTCTGCCCTGTCCCAAAATGAACTAGCGGCAGCGACAGCAGTATCATTCCTCTTTCAGCGGGAGCGCGCTCCAAGGGCCACAATGTTCAAGTCCAGTTCGATCATAATAGCGAACAGGCCACATTAGTGAGAGAAATTAAACTTCGTTAAAAAGATTAGCATTCAAATGTGGTCTTCTTATACCAGGATATATCGCTGGATGCTGCGTTACGTGCACGGTGGACGCGCGTGCTTTTAGGTTACGACAAAAGTATAGGGCCAAATACTTTTAATTTGGTTTAAG |
| >SG4833830  CTGCCTCTTACACTAGTTATATCCTGCATGAATTTCCCGAGATCTCACAAAATTTTTCAGTTATTTTTAAATTGAATGTGTACGCATATGTGTTATAATCTTTTAGGTCTAAGAATGTTTAGCACCTTCTTTTTCGAAAAGCAGTAAGTGCAACATGTACGTCTATGCGTGCCGGGGCATCGGCTCCCAAGGAGACTGAATGGCGCAGGCGTCCATGCACTGGCGCAGGGTCTCGAAGTTGTTCCCGTTGCTGTGGCAGCCTCCGTAGATGAACTGCCGGCATTGCTTTGTTTGAGCATCGTAGTAGAAGCGCGGGATGCTAGCCATGCAAATGCCGGAGTATGCAGGGCGACGGCACACATCAGCCACGGATGCGAACAGTCCACTGCGCACTCCCCAATGCCTTTCGGGCCGGAAAGAAGGTTTACTCGCAGAACACGTCATTTCACACTGTCGCTTGCTCTCGTATCGGTTCTCGTTTCCTCCGCAGCCGCCGTAGTTGAAAAGCTCGCACTTTCCAGTCCTGGCATTGAACCACCACATCGGCATCATCGCCTTGCAGGGACCGCTATCGGGCAGCTGGGTGCATCTCATGTCAAACGCAGTCGCTGCGAAAGCGGCGCTGACCACGGCAAGCAAAATGAAGACCTTCA |
| >SG4834609  GTTGCGACCCGCGTGCTTTGGAAGGAGGCATGAATCTTGGGCGAATTTTCCACATTGCGCCTGGCACTCCTTTTCAGTCTGGAAATGGTTCTCATTGTGCTCGCAGTAGATGTAACGCACGCATCTTTGCTTGAAAGGATCGAACCGGTACCCTTTATATCCAGCTCGGCAAATCTGTTTGGGCTCTGGCAGGGCACACACTCCGAGAACAGGGTCTCGACATTTCTTACTGCATTCCGCAAATTTGTTGAACCTGTTGCCGTTTCCATTGCAGCCTCCGTAGTAAAATACCTCGCAACGGTACGACGTCGCGTTGTAAAACCATTTCTTGAACATTCCTCTGCATATTCCGGGATCCGGCTCTTGCATGCAGTCTTCGCTGGGCGGCACAGTTTTACTGGCAGCGCAGATTCCTGCTAGGATGAATATGAAGATTGCAGTGAAAAGACTTTGTACCATGATGAGCAGCTCGGGTTCTTGAGTCGAAAGCGGCACCTGACAG |
| >SG4837489  CTGTGCCATAACTTTGTAAGTGGGACAGCAGGTCTCACTTAGGTGTTTCAGTCAGGAACATAAGGTACGTAGCGTAATGCGATGCTAGCGCTGTCATTGGTACTTCCGATCCAGAAGTGATGTCAGCCGCAGACGCGGAGGCATTCCTCATATGAAACAAAGCGATTCTGGTTTCCTTGGCATCCGCCGTAAACGAACTCTCGGCACGTCCCGGATGAAATGTCATAATAGAACGAGGGGAAGTAGGCGAAGCAGACTCCGGGACTAGGCGGCAGGCTGCAAAATCTGGGCCTTCTCTGAGCATTGGCACCATTGAACGAGATGGCCAAAAGCACAGCCGCCAGCACAGTCAGTCGAAGCATCGTCGACACCTGAGATGGTCG |
| >SG4839196  ATTTTGGCGCGCTCAGCCTGTTTTTTGGTCAAAACACGCGCGTCGCCTGCGGCAGTGCATTTCGAGCTGGCGCTTCTTTGAGCGTACGAGCCCTAATATCGGCCGCAGACTCTGCGGCATTGTCCAACAGAGTTGAATCTGTTCGCGTTGCCACCGCATCCGCCGTAGGTGAACGAGAGGCATCTTCCCGATCTACTATCGAAGTAGAAGGCTGGGAAGGCGCCGCGGCAGGGTCCCTGGCTAGGAGGAAGGAAGCACTGGCCTTGAGCATTGGTGCCTG |
| >SG4843029  GTTGTAAGATAATCATCGCAGATTTGTCGTGGGTTTCCGTTACCAGCGCATGTCTGCATGCAGCTGTAGCAGGTCTCGAACACGTTCCACTTAGCGTTGCATCCGTTGTAGTTGAACTGAAAGCACAAGGTGTAGTTTCGATTATAGTACCACCTTTTAAAATTTCCTTTGAAGCATGGTGTTGACTTGGCAGGTTTTTTATCGAAGCAGTCTCTGGGATTGTGGTGACGTTTTTTGACGGAGCCACAGAT |
| >SG4843518  GCTTTTTCCGTTGCGATTAACTGGCACTAAACTTGCCATGTTGTCCTGCTGTGACTCTCTCCTTTGCGGGAGTGAAACAGTTTATTGGGGTTTTGCATTTNNNNNNNNNNNNNNNNNNNNNNNTGAGCGCTCGCAATCTCTATTTGTTTTAAAGGAAGCGCCACCTCGGTGACATTTCCTGGGCTGTTCTCGGCAGGCGCCTTTATTTTCGTCATAATAATACGTCTTATACGTCCCTTGACATATATCATCATCATCCTTTGGCGGGGTGATGCAGTTCAATCCTTGACAGGTCACNNNNNNNNNNNNAACTGTCGAAAACTTGTTACCATTATCTTTGCATCCCTTATATAGAAAGAGTACACACTGGCAAGTTGTGCTGTCATAATAGTACATAGGCTCATGTGTGCCGCAATCGTTTTC |

| >MG120199  GGGCTACGTAAGCTTGCAGGCAATCTCGCACTCTTCCTTCGACTCGTAGTTGTTGTCGTTTCCACCGCAGCCACTGTAGAGGAACGTCTCGCACTGACCCGAGGTCACGTTGAAGAACCAGTGGTCTAAGAAGCCCTTGCAAAAGCCGCGTTGCGGTTTTGGCGCGCAACCAGTCTCGAAGTCAGCTCCCTCGAAGTCACTGGACCTCTCTGGCTCGCCACATGTTTTTTCGCATTCCTCGATAGTTTCGAAGTTGTTTTCGTTTCCTTCACAACCACCGTAGATAAAGAAGGAGCATTTTCCTTCAGCTGGGTTGAAGTAGAACCGCGGAATGCTTGCCCGGCAGA |
| --- |
| >MG120366  GTTGTGCTTTAAAGTTGTTGCCGTTGCTGCGGCAGCCGCCGTAGGTGAACTGGCGGCAAGTATTGCTGCTGGCGTCGTAGTAGAATCGGAGGAAGCTCGCCTTGCAAGGCCCAGTGTATGGCGGCTCCATGCATACGCCTACGCCGGATCCGGGAAGCCGCCCCTTCTTGTCATCGAAGTTGGCCTTCTTGTTGCTGAATGTGGTGAGAGGCGTTTCGTTCAGTGTTTTCGGAGCGCATGTCTTTTCGCAGTCCTCCTTGTAGAGGTATCTGTTCTGGTTGCCACCGCAGCCTCCGTAGTAGAAGAGCTCGCACTTGCCAGAATCCGTATTGAACCACCACCTTGGAAGCTTAGCCTTGCAAAGTCCCTTTTCCGCTTTTGCAGTGCACTGTTTGTCAACATTTGTAGCCGCACAAGCGGCGCCGATGAAAGCAAGGAACAAGTAGGCCTTCATACTGAGAAGGAACAGTCCTCCACCTTGGAAGCTTAGCCTTG |
| >MG1201637  GGCAGCGGCTGAGACGCCATGGCCTCTGCGGCTGCTTTGTCGTGGTTCCTTGTCACCTCGCTGACGGGCCTTGGAATACTGCCCGCGCCGACGACACCGGCAGAGGCCCTGGTGTGTGTCCCCAAGCCGGCCAGCGCCCTCGAGGACCTCGATTACAAGAAGNNNNNNNNNNNNNNNNNNNNNNNNNNNNNNNNNNNNNNNNNNNNNNNNNNNNCGAGGACCTCGATTACAAGAAGTTCTCGGGAGACTGGCGAGAGGTGTTGAAGAGCAAGCACGCTCCGGTTCTCCTGCAGAAAGGCTCTTGGCGCTTTGACTTCGACACCAACACGTTCGCCTTTAGCGCAGCACTTGCAGGCAATGAGACATGCCTGCCGGCCATTGTCGGCAAAGTCCACAAAGTGCTGCCCGTCGGACAATTTCAGCTCGAGTACGACTTTTTTGGGCAGCCTGTACAAGAGACGCTGGCCGTGGTAGCCACGGACTACGGGAGCTACGCCGTGCTGCACCGGTGCGCGCATGAGCAGCCCCACGCGTCGGTGTGTGCCCCGGATGCGACACACGTCAGCGTCCTTGCGAGGCACGTGCTCGAACGAGACACCTCTCTGGAGATCACGCGCCACTTGGAGAACGTCTGCGTTGCTGTCGCACAGCTGGAACCGCACGAGTTTGACGAAGCATGCACTCTACCGTCCGATGAAGATCCCACCTTGGCCGAAATACCACCAGGCACGGGAATATGCTTCCTGGAACGAAAGGAAGGCTTCTGTGCGAATAAGCAGACAGTCTTCTACTACGACCGAGAGCAACGCAGCTGCCTGAACTTCACTTTCACCGGTTGTGGAGCCAACGAAAACCACTTCCTTACTCGCCAAGAATGTGTTGAAAGATGCGAGAAGCCACTGGAGCAAGCTCGAGCTCCACCTGTGGAATCCGAATCAGCCCAGTGTCCCCAGCGTGCAGCATGCGGCCTAGCCTGCGCTCACTGCTGCCAGCGGCGAGAGAAAGACAGCTGTATTGCGTGCAATTGCCTCGCGTCCGAAACAGAGCCCCTTCTCAAGAAAGCTGACTGCCTCGCGGCTCCCGAGCTGTGCCCGGCTGGTTGTCAGAGTAGGGAGGTGTCTGCGTCGTGCTACGCCTGCGAATGTGCTGCTGCAGACGGCACTGTTGAATATGCACTGGAGTACGTGGATGCCCAAATCGCAGTGCCCTGCCCAGTGACTTGCGTGAAGCGAATTTCGAGCAGCGGCGCCCAGAGCTGTCACTGTCCGTCTGATGGTGCGTGCAAGTTGCCGGTGAAAGAAGGCAACTGCAATGAAAGGATTCCCCGCTACTTCTTCAACGTCACTTCCGGCGTTTGCGACGTGTTCTACTACACCGGCTGTGGTGGGAACGAGAACAGCTTCGCCTCCCATGAAGAGTGCATTGCCCAGTGTGAAGATCCCTGCCGCCTGCCAATGGATCCTGGCAGCTGCAACGACACGCAGGAGCGCTACTACTTCAACTCACAGACGGGATTGTGTGAGACCTTCGAGTACGGCGGCTGCGAAGGGAACAAGAACAACTTTGCAGACCTCGACGCGTGCAAGACGCTTTGCGAAGATGTTTGCTCGCAGCCCCAAGACCCGGGCCCATGCTACGCGTACTTCCGGCGCTTCTACTACAATAAGCAGGAGGATCGCTGCCTGCCTTTCATCTTCGGTGGCTGCATGGGCAACGGAAACAACTTTTACACCAGCACCCAGTGCAACGGACGCTGCCGATTGCATGTTGCTGAGGACGGCGCAGCCGGAAAGGACAGGGTGCATGTTGCTGAGGACGGCGTAGCCGGAAAGGACAGGGAAATGTGTCACCAGCCGGTGGACGAAGGCCACTGCGACACCGACAGCAGTGGCCCAGGGGCCACTACACCAGAGATCCGCTTCTACTACGACGTGCAGAAAGAACTGTGCGAGCGGTTCAACTACCAGGGATGTGGTGGAAACGACAACAACTTCCGTACCGTAGACGGATGCAACATGACCTGCTTTGGTGTGCGCATCAACTTGGCCAGGACGGCTGCGAGCTGCCCGGAGTCCATGCAGTGCAGCTGCGGTGATGAGCAGGAGGCCAAGTGCGGGTCATGCGAGTGCGGGAACGCAGCCCCGCGTAGCAACGGGGCCGCCCTCTACGCTCTAGTGTTCCTCACTGGTGCATCGGTGACAGCGTCGCTCTGCACTCTCTGAAGAAGCGACGAGGAGGACGGAAATATTTCTTTTTTGTTCCCAGATTCTCGATTAGCGCATGCGTGATCACAGGCGCACGCGGTGTGTAGTGAAATCTGCGCGCACTGGCAGTAACTGCCAAGGACGTCCACGAATGCAATTTGTGTCCCTGGCGATATGAACTTTCTGTCGCATTCCATCGGTGCGCCCAATCTCTAAAATATGCATTGTCTTTGTGGCAGTCAACGGAATTTGCTTACAAGCAGCGTCTTTTTCAGCAACACTGGATAACTGCAGCCAGGCAATCTTGCCCTCTGCACAAAATTTTCTAACGCAACAAATGCCTCTGGCGGCGTGGAGACTATCGGCAAGTTCAACCACGGCGCATCTGCCTCGACGCGCTCCTCCACTTAGCGTGAGCCTGCCACAGATAGAGCCGAGCCGACGGGCAGCGCGGCACAGCAATTGTAAGGATGTGCGCGATTGTGTGCGGTGCCGCTCGTAGTCTTGGCACATGCGCCGCAACTAACGATAGGGACGCAGTGGCGTTAGGCATAGTGCGCGCGAAAAGAATTTGGAACAACCGAGGTTCCATGGTGGCACGGGGAAGGAGCAACACGAGGTTTTTAGGTCGCACAAGCAAATCGGTGACGTGAATGCCTGGTTGCTGTCATTACCCTAGCACGGGGAATGTCGGTCGACGACAGCCGTTCGCCAAGCGCGCATACTATCGCCTACTAGTCCAAGGTTCGACAGCCCGTTTGGCCAGGAAAAGACATTGCAGGAGGAGAAATCGACGAGTGCCAAACAGAAGGGTAAAATACAAAGGCGGGGGCTTTTCGAGACCCTTACAAGCGCAATGTTCACATGTAAAAGGAATGACTGGACAGCCGCCTTGATATGCGCGATCAGCGCCACAGTGATCATGAGTATGTTTCGGGCAAGGGCTCACGTGTCTGTGCGCGATTGGTTGTGTGGATATGAAATGACATCAGGTGCAGACATGATAAGAATGGCTTTTCGATAGCCATCATTGTAATTCTGTCCCAGACAATAGTGTGTCTGGCGCATTCGTGGTGTTCTACCAGGCCATTCAATGCCTTTTACTTTTAGTGACACAAAACGAAGAAATTATCTCTGTGGTTTCAGTAAGCACTTTCAAAAATTCTTAATCTTATGGTTAAAGAGGAAAAATCTCCGTATAAATACATTCGCACCCTCGGCGGACGTCAGTTACTAAACGATTCGCACTAGTAAGGCATATGACAAAGTACGCTTTAAGATTAGCGTTTTGGTTAAAACGGTGTGGTGACTAGTGCTAATTGAGAGCTGGAAGCGAAGGGAAAGACGATGCCTTATCAGTTTCTGAAAATGTTCTGTAAAACCGTGCAGTGCGAGTAAATGCGAGGCTCTTTTTCGCACAATACCGAAACTGAGTGCATGAGGTGTGCAGGGAGCCCAGTGCAAACACAACGACCGCTGGTGACACGGTGCAGGGCCCACAGTCTCAACACAAATACACCGCTATTGCAGTGAAAATGAAGTAATCTGTCCTAAAGTGCACTCCCTTTCACAAAAAAAGTGCGCTAGAGGAAAGCGTACACCTGTTTTATTCTTTCTGCGTTTAGAGTGTACGGCGCCCAATTCTAACTGCCCGCACATTTTGCACCACGGCATACACTTTTGTTTGCTTTGCCACTTGGTCCAAAAAAATTATAGTCGTAGCCACCCGCGTATCTGTTATTTTTTAGAGGGGCGAAGCAAAATGAAGTGCATGCCGTGGTCCAATGAAGCTTATATTTAACAGCGTTTTTCGGCGAGCCCTTCCAAAATTGCTCAAAATAGGGTGGTCTCAAAAGAGACTGCTATCCGCGGCAGTTCTGAATTATTTTGAAAATATGCTTTTTTCTTGCTAAATAATTAGCTCTCACTACTGGCAGCCCCGTAGTTTACGATGAGTATTCATTTTCTTGCACATTGCGACGAAGCTTAAGAGCACTTTCCGTGGTGTATTAGCAAAAGCAGTTCGAAGTACCGTGTCTATGGTTCCTAGGATTATCTTGCCAGCATTGGTCTGTTTTCATGTTACATTTTTGTTATGCTAACGACCAGACTCTGTTCATTCCAAAATAATTTATTAAATCTCATTAAAGTAGCGATTTGATTACTATAGTTGTAAAGTCACTCACCTCCACTGCGGCCGATTGTGTGTTCCTGAACAGTTTTCCTCTGTGTTTGAAACACCCTATAATTAGGAATGCTTGAAAGTGCTGGTGACATACCGGGCAAACGAACACATATCAAAAACCTCCGCATTCAAGAAGTGAATTGCTATGTCACACGTGAGCTGGCATCGTGTGAGGGTAGCAGCTCCTCAAAGTAAATTTTTTAACTCTCTTACAATACCCTCCTTCCCCCTATAATGATAGGTGCGAACGGTCCAGCCAGCCTCATCATCAGTAACTACGGCACCAGTTAAAACTCGATTTAACGAAAGCCGATTTTGTGAAGTTCTCGATCTAACGAAAAAAATTTTATACCCCGGCAAGTATTCATAGGGTTCAATGTTCGAATAACCTGAAATAACGAAAGAAACGATCACTAAACCCTATTTAATGAAGTTTTTCCGGGAATAAACGACGATGTCTCGCAGCTTGTTGGAAGCGTGCCAGCAGTACATCTCCGCGCCCAACTCACGGCCCTGGTTTTCTTCTGACGGGGCTGCATGCTGCGCTGCTGAACGGAACATAGGACTCGGCAGTCGGCGGTGCTTTCCACCTTGGCCTCGAATCCGGTTTGTCATATGGTGCTTTTATGCACGAGCGATTCGGGCCGCTTTCGCGGACTTTTCATACGCGAAGGCGTGGGCTGTCGGCAAATGCGACGCCTCGGTAGCTTTTCAGTGCCGCTACGTTAGAATTTAAGATCTCAAAGGCCGTAAAAATGCCGTCTTCGATTTTACGAACTTCCCGATTTAACGAAATTTTTAACGGCTGCTTCTCGCTTAGTTGAATCGAGTTTTAACTGCATTTATTTTGATTCTGGTTTACAGGTGCGAAAGAGCACGTGACTACAGCTTGTCCGCCTACATGAGCAGTTGTTGGCGCGTGCCGTGCGTTAAACTACGACGTTGTCATTTGCTGTGTTCGAGGTTAACTCGCGGATCACGAAGGCACAATTTTCAAGCAGAACTTAGCGGAGAAAGAAAGTACCACATTAGCAAGGACATTTCTGTGCAAGAGTTGCGCATTATGGTCAGCAATGAATGGCAATTTCATAACAGGTGGCATCTTTGCCATCGTTTTGCGAGAAGCTGCCGTCCCCACTGCCGGCTTTGAAGGGGGCGTGCAGCTGCTTCATCGCCGACTGAGCGTGCCTACCATGGTCATTCTGCTCTTTGTTAGGGAAGGTCCACCAATAATTCTTGTTGGGCATTTGGTCAAAATAGCAAACCACACCAAAAAGCTTCCATGAGATGGCATCACAGGTGGTGTACCTTGTTGGTGCATAGCATAAATATTAATTCCTACAAAGTCCATACTTATCTCACTTTCCTGCCCAGTCATTCACGAAGATTATATCAACGCATTAACGGGAAGTGCTTGAGCATGCAACAAACTTTTATGGCTTTTAATGCTGTTCGTCGGAACGGGTACAAAAAGCATATGATACGGGGCGAGCTATAAAGAAGTGGAATAGATTTTTAAACAAGAACAACTGAAAAGAAACCTATATCAACCATCTAAAATCATCGGCAGTAAAATATTTGCATTGCATGCCGCGCGGTAAAGAGCTGAAAACTCAAGTGTGGGTGTGTGCGTTTTTAAGGTTCATGTTTACTTTTATTTCTTTTTTCGAGAGCGAGTTGATGGGATAGTTTGTGCTCCATGGTGCAAACGCAGCGCAAAGGACAGAGACATTCAATTCAACTCAATTCAGTTCAGTTTATTTTCCAGAAAAAAGAACATGTCGCGGACAGATTTAAAGGCTAAAAGTTGTTAGGAACAGCTTGACTGCTCTCAAGTACCATTAAATGACATTCAACGACGGAAGGCACAAAAACTGGTTGAAAGCAAATAGCACAAGGGCATTTAAAACAAAACGAAAATAATCAGGGGGGACATTGTTTCTAGGAAGTCCGAGGAATTCAAGACGGGAGCCCCCATAGTAAATCGACATATCAACCCTATTGGTGATTGGTGACGTCATGAGTATACCGATCTGGTGATTGATTGGTGACGTCACATATTTATCCAATTAGTGATTGATCGGTAACGTCACTTATAGAACCAAGATGTCAAATTCGTATTTCCATTGGTGTACCTCACTTTAACCTCATTCATATCAATTCTAGCAAACCAAACAGCTAACGCTCGTTACTTCAACGTCTTCCAGAC |
| >MG1204909  CCGCAACGCTCAAGGGCATGACCAGGAATTTTCTTGCTAAAGTGTTCTTGCTTGATCATGGGGAGTTGAGAATATTGCGACAAGCGCCTTCGGGGTCTTGGTGAGTCGAACATTTAGTCATGCAGGCTGTACAGTTGGGAAAGATGTTCTTATGTCCGCCGCATCCGCCCCACAAGAAGGGACCGCAGTAGCCGTATTTCGAATCATAGTACCATCTTTCAAGTGTAGGCCGCTCATGTCCGCATTGTCCTTCCTTTGGCGGTGTGTTGCAATATTTTGGGGGTGTGTACCCTG |
| >MG1205880  TCCTGCTTTGCAAGTGCGCCGACATTCGCCTAAGGTTGGAAAGTTGTTTCCTGTGTAGCTGCAGCCCATATCGCTGTGACACCTGCCGGTTTTAGGACTAAAATAGTAAACTGGAAATATCCTCCCGCGGCAAAGGGCAGACGGTCTCGGAGCAGTGCAGACGGCAACACTTCGACAGGTAGAATCGCATTCTAGTTTGGTTTCAAAAGGAGCTCGGCCACATGTCCATGTGCACTGCTTGGTAGAACGGCTGAATGCGTAGCT |
| >MG1207783  CATACTTGTCTTTTTATTTCGCTTGTTATGGCACATACAGTGCCGCAAGGATCAAGAGCAGGACCGGCAATTTCCTTGGTCAGGTGTTCTTGCTTGATCATGGGGAGTTGATAATATCGCGACAGGCGCCTTCGGGATCTGGGTGAGTCGTACAAGTAGTCATGCAGGCTGTGCAGTTGGGAAAGTTGTTTTTATTTCCGCCGCATCCGCCCCACAAGAAGGGACCGCAGTACCCGTATCTCACATCAAAGTACCATCTTT |
| >MG12010634  GACACACGCAAAAACACCAGTACGGCAACATGAAGGCTTACATTCTCCTCGCTGTTGTCAGCTCCGCATTTGCTATCTTCCAGCCAAAACAAGAAGCCACAGTGTTGCTCAGTGAGCCTGCGAAGAAAAAGCCTGATCTGTGTTTTCTCCCACCGTACCCCGGCCCGTGTGTGGCTTTCATCCCACGCTTCTACTACGAAGCTAAGACAAACCAATGCAAGTCGTTCCTCTATGGAGGCTGCCACGGCAACGGCAACAGCTTCGAGACAATGCGCGAGTGCTTCGCCGTCTGCGCCACCCTGGGGCCCGTGCCTATAATTCATTAGAGGCTGTTTCAAGGTGGAAACAGCAACTTGACATCACTCTAGAAGAACAACAAATAAACCATGAGTGTTAGACAAAAGAACGATGTTTGGCTGCAATGATTCTCGGTCACTGGTGGGTCCTGAAAGAGAGAGGACGAAGAGTTTGTGCCTACCCTTTCTTAGGCTCACTGCAGCCAGATTTATTATGGAAGCTGAGAGGACGGAAAATGTGTTAAGAATAAGAATAGACTCTGACGGGCGCTGAATTAAAACTGCAGCTGTCGGGCGGAGAATATGACCCACG |
| >MG12011731  TTTTTTTCTAAGTGACCTATATTTTCATTTTATTGCCTATACTGAGATGTGTAAACTAAAGTCATTTTCAAGATGAAAATTATTATGTCGCCTACGACTTCACGGACGTCTTCATCAGTTTCCTCTATACTCGTGAAGTTCGACTTTAGTTATTTTATTTCTGCGTTTCTTTTGCTTTCACTTTCACAGCGTGCTCCGCACCCTACGCTGTCGATGGCGTGGGCTGTGTGGCGAGTGGAGGGGCGCAGAAATCCAGGCAGTCGCGTCGAGTCTCGAAGTTGTTTCCGTTGCTGTGGCAGCCACCGTAGACGAACGGCTTGCAGGAGTTGGTCGTGGCGTCGTAGTAGAAGCGCGGTAAGAAGTCCAAGCAGTAACCAGTGTATGGGGGCCTACGGCACACGTCTTNNNNNNNNNNNNNNNNNNNNNNNNNNNNNNNNNNNNNNNNNNNGCTTTTCATCAGCGCAAGTCTTTTCGCATTCTTCCTTTGTCTCGTATCTGTTCTCGTTGCCACCGCAGCCGCCGTAGAAGAATTCCTCACACTGGTGAGTCCTCACGTTGAACCACCACCTCGGCAGTAAAGCTTTGCATAAACCAACTTCTGGGTCCTTCGTGCATTTCTCCTCGTA |
| >MG12014102  GAGCCCATTCCGAAGAGTGTCAAGCCAGATACATCGCAAACGAACAAGAGGCTGAGTAAATCAGCTTTATTAATCTGCCTCTTACACTAGTTATATCCTGCATGAATTTCCCGAGATCTCACAAAATTTTTCAGTTATTTTTAAATTGAATGTGTACGCATATGTGTTATAATCTTTTAGGTCTAAGAATGTTTAGCACCTTCTTTTTCGAAAAGCAGTAAGTGCAACATGTACGTCTATGCGTGCCGGGGCATCGGCTCCCAAGGAGACTGAATGGCGCAGGCGTCCATGCACTGGCGCAGGGTCTCGAAGTTGTTCCCGTTGCTGTGGCAGCCTCCGTAGATGAACTGCCGGCATTGCTTTGTTTGAGCATCGTAGTAGAAGCGCGGGATGCTAGCCATGCAAATGCCGGAGTATGCAGGGCGACGGCACACATCAGTCACGGATGCGAACAGTCCACTGCGCACTCCCCAATGCCTTTCGGGCCAGAAAGAAGGTTTACTCGCAGAACACGTCATTTCACACTGTCGCTTGCTCTCATATCGGTTCTCGTTTCCTCCGCAGCCGCCGTAGTTGAAAAGCTCACACTTTCCAGTCCTGGCATTGAACCACCACATCGGCATCATCGCCTTGCAGGGACCGCTATCGGGCAGCTGGGTGCATCTCATGTCAAACGCAGTCGCTGCGAAAGCGGCGCTGACCACGGCAAGCAAAATGAAGACCTTCATGATGGCCAAAAGTGGGGACAGCACGATGGAACCTGTCCCGCAGCGGCCGATTTTTAAACCCTTTTCCTTGGAGGTGGTGCCGGGCAGGTGCTTTTTTATTAGTACTCAG |
| >MG12015660  CTTTATTTTCCGGTGCCAGGGGCAGAACTAGAATCCAAAATATGCGTGCAGCACTTTCTGGCTTTCGCAAGCATTAGACAGTTTAATGAACTCGCTGAAGTACTTTTCGAGAAAGGAGATTGTCGCGTATTTTGACAGCCTTAATTAAGCTTGGCGGTTCCGGTTATTTCGGCATGGCCGTCGGGAAGTATTTCTTGTTGGCTTTTTCAGTCAACTGTTGGCAATACTCTCTGTCTTTCTTAGTAAATTTTTTAGCACAAATGTTCATGCATTCATAGCAGCTGTCAAATCTGTTCCGAGTTCCTCGACATCCGCCGTAGATAAATGCTTCGCACAAGCCGCTTTTTGTGTTATATGACCACTTGAGGAAGAAAGCTTTGCAGCGTCCAGTTTTCCGCCCAGGTTCTCTGCAGATTGGGTTTGGGGGACGATACCCGCTTACGAAGGTCGCAGCGAGCAGCAGCACGCTGAGTATGGCTATGAGCTTCATAGTCGATTTCGCTCCTTTTTTCGTAGAAATCGGGAACGCAGCTCATAGCCATACTCAGCGTGCTGCTGCTCGCTGCGACCTTCGTAAGCGGGTATCGTCCACCAAA |
| >MG12019060  CGGGGATGAAATTTCTATTTATTTATTAATACTGTAAACATATTTAGGCTCATACAGGGATGAGCTACTTCACGCACACAAATAAACAACATTGAATGGTTGCACCATGTTTGTTTCAATCGCATTTGGATGGTGCTCTGATTTTTGCTTAAACGCTTGCCTTGGGTTTTTGTTCCATAGGTTCGAAGTTGCTTCCGAAGTACATCAGCCAGATCCGCGCATGCAGGCCTTCTGGCATTCCCACTCGGTGTTGTACCGGTTGGCATTGCCACCGCATCCACCGTAGACGAAGGGCTTGCAGACACCGTCGGCCACGTCGAAGTACCAGCGCTCAAAGTCTGCCCTGCACATTCCGCTCTCAGGGGGAGGCCTGCAGCCCAAGTCAAAGTTCACGCCGCGGTGCTCATTTCGGACTTGCACCTCCGCCGTTGCTGCTGCGAAAACAGCGCCAGCCAGGAAGCAAAGAAGCACAGCCTTTTTCATGTTATTACAGTTGACAGGAAACTAGTGAAAGTGCGAACACGCCTTCGCAGTTTTCTG |
| >MG12020017  TTTCCACAGAAGGCATGCGCCGAAATTACAACCATTGCTTCATCAGTACCCATTGCCCAGTGGGGTTTAATCTTCAAGGCGAAATTCTTCAAATCCAAACTTCTCCCTCAGTCGCTGCAGTGGTTGATGACAAACTTTAGAAGTGAACCAAAGGAAAATGCAACGCCATCGGTTGTGCCGGTGGTTGCGCTCTTCCTCTTAAGCAGACTCTTTCGCATTCTTCTAGGCTTGCAAAGTTATTACCCATCAGAGTACAACCAACATCTTGAAAACATGATTGACTCCCAGGATAATAAAAAAAGACGGGAAAGACGTTTTCAGGTCTATCGCAGTAGCTGGCAGGGCGTGGAGCATAGCACACGGCAGGGTCTTTCGGGCTTATGCTGCTATGCATGGCCAAGCACATTGGTAGAAGAAAGAAGAAGCCTAAAGCTTGGTAGATATTAGATC |
| >MG12021154  CCTCTTCTGAGTTGTAGTTGTTGCGATTTCCTTCGCAGCCACCGTATGCGAACAAATTGCAAGTCGCCGACTCCGGTTCGAAGTTATAACGATAAATAGTTTTATCGCACGGTCCCTTGTCTGCCTCCACAAAGCAGTCCAGAAATGCTTGCGGGTCCGTTTCCTTTATGTCCGGATTGCTCGTTTGCGAACCTCCTCCTCGCCTCAAACAGGTAAGTTCACATGCCTCCTTGCTGACGTAGTTGTTGTAGTTGCCCTCACAACCGGTGTACACGAATGGCTCGCACGTTCTCACCACGGCATTGAACCACCAGCTAGGAAAGTGACCTCTGCAAGGACCACGCTCAGCACGCGGCGTGCAGTATGTCTCGAAGTCGTAGTCG |
| >MG12024109  AAGTGCGTAGAACCGGGTCCCAAGAATAGGTTTGGACTTGTGAAAAAATACTACTACGACGTCGAGAGCGACAGCTGCAAGACTTCGCGATACTCTCCGTTCTCAAGCAAGAAGAACAGGTTCAACACTAAGGATGACTGTGAGGCGACATGCAAAGCTAACTACACCACTAGCATCGTAAGCTTCTGATGCCACCGGAGCAGTCATCAATGGCGAGC |
| >MG12024725  GAAACCGCTGTTAATTATAGCATCTTTAGACTGTGGTCATGGAAATCAAGAGATGCGTTCATGTTCTTTTAATTCTGGCCACCGTGTACGGTACATCAGTCAGCATTTCCTCCATCTGCTCTTTGCCTCAAGTAAAAGGCAACTGCAGAGGGCTATTTGACATGTGGCACTACAACTCTACCAATGATAGATGCTCACTCTTCACATATGGAGGATGCGGCGGCAACGAAAACCGGTTTGAGAACTGCACGCACTGCATGGATTCATGTAGCGCAAACGAAAATCTAACAGAAATTTGCCGACTGCTTGAAAAAGAGGCTGATGAAGAGTACAATTCGGGATGGGAAGATTATACAGGCGGTGATGGCTACAC |
| >MG12026313  TCTTGCGCCGCCAGCATGAAGTCCCTGGTCTGCCTGCTCCTCATCTCCCTTTCACTCGTGTGCCATGCGGCGCACAAGAAAGCTTGCAACCTGAAGAAGGACCCCGGCAACTGCGAGGACGCCTCCACCAAATGGTACTACGACAGCAAGACCAACGCGTGCAAGCTGTTCGTGTACGGCGGATGCGACGGCAACGACAACCGCTTCGACACAGAGGCCAAGTGCAAGGCGGAATGCGTGCATCCCCGCAAGACGACCAGCGGATAGGGGCGGCAGCAGCAGAGCCAGCCTCACTGACGGCAGCGGCGATCCGGTTCGACACATTAAAGACTGCTTGCAC |
| >MG12028620  GTGTAGCCATCACCGCCTGTATTCTCCTCCCATCCGTAAAAGTACTCTTCGTCAGCCTCTTTTTCAAGCTTTAGGCATATTTCTGTTCGATTTTCGTTTGTGCTACATGACTCCATGCACAGCGTGCAGTTGTCAAATCGGTTTTCGTTGCCGCCGCATCCTCCATAATTGAAGGGTGAGCATATATCTTTGGTAGAGTTATAGTGCCACATCTCAGATATCCCTCTGCAGTTGCCTTTTTCTTGATGCAAAAAGCAGATGGAGGAAATGCTGACTGATGTACCGTACACGGTGGCCAGAATTAAAAGAAAATAAACGCATCTTTTGATTTCCATGACCACAGTCT |
| >MG12029782  TCTGGCCACCGTGTACGGTACACCACTCAACAATTCGTCCATCTGCTTTCTGCCTCAAGTAAAAGGCAGATGCAGAGGGCTGTTTGATATGTGGCACTATAACTCTACCAATGATATATGCTCACCCTTCACATATGGAGGATGCGACGGCAACCAAAACCGGTTTGATAACTGCACGCACTGCATGGAGTCATGTAGCACAAACGAAAATCGAACACAAATTTGCCAAAAGCTCGAACAAGAGGCTGACGAAGAGTACTATTCGGGATGGGACGATAATACAGGCGGTGGTGGCTACACCGCTCCGCCACGTGAGA |
| >MG12031297  ATCTCAGGTGTCGACGATGCTTCGACTGACTGTGCTGGCGGCTGTGCTTTTGGCCATCTCGTTCAATGGTGCCAATGCTCAGAGAAGGCCCAGATTTTGCAGCCTGCCGCCTAGTCCCGGAGTCTGCTTCGCCTACTTCCCCTCGTTCTATTATGACATTTCATCCGGGACGTGCCGAGAGTTCGTTTACGGCGGATGCCAAGGAAACCAGAATCGCTTTGTTTCATATGAGGAATGCCTCCGCGTCTGCGG |
| >MG12031323  ATGTTCAAGAAATGGTTTTACAACGCGACGTCGTACCGTTGCGAGGTATTTTACTACGGAGGCTGCAATGGAAACGGCAACAGGTTCAACAAATTTGCGGAATGCAGTAAGAAATGTCGAGACCCTGTTCTCGGAGTGTGTGCCCTGCCAGAGCCCAAACAGATTTGCCGAGCTGGATATAAGGGGTACCGGTTCAATCCGTTCAAGCAAAGATGCGTGCGTTACATCTACTGCGAGCACAATGAGAACCATTTCCCGACTGAGGAGGAGTGCCAGGCACAATGTGGAAGATTCGCCCAAGATTCATGCCTCCTCCCAAAGCACTCGGGTCGAAACTGCAGCAGGTCGAGCCGCATGCAGAACTTCTGGTTCAACAGTCAAACAAAAGCCTGCGAACAGTTTGACTACGAAGGCTGCGGAGGAAATGGAAACAATTTTCTCTACGAATCCGAATGCTGGAAAACATGCGGCAAATACGTCGAAAGCAATTGCAGTTACCCAATACATCGCGGGAAGGAGTGCCCAAATGGTTCACGTAAGGTAGCATTTGGGTACAACAACAAGAACAAAAGGTGTGAAAGGTTCGTCTACTACGGCTGCGGTGGCTACCCCAACAGGTTCGACTCCGCAAGGGAGTGCTGGAACACTTGTGGCC |
| >MG12041908  CTCGTGCAAGACACCCCGTCTGAAGCACTCCTTCCGTATTTTTGCAGGTGCAAGCATCGCTGCAAATACCTGGTGGAGGCCATCCTACGTGTGTCGCCTGCCGAGGAAAGACGGACCATGCGCCAATGTGATTCCTCGCTTCTATTTTCACTGGAGGACTGGCTGTCGTAGATTCTCCTACAGTGGCTGCTATGGAAACGCCAACAACTTCGCGACCGAGGAACAGTGCTTGAGAAAGTGTGGTCATTTCATACGGAGAAAAGATCCTGATCATACTAAAAACGGCACTCGTTGCAACGGAGAAGTGGGGCCTAAACAGTCTTTCTGCAGACTGCCACCGGTAGATGGACCATGAGCGACGGTGTACTCCCACTTTTACTTCGACACAAAAAAAGGGTGCACGAGATTTTCTTACAGCGGTTGCGAAGGGAACAGTAACAACTTTAAAACTGAA |
| >MG12042158  CCAGCATCCTCACTCGTGAGAAACGCACCCACCCGGACCGCCCGTTCGCAAGATGCCTCGTTCGCTTGTGGTCGTCGCCTTGCTGCTGGCTATTGTCGTAGCAGGCACCAATGCTCAAGGCCAGTGCTTCCTTCCTCCTAGCCAGGGACCCTGCCGCGGCGCCTTCCCAGCCTTCTACTTCGATAGTAGATCGGGAAGATGCCTCTCGTTCACCTACGGCGGATGCGGTGGCAACGCGAACAGATTCAACTCTGTTGGACAATGCCGCAGAGTCTGCGGCCGATATTAGGGCTCGTACGCTCAAAGAAGCGCCAGCTCGAAATGCACTGCCGCAGGCGACGCGCGTGTTTTGACCAAAAAACAGGCTGAGC |
| >MG12048021  TTCCAGTTTATTTTTTATTAAGTCACATAAGTCACAAAGACTATTGAGCCAGAGTATCAGAACCAGTTCCTCCTTAAACCATTTTCACATGTGTTCCCAATTTCGTTTAATGCATTATCAAATAAAAGGATCAAAAAGATTATCTCAAGAAAGAAAAGAATCGGCCACAAGCGCACTGAGCAAGTAAGCTAGCCATATAATTATNNNNNNNNNNNNNNNNNNAAAAATTCACAAGATGAGGCAATTAGCCTGCTATAAACTCTGTTCTCTTTTTGTGCTCTATTTTCTGCTGCCGTCTCATGTTCAAGCGCGAGGGAGAATGCCCATGGACGTCTTGCATTTGTTTTCGCAGTCGGCGTAGCTGTAGAAGTTGTTCGCATTCCCTCCACATCCTCCGTAGATGAACTGCTCGCAGGACTGGGTCGTCGCGTTGTAGTAGAAACGTGGCATGTAAG |

| >MG96664  CTTACTGTCTGTAAAAATGCGTGAATGAACACATTAAACACCTCATGTGTGTTTAAACAGTGCTCTGGCTCAGGAAACGGGCATACCCTGGGGTATTTTCTTTTTATTTCTCTGAAAAATTACAACAGCCGTTCTCTCATGTCTTGCAGGCTTTGTGTTCGCCCAATGCATGTGCGGAATGAGCAGGGCAAAAGAGAGAGCGAAGTGTTCATTTCATCTGTCCAGCGGGCTACGTAAGCTTGCAGGCAATCTCGCACTCTTCCTTCGACTCGTAGTTGTTGTCGTTTCCACCGCAGCCACTGTAGAGGAACGTCTCGCACTGACCCGAGGTCACGTTGAAGAACCAGT |
| --- |
| >MG96665  GGCTTTCGCGCAGCGAAACGCCATCTGCCGGCTGCCGCCAGACGAAGGCATCTGCCGGGCAAGCATTCCGCGGTTCTACTTCAACCCAGCTGAAGGAAAATGCTCCTTCTTTATCTACGGTGGTTGTGAAGGAAACGAAAACAACTTCGAAACTATCGAGGAATGCGAAAAAACATGTGGTGAACCAGAGAAGCCCAGTGACTTCGAGGGAGCTGACTTCGAGACTGGTTGCGCGCCAAAACCGCAACGNNNNNNNNNNNNNNNNNNNNNNNNNNNNGCCCGCTGGACAAATGAAATGAAGACTTCACTCTCTCTTTTGCCCTGCTCTATCCGCACATGCATTGGGCGAACACAAAGCCTGCAAGACATGAGAGAACGGCTGTTGTAATTTTTCAGAGAAATAAAGAGAAACTATCCCAGGGTATGCCTGTATCCTGAGCCAGAGCACTGCTTAAACACACATGAGGTGTTTAATGTGATCATTCACGCATTTTTGCAGACATTATTGAAGAAGCAGT |
| >MG96681  GTTCAATACGGGGGGGTTTAGTGCCGACTGTTCCTTCTCAGTATGAAGGCCTACTTGTTCCTTGCTTTCATCGGCGCCGCTTGTGCGGCTACAAATGTTGACAAACAGTGCACTGCAAAAGCGGAAAAGGGACTTTGCAAGGCTAAGCTTCCAAGGTGGTGGTTCAATACGGATTCTGGCAAGTGCGAGCTCTTCTACTACGGAGGCTGCGGTGGCAACCAGAACAGATACCTCTACAAGGAGGACTGCGAAAAGACATGCGCTCCGAAAACACTGAACGAAACGCCTCTCACCACATTCAGCAACAAGAAGGCCAACTTCGATGACAAGAAGGGGCGGCTTCCCGGATCCGGCGTAGGCGTATGCATGGAGCCGCCATACACTGGGCCTTGCAAGGCGAGCTTCCTCCGATTCTACTACGACGCCAGCAGCAATACTTGCCGCCAGTTCACCTACGGCGGCTGCCGCAGCAACGGCAACAACTTTAAAGCACAACGTGATTGCATGAGGGCTTGCGGAGGTCGACGGCGCGGAGGTCTTCGGCCGCGCTAAGAAGAGCTCGAAGAATAATTCGTCGTGCCATTCTTTACACTATATGTATTTTTGCGAGTAGCGCTAAAGGGAACAGGGAAGAAAAAGAATCTAATGTAACGTAGTGATTCCGTACTTCTTAATTGTTGTTGTTAGCCCATCCTTTGATACGCGAACAACAACAACATCTTAATTATAAGATTGCGCTGCTGCTTTGCTGCTTTTGCTTCCGTTAATAAAGATAATCTTTCAAGCGTTTTGTAGGGTTTTATGACTAAGTTAACATTTCATAAGTGCGAAAAAATATCATTTCAAACGATACCACGCGTAAGTCTGCTCCAAATATTTCCTGACTCTTAGAAACAATCACAACGCTTAGAGCACTGAGTGTCCTGCTCTAAACTCTGATAACTGCACAACTTGGCACAAGTCGAAAAAACCTGGGACACTAAATGCAACTGCACAGCTCTGAAACAAAATATTTGGTTTTTAAGGCATCCGCGCGGGCAGCAAATAAGGCGGCAAGTTACTTCAGGCGCAAGTAAGGGAGCGTTGCATTTCGTATATATTTCATCACGTATCTCTGT |
| >MG962085  GAAGATTCGAACGCTTTATTTTCCCTCAACAGACGTAATTTTTTTTCCTTGACACCACGCTTCGTATAAAAACTCCCTCATTTCCCTATGAATGGAATCCTTAGTGACAGCTGGGGCCACGACGGTTTTTTGTGATGCTGGTTTGATCCCAGGTTGTTTTCTTCGAGAAATTTTTTAGTTTGCTTCTTGCATATTTTTCTTGCGTTGTTATTTCCGCTGCATCGTTTCATGCACTTGAGACAAGTTTCAAACCTATTGGCGTTTCCTTTGCAACCACCGTAAATGAGGCCACGACATTCGTCCAATTCGGGGTCGTAGTACCATAATGGAATAGCGGCCTTGCATTTACCAGTTTTCGGCTTGAGGCTACATACCTCCTTGCGCTTAGATTTGCGTAAGCAGGATTTCTGACATTCTTCTTCGGTTGGAAACCTGTTCGAATTCCCGCCACATCCGCTGTAGAGGAACCCCTTGCAACGGAAGCTCCAAAAATCATAGTACCAGGCCGGAACCTTATTTTGACAGTTCCCCACAGCCTTCGGTCGGAAACAACGCGACCACTTTATACCTTCAGCTTGCCTGAGTACCAAAGTAAAGACGAAAGCACCTATGAAAGCTTGTGGTCTCATATTGCAAGCTGCGGCACTGAGGTAACTGAAGCCAAACAGCAACCGTTGGCCACTTCTTT |
| >MG962401  AAGACGCCAACCTGCTTCTTATTCGCCCTGCTCTGTCTCACATTAGTATTAGGGCAAGGGTACAAACCGCCAAGATATTGCAAGGCAGAACCAAATGACGGACAATGCGGTAATGTGCGGCCTTCAATTGAAAGATGGTACTTTGATGACAGATACGGGTACTGCGGTCCCTTCTTGTGGGGCGGATGCGGCGGAAATAAAAACAACTTTCCCAACTGTACAGCCTGCATGACTACTTGTACGACTCACCCAGATCCTGAAGGCGCCTGTCGCTATATTATCAACTCCCCGTGATCAAGCAAGAACACCTAAAAAAGAAATTTCCCTGTCCTGCTCTTCATCATTGCGGCACTGTTTGTGC |
| >MG962579  GGCGAGTGCGAGGCGTTCCTCTACAGTGGCTGCGGTGGAAACGACAACAACTACGAGTCGAAGGAAGAGTGCGAGATTGCCTGCAAGCTTACGTAGCCCGCTGGACAAATGAAATGAGCAATTCGCTCTCTCTTTTGCCCTCCTCATTCCGCACATGCATTAGGCGAACACAAAGCCTGCAGGACATGAGAGAACGGCTGTTGT |
| >MG963002  CAGGTATAAAATGGAGGCATGCCTGGGGCGTTTCAGTACGTCTTCCCTAGGAGGTCTAGGGTGATAGTGTGAAGCTCATTTAAACTTCAGCTCCGAGGTAACTACCGCAGAGCCTGTCAGGTGCCGCTTTCGACTCAAGAACCCGAGCTGCTCATCATGGTACAAAGTCTTTTCACTGCAATCTTCATATTCATCCTAGCAGGAATCTGCGCTGCCAGTAAAACTGTGCCGCCCAGCGAAGACTGCATGCAAGAGCCGGATCCCGGAATATGCAGAGGAATGTTCAAGAAATGGTTTTACAACGCGACGTCGTACCGTTGCGAGGTATTTTACTACGGAGGCTGCAATGGAAACGGCAACAGGTTCAACAAATTTGCGGAATGCAGTAAGAAATGTCGAGACCCTGTTCTCGGAGTGTGTGCCCTGCCAGAGCCCAAACAGATTTGCCGAGCTGGATATAAAGGGTACCGGTTCGATCCTTTCAAGCAAAGATGCGTGCGTTACATCTACTGCGAGCACAATGAGAACCATTTCCAGACTGAAAAGGAGTGCCAGGCGCAATGTGGAAAATTCGCCCAAGATTCATGCCTCCTTCCAAAGCACGCGGGTCGAAACTGCAGCAGGTCGAGCCGCATGCAGAACTTCTGGTTCAACAGTAAAACAAAAGTCTGCGAACAGTTTGACTACGAAGGCTGCGGAGGAAATGGAAACAATTTTCTCTACGAATCCGAATGCTGGAAAACATGCGGCAAATACGTCGAAAGCAATTGCAGTTACCCAATACATCGCGGAAAGAAGTGCCCAAATGGTACACGTAAGGTAGCATTTGGATACAACAACAAGAACAAAAGGTGTGAAAGGTTCGTCTACTACGGCTGCGGTGGCTACCCCAACAGGTTCGACTCCGCAAGGGAGTGCTGGAACACTTGTGGCCAGAACTCTGGGAGCAAGTGCGTAGAACCGGGTCCCAAGAATAGGTTTGGAATTGTGAAAAAATACTACTACGACGTCGAGAGCGACAGCTGCAAGACTTCGCGATACTCTCCGTTCTCAAGCAAGAAGAACAGGTTCAACACTAAGGATGACTGTGAGGCGACATGCAAAGCTAACTACACCACTAGCATCGTAAGCTTCTGATGCCACCGGAGCAGTCATCAATGGCGAGCTTCCAGACGAATGGCTTCGCTCGTCATCGGTCATCGTGGCTGATGCGGACTTGGAGAGCATGTTGAAAGACAATCGCATTCGAGCAGAATGCACCTCCAGACACTGGTTCCATTTTATAATATGATGTGAGCATTGCAGTCAACCTGTGCTGCAGGCTACGACCCCAATGAAATGAAATGTGTAATAAAGTTTGTTGATTGAGAAAAAAA |
| >MG963314  TTTTTTAATAGGTAAGAAAAAATTTTATTTTTTGTGTCCACGTTTCCATCATACAAAATATTGACGTCAGCAGCCTCGGGGAATGCGTTTGTGGGGAAAGCAGACTTCCAGGCATTCTTCCTTCGAGTCAAACAAGTTTGGTCCGCCGTTGCAGCCGCCGTATAAGAACTGAAAGCAATTCTTGTGGTAGTGGTCGTAGTACCACTTGAAAGCAAATGCGTAACATGGTCCACGGTTTGGCCAGTGACGACAGTCCTCCGGGGATCTTCGTCGTTCGCAGACCTTGCGGCACTCCGGCATGCTGGGGAAGTTGTTACCGCGATTGTGACAGCCCGATCTTTTCTCGCATAATTTCGTGTCCTGGTTGTAGGAGTACACGCTGCGCTCCTTCTCGGAGCAAGAATGGGGTAGGGGGGGTTGGCTGCACACATCTCGGCTCCTGCACACTTCACCGCAAAGCTGCCTTGAGGAAAAGGAGTTTGCCCGCGCAAGGTGACAGCCAACACCCAGGCGGCATTCCCTGGTGTGTCTGTCGTAGTACCACATGTGCCTTGACGGCGCGTAGTTGGCACAAGCAGGGTCCTCGGTTGGTCTGGGAAAGTGGCAGAAGGACTCTGACGGGCATGTCTTTTCGCAGGTGCTCCTTTTCACGAAGCTGTTGCCAGCCTTAGGGCAGACGAACCGGCGTTCGCACCTCTTGGCGTCCAAATTGTAGACCCACCTCTCCATAAGGCGTCCACAGCCGTGGGTTGGCGGGAAGGAGTAGCACACGGGACTCGGCATTGGTACCGGCGAGACTCGTCTTACGCAGACCCTGCGGCATTCTTGTAGGCTTGGGAAGTTATTCCCCCTGTTATGGCAGTTAACGTCTTTCAGGCACTGGCCGGAGGTTGGGTCGTAGTACCACACGGTCCTCTGTCCGCTGTCGCACAGTTCCAAAGGTCTCGGTGCATCACACACGTCGACTGCTCTGCACATCGAGACGCATTCTGATCTTGTGACGAAGGCATTGGCTCCGATCACACAATTGGCCGATTTTTTGCACCGATGAGTTTTTGCGTCGAAGAACCATTGGCTTTGTGCGGTGGTATTGGAGCAGTGTTGATCAACTTTCGGGAAGAGGCAGAAAGAATACGCCGGGCAAGCGTACTCGCAGTCCTCCCTGCTGTGGAAGTTGTTTCGATCCTCGGCGCAGGTCTGGCTTCTTAGGCACGACTTGGTGAGCATGTCGAAGTGCCACCTCTGTCCGGCATTTGCCACCCGGCACGGGTGGTAGCCCACCGCGTCATAGCACTTCGACTGAGGGTTCTTTGGTCCCCAAAACGCATCCTCGTACTCGCGTTGGCCGCTCGCGAGTCCCGCGAGCAGAAGGCACAGCATCCAGGGCACGGCGGCGTTCTTCATGTTCTTCCCTCTCGTGCGGTGGTCCCGCCCCGCGCACTCAACGCCGTCTGGCCGATGGGAACGCTCTTCTCAGACCGCCAACACGCGGCACGCA |
| >MG965806  ACGAGCGTTAGCTGTTTGGTTTGCTAGAATTGATATCAATGAGGTTAAAGTGAGGTACACCAATGGAAATACGAATTTGACATCTTGGTTCTATAAGTGACGTTACCGATCAATCACTAATTGGATAAATATGTGACGTCACCAATCAATCACCAGATCGGTATACTCATGACGTCACCAATCACCAATAGGGTTGATATGTCGATTTACTATGGGGGCTCCCGTCTTGAATTCCTCGGACTTCCTAGAAACAATGTCCCCCCTGATTATTTTCGTTTTGTTTTAAATGCCCTTGTGCTATTTGCTTTCAACCAGTTTTTGTGCCTTCCGTCGTTGAATGTCATTTAATGGTACTTGAGAGCAGTCAAGCTGTTCCTAACAACTTTTAGCCTTTAAATCTGTCCGCGACATGTTCTTTTTTCTGGAAAATAAACTGAACTGAATTGAGTTGAATTGAATGTCTCTGTCCTTTGCGCTGCGTTTGCACCATGGAGCACAAACTATCCCATCAACTCGCTCTCGAAAAAAGAAATAAAAGTAAACATGAACCTTAAAAACGCACACACCCACACTTGAGTTTTCAGCTCTTTACCGCGCGGCATGCAATGCAAATATTTTACTGCCGATGATTTTAGATGGTTGATATAGGTTTCTTTTCAGTTGTTCTTGTTTAAAAATCTATTCCACTTCTTTATAGCTCGCCCCGTATCATATGCTTTTTGTACCCGTTCCGACGAACAGCATTAAAAGCCATAAAAGTTTGTTGCATGCTCAAGCACTTCCCGTTAATGCGTTGATATAATCTTCGTGAATGACTGGGCAGGAAAGTGAGATAAGTATGGACTTTGTAGGAATTAATATTTATGCTATGCACCAACAAGGTACACCACCTGTGATGCCATCTCATGGAAGCTTTTTGGTGTGGTTTGCTATTTTGACCAAATGCCCAACAAGAATTATTGGTGGACCTTCCCTAACAAAGAGCAGAATGACCATGGTAGGCACGCTCAGTCGGCGATGAAGCAGCTGCACGCCCCCTTCAAAGCCGGCAGTGGGGACGGCAGCTTCTCGCAAAACGATGGCAAAGATGCCACCTGTTATGAAATTGCCATTCATTGCTGACCATAATGCGCAACTCTTGCACAGAAATGTCCTTGCTAATGTGGTACTTTCTTTCTCCGCTAAGTTCTGCTTGAAAATTGTGCCTTCGTGATCCGCGAGTTAACCTCGAACACAGCAAATGACAACGTCGTAGTTTAACGCACGGCACGCGCCAACAACTGCTCATGTAGGCGGACAAGCTGTAGTCACGTGCTCTTTCGCACCTGTAAACCAGAATCAAAATAAATGCAGTTAAAACTCGATTCAACTAAGCGAGAAGCAGCCGTTAAAAATTTCGTTAAATCGGGAAGTTCGTAAAATCGAAGACGGCATTTTTACGGCCTTTGAGATCTTAAATTCTAACGTAGCGGCACTGAAAAGCTACCGAGGCGTCGCATTTGCCGACAGCCCACGCCTTCGCGTATGAAAAGTCCGCGAAAGCGGCCCGAATCGCTCGTGCATAAAAGCACCATATGACAAACCGGATTCGAGGCCAAGGTGGAAAGCACCGCCGACTGCCGAGTCCTATGTTCCGTTCAGCAGCGCAGCATGCAGCCCCGTCAGAAGAAAACCAGGGTCGTGAGTTGGGCGCGGAGATGTACTGCTGGCACGCTTCCAACAAGCTGCGAGACATCGTCGTTTATTCCCGGAAAAACTTCATTAAATAGGGTTTAGTGATCGTTTCTTTCGTTATTTCAGGTTATTCGAACATTGAACCCTATGAATACTTGCCGGGGTATAAAATTTTTTTCGTTAGATCGAGAACTTCACAAAATCGGCTTTCGTTAAATCGAGTTTTAACTGGTGCCGTAGTTACTGATGATGAGGCTGGCTGGACCGTTCGCACCTATCATTATAGGGGGAAGGAGGGTATTGTAAGAGAGTTAAAAAATTTACTTTGAGGAGCTGCTACCCTCACACGATGCCAGCTCACGTGTGACATAGCAATTCACTTCTTGAATGCGGAGGTTTTTGATATGTGCTCGTTTGCCCGGTATGTCACCAGCACTTTCAAGCATTCCTAATTATAGGGTGTTTCAAACACAGAGGAAAACTGTTCAGGAACACACAATCGGCCGCAGTGGAGGTGAGTGACTTTACAACTATAGTAATCAAATCGCTACTTTAATGAGATTTAATAAATTATTTTGGAATGAACAGAGTCTGGTCGTTAGCATAACAAAAATGTAACATGAAAACAGACCAATGCTGGCAAGATAATCCTAGGAACCATAGACACGGTACTTCGAACTGCTTTTGCTAATACACCACGGAAAGTGCTCTTAAGCTTCGTCGCAATGTGCAAGAAAATGAATACTCATCGTAAACTACGGGGCTGCCAGTAGTGAGAGCTAATTATTTAGCAAGAAAAAAGCATATTTTCAAAATAATTCAGAACTGCCGCGGATAGCAGTCTCTTTTGAGACCACCCTATTTTGAGCAATTTTGGAAGGGCTCGCCGAAAAACGCTGTTAAATATAAGCTTCATTGGACCACGGCATGCACTTCATTTTGCTTCGCCCCTCTAAAAAATAACAGATACGCGGGTGGCTACGACTATAATTTTTTTGGACCAAGTGGCAAAGCAAACAAAAGTGTATGCCGTGGTGCAAAATGTGCGGGCAGTTAGAATTGGGCGCCGTACACTCTAAACGCAGAAAGAATAAAACAGGTGTACGCTTTCCTCTAGCGCACTTTTTTTGTGAAAGGGAGTGCACTTTAGGACAGATTACTTCATTTTCACTGCAATAGCGGTGTATTTGTGTTGAGACTGTGGGCCCTGCACCGTGTCACCAGCGGTCGTTGTGTTTGCACTGGGCTCCCTGCACACCTCATGCACTCAGTTTCGGTATTGTGCGAAAAAGAGCCTCGCATTTACTCGCACTGCACGGTTTTACAGAACATTTTCAGAAACTGATAAGGCATCGTCTTTCCCTTCGCTTCCAGCTCTCAATTAGCACTAGTCACCACACCGTTTTAACCAAAACGCTAATCTTAAAGCGTACTTTGTCATATGCCTTACTAGTGCGAATCGTTTAGTAACTGACGTCCGCCGAGGGTGCGAATGTATTTATACGGAGATTTTTCCTCTTTAACCATAAGATTAAGAATTTTTGAAAGTGCTTACTGAAACCACAGAGATAATTTCTTCGTTTTGTGTCACTAAAAGTAAAAGGCATTGAATGGCCTGGTAGAACACCACGAATGCGCCAGACACACTATTGTCTGGGACAGAATTACAATGATGGCTATCGAAAAGCCATTCTTATCATGTCTGCACCTGATGTCATTTCATATCCACACAACCAATCGCGCACAGACACGTGAGCCCTTGCCCGAAACATACTCATGATCACTGTGGCGCTGATCGCGCATATCAAGGCGGCTGTCCAGTCATTCCTTTTACATGTGAACATTGCGCTTGTAAGGGTCTCGAAAAGCCCCCGCCTTTGTATTTTACCCTTCTGTTTGGCACTCGTCGATTTCTCCTCCTGCAATGTCTTTTCCTGGCCAAACGGGCTGTCGAACCTTGGACTAGTAGGCGATAGTATGCGCTCTTGGCGAACGGCTGTCGTCGACCGACATTCCCCGTGCTAGGGTAATGACAGCAACCAGGCATTCACGTCACCGATTTGCTTGTGCGACCTAAAAACCTCGTGTTGCTCCTTCCCCGTGCCACCATGGAACCTCGGTTGTTCCAAATTCTTTTCGCGCGCACTATGCCTAACGCCACTGCGTCCCTATCGTTAGTTGCGGCGCATGTGCCAAGACTACGAGCGGCACCGCACACAATCGCGCACATCCTTACAATTGCTGTGCCGCGCTGCCCGTCGGCTCGGCTCTATCTGTGGCAGGCTCACGCTAAGTGGAGGAGCGCGTCGAGGCAGATGCGCCGTGGTTGAACTTGCCGATAGTCTCCACGCCGCCAGAGGCATTTGTTGCGTTAGAAAATTTTGTGCAGAGGGCAAGATTGCCTGGCTGCAGTTATCCAGTGTTGCTGAAAAAGACGCTGCTTGTAAGCAAATTCCGTTGACTGCCACAAAGACAATGCATATTTTAGAGATTGGGCGCACCGATGGAATGCGACAGAAAGTTCATATCGCCAGGGACACAAATTGCATTCGTGGACGTCCTTGGCAGTTACTGCCAGTGCGCGCAGATTTCACTACACACCGCGTGCGCCTGTGATCACGCATGCGCTAATCGAGAATCTGGGAACAAAAAAGAAATATTTCCGTCCTCCTCGTCGCTTCTTCAGAGAGTGCAGAGCGACGCTGTCACCGATGCACCAGTGAGGAACACTAGAGCGTAGAGGGCGGCCCCGTTGCTACGCGGGGCTGCGTTCCCGCACTCGCATGACCCGCACTTGGCCTCCTGCTCATCACCGCAGCTGCACTGCATGGACTCCGGGCAGCTCGCAGCCGTCCTGGCCAAGTTGATGCGCACACCAAAGCAGGTCATGTTGCATCCGTCTACGGTACGGAAGTTGTTGTCGTTTCCACCACATCCCTGGTAGTTGAACCGCTCGCACAGTTCTTTCTGCACGTCGTAGTAGAAGCGGATCTCTGGTGTAGTGGCCCCTGGGCCACTGCTGTCGGTGTCGCAGTGGCCTTCGTCCACCGGCTGGTGACACATTTCCCTGTCCTTTCCGGCTACGCCGTCCTCAGCAACATGCACCCTGTCCTTTCCGGCTGCGCCGTCCTCAGCAACATGCAATCGGCAGCGTCCGTTGCACTGGGTGCTGGTGTAAAAGTTGTTTCCGTTGCCCATGCAGCCACCGAAGATGAAAGGCAGGCAGCGATCCTCCTGCTTATTGTAGTAGAAGCGCCGGAAGTACGCGTAGCATGGGCCCGGGTCTTGGGGCTGCGAGCAAACATCTTCGCAAAGCGTCTTGCACGCGTCGAGGTCTGCAAAGTTGTTCTTGTTCCCTTCGCAGCCGCCGTACTCGAAGGTCTCACACAATCCCGTCTGTGAGTTGAAGTAGTAGCGCTCCTGCGTGTCGTTGCAGCTGCCAGGATCCATTGGCAGGCGGCAGGGATCTTCACACTGGGCAATGCACTCTTCATGGGAGGCGAAGCTGTTCTCGTTCCCACCACAGCCGGTGTAGTAGAACACGTCGCAAACGCCGGAAGTGACGTTGAAGAAGTAGCGGGGAATCCTTTCATTGCAGTTGCCTTCTTTCACCGGCAACTTGCACGCACCATCAGACGGACAGTGACAGCTCTGGGCGCCGCTGCTCGAAATTCGCTTCACGCAAGTCACTGGGCAGGGCACTGCGATTTGGGCATCCACGTACTCCAGTGCATATTCAACAGTGCCGTCTGCAGCAGCACATTCGCAGGCGTAGCACGACGCAGACACCTCCCTACTCTGACAACCAGCCGGGCACAGCTCGGGAGCCGCGAGGCAGTCAGCTTTCTTGAGAAGGGGCTCTGTTTCGGACGCGAGGCAATTGCACGCAATACAGCTGTCTTTCTCTCGCCGCTGGCAGCAGTGAGCGCAGGCTAGGCCGCATGCTGCACGCTGGGGACACTGGGCTGATTCGGATTCCACAGGTGGAGCTCGAGCTTGCTCCAGTGGCTTCTCGCATCTTTCAACACATTCTTGGCGAGTAAGGAAGTGGTTTTCGTTGGCTCCACAACCGGTGAAAGTGAAGTTCAGGCAGCTGCGTTGCTCTCGGTCGTAGTAGAAGACTGTCTGCTTATTCGCACAGAAGCCTTCCTTTCGTTCCAGGAAGCATATTCCCGTGCCTGGTGGTATTTCGGCCAAGGTGGGATCTTCATCGGACGGTAGAGTGCATGCTTCGTCAAACTCGTGCGGTTCCAGCTGTGCGACAGCAACGCAGACGTTCTCCAAGTGGCGCGTGATCTCCAGAGAGGTGTCTCGTTCGAGCACGTGCCTCGCAAGGACGCTGACGTGTGTCGCATCCGGGGCACACACCGACGCGTGGGGCTGCTCATGCGCGCACCGGTGCAGCACGGCGTAGCTCCCGTAGTCCGTGGCTACCACGGCCAGCGTCTCTTGTACAGGCTGCCCAAAAAAGTCGTACTCGAGCTGAAATTGTCCGACGGGCAGCACTTTGTGGACTTTGCCGACAATGGCCGGCAGGCATGTCTCATTGCCTGCAAGTGCTGCGCTAAAGGCGAACGTGTTGGTGTCGAAGTCAAAGCGCCAAGAGCCTTTCTGCAGGAGAACCGGAGCGTGCTTGCTCTTCAACACCTCTCGCCAGTCTCCCGAGAACTTCTTGTAATCGAGGTCCTCGAGGGCGCTGGCCGGCTTGGGGACACACACCAGGGCCTCTGCCGGTGTCGTCGGCGCGGGCAGTATTCCAAGGCCCGTCAGCGAGGTGACAAGGAACCACGACAAAGCAGCCGCAGAGGCCATGGCGTCTCAGCCGCTGCCTGCTTCGCTC |
| >MG966001  GGGCGAGTGTCTAACATGAATATCTACCAAGTTTTAGGCTTCTTCTTTCTTCTGCCAGTGTGCATGGCCGTGCATCACAGCAGCAGCTTGAAAGACCCAGCCGTGTGCACTGCCCCACGCCCTGCCAGCTATTGCTATGGTGGAGCCTTTGAGGTCTTTTATTTTGAACCCTTGAGTAACTCATGTTTAAAAGAATTAGGTTGTACTCTGCACGGCAATAACTTTAAAAGCAGAGAAGAATGTGAAAGACTCTGCTTAAGAGGAACCGCGCAACCACAGCCACGCCCGATGGCCTTATTTTATCCTTGGGTTTGGCTCTAAGGTTTATCATCACCCAGTGCAGCGACAAAGGGAGCACTCTTGATTTGAAGAATTTCACCTTGAAGAATAAAACCCACCGGGCAATGGGCATTGATGAATCAGTGGTTATACTTTTGGTGCACGTCTTCTGTGGAAATCCTCAAAAATAAAAAAAGGCTAACATTATAAAAATTCCACTGACGCAGATATCTTTTAAATCGAGTCAAAATGCACCAATCTGGGTCGAGAACAGCGGGCTCTGCCATGGTTGCAGTTTAAATGTAAAGGAGAGAAACTTACCACTTCATTTTAGCCATATATAGCTGCTCAAAATTTGCCTGACAAAAAATTTTAGTAACATTATTTAGAAAGTCTTTAGACAAGTGTTAATAGAATAC |
| >MG966072  CTTTTTCAATAGTTTATTTCTTGTTACCGAGAATAAGAAAAATGCGTAGTAGCAAAATATTTTCAAATTTCCTCGCTACTCTGCAGCACTGTATGTCTTCATATCAATTGAATCCCTCTTGACGAAAACGTTTACTTCTTATTTTCCTGCTTTGCAAGTGCGCCGACATTCGCCTAAGGTTGGAAAGTTGTTTCCTGTGTAGCTGCAGCCCATATCGCTGTGACACCTGCCGGTTTTAGGACTAAAATAGTAAACTGGAAATATCCTCCCGCGGCAAAGGGCAGACGGTCTCGGAGCAGTGCAGACGGCAACACTTCGACAGGTAGAATCGCATTCTAGTTTGGTTTCAAAAGGAGCTCGGCCACATGTCCATGTGCACTGCTTGGTAGAACGGCTGAATGCGTAGCTGCGGTGACGGCAATCATTTTGCAGTGGCGGGCGGCTAGATTTGCACCGCGGATCGTGTGGGTTTGGCCGCCTGGCACTACAAAGTGCCAATAG |
| >MG966825  CCCACTTGTGGCCATCATGAAGGTCTTCATTTTGCTTGCCGTGGTCAGCGCCGCTTTCGCAGCGACTGCGTTTGACATGAGATGCACCCAGCTGCCCGATAGCGGTCCCTGCAAGGCGATGATGCCGATGTGGTGGTTCAATGCCAGGACTGGAAAGTGCGAGCTTTTCAACTACGGCGGCTGCGGAGGAAACGAGAACCGATACGAGAGCAAGCGACAGTGTGAAATGACGTGTTCTGCGAGTAAACCTTCTTTCCGGCCCGAAAGGCATTGGGGAGTGCGCAGTGGACTGTTCGCATCCGTAGCTGATGTGTGCCGTCGCCCTCCATACTCCGGCCCTTGCATGGCTAGCATCCCGCGCTTCTACTACGATGCTCCAACAAAGCAATGCCGGCAGTTCATCTACGGAGGCTGCCACAGCAACGGGAACAACTTCGAGACCCTGCGCCAGTGCATGGACGCCTGCGCCATTCAGTCTCCTTGGGAGCCGATGCCCCGGCACGCATAGACGTACATGTTGCACTTACTGCTTTTCGAAAAAGAAGGTGCTAAACATTCTTAGACCTAAAAGATTATAACACATATGCGTACACATTCAATTTAAAAATAACTGAAAAATTTTGTGAGATCTCGGGAAATTCATGCAGGATATAACTAGTGTAAGAGGCAGATTAATAAAGCTGATTTACTCAGCCTCTTGTTCGTTTGCGATGTATTTGGCTTGACACTCTTCGGAATGGGCTCTCCCCCATGTCTCCGCGTGAAGTAAATAATTTCGAGCAGACCATTTCTTTGCTTACATATTTTAACTGTTTTCCTC |
| >MG967997  TTTTTCTTTATTTAGGGCTCTGCGCAGCAACAAGCCGATCCACGAGAGATCCAAGATGCATCTCAAACAAACCAATCGTTAATACAAGACGATGCTCAACACCTTCATGGCAGTTCAACTTAATTGAAAAGAAGTGTAATGAGACGTGCAACAAGGACGGCCCGTTCGACAGCAAGCTTGCTTGCGATGGATACTGTCGTAGTGTTGACGTGTGCACGGCTCCACGTGCTGTGTCTTCCTGCGCCGGCGATGTACACCCTGTTTTCTACTATGATCCCTCGACACGCAGCTGCCTCAAGGACATGGGGTGCATATACTATGGAAATAACTTTCCCACCATTAAGGAATGCCAGGAAACGTGTATGAGACGTCGACCGAAGCCAACAATACCTCGGGAGTGTTTTGTGTCTCCGACTCATGGGTACCCTTGTCGATGGGGTTTCGTCTCAATACGTTTCTACTACAAACCTCGCACCGGCACGTGCATACCTTTTTGGTACTGGGGCTGCGGAGGGAGCGCAAATAGTTTCCCCTCCCGCAACTACTGCATCAAGCACTGCGCAAGAGACATGCCGGAGAGATAAAG |
| >MG9610040  CACCGTACCCCGGGATGCAGAAAGCAGACGCCACCAGTACGGCAACATGAAGGCTTACATTCTCCTCGCTGTTGTCAGCTCCGCATTGGCTATCTTCCAGCCAAAACAAGAAGCCACAGTGTTGCTCAGTGAGCCTGCGAAGAAAAAGCCTGATCTGTGTTTTCTCCCACCGTACCCCGGCCCGTGTGTGGCTTTCATCCCACGCTTCTACTACGAAGCTAAGACAAACCAATGCAAGTCGTTCCTCTATGGAGGCTGCCACGGCAACGGCAACAGCTTCGAGACGATGCGCGAGTGCTTCGCCGTCTGCGCCACCCTGGGGCCCGTGCCTATAATTCATTAGAGGCTGTTTCAAGGTGGAAACAGCAACTTGACATCACTCTAGAAGAACAACAAATAAAGCATGAGTGTTAGACAAAAGAAAAA |
| >MG9611087  TTTCTACTATGATCCCTCGACAAGCAGCTGCCTCAAGGACATGGGGTGCATATACTATGGAAATAACTTTCCCACGATTAAGGAATGCCAGAAAACGTGTATGAGACGTAGACCGAAGCCAACAAAACCTTGGAAGTGTTTAGTGTTTCCGACTCAAGGGTACCCTTGTCGATGGGGTTCCGGCTCAGTACGTTTCTACTACAAACCTCGCACCGGCCAGTGCATACCTTTTTGGTACTGGGGCTGCGGAGGGACCGCAAATAATTTCTCGTCCTACCGACACTGCATGAAACATTGTGCAAAGCACTAGTGTCTGCTTGAAGTATTTGATTCTTCCCACTGAAACATGGAGACATGCCGGAGAGATAAAG |
| >MG9612847  CGAAGTCCACAGCAGCTCCTGCTTTGCCGGGAAGAACGCCGGGCGACCCCTTGCTGCTGCGGTGGACGTCGCTTCGCTGGCAAAGCAGAAGCTGCTGTGGACTTGGTGTTTTGCGGGTTTTCTCTCGATCTGTGCGCGAGCGGATTGGTAATGGCAAGAAGGTGGCTGAGATATGGCGCTAGGAGTCGATTTAAACTTCTGGGAGTGAGGAACGGTACATACCTACTGGCCGTATTGCTGGTGTTATCGGCATGTCTCGCCACCGTCACGGCTCAGGCCAACGTCAGTTCCAACGTCACTGCCAACGTAGGCGCCAACGAGACACATGCCGAAGGTGACACCACCACCGAGATGCGTTCCAGACCTCGCGAAAGAACGGAGCCCAACTGTTTCGTGCCTCCAAATGCCGGAAGCTGCAACGGGAGGCTGTTCCGCTACTACTTCCACTGGGGCGTGGGACGGTGCATCCGCTACGTCTACACGGGTTGCGAGGGCACCGGAAACAATTTCAGAAGCAGACAGATGTGCATGAGGACTTGCTGGTTCCAAAACTACAACATCTACTGCAAGCTTCCAATGGACCGAGGCCAATGCGGCTCCTGGGTGGTGCGGTATCACTACGACAAAGAAAAGAACCACTGCTTTCCTTTTTGGTTTTCCGGCTGTGGCGGCAACCTCAACAGATTCCGCTCCGCCCAGGAATGCCGGGCGGTCTGCATCCCGGACTGACCGCAAGAGGGCGCCGCGGTCGCGGACAGGGCAGCTCACTGGACCNNNNNNNNNNNNNTCTATGGACTGTCTATAGACTTCTTATGGACTCTATTGCCTTCCTACATGTCCCTTTTTGTCTATTTATAGTCTATAGACTATAGACAGAAGTCTACTAA |
| >MG9615920  ATTTATTTATTAATACTGTAAACATATTTAGGCTCATACAGGGATGAGCTACTTCACGCACACAAATAAACAACATTGAATGGTTGCACCATGTTTGTTTCAATCGCATTTGGATGGTGCTCTGATTTTTGCTTAAACGCTTGCCTTGGGTTTTTGTTCCATAGGTTCGAAGTTGCTTCCGAAGTACATCAGCCAGATCCGCGCATGCAGGCCTTCTGGCATTCCCACTCGGTGTTGTACCGGTTGGCATTGCCACCGCATCCACCGTAGACGAAGGGCTTGCAGACACCGTCGGCCACGTCGAAGTACCAGCGCTCAAAGTCTGCCCTGCACATTCCGCTCTCAGGGGGAGGCCTGCAGCCCAAGTCAAAGTTCACGCCGCGGTGCTCATTTCGGACTTGCACCTCCGCCGTTGCTGCTGCGAAAACAGCGCCAGCCAGGAAGCAAAGAAGCACAGCCTTTTTCATGTTATTACAGTTGACAGGAAACCAGTGAAAGTGCGAAAACGCCCTCCGCGGCGCTCAT |
| >MG9616059  CTCCGCCAATATTTATTTGTTAGTATGCATTCGCAATTGTGTTGTTACACGAGAGCCTTCTATTTCGTATCTTCGGAAGATGATTGTACGTAGTATCAATATTGTGCCTTCTCTTCTGATGAATCGCCCAATAACATCAGCCGTGTGAAATTGAACCTGCCACATGCCTTCTGGCATTGATCCCGGGATTCAAAGTTGTTTAGGTTGCCGCCGCAGCCGCCGTAGAAAAATCCCTCGCACCATTGCCCGTTGTACCACCAACGAGGGATAAGAGCTCTACAAGGTCCTGGGACCGGGTCCTCCCTGCATATATCTGCGCACGTTTCGTTGTCCCGACCTTGACTGCATATTTCCGCCCACTTTTCTGCTTCGTCAGCGAAGGCCGCACCTCCTAGAGAAAGAAGAAGAGT |
| >MG9617976  ACTCTTCTTCTTTCTCTAGGAGATGTGGATCCTGGTCGCGGCAGAGCAATCGCGGCAGCGGTGACGCCGAACTCTTTGGATGCAAGTTTGGGACCATTGGCGAGGAATTTATCTCCAGGCGCAGGTGTAGCCAGCAGAGAGGACGCAGTTGATTGGGCTGAAATATGCAGTGGGGATAAGAAGAACGAAACGTGCGAAGATGTATGCAGGGAGGGCCCCGTCGAAGGACCTTGTAGAGCTCTTATCTCTCGTTGGTGGTACAACGGGCAATTTTGCGAGAAGTTTACGTACGGCGGCTGCAGCGGTGGCCGGAACAACTTCGAATCCCCGGAGCAATGCCAGAATGCATGTGGCAACTTCAGTTTCCCGCTGCTGGTGTTATTGGACGATTGAGCGTAACTCAAGCAAGAATATACATACCGTGGACAATAATCTCCTGATGATTCGAAATGGAAGGTTCACTTATAAAAACCAAATTGGGAAAGCATACGCAAC |
| >MG9618880  TTCTGCGTTTCTTTTGCTTTCAATTTTACAGCATGCTCCGGACCCTACGCTGTCGATGGCGTGGGCTGCGTGGCGACTGGAGGGGCGCAGAAATCCAGGCAGCCGCGTCGGGTCTCGAAGTTGTTTCCGTTGCTGTGGCAGCCACCGTAGACGAACGGCTTGCAGGAGTTGGTCGTGGCGTCATAGTAGAAGCGCGGTAAGAAGGCCAAGCAGTAACCAGTGTATGG |
| >MG9618881  CGACTTCACGGACGTCTTCATCAGTTTCCTCTATACTCGTGAAGTTCGACTTTAGTTATTTTATTTCTGCGTTTCTTTTGCTTTCACTTTCACAGCGTGCTCCGCACCCTACGCTGTCGATGGCGTGGGCTGTGTGGCGAGTGGAGGGGCGCAGAAATCCAGGCAGTCGCGTCGAGTCTCGAAGTTGTTTCCGTTGCTGTGGCAGCCACCGTAGACGAACGGCTTGCAGGAGTTGGTCGTGGCGTCGTAGTAGAAGCGCGGTAAGAAGNNNNNNNNNNNNNNNNNNNNNNNNNCCAAGCAGTAACCAGTGTATGGGGGCCTACGGCACACGTCTTCTACGGAGGGCTTTTCATCAGCGCAAGTCTTTTCGCATTCTTCCTTTGTCTCGTATCTGTTCTCGTTGCCACCGCAGCCGCCGTAGAAGAATTCCTCACACTGGTGAGTCCTCACGTTGAACCACCACCTCGGCAGTAAAGCTTTGCATAAACCAACTTCTGGGTCCTTCGTGCATTTCTCCTCGTAGAAGGTGGCCGCAAATGTAGTGCAGATGAGAGGGAATATGACACAGAGCTTCATGTTGAGGCTGATGATGCCGCGAGATGGCTGAAGCTTTCCTAGCACACCTCGATTTTTATACGCCAG |
| >MG9621991  CAGAAGGCATGCGCCGAAATTACAACCATTGCTTCATCAGTACCCATTGCCCAGTGGGGTTTAATCTTCAAGGCGAAATTCTTCAAATCCAAACTTCTCCCTCAGTCGCTGCAGTGGTTGATGACAAACTTTAGAAGTGAACCAAAGGAAAATGCAACGCCATCGGTTGTGCCGGTGGTTGCGCTCTTCCTCTTAAGCAGACTCTTTCGCATTCTTCTAGGCTTGCAAAGTTATTACCCATCAGAGTACAACCAACATCTTGAAAACATGATTGACTCCCAGGATAATAAAAAAAGACGGGAAAGACGTTTTCAGGTCTATCGCAGTAGCTGGCAGGGCGTGGAGCATAGCACACGGCAGGGTCTTTCGGGCTTATGCTGCTATGCATGGCCAAGCACATTGGTAGAAGAAAGAAGAAGCCTAAAGCTTGGTAGATATTCATG |
| >MG9624116  AATCAACGAAGCTCTCAGAGTCTTTGTTGCGTTTGGCTTCTGGGCAGCCATTATTCCCGGCCACGCACTTCTCAGCGACGATATCTGCAATCGTCCACGCGCAGTGCCCACCTGTCTGGACACCGCATTTGAAGTTTACTTTTATTCACCAGACACCGGAATGTGCCACAGCGATGTGAGCTGTACTCTCGAAGGCAACAACTTTCGCACGCTTCAAGAGTGCATGAGTGCTTGTGGAAGTAGTTCGCCGATGTCGACTGCACACACAGGGATGACGATAGCTGAGCCCTTGTACGCCATCTTTTATTGAAAATGAATTCCGGTTTGTGGTGTGCTTTTGACCTCGTTGGGCGTCGATGGAGCTCTTTCAGCGATATGAGG |
| >MG9628098  AACAACTCCATCGTCGTCCATCCCCAGCAATGACGTTTTACCTGGCCCCAACCCAGGTGTCAAGACCCAAACTACGGACCCACGCTGTCAACACGCGCCCAAGAAGAACGTTGCAGACGGCGTGCAAAAAGTGCAAAGGGCATGTGCCATAATGAAAAAGAGCCTAAACTGCGCTTTACATATAACCCTAGCACTGGAAAGTGCGACCATTTCTGGGATTTAAGCTGCGATGGACAAATTCTCAACAGTTTTGAGAATTTCACGGAATGTATGACAGCGTGCAACCCCGATTCAAAATGCCTGGCGACCCCTGACAAGC |
| >MG9628654  CCTTTGGCTTGATCAGGCACTTTCTGTAGTACTCACTTACAGGGTTGCACTTGGCCATGCATTCCTCTTCTGAGTTGTAGTTGTTGCGATTTCCTTCGCAGCCACCGTATGCGAACAAATTGCAAGTCGCCGACTCCGGTTCGAAGTTATAACGATAAATAGTTTTATCGCACGGTCCCTTGTCTGCCTCCACAAAGCAGTCCAGAAATGCTTGCGGGTCCGTTTCCTTTATGTCCGGATTGCTCGTTTGCGAACCTCCT |
| >MG9632571  GGAGGATGTGGAGGGAATGCGAACAACTTCTACAGCTACGCCGACTGCGAAAACAAATGCAAGACGTCCATGGGCATTCTCCCTCGCGCTTGAACATGAGACGGCAGCAGAAAATAGAGCACAAAAAGAGAACAGAGTTTATAGCAGGCTAATTGCCTCATCTTGTGAATTTTTAAGAGCTGTAACAAACAAATAATTATATGGCTAGCTTACTTGCTCAGTGCGCTTGTGGCCGATTCTTTTCTTTCTTGAGATAATCTTTTTGATCCTTTTATTTGATAATGCATTAAACGAAATTGGGAACACATGTGAAAATGGTTTAAGGAGGAACTGGTTCTGATACTCTGGCTCAATAGTCTTTGTGACTTATGTGACTTAATAAAAAATAAA |
| >MG9635308  CTTCAGTAGTCAGTTTTTGGCAGATTCTCCATGCATTTTTGGGTTTTGCACCGCTGCATCTTTTCATGCAATCCAGACAGTTTGAAAACCTGTTGGCGTTTCGGTTGCAGTGGCCGTGCAGGAAAAAGCGACAGCGGCCAGCATCCGAGTCATATGCCCATGTATGAACTCCCGTCTTGCAAGATCTGTTTGGTAGCCTAAGGCTACAAACTCGTTTCACGGGAGCTCCAGGCAGGCAGGTTTTTTGGCATTCGACCTCACTTTCAAACCTGTTGGAATTGCCACCACATCCGCCGTATATAAATACTTTGCAGTGCTTTGTTGAGACATCATAATACCATCTCGGGAACTTTGCTTTGCAGTGGCCAACTGCAGGTAACTGCAAACAACTTTGTGGTGTCCGATAACTAGCCGCATGCGCGGACACCAGAAACTCCAGAAGAATAAGGAAATGAGCTATAATCATAGTCGACGCTGTTGTGC |
| >MG9636497  TGCAGACTAGTTTTTTACTGTGCCATAACTTTGTAAGTGGGACAGCAGGTCTCACTTAGGTGTTTCAGTCAGGAACATAAGGTACGTAGCGCTGTAAATGGTAGTTCCGATCCAGAAGTGATGTCAGCCGCAGACGCGGAGGCATTCCTCATATGAAACAAAGCGATTCTGGTTTCCTTGGCATCCGCCGTAAACGAACTCTCGGCACGTCCCGGATGAAATGTCGTAATAGAACGAGCGGAAGTAGGCGAAGCAGACTCCGGGACTAGGCGGCAGGCTGCAAAATCTGGGCCTTCTCTGAGCATTGGCACCATTGAACGAGATGGCCAACAGGACGGCCGCCAGCACAGTCAATCGAAGCATCGTCGACACCTGAGATGGTCGCGGACGCCGG |
| >MG9638532  CTTGCCGCCTCTTCGTCTACGGCGGGGGCGAAGGCAACGACAAAAACTTCAAAAGCCGCAAGCAGTGCCTACGACGATGCGGAAGCGTTATTACGTCGAGAATCTGCAAGCTGCCCCCTAATCGTGGCTACGGCCCTTCGCGCGTGCTGCATTACTACTTCGACTCGAAGAAGCAGTCGTGCCGCCCATTCGTCTTCCTGGGCTTCGGAGGGAACCGCAACAACTTCATATCCAGCGACGAGTGCCGCATGCAGTGCCTGGGCAAGGAGGCCCACGAGAGGGGGGAAGACTGATGCGAGCCGGAACACGCACGCGAAGCCAGCAACGCTACCCTACGCTTTGGAAGTCGACTCTGGACGCTTGGACTGTGAATGCCTGGGGAAAAACGCG |
| >MG9640383  TGAAGTCCCTGGTCTGCCTGCTCCTCATCTCCCTTTCACTCGTGTGCCATGCGGCGCACAAGAAAGCTTGCAACCTGAAGAAGGACCCCGGCAACTGCGAGGACGCCTCCACCAAATGGTACTACGACAGCAAGACCAACGCGTGCAAGCTGTTCGTGTACGGCGGATGCGACGGCAACGACAACCGCTTCGACACAGAGGCCAAGTGCCGGAATGCGTGCATCCCCGCAAGACGACCAGCGGATAGGGGCGGCAGCAGCAGAGCCAGCCTCACTGACGGCAGCGGCGATCCGGTTCGACA |

| >MG4828  TCGCACTTATGAAATGTTAACTTAGTCATAAAACCCTACAAAACGCTTGAAAGATTATCTTTATTAACGGAAGCAAAAGCAGCAAAGCAGCAGCGCAATCTTATAATTAAGATGTTGTTGTTGTTAGCGTATCAAAGGATGGGCTAACAACAACAATTAAGAAGTACGGAATCACTACGTTACATTAGATTCTTTTTCTTCCCCGTTCCCTTTAGCGCTACTCGCAAAAATACATATAGTGTAAAGAATGGCACGACGAATTATTCTTCGATCTCTTCTTAGCGCGGCCGAAGACCTCCGCNNNNNNNNNNNNNNNNNNNNNNNGACCTCCGCAAGCCCTCATGCAATCACGTTGTGCTTTAAAGTTGTTGCCGTTGCTGCGGCAGCCGCCGTAGGTGAACTGGCGGCAAGTATTGCTGCTGGCGTCGTAGTAGAATCGGAGGAAGCTCGCCTTGCAAGGCCCAGTGTATGGCGGCTCCATGCATACGCCTACGCCGGATCCGGGAAGCCGCCCCTTCTTGTCATCGAAGTTGGCCTTCTTGTTG |
| --- |
| >MG4829  CCGCTTGTGCGGCTACAAATGTTGACAAACAGTGCACTGCAAAAGCGGAAAAGGGACTTTGCAAGGCTAAGCTTCCAAGGTGGTGGTTCAATACGGATTCTGGCAAGTGCGAGCTCTTCTACTACGGAGGCTGCGGTGGCAACCAGAACAGATACCTCTACAAGGAGGACTGCGAAAAGACATGCGCTCCGAAAACACTGAACGAAACGCCTCTCACCACATTCAGCAACAAGAAGGCCAACTTCGAT |
| >MG4830  CCGCTTGTGCGGCTACAAATGTAGACAAACAGTGCACTGCAAAAGCGGAAAAGGGACTTTGCAAGGCTAAGCTTCCAAGGTGGTGGTTCAATACGGATTGTGGCAAGTGCGAGCTCTTCTACTACGGAGGCTGCGGTGGCAACCAGAACAGATACCTCTACAAGGAGGACTGCGAAAAGACATGCGCTTCACAGACACTGAACGAAACGCCTCTCACCACATTCAACAACAAGAAGGCCAACTTCGAT |
| >MG48609  GCGCAGCAGAGAGTTCCCACGAGAATTGCAGCGGTCGCAAGAAGGTGGTTATCAGTGCTCTTACAAAACACATTCGCGTTCGGTCTATAAAGCGCGCTCTGTCTCTGCCGACAGTAATTCATCTCCAGATCTCACCAAGAAAGCAATGGCAATTAATTACTGCATCCTGCTCACCCTTTTTGCAGCGGCTTTGGCGCAGCGAAACGCCATCTGCAGGCTGCCGCCAGACGAAGGCGTCTGCCGGGCAAGCATTCCGCGGTTCTACTTCAACCCAGCTGAAGGAAAATGCTCCTTCTTTATCTACGGTGGTTGTGAAGGAAACGAAAACAACTTCGAAACTATCGAGGAATGCGAAAAAACATGTGGTGAACCAGAGAAGCCCAGTGACTTCGAGGGAGCTGACTTCGAGACTGGTTGCGCGCCAAAACCGCAACGCGGCTTTTGCAAGGGCTTCTTAGA |
| >MG48669  GTGTTGAACCTGTTCTTCTTGCTTGAGAACGGAGAGTATCGCGAAGTCTTGCAGCTGTCGCTCTCGACGTCGTAGTAGTATTTTTTCACAATTCCAAACCTATTCTTGGGACCCGGTTCTACGCACTTGCTCCCAGAGTTCTGGCCACAAGTGTTCCAGCACTCCCTTGCGGAGTCGAACCTGTTGGGGTAGCCACCGCAGCCGTAGTAGACGAACCTTTCACACCTTTTGTTCTTGTTGTTGTATCCAAATGCTACCTTACGTGTACCATTTGGGCACTTCTTTCCGCGATGTATTGGGTAACTGC |
| >MG48670  CCGCGATGTATTGGGTAACTGCAATTGCTTTCGACGTATTTGCCGCATGTTTTCCAGCATTCGGATTCGTAGAGAAAATTGTTTCCATTTCCTCCGCAGCCTTCGTAGTCAAACTGTTCGCAGGCTTTTGTTTGACTGTTGAACCAGAAGTTCTGCATGCGGCTCGACCTGCTGCAGTTTCGACCCGAGTGCTTTGGGAGGAGGCATGAATCTTGGGCGAATCTTCCACATTGTGCCTGGCACTCCTTTTCAGTCTGGAAATGGTTCTCATTGTGCTCGCAGTAGATGTAACGCACGCATCTTTGCTTGAAAGGATCGAACCGGTACCCCTTATATCCAGCTCGGCAAATCTGTTTGGGCTCTGGCAGGGCACACACTCCGAGAACAGGGTCTCGACATTTCTTACTGCATTCCGCAAATTTGTTGAACCTGTTGCCGTTTCCATTGCAGCCTCCGTAGTAAAATACCTCGCAACGGTACGACGTCGCGTTGTAAAACCATTTCTTGAACATTCCTCTGCATATTCCGGGATCCGGCTCTTGCATGCAGTCTTCACTGGGTGACGCAGTTTTACTGGCAGCGCAGATTCCTGCTAGGATGCATATGAAGAGTGCGATGAAAAGACGTTGTACCATGATGAGCAGCTCGGGTCCTTGAGTCGAAAGCGGCACCTGACAGGCTCTGCGGTAGTTACCTCGGAGCTGAAGTTTAAATGAGCTTCACACTATCACCCTAGACCTCCTAGGGAAGACGTACTGAAACGCCCCAGGCATGCCTCCATTTTATACCTG |
| >MG48686  CACTTGTGGCCATCATGAAGGTTTTCATTTTGCTTGCCGTGGTCAGCGCCGCTTTCGCAGCGACTGCGTTTGACATGAGATGCACCCAGCTGCCCGATAGCGGTCCCTGCAAGGCGATGATGCCGATGTGGTGGTTCAATGCCAGGACTGGAAAGTGTGAGCTTTTCAACTACGGCGGCTGCGGAGGAAACGAGAACCGATATGAGAGCAAGCGACAGTGTGAAATGACGTGTTCTGCGAGTAAACCTT |
| >MG48787  GTGCGTGTGGCGGTGGGAGAGCGAAGCAGGCAGCGGCTGAGACGCCATGGCCTCTGCGGCTGCTTTGTCGTGGTTCCTTGTCACCTCGCTGACGGGCCTTGGAATACTGCCCGCGCCGACGACACCGGCAGAGGCCCTGGTGTGTGTCCCCAAGCCGGCCAGCGCCCTCGAGGACCTCGATTACAAGAAGTTCTCGGGAGACTGGCGAGAGGTGTTGAAGAGCAAGCACGCTCCGGTTCTCCTGCAGAAAGGCTCTTGGCGCTTTGACTTCGACACCAACACGTTCGCCTTTAGCGCAGCACTTGCAGGCAATGAGACATGCCTGCCGGCCATTGTCGGCAAAGTCCACAAAGTGCTGCCCGTCGGACAATTTCAGCTCGAGTACGACTTTTTTGGGCAGCCTGTACAAGAGACGCTGGCCGTGGTAGCCACGGACTACGGGAGCTACGCCGTGCTGCACCGGTGCGCGCATGAGCAGCCCCACGCGTCGGTGTGTGCCCCGGATGCGACACACGTCAGCGTCCTTGCGAGGCACGTGCTCGAACGAGACACCTCTCTGGAGATCACGCGCCACTTGGAGAACGTCTGCGTTGCTGTCGCACAGCTGGAACCGCACGAGTTTGACGAAGCATGCACTCTACCGTCCGATGAAGATCCCACCTTGGCCGAAATACCACCAGGCACGGGAATATGCTTCCTGGAACGAAAGGAAGGCTTCTGTGCGAATAAGCAGACAGTCTTCTACTACGACCGAGAGCAACGCAGCTGCCTGAACTTCACTTTCACCGGTTGTGGAGCCAACGAAAACCACTTCCTTACTCGCCAAGAATGTGTTGAAAGATGCGAGAAGCCACTGGAGCAAGCTCGAGCTCCACCTGTGGAATCCGAATCAGCCCAGTGTCCCCAGCGTGCAGCATGCGGCCTAGCCTGCGCTCACTGCTGCCAGCGGCGAGAGAAAGACAGCTGTATTGCGTGCAATTGCCTCGCGTCCGAAACAGAGCCCCTTCTCAAGAAAGCTGACTGCCTCGCGGCTCCCGAGCTGTGCCCGGCTGGTTGTCAGAGTAGGGAGGTGTCTGCGTCGTGCTACGCCTGCGAATGTGCTGCTGCAGACGGCACTGTTGAATATGCACTGGAGTACGTGGATGCCCAAATCGCAGTGCCCTGCCCAGTGACTTGCGTGAAGCGAATTTCGAGCAGCGGCGCCCAGAGCTGTCACTGTCCGTCTGATGGTGCGTGCAAGTTGCCGGTGAAAGAAGGCAACTGCAATGAAAGGATTCCCCGCTACTTCTTCAACGTCACTTCCGGCGTTTGCGACGTGTTCTACTACACCGGCTGTGGTGGGAACGAGAACAGCTTCGCCTCCCATGAAGAGTGCATTGCCCAGTGTGAAGATCCCTGCCGCCTGCCAATGGATCCTGGCAGCTGCAACGACACGCAGGAGCGCTACTACTTCAACTCACAGACGGGATTGTGTGAGACCTTCGAGTACGGCGGCTGCGAAGGGAACAAGAACAACTTTGCAGACCTCGACGCGTGCAAGACGCTTTGCGAAGATGTTTGCTCGCAGCCCCAAGACCCGGGCCCATGCTACGCGTACTTCCGGCGCTTCTACTACAATAAGCAGGAGGATCGCTGCCTGCCTTTCATCTTCGGTGGCTGCATGGGCAACGGAAACAACTTTTACACCAGCACCCAGTGCAACGGACGCTGCCGATTGCATGTTGCTGAGGACGGCGCAGCCGGAAAGGACAGGGTGCATGTTGCTGAGGACGGCGTAGCCGGAAAGGACAGGGAAATGTGTCACCAGCCGGTGGACGAAGGCCACTGCGACACCGACAGCAGTGGCCCAGGGGCCACTACACCAGAGATCCGCTTCTACTACGACGTGCAGAAAGAACTGTGCGAGCGGTTCAACTACCAGGGATGTGGTGGAAACGACAACAACTTCCGTACCGTAGACGGATGCAACATGACCTGCTTTGGTGTGCGCATCAACTTGGCCAGGACGGCTGCGAGCTGCCCGGAGTCCATGCAGTGCAGCTGCGGTGATGAGCAGGAGGCCAAGTGCGGGTCATGCGAGTGCGGGAACGCAGCCCCGCGTAGCAACGGGGCCGCCCTCTACGCTCTAGTGTTCCTCACTGGTGCATCGGTGACAGCGTCGCTCTGCACTCTCTGAAGAAGCGACGAGGAGGACGGAAATATTTCTTTTTTGTTCCCAGATTCTCGATTAGCGCATGCGTGATCACAGGCGCACGCGGTGTGTAGTGAAATCTGCGCGCACTGGCAGTAACTGCCAAGGACGTCCACGAATGCAATTTGTGTCCCTGGCGATATGAACTTTCTGTCGCATTCCATCGGTGCGCCCAATCTCTAAAATATGCATTGTCTTTGTGGCAGTCAACGGAATTTGCTTACAAGCAGCGTCTTTTTCAGCAACACTGGATAACTGCAGCCAGGCAATCTTGCCCTCTGCACAAAATTTTCTAACGCAACAAATGCCTCTGGCGGCGTGGAGACTATCGGCAAGTTCAACCACGGCGCATCTGCCTCGACGCGCTCCTCCACTTAGCGTGAGCCTGCCACAGATAGAGCCGAGCCGACGGGCAGCGCGGCACAGCAATTGTAAGGATGTGCGCGATTGTGTGCGGTGCCGCTCGTAGTCTTGGCACATGCGCCGCAACTAACGATAGGGACGCAGTGGCGTTAGGCATAGTGCGCGCGAAAAGAATTTGGAACAACCGAGGTTCCATGGTGGCACGGGGAAGGAGCAACACGAGGTTTTTAGGTCGCACAAGCAAATCGGTGACGTGAATGCCTGGTTGCTGTCATTACCCTAGCACGGGGAATGTCGGTCGACGACAGCCGTTCGCCAAGCGCGCATACTATCGCCTACTAGTCCAAGGTTCGACAGCCCGTTTGGCCAGGAAAAGACATTGCAGGAGGAGAAATCGACGAGTGCCAAACAGAAGGGTAAAATACAAAGGCGGGGGCTTTTCGAGACCCTTACAAGCGCAATGTTCACATGTAAAAGGAATGACTGGACAGCCGCCTTGATATGCGCGATCAGCGCCACAGTGATCATGAGTATGTTTCGGGCAAGGGCTCACGTGTCTGTGCGCGATTGGTTGTGTGGATATGAAACGACATCAGGTGCAGACATGATAAGAATGGCTTTTCGATAGCCATCATTGTAATTCTGTCCCAGACAATAGTGTGTCTGGCGCATTCGTGGTGTTCTACCAGGCCATTCAATGCCTTTTACTTTTAGTGACACAAAACGAAGAAATTATCTCTGTGGTTTCAGTAAGCACTTTCAAAAATTCTTAATCTTATGGTTAAAGAGGAAAAATCTCCGTATAAATACATTCGCACCCTCGGCGGACGTCAGTTACTAAACGATTCGCACTAGTAAGGCATATGACAAAGTACGCTTTAAGATTAGCGTTTTGGTTAAAACGGTGTGGTGACTAGTGCTAATTGAGAGCTGGAAGCGAAGGGAAAGACGATGCCTTATCAGTTTCTGAAAATGTTCTGTAAAACCGTGCAGTGCGAGTAAATGCGAGGCTCTTTTTCGCACAATACCGAAACTGAGTGCATGAGGTGTGCAGGGAGCCCAGTGCAAACACAACGACCGCTGGTGACACGGTGCAGGGCCCACAGTCTCAACACAAATACACCGCTATTGCAGTGAAAATGAAGTAATCTGTCCTAAAGTGCACTCCCTTTCACAAAAAAAGTGCGCTAGAGGAAAGCGTACACCTGTTTTATTCTTTCTGCGTTTAGAGTGTACGGCGCCCAATTCTAACTGCCCGCACATTTTGCACCACGGCATACACTTTTGTTTGCTTTGCCACTTGGTCCAAAAAAATTATAGTCGTAGCCACCCGCGTATCTGTTATTTTTTAGAGGGGCGAAGCAAAATGAAGTGCATGCCGTGGTCCAATGAAGCTTATATTTAACAGCGTTTTTCGGCGAGCCCTTCCAAAATTGCTCAAAATAGGGTGGTCTCAAAAGAGACTGCTATCCGCGGCAGTTCTGAATTATTTTGAAAATATGCTTTTTTCTTGCTAAATAATTAGCTCTCACTACTGGCAGCCCCGTAGTTTACGATGAGTATTCATTTTCTTGCACATTGCGACGAAGCTTAAGAGCACTTTCCGTGGTGTATTAGCAAAAGCAGTTCGAAGTACCGTGTCTATGGTTCCTAGGATTATCTTGCCAGCATTGGTCTGTTTTCATGTTACATTTTTGTTATGCTAACGACCAGACTCTGTTCATTCCAAAATAATTTATTAAATCTCATTAAAGTAGCGATTTGATTACTATAGTTGTAAAGTCACTCACCTCCACTGCGGCCGATTGTGTGTTCCTGAACAGTTTTCCTCTGTGTTTGAAACACCCTATAATTAGGAATGCTTGAAAGTGCTGGTGACATACCGGGCAAACGAACACATATCAAAAACCTCCGCATTCAAGAAGTGAATTGCTATGTCACACGTGAGCTGGCATCGTGTGAGGGTAGCAGCTCCTCAAAGTAAATTTTTTAACTCTCTTACAATACCCTCCTTCCCCCTATAATGATAGGTGCGAAAGGTCCAGCCAGCCTCATCATCAGTGACTACGGCACCAGTTAAAACTCGATTTAACGAAAGCCGATTTTGTGAAGTTCTCGATCTAACGAAAAAAATTTTATACCCCGGCAAGTATTCATAGGGTTCAATGTTCGAATAACCTGAAATACCGAAAGAAACGATCACTAAACCCTATTTAATGAAGTTTTTCCGGGAATAAACGACGATGTCTCGCAGCTTGTTGGAAGCGTGCCAGCAGTACATCTCCGCGCCCAACTCACGGCCCTGGTTTTCTTCTGACGGGGCTGCATGCTGCGCTGCTGAACGGAACATAGGACTCGGCAGTCGGCGGTGCTTTCCACCTTGGCCTCGAATCCGGTTTGTCATATGGTGCTTTTATGCACGAGCGATTCGGGCCGCTTTCGCGGACTTTTCATACGCGAAGGCGTGGGCTGTCGGCAAATGCGACGCCTCGGTAGCTTTTCAGTGCCGCTACGTTAGAATTTAAGATCTCAAAGGCCGTAAAAATGCCGTCTTCGATTTTACGAACTTCCCGATTTAACGAAATTTTTAACGGCTGCTTCTCGCTTAGTTGAATCGAGTTTTAACTGCATTTATTTTGATTCTGGTTTACAGGTGCGAAAGAGCACGTGACTACAGCTTGTCCGCCTACATGAGCAGTTGTTGGCGCGTGCCGTGCGTTAAACTACGACGTTGTCATTTGCTGTGTTCGAGGTTAACTCGCGGATCACGAAGGCACAATTTTCAAGCAGAACTTAGCGGAGAAAGAAAGTACCACATTAGCAAGGACATTTCTGTGCAAGAGTTGCGCATTATGGTCAGCAATGAATGGCAATTTCATAACAGGTGGCATCTTTGCCATCGTTTTGCGAGAAGCTGCCGTCCCCACTGCCGGCTTTGAAGGGGGCGTGCAGCTGCTTCATCGCCGACTGAGCGTGCTTACCATGGTCATTCTGCTCTTTGTTAGGGAAGGTCCACCAATAATTCTTGTTGGGCATTTGGTCAAAATAGCAAACCACACCAAAAAGCTTCCATGAGATGGCATCACAGGTGGTGTACCTTGTTGGTGCATAGCATAAATATTAATTCCTACAAAGTCCATACTTATCTCACTTTCCTGCCCAGTCATTCACGAATATTATATCAACGCATTAACGGGAAGTGCTTGAGCATGCAACAAACTTTTATGGCTTTTAATGCTGTTCGTCGGAACGGGTACAAAAAGCATATGATACGGGGCGAGCTATAAAGAAGTGGAATAGATTTTTAAACAAGAACAACTGAAAAGAAACCTATATCAACCATCTAAAATCATCGGCAGTAAAATATTTGCATTGCATGCCGCGCGGTAAAGAGCTGAAAACTCAAGTGTGGGTGTGTGCGTTTTTAAGGTTCATGTTTACTTTTATTTCTTTTTTCGAGAGCGAGTTGATGGGATAGTTTGTGCTCCATGGTGCAAACGCAGCGCAAAGGACAGAGACATTCAATTCAACTCAATTCAGTTCAGTTTATTTTCCAGGAAAAAGAACATGTCCCGGACAGATTTAAAGGCTAAAAGTTGTTAGGAACAGCTTGACTGCTCTCAAGTACCATTAAATGACATTCAACGACGGAAGGCACAAAAACTGGTTGAAAGCAAATAGCACAAGGGCATTTAAAACAAAACGAAAATAATCAGGGGGGACATTGTTTCTAGGAAGTCCGAGGAATTCAAGACGGGAGCCCCCATAGTAAATCGACATATCAACCCTATTGGTGATTGGTGACGTCATGAGTATACCGATCTGGTGATTGATTGGTGACGTCACATATTTATCCAATTAGTGATTGATCGGTAACGTCACTTATAGAACCAAGATGTCAAATTCGTATTTCCATTGGTGTACCTCACTTTAACCTCATTCATATCAATTCTAGCAAACCAAACAGCTAACGCTCGTTACTTCAACGTCTTCCAGACCGTGGCGGCATCTTTTTTTGCACGCTTTTTATTATTTTGTTGTATATTTTTCTGGTGATAGACGCGCATGAATGTAGTTTTGCGTGAAAAAAAAATCATTGTGAGCTGTTTTTGTTTCGTGCTTTTGATTTCTTCCCCGTACAATCGTGGTACAGTTGAAAGAATGATTAAAGAATACATAGCCAGGGTTTATATTGTTTAACCAGGAACTCGGCTCGCAAGATATATCTGTGACATGCTCCAAATGTGCGCACCGAGTGCATGATGCGGGCAGCGAAATGCTACGCGCGCAGTGAGGGTTCGAAGAGTTCTGGAGCTGCTATGCTGAGGCGCGCTTATCCAAGAGAACGTTTCCTTGTTGACGCCGAGGCTGATTGAGATGGAATGCTGCAGACCAGGAGGGAAGGAAGGTAGTCACCTGCAGAGAAGCATTCACCAGGAACGATTGACATCAGCGTCGCTTGGTGCAATTCGTTTTATTTTATATGCTGTACCTGTGAAAGGAAAGAGTTTATTTTGACCGTGTTATTTGTGTCCATTTCCTTAAGCGCGCTACTCCTCCTTGAGGATCTGTCCTGAACTGCAAGCTGAAGCAAGTTGAAGTTCCAAGTAAACGCTGAAGGCATAATGAAATGAACACAAATAAAACGCTCCTCGAGTAGCACTG |
| >MG481105  AACTTTTCCAAACTAGAAATACAGATAGACGTCGTAACGTTCTTCAATTCCTGCAGGAATTTCTTTTGTACTAAATCACAGTTTATTGAAGTTTACGCTTTGGATGGTAAGACAGTTAATTGGTCATAAATGTAAAACATAGGTGAATAAAGCTTCAATTCATGGTGCAGATATCTGCGGGGAAATGCCGTTCCTTAAAATTCAGAGCCGTTCCTGTTTTTTTTTTTCAATGTAGAGCATTCTAAATTTGTACACATACATTTTCAGATTTTAGTCGATAGTAGAACATAATCCGTGTTTGAAAGGATAATAATTTAGTGCATTTTATGAAGAAACCGAGCAGCCGTCGCACCCTTCGTTTGAAAGGAGCCAAAATAGCATTTTAGAAGGTTTATTTAGGCAGCAGCTGGTTGTTATTTTATGTGGCTACAAATGCAGCACAGGTATTTCAGCGCCAGCTAAAGTCACAGCTCTGAGGGATGTCCCTGCCACCTGATGCGCATTCGCCGCTATGCAAATTACTTTTTCGGAAATGGTGGCGTTAGTTGAGCAGGAGGAAAACAGTTAAAATATCTAAGCAAAGAAATGGTCTGCTCGAAATTATTTACTTCACGCGGAGACATGGGGGAGAGCCCATTCCGAAGAGTGTCAAGCCAAATACATCGCAAACGAACAAGAGGCTGAGTAAATCAGCTTTATTAATCTGCCTCTTACACTAGTTATATCCTGCATGAATTTCCCGAGATCTCACAAAATTTTTCAGTTATTTTTAAATTGAATGTGTACGCATATGTGTTATAATCTTTTAGGTCTAAGAATGTTTAGCACGTTCTTTTTCGAAAAGCAGTAAGTGCAACATGTACGTCTATGCGTGCCGGGGCATCGGCTCCCAAGGAGACTGAATGGCGCAGGCGTCCATGCACTGGCGCAGGGTCTCGAAGTTGTTCCCGTTGCTGTGGCAGCCTCCGTAGATGAACTGCCGGCATTGCTTTGTTTGAGCATCGTAGTAGAAGCGCGGGATGCTAGCCATGCAA |
| >MG481759  CTTGAGCACCCCTCGTGACAGCATGAAGGTACTCGTACTTCTGTCGCTTTTCGGCGTCGGGTTGGAGTGCACCGCCCAGGCTCCGAATACCATCACGCTCCTTGCCGAAGAAGAAACGGAGGCCGACTACGACTTCGAGACATACTGCACGCCGCGTGCTGAGCGTGGTCCTTGCAGAGGTCACTTTCCTAGCTGGTGGTTCAATGCCGTGGTGAGAACGTGCGAGCCATTCGTGTACACCGGTTGTGAGGGCAACTACAACAACTACGTCAGCAAGGAGGCATGTGAACTTACCTGTTTGAGGCGAGGAGGAGGTTCGCAAACGAGCAATCCGGACATAAAGGAAACGGACCCGCAAGCATTTCTGGACTGCTTTGTGGAGGCAGACAAGGGACCGTGCGATAAAACTATTTATCGTTATAACTTCGAACCGGAGTCGGCGACTTGCAATTTGTTCGCATACGGTGGCTGCGAAGGAAATCGCAACAACTACAACTCAGAAGAGGAATGCATGGCCAAGTGCAACCCTGTAAGTGAGTACTACAGAAAGTGCCTGATCAAGCCAAAGGAAGGGCGTTGCAGGATGTTGCAGAACTTGTGGACGTACAACGTGACCCTTGCCCAGTGCCAGCAGTTTTGGTACGTTGGATGCATGGACAACGATAACAAATATAGGACCAAGGAGGAGTGCGAAATGACTTGCCTGCGTCAGCCAGGGAATGTTAACCCCCTCTGTTTCGAGCCAAAGTATCGCGGGCCATGCGGCGCGCACTACCCCCGCTACTACTACAACCGGTGGTCAAAAACCTGCGAGAAGTTCATCTACGGCGGCTGCCGGGGCAACGAGAACAATTTCGAGACTCTGGAAGAGTGCGAAAACACATGCTGGGTCTCAAGGAAACAGGATCCAGCAGATGTCAGCGAAGCATTTCAAGCTCCATACAGGCCGTGGGCAACGCCTTCGGAGTGCACTTATCCGGCAGAGGCAGGACGTTGCTTGGCTTACATGCCACGTTTCTACTACAACGCGACGACCCAGTCCTGCGAGCAGTTCATCTACGGAGGATGTGGAGGGAATGCGAACAACTTCTACAGCTACGCCGACTGCGAAAACAAATGCAAGACGTCCATGGGCATTCTCCCTCGCGCTTGAACATGAGACGGCAGCAGAAAATAGAGCACAAAAAGAGAACAGAGTTTATAGCAGGCTAATTGCCTCATCTTGTGAATTTTTAAGAGCTGTAACAAACAAATAATTATATGGCTAGCTTACTTGCTCAGTGCGCTTGTGGCCGATTCTTTTCTTTCTTGAGATAATCTTTTTGATCCTTTTATTTGATAATGCATTAAACGAAATTGGGAACACATGTGAAAATGGTTTAAGGAGGAACTGGTTCTGATACTCTGGCTCAATAGTCTTTGTGACTTATGTGACTTAATAAAAAATAAACTGGAA |
| >MG482245  TGGGATTCCGGGTTCCCTCTTCGCCAGCAAGTGGCACTGGCTTCCTCTAGCAGGAAAAAGGTTGGTGAAGATGTAGGTGCGCCTATACGCACCGGAGTCATCCTTTATTTTCCGGTGCCAGGGGCAGAACTAGAATCCAAAATATGCGTGCAGCACTTTCTGGCTTTCGCAAGCATTAGACAGTTTAATGAACTCGCTGAAGTACTTTTCGAGAAAGGAGATTGTCGCGTATTTTGACAGCCTTAATTAAGCTTGGCGGTTCCGGTTATTTCGGCATGGCCGTCGGGAAGTATTTCTTGTTGGCTTTTTCAGTCAACTGTTGGCAATACTCTCTGTCTTTCTTAGTAAATTTTTTAGCACAAATGTTCATGCATTCATAGCAGCTGTCAAATCTGTTCCGAGTTCCTCGACATCCGCCGTAGATAAATGCTTCGCACAAGCCGCTTTTTGTGTTATATGACCA |
| >MG482444  CTTCTTTCTTCTACCAATGTGCTTGGCCATGCATAGCAGCATAAGCCCGAAAGACCCTGCCGTGTGCTATGCTCCACGCCCTGCCAGCTACTGCGATAGACCTGAAAACGTCTTTCCCGTCTTTTTTTATTATCCTGGGAGTCAATCATGTTTTCAAGATGTTGGTTGTACTCTGATGGGTAATAACTTTGCAAGCCTAGAAGAATGCGAAAGAGTCTGCTTAAGAGGAAGAGCGCAACCACCGGCACAACCGATGGCGTTGCATTTTCCTTTGGTTCACTTCTAAAGTTTGTCATCAACCACTGCAGCGACTGAGGGAGAAGTTTGGATTTGAAGAATTTCGCCTTGAAGATTAAACCCCACTGGGCAATGGGTACTGATGAAGCAATGGTTGTAATTTCGGCGCATGCCTTCTGTGGAAATCCTCTAAAATAAAAAAGTTTATATTCTAAGCATTTAATTTATGCAGATCTCTTTTCCATCAAGTCAAAATGCTCCAATTTCGCCCGAAAATAG |
| >MG482575  AAGCTTCAGCCATCTCGCGGCATCATCAGCCTCAACATGAAGCTCTGTGTCATATTCCCTCTCATCTGCACTACATTTGCGGCCACCTTCTACGAGGAGAAATGCACGAAGGACCCAGAAGTTGGTTTATGCAAAGCTTTACTGCCGAGGTGGTGGTTCAACGTGAGGACTCACCAGTGTGAGGAATTCTTCTACGGCGGCTGCGGTGGCAACGAGAACAGATACGAGACAAAGGAAGAATGCGAAAAGACTTGCGCTGATGAAAAGC |
| >MG487956  GAGTCTTTGTTGCGTTTGGCTTGTGGGCAGCCATTGTGCCCGGCCACGCACTTCTCAGAGACGATATCTGCAATCGTCCACGCCCAGTGCCCACCTGTCTGGGGCACGTATTTGAAGTTTACTATTATTCACCAGTGACAGGAAAGTGCCACAGCGATTTGACCTGTACTCACGAAGGCAACAACTTTCCCACGTATCACGAGTGCATGAGAACTTGTGGAAGGATGGCGCCGATGTCGACTGCACCCATGGGGATGACGATGGAAGGGCCCTTTTACGCCTTCTTTTTATGAAAATGAATTCCAATTTCTGGTGTGGTTTTGACCTCATTGGGCGTCGATGGAGCTTTGTCAACGATATGAGGAAACCACCTAAACGACCTCAAAATAAAATGAGTTCTTTT |
| >MG488161  GGACCCACCAGTGACCGAGAATCATTGCAGCCAAACATCGTTCTTTTGTCTAACACTCATGGTTTATTTGTTGTTCTTCTAGAGTGATGTCAAGTTGCTGTTTCCACCTTGAAACAGCCTCTAATGAATTATAGGCACGGGCCCCAGGGTGGCGCAGACGGCGAAGCACTCGCGCATCGTCTCGAAGCTGTTGCCGTTGCCGTGGCAGCCTCCATAGAGGAACGACTTGCATTGGTTTGTCTTAGCTTCGTAGTAGAAGCGTGGGATGAAAGCCACACACGGGCCGGGGTACGGTGGGAGAAAACACAGATCAGGCTTTTTCTTCGCAGGCTCACTGAGCAACACTGTGGCTTCTTGTTTTGGCTGGAAGATAGCAAATGCGGAGCTGACAACAGCGAGGAGAATGTAAGCCTTCATGTTGCCGTACTGGTGGCGTCTCCGCATTTGCTATCTTCCAGCCAAAACAAGAAGCCACAGTGTTGCTCAGTGAGCC |
| >MG489096  AAATTTTATTTTTTGTGTCCACGTTTCCATCATACAAAATATTGACGTCAGCAGCCTCGGGGAATGCGTTTGTGGGGAAAGCAGACTTCCAGGCATTCTTCCTTCGAGTCAAACAAGTTTGGTCCGCCGTTGCAGCCGCCGTATAAGAACTGAAAGCAGTTCTTGTGGTAGTGGTCGTAGTACCACTTGAAAGCAAATGCGTAACATGGTCCACGGTTTGGCCAGTGACGACAGTCCTCCGGGGATCTTCGTCGTTCGCAGACCTTGCGGCACTCCGGCATGCTGGGGAAGTTGTTACCGCGATTGTGACAGCCCGATCTTTTCTCGCACAATTTCGTGTCCTGGTTGTAGGAGTACACGCTGCGCTCCTTCTCGGAGCACGAATGGGGTAGGGGGNNNNNNNNNNNNNNAGCTGCCTTGAGGAAAAGGAGTTTGCCCGCGCAAGGTGACAGCCAACACCCAGGCGGCATTCCCTGGTGTGTCTGTCGTAGTACCACGTGTGCCTTGACGGCGCGTAGTTGGCACAAGCAGGGTCCTCGGTTGGTCTGGGAAAGTGGCAGAAGGACTCTGACGGGCATGTTTTTTCGCAGGTGCTCCTCTTCACGAAGCTGTTGCCAGCCTTAGGGCAGACGAACCGGCGTTCGCACCTCTTGGCGTCCAAATTGTAGACCCACCTCTCCATAAGGCGTCCACAGCCGTGGGTTGGCGGGAAGGAGTAGCACACGGGACTCGGCATTGGTACCGGCGAGACTCGTCTTACGCAGACCCTGCGGCATTCTTGTAGGCTTGGGAAATTATTCCCCCGGTTATGGCAGTTGACGTCTTTCAGGCACTGGCCGGAGGTTGGGTCGTAGTACCACACGGTCCTCTGTCCGCTGTCGCACAGTTCCAATGGCCTCGGTGCATCGCACACGTCGACTGCTCTGCACATCGAGACGCATTCTGATCTCGTGACGAAGGCATTGGCTCCGATCACACAATTGGCCGATTTTTTGCACCGATGAGTCTTTGCGTCGAAGAACCATTGGCTTTGTGCGGTGGTATTGGAGCAGTGTTGATCAACTTTCGGGAAGAGGCAGAAAGAATACGCCGGGCAAGCGTACTCGCAGTCCTCCCTGCTGTGGAAGTTGTTTCGATCCTCGGCGCAGGTCTGGCTTCTTAGGCACGACTTGGTGAGCATGTCGAAGTGCCACCTCTGTCCGGCATTTGCCACCCGGCACGGGTGGTAGCCCACGGCGTCATAGCACTTCGACTGAGGGTTCTTTGGTCCCCAAAACGCATCCTCGTACTCGCGTTGGCCGCTCGCGAGTCCCGCGAGCAGAACGCACAGCATCCAGGGCACGGCGGCGTTCTTCATGTTCTTCCCTCTCGTGCGGTGGTCCCGCCCCGCGCACTCAACGCCGTCTGGCCGATGGGAACGCTCTTCTCCGACCGCCAACACGCGGCACGCACGCAGCGTAGTAAAGCGCCTATCTTGCGCTTCCTCCGGGCAACCAACCTCTTGCTTTCTTCTTGCGCGCCT |
| >MG4812549  TTTTTTTTGATGCTTTTTCAATACTTTATTCCTTGTTCCCGAGGATAAGAGAAATGCGTAGGAGCCAAATATTTTCACATTTCCTCTCTGCTCTGTAGCACTGTTTGTCTTCTTTTCAGTTGAATCCCCTGGACAAGAACGATTATTTCTTATTATCCTGCTTTGCAAGTGCGCCGACATTCGCCTAAGGTTGGAAAGTTGTTTCCTGCGTAGCTGCAGCCCATATCGCTGTGGCACATGCCGGTCCAAGGACTAAAATAGAAAACTGGAAATGTCTTCCCGCGGCAAAGGGTATACGGTCTCGGAGCAGTGCAGACGGCAACACTTCGACAGGTAGCATCGCATTCTAATTGGGTTTCAAAAGGAGCTCGGCCGCATGTCCATGTGCACTGCTTGGTATTACGGTTGAATGCGTAGCTGCGGTGAGGGCAATTACTTTTCAGCACCGGGAGGCTTGATCTGCATCGTGAATCGTGTGGGTTTGGCCGCCTGGCACTACAAAGTGCCAATAGAGCAAGCAACAGAACCGCCTTCCAGATGGCCATGGTCCCAATTGTCTCGTGTGGGTTTGAGATCGG |

| >MG4814113  TTGCTTTGTGTGCAGCAATATTTTATTTTTCAATAAATCCGCTGCATAAACAGCTAACTGCTGCTTAGAAGTCATCCAAAGACCCTGTGATGTGACGTAACCAATGAGATAAGTTCTTTAACTCTCCGGCATGTCTCCCATGTTTCGGTGGGAAGAATAAAATACTTCAAGCAGACACTAGTGCTTTGCACAATGTTTCATGCAGTGTCGGTAGGACGAGAAATTATTTGCGGTCCCTCCGCAGCCCCAGTACCAAAAAGGTATGCACTGGCCGGTGCGAGGTTTGTAGTAGAATCGTACTGAGCCGGAACCCCATCGACAAGGGTACCCTTGAGTCGGAAACACTAAACACTTCCAAGGTTTTGTTGGCTTCGGTCGACGTCTCATACACGTTTCCTGGCATTCCTCAATGGTGGGAAAGTTATTTCCATAGTATATGCACCCCATGTCCCTGAGGCAGCTGCGTGTCGTGGGATCATAGTAGAAAACAGGGTGTATATCGCTGGCGCAGGAGGACACAGCACGTGGAGCCGTGCACACGTCAACACTACGACAGTATCCATCGCAAGCAAGCTTGCTGTCGAACGGGCCGTCCTTGTTGCACGTCTCATTACACTTCTTTTCAATTAAGTTGAACTGCCATGAAGGTGTTGAGCATCGTCTTGTATTAACGATTGGTTTGTTTGAGATGCATCTTGGATCTCTCGTGGATCGGCTTGTTGCTGCGCAGAGCCCTAAATAAAGAAAAACTGCCAACGCCGGGTACATTTCCATGTCTTTTCAGCCAAGTGGAAGCTTGCTTGGGAAATGTTCTTGCCTCTTCGTTCTCTTATAAGCTCACTGCGG |
| --- |
| >MG4814561  TGAATATCTACCAAGTTTTAGGCTTCTTCTTTCTTCTGCCAGTGTGCATGGCCGTGCATCACAGCAGCAGCTTGAAAGACCCAGCCGTGTGCACTGCCCCACGCCCTGCCAGCTATTGCTATGGTGGAGCCTTTGAGGTCTTTTATTTTGAACCCTTGAGTAACTCATGTTTAAAAGAATTAGGTTGTACTCTGCACGGCAATAACTTTAAAAGCAGAGAAGAATGTGAAAGACTCTGCTTAAGAGGAACCGCGCAACCACAGCCACGCCCGATGGCCTTATTTTATCCTTGGGTTTGGCTCTAAGGTTTATCATCACCCAGTGCAGCGACAAAGGGAGCACTCTTGATTTGAAGAATTTCACCTTGA |
| >MG4814757  TTTTTTTTGAAAATAAGGAGAGTCTTTATTTATTAAATGTTTACTCTTCGTCGTTGTCCTAATAGTAATTCTCACGTGGCGGAGCGGTGTAGCCATCACCGCCTGTATAATCTTCCCATCCCGAATTGTACTCTTCATCAGCCTCTTTTTCAAGCAGTCGGCAAATTTCTGTTAGATTTTCGTTTGCGCTACATGAATCCATGCAGTGCGTGCAGTTCTCAAACCGGTTTTCGTTGCCGCCGCATCCTCCATATGTGAAGAGTGAGCATCTATCATTGGTAGAGTTGTAGTGCCACATGTCAAATAGCCCTCTGCAGTTGCCTTTTACTTGAGGCAAAGAGCAGATGGAGGAAATGCTGACTGATGTACCGTACACGGTGGCCAGAATTAAAAGAA |
| >MG4814758  TATCCCTCTGCAGTTGCCTTTTTCTTGATGCAAAAAGCAGATGGAGGAAATGCTGACNNAGCAGCTTTAGGCTGTGGTCATGGAAATCAAAAGATGCGTTTATTTTCTTTTAATTCTGGCCACCGTGTACGGTACATCAGTCAGCATTTCCTCCATCTGCTTTTTGCATCAAGAAAAAGGCAACTGCAGAGGGATATCTGAGATGTGGCACTATAACTCTACCAAAGATATATGCTCACCCTTCAATT |
| >MG4815417  CGTATGCTTTCCCAATTTGGTTTTTATAAGTGAACCTTCCATTTCGAATCATCAGGAGATTATTGTCCACGGTATGTATATTCTTGCTTGAGTTACGCTCAATCGTCCAATAACACCAGCAGCGGGAAACTGAAGTTGCCACATGCATTCTGGCATTGCTCCGGGGATTCGAAGTTGTTCCGGCCACCGCTGCAGCCGCCGTACGTAAACTTCTCGCAAAATTGCCCGTTGTACCACCAACGAGAGATAAGAGCTCTACAAGGTCCTTCGACGGGGCCCTCCCTGCATACATCTTCGCACGTTTCGTTCTTCTTATCCCCACTGCATATTTCAGCCCAATCAACTGCGTCCTCTCTGCTGGCTACACCTGCGCCTGGAGATAAATTCCTCGCCAATGGTCCCAAACTTGCATCCAAAGAGTTCGGCGTCACCGCTGCCGCGATTGCTCTGCCGCGACCAGGATCCACATCTCCTAGAGAAAGAAGAAGAGTAAACAGAAGGAACTTCATTTTCAGTCGCTCGTTTAGCCAGGAATTAGGAGTCCAGCCCAAGCAGG |
| >MG4815738  AAGACGTGTGCCGTAGGCCCCCATACACTGGTTACTGCTTGGCCTTCTTACCGCGCTTCTACTACGACGCCACGACCAACTCCTGCAAGCCGTTCGTCTACGGTGGCTGCCACAGCAACGGAAACAACTTCGAGACTCGACGCGACTGCCTGGATTTCTGCGCCCCTCCACTCGCCACACAGCCCACGCCATCGACAGCGTAGGGTGCGGAGCACGCTGTGAAAGTGAAAGCAAAAGAAACGCAGAAATAAAATAACTAAAGTCGAACTTCACGAGTATAGAGGAAACTGATGAAGACGTCCGTGAAGTCGTAGGCGACATAATAATTTTCATCTTGAAAATGACTTTAGTTTACACATCTCAGTATAGGCAATAAAAT |
| >MG4815767  GCAAAAGCTGTGGACTTCGTGTTTTGCGGGTTTTCCCTCGGTCTGTGCGCGAGCGGATTGGTAATGGCAAGAAGGTGGCTGAGATATGGCGCTAGGAGTCGATTTAAACTTCTGGTGGTGACGAACGGTACATACCTACTGGCCATATTGCTGGTGTTATCGGCGTGTCTCGCCACCGTCACGGCTCAGGCCAACGTCAGTGCCAACGTCACTGACAACGTCGGCGCCAACGAGACAAATGCCGAAGGTGACACCACCAGCGAGCTGCGTTCCAGACCTCACGAAAGAACGGAGCCCAACTGTTTCGTGCCTCCCAATGCCGGAAGGTGCAACGGGAGGCTGTTCCGCTACTACTTCCACTGGGGCGTGGGACGGTGCATCCGCTACGTCTACACTGGTTGCGAGGGCACCGGAAACAATTTCAGAAGCAGACAGATGTGCATGAGGACTTGCTGGTTCCAAAACTACAACATCTACTGCAAGCTTCCAATGGACCGAGGCCAATGCGGCTCCTGGGTGGTGCGCTATCACTACGACAAAGAAAAGAACCACTGCTTTCCTTTTTGGTTTTCCGGCTGTGGCGGCAACCTCAACAGATTCCGCTCCGCCCAGGAATGCCGGGCGGTCTGCATCCCGGACTGACCGCAAGAGGGCGCCGCGGTCGCGGACAGGGAAGCTCACTGGACCCTTATAAAAATGGTCT |
| >MG4819197  CGCAGAGTATATAAGGCATGCCGAACAACCGAGCAGAAAACTGCGAAGGCGTGTTCGCACTTTCACTGGTTTCCTGTCAACTGTAATAACATGAAAAAGGCTGTGCTTCTTTGCTTCCTGGCTGGCGCTGTTTTCGCAGCAGCAACGGCGGAGGTGCAAGTCCGAAATGAGCACCGCGGCGTGAACTTTGACTTGGGCTGCAGGCCTCCCCCTGAGAGCGGAATGTGCAGGGCAGACTTTGAGCGCTGGTACTTCGACGTGGCCGACGGTGTCTGCAAGCCCTTCGTCTACGGTGGATGCGGTGGCAATGCCAACCGGTACAACACCGAGTGGGAATGCCAGAAGGCCTGCATGCGCGGATCTGGCTGATGTACTTCGGAAGCAACTTCGAACCTATGGAACAAAAACCCAAGGCAAGCGTTTAAGCAAAAATCAGAGCACCATCCAAATGCGATTGAAACAAACATGGTGCAACCATTCAATGTTGTTTATTTGTGTGCGTGAAGTAGCTCATCCCTGTATGAGCCTAAATATGTTTACAGTATTAATAAATAAATAGAAATTTCAT |
| >MG4824755  AAGGGCCAGTGTATGGCGGCCGCGTGCATACACCTTCGCCGGATCCGAGTACGCTGCCCTTCTTGTCGCTTAATGCACCGAAAGGCGTTCGGTTCACGAGTACTTGGCCTTTCTTATCATGGAATGTGGCTCTCTTGACGCTGTGAGGCGTTTGGTTGAGTGTCAATGGAGCGCATGTCTTTTCGCACTGCTCCTTGTAGAGGTATCTGTTCTTGTTGCCACCGCAGCCGCCGTAATAGAAGGGCTCGCACTTGCCAGACTCCGTATTGAACCACCACCTCGGAAGCTTAGCCTTGCAGGGCCCAACTTCCGCCGTTGGAGTGCATTGTTTCTCAAAATCTGTAGCCGCACAAGCGGCGCCG |
| >MG4828264  CGCGTTTTTCCCCAGGCATTCACAGTCCAAGCGTCCAGAGTCGACTTCCAAAGCGTAGTGTAGCGTTGCTGGCTTCGCGTGCGTGTTCCTGTTCGCATCAGTCTTCCCCCCTCTCGTGGGCCTCCTTGCCCAGGCACTGCATGCGGCACTCGTCGCTGGATATGAAGTTGTTGCGGTTCCCTCCGAAGCCCAGGAAGACGAACGGGCGGCACGACTGCTTCTTCGAGTCGAAGTAGTAATGCCACACGCGCGAAGGGCCGTAGCCACGATTAGGGGGCAGCTTGCAGATTCTCGACGTAATAACGCTTGCGCATCGTCGCAGGCACTGCTTGCGGCTTTTGAAGTTATTGTCGTTGCCTTCGC |
| >MG4829138  CAACGTTCATCATGAAGCTGTACCTCATCCTAGCTTTCATTGGAGCCGCACTTGCGGATGCAAAGTTTGAAAGCCAGTGCGCCCAGAAGCCGGATCCTGGATTCTGCAAAGCTCGGCTGCCCAGGTGGTTTTTAAACACGACGACTGGCAAGTGCGAGAAGTTCTCCTACAGCGGTTGCGGGGGCAACCAGAACCGATTCCTGATCAAGGAAAAATGTGAACTGACCTGTATCACTGAAATGCAAA |
| >MG4830030  CTTTCTTCTACCAATGTGCATGGCCATGCATAGCAGCATAAGCCCGAAAGACCCTGCCGTGTGCTCTGCTCCACGTCCTGCCAGCTACTGCTTTAGAGTTGATGACATGTTCCCCGTATATTATTATGAACCCTGGAGTAGATCATGCATTCAGGAACCAGGTTGTACTCTGGAGGGCAATAACTTTAGTTCCAAAGAAGAATGCGAAGAACTCTGCTTAAAAGGAAGCGCGCAACCAGAGCCACGCCCGATGGCCTTTTTTTTGCCTTGGGGTTACTTCTAAAGTTTGTCATCAGGCACTGCAGCGACTGAGGTAGCACTCTGGATTTGAAGTTT |
| >MG4831901  AACTGGATCACCAAAACCCGTGTTCACAAGCCCGAACACGTTGCGTTCGTAATGCGTTCCTGAGGAGAAATATCAAGGAACAAATAACGTAGGCTTGAAGTTGAAAGCAACAAGGTTTTCGAGGGCGGGCTTTTTGTTGTTTCCGACTTCCATGATGTCCGGCCCCATCTGGCTGTCGCCAAAGCACGAACACATATGTGTTTATACCAAACGGGGCTCCATCCAAAATTCTTGCAGCTCAACCACAGCGCTTCTGGCATTCTTGGGGCGTTTCGAAGTTGTTCCTGTTGCCCTGGCAACCACCGTAGATGAACTGCTCGCAGGTCTTCGNNNNNNNNNNNNNNNNNNNNNNNNNNNNNNNGCCCATGCAAGAACCAGGATCCTTTGGCTCCTGGCACCTAGTATCAGAGCTTGCCAGTGCAGCACCAAGAATCGCAAACAGTACGAGTAACTTCATGGTACGTCCGTCCATAGTGGTAGACGCTAGCCCTGCACAAGTGACGGGACGAGAGCTTCAGGCCGCTGTTCACTTTTCGGGTGTTCGGC |
| >MG4832500  CTCGAACCTGTTGGCGTTGCCCTCACAGCCTCCATAGTAGAACTGCTCGCAACGACCAGCGGCCATGTTGTAGTACCAGTGCACAATGGCTGCCCGACACGGACCCTCCTGCTTGGGCAAAGCGCAAATGTCTCGGGGCACGGTCACCCTGGTGGAGTTGCACATGCGCTCGCATTGCTCACGGGTGTTGAAGCGGTTTCGGTTGCCCTGGCAACCTGTGTAGACAAACTCCTTGCAGAGCCCCTCC |
| >MG4833866  CGCGACCATCTCAGGTGTCGACGATGCTTCGACTGACTGTGCTGGCGGCTGTGCTTTTGGCCATCTCGTTCAATGGTGCCAATGCTCAGAGAAGGCCCAGATTTTGCAGCCTGCCGCCTAGTCCCGGAGTCTGCTTCGCCTACTTCCCCTCGTTCTATTATGACATTTCATCCGGGACGTGCCGAGAGTTCGTTTACGGCGGATGCCAAGGAAACCAGAATCGCTTTGTTTCATATGAGGAATGCCTCCGCGTCTGCGGCTGACATCACTTCTGGATCGGAAGTACCAATGACAGCGCTAGCATCGCATTACGCTACGTACCTTATGTTCCTGACTGAAACACCTAAGTGAGACCTGCTGTCCCACTTACAAAGTTATGG |
| >MG4836233  AAGCTTCAGCCATCTCGCGGCAGTATCATCCTCAACATGAAACTCTGTGTCATCTTCGCTCTCATCTGCACCACACTTGCGGCCACCTCCTACGAGGAGAAATGCACGAAGGACCCAGAAGTTGGTTTCTGCAAAGCTTTACTGCCGAGGTGGTGGTTCAACGTGAGGACTCACCAGTGTGAGGAATTCTTCTACGGCGGCTGCGGTGGCAAC |
| >MG4836573  CTAACCACCACTATGCGCAGCCTTACTTTCTTGGTGTTGCTGATTGTATTTTTGTGCGCCGTGCTCCTCGTAGACGGCGAGAAGAAGAAACCTAAACCGGTATGCAGCCTTCCACCCGAACACAAGAAGTGTGCAAGGCTTAAAAGAAAAGGATGTTACTGCCCGAAGAATTTAGGTATTCCCGGCTTCGTACGCGAAGAGCGCTGGTTCTACAACAAAAAGAAGAAACAATGCGAATCGTTCGCGTGGTCGACTAATGGTGGAAACTGCAACAATTTCCCATCAAGAGAGAAATGCTACGAAACGTGTTCAAATTTCTAATGACGAAATAAATAGAACACAG |
| >MG4839134  GCTCCACGCCCTGCCAGCTATTGCTACAATGGAGTCTTTTACGTCTTTTATTTTGACCCCTGGAGTAACTCATGTTTAAGGGAAGTAGGTTGTACTCTGAACGGCAATAACTTTGGAAGCAGAGAAGAATGCGAAAGACTGTGCTTAAGAGGAACCGCGCGACCACAGCCACGCCCGAGCGCCTTCTTTTATCCTTGGGTTCAGTTCTAAGGTTTGCCATCAGCCACTGCAGCGACAAAGGGAGCACTCTGGATTTGAAGAATTTCACCTTGA |
| >MG4839887  GCCTCGTATCGCATGAAGCAGGACTCTTGTATGAACTCTTCGATGGGAGCATCTGTCACAGCCTGCCTCACACACGCGCGTTCGCACTTCAGGTGTGAGGCGAAGTTGTTCTCGTTTCCGCCCGAGCCGCCGTAGTAGAAAGGATGGCAGCGGCCGTCATCGGCATTGTAGTACCAACGCTCGCGCAAATTCACGCGTAGTACCAACGCTCGCGCAAATTCACGCAGGTTCCGGCGTCCTGCGGAAGTCCGCAGATGCTCCGGTTGGGACTGACAT |
| >MG4843486  CGTATGCTTTCCCAATTTGGTTGTTATAAGTGAACCTTCCATTTCCAGTCATCAGGAGATTATTGTCCATGGTATGTATTTTCTTGCTTGAGTTACGCTCAATCGTCTAATAACACCAGCTGCGGGAATTTGAACTTGCCACATGCCTTCTGGCATTGTTCCCGTGATTCGAAGTTGTTTCGGCCACCGTAACAGCCGCCGTAGTAAAATTCTTCGCACCATTGTCCGTTGTACCACCAGCGAGGGATAAGAGCTCTACAATAAGATCTTAATACTGGGTGCTCGCTGCATACATCTTCGCACGTTT |
| >MG4844417  CGTATTGCTACTGAGAGCACAACAGCGTCGACTATGATTATAGCTCATTTCCTTATTCTTCTGGAGTTTCTGGTGTCCGCGCATGCGGCTAGTTATCGGACACCACAAAGTTGTTTGCAGTTACCTGCAGTTGGCCACTGCAAAGCAAAGTTCCCGAGATGGTATTATGATGTCTCAACAAAGCACTGCAAAGTATTTATATACGGCGGATGTGGTGGCAATTCCAACAGGTTTGAAAGTGAGGTCGAATGCCAAAAAAC |
| >MG4844989  CGCCGCTGCCGTCAGTGAGGCTGGCTCTGCTGCTGCCGCCCCTATCCGCTGGTCGTCTTGCGGGGATGCACGCATTCCGCCTTGCACTTGGCCTCTGTGTCGAAGCGGTTGTCGTTGCCGTCGCATCCGCCGTACACGAACAGCTTGCACGCGTTGGTCTTGCTGTCGTAGTACCATTTGGTGGAGGCGTCCTCGCAGTTGCCGGGGTCCTTCTTCAGGTTGCAAGCTTTCTTGTGCGCCGCATGGCACACGAGTGAAAGGGAGATGAGGAGCAGGCAGACCAGGGACT |

| >AAFF150  TGCAATCCTTCTTGACTGGAATAGCATTCGGAGAAAGGAGCCTGAAGATGGACCCTCGAACCTTCTTCTTTCTATTTGCTGTCCTGACTGTGGTGTCGGCCAAGTCGTTTTCACTCCCTCCAGAGTGCCTTGAAAAAGCCGACATCGGTCGGTGCAACAGCTTTGAACCTAAGTGGTTCTTTGATGCGGATGCAGGGCACTGCAAGCCATTTCAGTATGGCGGATGCGGGCAAAACAAAAACGTTTTTTCTAATTGCACTGCCTGCATGAGTCGATGCACTGCCCATGATGATCCCAATAGAGCATGCGAATCATATCTTGAAGTAATGTATGGCCAGCAAGGATGAAGGCAGCAAGCACCACGGCGGGGATTTTCAGCATATCTTGGTTTACACATGTGAAGATTTGGTCGGACATTAGGAGCAGTGAATTTTACAACTGATTCACTCTGTGGACCAAGCAATAAACTAAGGTTTTTCGCATTTCCGTGAGCATCCTTCT |
| --- |
| >AAFF203  TCTGTCTCACATTAGTAGTTGTGGAAGGGTACAAACCGCCAAGATATTGCAAGGCAAAACCAAATGACGGACAATGCGGGGGCGTGCGGCCTTCAATTGAAAGATGGTACTTTGATGTGAGATACGGGTACTGCGGTCCCTTCTTGTGGGGCGGATGCGGCGGAAATAACAACAACTTTCCCAACTGTACAGCCTGCATGACTACTTGTACGACTCACCCAGATCCCGAAGGCGCCTGTCGCTATATTATCAACTCCCCATGATCAAGCAAGAACACCTGACCAAGAAAATTGCCGGTCCTGCTCTTGATCAT |
| >AAFF281  GGTCTGCAATTGGCACCATGCGTTCGAAACCTGCACCCGTTTCTTCCCGGTTTACTATCTGGAAAAGTCGAGCGGCAAGTGCGTGCTGGATGTCGGAGGCTGCAGATACTATGGCAACAACTTTCCCACGTTAGCAGAGTGTCAGAATACGTGCAAAGCAGGTTAAAGATCCATGGTTATTTTCAACTGGAACTTCCATTTGAAGTGAGGTATTTGCAGCCAAACAGCAAGCAGCAAAGCTGAAATGAATAGGTGCACCGAATTTTTTCTCTTCCAGGAAAGATTCAATAAATAAGTGGCAAGGAATCCCTGTCG |
| >AAFF308  TGGATCTTTAACCTGCTTTGCACGTATTCTGACACTCTCCTAACGTGGGAAAGTTGTTGCCATAGTATCTGCAGCCTCCGACATCCAGCACGCACTTGCCGCTCGACTTTTGCAGATAGTAAACCGGGAAGAAACGGGTGCAGGTTTCGAACGCATGGTGCCAATTGCAGACCACCGGAGTGCGACAGGTTCCGTCGCATTCTCTTCTGGTTACAAATGAGCCTCGCCCACATGTCCATGCGCATTTCTTCTTAGAAGCGACATATACGAAGCTCTTATGCTGGCAAGGTCTATTTTGCAGC |
| >AAFF646  GCAATGATCAAGAGCAGGACCGGGAAATTTCTTGGTTAGGTGTTCTTGCTTGATCACGGGGAGTTGATAATATAGCGACAGGCGCCTTCAGGATCTGGGTGAGTCGTACAAGTAGTCATGCAGGCTGTACAGTTGGGAAAGTTGTTTTTATTTCCGCCGCATCCGCCCCACAAGAAGGGACCGCAGTACCCGTATCTCACATCAAAGTACCATCTTTCAATTGAAGGCCGCACATCACCGCATTGTCCGTCATTTGGTTTTGCCTTGCAATATCTTGGGGGTTTGTACCCTTGCACT |
| >AAFF916  GGCGGCCAAACCCACACGATTCACGATGCAGATCAAGCCTCCCGGTGCTGAAAAGTAATTGCCCTCACCGCAGCTACGCATTCAACCGTAATACCAAGCAGTGCACATGGACATGCGGCCGAGCTCCTTTTGAAACCCAATTAGAATGCGATGTTACCTGTCGAAGTGTTGCCGTCTGCACTGCTCCGAGACCGTATGCAATTTGCAGCGGGAGGACATTTGCAGTTTACTATTTTAATCCTGGGACCGGCATGTGCCACGCGGATATGGGCTGCAGCTACGCAGGAAACAACTTTCCAACCTTAAGCGAATGTCGGCGCACTTGCAAAGCAGGATAATAAGAAACAGACGTTTTTGTCCAGGGAACTCAATTGAAAAAAAGACAAACAGGGCTACAGAGTAGAGAGGAAATGTGAAAATATTTGTCTGCTACGCATTTCTCTTATCCTCG |
| >AAFF980  TTTTTTCAATCTAGGTACTTTTATTTTGGCTGAGAAAAATTTCCCGTATGTCTTACTTTGGCTGGAGTATTGCCCCGATAATTCCCATTAAACACAGCTCGTGGTTGCCCACTGAGCTCTCCCTGCCGAAGATGTTATTTCGCCAGGCGGGCAGTTCCATACTTCTTGTTGAATTCCTCCTTCAGCTTCTTACAGAGGCGTCGTGGATTCGAACCCCTGGCGCATGTTTTCATGCATTTTTCACAGCTTGCAAATCTGTTGCCATTTCCTCGGCAACCACCATACACGAATCCAGTGCAGCGACGTAATTCAGAACTATAGTGCCACATTGGTTGATAAGCCCTGCAAGGCCCTGGATCTGCTTTTTTCATACAAACTGCACGATCTGGACGTCTTGCGTGTGAGAGTCCAAGGCAGAAGAGGGCTAGAAGAAGACAGACTTGTACTTTGACCATAATGCTCACACCTTAAAGCTCTGTGAAAAACTCTTGTTCGTAATGCCACTGATGCCACTCACCTGACAGCCTA |
| >AAFF1019  AGAGCAGGACCGGGAATTTTCTTGGTCAGGTGTTCTTTCTTGATCATGGGGAGTGAGAATATTGCGACAAGCGCCTTCGGGATCTGGGTGAGTCGTACAAGTAGTCATGCAGGCTGTACAGTTGGGAAAGTTGTTTTTATTTCCGCCGCATCCGCCCCACAAGAAGGGACCGCAGTAGCCGTATCTCGCATCATAGTACCATCTTTCAAGTGTAGGCCGCTCGTCTCCGCATTGTCCGTCATTTGGTTCTGCCTCGCAATATTTTGGGCGTTTGTACCCTTGCACTAATACTACTGTGAGACACAGCAGGG |
| >AAFF1286  GAGACGTGCAACAAGGACGGCTCGTTCGACAGCAAGCTTGCTTGCGATGGATACTGTCGTAGTGTTGACGTGTGCACGGCTCCACGTGCTGTGTCCTCCTGCGCCAGCGATATACACCCTGTTTTCTACTATGATCCCTCGACACGCAGCTGCCTCAGGGACATGGGGTGCATATACTATGGAAATAACTTTCCCACCATTGAGGAATGCCAGGAAACGTGTATGAGACGTCGACCGAAGCCAACGAAACCTCGGGAGTGTTTTGTGTCTCCGACTCAAGGGTACCCTTGTCGATGGGGTTTCGTCTCAATACGTTTCTACT |
| >AAFF1287  TTTCTCATGCGACGTGCTCCTGCAAGTACATTGACAGGAAAAGGCTTCAAAATAACCTGCTCTCCATATGCAATCAAAGCACCGCAGTGAGCTTATAAGAGAACGAAGAGGCAAGAACATTTCCCAAGCAAGCTTCCACTTCGCTGAAAAGACATGGAAATGTACCCGGCGTTGGCAGTTTTTCTTTATTTAGGGCTCTGCGCAGCAACAAGCCGATCCACGAGAGATCCAAGATGCATCTCAAACAAACCAATCGTTAATACAAGACGATGCTCAACACCTTCATGGCAGTTCAACTTAATTGAAAAGAAGTGTAATGAGACGTGCAACAAGGACGGCCCGTTCGACAGCAAGCTTGCTTGCGATGGATACTGTCGTAGTGTTGACGTGTGCACGGCTCCACGTGCTGTGTCTTCCTGCGCCAGCGATGTACACCCTGTTTTCTACTATGATCCCTCGACACGCAGCTGCCTCAAGGACATGGGGTGCATATACTATGGAAATAACTTTCCCACCATTAAGGAATGCCAGGAAACGTGTATGAGACGTAGACCGAAGCCAACAAAACCTCGGGAGTGTTTTGTGTCTCCGACTCAAGGGTACCCTTGTCGATGGGGCTTCGTCTCAATACGTTTCTACT |
| >AAFF1535  CGGTGCAAAAAATCGGCCAATTGTGTGATCGGAGCCAATGCCTTCGTCACGAGATCAGAATGCGTCTCGATGTGCAGAGCAGTCGACGTGTGTGATGCACCGAGGCCTTTGGAACTGTGCGACAGCGGACAGAGGACCGTGTGGTACTACGACCCAACCTCCGGCCAGTGCCTGAAAGACGTCAACTGCCATAACCGGGGGAATAATTTCCCAAGCCTACAAGAATGCCGCAGGGTCTGCGTAAGACGAGTCTCGCCGGTACCAATGC |
| >AAFF1536  AAACTCATCGGTGCAAAAAATCGGCCAATTGTGTGATCGGAGCCAATGCCTTCGTCACGAGATCAGAATGCGTCTCGATGTGCAGAGCAGTCGACGTGTGCGATGCACCGAGGCCATTGGAACTGTGCGACAGCGGACAGAGGACCGTATGGTACTACGACCCAACCTCCGGCCAGTGCCTTAAAGACGTCAACTGCCATAACCGAGGGAATAACTTCCCAAGCCTACAAGAATGCCGCAGGGTCTGCGTAAGACGAGTCTCGCCGGTACCAATGCCGAGTCCCGTGTGCTACTCCTTCCCGCCAA |
| >AAFF1537  AAAAATCCACATTCATGTGAAGTGAAACGCTTAAGGTTATCACAGTGAATATTTTAATAGGTCAGAAAAAATTTTATTTTTTGTGTCCACGTTTCCATCATACAAAATATTGACGTCAGCAGCCTCGGGGAATGCGTTTGTGGGGAAAGCAGACTTCCAGGCATTCTTCCTTCGAGTCAAACAAGTTTGGTCCGCCGTTGCAGCCGCCGTATAAGAACTGAAAGCAATTCTTGTGGTAGTGGTCGTAGTACCACTTGAAAGCAAATGCGTAACATGGTCCACGGTTTGGCCAGTGACGACAGTCCTCCGGGGATCTTCGTCGTTCGCAGACCTTGCGGCACTCCGGCATGCTGGGGAAGTTGTTACCGCGATTGTGACAGCCCGATCTTTTCTCGCACAATTTCGTGTCCTGGTTGTAGGAGTACACGCTGCGCTCCTTCTCGGAGCACGAATGGGGTAGGGGGGGTTGGCTGCACACATCTCGGCTCCTGCACACTTCACTGCACAGCTGCCTTGAGGAAAAGGAGTTTGCCCGCGCAAGGTGACAGCCAACACCCAGGCGGCATTCCCTGGTGTGTCTGTCGTAGTACCACGTGTGCCTTGACGGCGCGTAGTTGGCACAAGCAGGGTCCTCGGTTGGTCTGGGAAAGTGGCAGAAGGACTCTGACGGGCATGTTTTTTCGCAGGTGCTCCTCTTCACGAAGCTGTTGCCAGCCTTAGGGCAGACGAACCGGCGTTCGCACCTCTTGGCGTCCAAATTGTAGACCCACCTCTCCATAAGGCGTCCACAGCCGTGGGTTGGCGGGAAGGAGTAGCACAC |
| >AAFF1687  TTACAACAGCCGTTCTCTCATGTCTTGCAGGCTTTGTGTTCGCCCAATGCATGTGCTGAATGAACACGGCAAAAGAGAGAGCGAAGTGCCCATTTCATTTGTCCAGCAGGCTACGTAAGCTTGCAGGCAATCTCGCACTCTTCCTTCGACTCGTAGTTGTTGTCGTTTCCACCGCAGCCACTGTAGAGGAACGTCTCGCACTCTCCCGAGGTCACGTTGAAGAACCAGTGATCTAAGAAGCCCTTGCAAAAGCCGCGTTGCGGTTTTGGCGCGCAACCAGTCTCGAAGTCAGCTCCCTCGAAGTCACTGGGCCTCTCTGGTTCGCCACATGTTTTTTCGCATTCCTCGATAGTTTCGAAGTTGTTTTCGTTTCCTTCACAACCACCGTAGATGAAGAAGGAGCATTTTCCTTCAGCTGGGTTGAAGTAGAACCGCGGAATGCTTGCCCGGCAGACGCCTTCGTCTGGCGGCAGCCGGCAGATGGCGTTTCGCTGCGCGAAAGCC |
| >AAFF1989  TTTTCGTTTCCACCGCAGCCGTTGAAGCTGAAAGCTTTGCAAGACGTTCCGTCGAAGTGCCAGCGAATCTCAGTTGTCTTAGTTGAGTTTTTGCATGATTCGTCTCCCTCATCCTTCTCCAGTTTGCAAACTTTCTTGCTTTTCGCTGTCTGTGACTCAACACATGTACCCGTGAAGATTATGAGTGATAGAACGGCAAGTTGGCGCATTT |
| >AAFF2100  AGAGCATTCTAAATTTGTACACATACATTTTCAGATTTTAGTCGATAGTAGAACATAATCCGTGTTTGAAAGGATAATAATTTAGTGCATTTTATGAAGAATCCGAGCAGCCGTCGCACCCTTCGTTTGAAAGGAGCCAAAATAGCATTTTAGAAGGTTTATTTAGGCAGCAGCTGGTTGGTATTTTATGTGGCTACAAATGCAGCACAGGTATTTCAGCGCCAGCTATAGTCACAGCTTTGTGGGATGTCCCTGCCACCTGATGCGCATTCGCCGCTATGCAAATTACTTTTTCGGAAATGGTGGCGTTAGTTGAGCAGGAGGAAAACAGTTAAAATATGTAAGCAAAGAAATGGTCTGCTCGAAATTATTTACTTCACGCGGAGACATGGGGGAGAGCCCATTCCGAAGAGTGTCAAGCCAGATACATCGCAAACGAACAAGAGGCTGAGTAAATCAGCTTTATTAATCTGCCTCTTACACTAGTTATATCCTGCATGAATTTCCCGAGATCTCACAAAATTTTTCAGTTATTTTTAAATTGAATGTGTACGCATATGTGTTATAATCTTTTAGGTCTAAGAATGTTTAGCACCTTCTTTTTCGAAAAGCAGTAAGTGCAACATGTACGTCTATGCGTGCCGGGGCATCGGCTCCCAAGGAGACTGAATGGCGCAGGCGTCCATGCACTGGCGCAGGGTCTCGAAGTTGTTCCCGTTGCTGTGGCAGCCTCCGTAGATGAACTGCCGGCATTGCTTTGTTTGAGCATCGTAGTAGAAGCGCGGGATGCTAGCCATGCAAATGCCGGAGTATGCAGGGCGACGGCACACATCAGCCACGGATGCGAACAGTCCACTGCGCACTCCCCAATGCCTTTCGGGCCAGAAAGAAGGTTTACTCGCAGAACACGTCATTTCACACTGTCGCTTGCTCTCATATCGGTTCTCGTTTCCTCCGCAGCCGCCGTAGTTGAAAAGCTCACACTTTCCAGTCCTGGCATTGAACCACCACATCGGCATCATCGCCTTGCAGGGACCGCTATCGGGCAGCTGGGTGCATCTCATGTCAAACGCAGTCGCTGCGAAAGCGGCGCTGACCACGGCAAGCAAAATGAAGACCTTCATGATGGCCACAAGTGGGGACAGCACGATGGAA |
| >AAFF2240  TTTTAATGTCCATCTAGCCACCGAAAGCTGAGTTTATTTCAAGTGCGCGAACTTTTCCTGAAGTGCCGAGAGAGAAATGTGTTTCCAGTACTCAGCACACTCTAGATTACGCTGATTACTTCGGTCTTCTTGCATAGTTTGGCTTGTAAGGGCCGGTGTTTGTACCAATTGGCTTCTCCAGCTTCCTGCAAAACTTCTTCGCTTTCCGCCAGCCCATTCCACCGCATTTCTTCATACACTCGTGGCAGTGTTGAAATTTATTCTCATTTCCTCCACAGCCACCGTAGGTAAACAAAGTGCAGTATCCTCTGTAAGCGTCATACCACCACATCGGTATCGATGCTCTGCAGGGTCCAACTTCTTTTCGCTGGGTGCAGACTTTTTTTCTGTTAAACCAGGCATGTGAAAAAGCTACGCCGACGAGAAGCAGAAGCAGCCACGCTGGTAATCTGGTCATTGCGGTCATTTTTTGAAGCTTTGCTCAGAAGGCGCCTTTGATGATATGCGAGTGGTCCAATTCACTCCTTCCAGCCTTTTATTTGCGAATTTTCTCGCTGAATTCCTAGTCACCTGACTCAATACTCAATCATGTCGTGGACATTACAATAGAAAATCCGTATTAACACAGTACATTCAGCTCCATGTTGTTGGCAGGTTTGGGTCTATACACAAAATTCACATTTACAAATGCATGATGTTTTCAGTTTCAGGGC |
| >AAFF2716  TTTGCTTTTTCAATAGTTTATTCCTTGTTCCCGAGGATAAGAGAAATGCGTAGGAGCCAAATATTTTCACATTTCCTCTCTGCTCTGTAGCACTGTTTGTCTTCTTTTCAGTTGAATCCCCTGGACAAGAACGATTATTTCTTATTATCCTGCTTTGCAAGTGCGCCGACATTCGCCTAAGGTTGGAAAGTTGTTTCCTGCGTAGCTGCAGCCCATATCGCTGTGGCACATGCCGGTCGAAGGACTAAAATAGAAAACTGGAAATGTCTTCCCGCGGCAAAGGGTATACGGTCTCGGAGCAGTGCAGACGGCAACACTTCGACAGGTAGAATCGCATTCTCTTTGGGTTTCAAAAGGAGCTCGGCCGCATGTCCATGTGCACTGCTTGGTCCTACGGTTGAAAGCGTAGCTGCGGTGCTGGCAATCATTTTGCACCGCCGGGCGGCTAGATCTGCATCGCGAATCGTGTGGGTTTGGCCGCC |
| >AAFF2717  GAATTTATTACTTTTCCCAATCATAAGAAAGATATAGAAGGCAAGGTGGTCAAGTATCCTCCGATTTTCTTCTTCCTCAGCAGCCTTTTCGTCTTTCTTAAAAATGAATTCCGAGGATGAAAAAAATTTTTCATTCTTTTCCTGCTTTGCACGTGCGCTGGCATTCCCCTAAGGTTGGAAAGTTGTTTCCTCTGTAGCTGCAGCCCATATCTAAGTGGCACTTGCCGGTCCTATTATTCAAATAGTAAACAGGAAATGCCCTGCCCTGGCACTGAACAAATGGCCTGGGCGCAGCGCAGACGGCCGCACTTCGACAGACTGAATCGCACTGTACTTTGGTTACAAAAGGGCCTCGGCCGCATGTCCAAGAACACTGTTTTGTAGAACGGTTGAATACGTAGCTGCGGTGATTACAATTATTTTGCAGTGCTGGGCGGCTTGATTTGCATCGCGAATCGTATGGTGTTGGCCTCCGGGCGCTGCATAGGGCCAATAGACCAAGCAACAGAAACGGCTTCCAGATGGCCATGATGGAACCTGTGTTTTGCCCTGTGCGCACTGGAACATTTATAATCATCTAGGCGTATTGTT |
| >AAFF2721  ATCCTTTAATTAAAACATACATATTTATTATCCAAATACAGCCAGGATTTACCTCCCGTGTAATTACTGAAATATGTAAACTACTTTCACGTCTAGCTTTGCTTCCAAAATGGTATTATTCCGGGGAGCAATTTCTTTTATAAATGATTTCGATAGATTACGGCATCCATATCCGCAATGAGGAAATAATTTTGTGGAAACTGCGTGCCCATCGACAAGTGCAGGTGTCAATAAGTGAACTCTTTCCCGTTTACTCTGTGCCTGGGCTTGCACTCTCTGATGCATTTCTGCTGGGAACGGAACAAATTTCCCTTCGGCCAAACGTTTTTTGCCACAATGTTGAGCACAGTTTCGTAGCAGAAGTCGTCTTCAGAGTCATAATAGTACAACGGTTTCTTTTGTTTGTTATGTTGCAATGTTGGTGTATCATTCTCCAAGCAGACCGAGCTCTGATTGCATGCTTTCCAACACTCCTCCCGTGTTTCAAACTCGTTGCCGTTTTTCTGAAAGCAGCCTTCACGTACATCGTAATCCACGCATTTTCCTGTCTTTTTATTGTAAGCGTAGCGCTCTATCAATTCTCCGCTGCACCCATTCCTCTTTGGCTTGATTTTCATGCTGCATCTTGGATCTTGAGGGAGGGCTCTAGATAACACGTCGTCTCGTTTCTCCGCTTTCGGCGCAGTTGCGTAGCAGTCCAGAGTCAAGCCATACAAGCACAGCAGTTGAAGAAAAATACTCAAGATCATTGCTTTTGCTCCGTTTGCAAAAAAAAAAAAGTGGTAAGTATACGCTGTTCCTGGCC |
| >AAFF5138  CAGCCTGTTTTTTGGTCAAAACGCGCGCGTCGCCTGCGGCAGTGCATTTCGAGCTGGTGCTGCCTTGAGGGTATGAGCCCTAATATCGGCCGCAGACTCTGCGGCATTGTCCAACAGAGTTGAATCTGTTCGCGTTGCCACCGCATCCGCCGTAGGTGAACGGGAGGCATCTTCCCGATCTACTATCGAAGTAGAAGGCTGGGAAGGCGCCGCGGCAGGGTCCCTGGCTAGGATGAAGGAAGCACTGGCCTTGAGCATTGGTGCCTGCTACGACAATAGCCAGCAGCAAGGCGACGACCACGAGCGAACGAGGCATCTTGAGAACGGGCGGTCCCGGTGGG |
| >AAFF5139  CGGCAGTGCATTTCGAGCTGGCGCTTCTTTGAGCGTACGAGCCCTAATATCGGCCGCAGACTCTGCGGCATTGTCCAACAGAGTTGAATCTGTTCGCGTTGCCACCGCATCCGCCGTAGGTGAACGAGAGGCATCTTCCCGATCTACTATCGAAGTAGAAGGCTGGGAAGGCGCCGCGGCAGGGTCCCTGGCTAGGAGGAAGGAAGCACTGGCCTTGAGCATTGGTGCCTGCTACGACAATAGCCAGCAGCAAGGCGACGACCACAAGCGAACGAGGCATCTTGCGAACGGGCGGTCCCGGTGGGTGCGTTTCTCACGAGTGAGGATGCTGGGCAGCCTTGAGTCGGTCAAATATGCGCGCGGCGGCGTCCTGCCGAGCAGCAACGGCACCTTCGAGATTCCGGTTTCCTGGGCGCGCAGCGGCGCACGTGTGGTCGGTTGGA |
| >AAFF5280  TCGAGGAATGCGAAAAAACATGCGGCGAGCCAGAGAGGTCAAGTGACTTCGAGGGAGCTGACTTCGAGACTGGTTGCGCGCCAAAACCGCAACGCGGCTTTTGCAAGGGCTTCTTAGACCACTGGTTCTTCAACGTGACCTCGGGTCAGTGCGAGACGTTCCTCTACAGTGGCTGCGGTGGAAACGACAACAACTATGAGTCGAAGGAAGAGTGCGAGATTGCCTGCCAGCTTACGTAGCCCGCTGGACAAATGAAATGGGCACTTCGCTCTCTTTTTTGCCGTGTTCATTCAGCACATGCATTGGGCGAACAC |
| >AAFF5281  GCTTTTGCAAGGGCTTCTTAGATCACTGGTTCTTCAACGTGACCTCGGGCGAGTGCGAGGCGTTCCTCTACAGTGGCTGCGGTGGAAACGACAACAACTACGAGTCGAAGGAAGAGTGCGAGATTGCCTGCAAGCTTACGTAGCCCGCTGGACAAATGAAATGAAGACTTCACTCTCTCTTTTGCCCTGCTCTATCCGCACATGCATTGGGCGAAC |
| >AAFF6176  ATTTCCCCCACAGCCGCCGTAAATAAAAACCCTACATTCTCCTTTGAACTGGTTAAAATACCACTTTGGAAAGTAGGCTCTGCACACTCCAGACTCTTTTTGAAGCCTGCAGACTTTGTTTCGGGCACGTCCTTGGGAGACAGAGAGGCAGAAGAGGAGGATAAGAAGGTACAGCTGCAATCTCGGAATTGATTTCATCTTTCAGATTTGGCGCTGCCCTGGGCTTTCTTGCGATACCGCTAAAAATTGACAGTTCAACGCCGCCTGTTACTTGCGGCAGTCTATAGGTGAATTCCCTGTTATGGGACTGAATGCGCAGCTATTCTGGGAGCGTCGCAAGAGGAGAGCACGGTTGGCACAAGTATCGGCGCCAAATCTGAAAGATGAAA |
| >AAFF6560  TTTTTTTGAAGTGAACAGCTCCGCCAATATTTATTTGTTAGTATGCATTCCCAATTGTGTTGTTACACGAGAGCCTTCTATTTCGTATCATCGGAAGATGATTGTACGTAGTATCAATCTTGTGCCTTCTCTTCTGATGAATCGCCCAATAACATCAGCTGTGTGAAATTGAACCTGCCACATGCATTCCGGCATTGATCCCAGGATTCGAAGTTGTTTCGGTTACCGTTGCAGCCGCCGTAGATAAATACTTCGCACCATTGACCGTTGTACCACCAACGAGGGATATGAGCTCTACAAGGTCCTTCGACTGGGTCCTCCCTGCATATATCTGCGCATGCTGTGTTGTTCAGACCTTGACTGCATATTTTCGCCCATTTTTCTGCTTCGTCTTCGAAGGCCGCACCTCCTAGAGAAAGAAGAAGAGTGAACAGAAGGAACTTCATTTTCA |
| >AAFF7505  CGTATGCTTTCCCAATTTGGTTGGTATAAGTGATCCTTCCATTTCGAATCATCAGGAGAATATTGTTCATGGTATGTATTTTCTTGCTTGAGTTACGCTCAATCGTCCAATAACACCAGCAGCGGGAAACTGAAGTTGCCGCATGCCTTCTTGCATTGCTCCTCGGATTCGAAGCTGTTCCGGCCACCGTTACAGCCGCCGTACATAAAATTTTCGCAATATTGCCCGTTGTACCACCACCGAGAGATAAGAGCTCTACAAGGTCCAGGGACCGGGTCCTCCCTGCATACATCTTCGCACGTTTTGTTGTCCCCACCCCTACTGCATATGTCAGCCCAATCTACTGTGTCGTTTCTGCTGACCACACCTGTGGCTGGAGATAAATTTCTCGCC |
| >AAFF7581  CAGGAAACGTGTATGAGACGTCCACCGAAACCAACAAAACCTCGGAAGTGTTTTGTGTTTCCGACTCAAGGGTACCCTTGTCGATGGAGTTCCGGCTCAGTACGTTTCTACTACGAACCTCGCATCGGCCAGTGCATACCTTTTTGGTACTGGGGCTGCGGAGGGACCGCAAATAATTTCTCCTCCTTCAGACACTGCATGAAACATTGCGCAAAGCACTAGTGTCTGCTCGAAGTATTTGATTCTTTCTACTGAAACTAGGGAGACATGCCGGAGAGTTAA |
| >AAFF8679  GGAACCTTGGGCAGGATGGGGTGGCGTATATATTTAAACGCAGCCGGGATGCCTGCCACTGAACTCCACCGGCGTCCGCGACCATCTCAGGTGTCGACGATGCTTCGACTGACTGTGCTGGCGGCTGTGCTTTTGGCCATCTCGTTCAATGGTGCCAATGCTCAGAGAAGGCCCAGATTTTGCAGCCTGCCGCCTAGTCCCGGAGTCTGCTTCGCCTACTTCCGCTCGTTCTATTACGACATTTCATCCGGGACGTGCCGAGAGTTCGTTTACGGCGGATGCCAAGGAAACCAGAATCGCTTTGTTTCATATGAGGAATGCCTCCGCGTCTGCGGCTGACATC |
| >AAFF8862  AGAGTTTTTCACAGAGCTTTAAAGTGTGAGCATTATGGTCCGAGTACAAGTCTGTCTTCTTCTAGCCCTCTTCTGCCTCGGACTCTCGCACGCAATACGACCACGTGTCCCAGTCTGTAAGCAAAAAGCAAATCCAGGGCCTTGCAGGGCTTATCAACCACAGTGGTACTATAGTCCCAAATTACGTCACTGCACTGGATTCGTGTATGGTGGTTGCAAAGGGAACGATAACAGATTTGGGAGCTGTGAGCAATGTATGAAGAGATGTGCCAAGGGTTCGAATCCACGACGCCTCTGTAAGAAGCTGAAGGAGGAATTCAACAAGAAGTATGGAACTGCCCGCCTGGCGAAATAACATCTTCGGCA |
| >AAFF11214  TCCAAACTGCACACTAAGAAACAACCAGATAATATCTGTCGGAACAACGCCATCTATATATAAAGCCACACAGATTGACCGTTTTCAGCTGCGTTCCCGATTTCTACGAAAAAAGGAGCGAAATCGACTATGAAGCTCATAGCCATACTCAGCGTGCTGCTGCTCGCTGCGACCTTCGTAAGCGGGTATCGTCCCCCAAACCCAATCTGCAGAGAACCTGGGCGGAAAACTGGACGCTGCAAAGCTTTCTTCCTCAAGTGGTCATATAACCCAAAAAGCGGCTTGTGCGAAGCATTTATCTACGGCGGATGTCGAGGAACTCGGAACAGATTTGACAGCTGCTATGAATGCATGAACATTTGTGCTAAAAAATTTACTAAGAAAGACAGAGAGTATTGCCATCAGTTGACTGAAAAAGCCAACAAGAAATACTTCCCGACGGCCATGCCGAAATAACCGGAACCGCCAAGCTTAATTAAGGTTGTCAAAATACGCGACAATCTTCTTTCTCGAAAAGTACTTCAGCCAGTTCATTAAACTGTCTAGTGCTTGCGAAAGCGAGAAAGTGGTGCACGCAAATTTTGGATTATAGTTCTGCCCCTGGCACCGGAAAATAAAGGATGACT |
| >AAFF11250  CGAGTTCCTCGACATCCGCCGTAGATAAATGCTTCGCACAAGCCGCTTTTTGTGTTATATGACCAGTTGAGGAAGAAAGCTTTGCAGCGTCCAGTTTTCCGCCCAGGTTCTCTGCATATTGGGTTTGGTGGACGATACCCGCTTACGAAGGTCGCAGCGAGCAGCAGCACGCTGAGTATGGCTATTAGCTTCATAGTCGTTTTCGCTCCTTTTTTCGTAGAA |
| >AAFF11255  AAATGCGCGTACTGCTAGTCGCGGCTCTTCTCTTCACGGCGTGTGCGGCCTTTTTCTACAATGAAAGCCCTGGTGCGACAGTATGTTTTAATCCTGACAGACAAGACGAATGCAAAGAATTATGCAGAACGGTGCCCGAGGACAGTCCTTGCAGAGCCCTGATTCCTGCTTGGTACTTTAATGGACAATCATGCCAGAAATTCCATTACGGAGGCTGTGGGGACGACTTAAATAACTTCGATACCAAAGAGGAATGCATGAAAGCATGTGGCAATTACACGTACCCACGATTAAACATGACCTGGGAAAACGATTACCCTGATAACCACAACAACCGCACACAGTAAATTAGAGGTGACTTGGATGAGCAGCAGCTGTTAATCTGTGCAAAAAGTATCCTAAAATAAATTCGGTTTGAGCAGT |
| >AAFF11430  GCCTGTATAAAAAAAAGAAATTAACAAACAATTCAGGCCGCAATTTATTCGTTGCGTATGCTTTCCCAATTTGGTTTTTATAAGTGAACCTTCCATTTCCAGTCATCAGGAGATTATTGTCCATGGTATGTATTTTCTTGCTTGAGTTACGCTCAATCGTCTAATAACACCAGCTGCGGGAATTTGAACTTGCCACATGCCTTCTGGCATTGCTCCGGGGATTCGAAGTTGTTCCGACCACCGCTGCAGCCGCCATACGTAAACTTTTCGCAATATTGCCCGTTGTACCACCAACGAGGGATAAAAGCTCTACAAGGTCCTTCGACGGGGCCCTCCCTGCATACATCTTCGCACGTTTCGTTCTTCTTATCCCCACTGCATATTTCAGCCCAATCAACTGCGTCCTCTCTGCTGGCTACACCTGCGCCTGGAGAGAAATTTCTCGCCAATGGTCCCAAACTTGCATCCAAAGAGTTCGGTGTCACCGCTGCCGCGATTGCTCTGCCACGACCAGGATCCACATCTCCTA |
| >AAFF11495  TGTGTGCGGTTGTTGTGGTTATCAGGGTAATCGTTTTCCCAGGTCATGTTTAATCGTGGGTACGTGTAATTGCCACATGCTTTCATGCATTCCTCTCTGGTGTCGAAGTTATTTAAGTCGTCCCCACAGCCTCCGTAAGTGAATTCCTGGCATGATAGTCCATTAAAGAACCAAGCAGGAATCAGGGCTCTGCAAGGACTGTCCTCGGGCATCTTTCTGCATAATTCTTTGCATTCCTTTTGTTTGTTAGGATCAGAACATATTTTCGCACCAGGGCTTTCATCGTAGAACCAGGCCGCACACGCCGTGAAGAGAAGAGGAGCGACTAGCAGTACGCGCATTT |
| >AAFF11538  AAGAAGTGGCCAACGGTTGCTGTTTGGCTTCAGTTACCTCAGTGCCGCAGCTTGCAATATGAGACCACAAGCTTTCATAGGTGCTTTCGTCTTTACTTTGGTACTCAGGCAAGCTGCAGGTATAAAGTGGTCGCGTTGTTTCCGACCGAAGGCTGTGGGGAACTGTCAAAATAAGGTTCCGGCCTGGTACTATGATTTTTGGAGCTTCCGTTGCAAGGGGTTCCTCTACAGCGGATGTGGCGGGAATTCGAACAGGTTTCCAACCGAAGAAGAATACTGGTAAATGCAAGGCCGCTATTCCATTATGGTACTACGACCCCGAATTGGACGAATGCCGTGGCCTCATTTACGGTGGCTGCAAAGGAAACGCCAATAGGTTTGAAACTTGTCTCAAGTGCATGAAACGATGCAGCGGAAATAACAACGCAAGAAAAATATGCAAGAAGCAAACTAAAA |
| >AAFF12081  ATTTCCCCCACAGCCGCCGTAAATAAAAACTCTACATCCACCTTTGGACCAGTTAAAATACCACATTGGAAAGTAGGCTCTGCAGACCCCAGTCTCTTTTGGAAGCCTGCAGACTTTGTTTCGTAGACGTCCTTGTGAGACAGAGAGGCAGAAGAGGAGGAGAAGAAGGCACGACTGTAGTCTCGTAAATGATTTCATGTTTCAGATTTGGCGCAACGCTGGG |
| >AAFF12395  CCTCATGGAGAAGGAGATGTGCATCGCCGACATGCCCACCTGCAAGGACGCGCCAGAGGTGCTGGACCCCAACTGCGCGGTGACCCAGTGGGCCGAGTGGTCCCCCTGCACGGCAACTTGTGGCAAGGGGATCAAAGTGCGCACGCGAGCCTACCTCAATGCGATGGCGGCCGCCATGGCCATGTGCAACGTGGAGCAGATACAGAAGGCCCCCTGCATGGCCGAGAACACCGACTGCAAGATCGACAGCCAGGAGGCGTACGAGATCTGCCTGCTGCCCAAGGACATCGGACCGTGCCGTGGCTACTTCCCGCGCTGGTACTACGACTCGACCAAGCGCATGTGCCTGCAGTTCGTCTACGGCGGATGTCGAGGCAACAGGAACCGCTTCGAGAGATACGCGGAGTGCAACAAAATGTGCGAGGTCACAATATCACCGCCTATCGGCAAGCTAAACGGCCTGTCAGCAGTGAACGTGCTTCCGACGACAAGCGACGAACCGACCTCTCCGGTCATCGACTGCGTGCTGACGCCGTGGTCACAGTGGGGTCCCTGCTCCAAGACGTGCGGCAATGGACGCCGGGAACGGCGACGCATGATCAAGCTGAACCCGCAGAACGGCGGAAAGCCGTGCCCCAAGCGACTGGTCCAGAGACGAAAGTGCAAGGAGAACCCACCATGCCCGGTGGACTGCATGCTGACGCCGTGGAGCGAGTGGCGTCCCTGTTCCAAGACGTGCGGACCAGGCGCCGTGCAAGAGCGGCACCGCACCATCAAGCGGCACCCGAAGAACGGAGGTTCCAGCTGTGACGCCACTTTCGAGCGGCGCTACTGCACGCTGCCACC |
| >AAFF13810  TACGGCGGCTGTGGGGGAAATGGCAACAAATTTGACACCTGCGAGAAATGCATGCGGGTCTGCACAGGAAAACCCCGGCAGAGAATCAAGAGGATTTGTCGAAAACTGGAGAAACTAGCGAGTGCGAACCTCAGACCGTGGGGTGCGCCCAACGCAAGACGATTCTAATAACGGCCACCTTCTAACATTTATAAACTGCTAGCATCAATTATGTCTAGCCATAGCATAAGAAAGCCTCTCCAGCACAATAAATGGTTGTCG |
| >AAFF13811  TACGGCGGCTGTGGGGGAAATGACAACAAATTTGACACCTGCGAGAAATGCATGCGGGTCTGCACAGGAAAACGCCGTCAGAGCATCAAGAGGATTTGTCGAAAACTGGAGAAACTAGCGAGTGCGAACCTCAGACCGAGGGGTACGCCCAACGCAAGACGATTCCAATAACGGTCACCTTCTAACATTTATAAAGTGCTAGCATCAATTATGTTTAGCCATGTGCATAAGAAAGCCTCTCCAGCAAAATAAATGGTTGTC |
| >AAFF14737  TTTAAAGCCATTTTGGCAAGCCAAAACGTCTAGTCTTGAAGCTGGAGCAAATAATCATCGTCTTCATCATTTTCTTCGCTTTTTATCGAAATTGCAGGCAGAGTTTTATTGGCAATATCGTCGAATATCCAATAATCCGTTACTTTGAAACAAACTTGAGCAAGACCATCATTTTCATTTTTCTTGCTTTCTATCGAAATTTCAGTAAGATTCTTCTTGGCAATATACTCCATTATTACTTTGAAACAAACGTATTTCGGTGATTCGAAGTGAATGTGCGTTTCCTCCTCGCACAGCCTTTGGCAGTCGTCTATCCTGCCGAAATTGTTTTCGTTTCCACCACATCCGTAGAAGCGGAAAGCTTTGCAAGACATTCCGTCGAAGTACCAGCGAATCTGAGTTGTCTTAGTTGAGTTTTTGCATGATTCGTCTCCCCCATCCATTTTCAGTTTGCAAACTTCCTTGCTTTTCGCTGTCCGTGACTCAACACACGTACCCGTGAAGATTATGAGCGACAGAACGGCAAGTTGGCGCATTTCTGAGCGAAGTGGGTTCCTGTTGACTCCCTTTCGAAAAATCCG |
| >AAFF14934  TTATTTCCCAAATACGTGCGACGACCGTTAACTGGATCACCAAAACCCGTGTTCACAAGCCCGAACACGTTGCGTTCGTAATGCGTTGCTGAGGAGAAATATCAAGGAACAAATAACGTAGGCTTGAAGTTGAAAGCAACAAGGTTTTCGAGGGCGGGCTTTTTGTTGTTTCCGACTTCCATGATGTCCGGCCCCATCTGGCTGTCGCCAAAGCACGAACACATATGTGTTTATACCAAACGGGGCTCCATCCAAAATTCTTGCAGCTCAACCACAGCGCTTCTGGCATTCTTGGGGCGTTTCGAAGTTGTTCCTGTTGCCCTGGCAACCACCGTAGATGAACTGCTCGCAGGTCTTCGTGTCTTTGTTGAAGTAGTAGCGAGGGAAGTAGCCCATGCAAG |
| >AAFF15717  TCCGCATCAGCCACGATGACCGGTGATGAGCGAAGCCCTTCGTCTCGAAGCTCGCCCTTGATGACTGCTCCTGTGGCATCAGAAGCTTGCGATGCTAGAGGTGTAGTTAGCTTTGCATGTCGCCTCGCAGTCATCCTTGGTGTTGAACCTGTTCTTCTTGCTCGAGAACGGAGAGTATCGCGAAGTCTTGCAGCTGTCGCTCTCGACGTCGTAGTAGTATTTTTTCACAAGTCCAAACCTATTCTTGGGACCCGGTTCTACGCACTTGCTCCCAGAGTTCCGGCCACAAGTGTTCCAGCACTCCCTTGCGGAGTCAAACCTGTTGGGGTAGCCACCGCAGCCGTAGTAGACGAACCTTTCACACCTTTTGTTCTTGTTGTTGTATCCAAATGCTACCTTACGTGAACCATTTGAGCACTCCTTTCCGCGATGTATTGGGTAACTGCAATTGCTTTCGACGTATTTGCCGCATGTTTTCCAGCATTCGGATTCGTAGAGAAAATTGTTTCCATTTCCTCCGCAGCCTTCGTAGTCAAACTGTTCGCAGGCTTTTGTTTCACTGTTGAACCAGAAGTTCTGCATGCGGCTCGACCTGCTGCAGTTGCGACCCGCGTGCTTTGGAAGGAGGCATGAATCTTGGGCGAATTTTCCACATTGCGCCTGGCACTCCTCCTCAGTCGGGAAATGGTTCTCATTGTGCTCGCAGTAGATGTAACGCACGCATCTTTGCTTGAACGGATTGAACCGGTACCCCTTATATCCAGCTCGGCAAATTTGTTTGGGCTCTGGCAGGGCACACACTCCGAGAACAGGGTCTCGACATTTCTTACTGCATTCCGCAAATTTGTCGAACCTGTTGCCGTTTCCATTGCAGCCTCCGTAGTAAAATACCTCGCAACGGTACGACGTCGCGTTGTAAAACCATTTCTTGAACATTCCTCTGCATATTCCGGGATCCGGCTCTTGCATGCAGTCTTCACTGGGTGACACAGTTTTACTGGCAGCGCAGATTCCTGCTAGGATGCATATGAAGAGTGCGATGAAAAGACGTTGTACCATGATGAGCAGCTCGGGTCCTTGAGTCGAAAGCGGCACCTGACAGGCTCTGCGGTAGTTAACTCGGAGCTGAAGTTTAAATGAGCTTCACACTATCACCCTTAGGTTCAAAGCTGTTGCAC |
| >AAFF15887  TTTTGTCTAACACTCATGCTTTATTTGTTGTTCTTCTAGAGTGATGTCAAGTTGCTGTTTCCACCTTGAAACAGCCTCTAATGAATTATAGGCACGGGCCCCAGGGTGGCGCAGACGGCGAAGCACTCGCGCATCGTCTCGAAGCTGTTGCCGTTGCCGTGGCAGCCTCCATAGAGGAACGACTTGCATTGGTTTGTCTTAGCTTCGTAGTAGAAGCGTGGGATGAAAGCCACACACGGGCCGGGGTACGGTGGGAGAAAACACAGATCAGGCTTTTTCTTCGCAGGCTCACTGAGCAACACTGTGGCTTCTTGTTTTGGCTGGAAGATAGCAAATGCGGAGCTGACAACAGCGAGGAGAATGTAAGCCTTCATGTTGCCGTACTGGTGGCGTCTCCGCATTTGCTATCTT |
| >AAFF16817  GGCGAGAAATTTATCTCCAGCCCCAGGTGTGGTCAGCAGAAACGACACACTAGATTGGGCTGAAATATGCAGTGGGGATAAGAAGAACGAAACGTGCGAAGATTTATGCAGGGAGGACCCGGTCCCTGGACCTTGTAGAGCTCTTATCCCTCGTTGGTGGTACAACGGGCAATTTTGCGAAAAGTTTACGTACGGCGGCTGCAACGGTCGCCGGAACAACTTCGAATCCGAGGAGAAATGCATGAAGGCATGCGGCAAGTTCAAATTCCCGCAGCTGGTGTTATTAGACGATTGAGCGTAACTCAAGC |
| >AAFF17636  TTCAATACGGATTCTGAGTGTCGACTGTTCCTTCTCAGTATGAAGGCCTACTTGTTCCTTGCTTTCATCGGCGCCGCTTGTGCGGCTACAAATGTTGACAAACAGTGCACTGCAAAAGCGGAAAAGGGACTTTGCAAGGCTAAGCTTCCAAGGTGGTGGTTCAATACGGATTCTGGCAAGTGCGAGCTCTTCTACTACGGAGGCTGCGGTGGCAACCAGAACAGATACCTCTACAAGGAGGATTGCGAAAAGACATGCGCTCCGAAAACACTGAACGAAACGCCTCTCACCACATTCAGCAACAAGAAGGCCAACTTCGATGACAAGAAGGGGCGGCTTCCCGGATCCGGCGTAGGCGTATGCATGGAGCCGCCATACACTGGGCCTTGCAAGGCGAGCTTCCTCCGATTCTACTACGACGCCAGCAGCAATACTTGCCGCCAGTTCACCTACGGCGGCTGCCGCAGCAACGGCAACAACTTTAAAGCACAACGTGATTGCATGAGGGCTTGCGGAGGTCGACGGCGCGGAGGTCTTCGGCCGCGCTAAGAAGAGATCGAAGAATAATTCGTCGTGCCATTCTTTACACTATATGTATTTTTGCGAGTAGCGCTAAAGGGAACGGGGAAGAAAAAGAATCTAATGTAACGTAGTGATTCCGTACTTCTTAATTGTTGTTGTTAGCCCATCCTTTGATACGCTAACAACAACAACATCTTAATTATAAGATTGCGCTGCTGCTTTGCTGCTTTTGCTTCCGTTAATAAAGATAATCTCTCAAAAAA |
| >AAFF18831  CTCTCGTGGGCCTCCTTGCCCAGGCACTGCATGCGGCACTCGTCGCTGGATATGAAGTTGTTGCGGTTCCCTCCGAAGCCCAGGAAGACGAACGGGCGGCACGACTGCTTCTTCGAGTCGAAGTAGTAATGCCACACGCGCGAAGGGCCGTAGCCACGATTAGGGGGCAGCTTGCAGATTCTCGACGTAATAACGCTTGCGCATCGTCGTAGGCACTGCTTGCGGCTTTTGAAGTTGTTGTCGTTGCCTTCGCACCCGCCGTAGACGAAGAGGCGGCAAGTCCCTCGCATGAAGTTGAAGTAGTAGCGGTACACTGTGCCTTTGCAGGGTCCCCGATCGGGATATTCCGTGCACTGCATGTAGGCTTTGTCTTCCTCGAAACACACGCATCCTGTTAACGACACCAGAAATACGGTTGCTGCAAGCGCAATCCAAGTCATATTTTCCAGAGATGTCG |
| >AAFF18941  CTCAGCCTGGCTCTTTCACAGGCCCGTTTCAACAGAAAAAAAGTCTGCTCCCTTCCGAAAGACGTTGGGCCCTGCAAAGCATCGATGCCCAAGTGGTGGTATAACAAAAATAAAAATTCCTGTTTTTTGTTTATCTACGGCGGCTGCCAAGGAAATGCGAATAATTTTGAACACTGCGAGGATTGCATGAGGAAATGTGGAGGACTGAACCTGCGAAAAGCAA |
| >AAFF19194  TTCAAGTATTTTATTTAAGCGCTAAGAACATTTTGGTTTAATATGCTTTGTACACATACTTAGTTCTTTATGAACGGAATGCTTAATATGAAAGGCCACTTGGATGTTTTGCGCTTTGGCTGAAGCCCTAAGTTGTATCTCTTGCGAAATGCTTCGGTCCTCTTCTTGCAGATTTTCCAAGCGGGGTTCTTCCCGCTGCACCGCTTCATGCACTTCATGCAGCTGTCAAACCTGTTGGCGTTCCCGTAGCATCCCCCATAAATGAAACCACGACACAGATCCACATCGGCGTCGTAGTACCATGACGGAGAAAAGCCCGTGCAATTTCCAGTTCTCGGCTTCAGGCTGCAGACTAGTTTTCGCTTAGATTTACGTGTGCAGGCTTTCTGGCATTCTTGTTCAGATGTAAACCTGTTCGAGTTCTCACCACATCCGCTGTAGAGGAACCCTTTGCAACTCAAGGTCCACAAATCGTAGTACCAAGAAGGAATCTTAGCTGTACAATTTCCGACAGCCTTTCGTCGCCAACAACGGGTCCATTTAAAACTTTCAGCTTGCACGACTAATACGTACACAGCGAAAGCACTTAAGAAAGCTTTCGCTCTCATTTTACAAGCTCCTGCTTTGAGGTAACGAAAGCCAAGAACTCA |
| >AAFF21925  GCATGCCTGCTTTGTGAGATCCGCAGGGGCACTACCCTTGGCCTTTTTATTGCATGCAGCCAACAGAAGTCCTCAAAGAGCATTTTGTTCCCGCACAGTCCTTCTGCTAAGTATTAAGAAATTCGGAGATGAAACCCCAAATCATTGGCCTTCTATTCTCCATGCTTATTCTGGTATTTGCTGCACCAAAAAGAGGTGAAAAGAATCGAGGAGATTGTTTTAAAAATACACGCCGCCCCGGTCAATGTGTAAAGAAGTTGACTAGATGGAGATATGACGAAACTCGCAAACTGTGCCTCTCGTTCACCCATGAAGGTTGTGAACATCCAAGAAACATTTTCCTCACGTGCCAAAAATGCATGTTGACATGTACAGAAAACAGAAACCCAGTCGGAATCTGCGGGTATCTAAATAATACTTCTCCAGAGTAGCACCTTCTCAAGAAAAGTCAAATGCTGAAGCGCTGGAGATACGGAAAACTTCTGAGAGTGGTCCAAACAGAAAAGTAGTTCGAATAAATGT |
| >AAFF22171  GCTCCTCACGATTTTTGCTCTCTTTTGCCTGTTGGGAACCACGCTGTCAGCACGCGCCCAAGAAGACCGTTGCAAACGGCGTCCAAAAAGTGCTACGGGCATGTGCCATAATGGAAAAGTGCCTAAACTGCGCTTTACATATAACCCTAGCACTGGAAAGTGCGACCATTTCTATGATTTAAGATGCAATGGACAAATTCTCAACAGTTTTGAGAATTTTACGGAATGTATGACAGCGTGCAACCCCGATTCAAAATGCCTGGCGACTCCTGATAAGCCTTTCAAGTGGATTCGATTGACAACCTCTTTCGTGTTTGACATAAACTCAATGAAGTGTAAAAAGGAGAAATCGCTCCGACGACCGGGCATTGGCCCTAAAATTAACAGGTTCTTAGAAGAGGACGAGTGCAAAAAAACATGCGAGCCGCATCTTATTAAAATCATTAAGAGTACAGGTTAAGCAACCGACAATAAAGTCAGTTCCAA |
| >AAFF22633  TGGCGTATAAAAATCGAGGTGTGCTAGGAAAGCTTCAGCCATCTCGCGGCATCATCAGCCTCAACATGAAGCTCTGTGTCATATTCCCTCTCATCTGCACTACATTTGCGGCCACCTTCTACGAGGAGAAATGCACGAAGGACCCAGAAGTTGGTTTATGCAAAGCTTTACTGCCGAGGTGGTGGTTCAACGTGAGGACTCACCAGTGTGAGGAATTCTTCTACGGCGGCTGCGGTGGCAACGAGAACAGATACGAGACAAAGGAAGAATGCGAAAAGACTTGCGCTGATGAAAAGCCCTCCGTAGAAGACGTGTGCCGTAGGCCCCCATACACTGGTTACTGCTTGGACTTCTTACCGCGCTTCTACTACGACGCCACGACCAACTCCTGCAAGCCGTTCGTCTACGGTGGCTGCCACAGCAACGGAAACAACTTCGAGACTCGACGCGACTGCCTGGATTTCTGCGCCCCTCCACTCGCCACACAGCCCACGCCATCGACAGCGTAGGGTGCGGAGCACGCTGTGAAAGTGAAAGCAAAAGAAACGCAGAAATAAAATAACTAAAGTCGAACTTCA |
| >AAFF24869  AGCTGTCACTGTCCGTCTGATGTGCATGTTGCTGAGGACGGCGCAGCCGGAAAGGACAGGGTGCATGTTGCTGAGGACGGCGTAGCCGGAAAGGACAGGGAAATGTGTCACCAGCCGGTGGACGAAGGCCACTGCGACACCGACAGCAGTGGCCCAGGGGCCACTACACCAGAGATCCGCTTCTACTACGACGTGCAGAAAGAACTGTGCGAGCGGTTCAACTACCAGGGATGTGGTGGAAACGACAACAACTTCCGTACCGTAGACGGATGCAACATGACCTGCTTTGGTGTGCGCATCAACTTGGCCAGGACGGCTGCGAGCTGCCCGGAGTCCATGCAGTGCAGCTGCGGTGATGAGCAGGAGGCCAAGTGCGGGTCATGCGAGTGCGGGAACGCAGCCCCGCGTAGCAACGGGGCCGCCCTCTACGCTCTAGTGTTCCTCACTGGTGCATCGGTGACAGCGTCGCTCTGCACTCTCTGAAGAAGCGACGAGGAGGACGGAAATATTTCTTTTTTGTTCCCAGATTCTCGATTAGCGCATGCGTGATCACAGGCGCACGCGGTGTGTAGTGAAATCTGCGCGCACTGGCAGTAACTGCCAAGGACGTCCACGAATGCAATTTGTGTCCCTGGCGATATGAACTTTCTGTC |
| >AAFF25745  GCCCTTCGGACTGCCCCATATTACCGTCGGTAAAATGAGAGCTCAAGCTATATACTCAGCATGTTTGGTTTACTTCGCTTTTGTGCACGCCGCAAGTTTCCGCATATGGCCACGTTGTTGGCGAGGAAAGGCTGTTGGAAGCTGCGGGAAGAAGATTCCGTCGTGGTATTATGACTTTTGGTCCGGGAAATGTAAAGGCTTTCTCTACAGCGGTTGTGGCGGGAATCCCAACAGATTTTCGTCTGAAGTCGAATGCCAAAGGTGGTGCATA |
| >AAFF25967  CATCAGACGGACAGTGACAGCTCTGGGCGCCGCTGCTCGAAATTCGCTTCACGCAAGTCACTGGGCAGGGCACTGCGATTTGGGCATCCACGTACTCCAGTGCATATTCAACAGTGCCGTCTGCAGCAGCACATTCGCAGGCGTAGCACGACGCAGACACCTCCCTACTCTGACAACCAGCCGGGCACAGCTCGGGAGCCGCGAGGCAGTCAGCTTTCTTGAGAAGGGGCTCTGTTTCGGACGCGAGGCAATTGCACGCAATACAGCTGTCTTTCTCTCGCCGCTGGCAGCAGTGAGCGCAGGCTAGGCCGCATGCTGCACGCTGGGGACACTGGGCTGATTCGGATTCCACAGGTGGAGCTCGAGCTTGCTCCAGTGGCTTCTCGCATCTTTCAACACATTCTTGGCGAGTAAGGAAGTGGTTTTCGTTGGCTCCACAACCGGTGAAAGTGAAGTTCAGGCAGCTGCGTTGCTCTCGGTCGTAGTAGAAGACTGTCTGCTTATTCGCACAGAAGCCTTCCTTTCGTTCCAGGAAGCATATTCCCGTGCCTGGTGGTATTTCGGCCAAGGTGGGATCTTCATCGGACGGTAGAGTGCATGCTTCGTCAAACTCGTGCGGTTCCAGCTGTGCGACAGCAACGCAGACGTTCTCCAAGTGGCGCGTGATCTCCAGAGAGGTGTCTCGTTCGAGCACGTGCCTCGCAAGGACGCTGACGTGTGTCGCATCCGGGGCACACACCGACGCGTGGGGCTGCTCATGCGCGCACCGGTGCAGCACGGCGTAGCTCCCGTAGTCCGTGGCTACCACGGCCAGCGTCTCTTGTACAGGCTGCCCAAAAAAGTCGTACTCGAGCTGAAATTGTCCGACGGGCAGCACTTTGTGGACTTTGCCGACAATGGCCGGCAGGCATGTCTCATTGCCTGCAAGTGCTGCGCTAAAGGCGAACGTGTTGGTGTCGAAGTCAAAGCGCCAAGAGCCTTTCTGCAGGAGAACCGGAGCGTGCTTGCTCTTCAACACCTCTCGCCAGTCTCCCGAGAACTTCTTGTAATCGAGGTCCTCG |
| >AAFF27299  GCGCGTTTCTTATGATATATGCGGTCTCCAATTGGCCACAACAGCAGTGCGCTGACATGCCAGCGTAAACAACTAGTTGGCAAGGCGTCCCGTAGGTACACCCTGCGAGTTTTTTGCCCCGCGGACAGTGCAGCTTGAGCACCCCTCGAGACAGCATGAAGGTGCTCGTACTTCTGTCGCTTTTCGGCGTCGGGTTGGAGTGCACCGCCCAGGCTCCGAATACCATCACGCTCCTTGCCGAAGAAGAAACGGAGGCCGACTACGACTTCGAGACATACTGCACGCCGCGTGCTGAGCGTGGTCCTTGCAGAGGTCACTTTCCAAGCTGGTGGTTCAATGCCGTGGTGAGAACGTGCGAGCGATTCGTGTACACCGGTTGTGAGGGCAACTACAACAACTACGTCAGCAAGGAGGCATGTGAACTTACCTGTTTGAGGCGAGGAGGAGGTTCGCAAACGAGCAATCCGGACATAAAGGAAACGGACCCGCAAGCATTTCTGGACTGCTTTGTGGAGGCAGACAAGGGACCGTGCGATAAAACTATTTATCGTTATAACTTCGAACCGGAGTCGGCGACTTGCAATTTGTTCGCATACGGTGGCTGCGAAGGAAACCGCAACAACTACAACTCAGAAGAGGAATGCATGGCCAAGTGCAACCCTCTAAATGACTACTACAGAAAGTGCCTGATCAAGCCAATGGAAGGGCATTGCAGGATGTTGCAGAAATTGTGGACCTACAACGTGACCCTTGCCAAATGCCAGCAGTTTTGGTACGTTGGATGCATGGACAACGATAACAAATATAGGACCAAGGAGGAGTGCGAAATGACTTGCCTGCGTCAGCCAGGGAATGTTAACCCCCTCTGTTTCGAGCCAAAGTATCCTGGGCCATGCGGGGCGCACTACCCCCGCTACTACTACAACCGGTGGTCAAAAACCTGCGAGAAGTTCATCTACGGCGGCTGCCGGGGCAACGAGAACAATTTCGAGACTCTGGAAGAGTGCGAGAACACATGCTGGGTCTCAAGGAAACAGGATCCAGCAGATGTCAGCGAAGCATTTCAAGCTCCATTCAGGCCGTGGGCAACGCCTTTGGAGTGCACTTATCCGGCAGAGGCAGGACGTTGCTTGGCTTACATGCCACGTTTCTACTACAACGCGACGACCCAGTCCTGCGAGCAGTTCATCTACGGAGGATGTGGAGGGAATGCGAACAACTTCTACAGCTACGCCGACTGCGAAAGCAAATGCAAGACGTCCATGGGCATTCTCCCTCGCGCTTGAGCATGAGACGGCAGCAGAAAATAGAACACAAAAAGAGAACAGAGTTTATAGCAGGCTAATTGCCTCAACCTGTGAATTTTTAAGAGTTGTAACAAACAAATATTTCTATGGCTAGCTTACTTGCTCAGTGCGCTTGTGGCCGATTCTTTTCTTTCTTGAGATAATCTTTTTGATCATTTTATTTGATAATGCATTAAACAAAATTGGGAACACATGTG |
| >AAFF27783  TTCAGATGCAGCGAAACTGTAACCAGCATGCACGACCGACACCTCATGGCAGAAAAAATGCTTTGGGGCTAAATTACAATGATCCCGCAATGCCTGGTGCTGCGAGTACACGATACTGATGGGTTTGTGGTGCATTTCTACTCTTGATGTCTTTCTGGATCTCCGGAGCCTCGGCGGCACTGTCGTTCGCACATGGCGATGGTGTGGAACCTGTTGCCGTTTCCCTGGCACAGGCTGTAGAGGAAGGGCAGGCACTGGTCCTTCTCCTGGTCGTAGTAGAAACGCCGCC |
| >AAFF27784  TTCAGATGCAGCGAAACTGTAATCAGCATGCACGACCGACACCTCATGGCAGAAAAAATGATTTGAGGCTAAATTACAATGATCCCGCAATGCCTGGTGCTGCCAGGAGTACACGATACTGATGGGTTCGTGGTGCATTTCTACTCTTGATGTCTTTCTGGATCTCCGGAGCCTCGACGGCACTGTCGTTCGCACATCGCGATGGTGTGGAACCTGTTGCCGTTTCCCTGGCACAGGCTGTAGAGGAAGGGCAGGCACTGGTCCTTCTCTTGGTCGTAGTAGAAACGCCGCC |
| >AAFF27907  AATTTTATTACAAGCAATCAGATACTTCTAAAGCCTTAAGCACAAAGCTAGTGCATTGCACCGAACGCAAAAGAGAAACATTGTGTCAAGCTTGCAGTTAACTTGCTTGTGCATCTCGCCGTCCCATCCCCGGTTCTCATTCGGCTCTGCTTCCTCCTGCGGCAGTCACCGGCTGTCTGTTGTGGAGCAGCTGACAGTATGCCTTTCCGTTGAAATTTGTACATCTTCTCATGCAATCCTGGCAAGTTCTAAAACTGTTGCGGTTTTTTCCGCAGGCACCGTATGGGAACCTAACACAGACGCCCTCTTGTTGATTGTAGTACCACCGCAGTCTGTTCCCCGTGCACCGTCCTGGGTTGGGAGGCTGGCTACAGGTCAATAGAGGTGTGCGTCGCGGTAGGCACGTTTGCAGACATTTTCTCTCCGAAGGAAACTGGTTCCTGTTCCCTCCGCAGCCACCCCAGATGAAAATCTTGCAGACTCCAGCTTTCGATTCATAAAACCACTTCAGATATTTTGCTCTGCAGGAACCAGTTTCTGGCGCTTTCCTGCAATAGTTAGGAGCCGCGCACACATAGACAACGGAGACGAGTAGCACGCATACTGCACCGAAAAATTTCATATTTGATCTTTCTGTGGAGGGTAGATGCGTGCTACTC |
| >AAFF28621  GAAAGCCCAGTTCTTAGCAAGGGTTGTACAAAGTTAGCGCGATGATCAGAATGCAAGCATACGTCTTTCTGCTTATCACCTGCCTCGTCCTCTCACATGTGGCTGCTAATAGACCCCAACGCTGTATGCAAAGAGGAGGAGTAAAAGGAAACTGCAGAGCTTCTATTCTTAGCTGGTCATACGACTCGAAAAGGGACAAATGTTACCCATTTTTATACGGTGGATGTGAAGGAAATCAGAACAACTTCCCAAGCTGTAAAACGTGCATGGAAAAGTGCAATCGAGGCAACAGACGGGCGACGCAACGATTTTGTAAAAGAGCGAACAAAGAAGCCAAGAAGCAATTCAAGCCACAGGGTTCTCCAAATTGAAGACGAGGCCGTCAACCGGAACTACCAAGCTTTACCTCTTCAGGAAAACTGGTTCGGTC |
| >AAFF29345  TCCCGTCTTGCATGTAGCTTTGGGTGGATTAAGGCTGCACACTGGTTCAACGGGAACTCCAGGCAAGCAGGTTTGTTGGCATTTGACCTCGCTTGGAAACCGGTTCGAGTTACCTTGACATCCTCCGTAAATGAATGCTTTGCACTTCTTCGTTGCGGGATCATAATACCAACTCCGATGCAATGCTTTGCAGGGGCCAACTACTGGTGGCTTCAAGCAAATGTGCGGCGTTGCATAAGTAGCCGCAAGCGCGGACACCATAAACCCTGGAAGAATAAGAAAAATAGGTAACTTCATGGTCGACGCCGTTGTTTGGCTCTGA |
| >AAFF30538  TGTGAGAACCCATGCCGGACTCCTTTAGAAGGAGGTCGCTGCAGGGAGGGGGTCGAAGGTCGCTACTACTACAACATTGAAACAGGCCGCTGTGAGTCTACCCACTACGGATTCTGCGAAGGATCCTGGGACAGCTTCCTCCGTCTTGAAGAATGCAAGACTCGCTGTGAAGACGTGTGCTCGCGGCCTGTGGACCCTGGTCCCTGTGACTTGAGTTATCCACGGTTGTGGCCAAATAGGCGGCGTTTCTACTACGACCA |
| >AAFF31248  GTGTTATTCTGGTTGCGAATAGGGAATCCGCTGCAATGAAATATGCAAAGTGTTTTCGTGCTGAACGAGTTCTTTTCCATCTCCGCTCCTGCGTTCTCGCACATTGAATACTCCTGGCAAACTCTTGATGTTGAGTTGTAGAAGAATCTTTTCATTTCTCTGTTGTAGTTTTGCCCAACGCACAGACCCGGGTGAGGAAGCATTCCGCATATATCCAGGGTTTCACTTTGGTTGCACTTCATCGCGCAGTCAATTCGTTTCTCATAGACATCCTCCTTAAGGCACCCGTTCCATCGAAACTGCTCGCAACGGCGTGATGTCGTATTGTAAAAATATCTTGGGACTTTTGCTTCGCATCGAGGTTCATATTCCATTAGCGGTATCTTGCAGGCTCCATGATTGGCCACCTTTCGTTGCTCTGAACGTGATGCTGTCACAAGAGTCAACCCGAGGAGACAAATGAGCAGACAGTGCATCACAGTTTCCTTTCAGGCGTAGTGGCAGAAGTGAGA |
| >AAFF32203  ATAAAAATTTTCTTGGTGTAACAGAAAGCTATGGCGGTGATCTTACTTTACGCTGCGACTTTATTGCTGGTATCAGGAATTACAACCTTTGGAGAAAACATTCAACAACCTCAATGTGAAGACGATACTAAGAACACGACATCGCCGGGGTACTGGATGTGCGACAAAAAAAATGGAATCACAGTATGCCGCCGGCATGACTACTACTATGACCCAGCAACGAACAGATGCAACTTCCTTGGCTTTCTTGGATGTGGTCGCAACGGTAATAACTTTCCGTCACACCCAGATTGCATCGCTCACTGCAAACAGGGCGCTGATTCACGCTGGATTGAATTCTTCCGAAGACGTTTCCCAGGGTGCCACATGAAATCTAATCCACTCAATGACACGGGTGACATTCGGCGGTTCTATTACAATTCTACCTCGAGAGAGTGCGAAGCGGTGGATGTTAAAACAGGGGATCATTATTTTCCCAGCATGAATTTCTGCCTTGAAGTGTGCCCAACATCTAAGAAAGGACTTGAACGCTGCAACGAGGAAAGCGAGAC |
| >AAFF32388  TTGCACGAACAGTACAAAATCTGACAGATGCTCAAATCACGGAAGATGCACGGCATGTAGCTGGCCACTCAGTGCTTCTTAACGAAGACGCTATTTAGGCTGGCGTGGCTTGTTGAAGGCTCCACCCACGCTTGCGTAGTTCCTCTCGAATTCTTGTGTGAGCTTCTTACAGAGATGCATGTTATGACCTCTGCCACATTTACTCATGCACTCCCAGCAGGTACCAAATCTGTTGTCGTTTCCTTTGCAGCCTCCATACACGAATCCCATACAGTACCCTCTGCTGTAATCGTAGTACCAGCGCGGTTTGTAAGCCTTGCAGATTCCTGTAGCAGGCTTTAGACGACAAACCGAACCAGGCCGACGTTTCGGTCGGCAGACTTCCAGACACCTCTTCTTCGTTTTGAAGTTGTTGGCGTTTCCCTTACAGCCACCGTAGTAGAAAAATTTACATGTTTGGGTAAAGGGATCAAAGTACCACCTTTGAATGCGAGCTCTGCACAGACCAACCTGCGGCTTCTTGTTGCAGTAGTCTGGAGCTCGCTGGGTCACCACAGAAGCAACGAATGCGGTGGCCAAAAGTAGGCTGAAAGAGGAGCGATTATACATGGTGGAAAA |
| >AAFF32773  TACATTCGGCGCAGGAGTCAAATTTGTTTGCATTTCCCCCACCGCTTATTTATTTCCCTGGGGCGTCTTCTTGCGTTAATTCCGGATAGAAATACGTTGTTTGAATTTGCTCTGGTTTCCAAAGAATGCGCTTATTTAGGTAATCATGTACTACCTGCATTTAGCTTGAGTTTCTTCTCCTCTTTTAGCGTAAGCTCATGGCAAAGCTTCTTAGTCTTCTTACCACGTTTTCCCATGCACTTCAACATACATTCGGCGCAGGAGTCAAATTTGTTTGCATTTCCCCCACAGCCGCCATATATGAACATCCTGCACCCTCCAGATTCGGAATAATACCATTTTGGGATGGAGGCTCTGCATCTTCCTGGATCCTTTTTAAAGCCGCAATATTTCTTCAAAGATTGTGCATTAGACAGGGCCACTAAGAAGAGCAGCAGAAGAAGGCAGGATTGTGCTTTCATAATCGCGCGTATCTTGATGATTTGATGCTAACACGATATATGTCAGCTGCAAATGGCCAGAATATTTGAGTGCACGCTTTTATTGGCTAGCGTTTCTGCATGTATTCTGGCGGCGCGGCATTGAA |
| >AAFF33328  TCCACTCCAGGTCATTCGAGAACCACCGGCTTATCCTTGGCCTTTGCTGTTTCTTCGTTGAAACCTCCGCATTGCTTTTCGCAGTATCCTCTTGCGATGAAATAATTATTATCGAGCAAGTTCTGCCATCCTCCGCAGAAGTTGTACTCCTCGCAGGTTTTTGTCGTTACGTTGTAGTAATACCGTTTTCTCCCTTTTTTATCTTTCTCGTCATCGCAAGGACTTGGTGGAGGATTAGCGCAGAATGGAGCGCCTTGTTTTTCATTGCATGTGGAGACACATTCGTAGCGAGATCCAAACAAGCCTTTTGTGCCACAGCCATTCCACCGGAAGTGTTCGCACATTTCGGACGTTGTATTGTAAAAGTACTTCAAGGTGATCTGGCGACAGTTTGGCCCGCTTACAATATCTGGCATCACGCATTCCGTTTTTTTGGGCTCGGAGAGAACCGAAGCCACGAGAGCGCATACGGTGAGGATGGAA |
| >AAFF33329  TTCCATCCTCACCGTATGCGCTATCGTGGCTTCGGTTCTCTCCGAGCGCGACGAAACGCAGTGCGTGATGCCAGATATTGAAAGCGGGCCAAACTGTCGCCAGATTACCTTGAAGTACTTTTACAATACAACGTCCGGAATGTGCGAACACTTCCGGTGGAATGGCTGTGGCACGAAAGGCGTCTTTGAATCTCGCTACAAGTGTGTCTCCACATGCAATGAGATGCAAGGCGCTCCATTCTGCGCTAATTCTCCACCAAGTCCTTGCGAGGGCGAGAAAACTAGAAAATGGAGACCAAGGTATTATTACAACATAACGACACAAACCTGCGAGAAGTACAACTTCTGCGGAGGAAGTCAGAAATTGCTGGATAATAATTATTTCATCGCAAGAGGATATTGCGAAAAGCAATGCGGAGGTT |
| >AAFF34313  GCTCCTCACGATTTTTGCTCTCCTTTGCCTGTTGGGACCCACGCTGTCAGCACACGCCTTAAAAGAGCGTTGCCGAAAGCCTGTAGCAAGTGCTACAAGAACGTGTCCTAAGGGAGAAACGCATACGCTGCGCTTCACATATTACCCTGGAACTGGAAAGTGCGGCCAATACTGGCACTCAGGCTGCTACAGAGGAAGAAATAGGAACAGTTTTCACAATTTCACGGAATGCATGAAGGAGTGCAACCCAACTTCAATATGCCTGAAGACTCCTACCCAGCATAGAGGGTTGATTCCAAGGGTAACCTCTTTCGTGTTCGACATAAATACATTGA |
| >AAFF34314  TCACGATTTTTGCTCTCCTTTGTGTGTTGGGATCCACGCTGTCAGATGACACTCTACCAAAACGTTGCAAAAAGCCTGCAATACATGCCACAAGCACGTGCCCTAACGGAGAAGAACCTAAATTGCGCTTTACATATATCCCTGGCACTAAAAAGTGCGACCAATTCTGGGAGTCAGGCTGCGGGAAAAAAAATATTAACAGTTTTGCCAATTTCAAGGAATGCATGACAGTGTGCAACCCCTCTTCAAAATGCTTGAAGACTCCTACCCAGCATAG |
| >AAFF34725  GCAGCCGCACTTATAGGCAGGTGAAAGTCGGACGAAGTGGACTTGAGGAACGGACCGTACTCCTGATTTAGGGAAGTATATAATACAGGAGAGCGCCAACAAGAGTCCAGAAACAGGATCTAAAAGATGAAATTTGTAAACATTGCTTTTCTTGTGACCATATTTTTGGTGGTGGCAGGAATGAAACGTTCAACGAGAATCCCGAGGCGTGAAGATTGCTTCAGTTCTAGGAATGTGGCGGGAAAAAACGCATGCATGGGAAGTGTCCAAAGGTGGCATTACGACAACAACCATAGCACTTGTCTTCCGTTCATTCATTCCGGGTGTAGCGTTCCAAAAAACCTTTTTCGGACTTGCGAAAGTTGCATGAAGACATGTCTCCGGAGGAGTAATCCACAAGCAGAATGCAAAATTCTTTCCGAGATTTCAGACTCATAGGGGATGTGACACACCGGTTTTGCTTTATATCGGTAAAGCTGAGACTGCATGGCAAGCTAGATATGTCGTTTGGTGAATAAATAAAATACATACTGGCATTACGACAACAACCATAGCACTTGTCTTCC |
| >AAFF35151  AGCCCCACGCGGTGCACATCGCCTTGGCTCCCTGGCCCAAGCACAGCCCCCACCGCAAGCCGGACCTGTGCACGCTGAAGCCGAACCGCGGCAGTCGCTGGCCCTGTTCCCAGCGCTGGTACTACAACGTGGTCGAGGATGCCTGCAAGATGTTCACCTTCTGCGGACACAAGGGGAACAGGAACAACTTCCCCTCGCACGAGACCTGTCACAAGGAGTGCCACGGCGCAAGTCTCGAAAAACGAAGCTAAGCACCAACCAAGATGTCTTCCTCCGGAAGCGTAAGGCACGCAAGGAGTCATTCAACGCGTGCTCCCCGTGCGCTGGAACGTGTGGTGGAGATACGGCACCAGCCTGAAACAGACTTTTCTCGGCACTTCTTTCTCAAGA |
| >AAFF36833  GCGTACCCTTATTACGCGTACCCTCAGCTCTGGTGAGAGGCACAGACTCGGACCAAGACCATGTGCAAGCAGTCTTTAATGTGTCGAACCGGATCGCCGCTGCCGTCAGTGAGGCTGGCTCTGCTGCTGCCGCCCCTATCCGCTGGTCGTCTTGCGGGGATGCACGCATTCCGCCTTGCACTTGGCCTCTGTGTCGAAGCGGTTGTCGTTGCCGTCGCATCCGCCGTACACGAACAGCTTGCACGCGTTGGTCTTGCTGTCGTAGTACCATTTGGTGGAGGCGTCCTCGCAGTTGCCGGGGTCCTTCTTCAGGTTGCAAGCTTTCTTGTGCGCCGCATGGCACACGAGTGAAAGGGAGATGAGGAGCAGGCAGACCAGGGACTTCATGCTGGCGG |
| >AAFF37485  CGCCTCCTGTGGCAGCACGCAGATGTTCCGCCCTCCGGGGGAGAGGCAGGCTCGCTCACAGTCACGCCTTGACTCGAACCTGTTGGCGTTGCCCTCACAGCCTCCATAGTAGAACTGCTCGCAACGACCAGCGGCCATGTTGTAGTACCAGTGCACAATGGCTGCCCGACACGGACCCTCCTGCTTGGGCAAGGCGCAAACGTCTCGGGGCACGGTCACCCTGGTGGAGTTGCACATGCGCTCGCATTGCTCACGGGTGTTGAAGCGGTTTCGGTTGCCCTGGCAACCTGTGTAGACAAATTCCTTGCAGAGCCCCTCCACATAGTCGTAGTACCACTTGCGGTCTTCCGACAGGCACTGGCCTACCTCCTTGGACAGGATGCAGATATCCTTTGCACCGAGAGAGACACATCGTTCGGAGCATTCTTCTTCAGTGGCAAAGTTGTTGGCATTACCCTGACAGCCACCATAAGTGAACTGCTTGCACCGATTTTCCTGCCGGTCATAGTAAAACCGCGTATATGAGCCCCTACACGATCCAGGTGCAACCGACTGTTCACATGGGTCCAGTGTCTCCTGATGGAGGCA |
| >AAFF37541  AATTTGTTTCGGTGCTACAGCAGGCCAGGATGGGGATGAAGTTTTTCGTCGCGGCAGTATTGTTGGCATCAGCGGTCACGATTTTGGGAGGAAAACAAGAAAAAATAGAAGAACCGAAATGTGTCGACAGAAAAGTGAGCAGGTATTGGATGTGCAACCATGAAAACGGAAACGAAACCTGCCGACGGCGCGACTACTACTACCAGGAGTCTTCCAAGAAGTGTCTATTCCTTGGCTTTCTGGGATGTGAAGGCAACGAAAATAATTTTCCCTCGCGAGAAGACTGCATCGATCATTGCGGGAAGGCTCCAAAGCCAAAGAATGCCGCTTACTTGAAGTTCCTGAACATACTGCCAAACTGTAACGTGACCTTTAACCCGAAAATTGACAATGGTACTGTACAGCGGTTCTATTATAATGAAACCGCGCAAAAGTGTGTGCCGGTGAGTGTTCGCTACGGCGATCGCTATTTCCCAGGAATGCATTTCTGTGAAGACAAGTGCAGCAAGGAGAAACATGACCTTCCACGCT |
| >AAFF37779  AGGTCTTTGCTGACAAAGCGGTTGTTGTTTCCGAGGCACCCTCCATACATGAACGACTCGCAGGTGCGAGTCGAAGCATCGAAGTACCAGTGCTCGTACTGGCCATCACAAGGGCCAACCACCTTGGGGAGCTGGCAGGCATCAGGTCCTTCTGGTTTGACACAAGTGTTCTCACACTCCTCTTCAGAGTTGAACCGGTTTTCGTTGCCCTCACAGCCGCCATACCAGAACCGGTTGCAGCGGCCGTCAGCAACGCTGAAGAACCAGTTGACAGTGTAGTTTCGGCACGGGCCAAGGTCCTCGGGCAGGCCACATACAC |
| >AAFF37951  AGCGACACTGGACACACCCACGGTGGTCCACAGTCTTATTCTTTCCCAAGGGGCACTGCAGTTCCTCACAGTTTGACGGCAGGCACTCTGGCGCTGGCGTGCGGGGCACAGTTGCCGGTGTCAACGGACTAGGCTCTGGAAGTGGTGTGACTGCCATTGGTGGCTTCACACGTGGGTCCAACTCTTCGCTTGTGTCACTAGGCTCTGGAGGGGAAGGCGGCATCCATTTCCTGTCCGCATCAGGATCGATGATTTCGTCATCAATGCTGTCCATTGTGACCCCAGAGCAGATCTTCCGGCAGATCTCGGCCGACTTGAAGCGGTTCTTATTGCCTCCACAGCCGGTGTACACAAATGGCAGGCAGAGCCCGCTCTTTACGTCGTAGAACCATTTAGCATGCATTTCACTGCAGGTTCCCGCATCAGCCATTTTCTTGCAAATGCCTTTGCTAATGCGCCTAGGCTTAGTGGGACGCCGAGGAGGTGGCACTACGACTGGTGGTACAGCACGGCCGCAGCGCTGTTCGCAGAGCTCCCGAGACTCGAAGCGATTGTCGTTGCCCTCACAGCCACCGTAGGTGAAGTTGCGGCACTCGCGGGTCGTGGTGTCGTAGTACCACATCATCAGCACACCGTAGCAAGGTCCCGGCTGCTTAGGCTGTGCACAGACATCAAGTGGCGTGGGATGGTCGACAGGGTGCTCAGTGGGCAGTGGTGCGCCCTTGCGGCACCGAGCCTCGCAGGCCTCACGATCATTGAAGCGGTTGGCATTGCCCTGGCAACCACCGTATGTGAACTCATGGCACCTGTCTGTCTCGGGATCGTAGAACCACTGTATGAAGTTGCCACTGCAAGGGCCCTGCACGTTCGGCAGCATGCACAGATCTTGTGCACTGAAGCACCTCTCTTCGCAGTCCCGGCGAGACCTGAAGCGGTTGCCATTGCCAAGGCAGCCACCG |
| >AAFF37964  TCTGCTCCCTTCCAAAAGTCGTTGGGCCCTGCAAAGCATCCATGCCCAAGTGGTGGTATAACATCAAAACAGGTTCCTGTGTTTTCTTTATCTACGGCGGCTGCCAGGGAAATGATAACAGTTTTGATCACTGCGAGGACTGCATGACGAAATGTGGCGGACTGAGCAGGCGAAAAGCAAAGTTCGTTTGCAAGAAGCTGGAGAAGCAGAATGGTGGAAATTGGAATCCTAACAAGCCAAGCAATGCAAGAAGAGCCAAGTAACCAGCGTCATCTAGTTTTTACTCCCTGCTGGAAACATATTCTCTCTGCACTTGTGAAAAAATTCCGCACAGATCGAATAAACTATCTAAAAAAAATAACCACCCATTATCA |
| >AAFF38822  TAGCGAATACGCGACACAGGAACATGCAGTCTGTGAAAGGGAGGACAAGCGGAGTCGGAGGCTAAACGGAACAGATAAAGCCATTGCGCTCGGCTTCAGCGGGACTCTTAGCGTCGCTCTGTAACACAGTCTTCTTGAGAGGTCGTTGCCGTTGCCTGTGGACGTCGCCTTGGAGTGAAGCAAAAAGACTAACGGCGATGCAGGTCCTGAAGACATGCATCTTTCTTCTAGCCAACGTGGCATGGTGTTCTGCTCAAGGAGCCGTAGTGTGCAAGCTTCCACTGCACGAAGGCACGTGCAACGGAACCACTTCGCGGTACTTTTTTAATGTAACGTCTGGCTTTTGTCAGTCGTTCAGCGGCTGCGAGAGCAATGGGAACAATTTCGCCCACCACAAGGACTGCTATGACGAATGTGAGAACCCATGCCGGACTC |
| >AAFF40907  CTCTACAGTGGATGTGGTGAGAACTCAAACAGGTTTACGTCCGAACAAGAATGCCAGAAAGCCTGCACACGTAAAGCTAAGCGAAAACCAGTTTGCAGCCTGAAGCCGAAAACTGGAAATTGCACGGGCTTTTCTCCGTCATGGTACTACGACCCCGATGTAGACCTGTGTCGTGGTTTCATTTACGGGGGCTGCTACGGAAACGCCAACAGGTTTGACAGCTGCACGAAGTGCATGAAGCGGTGCAGTGGGAAGAAAAACGCTTGGAAAATCTGCAAGAAGAGGACCGAAGCATTTCGCAAAAGATACAACTTCGGGCTTCAACCAAAGCG |
| >AAFF41614  CTGGGGCCTCCCCGGGTCTTGGGAACCTTTTGAGTGGAGGCAGATCAGCTCGCAGGCCGTCTTGGTGTGGTATTTGTTCTGGTTTCCGTCGCAGCCGCCGTAGGTGAACCGCTGGCAGCGTCCCTTTGTGACGTTGTAGGCCCACATGGGGATGCGGGCGCGGCATGGTCCTCGATCAGCCGCTGGTGTGCATTTGCTGTGGAAGTCTGCGAGCACAACCACAGCGAAGAGAAGCGTCAACGAGACAGTGACCCCCATAGCGTCAGCAGGTGTGATGCGAAGAACTGGCAGAGAGCCCTGACCCA |
| >AAFF41927  TCGTGCCATCTCCTGCCGGGTACACCGCTACTATGCGTATCCTTGCTTTCTTACTGCTTTTGCTTGGCCTCTTCTGCGCTATGCTTCGCGTAGAAGGCCAGAGGAAGAAAGGGAATCCAGCATGCAGAAAGCCACCCACCCATCGAAAATGTAAACTCTTCAGAAAACTAGTGTGTGACTGCCCTTTGGATTTGGGTACTCCAACCTACTTCCGGGAGCTGCGGTGGTTTTACGACAATGAAACCAAACTGTGCGAACCATTTGCATGGTCGCGCATGGGGGGAAATTGCAACAACTTTCCCTCAGATGACGAATGCTATAATGTGTGTGGA |
| >AAFF45697  TCTGGTCCTCCAACGATGGGGCGCAGACGATTGCCATGTTTTTCAATAACTTCAGCAGTTAGTTTCAAGCAGAGTTTCTGTGCCTTTCCGGGTTGCATTCCGCTGCATCTGCCCATGCACTCGAGACAGCTTCGAAAGCTGTTGGCGTTTCGTTTGCATTCACCGTGCAAAAAAAAGCCACAGCGACCAGCATTCGAGTCAAACGCCCATGAATAAACTCGCCTCCCGCATTTTCCTTTTGGTGGCTTAAGGCTACAAACTGGTCTCACAGGAGCTCCAGGCAAGCAGGCTTCTTGGCATTTAACCTCGGTGTGAAACCTGTTGGAATTACCTCCACATCCACCGTATATAAATGCTTTGCACTTCTTGTTTGAGGGATCATAATACCATCTCGGGAACTTTGCTTTGCAGGGGCCAACTGCAGGTAACTTCAAACAAC |
| >AAFF47234  CTTCGCAAAGCGTCTTGCACGCGTCGAGGTCTGCAAAGTTGTTCTTGTTCCCTTCGCAGCCGCCGTACTCGAAGGTCTCACACAATCCCGTCTGTGAGTTGAAGTAGTAGCGCTCCTGCGTGTCGTTGCAGCTGCCAGGATCCATTGGCAGGCGGCAGGGATCTTCACACTGGGCAATGCACTCTTCATGGGAGGCGAAGCTGTTCTCGTTCCCACCACAGCCGGTGTAGTAGAACACGTCGCAAACGCCGGAAGTGACGTTGAAGAAGTAGCGGGGAATCCTTTCATTGCAGTTGCCTTCTTTCACCGGCAACTTGCACGCACCATCAGACGGACAGTGACAGCT |
| >AAFF48589  GAAGTTTCCTCATATCGCTGAAAGAGCTCCATCGACGCCCAACGAGGTCAAAAGCACACCACAAACCGGAATTCATTTTCAATAAAAGATGGCGTACAAGGGCTCAGCTATCGTCATCCCTGTGTGTGCAGTCGACATCGGCGAACTACTTCCACAAGCACTCATGCACTCTTGAAGCGTGCGAAAGTTGTTGCCTTCGAGAGTACAGCTCACATCGCTGTGGCACATTCCGGTGTCTGGTGAATAAAAGTAAACTTCAAATGCGGTGTCCAGACAGGTGGGCACTGCGCGTGGACGATTGCAGATATCGTCGCTGAGAAGTGCGTGGCCGGGAATAATGGCTGCCCAGAAGCCAAACGCAACAA |
| >AAFF49119  TTTAGGCTCATACAGGGATGAGCTACTTCACGCACACAAATAAACAACATTGAATGGTTGCACCATGTTTGTTTCAATCGCATTTGGATGGTGCTCTGATTTTTGCTTAAACGCTTGCCTTGGGTTTTTGTTCCATAGGTTCGAAGTTGCTTCCGAAGTACATCAGCCAGATCCGCGCATGCAGGCCTTCTGGCATTCCCACTCGGTGTTGTACCGGTTGGCATTGCCACCGCATCCACCGTAGACGAAGGGCTTGCAGACACCGTCGGCCACGTCGAAGTACCAGCGCTCAAAGTCTGCCCTGCACATTCCGCTCTCAGGGGGAGGCCTGCAGCCCAAGTCAAAGTTCACGCCGCGGTGCTCATTTCGGACTTGCACCTCCGCCGTTGCTGCTGCGAAAACAGCGCCAGCCAGGAAGCAAAGAAGCACAGCCTTTTTCATGTTATTACAGTTGACAGGAAACCAGTGAAAGTGCGAACACGC |

| >AAUM1908  CCCGATTTCTACGAAAAAAGGAGCGAAATCGACTATGAAGCTCATAGCCATACTCAGCGTGCTGCTGCTCGCTGCGACCTTCGTAAGCGGGTATCGTCCACCAAACCCAATATGCAGAGAACCTGGGCGGAAAACTGGACGCTGCAAAGCTTTCTTCCTCAAGTGGTCATATAACACAAAAAGCGGCTTGTGCGAAGCATTTATCTACGGCGGATGTCGAGGAACTCGGAACAGATTTGACAGCTGCTATGAATGCATGAACATTTGTGCTAAAAAATTTACTAAGAAAGACAGAGAGTATTGCCAACAGTTGACTGAAAAAGCCAACAAGAAATACTTCCCGACGGCCATGCCGAAATAACCGGAACCGCCAAGCTTAATTAAGGCTGTCAAAATACGCGACAATCTCCTTTCTCGAAAAGTACTTCAGCGAGTTCATTAAACTGTCTAATGCTTGCGAAAGCCAGAAAGTGCTGCACGCATATTTTGGATTCTAGTTCTGCCCCTGGCTCCGGAGAATAAAG |
| --- |
| >AAUM3234  GATTGTGGCGTACCACCGCCACCGCCATGCGTCTATCCAGATGGTTGCAAGGAGCCGCAAAAAGTTGGACCATGCAATACAATCGTGCTGAGGTACTATTTCAACCCTGTGACCAAGAAGTGCGAGCAGTTCAAATGGGGTGGATGCTGCCCAAACTGCAACAATTTCGCAACGCTTCAGGAATGCCAAAGAACATGCGTTCGTTAATCTTCAAAAGCTGTTCGAGTTCCCTTTGCGTGTTTCGCATTGACCAGCGGCAAAGAGCGTTGTGAAATA |
| >AAUM5095  TTTTTTTTAATAGGTCAGAAAAAATTTTATTTTTTGTGTCCACGTTTCCATCATACAAAATATTGACGTCAGCAGCCTCGGGGAATGCGTTTGTGGGGAAAGCAGACTTCCAGGCATTCTTCCTTCGAGTCAAACAAGTTTGGTCCGCCGTTGCAGCCGCCGTATAAGAACTGAAAGCAATTCTTGTGGTAGTGGTCGTAGTACCACTTGAAAGCAAATGCGTAACATGGTCCACGGTTTGGCCAGTGACGACAGTCCTCCGGGGATCTTCGTCGTTCGCAGACCTTGCGGCACTCCGGCATGCTGGGGAAGTTGTTACCGCGATTGTGACAGCCCGATCTTTTCTCGCACAATTTCGTGTCCTGGTTGTAGGAGTACACGCTGCGCTCCTTCTCGGAGCACGAATGGGGTAGGGGGGGTTGGCTGCACACATCTCGGCTCCTGCACACTTCACTGCACAGCTGCCTTGAGGAAAAGGAGTTTGCCCGCGCAAGGTGACAGCCAACACCCAGGCGGCATTCCCTGGTGTGTCTGTCGTAGTACCACGTGTGCCTTGACGGCGCGTAGTTGGCACAAGCAGGGTCCTCAGTTGGTCTAGGAAAGTGGCAGAAGGACTCTGACGGGCATGTTTTTTCGCAGGTGCTCCTCTTCACGAAGCTGTTGCCAGCCTTAGGGCAGACGAACCGGCGTTCGCACCTCTTGGCGTCCAAATTGTAGACCCACCTCTCCATAAGGCGTCCACAGCCGTGGGTTGGCGGGAAGGAGTAGCACACGGGACTCGGCATTGGTACCGGCGAGACTCGTCTTACGCAGACCCTGCGGCATTCTTGTAGGCTTGGGAAGTTATTCCCCCTGTTATGGCAGTTAACGTCTTTCAGGCACTGGCCGGAGGTTGGGTCGTAGTACCA |
| >AAUM6652  CTTTGAGCGTACGAGCCCTAATATCGGCCGCACACTCTGCGGCATTGTCCAACAGAGTTGAATCTGTTCGCGTTGCCACCGCATCCGCCGTAGGTGAACGAGAGGCATCTTCCCGATCTACTATCGAAGTAGAAGGCTGGGAAGGCGCCGCGGCAGGGTCCCTGGCTAGGAGGAAGGAAGCACTGGCCTTGAGCATTGGTGCCTGCTACGACAATAGCCAGCAGCAAGGCGACGACCACAAGCGAACGAGGCATCTTGCGAACGGGCGGTCC |
| >AAUM6930  CCTTTTTCTTTTTATTTTGCTTGATATGGCACAAACAGTGCCGCAATGATGAAGAGCAGGACAGGGAAATTTCTTTTTTAGGTGTTCTTGCTTGATCACGGGGAGTTGATAATATAGCGACAGGCGCCTTCAGGATCTGGGTGAGTCGTACAAGTAGTCATGCAGGCTGTACAGTTGGGAAAGTTGTTTTTATTTCCGCCGCATCCGCCCCACAAGAAGGGACCACAGTATCCGTATCTCCCATCAAAGTACCATCTTTCAATTGAAGGCCGCACATGACCGCATTGTCCGTCATTTGGTTTTGCCTGGCAATATCTTGGGGGTTTGTACCCTTGCACTAATA |
| >AAUM6931  GATCATGGGGAGTTGATAATATCGCGACAGGCGCCTTCGGGATCTGGGTGAGTCGTACAAGTAGTCATGCAGGCTGTGCAGTTGGGAAAGTTGTTTTTATTTCCGCCGCATCCGCCCCACAAGAAGGGACCGCAGTACCCGTATCTCACATCAAAGTACCATCTTTCAATTGAAGGTCGCACAGCCCCGCATTGTCCGTCATTTGGTT |
| >AAUM7465  ATGTCTTTCTGGATCTCCGGAGCCTCGACGGCACTGTCGTTCGCACATTGCGATGGTGTGGAACCTGTTGCCGTTTCCCTGGCACAGGCTGTAGAGGAAGGGCAGGCACTGGTCCTTCTCTTGGTCGTAGTAGAAACGCCGCCTATATGGCCACAACCGTGGATAACTCAAATCACAGGGACCCGGGTCCACAGGCCGCGAGCACACGTCTTCACAGCGAGTCTTGCATTCTTCAAGACGGAGGAAGCTGTCCCAGGCTCCTTCGCAGAATCCGTAGTGGGTAGACTCACAGCGGCCTGTTTCAATGTTGTAGTAGTAGCGACCTTCGACCCCCTCCCTGCAGCGACCTCCTTCTAAAGGAGTCCGGCATGGGTTCTCACACTCGTCATAGCAGTCCTTGTAGTGGGCGAAATTGTTACCATTGCTGTCGCAGCCGTTGAACGACTGACAAACACCGGACGTTACATTAAAAAAGTACCGCAACGTGGTTCCGTTGCACGTGCCTTCGTGCAGTGGAAGCTTGCACACTTCGGCTCCTTGAGCAGAACACCATGCCAGGTTGGCTTGAAGAAAGATGCATGCCTTCAGGACCTGCATCACCGTTAGTCTTTTGGCTTCACTCCAAGGCGACGTCCACAGGCAACGGCAACGACCTCTCAAGAAGACTGTTGCAGAGTGACGCTAGGAGTCCCGATGAAG |
| >AAUM7916  CTGCTTGAGGCCTGACGATCCGAGACAGCTGGACGGTTGGACCGATTACCCTGCCCAATGTGAGGGTCGACACTGCAGCGCTCCCTGAAACTACGGCTTAAAGACTTGGGACGACATCTCTGTTTTTCTTATTTTTTTCAGGAAAATATGACTTGGATTGCGCTTGCAGCAACCGTATTTCTGGTGTCGTTAACAGGATGCGTGTGTTTCGAGGAAGACAAAGCCTACATGCAGTGCACGGAATATCCCGATCGGGGACCCTGCAAAGGCACAGTGTACCGCTACTACTTCAACTTCATGCGAGGGACTTGCCGCCTCTTCGTCTACGGCGGATGCGAAGGCAACGACAACAACTTCAAAAGCCGCAAGCAGTGCCTACGACGATGCGGAAGCGTTATTACGTCGAGAATCTGCAAGCTGCCCCCTAATCGTGGCTACGGCCCTTCGCGCGTGTGGCATTACTACTTCGACTCGAAGAAGCAGTCGTGCCGCCCATTCGTCTTCCTGGGCTTCGGAGGGAACCGCAACAACTTCATATCCAGCGACGAGTGCCGCATGCAGTGCCTGGGCAAGGAGGCCCACGAGAGGGGGGAAGACTGATGCGAGCCGGAACACGCACGCGAAGCCATCAACGCTACACTACGCTTTGGAAGTCGACTCTGGACGCTTGGACTGTGAATGCCTGGGGAAAAACGCGATGGCATTGCTATCAAAAATAAAAA |
| >AAUM9313  TTCCTTTTTTGCAATCTTTTTATGCTCCCTCTGTTTGTCATGAATGAGAATCATACAGAGACATCTGTGCAAAGAATGTCATCATTATATGCAGGACTTTTCGCACTCTGCCTTTGTATAGAACGTGTTGCAGTTTATGCAGCAGCCGAATCCAATCTTGAACTCCTCACACTTCCTGGTCGTTGGATTGTAACGGTACCTGACCAGGACTGGGAAAATCAGGCATGTTCCTCCTGGTTTTGGTAGACTGCAATCTTTTTTATATGTGCATGGTGGCGGGCAAAAAATGGAGTCTCCATATGAGACGGTAGCGAGAAGAACCGCCACCGTAAATAGAGCTACTGCTGCATTCATGGTGCCTAT |
| >AAUM9975  TTCGAAGTTGTTCCTGTTGCCCTGGCAACCACCGTAGATGAACTGCTCGCAGGTCTTCGTGTCTTTGTTGAAGTAGTAGCGAGGGAAGTAGCCCATGCAAGAACCAGGATCCTTTGGCTCCTGGCACCTAGTATCAGAGCTTGCCAGTGCAGCACCAAGAATCGCAAACAGTACGAGGAACTTCATGGTACGTCCGTCCATAGTGGTAGACGCTAGCC |
| >AAUM9976  TTCGAAGTTGTTCCTGTTGCCCCGGCAACCACCGTAGATGAACTGCTCGCAGGTCTTCGTGTCTTTGTTGAAGTAGTAGCGAGGTAAGTAGTCCATGCAAGGACCAGGATCCTTTGGCTCCTGGCACCTAGTATAAGAGCTTGCCAGTGCAGCACCAAGAATCGCAAACAGTACGAGGAACTTCATGGTACGTCCGCCCATAGTGGTAGACGCTAGCC |
| >AAUM15770  CTGAAACTGAAAACATCATGCATTTGTAAATGTGAATTTTGTGTATAGACCCAAACCTGCCAACAACATGGAGCTGAATGTACTGTGTTAATACGGATTTTCTATTGTAATGTCCACGACATGATCGAGTATTGAGTCAGGTGACTAGGAATTCAGCGAGAAAATTCGCAAATAAAAGGCTGGAAGGAGTGAATTGGACCATTCGCATATCAACAAAGGCGCCTTCTGAGCAAAGCTTCAAAAAATGACCGCAATGACCAGATTACCAGCGTGGCTGCTTCTGCTTCTCGTCGGCGTAGCTTTTTCACATGCCTGGTTTAACAGAAAAAAAGTCTGCACCCAGCGAAAAGAAGTTGGACCCTGCAGAGCATCGATACCTATGTGGTGGTATAACCCAAAAAGACAATACTGCAATTTGTTTATCTACGGTGGCTGTCAAGGAAATCAGAATAAATTTCAACACTGCGACGAGTGTATGAAGAAATGCGGTGGAATGGGCTGGCAGAAAGCGAAGAAGTTTTGCAGTAAGCTGGAGAAGCCAATTGGTACAAACACCGGCCCTTACAAGCCAAACTATGCAAGAAGACCGAAGTAATCAGCGTAATCTAAAGTGTGCTGAGTACTGGAAACACATTTCTCTCTCGGCACTTCAGGAAAAGTTCCCGCACTTGAAATAAACTCAGCTTTCGGTGGCTAGATGGACATTAAAAAAA |
| >AAUM15921  TCGAGGAATGCGAAAAAACATGCGGCGAGCCAGAGAGGTCAAGTGACTTCGAGGGAGCTGACTTCGAGACTGGTTGCGCGCCAAAACCGCAACGCGGCTATTGCAAGGGCTTCTTAGACCACTGGTTCTTCAACGTGACCTCGGGTCAGTGCGAGACGTTCCTCTACAGTGGCTGCGGTGGAAACGACAACAACTATGAGTCGAAGGAAGAGTGCGAGATTGCCTGCCAGCTTACGTAGCCCGCTGGACAAATGAAATGGGCACTTCGCTCTCTTTTTTGCCGTGTTCATTCAGCACATGCATTGGGCGAACACA |
| >AAUM15922  GGTTCTTCAACGTGACCTCGGGTCAGTGCGAGACGTTCCTTTATAGTGGCTGCGGTGGAAACGACAACAACTACGAGTCGAAGGAAGAGTGCGAGATTGCCTGCAAGCTTACATAGCCCCCTGGACAAATGAAATGAGCACTTCGCTCTCACTTTTGCCCTGCTCATTCCGCACATGCATTGGGCGACCACAAAGCCTGCAAGACATGAG |
| >AAUM16123  AGAAATGCGTAGCAGACAAATATTTTCACATTTCCTCACTACTCTGTAGCACTGTTTGTCTTCCTTTCAATTGAGTTCCCTGGACAAAAACGTCTGTTTCTTATTATCCTGCTTTGCAAGTGCGCCGACATTCGCCTAAGGTTGGAAAGTTGTTTCCTGCGTAGCTGCAGCCCATATCCGCGTGGCACATGCCGGTCCAAGGATTAAAATAGTAAACTGCAAATGTCCTCCCGCTGCAAAGTGCATACGGTCTCGGAGCAGTGCAGACGGCAACACTTCGACAGGTAACATCGCATTCTAATTGGGTTTCAAAAGGAGCTCGGCCGCATGTCCATGTGCACTGCTTGGTATTACGGTTGAATGCGTAGCTGCGGTGA |
| >AAUM17933  GTTCCATATTCGTACACGAATCATTCATGCGCACCTGTCATTCCTCTTTATGCTTGTCATTCCATTTCCTGAGGATTTCGCTTGCCTACATCTGGCTGCCATTTCTATTAAGTCACGGTATAGAGTCTCGTTAAGCGAACCGCGTTCCGTTAAGGCAGCGGTTAGTTTTTTTTTTTCCCCTTGGGTGATTAGTTTGGTCGTTTGTTGCAACTTTAGCTAACAAAAAAAAAATATATCGGTTTAGTACGCATCCGAGCAAGTGGAATGCAGGAAGTGTTCTTGAGAAAGAAGTGCCGAGAAAAGTCTGGTTCAGGCTGTTGCCGTATCTCCACCACACGTTCCAGCGCACGGGGAGCACGCGTTGAATGACTCCTTGCGTGCCTTACGCTTCCGGAGGAAGACATCTTGGTTGGTGCTTAGCTTCGTTTTTCGAGACTTGCGCCGTGGCACTCCTTGTGACAGGTCTCGTGCGAGGGGAAGTTGTTCCTGTTCCCCTTGTGTCCGCAGAAGGTGAACGTCTTGCAGGCATCCTCGACCACGTTGTAGTACCAGCGCTGGGAACAGGGCCAGCGACTGCCGCGGTTCGGCTTCAGCGTGCACAGGTCCGGCTTGCGGTGGGGGCTGTGCTTGGGCCAGGGAGCCAAGGCGATGTGCACCGCGTGGGGCTGCGTGACGCCAAACTCGAACGTCGCCTCGTTGGCGCCCAGTGGCTCGACCGGCGATGCAGCCCGGGCCGTTGCCGGACGCTGGGACGTAG |
| >AAUM18469  CTAACATGAATATCTACCAAGTTTTAGGCTTCTACTTTCTTCTGCCAGTGTGCATGGCCGTGCATCACAGCAGCAGCTTGAAAGACCCAGCCGTGTGCACTGCTCCACGCCCTGCCAGCTATTGCTACAATGGAGTCTTTTACGTCTTTTATTTTGACCCCTGGAGTAACTCATGTTTAAGGGAAGTAGGTTGTACTCTGAACGGCAATAACTTTGGAAGCAGAGAAGAATGCGAAAGACTGTGCTTAAGAGGAACCGCGCGACCACAGCCACGCCCGAGCGCCTTCTTTTATCCTTGGGTTCAGTTCTAAGGTTTGCCATCAGCCACTGCAGCGACAAAGGGAGCACTCTGGATTTGAAGAATTTCACCTTGAAGAATAAAACCCACCGGGCAATGGGCATTGATGAATCAATGGTTGTAATTTTGGTGCACGTGTTCTGTGGAAATCCTCAAAAATAAAAAAGGCTAACATTCTAAAAATTCCACTGACGCAGATATCTTTTAAATAAAGTCAAAATGAGA |
| >AAUM18663  AACCTGTTGGGGTAGCCACCGCAGCCGTAGTAGACGAACCTTTCACACCTTTTGTTCTTGTTGTTGTATCCAAATGCTACCTTAGGTGAACCATTTGGGCACTCCTTCCCGCGATGTATTGGGTAACTGCAATTGCTTTCGACGTATTTGCCGCATGTTTTCCAGCATTCGGATTCGTAGAGAAAATTGTTTCCATTTCCTCCGCAGCCTTCGTAGTCGAACTGTTCGCAGACTTTTGTTTTACTGTTGAACCAGAAGTTCTGCATGCGGCTCGACCTGCTGCAGTTGCGACCCACGTGCTTTGGGAGGAGGCATGAATCTTGGGCGAATTTTCCACATTGTGCCTGGCACTCCTCCTCAGTCGGGAAATGGTTCTCAT |
| >AAUM19276  CCGCTCAGTTCGTGCGTGTGGCGGTGGGAGAGCGAAGCAGGCAGCGGCTGAGACGCCATGGCCTCTGCGGCTGCTTTGTCGTGGTTCCTTGTCACCTCGCTGACGGGCCTTGGAATACTGCCCGCGCCGACGACACCGGCAGAGGCCCTGGTGTGTGTCCCCAAGCCGGCCAGCGCCCTCGAGGACCTCGATTACAAGAAGNCGAGGACCTCGATTACAAGAAGTTCTCGGGAGACTGGCGAGAGGTGTTGAAGAGCAAGCACGCTCCGGTTCTCCTGCAGAAAGGCTCTTGGCGCTTTGACTTCGACACCAACACGTTCGCCTTTAGCGCAGCACTTGCAGGCAATGAGACATGCCTGCCGGCCATTGTCGGCAAAGTCCACAAAGTGCTGCCCGTCGGACAATTTCAGCTCGAGTACGACTTTTTTGGGCAGCCTGTACAAGAGACGCTGGCCGTGGTAGCCACGGACTACGGGAGCTACGCCGTGCTGCACCGGTGCGCGCATGAGCAGCCCCACGCGTCGGTGTGTGCCCCGGATGCGACACACGTCAGCGTCCTTGCGAGGCACGTGCTCGAACGAGACACCACTCTGGAGATCACGCGCCACTTGGAGAACGTCTGCGTTGCTGTCGCACAGCTGGAACCGCACGAGTTTGACGAAGCATGCACTCTACCGTCCGATGAAGATCCCACCTTGGCCGAAATACCACCAGGCACGGGAATATGCTTCCTGGAACGAAAGGAAGGCTTCTGTGCGAATAAGCAGACAGTCTTCTACTACGACCGAGAGCAACGCAGCTGCCTGAACTTCACTTTCACCGGTTGTGGAGCCAACGAAAACCACTTCCTTACTCGCCAAGAATGTGTTGAAAGATGCGAGAAGCCACTGGAGCAAGCTCGAGCTCCACCTGTGGAATCCGAATCAGCCCAGTGTCCCCAGCGTGCAGCATGCGGCCTAGCCTGCGCTCACTGCTGCCAGCGGCGAGAGAAAGACAGCTGTATTGCGTGCAATTGCCTCGCGTCCGAAACAGAGCCCCTTCTCAAGAAAGCTGACTGCCTCGCGGCTCCCGAGCTGTGCCCGGCTGGTTGTCAGAGTAGGGAGGTGTCTGCGTCGTGCTACGCCTGCGAATGTGCTGCTGCAGACGGCACTGTTGAATATGCACTGGGTGCGTGCAAGTTGCCGGTGAAAGAAGGCAACTGCAATGAAAGGATTCCCCGCTACTTCTTCAACGTCACTTCCGGCGTTTGCGACGTGTTCTACTACACCGGCTGTGGTGGGAACGAGAACAGCTTCGCCTCCCATGAAGAGTGCATTGCCCAGTGTGAAGATCCCTGCCGCCTGCCAATGGATCCTGGCAGCTGCAACGACACGCAGGAGCGCTACTACTTCAACTCACAGACGGGATTGTGTGAGACCTTCGAGTACGGCGGCTGCGAAGGGAACAAGAACAACTTTGCAGACCTCGACGCGTGCAAGACGCTTTGCGAAGATGTTTGCTCGCAGCCCCAAGACCCGGGCCCATGCTACGCGTACTTCCGGCGCTTCTACTACAATAAGCAGGAGGATCGCTGCCTGCCTTTCATCTTCGGTGGCTGCATGGGCAACGGAAACAACTTTTACACCAGCACCCAGTGCAACGGACGCTGCCGATTGCATGTTGCTGAGGACGGCGCAGCCGGAAAGGACAGGGTGCATGTTGCTGAGGACGGCGTAGCCGGAAAGGACAGGGAAATGTGTCACCAGCCGGTGGACGAAGGCCACTGCGACACCGACAGCAGTGGCCCAGGGGCCACTACACCAGAGATCCGCTTCTACTACGACGTGCAGAAAGAACTGTGCGAGCGGTTCAACTACCAGGGATGTGGTGGAAACGACAACAACTTCCGTACCGTAGACGGATGCAACATGACCTGCTTTGGTGTGCGCATCAACTTGGCCAGGACGGCTGCGAGCTGCCCGGAGTCCATGCAGTGCAGCTGCGGTGATGAGCAGGAGGCCAAGTGCGGGTCATGCGAGTGCGGGAACGCAGCCCCGCGTAGCAACGGGGCCGCCCTCTACGCTCTAGTGTTCCTCACTGGTGCATCGGTGACA |
| >AAUM20559  CAAAGTACCATTTTATTTCCTCGTTGTGTACCTGCATTCATTTACTAGAGCAGTAACAGGCGTTTATACTGGTGTTAATTAGCTTACGCTGTAATGCATTGCTAATGAGAGGTATCAGAGAAAAATTAGCATTCAGTTGGAGTTGAAAGTGACAAGTTTTTTTGCGGTGGGCTTTGCGTTCAAAACTTCAATGATCTCCATCCTCATCTTACTGTCGACAAAAGAAGGACACCTAGGTGCTCTATACGAAACAGGGGCTCCTACTCAACATCCTGGGTGCTCAACCACAGCGGCTCTGGCATTCTTCGAGTGTTTGGAAGTTGTTTCCGTTGGGCCGGCAACCACCGTAGATGAACTG |
| >AAUM21589  TTTTTTTTGAAATGCGAAAATCTTAGTTTATTGCGTGATCCACTTAGTGAATTCGTTATAAAATTCTCTTCTCTTGATGTCCCACCAAAGCTTCACATGTGTAAACCAAGATACGCTGAAAACCAGTGCCGAGGTGCTTGCTGCCTTCACCCTAGTGGGCCATAGATTTCTTCTAAATATAATTCGCATGCTTTCTTGGGGTCTTCATGGCCACTGCATCGACGCATGCAGGTAGTGCAGTCAGAATAAACGTTTTCGTTTCTTTGGCATCCTCCGTACTGGAATGGTCTGCAGTGCCCTTTTTCGACATTAAAAAACCACTTTGCTTCATAGCTAGTGCACAGATCG |
| >AAUM21753  CAAAAACTCAACTAAGACAACTGAGATCCGCTGGCACTTCGACGGAACGTCTTGCAAAGCTTTCAGCTTCAACGGCTGCGGTGGAAACGAAAATAATTTCGACAGGATAGACGACTGCCAGAGGCTGTGCGAGTNNNNNNCACACATTCACTTCGAATCACCGAAATACGTTTGTTTCAAAGCAATAATAGGCTATATTGCCAATAAGAATCTTACTGAAATTTCGATACAAAGCAAGAAAAATGAAAATGATGGTCCTGCTCAAGTTTGTTTCAAAGTAACGGATTATTGGATATTCGACGATATTGCCAATAACACTCTGCCTGCAGTTTCGATAAAAGAAGAAGAAAATGAAGAAGATGATGNNGTTTTTTTCCAACTTGAATGAAGACTAGACGTTTTGGTTTGCCAAAACGGCTTTGAAGATATGTACCTGGAAGCAGAAACTAGAAATTAAA |
| >AAUM22911  AATTACAATGATCCCGCAATGCCTGGTGCTGCCAGGAGTACACGATACTGATGGGTTCGTGGTGCATTTCTACTCTTGATGTCTTTCTGGATCTCCGGAGCCTCGGCGGCACTGTCGTTCGCACATGGCGATGGTGTGGAACCTGTTGCCGTTTCCCTGGCACAGGCTGTAGAGGAATGGCAGGCACTGGTCCTTCTCCTGGTCGTAGTAGAAACGCCGCCTATTTGGCCACAACCGTGGATAACTC |
| >AAUM25346  CGACAAGTTCAACGACGCGTTCACGAGGTGTGTCCAGGACCCGTGCTCGCAGCCCCCAGAGCCCGGCTGGTGCCTGCAGAACATGGTGATGTTCTACTACGACCCGGACAGGGCCTCCTGCAACGAGTTCACCTACACCGGCTGCGGGAGGAACTTCAACAACTTCCTCACAAAGCAGGAGTGCCGCAAGATCTGCGTAGAAGGTCGGACGTGATCGGGACATTCGGCACGCGGGTGGATGACTGGGAAGACCACGGCGCGCACGAACACAGCACGCA |
| >AAUM27386  GGAAGCTCTGTCCCTCGAAAACTGAAGATATGAATGCGCTGACCAGAGTACAAGCATGCCTTCTTATCTTCCTTGTCTGCTTGACTCTCTCACATGCACGTACAGCACAAAAGAGATTGTGCTTTCTTGGCAGCAAGACAGGGCTTTGCAGAGGATATTTTCCTCGTTGGTCTTACAACCGCTGGTCTGGTGTCTGTGAAGTTTTTATCTTTGGCGGCTGTGGTGGAAATAAAAACAACTTTGAAGACTGCCAAACTTGTATGAAAACATGCACTGTGAACATTAGTTATCAAAAACGGAAAAACATTTGCCACAGGCAAACTGTAAAATACCAGCGCTTGCTAAACCCCACAGGTAGAAGGCCCAAATAACCGACCATGCCTAGATCTGTGACCCAAGGAACTTTCGCTGCAATCAACCTTCTGCAGTATCTTTCGACCAATTGAAATAAACTAATATTTGCATGA |
| >AAUM27941  CTCCAGACTACTGCAACAAGAAGCCGCAGGTTGGTCTGTGCAGAGCTCGCATTCAAAGGTGGTACTTTGATCCTTTTACCCAAACATGTAAATTTTTCTACTACGGTGGCTGTAAGGGAAACGCCAACAACTTCAAAACGAAGAAGAGGTGTCTGGAAGTCTGCCGACCGAAACGTCGGCCTGGTTCGGTTTGTCGTCTAAAGCCTGCTACAGGAATCTGCAAGGCTTACAAACCGCGCTGGTACTACGATTACAGCAGAGGGTACTGTATGGGATTCGTGTATGGAGGCTGCAAAGGAAACGACAACAGATTTGGTACCTGCTGGGAGTGCATGAGTAAATGTGGCAGAGGTCATAACATGCATCTCTGTAAGAAGCTCACACAAGAGTTCGAGAGGAACTACACAAGAGTGGGTGGAGCCTTCAAC |
| >AAUM30584  TCCGATCTCTTCACTCGAAGCTTCAAGTACTTCTTTCCTCCTTCGCTTTTAATATCATCTCCCATTGTTTTGTGCAATGATTCTCAGTTGGAAGCCGGTGCTCGCCTTAGGCGCGAGGGATGACGCCCAGGAACTTCTTGCACTTGTTGTCGCAGTCGGCGAATGTGCGGAACCTGTTCTCGTTTCCACCGCACTATCCGTAGACGAACTCCTCGCAAGTTTTCGTCAGGGTGTTGAAGTACCACATCGGCATATAAGCCAAGCATGGCCCGGAATCTTTCGGATAGGTGCACACTTGAGGCGGCTTGAATGGCCAGAACGGGGGATCAAATGTGCCAATGATGTCGATTTGCGCTTGGCTCAGGGATGCCCAACACCTGTTCTTGCACTCTTCGAGCGTCCCAAAGTTGTTTCCGTTGCCCTGGCAACCACCGTAG |
| >AAUM32925  TAAGAAGTCTATAGACTGTCTATAGACCATTTTATAAGGGTCCAGTGAGCTGCCCTGTCCGCGACCGCGGCGCCCTCTTGCGGTCAGTCCGGGATGCAGACCGCCCGGCATTCCTGGGCGGAGCGGAATCTGTTAAGGTTGCCGCCACAGCCGGAAAACCAAAAAGGAAAGCAGTGGTTCTTCTCTTTGTCGTAGTGATACCGCACCACCCAGGAGCCGCATTGGCCTCGGTCCATTGGAAGCTTGCAGTAGATGTTGTAGTTTTGGAACCAGCAAGTCCTCATGCACATCTGTCTGCTTCTGAAATTGTTTCCGGTGCCCTCGCAACCAGTGTAGACGTAGCGGATGCACCGTCCCACGCCCCAGTGGAAGTAGTAGCGGAACAGCCTCCCGTTGCAGCTTCCGGCATTTGGAGGCACGAAACAGTTGGGCTCCGTTCTTTCGTGAGGTCTGGAACGCAGCTCGCCGGTGGTGTCACCTTCGGCATGTGTCTCGTTGGCGCCTACGTTGGCAGTGACGTTGGAACTGACGTTGGCCTGAGCCGTG |
| >AAUM33331  GAAGACTGCATGCAAGAGCCGGATCCCGGAATATGCAGAGGAATGTTCAAGAAATGGTTTTACAACGCGACGTCGTACCGTTGCGAGGTATTTTACTACGGAGGCTGCAATGGAAACGGCAACAGGTTCAACAAATTTGCGGAATGCAGTAAGAAATGTCGAGACCCTGTTCTCGGAGTGTGTGCCCTGCCAGAGCCCAAACAAATTTGCCGAGCTGGATATAAGGGGTACCGGTTCAATCCGTTCAAGCAAAGATGCGTGCGTTACATCTACTGCGAGCACAATGAGAACCATTTCC |
| >AAUM34355  TGAACACAAAAGAAGTGGCCAACGGTTGCTGTTTTGCTTCAGTTACCTCAGTGCCGCAGCTTGCAATATGAGACCACAAGCTTTCATAGGTGCTTTCGTCTTTACTTTGGTACTCAGGCAAGCTGCAGGTATAAAGTGGTCGCGTTGTTTCCGACCGAAGGCTGTGGGGAACTGTCAAAATAAGGTTCCGGCCTGGTACTATGATTTTTGGAGCTTCCGTTGCAAGGGGTTCCTCTACAGCGGATGTGGCGGGNNNNNNNNNNNNNNNNNNNNNNNNNNNNNNNNNNNNNNNNNNNNNNNACCGAAGAAGAATGTCAGAAATCCTGCTTACGCAAATCTAAGCGGAAGGAGGTTTGCAGCTTGAAGCCGAAAACTGGTAAATGCAAGGCCGCTATTCCATTATGGTACTACGACCCCGAATTGGACGAATGCCGTGGCCTCATTTACGGGGGCTGTAAAGGAAACG |
| >AAUM34683  CGAAGTTATAACGATAAATAGTTTTATCGCACGGTCCCTTGTCTGCCTCCACAAAGCAGTCCAGAAATGCTTGCGGGTCCGTTTCCTTTATGTCCGGATTGCTCGTTTGCGAACCTCCTCCTCGCCTCAAACAGGTAAGTTCACATGCCTCCTTGCTGACGTAGTTGTTGTAGTTGCCCTCACAACCGGTGTACACGAATCGCTCGCACGTTCTCACCACGGCATTGAACCACCAGCTTGGAAAGTGACCTCTGCAAGGACCACGCTCAGCACGCGGCGTGCAGTATGTCTCGAAGTCGTAGTCGGCCTCCGTTTCTTCTTCGGCAAGGAGCGTGATGGTATTCGGAGCCTGGGCG |
| >AAUM37519  TTCACATGTGTAAACCAAGATATGCTGAAAATCCCCGCCGTGGTGCTTGCTGCCTTCATCCTTGCTGGCCATACATTACTTCAAGATATGATTCGCATGCTCTATTGGGATCATCATGGGCAGTGCATCGACTCATGCAGGCAGTGCAATTAGAAAAAACGTTTTTGTTTTGCCCGCATCCGCCATACTGAAATGGCTTGCAGTGCCCTGCATCCGCATCAAAGAACCACTTAGGTTCAAAGCTGTTGCACCGACCGATGTCGGCTTTTTCAAGGCACTCTGGAGGGAGTGAAAACGACTTGGCCGACACCACAGTCAGGACAGCAAATAGAAAGAAGAAGGTTCGAGGGTCCATCTTCAGGCTCCTTTCTCCGAATGCTATTCCAGTCAAG |
| >AAUM39887  CTAAAATACCGGCTTTCGATTGCTGCCATGGAAATCCGGAGCTGTGCTTATGTTCTTTTAATTCTGGCCACCGTCCACGGTACACCAGTAAACAATTCGTCCAGCTGCTCTTTGCCTCAAGTAAAAGGCAACTGCAGAGGGCTATTTGACATGTGGCACTACAACTCTACCAATGATAGATGCTCACTCTTCACATATGGAGGATGCGGCGGCAACGAAAACCGGTTTGAGAACTGCACGCACTGCATGGATTCATGTAGCGCAAACGAAAATCTAACAGAAATTTGCCGACTGCTTGAAAAAGAGGCTGATGAAGAGTACAATTCGGGATGGGAAGATTATAC |
| >AAUM41053  AGCGGCCTGAAGCTCTCGTCCCGTCACTTGTGCAGGGCCAGTGTCTACCGGTATGGACGGACGCACAATGAAGTTCCTCGTAGTGCTTGCGATTTTTGGAGCAGCACTGGCAAGCTCTGATACTTCGTGCCAGGAGCCAAAGTATCCGGGTCCTTGCAAGGGGTACTTCCCTCGCTACTACTTCAACAAAGNNGTTCATCTACGGTGGTTGCCAGACCAACGGAAACAACTTCGCAACGCTCCAAGAATGTCAGAGCCACTGTGGTTGAGCTGCAAGATATTTGGATGGAGCCCCGTTTGGAATAAACACATATCTGTTCGTGCTTTGGCGACAGCC |
| >AAUM43642  TTGCCAAGACTGTGAGGACCCTTCAAAATGTACATATCCAGATGGTTGCAAGGAACCTCAAAAAATTGGACCATGCAAGGCGCTTGTGCCGAAGTACTTTTTCAACTCTACAACAATGAAGTGCGAGCCGTTCAAATGGGGAGGGTGCTGCGCAAACTGCAATAATTTCCAAGCACTGGACGAATGTGAACGAAACTGTCTTAACCAATAATGGCATGCAGTTCGCATTTACCTCTGTGTGATATGCATTCGTCAGAAGCACCGTCAGCATGAAGAAATAAAAGTAAAAAAG |
| >AAUM43647  ATGAATATCTACCAAGCTTTAGGCTTCTTCTTTCTTCTACCAATGTGCTTGGCCATGCATAGCAGCATAAGCCCGAAAGACCCTGCCGTGTGCTATGCTCCACGCCCTGCCAGCTACTGCGATAGACCTGAAAACGTCTTTCCCGTCTTTTTTTATTATCCTGGGAGTCAATCATGTTTTCAAGATGTTGGTTGTACTCTGATGGGTAATAACTTTGCAAGCCTAGAAGAATGCGAAAGAGTCTGCTTAAGAGGAA |
| >AAUM44374  CTACTTCACGCACACAAATAAACAACATTGAATGGTTGCACCATGTTTGTTTCAATCGCATTTGGATGGTGCTCTGATTTTTGCTTAAACGCTTGCCTTGGGTTTTTGTTCCATAGGTTCGAAGTTGCTTCCGAAGTACATCAGCCAGATCCGCGCATGCAGGCCTTCTGGCATTCCCACTCGGTGTTGTACCGGTTGGCACTGCCACCGCATCCACCGTAGACGAAGGGCTTGCAGACACCGTCGGCCACGTCGAAGTACCAGCGCTCAAAGTCTGCCCTGCACATTCCGCTCTCAGGGGGAGGCCTGCAGCCCAAGTCAAAGTTCACGCCGCGGTGCTCATTTCGGACTTGCACCTCCGCCGTTGCTGCTG |
| >AAUM44564  CGGGAACCTGCAGTGAAATGCATGCTAAATGGTTCTACGACGTAAAGAGCGGGCTCTGCCTGCCATTTGTGTACACCGGCTGTGGAGGCAATAAGAACCGCTTCAAGTCGGCCGAGATCTGCCGGAAGATCTGCTCTGGGGTCACAATGGACAGCATTGATGACGAAATCATCGATCCTGATGCGGACAGGAAATGGATGCCGCCTTCCCCTCCAGAGCCTAGTGACACAAGCGAAGAGTTGGACCCACGTGTGAAGCCACCAATGGCAGTCACACCACTTCCAGAGCCTAGTTC |
| >AAUM45648  TTTTCAAAAATGCAGAGAGAAGAATATCTTTCCAGCAGGCAGTAAAATCTAGGTGACGCTGGTTATTCGGCTCTTCTTGCATTGATTGGCTTGTGCAAAAAGCTATTTCCACTGTTCTGCTTCTCCAGCCTTTTGCAAGCGATCTTTGCTTTTCGCCATCTCAGTCCGCCACATTTCGTCATGCAGTCCTCGCAGTGATCAAAACTGTTATCATTTCCCTGGCAGCCGCCGTAGATAAAGAAAACACAGGAACCTGTTTTGATGTTATACCACCACTTGGGCATGGATGCTTTGCAGGGCCCAACGACTTTTGGAAGGGAGCAGACTTTTTTTCTGTTGAAACGGGCCTGTGAAAGAGCCAGGCCGAGGAGGAGCAGAAAGAGGCACGCCGGTAACCTGGCCATCGTAGTCATTTTTCGAATCTTTGCCAAAAATGCGGCTTTGTCG |
| >AAUM49763  CTTGACTCGAACCTGTTGGCGTTGCCCTCACAGCCTCCATAGTAGAACTGCTCGCAACGACCAGCGGCCATGTTGTAGTACCAGTGCACAATGGCTGCCCGACACGGACCCTCCTGCTTGGGCAAGGCGCAAACGTCTCGGGGCACGGTCACCCTGGTGGAGTTGCACATGCGCTCGCATTGCTCACGGGTGTTGAAGCGGTTTCGGTTGCCCTGGCAACCTGTGTAGACAAACTCCTTGCAGAGCCCCTCCACATAGTCGTAGTACCACTTGCGGTCTTCCGACAGGCACTGGCCTACCTCCTTGGACAGGATGCAGATATCCTTTGCACCGAGAGAGACACATCGTTCGGAGCATTCTTC |
| >AAUM53436  GTTCTGTGTCGTCCTCCATCTCCATCGGCTCGCACCAGTCTTTGACCTGCCTGTACCCTGGCATGCAGGCACATGTCTGAGCGCTGCACATGAGGTGCGAGCTGCACTCGTTGTCGTGGCTGCAGTTTTCACCGAGGAGCATCAGCGGAATGCAACTGTTGTTCTTCCAGTTGTAATAGTGGCCCCGGATGCAGAAACAACTGGAGTACATGCACTGGCTCTTGGGCAGTAGATTTTGGCACTGTGACGTTGTGTTGCAGGCGGTCCCGATCAGACGCTTCTGGATTATAGCGGAGTCGACCGTCATCGCGAAACAGATAACTTCCAGCACGGCGGTAGTAAGCAACAACCAGGGCATCTTGGTCTTTGTGTGTCGCTACGGTGAAGGTTCTGGCCTC |
| >AAUM57402  AAGGAGGAGTGCGAAATGACTTGCCTGCGTCAGCCAGGGAATGTTAACCCCCTCTGTTTCGAGCCAAAGTATCCTGGGCCATGCGGGGCGCACTACCCCCGCTACTACTACAACCGGTGGTCAAAAACCTGCGAGAAGTTCATCTACGGCGGCTGCCGGGGCAACGAGAACAATTTCGAGACTCTGGAAGAGTGCGAGAACACATGCTGGGTCTCAAGGAAACAGGATCCAGCAGATGTCAGCGAAGCATTTCAAGCTCCATTCAGGCCGTGGGCAACGCCTTTGGAGTGCACTTATCCGGCAGAGGCAGGACGTTGCTTGGCTTACATGCCACGTTTCTACTACAACGCGACGACCCAGTCCTGCGAGCAGTTCATCTACGGAGGATGTGGAGGGAATGCGAACAACTTCTACAGCTACGCCGACTGCGAAAGCAAATGCAAGACGTCCATGGGCATTCTCCCTCGCGCTTGAGCATGAGACGGCAGCAGAAAATAGAACACAAAAAGAGAACAGAGTTTATAGCAGGCTAATTGCCTCAACCTGTGAATTTTTAAGAGTTGTAACAAACAAATATTTCTATGGCTAGCTTACTTGCTCAGTGCGCTTGTGGCCGATTCTTTTCTTTCTTGAGATAATCTTTTTGATCATTTTATTTGATAATGCATTAAACAAAATTGGGAACACATGTGAAAATGATTTAAGGAGGAACTGGTTCTGATACTCTGGCTCAATAGTCTTTGTGACATATGTGACTTAATAAAAAAT |
| >AAUM59577  CTTTCTTATGCACATGGCTAGACATAATTGATGCTATCAGTTTGTATATAATGGAAGGTGGCCGTTATTAGAATCGTCTTGCGTTGGGTGTACCCCTCGGTGTGAGGTTCGCACTTGCTTGTTTTTCCAGTTTTTGACAAATCCTCTTTATACTCGGCCAGGGTTTTANNNNNNNNNCCCGCATGCATTTCTCGCAGGTTCCAAATTTGTTCCTATTTCCCCCACAGCCGCCGTAAATAAAAACTCTACATCCACCTTTAGACCAGTTAAAATACCACATTGGAAAGTAGGCTCTGCAGACCCCAGTCTCTTTTGGAAGCCTGCAGACTTTGTTTCGTAGACGTCCTTGTGAGACAGAGAGGCAGAAGAGGAGGAGAAGAAGGCACGACTGTAGTCTCGTAAATGAATTCATGTTTCAG |
| >AAUM59928  GCTTTATTTGTTGTTCTTCTAGAGTGATGTCAAGTTGCTGTTTCCACCTTGAAACAGCCTCTAATGAATTATAGGCACGGGCCCCAGGGTGGCGCAGACGGCGAAGCACTCGCGCATTGTCTCGAAGCTGTTGCCGTTGCCGTGGCAGCCTCCATAGAGGAACGACTTGCATTGGTTTGTCTTAGCTTCGTAGTAGAAGCGTGGGATGAAAGCCACACACGGGCCGGGGTACGGTGGGAGAAAACACAGATCAGGCTTTTTCTTCGCAGGCTCACTGAGCAACACTGTGGCTTCTTGTTTTGGCTGGAAGATAGCAAATGCGGAGCTGACAACAGCGAGGAGAATGTAAGCCTTCATGTTGCCGTACTGGTGGCGTCTGC |
| >AAUM61041  AAAAAATACATAGCAGGTGTCTGGTAGTAACACAAGCGCATCCCCTTTTTGAAATGTTATGACTGTCGAACCCTGTACGGGTACCCAAACCTTTCCAACAACAATGACCTAAATCTTTCCATTTCTCTAATCGGCCCGTGGCACACCCGCAATATGTTCGAGACTTTTCTCCCGGTGCAACTCTTCATGCATCCCACGCAAGTTCTAAAGTTGTTGGCATTTCCGTAGCATCCGCCGTATACAAAAAAACGACAGTGGCCTGCAGCTGGGTCGTACGCCCAAG |
| >AAUM62165  CTCTCGTATCGGTTCTCGTTTCCTCCGCAGCCGCCGTAGTTGAAAAGCTCGCACTTTCCAGTCCTGGTGTTGAACCACCACATCGGCATCATCGCCTTGCAGGGACCGCTATCGGGCAGCTGGGTGCATCTCATGTCAAACGCAGTCGCTGATCCGCCACATCTTCAGCTTGCTCCACTGGACAGAAAAGCACTCCATCACTACCGATTGCGGAGACTTGAGATGGCTGCGCCACTAGGAGCCACCTCAATGCGCTGAAAACCTCAACAGATGGGCTTGGGCAAGTTCTGTCTTGTCC |
| >AAUM64840  TATTGCATGAAGCAGATCTCTTGTCTGAGCTCTTCCTCAGGAGCATCTGTCACAGCTGGCCGCCCGCAGGCCTGTTCACATTCCAGGTGTGAAGCAAAGTTGTTCTCATTTCCGTCGCAGCCGCCATAGTAGAAACGATGGCAGCGGCCTTCCTCAGCATTGTAGTACCACCGCTCACGGAAGTCGACACAGTTTCCAGCCTCCTGTGGCAGCACGCAGATGTTCCGCCCTCCGGGGGAGAGGCAGGCTCGCTCACAGTCACGCCTTGACTCGAACCTGTTGG |

| >AAUM65109  CACGGTTGCTGTCCTGATGGACGAACGCCGGCTCGGGGTCCGGATGCCGATGGCTGCACCTGTGCCCTGACTGCGTATGGCTGCTGTCGTGATGGCGTCACGGCTGCAAGGGGGCACCACTTTGAGGGCTGTCCGGACAAGATCATTGTCCCNNACAGGAATGTCAGCGCCAGTGTATGTGGCCTGCCCGNNNNNNNNNNNNNNNNNNNNNNNNNNNNNNNNNNNNNATTCTTCAGCGTTGCTGACGGCCGCTGCAACCGGTTCTGGTATGGCGGCTGTGAGGGCAACGAAAACCGGTTCAACTCTGAAGAGGAGTGTGAGAACACT |
| --- |

| >AAUF35  ACTGAACTCCACCGGCGTCCGCGACCATCTCAGGTGTCGACGATGCTTCGATTGACTGTGCTGGCGGCTGTGCTTTTGGCCATCTCGTTCAATGGTGCCAATGCTCAGAGAAGGCCCAGATTTTGCAGCCTGCCGCCTAGTCCCGGAGTCTGCTTCGCCTACTTCCCCTCGTTCTATTATGACATTTCATCCAGGACGTGCCGAGAGTTCGTTTACGGCGGATGCCAAGGAAACCAGAATCGCTTTGTTTCATATGAGGAATGCCTCCGCGTCTGCGGCTGACATCACTTCTGGATCGGAAGTACCAATGACAGCAATAGCATCGCATTACGCTACGTACCTTATGTTCCTGACTGAAACACCTAAGTGAGACCTGCTGTCCCACTTACAAAGTTATGGCACAGTAAAAAACTAGTCTGCAACAACCGAGTCTCATGAATATTTCCTTTTCATAACAGCTCCATACCACGTTGCTGTTGCGATTCAACTCGCCCGCTCAG |
| --- |
| >AAUF3188  GTCGGTGGCACTCTGCTGGGAAATCCACAGGCAACGAGAAGGCAATCCGAACGCTGCGGAGCGAGGGTTTGCGCCTGCGCCTAACCTGACGCACTAGATCGGGTCCCGGATGATGTCAGCTCCAACCGACCACACGTGCGCCGCTGCGCGCCCAGGAAACCGGAATCTCGAAGGTGCCGTTGCTGCTCGGCAGAACGCCGCCGCGCGCATATTTGACCGACTCAAGGCTGCCCAGCATCCTCACTCGTGAGAAACGCACCCACCCGGACCGCCCGTTCGCAAGATGCCTCGTTCGCTTGTGGTCGTCGCCTTGCTGCTGGCTATTGTCGTAGCAGGCACCAATGCTCAAGGCCAGTGCTTCCTTCCTCCTAGCCAGGGACCCTGCCGCGGCGCCTTCCCAGCCTTCTACTTCGATAGTAGATCGGGAAGATGCCTCTCGTTCACCTACGGCGGATGCGGTGGCAACGCGAACAGATTCAACTCTGTTGGACAATGCCGCAGAGTGTGCGGCCGATATTAGGGCTCGTACGCTCAAAGCAGCGCCAGCTCGAAATGCACTGCCGCAGGCGACGCGCGCGTTTTGACGAAAAAACAGGCTGAGCGCGCCAAAAATAAAGGTTTTCTATGTCATTTTTTATCTTATTTCTTGTGTTGTGTGTGTGTTTTACTGCTTTTTTGAGTGTTTCCTAGGTTCACGCTACCAAAGTGTGGCTTTTGTAAGGCTACTGTAGCCCATTTTGTCTTGCTACTGACTGTGTGTAGTCGGTCACTAGAGCGGCTATCGAATCGGTGAGACCAGGAGCTATTTTAATAAAAAATCTGCATCAACTATCAGTCCAAGCAACAATGAAAATGAATCCACATTTATTTGTATTAAATATGCAAGAATGCTTGTGTACTCAGCTGTTGCAGGCTGCAGAACCACTCACCTTATCTAAGATAAACCGAGCGCCCCTCTGCAGCGTGAGTCATAGCCCACAGTTATTTTTTATAAGTAAAAATATTTATCAAAGAATTAACGTTGTATTCCTTCACGATTTTTTTTCTCTCCACTTCTACTCTTGAGGCCTTCCTGTATATTGTACCGCGGAGAGTGGCTGACGGGCATACTCACACGCAGGGTTCCTCAGCTCTGCTCCGATTAAACAAGGAAGACGTAACAATTGTTCTCCCCCTTTGCATGCGTATATGAGGAAGTCAGAGGCCGACCATTAATATGAAATGTTTTTGTGTGAATATTTCACACAAAGCCCCGTTTGTTTGTTTTTTCTTTCAACAAAAGCGGTCCAGCAAATTCATATGCAATCTTGACAGAAATGGTCCCAGATGCTTGCTACCATAAGTTATTTTAGAACTAGCTCATGCACTCCGGATGCTTCTCAGAACTTCGAAATTATTCCAAGCTTTTTATACTCCGAATTTTCTTTGGCCG |
| >AAUF5073  TTCGCATGTGGCCAAGACTGTGGTCCACCTCCCCCGCCATGCAACTATCCAGATGGTTGCAAGCAGCAGCAACAAGTTGGGCCATGCAAGGCAATTGTGCCGAGGTGGTATTTCAACCCTCTGACCAAGAAGTGCGAGCAGTTCAAATGGGGAGGATGCTGCCCAAATTGCAACAATTTCGCAACGTTTGAAGAATGCCAAAGGAGATGCCTTCCTCAACCATAACATTCTGTTCGCAGTTCCTTCTATCTGCGTCTTGCATTAAGCAAGAGAGACGAACAGTGTTTTGAAATAAACAAGAAAATCGTCGGAATGTAAGTTTTCGCTCCTTCACGTGGAGGCAAAAGCTGGCATCATTTTGTCCCAGTAAACGTAATATCCT |
| >AAUF5123  TTTATTTCACAACGCTCTTTGCCGCTGGTCAATGCGAAACACGCAAAGGGAACTCGAACAGCTTTTGAAGATTAACGAACGCATGTTCTTTGGCATTCCTGAAGCGTTGCGAAATTGTTGCAGTTTGGGCAGCATCCACCCCATTTGAACTGCTCGCACTTCTTGGTCACAGGGTTGAAATAGTACCTCAGCACGATTGTATTGCATGGTCCAACTTTTTG |
| >AAUF5151  TTTATTTCACAACGCTCTTTGCTGCTGCTCAATGCGAAGCATGCAGAGGGAACTCGAACAGCTTTTGAAGATTAACGAAGGCATGTTCTTTGGCAATCCTGTAGCGTTGCGAAATTGTTGCAGTTTGGGCAGCATCCACCCCATTTGAACTGCTCGCACATCTTGGTCACAGGGTTGAAATAGTACCTCAGCACAAATTTATTGCATGGTCCAACTTTTTG |
| >AAUF6393  TTTATGGTTATCACAGTGAATATTTTATTAGGTCAGAAAAAATTTTATTTATTGTGTCCACGTTTCCATCATACAAAATATTGACGTCAGCAGCCTCGGGGAATGCGTTTGTGGGGAAAGCAGACTTCCAGGCATTCTTCCTTCGAGTCAAACAAGTTTGGTCCGCCGTTGCAGCCGCCGTATAAGAACTGAAAGCAATTCTTGTGGTAGTGGTCGTAGTACCACTTGAAAGCAAATGCGTAACATGGTCCACGGTTTGGCCAGTGACGACAGTCCTCCGGGGATCTTCGTCGTTCGCAGACCTTGCGGCACTCCGGCATGCTGGGGAAGTTGTTACCGCGATTGTGACAGCC |
| >AAUF6421  AACAAAGGCGCCTTCTGAGCAAAGCTTCAAAAAATGACCGCAATGACCAGATTACCAGCGTGGCTGCTTCTGCTTCTCGTCGGCGTAGCTTTTTCACATGCCTGGTTTAACAGAAAAAAAGTCTGCACCCAGCGAAAAGAAGTTGGACCCTGCAGAGCATCGATACCTATGTGGTGGTATAACCCAAAAAGACAATACTGCAATTTGTTTATCTACGGTGGCTGTCAAGGAAATCAGAATAAATTTCAACACTGCGACGAGTGTATGAAGAAATGCGGTGGAATGGGCTGGCAGAAAGCGAAGAAGTTTTGCAGTAAGCTGGAGAAGCCAATTGGTACAAACACCGGCCCTTACAAGCCAAACTATGCAAGAAGACCGAAGTAATCAGCGTAATCTAAAGTGTGCTGAGTACTGGAAACACATTTCTCTCTCGGCACTTCAGGAAAAGTTCCCGCACTTGAAATAAACTCAGCTTTCGG |
| >AAUF8938  ACCCAGCTGAAGGAAAATGCTCTTTCTTCATCTACGGTGGTTGTGAAGGAAACGAAAACAACTTCGAAACTATCGAGGAATGCGAAAAAACATGCGGCGAGCCAGAGAGGTCAAGTGACTTCGAGGGAGCTGACTTCGAGACTGGTTGCGCGCCAAAACCGCAACGCGGCTATTGCAAGGGCTTCTTAGACCACTGGTTCTTCAACGTGACCTCGGGTCAGTGCGAGACGTTCCTCTACAGTGGCTGCGGTGGAAACGACAACAACTATGAGTCGAAGGAAGAGTGCGAGATTGCCTGCCAGCTTACGTAGCCCGCTGGACAAATGAAATGGGCACTTCGCTCTCTTTTTTGCCGTGTTCATTCAGCACATGCATTGGGCGAACACAAAGCCTGCAAGACATGAGAGAACGGCTGTTGTAGTTTTTCAGAGAAATAAAAACAAACTATACAATAAAAAAA |
| >AAUF8939  AGGAATGCGAAAAAACATGTGGTGAACCAGAGAAGCCCAGTGACTTCGAGGGAGCTGACTTCGAGACTGGTTGCGCGCCAAAACCGCAACGCGGCTTTTGCAAGGGCTTCTTAGATCACTGGTTCTTCAACGTGACCTCGGGTCAGTGCGAGACGTTCCTTTATAGTGGCTGCGGTGGAAACGACAACAACTACGAGTCGAAGGAAGAGTGCGAGATTGCCTGCAAGCTTACATAGCCCCCTGGACAAATGAAATGAGCACTTCGCTCTCACTTTTGCCCTGCTCATTCCGCACATGCATTGGGCGAACACA |
| >AAUF9570  TCCCGCGATGTATTGGGTAACTGCAATTGCTTTCGACGTATTTGCCGCATGTTTTCCAGCATTCGGATTCGTAGAGAAAATTGTTTCCATTTCCTCCGCAGCCTTCGTAGTCAAACTGTTCGCAGGCTTTTGTTTGACTGTTGAACCAGAAGTTCTGCATGCGGCTCGACCTGCTGCAGTTTCGACCCGAGTGCTTTGGGAGGAGGCATGAATCTTGGGCGAATTTTCCACATTGCGCCTGGCACTCCTTTTCAGTCGGGAAATGGTTCTCATTGTGCTCGCAGTAGATGTAACGCACGCATCTTTGCTTGAACGGATTGAACCGGTACCCCTTATATCCAGCTCGGCAAATTTGTTTGGGCTCTGGCAGGGCACACACTCCGAGAACAGGGTCTCGACATTTCTTACTGCATTCCGCAAATTTGTTGAACCTGTTGCCGTTTCCATTGCAGCCTCCGTAGTAAAATACCTCGCAACGGTACGACGTCGCGTTGTAAAACCATTTCTTGAACATTCCTCTGCATATTCCGGGATCCGGCTCTTGCATGCAGTCTTC |
| >AAUF9721  TAGATTTCTTCTAAATATGATTCGCATGCTTTCTTGGGGTCTTCATGGCCAGTGCATCGACTCATGCAGGCACTGCAGTCAGGATAAACGTTTTCGTTTCTTGGGCATCCTCCGTGCTGGAATGGTCTGCAGTGCCCTTTGTCGGCATCAAAAAACCACTTTGGTTCATAGCTAGTGCACAGATTGATGTCGGGTTCTTTAAGGCATTCTGGAGGCATTGAGAATACTGCGAAAAAAAGAATTCAAGCTTGAAAACCCGTCATTACCGACGTTTTCCTGGCGTAACGCAGCCTGACAGCTCTGCACACTTCTGCTATTGCGATTAGCAAAAGAGAAGAAAAAAATTAAACATGTTTACTTGACTTTTGCTCAAGGCGCTCATGAACGCTTTTCATTTCTATACGCCTTTTTGAGCACGGAGTGAACGATGCAATAAAAGAGAAGATATATAGAATGCTAGTGGACCTTTTCAATATGCGAAAAAAATGTGAAGATACCTACACTAGGAAAAATTATCGACAATTCTCTTGATGCACATTAAGGATGAGCAAAGAATAAAGAATACAGTGTATCTAACCATTCTCTTTCAATCTGGGCAAGGCTTACTGTCACCCTCATAAAACGCGTCATCGGCAAATTAATCAAAGTGGGCATGAAGATGGAAGTACATTTTTATGTTTTTCAAAGTAAGGAGCCCGAATTTAGACCAATCGTCTGTGGGAGTAAGTACCATGTGTCAATACGATACTCAGTTCTACTACTTGATGTGTGACTATGTTCCTTGCGCATTCGCTGGTTGACAGCATCTATATGCATGCATATGGTTTCTCCAAATTAAACAGCATCTATATGCATGCATGTGGTTTCTGCAAATTAAACCAGTAGTGAGTGGTGCTCTGTCCCGTTCCCTTCTCTCGTGTGCATGTGTCCTTTTAGTGCCTTTAACGTACAACGATCAATTACCAACTAGCCCAACAAGAAGTTCTCTTAGGCTGACGAAAGAATAAGACACGATAAGTTGTCCTCTGCTCTGTTGACAAATGGTCGTGCGACCTTGCTCTGTCTAAACTTCACTCAAAGCGGATCCTGAAGGCGTGATCGCAGAAACGAGCGCTTGCAAGAACTCACGCAGAAGAACTAAGGTTCACTAATGTGAGACACTCACACCTGGCCGACACCACAGTAAGGATAGTAAGTAGAAAAAAGCAGGTTCGAGGGTCCATCTTCGCGCTCCTTTCTCTGAATACTATTCCAGAGAAGAAGGATTACAAAGGCTGGCCTGGGGCGGCTCAGGACCCCGCTTTATATACGAAAGCACAACGTGGGTTTCTGTTCACGGGACACCATATTGCGGTCATCGCAAGACCAACTTGTTAAGTCATAGCAGGGGTCTTTTTTCAGTGATGGCGATCAAGCATTTTTCTGGAAGCGTTATTGTTCCACCATTACCAGAAACAATGTTTCTGAAATGACGTCATTGGCAGGGTTCGGAGAAGCACATGCGTTGTATATGTCAAGCAGAATTATATATTCATACAAAACTGGCCCTGATACATATGTGTCTGCTCTAATGGCGATGCACTGATTTTTCTAGTTTCCTCTTGCAAAACAAAGCGCTGAAAATTGATACAGTGCAGGCATAAGTCGTTTTTTTTCGTAAACAGAGATCCAAACAGCCTTTCAACAGCCTTCGTTTTTCTTCTTTTTTACCTCTCTCGTCATTATCTTGGCGTCTGTGGTAATGTTCTAACGAACCATTGCATCACATGTGTCTAGCAGGAATATGTATTCAATGCAAATAAAGCAACCTGAAATGCCCGCGTCCAGTGCAATACCGAAGCGT |

| >AAUF10905  CACGGGGTGACACTGCACCTGCTTGAGGCCTGACGATCCGAGACAGCTGGACGGTTGGACCGATTACCCTGCCCAATGTGAGGGCCGAAACTGCAGCGCTCCCTGAAGCTACGGCTTAAAGACTTGGGACGACATCTCTGGAAAATATGACTTGGATTGCGCTTGCAGCAACCGTATTTCTGGTGTCGTTAACAGGATGCGTGTGTTTCGAGGAAGACAAAGCCTACATGCAGTGCACGGAATATCCCGATCGGGGACCCTGCAAAGGCACAGTGTACCGCTACTACTTCAACTTCATGCGAGGGACTTGCCGCCTCTTCGTCTACGGCGGATGCGAAGGCAACGACAACAACTTCAAAAGCCGCAAGCAGTGCCTGCGACGATGCGCAAGCGTTATTACGTCGAGAATCTGCAAGCTGCCCCCTAATCGTGGCTACGGCCCTTCGCGCGTGTGGCATTACTACTTCGACTCGAAGAAGCAGTCGTGCCGCCCGTTCGTCTTCCTGGGCTTCGGAGGGAACCGCAACAACTTCATATCCAGCGACGAGTGCCGCATGCAGTGCCTGGGCAAGGAGGCCCACGAGAGGGGGGAAGACTGATGCGAGCCGGAACACGCAAGCGAAAGCAGCCACTCTACACTACGCTTTGGAAGTCGACTCTGGACGCTTGGACTGTGAATGCCTGGGGAAAAACGCGATGGCATCGTTATCAAAAATAAAAACATAAGCGACAAAAG |
| --- |
| >AAUF14175  CCTTCGTAAGCGGGTATCGTCCACCAAACCCAATATGCAGAGAACCTGGGCGGAAAACTGGACGCTGCAAAGCTTTCTTCCTCAACTGGTCATATAACACAAAAAGCGGCTTGTGCGAAGCATTTATCTACGGCGGATGTCGAGGAACTCGGAACAGATTTGACAGCTGCAATGCATGCATGAAAAAGTGTGCTCAAAAATTTACTAAGAAAGACAGAGAGTATTGCCAACAGTTGACTGAAAAAGCCAACAAGAAATACTTCCCGACGGCCATGCCGAAATAACCGGAACCGCCAAGCTTAATTAAGGCTGTCAAAATACGCGACAATCTCCTTTCTCGAAAAGTACTTCAGCGAGTTCATTAAACTGTCTAATGCTTGCGAAAGCCAGAAAGTGCTGCACGCATATTTTGGATTCTAGTTCTGCCCCTGGCTCCGGAGAATAAAG |
| >AAUF15425  TAGATTTCTTCTAAATATGATTGGCATGCATTCTTGGGGTCTCCATGGCCACTGCATCGACGCATGCAGGTAGTGCAGTCAGGATAAACGTTTTCGTTTCTTTGACATCCTCCGTACTGGAATGGTCTGCAGTGCCCTTTTTCGACATCATAAAACCACTTTGGTTCATAGCTAGTGCACAGATCGATGTCGGGTTCTTTAAGGCAT |
| >AAUF15546  CTGAAGGAAAATGCTCCTTCTTTATCTACGGTGGTTGTGAAGGAAACGAAAACAACTTCGAAACTATCGAGGAATGCGAAAAAACATGTGCCGAACCAGAGAGGTCCAGTGACTTCGATGGAGCTGACTTCGAGACTGGTTGCGCGCCAAAACCGCAACGCGGCTTTTGCAAGGGCTTCTTAGATCATTGGTTCTTCAACGTGACCTCGGGCGAGTGCGAGGCGTTCCTCTACAGTGGCTGCGGTGGAAACGACAACAACTACGAGTCGAAGGAAGAGTGCGAGATTGCCTGCAAGCTTACGTAGCCCGCTGGACAAATGAAATGAACACTTCGCTCTCTCTTTTGCCGTGTTCGTTCCTCACATGCATTGGGCGAACACAAAGCCTGCAAGACATGAG |
| >AAUF16868  GTTGAAAATAACCATGGATCTTTAACCTGCTTTGCACGTATTCTGACACTCTGCTAACGTGGGAAAGTTGTTGCCGTAGTATCTGCAGCCTCCGACATCCAGCACGCACTTGCCGCTCGACTTTTCCAGATAGTAAACCGGGAAGAAACGGGTGCAGGTTTCGAACGCATGGTGCCAATTGCAGACCACCGGAGTGCGACAGGTTCCGTCGCATTCTCTTCTGGTTACAAATGAGCCTCGCCCACATGTCCATGCGCATTTCTTCTTAGAAGCTACATATACGAAGCTCTTATGCTGGCAAGGTCTAT |
| >AAUF17017  GTGAAACTTGGATGGCTGGTTTATTCGGAACCTCTAAATGTGTTTCAATTTCATATTCAGAGCTGTTTTAAGCCTTTAGTGGCTGTGTCCCATTTTCATTGTCTTCTAGACGAGTTCTTTCCACCTTTGGTTGTGTTATTCTGGTTGCGAATAGGGAATCCGCTGCAATGAAATATGCAAAGTGTTTTCGTGCTGAACGAGTTCTTTTCCATCTCCGCTCCTGCGTTCTCGCACATTAAATACTCCTGGCAAACTCCTGATGTTGAGTTGTAGAAGAATCTTTTCATTTCTCTGTTGTAGTTTTGCCCAACGCACAGACCCGGGTGAGGAGTCATTCCGCATATATCCAGGGTTTCACTTTGGTTGCACTTCATCGCGCAGTCAATTCGTTTCTCATAGACATCCTCTTTAAGGCACCCGTTCCATCGAAATTGCTGGCAACGGCGTGATGTCGTATTGTAAAAATATCTTGGGACTTTTGCTTCGCATCGAGGTTCATATTCCATCAGCGGTATCTTGCAGGCTCCATGATTGGCCACCTTTCGTTGCTCTGAACGTGATGCTGTCACAAGAGTCAACCCGAGGAGACAAATGAGCAGACAGTGCATCGCAGTTTCCTTTCAGGCGTAGTGGCAGAAGTGAG |
| >AAUF19034  CTGCAATGCTCACATCATATTATAAAATGGAACCAGTGTCTGGAGGTGCATTCTGCTCGAATGCGATTGTCTTTCAACATGCTCTCCAAGGCCGCATCAGCCACGAAGAGCGGTGATGAGCGAAGCCCTTCGTCTCGAAGCTCGCCCTTGATGACTGCCCCTGTGGCATCAGAAGCTTGCGATGCTAGTGGTGTAGTTAGCTTTGCATGTCGCCTCACAGTCACCCTTGGTGTTGAACCTGTTCTTCTTGCTTGAGAACGGAGAGTATCGCGAAGTATTGCAGCTGTCGCTCTCGACGTCGTAGTAGTATTTTTTCACAAGTCCAAACCTATTCTTAGGACCCGGTTCTACGCACTTGCTCCCAGAGTTCCGGCCACAAGTGTTCCAGCACTCCCTTGCGGAGTCAAACTTGTTGGGGTAGCCACCGCAGCCGTAGTAGACGAACCTTTCACACCTTTTGTTCTTGTTGTTGTATCCAAATGCTACCTTATGTGAACCATTTGGGCACTCCCT |
| >AAUF19035  CTCAGCACTTTTTTATTTGTGGGTCATTTCAATGCTTGTTCTTAAGCAGCATCGAATACTAAGAGCGTCTCAATCAACAAACTTTATTACACATTTCATTTCATTGGGGTCGTAGCCTGCAGCGCAGGTTGACTGCAATGCTCAGATCGTATTAAAAAATGGCACCAGTGTCTGGAAGTGCATTCTGCTCGAATGCGATTGTCTTTCAACATGCTCTCCAAGTCCGCATCAGCCACGATGACCAATGACGAGCGAAGCCATTCGTCTGGAAGCTCGCCATTGATGACTGCTCCTGTGGCATCAGAAGCTTACGATGCTAGTGGTGTAGTTAGCTTTGCATGTCGCCTCACAGTCATCCTTGGTGTTGAACCTGTTCTTCTTGCTTGAGAACGGAGAGTATCGCGAAGTCTTGCAGCTGTCGCTCTCGACGTCGTAGTAGAATTTTTTCACAAGTCCAAACCTATTCTTGGGACCAGGCTCTACGCACTTGCTCCCAGAGTTCTGGCCACAAGTGTTCCAGCACTCCCTTGCGGAGTCGAACCTGTTGGGGTAGCCACCGCAGCCGTAGTAGACGAACCTTTCACACCTTTTGTTCTTGTTGTTGTATCCAAATGCTACCTTAGGTGAACCATTTGGGCACTCC |
| >AAUF19644  GCGGTGGCTACCCCAACAGGTTTGACTCCGCAAGGGAGTGCTGGAACACTTGTGGCCGGGACTCTGGCAGCAAGTGCGTAGAGCCTGGTCCCAAGAATAGGTTTGGACTTGTGAAAAAATACTACTACGACCTCGAGAGCGACAGCTGCAAGACTTCGCGATACTCTCCGTTCTCGAGCAAGAAGAACAGGTTCAACACCAAGGATGACTGCGAGGCGACATGCAGAGCTAACTACACCTCTAGCATCGCAAGCTTCTGATGCCACAGGGGCAGTCATCAAGGGCGAGCTTCGAGACGAAGGGCTTCGCTCATCAGCGGTCATCGTGGCTGATGCGGCCTTGGAGAGCATGTTGAAAGACAATCGCATTCGAGCAGAATGCACCTCCAGACACTGGTTCCATTTCCTAATATGATGTGAGCATTGCA |
| >AAUF20767  GGCGGCGTTTCTACTACGACCAGGAGAAGGACCAGTGCCTGCCATTCCTCTACAGCCTGTGCCAGGGAAACGGCAACAGGTTCCACACCATCGCCATGTGCGAACGACAGTGCCGCCGAGGCTCCGGAGATCCAGAAAGACATCAAGAGTAGAAATGCACCACGAACCCATCAGTATCGTGTTCTCCTCGCAGCGCCAGGCATTGCGCGATCATTGTAATTTAGCCTCAA |
| >AAUF20805  CGCGACTTGAACACAAAAGAAGTGGCCAACGGTTGCTGTTTTGCTTCAGTTACCTCAGTGCCGCAGCTTGCAATATGAGACCACAAGCTTTCATAGGTGTTATCGTCTTTACTTTGGTACTCAGGCAAGCTGCAGGTATAAAGTGGTCGCGTTGTTTCCGACCGAAGGCTGTGGGGAACTGTCAAAATAAGGTTCCGGCCTGGTACTATGATTTTTGGAGCTTCCGTTGCAAGGGGTTCCTCTACAGCGGATGTGGCGGGAATTCGAACAGGTTTCCAACCGAAGAAGAATGTCAGAAATCCTGCTTACGCAAATCTAAGCGGAAGGAGGTTTGCAGCCTGAAGCCGAAAACTGGTAAATGCAAGGCCGCTATTCCATTATGGTACTACGATCCCGAATTGGATGAATGCCGTGGCCTCATTTACGGTGGCTGCAAAGGAAACGCCAATAGGTTTGAAACTTGTCTCAAGTGCATGAAACGATGCAGCGGAAATAACAACGCAAGAAAAATATGCAAGAAGCAAACTAAAAAATTTCTCGAAAAAAACAACCTGGGATCAAACCAGCATCAGAAAAAACCTTCGTGGCCCCAGCTGTCACTAAGGTTTCCATTCATAGGGAAATGAGGATGTATATACGAAGCGTGGTGTCAAGGAAAAAAAAAATTACGTCTGTG |
| >AAUF20964  CCAGGTTTCTTTTGACACCACCTCCTAGTTAGAATGCTCTTTAGGTTAGATTCCAAGCGCAGTGATTCTTCAAAGCATCGCCTTGACATGACGCAATAACAAGGCATCGAAAGCCAATCCTTTTTTACAGCCTTTTTATGCTCCCTCTGTTTGTCATGAATGAGAATCATACAGAGACATCTGTGCAAAGAATGTCATCATTATATGCAGGACTTCTCGCACTCTGCCTTCGTATAGAACGTGTTGCAGTTTACGCAGCAGCCGAATCCAATCTTGAACTCCTCACACTTCCTGGTCGTTGGATTGTAACGGTACCTGACCAGGACTGGGAAAATCAGGCATGTTCCTCCTGGTTTTGGTAGACTGCAATCTTTTTTATATGTGCATGGTGGCGGGCAAAAAATGGAGTCTCCATATGAGACGGTAGCGAGAAGAACCGCCACCGTAAATAGAGCTACTGCTGCATTCATGGTGCCTATAAGAATAGGTCCGGGCG |
| >AAUF22249  CGACAGTCATGCAAATATTAGTTTATTTCAATTGGTCGAAAGATACTGCAGAAGGTTGATTGCAGCGAAAGTTCCTTGGGTCACAGATCTAGGCATGGTCGGTTATTTGGGCCTTCTACCTGTGGGGTTTAGCAAGCGCTGGTATTTTACAGTTTGCCTGTGGCAAATGTTTTTCCGTTTTTGATAACTAATGTTCACAGTGCATGTTTTCATACAAGTTTGGCAGTCTTCAAAGTTGTTTTTATTTCCACCACAGCCGCCAAAGATAAAAACTTCACAGACACCAGACCAGCGGTTGTAAGACCAACGAGGAAAATATCCTCTGCAAAGCCCTGTCTTGCTGCCAAGAAAGCACAATCTCTTTTGTGCTGTACGTGCATGTGAGAGAGTCAAGCAGACAAGGAAGATAAGAAGGCATGCTTGTACTCTGGTCAGCGCATTCATATCTTCAGTTTTCGAGGGACAGAGCTTCCTCGTGATGCAACTCCTCACAGACACCAG |
| >AAUF24786  CGGTCGAGGTGGCCTCAGCACCGTCATCCGAGGAAGGCCGCGGCATAATCGACACGGCGCACAACCTGCGGGTGTTCAAGCCGCGGGTCAAGCACTGCATCAAGAACGTCATCAACTACCGGGACGCGCCGCCGGACAGGAGAGGGCGCTTCCACATACTGCTCGGCCGCATGGTGGAGTACGTCGACCAGATGACCGAGCAGAACTACAAAGCCAAGGTCAAGCTGCTGTCCAAGAGCGGGACCATGCTGCACGACAAGTTCAACGACGCTTTCACGAGGTGTGTCCAGGACCCGTGCTCGCAGCCCCCGGAGCCCGGCTGGTGCCTGCAGAACATGGTGATGTTCTACTACGACCCGGACAGGGCCTCCTGCAACGAGTTCACCTACACCGGCTGCGGGAGGAACTTCAACAACTTCCTCACCAAGCAGGAGTGCCGCAAGATCTGCGTAGAAGGTAGGACGTGATCGGGACATTCGGCACGCGGGTGGATGACTGGGAAGACCACGGCGCGCACGAACACAGCACGCAAACTGGCCCGCGG |
| >AAUF28154  CCTTCTTGACTGGAATAGCATTCGGAGAAAGGAGCCTGAAGATGGACCCTCGAACCTTCTTCTTTCTATTTGCTGTCCTGACTGTGGTGTCGGCCAAGTCGTTTTCACTCCCTCCAGAGTGCCTTGAAAAAGCCGACATCGGTCGGTGCAACAGCTTTGAACCTAAGTGGTTCTTTGATGCGGATGCAGGGCACTGCAAGCCATTTCAGTATGGCGGATGCGGGCAAAACAAAAACGTTTTTTCTAATTGCACTGCCTGCATGAGTCGATGCACT |
| >AAUF28791  GGTGCGTGCAAGTTGCCGGTGAAAGAAGGCAACTGCAATGAAAGGATTCCCCGCTACTTCTTCAACGTCACTTCCGGCGTTTGCGACGTGTTCTACTACACCGGCTGTGGTGGGAACGAGAACAGCTTCGCCTCCCATGAAGAGTGCATTGCCCAGTGTGAAGATCCCTGCCGCCTGCCAATGGATCCTGGCAGCTGCAACGACACGCAGGAGCGCTACTACTTCAACTCACAGACGGGATTGTGTGAGACCTTCGAGTACGGCGGCTGCGAAGGGAACAAGAACAACTTTGCAGACCTCGACGCGTGCAAGACGCTTTGCGAAGATGTTTGCTCGCAGCCCCAAGACCCGGGCCCATGCTACGCGTACTTCCGGCGCTTCTACTACAATAAGCAGGAGGATCGCTGCCTGCCTTTCATCTTCGGTGGCTGCATGGGCAACGGAAACAACTTTTACACCAGCACCCAGTGCAACGGACGCTGCCGATTGCATGTTGCTGAGGACGGCGCAGCCGGAAAGGACAGGGTGCATGTTGCTGAGGACGGCGTAGCCGGAAAGGACAGGGAAATGTGTCACCAGCCGGTGGACGAAGGCCACTGCGACACCGACAGCAGTGGCCCAGGGGCCACTACACCAGAGATCCGCTTCTACTACGACGTGCAGAAAGAACTGTGCGAGCGGTTCAACTACCAGGGATGTGGTGGAAACGACAACAACTTCCGTACCGTAGACGGATGCAACATGACCTGCTTTGGTGTGCGCATCAACTTGGCCAGGACGGCTGCGAGCTGCCCGGAGTCCATGCAGTGCAGCTGCGGTGATGAGCAGGAGGCCAAGTGCGGGTCATGCGAGTGCGGGAACGCAGCCCCGCGTAGCAACGGGGCCGCCCTCTACGCTCTAGTGTTCCTCACTGGTGCATCGGTGACAGCGTCGCTCTGCACTCTCTGAAGAAGCGACGAGGAGGACGGAAATATTTCTTTTTTGTTCCCAGATTCTCGATTAGCGCATGCGTGATCACAGGCGCACGCGGTGTGTAGTGAAATCTGCGCGCACTGGCAGTATCTGCCAAGGACGTCCACGAATGCAATTTGTGTCCCTGGCGATATGAACTTTCTGTCGCATTCCATCGGTGCGCCCAATCTCTAAAATATGCATTGTCTTTGTGGCAGTCAACGGAATTTGCTTACAAGCAGCGTCTTTTTCAGCAACACTGGATAACTGCAGCCAGGCAATCTTGCCCTCTGCACAAAATTTTCTAACGCAACAAATGCCTCTGGCGGCGTGGAGACTATCGGCAAGTTCAACCACGGCGCATCTGCCTCGACGCGCTCCTCCACTTAGCGTGAGCCT |
| >AAUF28986  TCACCGGCAACTTGCACGCACCATCAGACGGACAGTGACAGCTCTGGGCGCCGCTGCTCGAAATTCGCTTCACGCAAGTCACTGGGCAGGGCACTGCGATTTGGGCATCCACGTACTCCAGTGCATATTCAACAGTGCCGTCTGCAGCAGCACATTCGCAGGCGTAGCACGACGCAGACACCTCCCTACTCTGACAACCAGCCGGGCACAGCTCGGGAGCCGCGAGGCAGTCAGCTTTCTTGAGAAGGGGCTCTGTTTCGGACGCGAGGCAATTGCACGCAATACAGCTGTCTTTCTCTCGCCGCTGGCAGCAGTGAGCGCAGGCTAGGCCGCATGCTGCACGCTGGGGACACTGGGCTGATTCGGATTCCACAGGTGGAGCTCGAGCTTGCTCCAGTGGCTTCTCGCATCTTTCAACACATTCTTGGCGAGTAAGGAAGTGGTTTTCGTTGGCTCCACAACCGGTGAAAGTGAAGTTCAGGCAGCTGCGTTGCTCTCGGTCGTAGTAGAAGACTGTCTGCTTATTCGCACAGAAGCCTTCCTTTCGTTCCAGGAAGCATATTCCCGTGCCTGGTGGTATTTCGGCCAAGGTGGGATCTTCATCGGACGGTAGAGTGCATGCTTC |
| >AAUF32278  GTTTCATCTTCTAGCCGCGAAAAGTGGATAACGTCCACTCCACGTCATTCGAGAGCCACCGACTCATCCTTGGCCTTTGCAGTTTCTTCGTTGAAACCTCCGCATTGCTTTTCGCAATATCCTCTTGCGATGAAATAATTATTATCCAGCAATTTCTGACTTCCTCCGCAGAAGTTGTACTTCTCGCAGGTTTGTGTCGTTATGTTGTAATAATACCTTGGTCTCCATTTTCTAGTTTTCTCGCCTTCGCAAGGACTTGGTGGAGAATTAGCGCAGAATGGAGCGCCTTGCTTCTCATTGCATGTGGAGACACATTCGTAGCGAGATTCAAACAAGCCTTCTGTGCCGCAGCCGTTCCACCGCAAGTGTTGGCACATTCCGGACGTTGTATTG |
| >AAUF37396  AAAGTGGATAACGTCCATTGCATGTCATTCGAGTGCCACCGGTCCACCCTTGGCCTTTGCTGTTTCTTCGTTGAATCCTCCGCATTGCTTTTCGCAGTATCCTTTTGCGATGAAATAATTATTATCCAGCAAGTTCTGCCGTCCTCCGCAGAAGTTGTACTCCTCGCAGGTTTTTGTCGTTATGTTGTAGTAATACCTTTTTCTCCCTTTTTTAGCTTTCTCCTCCTCGCAAGGACTTGATGGAGGATCAGCGCAGAATGGAGCGCCTTGTTCTTTATTGCATGTGGCGACACATTGGTAGCGGGATGTAAACAAGCCTTCTGTGCCACAGCCGTTCCACCGCAAGTGTTCGCACATTTCGGACGTTTTATTGTAAAAGTACCTCAAGGTGATTTGGCGACAGTTTGGCCCGCTTACAATTTTTGGCATCACGCACTGCGTTTCGTCGCGCTCGGAGATAACCGAAGCCACGAGTGCGCATACGGTGAGGATGGAAG |
| >AAUF39948  GCGGCAGTGGAATTTTTAGAATGTTAGCCTTTTTTATTTTTGAGGATTTCCACAGAACACGTGCACCAAAATTACAACCATTGATTCATCAATGCCCATTGCCCGGTGGGTTTTATTCTTCAAGGTGAAATTCTTCAAATCCAGAGTGCTCCCTTTGTCGCTGCAGTGGCTGATGGCAAACCTTAGAACTGAACCCAAGGATAAAAGAAGGCGCTCGGGCGTGGCTGTGGTCGCGCGGTTCCTCTTAAGCACAGTCTTTCGCATTCTTCTCTGCTTCCAAAGTTATTGCCGTTCAGAGTACAACCTACTTCCCTTAAACATGAGTTACTCCAGGGGTCAAAATAAAAGACGTAAAAGACTCCATTGTAGCAATAGCTGGCAGGGCGTGGAGCAGTGCACACGGCTGGGTCTTTCAAGCTGCTGCTGTGATGCACGGCCATGCACACTGGCAGAAGAAAGTAGAAGCCTAAAACTTGGTAGATATT |
| >AAUF42040  CTTTAATGTGTCGAACCGGATCGCCGCTGCCGTCAGTGAGGCTGGCTCTGCTGCTGCCGCCCCTATCCGCTGGTCGTCTTGCGGGGATGCACGCATTCCGACTTGCACTTGGCCTCTGTGTCGAAGCGGTTGTCGTTGCCGTCGCATCCGCCGTACACGAACAGCTTGCACGCGTTGGTCTTGCTGTCGTAGTACCATTTGGTGGAGGCGTCCTCGCAGTTGCCGGGGTCCTTCTTCAGGTTGCAAGCTTTCTTGTGCGC |
| >AAUF43411  GTTCAAGTTTTTTATTTAAGCGCTAAGAACATTTTGGTTTAATATGCTTTGTACACATACTTAGTTCTTTATGAATGGAATGCTCAATATGAAAGGCCACTTGGATGTTTTGCGCTTTGGCTGAAGCCCTAAGTTGTATCTCTTGCGAAATGCTTCGGTCCTCTTCTTGCAGATTTTCCAAGCGTGGTTCTTCCGTGTGCAGGCTTTCTGGCATTCTTGTTCAGATGTAAACCTGTTCGAGTTCTCACCACATCCGCTGTAGAGGAACCCTTTGCAACTCAAGGTCCACAAATCGTAGTACCAAGAAGGAATCTTTGCTGTACAATTTCCGACAGCCTTTCGTCGCCAACAACGGGTCCATTTAAAGCTTTCAGCTTTTATTTGTTTTAGTGTTGCTTGGATGCTGCTCCAGCAGAAAGAATCAGGGCATCTTGGCAACGAGATGTATTTTTCCAACGCGCTATGCAAGAATGGTGGTAAACCTGATTACAGTTT |
| >AAUF44122  CCTTGCTCCCGAGGATAAGAGAAATGCGTAGCAGACAAATATTTTCACATTTCCTCTCTACTCTGTAGCACTGTTTGTCTTCCTTTCAATTGAGTTCCCTGGACAAAAACGTCTGTTTCTTATTATCCTGCTTTGCAAGTGCGCCGACATTCGCCTAAGGTTGGAAAGTTGTTTCCTGCGTAGCTGCAGCCCATATCCGCGTGGCACATGCCGGTCCAAGGATTAAAATAGTAAACTGCAAATGTCCTCCCGCTGCAAAGTGCATACGGTCTCGGAGCAGTGCAGACGGCAACACTTCGACAGGTAACATCGCATTCTAATTGGGTTTCAAAAGGAGCTCGGCCGCATGTCCATGTGCACTGCTTGGTATTACGGTTGAATGCGTAGCTGCGGTGAGGGCAATTACTTTTCAGCACCGGGAGGCTTGATCTGCATCGTGAATCGTGTGGGTTTGGCCGCCTGGCACTACAAAGTGCCAATAGAGCAAGCAACAGAACCGCCTTCCAGATGGC |
| >AAUF44563  CTCCTTTAGAAGGAGGTCGCTGCAGGGAGGGGGTCGAAGGTCGCTACTACTACAACATTGAAACAGGCCGCTGTGAGTCTACCCACTACGGATTCTGCGAAGGAGCCTGGGACAGCTTCCTCCGTCTTGAAGAATGCAAGACTCGCTGTGAAGACGTGTGCTCGCGGCCTGTGGACCCGGGTCCCTGTGATTTGAGTTATCCACGGTTGTGGCCA |
| >AAUF44843  GTGAAATGCATGCTAAATGGTTCTACGACGTAAAGAGCGGGCTCTGCCTGCCATTTGTGTACACCGGCTGTGGAGGCAATAAGAACCGCTTCAAGTCGGCCGAGATCTGCCGGAAGATCTGCTCTGGGGTCACAATGGACAGCATTGATGACGAAATCATCGATCCTGATGCGGACAGGAAATGGATGCCGCCTTCCCCTCCAGAGCCTAGTGACACAAGCGAAGAGTGGGACCCACGTGTGAAGCCACCAATGGCAGTCACACCACTTCCAGAGCCTAGTTCGTTGACACCGGCAACTGTGCCCCGCACGCCAGCGCCAGAGTGCCTGCCGTCAAACTGTGACGAACTGCAGTGCCCCTTGGGAAAGAATAAGACTGTGGACCACCGTGGGTGTGTCCAGTGTCGCTGCAGCAACCCTTGCGAGACGTTCTCGTGCCATGAGGAAGAGCTGTGCAGGATTGAAGCGTACCGAAGTGCCGACGGGAAACCCAACTACCGAC |
| >AAUF47392  GGAACACACTTCGCTCAGAAATGCGCCAACTTGCCGTTCTATCGCTCATACTCTTCACGGGTACGTGTGTTGAGTCCCAGACAGCGAAAAGCAAGGAAGTTTGCAAACTGAAAATGGATGGGGGAGACGAATCATGCAAAAACTCAACTAAGACAACTGAGATCCGCTGGCACTTCGACGGAACGTCTTGCAAAGCTTTCAGCTTCAACGGCTGCGGTGGAAACGAAAATAATTTCGACAGGATAGACGACTGCCAGAGGCTGTGCGAGGAGGAAACACACATTCACTTCGAATCACCGAAATACGTTTGTTTCAAAGCAATAATAGGCTATATTGCCAATAAGAATCTTACTGAAATTTCGATACAAAGCAAGAAAAATTAAAATGATGG |
| >AAUF49722  CGGATCCGGGAAGCCGCCCCTTCTTGTCATCGAAGTTGGCCTTCTTGTTGCTGAATGTGGTGAGAGGCGTTTCGTTCAGTGTTTTCGGACCGCATGTCTTTTCGCAGTCCTCCTTGTAGAGGTATCTGTTCTGGTTGCCACCGCAGCCTCCGTAGTAGAAGAGCTCGCACTTGCCAGAATCCGTATTGAACCACCACCTTGGAAGCTTAGCCTTGCAAAGTCCCTTTTCCGCTTTTGCAGTGCACTGTTTGTCAACATTTGTAGCCGCACAAGCGGCGCCGATGAAAGCAAGGAACAAGTAG |
| >AAUF50423  CCTTATGCACATGGCTAGACATAATTGATGCTATCAGTTTGTATATAATGGAAGGTGGCCGTTATTAGAATCGTCTTGCGTTGGGTGTACCCCTCGGTGTGAGGTTCGCACTTGCTTGTTTTTCCAGTTTTTGACAAATCCTCTTTATACTCGGCCAGGGTTTTCCTGAGCACGCCCGCATGCATTTCTCGCAGGTTCCAAATTTGTTCCTATTTCCCCCACAGCCGCCGTAAATAAAAACTCTACATCCACCTTTGGACCAGTTAAAATACCACATTGGAAAGTAGGCTCTGCAGACCCCAGTCTCTTTTGGAAGCCTGCAGACTTTGTTT |
| >AAUF50530  GTGTTCTATTTTCTGCCCCCGTCTCATGCTCAAGCGCGAGGGAGAATGCCCATGGACGTCTTGCATTTGCTTTCGCAGTCGGCGTAGCTGTAGAAGTTGTTCGCATTCCCTCCACATCCTCCGTAGATGAACTGCTCGCAGGACTGGGTCGTCGCGTTGTAGTAGNNNNNNNNNNNNNNNNNNAAGCAACGTCCTGCCTCTGCCGGATAAGTGCACTCCAAAGGCGTTGCCCACGGCCTGAATGGAGCTTGAAATGCTTCGCTGACATCTGCTGGATCCTGTTTCCTTGAGACCCAGCATGTGTTCTCGCACTCTTCCAGAGTCTCGAAATTGTTCTCGTTGCCCCGGCAGCCGCCGTAGATGAACTTCTCGCAGGTTTTTGACCACCGGTTGTAGTAGTAGCGGGGGTAGTGCGCCCCGCATGGCCCAGGATACTTTGGCTCGAAACAGAGGGGG |
| >AAUF54368  GAAGTTGCCACATGCATTCTGGCACTGCTCCGGGGATTCGAAGTTGTTCCGGCCACCGCTGCAGCCGCCGTACGTAAACTTCTCGCAAAATTGCCCGTTGTACCACCAACGAGAGATAAGAGCTCTACAAGGTCCTTCGACGGGGCCCTCCCTGCATACATCTTCGCACGTTTCGTTCTTCTTATCCCCACTGCATATTTCAGGACAATCAACTGCGTCCTCTCTGCTGGCTACACCTGCGCCTGGAAATAAATTCCTCGCCAATGGTCCCAAACTTGCATCCAAAGAGTTCGGCGTCACCGCTGCCGCGATTGCTCTGCCGCGACCAGGATCCACATCTCCTAGAGAAAGAAGAAGAGTAAACAGAAGGAACTTCATTTTCAGTCGCTTGTTTAGCCAGGAATTAGA |
| >AAUF54513  TAAAATCAACGAAGCTCTCAGAGTCTTTGTTGCGTTTGGCTTCTGGGCAGCCATTATTCCCGGCCACGCACTTCTCAGCGACGATATCTGCAATCGTCCAGGCGCAGTGCCCACCTGTCTGGACACCGCATTTGAAGTTTACTTTTATTCACCAGACACCGGAATGTGCCACAGCGATGTGAGCTGTACTCTCGAAGGCAACAACTTTCGCACGCTTCAAGAGTGCATGAGTGCTTGTGGAAGTAGTTCGCCGATGTCGACTGCACACACAGGGATGACGATAGCTGAGCCCTTGTACGCCATCTTTTATTGAAAATGAATTCCGGTTTGTGGTGTGCTTTTGACCTCGTTGGGCGTCGATGGAGCTCTTTCAGCGATATGAG |
| >AAUF54635  GTTCCTCTCGAACTCTTGTGTGAGCTTCTTACAGAGATGCATGTTATGACCTNNNNNNNNNNNNNTCATGCACTCCCAGCAGGTACCAAATCTGTTGTCGTTTCCTTTGCAGCCTCCATACACGAATCCCATACAGTACCCTCTGCTGTAATCGTAGTACCAGCGCGGTTTGTAAGCCTTGCAGATTCCTGTAGCAGGCTTTAGACGACAAACCGAACCAGGCCGACGTTTCGGTCGGCAGACTTCCAGACACCTCTTCTTCGTTTTGAAGTTGTTGGCGTTTCCCTTACAGCCACCGTAGTAGAAAAATTTACATGTTTGGGTAAAAGGATCAAAGTACCACCTTTGAATGCGAGCTCTGCACAGACCAACCTGCGGCTTCTTGTTGCAGTAGTCTGGAGCTCGCTAGGTCACCACTGAAGCAACGAATGCGGTGGCCAAAAGTAGGCTGAAAGAGGAGCGATTATACATGGTGGAAAATCTCTTCTTAATGGGGCACGGAACGTG |
| >AAUF54652  CCGCCCTCCGGGGGAGAGGCAGGCTCGTTCACAGTCACGCCTTGACTCGAACCTGTTGGCGTTGCCCTCACAGCCTCCATAGTAGAACTGCTCGCAACGACCAGCGGCCATGTTGTAGTACCAGTGCACAATGGCTGCCCGACACGGACCCTCCTGCTTGGGCAAGGCGCAAACGTCTCGGGGCACGGTCACCCTGGTGGAGTTGCACATGCGCTCGCATTGCTCACGGGTGTTGAAGCGGTTTCGGTTGCCCTGGCAACCTGTGTAGACAAATTCCTTGCAGAGCCCCTCCACATAGTCGTAGTACCACTTGCGGTCTTCCGACAAGCACTGGCCTACCTCCTTGGACAGGATGCAGATATCCTTTGCACCGAGAGAGACACATCGTTCGGAGCATTCTTCTTCAGTGGCAAAGTTGTTGGCATTACCCTGACAGCCACCATAAGTGAACTGCTTGCACCGATTTTCCTGCCGGTCATAGTAAAACCGCGTATATGAGCCCCTACACGATCCAGGTGCAACCGACTGTTCACATGGGTCCAGTGTCTCCTGATGGAGGCAGGTCTGTTCACAGAGGTCTTTGCTGACAAAGCGGTTGTTGTTTCCGAGGCACCCTCCATACATGAACGACTCGCAGGTACGAGTCGATGCATCGAAGTACCAGTGCTCGTACTGGCCATCACAAGGGCCGACCACCTTGGGGAGCTGGCAGGCATCAGGTCCTTCTGGTTTGACACAAGTGTTCTCACACTCCTCTTCAGAGTTGAACCGGTTTTCGTTGCCCTCACAGCCG |
| >AAUF62775  CATCACCGTAGCGACACACAAAGACCAAGATGCCCTGGTTGTTGCTTACTACCGCCGTGCTGGAAGTTATCTGTTTCGCGATGACGGTCGACTCCGCTATAATCCAGAAGCGTCTGATCGGGACCGCCTGCAACACAACGTCACAGTGCCAAAATCTACTGCCCAAGAGCCAGTGCATGTACTCCAGTTGTTTCTGCATCCGGGGCCACTATTACAACTGGAAGAACAACAGTTGCATTCCGCTGATGCTCCTCGGTGAAAACTGCAGCCACGACAACGAGTGCAGCTCGCACCTCATGTGCAGCGCTCAGACATGTGCCTGCATGCCTGGGTACAGGCAGGTCAAAGACTGGTGCGAGCCGATGGAGATGGAGGACGACACAGAACGGTTCATGGAGGACGACACAGAAC |
| >AAUF63715  ATAGGCACGGGCCCCAGGGTGGCGCAGACGGCGAAGCACTCGCGCATCGTCTCGAAGCTGTTGCCGTTGCCGTGGCAGCCTCCATAGAGGAACGACTTGCATTGGTTTGTCTTAGCTTCGTAGTAGAAGCGTGGGATGAAAGCCACACACGGGCCGGGGTACGGTGGGAGAAAACACAGATCAGGCTTTTTCTTCGCAGGCTCACTGAGCAACACTGTGGCTTCTTGTTTTGGCTGGAAGATAGCAAATGCGGAGCTGACAACAGCGAGGAGAATGTAAGCCTTCATG |

| >AAFM346  TCCGGTGCCAGGGGCAGAACTATAATCCAAAATTTGCGTGCACCACTTTCTCGCTTTCGCAAGCACTAGACAGTTTAATGAACTGGCTGAAGTACTTTTCGAGAAAGAAGATTGTCGCGTATTTTGACAACCTTAATTAAGCTTGGCGGTTCCGGTTATTTCGGCATGGCCGTCGGGAAGTATTTCTTGTTGGCTTTTTCAGTCAACTGATGGCAATACTCTCTGTCTTTCTTAGTAAATTTTTTAGCACAAATGTTCATGCATTCATAGCAGCTGTCAAATCTGTTCCGAGTTCCTCGACATCCGCCGTAGATAAATGCTTCGCACAAGCCGCTTTTTGGGTTATATGACCACTTGAGGAAGAAAGCTTTGCAGCGTCCAGTTTTCCGCCCAGGTTCTCTGCAGATTGGGTTTGGGGGACGATACCCGCTTACGAAGGTCGCAGCGAGCAGCAGCACGCTGAGTATGGCTATGAGCTTCATAGTCGATTTCGCTCCTTTTTTCGTAGAAATCGGGAACGCAGCTGAAAACGGTCAATCTGTGTGGCTTTATATATCTATGGCGTTGTTCCGACAGATATTATCTGGTTGTTTTTTAGTGTGCAGTTTG |
| --- |
| >AAFM347  AGTCATCCTTTATTTTCCGGTGACAGGGGCAGAACTAGAATCCAAAATATGCGTGCAGCACTTTCTGGCTTTCGCAAGCATTAGACAGTTTAATGAACTCGCTGAAGTACTTTTCGAGAAAGGAGATTGTCGCGTATTTTGACAGCCTTAATTAAGCTTGGCGGTTCCGGTTATTTCGGCATGGCCGTCGGGAAGTATTTCTTGTTGGCTTTTTCAGTCAACTGTTGGCAATACTCTCTGTCTTTCTTAGTAAATTTTTTAGCACAAGTGTTCATGCATGCATTGCAGCTGTCAAATCTGTTGCGAGTTCCTCGACATCCGCCGTAGATAAATGCTTCGCACAAGCCGCTTTTTGTGTTATATGACCAGTTGAGGAAGAAAGCTTTGCAGCGTCCAGTTTTCCGCCCAGGTTCTCTGCATATTGGGTTTGGTGGACGATACCCGCTTACGAAGGTCGCAGCGAGCAGCAGCACGCTGAGTATGGCTATGAGCTTCATAGTCGATTTCGCTCCTTTTTTCGTAGAA |
| >AAFM456  GACTCCATTGTAGCAATAGCTGACTGCGCGTGTCTAACATGAATATCTACCAAGTTTTAGGCTTCTACTTTCTTCTGCCAGTGTGCATGGCCGTGCATCACAGCAGCAGCTTGAAAGACCCAGCCGTGTGCACTGCTCCACGCCCTGCCAGCTATTGCTACAATGGAGTCTTTTACGTCTTTTATTTTGACCCCTGGAGTAACTCATGTTTAAGGGAAGTAGGTTGTACTCTGAACGGCAATAACTTTGGAAGCAGAGAAGAATGCGAAAGACTGTGCTTAAGAGGAACCGCGCGACCACAGCCACGCCCGAGCGCCTTCTTTTATCCTTGGGTTCAGTTCTAAGGTTTGCCATCAGCCACTGCAGCGACAAAGGGAGCACTCTGGATTTGAAGAATTTCACCTTGAAGAATAAAACCCACCGGGCAATGGGCATTGATGAATCAATGGTTGTAATTTTGGTGCACGTGTTCTGTGGAAATCCTCAAAAATAAAAAAGGCTAACATTCTAAAAATTCCACTGACGCAGATATCTTTTAAATCGAGTCAAAATGCACCAATCTGGGTCGAGAACAGCGGGCTCTGCCATGGTTGCAGTTTAAATGTAAAGGAGAGAAACTTACCACTTCATTTTAGCCATATATAGCTGCTCAAAATTTGCCTGACAAAAAATTTTAGTAACATTATTTAGAAAGTCTTTAGACAAGTGTTAATAGAATACATTGACGTTGAC |
| >AAFM457  TCTACCAAGTTTTAGGCTTCTTCTTTCTTCTACCAGTCTGCATGAGCCAGCATCCCGAAAGCGGTGCGAAAAACCCTGCCGTGTGCAGTGTTCCACGCCCTGCTAGCTACTGCTCTGGGAGTGATGTCTTTCCAGTCTATTTTTTTGACACCTGGAGTCACACATGTTTAAGAGAAGAAGTAGGTTGTACTCTGCTGGGCAATAACTTTAGAACCAGAGAAGAATGCGAAGCACTCTGCTTAGGAGCAAACCCGCTACCACAGCCGCAGCCGATGACCTATCTTAACGGTTGGTTTTTCTACTAAAGTACATCATGAGCCACTGCAGCGCGTGAGGGAGCACTCTGGATTTGGAGAATTTCACCTTGATGAATCAACCGTACCGGGCGATGGGCACTGATCCAGCAATGGTTGTAATTTCGGCGCAAGTCTTCTGCAGAAACCCTCTAAAATAAAAAATGCGCATATTCTAAAC |
| >AAFM638  TCTACCAAGCTTTAGGCTTCTTTCTTCTACCAATGTGCATGGCCATGCATAGCAGCATAAGCCCGAAAGACCCTGCCGTGTGCTATGCTCCACGCCCTGCCAGCTACTGCGATAGACATGAAAACGTCTTTCCCGTCTTTTTTTTTTATCCTGGGAGTCAATCATGTTTTCAAGAAGTTGGTTGTACTCTGGTGGGTAATAACTTTGCCACCCTAGAAGAATGCCAAAGAGTCTGCTTAAGAGGAAGTGCGCAACCACCGGCACAACCGATGGCCTTGTATTTTCCTTTGGTTCACTTCTAAAGTTTGTCATCAACCACTGCAGCGACTGAGGGAGAAGTTTGGATTTGAAGAATTTCGCCTTGAAGAATAAACTTCACTGGGCAATGGGTACTGATG |
| >AAFM640  TGCAACATGAATATCTACCAAGCTTTAGGCTTCTTCTTTCTTCTACCAATGTGCTTGGCCATGCATAGCAGCATAAGCCCGAAAGACCCTGCCGTGTGCTATGCTCCACGCCCTGCCAGCTACTGCGATAGACCTGAAAACGTCTTTCCCGTCTTTTTTTATTATCCTGGGAGTCAATCATGTTTTCAAGATGTTGGTTGTACTCTGATGGGTAATAACTTTGCAAGCCTAGAAGAATGCGAAAGAGTCTGCTTAAGAGGAAGAGCGCAACCACCGGCACAACCGATGGCGTTGCATTTTCCTTTGGTTCACTTCTAAAGTTTGTCATCAACCACTGCAGCGACTGAGGGAGAAGTTTGGATTTGAAGAATTTCGCCTTGAAGATTAAACCCCACTGGGCAATGGGTACTGATG |
| >AAFM641  GCTCCACGTCCTGCCGGACTACGCCTGTGCGACATGAATCTCTACCAAGTTTTAGGCTTTTTATTTCTGCTACCAGTGTGCATGGGCCAGCATCGTAGCAGCGATGAGAAAAACCCTGCCGTGTGCTATGCTCCACGTCCTGCCAGCTATTGCTATGAAGGGGGAGTCTTTCCGGTCTTTTATTTTGACCCTGGGAGTAATTCATGTTTAATGGAAGAACTAGGTTGTACTCTGTACGGCAATAACTTTAAAAGCAAAGAAGATTGCGAAGCACTCTGCTTAAGAGAACACGCGCAGCCACAGCCAAACGTGATGAACTGGCATAATTTTTGGTATTTTTAGTAAAGTTTATTATGAGCCAATGCAGCGATTGAGGGAGCACTCTGGATTTAAAGAACTTCACCTTGAAGAATAAACCCCACCGGGCAATGGGCACTGATGAAGCAATGGTTGCAATTTCGGCGCATGCCTTGTGCGGAAATTCTCTAAAATAAAAAGGCTCATATTCTAAACATCCCAAAA |
| >AAFM1376  TTACAACAGCCGTTCTCTCATGCCTTGCAGGCTTTGTGTTCGCCCAATGCATGTGCTGAATGAACACGGCAAAAGAGAGAGCGAAGTGCCCATTTCATTTGTCCAGCAGGCTACGTAAGCTTGCAGGCAATCTCGCACTCTTCCTTCGACTCGTAGTTGTTGTCGTTTCCACCGCAGCCACTGTAGAGGAACGTCTCGCACTCTCCCGAGGTCACGTTGAAGAACC |
| >AAFM1377  GTGTTCGCCCAATGCATGTGCTGAATGAACACGGCAAAAAAGAGAGCGAAGTGCCCATTTCATTTGTCCAGCGGGCTACGTAAGCTGGCAGGCAATCTCGCACTCTTCCTTCGACTCATAGTTGTTGTCGTTTCCACCGCAGCCACTGTAGAGGAACGTCTCGCACTGACCCGAGNNNNNNNNNNNNNNNNNNNNNNNNGTGGTCTAAGAAGCCCTTGCAATAGCCGCGTTGCGGTTTTGGCGCGCAACCAGTCTCGAAGTCAGCTCCCTCGAAGTCACTTGACCTCTCTGGCTCGCCGCATGTTTTTTCGCATTCCTCGA |
| >AAFM1626  ATCTTCAGGCTCCTTTCTCCGAGACTTTGCAATCCTTCTTGACTGGAATAGCATTCGGAGAAAGGAGCCTGAAGATGGACCCTCGAACCTTCTTCTTTCTATTTGCTGTCCTGACTGTGGTGTCGGCCAAGTCGTTTTCACTCCCTCCAGAGTGCCTTGAAAAAGCCGACATCGGTCGGTGCAACAGCTTTGAACCTAAGTGGTTCTTTGATGCGGATGCAGGGCACTGCAAGCCATTTCAGTATGGCGGATGCGGGCAAAACAAAAACGTTTTTTCTAATTGCACTGCCTGCATGAGTCGATGCACTGCCCATGATGATCCCAATAGAGCATGCGAATCATATCTTGAAGTAATGTATGGCCAGCAAGGATGAAGGCAGCAAGCACCACGGCGGGGATTTTCAGCATATCTTGGTTTACACATGTGAAGATTTGGTCGGACATTAGGAGCAGTGAATTTTACAACTGATTCACTCTGTGGACCAAGCAATAAACTAAGGTTTTTCGCATTTCCGTGAGCATCCTTCTGAAATCCAATCCTACTGTACACCCCTCTTTCTTTAAATCATAGTCTTTAAGCTGCCAATGCAAGAGGAAT |
| >AAFM2032  CTTTTTTTCAGTTGCTAGCAAATTTTATTGATCCTTTAATTAAAACATACATATTTATTATCCAAATACAGCCAGGATTTACCTCCCGTGTAATTACTGAAATATGTAAACTACTTTCACGTCTAGCTTTGCTTCCAAAATGGTATTATTCCGGGGAGCAATTTCTTTTATAAATGATTTCGATAGATTACGGCATCCATATCCGCAATGAGGAAATAATTTTGTGGAAACTGCGTGCCCATCGACAAGTGCAGGTGTCAATAAGTGAACTCTTTCCCGTTTACTCTGTGCCTGGGCTTGCACTCTCTGATGCATTTCTGCTGGGAACGGAACAAATTTCCCTTCGGCCAAACGTTTTTTGCCACAATGTTGAGCACAGTTTCGTAGCAGAAGTCGTCTTCAGAGTCATAATAGTACAACGGTTTCTTTTGTTTGTTATGTTGCAATGTTGGTGTATCATTCTCCAAGCAGACCGAGCTCTGATTGCATGCTTTCCAACACTCCTCCCGTGTTTCAAACTCGTTGCCGTTTTTCTGAAAGCAGCCTTCACGTACATCGTAATCCACGCATTTTCCTGTCTTTTTATTGTAAGCGTAGCGCTCTATCAATTCTCCGCTGCACCCATTCCTCTTTGGCTTGATTTTCATGCTGCATCTTGGATCTTGAGGGAGGGCTCTAGATAACACGTCGTCTCGTTTCTCCGCTTTCGGCGCAGTTGCGTAGCAGTCCAGAGTCAAGCCATACAAGCACAGCAGTTGAAGAAAAATACTCAAGATCATTGCTTTTGCTCCGTTTGCAAAAAAAAAAAAGTGGTAAGTATACGCTGTTCCTGGCC |
| >AAFM2165  TGAAAATGAAGTTCCTTCTGTTCACTCTTCTTCTTTCTCTAGGAGGTGCGGCCTTCGAAGACGAAGCAGAAAAATGGGCGAAAATATGCAGTCAAGGTCTGAACAACACAGCATGCGCAGATATATGCAGGGAGGACCCAGTCGAAGGACCTTGTAGAGCTCATATCCCTCGTTGGTGGTACAACGGTCAATGGTGCGAAGTATTTATCTACGGCGGCTGCAACGGTAACCGAAACAACTTCGAATCCTGGGATCAATGCCGGAATGCATGTGGCAGGTTCAATTTCACACAGCTGATGTTATTGGGCGATTCATCAGAAGAGAAGGCACAAGATTGATACTACGTACAATCATCTTCCGATGATACGAAATAGAAGGCTCTCGTGTAACAACACAATTGGGAATGCATACTAACAAATAAATATTGGCGGAGCTGTTCACTTCAGTTAACGTGTATTTCGCACACTGCACGTGAATTTCCA |
| >AAFM2166  GTAGGACCTTGTAGGGCCCATGGCTGGACTCCTAATTCTTGGCTAAACGAGCGACCGAATGAAGTTCCTTCTGTTTACTCTTCTTCTTTCTCTAGGAGGTGCGGCCTTCGCAGACGAAGCAGAAAAATGGGCGGAAATATGCAGTCAAGGTCTGGACAACAAAACGTGCGCAGAGATCTGCAGGGATGACCCGGTCGTAGGACCTTGTAGGGCCCATATCTCTCGTTGGTGGTATAACGGAGAATGGTGCGAAGAGTTTTTCTACGGCGGCTGCAAAGGCAACCTAAACAACTTCGAATCCTTGGATCAATGCCAGAAGGCATGTGGCAGGTTCAATTTCACACGGCTGATGTTATTGGGCGATTCA |
| >AAFM2649  CACATATGAAGGTTAGAAGGGTGGGTGCTGATCTTGCTTATTCCTCTTATTCTGGGTCGCAAGCTGCGTAGCAGTCGGCTGCTGACTTGAAGTTGTTACAATTCCCACCATTCTGGTGCCAGGCGAACATCTCGCACAGTCCAGTTTCACTGTTATAGTACCAGCGCTCTTCTCGTATGTAGTTAGGAACGCCCGCCACTTTAGGGCAGCTGCATGTTTCAGGGTACCGACATTGATCGCTTGGTCCTGTTCCGCAGTCTTGACTGGTGCCTTCCGATAACAGCAGTGTCCCAGACACTAGAAAGAAGAGAGCCACGAAAATTAGCTGGTTCATCATTGTAGCTAGATGAGGATGACAGC |
| >AAFM2650  CTTAAATTGCAAACACTTTTATTTCAACGGAAAAAGAAGCATCGGTCACCCCATATGAAGAGTAGAAGGATAGGTGCTGATCTTGCTTATTCCTCTTCTGGGACGCAAGCTGCGTAGCAGTCGTCTTCTGACTCGAAGTTGTTACAATTCCCACCATTCTGGTGCCAGGCGAACATCTCGCACAGTCCAGTTTGATTGTTATAGTAATAGCGCTCTAGTCGTATATAGTTAGGAGGCCCCGCCACTTTAGGGCAGCTGCATGCTTCAGGGTACCGACATTGATCGCTTGGTCCTGTCCCGCAGTCTTGACTGGCGCCTTCAGATAAAAGCAGTGTTCCAGACACTAGAAAGAAGAGAGCCACAAAAAATAGCTGGTGCACGCCCTCCACGCATGTTCTGATGAAGTACCAGAACATGC |
| >AAFM2811  TTTTTGTCCATCTAGCCACCGAAAGCTGAGTTTATTTCAAGTGCGCGAACTTTTCCTGAAGTGCCGAGAGAGAAATGTGTTTCCAGTACTCAGCACACTCTAGATTACGCTGATTACTTCGGTCTTCTTGCATAGTTTGGCTTGTAAGGGCCGGTGTTTGTACCAATTGGCTTCTCCAGCTTCCTGCAAAACTTCTTCGCTTTCCGCCAGCCCATTCCACCGCATTTCTTCATACACTCGTGGCAGTGTTGAAATTTATTCTCATTTCCTCCACAGCCACCGTAGGTAAACAAAGTGCAGTATCCTCTGTAAGCGTCATACCACCACATCGGTATCGATGCTCTGCAGGGTCCAACTTCTTTTCGCTGGGTGCAGACTTTTTTTCTGTTAAACCAGGCATGTGAAAAAGCTACGCCGACGAGAAGCAGAAGCAGCCACGCTGGTAATCTGGTCATTGCGGTCATTTTTTGAAGCTTTGCTCAGAAGGCGCCTTTGATGATATGCGAGTGGTCCAATTCACTCCTTCCAGCCTTTTATTTGCAAATTTTCTCGC |
| >AAFM2888  CCCCACATTATTGCAAGCACCAACCAAAGGAAGGACAATGCGGGCACGAGCGGCCTACAATGGAAAGATGGTACTTTGATGAACGATACGGCTACTGCGGTCCCTTCTTGTGGGGCGGATGCAGCGAAGATAAGAACAACTTTCCCAACTGTACATCCTGCATGACTACTTGTTCCACTCACCTAGATCCCGAAGGCGCTTGTCGCAATATTATCAGCGCCCCGTGATCAAGCAAGAACACTTCAGAAAGAAAATTTACAGTCCTGCTCTTGAACATTGCGGCACTGTGTGTTTTTGCCATATAAAGCAAAATAAAAAGACAAAA |
| >AAFM5058  CCGCCAACAACTATTTATTGTGCTGAAGAGGCTTTCTTATGCACATGGCTAGACATAATTGATGCTATCAGTTTGTATATAATGGAAGGTGGTCGTTATTAGAATCGTCTTGCGTTGGGTGTACCCCTCGGTCTGAGGTTCGCACTTGCTTGTTTTTCCAGTTTTTGACAAATCCTCTTTATACTCCNNNNNNNNNNNNNNNNNNNNNNNNNNATTTCCCCCACAGCCGCCGTAAATAAAAACTCTACATCCACCTTTGGACCAGTTAAAATACCACATTGGAAAGTAGGCTCTGCAGACCCCAGTCTCTTTTGGAAGCCTGCAGACTTTGTTTCGTAGACGTCCTTGTGAGACAGAGAGGCAGAAGAGGAGGAGAAGAAGGCACGACTGTAGTCTCGTAAATGATTTCATGTTTCAGATTTGGCGCAACGCTGGGCTTTCTTGCGATACCGC |
| >AAFM5059  TTTCAAGCCACGTGCGCGCCGATACTTGTGCCAACCGAGCTCTCCACTTGCGACGCTCCCAGAATAGCTGCGCATTCAGTCCCATAACAGGGAATTCACCTATAGACTGCCGCAAGTAACAGGCGGCGTTGAACTGTCAATTTTTAGCGGTATCGCAAGAAAGCCCAGGGCAGCGCCAAATCTGAAAGATGAAATCAATTCCGAGATTGCAGCTGTACCTTCTTATCCTCCTCTTCTGCCTCTCTGTCTCCCAAGGACGTGCCCGAAACAAAGTCTGCAGGCTTCAAAAAGAGTCTGGAGTGTGCAGAGCCTACTTTCCAAAGTGGTATTTTAACCAGTTCAAAGGAGAATGTAGGGTTTTTATTTACGGCGGCTGTGGGGGAAATGGCAACAAATTTGACACCTGCGAGAAATGCATGCGGGTCTGCACAGGAAAAC |
| >AAFM5066  GGAAAATATGACTTGGATTGCGCTTGCAGCAACCGTATTTCTGGTGTCGTTAACAGGATGCGTGTGTTTCGAGGAAGACAAAGCCTACATGCAGTGCACGGAATATCCCGATCGGGGACCCTGCAAAGGCACAGTGTACCGCTACTACTTCAACTTCATGCGAGGGACTTGCCGCCTCTTCGTCTACGGCGGATGCGAAGGCAACGACAACAACTTCAAAAGCCGCAAGCAGTGCCTGCGACGATGCGCAAGCGTTATTACGTCGAGAATCTGCAAGCTGCCCCCTAATCGTGGCTACGGCCCTTCGCGCGTGTGGCATTACTACTTCGACTCGAAGAAGCAGTCGTGCCGCCCGTTCGTCTTCCTGGGCTTCGGAGGGAACCGCAACAACTTCATATCCAGCGACGAGTGCCGCATGCAGTGCCTGGGCAAGGAGGCCCACGAGAGGGGGGAAGACTGATGCGA |
| >AAFM5132  CCGCAAGAAAGCCCAGTTCTTAGCAAGGGTTGTACAAAGTTAGCGCGATGATCAGAATGCAAGCATACGTCTTTCTGCTTATCACCTGCCTCGTCCTCTCACATGTGGCTGCTAATAGACCCCAACGCTGTATGCAAAGAGGAGGAGTAAAAGGAAACTGCAGAGCTTCTATTCTTAGCTGGTCATACGACTCGAAAAGGGACAAATGTTACCCATTTTTATACGGTGGATGTGAAGGAAATCAGAACAACTTCCCAAGCTGTAAAACGTGCATGGAAAAGTGCAATCGAGGCAACAGACGGGCGACGCAACGATTTTGTAAAAGAGCGAACAAAGAAGCCAAGAAGCAATTCAAGCCACAGGGTTCTCCAAATTGAAGACGAGGCCGTCAACCGGAACTACCAAGCTTTACCTCTTCAGGAAAACTGGTTCGGTCAGTTGAATAAACCACCCTGTGTTATACCACATGTAAAGCGCTGAAAGTGAATAAAAGTATATCGGCAATCAGGCCGCTCAGTGACAGCACTACGGCGGTGTGGAACCAACG |
| >AAFM5225  GGCGTGTTCGCACTTTCACTGGTTTCCTGTCAACTGTAATAACATGAAAAAGGCTGTGCTTCTTTGCTTCCTGGCTGGCGCTGTTTTCGCAGCAGCAACGGCGGAGGTGCAAGTCCGAAATGAGCACCGCGGCGTGAACTTTGACTTGGGCTGCAGGCCTCCCCCTGAGAGCGGAATGTGCAGGGCAGACTTTGAGCGCTGGTACTTCGACGTGGCCGACGGTGTCTGCAAGCCCTTCGTCTACGGTGGATGCGGTGGCAATGCCAACCGGTACAACACCGAGTGGGAATGCCAGAAGGCCTGCATGCGCGGATCTGGCTGATGTACTTCGGAAGCAACTTCGAACCTATGGAACAAAAACCCAAGGCAAGCGTTTAAGCAAAAATCAGAGCACCATCCAAATGCGATTGAAACAAACATGGTGCAACCATTCAATGTTGTTTATTTGTGTGCGTG |
| >AAFM5747  AATGGTACAACAGCAAGTGCAGCGCCCATCGGATTCGCTGCGCAANNNNNNNNNNNGCGGCTTCTAAAAAAAAAAAAAGATGATGGTGCTGTGCGAGGATTTGCGAAAGACGGAACTTCGGCACGTGGGCTTCGCACGACCAGCCTTCCCGGAAATGCCCCGCATTAAGAGAAGCTGCCGCAGGTGCAGAGACAGCGGATGCCACGGGATGCCTGCCTCTACCGGCAGCGCTGCTGACGGGGCGAGCAGTGGGGGCGCTGCTGCTTGCGGCCGCCTCGTGCCAGCCCTGGTGGCGGCCGCCGCGTTACTGGGCTGCGCCCTGGGGGCGTCGGCCGCCAGCGTCACTGTGGAGGTGGCCTCGGCACCGTCATCCGAGGAAGGCCGCGGCATAATCGACACGGCGCACAACCTGCGGGTGTTCAAGCCGCGGGCCAAGCACTGCATCAAGAACGTCATCAACTACCGGGACGCGCCGCCGGACAGGAGAGGGCGCTTCCACATACTGCTCGGCCGCATGGTGGAGTACGTCGACCAGATGACCGAGCAGAACTACAAAGCCAAGGTCAAGCTGCTGTCCAAGAGCGGGACCATGCTGCACGACAAGTTCAACGACGCGTTCACGAGGTGTGTCCAGGACCCGTGCTCGCAGCCCCCGGAGCCCGGCTGGTGCCTGCAGAACATGGTGATGTTCTACTACGACCCGGACAGGGCCTCCTGCAACGAGTTCACCTACACCGGCTGCGGGAGGAACTTCAACAACTTCCTCAC |
| >AAFM5870  CTTCACAGCGTAATTATGCAGTAAGATGCCCGATGAAGGACCTTGCAGAGCCTTGATTCCTGCTTGGTTTTTTAATGGAATATCATGCCAGAAATTCACATACGGAGGCTGCGGGGACGACTACAATAACTTCGATTCCGAAGAAGAATGCATGGAAGCATGTGGCAATTACACGTACCCACGATTAAACATGACCTGGGAAAGCAATTACCTTGGTAACCACAACAACCGCACACAGTAAATTAGAGGTGACTTGGATGAGCAGCAGCTGTTAATCTGTGCAAAA |
| >AAFM5871  TTTCACTACTCAAACCAAATTTATTTTAGGATACGTTTTGCACAGATTAACAGCTGCTGCTCATCCAAGTCACCTCTAATTTACTGTGTGCGGTTGTTGTGGTTATAAGGGTAATCGTTTTCCCAGGTCATGTTTAATCGTGGGTACGTGTAATTGCCACATGCTTTCATGCATTCCTCTTTGGTATCGAAGTTATTTAAGTCGTCCCCACAGCCTCCGTAATGGAATTTCTGGCATGATTGTCCATTAAAGTACCAAGCAGGAATCAGGGCTCTGCAAGGACTGTCCTCGGGCACCGTTCTGCATAATTCTTTGCATTCGTCTTGTCTGTCAGGATTAAAACATACTGTCGCACCAGGGCTTTCATTGTAGAAAAAGGCCGCACACGCCGTGAAGAG |
| >AAFM6352  GCTCCTCACGATTTTTGCTCTCTTTTGCCTGTTGGGAACCACGCTGTCAGCACGCGCCCAAGAAGACCGTTGCAAACGGCGTCCAAAAAGTGCTACGGGCATGTGCCATAATGGAAAAGTGCCTAAACTGCGCTTTACATATAACCCTAGCACTGGAAAGTGCGACCATTTCTATGATTTAAGATGCAATGGACAAATTCTCAACAGTTTTGAGAATTTTACGGAATGTATGACAGCGTGCAACCCCGATTCAAAATGCCTGGCGACTCCTGATAAGCCTTTCAAGTGGATTCGATTGACAACCTCTTTCGTGTTTGACATAAACTCAATGAAGTGTAAAAAGGAGAAATCGCTCCGACGACCGGGCATTGGCCCTAAAATTAACAGGTTCTTAGAAGAGGACGAGTGCAAAAAAACATGCGAGCCGCATCTTATTAAAATCATTAAGAGTACAGGTTAAGCAACCGACAATAAAGTCAGTTCCAAGGCAAAAA |
| >AAFM6363  TTTTTTTTTTGCTTTGTGTGCAGCAATATTTTATTTTTCAATAAATACGCTGCATAAACAGTTAACTGCTGCTTAGACGTCATCGAAAGACCCTGTGATGTGACGTAACCAATGAGATAAGTTCTTTAACTCTCCGGCATGTCTCCCATGTTTCAGTCTGAAGAACCAAATACTTCGAGCATACACTAGTGCTTTGCGCAGTGCTTGATGCAGTAGTTGCGGGAGGGGAAACTATTTGCGCTCCCTCCGCAGCCCCAGTACCAAAAAGGTACGCACGTGCCGGTGCGAGGTTTGTAGTAGAAACGTATTGAGACGAAACCCCATCGACAAGGGTACCC |
| >AAFM6364  TTTTTTTGTGTGCAGCAAGATTTTATTTTTCAATAAATCCGCTGCTTAAACATCTAACTGCTGCTTAGACGTGATCGAAATACCCTTTGATGCGACGTAACCAATGAGATAAGTTCTTTAACTCTCCGGCATGTCTCCCATGTTTCAGTGGGAAGAATCAAATACTTCGAGCAGACACTAGTGCTTTGCGCAGTGCTTGATGCAGTAGTTGCGGGAGGGGAAACTATTTGCGCTCCCTCCGCAGCCCCAGTACCAAAAAGGTATGCACGTGCCGGTGCGAGGTTTGTAGTAGAAACGTATTGAGACGAA |
| >AAFM7456  GTAGACGGCCAAAGGAAGAAAGGTCCGCGTTCGCGACACTGCGGAAAAGGACCCGAGGACGAAAAATGTCAGAGCCTTATAGATAATAAACAAGTGTGTGCCTGCCCGAAGGAGGAAGGCAGACCCGGGTTCATTCGAGACCCGCGCTGGTTCTTTGACAAAAAGTCTGGACAGTGCCAGATGTTCTCGTGGTCCACTGATGGCGGTAACTGCAACAACTT |
| >AAFM7525  GCTGCGGGAATTTGAACTTGCCACATGCCTTCTGGCATTGCTCCGGGGATTCGAAGTTGTTCCGACCACCGCTGCAGCCGCCATACGTAAACTTTTCGCAATATTGCCCGTTGTACCACCAACGAGGGATAAAAGCTCTACAAGGTCCTTCGACGGGGCCCTCCCTGCATACATCTTCGCACGTTTCGTTCTTCTTATCCCCACTGCATATTTCAGCCCAATCAACTGCGTCCTCTCTGCTGGCTACACCTGCGCCTGGAGAGAAATTTCTCGCCAATGGTCCCAAACTTGCATCCAAAGAGTTCGGCGTCACCGCTGCCGCGATTGCTCTGCCGCGACCAGGATCCACATCTCCTAGAGAAAGAAGAAGAGTAAACAGAAGGAACCTCATTTTCAGTCGCTCGGTTAGCCAGGAATTAGGAGTCCAGCATTTTTGTAGGCAGATATGAGCTTATTTATCTACTCAGCCCCTGACATTAATCCAAATTCTCCATTCAGACCTAAAACGGCC |
| >AAFM7608  GTATGCTTTCCCAATTTGGTTGGTATAAGTGATCCTTCCATTTCGAATCATCAGGAGAATATTGTTCATGGTATGTATTTTCTTGCTTGAGTTACGCTCAATCGTCCAATAACACCAGCAGCGGGAAACTGAAGTTGCCGCATGCCTTCTTGCATTGCTCCTCGGATTCGAAGCTGTTCCGGCCACCGTTACAGCCGCCGTACATAAAATTTTCGCAATATTGCCCGTTGTACCACCACCGAGAGATAAGAGCTCTACAAGGTCCAGGGACCGGGTCCTCCCTGCATACATCTTCGCACGTTTTGTTGTCCCCACCCCTACTGCATATGTCAGCCCAATCTACTGTGTCGT |
| >AAFM8318  CTGGAATGTTATGAAAAGTGCGGTCGGCTCGCAGGTAACCCGTGCGTAATGCCTATTGCCCATGCTAAAGAGGCATGCATAAATGGCGAGAACCATACCCTGAGTTACGGATACAACTATTCTACGCAAAAGTGCGAGCAGTTCCTGCAGTCTGGCTGCAGCGGGAATAAGAATAGTTTTCCAACGTTACGGGAATGCCTCCAAAAGTGCCGGCCCGAGTCACGATGCCTGAAGCCTCCTAAAAAGGCAATGAAAATTTGGAACTGGGGGAAATCCTCTTACATATTCGATGTGAATAATATTGTATGCAAAGAGGAAAAAACTTTGTTTCGGCAGAGTTCGGGCCCCGGGTATAACCGATTTAACACAAAGCAAGAGTGCGAAAGCGAATGCATGCCACGTTTTGAGGAAGTTGTAATAACCATTGAGCAAAATCAATAAAGTGAT |
| >AAFM9317  TCGTGTTTATTTTGTTCGTTCTGCTTACCGCCGCCACTATGATTCGCAGTCAAACTGGTGCCTCAGCCACGGTGTTTTGCTGGCTGTTGGCTGCCTTTGCCACCAGACCTTGCATGTTCAGGGACCTCAAACGCGCAGGTTTTCATGCACTGCTCGTGGGACTTGAAGGCATTGGAATCCTCTCCTCCGCAGCCGCCGTACAGGTACTGCTTGCAGAGCAGGTAGGTCGTGTCGTAGTACCACCGCGGCTCGTAGTCTTTGCAGTGCTGCTGGTTGGGCTTCAGCCAGCACTTGTTCTTGAGGCGCTGGTCTGTCGGGTTGTCCTGTTCCGACGACCGTCTGGCGTGCTTCGCCTCAAGTATTCCTGTCAGCGTTCCAGATATGATCAAGGCGTACACAAGCATCCATTTCGTGCTGTCCATGTTGAAGGACTTCGAAGATGTACGACCGAAGCTTCTCCCCTGTTCACGACTACTTCATCGAAACCCCTAAACAGGCCTCTTNNNNNNNNNNNNNNNNNNNNNNNNNNNNNNNNNNNNNNNNNNNNNNNNNNNNNNNNNNNNNNNNNNNNAACGGAGCGCCGCCACACAAGAGGTCTGAGTATATGAACCGCGAGTGCAGGAGTATTAGGGAATGAAAAAAAAATGAATTCGACGATTGCTAGGAAAATCCACGGGATGCGGCATAAGCAGTCTTTTAGTTGAGGAGACGTACCCTCTCACACCCTTTGTACGC |
| >AAFM10492  CGACCGGCCTTCAACTCCAAGATCCCCGAGGACTAATCTTTTCCTAATTTTTTTAACGATGAAGACGGCAACCTCCTTTCTATACGCCCTACTCTGTCTCACAGTAGTAGTAGTGCAAGGGTACAAACCCCCGAAATATTGCAATGCACGACCAATCGTTGGACAATGCGGGCACGACCGGCCTTCAACTCCAAGATGGTACTTTGATGCGAGATACGGCTACTGCGGTCGCTTCTTGTGGGGCGGATGCGGCGGGAATAAGAACAACTTTCCCAACTGTACATCCTGCATGACTACTTGCTCGACTCACCCAGATCCCGCAGGCGCCTGTCGCCACATTCTCCACTCCCCATGATCGAGGAAGAACAGCTTATCCAGAAAATTCCCGGTCCTGCTCTTGAACATTGTGGCACTCTTTGTGCCCTATTAAAGAAAATAAAAAGACAAAATTGAAC |
| >AAFM10511  TAGTATTAGTGCAAGGGTACACCCCCCCACGATATTGCAAAGCCCCACCAAAGGTTGGACAATGCGGGCACGACCGGCCTTCAACTCCAAGATGGTACTATGATGCGAGATACAACCTCTGCGGTCCCTTCTTGTGGGGCGGATGTGGCGGAAATAAGAACAATTTTGATGACTGTACAACCTGCATGAAGAGATGTTCAACTCACCCAGATCCCGCAGGCGCCTGCCGGCATATTATCAGCGCCCCGTGATCAAACAAGAACACTTCAGCAAGAAAATTCGCAGTCCTGCTCTTGAACATTGCGG |
| >AAFM11667  GTCACCCAGTGAAGACTGCATGCAAGAGCCGGATCCCGGAATATGCAGAGGAATGTTCAAGAAATGGTTTTACAACGCGACGTCGTACCGTTGCGAGGTATTTTACTACGGAGGCTGCAATGGAAACGGCAACAGGTTCGACAAATTTGCGGAATGCAGTAAGAAATGTCGAGACCCTGTTCTCGGAGTGTGTGCCCTGCCAGAGCCCAAACAAATTTGCCGAGCTGGATATAAGGGGTACCGGTTCAATCCGTTCAAGCAAAGATGCGTGCGTTACATCTACTGCGAGCACAATGAGAACCATTTCCCGACTGAAGAGGAGTGCCAGGCGCAATGTGGAAAATTCGCCCAAGATTCATGCCTCCTTCCAAAGCACGCGGGTCGCAACTGCAGCAGGTCGAGCCGCATGCAGAACTTCTGGTTCAACAGTAAAACAAAAGCCTGCGAACAGTTTGACTACGAAGGCTGCGGAGGAAATGGAAACAATTTTCTCTACGAATCCGAATGCTGGAAAACATGCGGCAAATACGTCGAAAGCAATTGCAGTTACCCAATACATCGCGGAAAGGAGTGCTCAAATGGTTCACGTAAGGTAGCATTTGGATACAACAACAAGAACAAAAGGTGTGAAAGGTTCGTCTACTACGGCTGCGGTGGCTACCCCAACAGGTTTGACTCCGCAAGGGAGTGCTGGAACACTTGTGGCCGGAACTCTGGGAGCAAGTGCGTAGAACCGGGTCCCAAGAATAGGTTTGGACTTGTGAAAAAATACTACTACGACGTCGAGAGCGACAGCTGCAAGACTTCGCGATACTCTCCGTTCTC |
| >AAFM12060  CTTCACTTGGTGCTACTCAGAGCCAAACAACGGCGTCGACCATGAAGTTACCTATTTTTCTTATTCTTCCAGGGTTTATGGTGTCCGCGCTTGCGGCTACTTATGCAACGCCGCACATTTGCTTGAAGCCACCAGTAGTTGGCCCCTGCAAAGCATTGCATCGGAGTTGGTATTATGATCCCGCAACGAAGAAGTGCAAAGCATTCATTTACGGAGGATGTCAAGGTAACTCGAACCGGTTTCCAAGCGAGGTCAAATGCCAACAAACCTGCTTGCCTGGAGTTCCCGTTGAACCAGTGTGCAGCCTTAATCCACCCAAAGCTACATGCAAGACGGGAGTTTATTCATGGTCATATGATCAGGGAGCTGGCCACTGTCGCTTTCATCTCCACGCTGGGTGCAAAACAAACGCAAACAGATTCAGAAGCTGCCTCGAGTGCATGGACAGATGCCGAGGTATGGAACCCAGAAAGGCACACAAACTCTGCTGGAAACTCACTGCTCAAGTGGTCAAAAAATATGGCAATCGGCTGCGCCCCTCCCTTGAAGGACCAGAGTAGGCTCGTCTATGCATGCCTTCCGGAAATGATAAGGCGATGCTTTGCTACGCAATAAAA |
| >AAFM12061  CGCGTAACGAGGCATGTCTTACAATTTCCGGAAGGCATGCGTAGAGGAACCTACTCTCGTCCTCCCAGGATGGGGCGGAGAACATCGCCATATTTTTGGATCGCTTCAAGAGCCAGTTTCGCGCAGATTCTCCGTGCATCTTTGGGTTTCGTTCCGCTGCATCTGTCGATGCAGTCGAGACAGGTTTTAAACCTGTTGTCGTTTCTATTGCAGTCGCCGTGCAGAAAAAAGCGGCAGCCGCCAGCTTTCCAGTCAAATGACCAAGTATGAACTCCCGTCTTGCAAGTTCTATTTGGTGGCTGAAGGCTACAAACTGGTTTCGCGGGAACTCCAGGCAAGCAGGTTTTCAGGCATCCGACCTCATATTGAAACCTGTTGTTATTACCTTCACATCCGCCGTATATAAATAGTTTGCACTTGTTTGCTGACGGATCATAATACCATCTCGGGACCTTCGCTTTGCAGCGTCCAACTGCAGGTGGCTTCAAACAACGT |
| >AAFM12105  GTATTTGTTTCAGCCATCGTGGTCGTTTTCCTAGCCGCCTATTCATATGGCCAACAAGATTGTGGCGTACCACCGCCACCGCCATGCGTCTATCCTGATGGTTGCAAGGAGCCGCAAAAAGTTGGACCATGCAATACAATCGTGCTGAGATACTATTTCAACCCTGTGACCAAGAAGTGCGAGCAGTTCAAATGGGGTGGATGCTGCCCAAACTGCAACAATTTCGCAACGCTTCAGGAATGCCAAAGAACATGCGTTCGTTAATCTTCAAAAGCTGTTCGAGTTCCCTTTGCGTGTTTTGCATTGACCAGCGGCAAAGAGCGTTGTGAAATAAATAACAAAATGGTCGGAG |
| >AAFM12472  TTTTGAAATGCGAAAATCTTAGTTTATTGGGTGATCCACTTAGTGAATTCGTTATAAAATTCCCTTCTCTTGATGTCCCACCAAAGCTTCACATGTGTAAACCAAGATACGCTGAAAACCAGTGCCGAGGTGCTTGCTGCCTTCACCCTAGTGGGCCATAGATTTCTTCTAAATATAATTCGCATGCTTTCTTGGGGTCTTCATGGCCACTGCATCGACGCATGCAGGTAGTGCAGTCAGAATAAACGTTTTCGTTTCTTTGGCATCCTCCGTACTGGAATGGTCTGCAGTGCCCTTTTTCGACATTAAAAAACCACTTTGCTTCATAGCTAGTGCACAGATCG |
| >AAFM12716  GTCCAGTGTCTCCTGATGGAGGCAGGTCTGTTCACAGAGGTCTTTGCTGACAAAGCGGTTGTTGTTTCCGAGGCACCCTCCATACATGAACGACTCGCAGGTGCGAGTCGAAGCATCGAAGTACCAGTGCTCGTACTGGCCATCACAAGGGCCAACCACCTTGGGGAGCTGGCAGGCATCAGGTCCTTCTGGTTTGACACAAGTGTTCTCACACTCCTCTTCAGAGTTGAACCGGTTTTCGTTGCCCTCACAGCCGCCATACCAGAACCGGTTGCAGCGGCCATCAGCAACGCTGAAGAACCAGTTGACAGTGTAGTTTCGGCATGGGCCAAGGTCTTCGGGCAGGCCACATACACTGGCGCTGACATTCCTGGGGGGGACAATGATCTTGTCCGGACAGCCCTCAAAGTGGTGCCCCCTTGCAGCCGTGACGCCATCACGACAGCAGCCATACGCAGCCAGGGCACAGGTGCAGCCATCGGCATCCGGACAAGATCATTGTCCCCCCCCGGAAGGAATGCCTATGCAAGGGATGCCCATGCAAGGGATGAATATGCA |
| >AAFM13495  TTTTGCTTTTTCAATAGTTTATTCCTTGTTCCCGAGGATAAGAGAAATGCGTAGGAGCCAAATATTTTCACATTTCCTCTCTGCTCTGTAGCACTGTTTGTCTTCTTTTCAGTTGAATCCCCTGGACAAGAACGATTATTTCTTATTATCCTGCTTTGCAAGTGCGCCGACATTCGCCTAAGGTTGGAAAGTTGTTTCCTGCGTAGCTGCAGCCCATATCGCTGTGGCACATGCCGGTCGAAGGACTAAAATAGAAAACTGGAAATGTCTTCCCGCGGCAAAGGGTATACGGTCTCGGAGCAGTGCAGACGGCAACACTTCGACAGGTAGAATCGCATTCTCTTTGGGTTTCAAAAGGAGCTCGGCCGCATGTCCATGTGCACTGCTTGGTCCTACGGTTGAAAGCGTAGCTGCGGTGCTGGCAATCATTTTGCACCGCCGGGCGGCTAGATCTGCATCGCGAATCGTGTGGGTTTGGCCGCCTGGCACTACAAAGTGCCAATAGAGCAAGCAACATAACCGCCTTCCAGATGGCCATGGTCCCAATTGTCTTTGGGTTTCAAAAGGAGCTCG |
| >AAFM13563  GTTCCATCGTGCTGTCCCCACTTGTGGCCATCATGAAGGTCTTCATTTTGCTTGCCGTGGTCAGCGCCGCTTTCGCAGCGACTGCGTTTGACATGAGATGCACCCAGCTGCCCGATAGCGGTCCCTGCAAGGCGATGATGCCGATGTGGTGGTTCAATGCCAGGACTGGAAAGTGTGAGCTTTTCAACTACGGCGGCTGCGGAGGAAACGAGAACCGATATGAGAGCAAGCGACAGTGTGAAATGACGTGTTCTGCGAGTAAACCTTCTTTCTGGCCCGAAAGGCATTGGGGAGTGCGCAGTGGACTGTTCGCATCCGTGGCTGATGTGTGCCGTCGCCCTGCATACTCCGGCATTTGCATGGCTAGCATCCCGCGCTTCTACTACGATGCTCAAACAAAGCAATGCCGGCAGTTCATCTACGGAGGCTGCCACAGCAACGGGAACAACTTCGAGACCCTGCGCCAGTGCATGGACGCCTGCGCCATTCAGTCTCCTTGGGAGCCGATGCCCCGGCACGCATAGACGTACATGTTGCACTTACTGCTTTTCGAAAAAGAAGGTGCTAAACATTCTTAGACCTAAAAGATTATAACACATATGCGTACACATTCAATTTAAAAATAACTGAAAAATTTTGTGAGATCTCGGGAAATTCATGCAGGATATAACTAGTGTAAGAGGCAGATTAATAAAGCTGATTTACTCAGCCTCTTGTTCGTTTGCGATGTATCTGGCTTGACACTCTTCGGAATGGGCTCTCCCCCATGTCTCCGCGTGAAGTAAATAATTTCGAGCAGACCATTTCTTTGCTTACATATTTTAACTGTTTTCCTCCTGCTCAACTAACGCCACCATTTCCGAAAAAGTAATTTGCATAGCGGCGAATGCGCATCAGGTGGCAGGGACATCCCACAAAGCTGTGACTATAGCTGGCGCTGAAATACCTGTGCTGCATTTGT |
| >AAFM14636  CGCGGCAATGTGAAGAGAAATTCATATTTATTGGAATCACTGCAGTTTATTTACCCTGCCAGTTCTTTGGTACTATCTCGGGTGCGCACTCATCGTAGCATTCTTTCTCTGTTGCGAATCTGTTCTTTTTAGGGCTGAAGTTTGCTTTTTTATACTTCGCACTGAGGCAGTAGTCGACACTTGCGTAGTAAGAGTAGCCTTTAACTGTGCCGTTCTCTCTTCCTTTTCTTGGCCTCAAGCAAGGAGAGTCCTCCATACAGGTTTTCATACAGTCGTGTCGTTTTGCGAAATTATTTCCAACATCGTAATTATTTCCGCACTTTTCCGGAATGAACAACACGCATTTTTTTGTCGTTCTGTTATAAAAGTAGCGCCTTTCGTGAAATGGGTTTCCGCAGTGTTTTCCAACTAAACTTTCTTCTTGTTCGCAGTCCCGTCGGGAGAAATTCCGCGGCCAATTGAATTCCTGCGTTGAAAAGATTCCTCCGCCAACTCCAAGTGCAGCTCCACTCCAACACCACAGAAGAGCTATCGCAAGGAATTTCATGTCTCGCTGCTTCGGTGCACCTTGCGATAGCTCTTCTGTGGTGTTGGAGTGGAGCTGCACTTGG |
| >AAFM14668  CAGATGCGCCGTGGTTGAACTTGCCGATAGTCTCCACGCCGCCAGAGGCATTTGTTGCGTTAGAAAATTTTGTGCAGAGGGCAAGATTGCCTGGCTGCAGTTATCCAGTGTTGCTGAAAAAGACGCTGCTTGTAAGCAAATTCCGTTGACTGCCACAAAGACAATGCATATTTTAGAGATTGGGCGCACCGATGGAATGCGACAGAAAGTTCATATCGCCAGGGACACAAATTGCATTCGTGGACGTCCTTGGCAGTTACTGCCAGTGCGCGCAGATTTCACTACACACCGCGTGCGCCTGTGATCACGCATGCGCTAATCGAGAATCTGGGAACAAAAAAGAAATATTTCCGTCCTCCTCGTCGCTTCTTCAGAGAGTGCAGAGCGACGCTGTCACCGATGCACCAGTGAGGAACACTAGAGCGTAGAGGGCGGCCCCGTTGCTACGCGGGGCTGCGTTCCCGCACTCGCATGACCCGCACTTGGCCTCCTGCTCATCACCGCAGCTGCACTGCATGGACTCCGGGCAGCTCGCAGCCGTCCTGGCCAAGTTGATGCGCACACCAAAGCAGGTCATGTTGCATCCGTCTACGGTACGGAAGTTGTTGTCGTTTCCACCACATCCCTGGTAGTTGAACCGCTCGCACAGTTCTTTCTGCACGTCGTAGTAGAAGCGGATCTCTGGTGTAGTGGCCCCTGGGCCACTGCTGTCGGTGTCGCAGTGGCCTTCGTCCACCGGCTGGTGACACATTTCCCTGTCCTTTCCGGCTACGCCGTCCTCAGCAACATGCACCCTGTCCTTTCCGGCTGCGCCGTCCTCAGCAACATGCAATCGGCAGCGTCCGTTGCACTGGGTGCTGGTGTAAAAGTTGTTTCCGTTGCCCATGCAGCCACCGAAGATGAAAGGCAGGCAGCGATCCTCCTGCTTATTGTAGTAGAAGCGCCGGAAGTACGCGTAGCATGGGCCCGGGTCTTGGGGCTGCGAGCAAACATCTTCGCAAAGCGTCTTGCACGCGTCGAGGTCTGCAAAGTTGTTCTTGTTCCCTTCGCAGCCGCCGTACTCGAAGGTCTCACACAATCCCGTCTGTGAGTTGAAGTAGTAGCGCTCCTGCGTGTCGTTGCAGCTGCCAGGATCCATTGGCAGGCGGCAGGGATCTTCACACTGGGCAATGCACTCTTCATGGGAGGCGAAGCTGTTCTCGTTCCCACCACAGCCGGTGTAGTAGAACACGTCGCAAACGCCGGAAGTGACGTTGAAGAAGTAGCGGGGAATCCTTTCATTGCAGTTGCCTTCTTTCACCGGCAACTTGCACGCACCATCAGACGGACAGTGACAGCTCTGGGCGCCGCTGCTCGAAATTCGCTTCACGCAAGTCACTGGGCAGGGCACTGCGATTTGGGCATCCACGTACTCCAGTGCATATTCAACAGTGCCGTCTGCAGCAGCACATTCGCAGGCGTAGCACGACGCAGACACCTCCCTACTCTGACAACCAGCCGGGCACAGCTCGGGAGCCGCGAGGCAGTCAGCTTTCTTGAGAAGGGGCTCTGTTTCGGACGCGAGGCAATTGCACGCAATACAGCTGTCTTTCTCTCGCCGCTGGCAGCAGTGAGCGCAGGCTAGGCCGCATGCTGCACGCTGGGGACACTGGGCTGAATCGGATTCCACAGGTGGAGCTCGAGCTTGCTCCAGTGGCTTCTCGCATCTTTCAACACATTCTTGGCGAGTAAGGAAGTGGTTTTCGTTGGCTCCACAACCGGTGAAAGTGAAGTTCAGGCAGCTGCGTTGCTCTCGGTCGTAGTAGAAGACTGTCTGCTTATTCGCACAGAAGCCTTCCTTTCGTTCCAGGAAGCATATTCCCGTGCCTGGTGGTATTTCGGCCAAGGTGGGATCTTCATCGGACGGTAGAGTGCATGCTTCGTCAAACTCGTGCGGTTCCAGCTGTGCGACAGCAACGCAGACGTTCTCCAAGTGGCGCGTGATCTCCAGAGAGGTGTCTCGTTCGAGCACGTGCCTCGCAAGGACGCTGACGTGTGTCGCATCCGGGGCACACACCGACGCGTGGGGCTGCTCATGCGCGCACCGGTGCAGCACGGCGTAGCTCCCGTAGTCCGTGGCTACCACGGCCAGCGTCTCTTGTACAGGCTGCCCAAAAAAGTCGTACTCGAGC |
| >AAFM14814  TTACGAACAAGAGTTTTTCACAGAGCTTTAAGGTGTGAGCATTATGGTCAAAGTACAAGTCTGTCTTCTTCTAGCCCTCTTCTGCCTTGGACTCTCACACGCAAGACGTCCAGATCGTGCAGTTTGTATGAAAAAAGCAGATCCAGGGCCTTGCAGGGCTTATCAACCAATGTGGCACTATAGTTCTGAATTACGTCGCTGCACTGGATTCGTGTATGGTGGTTGCCGAGGAAATGGCAACAGATTTGCAAGCTGTGAAAAATGCATGAAAACATGCGCCAGGGGTTCGAATCCACGACGCCTCTGTAAGAAGCTGAAGGAGGAATTCAACAAGAAGTATGGAACTGCCCGCCTGGCGAAATAACATCTTCGGCAGGGAGAGCTCAGTGGGCAACCACGAGCTGTGTTTAATGGGAATTATCGGGGCAATACTCCAGCCAAA |
| >AAFM14825  GGCTGAGCAAAATTTCCCGTATGTCTTACTTTGGCTGGAGTATTGCCCCGAAAATTCCCATTAAACACAGCTCGTGGTTGCCTACTGAGCTCTCCCTGCCGAAGGTGTTATTTCGGCGGGCGGTAAATTCCGTACTTCTCGTTGAATTGCTTCTTCAGCTTTTTACAGAGGCGTTGTGAATTCCAACCCTTGGCGCATCTTTTCATACATTGCTCACAGCTCCCAAATCTGTTGTCGTTCCCTTTGCAACCACCATACACGAATCCAATGCAGTGACCTAATTTGGGACTATAGTGCCACTGTGGTTGATAAGCCCTGCAAGGCCCTGGATTTGCTTCTTGCTTACAGACTGGGACATGTGGTCGTCTTGGTAGGCAGACTTCCAGACACTTCTTCTGCGTGTTGAAGTTGTTGGCGTTCCCATTACAGCCACCGTAATAGAAACCTTTACATCTTTGGTCACGGTGATCGAAATACCACTTTTGAATGCGAGCTCTGCACGGGCCCACCTCTGGCTCTTTGTGGCAGTAGTGTGGAGCCCGCCGGGTGACCACAGGAGCAACGAATGCGGTGGCCAAAAGTAGGGTGAAAGAGGTGCGATTGCACATCATGGAAAATCTCTCCTTAATGGGGCACGAAC |
| >AAFM15051  CAGATTTGCTTTCTCGAAGGAATTCCCTGCGAGAGGCTTAGACAGACGAGTGGCATCACCAGGAAGCGTAGTTTTTCGCCAACCTCGCAGATATGAGTTCGATGGTCGGCGTACACACCTGCCTTATTCTGCTCCTCGTCTGTCTAAGCCTCTCGCAGGGAATTCCTTCGAGAAAGCAAATCTGCAACCTTCGAAAAGACACGGGACCCTGCAGAGCCTCAATTCTTATGTGGTATTACGAACCACGTGATGGCCTATGCCGCCCGTTTTTATATGGCGGCTGTGCAGGAAACCAGAACAAATTCCGCAGCTGCACAATCTGCATGTCCGAATGCACAAGGAAGCGGGACAAAAAAATTAAACAAATTTGCCGTGGCCTGAGGAAAGAAGCTGAGCGGTTCGGGCGGCCTGGAGGGGCCAGTGCAAGAAACAAGTAGCCAGCATTGTGTACAACGCGCAGTATACAATAAATTTCGTTTCAGTTGAAATTTAACGAATAATTTGTTTGTTATGCAATAAATTCTTAAA |
| >AAFM15677  GCTTCACACCTTGGCGCTCCTTGACCTGTCCTGCACTTGCTGACGCATTGATACAAGGAATCAAATATGTCTCCTCCACCGCAGCCAAAATTGGTGACTACATGACAGCGACGATCATTTTCGTTGTAGTAGTACCCTTGGCTGGTTCCGCTGCAATCTCGCAGTGCGCTCCAATTTGGAGTCTTACAGACCTGATCGTTAGCGGCGGAGTTTGACGCATTGCTGATAGCTGGGAATATAGAAGCTACCATAACGGATGCTGCAAGGACTC |
| >AAFM15698  CCGGGATTTTGAATTTTATTACAAGCAATCAGTTACTTCTAAAGCCTTAAGCACAAAGCTAGTGCATTGCACCGAACGCAAAAGAGAAACATTGTGTCAAGCTTGCAGTTAACTTGCTTGTGCATCTCGCCGTCCCATCCCCGGTTCTCATTCGGCTCTGCTTCCTCCTGCGGCAGTCACCGGCTGTCTGTTGTGGAGCAGCTGACAGTATGCCTTTCCGTTGAAATTTGTACATCTTCTCATGCAATCCTGGCAAGTTCTAAAACTGTTGCGGTTTTTTCCGCAGGCACCGTATGGGAACCTAACACAGACGCCCTCTTGTTGATTGTAGTACCACCGCAGTCTGTTCCCCGTGCACCGTCCTGGGTTGGGAGGCTGGCTACAGGTCAATAGAGGTGTGCGTCGCGGTAGGCACGTTTGCAGACATTTTCTCTCCGAAGGAAACTGGTTCCTGTTCCCTCCGCAGCCACCCCAGATGAAAATCTTGCAGACTCCAGCTTTCGATTCATAAAACCACTTCAGATATTTTGCTCTGCAGGAACCAGTTTCTGGCGCTTTCCTGCAATAGTTAGGAGCCGCGCACACATAGACAACGGAGACGAGTAGCACGCATACTGCACCGAAAAATTTCATATTTGATCTTTCTGTGGAGGGTAGATGCGGCCTTTTTACTGAAAAAACAACCCAG |
| >AAFM16108  TAGATTGGGCTGAAATATGCAGTGGGGATAAGAAGAACGAAACGTGCGAAGATTTATGCAGGGAGGACCCGGTCCCTGGACCTTGTAGAGCTCTTATCCCTCGTTGGTGGTACAACGGGCAATTTTGCGAAAAGTTTACGTACGGCGGCTGCAACGGTCGCCGGAACAACTTCGAATCCGAGGAGAAATGCATGAAGGCATGCGGCAAGTTCAAATTCCCGCAGC |
| >AAFM16995  GGTGCAACTCTTCATGCATCCCACGCAAGTTCTAAAGTTGTTGGCATTTCCGTAGCATCCGCCGTATACAAAAAAACGACAGTGGCCTGCAGCTGGGTCGTACGCCCAAGCAAAATAGAGGGCACTGCAAGGACCTCTTACAGGAGGAAGGCTGCAAACTGGTCTTGGTGGAAATCCAAATGGATTACGGTTTCCTTGAGGTACCACCTGATTCCACATTCCACCCTCTGCACTGACAGCGAACGCCAGAAGAACGAGGACGTTAATTTGCAGTTTCAT |
| >AAFM17784  CTGTGAATTCACCGTCCCTTTGCAGCTTTTATGATGTAGTGGTCCAGGCTGAGAGACGGATCTTGTCCAGGTTGTTTCTGGGCACATACTTGAGTCCCGCATGCCGTCTTGCATTCCGGACAGCCAAGAAAGATGTTCTCATTCATTCCGCAGCCACCGTAAAGAAATGGTCTGCAGGCTCCCCGTTCCTCGTCGTAGCACCATTTCCACGAAAAACTCGCTGTCTCACATTTGTTGTCGACAACTGGTACTGAGCACTCCTGTGTTCGCACTTCACCGAATAAAAACCACACATTCAAGGCAAGCAAAGCAGGCGGTAATGCTAATCTCATTGTCACATTAACATTAGAAATTGGTGCTATAGGATTCGCT |
| >AAFM17939  TAGTGAATTCGTTATAAAATTCTCTTCTCTTGATGTCCCACCAAAGCTTCACATGTGTAAACCAAGATACGCTGAAAACCAGTGCCGAGGTGCTTGCTGCCTTCACCCTAGTCTGCCATAGATTACTTCTAAATATGATTCGCATGATCTCTTGGGGTCTTCATGGCCACTGCATCGAAGCATGCAGGCAGTGCAGTCAGGATAAACGTTTTTGTTTCTTTGGCATCCTCCGTACTGGAATGGTCTGCAGTGCCCTTTTTCGACATCATAAAACCACTTTGGTTCATAGCTAGTGCACAGATCG |
| >AAFM18150  CTGCACATTTATTTTATTTTAGTGAAATGACCACGAGCTGTAGTCTGGCGCGAATCTTCTTAACTTCAGAGCACATGCTCTGCGCGTGGCTGGATACTCGACCGCTTCTGGCAAGGATCCTAATCGCAGGAGTGCTCACCCCATACGGTTGTTCCCAGTCGTGAAGTTTTTTGCGAATTGCGCTTCCAGCTTTCTGCATAGAAGTTCTCTTCTCCTTCTGGTACTGCATGTTTTCATGCACACCGAGCAGGAATCAAATTTGTTGTCATTTCCACCACAGCCACCGTAGACAAATCCAGAACAGAGGCGTGTCTGCGGATTGTAGTACCACATCGGTTGGTATGCCCTGCACCTGCCATTTTCTGGTCTTAGCCGGCAAAACCCAGGCTTAGATCTCGCGTGTGAGGCAGTCAGGTAGACAATGAATAGAAGGAGGCATACTTGTATTCGGATTGCCGCTCTCATATCGCAAAGCCGTGCAAAAATAGAACTTCCTTCTTGTGCTTCTAATGAGTAACCGTTGTGCCGAGCCTTTTTGAAGCGGGCGTTGCTAAGAATTCTTCCCGCTTTCGCTAGATCCCCTTTATATGGTAGGCGTCACAATAGGAGTGCTCCCC |
| >AAFM18301  GTGAATTTTCAAAAATGCAGAGAGAAGAATATCTTTCCAGCAGGCAGTAAAATCTAGGTGACGCTGGTTATTCGGCTCTTCTTGCATTGATTGGCTTGTGCAAAAAGCTATTTCCACTGTTCTGCTTCTCCAGCCTTTTGCAAGCGATCTTTGCTTTTCGCCATCTCAGTCCGCCACATTTCGTCATGCAGTCCTCGCAGCGATCAAAACTGTTATCATTTCCCTGGCAGCCGCCGTAGATAAAGAAAACACAGGAACCTGTTTTGATGTTATACCACCACTTGGGCATGGATGCTTTGCAGGGCCCAACCACTTTTGGAAGGGAGCAGACTTTTTGTCTGTTGAAACGGGCATGTGAAAGAGCCAGGCCGAGGAGGAGCAGAAAGAAGCACGCCGGTAACCTGGCCATCGTGGTCATTTTTCGAATCTTTGCCAAGAAGGCGGCTTTGTCGAG |
| >AAFM18828  CCACACGGCAAACATCTCTGCAGTCGGCTCGCACCACAGCGGCAGGCTGTGCCTCGACTTCAGCTACAGGGCAGTGGCCAGCTTTGTTCACCAGTCGGCATACTGGTCGGTAGTTGGGTTTCCCGTCGGCACTTCGGTACGCTTCAATCCTGCACAGCTCTTCCTCATGGCACGAGAACGTCTCGCAAGGGTTGCTGCAGCGACACTGGACACACCCACGGTGGTCCACAGTCTTATTCTTTCCCAAGGGGCACTGCAGTTCCTCACAGTTTGACGGCAGGCACTCTGGCGCTGGCGTGCGGGGCACAGTTGCCGGTGTCAACGGACTAGGCTCTGGAAGTGGTGTGACTGCCATTGGTGGCTTCACACGTGGGTCCAACTCTTCGCTTGTGTCACTAGGCTCTGGAGGGGAAGGCGGCATCCATTTCCTGTCCGCATCAGGATCGATGATTTCGTCATCAATGCTGTCCATTGTGACCCCAGAGCAGATCTTCCGGCAGATCTCGGCCGACTTGAAGCGGTTCTTATTGCCTCCACAGCCGGTGTACACAAATGGCAGGCAGAGCCCGCTCTTTACGTCGTAGAACCATTTAGCATGCATTTCACTGCAGGTTCCCG |
| >AAFM19177  AAGGCCGCATCAGCCACGATGACCGGTGATGAGCGAAGTCCTTCGTCTGGAAGCTCGCCATTGATGACTGCTCTGGTGGCATCAGAAGCTTGCGATACTAGAGGTGTAGTTAGCTTTGCATGTCGCCTCGCAGTCACCCTTCGTGTTGAACCTGTTCTTCTTGCTCGAGAACGGAGAGTATCGCGAAGTCTTGCAGCTGTCGCTCTCGACATCGTAGTAGTATTTTTTCACAA |
| >AAFM19479  GCCACACACTTTTAGTAGACTTTTGTCTATAGTCTATAGACTATAAATACGCAAAAAGATATATGTAGGAAGGCAATAGAGTCCATAAGAAGTCTATAGACGGTCCATAGACCATTTTTATAAGGGTCCAGTGAGCTTCCCTGTCCGCGACCGCGGCGCCCTCTTGCGGTCAGTCCGGGATGCAGACCGCCCGGCATTCCTGGGCGGAGCGGAATCTGTTGAGGTTGCCGCCACAGCCGGAAAACCAAAAAGGAAAGCAGTGGTTCTTTTCTTTGTCGTAGTGATACCGCACCACCCAGGAGCCGCATTGGCCTCGGTCCATTGGAAGCTTGCAGTAGATGTTGTAGTTTTGGAACCAGCANNNNNNNNNNNNNNNNNNNNNNNNNNNNNNNNNNNNNNNNGTGTAGACGTAGCGGATGCACCGTCCCACGCCCCAGTGGAAGTAGTAGCGGAACAGCCTCCCGTTGCAGCTTCCGGCATTTGGAGGCACGAAACAGTTGGGCTCCGTTCTTTCGCGAGGTCTGGAACGCATCTCGGCGGCGGTCTCAC |
| >AAFM19650  GTTCGTCTCACTTCTGCCACTACGCCTGAAAGGAAACTGCGATGCACTGTCTGCTCATTTGTCTCCTCGGGTTGACTCTTGTGACAGCATCACGTTCAGAGCAACGAAAGGTGGCCAATCATGGAGCCTGCAAGATACCGCTGATGGAATATGAACCTCGATGCGAAGCAAAAGTCCCAAGATATTTTTACAATACGACATCACGCCGTTGCGAGCAGTTTCGATGGAACGGGTGCCTTAAGGAGGATGTCTATGAGAAACGAATTGACTGCGCGATGAAGTGCAACCAAAGTGAAACCCTGGATATATGCGGAATG |
| >AAFM19774  CCACTTCTTCACGAATACTATAGAGCATGCAGTTTTACGCCGAACTATGCATTGCGCTCCAAGTAGTTGAACATAGTCGCTGGTGGTCACTTCATGAAAAAATATTCAGGGTGCATGTGGAAGGCGTCCTCACAACGACCACTTCAATCAGGAGCGAGGCGTGATGTTCTGCCTTGGACTACAGGCTTGATAGCACTCGCCGAGACTGCCGAAGTTGTTGGCGTTGCCGCCACAGCCACCATAAGCAAATCCTTGGCACATTCTGTTGCTGACGTCGTAGTAAAATCGGTGAATGAAGCCGGAACACATCCCACGGACCGGGGGCAGTAGGCAAGTCGGATTTGTCAGAACAGACCACATCAGGGAAGGGACCTGCGGCGGAGAGAAGACTTCCATGATGTCCTCTCGCAACTCCTGGGGCCTCCCCGGGTCTTGAGAACCTTTTGAGTGGAGGCAGATCAGCTCGCAGGCCGTCTTGGTGTGGTATTTGTTCTGGTTGCCGTCGCAGCCACCGTAGGTGAACCGGAGGCAGCGTCCCTTTGTTACGTTGTAGGCCCACATGGG |
| >AAFM20326  AACTGGAAATTGCACGGGCTTTTCTCCGTCATGGTACTACGACGCCGATGTGGATCTGTGTCGTGGTTTCATTTATGGGGGATGCTACGGGAACGCCAACAGGTTTGACAGCTGCATGAAGTGCATGAAGCGGTGCAGCGGGAACAAAAACGCTTGGAAAATCTGCATGAAGAGGACCGAAGCATTTCGCAAGAGATACAACTTAGGGCTTCAACCAAAGCGCAAAACATCCAAGTGGCCTTTCATATTAAGTATTCCGTTCTTAAAGAACTAAGTATGTGTACAAAGCATATTAAACCAAAATGTTC |
| >AAFM20473  CTTGCAGGCTTTGTGTTCGCCCGATGCATAAGCGGAATGAGCAGGGCAAAAGAGAGAGCCAAGTGTTCCTTTCATCTGTCCAGCGGGCTACGTAAGCTTGCAGGCAATCTCGCACTCTTCCTTCGACTCGTAGTTGTTGTCGTTTCCACCGCAGCCACTGTAGAGGAACGTCTCGCACTGACCTGAGGTCACGTTGAAGAACCAGT |
| >AAFM20724  TCCACCAATAAATCAAATGTGTTCTGTTTATTTCATCATTAGTATTTTGAACACGTTTCCATGCATTTCTCTATTGATGGGAAGTTGTTGCAGTTTCCACCAACAGTCGACCACGAGAATAATTCGCAGTGTCTGGTGTCTTTGTTGTAGAACCAGCGCTCTTGTCGTATGTAGCCAAGAGTACCTAAATGTTTCGGACAGTGACAACCTTTTTTTGGAAGTGCTTGACACATCGGGCTGTTCGGTAGAATACTGCACGGTCGTGTACGTGTCCTGTTCCTCTTGCCCTCTGCGTGGAGCACGGCGCAGAGAAGGACCACAAAACACACCAGGAAATTAAGGCTGCGCATAGTGTTGGTCACCTAGGCTGGAGTTGGTTG |
| >AAFM20982  CCGAAAAATCAAAGACGCCTTTATTCCCGAGCACAGGATAGAAGCATAACAGCGGAGGCGTTTACACACACTGACAGTCCACAACGACAAAGGTCAAGAATGCGGCATGAGAACCGGATTTGAGGAATAGCCGTGTTCACTCCTGTTCATCGTCACCACTATGCCACACGCATGGCCATTGCTCAGTCCTTCACTGATGGCCGCACGCTTCCTGGCAAGCCTTCCGGGTTTCGAAACGGTTCTTGTTCCCTCCGCAGCCTCCGTACACAAACATTTCGCACTTCTTGCTGTCCTTACGGTAGTAGTACATGGGTTTGGAGCTGAAGCATGGACCACTGTCAAAATCTAGGCTGCACGCTGGGTTTCCGGACCGAGTATTGGACGTTGTTGAGGGCGAGGGCTCGACGGTCGTCTGCTTTTCCGCAATCTCAGCGCCACGGTGTGGCTTCTTTTCGTGCTTGTCCTCGTGGTTCTCATCCACGTTCCATCCTTTCTCTTCGTTGGAGTCACCTTCATAGCAGACTTGCTCTAACTTTGGACCACACCAGTACGGGTCACCCGATCCTTTCTTCACTGTTTCCTTTGTAGGCTTTTTATTGTTGTTCGCTCCACAGATGACACCAACGCTTGCTAGAAGCCACCACAGCGTCTTCACTGTCTTCCGCATAGCGACTACATGCACGTTTGCGATTTAACTACCGTTTCCAGCGGTAGTACGTGTGATCGACCGGCGTAGTGACGCCGAAGAAATGTACTCCAAG |
| >AAFM21026  TTTAAACGCAGCCGGGATGTCTGCCACTGAACTCCACCGGCGTCCGCGACCATCTCAGGTGTCGACGATGCTTCGACTGACTGTGCTGGCGGCTGTGCTTTTGGCCATCTCGTTCAATGGTGCCAATGCTCAGAGAAGGCCCAGATTTTGCAGCCTGCCGCCTAGTCCCGGAGTCTGCTTCGCCTACTTCCGCTCGTTCTATTACGACATTTCATCCGGGACGTGCCGAGAGTTCGTTTACGGCGGATGCCAAGGAAACCAGAATCGCTTTGTTTCATATGAGGAATGCCTCCGCGTCTGCGGCTGACATCACTTCTGGATCGGAA |
| >AAFM22472  TTCAGAATACATGCAGAAACGCTAGCCAATAAAAGCGTGCACTCAAATATTCTGGCCATTTGCAGCTGACATATATCGTGTTAGCATCAAATCATCAAGATACGCGCGATTATGAAAGCACAATCCTGCCTTCTTCTGCTGCTCTTCTTAGTGGCCCTGTCTAATGCACAATCTTTGAAGAAATATTGCGGCTTTAAAAAGGATCCAGGAAGATGCAGAGCCTCCATCCCAAAATGGTATTATTCCGAATCTGGAGGGTGCAGGATGTTCATATATGGCGGCTGTGGGGGAAATGCAAACAAATTTGACTCCTGCGCCGAATGTATGTTGAAGTGCATGGGAAAACGTGGTAAGAAGACTAAGAAGCTTTGCCATGAGCTTACGCTAAAAGAGGAGAAGAAACTCAAGCTAAATGCAGGTAGTACATGATTACCTAAATAAGCGCATTCTTTGGAAACCAGAGCAAATTCAAACAACGTATTTCTATCCGGAATTAACGCAAGAAGACGCCCCAGGGAAATAAATAAGCGGGAGGATTTTTCCGCACATATAAAAAAA |
| >AAFM23137  CTGTGCGCGAGCGGATTGGTAATGGCAAGAAGGTGGCTGAGATATGGCGCTAGGAGTCGATTTAAACTTCTGGGAGTGAGGAACGGTACATACCTACTGGCCGTATTGCTGGTGTTATCGGCGTGTCTCGCCACCGTCACGGCTCAGGCCAACGTCAGTNNNNNNNNNNNNNNNNNNNNNNNNNNNNNNNNNNNNNNNNNNNNNNNNNNNNNNNNNNNNNNNNNNNNNNNNNNNNNNNNNNNNNNNNNNNNNNGCGAGCTGCGTTCCAGACCTCACGAAAGAACGGAGCCCAACTGTTTCGTGCCTCCCAATGCCGGAAGGTGCAACGGGAGGCTGTTCCGCTACTACTTCCACTGGGGCGTGGGACGGTGCATCCGCTACGTCTACACTGGTTGCGAGGGCACCGGAAACA |
| >AAFM24164  GGTTAGACCATTGTGACCAGTTGGAAAAGCATAATGACTGCGTTTTCGGGGTTCGTGATTGCTCTAACAGGGTTCTTCGCCACTGTGGGGTCTGAAGCAGAAGATAAATGCAATTCGAACTGGAAACAATGGTCTAACCTCAACACCGGCTACTATTATAATGAGTCAAAACATGAATGTGTCAAAACGACACGGACTGCTCATCCTGGTGTCTATCTGTTCTCTACAGAGGAAATGTGCAACTGGAGCTGTCGAGCAGGGACGGACTGCTTAATGCCGATGAAAGCCGGAAGCGAATGTGGAACCCCTCAGTTGATGTACTATTATGACAACAAAAGTTGCAATTGCATATTGTTTTTGTACAAAGGATGCGATGGAAACGGCAACAGATTTTCTTCATTGCGAGAATGTCAAGTGACTTGCAAAGGACAGCAGTGCATAAGCCCTCCACTTGACAGCGAGGTCTGTGAAAAAAAACTTACCACGTACTTCTACGACCAAACCACGGGTACCTGCAAAAAGCGCTCTTATGGATGCAACCGAGCAGGCGCCAACTTTAAAACTGAGAAAGAGTGCCTTCGGACGTGCATTTTGCGAGCTCCCACAAAGCCCACATAATGTAACCACATCGGAAT |
| >AAFM24165  TGCAAAGGACAGCAGTGCATAACCCCTCCACATGACAGCGAGGTCTGTGGTGAAAAGCTTAACACGTACTTCTACGACAAAACCACAGGTGCCTGTAAAAACCGCCCTTATGGATGCAACCGAGAAGGTTCCAACTTTAAAACTGAGAGAGACTGCCTTCGGACTTGCATTTTGCGAGGTCCCACAAAGCCCGCATAATGTAACCACATCGGAATCACAAGAAGCCCTTTGCGTCTGCTAAAAGAATATGCAAATAAAAGTTATGCGATTTAAAAAAA |
| >AAFM24255  CTTCCAGTTTATTTTTTATTAAGTCACATATGTCACAAAGACTATTGAGCCAGAGTATCAGAACCAGTTCCTCCTTAAATCATTTTCACATGTGTTCCCAATTTTGTTTAATGCATTATCAAATAAAATGATCAAAAAGATTATCTCAAGAAAGAAAAGAATCGGCCACAAGCGCACTGAGCAAGTAAGCTAGCCATAGAAATATTTGTTTGTTACAACTCTTAAAAATTCACAGGTTGAGGCAATTAGCCTGCTATAAACTCTGTTCTCTTTTTGTGTTCTATTTTCTGCTGCCGTCTCATGCTCAAGCGCGAGGGAGAATGCCCATGGACGTCTTGCATTTGCTTTCGCAGTCGGCGTAGCTGTAGAAGTTGTTCGCATTCCCTCCACATCCTCCGTAGATGAACTGCTCGCAGGACTGGGTCGTCGCGTTGTAGTAGAAACGTGGCATGTAAGCCAAGCAACGTCCTGCCTCTGCCGGATAAGTGCACTCCAAAGGCGTTGCCCACGGCCTGAATGGAGCTTGAAATGCTTCGCTGACATCTGCTGGATCCTGTTTCCTTGAGACCCAGCATGTGTTCTCGCACTCTTCCAGAGTCTCGAAATTGTTCTCGTTGCCCCGGCAGCCGCCGTAGATGAACTTCTCGCAGGTTTTTGACCACCGGTTGTAGTAGTAGCGGGGGTAGTGCGCCCCGCATGGCCCAGGATACTTTGGCTCGAAACAGAGGGGGTTAACATTCCCTGGCTGACGCAGGCAAGTCATTTCGCACTCCTCCTTGGTCCTATATTTGTTATCGTTGTCCATGCATCCAACGTACCAAAACTGCTGGCATTTGGCAAGGGTCACGTTGTAGGTCCACAATTTCTGCAACATCCTGCAATGCCCTTCCATTGGCTTGATCAGGCACTTTCTGTAGTAGTCATTTAGAGGGTTGCACTTGGCCATGCATTCCTCTTCTGAGTTGTAGTTGTTGCGGTTTCCTTCGCAGCCACCGTATGCGAACAAATTGCAAGTCGCCGACTCCGGTTCGAAGTTATAACGATAAATAGTTTTATCGCACGGTCCCTTGTCTGCCTCCACAAAGCAGTCCAGAAATGCTTGCGGGTCCGTTTCCTTTATGTCCGGATTGCTCGTTTGCGAACCTCCTCCTCGCCTCAAACAGGTAAGTTCACATGCCTCCTTGCTGACGTAGTTGTTGTAGTTGCCCTCACAACCGGTGTACACGAATCGCTCGCACGTTCTCACCACGGCATTGAACCACCAGCTTGGAAAGTGACCTCTGCAAGGACCACGCTCAGCACGCGGCGTGCAGTATGTCTCGAAGTCGTAGTCGGCCTCCGTTTCTTCTTCGGCAAGGAGCGTGATGGTATTCGGAGCCTGGGCGGTGCACTCCAACCCGACGCCGAAAAGCGACAGAAGTACGAGCACCTTCATGCTGTCTCGA |
| >AAFM24522  TGGCAAAGATTCGAAAAATGACTACGATGGCCAGGTTACCGGCGTGCCTCTTTCTGCTCCTCCTCAGCCTGGCTCTTTCACAGGCCCGTTTCAACAGAAAAAAAGTCTGCTCCCTTCCGAAAGACGTTGGGCCCTGCAAAGCATCGATGCCCAAGTGGTGGTATAACAAAAATAAAAATTCCTGTTTTTTGTTTATCTACGGCGGCTGCCAAGGAAATGCGAATAATTTTGAACACTGCGAGGATTGCATGAGGAAATGTGGAGGACTGAACCTGCGAAAAGCAAAGTTCGTTTGCAAGAAGCTGGAGAAGCAGAATGGTGGAAATTGGAATCCTAACAAGCCAAGCAATGCAAGAAGAGCCAAGTAACCAGCGTCATCTAGTTTTTACCACCCGCTGGGAGCAAATTTTTCTCTCTGAATTATTGAAAATCCTCACACAG |
| >AAFM26077  CCGCTCTAAAATAGCGGCTTTCGATTGCTGCCATGGAAATCAAGAGATGCGTTCATGTTCTTTTAATTCTGGCCACCGTCCACGGTACACCAGTAAACAATTCGTCCAGCTGCTCTTTGCCTCAAGTAAAAGGCAACTGCAGAGGGATATTTGAAAAGTGGCACTATAACTCTACCAATGATATATGCTCACCCTTCACATATGGAGGATGCGACGGCAACCAAAACCGGTTTGATAACTGCACGCACTGCATGGAGTCATGTAGCACAAACGAAAATCGAACAGAAATATGC |
| >AAFM26078  CCGCTGTTAATTATAGCATCTTTAGACTGTGGTCATGGAAATCAAGAGATGCGTTCATGTTCTTTTAATTCTGGCCACCGTGTACGGTACATCAGTCAGCATTTCCTCCATCTGCTCTTTGCCTCAAGTAAAAGGCAACTGCAGAGGGCTATTTGACATGTGGCACTACAACTCTACCAATGATAGATGCTCACTCTTCACATATGGAGGATGCGGCGGCAACGAAAACCGATTTGACAACTGCACGCTGTGCATGGAGTCATGTAGCACAAACGAAAATCGAACAGAAATATGC |
| >AAFM27360  ATACGCTACGAGGCAAAGAGTATTTGGCTACAATCGAGTTACGCAGAGGTGTGAAGGGTACATATATTTCGGCTGCGGTGGGTATGGTAATAGTTTTGCCACACCCCAACAGTGCTGGAAGAAATGTGGAGTAAAACACAAGAGCAAGTGTGCCCAATCCAGTGGCGGAGCCTCTGGGGCTTACGGGCGATATTATTACGACATAAATTCCGACACCTGCCAGTACGGACACTACCTCACACCCGGCATCGACGGAGTCAATCGGTTCTACTATCTTGAGGACTGCGAAAGAGAATG |
| >AAFM27388  CGCCGCCAGCATGAAGTCCCTGGTCTGCCTGCTCCTCATCTCCCTTTCACTCGTGTGCCATGCGGCGCACAAGAAAGCTTGCAACCTGAAGAAGGACCCCGGCAACTGCGAGGACGCCTCCACCAAATGGTACTACGACAGCAAGACCAACGCGTGCAAGCTGTTCGTGTACGGCGGATGCGACGGCAACGACAACCGCTTCGACACAGAGGCCAAGTGCAAGGCGGAATGCGTGCATCCCCGCAAGACGACCAGCG |
| >AAFM29546  TTTTCAATCAATAAATCAGTTGTGTTCTTTTATTTCATCATGCGTATTGTGAACACGTATTCATGCATTCTTCTTGTGATTGGAAGTTGTTGCAGTTGCCACCATTATTCGACCACCCAAATAATTCGCATTGTTTGGTGTCTTTGTTGTAGAACCAACGTTGGAGTCGTATGTAGCCGGGAGTACCTATTTTTTTCGGGCAGAAACAACCTTTTCTTGGAAGTTGAGCACACAACCGGCTGTTGGGTGGAACAGCGCATCCTCGCATGACTATCTTCTTCCCCTGGACCTCTACGAGGAGCATAGCGCAGAAAAGGGCCAGGAACAACAAGCAAGTAAGGCTGCGCATAGTGGT |
| >AAFM30408  AATCATTGCAGCCAAACATCGTTCTTTTGTCTAACACTCATGCTTTATTTGTTGTTCTTCTAGAGTGATGTCAAGTTGCTGTTTCCACCTTGAAACAGCCTCTAATGAATTATAGGCACGGGCCCCAGGGTGGCGCAGACGGCGAAGCACTCGCGCATCGTCTCGAAGCTGTTGCCGTTGCCGTGGCAGCCTCCATAGAGGAACGACTTGCATTGGTTTGTCTTAGCTTCGTAGTAGAAGCGTGGGATGAAAGCCACACACGGGCCGGGGTACGGTGGGAGAAAACACAGATCAGGCTTTTTCTTCGCAGGCTCACTGAGCAACACTGTGGCTTCTTGTTTTGGCTGGAAGATAGCCAATGCGGAGCTGACAACAGCGAGGAGAATGTAAGCCTTCATGTTGCCGTACTGGTGGCGTCTGCTTT |
| >AAFM31808  GTCGACTGTTCCTTCTCAGTATGAAGGCCTACTTGTTCCTTGCTTTCATCGGCGCCGCTTGTGCGGCTACAAATGTTGACAAACAGTGCACTGCAAAAGCGGAAAAGGGACTTTGCAAGGCTAAGCTTCCAAGGTGGTGGTTCAATACGGATTCTGGCAAGTGCGAGCTCTTCTACTACGGAGGCTGCGGTGGCAACCAGAACAGATACCTCTACAAGGAGGATTGCGAAAAGACATGCGCTCCGAAAACACTGAACGAAACGCCTCTCACCACATTCAGCAACAAGAAGGCCAACTTCGATGACAAGAAGGGGCGGCTTCCCGGATCCGGCGTAGGCGTATGCATGGAGCCGCCATACACTGGGCCTTGCAAGGCGAGCTTCCTCCGATTCTACTACGACGCCAGCAGCAATACTTGCCGCCAGTTCACCTACGGCGGCTGCCGCAGCAACGGCAACAACTTTAAAGCACAACGTGATTGCATGAGGGCTTGCGGAGGTCGACGGCGCGGAGGTCTTCGGCCGCGCTAAGAAGAGATCGAAGAATAATTCGTCGTGCCATTCTTTACACTATATGTATTTTTGCGAGTAGCGCTAAAGGGAACGGGGAAGAAAAAGAATCTAATGTAACGTAGTGATTCCGTACTTCTTAATTGTTG |
| >AAFM32109  TTTTTAAATGACATATAAAAGCATTTATTTTGGCGCGCTCAGCCTGTTTTTTGGTCAAAACACGCGCGTCGCTTGCGGCAGTGCATTTCGAGCTGGCGCTTCTTTGAGCGTACGAGCCCTAATATCGGCCGCAGACTCTGCGGCATTGTCCAACAGAGTTGAATCTGTTCGCGTTGCCACCGCATCCGCCGTAGGTGAACGAGAGGCATCTTCCCGATCTACTATCGAAGTAGAAGGCTGGGAAGGCGCCGCGGCAGGGTCCCTGGCTAGGAGGAAGGAAGCACTGGCCTTGAGCATTGGTGCCTGCTACGACAATAGCCAGCAGCAAGGCGACGACCACAAGCGAACGAGGCATCTTGCGAACGGGCGGTCCGGGTGGGTGCGTTTCTCACGAGTGAGGATGCTGGGCAGCCTTGAGTCGGTCAAATATGCGCG |
| >AAFM32527  CCCGAGTGCCTGCAGCCCGGCCGACCGGTGAACAAAGGCTAAGAGCGAAGCCAGGACCGAACAGCCTACGCGGCAATGCCGGCTCCGGGTTCATACCGCCGCTGCTGTGCGCTCTGGACCACCGCGGTAGCCGCGCTCCTCACCGCATGCGCCGCCCTGGAGCCCGAGTGCCTGCAGCCCCTGGAGAAGGGCTCTTGCGGCAAGCTGCACGGCCGCTTCTTCTACAACGCCAGCGCCCGCCAGTGCCAGGAGTTCTTCTGGCTGGGCTGCGCCGAGAACGCCAACAACTTCGAGAACCGCGAGGACTGTGAGAAGGAGTGCCAGCACACGGGAACACCACTCCCAAGACCCAAGGACGAGCCGGGCCGTCGCAGCGCGGTCTGCTACCGGCGTCCGCGGCGTGGCCACTGCAAGGCCATCCACCGGCGCTGGTTCTTCAACTACCGCACGCTCATGTGCGACCGCTTCCTGTGGGGAGGCTGCGACACCAACGGCAACAACTTCCACACGCGCCGAGAGTGCCGCATGGCCTGCGGCATAGACGCCCACGCGCGGCCTGACCGTGACCTTGCCGAAGGGGGGCGCAGTAAAGCGAAAGTACTCCACTGAAAAAGTCTACGCGTGAAATACAAATAAAAGACGGCATAAGCAGGAAAA |
| >AAFM33290  CCACGTTCCGTGCCCCATTAAGAAGAGATTTTCCACCATGTATAATCGCTCCTCTTTCAGCCTACTTTTGGCCACCGCATTCGTTGCTTCTGTGGTGACCCAGCGAGCTCCAGACTACTGCAACAAGAAGCCGCAGGTTGGTCTGTGCAGAGCTCGCATTCAAAGGTGGTACTTTGATCCCTTTACCCAAACATGTAAATTTTTCTACTACGGTGGCTGTAAGGGAAACGCCAACAACTTCAAAACGAAGAAGAGGTGTCTGGAAGTCTGCCGACCGAAACGTCGGCCTGGTTCGGTTTGTCGTCTAAAGCCTGCTACAGGAATCTGCAAGGCTTACAAACCGCGCTGGTACTACGATTACAGCAGAGGGTACTGTATGGGATTCGTGTATGGAGGCTGCAAAGGAAACGACAACAGATTTGGTACCTGCTGGGAGTGCATGAGTAAATGTGGCAGAGGTCATAACATGCATCTCTGTAAGAAGCTCACACAAGAATTCGAGAGGAACTACGCAAGCGTGGGTGGAGCCTTCAACAAGCCACGCCAGCCTAAATAGCGTCTTCGTTAAGAAGCACTGAGTGGCCAGCTACATGCCGTGCATCTTCCGTGATTTGAG |
| >AAFM34222  GTTTCTTTTGCTTTCACTTTCACAGCGTGCTCCGCACCCTACGCTGTCGATGGCGTGGGCTGTGTGGCGAGTGGAGGGGCGCAGAAATCCAGGCAGTCGCGTCGGGTCTCGAAGTTGTTTCCGTTGCTGTGGCAGCCACCGTAGACGAACGGCTTGCAGGAGTTGGTCGTGGCGTCGTAGTAGAAGCGCGGTAAGAAGGCCAAGCAGTAACCAGTGTATGGGGGCCTACGGCACACGTCTTCTACGGAGGGCTTTTCATCAGCGCAAGTCTT |
| >AAFM34223  CTCAACATGAAACTCTGTGTCATCTTCGCTCTCATCTGCACCACACTTGCGGCCACCTCCTACGAGGAGAAATGCACGAAGGACCCAGAAGTTGGTTTCTGCAAAGCTTTACTGCCGAGGTGGTGGTTCAACGTGAGGACTCACCAGTGTGAGGAATTCTTCTACGGCGGCTGCGGTGGCAACGAGAACAGATACGAGACAAAGGAAGAATGCGAAAAGACTTGCGCTGATGAAAAGC |
| >AAFM34588  AGAAACGTGTCACTTGCTGACAAAACCGAATCGCAAGAAGACGCACCACAAGATCTAGTTTACGTCAAAAATTGAGGATGAAATTTCCAAGAACCCCTCTCATTTTTCTTTTGTGCCTGTTGACTAAAAGATGACAAGCCCGATCGGATGATTGCTATAAAAAAAAATATTTCGGCCGATGCCAGATCTACGAGTTTTCTAATAGATAGTATTTTGATCAGAATATACAGGCATGCCGAAAGTTCCTTTACTCAGGTTGTGGTGTGACCACAAACAATTTCCGTTCATGTGAAGAATTCCAGTTTCACTGCGACAAGGTCGCAACATGTGGAGCACTGAAGAATCTTGCTGAGCCGCCTGCTGTAACCTGCTGTTGCGCCAAAGTAACTCATTAGGGGTACGTTTTGG |
| >AAFM35295  CCTCCGCATTGCTTTTCGCAATATCCTCTTGCGATGAAATAATTATTATCCAGCAATTTCTGACTTCCTCCGCAGAAGTTGTACTTCTCGCAGGTTTGTGTCGTTATGTTGTAATAATACCTTGGTCTCCATTTTCTAGTTTTCTCGCCCTCGCAAGGACTTGGTGGAGAATTAGCGCAGAATGGAGCGCCTTGCATCTCATTGCATGTGGAGACACACTTGTAGCGAGATTCAAAGACGCCTTTCGTGCCACAGCCATTCCACCGGAAGTGTTCGCACATTCCGGACGTTGTATTGTAAAAGTACTTCAAGGTAATCTGGCGACAGTTTGGCCCGCTTTCAATATCTGGCATCACGCACTGCGTTTCGTCGCGCTCGGAGAGAACCGAAGCCACGATAGCGCATACGGTGAGGATGGAA |
| >AAFM35296  AACCTCCGCATTGCTTTTCGCAGTATCCTTTTGCGATGAAATAATTATTATCGAGCAAGTTCTGCCGTCCTCCGCAGAATTTGTACTCCTCGCAGGTTTTTGTCGTTATGTTGTAGTAATACCTTTTTCTCCCTTTTCTATCTTTCTCGTCCTCGCAAGGACTTGATGGAGGATCAGCGCAGAATGGAGCGCCTTGTTTCTCATTGCATGTGGAGACACACTTGTAGCGGGATTCAAACAAGCCTTTCGTGCCACAGCCATTCCACCGGAAGTGTTCGCACATTCCGGACGTTGTATTGTAAAAGTACCTCAAGGTGATCTGGCGACAGTTTGGCCCGCTTACAATTTTTGGCATCACGCACTGCGTTTCGTCGCGCTCGGAGAGTACCGAAGCCACGAGAGCGCATACGGTGAGGATGGAAGTTTGGAGATGCATCTTGGTTTCTTAGCGCGAGATCACTAATTGTGTCCAGCCTTCTCAAGGTGATCTGGCGACAGTTTGGCC |
| >AAFM35499  GCAAAATGTTCTTCTCTTCTGGGGCAATGTCGAACAATTTCCGATCCTCAACAAAATGAACACGTCTCTCACGTGTGTTTTGGCCTGGGTTGTTTTCGGAATTGTCGCATCACAACTCAACCTTATACGCAACAAGATTGACTGTAAATCGCCACCTGACATGCAGCTTTGTCGCCAGTATGTCCTGCAGTGGTATTTCGATCCCGACGAGTGGCACTGCAAGACATTTTACTACGGCGGGTGCCACAACAATGACAACCATTACAACACGAGGGAGGCGTGTTATCTTGCTTGCCGCATCTGGAAACCGCTCGAGCAGCTGATGAATATACTTATGTTCGGGAAAAATGAACCAAAAAACTCCGAAATGGAGCGCCAAGATTAGGTGTCACATTAAACTCAA |
| >AAFM35688  CTAAACCGGTATGCAGCCTTCCACCCGAACACAAGAAGTGTGCAAGGCTTAAAAGAAAAGGATGTCACTGCCCGAAGAATTTAGGTATTCCTGGCTTCATACGCGAAGAGCGCTGGTTCTACAACAAAAAGAAGAAACAATGCGAATCGTTCGCGTGGTCGACTAAAGGTGGAAACTGCAACAATTTCCCATCAAGTGAGAAATGCTACGAA |
| >AAFM35945  AAGACTTGCGCTGATGAAAAGCGAATCGAAAGGCCTGGAGGATTTACCGAGAGTCTTCTATATGCCTCCGTAAAAGACGTGTGCCGTAGGCCCCCATACACTGGTTACTGCTTGGCCTTCTTACCGCGCTTCTACTACGACGCCACGACCAACTCCTGCAAGCCGTTCGTCTACGGTGGCTGCCACAGCAACGGAAACAACTTCGAGACCCGACGCGACTGCCTGGATTTCTGCTCCCCTCCAGTCGCCACGCAGCCTACGCCATCGACAGCGTAAGGTCCGG |
| >AAFM36151  CAAGAGTTAGCGGTGAAGACAGCGTAAAGAAAGGATGAACTTAGCTGCAGTCTCTCTGTCCTTACTGTTGACAGGCATCATTTCTACAGATGATGTAAAAGCTGCAGGGGCTAAGCCAGAGGAGTGCGGCTATGGTTATGAAGTGTCCCCCAATTGCAAGCACGGTGGATGGGGCTACAGGTACACGCCCAGTAAGGAGCGATGTGAGCGACACTACTTTAAGTGCCAACCGAAGAAAGGCTTTTTTAGCACTATGCAAGCCTGCAAAGAGAAATGCGGAAACTTCAGTAAAGACCCGTGCACTATGCCGAAGGATCAAGGAATAAGCTGCGGTGGTTTCTTAGAGCCGTATCCTGCTTTTTGGTTCAACAGAGATTCGCAAAGGTGTGAACACTTCACGCATAATGGCTGCAATGCGAAAGGCAACACATTCAACGAAGCAGCGGAATGTTGGGACGAATGCGCACATTACGTTAAAGACGTGTGTCACCGGCCATTTTCGAGTGGCTACTTCTGCCCGAATGATACAAAATACACTACAAGGCAAAAAGTGTTTGGCTACAATCGAGTTACGAAGAGGTGTGAAG |
| >AAFM36629  GATTTTTCTTGCAAACTGTGTGAAGTATCGTCTCGTTCACAGTGCACGTCCAACCTATCGGTGGTTTGCCGGGCTCCATCGAGAGGCTGCAGCGCTCAAGTCCCGTTTTAGTTGTATTGCACTTTTCCATGCAGTGACGCATGTCTGGGAAATAGCCTGTGCTTGTCCTAACGTTCACCGGCGTGCACTCTTGCAAGGTAGCATTGTAGTAGAATCGCCGAACGGTGCCGTTATCTTTGATTGGGTCAGCCTTTCCGTCGCATTTCGGGAGGTATGCCAGCCACTTTTCATAACCCGGATATTTTCGAGTCTTAATCTTGCAGTGGTCAAGGCAGTCAGGCAGCGATGGGAAATTGTTATCGTTACCGTCACATCCCATAAAGCCAAGGAATCGGCACGTGTCGCCTTTACTGTCGTAGTAGAAATCACGCCGCCAGCACTCGGTCTTGTCGTCGTTGCAGGTCCAAAAATCGGACGCTTTGTTATCGCCCACAAGGTTTCTGACACTTNGACAGTCCAGCTTTTGTCTGGTTTTAACTCCAGAAACTGTAAATGCTGATGCCAGCAATAAAGCCACGGCGTAAAAAGCCATGGTAGCAGTTGCTTCTTCCGACGGCAAGAAAATTACCAAGCAAAACAACCGGTTTACAGTTTTCT |
| >AAFM37650  AATATAGTCCATTATTACTTTGAAACAAACGTATTTCGGTGATTCGAAGTGAATGTGCGTTTCGTTTTCGCACAGCCTTTGGCAGTCGTCTATCCTGCCGAAATTATTTTCGTTTCCACCGCAGCCGTAGAAGCGGAAAGCTTTGCAAGACGTTCCGTCGAAGTACCAGCGAATCTCAGTGGTCTCAGTTGAGTTTTTGCATGATTCGTCTCCCCCATCCATTTTCAGTTTGCAAACTTCCTTGCTTTTCGCTGTCTGGGACTCAACACACGTACCCGTGAAGATTATGAGCGACAGAACGGCAAGTTGGCGCATTTCTGAGCGAAGTTGGTTCCTG |
| >AAFM37862  CGTCGGACCATGTGAGGCCGCCATGTCCCGGTTCTTCTACAACGCCACCAGCCAGGCCTGCGAGCACTTCGTCTACGGAGGCTGTCGCGGGAACGGCAACAACTTCCGAACCGAGCAAGAATGCCAGGAAACCTGCGAACCGCACCAAGTCGTGCTGCCCAGGGCATAAGGAAAATGAGACTCATTAAGGGCAGCCGCGGGTTCTGCAAGCTCCGGGCTTCTGGAAGAAAAAAACCACAAAATATTGTACTTTTCAAAATTTTAAATGAGAGCGAGAAAGGTGACGCTGTGACACTAATCTCAACGAGAA |
| >AAFM38896  CGTCGCGTCGCACGGCTCGCCGTACGAGCCCCAGCGCTGGCAGGGGCCCAGGCCCAGGGAGTGCAGGCCCTCGGGGCACTGGCACGTGCCGTTCACGCACCGCGAGTTGGTGTCCGCAGAGGAGCATGTCTCCGAGAAGAGGCAGTGCTCGTGCAGGGTTCTGCCCTTGTAGCAGACGTCGTAGAAGCGGTCGTAGGCGGCCCTGACGTCCGGGCAAGAGCACAGGGAGGTCCTGATCTGGGAGGAGAGCCTGCAGACGGCCCCGATGGTTTGGCAC |
| >AAFM40542  TGTACAGTTGGGAAAGTTGTTATGATTTCCACCGCATCCGCCCCACAAGAAGGGGCCGCAGTACCCGAATCTGGCATCAAAGTACCATCTTGTAATTGAAGGCCGCTCGTACCCGCATTGTCCTTCCTTTCGTGGTGCATTGCAATATTTTGGGCGTCTGTACCCTTGCACTAATACGGTCAGCTGCGTCTCTTAGGTGGGTGAGCAAGCTCCGACTCATGAGGAGGATTCCTGGACCCGTCGGCTGCCGGTTGTAGATTAGCGTCTATCGTGTCT |
| >AAFM42266  CGGCAGTCTTATTGCACAATACGAAACCATCATGCGTGCTGCAACTCCAACCTTGCGGTGACTGGCCAGTCTCGCTTTCCTCGTTGCAGCGTTCAAGTCCTTTCTTAGATGTTGGGCACACTTCAAGGCAGAAATTCATGCTGGGAAAATAATGATCCCCTGTTTTAACATCCACCGCTTCGCACTCTCTCGAGGTAGAATTGTAATAGAACCGCCGAATGTCACCCGTGTCATTGAGTGGATTAGATTTCATGTGGCACCCTGGGAAACGTCTTCGGAAGAATTCAATCCAGCGTGAATCAGCGCCCTGTTTGCAGTGAGCGATGCAATCTGGGTGTGACGGAAAGTTATTACCGTTGCGACCACATCCAAGAAAGCCAAGGAAGTTGCATCTGTTCGTTGCTGGGTCATAGTAGTAGTCATGCCGGCGGCATACTGTGATTCCATTTTTTTTGTCGCACATCCAGTACCCCGGCGATGTCGTGTTCTTAGTATCGTCTTCACATTGAGGTTGTTGAATGTTTTCTCCAAAGGTTGTAATTCCTGATACCAGCAATAAAGTCGCAGCGTAAAGTAAGATCACCGCCATAGCTTTCTGTTACACCAAGAAAAT |
| >AAFM42492  CTTCCTCAGGAGCATCTGTCACAGCTGGCCGCCCGCAGGCCTGTTCACATTCCAGGTGTGAAGCAAAGTTGTTCTCATTTCCGTCGCAGCCGCCATAGTAGAAACGATGGCAGCGGCCTTCCTCAGCATTGTAGTACCACCGCTCACGGAAGTCGACACAGTTTCCAGCCTCCTGTGGCAGCACGCAGATGTTCCGCCCTCCGGGGGAGAGGCAGGCTCGCTCACAGTCACGCCTTGACTCGAACCTGTTGGCGTTGCCCTCACAGCCTCCGTAGTAGAACTGCTCGCAACGACCAGCGGCCATGTTGTAG |
| >AAFM44318  GTTCAACTCATGAGAGTGGTTACTATTATGACGACGAAAAGCATGAATGCGTGTATACAGACAAAAAGTGTATCTACGGTCACATCTTCAAAACCATGGAATTGTGCGCTTGGACATGTCAGGCAGTTATTGACTGCACTCGCAAGCCTGAAGATCTTACTCGCAAATGCCGTGAACAACCCCGGTTAATGTACTACTTTGATGGTTCAAAAAATTCGTGTGCACTTTTCCTGAATACTGGCTGTGGAGACAACGGAAATAAGTTCAACACATTACGACAGTGT |
| >AAFM45263  CAGTCGGAGATTGCGCCGTAAATATACTCAAGTACTTTTTCAACCAGACATCCCAAATGTGTGAACGCTTTTTATGGAATGGCTGCTTGAGCGATGGTGTCTTTGAAACCCGCTACGACTGCGTGCGGTACTGCCACAGGAATGAACGTGCAGGAGTATGCGCCCAGCGCCGCCCTGACAACTGTGCTGAACTAAAAGCCAAAGGCAAAAGATTGCCCCGATTCAGTATGGATGCATGGTACTACGACTTGCAGAGGAGAAGATGCATGAAGTTTCAGTTTTGTGGACCATCAGTGCCGATCATGTCTAATTACTTTACGACATTGACGGTGTGCATGATGGAATGCAG |
| >AAFM45865  TTTCAATAAGCTAACCGTTGGCGCTTTATTTCATGATAACTCTTATAGACAGGCGTCCTCGCATTCTTCTTCAGTTTCGAAGTTGTTGCAGTTACCGCCATTCGTGGACCAGGCGAACATCTCGCACTGTCGAGTCTCTTGGTCGTAGTACCAGCGTGGGTCACGAATATAGCCGGGTTTTCCTTCCTTCTTTGGGCAGGCACACACTTCTTTATTTTCTATAAGGCCTGGACATTTTTGGTCGTCGGGTCCTTTTCCGCAGTTTCGAGGACGAGAAGCTTTCTTCCTTTGGCCGTCTAC |
| >AAFM48714  GTATGCTTTAAAATCTACTTGAACAAGAATTGTGCGCATTCACTGGAATTTCATCTGAACATATACGTCCAAGTAGCGTACGCCTGCACCGTTGCCATCATGAAGAAGGGGACACATGTACTCCTAGCTGTCTTGTTTTTGGTTATGTATATAGGTGGCTCGGCTGCGGTGCCCCGCTACTGCTCGTTCCGCAGCCACCCTGGCTACTGCGAGTTCGGCATCAAGCGCTTCTACTGGGACCGGCGCTCGGGAAAGTGCCGCCAGTTCCACTACAACGGCCGCTTCGGCAACGCCAATCGCTTCGACACGCTCGCCGAGTGCCGCAGCGCCTGCGACGTCCGGAGGAGCTACGACAAGTATGTCTAGGAGAAAGGCACTGGCTTTCTAACTTTGCAAGAACTACTAATAAAGAAGGTTGACGTTCAT |
| >AAFM48894  CCTGGGACGCTCAGTACCTGACGGAGACAAGCCCAGCTGCCGAACAGTTCTCAGCGGTCTGAATCTCTCATCAGTTTTGCAGGGCCACAGTCTACCACAATGGACAGACGCACCATGACGTTCCTCGCACTGCTGGCGGCTTGTGTCACTGCCCTGGCAAGCTCGGTTTCTGCGTGCCTGGAACCAAAGTATCCAGGTCCCTGCAAAGGCTACTTCCCTCGTTACTACTTCAACAAAGACAAGGAGACCTGCCTGCAGTTCATCTACGGTGGTTGC |
| >AAFM48895  CAGTTCATCTACGGTGGTTGCCGGCCCAACGGAAACAACTTCCAAACACTCGAAGAATGCCAGAGCCGCTGTGGTTGAGCACCCAGGATGTTGAGTAGGAGCCCCTGTTTCGTATAGAGCACCTAGGTGTCCTTCTTTTGTCGACAGTAAGATGAGGNNNNNTATCATTGAAGTTTTGAACGCAAAGCCCACCGCAAAAAAACTTGTCACTTTCAACTCCAACTGAATGCTAATTTTTCTCTGATACCTCTCATTAGCAATGCATTACAGC |
| >AAFM49026  ATTACTTGCAGTATTTTTCACAATCCTCCTTCTTTTGGAAAGTGTTGCAGGTGGGGCAGCAATTTAATCCAAATCCCAGTTTGAACACCTCGCACTTTCTGGTCCTAGGATTAAAATAGTACCGGTCGAGGACCTGGCCGTAGCATGTTCCGGTGTATTTTGGCTCGGTGCAACCTTTTGGATAGGTGCACTTNAGATTGGTGCAGAAAAAGGTGGATTCTCCATATTCGACGGCAGCTAGAAGTGCCACTGCAAATAGAGGCAGTGGCACTTCTAGCTGCCGTCGAATATGGAG |
| >AAFM49936  GTTCTCAGTGCCACTCGCAGCTTTTCCCTTCGCTTGTGTAAATAACTGTAATATTTTTTCCCGCAAAAATATCCTCAGCAAGCCCTTTTGGCTACTGCGAGAACCACTACTCGTAACCAGTGTTGCAACAGTAGAACCTGCCTCCACAACTCAGATTTGAGTAATCTGCACGCTCTTTTCTAGTGTCCGCTGGAAGGCTTAATCAACCCGAAGAAGGCACCTACCTCTACCGATCCGAGATGCAAGGTATTCCTCCCAACGACGGGGAATTTACGTTGTCCAAGGTTGAAATACATCTACAACAACCAGCGTAAGACGTGTGTACCGACATGTCGGAGGATTGGCCAGTTTTCAAGCAAAGAACAGTGCGACACAATCTGCCGGAGCTCTGCTGTCTGCTATGTACCACGACCGTATGGGCTCTGCAGAGGCCCCACCTGGCCCGTCTACTACTTTAGCCAGAGCTTGCGCCGCTGTCTTGTTGAACGAGGTTGTTCCTTCCGAGGAAACAATTTTCCAAGTTTGGGAGAGTGCCAGCGCACATGTGAAAG |
| >AAFM51746  CTTTATGCATTTCCTCCACACACTTCATAGCATATGTCCTCTGATGGAAAGTTGTTGCAATTTCCCCCAACATGCGACCATGCAAATGGTTCGCACAGTTTGGTTGCATTGTCGTAAAACCACCGGAGGTCTCGAATGTAGCTTGGAGTACCCAATTCCAAAGGGCAGTCACACGCTTTTTTTTTGAAGAGTTTACATTTGGGATGGGTGGGTGGCTTTCTGCATGCTGGATTCCCTTTCCTCCTCTTGCCTTCTACGAGGAGCACAGCGCAGAAGAGGCCAAGCAACAACAGTAACAAAGCAAGATTACGCATAGTGGCGGTGTACCCGGCAGG |
| >AAFM54574  CCTGCACCAACTTGGAGGTGCGCTGGTTCTACGACAAGCAGGACGGTGTCTGCCGGGAGTTCTACTACGGTGGCTGCCTTGGCAATGGCAACCGCTTCAGGTCTCGCCGGGACTGCGAAGAGAGGTGCTTCAGTGCACAAGATCTGTGCATGCTGCCGAACGTGCAGGGCCCTTGCAGNNNNNNNNNNNNNNNNNNNNNNNNNNNNNNNNNNACAGACAGGTGCCATGAGTTCACATACGGTGGTTGCCAGGGCAATGCCAACCGCTTCAACGATCGTGAGGCCTGCGAGGCGCGGTGCCGCAAGGGCGCAC |
| >AAFM57158  CGTGTGCTCGCGGCCTGTCGACCCAGGTCCCTGTGACTTGAGTTATCCACGGTTGTGGCCATATAGGCGGCGTTTCTACTACGACCAAGAGAAGGACCAGTGCCTGCCCTTCCTCTACAGCCTGTGCCAGGGAAACGGCAACAGGTTCCACACCATCGCAATGTGCGAACGACAGTGCCGTCGAGGCTCCGGAGATCCAGAAAGACATCAAGAGTAGAAATGCACCACGAACCCATCAGTATCGTGTACTCGCAGCACCAGGCATTGCGGGTTCAT |
