# Supplementary material for: A repertoire of protease inhibitor families in Amblyomma americanum and other tick species: inter-species comparative analyses
Source: Parasit Vectors. 2017 Mar 22;10:152. doi: 10.1186/s13071-017-2080-1 (PMC5361777; doi:10.1186/s13071-017-2080-1)
Supplement: Supplementary file 3 — FASTA sequences for Amblyomma americanum contigs from Illumina sequencing, by PI family. (ZIP 638 kb) [file 13071_2017_2080_MOESM3_ESM.zip › A. americanum I21.docx]

>AAFM13415

TTTTTTTCAACAACATAAATTATTTTATTCGCTTAAACAGTGTCCGGAGCGTTCGTCCATGACAATAATACATAAATGGCGAAGAGGGAAACAGATATGACCTTCAGTTTTAAGTAGTGCACATAATATATCACAACACGATTTACAGGAGTCGCAACTGTTTCATATATTTTTAGATGAGATATAAGCACGATCTTTTCTTTTTTACTTGCGTGGCGGAACGGGAGCCTTTTTGGCTACAACAGGAAGCTTGTCGCCCTGCAGGTAAGGATTTTTCGCGGGTGTAGTCTTTGGCTGCTTCACGGATTTCTTCGATTTTCGCAAGTACCGCTTCGCGTAGGAGGCTTGATGCTCCTTCTTGAAGAACTTCTTGGCCATGACTGTCTTGTGCTGTCCTTCGACCGGCACCGTGGGGTCGATGTGCTGCACGATGCGGTCGAGCGTAGATTCCATGAGACCCTCGTTCTGGATCGATCGTGCCAGCGCGTCGATCTCGTTCTCCTGCGTGGCCCCGGGGCAGTCGAACATGTGCTCCGAGTCACACATGCAGTCCTGAGCCGCCTGGTAGTCGCGGCTGAACGCCGCCGTGTTCTCGAACTCTTCCAGGCAACCGTCCTCGGCTGTGTATCCTTTGGGACAAGGGTTGGGCGGGTTGCAGTAGGCGGGCAGCACGGCGTCGGTCTTCACTACCTGGACGTTCTTGGCGCTCCCGTCAGGTTTGAGGCGCTGAAAGCCCTCGCCGGCGCCACCGGCGACGTACTGGTGGCCCCAGAGCGAGCTGTGCTGCAGGTACTCCTGGTCCCGGATGGAGGGCCCTCCGAGGATGTCCACGTGTGCGGCCTGTCCGGCCCCCTCTTTGCGGTACCTGCCCAGGTGGCGGGCCCCGAAGCTCGGGTCTCGCTGGTAGACGTCCGCCGCCGACAGGGCTGGCTCCTGCTGCTGCTTGGCGCGCTGCTCGGCGGCGAACTCGAGCGAGCTGTCCGACAGGTCCAAGCCGGAGCCTTGGATCCTGGACATCACCTGCTTGAGGAAGCCATCTGTGAGCCCCTCAAGACCCCGGTCGTAGGAAACCGGATTGAACGTGAAGCTCCGTGTTGCCGCGTTTAGTAGCACGGCAACAAAGAAGAGTCTGC

>AAFF3827

GCAGACTCTTCTTTGTTGCCGTGCTACTAAACGCGGCAACACGGAGCTTCACGTTCAATCCGGTTTCCTACGACCGGGGTCTTGAGGGGCTCACAGATGGCTTCCTCAAGCAGGTGATGTCCAGGATCCAAGGCTCCGGCTTGGACCTGTCGGACAGCTCGCTCGAGTTCGCCGCCGAGCAGCGCGCCAAGCAGCAGCAGGAGCCAGCCCTGTCGGCGGCGGACGTCTACCAGCGAGACCCGAGCTTCGGGGCCCGCCACCTGGGCAGGTACCGCAAAGAGGGGGCCGGACAGGCCGCACACGTGGACATCCTCGGAGGGCCCTCCATCCGGGACCAGGAGTACCTGCAGCACAGCTCGCTCTGGGGCCACCAGTACGTCGCCGGTGGCGCCGGCGAGGGCTTTCAGCGCCTCAAACCTGACGGGAGCGCCAAGAACGTCCAGGTAGTGAAGACCGACGCCGTGCTGCCCGCCTACTGCAACCCGCCCAACCCTTGTCCCAAAGGATACACAGCCGAGGACGGTTGCCTGGAAGAGTTCGAGAACACGGCGGCGTTCAGCCGCGACTACCAGGCGGCTCAGGACTGCATGTGTGACTCGGAGCACATGTTCGACTGCCCCGGGGCCACGCAGGAGAACGAGATCGACGCGCTGGCACGATCGATCCAGAACGAGGGTCTCATGGAATCTACGCTCGACCGCATCGTGCAGCACATCGACCCCACGGTGCCGGTCGAAGGACAGCACAAGACAGTCATGGCCAAGAAGTTCTTCAAGAAGGAGCATCAAGCCTCCTACGCGAAGCGGTACTTGCGAAAATCGAAGAAATCCGTGAAGCAGCCAAAGACTACACCCGCGAAAAATCCTTACCTGCAGGGCGACAAGCTTCCTGTTGTAGC

>AAUM1235

GCCGGGGAAGACTCCAGCTGACAAGGCCATTTCCGACGAGCCCGCACTGGACACTAAAACTAGGCACGCCGAGTCGCTTTGACGGTATTTCCGAAGGACTTTTGGTGACATATAGTCACCATGATCAACGGCAGACTCTTCTTTGTTGCCGTGCTACTAAACGCGGCAACACGGAGCTTCACGTTCAACCCGGTTTCCTACGACCGGGGTCTTGAGGGGCTCACAGATGGCTTCCTCGAGCAGGTGATGTCCAGGATCCAAGGCTCCGGCTTGGACCTGTCGGACAGCTCGCTCGAGTTCGCCGCCGAGCAGCGCGCCAAGCAGCAGCAGGAGCCAGCCCTGTCGGCGGCGGACGTCTACCAGCGAGACCCGAGCTTCGGGGCCCGCCACCTGGGCAGGTATCGCAAAGAGGGGGCCGGACAGGCCGCACACGTAGACATCCTCGGAGGGCCCTCCATCCGGGACCAGGAGTACCTGCAGCACAGCTCGCTCTGGGGCCACCAGTACGTCGCCGGTGGCGCCGGTGAGGGATTTCAGCGCCTCAAACCTGACGGGAGCGCCAAGAACGTCCAGGTAGTGAAGACCGACGCCGTGCTGCCCGCCTACTGCAACCCGCCCAACCCTTGTCCCAAAGGATACACAGCCGAGGACGGTTGCCTGGAAGAGTTCGAGAACACGGCGGCGTTCAGCCGCGACTACCAGGCGGCTCAGGACTGCATGTGTGACTCGGAGCACATGTTCGACTGCCCCGGGGCCACGCAGGAGAACGAGATCGACGCGCTGGCACGATCGATCCAGAACGAGGGTCTCATGGAATCTACGCTCGACCGCATCGTGCAGCACATCGACCCCACGGTGCCGGTCGAAGGACAGCACAAGACAGTCATGGCCAAGAAGTTCTTCAAGAAGGAGCATCAAGCCTCCTACGCGAAGCGGTACTTGCGAAAATCGAAGAAATCCGTGAAGCAGCCAAAGACTACACCCGCGAAAAATCCTTACCTGCAGGGCGACAAGCTTCCTGTTGTAGC

>AAUF3468

TCCGGCTTGGACCTGTCGGACAGCTCGCTCGAGTTCGCCGCCGAGCAGCGCGCCAAGCAGCAGCAGGACCCAGCCCTGTCGGCGGCGGACGTCTACCAGCGAGACCCGAGCTTCGGGGCCCGCCACCTGGGCAGGTACCGCAAAGAGGGGGCCGGACAGGCCGCACACGTGGACATCCTCGGAGGGCCCTCCATCCGGGACCAGGAGTACCTGCAGCACAGCTCGCTCTGGGGCCACCAGTACGTCGCCGGTGGCGCCGGTGAGGGATTTCAGCGCCTCAAACCTGACGGGAGCGCCAAGAACGTCCAGGTGGTGAAGACCGACGCCGTGCTGCCCGCCTACTGCAACCCGCCCAACCCTTGTCCCAAAGGATACACAGCCGAGGACGGTTGCCTGGAAGAGTTCGAGAACACGGCGGCGTTCAGCCGCGACTACCAGGCGGCTCAGGACTGCATGTGTGACTCGGAGCACATGTTCGACTGCCCCGGGGCCACGCAGGAGAACGAGATCGACGCGCTGGCACGATCGATCCAGAGCGAGGGTCTCATGGAATCTACGCTCGACCGCATCGTGCAGCACATCGACCCCACGGTGCCGGTCGAAGGACAGCACAAGACAGTCATGGCCAAGAAGTTCTTCAAGAAGGAGCATCAAGCCTCCTACGCGAAGCGGTACTTGCGAAAATCGAAGAAATCCGTGAAGCAGCCAAAGACTACACCCGCGAAAAATCCTTACCTGCAGGGCGACAAGCTTCCTGTTGTAGCTAAAAAGGCTCCCG

>MG12028947

GCCGGTGGCGCCGGCGAGGGCTTTCAGCGCCTCAAACCTGACGGGAGCGCCAAGAACGTCCAGGTAGTGAAGACCGACGCCGTGCTGCCCGCCTACTGCAACCCGCCCAACCCTTGTCCCAAAGGATACACAGCCGAGGATGGTTGCCTGGAAGAGTTCGAGAACACGGCGGCGTTCAGCCGCGACTACCAGGCGGCTCAGGACTGCATGTGTGACTCGGAGCACATGTTCGACTGCCCCGGGGCCACGCAGGAGAACGAGATCGACGCGCTGGCACGATCGATCCAGAACGAGGGTCTCATGGAATCTACGCTCGACCGCATCGTGCAGCACATCGACCCCACGGTGCCGGTCGAAGGACAGCACAAGACAGTCATGGCCAAGAAGTTCTTCAAGAAGGAGCATCAAGCCTCCTACGCGAAGCGGTACTTGCGAAAATCGAAGAAATCCGTGAAGCAGCCAAAGACTACACCCGCGA

>MG9624863

CGTCGATCTCGTTCTCCTGCGTGGCCCCGGGGCAGTCGAACATGTGCTCCGAGTCACACATGCAGTCCTGAGCCGCCTGGTAGTCGCGGCTGAACGCCGCCGTGTTCTCGAACTCTTCCAGGCAACCATCCTCGGCTGTGTATCCTTTGGGACAAGGGTTGGGCGGGTTGCAGTAGGCGGGCAGCACGGCGTCGGTCTTCACTACCTGGACGTCCTTG

>MG4832170

CCATGACAATAATACATAAATGGCGAAGAGGGAAACAGATATGACCTTCAGTTTTAAGTAGTGCACATAATATATCACAACACGATTTACAGGAGTCGCAACTGTTTCATATATTTTTAGATGAGATATAAGCATGATCTTTTCTTTTTTACTTGCGTGGCGGAACGGGAGCCTTTTTGGCTACAACAGGAAGCTTGTCGCCCTGCAGGTAAGGATTTTTCGCGGGTGTAGTCTTTGGCTGCTTCACGGATTTCTTCGATTTTCGCAAGTACCGCTTCGCGTAGGAGGCTTGATGCTCCTTCTTGAAGAACTTCTTGGCCATGACTGTCTTGTGCTGTCCTTCGACCGGCACCGTGGGGTCGATGTGCTGCACGATGCGGTCGAGCGTAGATTCCATGAGACCCTCGTTCTGGATCGATCGTGCCAGCGCGTCGATCTCGTTCTCCTGCGTGGCCCCGGGGCAGTCGAACATGTGCTCCGAGTCANNNNNNNNNNNCTGAGCCGCCTGGTAGTCGCGGCTGAACGCCGCCGTGTTCTCGAACTCTTCCAGGCAACCGTCCTCGGCTGTGTATCCTTTGGGACAAGGGTTGGGCGGGTTGCAGTAGGCGGGCAACACGGCGTCGGTCTTCACTACCTGGACGTTCTTGGCGC

>SG967400

CTCGGCGCTTCGACCGAGGCCGGGGGAAGACTCCAGCTGACGAGGCCATTTCCGACAAGCCCGCACTGAACACTAAAATTAGGCACGCCGAGTCGCTTTGACGGTATTTCCGAAGGACTTCTGGTGACATACAGTCACCATGATCAACGGCAGACTCTTCTTTGTTGCCGTGCTACTAAACGCGGCAACACGGAGCTTCACGTTCAACCCGGTTTCCTACGACCGGGGTCTTGAGGGGCTCACAGATGGCTTCCTCAAGCAGGTGATGTCCAGGATCCAAGGCTCCGGCTTGGACCTGTCGGACAGCTCGCTCGAGTTCGCCGCCGAGCAGCGCGCCAAGCAGCAGCAGGACCCAGCCCTGTCGGCGGCGGACGTCTACCAGCGAGACCCGAGCTTCGGGGCCCGCCACCTGGGCAGGTATCGCAAAGAGGGGGCCGGACAGGCCGCACACGTAGACATCCTCGGAGGGCCCTCCATCCGGGACCAGGAGTACCTGCAGCACAGCTCGCTCTGGGGCCACCAGTACGTCGCCGGTGGCGCCGGCGAGGGCTTTCAGCGCCTCAAACCTGACGGGAGCGCCAAGAACGTCCAGGTAGTGAAGACCGACGCCGTGCTGCCCGCCTACTGCAACCCGCCCAACCCTTGTCCCAAAGGATACACAGCCGAGGATGGTTGCCTGGAAGAGTTCGAGAACACGGCGGCGTTCAGCCGCGACTACCAGGCGGCTCAGGACTGCATGTGTGACTCGGAGCACATGTTCGACTGCCCCGGGGCCACGCAGGAGAACGAGATCGACGCGCTGGCACGATCGATCCAGAACGAGGGTCTCATGGAATCTACGCTCGACCGCATCGTGCAGCACATCGACCCCACGGTGCCGGTCGAAGGACAGCACAAGACAGTCATGGCCAAGAAGTTCTTCAAGAAGGAGCATCAAGCCTCCTACGCGAAGCGGTACTTGCGAAAATCGAAGAAATCCGTGAAGCAGCCAAAGACTACACCCGCGAAAAATCCTTACCTGCAGGGCGACAAGCTTCCTGTTGTAGCCAAAAAGGCTCCCGTTCCGCCACGCAAGTAAAAAAGAAAAGATCATGCTTATATCTCATCTAAAAATATATGAAACAGTTGCGACTCCTGTAAATCGTGTTGTGATATATTATGTGCACTACTTAAAACTGAAGGTCATATCTGTTTCCCTCTTCGCCATTTATGTATTATTGTCATGGACGAACGCTCCGGACACTGTTTAAGCGAAT
