# Supplementary material for: A repertoire of protease inhibitor families in Amblyomma americanum and other tick species: inter-species comparative analyses
Source: Parasit Vectors. 2017 Mar 22;10:152. doi: 10.1186/s13071-017-2080-1 (PMC5361777; doi:10.1186/s13071-017-2080-1)
Supplement: Supplementary file 3 — FASTA sequences for Amblyomma americanum contigs from Illumina sequencing, by PI family. (ZIP 638 kb) [file 13071_2017_2080_MOESM3_ESM.zip › A. americanum I25.docx]

>AAFM850

GGGCTATATCTTCCTGCACTCGAAGGCCGCGCCACCCGTCTGGAAGGCGTCTCGTTTGCCGCACTCCAACGCCCTCGCCCTTTTCCCTCTCTCAGCTCTTGTGTTGTCTTCCGATCGCAGCCGCCGTCGGAAAAGCAGCGCTCTCGTCGACGACGCCTACAGCCGACTCACCCGCTGACGCTGACACATCGCCTCCAAACACCCAAGGTTACTTGCGACTACCAAGCCATGGCTTCCCCGATCGTGCTGGTGGCGATGCTCTTGGCGGCATCCGTGTGCTGTGGAGCTGCGGCTGGCTCCCGACTGGTAGGCGGCTGGCAAAAGAAGAACGTGGACGGCAACGACCTGTTCACAGAGCTGGCGCACTTTGCAGTCGGCCAGCAGGTCGGCGACAGAGAGTTCTTCGACACGGTGCTCGAGGTCACCGACGCAGAGACCCAGGTTGTGGCCGGCACGAACTACCGGCTCACTTTCAAGATTGCCGAATCAACGTGCCGCGTGACCGAGACGTACACCAAGGAGCTGTGCCTGCCCAAGACGCGAGACGTGAAGGACACCTGTACCGCGGTCATCTATGATGTTCCTTGGCTGAACCAGCGCTCCGTCTCCTCGTTCACTTGCAGCGCCGTTGCTGCTTCCACGTGAACACGTAGGACACGANNNNNNNNNNNNNNNNNNNNNNNNNNNNNNNNNNNNNNNNTAGAACATGAAATAAAGCTCATAGAGATTTCTTGCTCATTTCCGTAACCTCTTCAGGCGCTGTGCCTACAACTGTGGCCGCGAACTAACATATTTTTTTTTTTCACCTCTACAAGACAAAGGCGAAAGATTGCTACGAGAGTGGGGAGAGGCCTGGTGCACGTATCCCGCCTCTCACCCCGACCTGGACAGGGCGCAAGCCACGATACTCGGACGCCTACAAACAAAGGCAATTCTTAGCCCTTACCGATTATGTTCAGTTACAAACGGTGAATATCACCCTGCATGCTCGCACTGCGGGGACGGGACGCACGCCCCCCAAGACCATATATTACGGGAATGCGCTAACAAACCCCCTCCGACGACACTCTTGCCAGCTGCCTCGGCGGAGGAATGAGACACGCTCTAGGCCAGTTCAAGAAAAACAACGAAACTGGCCATCATCACGCGAGCTCAAGAGGTCGCCCTCGGCTTGCTGCCACCCTGGGCTCGCGCCTGCCCCAACTAATCCCTTCCACTTTGTGGCAATAAAGTTGTAACCACAACCNNNNNNNNNNNNNNNNNNNNNNTGGTTTTTGAGGAAAGGAAATGCCGCAGTAATTGTCTCATCTCGGTGGACACCCGAACCGCG

>AAFM1323

TGGCACTGATCATGGCAGGGCTGTGCCAAGGGCAAAAACTCATAGGAGGCTGGCTGGAGCAGGACCCATCGAGCGATCCTAAATACCTGCAACTGGCTCACTTTGCGATCGCACAGGAAACAACCGGCCTCACTTACTACCACACCGTTCTTCGGCTCCTCAAGGTTGAGACGCAGATTGTGGCTGGAGTCAACTACAAGCTGATTTTCGAGACGGCACCAACCAACTGCAAAGTCAGCGATGGTCCTTATTCAAGTGAGAGGTGTCAGCCTACATCTAACCAGGCATCAGCAGCATGCACTGCAATCATCTACGAGCGCCCTTGGGACAACTTCAAGGCTGTTACATCGTTTAGGTGCCACAAGTAAACGTGAAAAAACAATTCTTGATGTCAGTGCTTGCCAAAGTGTAATAAAATACATGAAAGTTAAATAAACATTTTGGGTGCACTGCAATCATCTACGAGC

>AAFM1418

CTGAGACTGGGCGTTCTTTTGTCCTTCCGACGCCCTCCCTTCACCCACCGAGAATAAGCTAGCTTTGTGAGCATTTTCAGGGGCTGCCTGGACGATTTACACCAGAGAGGACAGCGGAAAGTAGCATGCTTTCGACAACATTACTGATCTTTGCTGCCTGCGCCGGTGTATCCAAGGTTGAAGAAATGCAAGGAGCACCTTCGAATGTTAATCCCTACAGTGATCCGAAATACTATCATCTTGCGGAGTATGCTCTGTCAGAACGGCCTCTGAAAGTCAACTCGAGATATGTCACTGTGTTGGAACTGACAAGCGCATCAAAACAGCTGGTTGCCGGATTTATCTACCGGGTGAATTTCACTACGGCTGTGACAAGCTGCAGGCGCCGTTACAAAATGCACAAGTGCACACCGATAAGTGATAAGGCCGAGCACATTTGCCGAGTTGCATTCTGGGAAAAAGAGTGGGAAGACTTTCTTGAGATGCAAGAATTTGACTGTCAACGTGTACATGACAATGACTATTACGATTAAAGAATGTCTAAGCACGGTATTATGAACGACTCCATTGAGGCCTTGTGGATATCTTGAGGTTGAAATGAAATAAAAAGCGCTGAAAACGACAAAAAA

>AAFM1492

TATTCAGTACCAGCAAGGAGAACATTTATGCACTCCCAATATTTTTATGAATATGCGAAGTCGGTACCCAGAGAACGGTTTCTTTCATTTTGGCGTCACACAGTCGAACGCCAGAAGCTTCTTGATGTTCTGGTGCAGTGGCTCGTGAATGATTGCAGTGCACGTTTCTCTGACATAGTAATTGGCAGGCCTGCAGATGATTCCACTTCTTTGGCAGTGTCTTCTTGCAATGAGGAGTTCTATCTTGTAGTTCATGCCAGCCACAACCTGCGTGGAGACACTCTTGAGCCATAGAGCCGCGTAGTTACGTCTGAGGCCAGANNNNNNNNNNNNNNNNNNNNNNNNNNNNNNNNNNNNNNNNNNNNNNNNNNNNNNNNNNNNNNNNNNNNNAGTTTCAAGTATTTAGTATCGCCCCATGGGTCCGCCTCTGTCCACCCTCCAATTTCAACAGGTGGAAATGAAGCGGCGTTGCAGAGCGTTACAGCGGTTACTGATAGAAACGCAAGCCCTA

>AAFM1731

GTTTGTGTTTTGGGGCGGCACAGTTCCTTCGTGTACGTCTGGGTCACTGGGCACGTAGATTCGGCAGTCTTGAAAGTGATGCGGTAGTTCGTGCCTGCGATAACCTGGGTCTCTGCATCGGTGACCTCGAGCACGGTGTCGAAGAACTCTTTGCCTTGAACCTGCCTGCTGATGGCGAAATGCGCGAGCTCTTGAAATTCGTCGTTGCCAGCAACGGTCTTCTTCTGCCAGCCGCCCGGAACCCCCAGCTGCGCTTGGCAGCACAAGAAGGCACCCAAGAGGACTGACAGCAGCACCAGCGAAGAAGCCATGGCTTGGTTGTAACAGATATCCGTGGTCAGTCGGAAGCAGGAGTCGTCGCTGAGAGCGCGGTTTCTCCGGCGATGACGGCGAGAAGAGGACAACAGCAACGTTCAGCACCCCGGAGATTACGGCTGTTGTTGAAGTGCAAGCGAGACTCCTCACAGAC

>AAFM2306

TGCGGTGGCTACGGGAGATCTGCATTCGTCGGTGGGTGGCAAGAACAACAACCTTACCGCGACCCCAAATATCTCCAGCTGGCTCACTACGCCGTCTCGACACAAACGCAAAACCGGAAGATGTACGACACTGTTGTCAGACTCACCCATGTCTCCACGCAGGTCGTCGCAGGAGTGAACTACAACCTTACATTCACTACGGCACGATCAAACTGTACGATCGGGAAAGATATGTACATGGCGGAACGCTGCGTTCCATCTGGACCGGTCAACGGGCTGTGTTCGGCAATTGTATACGTTGTTCC

>AAFM3158

CTGGAGAACTACGACACTGTTCTCGAGCTCACCAGAGTGGAAACTCAGATTGTAGCGGGTGTCAACTACCGCCTAACATTCACCATCGCTGGCTCGGAATGCAAAATCGGAGAGATTGAATACAGCGAGGAGCGCTGCCCACCCAAGGAGAACGTGGCAAAGGCAACCTGCACGGCCGTTGTATACGAGAAGCCCTGGCAAAACCTCCGCTCTGTCACATCATTCACCTGCCAATGATTACAGAGAAGCCAAGGAGGTGTCGAATACATCCGAATACTCACTGCTGTTATTCTGAGAATAAAATTTCAATTGTATATTTGAAACCAAAAAAAA

>AAFM4936

CCTGAACGATTTTTTGTGCGTTTATAGCCGACATTAGAGGTTTCCACGTCAGCGTTCTCTCATTTTCTCCTCGACTGCCTTTTCTAGTAACTTATCGCGATCGTGGAGCTATCTGCAATCATTTTATTTCTCCTCAGCACAAATAAGACAACCCTCAGAAAGGAAATGTGCAACGGAGCACTTTTGTTAATCGCTGTTTACGCCATTGGGGCAGAGGCTCGTGATATAGAAATCCTTGGTGGCTATGATACGGTCAATCCTTACAGTGATCCCAAATACTACGAAATGGCAATGTTTGCTCTTCGTGAACAACCCCAAAGTGTCGACGGCACATATGTCACCGTGCTCAGGTTGACAGACGTTCAAGTGCAGCAAGTTTCAGGAACGAACTACAAGGTGGCTTTCGAAACAGCTTTGACAGACTGTGTTAACTCTTACATTCCTAATGAATGCTGGAGGGTAAATAAACAGCCCCAACAGGGTTGCACTGCGGTAATCTTTGAAGATCTGGAGGCCGAAAACTGGGAACTGACCAGCCTCGAATGCTATAGAATAGCTGGAAATGACGAAAGCTATATTGACCGTTACTGAAATTCGTCAAATTATTTTCTGTCCTTTGGAAACGGGCTACCTTCCCACGTTACAATTCTGAAATAAATGAACCGGCGTTTGAAAAAATGTAGACGAGGTGAATCCTGCAGAAGATGCCCAACTTGCTCTGAAGAATATGTGAAATGAGAAAAATGGTAGGGTACTTTGAAA

>AAFM5773

GAGCCTGGCAGTAACCCCAAATACGTCGACCTGGCCCATTATGCCATTTCCCAGCAAAGAGAAGGCCGCAAGATATACGACACGCTTGTCAACCTCACCGAAGTCTACACTCAGGTGGTGGCAGGCATTAACTACAGGCTAACCTTCACTACCGCTCCTTCGAACTGCACGATCGGCAAAGACACTTACTCTGCCCAGCGTTGTGTTCCAGTCGAAGAGGTGAACGGGAGGTGCACGGCTGTTGTCTACGAAGTCCCATGGACGAACACAACCGGACTCACCAGCTACGCGTGTTCTCCCACCGGTCAAGAACAGACATCCTGAGGCCCGGTCGACCAA

>AAFM7511

TTTTTTGCAGAGTTGGGTGAAGTAGTTTATTCAGTTCCAGCAAGGAAAATATTCATTCACTCGCCGTATTCTTATGAATATGCGAAGTCTGTACCCAGAGAACGGTTTCTTTCATTTTGGCGTTACACAGTCGAACGCCACAACGTTTTTGATGTTCTGGTGTAGTGGCTCGTGAATGACCGTAGTGCACTTTTCTGTGACGTAGTATTTCGCAGGCCTGCAGTTGGCTCCACGTTTCTTGCAGTCTCTTCTTGCAATGAGGATTTCTATCTTGTAGTTAATTCCAGCGACAACCTGCGTGGAGACACTGTTGAGCCACAGAGCCGCATAGTTACGTCTGAGGCCAGA

>AAFM9694

CCGGGTTCTAGGAGCGCAGAATGAATGGCCAGTTAGCATTTTTGGTGCCGGGAAGAAACGCTGTTCTGGCCATCCATTCCGGGTTGTAGGAGCGCAGATATTCAAAAACTAAAATCATGTTTAGCTCAGTAGGTGTGATCGCAACGCTGGCTATTGTGGCTGCTGTGTACGTGGCAGCCCTACCGGGTGGCTGGACAACCAGGGATCCCGAGTCCAGTCCAAAGTACAAGCAGCTGGCACACTACGCTGTAGCCCGGCACGTTGACGGCCTGCAGAACTACGACACTGTGCTAGAGCTCACCAAAGTGGAAACGCAGGTCGTGGCAGGTGTGAAGTACCGTCTCACCTTCACCACCGCCGCAACGGAATGCAAAATTGGTGAGATCGAATACAGCGAGGAGCGATGTCCCCCCAAGGATAACGTGCCGAAGGAAACCTGCACAGCAGTCGTCTACGANNNNNNNNNNNNNNNNNNNNNNNNNNNNNNNNNNNNNNNNNNNNNNNNNTTATTAAATTTCACCTGTGAAGTGGCTGCACTGCAGACATGTGCTTCAGATGTACACTAAGAAGAAAAAGCACATTTCCTCCAGCAAAGTCGGGCGCTCTGCAGGAAACCATGAAATAACCTGTGTGAAACCTATCTTCAAAAATAAAGTAGTGATTGCTTGCAAAAAAAA

>AAFM14894

ATTTCCGTCACACAACAAACGGCTTCCTCGAAGCCGCGCCACTGCCTGCTTCGTCTTCTTCGGGACAACTGTTTACCGTGAATTCAGTCATCCATTTAACACCCCCCTCCCCCCAAACCTCTCTGAACACCAGGCAAAACGAAACATACGGCGGCAATGAGCCACACACCTTTGCTCCCGCTCGTTCTACTAGCCGCAGCTTCATCATGTTGGGCGACTGCCGTCGGTGTTTGGCAGACACGAGGCATCAGGGATGACTACAACTACTTGTTGATGGCGCAGTTCGCCCTCTCTCAACAAAAGAACATCCGTTTCTATAACAGCGTTCTCGAGCTGAAGAATGTTCAGACAATGGAATACGTCGGGAAACACTATTCCATTGAGTTTACAATAGCTCCCACGTACTGCAACGCTCGTATAAAATATAACAGCGACGTATGCAAACCCATCATCAATAGACCAATAGGCTTGTGCAACGCAGTTGTTCACGACAAGCCCTGGGAAAATTGGCGCAACGTGACGTACTTCGCCTGCATTCCTCCTGAGGACAAGGCAAAGGTCTTCGAAGGCTTGTTAGAGAACTTGTCTCCTGCATAACACCCTACTTCCTTCATGATTCATTATCTCCACGCTGCCCAACATTCCCCGATTCATTACAATGCACATTTTGTGAAGGATATAAATTAGCAACTTAAGCGATGAGTCCTGAATTTATATACACCCACCTAATACTAGAAAATTAGCATCCTTGTAGCAATAAGAGGGTTTCATGTCGGGCTAGTTGGTTTTTAA

>AAFM17929

CGCCGTTTTATTTTACTTTTTTGCAACTGAATGAGGTAACCTTTCTTGTTTTCGTCCACGGCTGGTTGTAGATGGTCGCATTGCAGATTGTTATGGCATAGTAGTTGGCATGCTTGCACGACCGAATCGAAGTGGGAAAACCATCCTTCACGCAAGGCCTTCGTGCAATTTTGAACCGTATCCTGTAGTTGACTCCAGACACGATCTGCGTCTCCACCTTGAGTAGCCAAAGCGCTGAGTAACGACTGCTGAAGCCGGTTTTCTCCTCCTGCTGCTTAATAGCGAATTTTGTCATTTCCAAGTATTCAACGTTGTTCCAAGGGTTTTGCTGTGTCCAGCCTCCAACCATGTAGCAAGTAGCGTAGTGAAGGCCTACTGCGATTGCTGCTAGGAAAGCAAGTACTTGCACAAGCTTCATTGTACGAGACATGCGCGGAGGAGTCCTCTT

>AAFM17966

CTATTGGTGCAGGAGTGACTATAAGTAGCGTTCAGATACTTTGGCACACTAGCACGTAACGAGTAGGTGGACGAAGAAAGGCGAGGCATCGGGAATCTTGGTGCATCATGAAGATCACCTGTTCCCTGCTGGTGGTTGTGGCGGCCCTGGTCGTCGGTGCCGGCACTGGTGCGGCCGCAAGCAAGAAGGGCAGTATTACCGGAGGAATAGTCAAGCAGCCAAAGTCTCGGTTCCATTACTACCAGAGTTACGCGGACGAATTGGCTCGAGAGATGTCCATGGAAGCGAAAAATCGATTAATAGTGCTGCGCATTACGGAAGTCAAGACCCAGGTAGTGTCGGGTATAAACATCTGGATAACGATCCGGGTTGCATACTCGAACTGCAAACCGTGGACTCCGAAGCCGTTTAAGTGTAAGCCACGGCCTGGCACGCGTCATCATACCTGCAAAGCTATGATCTATGATAACTTTGGGGAAATAACATTCAAGAAGAAGAGCTGCAACATAAGGAAATGAAAA

>AAFM21355

CTTTTGTGTAATTGTCAATATCTCTCAGACTTGTTAGATGGAACAATTCTAAGCAGTCAGTAGTTTATTTACTATTTAAACAGTGGAGAGGTTTGAATACTGTGATTATAACTTCCTATGTGAAGTCTTGTTTACAAGAATAGACATTGCATTGCACCAAGCTAATAATTTTTGGCATCGGGTGCTACATAGCTAGTAGAAGGATAAAGATGAAACATTTCCCGCCCAATGAGAGTAGCCAAATTACGACGGCATTTCAGCAGGTTTTTCGACATTATTCGCAGGTGAGTGAGCTGAGGGAGCGGAAGTTCTCCCAGGGCTTCTCGTAGACGACCGCTGTACAGGTTGCCTTCGGCACGTTGGTTTTTGGTGGGCAGCGCTCCTTGCTGTAGTCAACCTCACCGATCTTGCAGTCCGAAGCAGCGATGGTGAAGGTGAGCCGGTAATTTACACCTGCTACGACCTGAGTTTCCACCTTTGTGATGTCTAGCACAGTATTGTAGTTAACTTGACCTTCGACGCGTTGAGATACAGCATAGTGGGCCAGTTCTCTGTATTTTGGGCTGGACTCGGGATCCTTGGAGGTCCATCTTCCTGGAACAGCCGCCAAGCATGCAACACCACAGATGGCCAGCATGGTGATAAGGCCTACTGGGAGAAACATTCCTTCCTTAGTCTGAGCTTCTGAGATCTGAAATAAAACGTTAAGGCCACCTTTCTGCTGGCTGCAAACCACGCTAACTGATGTCCATGGGCCGCACTATCC

>AAFM23344

CTGCTTCGTAGCCTATAGGTACATAACGCTCGTGAGGAGTCCAAGACGATTCCCTTTCACTTTCAGGTCGCTAATATAAAACATCCCTGCGAAAGATCACGTACGGGGCGACATCGCTGACAGAGGGAGTCGGTAATACTTGCCCTGGATTCTGCGAATGACGGCGAGAATTCAACATGACCGGCCTGTCGCTCGTCTTGCGGAACATCCAGATTGTCTTGCCTAAGGAAATGTACCAGCGAACACTGCTATTCCTCGCTATTTGGGGCACTGCGTCGCCGTCTGCCATTATGCATGGTTGGAGAATACATCCTAACCACAATCCCAAGTACCAGCGTTTAGCGCAGTTTGCTCTCCAGGAGCGCCCTCAACTAGCCGGCTCGAAATACGTCACGATGCTCAACGTGACAGATGTGGAAATATTGTGGTCTGAGGGATGGGACATCCAAGTGACCTTCACAACAGCTGCGACAAACTGCGGTCGCAGATATGACCCCCGCATATGTCGTCCGGTAAATTCTAAGACGCAGCACATTTGCACAGTGCGCATCTATGAAGCACCCACGAGTCATACTCCTAAGTTGACTCAGTTTAACTGCACGCGCACGAGTGGTGATAACGACGAGTACGAAGATGAAGAAGAATGAAATTCGCCAAAACATTCTCTGCTGGATCGCAAGGAGGCACACTGCCAAGTTTTCATCCTGGAGTAAATAAGCGAG

>AAFM30391

CTTTTTTCGACTTCAAGGACTCGTGTAGTTGTATGGTGCACACATCCAGCACCTTCCGAGTCAAGCAGAAAAGATTCCTATTAGTACCCGACTGTTTAAAACCACATAAAATTTTGGATATCTCCACTTCAATCTTGTAGTTGAGACCATTCACGACCTGTGTAGATGCCTTAAGAATCTTCACATTGGTGCCTTGCAGGTAGGGTCGTTGGAGATTAGCAGAGAATGCTTGCAAAGTGAGATTAAAGAATTTCATAGAACTGGAAGGGTTCTCATGGTGCGTCCAGCCGCCCAGGATAGTTGGTGCGCGACCTTGAGCAACCACCACGCTACAAAAAACAAGCGCGATTCTCAGATAGACACTCATTGTGGATTTTTCTATCCC

>AAFM48127

CTTCACACACAAGTTCCCACACTCGTCGAGTGTGTTCTTGCAAATTGTCCTCGAGGACGGCTGTGCACCGATGTGTGGGAGTTTCCCGCTGCAGCTGGCAGCTTGTGCGGCTGAATGTGTCACGTGCTGCGGTGCAGTTCGTTGGTCCAGCGACGAAGACGAACTGGTAAAGAGTTGGCTCCCCGATCCGCTCTTTAACCGTCAGCAACTTCGTCATCATGCCATAATGTGTGTCAAGAACTAGAGGGTATCTCTCGATTGCGTACTGCACCAGTTGAGGGTACTGCGGATCGCTGTTAGGGTCAGTCTCCACCCAATCAACCCAGCCATTCACTTCCACCAGAGCGACGACACCACAAATGACGGCAAAGACGATTGTAGGAGCCATGTTTGACTCAACTGTCAGCATTTCCGACGAACTCCTGGCACTGTACTTTCCTTTTTGTGTGGGCATGAGTTCAGCAAAATAAATGACGTGTGGCGCTG

>AAFM50162

GAGAGAGCAGGGAGGAAGTCCAGGACACCGAACGGCCGCATTTACGCGACGTCGAGCAAAATTTCACAAGTTGCCGACCCAGAGACTGAACGAACGTCCCTCGTGTCGCATGACCCATTTCATTTGTTGCCGCTTGTGAAACCTGTGAGAAACGCCTCCGAGTTCCGCTGAGTTCAGAGAAATCGCGCTTTTTTTTCTGACTCCGTTAATGTTCACTCAACTCAACACGGGGCTTGATAAAAGGGCCTGAATCAGCCGCTCAGAGCATTAGTGAAGACTAAGGGAGGCCAGCAGAGTAGACGAAGATCGGAATGTAGTGACTGTCGTCCGCATTGTCAAGCAAGTCAAGAGTATCCAACAAACCTGCTGGAAGAAGGTACTTCAATAAGGCCGGTATATTCATCAAGATTGGTGGCTGGGAGGAGGAGAGCTAAAGAGAGAAAAATATTTGTTGGCGCTACTTGGAATTTAGAGACCCGATTTTCAGGAATAGGTGTACAAGCAAAGATGTCAGGCATGCAGCTCTTAGCTCTCTGCGTTGTCTTCCTGCCTGGAGTTGTGCTCGGCGCCAGCCTTCCGGGCGGTTTCTACCGTAGGAGATGGGACGCCGATCCCATCTACAGCCAGATGGCGCACTTCGCCGTAGCCCAGCACACAGCGGGACACGAGTACTTCGACACGGTTCTCGAGCTCTTCGAAGTGGACGTCCAGAGCGCCGCTGGTTTCAACTACCGGCTGAGATTCACGACAGCTGAGTCCACATGCAGAGGAGCAGAGACCTACAGCCCAGACATATGTCGGCCCAAGAAAAAGCAGGCCAAGGAAGTGTGCACCGCTTTCGTCTTTTACGTTCCGTGGGTCGGGCGTCGCTCTGTCAAGTCCATGCGTTGCCAGCCGGCCAGA

>AAUF154

AGAGTGTCGTCGGAGGGGGTTTGTTAGCGCATTCCCATAATATATGGTCTTGGGGGGCGTGCGTCCCGTCCCCGCAGTGCGAGCATGCAGGGTGATATTCACCGTTTGTAACTGAACATAATCGGTAAGGGCTAAGAATTGCCTTTGTTTGTAGGCGTCCGAGTATCGTGGCTTGCGCCCTGTCCAGGTCGGGGTGAGAGGCGGGATACGTGCACCAGGCCTCTCCCCACTCTCGTAGCAATCTTTCGCCTTTGTCTTGTAGAGGTGAAAAAAAAATATGTTAGTTCGCGGCCACAGTTGTAGGCACAACGCCTGAAGAGGTTACGGAAATGAGCAAGAAATCTCTATGAGCTTTATTTCATGTTCTACTGAAATTAGGCAGTGCAAATTTGCTTTTATATCTAAGTAGAGGTCCGACACTTAAATTATTCGTGTCCTACGTGTTCACGTGGAAGCAGCAACGGCGCTGCAAGTGAACGAGGAGACGGAGCGCTGGTTCAGCCAAGGAACATCATAGATGACCGCGGTACAGGTGTCCTTCACGTCTCGCGTCTTGGGCAGGCACAGCTCCTTGGTGTACGTCTCGGTCACGCGGCACGTTGATTCGGCAATCTTGAAAGTGAGCCGGTAGTTCGTGCCGGCCACAACCTGGGTCTCTGCGTCGGTGACCTCGAGCACCGTGTCGAAGAACTCTCTGTCGCCGACCTGCTGGCCGACTGCAAAGTGCGCCAGCTCTGTGAACAGGTCGTTGCCGTCCACGTTCTTCTTTTGCCAGCCGCCTACCAGTCGGGAGCCAGCCGCAGCTCCACAGCACACGGATGCCGCCAAGAGCATCGCCACCAGCACGATCGGGGAAGCCATGGCTTGGTAGTCGCAAGTAACCTTGGGTGTT

>AAUF1871

ATCGGGGCAACTGCTCTCAACAGAGATTACTTTGACGCCGCAAGCGAAGTTTTAAAAACTTTTCGATATTAAGTCTTTATTTCTGAAGTGACGATGTGGAGGAATGAGACATCAGTTTCGGCGCCTGAGTCTCTGCAACAATGTCGCTGGTTGCAATAAGGGGTTTTCTTTAAAACAATGCATACAAATAAGTGCACAATTAGAAAAATTCTCAGATATGGTTCATCGTGATTTATTTCCAGTTTCATCGAAGAGACCTCGTCTACAAAGGTTCTTTCTCTGCTAGGTATACAGGTGCAATACGTAAATGAATTACGCTCCAGATTTTCACTTCGAAGCGGCGCCGCATGTGTACGAGGAGACGAAGCGCTCCTCCCTCAATGGAACGGTGATGACAGCGGTGCAGGTGTCCTTGACGGTTTGTGTTTTGGGGCGGCACAGTTCCTTCGTGTACGTCTGGGTCACTGGGCACGTAGATTCGGCAGTCTTGAAAGTGATGCGGTAGTTCGTGCCGGCGATAACCTGGGTCTCTGCATCGGTGACCTCGAGCACGGTGTCGAAGAACTCTTTGCCTTGAACCTGCCTGCTGATGGCGAAATGCGCGAGCTCTTGAAATTCGTCGTTGCCAGCAACGGTCTTCTTCTGCCAGCCGCCCGGAACCCCCAGCTGCGCTTGGCAGCACA

>AAUF1998

CGACGATAAAAAAGAGGGGAGCGCAGTCGTTTTCCATCAGTTGCCGCCATTGGCGCCCACGATAAAAGCAATCGACGTGTTTCGTTTCAGCTTTGAGAAGCAGCACCGACCAAGTGAAACGCAACATGGCTCGCTCAGTAAGTGTGGTAGCCGTGCTGGCCGTCTGTATCGCAGCTTGCGTGGCCAGTATTCCTGGAGGCTGGTCAGTCCAAGAACCTCAGTCCAGTCCCAAATACAAGGAGCTGGCACACTATGCCGTCGCGCAACGCATCGAAGGTCTGGAGAACTACGACACTGTTCTCGAGCTCACCAGAGTGGAAACTCAGATTGTAGCGGGTGTCAACTACCGCCTAACATTCACCATCGCTGGCTCGGAGTGCAAAATCGGAGAGATTGAATACAGCGAGGAGCGCTGCCCACCCAAGGAGAACGTGGCAAAGGCAACCTGCACGGCCGTTGTATACGAGAAGCCCTGGCAAAACCTCCGCTCTGTCACATCATTCACCTGCCAATGATTACAGAGAAGCCAAGAAGGTGTCGGATACATCCGAATACTCACTGCTGTTATTCTGCGAATAAAATTTAAACTGTATATTTGAAAA

>AAUF10826

GATCTCAGTGTTTCGTTCCTTTTCCGTACGGGGAGCATGTGTAGCTTGTCACTTGAGTGGTATTCATCCATGGAACAACGTATACAATGGCCGAACACAGGCCGTTGACCGGTCCAGATGGAACGCAGCGTTCCGCCGTGTACATATCCTTCCCGATCGTACAGTTTGATCGTGCCGTAGTGAATGTAAGGTTGTAGTTCACTCCTGCGACGACCTGCGTGGAGACATGGGTGAGTCTGACAACAGTGTCGTACATCTTCCGGTTTTGCGTTTGTGTCGAGAC

>AAUF21197

GCAGGTGTTTCTTTTTAGTGAAAGCGAGATCTGGCCGGCTGGCAACGCATGGACTTGACAGAGCGACGCCCGACCCACGGAACGTAAAAGACGAAAGCGGTGCACACTTCCTTGGCCTGCTTTTTCTTGGGCCGACATATGTCTGGGCTGTAGGTCTCTGCTCCTCTGCATGTGGACTCAGCTGTCGTGAATCTCAGCCGGTAGTTGAAACCAGCGGCGCTCTGGACGTCCACTTCGAAGAGCTCGAGAACCGTGTCGAAGTACTCGTGTCCCGCTGTGTGCTGGGCTACGGCGAAGTGCGCCATCTGGCTGTAGATGGGATCGGCGTCCCATCTCCTACGGTAGAAACCGCCCGGAAGGCTTGCGCCGAGCACAACTCCAGGCAGGAAGACAACGCAGAGAGCTAAGAGCTGCATGCCTGACATCTTTGCTTGTACACCTATTCCT

>AAUF23131

TACTTGTGGCACCTAAACGATGTAACAGCCTTGAAGTTGTCCCAAGGGCGCTCGTAGATGATTGCAGTGCATGCTGCTGATGCCTGGTTAGATGTAGGCTGACACCTCTCACTTGAATAAGGACCATCGCTGACTTTGCAGTTGGTTGGTGCCGTCTCGAAAATCAGCTTGTAGTTGACTCCAGCCACAATCTGCGTCTCAACCTTGAGGAGCCGAAGAACGGTGTGGTAGTAAGTGAGGCCGGTTGTTTCCTGTGCGATCGCAAAGTGAGCCAGTTGCAGGTATTTAGGATCGCTCGATGGGTCCTGCTCCAGCCAGCCTCCTATGAGTTTTTGCCCTTGGCACAGCCCTGCCATGATCAGTGCCAAAAAACAAAAAACACCTACAGCCCTCATTATTTTCAAGACTAACGTGCAATTGGTCTCCCGCAGAATTCAGCAATCCTATGGACGACACTCTGCTGTCTCGGGATATTTATATGTGGAAACTTGGGGCGAAACACTCAGTGGTAGGTAGGCAATAAAGCTACTGACGTACGCGGTCAGATAACG

>AAUF24799

GTTTCACACAGGTTATTTCATGGTTTCCTGCAGAGCGCCCGACTTTGCTGGAGGAAATGTGCTTTTTCTTCTTAGTGTACATCTGAAGCACATGTCTGCAGTGCAGCCACTTCACAGGTGAAATTTAATAATAAGATCAAAACCACGGCGGCACTTCTGTCCTTGATGATTTTTATTCGCAGTCGATCGAAACCAGCGTGCGGCAGTTTTCCCAGGCTTTCTCGTAGACGACTGCTGTGCAGGTTTCCTTCGGCACGTTATCCTTGGGGGGACATCGCTCCTCGCTGTATTCGATCTCACCAATTTTGCATTCCGTTGCGGCGGTGGTGAAGGTGAGACGGTACTTCACACCTGCCACGACCTGCGTTTCCACTTTGGTGAGCTCTAGCACAGTGTCGTAGTTCTGCAGGCCGTCAACGTGCCGGGCTACAGCGTAGTGTGCCAGCTGCTTGTACTTTGGACTGGACTCGGGATCCCTGGTTGTCCAGCCACCCGGTAGGGCTGCCACGTACACAGCAGCCACAATAGCCAGCGTTGCGATCACACCTACTGAGCTAAACATGATTTTAGTTTTTGAATATCTGCGCTCCTACAACCCGGAATGGATGGCCAGAACAGCGTTTCTTC

>AAUF26564

AGCGTACGGGCCCAAATCGAACCCGGCTTCAAGGACACCTGTACCAGTAAATACGTGTGTTCACCTTCACAATATGAACGTGTTGCAAAGAGCTGCAGCAATGACTACGGTGAGGCAAGCCGCCCTCGTTCTCCTCTTCGCAACGGCGACAACCGCGCTTATTTGCTGCCACAACTCCACTTCCGTCCTGATCGGTGGCTGGACCAGGCAGGAGCCTGGCAGTAACCCCAAATACGTCGACCTGGCCCATTATGCCATTTCCCAGCAAAGAGAAGGCCGCAAGATATACGACACGCTTGTCAACCTCACCGAAGTCTACACTCAGGTGGTGGCAGGCGTTAACTACAGGCTCGTCTTCACTACCGCCCTTTCGAACTGCACGATCGGCAAAGACACTTACTCTGCCCAGCGTTGTGTTCCAGTCGAAGAGGTGAACGGGAGGTGCACGGCTGTTGTCTACGAAGTCCCATGGACGAACACAACCGGACTCACCAGCTACGCGTGTTCTCCCACCGGTCAAGAACAGACATCCTGAGGCCCGGTCGACCAACGTGCAAGGTCACTGACATCTTCGATGTCCACTGCACAGAACATCGTATTTATGCGCCAACTTCCGTAACATGCAAGGCCGCAATTATTTTGTGAGTTGTGAACGAATCATGAATAAAACCTTACTGTTTGGTGCTGTAACTTTTTTAAAAC

>AAUF43090

AAAATATTTATGCACTCCCAATATTTTCAAGAATATGTAAAAGCCTGTACCCAGAGAACGGTTTATTTCATTCTGGCGTCACGCAGTCGAACGCTTTAACGTTCTTGACGTTCGTGTGTAGTGGCTCGTAAATTATCGCAGTGCACGTTTCTGTGACATAGTATTTCGCTGGCCTGCAGTTGGTTCCACGTTTCTTGCAGTCTCTTCTTGCAATGAGGATTTCTATCTTGTAGTTAAAGCCAGCCACAACCTGCGTGGAGACACTGTTGAGCCACAGAGCCGCATAGTTACGTCTGAGGCCAGATTTCTCTCTCGAGGCTGCGTACTGAGCTAGTTTCAAGTATTTAGTATCGCTCCATGGGTCTATCTCGGTCCACCCTCCAGTTTCAACAGGTGCAAATGAAGCGGCGTTGCAGAGCGTTACAGCGGTCACTGATAGAAACGCAAGCCCTACCACGGCGTTCATTTTTGCAGACACTTGTTGGAACCTCCTTTTGCGCTGAGCCAGACAAGATTTCTGCAATTGCG

>AAUM878

TTTTTTTGCAAGCAATCACTACTTTATTTTTGAAGATAGGTTTCACACAGGTTATTTCATGGTTTCCTGCAGAGCGCCCGACTTTGCTGGAGGAAATGTGCTTTTTCTTCTTAGTGTACATCTGAAGCACATGTCTGCAGTGCAGCCACTTCACAGGTGAAATTTAATAATAAGATCGAAACCACGGCGGCACTTCTGTCCTTGATGATTTTTATTCGCAGTCGATCGAAACCAGCGTGCGGCAGTTTTCCCAGGCTTTCTCGTAGACGACTGCTGTGCAGGTTTCCTTCGGCACGTTATCCTTGGGGGGACATCGCTCCTCGCTGTATTCGATCTCACCAATTTTGCATTCCGTTGCGGCGGTGGTGAAGGTGAGACGGTACTTCACACCTGCCACGACCTGCGTTTCCACTTTGGTGAGCTCTAGCACAGTGTCGTAGTTCTGCAGGCCGTCAACGTGCCGGGCTACAGCGTAGTGTGCCAGCTGCTTGTACTTTGGACTGGACTCGGGATCCCTGGTTGTCCAGCCACCCGGTAGGGCTGCCACGTACACAGCAGCCACAATAGCCAGCGTTGCGATCACACCTACTGAGCTAAACATGATTTTAGTTTTTGAATATCTGCGCTCCTACAACCCGGAATGGATGGCCAGAACAGCGTTTCTTCCCGGCACCAATAATGCTAACTGATGTGTGTGGCCTGTGCACTTCATTTTAT

>AAUM1094

CCCCACTCTCGTAGCAATCGTTCGCCTTTGTCTTGTAGAGGTGAAAAAAAAATATGTTAGTTCGCGGCCACAGTTGTAGGCACAGCGCCTGAAGAGGTTACGGAAATGAGCAAGAAATCTCTATGAGCTTTATTTCATGTTCTACTGAAATTAGGCAGTGCAAATTTGCTTTTATATCTAAGTAGAGGTCCGACACTTAAATTATTCGTGTCCTACGTGTTCACGTGGAAGCAGCAACGGCGCTGCAAGTGAACGAGGAGACGGAGCGCTGGTTCAGCCAAGGAACATCATAGATGACCGCGGTACAGGTGTCCTTCACGTCTCGCGTCTTGGGCAGGCACAGCTCCTTGGTGTACGTCTCGGTCACGCGGCACGTTGATTCGGCAATCTTGAAAGTGAGCCGGTAGTTCGTGCCGGCCACAACCTGGGTCTCTGCGTCGGTGACCTCGAGCACCGTGTCGAAGAACTCTCTGTCGCCGACCTGCTGGCCGACTGCAAAGTGCGCCAGCTCTGTGAACAGGTCGTTGCCGTCCACGTTCTTCTTTTGCCAGCCGCCTACCAGTCGGGAGCCAGCCGCAGCTCCACAGCACACGGATGCCGCCAAGAGCATCGCCACCAGCANNNNNNNNNNNNNNNNNNNNNNNNNNNNNNNNNNNNNNNNNNNNNNNNNNNNNNNNNNNNNNNNNNNNNNNNNNNNGTCGCAAGTAACCTTGGGTGTTCGGAGGCGATGTGTCAGCGTCAGCGGGTGAGTCGGCTGTAGGCGTCGTCGGCGAGAGCGCTGCTTTTCCGACGGCGGCTGAGAACGGAAGACAACACAAGAGCTCAGAGAGGAAAAAGGGCGAGGGCGTTGGAGT

>AAUM2711

GGTTGTAACAGATATCCGTGGTGAGTCTGTGAGGAGTCTCGCTTGCCCTTCAACAACAGCCGTAATCTCCGGGGTGCTGAACGTTGCTGTTGTCCTCTTCTCGCCGTCATCGCCGGAGAAACCGCGCTCTCAGCGACGACTCCTGCTTCCGACTGACCACGGNNNNNNNNNNNNNNNNNNNNNNNNNNNNNNNNNNNNNNNNNNNNNNNNNNNNNNNNNNNNNNNNNNNNNNNNNNNNNNNNNNNNNNNNTGTGCTGCCAAGCGCAGCTGGGGGTTCCGGGCGGCTGGCAGAAGAAGACCGTTGCTGGCAACGACGAATTTCAAGAGCTCGCGCATTTCGCCATCAGCAGGCAGGTTCAAGGCAAAGAGTTCTTCGACACCGTGCTCGAGGTCACCGATGCAGAGACCCAGGTTATCGCAGGCACGAACTACCGCATCACTTTCAAGACTGCCGAATCTACGTGCCCAGTGACCCAGACGTACACGAAGGAACTGTGCCGCCCCAAAACACAAACTGTCAAGGACACCTGCACCGCTGTCATCACCGTTCCATTGAGGGAGGAGCGCTTCGTCTCCTCGTACACATGCGGCNNNNNNNNNNNNNNNNNNNNNNNNNNNNNNNNNNNNNNNNNNNNNNNNNNNNNNNNNNNNNNNNNNNNTCTCTTCGATGAAACTGGAAATAAATCACGATGAACCATATCTGAGAATTTTTCTAATTGTGCACTTATTTGTATGCATTGTTTTAAAGAAAACCCCTTATTGCAACCAGCGACATTGTTGCAGAGACTCACGCGCCGAAACTGATGTCTCATTCCTCCACATCGTCACTTCAGAAATAAAGACTTAATATCGAAAAGTTTTTAAAACTTCGCTTGCGGCGT

>AAUM5958

TTGAGAAGCAGCACCGACCAAGGGAGACGCAACATGGCTCGCTCAGTAAGTGTGGTTGCCGTGCTGGCCGTCTGTATCGCAGCTTGCGTGGCCAGTATTCCTGGAGGCTGGTCAGCCCAAGAACCTCAGTCCAGTCCCAAATACAAGGAGCTGGCACACTATGCCGTCGCGCAACGCATCGAAGGCCTGGAGAACTACGACACTGTTCTCGAGCTCACCAGAGTGGAAACTCAGATTGTAGCGGGTGTCAACTACCGCCTAACATTCACCATCGCTGGCTCGGAGTGCAAAATCGGAGAGATTGAATACAGCGAGGAGCGCTGCCCACCCAAGGAGAACGTGGCAAAGGCAACCTGCACGGCCGTTGTATACGAGAAGCCCTGGCAAAACCTCCGCTCTGTCACATCATTCACCTGCCAATGATTACAGAGAAGCCAAGAAGGTGTCGGATACATCCGAATACTCACTGCTGTTATTCTGAGAATAAAATTTCAATTGTATATTTGAAAAAAA

>AAUM10870

TCGCGGTGTTGGCGGCGGTGTCGCTCTGCGGTGGCTACGGAAGATCTGCATTCGTCGGTGGGTGGCAAGAACAACAACCTTACCGCGACCCCAAATATCTCCAGCTGGCTCACTACGCCGTCTCGACACAAACGCAAAACCGGAAGATGTACGACACTGTTGTCAGACTCACCCAAGTCTCGACGCAGGTCGTCGCAGGAGTGAACTACAACCTTACATTCACTACGGCACGATCAAACTGTACGATCGGGAAAGATATGTACACGGCAGAACGCTGCGTTCCATCTGGACCGGTCAACGGGCTGTGNNNNNNNNNNNNNNNNNNNNNNNNNNNNNNATTGTATACGTTGTTCCATGGATGAATACCACTCAAGTGACAAGCTACACATGCTCCCCGTACGGCAAAGGAACGAAACACTGAGATCAAAGCAACGGCACATCACTGAAGCGATTCACATAGC

>AAUM11226

CGCTGAGTTCAGAGAAATCTCGCTTTTTTTTCTGACTCCGTTAATGTTCACTCAACTCAACACGGGGCTTGATAAAAGGGCCCGAATCAGCCGCTCAGAGCATTAGTGAAGACTAAGGGAGGCCAGCAGAGTAGACGAAGATCGGAATGTAGTGACTGTCGTCCGCATTGTCAAGCAAGTCAAGAGTATCCAACAAACCTGCTGGAAGAAGGTACTTCAATAAGGCCGGTATATTCATCAAGATTGGTGGCTGGGAGGAGGAGAGCTAAAGAGAGAAAAATATTTGTTGGCGCTACTTGGAATTTAGAGACCCGATTTTCAGGAATAGGTGTACAAGCAAAGATGTCAGGCATGCAGCTCTTAGCTCTCTGCGTTGTCTTCCTGCCTGGAGTTGTGCTCGGCGCAAGCCTTCCGGGCGGTTTCTACCGTAGGAGATGGGACGCCGATCCCATCTACAGCCAGATGGCGCACTTCGCCGTAGCCCAGCACACAGCGGGACACGAGTACTTCGACACGGTTCTCGAGCTCTTCGAAGTGGACGTCCAGAGCGCCGCTGGTTTCAACTACCGGCTGAGATTCACGACAGCTGAGTCCACATGCAGAGGAGCAGAGACCTACAGCCCAGACATATGTCGGCCCAAGAAAAAGCAGGCCAAGGAAGTGTGCACCGCTTTCGTCTTTTACGTTCCGTGGGTCGGGCGTCGCTCTGTCAAGTCCATGCGTTGCCAGCCGGCCAGATCTCGCTTTCACTAAAAAGAAACACCTGCACCCCCCCCCCCCCCCCCCCC

>AAUM18079

CCACTTTGTAGCAAGAACTCCTCGTTGCCATACATGCTGGATCGAGAAGAAAAGATCCACAATTGTGCGTTTTTCCATTTTACTACTTGCACCTGAATGATGTAACCTTCCTGGTTTTAGTCCACGGCTGATCATAGATGGTTGCATAGCAGTTTTCTACGGTATAGTATTTTGCATGCTGGCACGTCTGAAGCGAGTCTGAAAAGCCGCCTTTTATGCAGGGTCTTCGTGCGATTTTGAACCGTATCTTGTAGTTCATTCCAGACACCACCTGCGTCTGAACCTTGATGAGCCACAGCGCGGCATAACGACCACTTAACTTGATATTCTTCTGCTCCTGCTTAATCGCAAACTTCGCCAATTCCAGATATTTCACATTGCCCCACGGGTTTTGCTCCGTCCAGCCTCCAACTAAATATCCAGTTGCATAGTGCAGCCCTAAAGTAGCTGATATTAGAAAAACAAGAACGTGCACAGGCTTCATTGTTATAGAACTCGAGGCGAGCTCCTCTTAAAGAGAGCTCGATAAAGGTTATCGGG

>AAUM19672

TGGATGAGCTGCAGTGATTTCGCAGTACCCCAAAATGTTTATTTAACTTTCATGTATTTTATTACACTTTGGCAAGCACTGACATCAAGAATTGTTTTTTCACGTTTACTTGTGGCACCTAAACGATGTAACAGCCTTGAAGTTGTCCCAAGGGCGCTCGTAGATGATTGCAGTGCATGCTGCTGATGCCTGGTTAGATGTAGGCTGACACCTCTCACTTGAATAAGGACCATCGCTGACTTTGCAGTTGGTTGGTGCCGTCTCGAAAATCAGCTTGTAGTTGACTCCAGCCACAATCTGCGTCTCAACCTTGAGGAGCCGAAGAACGGTGTGGTAGTAAGTGAGGCCGGTTGTTTCCTGTGCGATCGCAAAGTGAGCCAGTTGCAGGTATTTAGGATCGCTCGATGGGTCCTGCTCCAGCCAGCCTCCTATGAGTTTTTGCCCTTGGCACAGCCCTGCCATGATCAGTGCCAAAAAACAAAAAACACCTACAGCCCTCATTATTTTCAAGACTAACGTGCAATTGGTCTCCCGCAGAATTCAGCAATCCTATGGACGACACTCTGCTGTCTCGGGATATTTATATGTGGAAACTTGTGGCGAAACACTCAGTGGTAGGTAGGCAATAAAGC

>AAUM27388

CGAAAACCTGTGTGCAATTGCTGCGACTTTTATTGACAGTAATATGAAAAAAACAATCAGTAAAAAGAAGCTTTACAGCAAAAATACATGCCCGATATATAAACGAATTCTGTTGTAGTGTTCACGCGGAGTCACACGTGAACGAAGAGATGGCACGCGGCTCTTCCAGTGGGACGTAGATGACCGCAGTGCAGATGTCTTTGACATTTCCCGTCTTGGGAAGGCACCGTTCCTTGGTGTACGGCTCGGTCACCCGGCACGTGGATTCGGCAGTCTTGAAAGTGAGCCGGTAGGTCGCACCGGTCACAACCTGAGATTCTACGGCGGTGAGCTCGAGCACCGTGTCGAAGAAATCTCTGTTGGTCTTCTGCTTGCTAATGGCGAAATGCGCTAATTCCGCGTAGAGCGGGTCTCCGTCCACGCTGTGCTTCACCCAGCCGCCAACAAGGCGCTCCTCCTCTGCTCCGCCACACGCGAAGGCAGCCATGAGGATCACCGCAAGTACGAACGAAGACGCCATGGCTTGGTTCTCACAGGCAGCCTTGGCTGTTTGGAGGCGATGCGTCAGCGCTGTCAGCCGGATTGAGTCAACGGCAGGCGAATAGTGGGCGTCCGTGCAAAGGCTGCTGCTTCTCCGACCGTGGCTGCCGATAGAAGACAACGCAGGAGCGGAGCGAGGACTGAACAGATC

>AAUM28772

TGGCTTTATTCATGATTCGTTCACAACTCACAAATTAATTGCGGCTTTGCATGTTACGGAAGTTGGCGCATAAAGACGATGTTCTGTGCGGTGGGCATTGAAGATGTCAGTGACCTTGCACGTTGGTCGACCGGGCCTCAGGATGTCTGTTCTTGACCGGTGGGAGAACACGCGTAGCTGGTGAGTCCGGTTGTGTTCGTCCATGGGACTTCGTAGACAACAGCCGTGCACCTCCCGTTCACCTCTTCGACTGGAACACAACGCAGGGCAGAGTAAGTGTCTTTGCCGATCGTGCAGTTCGAAGGAGCGGTAGTGAAGACGAGCCTGTAGTTANNGCCTGCCACCACCTGAGTGTAGACTTCGGTGAGGTTGACAAGCGTGTCGTATATCTTGCGGCCTTCTCTTTGCTGGGAAATGGCATAATGGGCCAGGTCGACGTATTTGGGGTTACTGCGAGGCTCCTGCCTGGTCCAGCCACCGACAAGGACGGAAGTGGAGTTGTGGCAGCAAATAAGCGCGGTTGTCGCCGTTGCGAAGAGGAGAACGAGGGCGGCTTGCCTCACC

>AAUM52686

CTAAATACCTGCAACTGGCTCATTTTGCGATCGCGCAGCAGGCAACTGGCCTCACTTACTACCAAACCGTTCTGCGGCTGCTCAAGGTTGAAACACAGGTTGTGGCTGGAGTGAACTACAAGCTGATATTCGTGACTGCGCCTACTAACTGCAAATTTAGCGATGGTCCCTACTCAAGTGAGAGGTGTCAGCCTACATCTAACCAGGCATCAGCAGC

>AAFF751

CACAGGCCACACACATCAGTTAGCATTTTTGGTGCCGGGAAGAAACGCTGTTCTGGCCATCCATTCCGGGTTGTAGGAGCGCAGATATTCAAAAACTAAAATCATGTTTAGCTCAGTAGGTGTGATCGCAACGCTGGCTATTGTGGCTGCTGTGTACGTGGCAGCCCTACCGGGTGGCTGGACAACCAGGGATCCCGAGTCCAGTCCAAAGTACAAGCAGCTGGCACACTACGCTGTAGCCCGGCACGTTGACGGCCTGCAGAACTACGACACTGTGCTAGAGCTCACCAAAGTGGAAACGCAGGTCGTGGCAGGTGTGAAGTACCGTCTCACCTTCACCACCGCCGCAACGGAATGCAAAATTGGTGAGATCGAATACAGCGAGGAGCGATGTCCCCCCAAGGATAACGTGCCGAAGGAAACCTGCACAGCAGTCGTCTACGAGAAAGCCTGGGAAAACTGCCGCACGCTGGTTTCGATCGACTGCGAATAAAAATCATCAAGGACAGAAGTGCCGCCGTGGTTTCGATCTTATTATTAAATTTCACCTGTGAAGTGGCTGCACTGCAGACATGTGCTTCAGATGTACACTAAGAAGAAAAAGCACATTTCCTCCAGCAAAGTCGGGCGCTCTGCAGGAAACCATGAAATAACCTGTGTGAAACCTATCTTCAAAAATAAAGTAGTGATTGCT

>AAFF1309

CTCGTCTACAAAGGTTCTTTCTCTGCTAGGTATACAGGTGCAATACGTAAATGAATTACGCTCCAGATTTTCACTTCGAAGCGGCGCCGCATGTGTACGAGGAGACGAAGCGCTCCTCCCTCAATGGAACGGTGATGACAGCGGTGCAGGTGTCCTTGACAGTTTGTGTTTTGGGGCGGCACAGTTCCTTCGTGTACGTCTGGGTCACTGGGCACGTAGATTCGGCAGTCTTGAAAGTGATGCGGTAGTTCGTGCCGGCGATAACCTGGGTCTCTGCATCGGTGACCTCGAGCACGGTGTCGAAGAACTCTTTGCCTTGAACCTGCCTGCTGATGGCGAAATGCGCGAGCTCTTGAAATTCGTCGTTGCCAGCAACGGTCTTCTTCTGCCAGCCGCCCGGAACCCCCAGCTGCGCTTGGCAGCACAAGA

>AAFF1415

NNCTGGAGAACTACGACACTGTTCTCGAGCTCACCAGAGTGGAAACTCAGATTGTAGCGGGTGTCAACTACCGCCTAACATTCACCATCGCTGGCTCGGAGTGCAAAATCGGAGAGATTGAATACAGCGAGGAGCGCTGCCCACCCAAGGAGAACGTGGCAAAGGCAACCTGCACGGCCGTTGTATACGAGAAGCCCTGGCAAAACCTCCGCTCTGTCACATCATTCACCTGCCAATGATTACAGAGAAGCCAAGGAGGTGTCGAATACATCCGAATACTCACTGCTGTTATTCTGAGAATAAAATTTCAATTGTATATTTGAAAAAAAAAAA

>AAFF2807

TGGCAAGAGTGTCGTCGGAGGGGGTTTGTTAGCGCATTCCCGTAATATATGGTCTTGGGGGGCGTGCGTCCCGTCCCCGCAGTGCGAGCATGCAGGGTGATATTCACCGTTTGTAACTGAACATAATCGGTAAGGGCTAAGAATTGCCTTTGTTTGTAGGCGTCCGAGTATCGTGGCTTGCGCCCTGTCCAGGTCGGGGTGAGAGGCGGGATACGTGCACCAGGCCTCTCCCCACTCTCGTAGCAATCTTTTGCCTTTGTCTTGTAGAGGTGAAAAAAAAAAATATGTTAGTTCGCGGCCACAGTTGTAGGCACAGCGCCTGAAGAGGTTACGGAAATGAGCAAGAAATCTCTATGAGCTTTATTTCATGTTCTACTGAAATTAGGCAGTGAAAATTTGCTTTTATATCTAAGTAGAGGTCCGACACTTAAATTATTCGTGTCCTACGTGTTCACGTGGAAGCAGCAACGGCGCTGCAAGTGAACGAGGAGACGGAGCGCTGGTTCAGCCAAGGAACATCATAGATGACCGCGGTACAGGTGTCCTTCACGTCTCGCGTCTTGGGCAGGCACAGCTCCTTGGTGTACGTCTCGGTCACGCGGCACGTTGATTCGGCAATCTTGAAAGTGAGCCGGTAGTTCGTGCCGGCCACAACCTGGGTCTCTGCGTCGGTGACCTCGAGCACCGTGTCGAAGAACTCTCTGTCGCCGACCTGCTGGCCGACTGCAAAGTGCGCCAGCTCTGTGAACAGGTCGTTGCCGTCCACGTTCTTCTTTTGCCAGCCGCCTACCAGTCGGGAGCCAGCCGCAGCTCCACAGCACACGGATGCCGCCAAGAGCATCGCCACCAGCACGATCGGGGAAGCCATGGCTTGGTAGTCGCAAGTAACCTTGGGTGTTTGGAGGCGATGTGTCAGCGTCAGCGGGTGAGTCGGCTGTAGGCGTCGTCGACGAGAGCGCTGCTTTTCCGACGGCGGCTGCGATCGGAAGACAACACAAGAGCTGAGAGAGGGAAAAGGGCGAGGGCGTTGGAGTGCGGCAAACGAGACGCCTTCCAGACGGGTGGCGCGGCCTTCGAGTGCGGGAAGATATA

>AAFF2808

NNTCCTTGGTGTACGTCTCGGTCACCCGGCACGTGGATTCGGCAGTCTTGAAAGTGAGCCGGTAGGTCGCACCGGTCACAACCTGAGATTCTACGGCGGTGAGCTCGAGCACCGTGTCGAAGAAATCTCTGTTGGTCTTCTGCTTGCTAATGGCGAAATGCGCTAATTCCGCGTAGAGCGGGTCTCCGTCCACGCTGTGCTTCACCCAGCCGCCAACAAGGCGCTCCTCCTCTGCTCCGCCACACGCGAAGGCAGCCATGAGGATCACCGCAAGTACGAACGAAGACGCCATGGCTTGGTTCTCACAGGCAGCCTTGGCTGTTTGGAGGCGATGCGTCAGCGCTGTCAGCCGGATTGAGTCAACGGCAGGCGAATAGTGGGCGTCCGTGCAAAGGCTGCTGCTTCTCCGACCGTGGCTGCCGATAGAAGACAACGCA

>AAFF4173

GCAGTGATTTCGCAGTACCCCAAAATGTTTATTTAACTTTCATGTATTTTATTACACTTTGGCAAGCACTGACATCAAGAATTGTTTTTTCACGTTTACTTGTGGCACCTAAACGATGTAACAGCCTTGAAGTTGTCCCAAGGGCGCTCGTAGATGATTGCAGTGCATGCTGCTGATGCCTGGTTAGATGTAGGCTGACACCTCTCACTTGAATAAGGACCATCGCTGACTTTGCAGTTGGTTGGTGCCGTCTCGAAAATCAGCTTGTAGTTGACTCCAGCCACAATCTGCGTCTCAACCTTGAGGAGCCGAAGAACGGTGTGGTAGTAAGTGAGGCCGGTTGTTTCCTGTGCGATCGCAAAGTGAGCCAGTTGCAGGTATTTAGGATCGCTCGATGGGTCCTGCTCCAGCCAGCCTCCTATGAGTTTTTGCCCTTGGCACAGCCCTGCCATGATCAGTGCCAAAAAACAAAAAACACCTACAGCC

>AAFF10618

TTCTCTCTTACGTTTTGAAAAAGTTACTGCACCAAACAGTAAGGCTTTATTCATGATGCGTTCACAACTCACAAAATAATTGCGGCTTTGCATGTTACGGAAGTTGGCGCATAAAGACGATGTTCTGTGCGGTGGACATCGAAGATGTCAGTGACCTTGCACTTTGGTCGACCGGGCCTCAGGATGTCTGTTCTTGACCGGTGGGAGAACACGCGTAGCTGGTGAGTCCGGTTGTGTTCGTCCATGGGACTTCGTAGACAACAGCCGTGCACCTCCCGTTCACCTCTTCGACTGGAACACAACGCTGGGCAGAGTAAGTGTCTTTGCCGATCGTGCAGTTCGAAGGAGCGGTAGTGAAGACGAGCCTGTAGTTAATGCCTGCCACCACCTGAGTGTAGACTTCGGTGAGGTTGACAAGCGTGTCGTATATCTTGCGGCCTTCTCTTTGCTGGGAAATGGCATAATGGGCCAGGTCGACGTATTTGGGGTTACTGCCAGGCTCCTGCCTGGTCCAGCCACCGACCAGGACGGAAGTGGAGTTGTGGCAGCAAATAAGCGCGGTTGTCGCCGTTGCGAAGAGAACGAGGGCGGCTTGCCTCACTGTAGTCATTGCTGCAGCTCTTTGCAACGCTTTCATATTGTGAAGGTGAACACACGTATTTACTGGTACAGGTGTCCTTGAAGCCGGGTTCGATTTGGGCCCGTACGCTGAAGCTGCCTGGAGACAAGGGGCAGAGCGCAGTTGCTGTTCTAGATCGAACGTCAAGCAGTCTAATACTACTTTCGTTAGAGGCTCTACCGTCAAACGTCTCTGCTCGCACGTGGCGCGAGTGCGGGCCCAAATCGAA

>AAFF18650

AACACGGGGCTTGATAAAAGGGCCTGAATCAGCCGCTCAGAGCATTAGTGAAGACTAAGGGAGGCCAGCAGAGTAGACGAAGATCGGAATGTAGTGACTGTCGTCCGCATTGTCAAGCAAGTCAAGAGTATCCAACAAACCTGCTGGAAGAAGGTACTTCAATAAGGCCGGTATATTCATCAAGATTGGTGGCTGGGAGGAGGAGAGCTAAAGAGAGAAAAATATTTGTTGGCGCTACTTGGAATTTAGAGACCCGATTTTCAGGAATAGGTGTACAAGCAAAGATGTCAGGCATACAGCTCTTAGCTCTCTGCGTTGTCTTCCTGCCTGGAGTTGTGCTCGGCGCAAGCCTTCCGGGCGGTTTCTACCGTAGGAGATGGGACGCCGATCCCATCTACAGCCAGATGGCGCACTTCGCCGTAGCCCAGCACACAGCGGGACACGAGTACTTCGACACGGTTCTCGAGCTCTTCGAAGTGGACGTCCAGAGCGCCGCTGGTTTCAACTACCGGCTGAGATTCACGACAGCTGAGTCCACATGCAGAGGAGCAGAGACCTACAGCCCAGACATATGTCGGCCCAAGAAAAAGCAGGCCAAGGAAGTGTGCACCGCTTTCGTCTTTTACGTTCCGTGGGTCGGGCGTCGCTCTGTCAAGTCCATGCGTTGCCAGCCGGCCAGATCTCGCTTTCACTAAAA

>AAFF27602

CAGAAGGCTGTAAAATTTAAGATAATATAAGAAGAAAACTGCTGAAAATTGATCAGCGCAACGACTCTTTGAAGTCTGTGCGCAAAAATGCTCCTTTTTTGGCTATGTGAATCGCTTCAGTGCTGTGCCGTTGCTTTGATCTCAGTGTTTCGTTCCTTTGCCGTACGGGGAGCATGTGTAGCTTGTCACTTGAGTGGTATTCATCCATGGAACAACGTATACAATTGCCGAACACAGCCCGTTGACCGGTCCAGATGGAACGCAGCGTTCCGCCGTGTACATATCCTTCCCGATCGTACAGTTTGATCGTGCCGTAGTGAATGTAAGGTTGTAGTTCACTCCTGCGACGACCTGCGTCGAGACTTGGGTGAGTCTGACAACAGTGTCGTACATCTTCCGGTTTTGCGTTTGTGTCGAGACGGCGTAGTGAGCCAGCTGGAGATATTTGGGGTCGCGGTAAGGTTGTTGTTCTTGCCACCCACCGACGAATGCAGATCTCCCGTAGCCACCGCAGAGCGACACCGCCGCCAACACCGCGACGAGGAGCAGACAGGCTTGCTTTACGGCCGTCATTCCTGCTGGAAACTTCAGCGAAACCTGTACTTATGGAGCTCTCGTCAGCCTGGAGGTTTGTGCGAACCTGCGCGGACAGCCCGGAAAGGGGACATCTCCCGCATTCGTCGCTCGATACAGGAAGCCTGTTGACGATGGCTTCAGCGGTGCTTCGCCGGCGGTCGCTGCCCCGCTAATTTATCTTGCTTATACGTCCTTGATGGAGGCGCGGAGCTCAAACCGCCTTTCTGGCCCTTTGTTTTTCGACGCGGGCCGGCGAACGCGGCGTTGTTTTGGCGGTTCCCTTCCCCTCGACCCAGGCGCTACCCTCGACCCAGGTGTGCGAGTGTATACAAGCGGGCAGCT

>AAFF38186

AGCGCTCCTTGCTGTAGTCAACCTCACCGATCTTGCAGTCCGAAGCAGCGATGGTGAAGGTGAGCCGGTAATTTACACCTGCTACGACCTGAGTTTCCACCTTTGTGATGTCTAGCACAGTATTGTAGTTAACTTGACCTTCGACGCGTTGAGATACAGCATAGTGGGCCAGTTCTCTGTATTTTGGGCTGGACTCGGGATCCTTGGAGGTCCATCTTCCTGGAACAGCCGCCAAGCATGCAACACCACAGATGGCCAGCATGGTGATAAGGCCTACTGGGAGAAACATTCCTTCCTTAGTCTGAGCTTCTGAGATCTGAAATAAAACGTTAAGGCCACCTTTCTGCTGGCTGCAAA

>SG1202209

GCTCTGCAACGCCGCTTCATTTCCACCTGTTGAAATTGGAGGGTGGACAGAGGCGGACCCATGGGGCGATACTAAATACTTGAAACTAGCTAAGTACGCAGCCTCGCGAGAGAAGTCTGGCCTCAGACGTAACTATGCGGCTCTGTGGCTCAACAGTGTCTCCACGCAGGTTGTGGCTGGCATGAACTACAAGATAGAACTCCTCATTGCAAGAAGACACTGCCAAAGAAGTGGAATCATCTGCAGGCCTGCCAATTACTATGTCAGAGAAACGTGCACTGCAATCATTCACGAGCCCCTGCACCAGAACATCAAGAAGGTTCTGGCGTTCGACTGTGTGACGCCAAAATGAAAGAAACCGTTTTCTGGGTACAGACTTCGCATATTCATAAAAATATTGGGAGTGCATAAATGTTCTCCTTGCTGGTACTGAATAAACAACTTCACCCAACTCTAAAAAAA

>SG1203445

TTGCAGAAATCTTGTCTGGCTCAGCGCAAAAGGAGGTTCCAACAAGTGTCTGCAAAAATGAACGCCGTGGTAGGGCTTGCGTTTCTATCAGTAACCGCTGTAACGCTCTGCAACGCCGCTTCATTCGCACCTGCTGAAACTGGAAAGTGGATCGAGATAGACCCATGGAGCGATACTAAATACTTGAAACTAGCTAAGTACGCAGCCTCGCGAGAGAAATCTGGCCTCAGACGTAACTATGTGGCTCTGTGGCTCAAGAGTGTCTCCACGCAGGTTGTCGCTGGAATTAACTACAAGATAGAAATCCTCATTGCAAGAAGAGACTGCAAGAAACGTGGAGCCAACTGCAGGCCTGCGAAATACTATGTCACAGAAACATGCACTACGGTCATTCATGAGCCACTACATCAGAACATGAGGAACATTCTGGCGTTCGACTGTGTAACGCTAAAATGAAAGAAACCGTTATCTGGGTACAGACTTCGCATATTCATAAGCATATTCGGAGTGCATAAATATTTTCCTTGCTGGAACTGAATAAACAACATCAC

>SG12010407

CTGAATTCTGCGGGAGACCAATTGCACGTTAGTCTTGAAAATAATGAGGGCTGTAGGTGTTTTTTGTTTTTTGGCACTGATCATGGCAGGGCTGTGCCAAGGGCAAAAACTCATAGGAGGCTGGCTGGAGCAGGACCCATCGAGCGATCCTAAATACCTGCAACTGGCTCACTTTGCGATCGCACAGGAAACAACCGGCCTCACTTACTACCACACCGTTCTTCGGCTCCTCAAGGTTGAGACTCAGATTGTGGCTGGAGTCAACTACAAGCTGATTTTCGAGACGGCACCAACCAACTGCAAAGTCAGCGATGGTCCTTATTCAAGTGAGAGGTGTCAGCCTACATCTAACCAGGCATCAGCAGCATGCACTGCAATCATCTACGAGCGCCCTTGGGACAACTTCAAGGCTGTTACATCGTTTAGGTGCCACAAGTA

>SG12012945

CTTCCGTTCGCCGCCGCCGTCGGAAAAGCAGCGCTCTCGTCGACGACGCCTACAGCCGACTCACCCGCTGACGCTGACACATCGCCTCCAAACACCCAAGGTTACTTGTGACTACCAAGCCATGGCTTCCCCGTTCGTGCTGGTGGCGATGCTCTTGGCGGCATCCGTGTGCTGTGGAGCTGCGGCTGGCTCCCGACTGGTAGGCGGCTGGCAAAAGAAGAACGTGGACGGCAACGAGCTGTTCACAGAGCTGGCGCACTTTGCAGTCGGCCAGCAGGTCGGCGACAGAGAGTTCTTCGACACGGTGCTCGAGGTCACCGACGCAGAGACCCAGGTTGTGGCCGGCACGAACTACCGGCTCACTTTCAAGATTGCCGAATCAACGTGCCGCGTGACCGAGACGTACACCAAGGAGCTGTGCCTGCCCAAGACGCGAGACGTGAAGGACACCTGTACCGCGGTCATCTATGATGTTCCTTGGCTGAACCAGCGCTCCGTCTCCTCGTTCACTTGCAGCGCCGTTGCTGCTTCCACGTGAACACGTAGGACACGAATAATTTAAGTGTCGGACCTCTACTTAGATATAAAAGCAAATTTGCACTGCCTAATTTCAGTAGAACATGAAATAAAGATCATAGAGATTTCTTGAAAAA

>SG12014079

CTGGCGCCTGCAGGGACTCCTGCCACGATAACCTTTATCGAGCTCTCTTTAAGAGGAGCTCGCCTCGAGTTCTATAACAATGAAGCCTGTGCACGTTCTTGTTTTTCTAATATCAGCTACTTTAGGGCTGCACTATGCAACTGGATATTTAGTTGGAGGCTGGACGGAGCAAAACCCGTGGGGCAATGTGAAATATCTGGAATTGGCGAAGTTTGCGATTAAGCAGGAGCAGAAGAAAATCAAGTTAAGTGGTCGTTATGCCGCGCTGTGGCTCATCAAGGTTCAGACGCAGGTGGTGTCTGGAATGAACTACAAGATACGGTTCAAAATCGCACGAAGACCCTGCATAAAAGGCGGCTTTTCAGACTCGCTTCATACGTGCCAGCATGCAAAATACTATACCGTAGAAAACTGCTATGCAACCATCTATGATCAGCCGTGGACTAAAACCAGGAAGGTTACATCATTCAGGTGCAAGTAGTAAAATGGAAAAACGCACAATTGTGGATCTTTTCTTCTCGATCCAGCATGTCAGTCGCCGTGCCACTGAGCAACATCGACTGATATGTGGAGAAGGTGTGCGAGACCAGTCCCTGCCTCATCAAAGCAGAGGACGTGTACTATGCAGGCCATGTAGTTGAGTGCTCCGTTGAAGCGGATGAAAATCGTCGGTTCAGTTTCCTGGCGTCTGTTCTGCAAACGACCGCACTCAATAATGCCCCACACGAAGTTAAGATCACCGTTTGCAATTCGGAGGTTGTTGGAGTCAGCTGCTCCCGCAGGGCAGGGAACAACAAATGTAAGCACATTGTGGCTTCTCTCCTACATATTAACGCAGCAAGAACTTTTGACAAACTGTCGCCGACTGATCAGCCACAGAAGTGGAACAAAGCTCAGAAGGAGAAGCAGTACGAACCAAGGGCTATACTTGACCTTCCGCGCGCAAAAAGGCAAAGATCAAAGATCCTGCTCAACCTTAAGGTAGCATCTTTGAACAGCTCCTCAAGAATTTGGGCCATCAATCTGCAGAAAAAATGCATTCGGCGCGCGCCACTAAAACTGCTGTAGCAGACAATGGTGCACATCCCTACCCCTGTGAGCCTGCATCTAGAGACATCACTCCGATGGACATCTTTCACGAGTTTAGAAGGTTCCTTGGGAACTGTGACTTGCAAAAGCTGCTCGAACACTTAAACAACGCAAGAAGTTGCGAAGATATAATGCAATTGAATAGTCGACACGACACCAAGCAAAATCTAGCCTCTGGTGGCACCAAAGGGTTGGCATGATAACTGTCTCCATCGACTACTGTGTCTTCACAAGGGTTAAGACACAACGGACAAAGATGGAACCATATGACCTGAGACCTTTACTAAAGAGATCATGCGGCAAACCAACGTCTGCACACCAGCAATGCCTCATG

>SG12014216

CGCACAATGAAGCATGTGCAAGTACTTGCTTTCCTAGCAGCAATCGCAGTAGGCCTTCACTACGCTACTTGCTACATGGTTGGAGGCTGGACACAGCAAAACCCTTGGAACAACGTTGAATACTTGGAAATGACAAAATTCGCTATTAAGCAGCAGGAGGAGAAAACCGGCTTCAGCAGTCGTTACTAAGCGCTTTGGCTACTCAAGGTGGAGAAGCAGATCGTGTCTGGAGTCAACTACAGGATACGGTTCAAAATTGCACGAAGGCCTTGCGTGATGGGTGGTTTTCCCACTTCGATTCGGTCGTGCAAGCATGCAAACTACTATGCCATAACAATCT

>SG12015645

CCGCTCCTGCGTTGTCTTCTATCGGCAGCCGCGGTCGGAGAAGCAGCAGCCTTTGCACGGACGCCCACTATTCGCCTGCCGTTGACTCAATCCGGCTGACAGCGCTGACGCATCGCCTCCAAACAGCCAAGGCTGCCTGTGAGAACCAAGCCATGGCGTCTTCGTTCGTACTTGCGGTGATCCTCATGGCTGCCTTCGCGTGTGGCGGAGCAGAGGAGGAGCGCCTTGTTGGCGGCTGGGTGAAGCACAGCGTGGACGGAGACCCGCTCTACGCGGAATTGGCGCATTTCGCCATTAGCAAGCAGAAGACCAACAGAGATTTCTTCGACACGGTGCTCGAGCTCACCGCCGTAGAATCTCAGGTTGTGACCGGTGCGACCTACCGGCTCACTTTCAAGACTGCCGAATCCACGTGCCGGGTGACCGAGACGTACACCAAGGAACGGTGCCTTCCCAAGACGGGAAATGTCAAAGACATCTGCACTGCGGTCATCTACGTCCCACTGGAAGAGCCGCGTGCCATCTCTTCGTTCACGTGTGACTCCACGTGAACACTACAACAGAATTCGTTTATATATCGGGCATGTATTTTTGCTGTAAAGCTTCTTTTTACTGATTGTTTTTTCATATCACTGTCAATAAAAGTCGCAGCAATTGCGCACAGGTTTTCGTAGGGTCCCCCAATGGTCGCATTGAATTCGTTT

>SG12031848

AGATTGTTATGGCATAGTAGTTGGCATGCTTGCACGACCGAATCAAAGTGGGAAAACCACCCTTCACGCAAGGCCTTCGTGCAAGTTTGAACCATATCCTGTAGTTGACTCCAGCCACGATCTGCGTCTCCACCTTGAGTAGCCAAAGCGCTGAGTAACGACTGCTGAAGCCGGTTTTCTCCTCCTGCTGCTTAATAGCGAATTTTGTCATTTCCAAGTATT

>SG12040601

CGCGTAGCTGGTGAGTCCGGTTGTGTTCGTCCATGGGACTTCGTAGACAACAGCCGTGCACCTCCCGTTCACCTCTCCGACTGGAACACAACGCTGGGCAGAGTAAGTGTCTTTGCCGATCGTGCAGTTCGAAGGAGCGGTAGTGAAGGTTAGCCTGTAGTTAATGCCTGCCACCACCTGAGTGTAGACTTCGGTGAGGTTGACAAGCGTGTCGTATATCTTGCGGCCTTCTCTTTGCTGGGAAATGGCATAATGGGCCAGGTCGACGTATTTGTGGTTACTGCCAGGCTCCTGCCTGGTCCAGCCACCGACCAGGACGGAAGTGGAGTTGTGGCAGC

>SG96810

ACTTGTTGGAACCTCCTTTTGCACTGTGCGCGCAATTGCAGAAATCTTGTCTGGCTCAGCGCAAAAGGAGGTTCCAACAAGTGTCTGCAAAAATGAACGCCGTGGTAGGGCTTGCGTTTCTATCAGTGACCGCTGTAACGCTCTGCAACGCCGCTTCATTTGCACCTGTTGAAACTGGAGGGTGGACCGAGATAGACCCATGGAGCGATACTAAATACTTGAAACTAGCTCAGTACGCAGCCTCGAGAGAGAAATCTGGCCTCAGACGTAACTACGAGGCTCTATGGCTCAAGAGTGTCTTTACGCAGGTGGTCGCTGGAATTAACTACAAGATAGAAATCCTCATTGCAAGAAGAGACTGCAAGAAACGTGGAACCAACTGCAGGCCAGCGAAATACTATGTCACAGAAACGTGCACTGCGATAATTTACGAGCCACTACACACGAACGTCAAGAACGTTAAAGCGTTCGACTGCGTGACGCCAGAATGAAATAAACCGTTCTCTGGGTACAGGCTTTTGCATATCCTTGAAAATATTGGGAGTGCATAAATATTTTCCTTGCTGCTACTGAATAAACTTCTTCACCCAACTCTGCAAAAAAAA

>SG964345

TTTTTTGCAAGCAATCACTACTTTATTTTTGAAGATAGGTTTCACACAGGTTATTTCATGGTTTCCTGCAGAGCGCCCGACTTTGATGGAGGAAATGTGCTTTTTCTTCTTAGTGTACATCTGAAGCACATGTCTGCAGTGCAGCCACTTCACAGGTGAAATTTAATAAAGATCAAAACCACGGCGGCACTTCTGTCCTTGATGATTTTTATTCGCAGTCGATCGAAACCAGCGTGCGGCAGTTTTCCCAGGCTTTCTCGTAGACGACTGCTGTGCAGGTTTCCTTCGGCACGTTATCCTTGGGGGGACATCGCTCCTCGCTGTATTCGATCTCACCAATTTTGCATTCCGTTGCGGCGGTGGTGAAGGTGAGACGGTACTTCACACCTGCCACGACCTGCGTTTCCACTTTGGTGAGCTCTAGCACAGTGTCGTAGTTCTGCAGGCCGTCAACGTGCCGGGCTACAGCGTAGTGTGCCAGCTGCTTGTACTTTGGACTGGACTCGGGATCCCTGGTTGCCCAGCCACCCGGTAGGGCTGCCACGTACACAGCAGCCACAATAGCCAGCGTTGCGATCACACCTACTGAGCTAAACATGATTTTAGTTTTTGAATATCTGCGCTCCTACAACCCGGAATGGATGGCCAGAACAGCGTTTCTTCCCGGCAC

>SG967531

TCGTGCTGGTGGCGATGCTCTTGGCGGCATCCGTGTGCTGTGGAGCTGCGGCTGGCTCCCGACTGGTAGGCGGCTGGCAAAAGAAGAACGTGGACGGCAACGAGCTGTTCACAGAGCTGGCGCACTTTGCAGTCGGCCAGCAGGTCGGCGACAGAGAGTTCTTCGACACGGTGCTCGAGGTCACCGACGCAGAGACCCAGGTTGTGGCCGGCACGAACTACCGGCTCACTTTCAAGATTGCCGAATCAACGTGCCGCGTGACCGAGACGTACACCAAGGAGCTGTGCCTGCCCAAGACGCGAGACGTGAAGGACACCTGTACCGCGGTCATCTATGATGTTCCTTGGCTGAACCAGCGCTCCGTCTCCTCGTTCACTTGCAGCGCCGTTGCTGCTTCCACGTGAACACGTAGGACACGAATAATTTAAGTGTCGGACCTCTACTTAGATATAAAAGCAAATTTGCACTGCCTAATTTCAGTAGAACATGAAATAAAGCTCATAGAGATTTCTTGCTCATTTCCGTAACCT

>SG967558

CTGCGACTTTTATTGACAGTGATATGAAAAAACAATCAGTAAAAAGAAGCTTTACAGCAAAAATACATGCCCGATATATAAACGAATTCTGTTGTAGTGTTCACGCGGAGTCACACGTGAACGAAGAGATGGCACGCGGCTCTTCCAGTGGGACGTAGATGACCGCAGTGCAGATGTCTTTGACATTTCCCGTCTTGGGAAGGCACCGTTCCTTGGTGTACGTCTCGGTCACCCGGCACGTGGATTCGGCAGTCTTGAAAGTGAGCCGGTAGGTCGCACCGGCCACAACCTGAGATTCTACGGCGGTGAGCTCGAGCACCGTGTCGAAGAAATCTCTGTTGGTCTTCTGCTTGCTAATGGCGAAATGCGCTAATTCCGCGTAGAGCGGGTCTCCGTCCACGCTGTGCTTCACCCAGCCGCCAACAAGGCGCTCCTCCTCTGCTCCGCCACACGCGAAGGCAGCCATGAGGATCACCGCAAGTACGAACGAAGACGCCATGGCTTGGTTCTCACAGGCAGCCTTGGCTGTTTGGAGGCGATGCGTCAGCGCTGTCAGCCGGATTGAGTCAACGGCAGGCGAATAGTGGGCGTCCGTGCAAAGGCTGCTGCTTCTCCGACCGCGGCTGCCGATAGAAGACAACGCAGGAGCGGAGCGAGG

>SG968328

GTTTTGTTCCAGAAGGCTGTAAAATTTAAGAAAATATAACAAGAAAACTGCTGAAAATTGATCAGCGCAACGACTCTGAAGTCTGTGCGCAAAAATGCTCCTTTTTTGGCTATGTGAATCGCTTCAGTGCTGTGCCGTTGCTTTGATCTCAGTGTTTCTTTCCTTTGCCGTACGGGGAGCATGTGTAGCTTGTCACTTGAGTTGTATTCATCCATGGAACAACGTATACAATTGCCGAACACAGCCCGTTGACCGGTCCAGATGGAACGCAGCGTTCTGCCGTGTACATATCTTTCCCGATCGTACAGTTTGATAGTGCCGTAGTGAATGTAAGGTTGTAGTTCACTCCTGCGACGACCTGCGTGGAGACATGGGTGAGTCTGACAACAGTGTCGTACATCTTCAGGTTTTGCGTTTGTGTCGAGACGGCGTAGTGAGCCAGCTGGAGATATTTGGGGTCGCGGTAAGGTTGTTGTTCTTGCCACCCACCGACGAATGCAGATCTCCCGTAGTCACCGCAGAGCGACACCGCCGCCAACACCGCGACGAGGAGCAGACAGGCTTGCTTTACCGCCGTCATTCCTGCTGGACACTTCAGCGAAACCTGTACTTATGGAGCTCTCGTCAGCCTGGAGGTCTGTGCGAAGTTGCGCGGACAGCCCGGAAAGGGGACATCTCCCGCATTCGTCGCTCGATACAGGAAGCCTGTTGACGATGGCTTCAGCGGTGCTTCGCCGGAGGTCGCTGCCCCGCTAATTTATCTTGCTTATACGTCCTTGATGGAGGCACGGAGCTCAAACCGCCTTTCTGGCCCTTTGTTTTTCGACCCGGGCCGGCGAACGCGGCGTTGTTTTGGCGGTTCTCTTCCCCTCGACCCAGG

>SG969107

GTCGTTTTCCATTAGTTGCCGCCATTGGCGCCCACAATAAAAGCAATCGACGTGTTTCGTTTCAGCTTTGAGAAGCAGCACCGACCAAGGGAGACGCAACATGGCTCGCTCAGTAAGTGTGGTAGCCGTGCTGGCCGTCTGTATCGCAGCTTGCGTGGCCAGTATTCCTGGAGGCTGGTCAGCCCAAGAACCTCAGTCCAGTCCCAAATACAAGGAGCTGGCACACTATGCCGTCGCGCAACGCATCGAAGGCCTGGAGAACTACGACACTGTTCTCGAGCTCACCAGAGTGGAAACTCAGATTGTAGCGGGTGTCAACTACCGCCTAACATTCACCATCGCTGGCTCGGAGTGCAAAATCGGAGAGATTGAATACAGCGAGGAGCGCTGCCCACCCAAGGAGAACGTGGCAAAAGCAACCTGCACGGCCGTTGTATACGAGAAGCCCTGGCAAAACCTCCGCTCTGTCACATCATTCACCTGCCAATGATTACAGAGAAGCCAAGGAGGTGTCGAATACATCCGAATACTCACTGCTGTTATTCTGAGAATAAAATTTCAATTGTGTATTTGAAACCAAAAAAA

>SG969739

CTGAATTCTGCGGGAGACCAATTGCACGTTAGTCTTGAAAATAATGAGGGCTGTAGGTGTTTTTTGTTTTTTGGCACTGATCATGGCAGGGCTGTGCCAAGGGCAAAAACTCATAGGAGGCTGGCTGGAGCAGGACCCATCGAGCGATCCTAAATACCTGCAACTGGCTCACTTTGCGATCGCACAGGAAACAACCGGCCTCACTTACTACCACACCGTTCTTCGGCTCCTCAAGGTTGAGACTCAGATTGTGGCTGGAGTCAACTACAAGCTGATTTTCGAGACGGCACCAACCAACTGCAAAGTCAGCGATGGTCCTTATTCAAGTGAGAGGTGTCAGCCTACATCTAACCAGGCATCAGCAGCATGCACTGCAATCATCTACGAGCGCCCTTGGGACAACTTCAAGGCTGTTACATCGTTTAGGTGCCACAAGTAGACGTGAAAAAACAATTCTTGATGCCAGTGCTTGCCAAAGTGTAACAAAATACATGAAAGTCAAATAAACATTTTGGGG

>SG48397

CTTATGTGTAACCATAGCGCTGTTCTGCTACGCGAAGGGAGGCGTTAAAGGAGGCTGGAGAAATATAGACCCATGGAGCGATGTCAAAGTATTAGAAATGGCAAAGTATGCGCTACAGAATCAGAGAAATGGTTTCAGTGCTAAACACGGAGCTCTATGGCTCACAGAGGCTCAAATACAGGTTGAGACTGGAATGAAATACTACATCAGATTTGACATCGCACGAAGACCTTGCAAGAGCAGCTCTCGTTTGACCTTCAGTCGAAGATGCCAATTCCATGAAAAATATTATGTCGTATCGACATGCTTTACAACCGTCTGGGACAACGTTTTGGAGAAAAAGCGGACAGTTCTGAACTTCAGTTGCAAAACAAAACTGAATACAGGAAGACTGCTCCATGAGTAACAACCATAGCCTTTAGATTTTTTTTTTTCCTTACAGAGAAAGTATTCGACGCAACTCTCCAATG

>SG481143

TTGCAGTGCACGTTTCACTGACGTAGTATTTCGCAGGCCTGCAGTTGGTTCCACGTTTTTTGCAGTCTCTTCTTGCAATGAGGATTTCTATCTTGTAGTTAATACCAGCGACAACCTGCGTGGAGACACTGTTGAGCCACAGAGCCACATAGTTACGTCTGAGGCCAGATTTCTCTCGCGAGGCTGCGTACTGAGCTAGTTTCAAGTATTTAGTATCGCTCCATGGGTCTATCT

>SG481164

GAGAGAAATCTGGCCTCAGACGTAACTATGTGGCTCTGTGGCTCAAGAGTGTCTCCACGCAGGTTGTCGCTGGAATTAACTACAAGATAGAAATCCTCATTGCAAGAAGAGACTGCAAGAAACGTGGAGCCAACTGCAGGCCTGCGAAATACTATGTCACAGAAACATGCACTACGGTCATTCATGAGCCACTACATCAGAACATGAGGAACATTCTGGCGTTCGACTGTGTAACGCTAAAATGAAAGAAACCGTTATCTGGGTACAGACTTCGCATATTCATAAGCATATTCGGAGTGCATAAATATTTTCCTTGCTG

>SG484588

TACTTGTGGCACCTAAACGATGTAACAGCCTTGAAGTTGTCCCAAGGGCGCTCGTAGATGATTGCAGTGCATGCTGCTGATGCCTGGTTAGATGTAGGCTGACACCTCTCACTTGAATAAGGACCATCGCTGACTTTGCAGTTGGTTGGTGCCGTCTCGAAAATCAGCTTGTAGTTGACTCCAGCCACAATCTGCGTCTCAACCTTGAGGAGCCGAAGAACGGTGTGGTAGTAAGTGAGGCCGGTTGTTTCCTGTGCGATCGCAAAGTGAGCCAGTTGCAGGTATTTAGGATCGCTCGATGGGTCCTGCTCCAGCCAGCCTCCTATGAGTTTTTGCCCTTGGCACAGCCCTGCCATGATCAGTGCCAAAAAACAAAAAACACCTACAGCCCTCATTATTTTCAAGACTAACGTGCAATTGGTCTCCCGCAGAATTCAGCAATCCTATGGACGACACTCTGCTGTCTCG

>SG484923

CGTCGAGGTCTGCGAAAGAGGACTCCTCCGCGCATGTCTCGTACAATGAAGCTTGTGCAAGTACTTGCTTTCCTAGCAGCAATCGCAGTAGGCCTTCACTACGCTACTTGCTACATGGTTGGAGGCTGGACACAGCAAAACCCTTGGAACAACGTTGAATACTTGGAAATGACAAAATTCGCTATTAAGCAGCAGGAGGAGAAAACCGGCTTCAGCAGTCGTTACTAAGCGCTTTGGCTACTCAAGGTGGAGAAGCAGATCGTGTCTGGAGTCAACTACAGGATACGGTTCAAAATTGCACGAAGGCCTTGCGTGATGGGTGGTTTTCCCACTTCGATTCGGTCGTGCAAGCATGCAAACTACTATGCCATAACAATCTGCAATGCGACCATCTACAACCAGCCGTGGACGAAAACAAGAAAGGTTACCTCATTCAGTTGCAAAAAAGTAAAATAAAACGGCG

>SG487918

GCCGTCGGAAAAGCAGCGCTCTCGTCGACGACGCCTACAGCCGACTCACCCGCTGACGCTGACACATCGCCTCCAAACACCCAAGGTTACTTGTGACTACCAAGCCATGGCTTCCCCGATCGTGCTGGTGGCGATGCTCTTGGCGGCATCCGTGTGCTGTGGAGCTGCGGCTGGCTCCCGACTGGTAGGCGGCTGGCAAAAGAAGAACGTGGACGGCAACGAGCTGTTCACAGAGCTGGCGCACTTTGCAGTCGGCCAGCAGGTCGGCGACAGAGAGTTCTTCGACACGGTGCTCGAGGTCACCGACGCAGAGACCCAGGTTGTGGCCGGCACGAACTACCGGCTCACTTTCAAGATTGCCGAATCAACGTGCCGCGTGACCGAGACGTACACCAAGGAGCTGTGCCTGCCCAAGACGCGAGACGTGAAGGACACCTGTACCGCGGTCATCTATGATGTTCCTTGGCTGAACCAGCGCTCCGTCTCCTCGTTCACTTGCAGCGCCGTTGCTGCTTCCACGTGAACACGTAGGACACGAATAATTTAAGTGTCGGACCTCTACTTAGATATAAAAGCAAATTTGCACTGCCTAATTTCAGTAGAACATGAAATAAAGCTCATAGAGA

>SG488612

CAGCTCAGGTTGGTGTCATTTTTTGCTATTGTCTCCCAAATAATAAATGTACTGTACTCCGTGAATCTACAATTTGAGCCAACAGTCTACCGCAAAGAGATCATATCCAGCCCTGCGGGAGCAGCTGACTCCAACAACCTCCGAGTTGCAAACGGTGATCTTAACTTCGTGTGGGGCATTATTGAGTGCGGTCGTTTGCAGAACAGACGCCAGGAAACTGAACCGACGATTTTCATCCGCTTCAACGGAGCACTCAACTACATGGCCTGCATAGTACACGTCCTCTGCTTTGATGAGGCAGGGACTGGTCTCGCACACCTTCTCCACATATCAGTCGGTGTTGCTCGGTGGCAGGGCGACATACATGCTGGATCGAGAAGAAAAGATCCACAATTGTGCGTTTTTCCATTTTACTACTTGCACCTGAATGATGTAACCTTCCTGGTTTTAGTCCACGGCTGATCATAGATGGTTGCATAGCAGTTTTCTACGGTATAGTATTTTGCATGCTGGCACGTCTGAAGCGAGTCTGAAAAGCCGCCTTTTATGCAGGGTCTTCGTGCGATTTTGAACCGTATCTTGTAGTTCATTCCAGACACCACCTGCGTCTGAACCTTGAT

>SG4810114

TTTTTTTTCAAATACACAATTGAAATTTTATTCTCAGAATAACAGCAGTGAGTATTCGGATGTATTCGACACCTCCTTGGCTTCTCTGTAATCATTGGCAGGTGAATGATGTGACAGAGCGGAGGTTTTGCCAGGGCTTCTCGTATACAACGGCCGTGCAGGTTGCCTTTGCCACGTTCTCCTTGGGTGGGCAGCGCTCCTCGCTGTATTCAATCTCTCCGATTTTGCACTCCGAGCCAGCGATGGTGAATGTTAGGCGGTAGTTGACACCCGCTACAATCTGAGTTTCCACTCTGGTGAGCTCGAGAACAGTGTCGTAGTTCTCCAGACCTTCGATGCGTTGCGCGACGGCATAGTGTGCCAGCTCCTTGTATTTGGGACTGGACTGAGGTTCTTGGACTGACCAGCCTCCAGGAATACTGGCCACGCAAGCTGCGATACAGACGGCCAGCACGGCTACCACACTTACTGAGCGAGCCATGTTGCGTCTCACTTGGTCGGTGCTGCTTCTCAAAGCTGAAACGAAACACGTCGATTGCTTTTATTGTGGGCGCCAATGGC

>SG4819543

GCGATCACTACTTTATTTTTGAAGATAGGTTTCACACAGGTTATTTCATGGTTTCCTGCAGAGCGCCCGACTTTGATGGAGGAAATGTGCTTTTTCTTCTTAGGGTACATCTGAAGCACATGTCTGCAGTGCAGCCACTTCACAGGTGAAATTTAATAATAAGATCGAAACCACGGCGGCACTTCTGTCCTTGATGATTTTTATTCGCAGTTGATCGAAACCAGCGTGCGGCAGTTTTCCCAGGCTTTCTCGTAGACGACTGCTGTGCAGGTTTCCTTCGGCACGTTATCCTTGGGGGGACATCGCTCCTCGCTGTATTCGATCTCACCAATTTTGCATTCCGTTGCGGCGGTGGTGAAGGTGAGACGGTACTTCACACCTGCCACGACCTGCGTTTCCACTTTGGTGAGCTCTAGCACAGTGTCGTAGTTCTGCAGGCCGTCAACGTGCCGGGCTACAGCGTAGTGTGCCAGCTGCTTGTACTTTGGACTGGACTCGGGATCCCTGGTTGCCCAGCCACCCGGTAGGGCTGCCACGTACACAGCAGCCACAATAGCCAGCGTTGCGATCACACCTACTGAGCTAAACATGATTTTAGTTTTTGAATATCTGCGCTCCTACAACCCGGAATGGATGGCCAGAACAGCGTTTCTTCCCGGCACCAATAATGCTAACTGATGTGTGTG

>SG4824323

TGCGACTTTTATTGACAGTGATATGAAAAAACAATCAGTAAAAAGAAGCTTTACAGCAAAAATACATGCCGGATATATAAACGAATTCTGTTGTAGTGTTCACGCGGAGTCACACGTGAACGAAGAGATGGCACGCGGCTCTTCCAGTGGGACGTAGATGACCGCAGTGCAGATGTCTTTGACATTTCCCGTCTTGGGAAGGCACCGTTCCTTGGTGTACGTCTCGGTCACCCGGCACGTGGATTCGGCAGTCTTGAAAGTGAGCCGGTAGGTCGCACCGGTCACAACCTGAGATTCTACGGCGGTGAGCTCGAGCACCGTGTCGAAGAAATCTCTGTTGGTCTTCTGCTTGCTAATGGCGAAATGCGCTAATTCCGCGTAGAGCGGGTCTCCGTCCACGCTGTGCTTCACCCAGCCGCCAACAAGGCGCTCCTCCTCTGCTCCGCCACACGCGAAGGCAGCCATGAGGATCACCGCAAGTACGAACGAAGACGCCATGGCTTGGTTCTCACAGGCAGCCTTGGCTGTTTGGAGGCGATGCGTCAGCGCTGTCAGCCGGATTGAGTCAACGGCAGGCGAATAGTGGGCGTCCGTGCAAAGGCTGCTGCTTCTCCGACCGCGGCTGCCGATAGAAGACAACGCAGGA

>SG4831257

CACTTCCGTCCTGGTCGGTGGCTGGACCAGGCAGGAGCCTGGCAGTAACCCCAAATACGTCGACCTGGCCCATTATGCCATTTCCCAGCAAAGAGAAGGCCGCAAGATATACGACACGCTTGTCAACCTCACCGAAGTCTACACTCAGGTGGTGGCAGGCATTAACTACAGGCTAACCTTCACTACCGCTCCTTCGAACTGCACGATCGGCAAAGACACTTACTCTGCCCAGCGTTGTGTTCCAGTCGGAGAGGTGAACGGGAGGTGCACGGCTGTTGTCTACGAAGTCCCATGGACGAACACAACCGGACTCACCAGCTACGCGTGTTCTCCCACCGGTCAAGAACAGACATCCTGAGGCCCGGTCGACCAAAGTGCAAGGTCACTGACATCTTCGATGTCCACCGCACAGAACATCGTCTTTATGCGCCAACTTCCGTAACATGCAAAGCCG

>SG4840606

GAAGTGTCCAGCAGGAATGACGGCGGTAAAGCAAGCCTGTCTGCTCCTCGTCGCGGTGTTGGCGGCGGTGTCGCTCTGCGGTGGCTACGGGAGATCTGCATTCGTCGGTGGGTGGCAAGAACAACAACCTTACCGCGACCCCAAATATCTCCAGCTGGCTCACTACGCCGTCTCGACACAAACGCAAAACCTGAAGATGTACGACACTGTTGTCAGACTCACCCATGTCTCCACGCAGGTCGTCGCAGGAGTGAACTACAACCTTACATTCACTACGGCACGATCAAACTGTACGATCGGGAAAGATATGTACACGGCAG

>MG48125

TATAACAGAGGAGTTTCTCGTTCCTCTCGAGTCTTTTTCTTCGTAGCTGCCAGCTCTGATCAATCATGCCACTTTGTGGAGGACTCGCCGAGGAAGTTAAGGACGCCGATGCCACCGTCCAGGAGATCTGCGAAAAGGTCCGCTCCGAAGTGGAGGCGAAGCTCGCGAAGAAATTCGACGAGTTCACTCCGCTGAAGTATCGCACGCAGCTTGTGAACGGCGTAAACTATTTCGTCAAGGTCCACGTCGGAGGCGGCCAGCACATCCACGTGCGTGCGCACAAGGCCTTCCAGGGCGAGATATCGTTCTCTGCTGTGCAGGAGAACAAGGCGTTGGAGGACCCAATCGAGCATTTCCAGTGACGGCTGCGGCAAGACTTGAAACGTGGATTTGTACTGATGTAATAAAGTTTACTTCTTCCTGCAAAAAAAAAA

>MG48371

TCATTGGCAGGTGAATGATGGGTTTCAAATACACAATTGAAATTTTATTCTCAGAATAACAGCAGTGAGTATTCGGATGTATTCGACACCTCCTTGGCTTCTCTGTAATCATTGGCAGGTGAATGATGTGACAGAGCGGAGGTTTTGCCAGGGCTTCTCGTATACAACGGCCGTGCAGGTTGCCTTTGCCACGTTCTCCTTGGGTGGGCAGCGCTCCTCGCTGTATTCAATCTCTCCGATTTTGCACTCCGAGCCGGCGATGGTGAATGTTAGGCGGTAGTTGACACCCGCTACAATCTGAGTTTCCACTCTGGTGAGCTCGAGAACAGTGTCGTAGTTCTCCAG

>MG48606

TGTCAGCCGGATTGAGTCCTCGCTCCGCTCCTGCGTTGTCTTCTATCGGCAGCCGCGGTCGGAGAAGCAGCAGCCTTTGCACGGACGCCCACTATTCGCCTGCCGTTGACTCAATCCGGCTGACAGCGCTGACGCATCGCCTCCAAACAGCCAAGGCTGCCTGTGAGAACCAAGCCATGGCGTCTTCGTTCGTACTTGCGGTGATCCTCATGGCTGCCTTCGCGTGTGGCGGAGCAGAGGAGGAGCGCCTTGTTGGCGGCTGGGTGAAGCACAGCGTGGACGGAGACCCGCTCTACGCGGAATTAGCGCATTTCGCCATTAGCAAGCAGAAGACCAACAGAGATTTCTTCGACACGGTGCTCGAGCTCACCGCCGTAGAATCTCAGGTTGTGGCCGGTGCGACCTACCGGCTCACTTTCAAGACTGCCGAATCCACGTGCCGGGTGACCGAGACGTACACCAAGGNNNNNNNNNNNTGACCGAGACGTACACCAAGGAACGGTGCCTTCCCAAGACGGGAAATGTCAAAGACATCTGCACTGCGGTCATCTACGTCCCACTGGAAGAGCCGCGTGCCATCTCTTCGTTCGCGTGTGACTCCGCGTGAACACTACCACAGAATTCGTTTATATATCGGGCATGTATTTTTG

>MG48647

TTTCGACGTACCCCAAAATGTTTATTTGACTTTCATGTATTTTGTTACACTTTGGCAAGCACTGGCATCAAGAATTGTTTTTTCACGTCTACTTGTGGCACCTAAACGATGTAACAGCCTTGAAGTTGTCCCAAGGGCGCTCGTAGATGATTGCAGTGCATGCTGCTGATGCCTGGTTAGATGTAGGCTGACACCTCTCACTTGAATAAGGACCATCGCTGACTTTGCAGTTGGTTGGTGCCGTCTCGAAAATCAGCTTGTAGTTGACTCCAGCCACAATCTGCGTCTCAACCTTGAGGAGCCGAAGAACGGTGTGGTAGTAAGTGAGGCCGGTTGTTTCCTGTGCGATCGCAAAGTGAGCCAGTTGCAGGTATTTAGGATCGCTCGATGGGTCCTGCTCCAGCCAGCCTCCTATGAGTTTTTGCCCTTGGCACAGCCCTGCCATGATCAGTGCCAAAAAACAAAAAACACCTACAGCCCTCATTATTTTCAAGACTAACGTGCAATTGGTCTCCCGCAGAATTCAGCAATCCTATGGACGACACTCTGCTGTCTCGGGATATTTATATGTGGAAACTTGTGGCGAAACACTCAGTGGTAGGTAGGCAATAAAGCTACTGACGTACGCGGTCAGATAACGCTTTGTGCCTGCTTCCTATTATAAGCGCTTTTGCTGCATG

>MG482679

GGTCTATACAACCACTCATGCGCGCATGCTGGATCTGGCTGCTTCGATACTTATACGCTATTGCTTAATTAATAGGCAGACTTCCACCTCCTTTCTGCGTAGAAAGTGTGCCGTTTCAAAAAAGGACCAATACCCACCGCGGTGGATCAGTGGTTACAGCACTCGGCTACTTATACGGAGTTTCTGGGTTCGAACCCGACCGCGGCGGCTGCGTTTTTATGGAGGCAAAATGCTAAGGCGCCCGTGCGCTGTGCGATGTCAGTATACGTTAAATATCCTCAAGTGGTAGAAATTATTCCGGAGCCCTCCACTACGGCACCTTTTCCTTTCTTTCTTCTTTCACTCTCTCCTTTATCCCTTCCCTTACGGCGCGGGTGGTGGTGGTTGCAACTTTAATGCCACAAAATGGAAGGGATTTGTTGGGGCTGGGGCGAGTCAAGGGTGGCCTCCAGCCGGGGGCGACCTCTTGAGCTCGCGTGATGATGGCCAGTTTCGTTGTTTTTCTTGAACTGGCCTAGAGCGTGTCTCATTCCTCCGCCGAGGCAGCTGGCAAGAGTGTCGTCGGAGGGGGTTTGTTAGCGCATTCCCGTAATATATGGTCTTGGGGGGCGTGCGTCCCGTCCCTGCAGTGCGAGCATGCAGGGTGATATTCACCGTTTGTAACTGAACATAATCGGTAAGGGCTAAGAATTGCCTTTGTTTGTAGGCGTCCGAGTATCGTGGCTTGCGCCCTGTCCAGGTCGGGGGGAGAGGCGGGATACGTGCATCTGGCTTCTCCTCACTCTCGTAGCAATATTTCGCCTTTGTCTTGTAGAGGTGAAAAAAAAAAAATATGTTAGTTCGCGACCACAGTTGTAGGCACAGCGCCTGAAGAGGTTACGGAAATGAGCAAGAAATCTCTATGAGCTTTATTTCATGTTCTACTGAAATTAGGCAGTGCAAATTTGCTTTTATATCTAAGTAGAGGTCCGACACTTAAATTATTCGTGTCCTACGTGTTCACGTGGAAGCAGCAACGGCGCTGCAAGTGAACGAGGAGACGGAGCGCTGGTTCAGCCAAGGAACATCATAGATGACCGCGGTACAGGTGTCCTTCACGTCTCGCGTCTTGGGCAGGCACAGCTCCTTGGTGTACGTCTCGGTCACGCGGCACGTTGATTCGGCAATCTTGAAAGTGAGCCGGTAGTTCGTGCCGGCCACAACCTGGGTCTCTGCGTCGGTGACCTCGAGCACCGTGTCGAAGAACTCTCTGTCGCCGACCTGCTGGCCGACTGCAAAGTGCGCCAGCTCTGTGAACAGGTCGTTGCCGTCCACGTTCTTCTTTTGCCAGCCGCCTACCAGTCGGGAGCCAGCCGCAGCTCCACAGCACACGGATGCCGCCAAGAGCATCGCCACCAGCACGATCGGGGAAGCCATGGCTTGGTAGTCGCAAGTAACCTTGGGTGTTTGGAGGCGATGTGTCAGCGTCAGCGGGTGAGTCGGCTGTAGGCGTCGTCGACGAGAGCGCTGCTTTTCCGACGGCGGCTGCGATCGGAAGACAACACAAGAGCTGAGAGAGGGAAAAGGGCGAGGGCGTTGGAGTGCGGCAAACGAGACGCCTTCCAGACGGGTGGCGCGG

>MG488881

CGCAGTGCATGCTGCTGACGCCTGATTGGTTGTAGGCTGACACCTCTCACTTGAGTAGGGACCATCGCTAAATTTGCAGTTAGTTGGTGCAGTCACGAATATCAGCTTGTAGTTCACTCCAGCCACAACCTGTGTTTCAACCTTGAGCAGCCGCAGAACGGTTTGGTAGTAAGTGAGGCCAGTTGCCTGCTGCGCGATCGCAAAATGAGTCAGTTGCAGGTATTTAGNNNNNNNNNNNNNNNNNNNNNNNNNNNNNNNNNNNNNNNNNNGATCGCTCGATGGGTCCTGCTCTTCCCAGCCTCCCACAAGGCGTGTTCCTTGGCTAAGCCCTACGATGGTTACTGCCAAAAAACAAACAACTGCCACAGCCTTAATTATTTTTCGGCTAACCGGAATAGTCCTCCGAAAATTTCAGCAATGCTGCTCCAAATCCTCCGCCTGCGATGACAAGGCAACTATAAAG

>MG4819437

AGGCCTTATCACCATGCTGGCCATCTGTGGTGTTGCATGCTTGGCGGCTGTTCCAGGAAGATGGACCTCCAAGGATCCCGAGTCCAGCCCAAAATACAGAGAACTGGCCCACTATGCTGTATCTCAACGCGTCGAAGGTCAAGTTAACTACAATACTGTGCTAGACATCACAAAGGTGGAAACTCAGGTCGTAGCAGGTGTAAATTACCGGCTCACCTTCACCATCGCTGCTTCGGACTGCAAGATCGGTGAGGTTGACTACAGCAAGGAGCGCTGCCCACCAAAAACCAACGTGCCGAAGGCAACCTGTACAGCGGTCGTCTACGAGAAGCCCTGGGAGAACTTCCGCTCCCTCAGCTCACTCACCTGCGAATAATGTCGAAAAACCTGCTGAAACGCCGTCGTAATTTGGCTACTCTCATTGGGCGGGAAATGTTTCATCTTTATCCTTCTACTAGCTATGTAGCACCCGATGCCAAAAATTATTAGCTTGGTGCAATGCAATGTCTATTCTTGTAAACAAGACTTCACATAGGAAGTTATAATCACAGTATTCAAACCTCTCCACTGTTTAAATAGTAAATAAAC

>MG4822580

GATGTCTGTTCTTGACCGGTGGGAGAACACGCGTAGCTGGTGAGTCCGGTTGTGTTCGTCCATGGGACTTCGTAGACAACAGCCGTGCACCTCCCGTTCACCTCTCCGACTGGAACACAACGCTGGGCAGAGTAAGTGTCTTTGCCGATCGTGCAGTTCGAAGGAGCGGTAGTGAAGGTTAGCCTGTAGTTAATGCCTGCCACCACCTGAGTGTAGACTTCGGTGAGGTTGACAAGCGTGTCGTATATCTTGCGGCCTTCTCTTTGCTGGGAAATGGCATAATGGGCCAGGTCGACGTATTTGGGGTTACTGCCAGG

>MG4823368

TTGTAAGAAACGCTGTTCTGGCCATCCATTCCGGGTTGTAGGAGCGCAGATATTCAAAAACTAAAATCATGTTTAGCTCAGTAGGTGTGATCGCAACGCTGGCTATTGTGGCTGCTGTGTACGTGGCAGCCCTACCGGGTGGCTGGGCAACCAGGGATCCCGAGTCCAGTCCAAAGTACAAGCAGCTGGCACACTACGCTGTAGCCCGGCACGTTGACGGCCTGCAGAACTACGACACTGTGCTAGAGCTCACCAAAGTGGAAACGCAGGTCGTGGCAGGTGTGAAGTACCGTCTCACCTTCACCACCGCCGCAACGGAATGCAAAATTGGTGAGATCGAATACAGCGAGGAGCGATGTCCCCCCAAGGATAACGTGCCGAAGGAAACCTGCACAGCAGTCGTCTACGAGAAAGCCTGGGAAAACTGCCGCACGCTGGTTTCGATCGACTGCGAATAAAAATCATCAAGGACAGAAGTGCCGCCGTGGTTTTGATCTTTATTAAATTTCACCTGTGAAGTGGCTGCACTGCAGACATGTGCTTCAGATGTACACTAAGAAGAAAAAGCACATTTCCTCCATCAAAGTCGGGCGCTCTGCAGGAAACCATGAAATAA

>MG4834970

CGGTCATTGGCTTTTCTTGAATGCTGCTGAGTGTGCCGCCCTGGCAATGTGTCCTCGCACAGTGATTGGCATATCATACATGCTGGATCGAGAAGAAAAGNNNNNNNNNNNNNNGTTTTTCCATTTTACTACTTGCACCTGAATGATGTAACCTTCCTGGTTTTAGTCCACGGCTGATCATAGATGATTGCATAGCAGTTTTCTACGGTATAGTATTTTGCATGCTGGCACGTCTGAAGCGAGTCTGAAAAGCCGCCTTTTATGCAGGGTCTTCGTGCGATTTTGAACCGTATCTTGTAGTTCATTCCAGACACCACCTGCGTCTGAACCTTGATAAGCCACAGCGCGGCATAACGACCACTTAACTTGATTTTCTTCTGCTCCTGCTTAATCGCAAACTTCGCCAATTCCAGATATTTCACATTGCCCCACGGGTTTTGCTCCGTCCAGCCTCCAACTAAATATCCAGTTGCATAGTGCAGCCCTAAAGTAGCTGATATTAGAAAAACAAGAACGTGCACAGGCTTCATTGTTA

>MG4835778

TTTATTTCCAGTTTCATCGAAGAGAACTCGTCTACAAAGGTTCTTTCTCTGCTAGGTAGACAGGCGCAAAACGTAAATGAATTACGCTTCATATTTTCACTTAGAAGCGGCGCCGCATGTGTACGAGGAGACGAAGCGCTCCTCGCTCAATGGAACGGTGATGACAGCGGTGCAGGTGTCCTTGACGGTTTGTGTTTTGGGGCGGCACAGTTCCTTCGTGTACGTCTGGGTCACTGGGCACGTAGATTCGGCAGTCTTGAAAGTGATGCGGTAGTTCGTGCCGGCGATAACCTGGGTCTCTGCATCGGTGACCTCGAGCACGGTGTCGAAGAACTCTTTGCCTTGAACCTGCCTGCTGATGGCGAAATGCGCGAGCTCTTGAAATTCGTCGTTTCCAGCAACGGTCTTCTTCTGCCAGCCGCCCGGAACCCCCAGCTGCGCTTGGCAGCACAAGAAGGCACCCAAGAGGACTGACAGCAGCACCAGCGAGGAAGCCATGGCTTGGTTGTAACAGATATCCGTGGTCAGTCGGAAGCAGGAGTCGTCGCTGAGAGCGCGGTTTCTCCGGCGATGACGGCGAGAAGAGGACAGCAGCAACGTTCAGCACCCCGGAGATTACGGCTGTTGTTGAAGGGCAAGCGAGAC

>MG4839947

GTTTTGTTCCAGAAGGCTGTAAAATTTAAGAAAATATAACAAGAAAACTGCTGAAAATTGATCAGCGCAACGACTCTGAAGTCTGTGCGCAAAAATGCTCCTTTTTTGGCTATGTGAATCGCTTCAGTGCTGTGCCGTTGCTTTGATCTCAGTGTTTCTTTCCTTTGCCGTACGGGGAGCATGTGTAGCTTGTCACTTGAGTTGTATTCATCCATGGAACAACGTATACAATTGCCGAACACAGCCCGTTGACCGGTCCAGATGGAACGCAGCGTTCTGCCGTGTACATATCTTTCCCGATCGTACAGTTTGATCGTGCCGTAGTGAATGTAAGGTTGTAGTTCACTCCTGCGACGACCTGCGTGGAGACATGGGTGAGTCTGACAACAGTGTCGTACATCATCCGGTTTTGCGTTTGTGTCGAGACG

>MG9618

AGTTGCCGCCATTGGCGCCCACAATAAAAGCAATCGACGTGTTTCGTTTCAGCTTTGAGAAGCAGCACCGACCAAGGGAGACGCAACATGGCTCGCTCAGTAAGTGTGGTAGCCGTGCTGGCCGTCTGTATCGCAGCTTGCGTGGCCAGTATTCCTGGAGGCTGGTCAGCCCAAGAACCTCAGTCCAGTCCCAAATACAAGGAGCTGGCACACTATGCCGTCGCGCAACGCATCGAAGGCCTGGAGAACTACGACACTGTTCTCGAGCTCACCAGAGTGGAAACTCAGATTGTAGCGGGTGTCAACTACCGCCTAACATTCACCATCGCTGGCTCGGAGTGCAAAATCGGAGAGATTGAATACAGCGAGGAGCGCTGCCCACCCAAGGAGAACGTGGCAAAAGCAACCTGCACGGCCGTTGTATACGAGAAGCCCTGGCAAAACCTCCGCTCTGTCACATCATTCACCTGCCAATGATTACAGAGAAGCCAAGGAGGTGTCGAATACATCCGAATACTCACTGCTGTTATTCTGAGAATAAAATTTCAATTGTGTATTTGAAACCAAAAAAAA

>MG961041

GGGAAATAAAGGACGTCGTCTAATAGACATCAAAGATGTCCACTGGATGAGCTGCAATGATTTCGACGTACCCCAAAATGTTTATTTGACTTTCATGTATTTTGTTACACTTTGGCAAGCACTGGCATCAAGAATTGTTTTTTCACGTCTACTTGTGGCACCTAAACGATGTAACAGCCTTGAAGTTGTCCCAAGGGCGCTCGTAGATGATTGCAGTGCATGCTGCTGATGCCTGGTTAGATGTAGGCTGACACCTCTCACTTGAATAAGGACCATCGCTGACTTTGCAGTTGGTTGGTGCCGTCTCGAAAATCAGCTTGTAGTTGACTCCAGCCACAATCTGAGTCTCAACCTTGAGGAGCCGAAGAACGGTGTGGTAGTAAGTGAGGCCGGTTGTTTCCTGTGCGATCGCAAAGTGAGCCAGTTGCAGGTATTTAGGATCGCTCGATGGGTCCTGCTCCAGCCAGCCTCCTATGAGTTTTTGCCCTTGGCACAGCCCTGCCATGATCAGTGCCAAAAAACAAAAAACACCTACAGCCCTCATTATTTTCAAGACTAACGTGCAATTGGTCTCCCGCAGAATTCAGCAATCCTATGGACGACACTCTGCTGTCTCGGGATATTTATATGTGGAAACTTGTGGCGAAACACTCAGTGGTAGGTAGGCAATAAAGCTACTGACGTACGCGGTCATATAACGCTTTGTGCCTG

>MG963143

GTCTTCCCCCGCCGTATAATCAGAAAGAAGAAACGGACCCACGCCACGGGCTATATCTTCCCGCACTCGAAGGCCGCGCCACCCGTCTGGAAGGCGTCTCGCTTGCCGCACTCCAACGCCCTCGCCCTTTTCCTTCTCTCAGCTCTTGTGTTGTCTTCCGTTCGCAGCCGCCGTCGGAAAAGCAGCGCTCTCGTCGACGACGCCTACAGCCGACTCACCCGCTGACGCTGACACATCGCCTCCAAACACCCAAGGTTACTTGTGACTACCAAGCCATGGCTTCCCCGATCGTGCTGGTGGCGATGCTCTTGGCGGCATCCGTGTGCTGTGGAGCTGCGGCTGGCTCCCGACTGGTAGGCGGCTGGCAAAAGAAGAACGTGGACGGCAACGAGCTGTTCACAGAGCTGGCGCACTTTGCAGTCGGCCAGCAGGTCGGCGACAGAGAGTTCTTCGACACGGTGCTCGAGGTCACCGACGCAGAGACCCAGGTTGTGGCCGGCACGAACTACCGGCTCACTTTCAAGATTGCCGAATCAACGTGCCGCGTGACCGAGACGTACACCAAGGAGCTGTGCCTGCCCAAGACGCGAGACGTGAAGGACACCTGTACCGCGGTCATCTATGATGTTCCTTGGCTGAACCAGCGCTCCGTCTCCTCGTTCACTTGCAGCGCCGTTGCTGCTTCCACGTGAACACGTAGGACACGAATAATTTAAGTGTCGGACCTCTACTTAGATATAAAAGCAAATTTGCACTGCCTAATTTCAGTAGAACATGAAATAAAGCTCATAGAGATTTCTTGCTCATTTCCGTAACCTCTTCAGGCGCTGTGCCTACAACTGTGGCCGCGAACTAACATATTTTTTTTTTCACCTCTACAAGACAAAGGCGAAAGATTGCTACGAGAGTGGGGAGAGGCCTGGTGCACGTATCCCGCCTCTCACCCCGACCTGGACAGGGCG

>MG963879

CAGAGAGAGGAGGCGGAGAGGCGTTTGTGTACTGTCGCAGTCAGCTTTGCCGCTCCGCGCGCTCGCCCTGTCACTGTCGGCTGGTGGTGGATTCGAGCCTCACCAGCGGTGCTGCCGTTGATTTGCAAATAATCTGCCATCCTGCAACCGCACGAATAGATGCTATGGGTATGAGGCAAACTATTTCGGCACTACTCGTCGCAGTTCTGTGCGCCCTAGTGGCGCGCAGCTCTGCTGCTCTGGTCGGAGCTCCGATGAAGCATGACCCGAAAGAGTTTGAGAATTATCTTGACGAGGCTCACTTCGTGGCGTCGTCCCAGGTGGAGGGCCGTGAGTTCTACGACACCGTCGTCGAGATCCTCGAAGCCGAAACCCAGGTTGTGTCAGGAGTGATATACAGGCTCAAAATGAAGATGACAGAATCAACGTGCAAGGTCTCAAAAGGGAACTATTCCAAAGAGCTATGTGTGCCGAAGAAAGGGAAGCCAGTAAAGATTTGCGAAGCGGAAGTGTACAGCAGGGCGTGGGAAAACTACCACAATGTGAACTCCTTCACATGTGAAGCGGCCATGTCAATTGTCACACGGATATCGGGAGTTCTAGACCCAAAGGACTTGACTTTTGCCTACCTCTCGAAGCATCTCAAGTTGTCCCAGGAGCGGTCACTCTTCAGCGTATTTGTCAGGACTTACAACAAGACTTACAAAGACAAAGAAGAACACGAAGCCCGCTTCATGATCTTCAAGAACAACTTGAAAAGGATTGCCCTTTTCAACCGGCTCGAAGAGGGGACAGCTCATTATGGACTCACAGAGTTCTCGGACCTGTCTCCAAGCGAGTTTGAGCGCCGCCACCTCGGACTGAAGAAGGACCTCAATGAACACAAGGAAGAGGTCAAACCTATCAAGGTTGGACCTGTTCATGAACCACTGCCAGACCTTTTCGACTGGAGGACCAAGGGGGCAGTGACAGACGTGAAGAACCAGGG

>MG9614858

TTTTATGAATATGCGAAGTCTACCCAGACAACGGTTTCTTTCATTTTGGCGTCACACAGTCGAACGCCAGAACCTTCTTGATGTTCTGGTGTAGTGGCTCGTGAATAATTGCAGTGCACGTTTCACTGATATAGTAATTGGCAGGCCTACAGATGAATCCACTTCTCTGGCAGTGTCTTCTTGCAATGAGGAGTTCTATCTTGTAGTTCATGCCAGCCACAACCTGCGTGGAGACACTCTTGAGCCATAGAGCCGCGTAGTTACGTCTGAGGCCAGATTTCTCTCGCGA

>MG9618773

GAGCGCTGCTCTCGCATGCCGGCTCCCGCAGGTGTGAGGCCTTTCGGTTGAAATGGCGCCAGGCTGGCGGGGACTGAGACAGCTGCCCGCTTGTACACACTCGCACACCTGGGTCGAGGGTAGCGCCTGGGTCGAGGGGAAGAGAACCGCCAAAACAACGCCGCGTTCGCCGGCCCGGGTCGAAAAACAAAGGGCCAGAAAGGCGGTTTGAGCTCCGTGCCTCCATCAAGGACGTATAAGCAAGATAAATTAGCGGGGCAGCGACCGCCGGCGAAGCACCGCTGAAGCCATCGTCAACAGGCTTCCTGTATCGAGCGACGAATGCGGGAGATGTCCCCTTTCCGGGCTGTCCGCGCAACTTCGCACAGACCTCCAGGCTGACGAGAGCTCCATAAGTACAGGTTTCGCTGAAGTGTCCAGCAGGAATGACGGCGGTAAAGCAAGCCTGTCTGCTCCTCGTCGCGGTGTTGGCGGCGGTGTCGCTCTGCGGTGACTACGGGAGATCTGCATTCGTCGGTGGGTGGCAAGAACAACAACCTTACCGCGACCCCAAATATCTCCAGCTGGCTCACTACGCCGTCTCGACACAAACGCAAAACCTGAAGATGTACGACACTGTTGTCAGACTCACCCATGTCTCCACGCAGGTCGTCGCAGGAGTGAACTACAACCTTACATTCACTACGGCACTATCAAACTGTACGATCGGGAAAGATATGTACACGGCAGAACGCTGCGTTCCATCTGGACCGGTCAACGGGCTGTGTTCGGCAATTGTATACGTTGTTCCATGGATGAATACAACTCAAGTGACAAGCTACACATGCTCCCCGTACGGCAAAGGAAAGAAACACTGAGATCAAAGCAACGGCACAGCACTGAAGCGATTCACATAGCCAAAAAAGGAGCATTTTTGCGCACAGACTTCAGAGTCGTTGCGCTGATCAATTTTCAGCAGTTTTCTTGTTATATTTTCTTAAATTTTACAGCCTTCTGGAACAAAACTGGAATAATATATAATAAC

>MG9624205

TTTCAGCAGGTTTTTCGACATTATTCGCAGGTGAGTGAGCTGAGGGAGCGGAAGTTCTCCCAGGGCTTCTCGTAGACGACCGCTGTACAGGTTGCCTTCGGCACGTTGGTTTTTGGTGGGCAGCGCTCCTTGCTGTAGTCAACCTCACCGATCTTGCAGTCCGAAGCAGCGATGGTGAAGGTGAGCCGGTAATTTACACCTGCTACGACCTGAGTTTCCACCTTTGTNNNNNNNNNNNNNGTATTGTAGTTAACTTGACCTTCGACGCGTTGAGATACAGCATAGTGGGCCAGTTCTCTGTATTTTGGGCTGGACTCGGGATCCTTGGAGGTCCATCTTCCTGGAACAGCCGCCAAGCATGCAACACCACAGATGGCCAGCATGGTGATAAGGCCTACTGGGAGAAACATTCCTTCCTTAGTCTGAGCTTCTGAGATCTGAAATAAAACGTTAAG

>MG9632895

TTTTTCAGAGTTGGGTGATGTTGTTTATTCAGTTCCAGCAAGGAAAATATTTATGCACTCCGAATATGCTTATGAATATGCGAAGTCTGTACCCAGATAACGGTTTCTTTCATTTTAGCGTTACACAGTCGAACGCCAGAATGTTCCTCATGTTCTGATGTAGTGGCTCATGAATGACCGTAGTGCATGTTTCTGTGACATAGTATTTCGCAGGCCTGCAGTTGGCTCCACGTTTCTTGCAGTCTCTTCTTGCAATGAGGATTTCTATCTTGTAGTTAATTCCAGCGACCACCTGCGTAAAGACACTCTTGAGCCATAGAGCCTCGTAGTTACGTCTGA

>MG9644349

CCGAAGTCTACACTCAGGTGGTGGCAGGCATTAACTACAGGCTAACCTTCACTACCGCTCCTTCGAACTGCACGATCGGCAAAGACACTTACTCTGCCCAGCGTTGTGTTCCAGTCGAAGAGGTGAACGGGAGGTGCACGGCTGTTGTCTACGAAGTCCCGTGGACGAACACAACTGGACTCACCAGCTACGCGTGTTCTCCCACCGGTCAAGAACAGACATCCTGAGGCCCGGTCGACCAAAG

>MG9644760

GCCAACTGTTGAAAACCACATGTAACTTCGGCTTTCTCCACTTCAAGTTTGTAGTTGACACCATTCACGACCTGTGTAGAAGCCTTCACAACCTTCAAATTGATGCCACGTAGGTAGGGTGGATTGACTCTAGTAGCGAATGCTTGAAAGGCAAGATTGAAAAATTTCAGTGACCTGGACGGGTTTTCATGGTGCATCCACCCGCCCAGAATTGTAGGTGCGCGACCTTGAGCAACTACCGCGCCGCAGAAAAAAAGTGCGATGTTCA

>MG120298

TTTCGACGTACCCCAAAATGTTTATTTGACTTTCATGTATTTTGTTACACTTTGGCAAGCACTGGCATCAAGAATTGTTTTTTCACGTCTACTTGTGGCACCTAAACGATGTAACAGCCTTGAAGTTGTCCCAAGGGCGCTCGTAGATGATTGCAGTGCATGCTGCTGATGCCTGGTTAGATGTAGGCTGACACCTCTCACTTGAATAAGGACCATCGCTGACTTTGCAGTTGGTTGGTGCCGTCTCGAAAATCAGCTTGTAGTTGACTCCAGCCACAATCTGAGTCTCAACCTTGAGGAGCCGAAGAACGGTGTGGTAGTAAGTGAGGCCGGTTGTTTCCTGTGCGATCGCAAAGTGAGCCAGTTGCAGGTATTTAGGATCGCTCGATGGGTCCTGCTCCAGCCAGCCTCCTATGAGTTTTTGCCCTTGGCACAGCCCTGCCATGATCAGTGCCAAAAAACAAAAAACACCTACAGCCCTCATTATTTTCAAGACTAACGTGCAATTGGTCTCCCGCAGAATTCAGCAATCCTATGGACGACACTCTGCTGTCTCGGGATATTTATATGTGGAAACTTGTGGCGAAACACTCAGTGGTAGGTAGGCAATAAAGCTACTGACGTACGCGGTCAGATAACGCTTTGTGCCTGCTTCCTATTATAAGCGCTTCTGCTGCATGATAGTGTTTATCGAGCGATGCCAAG

>MG1201375

CTCGAAGGCCGCGCCACCCGTCTGGAAGGCGTCTCGTTTGTCGCACTCCAACGCCCTCGCCCTTTTCCCTCTCTGAGCTCTTGTGTTGTCTTCCGTTCTCAGCCGCCGTCGGAAAAGCAGCGCTCTCGTCGACGACGCCTACAGCCGACTCACCCGCTGACGCTGACACATCGCCTCCAAACACCCAAGGTTACTTGTGACTACCAAGCCATGGCTTCCCCGTTCGTGCTGGTGGCGATGCTCTTGGCGGCATCCGTGTGCTGTGGAGCTGCGGCTGGCTCCCGACTGGTAGGCGGCTGGCAAAAGAAGAACGTGGACGGCAACGAGCTGTTCACAGAGCTGGCGCACTTTGCAGTCGGCCAGCAGGTCGGCGACAGAGAGTTCTTCGACACGGTGCTCGAGGTCACCGACGCAGAGACCCAGGTTGTGGCCGGCACGAACTACCGGCTCACTTTCAAGATTGCCGAATCAACGTGCCGCGTGACCGAGACGTACACCAAGGAGCTGTGCCTGCCCAAGACGCGAGACGTGAAGGACACCTGTACCGCGGTCATCTATGATGTTCCTTGGCTGAACCAGCGCTCCGTCTCCTCGTTCACTTGCAGCGCCGTTGCTGCTTCCACGTGAACACGTAGGACACGAATAATTTAAGTGTCGGACCTCTACTTAGATATAAAAGCAAATTTGCACTGCCTAATTTCAGTAGAACATGAAATAAAGCTCATAGAGATTTCTTGCTCATTTCCGTAACCTCTTCAGGCGCTGTGCCTACAACTGTGGCCGCGAACTAACATATTTTTTTTTTCACCTCTACAAGACAAAGGCGAAAGATTGCTACGAGAGTGGGGAGAAACCTGGTGCACG

>MG12030641

CCGAAGTCTACACTCAGGTGGTGGCAGGCATTAACTACAGGCTAACCTTCACTACCGCTCCTTCGAACTGCACGATCGGCAAAGACACTTACTCTGCCCAGCGTTGTGTTCCAGTCGGAGAGGTGAACGGGAGGTGCACGGCTGTTGTCTACGAAGTCCCATGGACGAACACAACCGGACTCACCAGCTACGCGTGTTCTGCCACCGGTCAAGAACAGACATCCTGAGGCCCGGTCGACCAACGTGCAAGGTCACTGACATCTTC

>MG12033468

CCCAAATCGAACCCGGCTTCAAGGACACCTGTACCAGTAAATACGTGTGTTCACCTTCACAATATGAACGTGTTGCAAAGAGCTGCAGCAATGACTACGGTGAGGCAAGCCGTCCTCGTTCTTGTCTTCGCAACGGCGACAACCGCGCTTATTTGCTGCCACAACTCCACTTCCGTCCTGGTCGGTGGCTGGACCAGGCAAGAGCCTGGCAGTAACCCCAAATACGTCGACCTGGCCCATTATGCCATTTCCCAGCAAAGAGAAGGCCGCAAGATATACGACACGCTTGTCAACCTCACCGAAGTCTACACTCAGGTCGTCGCCGGCGTTAACTACAGGCTCGTCTTCACTACCGCTCCTTCGAACTGCA

>MG12046908

CTTGAAAGTGATGCGGTAGTTCGTGCCTGCGATAACCTGGGTCTCTGCATCGATGACCTCGAGCACGGTATCGAAGAACTCTTTGCCTTGAACCTGCCTGCTGATGGCGAAATGCGCGAGCTCTTGAAATTCGTCGTTGCCAGCAACGGTCTTCTTCTGCCAGCCGCCCGGAACCCCCAGCTGCGCTTGGCAGCACAAGAAGGCACCCAAGAGGACTGACAGCAGCACCAGCGAGGAAGCCATGGCTTGGTTGTAACAGATATCCGTGGTCAGTCGGAAGCAGGAGTCGTCGCTGAGAGCGCGGTTTCTCCGGCGATGACGGCGAGAAGAGGACAACAGCAACGTTCAGCACCCCGGAGATTACGGCTGTTGTTGAAGGGCAAGCGAGACTCCTCACAGACTGGAGAAGCG
