# Supplementary material for: A repertoire of protease inhibitor families in Amblyomma americanum and other tick species: inter-species comparative analyses
Source: Parasit Vectors. 2017 Mar 22;10:152. doi: 10.1186/s13071-017-2080-1 (PMC5361777; doi:10.1186/s13071-017-2080-1)
Supplement: Supplementary file 3 — FASTA sequences for Amblyomma americanum contigs from Illumina sequencing, by PI family. (ZIP 638 kb) [file 13071_2017_2080_MOESM3_ESM.zip › A. americanum I29.docx]

>MG4887

TGGTTCACTCCCAGCTTGTAAGAAACGAGGCCCTTGGAGTACTTTTCATTGTGCTTGGCAATCAGCAAGCTGTTCTCAGTGAAAATCTTGAACCTGAGGAGCTCCTCCACGTTGGACTTGTATGTCTTCTTGTGTGTTAGTTTGAAAGCCTCCCACTGGGTGCGGAGAATTTCGTGTGAACTGGCTGCCGTGGCTGCTGCCACAATGGCACAGAGCAGAGCGAATCGCAGCATTGTGGAGGAGGGCAAAGTCTCGTGAGTAGCTTCCTGCCTGTACGCACCCTGCGGGGTTTCTGCGTAATCACGCGTGTTTTATACCTGCCCGTGAAGACTGTTTGTCACACCTTGCCATTTCACTTGGCGATATTCATCGGGGCACGAGAAAATTCGACAATAGTATCGGAACCGATAAAATGAACTGATAGGTTTCGTTCCTGGACGGGTCTCGTG

>MG485027

CGGGAATCCTATCCGCAACTCCAGCAGTCTTGCGCTTTTAACTTTTGTCATGTGCTTCTACAACCTCGTGATTCTATCACAGTAGTGGATTTCACGCCCTGCATTTATCGCGCACTCCGAGATATACGGTCTTGTTCTGTCTGCCCAGCTGAACGAGCTAAAGCACCGTTCAGCAAAGATCTGATAAGGTCTGCAGGTTTGAGCTACGGACTGATCTGCATTCTACAATGATACGGCGCCGTCTCTTATTGGTGTTACTTGCGAGTGCATCCATGGCCCTTCAGCAAGAGTTCTTAAGGGACGAGTGGGAATCTTTCAAAGCAAAAAACGGAAAAACGTACACCACTGGCGAAGAGGAAGAAAGAAGAAAGTGTATTTTTGAAAAGAATCTTGAGCTGATTATACAGCACAACAAGAAATACGCCGAAGGAAATGTAACCTACAAACTTGCTATGAATCACTTCGGAGACATGCTTCCATCTGAGCTAACACCTGAAGTTACTTTTCCACCTGGAGCGGGAGACAACCTAGAAGTGCACTCCAGCCCAGCAGTTGCCGTGAATATCACTGACTTGCCAGATGAAGTCGACTGGAGGAAGAAGGGTGCAGTAACACCTGTTCCTGACCAAGGGTCGTGTTTGTCTTCCTACGCCTTCAGTGCGATCGGAGCTCTCGAAAGCCAGCAGTTTCTGAAGACAACTAAGCTGATTCCTCTGAGCGCTCAGAATGTTGTCGACTGTGGCGCTGTCTTTCATAATCGGAACTGTGATGGTGGTTTTATGGATTCTACTTACGGTTACATTAAGATCAACGGTGGCATTGACAGTGAAGAAAGCTACCCCTACGTTGGAGTTAAATCAGACTGCGCCTTTGATCCGACAGGGGTGGCGGCTACTGTATCCTC

>MG96513

GTACACCACTGGCGAAGAGGAAGAAAGAAGAAAGTGTATTTTTGAAAAGAATGTTGAGCTGATTATACAGCACAACAAGAAATACGCCGAAGGAAATGTCACCTACAAACTTGCTATGAATCACTTCGGAGACATGCTTCCATCTGAGCTAACACCTGAAGTTACTTTTCCACCTGGAGAGGGAGACAACCTAGAATTGCACTCCAGCCAAGCAGTTGCCGTGAATATCACTGACTTGCCAGATGAAGTCGACTGGAGGAAGAAGGGTGCAGTAACACCTGTTCCTGACCAAGGGTCGTGTTTGTCTTCCTACGCCTTCAGTGCGATCGGAGCTCTCGAAAGCCAGCAGTTTCTGAAGACAACTAAGCTGATTCCTCTGAGCGCTCAGAATGTTGTCGACTGTGGCGCTGTCTTTCATAATCGGAACTGTGATGGTGGTTATATGGATTCCACTTACGGTTACATTAAGATCAACGGTGGCATTGACAGTGAAGAAAGCTACCCCTACGTTGGAGTTAAATCAGACTGCGCCTTTGATCCGGCAGGGGTGGCGGCTACTGTATCCTCCTTTGTCAACGTCCGATCAGCAACAGAACTGACCCTCATGACAAAACTGGCAACGGTTGGCCCTATGTGCGCCTTCATTGACAACAGACTGACTTCATTTATTCTGTACTCCGAAGGTGTGTACCACGACCCGAACTGTGCCAAGGACACAATGAACCACGCCGTGCTGATTGTTGGCTACGGGGCCACGGAAAAAGGCGAGAAGTATTGGATTGTCAGGAATTCGTGGGGCACACAGTGGGGTCAGGGTGGATACATCCTCATCAGAAGAGACTCAAGTAATGAATGTGGAATCGCTAGCTTCATCACCTACCCTGTCATTTAGAGAAGTTCCCACGTTTTCCTGAAATCTTATTCAAGATTATAAGGTGCAGCTGATGTGGAAGCACCGGTCAAGAAGCTTTAAAAGGTTTTCTAAAACAATATTTACCATTCCTGTGAAAAATGTCGCAATAAAATTGTAGTTAAAGCAGAA

>MG963879

CAGAGAGAGGAGGCGGAGAGGCGTTTGTGTACTGTCGCAGTCAGCTTTGCCGCTCCGCGCGCTCGCCCTGTCACTGTCGGCTGGTGGTGGATTCGAGCCTCACCAGCGGTGCTGCCGTTGATTTGCAAATAATCTGCCATCCTGCAACCGCACGAATAGATGCTATGGGTATGAGGCAAACTATTTCGGCACTACTCGTCGCAGTTCTGTGCGCCCTAGTGGCGCGCAGCTCTGCTGCTCTGGTCGGAGCTCCGATGAAGCATGACCCGAAAGAGTTTGAGAATTATCTTGACGAGGCTCACTTCGTGGCGTCGTCCCAGGTGGAGGGCCGTGAGTTCTACGACACCGTCGTCGAGATCCTCGAAGCCGAAACCCAGGTTGTGTCAGGAGTGATATACAGGCTCAAAATGAAGATGACAGAATCAACGTGCAAGGTCTCAAAAGGGAACTATTCCAAAGAGCTATGTGTGCCGAAGAAAGGGAAGCCAGTAAAGATTTGCGAAGCGGAAGTGTACAGCAGGGCGTGGGAAAACTACCACAATGTGAACTCCTTCACATGTGAAGCGGCCATGTCAATTGTCACACGGATATCGGGAGTTCTAGACCCAAAGGACTTGACTTTTGCCTACCTCTCGAAGCATCTCAAGTTGTCCCAGGAGCGGTCACTCTTCAGCGTATTTGTCAGGACTTACAACAAGACTTACAAAGACAAAGAAGAACACGAAGCCCGCTTCATGATCTTCAAGAACAACTTGAAAAGGATTGCCCTTTTCAACCGGCTCGAAGAGGGGACAGCTCATTATGGACTCACAGAGTTCTCGGACCTGTCTCCAAGCGAGTTTGAGCGCCGCCACCTCGGACTGAAGAAGGACCTCAATGAACACAAGGAAGAGGTCAAACCTATCAAGGTTGGACCTGTTCATGAACCACTGCCAGACCTTTTCGACTGGAGGACCAAGGGGGCAGTGACAGACGTGAAGAACCAGGG

>MG1206079

CCCCATTTGGTTTAATCAACCACTCTTCTACATGAAATTAGCAATACAAAGTCAGTTTTTTAAAAAGCTCGCTTTTGCAAGTCAGCACTTTGTACAGGTGCATTTGTGATCAAAGGCATCAGGCAGGAACACCAGGTTTAGTCACACTTATTTTGCATAGTGCAGCTTCTGTCATCAGTGCAATATCCACAAGGGCAATCATCTGCCACATGACTGTTCCATGTCCGCAGCAAATGCAGCCTTTGCTTTAGGTATGAGCACATCTGGGTTGAGCAAAATGTCAAGACAAGCCAGGGCCATAGCTTTTGCAGCAACTAGTGTCGGTTCCTGAGCACATGGATGGCCTGCAGCATCCGTAAACTCCTTTGTATGGTTTGGTCCGGCCGTGTCAATGCGGTAAATGGGGTGAATGCTAGGCACTGTGTGACATACATCCCCCATGTCAGATGAAGCCACAAAAAGAGTTGCCTTGGGGTTGGCATCATCAAATTCAACACCAAGTGCCTCAGCATGCTTCTTGTACAGCTGTGCAATAACGGAGTTAGTAACAACAGGCTTGAAGCTGAAGGACCGAGTTATTTCCATGGAGCAACCTGTAGCTTCAGCGCCAGCAGAAATGCAGGACATAACCTTCTTGCGCAGTTCTTCCATCATTGCCACGGTGGGAGCGCGGTATAAGATGTCCATGGCTGAACGCTCTGGGATGATGTTGGCTGCTGCGCCCCCGTTAGTAAATATCGCTCCTACACGGCAATGTGGCTTGATGTGCTGCCGAAGATATGAGAGCCCATTGTAACACATTACTGCTGCATCCACAGCATTAACCCCGTCCCAAGGGGCACCTGATGAGTGTGCAGCCTTCCCATGGTACTCTACGCTGATATGGCCAACACCAATAAACAGTGGAGCCAGTTCATTGCTTCTGCTGGGATGAACCATCATGGCGACATCTATATCTGCAAATGCGCCTGCATTAATCAAGTCAATTTTTCCACCTCCGCTTTCTTCGGCTGGTGTTCCAAGGACAACAACCTTGCCCTTCAGCTTCGGATCGGACCGCAACGCTTCCTTCACGGCCACTCCAGCGGCAAACCCCGCTTCAGCTATTAAGTTGTGCCCACAGGCGTGTCCAATTACAGGCAGCGCATCGTATTCGCATATGATGCATATCACGGGCCCACCTAGCACGTTGGAATATTCAGCCCTGAAAGCGGTATCCAGGTGGTACTTCCGCTGCACCTGAAAGCCTTCGTCTTCAAGTGCTTGCGACAGATAGTCATGGGCTTTCACTTCTCTGTAATACAACTCTGGATTTTTCCAAATTGTTTGGCTCACTAAGTTTAGTTTCTCGCGGTACTTATCAATGGTGCTAACAATGAACTCAGACATGTCGGACATGGCAGCGGTACAGACGCTACAACACTGCAAATCCTATAAGCTGCTCCGGCTGCTCCTGGAAATACAGCACATGCCGGAGAGAGATAATTTTGCACCCGGCACTACACAACTGCTGCTCAGGTCAACGCCTCTTATTCAGTGACCTTCGTACCGCGCCGTTAGCGCCGTCGTCTGCGCGCTCCATGTCTGCGCGCCACGGCTACCAC

>MG1206080

AGGCTGTTTAGCATGTTGAGAGTAAAGTGGCATGAACTTTTTCATTCCGGGGCTTATTTTGAAGCATTACTGTTTGGAACTTGGTACTTGAAGTTTAGTACCAGCTTATTAGCAGGATGCTATCGCTACAGGCTGCCACAGAGCCATCTTGACTGAGCGATTTTAGCTGCACAGAAGCCATTTTTGAGCTGCATGTTTGGCAACTAAGCTTGTAGGAATGCCTATATGACTGCCTAAATCATGGCTCTTTAAGGGGCCATTTGCCATAGACTGCAATGGGTGGAAGGTGCCGATGCTTTTGATCTTCTCTCGGGAGCACTTTCTCGTTGTTGACATTTGGTGCAAGGGTTCTACCAGAGTTTCGGCTGGATGCTGTTTACAGAGAGAAGGTCACTGCGATGTCATAGTAATGCTTAGTGTTGACCCAATCATGCTGAGCTCTTTCATTCTTCGCTCTGGCTGGTACCTCGTGTCTCGGAACTCCAAGCCCACATAACCTTTGGGGCCTTTCTTGCAAATCATCTCTAGCACCTGTGGCTGTCCCGGTCGCATGTTTGTGCGGTTTCTACCGTTGCACATGTTTAGAAGTGACTGTTCACTC

>SG9621955

CTTGCCCTGGTAGCCATTCATCTTCTTAACGAACTCATGCGGGAGCAGGTCAGCAAACTGGTTCACTCCCAGCTTGTAAGAAACGAGGCCCTTGGAGTACTTTTCATTGTGCTTGGCAATCAGCAAGCTGTTCTCAGTGAAAATCTTGAACCTGAGGAGCTCCTCCACGTTGGACTTGTATGTCTTCTTGTGTGTTAGTTTGAAAGCCTCCCACTGGGTGCGGAGAATTTCGTGTGAACTGGCTGCCGTGGCTGCTGCCACAATGGCACAGAGCAGAGCGAATCGCAGCATTGTGGAGGAGGGCAAAGTCTCGTGAGTAGCTTCCTGCCTGTACGCACCCTGCGGGGTTTCTGCGTAATCACGCGTGTTTTATACCTGCCCGTGAAG

>SG9624711

CACGGCCTGGTCCTTCACAGGCGTCACTGCTCCATACAGCCTCCAGTCAACTTGGTCGGGCAACTTGGTACTGAATTTGTGGCGAGGAAATGGATCAGCGGTGGAAGTCCCATCGCTGGACTGGAGACGTCCACGGAGCACGCTGATCTCCTCCTGCGTGCGGTCAGCCAGATGGTTCACGGCCAGCTGGTAGCCCAGGTTAGCACGGTTTGTCGAGTCGATGAACCTGAGGTTTTGGCGGAAGATGTCGCGTCGTCTGTCATGCTCCACATC

>AAFF10869

TGCTTTAACTACAATTTTATTGCGACATTTTTCACAGGAATGGTAAATATTGTTTTAGAAAACCTTTTTAAAGCTTCTTGACCGGTGCTTCCACATCAGCTGCACCTTATCATCTTGAATAAGATTTCAGGAAAACGTTGGAACTTGTCTAAATGACAGGGTAGGTGATGAAGCTAGCGATTCCACATTCATTACTTGAGTCTCTTCTGATGAGGATGTATCCACCCTGGCCCCACTGTGTGCCCCACGAATTCCTGACAATCCAATACTTCTCGCCTTTTTCCGTGGCCCCGTAGCCAACAATCAGCACGGCATGGTTCATTGTGTCCTTGGCGCAGTTCGGGTCGTGGTACACACCTTCGGAGTACAGAATAAATGAAGTCAGTCTGTTGTCAATGAAGGCGCACATAGGGCCAACCGTTGCCAGTTTTGTCATGAGGGTCAGTTCTGTTGCTGATCGGACGTTGACAAAGGAGGATACAGTAGCCGCCACCCCTGCCGGATCAAAGGCGCAGTCTGATTTAACTCCAACGTAGGGGTAGCTTTCTTCACTGTCAATGCCACCGTTGATCTTAATGTAACCGTAAGTGGAATCCATATAACCACCATCACAGTTCCGATTATGAAAGACAGCGCCACAGTCGACAACATTCTGAGCGCTCAGAGGAATCAGCTTAGTTGTCTTCAGAAACTGCTGGCTTTCGAGAGCTCCGATCGCACTGAAGGCGTAGGAAGACAAACACGACCCTTGGTCAGGAACAGGTGTTACTGCACCCTTCTTCCTCCAGTCGACTTCATCTGGCAAGTCAGTGATATTCACGGCAACTGCTTGGCTGGAGTGCGCTTCTAGGTTGTCTCCCGCTCCAGGTGGAAAAGTAACTTCAGGTGTTAGCTCAGATGGAAGCATGTCTCCGAAGTGATTCATAGCAAGTTTGTAGGTTACTTTTCCTTCGGCGTATTTCTTGTTGTGCTGTATAATCAGCTCAAGATTCTTTTCAAAAATACACTTTCTTCTTTCTTCCTCTTCGCCAGTGGTGTACTTTTTTCCGTTTTTTGCTTTGAAAGATTCCCACTCGTCCCTTAAGAACTCTTGCTGAAGGGCCATGGATGCACTCGCAAGTAACACCAATAAGAGACGGCGCCGTATCATTGTAGAATGCAGATCAGTCCTTAGCTCAAACCTGCAGACCTTATCAGATCTTTGCTGAACGGTGCTTTAGCTCGTTCAGCTGGGCAGACAGAACAAGGCCGTATATCTCGGAGTGCGCGATAAATGCAGGGCGTGAAATCCACTACTGTGATAGAATCACGAGGTTGTAGAA

>AAFF36918

TGAAGTCCGAAAAGCGGGTCAGCCCGTAGCGTGCAGTGTCCTTCGAGCTGCGACTCTTGCGTTCGATCCGTCCCAGGCTGTCGCGGAAGGCGGCGAAGCGCCGCTCGTACTCGGCCGAGCCGGGCTCGTAGCTTTTGTTGTAGCGAGACACGTACTGGGCGAACGCGTCCTCGACGCTCGACGCCACGATCGCTCCCCGTGCGTCATCCGTCGGGATCGTGATCGAAGCTGCGGCGATTGACAGTAGCAGCAACAGCAGCAGCGGCGGNN

>AAUF695

GGTGCGTACAGGCAGGAAGCTCCTCACGAGACTTTGCCCTCCTCCACAATGCTGCGATTCGCTCTGCTCTGTGCCATTGTGGCAGCAGCCACGGCAGCCAGTTCACACGAAATTCTCCGCACTCAGTGGGAGGCTTTCAAACTTACACACAAGAAGACATACAAGTCCAACGTGGAGGAGCTCCTCAGGTTCAAGATTTTCACTGAGAACAGCTTGCTGATTGCCAAGCACAATGAAAAGTACTCCAAGGGCCTCGTTTCTTACAAGCTGGGAGTGAACCAGTTTGCTGACCTGCTCCCGCATGAGTTCGTTAAGATGATGAATGGCTACCAGGGCAAGCGCTTGGGAGGTAGTGGCTCCACCTACCTTCCACCCGCTAACCTGAACAACAGCAGCCTGCCAAAGACCGTTGACTGGCGCAAGAAGGGAGCCGTGACCCCTGTCAAGGACCAGGGACAGTGCGGATCCTGCTGGGCCT

>AAUF19955

CTAAAGAGCAGTTTTCTGCTTTCACTACAATTTTATTGCGACATTTTTCACAGGAATGGTAAATATTGTTTTAGAAAAACCTTTTTAAAGCTTCTTGACCGGTGCTTCCACATCAGCTGCACCTTATAATCTTGAATAAGATTTCAGGAAAACGTTGCAACTTGTCTAAATGACAGGGTAGGTGATGAAGCTAGCGATTCCACATTCATTACTTGAGTCTCTTCTGATGAGGATGTATCCACCCTGGCCCCACTGTGTGCCCCACGAATTCCTGACAATCCAATACTTCTCGCCTTTTTCCGTGGCCCCGTAGCCAACAATCAGCACGGCGTGGTTCATTGTGTCCTTGGCGCAGTTCGGGTCGTGGTACACACCTTCGGAGTACAGAATGAATGAAGTCAGTCTGTTGTCAACGAAGGCGCACATAGGGCCAACCGTTGCCAGTTTTGTCATGAGGGTCTGTTCTGTTGCTGATCGGACGTTGACAAAGGAGGATACAGTAGCCGCCACCCCAGTCGGATCAAAGGCGCAGTCTGATTTAACTCCAACGTAGGGGTAGCTTTCTTCACTGTCAATGCCACCGTTGATCTTAATGTAACCGTAAGTGGAATCCATATAACCACCATCACAGTTCCGATTATGAAAGACAGCGCCACAGTCGACAACATTCTGAGCGCTCAGAGGAATCAGCTTAGTGGTCTTCAGAAACTGCTGGCTTTCGAGAGCTCCGATCGCACTGAAGGCGTAGGAAGACAAACACGACCCTTGGTCAGGAACAGGTGTTACTGCACCCTTCTTCCTCCAGTCGACTTCATCTGGCAAGTCAGTGATATTCACGGCAACTGCTTGGCTGGAGTGCAATTCTAGGTTGTCTCCCTCTCCAGGTGGAAAAGTAACTTCAGGTGTTAGCTCAGATGGAAGCATGTCTCCGAAGTGATTCATAGCAAGTTTGTAGGTGACATTTCCTTCGGCGTATTTCTTGTTGTGCTGTATAATCAGCTCAAGATTCTTTTCAAAAATGCACTTTCTTCTTTCTTCCTCTTCGCCAGTGGTGTACTTCTTTCCTTTGTGCAGTCTTCGTTTGGCATTCTTGCTTAGGACTCTGATGAAGCCGTCGTCAGCCGAAGAGT

>AAUF29826

CAAACATGTGGGTCCGAAGAGTATATAACCCGTAACAGCAGCCAGCAACGCTGCGCGGTGTGGTCCACCTGGAAGCTCGTCAGCAGGAATTCTGTCCGTACCTCCATAATGCTAAGGTTCACCGTACTGTGTGCGCTTGTGGCGGCGACTACGGCAGCCAGTTCACAGGAAATACTCCGCACCCAGTGGGAGGCCTTCAAGGTAGCACACAAAAAAACCTATGAATCCCATGTGGAGGAGGTTCTCCGGTTCAAGATATTCACGGAAAACAGCCTTTTTATTGCCAAGCACAATGAGAAGTACGCCAGAGGCCTCGTATCTTACAAGCTGGGCATGAACAAGTTTGGTGACCTGCTGCCGCACGAGTTCGTTAAGATGATGAATG

>AAFM71

AATTTTATTGCGACATTTTTCATAGGAATGGTAAATATTGTTTTAGGAAAGTTGTTTAAAGCTTCTTGGCCGGTGCTTCCACATCCGCTGCACCTTATCATCTTGAATAAGATTTCAGAAAAACGTTGGAACTTGTCTAAATGACAGGGTAGGTGATGAAGCTAGCGATTCCACATTCATTACTTGAGTCTCTTCTGATGAGGATGTATCCACTCTGACCCCACTGTGTGCCCCACGAATTCCTGACAATCCAATACTTCTCGCCTTTTTCTGTGGCCCCGTAGCCGACAATCAGCACGGCGTGGTTCATTGTGTCCTTGGCACAGTTCGGGTCGTGGTACACACCTTCGGAGTACAGAATAAATGAAGTCAGTCTGTTGTCAATGAAGGCGCACATAGGGCCAACCGTTGCCAGTTTTGTCATGAGGGTCAGTTCTGTTGCTGATCGGACGTTGACAAAGGAGGATACAGTAGCCGCCACCCCTGCCGGATCAAAGGCGCAGTCTGATTTAACTCCAACGTAGGGGTAGCTTTCTTCACTGTCAATGCCACCGTTGATCTTAATGTAACCGTAAGTGGAATCCATATAACCACCATCACAGTTCCGATTATGAAAGACAGCGCCACAGTCGACAACATTCTGAGCGCTCAGAGGAATCAGCTTAGTTGTCTTCAGAAACTGCTGGCTTTCGAGAGCTCCGATCGCACTGAAGGCGTAGGAAGACAAACACGACCCTTGGTCAGGAACAGGTGTTACTGCACCCTTCTTCCTCCAGTCGACTTCATCTGGCAAGTCAGTGATATTCACGGCAACTGCTTGGCTGGAGTGCGCTTCTAGGTTGTCTCCCGCTCCAGGTGGAAAAGTAACTTCAGGTGTTAGCTCAGATGGAAGCATGTCTCCGAAGTGATTCATAGCAAGTTTGTAGGTTACTTTTCCTTCGGCGTATTTCTTGTTGTGCTGTATAATCAGCTCAAGATTCTTTTCAAAAATACACTTTCTTCTTTCTTCCTCTTCGCCAGTGGTGTACTTTTTTCCGTTTTTTGCTTTGAAAGATTCCCACTCGTCCCTTAAGAACTCTTGCTGAAGGGCCATGGATGCACTCGCAAGTAACACCAATAAGAGACGGCGCCGTATCATTGTAGAATGCAGATCAGTCCTTAGCTCAAACCTGCAGACCTTATCAGATCTTTGCTGAACGGTGCTTTAGCTCGTTCAGCTGGGCAGACAGAACAAGACCGTATATCTCGGAGTGCGCGATAAATGCAGGGCGTGAAATCCACTACTGTGATAGAATCACGAGGTTGTAG

>AAFM77

TTCCCCAATCCGTTCCCCAGCTTTTATTACTTCATATTAGCCGCAGCGGCTCTAGGCTACTGGAACGTATGCCCAGTTGGCGATGCCGCACTGGTTATCCTTGTCTCGGGCCATGAGTCCGTAACCGTTGTTTCCCCAATCCGTTCCCCAGCTGTTTTTGACGATCCAGTATTTCTTTCCATTTTTTTCTCCGTACCCAACAACAAGGAGAAGATGGTTGGTTGTACTCTTTGAGCACTCAGGATGGTCAAATATTTGCCCTGTTGTATGTCTGTATGTCATGAAAGCTTTCAGGCTGGCGTCTATAGCTACGGAGACTGGACCCTGAGTTGCTACCGCTATCTGGAGGTCCTTCTCTGTCGCAGGCACTTTGCGGAACTCTTTCAGGGTAGCGCCAATTGTTTTTTTATTAAACTTGCAAGGTCCTACTTCATCCTTGTAAGGGTAGCTCTTGTCTGTGTCGAGACCTCCATTATGAATGATGTAATTGAAGGAGCCGGGAGCGGAGCCACTGGTACATCCGTGATTTCCGTAAGAACTGGAGCAGTCCACGATATTTTGCGGACTTAAAACAACGAGCTTTTTTGTTTGCTTGAAATGTAGTGCTTCCAGCGCAGCAGTCGCACTGAACGCATAACAAGATCCACACGTCAGTTGATCCCTGATAGGTGTAACATAGCCTTTCTTCCGCCAGTCTACTTCTTTTGGAAGGCTACTTTCATTGAAGTTTTCCGGAGGCTGATAAGTCGGCAAGTCTTCCACTAGCATGTCTTGACCGCCGCAGCTCACTTTCTGGTTCCACTCGTCATCTGACATGTCCGCAAACTGGTTGACGCCAACTTCGTGGGACTCTAGACCTTTTGCAAATCTTTCATTGTGGTCTTTGATAAACTGGAGGGTTTCCGCAAATAACTTCCTTCTCTTGGCTTCTTCGGAATTAGAACTGTATTTTCTCTCATACTTCTCTTTGAAGGTCTTCCATTCTGCTTCCAGTCCTTCGTCCTCTGAAGCAAAAGAGAAGACAACAACTGCGGATATGAGCAGGAAGCGTGAAGTCATGTTGAACGTGCACATACGAAGGAAAAAGGCGAGTATATAAGGCTTTCCCCGCAACTAATGCAGATTTGTATTTTAGGAATTTCTAAAAGGCGAACATTTTATATGTTCATCGTGACTCTATTTTGACAAGTTGTTGCGAAATATATTTTCAGCTAAAATAACAATTTGAAGGCAATCTTTTTAAGCGCGGATTTCCAAAGGTCTGTACAATTCCCACTCAACAAAAATTGTGCCTTTGTATCTTTGCAGAGTCTACACGTTTAGTGAATGCAGAA

>AAFM98

CAATAAATCTCCACTGCGTGTAGAAAAGCGTTTATATACCCGCTCTTCTTTTCGGTACTGTCTCCATCATCGTCACCATGATGGACCTGTTGCTGATCCTATTGGGTGCTCTTGCAATCGCCTATTCTGCAGAAGACCAGGGTCTGCAAGCTGAATGGAAGGACTTCAAAGCAAAATATGGACGACAATACAAATCTGCTGCTGAAGAAGAAGAGAAATATAAAATATTTGCCGCCAACATGAAGTATATTCAAGACCACAACGTAAAGTTCACAAACGGTCAAACCACCTTTAAACTCGGAATAAACAAGTTCGCAGACATGTCGCACAGAGAGTTCGTTAAGAAGATGACTTGCTTCCGCGGAAACCGAACCGGAAGTGGTGGATCCACCTACCTGCCTCCGGCTAACTTGAATTACAGCCGCCTGCCACACACGGTCGACTGGCGTACAGAGGGCGCCGTGACTCCCGTCAAAGACCAGGACGGCTGTAGTGCCTGCTGGGCCTTCAGCGCCACTGGATCTCTGGAGGGTCAGTATTTTCGCAAGACTAGAAACCTCGTCTCGCTTAGTGAGCAAAACCTGGTCGACTGCTCCAGCGAATACCACAACTCCGGTTGCAGTGGAGGACGCGCGGACAATGCTTTTGATTACATTAAGGCCAACGGTGGCATTGACACCGAAGAAAGCTACCCATACGTAGCCCAGGACCAAGACTGCTCCTTCAAACAAGAGGACGTGGGTGCGACTTGCACAGGGTACATGGGCCTTGAGCCCGGTTCCGAGGATGCCCTGAAGTATGCTGTTGCTACGGTTGGTCCCGTTTCTGTTGCAATTGATGCGAGATCTGAGAGCTTCAAGCACTACACAGGAGGTGTGTACGATGAACCTGAGTGCACTACGACGTATCTGACCCACGCTGTTCTAGTGGTGGGCTACGGTTCGAAGGACGGCGAGAAGTACTGGCTGGTCAAAAACAGCTGGGGTGAAGACTGGGGTCTGAACGGCTACGCCTTCATGTCTCGCGACAAGGACAACCAGTGCGGCATCGCCAGTTTTGCAGTGTACCCTCTCGTCTGAGCTATTCCTACTGCCGCTGGAAAGCATCAAGCGACCACGTTGTGGACTACTTGCAGAATTAAAGCGGCTGTCACATGAAAGAAAAGTTCTTTCCCTGAAAGATATCAAGGGAAATTCCTTTTAACAGTTGAATAAAAAGCCACATTATTTCTGCAAAAAATAAGATAGAAATGAAGATGTGTTTTTGAGAAAACGAAATGGCGCAGTAACTGTCTCACATATCTCGGTGGA

>AAFM1721

TCTGCAAGCTGAATGGAAGGCCTTCAAAGAGAAATACGGAAAAAAGTACCAATCTACTGCTGAGGAAGAAGAAAGACTCCAGATTTTTGACACTAAGAGAAAGATAATTGCAAAGCATAACGAAAGATACGCAAAAGGTCTAGAATCCCATGCTCTCGGGATCAATCAGTTCTCAGATATGTCAGCAGAAGAGTGGGCGAACAAGCACAGTTGTCTCCAAGTTGACAGGGAGATTGAAAATGCAACGATGTATCAGCCGCCCAAGGATTTTGACATCAATAATCTTCCCGAAGAGGTAGACTGGCGCAAACACGGCTATGTTACGAAGGTGAAGGATCAAACCGCTAATTGTGGGTCGTGCTATGCGTTCGTTACGACTGGAATTGTGGAAGCCCAGCATTTCAAGAAGACTGGCAAGCTGGTTATTCTAAGCGAACAAAATATCGTTGACTGCACCAGTGGTGACGACTACAGAAACAAGGGCTGCGTAAAGGGCAGCGTATATTGGAGCCTAATGTATATCAAGGATCACGGAATTGACACAAACCAGAGCTATCCTTACATAGGAGATAATTGCACATGCAAGTACGACAAAAAGCATATTGGTGCGACTATAAAAAACGTAGCATTAATCAATAATACAGAACATGACTTCCAGCTTGCCGTAGCAGCTTCGGGGCCAATCGGCGTCAGCATCTTTGGAAATCACCCTGGATTTTCTGACTTATCTGGTGATGTGATTCTTGATTATCCAGACTGCAATAAAAAAAACATGACTCATGGCCTTCTTATTGTGGGATACGGGACTCGAAAAGGAAAGAAATATTGGATCGCAAAAAACAGCTGGGGAACAGACTGGGGAGCGGAAGGTTACGTGTACATAGCCCGAGGCAAGAATATGTGCGGCATATCCACCACCTGGGGATATACTGCTGTCGCCTAGCGCCACATTTTTCAAGCTAAATCAATAAAGAAA

>AAFM2087

TGATCCTACCGGCCATTGTTGCCTTCACTTTCTCTGAGCAAGACGAAGCTATGCGAGATGAATGGAAGGCTTTCAAAGCGAAATACGGACGCAAATACAAATCTACTGCTGAAGAAGACGAGAAATATAAGATATTTGCCGACAACAGCAAGTATATCGCGGACCACAACAAAAAGTTCGCTAATGGTCTGTCTTCTTATGAGCTCGCCATGAACGAGTTCGGGGACATGCGGCCGGACGAGTTTGTAAAGACGATGACTTGCCTCCGCGGAAAGCGAACAGGAAGTGGTGGTTCCACATACGTGCCTCCGGCTTACCTGAATGACAGTAGCCTGCCGGACAAGGTCGACTGGCGTGAAAAGGGGGCTGTGACTCCCGTCAAAAACCAGCGCCATTGCGG

>AAFM2147

AAAAGCCTTATATACCCGTGTTTCCCCTCGAATCTCCACGTTCACCATGATTCCACGCTTCTTACTCATATCCGCTGCTGTTGTCTTCTCTTTTGCTTCAGAAGACGAAAGTCTGCAAGCTGAATGGAAGGCCTTCAAAGAGAAGTATGGGAAAAAATACAGCTCTAATTCGGAAGAATCCGATCGAATGAAGATATTTGCGGAAACCCTCAAGTTTATCAAAGAGCACAATGAAAGATTCGCAAAAGGTCTGGAGTCCCACGATGTTGGTGTGAATCAGTTTGCGGACATGTCAGATGACGAGTGGAAGAGGAAAGTCAGCTGCTGCATTCCTCCTAGCAAGAAAAAACAATTTCCGACCTATCAGCCTCCGGAAAACATGCACCCATATCTGCCTAAAGAAAAAGACTGGACGAAAGAAGGCAAAGTTACGGAAGTGAAAAATCAAGGAGTGTGTGCATCTTGTTATGCATTCAGTGCGATTGCTGCGCTGGAAAGTCTACATGCGATCAAGACAGGAAACCTCGTCTCTCTAAGTGAGCAACAAATCATAGACTGCTCCAGACATGTGGGAAATCGTGGATGCAATGAGGGCTATCTTGACCGCACCTTCCAGTACATCATCGATAATAAAGGTGTCAACACAGAAAAGGAGTACCCTTACGTACACGGTTCCGAGAACAAAACACTCCACTGCAAGTTTGATAAATCAGCTTTCGTCGCTAGAATGAAAAGTTTCAGAAAAGTGAAGAGTGAGACAGACCTCCAGATGGCTGTAGCTTTTGATGGACCAGTCTCTGTGATAATAGACGGCAGCCTGAAAGCTTTCAAATCCTACCCGAAGAACGAATTAATATTTGACCATCCGGAGTGCTCAAAAAACAACAAAAAGGGCAAATACCATGTCGTGCTTGTTGTTGGGTATGGAGAAAAACATGGAAAGAAATACTGGAAAATCAAAAACAGCTGGGGAAAGGCATGGGGATACGACGGTTACATATATATGGCCCGGGGCAATAACCAGTGCGGCATCATCAACAACGCATTCATTCCTTCTGCCTAGAGCCACTCCGGCGAAGATGAAGCAATAAAAGTTGACTGCTTTTCCTTTTCCTGTGTATATATGCCGGATTAATAAACTACGGTCAATCTTGTTGCGGGTCAAACGCCCCTAAGACCTACCGTGTGCATACGAAAAAA

>AAFM2984

TTTTTTTTCTGATGGCAAGCACCAAATCGGTGCAGTAAAAGAAAATTTTATTTTCGTTTCTAACATGCATAATACAACGTGCGAGTTTTTTTGTTTTAAATGTGTTACGTTGCAATTCTTCATGAATACCACATAGCAGGAGTCATCTTTTAGTTCTTTTTTGCTTCAAACGTTTCTCATAGCCTCCCGTTGCGATTTTCCAAATATATCAGCAGCTGAGGTTGATTGACGCCTTTCGACAGCACATAAATTGGCGCGTGCTGCTTTCAATCTGTCCCNTCCTCTGTCCAGTCGGCGAAATGATTCACGCCAACTTTGTCTGATACTTCGCCCTTTCTGTATCTTTCATTGTGATGGATGATGAAGCGCAAGGTCTTTTCGAACAGCTTCATCCTATAGCGATCCTCTTCCCCATAGTACGTCCGATTGTATTCTTTCTTGAAAGTTCGCCACTTGTTCTCCAAGGAGTCATTGGATTCATAATATGACTCGTCGATATATGCAAGCACGGTGGGCAGCAAGACACAAAGTAGAATCATGTTGACAAGAACCGGAACCTGAAAATAATAAAGGCTAGCACGTTGTGCTCGTGTTGAGGTCTATTTGTG

>AAFM18552

AGCATGTCTCCAAACTCGTTCATGGCGAGTTGAAAGGAGGCTTCACCATTTGCAAACATTTTGTTGTGGCCATCTATGTACTTCTTGTTGGCGGCAAATATCTTATATTTCTCTTTTTCTTCAGCAGCAGATTTGTATTTGCGTCCGTATNNNNCTTTGAAAGCCTTCCATTCATCGTGCACAGTTTCGTCTTCTGCAGAATGTGCAATTGCCATAATAGCCGATAGAATCAGCAATAGATGCATCATGGTGAAGC
