# Supplementary material for: A repertoire of protease inhibitor families in Amblyomma americanum and other tick species: inter-species comparative analyses
Source: Parasit Vectors. 2017 Mar 22;10:152. doi: 10.1186/s13071-017-2080-1 (PMC5361777; doi:10.1186/s13071-017-2080-1)
Supplement: Supplementary file 3 — FASTA sequences for Amblyomma americanum contigs from Illumina sequencing, by PI family. (ZIP 638 kb) [file 13071_2017_2080_MOESM3_ESM.zip › A. americanum I32.docx]

>AAFF11569

AGATGATGCAGGTGTGTGGACACCCGGTGCAATGTCCACACACCTGCATCATCTCAAGTTTTAAAGGAAAACGACCTTTTGCTTCACCGACTCCAGGCGTCAGAGGAGGAGCGCTTCAACACGTTTTCGGATTGGCCGTTGGACTACCTGTCACCGCGGTTACTGGCCCAGGCAGGCTTCTACTACCTTCACGAGCAGGACAAGGTGCGGTGTGCCTTCTGCCGCGGCACGGTGCACAACTGGGAGCGTGGGGACGATCCGCTGAGGGAGCACGCGCGGCACTACCCGTGCTGCCCTTTCCTGCTGGACCCCAGCCTGGCCGGCCAGGACGAGTGCGGCCACGAGTCTCGGCACCGGTCTCGCTCAGTGCCCGAGGGCCGCCACCTGCTCATCGGAGCAAACGGTTCCCAGGGGGTGCAGCTGAAAGGGGACACCCCTCCGAGTGAACTTTCTGGCCTGGGTGTGTCTGTACACGTCGGCCCGAAGCACCCATCGCAAGCGTCCCCCGACGCCCGGTTACGTACGTTTGAAAAGTGGCCTTCCACGTGTGCGAAGAGACCACTGGAGCTGGTGCAAGCTGGGTTTTTTTACATTGGTGTGCAGGACTACACCAAGTGCTTCCACTGCGATGGTGGCCTTTGCAACTGGGACTCCGGGGACGACCCGTGGGAAGAGCATGCCCGGTGGTTCCCCCGCTGCCAATTTGTCTTGCTCGCCAAGGGGGAGGCGTACATCAATGACTGCCTGCGGAGACACCAGTCGCATCTCAACTCGGTTGCTGCCTCAACGAGCTCTCAGCAAGGGCAGACAGGAGCGGCGGATGAGGGCATGGCGACAGAGCTGGCGGCATTGATGCGGTCGGACGACGTGCAGTTCTACCTGTCGCAAGGGGTTCCTGCAGAGACACTGCGTGCTGCACTGCTGCGGCATATGCGTGGCCAGGGCCGAGGTTTCGCCAGCCGCGACGAGCTGCTGCAGGTCCTTGGCGAGCTGCTTGCCCTGCCCAAGGCGTCTGCTGACCAGACACCGCAGGAACGGGCCACCAATGGGGCTTCCTCCAAGAATATCTTTTCTGGCACTGGAAAGCAGTCGTCACCGGTGGCCGTGACGCCAGCAGGGTCCGAGCCCTCGGACCCGGAGGAAACGCGGCTCAAGGATCAGCGGCTGTGTAAGGTGTGCCTCGATGCTGAGGTAGGCGTCGTGTTCCTGCCGTGCGGCCACCTCGTTGCGTGCCCGTCGTGCGCCTCGGCTCTCGTAGACTGCCCCATCTGCCGCGCAGCCATCCGCGGCACCGTGCGGACATTCTTTGCATGAAACGCCTTGGTGCGAAGGAAGAGTGCATTGGGTGAAAAAGAAAGATGGCAGAGTTTCGGTGATGTGTTCATCCAGAGTGCTCCGACGGTGGCCGGGTGGGTCGAACCGAGGGGAGTAGCCGAGCACTGCTGTGCTTGTGAGAAGCGGCTGCAGCGTTCATGCCTGTCAGCTGTGAAGCAAGCGGTAAGGCACCGATGCAGTGAAAAAGCAAGCATTGGTAGACCCTGCAAAGAGCACTTGTCATGCCCATACAAGCATTGGTGGCGAAATCAGAGGGGGCTTCCGCTTCTACGAAGGGATTGGGAAGAACGCCTTGATGTGCTGAACGTTCCCTCTAGCATGGCCCCTCTGGCTTTTATTCTCGTTAGTCCTACTTGTAGCTGACCTGTAGGCTGTTGTGGCGGCGTTTCAGTGTTTTTAAACCGTGGTGTTTTGTGATGCTCTTGTTTCAAGTTTCCCCTGGTCCTTTTTCAACCCAAATTCTGGTCTCACCCCACGCTGCCCCCACCTCCTCTGCCCAAGCAGTCCGGTCCCGCGGCAGGTCTTGAAACACCTGCCACCCTGTATTTAGTGGAAGGACTCGTGGCTAGGTGAGTCCGCAGGGCATGCACGTGTTGTGGAAGGTAGCACTCATAGGCAAACAGAGAGGATGCAAGAGAACAAGGAAGACTGCAAGTCGTGCGTCCGGTTCCCTTGGGGTTGCCTCTGAATGCAGCCTTTCACAGTGCTGCCTGCTTCCGTGGGAGGTAGCTGTAGAGCTGCTGTTACCTCTACAGTTCTGACTGGTAGTGCTTGCAGATTGGCACATTTCAAAGAAA

>AAFF31037

CAACACATTTATTGGTGACACCAAAGGCTACTTCATAACACAAAGGTGAGATCCGAAAGCACTGAATAGCATGCAAGTCGCATCGGCGCCACCTAAATTCGCAGCGCAGCATACAACGGCTGACGATGCAAAAGCGCCATTTGCTTGGCTCGCCAGTAAGTGCACGTTATACGGAGCACGGACCCATAACGCCGGGTAAAAGATTAGGAAAAAATGCGTGGATTTGCGGATGGGGCGACGAAATCGTGGTCAACGCATCTTCCTACGCACCTTGCCCAGTTCGTAGGTGACCTTGCGCACAGCCTCTGCGACTTCCGTTTGGTGCAGCTCCACGAACTTGTTGGCTCTGTTCTTGGTGCGGGCCTTCTCGAGGTGCATGTAGTCGACGGCAGTCATTTCCCGGGCCTTCTTGCCCAAGCGAACAAATTCGCAGTTAACGGAGCGCGAGTGCTCCTTGGACGGGTCGTCGCTCGGATCCCAGCCGTCGAGCTCCTTGAAGCAGACGTAACAGCGAGCAAGGTCGGGCTCGTTCTCTGTAGGACAATAGTAGAAGCCCGCTTCAGCCATGCGCTCGGGCGTGCACATGCAGTCGCCCGTCAGCGGCCACTGATCGAAGGAAGCCAGGCGATTCTCGACAGAGTGCATGTCTTTGTCCGTTTGAAATATCAGCACGGCTTGAAGGGACAAGTCGACGGGCGGCTTCGATTTTGACGACCCGGTCATGGCGTCCACTGAACGGCGCCGACTGACGGGAGTGGGACCAACCTGTTCTTGGCACAGATCAAATGCAGCACCACTTTCCAAACGACGGCTGCCTGGAAACCGTCGAACGTGTACAATAACACATATAACCAAAAAATGTAATGCAACAGAGTAACACCAGTAACGCCGCAATATAGCCGCGATAACCGCAATTTCAATTTGAAAATTTCAAATAGGACCCGCCGAATGGCGAGCGCGCGAACGTT

>AAFF31402

GACAGTCGACTTGCATAGGGGTCGAATGCCTGGTGTTGTCGAATGAAGGGGGTGCTGTCGTAGCGGAGACTGGGCTCTGCGCAGCAGAGGCTGGCGCAGGCAGGGAGCGCACAAAAGCGCAATCCGGCCGAGCTTGCCGATGTCGCTTCACGACCTCGTCGGCGATCTTCCAGTCCCCAATTTCCAGCCCACACTGAAAGCACCGTGTGTGGTAGTCGTGACCCGCGTACACGAAACCACCCTGTGCCAGCCTCTTGGCGGGGATTGGGGCGTTAAGGGGCCACCCATGAAACGAGGCGATCCGGACATCTTCCTGGCTCAGGTCCGGGCTACGGCTGACGG

>SG1208099

AGGTGTTTCATGCAAAGAATGTCCGCACGGTGCCGCGGATGGCTGCGCGGCAGATGGGGCAGTCTACGAGAGCCGAGGCGCACGACGGGCACGCAACGAGGTGGCCGCACGGCAGGAACACGACGCCTACCTCAGCATCGAGGCACACCTTACACAGCCGCTGATCCTTGAGCCGCGTTTCCTCCGGGTCCGAGGGCTCGGACCCTGCTGGCGTCACAGCCACCGGTGACGACTGCTTTCCAGCGCCAGAAAAGATATTCTTGGAGGAAGCCCCATTGGTGGCCCGTTCCTGCGGTGTCTGGTCAGCAGACGCCTTGGGCAGGGCAAGCAGCTCGCCAAGGACCTGCAGCAGCTCGTCGCGGCTGGCGAAACCTCGGCCCTGGCCACGCATATGCCGCAGCAGTGCAGCACGCAGTGTCTCTGCAGGAACCCCTTGCGACAGGTAGAACTGCACGTCGTCCGACCGCATCAATGCCGCCAGCTCTGTCGCCATGCCCTCATCCGCCGCTCCTGTCTGCCCTTGCTGAGAGCTCGTTGAGGCAGCAACCGAGTTGAGATGCGACTGGTGTCTCCGCAGGCAGTCATTGATGTACGCCTCCCCCTTGGCGAGCAAGACAAATTGGCAGCGGGGGAACCACCGGGCATGCTCTTCCCACGGGTCGTCCCCGGAGTCCCAGTTGCAAAGGCCACCATCGCAGTGGAAGCACTTGGTGTAGTCCTGCACACCAATGTAAAAAAACCCAGCTTGCACCAGCTCCAGTGGTCTCTTCGCACACGTGGAAGGCCACTTTTCAAACGTACGTAACCGGGCGTCGGGGGACGCTTGCGATGGGTGCTTCGGGCCGACGTGTACAGACACACCCAGGCCAGAAAGTTCACTCGGAGGGGTGTCCCCTTTCAGCTGCACCCCCTGGGAACCGTTTGCTCCATTGAGCAGGTGGCGACCCTCGGGCACTGAGCGAGACCGGTGCCAAGACTCGTGGCCGCACTCGTCCTGGCCGGCTAGGCTGGGGTCCAGCAGGAAAGGGCAGCACGGGTAGTGCCGTGCGTGCTCCCTCAGCGGATCGTCCCCACGCTCCCAGTTGTGCACCGTGCCGCGGCAGAAGGCACACCGCACCTTGTCCTGCTCGTGAAGGTAGTAGAAGCCTGCCTGGGCCAGTAACCGCGGTGACAGGTAGTCCAACGGCCAATCCGAAAACGTGTTGAAGCGCTCCTCCTCTGACGCCTGGAGTCGGTGAAGCAAAAGGTCGTTTTCCTTTAAAACTTGAGATGATGCAGGTGTGTGGACACCCGGTGCAATGCCCACACGAGGTTGCGTCAGACAGTCGACTTGCATAGGGGTCGAATGCCTGGTGTTGTCGAATGAAGGGGGTGCTGTCGTAGCGGAGACTGGGCTCTGCGCAGCAGAGGCTGGCGCAGGCAGGGAGCGCACAAAAGCGCAATCCGGCCGAGCTTGCCGATGTCGCTTCACGACCTCGTCGGCGATCTTCCAGTCCCCAATTTCCAGCCCACACTGAAAGCACCGTGTGTGGTAGTCGTGACCCGCGTACACGAAACCACCCTGTGCCAGCCTCTTGGCGGGGATTGGGGCGTTAAGGGGCCACCCATGAAACGAGGCGATCCGGACATCTTCCTGGCTCAGGTCCGGGCTACGGCTGACGGAGGCCATGCTTGAAAACTTGACCCCCAGTGGCCCATCAGTCACCATGCGCTCAGGGGCGGGCCGGAACGCGGGTGGCTGAGCCAACCGTTCTGCCACTGTATGTACCATCATGGGNNNNNNNNNNNNNNNNNNNGCCATTGAGAACTTCAACAGCCACGATGTGTGACAGTCGGGATGCCGGGCGAATGCCTGAATGGTCCAGAAGGAATTTCATTCTACCGTCGCATGACTAAATTCACATGCCAGCGATAACGGACGCTCCCCCTCGGAGGAGTCAGCGCTGGCTTATCCATTGCACGTGGCGGGGGCGAAACCCTCCTCCACACCACCAACCG

>SG12041493

CAGGTTGGTCCCACTCCCGTCAGTCGGCGCCGTTCAGTGGACGCCATGACCGGGTCGTCAAAATCGAAGCCGCCCGTCGACTTGTCCCTTCAAGCCGTGCTGATATTTCAAACGGACAAAGACATGCACTCTGTCGAGAATCGCCTGGCTTCCTTCGATCAGTGGCCGCTGACGGGCGACTGCATGTGCACGCCCGAGCGCATGGCTGAAGCGGGCTTCTACTATTGTCCTACAGAGAACGAGCCCGACCTTGCTCGCTGTTACGTCTGCTTCAAGGAGCTCGACGGCTGGGATCCGAG

>SG966051

TCAGAAGCAATAAAAACATATTTACATGGGCAGTATACTACAAAATATAGTGTAGGCTATAGCATGTAAGAGACATGATTGACTAAGAAAAACCGTTTCCGTGCATAAAGCGATAGGCTATTTGTGCAAAGCATAAGCATTTACATAATCACCCAGACTGGGTGGAAATTCGCACACACGTCCGACGTGAAGAGTGGTAATAACCGTGTGATAAAAAAAATCGCCTAGCTACATGCAACTGTACATTTCACACATGAAATGCGCATTTACATAATTTTCACAATAGGCTAAAACACGTCTGACGTCCACACATAACGTCACGATAAAAAAGAAAATCGCTACGCTGCTTGTACGCTGTTCTTAAATAGCGTATATGAGTCCGAATAACTAGCTCCGTACTCTTTTCAGTTCCGATATAACATGTAGGAATAGGCATGTGCATAGAGTGATTCGCTCATTAATGAATGACGCAAACACAAATAATAAAATATACTTGATAAATTGCAGTCTAGTGCCTAGTGAACGGTGCAGCATGTTCATGTGCCTGAATGTGGTTGCTATCATACTTTATACACGCACCACTAAGGATGGTGCAAAATAGCTCCTTTTTGATCCTACCATGATTTGTTTCTTTTCGCTCTTTGCAAACACCTCAATGGATGCAATGCCTTCCTTTGGCTGAGCTCGAGTCAATAACAGAAGGAGCACGAAGCATGCCGCTACTTTGTTTGGTTTATTGGCTTAACATCCCAAGGCAACTTGGGCTATGGGGGATGCTGTAGTGGAAGGCTCAGGATAATTTCGACCACCTGGGGTTCTTTAACATGCACCGTCATCACACAGTACAAGGGCACCTAGAATGTCGCCTCCATTGAAATGAGACCGCCATGGCCAGGATCGAACCCGCATCTTTTGGGTCAACAGCCGAGCGCCATAACCACTNNNNNNNNNNNNNNNNNNNNNNGCCACCGTGGTGGCTGGAGCTACTTTAGTTAATCCCACAAAGTAAAAGAAAATTGTCATAGAATATAAGTGGAATTGTTATATTATACGCGCCTAGGTTTCATGCTGCCTTTTCTACTCCTAGGTGAGAATTGGCTGCAAAAATCCAGAGACAACAGCTGCATCTGATGCTGCACAGCTTTTGTTTCCATGAGGCAAGAAGGTGTGAAGTTCACGTAAGAACAACACAGAGGCATGAATATCTTTGCCAATGTATGGCCTTCTTGTATTATATTCATTTTCTTTTTCTCCTGTTTGGCACTGCCTCCTTGGTCCAAACAAAGTCTGCAGTACAGCACAAGAATAAAACTAAAAACAAAAAGGAGCGAAGCCCTGGCACAAATACAGGCATGCATGATACCAGTGCATATATATGCCAGAGATGGCATATTTGCCAGTGGAACCACTGCTTTCATTTGAACACTGTTACTCTACACCTTTTGCCCCCACAGGTATAACCGGAACTGACGAAACCTGCATGACGATTAAATATTTGGACGAGAAAGCAGCAAATGGGTCTGTGAAAGAGCAGCTGTTCACGTCGACAAAGCAAGCGAGGACTGTAGATGCAAAAGATATACTGTCATTCAGGACAAGCCAGTTATATACCTAATGGCACATACAAATACATATTTCTGATTAATTTTTTTACTCGTGCCAACGTAGTATTTGCCAGTGAGGATGGCTGCCCAATGCTTTGCGCCAACAAAGAAAGCCTTATATCTTTAAGCGGGCACTGATCCTCCTCCTAAGCACACACAAATCTGTTCCCAGAAGAGAATGAAAGAGACCATTATACTAGCTTGCAAAACTGAAGTAAATGATACAACACGTGCTTTGGACAACCATGCCGCATTTTCTGGTGTGTAAGGTGCACCCCGTGCAAATGACAGTTTTTCCGAAGAAGCAAAAAAAAAAAAGCGTTATTTCCACCAGTGAAAAGATACACCTTTTCATCTGGCAAGCACAATGAAAACCAGGTGAAGATCCCGTAGCAACTATGAACTGCAATGCACAATGCACGTGAAGCCAGCTGCTACGAATGCGATAAAGACAACATGGCAAAAGTGACACTGACGAAACTGACAGCAAAAATTAAACTTGCGAGGATATGCAAGTAGACCCCCAGAACAACATTTTAGAAATAGCTTCGTTTCTTTCCAGTTTTACAGATTGATTTTCTTCACAAGTGTGGCTAATGGGCACAGTGAAAGACATGCAATGGCGCGGCATTTTTCGTCGTATGTAAAAAAGCGCAACTTACACACGAGAAAGCACGGTACTGCATTCACAAAAAAATCAGTACGATGCACAATATAAGATTTTAATTGTCTCCAGCCACTTCAGCATTCCTGTTTCTAAGCTGTTTACAGTAAATGCACTGTTGCTACATAAAGAAATAAAAGCAGGCGAACACAAATTAGAAATTGGATTATACTAACCTGTGGAACACCTTTTCACCATGCTATTTTGCTGCATAGCAGCAACCATGCTGTACATTCACACCACAATCACTACTACCTTATGGAGTAAGATTAATTAACTGCACAACACCAAATAGAAAAAACTTCAGCGTTCCTGTCCACTTCTTTAGTTTTTTTTACATTTATTAATAGTAATAAGTTTCTCATGTATGAGGTTCTAAACACACTGATACCTTGCACAATTAGTAACAAGCATTCAGTGCCACTATTCTACTGTAGAATATCTGTAGTAGCCCTGCAGTAAACTGCTAGAAGTGTTGTATGTACAGCAGCATTATTTCTGCACAGCATGTACAAAGTCCAATGCCGCAATTACTGCTGTAAGGAGAGACAGAAACTGCAGTGACATTTTATTTCAACAAAATATGCATGTAACCACTAGCAAGTAAAATTCGTGCATTGTGGACATGACATAACCGACTAAACCAACGCTGCTTGCCTGCAAGCGCGCTACGGTTGTGTTTGTGAAGACAGAAGATAATTTTACGTTTCGTGCAACAACTCTTGCACAAAAAAGCACAACTAATTATTAAAACCAACCTATGAACTCAATCGAGTAAGAAATAACCAAGAGCAATTACTGGAAACAGACTATACTGCACAGCAGTGCTTCATAACACAAGTATGTGTGCTAGCTGCCGTGTAGTATGTGCGTGCGCATGCAGATGCATGTGTGTGTTAGTTTCTTTAATGCTCACTCAGCAAATGAAACATAGAAATGCACCCAGTGACTTCGCAATCACAAACAAGAACAATGACGAAAGTGAAGACGAACGTTCTGAACCATGACAAGACAAAAGGGAAAAAGGCAGGACGTTGCCCTCGACTCCTTTGCACAGCAAACACACATTCTTGCCTATTTCCCTGACATAATGGGAGCCCCATATCAGTAGCACAAATGTGAATGTGCTTGCGTGATTAAAAAATTGGCTATGGTTTAGCTCTGGTTAAACCTGGAGTGACGCGAGAGCTACATCTGGCAGAGTGGAACTCGCTCAGTCGAATTGCAAAGTTGGTCTTTCGCCGCTCTGTTTCGCTGGGCGTCGTTCCTTCATCTTCGTCCCTGGTTGTGGATTCACTGCCCCGCCCTCCCCCACTCGGTTTCGCCGGTGCGCCACAGGCAGCTGCTCCGCACCACGTGACCGACCACGTGACCAGCCACGCCGCCGCCACAGAGCTCAAGGGCGGCCACGAAACTCAAGGGGTGCCACGCTGAAGGCTCGAAGTGCTAGCGTAATGTAGCTATCCCTACAAAAGCCGCGTGAGCGTGCATATTTTGCTCCAGGAAGTTACCATTTAACTACGTACGACTAGAGACTGGCGACTAGGGACTCTGAACGGGCACTCCCAAAAAATAAAAGACCACACAGCTTAGTGCAGCCGCATTTCAGGCGGGCTCCCAACGTTTCTGCCTGTGGACCATACTGTTTCACGAGTCTACTGCCACGCACTGCAAAACAAAGTCTACAGATTGCAACTTTAGATCGTGCAGCTTCCATTTCTGGTTGCACTAACCTCTGCACTACAGAAAACCTAAGGAGGTCAGAAGTCTTTTTTTTATTTTTTTCCTTGCTTGTAAGTCCGTGCGCTTAGATGCGCTCTTACTTTCTTTGAAATGTGCCAATCTGCAAGCACTACCAGTCAGAACTGTAGGGGTAACAGCAGCTCAACAGCTACCTCCCACGGAAGCACGCAGCACTGTGAAAGGCTGCATTCAGAGGCAACTCCAAGGGAACCGGACGCACGACTTGCAGTCTTCCTTGTTCTCTTGCATCCTCTCTACCTTCCACAACACGTGCATGCCCTGCGGACTCACCCAGCCACGAGTCCTTCCACTAAATACAGGGTGGCAGGTGTTTCAAGACCTGCCGCGGGACCGGACTGCTTGGGCAGAGGAGGTGGGGGCAGCGTGGGGTGAGACCAGAATTTGGGTTGAAAGAGGACCAGGGGAAACTTGAAACAAGAGCATCACAAAACACCACGGTTTAAAAACACTGAAGCGCCGCCACAACAGCCTACAGGTCAGCTACAAGTAGGACTAACGAGAATAAAAGCCAGAGGGGCCATGCTAGAGGGAACGTTCAGCACATCAAGGCGTTCTTCCCGACCCCTTCGTAGAAGCGGAAGCCCCCTCTGATTTCGCCACCAATGCTTCTATGGGCATGACAAGTGCTCTTTGCAGGGTCTACCAATGCTTGCTTTTTCACTGCATCGGTGCCTTACTGCTTGCTTTACAGCTGACAGGCAGGAATGCTGCAGCCGCTTCTCACAAGCACAGCAGCGCTCGGCTACTCCACTCGGTTCGACCCACCCGGCCACCGTCGGAGCACTCTGTATGAACACATCACCGAAACTCTGCCATCTTTCTTTTTCACCCAATGCACTCTTCCTTCGCACCAAGGTGTTTCATGCAAAGAATGTCCGCACGGTGCCGCGGATGGCTGCGCGGCAGATGGGGCAGTCTACGAGAGCCGAGGCGCACGATGGGCACGCAACGAGGTGGCCACACGGCAGGAACACGACGCCTACCTCAGCATCGAGGCACACCTTACACAGCCGCTGATCCTTGAGACGCGTTTCCTCCGGGTCCGAGGGCTCGGACCCTGCTGGCGTCACGGCCACCGGTGACGACTGCTTTCCAGCGCCAGAAAAGATATTCTTGGAGGAAGCCCCATTGGTGGCCCGTTCCTGCGGTGTCTGGTCAGCAGACGCCTTGGGCAGGGCAAGCAGCTCGCCAAGGACCTGCAGCAGCTCGTCGCGGCTGGCGAAACCTCGGCCCTGGCCACGCATATGCCGCAGCAGTGCAGCACGCAGTGTCTCTGCAGGAACCCCTTGCGACAGGTAGAACTGCACGTCGTCCGACCGCATCAATGCCGCCAGCTCTGTCGCCATGCCCTCATCCGCCGCTCCTGTCTGCCCTTGCTGAGAGCTCGTTGAGGCAGCAACCGAGTTGAGATGCGACTGGTGTCTCCGCAGGCAGTCATTGATGTACGCCTCCCCCTTGGCGAGCAAGACAAATTGGCAGCGGGGGAACCACCGGGCATGCTCTTCCCACGGGTCGTCCCCGGAGTCCCAGTTGCAAAGGCCACCATCGCAGTGGAAGCACTTGGTGTAGTCCTGCACACCAATATAAAAAAAACCCAGCTTGCACCAGCTCCAGTGGTCTCTTCGCACACGTGGAAGGCCACTTTTCGAACGTACGTAACCGGGCGTCGGGGGACGCTTGCGATGGGTGCTTCGGGCCGACGTGTACAGACACACCCAGGCCAGAAAGTTCACTCGGAGGGGTGTCCCCTTTCAGCTGCACCCCCTGGGAACCGTTTGCTCCATTGAGCAGGTGGCGACCCTCGGGCACTGAGCGAGACCGGTGCCAAGACTCGTGGCCGCACTCGTCCTGGCCGGCTAGGCTGGGGTCCAGCAGGAAAGGGCAGCACGGGTAGTGCCGTGCGTGCTCCCTCAGCGGATCGTCCCCACGCTCCCAGTTGTGCACCGTGCCGCGGCAGAAGGCACACCGCACCTTGTCCTGCTCGTGAAGGTAGTAGAAGCCTGCCTGGGCCAGTAACCGCGGTGACAGGTAGTCCAACGGCCAATCCGAAAACGTGTTGAAGCGCTCCTCCTCTGACGCCTGGAGTCGGTGAAGCAAAAGGTCGTTTTCCTTTAAAACTTGAGATGATGCAGGTGTGTGGACACCCGGTGCAATGCCGACACGAGGTTGCGTCGGACAGTCGACTTGCATAGGGGTCGAATGCCTGGTGTTGTCGAATGAAGGGGGTGCTGTCGTAGCGGAGACTGGGCTCTGCGCAGCAGAGGCTGGCGCAGGCAGGGAGCGCACAAAAGCGCAATCCGGCCGAGCTTGCCGATGTCGCTTCACGACCTCGTCGGCGATCTTCCAGTCCCCAATTTCCAGCCCACACTGAAAGCACCGTGTGTGGTAGTCGTGACCCGCGTACACGAAACCACCCTGTGCCAGCCTCTTGGCGGGGATTGGGGCGTTAAGGGGCCACCCATGAAACGAGGCGATCCGGACATCTTCCTGGCTCAGGTCCGGGCTACGGCTGACGGAGGCCATGCTTGAAAACTTGACCCCCAGTGGCCCATCAGTCACCATGCGCTCAGGGGCGGGCCGGAACGCGGGTGGCTGAGCCAACCGTTCTGCCACTGTATGTACCATCATGGGCGCCTCGCGTGTCCTGTTTTTAGTTAGGTTCCAAACTGATCACGCGAAGACGCACTATGGTACTCCGGCCATAGGCTATGCTCCACAGCACGCCAGCAACGAGCGCGCTCGAGAGGGGGAAAGGGAACG

>SG9619100

GCCGTGCTGATATTTCAAACGGACAAAGACATGCACTCTGTCGAGAATCGCCTGGCTTCCTTCGATCAGTGGCCGCTGACGGGCGACTGCATGTGCACGCCCGAGCGCATGGCTGAAGCGGGCTTCTACTATTGTCCTACAGAGAACGAGCCCGACCTTGCTCGCTGTTACGTCTGCTTCAAGGAGCTCGACGGCTGGGATCCGAGCGACGACCCGTCCAAGGAGCACTCGCGCTCCGTTAACTGCGAATTTGTTCGCTTGGGCAAGAAGGCCCGGGAAATGACTGCCGTCGACTACATGCACCTCGAGAAGGCCCGCACCAAGAACAGAGCCAACAAGTTCGTGGAGCTGCACCAAACGGAAGTCGCAGAGGCTGTGCGCAAGGTCACCTACGAACTGGGCAAGGTGCGTAGGAAGATGCGTTGACCACAATTTCGTCGCCCCATCCGCAAATCCACGCATTTTTTCCTAATCTTTTACCCGGCGTTATGGGTCCGTGCTCCGTATAACGTGCACTTACTGGCGAGCCAAGCAAATGGCGCTTTTGCATCGTCAGCCGTTGTATGCTGCGCTGCGAATTTAGGTGGCGCCGATGCGACTTGCATGCTATTCAGTGCTTTCGGATCTCACCTTTGTGTTATGAAGTAGCCTTTGG

>SG486605

TGGCCGGCCAGGACGAGTGCGGCCACGAGTCTCGGCACCGGTCTCGCTCAGTGCCCGAGGGCCGCCACCTGCTCATCGGAGCAAACGGTTCCCAGGGGGTGCAGCTGAAAGGGGACACCCCTCCGAGTGAACTTTCTGGCCTGGGTGTGTCTGTACACGTCGGCCCGAAGCACCCATCGCAAGCGTCCCCCGACGCCCGGTTACGTACGTTTGAAAAGTGGCCTTCCACGTGTGCGAAGAGACCACTGGAGCTGGTGCAAGCTGGGTTTTTTTACATTGGTGTGCAGGACTACACCAAGTGCTTCCACTGCGATGGTGGCCTTTGCAACTGGGACTCCGGGGACGACCCGTGGGAAGAGCATGCCCGGTGGTTCCCCCGCTGCCAATTTGTCTTGCTCGCCAAGGGGGAGGCGTACATCAATGACTGCCTGCGGAGACACCAGTCGCATCTCAACTCGGTTGCTGCCTCAACGAGCTCTCAGCAAGGGCAGACAGGAGCGGCGGATGAGGGCATGGCGACAGAGCTGGCGGCATTGATGCGGTCGGACGACGTGCAGTTCTACCTGTCGCAAGGGGTTCCTGCAGAGACACTGCGTGCTGCACTGCTGCGGCATATGCGTGGCCAGGGCCGAGGTTTCGCCAGCCGCGACGAGCTGCTGCAGGTCCTTGGCGAGCTGCTTGCCCTGCCCAAGGCGTCTGCTGACCAGACACCGCAGGAACGGGCCACCAATGGGGCTTCCTCCAAGAATATCTTTTCTGGCGCTGGAAAGCAGTCGTCACCGGTGGCTGTGACGCCAGCAGGGTCCGAGCCCTCGGACCCGGAGGAAACGCGGCTCAAGGATCAGCGGCTGTGTAAGGTGTGCCTCGATGCTGAGGTAGGCGTCGTGTTCCTGCCGTGCGGCCACCTCGTTGCGTGCCCGTCGTGCGCCTCGGCTCTCGTAGACTGCCCCATCTGCCGCGCAGCCATCCGCGGCACCGTGCGGACATTCTTTGCATGAAACGCCTTGGTGCGAAGGAAGAGTGCATTGGGTGAAAAAGAAAGATGGCAGAGTTTCGGTGATGTGTTCATACAGAGTGCTCCGACGGTGGCCGGGTGGGTCGAACCGAGTGGAGTAGCCGAGCTCTGCTGTGCTTGTGAGAAGCGGCTGCAGCGTTCATGTCTGTCAGCTGGGAAGCAAGCGGTAAGGCACCGATGCAGTGAAAAAGCAAGCATTGGTAGACCCTGCAAAGAGCACTTGTCATGCCCATAGAAGCATTGGTGGCGAAATCAGAGGGGGCTTCCGCTTCTACGAAGGGGTCGGGAAGAACGCCTTGATGTGCTGAACGTTCCCTCTAGCATGGCCCCTCTGGCTTTTATTCTCGTTAGTCCTACTTGTAGCTGACCTGTAGGCTGTTGTGGCGGCGTTTCAGTGTTTTTAAACCGTGGTGTTTTGTGATGCTCTTGTTTCAAGTTTCCCCTGGTCCTCTTTCAACCCAAATTCTGGTCTCACCCCACGCTGCCCCCACCTCCTCTGCCCAAGCAGTCCGGTCCCGCGGCAGG

>SG4817490

GGCAGCACGGGTAGTGCCGCGCGTGCTCCCTCAGCGGATCGTCCCCACGCTCCCAGTTGTGCACCGTGCCGCGGCAGAAGGCACACCGCACCTTGTCCTGCTCGTGAAGGTAGTAGAAGCCTGCCTGGGCCAGTAACCGCGGTGACAGGTAGTCCAACGGCCAATCCGAAAACGTGTTGAAGCGCTCCTCCTCTGACGCCTGGAGTCGGTGAAGCAAAAGGTCGTTTTCCTTTAAAACTTGAGATGATGCAGGTGTGTGGACACCCGGTGCAATGCCCACACGAGGTTGCGTCAGACAGTCGACTTGCATAGGGGTCGAATGCCTGGTGTTGTCGAATGAAGGGGGTGCTGTCGTAGCGGAGACTGGGCTCTGCGCAGCAGAGGCTGGCGCAGGCAGGGAGCGCACAAAAGCGCAATCCGGCCGAGCTTGCCGATGTCGCTTCACGACCTCGTCGGCGATCTTCCAGTCCCCAATTTCCAGCCCACACTGAAAGCACCGTGTGTGGTAGTCGTGACCCGCGTACACGAAACCACCCTGTGCCAGCCTCTTGGCGGGGATTGGGGCGTTAAGGGGCCACCCATGAAACGAGGCGATCCGGACATCTTCCTGGCTCAGGTCCGGGCTACGGCTGACGGAGGCCATGCTTGAAAACTTGACCCCCAGTGGCCCATCAGTCACCATGCGCTCAGGGGCGGGCCGGAACGCGGGTGGCTGAGCCAACCGTTCTGCCACTGTATGTACCATCATGGGTGCC

>SG4841123

GTCGTCGCTCGGATCCCAGCCGTCGAGCTCCTTGAAGCAGACGTAACAGCGAGCAAGGTCGGGCTCGTTCTCTGTAGGACAATAGTAGAAGCCCGCTTCAGCCATGCGCTCGGGCGTGCACATGCAGTCGCCCGTCAGCGGCCACTGATCGAAGGAAGCCAGGCGATTCTCGACAGAGTGCATGTCTTTGTCCGTTTGAAATATCAGCACGGCTTGAAGGGACAAGTCGACGGGCGGCTTCGATTTTGACGACCCGGTCATGGCGTCCACTGAACGGCGCCGACTGACGGGAGTGGGACCAACCTGTTCTTGGCACAGATCAAATGCAGCACCACTTTCCAAACGACGGCTGCCTGGAAACCG

>MG12016306

GCTCGGCTACTCCACTCGGTTCGACCCACCCGGCCACCGTCGGAGCACTCTGTATGAACACATCACCGAAACTCTGCCATCTTTCTTTTTCACCCAATGCACTCTTCCTTCGCACCAAGGTGTTTCATGCAAAGAATGTCCGCACGGTGCCGCGGATGGCTGCGCGGCAGATGGGGCAGTCTACGAGAGCCGAGGCGCACGACGGGCACGCAACGAGGTGGCCGCACGGCAGGAACACGACGCCTACCTCAGCATCGAGGCACACCTTACACAGCCGCTGATCCTTGAGCCGCGTTTCCTCCGGGTCCGAGGGCTCGGACCCTGCTGGCGTCACGGCCACCGGTGACGACTGCTTTCCAGCGCCAGAAAAGATATTCTTGGAGGAAGCCCCATTGGTGGCCCGTTCCTGCGGTGTCTGGTCAGCAGACGCCTTGGGCAGGGCAAGCAGCTCGCCAAGGACCTGCAGCAGCTCGTCGCGGCTGGCGAAACCTCGGCCCTGGCCACGCATATGCCGCAGCAGTGCAGCACGCAGTGTCTCTGCAGGAACCCCTTGCGACAGGTAGAACTGCACGTCGTCCGACCGCATCAATGCCGCCAGCTCTGTCGCCATGCCCTCATCCGCCGCTCCTGTCTGCCCTTGCTGAGAGCTCGTTGAGGCAGCAACCGAGTTGAGATGCGACTGGTGTCTCCGCAGGCAGTCATTGATGTACGCCTCCCCCTTGGCGAGCAAGACAAATTGGCAGCGGGGGAACCACCGGGCATGCTCTTCCCACGGGTCGTCCCCGGAGTCCCAGTTGCAAAGGCCACCATCGCAGTGGAAGCACTTGGTGTAGTCCTGCACACCAATGTAAAAAAACCCAGCTTGCACCAGCTCCAGTGGTCTCTTCGCACACGTGGAAGGCCACTTTTC

>MG12028210

GGGCCGACGTGTACAGACACACCCAGGCCAGAAAGTTCACTCGGAGGGGTGTCCCCTTTCAGCTGCACCCCCTGGGAACCGTTTGCTCCATTGAGCAGGTGGCGACCCTCGGGCACTGAGCGAGACCGGTGCCAAGACTCGTGGCCGCACTCGTCCTGGCCGGCTAGGCTGGGGTCCAGCAGGAAAGGGCAGCACGGGTAGTGCCGTGCGTGCTCCCTCAGCGGATCGTCCCCACGCTCCCAGTTGTGCACCGTGCCGCGGCAGAAGGCACACCGCACCTTGTCCTGCTCGTGAAGGTAGTAGAAGCCTGCCTGGGCCAGTAACCGCGGTGACAGGTAGTCCAACGGCCAATCCGAAAACGTGTTGAAGCGCTCCTCCTCTGACGCCTGGAGTCGGTGAAGCAAAAGGTCGTTTTCCTTTAAAACTTGAGATGATGCAGGTGTGTGGACACCCGGTGCAATGCCCACACGAGGTTGCGTCAGACAGTCGACTTGCATAGGGGTCGAATGCCTGGTGTTGTCGAATGAAGGGGGTGCTGTCGTAGCGGAGACTGGGCTCTGCGCAGCAGAGGCTGGCGCAGGCAGGGAGCGCACAAAAGCGCAATCCGGCCGAGCTTGCCGATGTCGCTTCACGACCTCGTCGGCGATCTTCCAGTCCCCAATTTCCAGCCCACACTGAAAGCACCGTGTGTGGTAGTCGTGACCCGCGTACACGAAACCACCCTGTGCCAGCCTCTTGGCGGGGATTGGGGCGTTAAGGGGCCACCCATGAAACGAGGCGATCCGGACATCTTCCTGGCTCAGGTCCGGGCTACGGCTGACGGAGGCCATGCTTGAAAACTTGACCCCCAGTGGCCCATCAGTCACCATGCGCTCAGGGGCGGGCCGGAACGCGGGTGGCTGAGCCAACCGTTCTGCCACTGTATGTACCATCATGGG

>MG12030032

GGTCCTATTTGAAATTTTCAAATTGAAATTGCGGTTATCGCGGCTATATTGCGGCGTTACTGGTGTTACTCTGTTGCATTACATTTTTTGGTTATCTGTGTTATTGTACACGTTCGACGGTTTCCAGGCAGCCGTCGTTTGGAAAGTGGTGCTGCATTTGATCTGTGCCAAGAACAGGTTGGTCCCACTCCCGTCAGTCGGCGCCGTTCAGTGGACGCCATGACCGGGTCGTCAAAATCGAAGCCGCCCGTCGACTTGTCCCTTCAAGCCGTGCTGATATTTCAAACGGACAAAGACATGCACTCTGTCGAGAATCGCCTGGCTTCCTTCGATCAGTGGCCGCTGACGGGCGACTGCATGTGCACGCCCGAGCGCATGGCTGAAGCGGGCTTCTACTATTGTCCTACAGAGAACGAGCCCGACCTTGCTCGCTGTTACGTCTGCTTCAAGGAGCTCGACGGCTGGGATCCGAGCGACGACCCGTCCAAGGAGCACTCGCGCTCCGTTAACTGCGAATTTGTTCGCTTGGGCAAGAAGGCCCGGGAAATGACTGCCGTCGACTACATGCACCTCGAGAAGGCCCGCACCAAGAACAGAGCCAACAAGTTCGTGGAGCTGCACCAAACGGAAGTCGCAGAGGCTGTGCGCAAGGTCACCTACGAACTGGGCAAGGTGCGTAGGAAGATGCGTTGACCACAATTTCGTCGCCCCATCCGCAAATCCACGCATTTTTTCCTAATCTTTTACCCGGCGTTATGGGTCCGTGCTCCGTATAACGTGCACTTACTGGCGAGCCAAGCAAATGGCGCTTTTGCATCGTCAGCCGTTGTATGCTGCGCTGCGAATTTAGGTGGCGCCGATGCGACTTGCATGCTATTCAGTGCTTTCGGATCTCACCTTTGTGTTATGAAGTAGCCTTTGG

>MG969240

GTCGCAGCCGGTTGGTGGTGTGGAGGAGGGTTTCGCCCCCGCCACGCGCAATGGATAAGCCAGCGCTGACTCCTCTGAGGGGGAGCGTCCGTTATCGCTGGCATGTGAATTTAGTCATGCGACGGTAGAATGAAATTCCTTCTGGACCATTCAGGCATTCGCCCGGCATCCCGACTGTCACACATCGTGGCTGTTGAAGTTCTCAATGGCNNNNNNNNNNNNNNNNNNNNNNNCCCATGATGGTACATACAGTGGCAGAACGGTTGGCTCAGCCACCCGCGTTCCGGCCCGCCCCTGAGCGCATGGTGACTGATGGGCCACTGGGGGTCAAGTTTTCAAGCATGGCCTCCGTCAGCCGTAGCCCGGACCTGAGCCAGGAAGATGTCCGGATCGCCTCGTTTCATGGGTGGCCCCTTAACGCCCCAATCCCCGCCAAGAGGCTGGCACAGGGTGGTTTCGTGTACGCGGGTCACGACTACCACACACGGTGCTTTCAGTGTGGGCTGGAAATTGGGGACTGGAAGATCGCCGACGAGGTCGTGAAGCGACATCGGCAAGCTCGGCCGGATTGCGCTTTTGTGCGCTCCCTGCCTGCGCCAGCCTCTGCTGCGCAGAGCCCAGTCTCCGCTACGACAGCACCCCCTTCATTCGACAACACCAGGCATTCGACCCCTATGCAAGTCGACTGTCTGACGCAACCTCGTGTGGGCATTGCACCGGGTGTCCACACACCTGCATCATCTCAAGTTTTAAAGGAAAACGACCTTTTGCTTCACCGACTCCAGGCGTCAGAGGAGGAGCGCTTCAACACGTTTTCGGATTGGCCGTTGGACTACCTGTCACCGCGGTTACTGGCCCAGGCAGGCTTCTACTACCTTCACGAGCAGGACAAGGTGCGGTGTGCCTTCTGCCGCGGCACGGTGCACAACTGGGAGCGTGGGGACGATCCGCTGAGGGAGCACGCACGGCACTACCCGTGCTGCCCTTTCCTGCTGGACCCCAGCCTAGCCGGCCAGGACGAGTGCGGCCACGAGTCTTGGCACCGGTCTCGCTCAGTGCCCGAGGGCCGCCACCTGCTCATCGGAGCAAACGGTTCCCAGGGGGTGCAGCTGAAAGGGGACACCCCTCCGAGTGAACTTTCTGGCCTGGGTGTGTCTGTACACGTCGGCCCGAAGCACCCATCGCAAGCGTCCCCCGACGCCCGGTTACGTACGTTTGAAAAGTGGCCTTCCACGTGTGCGAAGAGACCACTGGAGCTGGTGCAAGCTGGGTTTTTTTATATTGGTGTGCAGGACTACACCAAGTGCTTCCACTGCGATGGTGGCCTTTGCAACTGGGACTCCGGGGACGACCCGTGGGAAGAGCATGCCCGGTGGTTCCCCCGCTGCCAATTTGTCTTGCTCGCCAAGGGGGAGGCGTACATCAATGACTGCCTGCGGAGACACCAGTCGCATCTCAACTCGGTTGCTGCCTCAACGAGCTCTCAGCAAGGGCAGACAGGAGCGGCGGATGAGGGCATGGCGACAGAGCTGGCGGCATTGATGCGGTCGGACGACGTGCAGTTCTACCTGTCGCAAGGGGTTCCTGCAGAGACACTGCGTGCTGCACTGCTGCGGCATATGCGTGGCCAGGGCCGAGGTTTCGCCAGCCGCGACGAGCTGCTGCAGGTCCTTGGCGAGCTGCTTGCCCTGCCCAAGGCGTCTGCTGACCAGACACCGCATGAACGTGCCACCAATGGGGCTTCCTCCAAGAATATCTTTTCTGGCGCTGGAAAGCAGTCGTCACCGGTGGCCGTGACGCCAGCAGGGTCCGAGCCCTCGGACCCGGAGGAAACGCGTCTCAAGGATCAGCGGCTGTGTAAGGTGTGCCTCGATGCTGAGGTAGGCGTCGTGTTCCTGCCGTGTGGCCACCTCGTTGCGTGCCCATCGTGCGCCTCGGCTCTCGTAGACTGCCCCATCTGCCGCGCAGCCATCCGCGGCACCGTGCGGACATTCTTTGCATGAAACACCTTGGTGCGAAGGAAGAGTGCATTGGGTGAAAAAGAAAGATGGCAGAGTTTCGGTGATGTGTTCATACAGAGTGCTCCGACGGTGGCCGGGTGGGTCGAACCGAGTGGAGTAGCCGAGCGCTGCTGTGCTTGTGAGAAGCGGCTGCAGCATTCCTGCCTGTCAGCTGTAAAGCAAGCAGTAAGGCACCGATGCAGTGAAAAAGCAAGCATTGGTAGACCCTGCAAAGAGCACTTGTCATGCCCATAGAAGCATTGGTGGCGAAATCAGAGGGGGCTTCCGCTTCTACGAAGGGGTCGGGAAGAACACCTTGATGTGCTGAACGTTCCCTCTAGCATGGCCCCTCTGGCTTTTATTCTCGTTAGTCCTACTTGTAGCTGACCTGTAGGCTGTTGTGGCGGCGCTTCAGTGTTTTTAAACCGTGGTGTTTTGTGATGCTCTTGTTTCAAGTTTCCCCTGGTCCTCTTTCAACCCAAATTCTGGTCTCACCCCACGCTGCCCCCACCTCCTCTGCCCAAGCAGTCCGGTCCCGCGGCAGGTCTTGAAACACCTGCCACCCTGTATTTAGTGGAAGGACTCGTGGCTGGGTGAGTCCGCAGGGCATGCACGTGTTGTGGAAGGTAGCACTCATAGGCAAACGAGAGGATGCAAGAGAACAAGGAAGACTGCAAGTCATGCGTCCGGTTCCCTTGGGGTTGCCTCTGAATGCAGCCTTTCACAGTGCTGTGTGCTTCCGTGGGAGGTAGCTGTTGAGCTGCTGTTACCGCTACAGTTCTGACTGGTAGTGCTTGCAGATTGGCACATTTCAAAGAAAGTAAGAGTGCATCTAAGCGCACGGACTTACAAGCAAGGAAAAAAATAAAAAAAGACTTCTGACCTCCTTAGGTTTCCTGTAGTGCAGAGGTTAGTGCAACCAGAAATGGAAGCTGCACAATCTAAAGTTGCAATCTGTAGACTTTGTTTTGCAGTGCGTGGCAGTAAACTCATGAAACAGTATGGCCCACAGGCAGAAACGTTGGGAG

>MG9628228

ACCAAAGGCTACTTCATAACACAAAGGTGAGATCCGAAAGCACTGAATAGCATGCAAGTCGCATCGGCGCCACCTAAATTCGCAGCGCAGCATACAACGGCTGACGATGCAAAAGCGCCATTTGCTTGGCTCGCCAGTAAGTGCACGTTATACGGAGCACGGACCCATAACGCCGGGTAAAAGATTAGGAAAAAATGCGTGGATTTGCGGATGGGGCGACGAAATTGTGGTCAACGCATCTTCCTACGCACCTTGCCCAGTTCGTAGGTGACCTTGCGCACAGCCTCTGCGACTTCCGTTTGGTGCAGCTCCACGAACTTGTTGGCTCTGTTCTTGGTGCGGGCCTTCTCGAGGTGCATGTAGTCGACGGCAGTCATTTCCCGGGCCTTCTTGCCCAAGCGAACAAATTCGCAGTTAACGGAGCGCGAGTGCTCCTTGGACGGGTCGTCGCTCGGATCCCAGCCGTCGAGCTCCTTGAAGCAGACGTAACAGCGAGCAAGGTCGGGCTCGTTCTCTGTAGGACAATAGTAGAAGCCCGCTTCAGCCATGCGCTCGGGCGTGCACATGCAGTCGCCCGTCAGCGGCCACTGATCGAAGGAAGCCAGGCGATTCTCGACAGAGTGCATGTCTTTGTCCGTTTGAAATATCAGCACGGCTTGAAGGGACAAGTCGACGGGCGGCTTCGATTTTGACGACCCGGTCATGGCGTCCACTGAACGGCGCCGACTGACGGGAGTGGGACCAACCTGTTCTTGGCACAGATCAAATGCAGCACCACTTTCCA

>MG489627

TTTTTTTTGACCTGTTGGCAACACATTTATTGGTGACACCAAAGGCTACTTCATAACACAAAGGTGAGATCCGAAAGCACTGAATAGCATGCAAGTCGCATCGGCGCCACCTAAATTCGCAGCGCAGCATACAACGGCTGACGATGCAAAAGCGCCATTTGCTTGGCTCGCCAGTAAGTGCACGTTATACGGAGCACGGACCCATAACGCCGGGTAAAAGATTAGGAAAAAATGCGTGGATTTGCGGATGGGGCGACGAAATTGTGGTCAACGCATCTTCCTACGCACCTTGCCCAGTTCGTAGGTGACCTTGCGCACAGCCTCTGCGACTTCCGTTTGGTGCAGCTCCACGAACTTGTTGGCTCTGTTCTTGGTGCGGGCCTTCTCGAGGTGCATGTAGTCGACGGCAGTCATTTCCCGGGCCTTCTTGCCCAAGCGAACAAATTCGCAGTTAACGGAGCGCGAGTGCTCCTTGGACGGGTCGTCGCTCGGATCCCAGCCGTCGAGCTCCTTGAAGCAGACGTAACAGCGAGCAAGGTCGGGCTCGTTCTCTGTAGGACAATAGTAGAAGCCCGCTTCAGCCATGCGCTCGGGCGTGCACATGCAGTCGCCCGTCAGCGGCCACTGATCGAAGGAAGCCAGGCGATTCTCGACAGAGTGCATGTCTTTGTCCGTTTGAAATATCAGCACGGCTTGAAGGGACAAGTCGACGGGCGGCTTCGATTTTGACGACCCGGTCATGGCGTCCACTGAACGGCGCCGACTGACGGGAGTGGGACCAACCTGTTCTTGGCACAGATCAAATGCAGCACCACTTTCCAAACGACGGCTGCCTGGAAACCGTCGAACGTGTACAATAACACAGATAACCAAAAAATGTAATGCAACAGAGTAACACCAGTAACGCCGCAATATAGCCGCGATAACCGCAATTTCAATTTGAAAATTTCAAATAGGACCCGCCGAATGGCGAGCGCGC

>MG4811841

CTCCCAACGTTTCTGCCTGTGGGCCATACTGTTTCATGAGTTTACTGCCACGCACTGCAAAACAAAGTCTACAGATTGCAACTTTAGATTGTGCAGCTTCCATTTCTGGTTGCACTAACCTCTGCACTACAGGAAACCTAAGGAGGTCAGAAGTCTTTTTTTATTTTTTTCCTTGCTTGTAAGTCCGTGCGCTTAGATGCACTCTTACTTTCTTTGAAATGTGCCAATCTGCAAGCACTACCAGTCAGAACTGTAGCGGTAACAGCAGCTCAACAGCTACCTCCCACGGAAGCACACAGCACTGTGAAAGGCTGCATTCAGAGGCAACCCCAAGGGAACCGGACGCATGACTTGCAGTCTTCCTTGTTCTCTTGCATCCTCTCGTTTGCCTATGAGTGCTACCTTCCACAACACGTGCATGCCCTGCGGACTCACCCAGCCACGAGTCCTTCCACTAAATACAGGGTGGCAGGTGTTTCAAGACCTGCCGCGGGACCGGACTGCTTGGGCAGAGGAGGTGGGGGCAGCGTGGGGTGAGACCAGAATTTGGGTTGAAAGAGGACCAGGGGAAACTTGAAACAAGAGCATCACAAAACACCACGGTTTAAAAACACTGAAACGCCGCCACAACAGCCTACAGGTCAGCTACAAGTAGGACTAACGAGAATAAAAGCCAGAGGGGCCATGCTAGAGGGAACGTTCAGCACATCAAGGCGTTCTTCCCGACCCCTTCGTAGAAGCGGAAGCCCCCTCTGATTTCGCCACCAATGCTTGTATGGGCATGACAAGTGCTCTTTGCAGGGTCTACCAATGCTTGCTTTTTCACTGCATCGGTGCCTTACCGCTTGCTTCCCAGCTGACAGACATGAACGCTGCAGCCGCTTCTCACAAGCACAGCAGTGCTCGGCTACTCCACTCGGTTCGACCCACCCGGCCACCGTCGGAGCACTCTGTATGAACACATCACCGAAACTCTGCCATCTTTCTTTTTCACCCAATGCACTCTTCCTTCGCACCAAGGTGTTTCATGCAAAGAATGTCCGCACGGTGCCGCGGATGGCTGCGCGGCAGATGGGGCAGTCTACGAGAGCCGAGGCGCACGACGGGCACGCAACGAGGTGGCCGCACGGCAGGAACACGACGCCTACCTCAGCATCGAGGCACACCTTACACAGCCGCTGATCCTTGAGCCGCGTTTCCTCCGGGTCCGAGGGCTCGGACCCTGCTGGCGTCACGGCCACCGGTGACGACTGCTTTCCAGTGCCAGAAAAGATATTCTTGGAGGAAGCCCCATTGGTGGCCCGTTCCTGCGGTGTCTGGTCAGCAGACGCCTTGGGCAGGGCAAGCAGCTCGCCAAGGACCTGCAGCAGCTCGTCGCGGCTGGCGAAACCTCGGCCCTGGCCACGCATATGCCGCAGCAGTGCAGCACGCAGTGTCTCTGCAGGAACCCCTTGCGACAGGTAGAACTGCACGTCGTCCGACCGCATCAATGCCGCCAGCTCTGTCGCCATGCCCTCATCCGCCGCTCCTGTCTGCCCTTGCTGAGAGCTCGTTGAGGCAGCAACCGAGTTGAGATGCGACTGGTGTCTCCGCAGGCAGTCATTGATGTACGCCTCCCCCTTGGCGAGCAAGACAAATTGGCAGCGGGGGAACCACCGGGCATGCTCTTCCCACGGGTCGTCCCCGGAGTCCCAGTTGCAAAGGCCACCATCGCAGTGGAAGCACTTGGTGTAGTCCTGCACACCAATGTAAAAAAACCCAGCTTGCACCAGCTCCAGTGGTCTCTTCGCACACGTGGAAGGCCACTTTTCAAACGTACGTAACCGGGCGTCGGGGGACGCTTGCGATGGGTGCTTCGGGCCGACGTGTACAGACACACCCAGGCCAGAAAGTTCACTCGGAGGGGTGTCCCCTTTCAGCTGCACCCCCTGGGAACCGTTTGCTCCGATGAGCAGGTGGCGGCCCTCGGGCACTGAGCGAGACCGGTGCCGAGACTCGTGGCCGCACTCGTCCTGGCCGGCCAGGCTGGGGTCCAGCAGGAAAGGGCAGCACGGGTAGTGCCGCGCGTGCTCCCTCAGCGGATCGTCCCCACGCTCCCAGTTGTGCACCGTGCCGCGGCAGAAGGCACACCGCACCTTGTCCTGCTCGTGAAGGTAGTAGAAGCCTGCCTGGGCCAGTAACCGCGGTGACAGGTAGTCCAACGGCCAATCCGAAAACGTGTTGAAGCGCTCCTCCTCTGACGCCTGGAGTCGGTGAAGCAAAAGGTCGTTTTCCTTTAAAACTTGAGATGATGCAGGTGTGTGGACACCCGGTGCAATGCCCACACGAGGTTGCGTCAGACAGTCGACTTGCATAGGGGTCGAATGCCTGGTGTTGTCGAATGAAGGGGGTGCTGTCGTAGCGGAGACTGGGCTCTGCGCAGCAGAGGCTGGCGCAGGCAGGGAGCGCACAAAAGCGCAATCCGGCCGAGCTTGCCGATGTCGCTTCACGACCTCGTCGGCGATCTTCCAGTCCCCAATTTCCAGCCCACACTGAAAGCACCGTGTGTGGTAGTCGTGACCCGCGTACACGAAACCACCCTGTGCCAGCCTCTTGGCGGGGATTGGGGCGTTAAGGGGCCACCCATGAAACGAGGCGATCCGGACATCTTCCTGGCTCAGGTCCGGGCTACGGCTGACGGAGGCCATGCTTGAAAACTTGACCCCCAGTGGCCCATCAGTCACCATGCGCTCAGGGGCGGGCCGGAACGCGGGTGGCTGAGCCAACCGTTCTGCCACTGTATGTACCATCATGGGCGCCATTGAGAACTTCAACAGCCACGATGTGTGACAGTCGGGATGCCGGGCGAATGCCTGAATGGTCCAGAAGGAATTTCATTCTACCGTCGCATGACTAAATTCACATGCCAGCGATAACGGACGCTCCCCCTCAGAGGAGTCAGCG

>AAUM999

ACTAAAAACAGGACACGCGAGGTAAGCTCTACTGTCATCCGATAGCGCGGCCCATTTTTTAATTATTTTTTTCGGCTGTCGGTCGCATATGCGCGTTCCACCGTGCCGCCGACTACGCCAGCCCTGTGGTGAGCTCGATCAGGAAGTGCTAAGCCTCTGAGCCGTGTGCTTGGGTTGTCGCGCTCTTTGGCGTGCTCCGTGACCACGGCCTGACCTTGTGGTGTTCGTTTATGAACGGCAACCTCGCTAATGCGTAGTTGACGCGTACGCCCATTCCGAAGGTCGAGTTGGTCCTTTGTTGCGCAAGGACGCGCTTAACGACGCGTTGCCTACGCTGTCGTCGTTACCGAGGGCGGCCATGTTGGGCATGCGCGCAGTGTTTCTCCAGCTGCCCACTGCGTTGTGAAAGCCGAAGAAATACCGGAGTCCGGGACGTCTTGGAGTGGTTGCGTTAGGTTTCCCTAGTTAGTGCTGTCGCAGCCGGTTGGTGGTGTGGAGGAGGGTTTCGCCCCCGCCACGCGCAATGGATAAGCCAGCGCTGACTCCTCTGAGGGGGAGCGTCCGTTATCGCTGGCATGTGAATTTAGTCATGCGACGGTAGAATGAAATTCCTTCTGGACCATTCAGGCATTCGCCCGGCATCCCGACTGTCACACATCGTGGCTGTTGAAGTTCTCAATGGCNNNNNNNNNNNNNNNNNNNNNNNNNNNNNNNNNNNNNNNNNNNNNNNNNNNCCCATGATGGTACATACAGTGGCAGAACGGTTGGCTCAGCCACCCGCGTTCCGGCCCGCCCCTGAGCGCATGGTGACTGATGGGCCACTGGGGGTCAAGTTTTCAAGCATGGCCACCGTCAGCCGTAGCCCGGACCTGAGCCAGGAAGATGTCCGGATCGCCTCGTTTCATGGGTGGCCCCTTAACGCCCCAATCCCCGCCAAGAGGCTGGCACAGGGTGGTTTCGTGTACGCGGGTCACGACTACCACACACGGTGCTTTCAGTGTGGGCTGGAAATTGGGGACTGGAAGATCGCCGACGAGGTCGTGAAGCGACATCGGCAAGCTCGGCCGGATTGCGCTTTTGTGCGCTCCCTGCCTGCGCCAGCCTCTGCTGCGCAGAGCCCAGTCTCCGCTACGACAGCACCCCCTTCATTCGACAACACCAGGCATTCGACCCCTATGCAAGTCGACTGTCGGACGCAACCTCGTGTCGGCATTGCACCGGGTGTCCACACACCTGCATCATCTCAAGTTTTAAAGGAAAACGACCTTTTGCTTCACCGACTCCAGGCGTCAGAGGAGGAGCGCTTCAACACGTTTTCGGATTGGCCGTTGGACTACCTGTCACCGCGGTTACTGGCCCAGGCAGGCTTCTACTACCTTCACGAGCAGGACAAGGTGCGGTGTGCCTTCTGCCGCGGCACGGTGCACAACTGGGAGCGTGGGGACGATCCGCTGAGGGAGCACGCGCGGCACTACCCGTGCTGCCCTTTCCTGCTGGACCCCAGCCTGGCCGGCCAGGACGAGTGCGGCCACGAGTCTCGGCACCGGTCTCGCTCAGTGCCCGAGGGCCGCCACCTGCTCATCGGAGCAAACGGTTCCCAGGGGGTGCAGCTGAAAGGGGACACCCCTCCGAGTGAACTTTCTGGCCTGGGTGTGTCTGTACACGTCGGCCCGAAGCACCCATCGCAAGCGTCCCCCGACGCCCGGTTACGTACGTTTGAAAAGTGGCCTTCCACGTGTGCGAAGAGACCACTGGAGCTGGTGCAAGCTGGGTTTTTTTACATTGGTGTGCAGGACTACACCAAGTGCTTCCACTGCGATGGTGGCCTTTGCAACTGGGACTCCGGGGACGACCCGTGGGAAGAGCATGCCCGGTGGTTCCCCCGCTGCCAATTTGTCTTGCTCGCCAAGGGGGAGGCGTACATCAATGACTGCCTGCGGAGACACCAGTCGCATCTCAACTCGGTTGCTGCCTCAACGAGCTCTCAGCAAGGGCAGACAGGAGCGGCGGATGAGGGCATGGCGACAGAGCTGGCGGCATTGATGCGGTCGGACGACGTGCAGTTCTACCTGTCGCAAGGGGTTCCTGCAGAGACACTGCGTGCTGCACTGCTGCGGCATATGCGTGGCCAGGGCCGAGGTTTCGCCAGCCGCGACGAGCTGCTGCAGGTCCTTGGCGAGCTGCTTGCCCTGCCCAAGGCGTCTGCTGACCAGACACCGCAGGAACGGGCCACCAATGGGGCTTCCTCCAAGAATATCTTTTCTGGTGCTGGAAAGCAGTCGTCACCGGTGGCTGTGACGCCAGCAGGGTCCGAGCCCTCGGACCCGGAGGAAACGCGGCTCAAGGATCAGCGGCTGTGTAAGGTGTGCCTCGATGCTGAGGTAGGCGTCGTGTTCCTGCCGTGCGGCCACCTCGTTGCGTGCCCGTCGTGCGCCTCGGCTCTCGTAGACTGCCCCATCTGCCGCGCAGCCATCCGTGGCACCGTGCGGACATTCTTTGCATAAAACGCCATGGTGCGAAGGAAGAATGCATTGGGTGAAAAAGAAAGATGGCAGAGTTTCGGTGATGTGTTCATCCAGAGTGCTCCGACGGTGGCCGGGTGGGTCGAACCGAGGGGAGTAGCCGAGCGCTGCTGTGCTTGTGAGAAGCGGCTGCAGCATTCATGCCTGTCAGCTG

>AAUM15739

TGACACCAAAGGCTACTTCATAACACAAAGGTGAGATCCGAAAGCACTGAATAGCATGCAAGTCGCATCGGCGCCACCTAAATTCGCAGCGCAGCATACAACGGCTGACGATGCAAAAGCGCCATTTGCTTGGCTCGCCAGTAAGTGCACGTTATACGGAGCACGGACCCATAACGCCGGGTAAAAGATTAGGAAAAAATGCGTGGATTTGCGGATGGGGCGACGAAATCGTGGTCAACGCATCTTCCTACGCACCTTGCCCAGTTCGTAGGTGACCTTGCGCACAGCCTCTGCGACTTCCGTTTGGTGCAGCTCCACGAACTTGTTGGCTCTGTTCTTGGTGCGGGCCTTCTCGAGGTGCATGTAGTCGACGGCAGTCATTTCCCGGGCCTTCTTGCCCAAGCGAACAAATTCGCAGTTAACGGAGCGCGAGTGCTCCTTGGACGGGTCGTCGCTCGGATCCCAGCCGTCGAGCTCCTTGAAGCAGACGTAACAGCGAGCAAGGTCGGGCTCGTTCTCTGTAGGACAATAGTAGAAGCCCGCTTCAGCCATGCGCTCGGGCGTGCACATGCAGTCGCCCGTCAGCGGCCACTGATCGAAGGAAGCCAGGCGATTCTCGACAGAGTGCATGTCTTTGTCCGTTTGAAATATCAGCACGGCTTGAAGGGACAAGTCGACGGGCGGCTTCGATTTTGACGACCCGGTCATGGCGTCCACTGAACGGCGCCGACTGACGGGAGTGGGACCAACCTGTTCTTGGCACAGATCAAATGCAGCACCACTTTCCAAACGACGGCTGCCTGGAAACCGTCGAACGTGTACAATAACACAGATAACCAAAAAATGTAATGCAACAGAGTAACACCAGTAACGCCGCAATATAGCCGCGATAACCGCAATTTCAATTTGAAAATTTCAAATAGGACCCGCCGAATGGCGAGCGCGCGAACGTTTTG

>AAUF11345

TCCGAAGGTCGAGTTGGTCCTTTGTTGCGTAAGGACGCGCTTAACGACGCGTTGCCTACGCTGTCGTCGTTACCGAGGGCGGCCATGTTGGGCATGCGCGCAGTGTTTCTCCAGCTGCCCACTGCGTTGTGAAAGCCGAAGAAATACCGGAGTCCGGGACGTCTTGGAGTGGTTGCGTTAGGTTTCCCTAGTTAGTGCTGTCGCAGCCGGTTGGTGGTGTGGAGGAGGGTTTCGCCCCCGCCACGCGCAATGGATAAGCCAGCGCTGACTCCTCTGAGGGGGAGCGTCCGTTATCGCTGGCATGTGAATTTAGTCATGCGACGGTAGAATGAAATTCCTTCTGGACCATTCAGGCATTCGCCCGGCATCCCGACTGTCACACATCGTGGCTGTTGAAGTTCTCAATGGCACCCATGATGGTACATACAGTGGCAGAACGGTTGGCTCAGCCACCCGCGTTCCGGCCCGCCCCTGAGCGCATGGTGACTGATGGGCCACTGGGGGTCAAGTTTTCAAGCATGGCCACCGTCAGCCGTAGCCCGGACCTGAGCCAGGAAGATGTCCGGATCGCCTCGTTTCATGGGTGGCCCCTTAACGCCCCAATCCCCGCCAAGAGGCTGGCACAGGGTGGTTTCGTGTACGCGGGTCACGACTACCACACACGGTGCTTTCAGTGTGGGCTGGAAATTGGGGACTGGAAGATCGCCGACGAGGTCGTGAAGCGACATCGGCAAGCTCGGCCGGATTGCGCTTTTGTGCGCTCCCTGCCTGCGCCAGCCTCTGCTGCGCAGAGCCCAGTCTCCGCTACGACAGCACCCCCTTCATTCGACAACACCAGGCATTCGACCCCTATGCAAGTCGACTGTCTGACGCAACCTCGTGTGGGCATTGCACCGGGTGTCCACACACCTGCATCATCTCAAGTTTTAAAGGAAAACGACCTTTTGCTTCACCGACTCCAGGCGTCAGAGGAGGAGCGCTTCAACACGTTTTCGGATTGGCCGTTGGACTACCTGTCACCGCGGTTACTGGCCCAGGCAGGCTTCTACTACCTTCACGAGCAGGACAAGGTGCGGTGTGCCTTCTGCCGCGGCACGGTGCACAACTGGGAGCGTGGGGACGATCCGCTGAGGGAGCACGCGCGGCACTACCCGTGCTGCCCTTTCCTGCTGGACCCCAGCCTGGCCGGCCAGGACGAGTGCGGCCACGAGTCTCGGCACCGGTCTCGCTCAGTGCCCGAGGGCCGCCACCTGCTCATCGGAGCAAACGGTTCCCAGGGGGTGCAGCTGAAAGGGGACACCCCTCCGAGTGAACTTTCTGGCCTGGGTGTGTCTGTACACGTCGGCCCGAAGCACCCATCGCAAGCGTCCCCCGACGCCCGGTTACGTACGTTTGAAAAGTGGCCTTCCACGTGTGCGAAGAGACCACTGGAGCTGGTGCAAGCTGGGTTTTTTTACATTGGTGTGCAGGACTACACCAAGTGCTTCCACTGCGATGGTGGCCTTTGCAACTGGGACTCCGGGGACGACCCGTGGGAAGAGCATGCCCGGTGGTTCCCCCGCTGCCAATTTGTCTTGCTCGCCAAGGGGGAGGCGTACATCAATGACTGCCTGCGGAGACACCAGTCGCATCTCAACTCGGTTGCTGCCTCAACGAGCTCTCAGCAAGGGCAGACAGGAGCGGCGGATGAGGGCATGGCGACAGAGCTGGCGGCATTGATGCGGTCGGACGACGTGCAGTTCTACCTGTCGCAAGGGGTTCCTGCAGAGACACTGCGTGCTGCACTGCTGCGGCATATGCGTGGCCAGGGCCGAGGTTTCGCCAGCCGCGACGAGCTGCTGCAGGTCCTTGGCGAGCTGCTTGCCCTGCCCAAGGCGTCTGCTGACCAGACACCGCAGGAACGGGCCACCAATGGGGCTTCCTCCAAGAATATCTTTTCTGGCGCTGGAAAGCAGTCGTCACCGGTGGCCGTGACGCCAGCAGGGTCCGAGCCCTCGGACCCGGAGGAAACGCGGCTCAAGGATCAGCGGCTGTGTAAGGTGTGCCTCGATGCTGAGGTAGGCGTCGTGTTCCTGCCGTGCGGCCACCTCGTTGCGTGCCCGTCGTGCGCCTCGGCTCTCGTAGACTGCCCCATCTGCCGCGCAGCCATCCGNNNNNNNNNNNNNNNNNNNNNNNNNNNNNNNNNNNNNNNNNNNNNNNNNNNNNNNNNNNNNNNNNNNNNNNNNNNNNNNNNNNNNNNNNNNNNNNNNNNNNNNNNNNNNNNNNNNNNNNNNNNNNNNNNNNNNNNNNNNNNNNNNCTGCTGTGCTTGTGAGAAGCGGCTGCAGCGTTCATGTCTGTCAGCTGGGAAGCAAGCGGTAAGGCACCGATGCAGTGAAAAAGCAAGCATTGGTAGACCCTGCAAAGAGCACTTGTCATGCCCATACAAGCATTGGTGGCGAAATCAGAGGGGGCTTCCGCTTCTACGAAGGGATTGGGAAGAACGCCTTGATGTGCTGAACGTTCCCTCTAGCATGGCCCCTCTGGCTTTTATTCTCGTTAGTCCTACTTGTAGCTGACCTGTAGGCTGTTGTGGCGGCGNNNNNNNNNNNNNNNNNNNNNNNNNNNNNNNNNNNNNNNNNNNNNNNNNNNNNNNNNNNNNNNNNNNNNNTTTCAACCCAAATTCTGGTCTCACCCCACGCTGCCCCCACCTCCTCTGCCCAAGCAGTCCGGTCCCGCGGCAGGTCTTGAAACACCTGCCACCCTGTATTTAGTGGAAGGACTCGTGGCTGGGTGAGTCCGCAGGGCATGCACGTGTTGTGGAAGGTAGCACTCATAGGCAAACAGAGAGGATGCAAGAGAACAAGGAAGACTGCAAGTCGTGCGTCCGGTTCCCTTAGAGTTGCCTCTGAATGCAGCCTTTCACAGTGCTGCCTGCTTC

>AAUF36430

TGACACCAAAGGCTACTTCATAACACAAAGGTGAGATCCGAAAGCACTGAATAGCATGCAAGTCGCATCGGCGCCACCTAAATTCGCAGCGCAGCATACAACGGCTGACGATGCAAAAGCGCCATTTGCTTGGCTCGCCAGTAAGTGCACGTTATACGGAGCACGGACCCGTAACGCCGGGTAAAAGATTAGGAAAAAATGCGTGGATTTGCGGATGGGGCGACGAAATCGTGGTCAACGCATCTTCCTACGCACCTTGCCCAGTTCGTAGGTGACCTTGCGCACAGCCTCTGCGACTTCCGTTTGGTGCAGCTCCACGAACTTGTTGGCTCTGTTCTTGGTGCGGGCCTTCTCGAGGTGCATGTAGTCGACGGCAGTCATTTCCCGGGCCTTCTTGCCCAAGCGAACAAATTCGCAGTTAACGGAGCGCGAGTGCTCCTTGGACGGGTCGTCGCTCGGATCCCAGCCGTCGAGCTCCTTGAAGCAGACGTAACAGCGAGCAAGGTCGGGCTCGTTCTCTGTAGGACAATAGTAGAAGCCCGCTTCAGCCATGCGCTCGGGCGTGCACATGCAGTCGCCCGTCAGCGGCCACTGATCGAAGGAAGCCAGGCGATTCTCGACAGAGTGCATGTCTTTGTCCGTTTGAAATATCAGCACGGCTTGAAGGGACAAGTCGACGGGCGGCTTCGATTTTGACGACCCGGTCATGGCGTCCACTGAACGGCGCCGACTGACGGGAGTGGGACCAACCTGTTCTTGGCACAGATCAAATGCAGCACCACTTTCCAAACGACGGCTGCCTGGAAACCGTCGAACGTGTACAATAACACATATAACCAAAAAATGTAATGCAACAGAGTAACACCAGTAACGCCGCAATATAGCCGCGATAACCGCAATTTCAATTTGAAAATTTC

>AAFM14654

ATGAACACATCACCGAAACTCTGCCATCTTTCTTTTTCACCCAATGCATTCTTCCTTCGCACCATGGCGTTTCATGCAAAGAATGTCCGCACGGTGCCACGGATGGCTGCGCGGCAGATGGGGCAGTCTACGAGAGCCGAGGCGCACGACGGGCACGCAACGAGGTGGCCGCACGGCAGGAACACGACGCCTACCTCAGCATCGAGGCACACCTTACACAGCCGCTGATCCTTGAGCCGCGTTTCCTCCGGGTCCGAGGGCTCGGACCCTGCTGGCGTCACGGCCACCGGTGACGACTGCTTTCCAGTGCCAGAAAAGATATTCTTGGAGGAAGCCCCATTGGTGGCCCGTTCCTGCGGTGTCTGGTCAGCAGACGCCTTGGGCAGGGCAAGCAGCTCGCCAAGGACCTGCAGCAGCTCGTCGCGGCTGGCGAAACCTCGGCCCTGGCCACGCATATGCCGCAGCAGTGCAGCACGCAGTGTCTCTGCAGGAACCCCTTGCGACAGGTAGAACTGCACGTCGTCCGACCGCATCAATGCCGCCAGCTCTGTCGCCATGCCCTCATCCGCCGCTCCTGTCTGCCCTTGCTGAGAGCTCGTTGAGGCAGCAACCGAGTTGAGATGCGACTGGTGTCTCCGCAGGCAGTCATTGATGTACGCCTCCCCCTTGGCGAGCAAGACAAATTGGCAGCGGGGGAACCACCGGGCATGCTCTTCCCACGGGTCGTCCCCGGAGTCCCAGTTGCAAAGGCCACCATCGCAGTGGAAGCACTTGGTGTAGTCCTGCACACCAATGTAAAAAAACCCAGCTTGCACCAGCTCCAGTGGTCTCTTCGCACACGTGGAAGGCCACTTTTCAAACGTACGTAACCGGGCGTCGGGGGACGCTTGCGATGGGTGCTTCGGGCCGACGTGTACAGACACACCCAGGCCAGAAAGTTCACTCGGAGGGGTGTCCCCTTTCAGCTGCACCCCCTGGGAACCGTTTGCTCCGATGAGCAGGTGGCGGCCCTCGGGCACTGAGCGAGACCGGTGCCGAGACTCGTGGCCGCACTCGTCCTGGCCGGCCAGGCTGGGGTCCAGCAGGAAAGGGCAGCACGGGTAGTGCCGCGCGTGCTCCCTCAGCGGATCGTCCCCACGCTCCCAGTTGTGCACCGTGCCGCGGCAGAAGGCACACCGCACCTTGTCCTGCTCGTGAAGGTAGTAGAAGCCTGCCTGGGCCAGTAACCGCGGTGACAGGTAGTCCAACGGCCAATCCGAAAACGTGTTGAAGCGCTCCTCCTCTGACGCCTGGAGTCGGTGAAGCAAAAGGTCGTTTTCCTTTAAAACTTGAGATGATGCAGGTGTGTGGACACCCGGTGCAATGCCGACACGAGGTTGCGTCCGACAGTCGACTTGCATAGGGGTCGAATGCCTGGTGTTGTCGAATGAAGGGGGTGCTGTCGTAGCGGAGACTGGGCTCTGCGCAGCAGAGGCTGGCGCAGGCAGGGAGCGCACAAAAGCGCAATCCGGCCGAGCTTGCCGATGTCGCTTCACGACCTCGTCGGCGATCTTCCAGTCCCCAATTTCCAGCCCACACTGAAAGCACCGTGTGTGGTAGTCGTGACCCGCGTACACGAAACCACCCTGTGCCAGCCTCTTGGCGGGGATTGGGGCGTTAAGGGGCCACCCATGAAACGAGGCGATCCGGACATCTTCCTGGCTCAGGTCCGGGCTACGGCTGACGGTGGCCATGCTTGAAAACTTGACCCCCAGTGGCCCATCAGTCACCATGCGCTCAGGGGCGGGCCGGAACGCGGGTGGCTGAGCCAACCGTTCTGCCACTGTATGTACCATCATGGGCGCCATTGAGAACTTCAACAGCCACGATGTGTGACAGTCGGGATGCCGGGCGAATGCCTGAATGGTCCAGAAGGAATTTCATTCTACCGTCGCATGACTAAATTCACATGCCAGCGATAACGGACGCTCCCCCTCAGAGGAGTCAGCGCTGGCTTATCCATTGCGCGTGGCGGGGGCGAAACCCTCCTCCACACCACCAACCGGCTGCGACAGCACTAACTAGGGAAACCTAACGCAACCACTCCAAGACGTCCCGGACTCCGGTATTTCTTCGGCTTTCACAACGCAGTGGGCAGCTGGAGAAACACTGCGCGCATGCCCAACATGGCCGCCCTCGGTAACGACGACAGCGTAG

>AAFM29280

CACAAAACGTTCGCGCGCTCGCCATTCGGCGGGTCCTATTTGAAATTTTCAAATTGAAATTGCGGTTATCGCGGCTATATTGCGGCGTTACTGGTGTTACTCTGTTGCATTACATTTTTTGGTTATCTGTGTTATTGTACACGTTCGACGGTTTCCAGGCAGCCGTCGTTTGGAAAGTGGTGCTGCATTTGATCTGTGCCAAGAACAGGTTGGTCCCACTCCCGTCAGTCGGCGCCGTTCAGTGGACGCCATGACCGGGTCGTCAAAATCGAAGCCGCCCGTCGACTTGTCCCTTCAAGCCGTGCTGATATTTCAAACGGACAAAGACATGCACTCTGTCGAGAATCGCCTGGCTTCCTTCGATCAGTGGCCGCTGACGGGCGACTGCATGTGCACGCCCGAGCGCATGGCTGAAGCGGGCTTCTACTATTGTCCTACAGAGAACGAGCCCGACCTTGCTCGCTGTTACGTCTGCTTCAAGGAGCTCGACGGCTGGGATCCGAGCGACGACCCGTCCAAGGAGCACTCGCGCTCCGTTAACTGCGAATTTGTTCGCTTGGGCAAGAAGGCCCGGGAAATGACTGCCGTCGACTACATGCACCTCGAGAAGGCCCGCACCAAGAACAGAGCCAACAAGTTCGTGGAGCTGCACCAAACGGAAGTCGCAGAGGCTGTGCGCAAGGTCACCTACGAACTGGGCAAGGTGCGTAGGAAGATGCGTTGACCACGATTTCGTCGCCCCATCCGCAAATCCACGCATTTTTTCCTAATCTTTTACCCGGCGTTATGGGTCCGTGCTCCGTATAACGTGCACTTACTGGCGAGCCAAGCAAATGGCGCTTTTGCATCGTCAGCCGTTGTATGCTGCGCTGCGAATTTAGGTGGCGCCGATGCGACTTGCATGCTATTCAGTGCTTTCGGATCTCACCTTTGTGTTATGAAGTAGCCTTTGGTGTCACCAATAAATGTGTTGCCAACAGGTCAAAAA
