# Supplementary material for: A repertoire of protease inhibitor families in Amblyomma americanum and other tick species: inter-species comparative analyses
Source: Parasit Vectors. 2017 Mar 22;10:152. doi: 10.1186/s13071-017-2080-1 (PMC5361777; doi:10.1186/s13071-017-2080-1)
Supplement: Supplementary file 3 — FASTA sequences for Amblyomma americanum contigs from Illumina sequencing, by PI family. (ZIP 638 kb) [file 13071_2017_2080_MOESM3_ESM.zip › A. americanum I35.docx]

>AAUF61286

GCCACTTTTGCAAGAGTGACGTTGTGATCCAGGCCACCGTGCTGTCGGTGGAGGTCGTGGAAGCGTCGCTGCGTTACGACGTGGCCATCTCCGAGTCCTACCGCAACCTGCTGGACCTGCAGCAGCGCGAGTACCTGTGGGCGCCCGACCGCCGCTGCGCGTGCCCGAGGCTGCGTGTGGGACGAGAGTACGTGGTCATGGCGCGCGAACAGCGCAACTTCGCCAACAAGGAGTCCAAGCTGGTCGTCGACTCCAAGTCGTTCGTGCGCCGCTTCA

>SG12038131

CATCCCACAAAATGACCGTTATATAGCCCATGTTCACGATATCCGTAATATGACAGCTGGTGTTGAGAAGGACGGCTCTCAACTACTTTGGCATCCTCCGGATCTTTGCATGCTTGCTGTAAGCCACTGTAGGACGGAGTTTATCCTGCTGGCGCTTTGTGATGTCCCAGCCGTTTTTGTGTCGAAGAGACGAACATGCTTATCCAGAATGTAGCGAGACACAACTACGCCGTCCTTCTCGAAGAGCTCGCCGTCTCGGCCGAAGACAAAGTAGCGTGAGCCTTCACTCATGTCAGCCGTGTTGCAGTGTTTGCGAGCTACGAACAGCTTAGTGCCCGTCAAGGCGGCCTCCCTCTGTTCAGCGCCTTGTTTGATCACCGTGTCAACACGAAGTTCAATTTGGCGATAACCATTGGTTACATGGTTCCGAGACACGGTTCCAACCCACACGAAGTCGTGCTCTTTGCAAGCTAGGTCGAGCAGCTTGCGCCTCTTTTCAGAAGTCGCTCCCACGTTGTCAATTGTTCTGAAGGGTTCTCTGGGTGGGCATTCATCTTCCACGCATCGGCACTCGTCTCGTTCACAGGTAAGCTTCAGAAGCGGGCTTGTCGAATCAGGACCGTAGAATTGCGTGCACGAACGCGTCGGGTCATA

>MG12016892

GCCAAACATCCTGAACGTCCAGTTCCATCTGCTGAGACTTGATTGACCCCCAAAGAGCTACACTAATCATTCTCTACAGCTCTGCCAGGCAGATTGCCTTTATAACGACATCACCCTCGTCAATCGAATAGCGCAGAAGCGTTCTTTTCTTAATGAAAGACCGCTGCTTGACAGACAGAAAAAACCTCCCGGAAGGCTGCATATCGATCTTGTACCCGTGTCGCAGCACTCGGTGGCATGGTATCGTATTGACGTTGACCGGAAATGCTCTCACAGAATATTTTGCCGCCTCCAGTTGTCGACGCAGCCGTCATGCAGTGAAGCTTTCATCCCACAAAATGACCGTTATATAGCCCATGTTCACGATATCCGTAATATGAGAGCTGGTGTTGAGAAGGACGGCTCTCAACTACTTTGGCATCCTCCCGATCTTTGCATGCTTGCTGTAAGCCACTGTAGGACGGAGTTTATCCTGCTGGCGCTTTGTGATGTCCCAGCCGTTTTTGTGTCGAAGAGACGAACATGCTTATCCAGAATGTAGCGAGACACAACTACGCCGTCCTTCTCGAAGAGCTCGCCGTCTCGGCCGAAGACAAAGTAGCGTGAGCCTTCACTCATGTCAGCCGTGTTGCAGTGTTTGCGAGCTACGAACAGCTTAGTGCCCGTCAAGGCGGCCTCCCTCTGTTCAGCGCCTTGTTTGATCACCGTGTCAACACGAAGTTCGATTTGGCGATAACCATTGGTGACATGGTTCCGAGACACGGTTCCAACCCACACGAAGTCGTGCTCTTTGCAAGCTAGGTCGAGCAGCTTGCGCCTCTTTTCAGAAGACGCTCCCACGTTGTCAATTGTTCTGAAGGGTTCTCTGGGTGGGCATTCATCTTCCACGCATCGGCACTCGTCTCGTTCACAGGTAAGCTTCAGGAGCGGGCTTGTCGAATCAGGACCGTAGAATTGCGTGCA

>MG4818486

CGACCTGGCCTGCAAATGCCCCAAGCTCAGACTCAAGAGCAGCTACCTCATTCTCGGCAACGAAGAGATCCACGACGGCCAGCCGGGCCTTACGGCGGACAAGAACAGCATCGTCATTGACTGGAAGGATGAGTGGGGAAGGAGGCTGCGAAANNNNNNNNNNNNNNNNNNGAAAGAATGGTCTGAAGAAGGCTATGAGGAGCCCTCGCACTTTACGCAGTGTCTAATGAGCATTTACCACAATGAAACGGTGTCCTTTGACATTGCCGATGATGACATTTTACCATCCTGATGTTGTGTCCCTAGCAGTGTTCAAACAGTTGTTGAGGTCCCCTCACAGAAAGAACTTGATGTTGTCAAAGTACTCCAGATTGCTTAGTCAAACCACTTTTTCCTTCATCTTTTCCAAGGTAAGGTGGTTAAACTCATGCTTAATGAGATCAAGC
