# Supplementary material for: A repertoire of protease inhibitor families in Amblyomma americanum and other tick species: inter-species comparative analyses
Source: Parasit Vectors. 2017 Mar 22;10:152. doi: 10.1186/s13071-017-2080-1 (PMC5361777; doi:10.1186/s13071-017-2080-1)
Supplement: Supplementary file 3 — FASTA sequences for Amblyomma americanum contigs from Illumina sequencing, by PI family. (ZIP 638 kb) [file 13071_2017_2080_MOESM3_ESM.zip › A. americanum I39.docx]

>AAFM5175

GGCGTCCTTGCCCATGATGATGTATCGGCTGCCTTCAGTCAGTTTGAAGGATTCGCAATTCTCGCGAGCCTTGACCACTCGCGTCTTGTTGAGAAGGTCCTGCTGTGACTCAATGCCTGGCTTGAGGATTTTGTTGATAAAGAAAGGAACAGTGATGAATCCATCTTTGTGGGCTCCATTAAGGGGCGTGCCAAGCCATACAAAATCAACATTGTCGCAGGCGTGGTATCTCATGTATTCTCGGCAATCTGAGGTTTTCAGAACAGCACCCTCTTCTTTCGTGAACATCTCCTCGACTTCTTGGGGCGGGCAACCACCTTCCAAGCATCTGCAGACTTCAGAGTGACCCCCATTGGCATCGCTGCAGTTTGACTCGAGCATGGGACTGGTGCTGTCTGGCGAGTAGAATTTCCTGCACGATATATCGGGGTCGTAGTAGGCGTACGCCTTCACGGAGCCCGACTGCAACTTGCCTACGGCGAATGCCTGCTGCAGGCCAAACTCGACGCACACGCTGGCGTTCGATGGAATGGTCGACAGGTAGAATACCACGCTACGCTGGCTCAACTCGTACGAGTCCACACGTTTGTCGGCCACCATCTTGTCCAAATCGGCCAACACGGGATTGAAGCCGGTTAGGAGACCTACGTCCAGGATGGCCATGCCTTTCAATTTTTTTTCAAGAGACCTCGCACAGACTTCCATCGAGTAAATCTCTTTGAAACTCCCTTTACCTTCCAACGCTTTCGACACGTTGACCTTGTGCTGTGTGAAGTTTATCTTCAGATCAAACTTGCACAAAAGGTCCACAGCGACAGGGGACTCGTAGCTTGTGACAACGTACATAGTCGCCGTTCCAGTTCCTTTCACATTGACAAATATCTTGTCATTGCTGTCTGGAATCTCGATCCTGTTGCGCTGTTGGGCGTTGTCTCGCTTGATTCGAAGGGTGCGGTTAAAGTTACGGTCCCCGCTCAACGTCACTTCACAGGTCAGGTCAATGTCTGCGTCTCGTGCGTAGAGGGCATACTTGGACAGGGCTTGAAGCGCCACCACGGTATCCTGGCTGGACCGCAGCGACCCGCTGGGGTTCATTCGCAGGTTAAGCCATTGCACCATGGCCGTGATGTCGTCCCTACTCTCGCCGGCATTGAGCAGTGCCATGAGCGCGTATGACGTGGCCTGCACCGACAGTGGCTCGGAACCGGCGGACACATACAGCCCTCCTTCATTCCCTCTCTGGAAAATGCCGCCGAGCCGGCGCAGGGTGTCGTTCTTTGCGGGGCTTTTGCCCAAGGAGAGCGCGTACGCTGCCAGCGAGAGCACGTAGGGCGAAGTGTTTGGATTCAGCTGTCCCTCGATGAACCCTGTTGCTTTTGCGAAGGACTCTGTCAGGCCGGCCACCTGAACGCCTTCTCGATCGCATTCTTGCAGGGTGAGAAGGGTGTACGCGGTCAGTGGAACCGGTCCATTCACACCGCCCAGGAGGTCACCGTGGATTAGGTTGGATATGTCGTGGAAACTGCCGTCCTGCTTCTGCTGGGTCAAAATGTAACGGAGCCCACTTGTGACGACCTTCTCGTCGATGAGGATAGACTTCCGGGCTTCGCACAAAGTTCTCACCACGAATGCTGTTAGCCATAGACTGGACCTGTAATGATTCCAAACCGCGAAGGACCCGTCAGGTTTCCGGAAATTGAGGATCCGCTGGTAGCCACTGCTGATGAAGCCCAGCGCTCTGTCCTCGTCCACGGGCGATATGCGGCTGGCGGTCTTCAGATACTCGTAGGCGTACAGCGTTGGTGCCAGGCCGATCATGGTCTGTTCACCGCAGCCATGTGGCATCCTCAGAAGCGTTCCCGGGTTCTTTACGGACGCCTCCAAAGCAGCGCCCATGTCATCACCAACGATGTCGATCTCACAGCGTTCGGAGCCAGGAATCACTTCCTTAGGGGATCGGCCAGGCCGCTTCATCTGGATGAGTTGCTTGCCTCCAAGCCCGAACTTTTCTTCGTAGCTGTACGTGGGGCTCGCGACGTTCCGCGCTGGCCGTTTCTGTGTGTTTTGAGGGTCGAGAATGAGATTGAAGCTCTCATTCTTGCTGACACCAGGTGGCTCTACCCTGAGCATCACTTTGACAGAGTCCCTAGCTGACCCATCGCTAAGGGCGGCCACGCGGATCTCCCTCTCGCCAGCAGCCAACGGCACCACGGGGAAGACCACCGTGCGGCCCTGTCCCGCCGGCACGGTCAGCGTGCGCACCGATGAAGGCTTGCCTTCTTTGACACCCGAGCACACGTCCTTCGTCCCGAGCATCGCCACCCTAACCTTGAGATCCTTGTGGTCGTAGTTGTACACCGTCGCCGGTATCTCGATCTGTTCCTCCTTGATCACGGAGTAAGGGAGGTTGACTTCGACGAAGAGCTTCTTGAAGACCGGAATCTGGAGAGGATCAAGTACACATATCCCGCCAGATGGGGCCACGCCCACAGCGCTGACCTCCCACGTGGTGATCGAGTCTGGGACGGTCACCTCAAGGCTAGCCTCTCCATGTTCCCGTAGGGTTAGTTGATTGAATATCCACGTCTCTCTAAAGTTTCGCCTTTCTTCACCCTCTAAATCTTTGAAGTTGCTCATGCCCAACACGTCGGCTTCCTCTGCAGATGACCTTCCGACAGCCTGGTTGTCTTCGACATGGATGCAGCACTTGCGAAAGGCTGCGACACATTCTATCGTGTAGAGGGCCACGCCGGCATCCAGATAGCTGAGCAGAGCCTCCTCGCGTTCCGAGCAACTGCGTAGGAGCTGGTCAGGCTGCATCCCCCGCGAGCAGCAGTCTCGGAGCGTCTCGTTTTCGTACTCTTTCACTATGTCCAGCTTGATTTCACGTTTTGCGCGCCGTCTCTCTACCGCTGGTGCGCCGTAGCTGGCCTGTGATGTGAGCAGCACAACGCCCGCATTCGCAAGGGTTTCCACCGCTTTCCTGCCAGCGTCCTGTCCGTTGCCCATGTCCTTCGCTTCCATGGACTGGAACACCTTCTTTCGCGTTAGGAGGTCCTTGCGTCGCAGAAGGTATACTGCCTGGTCCACGCCCAGCAAGCCAACTCGTGTGCCTTCTCTACCCTTCAGCAAGAGCTTCTCAATAGAACCGGGTTCCAGCACTGCTCCAGGGGTTCTCCGGGTCAAAGTGAAGTGCGAGTCCGTCGTGCACGTAGGCACAGCGTTGACGTAGACGGCGTCTGTGACCAGATCGTTGTCCACAAAGGCGACCACCAGGAGTCGGAAGCTGGGCGTCATCTCCGGAGTCACGACGAAGGCCACCTTCGGGTCGAGGACACCACCTTCCGTGGCGATCTTCGCTGCCTCCATTACCCGGCCGCGGGAGAGGACCACGTAATACGCTGATGTCAGCTGGGAGTTCTGAGGTTTGAAGATCGAGGCCTCATACGTGGAGCCCGCCGCAACTAAAGCCCTGGGATCCTTCCTCTCGATGGCGATGTATTTTGCTGATCCGTCTTTGTATGCCTGCAGCATGACAAAAGCTTTGGCCTGCTGATCCTCCGGTAAGTCCTGGTCGGCGGTTTCCACCGTAAACTTGAATTCACCGTCACCAGACGTGGAAATTATGAGGAAGGTCACCACGCCGTTAACGTCCGTCCTGTGACTCATAGTTTGCACTCGACCGTTTTCACTCGTGAGTCTTGTCGGCACGCGCGCGGCTGGCTCACCGTTGGTGTACGTCACTTCCGCCACCACGTAGAACTTGGTGCCCGGCTTGAAGCTTTTCTCGCTTCGTGCTGTCGATATCAGGTAAGGCGAGGTGGTGAAAACGGCCTGCTCATTACGCCCGGTCTCCTGGATGCCCGTGGCCTCCTCGGTGACGGTTGCCTCGACCACCAGGCGCGCTCTGGTTCGGAACAGGGCGTCCAGCTGTCCCTCGCCCAGCCGGGACACCAAGTCGGTGCGGGCCAGCTTGTAGGTGCAGTCACCTGCCACTAGCTGTTTCGGCTTGTTGCTCGTGCCGATCTTTGTCAAGTCGCCGACCTCGTTCTTGACGGAAAACTTAAATGACACGAAACCACGGACGGGCTGCTGGTTGACAAACTTTGCTCTGACATCAACCGTAATGTCGGCAAAACCAGGCAGAATGTAGCCGGGGGTGTGTGTTTGCACCGAAAATCTTGGAAGTACATATTTTTCCACAGCGAAAGTCGTTGTCGTGTTTTGCTCAAACTTGTGTCCGTAGCGCATGACCAACGACCACTCGCCGAGGATAGTGGGCTTTGGCACTGCGAACTGGTGGCTCAGCATGATGTCCTGCCCCGGATTGAACTCCTTGCCCTCCAAGACCACGTTTTGAGGATTCCTGACTTCAAGCTTGAACGGAATAGAGGATGGCTCCAGAGTTCCATTCAGAGCAATGAACCTGATGTTCACGGTGGATCCGGGATGGTAGATGGGCTTATCCGTCTGCAGGAAGAGGTGGTCTGCGGATGCCCCGCTCAAAGGCAGCAGCACGTCACGACTCCACGCTCCGCATTGCACAGACAATTTCACCGTCGCGTCTCCTTTCGTGAGCTGCATATCGGGAACGTCTTCGGGCCTGACCAATACTTCGCCAACCACCGTAGAGCCTGCTGCCACGTCCAGAGTCGTCTGAAAGAAGGCGCCGCGTCCAGGCCGGTTCCCCAGGAGCGAAATGCCGACCTTCTGGGCGCGGCCACTGGTCATGGCTACCACGGTCTCCCTGGCACCCAGGCGGAGAGCGTTGGGCGTCAGGACGACGCACTCATTATTTGCCTGGACCCGATTTGGCGTTGATGCGATGAGCGCGCAGACGGCCCAGAGGATGGGGCCCGCGTTCATGTTGGCGGCCGACAGCAGACTGAGCCCGAAGCCGATCTGGACGTAAGGATGAG

>AAFM8975

ACCTTTCGCGATAAGCTAGAGGCATATTTTGCGCTGCACACACCCACGGCCGAGATGACAAGTTTATACCGCAGGCAGATTTTGTCCCCTATATGACTATAGGATAGGTGCTCTGAAGTGGATCAGGGCCTATCAGAAGCTGATCAAATTGTTTTTCTGTGAAAACAGAACGTGTTATCGGCAGGAGATTGATAGAGGCTACCACTGAATGTATGCAACTCATTTAAGTTGTTGGAGACTTTCTCTACCAAAGTGCAAATGTAAAATTCGCTGCATGGAAGAATGGGGGTCCTGAGAGAACAGCCTCTAGATTGTAGGTTTGCTTAGCTTTCTTTTTTCATCCGACGCAGTATATTACTGTTTTAACGCCGCTGCTATCGAGAGTGATGCGTGTTCAACAAACCTGGAGGAAAGCAAATTGAGAGCCTCTAATGAGTATTCAATATGGATAATTTAAGTAAGGGCATCATATACAAAAAAGTCGTCCGCACGGATAGTTTGCTCCTACAAAAAATACAATAAACGGCACTTCTGAAATGAAAACAATAGTAAACTGACATGCATCTGAATGCCCAAAGTTCAGTGGCGATGACATCCCCGTGAGTCTACGTCTTGGAGCTTGGAGGCAGTGCCCTCTCTAGGGCTTCAGGACCTTAAGCTCCCACAGTTCCCATGATGCATGGCGCTGAGTATTCGCGGAATTTCCCATTGCCAAGCGGACGCGCTCATGACGTATGGTGGATGCTTGGCTGGAGCGCCGCAAAATGTGGGCGCGTTCACGCTGGCACCGGAATTTCCTCCAGGAAAATGCGAGCCGTGTGATTGGTGAGTGGCGGCTACGTCACTGGCCCTGGCAGCCGCCTTGCCTGGCTAATCCCGACGTGAGCCACATGAGCACCGAGTACAGTCTACTTCCTCTCGAGGATGCTCTGCCATCTTCGGTGTTAAAGAGTCGCACGTTCTTGTCCAAAACGTACCTCATGACAACTTCGCCGTCCTTCTCGTGGGGTTCGCTGTCTCGGCCGAATATGAAGTAGCTACGTCCCTTGGTGAGGTCGGCCGTGTTGCAAAACTCCCTCGCCACGAATACTTTCACACCCGTCATTGCGGAGGTGCTGTTCTCAGCGCCTTCTTTCACAACGCTGTCCACACGGAAGTCGATGTAACGGTAACCGTTGACGATCCTGTTGGCAGACACCATTCCGATCCAAACGAAATCATGCTCCTGGCACGTGAGTTCGACTAGCTTCTGCCTTTTCTTCACTGTCGTTCCTGATCTCTCGACGTCAATGAAGGGCTCTTTCCTTGGGCATGCCGCTTCGGCGCACTGGCACTGATCATTTTCACAGATCAGCTTCAGAAGGGGACTCGTGGATCCGGGCCCGTAGAACTGCGAACACGAGTGCATTGGGTTGTAATAGTCATACACCTTCACAACGGCAGACTGAACATTGTAGACCACATGCTGACGCTCTATTCTGAATTTGACGCACGTCGGGGATTCCCAAGGTATCTTGTCGAAATAAAGGATCACATTTTTCTCAGTCATTTCATACTTGGCCAAAAGGGAGTTGTTGGCTTCTCTTGCCGCTTCCAGATCTTTTTCCATGGGTTTGAATCCACTGAACAGGCCAACCTCTATAACGGCCATGTTGGAATCCTCAGAGCCAATGTAGCGTGAGCACACTTCGATGTCGTAAGTAAGCTTGCTCTGTCGGGGTCCTTCCACTGAGACGGCGGCGGTGTCACTGTTACCGCTTGAGAATGTGTCCCTATCAGGGAAGAAGGGGGCCGCTGGCGAGTCCCTTCTTCGTGATTCAGCCGCCCATGGAGACCTCACTGGCTTCGCCTCAAGGTAGTATGGTGTCGTTGTGGGTGATTGCCTTCTAAAACTTCGAAACCATGAGCCAACTGAACGACGTCTTCTGCCTCCGCCGAACAAATCTTCGAGAAGTTCTGGAGGGAAATCGTCGAATTTTTTGGCCGTCTTTGCTGCCGGTTTGTGGATGTCAGCCCGAACCGTGATGTTGAACTTGCACAGAACTTCTGGTGGCACGACCACATTGTACTTCAACTTGACCGAGAGCAGCCCACTACCAGTGCCGCTGGCTTTGACGAACATTTTGCCTTTAATGTCGTGAATGTCCACTTGCTGAAGGATGGCAGCGTTGTCACGCTTAAGCCGAATGCTTTTCTGGAAGCCCCGTTTACTGCTAAGGGTGACATTACACGTGAGGTCCACATTTGGCTCTCGAGATTTCAGAGCGAATTCCGTCAACGCTTCAAGGGCCACCACGGTGTCCTGTGTCGATGCGAAAGCGCCGGACGCCGAGCGATGCGTATTGAGCCAGTTGACCACTGTCTTGCTCGTTTCGATGTCGTTGTGGGCAAGTAGCGCTAGAAGCGCATAACCGGTGCCTTCGACGACCAGAGGACTGGCCTCCATTCCCGTGCTTCTCGTGCTGAGCATGGGATCATAGATGAGCTTTTCCTTGAGTGCGGACAGGGTGGCGTACTTCTCCGTGTCGTTGGCTTGGCTGAGCGCGTAAGCAACTAGGGCCGCGACGTAAGGCTCGTCGCTGTCGAACGCGTTCTCCCGCAGGTACCTCTGAGACAGCTCCACCGTCCGACGCAGTATCGCTTTGTTGGTAGTTGCAAAAGGGTCGTGTGGACCATTCGTCATGCTCGGAGGCTTCATGCACTCCAAGAAAGTTAGCAGGACAAAGGCTGTCATGGGCACAGACCCTCGGACTCCTCCCAGCATGATCTTGTGCATGATGGGCTTCTTTTCCACATAGCTTCCATCGTACTTTTGGGCAGCCGCGAGCCACAGCATGCCGCTCTCAATCACAGTGGGGTCGATGTGCGTGTAGCGCCTCGACTTGCAAAAGATGCGCAGCACGAAGGCCGTCAACCATACGCTGCTCTTCCTGTGCGTGAATGCAGAAAACGAGCCGTCGTCTTTTCGAAACGATAGTTCCTTTTGATAGCCTTCTTTGATGTATCGGTAACCCTTCTCCTCTAGTGCTTCGTCGATGAGCTTGTTTTGCTTGAGGTACTCGAGAGCGTACAGCGTCGGTGCCATAAGCATCATGGTCTGCTCTCCGCAGCCCGTGGGCATTGTGATGAGCGACTCGATGTTCTCAAGCGTCATTTGAACGGATGGTCCCAGCTGATTTCCTATGACCATGAGAGAGCAGGTCTTTGTATCCGGGATTGCGTCAGGTGGCATTCTCGTGTTTACAGAGATCACTTGTAAGTTTGCGCCAGGGTCCAGTGAATCTTGGTAGAGCTCTCCTTTGATGCTTCTGGTTCTTCGTCTACGAGCGTTCGTTGGGTCAATGGGCACAGAGACACTCTTCTCTACTGTCACCCCTTCTGGCACGACGTTCAGTTCCTTCTCGACGACATCCTCGCCCATTGAGCTTTTCACGAAGACCTTGATGACGAACACGCCTTCCTTGAGTGGTATCACGGGAAAAGTGACGCTGGACGCCGAGTTGGCCTCCACCACCACAGGGCGGCGCTCCGAGCGCTCTCCCGGAAGGGCCCCGGTG

>AAFM45159

GCCAAACACAGAGCGCTGCGAAATCGACATCGTCGGAGACGGAGTGACTGCCGTTCTTCAATCTATAATCAAGAAGCCGGACCAAGCGTTCATTCATCCCACTGATTGTGGAGAACAGACCACGGCCAAGCTGATGCCAGTACTGTACGCCTACGAGTTCTTCAAGACCACCAACCGCATCAGCATTGCTGAAGAGAACGACGCTCTTGACTATATTCGAAGAGC

>AAFM49811

CCGGAACGCGTCGGTCGTCTGTGACTTGGTCATAGCGCACGCTTTCCATCAGCCACTGGATCAGTCCATCTTTCCCCGTGTTGTTGGCCAAGGACAGGGCATAGGCAGCCAGCGCCAACGCTCCCGGTGAGTCTCCGCGGTGCAAATTCCGCTCCAGAAACGCCGCTGCTCTCGCCCTCGAGGTTTGCGAAACCGAGTATCCTCCTTCAGCGCACTCCTCGAAGGTGATGAGAATGAAGGCAGTCAGCGCTGATGGGTGCCCGAAATTTAGCACTAGTTCGCTGAGGCTATATTCATGGAAGCCCCCATTTCCTTGCTGCCTGCTAGTTATATAGCGCAGTCCGCTTCGGATGACGTTTTCATCAATCATGACGGATTTCGTAGCTTCGCACAAGTTGCGAATGACGAAAGCCGTCAGCCATGGGCTAGCACTACTCCATTCGAAAACACTGAAAGAGCCATCCGGTTTCCTGTACTTGAGTATTTGGTTGTAGGCTCTTCGAATATAGTCAA

>AAFM846

TTTTTTTGTGAAAAAAATTAGTTTTATTCACTCTTTATGACACTATTTATAACGGTGAAGCCTCGCAGACGATGACGCGCTTTTCTCGCAAGTAGAAAATATATAAAGCTACTCTTCCACTCAGCTGGATAAGACGCGCTACGGGAAACAACATGGCGACGTCAAAAGTTCACGGCTCTGTTCACGCACACAAAAGCACACACTGCGCAGCGCTCCCTCAGTCGTCCGCACCCAATATCGAGGCTGTAAGATAAAACATCATGTGACCATTGTCTTGATTTCTTCGCGATGTGCGCTGTGGAAGCCATTAATCTTTTTTTTTTCGTTCGCGACATGGCACGTATGTCCTTCTCAAGAAGTGTGACCGGTATAAAAAGCCTTTGGTGTTGAACACTACCGAGGGAGCAAGAAAGCATGATTCTGGTAAAAAGAAAATTGGCAACCAAAGGATAAATGCGCATTGAATGAATGGTATGTTGTGACTCTTATTGTCTTGGCGCGTTCGTTCGAAAAAAAGAGACATAAAAAAAAGGGAGAGTAAGAAAAACTGATGGCCGATGTTCACTAGCAGGACGCCACAAGAGAGTAGGGCACGGAGTTGCTGTTTTCTTGCTCGTAATAGTCGTAGACTTTAACTGTGGCAGGTTTGGCATCTTCAATAGCGAACTCGCGGTGAACGCGAACGTCGAAGCACTTGTTTTCGGACGTTATTTCTTCAAAGTAAAAGTTAACCTGGTTCTTTTCAACTTCATGCCTCTTCAGCTTGACGTCTTTCTCACGGTAGAGTCCAAAGATGTGATCCTCATCTGGAGTGTAGCCCGATACCAGCTTGAGCTCCACCACTGCCATGTTGGATGGTTGCTCACCGTCGAACCTCAGACAGATTTTGAGCTTGTGGTCATTGCAGTCAGACGCCTCTTGAGATGGAGTGGCTGTGAGCTCGAAACCCTCGCTCTTCGGTGCAGTGTGAACGTTGTACTTCAGAGTCGCCGATATAAGGGCACATCCAGGACCTGTTGCTTCTGAAGTGAGCTTGTTGGGCAAGCTGACAACCTTCTTCTCCTGTACGACCAGTTTGGTGTCCTCCTTAAGGTTATACGACTCACTGACGTCAGTACCGTCGACCTTAACTGAGATGTCAACGGGATCTTTGCTAACGTAAGTAGCGAACGCCGACAGAGCCTGGAGACCGAGAACTGTATCCTGTGTGGATGGGAAGCCTCCGCGGCTGTTTCTCTTGGTCGCCATCCAGCGGACAATGGGCTGCGCCTTGCTCAGGTTCTCTTGGGCGTTCAGCTTGAGGTAGGCGAGCACTGCATACGCCGCCGTTTCCACGTCGGCCGACGCTGATGGACCCTTCTTGCCCGCGTTGCTCCAGTAGGTCAGGGCACCTTTGTGAACAGCGATGGACTCCAATTTCTCGAGGTAATCTTTGGCAGACTCATGTCCCGCCAGGGCCGCGGCGTAGGCGGACAGGGCCAAGTTGTGGGCACTGGGGTCACGCTGGGCGCTGATGCAGCGGAGCGCAGACTCAACGACCTTCTCGTCCGCAAGGCCGCCCTCCAGAAGAGCGGTGAGCACGTACGCCGTCAGCGCTCCCGGGGCAGTGGAGTTAACCTTGCCCTTCAAGCCGGAGCTAAGAACAGTGCCGATGTTCTGGAAGCAGCCGTTGGTTTTCTGCTTCGTGATGATCCACTTTATGCTCTCGTTGAGGTTTCCGGCGTCAATAGGAATGTATTTCTCAGCCTGCTTGAAGGACTTGACTACAAACGCCGTGAGGAACATGCTGCCCGAGTTGTCGCGGTTTCCAAAGGCGCTGTACGAACCGTCGTAGTGCTTGTACTTCTGCTGGCGCTGGTAGCCTGTCTTGAGATTCTGCACAGCCTTCCTTTCGATGTCTTCCTGGTTCTTGCCCGTAGCCTTAAGGTAGTCCAGGACATAGACGTTTGGCGTAAACTTTACCATGTTCTGCTCACCACATCCCGTTGGAACTTGCACCAGTGAGTCGAGGTTCTTGATTGCTGGGCCCATAATATCTCCTGTGACTTGAACGTAGGCACGGGCCGATCCTTCCACCAAGTCTTCAGGAAGCACCAAGTCAAATTCGTTCTTTGCGCCGCCTTCTCCAGCGTCTTTAGGACAAACGAAGACACTCTGCGTCTCTTCCTTAGGGAAGCCTTCCGCCTCGATGATGAGCGGTCGTGTGACTGCGTCCCTGGCCACCACCTTCTCAGTTGGCTGCTCTCCACATGCGGCATCGCTCTGCGATCCGGCCGCCGACACGGTCAGGTTCACCTCTCCGATAGTTTGTGGCCGGATCTGGAACTTGTGCACTTGGCTCTTGCTGCCGCACACGCAAAGCGTCGTCTCAGACTCGCCCTCAATGTGGAAGTCCGCGGATTCAGCCAGCTTCAGATCGACCGGCAGGCACTTTTCGAGGTAGTTGAACACGGACACCTTGACGGGCACCAGTTCTCCGCGCACCACAGAGTAAGGCAGGTTGAACGAGGCGAAGAACGGCTGGAAGGCCTTGATCTTTGCCGGATCTGAGATGCCGATTCCGTCCTCTGAGTTGATGCACACAGTGCTTCCCACCCACTCGGTGATGGTGTGCGGAATCTTTTCTTTGAAGTTCAGTTCGCCGTGCTCATCCAGCTCCTTCAGGTCCCACAGCCACGTCTCGGGGAAGTAGGTGCGCACTTCAACGGCCGACTTGGCCGGGATGTTGGCCGATGGTGCGCTCTGCGCCACAGAGTCCAGGGCGACGGGCACGCCGGGCAGGCCACNNNNNNNNNNNNNNNNNNNNNNNNNNNAGAGCGTAGGGTGGTCTGTCGTAGATGTTCTTCCGGCATGGCCTGGTTTCAAGCGTCAAGTCTGACATGACCACCACCCCTGATTCGTCGAAGGCGGTGATCGAATCGACGTATTCAACATTTGAAGATCTCGGTCCATTCCAGATGTGGCGCTTGAACTGTTGCGGATTCTTCGCCAACTGCTTGCGACAGTAGTCGTACGACGCCTGCTTCGGCCACGTGTAGCGGGTGATATCCAGACGCTTCAAAATGTCATAAACTTTGTCCTTTGTCAGTTGGTTATCCTGCTTCAGGAGGTGAACACTCTTGTCTACGACGCCAACACCACAGAAGGACCGAGGAGAACCATTCACATGGATGGCAGCAGATGTTGCAGGCTGTACAGTTTCAGATCCGAATCTCATGGTTACGTTGTTCTGGAGGCACTTCTCCACTTCGAACTGCTCGGAGTCAGCGATGACCTCGCCGTCGGGGCGGACGTAGAAAGCGAGCACTTTGACGCGTGGCACGTGGCTGAAATCTGGCTCGAGCTCGAACTCGAAGCTCCCTGTCGACACGCTGCCCTCGGCCATGTTGGTCGGAAGCGTCTCGTTCAGGTCCTCCTCCACCAGGAAGCTGTCGTCAACAGAGAGCGCCTCTTCCGGCTTGAAGGTCACATCCAGAACCTTGTCCTTGAGGATCTTGCCGCGAGCTATCACCTGTAAATGGAACTGCTTCTCTGAGTCGGGCTGACCCGTGTAGCGGAGGCGCACGGGGTGCTTGCCCGAGCAGCGCAAGGGTCCCTTGGACGGCTCGATCTGGATGAAGTTGTTGCTGGCCGAGTACCAAGCCTGGAGGTACAGAGTGCTCTTGGGCTGGTTGATCTTCACGCCGTAGGTGTCGTACTTCACAGTCTCGTAGTTCATCGCCACCGCCTCGACGCTGATGGTGACCACAGTTGTCTTCAGGGGAGGGATGGTGAATTTGATGATGCCTGACTCATCCGAAGTGTAGTTCTTGCAGCTGAGACGGCGGTCGGTGC

>AAFM972

ACGGCGAAGAGTGCGTCGCGGTTCGGGTCAGTCTGAAGTCTCTCGAGAATCTCGAGGTGGTTGGTGGTCCCAACAGCACCGACGCACTGCTCTGCCCCAACTCCAGCACATCCGCGTCATTCCCGTTTGACGTGCGCGCTACCACGCTTGAGGAGTCCCGGCTGGAAGCCCGCGTCCAGACGAGACCCGAGGCCCAGGCAGAGTATCCGGACGTCAAACTGTCGAACGTCAACTCAAGCGATACTGTCATCCAGACCATCGATGTTAGGCCTGAAGGCTTCCCCATCAGGGAAATCAACACGTTCCTCCTATGTGCTTCAGGTGATGCCGACAGCGCTCCGGACACCCTGCAAGTCAACCTTCCGACACCTGTCGCTCTTGTTGAAGGCTCCCAGCAGGTTGTACTAGTGGGCACAGGAGACATCCTCGCTCTCAGCTTGAACGACCTATCAGTTCCCACCATCACCTATTCCAACGCCGAGGGAACACTGGCTGTGCTTGCCTCCAGCGTCTACCTCCACAAGTACCTGGAGCAGACGGGAACTCTGACTGACACGGTGAACTCTGGCCTCCGATCCCGCATTCGCCAAGCGTCCCAGGCTCAAAACAGCTTCCGGTCCTCCGACGGCTCGTACGCTCAGTTTGGCTCTAGCGAGTTCCCGAGAAGCGTGTTCCTCACCGCCTTCGCCGTGAAAGCCCTGAGCGCCGCCAAGGAGTACCTGGGCACCAGCGTCGAAGCCGACATCGAAACTAGCGTGCGCTACGTCCTGCAGCACTGGAACGCTGCCACCGGTTGTTTCGTTGAGAACCAGCCCGGCTCGTCACCCTTCGGCCCCCAGACAGCCCCCGACTTCACCGCGGCCATTGGCGTCATGCTTCTGGAGAGTGGCTACAACTACGAGAACATCACGAACGGTGTCCTCCAGTGCATTGACGCCAGCAATCTGCCTTCCAACCACACGACTGCGCTGAACGCCTACTTTTCGGCGCTCGCTGGGCGCACGGACCGAGCTAACAGCGCCTTGGACACGCTCCTGAGCGAAGCTGATAAAAGCAGTGGCTTGACGTCGTGGAGCGGCGATGGCCTGACTTACGGCTCGGCAGACACCGCTGGTTACGCAGTTCTCACTTTGAAGTTGCTCGACCGAAACCTGGGAGAAGCGCTGCCAATCGTCCGTTGGCTCATGCAGCAGACCTACGCCCGATACACTTTCTCCTATTCCGAGGTCTACACCGTCGCCATCCAAGCCCTGACCCAGTACTCGAGCGTGGCATTCTCGAAGAACACAAACTTGACTATGAACGTTGCTGTCGACAGTTCATCCCCCGAAACGGTCTCTTTCCCGATCTCTGAGCAGAACAAACTGCTATACCAGGAGCGTCTTTTGAACAGAAGCGATTCGTACAGCTTCAAGGCATCTCTTGCTGACGGCAGCGCAGGGTGCGCCGCTTTGCAGGTTAAGTACTACTACAACTCCAGGAACAGTCCGGTTCAGCGAGGAATACAAGTCAACGTGTCCACGACATCTGGACCAGATTGCAGCACCCTTGAACTGGAAATATGCACACGGTACACCGAAGGCTTTCTGCGCAGCTCCGCGATCGTCCAGATCACCCTGCTTTCTGGATACTCGGCAGATGACCAGTCTCTCAAAAANNNNNNNNNNNNNNNNNNNNNNNNNNNNNNNNNNNNNNNNNNNNNNNNNNNNNNNNNNNNNNNNNNNNNNNNNNNNNNNNNNNNNNNNNNNNNNNNNNNNNNNNNNNNNNNNNNNNGAAAGCAGGTACCTGCAAGTGGCGAATCTTCAGGATGCAGTGGTGGAGGTGTACGACTACTACCAGTACCAGTACAAGGCAACGGCTGGCTACAGAGTAGAGGGCAACTGCACGCCCGCTGAAGTCGCGCCACCCACGAGCCTCAACGAAGTGGACAGCGTGTTCTTCGTTTAGAGATCTTTTCTCACTTCCTTTCCATCCATCCGCACTTCTCTAAGCATCTGGACAAACGATCTCAAGTGCCTCCGAGTCATCTGCCTTGTGATACCTTGTGTTCGTCTGTATTTTAGCAATCGCTCCTCTCATCCTCAATAAATGTATCCTCT

>AAFM16075

TGTCATCACTGTACTGAAAGCCACCTGTTTNNNNNNNNAAACAGGTGGCTTTCAGTACAGTGATGACAGACTGTAAAACTTGGGCCAAGCAAGGTCACCCAACTTCACTCCACAAGAAAGATATGGGCACCTGTATTTAACATGTTGCTCAGTTGCACTTCCAAGTTCCAAATGAGGAAAGTTCGCATGCTCAATAGGTTAAGCACACATTTTGGTTTTCAGACGTAAACTCTGAACAAGTGTATTTTTTTGCTTACCACAAAAGCGAATAAAGCTTCAACTTAATAATTATATGCTTCTTTCAAAAGACAGTTCTGGCTCTTCTATGTTGTTCCTTATAGTGTTCTTTTTATAAAAACTGGAGTGCATTGAGGTTCAACTTGGAGCACATCCCAGCAACTTGCAAGTTCTGTCAGTATGAAAGTTTTCTGCACGTTTATGTCCTTTTGCTGGTCAGGTCTGAGGAGCAGGCACTGTAACAGGCATGTTTGATTCAGGGCCATCTGGGAAGTCCAAGTCTTGTTCGATATTTCGGAAGTTTCCAAGATGAGTCCTGGCTGAACGAATATCTTCTTGCATTATTTCTGACTGCAGGGGCATTTGTGAGTCACTAAGGTCTTGTTGATCCCTCTCACAACTTGGAATTGTGTAGTTCCTTAGTACAATGACACCAGGATCATAGTAGTCCTGCAATACAACAGCTGCAGGAGCACTGTCTTGCACTTCAAACTCCTGGGTCAACATTAAGGTGATGCACATTCTTCGATTACTTAGCTCATCAAAGTACAAGTTTATTTTGTCAGGCTCTTCCTCAGTCTTCCTAACTTCCAGGCCATCAACAACCTTGCTCAAACGGAACCCAGTTTGCAGATGGACTTGTATGATCACCATTCCAGATATGCCCCAGGAGAGGAGGTAACTAGCACAAAGCTGTATCTTTGGCTTACAGTCAGCATGGGATGCCAGTACCATGAGATCAAACTTGTTGGTATCAGCCACTGCAGGAATGTTGAACTTTGCAGAGGCCTGCACAAAGGCACACCCCTTCCCTGTGACGTTGAGGTTCACCATCGCCGGCACTGTAGGGATGACCACCTCCTGAAGCAGCATTGCATTGCTTGGTTCCACAAAAAGCTCTTTGGCCTCTGTGTGTTGGGACGACACTCTCATTGACAAGTGTGTGCTTTGCAGAACAGTTGCTTTGGCAAATGCTGCCAGGGCTTGCAAGGCTACAATAGTGTCCTGTGTAGACGCGAATCCTCCATGGTCACTCCTCTGCTTGACCAACCACTGGACTGCGGGCAAGGCTTTAGGTACACCTGCTACCTTGAGCAGTGTCATGCAGGAGAGAATTGCATAGCCGCTTGTCTCTACAGCCACTCCCAAAGACACGTTGTGGCTCCAATGTAACGTGCCATCAACGTCTGATTCAGCCCTGCTCCAAAGCAAGTCAAAAGTTTGCTGTGCATCATGATGGTTTGAAAGAGCCAAGGCATAAGCCTGAAGTGCCAGTGAGTAGCTGTCCTGCTCGTGAGGAGTCTCTTCTATGAGGCATTGGATGGCTGCATTTAGTACAGATCTGGAGAGAGTCACATTTGCTTCAAGCAGTGCGATTAACACATAGGATGTTAGTGGTGAGAGTGATGTCCCCTGTACGCCGCCCTTGAGCTGGGTGTTCAGCACACGGCCTACTGATGGGAAACAACCAGTATCATATTGATGCCCGAGTAGCCACTGAGTGCTTAGGCTCAGCTCAGCATCATCAAAGAAAACATATTGGCGGGCTTGTCCAAAGGTCCGCACCACAAATGCTGCCAGCCACAGGCTAGGTTCAGGATCTGCAGTGCCAAAGGCACTATAGCC

>AAFM31523

CAGAAAATGGCACAGCAACTCGCAGCTTTGTACTAAAGCTTCCCAGCAGTGCCATTCCCGGCTCCTCTAGAGGACTGTTTATGGTTTCAGGTGACATACTNNNNNNNNNNNNNNNNNNNNNNNNNNAGTCCCTCGTTCAACTGCCAACTGGCTGTGGCGAACAGAACTTGGCTATGTTGGCCACCCGTGTGGTGGTGCTGGACTACCTCTTCTCTGTGGGACAATCCAATCATCCCTTGGTGGCCAAGCTCAGGAGAAGCATCATCACAGGTTACCAACAGCAGCTCAACTACAGGCATCAGAACAATGGCTCTAGTGCCTTTGGCACTGCAGATCCTCAACCTAGCCTGT

>AAFM42206

AAAGTATCTCGACACAAACATTACTGAGTCACCCATCTTGCCTTTCCGACAAGATCGCTTGTGCGTGTTCGGCAGTAACAAGGCCACTTTATCTGTGGTTGGTGATGTGTTTGGTCCAGCATTCCCAACTATGCCTGTCAATTCCAGCAGCCTGTTGTCAAAGCCGTTTTACTGTGGGGAACAAAACATGTTCAGCTTTGGGGCAAATTTGTTCACGGTGCACTATCTACGACAGACAAACCAACGTGACATGCATTTGGAAAGACAGGCATTCAAATATCTGAACTTGGGATACCAAAGGCAGCTGACTTATCAGAATGAGGATGGAAGCTTCCAGGTGTTTCGATGGCACAGTCAGCCAAGTGTTTGGCTGACGGCATTTTGTGCACGAGTGTTCCACAAGGCGACATTTCAGGAATGGGAGCAGTTCTTATACATCGATCCCACTGTCATACAAAAGGCCATTTCATGGTTGCTAGACAGGCAGTCTCCCGAAGGCTCCTTTCACGAGACATCTTTCTATGCTTATGACCGCAAAATGTCTCTTCCGTCTGAGAGGCCAGAAGACCCGGG

>AAFM47389

CCCACAATAGCCAACATCAGCATTCCCATTCCAAAGAATGCTGTTCCCGGATCGGAACGAATTAGTATTTCTGCAATTGGTGACCTTCTCGGCCCCAGTGTGAACAATTTGGACCAACTGCTAGTAATGCCACATGGGTGCGGAGAGCAAAACATGCTGGATTTTGTACCTAACGTTGTTGTTCTTGACTACCTGAAACGGGCCAACCGCCTCTCGCCACCTGTCCAAAACAAAGCTATACGGAATCTTGAAGATGGCTATCAGCGAGAACTGACATACAAACGCGATGATAATTCATTTAGTGCTGTTGGCAATACAGACAAGAGTGGC

>AAFM49624

ATTCATTTAGTGCTTTTGGCAATACAGACAAGAGTGGCAGTACATGGTTGACAGCATTTGTGCTCAAGAGCTTTGCCCAAGCTGCTCCGTACACTGTCATTGACCCCAAAGTACTTGAAAATGCTACTGCATGGCTGGTAGCACGTCAGCAGAGTGATGGCTCATTCAATGAACCTGGAGAGATCATTTACAAGCCAATGCAGAGTGGCGCTGGAAGTGGGCCTGCACTAACAGCTTATGTTTTGATTGCCCATCTAGAAAACAAGGCCAAGCAGACACATCCAGA

>AAFM19667

GGAAATACAGGGCCGACCCGTTCCTGAAGGAGTGCTGCTCTCTTGGTATGAAGCCCGACCGGATCGGCCGTCGCTGCAGTACACGCGCACACATCATGCGGCGCTATATGCAAGGGGAAAGGGGCGAGAAGTGCGCCCAAGCCTTTGAAGAATGCTGCCTTAGCACCACCGCCAGACCCGCATCAGATCCAAGGGATCCGCACTTACCCACACCACCCACCTTTCCGCAACGCGTGCAAGATCGAGAGCGTTTTGGCACTGAGGTCACCATGGACGAGATCGATGGTGACGAATCGCCTGGCACCATGGTACGGAATGACTTTCGAGAGACATGGCTCTTCGACGAACAAGTGATTGGGCCTGACGGAGTAGCCGACTTCGCTGTGAGCCTGCCGCACAGCATCACCACGTGGTCGGTGCAGGCGGTGAGCGTCTCTCCAGCGGGAGGGGTGTGCGTGCCTAAGCCAGAAGAGGTGCGCTCCTTCCAGCCGGTCTTCCTCCAGGTGGCGCTACCTTACAAGGTGGTGCGCAACGAGCAGATCGAGGTGCTCGCCACCGTGTACAACTATGGAAACCAGATACTGCAGGGAGTCGTCCACATATACGGAGTCGAGGGACTGTGTACCGGTGCTCAGAAAGGCG

>AAFM34109

CTGCGTTCCATTTGTACCAAAGGTCTCTGTGTATTCCTGTTGGATGTTCCTCGTGCCTCGCTTTTTAGGGTTTTCGGGATCCAAGATGACGGCAAACGATCGTGTTCTCTGAACTCCCGGAGGCCGGACATTCAGCTCCACTTTGACCTCGTCCCCTTCGCCGCTGGTGCTGCGTGCCTTCACGTGGATCTGTTTTTTTCCTGCGGCAAGGGGCACCACTGGGAATATGGCGGTGCGGCCATGTCCGGGTGGAATCTCGAGCACCCGGACCGCGGATGGTTTTCCTAGTTTGGCCCCAGAGCAGATGTCGTTAGTCCCAAGCAGCACCACCTTAGCCGTGATTTGTTTCGTGCCGTAATTGTAGACGGTGGCCGGGATCTCGACTTGCTCTTTCTTGACGACCGAGTAGGGAACGTTGACCTCAACGAAGAACTTCTTCGTCGCCACGATTTCGAGAGGTTCCACCGCGCAGACTCCTCCGCTGGGCGACACGCTCACAGCGTTCACCTCCCATGTGGTGACCGACGCCGGCAAAGTGGCCGAGAATTCGGCGGTGCCGTCGTCCCTAATTGTCATGCTATGGAATATCCACGTCTCACGAAAGTCGTCTCGAATTTGGTTGTGTACTCCAAGATACTCGTCTGAGAGTTCATCGACTCCAGCGGATGAGGATCTTTGAGCGGCGTCGTGTTCAGCAATGTCGCAGCACCGCTCGAAGGCTTCCACGCAATCCTGCGTGATGTTGGCGCTACCCTGCTCCATGTACTTCCTCAGGATGCCCGCCCTCTTCGAGCATGAGCGAAGGAACC

>AAFM36533

ATGCCGTGAGAATGACCGTGGCCACTTCGTTGCGCTGCATGTACGCCGGCAACGTGAGCGACACGAATAATGGCTGGAATCCGGTGACGTTTGCCACTTGGGAGACGCCGAGGCCGTTTCGTGGGTGCAGGCATACAGCAGTGCCCTGCCATGTGGTGATTGTGTCCGGGAGCGTCTCGGAGTACACCAAGGAACCGTCAGGGCTCACTCTCTTGA

>AAFM36534

ATGCCGTGAGAATGACCGTGGCTACTTCGTTGCGTTGCATGTATGCCGGCAACGTGAGCGATACGAATAATGGCTGGAATCCGGTGACGTTTGCCACTTGGGAGACGCCGAGGCCGTTTCGCGGGTGCAGGCATACGGCAGTGCCCTGCCATGAGGTGATGGTGTCTGGGAGCGTCTCTGAGTACACCAAGGACCCGTCAGGGCTCACTCTCTTGA

>AAFM8389

TCGCCGGATCAGTTCGGGCTTGGGCTGTCGGAGGGTCTTGTCGGCCTCGTCTCGCACCACTCTCTGCGCCCCAACGCCGCCGCCTTAGAGCGCGTGTGTTGTGTTTTGCTTCGCCACGCGACGTCAAGCCAGGAGCGAGACAAGAAGGGTAGCAAGAAAAAGCTCCCGGAACCGGCCTTAGCGCGCGCAGCAGAGAAAGCTGTGCTGTACAGTCTACGGAAAGGTGTCTAAGGCTGGCTTAGACGCAAGAAAACTAGCACAGACGGTGACGAGAGGTGCCGGTGGCCAGAGCTAGAATGCATTGGGGCACCTTCATTTTAGTCCTTGGTGCCTTGGCATCGCGCCTTGCTGAAAGTGGGTACATCTTCACGGCGCCGAAGATCCTGCGCAGCGAAACGGACGAACAGTTTCGGCTAACGCTCACAGACGTCAAGGAGGATGGCAAGGTCACCGTCCGCCTGCTCAAGTACAACAACGACAGCATCGTCTTGGCGGAACAAGAGTACGACATCAAGAACGGTGAAAGCACCTTTCTGCCGTTCCGAGTGCCCGAGCACCTCGACAGCCAAGCCAAGATCGAAGTGAACGGAACCTTTGGAGACTATGTCTTCGGCGATCGGAAAGAGATCGACTTCCAGAAGTCCAAGAACACGATCCTGGTTCAGTCCGATAAGGCACTTTATAAGCCTGGACAGAGGGTTCAATTCCGCGTCCTCCCTATCAACAATGAGCTGAAACCAGTCACCGATGTGAAGGCAACTATTTACGTGACAAGTCCCAGCGATGTAAGAATAGCTCAGTGGAACGACGTGTCTTTTGAAAAAGGCATTGTTCAGAGAGACTTTCAGCTTACAGAAGAGCCTGAGTTGGGTCTGTGGCAGATAGTCGTCGAACTTCCCACGCAAACCGTGAGGCAGCATTTTGAGGTGAACGAATACGTACTTCCCAAATTTGAGGTGACCATCAAGCCTCCATCCTACGTCTTGGCTGATGCAAAGGAGATCACCTGGAAGATTTGTGCCCACTATACTTTTGGACAGCCAGTGGACGGCACCCTGACGGTGAACGTGACATACGAGCGTTACAGTTGGGAGAAGGACGACTACCCAAAGATCAACCACACAGGACCCATCAACGGCTGCTTCGACATGACGGTGAACACGAGCCTGCTGCGCTTCAACGAGAACTACGAGATCTACAAGCGCATCTACCTCGTTGCACAAGTGAACGAGACCGGCACGGGCATCACGATGAACAAGACGAACTACATCAGCCGCAGCTTCAACCCGCTTGAGCTCAACTTTTTGGAAGGAGACCGCGGAAAGAACTACTTCAAGCCCACCATGCCTTTCTACGGCAGGCTCCTGGTGAAGAAGCCTGACGGCGTTCCTGTGGGCGGCGAGCTGGTGCAACTTTGTCTGCTGTCACAGGCTGAGGAGATCAAGCCACGGTGGTGGCGCACCGACCGCCGTCTCAGCTG

>AAFM14608

CCAGTCGCGACGAGCACGGCGCGTGCGTACTCGAGCACGCGCGGATTCACCTGACGGCGAGGTCGGAACCCGGTACAGCCAACAACTCCCTGTCTGTTTGCGCTTCGTGGTTTGGCCCCAGTCAATCACACGCCCGGCAAAAAAAGGTGCCATAAGTCCCCACCATGGCGTCTGGTTGGACGCACCAGCTGCTACTGCTGGCAGCTGTGGTCGCGCTGGGAACAACGCCCGCCGCCGCCTCTATCGAGTACCTTGTGACTGCACCCGGATTCTTGACTCCCAATACCGACATCAAGATCACCGCTGTGGTGACCAACCCGGCGTCGGCGGGACAACTGAAAGTAGATCTGTGGGGCACTCGGGTGGGAATCAACGAGAACAGCACAGTACTGGCAAGTCGGACGTATGACATCGGCGCAGATGGCATCGACGCCGAGCTGCTGTTCCACGTTCCCGACCTAAGCCCCAGCGTCTTCTACAACCTGTACCTCGATGTGACCGGACAATTCGGCAGCGACCAATTCCAAAACAGATCGTCTGTTCAACTGGAATACGTTACCAATGTCAACGTCGTTGTTCAGACGGATAAGCCCATGTACCGTCAAGGAAGCACAGTGAACTACCGCATACTGCTGCTTGACAACAACCTCCTCCCCGTGACGAATCAGCTGGCCAACATTACCATCACGAACCCGTATGGCCAGCTCTTGTTCCAGCAGCAGTACGTCAACTTCACCGACGGCCTCCTTCAAGGCTCCTACGACCTCCTTGCCATTACCGACGAAGGCACGTGGACGATCAGCGTGTCGGCAGGCACAAACTCTGGTTCGGCAACGTTCCAAGTTCAGGACTACGTGCTGCCAAAGTTTTCCGTTACCATCACTCCTGACACCAATGACGTCGTCACGAACCCTACTGTGCTGTACACTATCTGCGCCAAGTATACTTATGGGGAAGATGTCAAGGGCACTGTGC

>AAFM20341

CTCAAGTACAACGTGCTCCTCCCACCTGAGCTCGTGTGCATGTTCAACATTTCAGTTCGAGCTGACATTCATACCCCGAAGGAGAAAAGCGCTGCAGTGTCGGATTTCCCGGAAGAGCTTCTGCTCGAGCTACTGGGAGAAAACAGGGCACGTCGATCCGGCCGTGCCGGCTGGTCCTTGCCAGGAATCACTGGTACTACGGCCTCCCAAAGCGGCTCGTCACAGCAGAGCAAGCTCATTTACGACGTCGAAGTGTGTGCACAGTACCTTAGTGAGTCGGACTCTAACATGACGGTCATAGAAGTTGGACTCCTGAGTGGCTTTGTTCCCATTGCAGAGGACCTGAAAGCGGTCAAAATTAAAAATCAGCTCCTGGCTAACTACGTCATAACTGAGAAGAATGTAATGCTATATTTTCATTGGATACCATGGCGAAATGCAACCTGCCTCAAGTTCAGGACAGAGCGTCAGCACGTCGTCTACAACGTTCAATCGGCTCTCGTGAAGGTGTACGACTACTATGACCCGACGCGTTCGTGCACGCAATTCTACGGTCCTGATTCGACAAGCCCGCTCCTGAAGCTTACCTGTGAACGAGACGAGTGCCGATGCGTGGAAGATGAATGCCCACCCAGAGAACCCTTCAGAACAATTGACAACGTGGGAGCGACTTCTGAAAATAGGCGCAAGCTGCTCGACCTAGCTTGCAAAGAGCACGACTTCGTGTGGGTTGGAACCGTGTCTCGGAACCATGTCACCAATGGTTATCGCCAAATCGAACTTCGTGTTGACACGGTGATCAAACAAGGCGCTGAACAGAGGGAGGCCGCCTTGACGGGCACTAAGCTGTTCGTAGCTCGCAAACACTGCAACACGGCTGACATGAGTGAAGGCTCACGCTACTTTGTCTTCGGCCGAGACGGCGAGCTCTTCGAGAAGGACGGCGTAGTTGTGTCTCGCTACATTCTGGATAAGCATGTTCGTCTCTTCGACACAAAAACGGCTGGGACATCACAAAGCGCCAGCAGGATAAACTCCGTCCTACAGTGGCTTACAGCAAGCATGCAAAGATCGGGAGGATGCCAAAGTAGTTGAGAGCCGTCCTTCTCAACACCAGCTGTCATATTGCGGATATCGTGAACATGGGCTATATAACGGTCATTTTGTGGGATGCAAGCTTCACTGCATGACGGCTGCGTCGACAACTGAAGGTGGCAAAATATTCTGTGAGAGCATTTCCGGTCAACGTCAATACGATACCATGCCACCGAGTGCTGCGACACGGGTACAAGATCGATATGCAGCCTTCCGGGAGATTTTTTCTGTCTGTCAAGCAGCGGTCTTTCATTAAGAAAAGAACGCTTCTGCGCTATTCGATTGACGAGGGTGATTAGTGTAGCCCTTTGGGGGTCAATCAAGTCTCAGCAGATGGAACTGGACGTTCAGGATGTTTGGCGCTTGTTCTACGCACTAACTTAAATGCGATCACACAAAAGTGAATAAAACTGCA

>AAFM22686

ATTTGGAGTAGCAGTGCTACAGGTCTCATGGTCATTCAACTTGGCCGTGTCCTCAGAATCTCCTTCATTTTTCTTGAATCCTTTGCTGGACAAGACTTCTACAGAAAACTATCTTCAGCTCAGTGTCTGCACGCACTACCGAGGGGAAGGAGACGCCAGCAACATGGCTGTCATGGAAGTGGCCCTGCCTTCGGGCTACCTCTTTGACTTTGACACCTTGAGCAGCATTCATCGGACCAAGGAAGTGCGCCGGGTTGAGAGCCAAGATGCCGACACAAATGTTGTCATCTACTTTGACAGGATTGGAAGACAGGAGCTGTGCGTCACTGTACCAGCCCATAGGGAGCACAAGGTTGCCAACCAAAAACCAGTCCCAGTCAAGGTTTACGACTACTACAACTTGGCTCGCAGTGCTAGAATGTTCTACAGCCCTCACAAGGCAACATTATGCGACATCTGTGAGGGTGTTGAGTGTGGCAT

>AAFM28075

GAGTATGCCTTCACGTAGCTTGACTGCAGTTTACCTGCATTGAATTCTTGTTCGAGAGAGAAATTAACGCAGTGCGTCATGTTTGCAGCAATGAAGGGTACGTAAAAGTCAACATGGCGACTTGTGATAGTATAAAGCTGAATCTTTCTTTCTTTCACGAGCTCATCCAACTCGGTAGCATTGGGCTTGAATCCAGTCAGAAGACCCACTTCCAACATGACCATGCCATCTGGAGCATCCTCGAGTGGACTTGCACAGGCCTCCATGCGGTAGTTTGGCTTCAGGTCAGATGGTGGACGTGAGGAGGTTTTTTCGACACTTCGAGAAACTCGTGTGAGTATGAGTTCTATGTCCGGTTTCGTTTCCACAAAGTTTGCTGTGATGTTAAACTTGCAAAGCTCACCGTCAAGAACTTTAGCGTCGTATGTGTAGTTAAAGTACAGGATGCCGGTACCGGACCCTTTGACTCTGACGAAGATTTTCTCTCCCGGTCGATCAATCTCGATCTTATTCAGGATGGTGGCGTTATCTCGCTTGATCCTAATATTCTTCTTGAAAATTTTGCTGTTGCTCAAGGTCACCTCGCATGCCAAGTCGAGGTTGTTTTCTTTGGCGTATGCGGCAAACTTGGTGAGCGCTTGCAACACCATCACAGTGTCCTGCGTAGATTGAACAGACCCAGTGGGGCCGGTCCTTTTGCTCAGCCAACGCTGAAAACTTCTTATGATCTCGCTTTGTGCGTTCTCCCTGATGAGCGCCATGAGTGCATATGCGGTTGCTTGTGCAGACAGCAGTTCGCTGCTCGCCGGAACGTGTCGGTCGTCTGTGACTTGGTCATAG

>AAFM35537

CTCACGTTACCGGTCAGAGTCGTTTTGTTCTCTTCTCCTTCCTTTATAACGAGGCTGATGTTGAACACTAGGTGCCTGTAGCCCGTGGTCACGTACTTGGAAACAACTTTTCCTTTCCACACGAAGTCATGCTTGTCACATGCTATTTTTTCTATGATGGCCCGCTGCTTCTGTGTTGTTTTGTTTAAAAGAAGTTGGAATGTTTCTTCAATGGGGCACTCCGCTGCGGTACATTTGCATTGGCTGCCTTCGCAAAAGAAGCCCACCAAGGAACCAGCGGGCCTGTAGAACTGACTGCAAGAATCCTCTGGCTTGTAGTAGTCGTAAACTTTCACCGTTGCAGTTTGGAGGTTTGTTACCGCAAAATCTTTTTTGATTCGGAAGGCCACACACGTCTCAATAGCTGACTTTATCGTGGCCAGGTAAAAGATGACGCTCTTCTCCCACCAAGGAACCAG

>AAFM56280

CTGTGTCAACTTTACTGCCCAGCGTTGGTATCCTGTTGCCAACATGACACGGTTTATTTCAATTCGTGTCTACGACTATTATGCACCGGAGCGGTTCAACGAGTCAATGTTTGAAGTGTACAACCTGTACGCACTCAGCATCTGTCACGTGTGTGGCTCCTACCAGTGCCCATTCTGCCCAGTGTTCAACACAGCACCCTCTTTTTTGCACCTGAACCAACGGCAAGCACTGTTGCCCCTGACTGTGCTTCTGGTATTGCTGTGGAGGCACCTCTTCAAAGGCTAGCAGTAGTTGTGGTGCTGTGACAG

>AAFM4035

GCTGACTGCGCAAGTTGTTCTCACGCTTTCCAAGGTCACCGATCTTACAGGAAGCATTCGAGAGCATGCCAACACTGCCAAGCGCGACGGCGTACGGTACCTGGAGACACAGCTGGGCCAGGTGACGGACCCATACACCGTAGCCCTGACGACTTACGCACTCCTTGAGGCTGGCTCCAGCGAAGCCCAATTCGGCTACAACCTGCTGGAATCCATGAAGAAAGATGCCGAGGGGGCTGTCTACTGGAGCAGCGTGCACATTCCGCCGCCCGTGATCGTGTCCCAGAGCCAGCGGCCGTTCCTGATGCCCCGACTACCGCACCCAGAGGACTCGTCCGCTGTGGAGGCCACTGCGTACGCCCTGCAGGTCTACCTCAAACTTGGCGGCCTCTTCCAGGACCAGATCGTGCGCTGGCTGAACCGCATGCGCTCCACAGACTATGGCTTCATCGGCACACAGGACACACTTGCAGCCCTCGAAGCGCTTACACAGTATTCCTTCCGAACGCACGTCCGAGGCATAACAGAAATGAAGGTCACTGTGGAGTCATCATCAAACCCCGGCTTTCC

>AAFM5885

GTCGAGGTGCTGCAGAACCGCCGCGTTGTCCCGCTTGATCCGGATGCTTTTCTGGAAGGCGCGCTTGCCACTGTGAGTCACGTTGCATGTCAGGTCCATGTCGGACTGCTGGTACCTGAGTGCGAACTCCGTCAACGCCTCTAGGGCTACCACAGAGTGGTGCGTGGGGATATCAGACGACGATCGGTGCAACTCCAGCCAGTTTGCCAACGTTTGGATCATCTCGGTGTCGCTGTCGTTGGAGGCCAGTAGAACCAGCAGAACGTAGGCTGTGCTTTCGACCACCAAGGGGCTGACATCACTTCCCGCACTTCGCGTGTTATGTACCGGGTCGTAGATGAGCTTCTGCTTGAGTATCGATGTTATGTTGTGAGGCTGTTGATGCTCCGTCATCAAGAATATGTATGCCATAAGCGCTGCACCATGAGGGTTGACTCTTTCCAAGTCGGCCATGTTCGTGTAAGCACCACCGGGACTGAAAGGCTGTGCTAGCGCCTGCCAGGTATTGACGTCGCTGCCTTCAGGCTTCACCACGTGCCAGCACTCCCTGAAAGTGCGGCCAACGAAGGCTGTCGTAGAAACGGCTCTATCACCAGGTCCCGGGATGCTCTTGTCCACCAAGGGCCACTCGAAGTGCAGGTTTCCACTCTCCTGCCGTTTGCGTATCAGCCACGCCAAGCCTGTCTTGAT

>AAFM45413

TTCAATCTCAACATACTTGAGGTCAGAAGGCAGCTCAAGGCGCTGCAGAACAAGAGCATTTCCACTATGAATCTTCAAGATGCGGCTTCTTCCATCTCCATACTTCACACTGGCTTCAATGGCAATTGTCTGAGAGACAACACTGGCTGCTACTCGTGCCAAAGCCTGAATGCCTATCACAGTATCCTGAGTAGAACTGTAGCCACCATTCTCATTTTGCTTGGAGACAAGCCACCTCATGACAGGAACAGCATTGCCAATGTCGCCCCTTTCAGTTAGGGTAAGCAGGGCATAGGCAGTCATCTCCACATCCTTGAAGCTGGGCTTGAAGAAATAGTCAGACTGCTTATCACTAGTGTTTACAGCCAGGGCAGGGTCCTTCCAATAGATAGTGTCATCTTCTCGTGTTGCAAGTGAGAG

>AAFM50567

GCCCGGTGTTGAAGGAGCCTCCACAGTGACTTTGATGTCAGTGACATCGCGCGAGCGAGAGGTGATCGAGAACTCTAGCAGTGCTTCCATCGCCAGCAGCGTGTCCTGAGTTGATGCCCATCCACCATATGAGAGGCGCTGTGTGTTCAGCCATTCTACAATTTCCTTCTGAACAACTGCCTGGCGTTTTACATGCACCAACAGCCCATACGCCGTAGTCTCCACGTTGGATGCATCATAAACGTAAGGAAGTCGTGGGTGCAGGTAAGGTTTGTTGTTTTCAATCACTACTGTTGGGGCTGGCAGATCAGTG

>AAUF656

CTCCCACAGCTTGGAAGGCAACGCGCTAAGCTGGCCACGAACTTCCTCGTTGGTTGCGGCACTTAAGTCGGCTGCCACGCTGTCCGGCCAACTAGTCACCCTCGAGCCAACATGGCGAGCACACTGGCACTCGTGTGTGCCCTCTTTGTGGCTTTGTGCACGCCGCGGGTGTCTGCCGAAGAGAACTGCTTTGTGGTGGCACCGAACGTCTTCCGGCTGGGCACCGACGAAACCTTCGCGGTGATGGTGGACGGTGTCAAGAAAAGGGTGACGGTCACACTTCAGAACTACCCGAGCAGTCGGGGTGCGTTTTTCCACTGGACGGGGGACGTGGCCAGTGGTACTCCACGAATAGTGGACATCGCCGTAAAGGAAACCGACTTACCTGAGCTGCTCTACGGGCGGGACCAAATCTACGTCACGCTGGAGGTCACTTGCGGCAGACTGTGGACGAGGAAAACGCAGGTCTTGGTCAGCCCTGCATCGGGAGAGCATTTCTTCCTCCAGACAGAAAAGCCGATCTACCACCCGGGAAGCACGGTTAACATCAGGTTCCTGGCCGTCGATGGAAAGCTGGAACCATCCGCGAGTTCCTTCAGGCTCGAAGTTCGGAACCCCCAAAACGTGATTGTGGAAAGGACCGATTTTCAGCCGAGCAAAGAGCTGATGCTCACGCACACCTACGAGCTGCCCGAGCGCACGCTGCTGGGAGAATGGGCGCTCGTCGTGAAGTACGGCTACAAGTTTCAGCAAAACACAACAGCCACATTTCTGGTTGATAAGTACGTGCTTCCCAGGTTCAAGGTTGATCTGAGCGTTCCGGATTACGTACTGAGCAATTTTTCCACAATACCCTGCAGAGTGAAAGCAAGCTTCGTGAACAAAATGCCCGTCCATGGAGTTGTGCTGCTCGAATTTGGGTTGAGGCACGAGGTTGGAGACATCTGGTGGTTTTCCAGCAGCAAATCGCCAAAACTTCTCGAGGGCGGCGAAGCTCATTACATTCTGAGACGAGAAGCCCTGGAACCAAACATTGGAGACAAACTGCCCGACTTACACGCCACCCACGGTCGCCTCGTTGTAAAAGCAACGGTCACAGAGGAAGCCACCGGCGCAAAAGAGTCGGCGCAAAGTGAAAAGACGGTCTTCTGCAAGACGCCATTCGTGGTATCCCTCAAGAAGAACCAGCGAAGCTTCAAACCTGGCTTGAAGATCTATATCACCGCTGAAGTGACGTATGTGAACGGAAACCCAGCCGCAAACGTGCTCACAAAGGCCACGCGCCTGAACTCAAGAGACACCTTCACGGCCAGGACACGCAGTGACGGCGTGGCAACCTTCCTAATTCCGGTGTCCTTCGAAGATGGAACCCTGTCCGTCAAGGTGGAAACTGCCGATAGCAGATACAGACCGGAGCACCAAGCCCGAGCACAGATTAGCCTGGAGCCGTACTCAACCGTTCAATCCGGTTTCATTGCCATCGAAAGGAAAGACCCAAAAAGCTTTCCAAGAGCACGCGACACATACGAAGCCGTGTTATTCACACATCGCTTCGACAAAGTGTCTTCTTCGATCTACTATGTGGTCCTGTCCAAGGGGAGGATACAGCTGGTCGACAAGCTGCTAGAAGGAAGGAGAATCGAACAAAACATAGCCTTCTCTGTCACACCGGAAATGACGCCAAGCTTCCGGGTGGTGGTGTTCGCCATACTTGATGGGCGCGTCGTAACCGACTCTATTTACGTAAACGCCGAACCGGCGTGCACAACGACCTCTCAGTTCACCCTTGAAAGGAGAAATTTGGGAAATCCACCCGAGCCTATGTCCCAGGAAACTCTGGTCCTTACTGGAACACGGGGCACGGTTGTCGGCTTGTTGGGTGTCGATCAAGCCATGTATCTGCTCAGGAAAAAGGACCTTCTGACGAGGGACAAGTTGTTCCGGTCGTTGGACTCCAAAGACCTCGGCTGCGGTGCGGGTGGAGGAACGAACGCGGCCGAGGCACTCTACAGCTCCGGCATCGTGCTGCTTACGCATGAGGACGTTGGCAACCCCATGAGAAGCGGTGTAGGTTGCCACGAGGAGCGAAGACGGAGACGGCGGGATGCACAGAACATACTGGAGGAGTACGAGGACGAGACGGTGCGAAAGTGCTGCTTGCGCGGTCAGTGGCCAGACAGGTTCCTTCGCTCATGCTCGAAGAGGGCGGGCATCCTGAGGAAGTACATGGAGCAGGGTAGCGCCAACATCACGCAGGATTGCGTGGAAGCCTTCGAGCGGTGCTGCGACATTGCTGAACACGACGCCGCTCAAAGATCCTCATCCGCTGGAGTCGATGAACTCTCAGACGAGTATCTTGGAGTACACAACCAAATTCGAGACGACTTTCGTGAGACGTGGATATTCCATAGCATGACAATTAGGGACGACGGCACCGCCGAATTCTCGGCCACTTTGCCGGCGTCGGTCACCACATGGGAGGTGAACGCTGTGAGCGTGTCGCCCAGCGGAGGAGTCTGCGCGGTGGAACCTCTCGAAATCGTGGCGACGAAGAAGTTCTTCGTTGAGGTCAACGTTCCCTACTCGGTCGTCAAGAAAGAGCAAGTCGAGATCCCGGCCACCGTCTACAATTACGGCACGAAACAAATCACGGCTAAGGTGGTGCTGCTTGGGACTAACGACATCTGCTCTGGGGCCAAACTAGGAAAACCATCCGCGGTCCGGGTGCTCGAGATTCCACCCGGACATGGCCGCACCGCCATATTCCCAGTGGTGCCCCTTGCCGCAGGAAAAAAACAGATCCACGTGAAGGCACGCAGCACCAGCGGCGAAGGGGACGAGGTCAAAGTGGAGCTGAATGTCCGGCCTCCGGGAGTTCAGAGAACACGATCGTTTGCCGTCATCTTGGATCCCGAAAACCCTAAAAAGCGAGGCACGAGGAACATCCAACAGGAATACACAGAGACCTTTGGTACAAATGGAACGCAGGTGATACACATTCGGTCGCCACGTCCTGATTTTGCTTTGCCAAACACAGAGCGCTGCGAAATCGACATCGTCGGAGACGGAGTGACTGCCGTTCTTCAATCTATAATCAAGAAGCCGGACCAAGCGTTCATTTATCCCTCTGATTGTGGAGAACAGACCACGGCCAAGCTGATGCCAGTACTGTACGCCTACGAGTTCTTCAAGACCACCAACCGCATCAGCATTGCTGAAGAGAACGACGCTCTTGACTATATTCGAAGAGCCTACAACCAAATACTCAAGTACAGGAAACCGGATGGCTCTTTCAGTGTTTTCGAATGGAGTAGTGCTAGCCCATGGCTGACGGCTTTCGTCATTCGCAACTTGTGCGAAGCTACGAAATCCGTCATGATTGATGAAAACGTCATCCGAAGCGGACTGCGCTATATAACTAGCAGGCAGCAAGGAAATGGGGGCTTCCATGAATATAGCCTCAGCGAACTAGTGCTAAATTTCGGGCACCCATCAGCGCTGACTGCCTTCATTCTCATCACCTTCGAGGAGTGCGCTGAAGGAGGATACTCGGTTTCGCAAACCTCGAGGGCGAGAGCAGCGGCGTTTCTGGAGCGGAATTTGCACCGCGGAGACTCACCGGGAGCGTTGGCGCTGGCTGCCTATGCCCTGTCCTTGGCCAACAACACGGGGAAAGATGGACTGATCCAGTGGCTGATGGAAAGCGTGCGCTATGACCAAGTCACAGACGACCGACACGTTCCGGCGAGCAGCGAACTGCTGTCTGCACAAGCAACCGCATATGCACTCATGGCGCTCATCAGGGAGAACGCACAAAGCGAGATCATAAGAAGTTTTCAGCGTTGGCTGAGCAAAAGGACCGGCCCCACTGGGTCTGTTCAATCTACGCAGGACACTGTGATGGTGTTGCAAGCGCTCACCAAGTTTGCCGCATACGCCAAAGAAAACAACCTCGACTTGGCATGCGAGGTCACCTTGAGCAACAGCAAAATTTTCAAGAAGAATATTAGGATCAAGCGAGATAACGCCACCATCCTGAATAAGATCGAGATTGATCGACCGGGAGAGAAAATCTTCGTCAGAGTCAAAGGGTCCGGTACCGGCATCCTGTACTTTAACTACACATACGACGCTAAAGTTCTTGACGGTGAGCTTTGCAAGTTTAACATCACAGCAAACTTTGTGGAAACGAAACCGGACATAGAACTCATACTCACACGAGTTTCTCGAAGTGTCGAAAAAACCTCCTCACGTCCACCATCTGACCTGAAGCCAAACTACCGCATGGAGGCCTGTGCAAGTCCACTCGAGGATGCTCCAGATGGCATGGTCATGTTGGAAGTGGGTCTTCTGACTGGATTCAAGCCCAATGCTACCGAGTTGGATGAGCTCGTGAAAGAAAGAAAGATTCAGCTTTATACTATCACAAGTCGCCATGTTGACTTTTACGTACCCTTCATTGCTGCAAACATGACGCACTGCGTTAATTTCTCTCTCGAACAAGAATTCAATGCAGGTAAACTGCAGTCAAGCTACGTGAAGGCATACTCTTACTACAAACCAGACTTTTCTTGCACACGATTCTACTCGCCCGACAAGACAAGCCCCCTACTAAAGGTTGATGATAAGTGTGACGACTCCGATGTCTGCGTTTGCGCGGAAGGTGGTTGCCCGCCGGAGAAGCCACTCGACAGGTTCATAAAAACACGGGACAACGAATACTTTGAAGACGAAGAACAGCGTGAGCTTCTGAGAGAATTCGCCTGTGACGGAGTTCACTACGTATGGAGAGGCAATTCGACAGCCAATGTATCGACGGATGGCTTCATAGAAGTGGCTTTCCTCATCACTCAAGTTCTCAAGCCAGGCCAAGAAGACGACTTGAAAGGAAAGATACGTCGCATCAAGGCTCGTGATACCTGCAATACATTCAATATTCCCAATGGCGAGGAGTATATTGTCATGGGAAAAGACTCCACGTATATCGAGAAAGAC

>AAUF4516

CAGCGCGCCGAACCCTACATCTTGCGTTACCAGTGTCAAGGCGCCGGAGGCAGTCGCGTGTAAATACTAAGTTGCATGGCTCCACACGCCGGTTTCTGCGAGAGACTCAAGGTGAAGTTTACTGTCCAATGCTGTAATTTTTTTCCCTAACTGACCAAGTACCTATGAAATAAACTGCTCTTCTTTCAGCTAGAAATAACCGGATGTATCCAGGCAGCTAGAGTTCAAAAATCCAAGTACCCAAAATTTATAATGGGCTTCCTGCATGTTTTATTATTTATTGACTTCAAGAAACTTTAGCAAAAGAAAAGCCCATAGACACTGTGCTCATGTGTTGCAACGCTTCGACTCGTCGGAAAACTCGTTGATGAACCAGCCGACGAGTTTTACGAAGTCGCACAGCTGGTCTTCTGGCTTTTGACGTGGCCTCTTTCCGCTCGGTTTCGTCCCGGGCCGGTTTCTGCCTCCTCCCTCTGTCCGACTTGGTTTTTTGGCTGCTTCCACTGGGATGACAACGGAGTCGCTGTCTATCACGTACACGTATTGTTCATCGCCGAAGCTGGGGTCCTTCTCCTTGTACTTGGCGTCCTTGCCCATGATGATGTATCGGCTGCCTTCAGTCAGTTTGAAGGATTCGCAATTCTCGCGAGCCTTGACCACTCGCGTCTTGTTGAGAAGGTCCTGCTGTGACTCAATGCCTGGCTTGAGGATTTTGTTGATAAAGAAAGGAACAGTGATGAATCCATCTTTGTGGGCTCCATTAAGGGGCGTGCCAAGCCATACAAAATCAACATTGTCGCAGGCGTGGTATCTCATGTATTCTCGGCAATCTGAGGTTTTCAGAACAGCACCCTCTTCTTTCGTGAACATCTCCTCGACTTCTTGGGGCGGGCAACCACCTTCCAAGCATCTGCAGACTTCAGAGTGACCCNNNNNNNNNNNNNNNNNNNNNNNNNNNNNNNNNNNNNNNNNNNNNNNNNNNNNNNNNNNNNNCACGATATATCGGGGTCGTAGTAGGCGTACGCCTTCACGGAGCCCGACTGCAACTTGCCTACGGCGAATGCCTGCTGCAGGCCAAACTCGACGCACACGCTGGCGTTCGATGGAATGGTCGACAGGTAGAATACCACGCTACGCTGGCTCAACTCGTACGAGTCCACACGTTTGTCGGCCACCATCTTGTCCAAATCGGCCAACACGGGATTGAAGCCGGTTAGGAGACCTACGTCCAGGATGGCCATGCCTTTCAATTTTTTTTCAAGAGACCTCGCACAGACTTCCATCGAGTAAATCTCTTTGAAACTCCCTTTACCTTCCAACGCTTTCGACACGTTGACCTTGTGCTGTGTGAAGTTTATCTTCAGATCAAACTTGCACAAAAGGTCCACACTGACAGGGGACTCGTAGCTTGTGACAACGTACATAGTCGCCGTTCCAGTTCCTTTCACATTGACAAATATCTTGTCATTGCTGTCTGGAATCTCGATCCTGTTGCGCTGTTGGGCGTTGTCTCGCTTGATTCGAAGGGTGCGGTTAAAGTTACGGTCCCCGCTCAACGTCACTTCACAGGTCAGGTCAATGTCTGCGTCTCGTGCGTAGAGGGCATACTTGGACAGGGCTTGAAGCGCCACCACGGTATCCTGGCTGGACCGCAGCGACCCGCTGGGGTTCATTCGCAGGTTAAGCCATTGCACCATGGCCGTGATGTCGTCCCTACTCTCGCCGGCATTGAGCAGTGCCATGAGCGCGTATGACGTGGCCTGCACCGATAGTGGCTCGGAACCGGCGGACACATACAGCCCTCCTTCATTCCCTCTCTGGAAAATGCCGCCGAGCCGGCGCAGGGTGTCGTTCTTTGCGGGGCTTTTGCCCAAGGAGAGCGCGTACGCTGCCAGCGAGAGCACGTAGGGCGAAGTGTTTGGATTCAGCTGTCCCTCGATGAACCCTGTTGCTTTTGCGAAGGACTCTGTCAGGCCGGCCACCTGAACGCCTTCTCGATCGCATTCTTGCAGGGTGAGAAGGGTGTACGCGGTCAGTGGAACCGGTCCATTCACACCGCCCAGGAGGTCACCGTGGATTAGGTTGGATATGTNNGAGGTCACCGTGGATTAGGTTGAAAATGTCGTGGAAACTGCCATCTTGCTTCTGCTGGGTCAAAATGTAACCGAGCCCACTTGGGATGACCTTCTTGTCGATGGGGATATATTTCTGGGCTTTGCACAAAGTTCTCACCACGAATGCTGTCAGCCATAGACTGGAGTTGTAATTCGACCAAACCGCGAAGGACCCGTCAGATTTCCGGTAGTTGAGGTTCATCTGATAGCCACGGCGGATGTAGCGCAGCGCTATGCGTTCGTCCTCGGGCGATATGCGGCTGGCGGTCTTCAGATACTCGTAAGCGTACAGCGTTGGCGCCAGGTATATCATGATCTGTTCACCGCAGTTTTTTGGCATCCTTTTCAAAAGATCCCCCGGGTTCTTTACCGACGCCTCCAAAGCAGCTCCCATTTCATCACCAACGATGTTGATCTCACAGCGTTCCGAGCCAGGAATCACTTCCTTGGGGGATCGGTCAGGCCGCTTTATCACGACGAACTGCTTGCCCCCAGGCGCGAAACTCTCTCTGTAGCCGTGCGTGGGGCTTGCGGCATTCCGCGCTGGTCGTTTCTGTGTGTTTTGAGGGTCGAGAATGAGATTGAAGCTCTCATTCCTGCTGAAACCAGGTGGCTCTACCCTGAGCATCACTTTGACAGAGTCCCTAGCTGACCCATCGCTAAGGGCGGCCACGCGGATCTCCCTCTCGCCAGCAGCCAACGGCACCACGGGGAAGACCACCGTGCGGCCCTGTCCCGCCGGCACGGTCAGCGTGCGCACCGATGAAGGCTTGCCTTCTTTGACACCCGAGCACACGTCCTTCGTCCCGAGCATCGCCACCCTAACCTTGAGATCCTTGTGGTCGTAGTTGTACACCGTCGCCGGTATCTCGATCTGTTCCTCCTTGATCACGGAGTAAGGGAGGTTGACTTCGACGAAGAGCTTCTTGAAGACCGGAATCTGGAGAGGATCAAGTACACATATCCCGCCAGATGGGGCCACGCCCACAGCGCTGACCTCCCACGTGGTGATCGAGTCTGGGACGGTCACCTCAAGGCTAGCCTCTCCATGTTCCCGTAGGGTTAGTTGATTGAATATCCACGTCTCTCTAAAGTTTCGCCTTTCTTCACCCTCTAAATCTTTGAAGTTGCTCATGCCCAACACGTCGGCTTCCTCTGCAGATGACCTTCCGACAGCCTGGTTGTCTTCGACATGGATGCAGCACTTGCGAAAGGCTGCGACACATTCTATCGTGTAGAGGGCCACGCCGGCATCCAGATAGCTGAGCAGAGCCTCCTCGCGTTCCGAGCAACTGCGTAGGAGCTGGTCAGGCTGCATCCCCCGCGAGCAGCAGTCTCGGAGCGTCTCGTTTTCGTACTCTTTCACTATGTCCAGCTTGATTTCACGTTTTCCGCGCTGTCTCTCTACCACTGGTGCGGCGTAGCTGGCCTGAGATGTGAGCAGCACAACGCCCGCGTTCGCAAGGGTTTCCACCGCTTTCCTGCCAGCGTCCTGTCCGTTGCCCATGTCCTTCGCTTCCATGGACTGGAACACCTTCTTTCGCGTTAGGAGGTCCTTGCGTCGCAGAAGGTATACTGCCTGGTCCACGCCCAGCAAGCCAACTC

>AAUF12163

CTGAGGTGTGCGAGCAATGCGTAGCTAGTGCCCTCCACGTCCACGCCAGTGGCGTCGTCTCCCGTGCTCAATGTGTTCATCTCAGTGTCTTCCAGAAGATGGCTTTTCAAGACATCCATCGACTTCAGCCGGTCGGCTCCTGGGACGAAGCTCAAGGCATAAGCTACAAGCGCGGCCACGTAAGGGTGCTTGATGACGTCGAGACGCTCCCTGAGGTAAAACCTGGCTCGCGCTACGGTCACACTTGCNNNNNNNTGGGTGCCGATGCAGATGCGCATTCGTGAAACGTAAGCAGGACGAATGCTGTCATGGCTGTCGTGCCGTGCACGCTGCCCAACAGTTCCCGGGGAAGAATTGGGTCACGTTCATCGAAGGTGCCATTTGCTTGTTGTTTCGTGGTAAGCCACCAGAGGCCGCTCTCTATGACTTCAGGGCTGATGTCGATGAC

>AAUF12321

GCTCAGGGTAGAGCCACCTGGTGTCAGCAGGAATGAGAGCTTCAATCTCATTCTCGACCCTCAAAACACACAGAAACGGCCAGCGCGGAACGTCGCGAGCCCCACGTACAGCTACGAAGAAAAGTTCGGGCTTGGAGGCAAGCAACTCATCCAGATGAAGCGGCCTGGCCGATCCCCTAAGGAAGTGATTCCTGGCTCCGAACGCTGTGAGATCGACATCGTTGGTGATGACATGGGCGCTGCTTTGGAGGCGTCCGTAAAGAACCCGGGAACGCTTCTGAGGATGCCACATGGCTGCGGTGAACAGACCATGATCGGCCTGGCACCAACGCTGTACGCCTACGAGTATCTGAAGACCGCCAGCCGCATATCGCCCGTGGACGAGGACAGAGCGCTGGGCTTCATCAGCAGTGGCTACCAGCGGATCCTCAATTTCCGGAAACCTGACGGGTCCTTCGCGGTTTGGAATCATTACAGGTCCAGTCTATGGCTAACAGCATTCGTGGTGAGAACTTTGTGCGAAGCCCGGAAGTCTATCCTCATCGACGAGAAGGTCGTCACAAGTGGGCTCCGTTACATTTTGACCCAGCAGAAGCAGGACGGCAGTTTCCACGACATATCCAACCTAATCCACGG

>AAUF186

TCGTGGAGCGGCGATGGCCTGACTTACGGCTCGGCAGACACCGCTGGTTACGCAGTTCTCACTTTGAAGTTGCTCGACCGAAACCTGGGAGAAGCGCTGCCAATCGTCCGTTGGCTCATGCAGCAGACCTACGCCCGATACACTTTCTCCTATTCCGAGGTCTACACCGTCGCCATCGAAGCCCTGACCCAGTACTCGAGCGTGGCATTCTCGAAGAACACAAACTTGACTATGAACGTTGCTGTCGACAGTTCATCCCCCGAAACGGTCTCTTTCCCGATCTCTGAGCAGAACAAACTGCTATACCAGGAGCGTCTTTTGAACAGAAGCGATTCGTACAGCTTCAAGGCATCTCTTGCTGACGGCAGCGCAGGGTGCGCCGCTTTGCAGGTTAAGTACTACTACAACTCCAGGAACAGTCCGGTTCAGCGAGGAATACAAGTCAACGTGTCCACGACATCTGGACCAGATTGCAGCACCCTTGAACTGGAAATATGCACACGGTACACCGAAGGCTTTCTGCGCAGCTCCGCGATCGTCCAGATCACCCTGCTTTCTGGATACTCGGCAGATGACCAGTCTCTCAAAAANNNNNNNNNNNNNNNNNNNNNNNNNNNCTTCAGGATGCAGTGGTGGAGGTGTACGACTACTACCAGTACCAGTACAAGGCAACGGCTGGCTACAGAGTAGAGGGCAACTGCACGCCCGCTGAAGTCGCGCCACCCACGAGCCTCAACGAAGTGGACAGCGTGTTCTTCGTTTAGAGATCTTTTCTCACTTCCTTTCCATCCATCCGCACTTCTCTAAGCATCTGGACAAACGATCTCAAGTGCCTCCGAGTCATCTGCCTTGTGATACCTTGTGTTCGTCTGTATTTTAGCAATCGCTCCTCTCATCCTCAATAAATGTATCCTCTCAAGAAAAAAAAAA

>AAUF3660

TTTTTTTGTTTTTTTTTGAAAAAAATTAGTTTTATTCACTCTTTATGACACTATTTATAACGGTGAAGCCTCGCAGACGATGACGCGCTTTTCTCGCAAGTAGAAAATATATAAAGCTACTCTTCCACTCAGCTGGATAAGACGCGCTACGGGAAACAACATGGCGACGTCAAAAGTTCACGGCTCTGTTCACGCACACAAAAGCACACACTGCGCAGTGCTCCCTCAGTCGTCCGCACCCAATATCGAGGCTGTAAGATAAAACATCATGTGACCATTGTCTTGATTTCTTCGCGATGTGCGCTGTGGAAGCCATTAATCTTTTTTTTTCGTTCGCGACATGGCACGTATGTCCTTCTCAAGAAGTGTGACCGGTATAAAAAGCCTTTGGTGTTGAACACTACCGAGGGAGCGAGAAAGCATGATTCTGGTAAAAAGAAAATTGGCAACCAAAGGATAAATGCGCATTGAATGAATGGTATGTTGTGACTCTTATTGTCNNNNNNNNNNNNNNNNNNNNNNNNNNNNNNNTGATGGCCGATGTTCACTAGCAGGACGCCACAAGAGAGTAGGGCACGGAGTTGCTGTTTTCTTGCTCGTAATAGTCGTAGACTTTAACTGTGGCAGGTTTGGCATCTTCAATAGCGAACTCGCGGTGAACGCGAACGTCGAAGCACTTGTTTTCGGACGTTATTTCTTCAAAGTAAAAGTTAACCTGGTTCTTTTCAACTTCATGCCTCTTCAGCTTGACATCTTTCTCACGGTAGAGTCCAAAGATGTGATCCTCATCTGGAGTATAGCCCGATACCAGCTTGAGCTCCACCACTGCCATGTTGGATGGTTGCTCACCGTCGAACCTCAGACAGATTTTGAGCTTGTGGTCATTGCAGTCAGACGCCTCTTGAGATGGAGTGGCTGTGAGCTCGAAACCCTCGCTCTTCGGTGCAGTATGAACGTTGTACTTCAGAGTCGCCGATATAAGGGCACATCCAGGACCTGTTGCTTCTGAAGTGAGCTTGTTGGGCAAGCTGACAACCTTCTTCTCCTGTACGACCAGTTTGGTGTCCTCCTTAAGGTTATACGACTCACTGACGTCAGTACCGTCGACCTTAACTGAGATGTCAACGGGATCTTTGCTAACGTAAGTAGCGAACGCCGACAGAGCCTGGAGACCAAGAACTGTATCCTGTGTGGATGGGAAGCCTCCGCGGCTGTTTCTCTTGGTCGCCATCCAGCGGACAATGGGCTGCGCCTTGCTCAGGTTCTCTTGGGCGTTCAGCTTGAGGTAGGCGAGCACTGCATACGCCGCCGTTTCCACGTCGGCCGACGCTGATGGACCCTTCTTGCCCGCGTTGCTCCAGTAGGTCAGGGCACCTTTGTGAACAGCGATGGACTCCAATTTCTCGAGGTAATCTTTGGCAGACTCATGTCCCGCCAGGGCCGCGGCGTAGGCGGACAGGGCCAAGTTGTGGGCACTGGGGTCACGCTGGGCGCTGATGCAGCGGAGCGCAGACTCAACGACCTTCTCGTCCGCAAGGCCGCCCTCCAGAAGAGCGGTGAGCACGTACGCCGTCAGCGCTCCCGGGGCAGTGGAGTTAACCTTGCCCTTCAAGCCGGAGCTAAGAACAGTGCCGATGTTCTGGAAGCAGCCGTTGGTTTTCTGCTTCGTGATGATCCACTTTATGCTCTCGTTGAGGTTTCCGGCGTCAATAGGAATGTATTTCTCAGCCTGCTTGAAGGACTTGACTACAAACGCCGTGAGGAACATGCTGCCCGAGTTGTCGCGGTTTCCAAAGGCGCTGTACGAACCATCGTAGTGCTTGTACTTCTGCTGGCGCTGGTAGCCTGTCTTGAGATTCTGCACAGCCTTCCTTTCGATGTCTTCCTGGTTCTTGCCCGTAGCCTTAAGGTAGTCCAGGACATAGACGTTTGGCGTAAACTTTACCATGTTCTGCTCACCACATCCCGTTGGAACTTGCACCAGTGAGTCGAGGTTCTTGATTGCTGGGCCCATAATATCTCCTGTGACTTGAACGTAGGCACGGGCTGATCCTTCCACCAAGTCTTCAGGAAGCACCAAGTCAAATTCGTTCTTTGCGCCGCCTTCTCCAGCGTCTTTAGGACAAACGAAGACACTCTGCGTCTCTTCCTTAGGGAAGCCTTCCGCCTCGATGATGAGCGGTCGTGTGACTGCGTCCCTGGCCACCACCTTCTCAGTTGGCTGCTCTCCACATGCGGCATCGCTCTGCGATCCGGCCGCCGACACGGTCAGGTTCACCTCTCCGATAGTTTGTGGCCGGATCTGGAACTTGTGCACTTGGCTCTTGCTGCCGCACACGCAAAGCGTCGTCTCGGACTCGCCCTCAATGTGGAAGTCCGCGGATTCAGCCAGCTTTAGGTCGACCGGCAGGCACTTTTCGAGGTAGTTGAACACGGACACCTTGACGGGCACCAGTTCTCCGCGCACCACAGAGTAAGGCAGGTTGAACGAGGCGAAGAACGGCTGGAAGGCCTTGATCTTTGCCGGATCTGAGATGCCGATTCCGTCCTCTGAGTTGATGCACACAGTGCTTCCCACCCACTCGGTGATGGTGTGCGGAATCTTTTCTTTGAAGTTCAGTTCGCCGTGCTCATCCAGCTCCTTCAGGTCCCACAGCCACGTCTCGGGGAAGTAGGTGCGCACTTCAACGGCCGACTTGGCCGGGATGTTGGCCGATGGTGCGCTCTGCGCCACAGAGTCCAGGGCGACGGGCACGCCGGGCAGGCCACCTACAATGCGGTTGGAAAAGGCCACCCCAGCCGGAGGTGGAGGACCCGACAGTGCGAGTGGTCTCGCTAGAGGGANNNNNNNNNNNNNNNNNNNNNNNNNNTCGCTGGCGCCGCGAGAGCGTAGGGTGGTCTGTCGTAGATGTTCTTCCGGCATGGCCTGGTTTCAAGCGTCAAGTCTGACATGACCACCACCCCTGATTCGTCGAAGGCGGTGATCGAATCGACGTATTCAACATTTGAAGATCTCGGTCCATTCCAGATGTGGCGCTTGAACTGTTGCGGATTCTTCGCCAACTGCTTGCGACAGTAGTCGTACGACGCCTGCTTCGGCCACGTGTAGCGGGTGATATCCAGACGCTTCAAAATGTCATAAACTTTGTCCTTTGTCAGTTGGTTATCCTGCTTCAGGAGGTGAACACTCTTGTCTACGACGCCAACACCACAGAAGGACCGAGGAGAACCATTCACATGGATGGCAGCAGATGTTGCAGGCTGTACAGTTTCAGATCCGAATCTCATGGTTACGTTGTTCTGGAGGCACTTCTCCACTTCGAACTGCTCGGAGTCAGCGATGACCTCGCCGTCGGGGCGGACGTAGAAAGCGAGCACTTTGACGCGTGGCACGTGGCTGAAATCTGGCTCGAGCTCGAACTCAAAGCTCCCTGTCGACACGCTGCCCTCGGCCATGTTGGTCGGAAGCGTCTCGTTCAGGTCCTCCTCCACCAGGAAGCTGTCGTCAACAGAGAGCGCCTCTTCCGGCTTGAAGGTCACATCCAGAACCTTGTCCTTGAGGATCTTGCCGCGAGCTATCACCTGTAAATGGAACTGCTTCTCTGAGTCGGGCTGACCCGTGTAGCGGAGGCGCACGGGGTGCTTGCCCGAGCAGCGCAAGGGTCCCTTGGACGGCTCGATCTGGATGAAGTTGTTGCTGGCCGAGTACCAAGCCTGGAGGTACAGAGTGCTCTTGGGCTGGTTGATCTTCACGCCGTAGGTGTCGTACTTCACAGTCTCGTAGTTCATCGCCACCGCCTCGACGCTGATGGTGACGACAGTTGTCTTCAGGGGAGGGATGGTGAACTTGATGATGCCTGACTCGTCCGAAGTGTAGTTCTTGCAGCTGAGACGGCGGTCGGTGCGCCACCACCGTGGCTTGATCTCCTCAGCCTGTGACAGCAGACAAAGTTGCACCAGCTCGCCGCCCACAGGAACGCCGTCAGGCTTCTTCACCAGGAGCCTGCCGTAGAAAGGCATGGTGGGCTTGAAGTAGTTCTTTCCGCGGTCTCCTTCCAAAAAGTTGAGCTCAAGCGGGTTGAAGCTGCGGCTGATGTAGTTCGTCTTGTTCATCGTGATGCCCGTGCCGGTCTCGTTCACTTGTGCGACGAGGTAGATGCGCTTGTAGATCTCGTAGTTCTCGTTGAAGCGCAGCAGGCTCGTGTTCACCGTCATGTCGAAGCAGCCGTTGATGGGTCCTGTGTGGTTGATCTTTGGGTAGTCGTCCTTCTCCCAACTGTAACGCTCGTATGTCACGTTCACCGTCAGGGTGCCGTCCACTGGCTGTCCAAAAGTATAGTGGGCACAAATCTTCCAGGTGATCTCCTTTGCATCAGCCAAGACGTAGGATGGAGGCTTGATGGTCACCTCAAATTTGGGAAGTACGTATTCGTTCACCTCAAAATGCTGCCTCACGGTTTGCGTGGGAAGTTCGACGACTATCTGCCACAGACCCAACTCAGGCTCTTCTGTAAGCTGAAAGTCTCTCTGGACAATGCCTTTTTCAAAAGACACGTCGTTCCACTGAGCTATTCTTACATCGCTGGGACTTGTCACGTAAATAGTTGCCTTCACATCGGTGACTGGTTTCAGCTCATTGTTGATAGGGAGGACGCGGAATTGAACCCTCTGTCCAGGCTTATAAAGTGCCTTATCGGACTGAACCAGGATCGTGTTCTTGGACTTCTGGAAGTCGATCTCTTTCCGATCGCCGAAGACATAGTCTCCAAAGGTTCCGTTCACTTCGATCTTGGCTTGGCTGTCGAGGTGCTCGGGCACTCGGAACGGCAGAAAGGTGCTTTCACCGTTCTTGATGTCGTACTCTTGTTCTGCCAAGACGATGCTGTCGTTGTTGTACTTGAGCAGGCGGACGATGACCTTGCCATCCTCCTTGACGTCTGTGAGCGTTAGCCGAAACTGTTCGTCCGTTTCGCTGCGCAGGATCTTCGGCGCCGTGAAGATGTACCCACTTTCAGCAAGGCGCGATGCCAAGGCACCAAGGACTAAAATGAAGGTGCCCCAATGCATTCTAGCTCTGGCCACCGGCACCTCTCGTCACCGTCTGTGCTAGTTTTCTTGCGTCTAAGCCAGCCTTAGACACCTTTCCGTAGACTGTACAGCACAGCTTTCTCTGCTGCGCGCGCTAAGGCCGGTTCCGGGAGCTTTTTCTTGCTACCCTTCTTGTCTCGCTCCTGGCTTGACGTCGCGTGGCGAAGCAAAACACAACACACGCGCTCTAAGGCGGCGGCGTTGGTGCGCAGAGAGTGCCGCGAGACGAGGCCGACAAGACCCTCCGACAGCCCAAGCCCGAACTGATCCGGCGGCTGCTCG

>AAUF15559

TCTTTCAAAAGACAGTTCTGGCTCTTCTATGTTGTTCCTTATAGTGTTCTTTTTATAAAAACTGGAGTGCATTGAGGTTCAACTTGGAGCACATCCCAGCAACTTGCAAGTTCTGTCAGTATGAAAGTTTTCTGCACGTTTATGTCCTTTTGCCGGTCAGGTCTGAGGAGCAGGCACTGTAACAGGCATGTTTGATTCAGGGCCATCAGGGAAGTCCAAGTCTTGTTCGATATTTCGGAAGTTTCCAAGATGAGTCCTGGCTGAACGAATATCTTCTTGCATTATTTCTGACTGCAGGGGCATTTGTGAGTCACTAAGGTCTTGTTGATCCCTCTCACAACTTGGAATTGTGTAGTTCCTTAGTACAATGACACCAGGATCATAGTAGTCCTGCAATACAACAGCTGCAGGAGCACTGTCTTGCACTTCAAACTCCTGGGTCAACATTAAGGTGATGCACATTCTTCGATTACTTAGCTCATCAAAGTACAAGTTTATTTTGTCAGGCTCTTCCTCAGTCTTCCTAACTTCCAGGCCATCAACAACCTTGCTCAAACGGAACCCAGTTTGCAGATGGACTTGTATGATCACCATTCCAGATATGCCCCAGGAGAGGAGGTAACTAGCACAAAGCTCTATCTTTGGCTTACAGTCCGCATGGGATGCCAGTACCATGAGATCAAACTTGTTGGTATCAGCCACTGCAGGAATGTTGAACTTTGCAGAGGCCTGCACAAAGGCACACCCCTTCCCTGTGACGTTGAGGTTCACCATCGCCGGCACTGTAGGGATGACCACCTCCTGAAGCAGCATTGCATTGCTTGGTTCCACAAAAAGCTCTTTGGCCTCTGTGTGTTGGGACGACACTCTCATTGACAAGTGTGTGCTTTGCAGAACAGTTGCTTTGGCAAATGCAGCCAGGGCTTGCAAGGCTACAATAGTGTCCTGTGTAGACGCGAATCCTCCATGGTCACTCCTCTGCTTGACCAACCACTGGATTGCGGGCAAGGCTTTAGGTACACCTGCTACCTTGAGCAGTGTCATGCAGGAGAGAATTGCATAGCCGCTTGTCTCTACAGCCACTCCCAAAGACACGTTGTGGCTCCAATGTAACGTGCCATCAACGTCTGATTCAGCCCTGCTCCAAAGCAAGTCAAAAGTTTGCTGTGCATCATGATGGTTTGAAAGAGCCAAGGCATAAGCCTGAAGTGCCAGTGAGTAGCTGTCCTGCTCGTGAGGAGTCTCTTCTATGAGGCATTGGATGGCTGCATTTAGTACAGATCTGGAGAGAGTCACATTTGCTTCAAGCAGTGCGATTAACACATAGGATGTTAGTGGTGAGAGTGATGTCCCCTGTACACCGCCCTTGAGCTGGGTGTTCAGCACACGGCCTACTGATGGGAAACAACCAGTATCATATTGATGCCCGAGTAGCCACTGAGTGCTTAGGCTCAGCTCAGCATCATCAAAGAAAACATATTGGCGGGCTTGTCCAAAGGTCCGCACCACAAATGCTGCCAGCCACAGGCTAGGTTCAGGATCTGCAGTGCCAAAGGCACTATAGCCATTGTTCTGATGCCTGTAGTTGAGCTGCTGTTGGTAACCTGTGATGATGCTTCTCCTGAGCTTGGCCACCAAGGGATGATTGGATTGTCCCACAGAGAAGAGGTAGTCCAGCACCACCACACGGGTGGCCAACATAGCCAAGTTCTGTTCGCCACAGCCAGTTGGCAGTTGAACGAGGGACTCCAGGCCTGATGCTACAACTGGGCTGAGTATGTCACCTGAAACCATAAACAGTCCTCTAGAGGAGCCGGGAATGGCATTGCTGGGAAGCTTTAGTACAAAGCTGCGAGTTGCTGTGCCATTTTCTGTGGTGTTAATGCATATGTATTCAGAGACTGACTTTGTCCTGGGTATCCCCTCTGGCAAAACATGCAGGCTTTTGAGCACAGCATCTCGAGCTCTTCTTGTTTCGAGAACACTGCCAGGATCACAAATGGAATCATCTCGAGGAAGGCTCAGGGCATGAGCCGTGATGTTGATTTGGCCCATACACTCTGCCCTTGCATAAAACCTCAATGAAGCCGTGTCATCACTGCACAGGCAGAAGCGTTTTTGCACCCTGCCAGTCACCTTAAAACAGTCGGCATCAGCAGTCACACCAAACTTGACAGGTATGCACGACTTCAGGTAACTAAACAGCGAAGCAGTGACAGGAATAATCTCTCCACGTCTGGTGATGTTGGGAAGCGACAGCTGAATGAAGAAGGGCTGCATAGCATCCACAGAATCTGTGGCGACACCCAGGCCGTGAGATGTGTGTACGCACACAGCGCCTGCTCTCCACTTAGTCACAGAATGGGG

>AAUF16994

TTCTAGAAGGGCAATCAAAACATAAGCTGTCAGTGCAGGCCCACTTCCAGCGCCACTCTGCATTGGCTTGTAAATGATCTCTCCAGGTTCATTGAATGAGCCATCGCTCTGCTGACGTGCTACCAGCCATGCAGTAGCATTTTCAAGTACTTTGGGGTCAATGACAGTGTACGGAGCAGCTTGGGCAAAGCTCTTGAGCACAAATGCTGTCAACCATGTACTGCCACTCTTGTCTGTATTGCCAAAAGCACTAAATGAATTATCATCGCGTTTGTATGTCAGTTCTCGCTGATAGCCATCTTCAAGATTCCGTATAGCTTTGTTTTGGACAGGTGGCGAGAGGCGGTTGGCCCGTTTCAGGTAGTCAAGAACAACAACGTTAGGTACAAAATCCAGCATGTTTTGCTCTCCGCACCCATGTGGCATTACTAGCAGTTGGTCCAAATTGTTCACACTGGGGCCGAGAAGGTCACCAATTGCAGAAATACTAATTCGTTCCGATCCGGGAACAGCATTCTTTGGAATGGGAATGCTGATGTTGGCTATTGTGGGAGGAGCTGACGGATTCCGTCTATCAATTAGGATGGCCTTGTTGAAGTATTGCTTCGAGCCTTCAGGCTTGACAAGAAGCTTCTTGAGTATGGCATCACCAGCCAGACTTGAAGTAGCCGACACACGAATGTCTATGTAACCCAGCTTGAGAGGAGTAATAAGAAATGACACAGGGATGCCATCTTGAGCTGGCACCATCACCTTCTTTGTGCGTCTCTCCTTGTTTTCTTCTCCCGTGACATCATTCGACAGAGAGCTAAAGATGAATTCCCCGCGGTTATTCTCCAGTGTCACTTCCGCCTCAACTGGCTTCTTGTTGTAGTTGAAAACAACACACTGGATTGCCACAGACTCTCCCCGAAGAACAGAATACGGCAGACTAGCAGTCACAAAAAATGGCCGGAAGACTGTCACTTTTGCTTGCGAGGGAGCAATTCCAAGACCAGTCAGAGAATCCAGAGCAAAAGCACTGATCACCCAAGATGTAATGGTGTCAGGGATTGTGCTTGCGATAACAGCCCTACCATCAGGACCTGTTAAGGTGCTATTCCACAGCCAAGTCTCAGGAAAGAAGCGTCGCAGTCGGTGCTTCCCCTCGTTACGACCTGCCGCTCCCCCAGAGTACGCTGAGTCGTCAAGCTCAAATTCACCACCAAATCCAGAACGATAGTAGATTGCATCATCAAACTCAT

>AAUF56853

GCCTTTGGTATCCCAAGTTCAGATATTTGAATGCCTGTCTTTCCAAATGCATGTCACGTTGGTTTGTCTGTCGTAGATAGTGCACCGTGAACAAATTTGCCCCAAAGCTGAACATGTTTTGTTCCCCACAGTAAAACGGCTTTGACAACAGGCTGCTGGAATTGACAGGCATAGTTGGGAATGCTGGACCAAACACATCACCAACCACAGATAAAGTGGCCTTGTTACTGCCGAACACGTACAAGCGATCTTGTCGGAAAGGCAAGATGGGTGACTCAGTAATGTTTGTGTCGAGATACTTT

>AAUF1099

AATCGCTACTATCCCAGTTTCGGCGGTTTCGGAGCTGTGAACAAAGTGGCATTTGCTCCGAGTGCCTCCCCCGGATTTGCTGGACCGGCACCCATCGCAGAGTCTGCGCAAGCCGATTTTTCGCCCGTCGTCCAGGGGTCAAACTCCGTCCGCACACTCTTCCCTGAAACATGGCTCTGGCAGATCAAGAGAGTGAGCCCTGACGGTTCCTTGGTGTACTCCGAGACGCTCCCGGACACAATCACCACATGGCAGGGCACTGCCGTATGCCTGCACCCGCGAAACGGCCTCGGCGTCTCCCAAGTGGCAAACGTCACCGGATTCCAGCCATTATTCGTATCGCTCACGTTGCCAGCGTACATGCAGCGCAACGAAGTGGCCACGGTCATTCTCACGGCATTCAACTACGGCGAAGAGTGCGTCGCGGTCCGGGTCAGTCTGAGGTCTCTGGAGAATCTCGAGGTGGTCGGTGGTCCCAACAGCACCGACGCACTGCTCTGCCCCAACTCCAGCACATCCGCGTCATTCCCGTTTGACGTGCGCGCTACCACGCTTGAGGAATCCCGGCTGGAAGCCCGTGTCCAGACGAGACCCGAGGCCCANNNNNNNNNNNNNNNNNNNNNNNGACAGCGCTCCGGACACCCTGCAAGTCAACCTTCCGACACCTGTCGCTCTTGTTGAAGGCTCTCAGCAGGTTGTACTAGTTGGCACAGGAGACATCCTCGCTCTCAGCTTGAACGACCTATCGGTTCCCACCATCACCTATTCCAACGCCGAGGGAACACTGGCTGTGCTTGCCTCCAGCGTCTACCTCCACAAGTACCTGGAGCAGACGGGGACTCTGACTGACACGGTGAACTCTGGCCTCCGATCCCGCATTCGCCAAGCGTCCCAGGCTCAAAACAGCTTCCGGTCCTCCGACGGCTCGTACGCTCAGTTTGGCTCTAGCGAGTTCCCGAGAAGCGTGTTCCTCACCGCCTTCGCCGTGAAAGCCCTGAGCGCCGCCAAGGAGTACCTGGGCACCAGCGTCGA

>AAUF4190

GAGACGACTTCGATGGGCCTTTCATCGGAGTCACGGTGCACGTAGTACACAAGGAGTTGCACCTTGGGGCTGGCTTCCGCGGGTAGCCTTGACTGGAACTCGAAGCTGCCCACCGACCGCACGGTCTCTGGATCTACCACCTGGGGCTCGGAGCTTCCAGCTGGAGGGTCGCCGTTTTTGATCTCGTTGCCGTTGGACTGGTCCATGGTGAGCTCGGCTTCGGTGAAGTGCTTTGTGAAATTCTGCGTCTCGAGGATGTGGCCGCCCGAAGTGAGCGTGAGGAAGATGTGGTAGTCAACGTTGGCGTCGGCAGTCATCACGATGGGGCGCGTGAACTGGGCCTGGCACGTGTACACACTTCCGTCTCCTCGGTCGATGCGGATAGAGCTGGCCGTGGGCGAGTAGAAGGGGACAAGGCTCAGCTGCGCCGAGGGCTTCTCCAGGGTGGGGCCATTCTTGACTACGATGCGCGGGAAATTGACAGCGATGGCCTGCACAATGACACCAGTGACCCTCTGATTGAAAGGTGGCAGGCTGAAGCCAACGAGACCGTCAGCATCGGAGGTGTAGTTTCGGCAGGCGAGCACGTTGTCCGTCTCCCATGAGCGCCACTTGAACGTGTTGCGCTCCACGAACATGCAGAGCTGCACTAGCTCATTCGGGCGCGCAGTTCCATCCAACTCTTGCACGTAAAGCTGTCCCTTGTAGAGGAGGTTAAGCTTGAAGGTGTTGTCGGTGCTAGTGTATTGGTTGTTGGAATTGTACTTCTGTCCGAAGGTCAGCCTCAGTCGTGTGGTACCACGGCTGTGCAGCTCGGTGGCAGTTTGAGAGACACCTGTGCCGTCTTCGATGACCTTGGCAGTGACGTTGATTGAAGGGTATGTGGGGTACGTGTAGTTCACGGTGTTGAACAGCGACACGTTGAAGACGTAGTCGTAGCAGCCGTTGATCTCAGCGACGCGAAGGATGACTGGCTTCTGGCGCACTGGGTAGTAGTAGTTGAAGGGAGATGTGTAGATTTGCACAGTGCCCTTGACATCTTCNNNNNNNNNNNNNNNNNNNNNNNNNNNNNNNNNNNNNNNNNNNNNNNNNNNNNNNNNNNNNNNNNNNNNNNNNNNNNNNNNNNNNNNNNNNNNNNNNNNNNNNNNNNNNNNGAGTTTGTGCCTGCCGACACGCTGATCGTCCACGTGCCTTCGTCGGTAATGGCAAGGAGGTCGTAGGAGCCTTGAAGGAGGCCGTCGGTGAAGTTGACGTACTGCTGCTGGAACAAGAGCTGGCCATACGGGTTCGTGATGGTAATGTTGGCCAGCTGATTCGTCACGGGGAGGAGGTTGTTGTCAAGCAGCAGTATTCGGTAGTTCACTGTGCTTCCTTGACGGTACATGGGCTTATCCGTCTGTACAACGACGTTGACATTGGTAACGTATTCCAGTTGAACAGACGATCTGTTTTGGAATTGGTCGCTGCCGAATTGTCCGGTCACATCGAGGTACAGGTTGTAGAAGACGCTGGGGCTCAGGTCGGGAACGTGGAACAGCAGCTCGGCGTCGATGCCATCTGCGCCGATGTCATACGTCCGACTTGCCAGCACTGTGCTGTTCTCGTTGATTCCCACCCGAGTGCCCCACAGATCTACTTTCAGTTGTCCCGCCGACGCCGGGTTGGTCACCACAGCGGTGATCTTGATGTCGGTATTGGGAGTCAAGAATCCGGGTGCAGTCACAAGGTACTCGATAGAGGCGGCGGTGGGCGTTGTTCCCAGCGCGACCACAGCTGCCAGCAGTAGCAGCTGGTGCGTCCAACCAGACGCCATGGTGGGGACTTATGGCACCTTTTTTTGCCGGGCGTGTGATTGACTGGGGCCAAACCACGAAGCGCAAACAGACAGGGAGTTGTTGGCTGTACCGGGTTCCGACCTCGCCGTCAGGTGAATCCGCGCGTGCTCGAGTACGCACGCGCCGTGCTCGTCGCGACTGGACAGTTCGCGACGAGCACGGCGCGTGCGTACTCGAGCACGCGCGGATTCACCTGACGGCGAGGTCGGAACCCGGTACAG

>AAUF23785

AACGGACGGACATGGGGGCGGCAGTCCTCTTCCTCTTGGCGTGTGTGCCTTTAGCTGCGTCTCAAAGCGGCGGCGTGTACACCATCGTGGCCCCGCAGAAGCTGCGACCTAACCTCAAGTACCACGTGAGTGCGTCGCTGAGCCAGAGCCGGTCGCCCGTCGACCTGCAGGTCACCGTTTCCGGTCGCGCAGACAGCGGAAACTTCAACACCATCACCAAGAATGCCCTGCTTCATGACCGTGACACACAAGTGTTGAGCTTCGAGATTGGCGACTGGGGCCCTGGGAAGTACAAAATTACAGCGCAGGGCTCAGGCGGTCTCAACTTCCGCAATGAAACTGACCTCACCTACGAACACAAGAGCTATTCTGTTTTCACGCAGACTGACAAAGCTGTCTACAAGCCTGGCCAGAAAGTTCTGTTTCGGGTAATTGTGGTGGACCCATATCTCCTGCCCACTGTGACTGGCGCCATGGATGTGTATATCGCTGATGCCAAGGGAAACAGAATCCATCAATGGGATCGAGTGTTCACGCAGAGAGGCATCTATTCTTCTGAGCTCCAACTCTCTGATCAACCTGTTCTTGGAGACTGGACTATCAATGTGGATGTGCTGGGTCAGAAGTTTAGGAAAAGTTTCACAGTTGCCGAATATGTGCTGCCCACATTCGAGGTTAAGGTGTCACTTCCTTCTTATGCCACATACAACAAGTCTGAAGTCGTTGCCACAGTTTCAGCTGCCTACACGTATGGCAAGCCAGTGAAAGGTACTGTTACACTGACAGTCACTCCCCGCACTCGATACCACCAGTTGAACCCAAGGCCTTATGAGCAGTACCAGACGAAGACAACGATTGATGGTAGCGTGGACATACCTGTGGCAATTGTACGAGACCTGTCACTTAAGACTGACTTCTTTTCACGGGAGATCGAGTTCTTTGCCCTGGTAACCGAAGAGCTGACTGGCCGGAAGTACAATTCCACCAGTTATATCACAGTTCATGACAAAGAAATCAAAGTGGAGCTTGTGAAGACATCTGAGACTTTCAAGCCAGGACTGAAGTACACTTGCTTTCTCAAGGTGGCTTACCAGGATGACAGCCCTGTGAATGATGCAGTGAACCAGCTGGTGCTGCACTATGGTTTCAATTTCAACGAAGAGCTGTGGCAGAGTAGCCAGCACTGGGTGCCGCAGAATGGAGTAATTCGGCTGGAGTTTTATCCACCCAATGAGAACAGCACGGTAATCCTGGGTTTCCGGGCCGAGTTCAGGGGCCAGACTTACTACTTGGATGGAATCTACCCAGCCAAGTCACCGAGTCGCAGCTTCCTCCAAGCTTTCATGGTCACTGAGGAGCCCAAGGTGGGCGACCTTATTGAGTTTGAGGTCAATGCCACAGAGC

>AAUF50228

CGGCCACTCTACAAGCAAGGCCAGACGGTCCATTTTCGTGCACTGCCAGTCACAACAGAGCTCAAGGCATTCTCAGATGCCGTCGACGTTTACATGCTGACCCCAAACCGGACTGTTGTTCGGCGTTGGCTCTCCAGGCGAACAAATCTGGGTGCCGTGAGCCTGGAGTACCCGTTGTCTCCGCAACCTGATTACGGAAAGTGGACGATTCAAGTCATTGCTCAGGGCCAAGTCGAAGAAAATTCGTTCGTTGTAGAAGAGTACTACCAAACAAGATTTGAAGTCAATGTCACACTGCCTACGTTCTTCATGGCGACGCAAGAGTACATTTACGGCACTGTCG

>AAUF3470

GTTGAGATTGAAGCCACTGGATTTGGAGTAGCAGTGCTACAGGTCTCATGGTCATTCAACTTGGCCGTGTCTTCAGAATCTCCTTCATTTTTCTTGAATCCTTTGCTGGACAAGACTTCTACAGAAAACTATCTTCAGCTCAGTGTCTGCACGCACTACCGAGGGGAAGGAGACGCCAGCAACATGGCTGTCATGGAAGTGGCCCTGCCTTCGGGCTACCTCTTTGACTTTGACACCTTGAGCAGCATTCATCGGACCAAGGAAGTGCGCCGGGTTGAGAGCCAAGATGCCGACACAAATGTTGTCATCTACTTTGACAGGATTGGAAGACAGGAGCTGTGCGTCACTGTACCAGCCCATAGGGAGCACAAGGTTGCCAACCAAAAACCAGTCCCAGTCAAGGTTTACGACTACTACAACTTGGCTCGCAGTGCTAGAATGTTCTACAGCCCTCACAAGGCAACATTATGCGACATCTGTGAGGGTGTTGAGTGTGGCAATGACTGCAATGCTGTGAAGCAGACAAAGTCAGATGCTACGCAGCTGGAAAGAGAATCTGAGCCGGACGGTGCTGCTGGTATCCATGCATCCCATCTTTCTGTAGCAGTGCTTGCAATTACTGTGGCACTGTTTTGGAGATAGTGGCATAGTGAGGCTGACTACCAAGCAGGACTGCTTGGTATACGTAGCGCTGCATTTCATGTTAATGCAGTTCTGTGACTTGTTCTCATCACAAAGGCGTGCTCAGATGTGTTAAGCTTTATTTTGGCATTCATACTGCGGCATCTGTGGTTTTGCTTGTGTACTACTCTGTGTCAACTATGTAGTGCATTTTTGCACAGTGTGAAGCTCGTGACTGTTTTAGTGTTTCTTGTGACCCTGGCAGTGATAAGTTGCTGCTTTATGTTTGTTGCTATCATATCTCTTTTTTCTATGCTTGCACAGACAAGTACCCTCGTCATTTGAGGTACAGTCAATATGACAGGGAACACATTACTTGCATGCAGCCAGCGGTTTCCACCAAGGTGCAAATGAGAACCTTGCATTTTATTTTCTGTGGAAGTTTTCCTGTTGACAAGTGGTCCTTATATTTCTTTCTAGTTTGATACAGGTTTAAAGAAAAAAAGTTGAGATTTGATTGTGAATGAAGTGTTTTCTTTTGTATTGCATGTACCTGTACATTGTAAAATTACTCTGGGGTTAACTGGTCATTGGAGATGCCTTGTAGATGTTGACATTGCTAGATGCTGTTATTTGTGTGGGTGAATTGTGAAAACATTAATCTACTCAACAAAGCCCTCCATTAGAGTCCATACTGTTTGCATCACATCGTGGCTCATGTTCGATCAGCATCAGTGCACCAGTAGGTTATGCTGTCAGAGTGCAGGATGCAAGGAGTTTTTTCATATTTTCTGCATTTTGGCCTTTAAGCAGCATGTTTCAAATTCATTTCATGCTTAATCATAACTCATCCTTTTACAAATTCATGTGTTACATTTCAAACATTACTTTTTTTTTTGTAATCGGTGGCTTTGCTTTGTTTAGTAACATTTGCTACAAGGCTGAGATAGTAATGTTCGAATATTGTCATGCAGCACATGTGATATGGAACATGTATTGCATCACTGTAGCATTTACTAGAACTGTTAGATGTACACTAACCTATGTATATGTATCAACGCATACCAATAGCCTTCTTTTTCTCTTTGTGTAGCTGGTTTTGTTTCATGCTCCATTTTTCCATGAGAATTGCATGTTGAGGTGTTCTTCATATAGGAACAAATTTTGTCAGCACTTGCTTGATTAATGTAGTAGTAACAAGAAGCTGCCATCATTTGCAAGCATGCTTCTTGAAAGGGCAAGCTCAAGAGTCAAATGGATCGATGTCGTCATTGCAGAGAGAAATTGTCACTGGTGTTCATTGAGTTTTGCCATTTTCATACAGAAGGCAACACTCTTGCCCTCCTAGTTGGCTGAAGTTGCTTTTTTGGGGGGGCTTGGCTTGCAATGCACGAGTTCAACATGAACCAGCAAAGTGCCGATGCCTAATTAGTTCTTCAAATTAAACAAACCACAAAATGTGATCATCTAAAATTTGATCAGCTGCAAAGAATGGCGAATTTTTGAAAAATGCTGCAGCAGATATATAGGGTGCTATACTAGGAGCCACATTTTTGTTGGGCTACAGGGAAACCCTTTCACTTGGACACCTTTTCATGCGGACTAAGTGGACAGGAGCATAGGATTTTTCTGCCTCATACTTTTTATATTTGAAAGTGCATCACGTCTTACCTAAATTGTTCTTGTAGAACAGCTGCCAGCATGGTGTTTTATCGTTTATAGGGTGACATGTAGCAATGTAGGTAGGGACATATCAATGGTTACAAGACAGTGATGTAAGTAATGTTAATAATTGCAACATGGTAATTCAGTATCAATGCTCAAGTGTTGTGCCAATGGCAAATAATTTATTACATACACAAGAAAAAAGATAATCTTTGTACTATAGTGACCAGTCAGTAGATCAAGGATTGTGAACATGTTCATGACACACAAAAGAAAGGTAATCTGAAAAGTTTTCAGTAGTGAAGCAATGTAGAAAGAAACTATGTTGAAGTTATTTTCCTGACATAATGCATATCTTGTGGAATTTTGTGTTGATTGTAGGGCAAGTGTAGCTTTCAAGGCTTAAACTGTGTCCTTGCATCTCCTTTTATCCATCTTTTTAACCCTCATCATCTTTCATGTAATTTGTATGCCATTTGGTGCATGTAATAATCACAAAAATATGCCACCATCTGTCCAAGAGGTAAAAAGAACTCATTTATGTGTACATTCAGATGAACAAAAAAATAGGGCACAGTACAGGGCTCTGTAGCCCCACACTCATCTCATGTTTTGCATTGTATATGCTTTGTACTTCCATCATGCAGTAATGCTGAGAGGAACGATAAAGCCTAAAAAATTGGCAGCTTTCTTAAGAGGTAGTTTTAGGGGTCCTCTGAAGCTGCTTTATATATGTTTGATACATTGTCACATGAGTATGGAAATACATCATGCACCTGTTGCATTGGAGCTCTTGTAATTGATTGGTATAGGTGCCACTTCACTAGTGCCTGAAATTTTTGCAAGAGGCCTTTGGAAGCCAGAAATTGCTCAGGGCTGACCATCTTGGGTCTAGTTATAAGCTGGAGCTGTTGGTGTGCTACAAGCATGTGGATAATAGTAGTTTGGGCATTGTAGATGGTTTTTGCTACTCCTTGTACGTTTTTATGAATTATTGTTTTAGTCATTAACCATATACTTTATTAAAGGCATGTAAATATAGGGCAGCTCCCTTGTGTGCTCCAGCTTTTTGAAGGCATGTCACTTCTTCATAGAGAAGAAACTCTTGTGCAGGTTTTTGCACAATTTTAACTCTGGCACACCTGGTTTAGCGTGTGAAGGAGATTTTTCTTAATAATTTTTAAAATAAAGCAGTGGCAAGGCAGGCTAATATGTGTATTTGTCCTGCTGCTTCTGCTGTATCTCTTATAAAAACTGTGCTGTGCTGTCATTTGTCTAAGCAGGTTGCAGCTAGCCAAAATGCTTATTTTAATTGGGATGCTTAAAGGTTCAGTGCTGATTTAGACATGTAGAATGGAACAGGTTCTTAATGGGGGAAGCTGGTCTT

>AAUF6161

TTTTTTTTGTGATCTAAAAATAATGAAATATTTATTGTCTTTATTTTTACAGTGACTAAACATTTCAGGACCAAAGTGCGAGCAAGGAAAGACCACACATTCATAATTTTATTTCCATCCTTTAGCTGCACTTCACTGACTTAGTGACCACGAGCCGGCACTCTTCGCTGCATAGCTTAGGCAGAAATTGTGCGATCCCGGGAGACGCCACATGATTAAGTAGTTTTGCCTCTTTGATGTTGCTATGAATGTGAAGTCTATGACCTGTGAGAAGACAAACCTTTCGCGATAAGCTAGAGGCATATTTTGCGCTGCACACACCCACGGCCGAGATGACAAGTTTATACCGCAGGCAGATTTTGTCCCCTATATGACTATAGGATAGGTGCTCTGAAGTGGATCAGGGCCTATCAGAAGCTGATCAAATTGTTTTTCTGTGAAAACAGAACGTGTTATCGGCAGGAGATTGATAGAGGCTACCACTGAATGTATGCAACTCATTTAAGTTGTTGGAGACTTTCTCTACCAAAGTGCAAATGTAAAATTCGCTGCATGGAAGAATGGGGGNNNNNNNNNNNNNNNNNTCGCTGCATGGAAGAATGGGGGTCCTGAGAGAACAGCCTCTAGATTGTAGGTTTGCTTAGCTTTCTTTTTTCATCCGACGCAGTATATTACTGTTTTAACGCCGCTGCTATCGAGAGTGATGCGTGTTCAACAAACCTGGAGGAAAGCAAATTGAGAGCCTCTAATGAGTATTCAATATGGATAATTTAAGTAAGGGCATCATATACAAAAAAGTCGTCCGCACGGATAGTTTGCTCCTACAAAAAATACAATAAACGGCACTTCTGAAATGAAAACAATAGTAAACTGACATGCATCTGAATGCCCAAAGTTCAGTGGCGATGACATCCCCGTGAGTCTACGTCTTGGAGCTTGGAGGCAGTGCCCTCTCTAGGGCTTCAGGACCTTAAGCTCCCACAGTTCCCATGATGCATGGCGCTGAGTATTCGCGGAATTTCCCATTGCCAAGCGGACGCGCTCATGACGTATGGTGGATGCTTGGCTGGAGCGCCGCAAAATGTGGGCGCGTTCACGCTGGCACCGGAATTTCCTCCAGGAAAATGCGAGCCGTGTGATTGGTGAGTGGCGGCTACGTCACTGGCCCTGGCAGCCGCCTTGCCTGGCTAATCCCGACGTGAGCCACATGAGCACCGAGTACAGTCTACTTCCTCTCGAGGATGCTCTGCCATCTTCGGTGTTAAAGAGTCGCACGTTCTTGTCCAAAACGTACCTCATGACAACTTCGCCGTCCTTCTCGTGGGGTTCGCTGTCTCGGCCGAATATGAAGTAGCTACGTCCCTTGGTGAGGTCGGCCGTGTTGCAAAACTCCCTCGCCACGAACACTTTCAGACCCGTCATTGCGGAGGTGCTGTTCTCAGCGCCTTCTTTCACAACGCTGTCCACACGGAAGTCGATGTAACGGTAACCGTTGACGATCCTGTTGGCAGACACCATTCCGATCCAAACGAAATCATGCTCCTGGCACGTGAGTTCGACTAGCTTCTGCCTTTTCTTCACTGTCGTTCCTGATCTCTCGACGTCAATGAAGGGCTCTTTCCTTGGGCATGCCGCTTCGGCGCACTGGCACTGATCATTTTCACAGATCAGCTTCAGAAGGGGACTCGTGGATCCGGGCCCGTAGAACTGCGAACACGAGTGCATTGGGTTGTAATAGTCATACACCTTCACAACGGCAGACTGAACATTGTAGACCACATGCTGACGCTCTATTCTGAATTTGACGCACGTCGGGGATTCCCAAGGTATCTTGTCGAAATAAAGGATCACATTTTTCTCAGTCATTTCATACTTGGCCAAAAGGGAGTTGTTGGCTTCTCTTGCCGCTTCCAGATCTTTTTCCATGGGTTTGAATCCACTGAACAGGCCAACCTCTATAACGGCCATGTTGGAATCCTCCCGGCCAATGTAGCGTGAGCACACTTCGATGTCGTAAGTAAGCTTGCTCTGTCGGGGTCCTTCCACTGAGACGGCGGCGGTGTCACTGTTACCGCTTGA

>AAUF6690

GCAATCTAAGGTTTTCATAACAGCACCGCCTTTTTTTTTCATGAACATCACCTCGACTTCTTGGGGCGGGCAAGCACCTTCCAAGCATTTGCAGACGTCAGAGTGACCCTCATTGTCATCGCTGCAGTTTGACTTGAGCAAGGGACTGGTGCTGTCTGGCGAGTAGAATTTCCTGCACGATATATCGGGGTCGTAGTAAGCGTACGCCTTCACGGAGCCCGACTGCAATTTGCCTACGGCGAATTCCTGCTGCAGGCCAAACTCGACGCACACGCTCGCGTGCGATGGAATGGTCGACAGGTAGAATACCACGCTACGCTGGCTCAACTCGTACGAGTCCACACGTTTGTCGGCCACCAT

>AAUF7490

ATGAGGAACGCCTTAGTGAAGGGTTCCGGAAATTTTGGCCACCTGGGGTTCTTTAACGGATACTGCCTCTAGAATTACTCCTCCATAAAAATTCGACCGCTGCGGCCGAGATCGAACCCGCACCTTTCGGGTCAGCAGCCGAACACCACAACCACTGAGCCACCGCAGTGGCCCTATGTTGGTATAACACTGCTATCAGTGTAATGAATGTCAGGCGACACCAATGGAGCCTTGCAGATTTATTCACTTTTGTGTGATTGCATTTAAGTTAGTGCGTAGAACAAGCGCCAAACATCCTGAACGTCCAGTTCCATCTGCTGAGACTTGATTGACCCCCAAAGGGCTACACTAATCATTCTCTACAGCTCTGCCAGGCAGATTGCCTTTATAACGACATCACCCTCGTCAATCGAATAGCGCAGAAGCGTTCTTTTCTTAATGAAAGACCGCTGCTTGACAGACAGAAAAAATCTCCCGGAACGCTGCATATCGATCTTGTACCCGTGTCGCAGCACTCGGTGGCATGGTATCGTATTGACACTGACCGGAAATGCTCTCACAGAATATTTTGCCACCTTCAGTTGTCGATGCAGCCGTCATGCAGTGAAGCTTGCATCCCACAAAATGACCGTTATATAGCCCATGTTCACGATATCCGCAATATGACAGCTGGTGTTGAGAAGGACGGCTCTCAACTACTTTGGCATCCTCCCGATCTTTGCATGCTTGCTGTAAGCCACTGTAGGACGGAGTTTATCCTGCTGGCGCTTTGTGATGTCCCAGCCGTTTTTGTGTCGAAGAGACGAACATGCTTATCCAGAATGTAGCGAGACACAACTACGCCGTCCTTCTCGAAGAGCTCGCCGTCTCGGCCGAAGACAAAGTAGCGTGAGCCTTCACTCATGTCAGCCGTGTTGCAGTGTTTGCGAGCTACGAACAGCTTAGTGCCCGTCAAGGCGGCCTCCCTCTGTTCAGCGCCTTGTTTGATCACCGTGTCAACACGAAGTTCGATTTGGCGATAACCATTGGTGACATGGTTCCGAGACACGGTTCCAACCCACACGAAGTCGTGCTCTTTGCAAGCTAGGTCGAGCAGCTTGCGCCTCTTTTCAGAAGTCGCTCCCACGTTGTCAATTGTTCTGAAGGGTTCTCTGGGTGGGCATTCATCTTCCACGCATCGGCACTCATCTCGTTCACAGGTAAGCTTCAGGAGCGGGCTTGTCGAATCAGGACCGTAGAATTGCGTGCACGAACGCGTCGGGTCATAGTAGTCGTACACCTTCACGAGAGCCGATTGAACGTTGTAGACGACGTGCTGACGCTCTGTCCTGAACTTGAGGCAGGTTGCATTTCGCCATGGTATCCAATGAAAATATAGCATTACATTCTTCTCAGTTATGACGTAGTTAGCCAGGAGCTGATTTTTAATTTTGACCGCTTTCAGGTCCTCTGCAATGGGAACAAAGCCACTCAGGAGTCCAACTTCTATGACCGTCATGTTAGAGTCCGACTCACTAAGGTACTGTGCACACACTTCGACGTCGTAAATGAGCTTGCTCTGCTGTGACGAGCCGCTTTGGGAGGCCGTAGTACCAGTGATTCCTGGCAAGGACCAGCCGGCACGGGCGGATCGACGTGCCCTGTTTTCTCCCAGTAGCTCGAGCAGAAGCTCTTCCGGGAAATCCGACACTGCAGCGCTTTTCTCCTTCGGGGTATGAATGTCAGCTCGAACTGAAATGTTGAACTTGCACACGAGCTCAGGTGGGAGGAGCACGTTGTACTTGAGTCTCACGGAGAGCAGTCCACTTCCGGTTCCGTTCGCTGTGACGAGCAACTTACCCGTCACGTCGTGAACGTCGAGGTGCTGCAGAACCGCCGCGTTGTCCCGCTTGATCCGGATGCTTTTCTGGAAGGCGCGCTTGCCACTGTGAGTCACGTTGCACGTCAGGTCCATGTCGGACTGCTGGTACTTGAGTGCGAACTCCGTCAACGCCTCTAGGGCTACCACAGAGTGGTGCGTGGGTATATCAGACGACGAGCGGTGCAACTCCAGCCAGTTTGCCAACGTTTGGATCATCTCGGTGTCGCTGTCGTTGGAGGCCAGTAGAACCAGCAGAACGTAGGCTGTGCTTTCGACCACCAAGGGGCTGGCATCACTTCCCGCACTTCGCGTGTTATGTACCGGGTCGTAGATGAGCTTCTGCTTGAGTATCGATGTTATGTTGTGAGGCTGTTGATGCTCCGTCATCAAGAATATGTATGCCATAAGCGCTGCACCATGAGGGTTGACTCTTTCCAAGTCGGCCATGTTCGTGTAAGCACCACCGGGACTGAAAGGCTGTGCTAGCGCCTGCCAGGTATTGACGTCGCTGCCTTCAGGCTTCACCACGTGCCAGCACTCCCTGAAAGTGCGGCCAATGAAGGCTGTCGTAGAAACGGCTCTATCACCAGGTCCCGGGATGCTCTTGTCCACCAAGGGCCACTCGAAGCGCAGGTTTCCACTCTCCTGCCGTTTGCGTATCAGCCA

>AAUF19807

CACAAAGGCCTTGACTTTTGTTTTATTGAATAGTTAATAAAAACGTTCAACTGCTTCAAATGTATCATGAATAGATTGGGAAACTACACACATTACTCACAACCTACTTTCAGAGTCACAGCATGGCTGCTTCAATACTGGAATGCACACCAATTCCTTGTGAAGCAACTCACACAACTGCGAGACCTGAAGTTTCACCCAGAGGCATTTACAAGAACATTTTATGTCTAAGTATTTACAGTTTTTTGGCAAGACAGCAAGATAAATAGTCCAGAGCAATGTGTTTTGGCCTTGGTAAAAAACCACTCTATATTATTGCTCGTCCTTCTTGCAGCACTTGAACGTTGCACGAGACATAAAAATGTGAACTCAATATATGTACCTTGTTCCAGGTGCCGAATACAATGTTAGCAACAGTACGGCAGCGATGCAACATTTGCTTTCACTGCAATACAGCATGTCATTCAACTTCTTTTTCAACAAGATGCACTTATCTGACGTCAATAACAGGTGAGCAAGTTGGCAGAGTTTTTAAGTTTGGGAGCTTGCAACTGTGAAAAAGCTAGGGGCATTGAAAGACTGTCTCTTACAATGAAAGCAGAATGAAAACCTGAAAAAGCCTCATAAAGATTGCAAAGGGTTATGATTATTTAAGTAGTCTTCTCAGTATGCCATTTGTTTCATTTTTGCTGAAGGACTTCATAACAACTGCTTTCAAACAGCAGTGAATCAGCCATACACTAATATTTGCAATGAATTGCTACTAACTTGAACCAAAAATTACAATTCCTTCTGGTAAGCTCTCACGGTAATTTTTGACCACATACATACCTTTACGACTTATGAGCAGGCCTTCCAGAATATTTTAGCAAATGAAAAACTGTGTGTAGCAATAATTTACTTCACATAAAACGTCATACAAATTGGGGGCAGTACATCTGACTTGCCTCCAGTGAACATTTTACCTCTTTAATCACAATGCCTTCCTGTGATGTTCAACTGTTTTCACATGAAGAATGAGGACTATTGCTTTCAGATTTTCAGTAAAAAAAAAATGAAATAGATGAACAAAGCTCGCAATAAACAGTATTCATCATCTCTTCTTTCAGATGGGATGTTCAAAAATTAACACTGCTGATAGCACATGATCATACAAAATGCAGCATATGAAAGAATGCAAAAGTAAACAAGCACTCAACACATACACACAACACAACTCTTGCTTAGATATGTCAAACTCTAGATGACTACTACTAGATGTTCATAACAGATGAATGCCAAACTAAGAATCAGCTCTGATGATCCAAAGAGAAAACTGCTGTAGCTAAGCTGAAAATGAACTCTCAATTTATAAACCCAAAAATAATGTGCACGTCTCCTGCACAAGGCCAAATGAAAGGTGCATACTAAAACCAGTACTCTAGAAAAATGCACAAAAAAGGAAAGGCGCCTAAACTCTGTTGACTGGCAGGTGCCTTCTTCCCAGGGCAGCTATGCTGAGCCAGGCAGTAATGCCGTGTCCTCTCTCGCAATAGCAAAATGGAACAGCTCCACACCACAATTCAGTGGCGAAAGCAAGCATTGAACCTGCCTCCAAACGGAATGATCCAGGCAGATACTGAAAGAAAAATTGGAGGCTGCAGCTCTGCAGAGCCAGCCCAAACAGGGAACACGTAACGTGCTGCACAAATGAGTCTTGACTGAAGCAGACTGCACAGCTGCTGTGGCAATGTCCTCTGAAAAGTTTGTTCTTGTTGCTTGAGAAAAATAACTGTCACAGCACCACAACTACTGCTAGCCTTTGAAGAGGTGCCTCCACAGCAATACCAGAAGCACAGTCAGGGGCAACAGTGCTTGCCGTTGGTTCAGGTGCAAAAAAGAGGGTGCTGTGTTGAACACTGGGCAGAATGGGCACTGGTAGGAGCCACACACGTGACAGATGCTGAGTGCGTACAGGTTGTACACTTCAAACATTGACTCGTTGAACCGCTCCGGTGCATAATAGTCGTAGACACGAATTGAAATAAACCGTGTCATGTTGGCAACAGGATACCAACGCTGGGCAGTAAAGTTGACACAGATGGGTGATGTATCGAGATAGTCAAAGTAGATTTCAGCCTTCTTCTCCTCATAACGAGCTTCCCGAAGGTTTCGCACAGCTCCAGATTGCACGTATGCATCCAGAGTCTGCTGCTGAATGTAGTAACCTGATGGCAAGTTCACTTCCAGCACTGCCATGCCACTACGGGGACTTTCATCAGTACGGATCCAGCTTTGGCACGACCTAAATGAAACATGAGAGCTGTTCCTTCCATATGAATACTGTCGTATGTTGAGTGCAAATGCAGGCACTGGCGGAGGTGTGACCAAATGCTTCCATGTGTCAACATTGTATTCCATGTGCAGCTGGACAATGGCCAGTCCTGTTCCTTGAGCTTTCACAATGATCACACCCCATGCATTGGGAATAGACAGTGTCTG

>AAUF36574

CAGCCACCTCGCCTTTCAAAGGGTCCAGCTAGCCAGTCGAGGGTCTTTTTGATAGGGCGTTTGCGATCACCTGTTCTGCGGTCGTAGATGCGAACGTACTGGTTGAGCGGGTACCTAAGACTGGTGGCTTCCTCTGTGACGTGCTGGTCACTGTCTTTGCCAAATATAATGTACTCCCTGTTCACATCAAGGTCTGTAGCGTTGCAAAGCTCTCGACCTTGGAATACCCTCACGTTACCGGTCAGAGTCGTTTTGTTCTCTTCTCCTTCCTTTATAACGAGGCTGATTTTGAACACTAGGTGCCTGTAGCCCGTGGTCACGTACTTGGAAACAACTTTTCCTTTCCACACGAAGTCATGCTTGTCACATGCTATTTTTTCTATGATGGCCCGCTGCTTCTGTGTTGTTTTGTTTAAAAGAAGTTGGAATGTTTCTTCAATGGGGCACTCCGCTGCGGTACATTTGCATTGGCTGCCTTCGCAAAAGAAGCCCACCAAGGAACCAGCGGGCCTGTAGAACTGACTGCAAGAATCCTCTGGCTTGTAGTAGTCGTAAACTTTCACCGTTGCAGTTTGGAGGTTTGTTACCGCAAAATCTTTTTTTGAT

>AAUF33432

GGGTCGTGGAGACATAGTGTTTGCACAAACTCTCCCCGTATCTGGTGTACGAAGCTATCGGTTCAGCTTCTCTGCCACATTCCGCATGGCTCCAAAGGCCCGGGTACTTGTCTACTATGTCAGGAAAGATGGCGAAGTCGTGGCTGATGCTGTTAATTACGATGTCGGAGGAATTCTTCGCACACCAGTGCATGTCCAGACAAGCATAGCTGAAACAAAGCCTGGTGGACAAGTGAATGTGTTGGTATCTACAAAACCCAATGCCTATATTGGGGTGCTCGGTGTTGACCAGAGTGTGCTGCTGCTTAAAAAGGGCAATGACTTGAGCCAAGAACAGGTTATAGAGGAACTGGAGTCATTTGACAGTGGGAAAAAGGCAAAGTTGTGGCCTCCATGGTACAGACGGCGTAGACGCTCTCTCTGGTGGCCAGGATCAACAACAGCTCATGATCTCTTCAAGGATGCTGGCATGGCTGTCCTTACTAACGGCCTTGTGTATGAGTTTGATGATGCAATCTA

>AAUF36715

CTCATTCAATGAACCTGGAGAGATCATTTACAAGCCAATGCAGAGTGGCGCTGGAAGTGGGCCTGCACTGACAGCTTATGTTTTGATTGCCCTTCTAGAAAACAAGGCCAAGCAGACACATCCAGATGTAATTCAAGCAGCGGAGGCTTACCTCCTCAAAGAGCTTCGAACGACTAATGACCCTTATGTTGTGTCCTTGGTGACGTATGCTTTGCACTTGTCTGGGCACAGTGCACAAGATGGCGCCTTCCAGAAGCTTCTCTCACTTGCAACACGAGAAGATGACACTATCTATTGGAAGGACCCTGCCCTGGCTGTAAACACTAGTGATAAGCAGTCTGACTATTTCTTCAAGCCCAGCTTCAAGGATGTGGAGATGACTGCCTATGCCCTGCTTACCCTAACTGAAAGGGGCGACATTGGCAATGCTGTTCCTGTCATGAGGTGGCTTGTCTCCAAGCAAAATGAGAATGGCGGCTACAGTTCTACTCAGGATACTGTGATAGGCATTCAGGCTTTGGCACGAGTAGCAGCCAGTGTTGTCTCTCAGACAATTGCCATTGAAGCCAGTGTGAAGTATGGAGATGGAAGAAGCCGCATCTTGAAGATTCATAGTGGAAATGCTCTTGTTCTGCAGCGCCTTGAGCTGCCTTCTGACCTCAAGTATGTTGAGATTGAAGCCACTGGATTTGGAGTAGCAGTGCTACAGGTCTCATGGTCATTCAACTTGGCCGTGTCTTCAGAATCTCCTTCATTTTTCTTGAATC

>AAUF39367

AAGGAATACTGTGTAAGCGCTTCGAGGGCTGCAAGTGTGTCCTGTGTGCCGATGAAGCCATAGTCTGTGGAGCGCATGCGGTTCAGCCAGCGCACGATCTGGTCCTGGAAGAGGCCGCCAAGTTTGAGGTAGACCTGCAGGGCGTACGCAGTGGCCTCCACAGCGGACGAGTCCTCTGGGTGCGGTAGTCGGGGCATCAGGAACGGCCGCTGGCTCTGGGACACGATCACGGGCGGCGGAATGTGCACGCTGCTCCAGTAGACAGCCCCCTCGGCATCTTTCTTCATGGATTCCAGCAGGTTGTAGCCGAATTGGGCTTCACTGGAGCCAGCCTCAAGGAGTGCGTAAGTCGTCAGGGCTACGGTGTATGGGTCCGTCACCTGGCCCAGCTGTGTCTCCAGGTACCGTACGCCGTCGCGCTTGG

>AAUF47824

GGATCGCGTGCAGCCACAGCTCGTGCTGCTGCTGCTCGGTACCTGGAGCGCATGCTGCACCTCATTCAGAAGTTGGAGGACCCCTACGAATTGGCCATTGTGGCATATGCACTCACACTGGTCAACAGCGCTNNNNNNNNNNNNNNNNNNNNNNTCCTTGACGAAAAGATGAGGGAGACAAGTGGCATGAGGTACTGGTCTCGCACTGATCTGCCAGCCCCAACAGTAGTGATTGAAAACAACAAACCTTACCTGCACCCACGACTTCCTTACGTTTATGATGCATCCAATGTGGAGACTACGGCGTATGGGCTGTTGGTGCATGTAAAACGCCAGGCAGTTGTTCAGAAGGAAATTGTAGAATGGCTGAACACACAGCGCCTCTCATATGGTGGATGGGCATCAACTCAGGACACACTGCTGGCGATGGAAGCACTGCTAGAGTTCTCGATCACCTCTCGCTCACGCGATGTCACTGACATCAAAGTCACTGTGGAGGCTCCTTCAACACCGGGTTTCACCAGGGAGCTTCACATTGGACCTGAAAACTTGTCCAAGC

>AAUF49173

AGCCACTCCTATTCCGGTTGCGTTGATGACTATTGGTCGTCTGGCGTCAAAGATATCAAACTCCTTCAACACACCAGCGTTGTCACGGTTGATTTCATAATAATACTTCGTATTATCGGCGAAGATTGAGACGGAGATATTAGGAGAAGCTTTCGTTGCTTGCATGGAGTACTCGGAAAGGGCTTGCAGTGCGACTACAGTGTCCTGGGTGGACACAAACGACCCAGACGCTGACCGGTGCCTCTGGAGCCAG

>AAUF54505

CCATCTTTGAGAAGTTGAACAAAGGCTGACTGCATTCAGCCTACGGATGTACATGCTGACGGTGTTTCCGGACTGGGAGGACTACATATACATCGATCCTCGGCTCATCACTCAAGGGGTCCAGTTCCTGCTCAAGCATCAGGACACCACGGGCGCTTTCTACGAGACAACGCGCAACCCATCGAATCGCAGAATGAGATCGGAGGCTAACAACAAGGACAAAAACATCACGCTGACTGCGCAAGTTGTTCTCACGCTTTCCAAGGTCACCGATCTTACAGGAAGCATTCGAGAGCATGCCAACACTGCCAAGCGCGACG

>AAUM2065

ATGCGCTACGGACACAAGTTTGAGCAAAACACGACAACGACTTTCGCTGTGGAAAAATATGTACTTCCAAGATTTTCGGTGCAAACACACACCCCCGGCTACATTCTGCCTGGTTTTGCCGACATTACGGTTGATGTCAGAGCAAAGTTTGTCAACCAGCAGCCCGTCCGTGGTTTCGTGTCATTTAAGTTTTCCGTCAAGAACGAGGTCGGCGACTTGACAAAGATCGGCACGAGCAACAAGCCGAAACAGCTAGTGGCAGGTGACTGCACCTACAAGCTGGCCCGCACCGACTTGGTGTCCCGGCTGGGCGAGGGACAGCTGGACGCCCTGTTCCGAACCAGAGCGCGCCTGGTGGTCGAGGCAACCGTCACCGAGGAGGCCACGGGCATCCAGGAGACCGGGCGTAATGAGCAGGCCGTTTTCACCACCTCGCCTTACCTGATATCGACAGCACGAAGCGAGAAAAGCTTCAAGCCGGGCACCAAGTTCTACGTGGTGGCGGAAGTGACGTACACCAACGGTGAGCCAGCCGCGCGCGTGCCGACAAGACTCACGAGTGAAAACGGTCGAGTGCAAACTATGAGTCACAGGACGGACGTTAACGGCGTGGTGACCTTCCTCATAATTTCCAAGTCTGGTGACGGTGAATTCAAGTTTACGGTGGAAACCGCCGACCAGGACTTACCGGAGGATCAGCAGGCCAAAGCTTTTGTCATGCTGCAGGCATACAAAGACGGATCAGCAAAATACATCGCCATCGAGAGGAAGGATCCCAGGGCTTTAGTTGCGGCGGGCTCCACGTATGAGGCCTCGATCTTCAAACCTCAGAACTCCCAGCTGACATCAGCGTATTACGTGGTCCTCTCCCGCGGCCGGGTAATGGAGGCAGCGAAGATCGCCACGGAAGGTGGTGTCCTCGACCCGAAGGTGGCCTTCGTCGTGACTCCGGAGATGACGCCCAGCTTCCGAATCCTGGTGGTCGCCTTTGTGGACAACGATCTGGTCACAGACGCCGTCTACGTCAACGCTGTGCCTACGTGCACGACGGACTCGCACTTCACTTTGACCCGGAGAACCCCTGGAGCAGTGCTGGAACCCGGTTCTATTGAGAAGCTCTTGCTGAAGGGTAGAGAAGGCACACGAGTTGGCTTGCTGGGCGTGGACCAGGCAGTATACCTTCTGCGACGCAAGGACCTCCTAACGCGAAAGAAGGTGTTCCAGTCCATGGAAGCGAAGGACATGGGCAACGGACAGGACGCTGGCAGGAAAGCGGTGGAAACCCTTGCGAATGCGGGCGTTGTGCTGCTCACATCACAGGCCAGCTACGGCGCACCAGCGGTAGAGAGACGGCGCGCAAAACGTGAAATCAAGCTGGACATAGTGAAAGAGTACGAAAACGAGACGCTCCGAGACTGCTGCTCGCGGGGGATGCAGCCTGACCAGCTCCTACGCAGTTGCTCGGAACGCGAGGAGGCTCTGCTCAGCTATCTGGATGCCGGCGTGGCCCTCTACACGAGAGAATGTGTCGCAGCCTTTGGCAAGTGCTGCATCCATGTCGAAGACAACCAGGCTGTCGGAAGGTCATCTGCAGAGGAAGCCGACGTGTTGGGCATGAGCAACTTCAAAGATTTAGAGGGTGAAGAAAGGCGAAACTTTAGAGAGACGTGGATATTCAATCAACTAACCCTACGGGAACATGGAGAGGCTAGCCTTGAGGTGACCGTCCCAGACTCGATCACCACGTGGGAGGTCAGCGCTGTGGGCGTGGCCCCATCTGGCGGGATATGTGTACTTGATCCTCTCCAGATTCCGGTCTTCAAGAAGCTCTTCGTCGAAGTCAACCTCCCTTACTCCGTGATCAAGGAGGAACAGATCGAGATACCGGCGACGGTGTACAACTACGACCACAAGGATCTCAAGGTTAGGGTGGCGATGCTCGGGACGAAGGACGTGTGCTCGGGTGTCAAAGAAGGCAAGCCTTCATCGGTGCGCACGCTGACCGTGCCGGCGGGACAGGGCCGCACGGTGGTCTTCCCCGTGGTGCCGTTGGCTGCTGGCGAGAGGGAGATCCGCGTGGCCGCCCTTAGCGATGGGTCAGCTAGGGACTCTGTCAAAGTGATGCTCAGGGTAGAGCCACCTGGTGTCAGCAGGAATGAGAGCTTCAATCTCATTCTCGACCCTCAAAACACACAGAAACGGCCAGCGCGGAACGTCGCGAGCCCCACGTACAGCTACGAAGAAAAGTTCGGGCTTGGAGGCAAGCAACTCATCCAGATGAAGCGGCCTGGCCGATCCCCTAAGGAAGTGATTCCTGGCTCCGAACGCTGTGAGATCGACATCGTTGGTGATGACATGGGCGCTGCTTTGGAGGCGTCCGTAAAGAACCCGGGAACGCTTCTGAGGATGCCACATGGCTGCGGTGAACAGACCATGATCGGCCTGGCACCAACGCTGTACGCCTACGAGTATCTGAAGACCGCCAGCCGCATATCGCCCGTGGACGAGGACAGAGCGCTGGGCTTCATCAGCAGTGGCTACCAGCGGATCCTCAATTTCCGGAAACCTGACGGGTCCTTCGCGGTTTGGAATCATTACAGGTCCAGTCTATGGCTAACAGCATTCGTGGTGAGAACTTTGTGCGAAGCCCGGAAGTCTATCCTCATCGACGAGAAGGTCGTCACAAGTGGGCTCCGTTACATTTTGACCCAGCAGAAGCAGGACGGCAGTTTCCACGACATATCCAACCTAATCCACGGTGACCTCCTGGGCGGTGTGAATGGACCGGTTCCACTGACCGCGTACACCCTTCTCACCCTGCAAGAATGCGATCGAGAAGGCGTTCAGGTGGCCGGCCTGACAGAGTCCTTCGCAAAAGCAACAGGGTTCATCGAGGGACAGCTGAATCCAAACACTTCGCCCTACGTGCTCTCGCTGGCAGCGTACGCGCTCTCCTTGGGCAAAAGCCCCGCAAAGAACGACACCCTGCGCCGGCTCGGCGGCATTTTCCAGAGAGGGAATGAAGGAGGGCTGTATGTGTCCGCCGGTTCCGAGCCACTATCGGTGCAGGCCACGTCATACGCGCTCATGGCACTGCTCAATGCCGGCGAGAGTAGGGACGACATCACGGCCATGGTGCAATGGCTTAACCTGCGAATGAACCCCAGCGGGTCGCTGCGGTCCAGCCAGGATACCGTGGTGGCGCTTCAAGCCCTGTCCAAGTATGCCCTCTACGCACGAGACGCAGACATTGACCTGACCTGTGAAGTGACGTTGAGCGGGGACCGTAACTTTAACCGCACCCTTCGAATCAAGCGAGACAACGCCCAACAGCGCAACAGGATCGAGATTCCAGACAGCAATGACAAGATATTTGTCAATGTGAAAGGAACTGGAACGGCGACTATGTACGTTGTCACAAGCTACGAGTCCCCTGTCAGTGTGGACCTTTTGTGCAAGTTTGATCTGAAGATAAACTTCACACAGCACAAGGTCAACGTGTCGAAAGCGTTGGAAGGTAAAGGGAGTTTCAAAGAGATTTACTCGATGGAAGTCTGTGCGAGGTCTCTTGAAAAAAAATTGAAAGGCATGGCCATCCTGGACGTAGGTCTCCTAACCGGCTTCAATCCCGTGTTGGCCGATTTGGACAAGATGGTGGCCGACAAACGTGTGGACTCGTACGAGTTGAGCCAGCGTAGCGTGGTATTCTACCTGTCGACCATTCCATCGAACGCCAGCGTGTGCGTCGAGTTTGGCCTGCAGCAGGCATTCGCCGTAGGCAAGTTGCAGTCGGGCTCCGTGAAGGCGTACGCCTACTACGACCCCGATATATCGTG

>AAUM3781

TACCGTAATTGCCATAGCCATGGCAAGGTTCGGGCCACTCATAAGAAACAGCGAGTTCTCTCATCGGAAAACTCATTGATGAACCACGCCGCAAGTCTATTCTGTTGTCTATTCTTGGGCTTCGCGGGGAAAACCATCGAGCTGCTGTCGATCAGATACAGGAACTGCTTGGTCTCGAAGTCGTCTTTCTCGATATACGTGGAGTCTTTTCCCATGACAATATACTCCTCGCCATTGGGAATATTGAATGTATTGCAGGTATCACGAGCCTTGATGCGACGTATCTTTCCTTTCAAGTCGTCTTCTTGGCCTGGCTTGAGAACTTGAGTGATGAGGAAAGCCACTTCTATGAAGCCATCCGTCGATACATTGGCTGTCGAATTGCCTCTCCATACGTAGTGAACTCCGTCACAGGCGAATTCTCTCAGAAGCTCACGCTGTTCTTCGTCTTCAAAGTATTCGTTGTCCCGTGTTTTTATGAACCTGTCGAGTGGCTTCTCCGGCGGGCAACCACCTTCCGCGCAAACGCAGACATCGGAGTCGTCACACTTATCATCAACCTTTAGTAGGGGGCTTGTCTTGTCGGGCGAGTAGAATCGTGTGCAAGAAAAGTCTGGTTTGTAGTAAGAGTATGCCTTCACGTAGCTTGACTGCAGTTTACCTGCATTGAATTCTTGTTCGAGAGAGAAATTAACGCAGTGCGTCATGTTTGCAGCAATGAAGGGTACGTAAAAGTCAACATGGCGACTTGTGATAGTATAAAGCTGAATCTTTCTTTCTTTCACGAGCTCATCCAACTCGGTAGCATTGGGCTTGAATCCAGTCAGAAGACCCACTTCCAACATGACCATGCCATCTGGAGCATCCTCGAGTGGACTTGCACAGGCCTCCATGCGGTAGTTTGGCTTCAGGTCAGATGGTGGACGTGAGGAGGTTTTTTCGACACTTCGAGAAACTCGTGTGAGTATGAGTTCTATGTCCGGTTTCGTTTCCACAAAGTTTGCTGTGATGTTAAACTTGCAAAGCTCACCGTCAAGAACTTTAGCGTCGTATGTGTAGTTAAAGTACAGGATGCCGGTACCGGACCCTTTGACTCTGACGAAGATTTTCTCTCCCGGTCGATCAATCTCGATCTTATTCAGGATGGTGGCGTTATCTCGCTTGATCCTAATATTCTTCTTGAAAATTTTGCTGTTGCTCAAGGTCACCTCGCATGCCAAGTCGAGGTTGTTTTCTTTGGCGTATGCGGCAAACTTGGTGAGCGCTTGCAACACCATCACAGTGTCCTGCGTAGATTGAACAGACCCAGTGGGGCCGGTCCTTTTGCTCAGCCAACGCTGAAAACTTCTTATGATCTCGCTTTGTGCGTTCTCCCTGATGAGCGCCATGAGTGCATATGCGGTTGCTTGTGCAGACAGCAGTTCGCTGCTCGCCGGAACGTGTCGGTCGTCTGTGACTTGGTCATAGCGCACGCTTTCCATCAGCCACTGGATCAGTCCATCTTTCCCCGTGTTGTTGGCCAAGGACAGGGCATAGGCAGCCAGCGCCAACGCTCCCGGTGAGTCTCCGCGGTGCAAATTCCGCTCCAGAAACGCCGCTGCTCTCGCCCTCGAGGTTTGCGAAACCGAGTATCCTCCTTCAGCGCACTCCTCGAAGGTGATGAGAATGAAGGCAGTCAGCGCTGATGGGTGCCCGAAATTTAGCACTAGTTCGCTGAGGCTATATTCATGGAAGCCCCCATTTCCTTGCTGCCTGCTAGTTATATAGCGCAGTCCGCTTCGGATGACGTTTTCATCAATCATGACGGATTTCGTAGCTTCGCACAAGTTGCGAATGACGAAAGCCGTCAGCCATGGGCTAGCACTACTCCATTCGAAAACACTGAAAGAGCCATCCGGTTTCCTGTACTTGAGTATTTGGTTGTAGGCTCTTCGAATATAGTCAAGAGCGTCGTTCTCTTCAGCAATGCTGATGCGGTTGGTGGTCTTGAAGAACTCGTAGGCGTACAGTACTGGCATCAGCTTGGCCGTGGTCTGTTCTCCACAATCAGAGGGATAAATGAACGCTTGGTCCGGCTTCTTGATTATAGATTGAAGAACGGCAGTCACTCCGTCTCCGACGATGTCGATTTCGCAGCGCTCTGTGTTTGGCAAAGCAAAATCAGGACGTGGCGACCGAATGTGTATCACCTGCGTTCCATTTGTACCAAAGGTCTCTGTGTATTCCTGTTGGATGTTCCTCGTGCCTCGCTTTTTAGGGTTTTCGGGATCCAAGATGACGGCAAACGATCGTGTTCTCTGAACTCCCGGAGGCCGGACATTCAGCTCCACTTTGACCTCGTCCCCTTCGCCGCTGGTGCTGCGTGCCTTCACGTGGATCTGTTTTTTTCCTGCGGCAAGGGGCACCACTGGGAATATGGCGGTGCGGCCATGTCCGGGTGGAATCTCGAGCACCCGGACCGCGGATGGTTTTCCTAGTTTGGCCCCAGAGCAGATGTCGTTAGTCCCAAGCAGCACCACCTTAGCCGTGATTTGTTTCGTGCCGTAATTGTAGACGGTGGCCGGGATCTCGACTTGCTCTTTCTTGACGACCGAGTAGGGAACGTTGACCTCAACGAAGAACTTCTTCGTCGCCACGATTTCGAGAGGTTCCACCGCGCAGACTCCTCCGCTGGGCGACACGCTCACAGCGTTCACCTCCCATGTGGTGACCGACGCCGGCAAAGTGGCCGAGAATTCGGCGGTGCCGTCGTCCCTAATTGTCATGCTATGGAATATCCACGTCTCACGAAAGTCGTCTCGAATTTGGTTGTGTACTCCAAGATACTCGTCTGAGAGTTCATCGACTCCAGCGGATGAGGATCTTTGAGCGGCGTCGTGTTCAGCAATGTCGCAGCACCGCTCGAAGGCTTCCACGCAATCCTGCGTGATGTTGGCGCTACCCTGATCCATGTACTTCCTCAGGATGCCCGCCCTCTTCGAGCATGAGCGAAGGAACCTGTCTGGCCACTGACCGCGCAAGCAGCACTTTCGCACCGTCTCGTCCTCGTACTCCTCCAGTATGTTCTGTGCATCCCGCCGTCTCCGTCTTCGCTCCTCGTGGCAACCTACACCGCTTCTCATGGGGTTGCCAACGTCCTCATGCGTAAGCAGCACGATGCCGGAGCTGTAGAGTGCCTCGGCCGCGTTCGTTCCTCCACCCGCACCGCAGCCGAGGTCTTTGGAGTCCAACGACCGGAACAACTTGTCCCTCGTCAGAAGGTCCTTTTTCCTGAGCAGATACATGGCTTGATCGACACCCAACAAGCCGACAACCGTGCCCCGTGTTCCAGTAAGGACCAGAGTTTCCTGGGACATAGGCTCGGGTGGATTTCCCAAATTTCTCCTTTCAAGGGTGAACTGAGAGGTCGTTGTGCACGCCGGTTCGGCGTTTACGTAAATAGAGTCGGTTACGACGCGCCCATCAAGTATGGCGAACACCACCACCCGGAAGCTTGGCGTCATTTCCGGTGTGACAGAGAAGGCTATGTTTTGTTCGATTCTCCTTCCTTCTAGCAGCTTGTCGACCAGCTGTATCCTCCCCTTGGACAGGACCACATAGTAGATCGAAGAAGACACTTTGTCGAAGCGATGTGTGAATAACACGGCTTCGTATGTGTCGCGTGCTCTTGGAAAGCTTTTTGGGTCTTTCCTTTCGATGGCAATGAAACCGGATTGAACGGTTGAGTACGGCTCCAGGCTAATCTGTGCTCGGGCTTGGTGCTCCGGTCTGTATCTGCTATCGGCAGTTTCCACCTTGACGGACAGGGTTCCATCTTCGAAGGACACCGGAATTAGGAAGGTTGCCACGCCGTCACTGCGTGTCGTGGCCGTGAAGGTGTCTCTTGAGTTCAGGCGCGTGGCCTTTGTGAACACGTTTGCGGCTGGGTTTCCGTTCACATACGTCACTTCAGCGGTGATATAGATCTTCAAGCCAGGTTTGAAGCTTCGCTGGTTCTTCTTGAGGGATACCACGAATGGCGTCTTGCAGAAGACCGTCTTTTCACTTTGCGCCGACTCTTTTGCGCCGGTGGCTTCCTCTGTGACCGTTGCTTTTACAACGAGGCGACCGTGGGTGGCGTGTAAGTCGGGCAGTTTGTCTCCAATGTTTGGTTCCAGGGCTTCTCGTCTCAGAATGTAATGAGCTTCGCCGCCCTCGAGAAGTTTTGGCGATTTGCTGCTGGAAAACCACCAGATGTCTCCAACCTCGTGCCTCAACCCAAATTCGAGCAGCACAACTCCATGGACGGGCATTTTGTTCACGAAGCTTGCTTTCACTCTGCAGGGTATTGTGGAAAAATTGCTCAGTACGTAATCCGGAACGCTCAGATCAACCTTGAACCTGGGAAGCACGTACTTATCAACCAGAAATGTGGCTGTTGTGTTTTGCTGAAACTTGTAGCCGTACTTCACGACGAGCGCCCATTCTCCCAGCAGCGTGCGCTCGGGCAGCTCGTAGGTGTGCGTGAGCATCAGCTCTTTGCTCGGCTGAAAATCGGTCCTTTCCACAATCACGTTTTGGGGGTTCCGAACTTCGAGCCTGAAGGAACTCGCGGATGGTTCCAGCTTTCCATCGACGGCCAGGAACCTGATGTTAACCGTGCTTCCCGGGTGGTAGATCGGCTTTTCTGTCTGGAGGAAGAAATGCTCTCCCGATGCAGGGCTGACCAAGACCTGCGTTTTCCTCGTCCACAGTCTGCCGCAAGTGACCTCCAGCGTGACGTAGATTTGGTCCCGCCCGTAGAGCAGCTCAGGTAAGTCGGTTTCCTTTACGGCGATGTCCACTATTCGTGGAGTACCACTGGCCACGTCCCCCGTCCAGTGGAAAAACGCACCCCGACTGCTCGGGTAGTTCTGAAGTGTGACCGTCACCCTTTTCTTGACACCGTCCACCATCACCGCGAAGGTTTCGTCGGTGCCCAGCCGGAAGACGTTCGGTGCCACCACAAAGCAGTTCTCTTTGGCAGACACCCGCGGCGTGCACAAAGCCACAAAGAGGGCACACACGAGTGCCAGTGTGCTCGCCATGTTGGCTCGAGGGTGACTAGTTGGCCGGACAGCGTGGCAGCCGACTTAAGTGCCGCAACCAACGAGGAAGTTCGTGGCCAGCTTAGCGCG

>AAUM1502

GGCCCAGGCACAGTATCCGGACGTCAAACTGTCCAACGTCAACTCAAGCGATACTGTCATCCAGACCATCGATGTTAGGCCTGAAGGCTTCCCCATCAGAGAAATCAACACGTTCCTCCTGTGTGCTTCAGGTGATGCAGACAGCGCTCCGGACACCCTGCAAGTCAACCTCCCGACTCCTGTCGCTCTTGTTGAAGGCTCTCAGCAGGTTGTACTAGTTGGCACAGGAGACATCCTCGCTCTCAGCTTGAACGACCTATCGGTTCCCACCATCACCTATTCCAACGCCGAGGGAACACTGGCTGTGCTTGCCTCCAGCGTCTACCTCCACAAGTACCTGGAGCAGACGGGGACTCTGACTGACACGGTGAACTCTGGCCTCCGATCCCGCATTCGCCAAGCGTCCCAGGCTCAAAACAGCTTCCGGTCCTCCGACGGCTCGTACGCTCAGTTTGGCTCTAGCGAGTTCCCGAGAAGCGTGTTCCTCACCGCCTTCGCCGTGAAAGCCCTGAGCGCCGCCAAGGAGTACCTGGGCACCAGCGTCGAAGCCGACATCGAAACTAGCGTGCGCTACGTCCTGCAGCACTGGAACGCTGCCACCGGTTGTTTCGTTGAGAACCAGCCCGGCTCGTCACCCTTCGGCCCCCAGACAGCCCCCGACTTCACCGCGGCCATTGGCGTCATGCTTCTGGAGAGTGGCTACAACTACGAGAACATCACGAACGGTGTCCTCCAGTGCATTGACGCCAGCAATCTGCCTTCCAACCACACGACTGCGCTGAACGCCTACTTTTCGGCGCTCGCTGGACGCACGGACCGAGCTAACAGCGCCTTGGACACGCTTCTAAGCGAAGCTGATAAAAGCAGTGGCTTGACATCGTGGAGCGGCGATGGCCTGACTTACGGCTCGGCAGACACCGCTGGTTACGCAGTTCTCACTTTGAAGTTGCTCGACCGAAACCTGGGAGAAGCGCTGCCAATCGTCCGTTGGCTCATGCAGCAGACCTACGCCCGATACACTTTCTCCTATTCCGAGGTCTACACCGTCGCCATCGAAGCCCTGACCCAGTACTCGAGCGTGGCATTCTCGAAGAACACAAACTTGACTATGAACGTTGCTGTCGACAGTTCATCCCCCGAAACGGTCTCTTTCCCGATCTCTGAGCAGAACAAACTGCTATACCAGGAGCGTCTTTTGAACAGAAGCGATTCGTACAGCTTCAAGGCATCTCTTGCTGACGGCAGCGCAGGGTGCGCCGCTTTGCAGGTTAAGTACTACTACAACTCCAGGAACAGTCCGGTTCAGCGAGGAATACAAGTCAACGTGTCCACGACATCTGGACCAGATTGCAGCACCCTTGAACTGGAAATATGCACACGGTACACCGAAGGCTTTCTGCGCAGCTCCGCGATCGTCCA

>AAUM1513

TTTTTTTGAAAAAAATTAGTTTTATTCACTCTTTATGACACTATTTATAACGGTGAAGCCTCGCAGACGATGACGCGCTTTTCTCGCAAGTAGAAAATATATAAAGCTACTCTTCCACTCAGCTGGATAAGACGCGCTACGGGAAACAACATGGCGACGTCAAAAGTTCACGGCTCTGTTCACGCACACAAAAGCACACACTGCGCAGCGCTCCCTCAGTCGTCCGCACCCAATATCGAGGCTGTAAGATAAAACATCATGTGACCATTGTCTTGATTTCTTCGCGATGTGCGCTGTGGAAGCCATTAATCTTTTTTTTTCGTTCGCGACATGGCACGTATGTCCTTCTCAAGAAGTGTGACCGGTATAAAAAGCCTTTGGTGTTGAACACTACCGAGGGAGCGAGAAAGCATGATTCTGGTAAAAAGAAAATTGGCAACCAAAGGATAAATGCGCATTGAATGAATGGTATGTTGTGACTCTTATTGTCNNNNNNNNNNNNNNNNNNNNNNNNNNNNNNNNNNNNNNNNNTGATGGCCGATGTTCACTAGCAGGACGCCACAAGAGAGTAGGGCACGGAGTTGCTGTTTTCTTGCTCGTAATAGTCGTAGACTTTAACTGTGGCAGGTTTGGCATCTTCAATAGCGAACTCGCGGTGAACGCGAACGTCGAAGCACTTGTTTTCGGACGTTATTTCTTCAAAGTAAAAGTTAACCTGGTTCTTTTCAACTTCATGCCTCTTCAGCTTGACATCTTTCTCACGGTAGAGTCCAAAGATGTGATCCTCATCTGGAGTATAGCCCGATACCAGCTTGAGCTCCACCACTGCCATGTTGGATGGTTGCTCACCGTCGAACCTCAGACAGATTTTGAGCTTGTGGTCATTGCAGTCAGACGCCTCTTGAGATGGAGTGGCTGTGAGCTCGAAACCCTCGCTCTTCGGTGCAGTATGAACGTTGTACTTCAGAGTCGCCGATATAAGGGCACATCCAGGACCTGTTGCTTCTGAAGTGAGCTTGTTGGGCAAGCTGACAACCTTCTTCTCCTGTACGACCAGTTTGGTGTCCTCCTTAAGGTTATACGACTCACTGACGTCAGTACCGTCGACCTTAACTGAGATGTCAACGGGATCTTTGCTAACGTAAGTAGCGAACGCCGACAGAGCCTGGAGACCAAGAACTGTATCCTGTGTGGATGGGAAGCCTCCGCGGCTGTTTCTCTTGGTCGCCATCCAGCGGACAATGGGCTGCGCCTTGCTCAGGTTCTCTTGGGCGTTCAGCTTGAGGTAGGCGAGCACTGCATACGCCGCCGTTTCCACGTCGGCCGACGCTGATGGACCCTTCTTGCCCGCGTTGCTCCAGTAGGTCAGGGCACCTTTGTGAACAGCGATGGACTCCAATTTCTCGAGGTAATCTTTGGCAGACTCATGTCCCGCCAGGGCCGCGGCGTAGGCTGACAGGGCCAAGTTGTGGGCACTGGGGTCACGCTGGGCGCTGATGCAGCGGAGCGCAGACTCAACGACCTTCTCGTCCGCAAGGCCGCCCTCCAGAAGAGCGGTGAGCACGTACGCCGTCAGCGCTCCCGGGGCAGTGGAGTTAACCTTGCCCTTCAAGCCGGAGCTAAGAACAGTGCCGATGTTCTGGAAGCAGCCGTTGGTTTTCTGCTTCGTGATGATCCACTTTATGCTCTCGTTGAGGTTTCCGGCGTCAATAGGAATGTATTTCTCAGCCTGCTTGAAGGACTTGACTACAAACGCCGTGAGGAACATGCTGCCCGAGTTGTCGCGGTTTCCAAAGGCGCTGTACGAACCATCGTAGTGCTTGTACTTCTGCTGGCGCTGGTAGCCTGTCTTGAGATTCTGCACAGCCTTCCTTTCGATGTCTTCCTGGTTCTTGCCCGTAGCCTTAAGGTAGTCCAGGACATAGACGTTTGGCGTAAACTTTACCATGTTCTGCTCACCACATCCCGTTGGAACTTGCACCAGTGAGTCGAGGTTCTTGATTGCTGGGCCCATAATATCTCCTGTGACTTGAACGTAGGCACGGGCCGATCCTTCCACCAAGTCTTCAGGAAGCACCAAGTCAAATTCGTTCTTTGCGCCGCCTTCTCCAGCGTCTTTAGGACAAACGAAGACACTCTGCGTCTCTTCCTTAGGGAAGCCTTCCGCCTCGATGATGAGCGGTCGTGTGACTGCGTCCCTGGCCACCACCTTCTCAGTTGGCTGCTCTCCACATGCGGCATCGCTCTGCGATCCGGCCGCCGACACGGTCAGGTTCACCTCTCCGATAGTTTGTGGCCGGATCTGGAACTTGTGCACTTGGCTCTTGCTGCCGCACACGCAAAGCGTCGTCTCAGACTCGCCCTCAATGTGGAAGTCCGCGGATTCAGCCAGCTTTAGGTCGACCGGCAGGCACTTTTCGAGGTAGTTGAACACGGACACCTTGACGGGCACCAGTTCTCCGCGCACCACAGAGTAAGGCAGGTTGAACGAGGCGAAGAACGGCTGGAAGGCCTTGATCTTTGCCGGATCTGAGATGCCGATTCCGTCCTCTGAGTTGATGCACACAGTGCTTCCCACCCACTCGGTGATGGTGTGCGGAATCTTTTCTTTGAAGTTCAGTTCGCCGTGCTCATCCAGCTCCTTCAGGTCCCACAGCCACGTCTCGGGGAAGTAGGTGCGCACTTCAACGGCCGACTTGGCCGGGATGTTGGCCGATGGTGCGCTCTGCGCCACAGAGTCCAGGGCGACGGGCACGCCGGGCAGGCCACNNNNNNNNNNNNNNNNNNNNNNNNNNNNNNTCGCTGGCGCCGCGAGAGCGTAGGGTGGTCTGTCGTAGATGTTCTTCCGGCATGGCCTGGTTTCAAGCGTCAAGTCTGACATGACCACCACCCCTGATTCGTCGAAGGCGGTGATCGAATCGACGTATTCAACATTTGAAGATCTCGGTCCATTCCAGATGTGGCGCTTGAACTGTTGCGGATTCTTCGCCAACTGCTTGCGACAGTAGTCGTACGACGCCTGCTTCGGCCACGTGTAGCGGGTGATATCCAGACGCTTCAAAATGTCATAAACTTTGTCCTTTGTCAGTTGGTTATCCTGCTTCAGGAGGTGAACACTCTTGTCTACGACGCCAACACCACAGAAGGACCGAGGAGAACCATTCACATGGATGGCAGCAGATGTTGCAGGCTGTACAGTTTCAGATCCGAATCTCATGGTTACGTTGTTCTGGAGGCACTTCTCCACTTCGAACTGCTCGGAGTCAGCGATGACCTCGCCGTCGGGGCGGACGTAGAAAGCGAGCACTTTGACGCGTGGCACGTGGCTGAAATCTGGCTCGAGCTCGAACTCGAAGCTCCCTGTCGACACGCTGCCCTCGGCCATGTTGGTCGGAAGCGTCTCGTTCAGGTCCTCCTCCACCAGGAAGCTGTCGTCAACAGAGAGCGCCTCTTCCGGCTTGAAGGTCACATCCAGAACCTTGTCCTTGAGGATCTTGCCGCGAGCTATCACCTGTAAATGGAACTGCTTCTCTGAGTCGGGCTGACCCGTGTAGCGGAGGCGCACGGGGTGCTTGCCCGAGCAGCGCAAGGGTCCCTTGGACGGCTCGATCTGGATGAAGTTGTTGCTGGCCGAGTACCAAGCCTGGAGGTACAGAGTGCTCTTGGGCTGGTTGATCTTCACGCCGTAGGTGTCGTACTTCAC

>AAUM15929

AGCTACGCAGCTTGCTGAGCCCTCTGGAAGCCAAGCCAAATCGTCCCAGGGTCCTGAAAGGTCACTGTGTACTACGAGAAGAGAACTGGCATTTTGCAGGGCCCACATTATTTCTTGACTCTGCTGCTGCCTTTGAGGAGTCGATGGTCCAAGTTATCACAGATATGAAGATTAATGTCCGGCCTTGTTGGGAGGACGGTGGCCTCTGGACTCCAGAGCATGGTGCACGTGCAGGAATCGGAGATGGGGGTGATGGTGAGATTGCTGAGCTGGCCCCAGTAGCTACAGTTTCAAAGTCTGCAGTGGACATCCGTAGTCACTTCCCTGATACCTGGCTCTGGGAGCTCCATCACATTCGAGGGGATGTACCACTCAACCTGAGCCGAGAGTTGCCCCATTCTGTGACTAAGTGGAGAGCAGGCGCTGTGTGCGTACACACATCTCACGGCCTGGGTGTCGCCACAGATTCTGTGGATGCTATGCAGCCCTTCTTCATTCAGCTGTCGCTTCCCAACATCACCAGACGTGGAGAGATTATTCCTGTCACTGCTTCGCTGTTTAGTTACCTGAAGTCGTGCATACCTGTCAAGTTTGGTGTGACTGCTGATGCCGACTGTTTTAAGGTGACTGGCAGGGTGCAAAAACGCTTCTGCCTGTGCAGTGATGACACGGCTTCATTGAGGTTTTATGCAAGGGCAGAATGTATGGGCCAAATCAACATCACGGCTCATGCCCTGAGCCTTCCTCGAGATGATTCCATTTGTGATCCTGGCAGTGTTCTCGAAACAAGAAGAGCTCGAGATGCTGTGCTCAAAAGCCTGCATGTTTTGCCAGAGGGGATACCCAGGACAAAGTCAGTCTCTGAATACATATGCATTAATACCACAGAAAATGGCACAGCAACTCGCAGCTTTGTACTAAAGCTTCCCAGCAATGCCATTCCCGGCTCCTCTAGAGGACTGTTTATGGTTTCAGGTGACATACTCAGCCCAGTTGTAGCATCAGGCCTGGAGTCCCTCGTTCAACTGCCAACTGGCTGTGGCGAACAGAACTTGGCTATGTTGGCCACCCGTGTGGTGGTGCTGGACTACCTCTTCTCTGTGGGACAATCCAATCATCCCTTGGTGGCCAAGCTCAGGAGAAGCATCATCACAGGTTACCAACAGCAGCTCAACTACAGGCATCAGAACAATGGCTATAGTGCCTTTGGCACTGCAGATCCTGAACCTAGCCTGTGGCTGGCAGCATTTGTGGTGCGGACCTTTGGACAAGCCCGCCAATATGTTTTCTTTGATGATGCTGAGCTGAGCCTAAGCACTCAGTGGCTACTCGGGCATCAATATGATACTGGTTGTTTCCCATCAGTAGGCCGTGTGCTGAACACCCAGCTCAAGGGCGGCGTACAGGGGACATCACTCTCACCACTAACATCCTATGTGTTAATCGCACTGCTTGAAGCAAATGTGACTCTCTCCAGATCTGTACTAAATGCAGCCATCCAATGCCTCATAGAAGAGACTCCTCACGAGCAGGACAGCTACTCACTGGCACTTCAGGCTTATGCCTTGGCTCTTTCAAACCATCATGATGCACAGCAAACTTTTGACTTGCTTTGGAGCAGGGCTGAATCAGACGTTGATGGCACGTTACATTGGAGCCACAACGTGTCTTTGGGAGTGGCTGTAGAGACAAGCGGCTATGCAATTCTCTCCTGCATGACACTGCTCAAGGTAGCAGGTGTACCTAAAGCCTTGCCCGCAGTCCAGTGGTTGGTCAAGCAGAGGAGTGACCATGGAGGATTCGCGTCTACACAGGACACTATTGTAGCCTTGCAAGCCCTGGCTGCATTTGCCAAAGCAACTGTTCTGCAAAGCACACACTTGTCAATGAGAGTGTCGTCCCAACACACAGAGGCCAAAGAGCTTTTTGTGGAACCAAGCAATGCAATGCTGCTTCAGGAGGTGGTCATCCCTACAGTGCCGGCGATGGTGAACCTCAACGTCACAGGGAAGGGGTGTGCCTTTGTGCAGGCCTCTGCAAAGTTCAACATTCCTGCAGTGGCTGATACCAACAAGTTTGATCTCATGGTACTGGCATCCCATGCGGACTGTAAGCCAAAGATAGAGCTTTGCGCTAGTTACCTCCTCTCCTGGGGCATATCTGGAATGGTGATCATACAAGTCCATCTGCAAACTGGGTTCCGTTTGAGCAAGGTTGTTGATGGCCTGGAAGTTAGGAAGACTGAGGAAGAGCCTGACAAAATAAACTTGTACTTTGATGAGCTAAGTAATCGAAGAATGTGCATCACCTTAATGTTGACCCAGGAGTTTGAAGTGCAAGACAGTGCTCCTGCAGCTGTTGTATTGCAGGACTACTATGATCCTGGTGTCATTGTACTAAGGAACTACACAATTCCAAGTTGTGAGAGGGATCAACAAGACCTTAGTGACTCACAAATGCCCCTGCAGTCAGAAATAATGCAAGAAGATATTCGTTCAGCCAGGACTCATCTTGGAAACTTCCGAAATATCGAACAAGACTTGGACTTCCCTGATGGCCCTGAATCAAACATGCCTGTTACAGTGCCTGCTCCTCAGACCTGACCGGCAAAAGGACATAAACGTGCAGAAAACTTTCATACTGACGGAACTTGCAAGTTGCTGGGATGTGCTCCAAGTTGAACCTCAATGCACTCCAGTTTTTATAAAAAGAACACTATAAGGAACAACATAGAAGAGCCAGAACTGTCTTTTGAAAGA

>AAUM29228

AGAGATTAGCTACTCTGAGGTGCTTAGGAAGATGAATTCTTTTGACACTATCATCAATGGTACATTAACTCAGGAGTGGTTCTCAAGAGATGGTAATATCTACAGTTATGCATGTTTCCCATCATCAACATATGGCCTCGACTCAAACAGAACTTTTGAGTTTACTGGACTGTTGGTCTTCAGTGATTCCAACGTCACCCGCATGAGAGATGACTGCAATTTAACAGCTGGTTACGGCACTTGCTATGATGGTGCTTGCTACCGCCTTGAGCGACAATGTGATGGCTACTTTGACTGTGAGGACGGCTCTGACGAAACAAGATGTGAGGACGAACAGCGGTTCAGCATCCCTAGGTTTGAAATGTATCGAACAAACAGATATCAGCGGCTATATACCAATTCATGGCTTTGGAAAGACATCAACATTGGGCCATTGGGTTACTACATCTTCAACATCCCAGTTCCTGATGTGCCAACAGAATGGATGATCAGTGCATTTGGAATGGACGAGTCCAGTGGATTTGGGATTTTGTCAAACAGCATCTCATTTTCAAGTGCAAAGCCATTCTACATGAACGTAGAAATGCCAACAAAGATTCGTCTTGGAGAGCAGCTTGGTATACGTGTCACAGTCTTCAACTACAGGCTCTATGAAACTGAGGTACTTATCACACTGGCTAGCTCCCCAGATTACAGATTTGTCTTGGTGGGACCTCTAGGCCGGGTAAATTCCTATGCACCAGATACTTCTTCTGGAGAACACCAACACCTGATTTTCATCAAGCCTGGGAAGTCTGCTGTTGTTTACATGCCTATAGTAGCTGTACGCACTGGAACAATTGAAGTCACTGTTCTTGCCAAGACGCAGATTGCAAAGGACATGGTGACGCGTTCTCTTCTTGTCGAGCCTGATGGCATTCCTCAATACCGTCACACATCCATGGTTCTGGACCTCACTCAGGGCGCCTACTTGATAAAGTATCTCGACACAAACATTACTGAGTCACCCATCTTGCCTTTCCGACAAGATCGCTTGTACGTGTTCGGCAGTAACAAGGCCACTTTATCTGTGGTTGGTGATGTGTTTGGTCCAGCATTCCCAACTATGCCTGTCAATTCCAGCAGCCTGTTGTCAAAGCCGTTTTACTGTGGGGAACAAAACATGTTCAGCTTTGGGGCAAATTTGTTCACGGTGCACTATCTACGACAGACAAACCAACGTGACATGCATTTGGAAAGACAGGCATTCAAATATCTGAACTTGGGATACCAAAGGCAGCTGACTTATCACAATGAGGATGGAAGCTTCCAGGTGTTTCGATGGCACAGTCAGCCAAGTGTTTGGCTGACGGCATTTTGTGCACGAGTGTTCCACAAGGCGACATTTCAGGAATGGGAGCAGTTCTTATACATCGATCCCACTGTCATACAAAAGGCCATTTCATGGTTGCTAGACAGGCAGTCTCCCGAAGGCTCCTTTCACGAGACATCTTTCTATGCTTATGACCGCAAAATGTCTCTTCCGTCTGAGAGGCCAGAAGACCCGGTGCGG

>AAUM34725

TAAGCTGTCAGTGCAGGCCCACTTCCAGCGCCACTCTGCATTGGCTTGTAAATGATCTCTCCAGGTTCATTGAATGAGCCATCACTCTGCTGACGTGCTACCAGCCATGCAGTAGCATTTTCAAGTACTTTGGGGTCAATGACAGTGTACGGAGCAGCTTGGGCAAAGCTCTTGAGCACAAATGCTGTCAACCATGTACTGCCACTCTTGTCTGTATTGCCAAAAGCACTAAATGAATTATCATCGCGTTTGTATGTCAGTTCTCGCTGATAGCCATCTTCAAGATTCCGTATAGCTTTGTTTTGGACAGGTGGCGAGAGGCGGATGGCCCGTTTCAGGTAGTCAAGAACAACAACGTTAGGTACAAAATCCAGCATGTTTTGCTCTCCGCACCCATGTGGCATTACTAGCAGTTGGTCCAAATTGTTCACACTGGGGCCGAGAAGGTCACCAATTGCAGAAATACTAATTCGTTCCGATCCGGGAACAGCATTCTTTGGAATGGGAATGCTGATGTTGGCTATTGTGGGAGGAGCTGACGGATTCCGTCTATCAATTAGGATGGCCTTGTTGAAGTATTGCTTCGAGCCTTCAGGCTTGACAAGAAGCTTCTTGAGTATGGCATCACCAGCCAGACTTGAAGTAGCCGACACACGAATGTCTATGTAACCCAGCTTGAGAGGAGTAATAAGAAATGACACAGGGATGCCATCTTGAGCTGGCACCATCACCTTCTTTGTGCGTCTCTCCTTGTTTTCTTCTCCCGTGACATCATTCGACAGAGAGCTAAAGATGAATTCCCCGCGGTTATTCTCCAGTGTCACTTCCGCCTCAACTGGCTTCTTGTTGTAGTTGAAAACAACACACTGGATTGCCACAGACTCTCCCCGAAGAACAGAATACGGCAGACTAGCAGTCACAAAAAATGGCCGGAAGACTGTCACTTTTGCTTGCGAGGGAGCAATTCCAAGACCAGTCAGAGAATCCAGAGCAAAAGCACTGATCACCCAAGATGTAATGGTGTCAGGGATTGTGCTTGCGATAACAGCCCTACCATCAGGACCTGTTAAGGTGCTATTCCAC

>AAUM55137

TGGAAAGTAGAAAATGATCCGTTGTCATTCCTAAACGACAGTTCTTGCTGATATCCTTTCCGCAGATAGGTTAAAGCCGCGTTGAACAACGTCTCGTTCAGCATGTTTTTCATCGTGAGATAGTTGTATGCATACAGGACTGGAGCCATTATCATCATGTTCTGCTCTCCGCATCCCATCGGCATCGATATCAGCGAGTCGACATTTGAAATAACTGCTTCTGTCAACATGGACGCTTCGCTACCAATGATTG

>AAUM55513

AACCTTTCAACCACATCACCATTCATGTTCCCATCGTCTCAACGAAGATCGGGCAAGTGGATGTGGGCCTTGTGGCCAAAACACAGGTAGCACTTAAGGAGGCCACTATCTCCATCAATTTTCTGCCGGATGGTGTGCCACTGCGGATGCACACGTCGCTGCTCATGGATCTGCGGGCGCAGGCCTATAACATCAAATTCCTCGACTTGAACGTCACCGAGGATCCTATCATCCCGTTCGAGAGCCAGTACCGGCGTTACCTGTTCGGATCGCCTGCTGCGCATGTTTCCGTCATTGGAGACATCGTCGGTACGCCCCTGGAAGGAGACGTGGAACCAGAAGAGTTCGGGTTCTCAAGCGCCACAAAATCCGGGGAGCATGCCATGTTCAGCTTCGCCTATCAGGTTATACGGCTCACCTATCTGCGCCTCACAGACCAGCTGACCCGCGACATTGCGAAGCCCATCTTTGAGAAGTTGAACAAAGCATACGTTTATCAGAGCTCTTACTTCAAGAATGGGGCATTCACCATGTTCAAGAAGGAGCCTAGTGTTTGGCTGACTGCATTCAGCCTACGGATGTACATGCTGACGGTGTTTCCGGACTGGGAGGACTACATATACATCGATCCTCGGCTCATCACTCAAGGAGTTCAGTTCCTGCTCAAGCATCAGGACACCACGGGCGCTTTCTACGAGACAACGCGCAACCCATCGAATCGCAGAATGAGATCGGAGGCTAACAACAAGGACAAAAACATCACGCTGACTGCGCAAGTTGTTC

>AAUM8707

ACCTGCCAATCCAGACGAGGCCGTGTCCCAACCGTGCTTTCCCTGGCTATCCCAACAATCAAGTCCTGGGATCGACAAATCGCTACTATCCCAGTTTCGGCGGTTTCGGAGCTGTGAACAAAGTGGCATTTGCTCCGAGTGCCTCCCCCGGATTTGCTGGACCGGCACCCATCGCAGAGTCTGCGCAAGCCGATTTTTCGCCCGTCGTCCAGGGGTCAAACTCCGTCCGCACACTCTTCCCTGAAACATGGCTCTGGCAGATCAAGAGAGTGAGCCCTGACGGTTCCTTGGTGTACTCCGAGACGCTCCCGGACACAATCACCACATGGCAGGGCACTGCCGTATGCCTGCACCCGCGAAACGGCCTCGGCGTCTCCCAAGTGGCAAACGTCACCGGATTCCAGCCATTATTCGTATCGCTCACGTTGCCAGCGTACATGCAGCGCAACGAAGTGGCCACGGTCATTCTCACGGCATTCAACTACGGCGAAGAGTGCGTCGCGGTCCGGGTCAGTCTGAGGTCTCTGGAGAATCTCGAGGTGGTCGGTGGTCCCAACAGCACCGACGCACTGCTCTGCCCCAACTCCAGCACATCCGCGTCATTCCCGTTTGACGTGCGCGCTACCACGCTTGAGGAATCCCGGCTGGAAGCCCGCGTCCAGACGAGACCCGAGGCCCAGGCACAGTATCCGGAC

>AAUM15812

AGTTGCCTTTGGTATGCTGCTGTTAGCAAGCCGCGGCTTTTCTCCTGCAGGCTTTTGTCTGTTAGGCTGCCCCGTGTCAGGTAGTCATGGGCGTACAACGCTTGCGCCATAAGCAGGGCGCTCTGCTCCCCGTTTCCCGCAGGTATCTTCAAGAGAGCTTCCACCTGTTCGAGCGTCTCCTGAACAGGCGGTCTCATCCGATTTCCTGTAACGGATAGCGAGCAGCTTTTCGTGCCGGGAACTGCGTCCGGTGGTAGCAGCGTGTTCACAGAGATAACCTGTCGGTTCGCTACCTGATCGATGACGTCTTCATAAATATCCGCCATCGCTCTCCGAGCGTTGATTCTTCCAGCATTGGTTGGGTCGATCGGAACGGAGACCCTTTTCTCTACTGGCACTCCTTCCGGCACGACATTGAGCTCTTTCTCGACGACGTCCACGCCTTGTGAGCTTAGCACCCGGACCTTGATTACGAAAGTGCCTTCCCGCAGTGGCACAACAGGAAACGTGACACTGGACGCTGAGTTGGCGGCCACCAGCACGTTGCGCCCTTCCGAACGCTCGCCTTTCTGAGCACCGGTACACAGTCCCTCGACTCCGTATATGTGGACGACTCCCTGCAGTAGCTGGTTTCCGTAGTTGTACACGGTGGCGAGCACCTCGATCTGCTCGTTGCGCACCACCTTGTAAGGTAGCGCCACCTGGAGGAAGACCGGCTGGAAGGCGCGCACCTCCTCTGCCTTGGGCACGCACACCCCTCCCGCGGGAGAGACGCTCACCGCCTGCACCGACCACGTGGTGATGCTGTGCGGCAAGCTGACCGCGAAGGCCGCAACGCCGTCCGCACCGATCACTTTCTCGTCGAAAAGCCATGTCTCCCGGAAGTCCTTGCGGGTGAGTGTGGCGCCAATATGGTCATCATCCATCCCATGTGTCTGGCCGGGCGGGATCCGAACCCTCGTAGAAACGATGAATGGGCTCACTCCCTGTGGATTTCTTCCCGGAAGGCCATCGCTGAAGGAGGGCTGCGGGTCGGTAGAGAAGCAGCACTCCTGGAAGGCCTGGGCGCAGCCTTGTCCCCTCGCTCCGGGAATGTAGCGTCGAACGATACGCGACCGCGTGACGCAGTCCCTGTTGGCCCTGTCCGGCTTCAGGCCCAGCGAGCAACATCTATCCAGGAATGGATCGCTGCTGTACTCGTGCGGCAAATTTTGTGAAACACGCCGGCGACGATGAATCCGGGACGCGCAGGCACTCACTGTTCCGGTAACCAGGTCGGCCGCCTCCTCGGCGATGATGACTACGCCGGATTCGGAGAGTACTTCATCGGGTGTCTCACCACCTCCAGGTCCACAACCCATGTCGTGGGACCGCAGTGTCTTAAACAACTTATCACGAGTGAGAAGACTTTTGCTGCTGAGTGTGTAGACAGCTTTGTCCACACCCAGTATGCCAACGCGCGTCCCAGCAGCTCCGAGTATCTTCAGGGAGCCGTTGCTGCCCGGGAGCCCGCTGCTAAACTCCGGCTTGATCGCTATGTTGGATGACTCCGAGCATACCGGCTCCACCTCGATGGCGATCGAGTCAGTGAACACGTGACCCCTATGAAAGTTTAGCACGAGCAGTCGGAAGATGGGGGCCATGTCCGGTGTTACCAAGATGCTCAGTGGGGGAAAGTCTGCATACAAACGCTGCTTTCGCAGAACTCCGTGATGGATGATCCGTCCGTTTTGAGTAACCAGGTAATAAACTGGTGCGCCGCCTGGCTGAGCGAATACTTGAGTCACATAGGTCTCTCCGACCTTGAAAAGCTTCCTGCCGCCATTTGGGTGCAGAACGCAGTAGGCGTCGAACGCATGGTTTAACGCCGGGATGTGCACCCTTCCTTGAGCTTGGTGCCGAGCTTCTTC

>AAUM2548

TCCGAAGTGTAGTTCTTGCAGCTGAGACGGCGGTCGGTGCGCCACCACCGTGGCTTGATCTCCTCAGCCTGTGACAGCAGACAAAGTTGCACCAGCTCGCCGCCCACAGGAACGCCGTCAGGCTTCTTCACCAGGAGCCTGCCGTAGAAAGGCATGGTGGGCTTGAAGTAGTTCTTTCCGCGGTCTCCTTCCAAAAAGTTGAGCTCAAGCGGGTTGAAGCTGCGGCTGATGTAGTTCGTCTTGTTCATCGTGATGCCCGTGCCGGTCTCGTTCACTTGTGCGACGAGGTAGATGCGCTTGTAGATCTCGTAGTTCTCGTTGAAGCGCAGCAGGCTCGTGTTCACCGTCATGTCGAAGCAGCCGTTGATGGGTCCTGTGTGGTTGATCTTTGGGTAGTCGTCCTTCTCCCAACTGTAACGCTCGTATGTCACGTTCACCGTCAGGGTGCCGTCCACTGGCTGTCCAAAAGTATAGTGGGCACAAATCTTCCAGGTGATCTCCTTTGCATCAGCCAAGACGTAGGATGGAGGCTTGATGGTCACCTCAAATTTGGGAAGTACGTATTCGTTCACCTCAAAATGCTGCCTCACGGTTTGCGTGGGAAGTTCGACGACTATCTGCCACAGACCCAACTCAGGCTCTTCTGTAAGCTGAAAGTCTCTCTGGACAATGCCTTTTTCAAAAGACACGTCGTTCCACTGAGCTATTCTTACATCGCTGGGACTTGTCACGTAAATAGTTGCCTTCACATCGGTGACTGGTTTCAGCTCATTGTTGATAGGGAGGACGCGGAATTGAACCCTCTGTCCAGGCTTATAAAGTGCCTTATCGGACTGAACCAGGATCGTGTTCTTGGACTTCTGGAAGTCGATCTCTTTCCGATCGCCGAAGACATAGTCTCCAAAGGTTCCGTTCACTTCGATCTTGGCTTGGCTGTCGAGGTGCTCGGGCACTCGGAACGGCAGAAAGGTGCTTTCACCGTTCTTGATGTCGTACTCTTGTTCTGCCAAGACGATGCTGTCGTTGTTGTACTTGAGCAGGCGGACGGTGACCTTGCCATCCTCCTTGACGTCTGTGAGCGTTAGCCGAAACTGTTCGTCCGTTTCGCTGCGCAGGATCTTCGGCGCCGTGAAGATGTACCCACTTTCAGCAAGGCGCGATGCCAAGGCACCAAGGACTAAAATGAAGGTGCCCCAATGCATTCTAGCTCTGGCCACCGGCACCTCTCGTCACCGTCTGTGCTAGTTTTCTTGCGTCTAAGCCAGCCTTAGACACCTTTCCGTAGACTGTACAGCACAGCTTTCTCTGCTGCGCGCGCTAAGGCCGGTTCCGGGAGCTTTTTCTTGCTACCCTTCTTGTCTCGCTCCTGGCTTGACGTCGCGTGGCGAAGCAAAACACAACACACGCGCTCTAAGGCGGCGGCGTTGGTGCGCAGAGAGTGCCGCGAGACGAGGCCGACAAGACCCTCCGACAGCCCAAGCCCGAACTGATCCGGCGGCTGCTCGCCGGTTGACT

>AAUM7630

GAGTTTGTGCCTGCCGACACGCTGATCGTCCACGTGCCTTCGTCGGTAATGGCAAGGAGGTCGTAGGAGCCTTGAAGGAGGCCGTCGGTGAAGTTGACGTACTGCTGCTGGAACAAGAGCTGGCCATACGGGTTCGTGATGGTAATGTTGGCCAGCTGATTCGTCACGGGGAGGAGGTTGTTGTCAAGCAGCAGTATTCGGTAGTTCACTGTGCTTCCTTGACGGTACATGGGCTTATCCGTCTGTACAACGACGTTGACATTGGTAACGTATTCCAGTTGAACAGACGATCTGTTTTGGAATTGGTCGCTGCCGAATTGTCCGGTCACATCGAGGTACAGGTTGTAGAAGACGCTGGGGCTCAGGTCGGGAACGTGGAACAGCAGCTCGGCGTCGATGCCATCTGCGCCGATGTCATACGTCCGACTTGCCAGCACTGTGCTGTTCTCGTTGATTCCCACCCGAGTGCCCCACAGATCTACTTTCAGTTGTCCCGCCGACGCCGGGTTGGTCACCACAGCGGTGATCTTGATGTCGGTATTGGGAGTCAAGAATCCGGGTGCAGTCACAAGGTACTCGATAGAGGCGGCGGTGGGCGTTGTTCCCAGCGCGACCACAGCTGCCAGCAGTAGCAGCTGGTGCGTCCAACCAGACGCCATGGTGGGGACTTATGGCACCTTTTTTTGCCGGGCGTGTGATTGACTGGGGCCAAACCACGAAGCGCAAACAGACAGGGAGTTGTTGGCTGTACCGGGTTCCGACCTCGCCGTCAGGTGAATCCGCGCGTGCTCGAGTACGCACGCGCCGTGCTCGTCGCGACTGGACAGTTCGCGCTTGAACAAAAGCGAACAGCTCCGGGGGCTTTTTGAACGCCTTCGCTTTGCGTCTCCCGTTCGCCCGGCGGCCAATCA

>AAUM9611

GGCAAATAATGAGTGCGTCGTCCTGACGCCCAACGCTCTCCGCCTGGGTGCCAGGGAGACCGTGGTAGCCATGACCAGTGGCCGCGCCCAGAAGGTCGGCATTTCGCTCCTGGGGAACCGGCCTGGACGCGGCGCCTTCTTTCAGACGACTCTGGACGTGGCAGCAGGCTCTACGGTGGTTGGCGAAGTATTGGTCAGGCCCGAAGACGTTCCCGATATGCAGCTCACGAAAGGAGACGCGACGGTGAAATTGTCTGTGCAATGCGGAGCGTGGAGTCGTGACGTGCTGCTGCCTTTGAGCGGGGCATCCGCAGACCACCTCTTCCTGCAGACGGATAAGCCCATCTACCATCCCGGATCCACCGTGAACATCAGGTTCATTGCTCTGAATGGAACTCTGGAGCCATCCTCTATTCCGTTCAAGCTTGAAGTCAGGAATCCTCAAAACGTGGTCTTGGAGG

>AAUM28713

CGCTGGCACTGCGGTTTTCTACGTGCACTGCACCGGACAGTTGGAAGTGAGGATGCTGAAAAGCAGATGCGGACCCGCTTTTCAGAGGCAGCTGTGCCATCTGGGTTTTTTGCCTGAATCAGTCCTCTGTAGACCAGACCTGGCTTGAAACTGGGCACCGAGCGTTTCGTTACAAATTCCAGGGTGAATGGTGACCAGACAAACTCTTTTGTAGTGTTGATGCGTACTGTTTCTCCAGTAGCACTATCTGTAACTTCGGCTGTTAGAAGGAGGGGACGACGATTGATATCTCTATTGGCGAAGAGCAGATGACTCCCATTGACCTCAAACTCATGGCAACCTCTGATCGGTGTGTCGATATCAATGCTTGGAAGAACTTTTGTCAGCTCAGAAGTCCAGGGGAAGCGTTTATATGTGAGCTGAGCTCTGAGCTGACCTCGTACAGGTTTATCATAGAGGTAGCTTGCACATATTTTCCATGTGGTACTAGATTCCTCAGGAAAAATGTACGGTGGTGCAGTAAGACGAACAGCAAATCGGGGCAATACATAGTCATCCACTTGAAATGCCTGGCAAGTCAGACGATCCAAGATCTGGCAGCAGATTGTCCATTGGCCCAACTGGGGCTCATGAGAGAGCTCCAGTTCCAGTTGGACAATACCTGTGGAGAAAGACACATTCCTCCACTGGGTGATTTTGAAGCGCATTGGGTTTTCAATCCAGACCTCACCTACATGTTCTGCAAATGGAAGNNNNNNNNNGTCAACTGCGAGGATGCGAAATTGAACTAACTGGCCTGGCCTGTAGACAGGTTTATCCGTCTGGATGATAAGTAGGAGTCTTTTATCACTTTCCTTGGTGACAGCAGGCACTGTTTTCTCAAAAGCGTAGCTGCCAAATTTCCCTGAGATTTCAAGCGTGGCATTCAAAGGCAGGCTTTCAGGCACAGACAGCACCATTACATCTGGT

>MG4820459

GCGACGCCGAAAATCTTCCATTGGTGCATGAAATTGCATTTATTGTTGGACAGGAGCCCTCATCAGCGAAGCCTACATAGAAACATATCTCGTATTCAACAGACATGGCCGTTTGACGTATGTCGGAGGCAGCTTATTGCTGCTAAGTTCATGCCTCTTTACTCGAAGGCTATGTGACGTTCCAATGAGGTTGAAAAATATGCCTCCTCTAGACACTTCCTTCCACATGCTAACTTCAGCTGTTCACACTTTAGGTGCAGAGTACGCCATGTGTCGGGTGCTGATATCTTAACGGGAGCACTGGTGTCACAACGAAAGGCAGCGTATCAGTTAGGGCAGCGTTTCCATCCTCGGAGGTTAACCTCTAGCCAGCGGAGAAGAGATCTTAGCGGCCTGGAAGCTTTTTGCGCGTCGAAGAGGCGAACATGTTTATCCAGGGTGTACTGCATTACTACTTCGCCGTCTTTCTTGAGGAACTCGCCGTCTCTGCCGAAGATATAGTAGCTTGCGCCTTCATTCAAGTCGGCAGTGTTGCAGTGCTTGCGCGCCACGAACAGCTTAGTGCCCGTCATCGTAGACTCCCTGTTTTCAGCGCCTTCTTTAATCACTCTAGCAACACGAACTTCGAGGTGACGGTAAGCATCAGTTGCATGGTTCCGAGACACAGTTGCAATCCACACAAAGTCGTGTTCCCTGCATGCGAGATCGAGCAGAGCTTGCCTGAGTGCCCGGAGCCTGCGTGTTGCTCCCAAGCTCCGAAGTGCCAGAAAGGGCTCTCTAGGAGGGCATTCATCTGCAAGACTTGCTCGCATCGACACTCGTCTTCCTGGCACGACAGCTTCATAAGCGGGCTTGTGGAGTCAGGACCGTAGAATTGCGTGCACGAACGCGTCGGATCGTAGAAGTCGTACACCTTCACTACAGCTGACTGAATGTTGTGGACGACCTCCTTGCGTTCTGTCCTGAACTTGAGGCATGTTGGAGCTTCATGCGGTACGTGAGGAAAGTAAAGGATAACACTCTTCTCCGTCACGACGTAGTTCATCAGAAAGGGGTTTTCGATTCGAACCGCCTTTAAATCCGCCTTAACTGGATAAAAGCCACTCAGGAGTCCAACTTCTATCACAGTTACATTGGAGTGTGACTCGTCGAGGTACTGTGCGCACACTTCTAGGTCGTAAGTGAGCTTCCTCTGCTTCGATGACAGGCTGCGTGAATCTGTTGTGTCCACGCTCTCTTGCAAAGACCGGTAGGAATGGGTCGCTCGGCGTGCCATGTGGTCACCAGACAGCTCCCTCAAAAGTTCTTCCGGGAAAACTGCGAATTCATCAGTTCTCTTTACTGGTGCACGAATGGTAGCCCGTACCGAAACATTATGCTTGCACGTGCGATCAGGTGGTAGAAGAACGTTGTACTTGAATATCACGGAGAGCAGTCCACTTCCGGTTCCGTTCGCTGTGACGAGCAACTTACCCGTCACGTCTTGGATGTTGAGCTGCTGGGGTATCGCAGCGTTCTCCGGCTTCACCCGAATGCTTTGCTGGAAGCCACGCACACCGCTAAGCGTCACGGTGCACGTGAAGTCGGTGTCTGGCTGTTGAGACCTTAGTGCGAATTCCGTCAGCGCCTCGAGGGCTACCACACTGTGATGCGTTGGAGTTTTCGTAGGCGGGCGGTGCAACCGGAGCCAGTTAGCGAGCGTTTGAATGGTCTCGGTGTCACTGTCGTTGCAGGCTAACAGATACAACAGGACGTAGCCTGTGCTTTCGATCACCAGGGGAGTGGCTTCGCTTCCCGTACTTCGTGTGTTATTTACCGGGTCGTAGAGCAGCCTCCGCTTCAATGTCGACAGTAAGCCCTCCGGCAGTCGAGAGTTTAGCTTCAGCTGCGAATACATCATTAACGCTGCTCCATGAGGGTTGGTCTTTTCGAGATTATTCTCGTTCAGGAAATCAGCAGCCCGCTCCATGGCTTGTGTAATTGCCCATTCGAGGTTTCTATATGTCACTAAACGGACGCACTCCGCAAAACAGCGGAGAACAAAGGCTTCCCTGGACACTTTTCTTTCGTCTTCTTCCGGGATGCTCGTGTCCACCACTGGCCACTGGTAGGCATCTCCAGTCCTCATTGGCTCCCACACCAACCAATACAAACCCTCCTTGATGACGCTGTCGTCGATGCTTGTGTACCGGGTTGCTTGGCACATGATGCGGGTCACGAAGGCTGTCAACCAAAAGCTCCCTCTGGTATCCTTGAATATACTAAAAGAGCCATCACCTTGACGGAAGGACAGCTGGCGTTGATATCCTTCTTTGAGGAAGCGGCGACTCTTCCCTTCCAGTGTGCTGTCCGTTAGGTTGCTCCGCGTCACGTACTCAAGGGCGTACAGGGCTTGGGCCATAAGCAGGGCGTTGTGCTCGCCATTTTCCGCAGGTATCTTCAAGAGAGTTTCGACGTGGTCAAGAGTTTCCTGAATGGCCAGGGTCATCCAGTTTCCTGCGACTGTAAGTGAGCAGCTTTTTGTGCCGGGCACTGCATCGGGTGGCAAGAACAGGTTCAAGGGGACGACTTGCTGATTCACCGCCGAATCAGCGGTGGCTTCGTAAATATCCACCAGGGAGCTCTGACTTTTCATTCTTCCAGGATTGTTCCGGTCTATCGTGACGGAGACTCTTTTCTCCACAGGCACTCCTTCAAGCACGACGTTGAGCTCTTTCTCGACGACGTCTATACTAACGCCTTGTGAGCTTTGGAGCTCAACCTTGATGACGAAGACGCCTTCTTGCAGTGGCACAGCGGAA

>MG4838981

CCGCCCTTAGCGATGGGTCAGCTAGGGACTCTGTCAAAGTGATGCTCAGGGTAGAGCCACCTGGTGTCAGCAAGAATGAGAGCTTCAATCTCATTCTCGACCCTCAAAACACACAGAAACGGCCAGCGCGGAACGTCGCGAGCCCCACGTACAGCTACGAAGAAAAGTTCGGNNNNNNNNNNAAGCAACTCATCCAGATGAAGCGGCCTGGCCGATCCCCTAAGGAAGTGATTCCTGGCTCCGAACGCTGTGAGATCGACATCGTTGGTGATGACATGGGCGCTGCTTTGGAGGCGTCCGTAAAGAACCCGGGAACGCTTCTGAGGATGCCACATGGCTGCGGTGAACAGACCATGATCGGCCTGGCACCAACGCTGTACGCCTACGAGTATCTGAAGACCGCCAGCCGCATATCGCCCGTGGACGAGGACAGAGCGCTGGGCTTCATCAGCTGTGGCTACCAGCGGATCCTCAATTTCCGGAAACCTGACGGGTCCTTCGCGGTTTGGAATCATTACAGGTCCAGTCTATGGCTAACAGCATTCGTGGTGAGAACTTTGTGCGAAGCCCTG

>MG4814902

CCGAGACGTGGCTGTGGGACCTGAAGGAGCTGGATGAGCACGGCGAACTGAACTTCAAAGAAAAGATTCCGCACACCATCACCGAGTGGGTGGGAAGCACTGTGTGCATCAACTCAGAGGACGGAATCGGCATCTCAGATCCGGCAAAGATCAAGGCCTTCCAGCCGTTCTTCGCCTCGTTCAACCTGCCTTACTCTGTGGTGCGCGGAGAACTGGTGCCCGTCAAGGTGTCCGTGTTCAACTACCTCGAAAAGTGCCTGCCGGTCGATCTGAAGCTGGCTGAATCCGCGGACTTCCACATTGAGGGCGAGTCTGAGACGACGCTTTGCGTGTGCGGCAGCAAGAGCCAAGTGCACAAGTTCCAGATCCGGCCACAAACTATCGGAGAGGTGAACCTGACCGTGTCGGCGGCCGGATCGCAGAGCGATGCCGCATGTGGAGAGCAGCCAACTGAGAAGGTGGTGGCCAGGGACGCAGTCACACGACCGCTCATCATCGAGGCGGAAGGCTTCCCTAAGGAAGAGACGCAGAGTGTCTTCGTTTGTCCTAAAGACGCTGGAGAAGGCGGTGCAAAGAACGAATTTGACTTGGTGCTTCCTGAAGACTTGGTGGAAGGATCGGCCCGTGCCTACGTTCAAGTCACAGGAGATATTATGGGCCCAGCAATCAAGAACCTCGACTCACTGGTGCAAGTTCCAACGGGATGTGGTGAGCAGAACATGGTAAAGTTTACGCCAAACGTCTATGTCCTCGACTACCTTAAGGCTACGGGCAAGAACCAGGAAGACATCGAAAGGAAGGCTGTGCAGAATCTCAAGACAGGCTACCAGCGCCAGCAGAAGTACAAGCACTACGATGGTTCGTACAGCGCCTTTGGAAACCGCGACAACTCGGGCAGCATGTTCCTCACGGC

>MG4820080

CGTCCGCAAGGCCGCCCTCCAGAAGAGCGGTGAGCACGTACGCCGTCAGCGCTCCCGGGGCAGTGGAGTTAACCTTGCCCTTCAAGCCGGAGCTAAGAACAGTGCCGATGTTCTGGAAGCAGCCGTTGGTTTTCTGCTTCGTGATGATCCACTTTATGCTCTCGTTGAGGTTTCCGGCGTCAATAGGAATGTATTTCTCAGCCTGCTTGAAGGACTTGACTACAAACGCCGTGAGGAACATACTGCCCGAGTTGTCGCG

>MG489649

CTCGGGGATCCATCACTGAAGTTTATTGCTGCGTTTTAAAGCTGGGGCAAGTCGTTCGCATCGAGAAGTTTCGAACACAGTTCGTACTCGCCAATGTTCGGTGCATGAATGGTTGTTAAACGAACGCACGCCATCTGTGTGTTATGTTAATGACAATACCGGTATTCTGCTGGCGAAAAATCACATTTACATTACCGTAATTGCCATAGCCATGAAAAGGTTCGGGCCACTCATAAGAAACAGCGAGTTCTCTCATCGGAAAACTCATTGATGAACCACGCCGCAAGTCTATTCTGTTGTTTATTCTTGGGCTTCGCGGGGAAAACCATCGAGCTGCTGTCGATCAGATACAGGAACTGCTTGGTCTCGAAGAGGTCTTTCTCGATATACGTGGAGTCTTTTCCCATGACAATATACTCCTCGCCATTGGGAATATTGAATGTATTGCAGGTATCACGAGCCTTGATGCGACGTATCTTTCCTTTCAAGTCGTCTTCTTGGCCTGGCTTGAGAACTTGAGTGATGAGGAAAGCCACTTCTATGAAGCCATCCGTCGATACATTGGCTGTCGAATTGCCTCTCCATACGTAGTGAACTCCGTCACAGGCGAATTCTCTCAGAAGCTCACGCTGTTCTTCGTCTTCAAAGTATTCGTTGTCCCGTGTTTTTATGAACCTGTCGAGTGGCTTCTCCGGCGGGCAACCACCTTCCGCGCAAACGCAGACATCGGAGGCGTCACACTTATCATCAACCTTTAGTAGGGGGCTTGTCTTGTCGGGCGAGTAGAATCGTGTGCAAGAAAAGTCTGGTTTGTAGTAAGAGTATGCCTTCACGTAGCTTGACTGCAGTTTACCTGCATTGAATTCTTGTTCGAGAGAGAAATTAACGCAGTGCGTCATGTTTGCAGCAATGAAGGGTACGTAAAAGTCAAC

>MG481390

TCAGTGCACGTTAAAGAACCCCAGGTGATCGAAATTTCCGGAGCCCTCCAATACTGCGTCCGCATAGCCCGAGTCGCTTTGGGACGTTAATCGCCAATAAACCAAACCAAACCAAAAGTGAAACCCTGATAGCAAGGTGGAAGGATTCCATGTACATTGGACACTAAGCCACTCCCATTCAGAGCAGCGCAGTGCTGAAGAGGAGTCAATGGCGACGATGTAGTCTACAGAGTAAAGGAGTTTAAAATCGACGTTCTGGATTCCACCAAAGAGCGTATAGTTCTTACCGTCTGAATGATGAGCTGGATTTGGTTGCCGTTGACGACATACCGCTTAACGACGCCACTGCTCACGAGATTTTTGAGAGACTGGTCATCTGCCGAGTATCCAGAAAGCAGGGTGATCTGGACGATCGCGGAGCTGCGCAGAAAGCCTTCGGTGTACCGTGTGCATATTTCNNNNNNNNNNNNNNNNNNNNNNNNNNNNNNNNNNNNNNNNNNNNNNNNNNNNNNNNNNNNNNNNAAAGCGGCGCACCCTGCGCTGCCGTCAGCAAGAGATGCCTTGAAGCTGTACGAATCGCTTCTGTTCAAAAGACGCTCCTGGTATAGCAGTTTGTTCTGCTCAGAGATCGGGAAAGAGACCGTTTCGGGGGATGAACTGTCGACAGCAACGTTCATAGTCAAGTTTGTGTTCTTCGAGAATGCCACGCTCGAGTACTGGGTCAGGGCTTGGATGGCGACGGTGTAGACCTCGGAATAGGAGAAAGTGTATCGGGCGTAGGTCTGCTGCATGAGCCAACGGACGATTGGCAGCGCTTCTCCCAGGTTTCGGTCGAGCAACTTCAAAGTGAGAACTGCGTAACCAGCGGTGTCTGCCGAGCCGTAAGTCAGGCCATCGCCGCTCCACGACGTCAAGCCACTGCTTTTATCAGCTTCGCTCAGAAGCGTGTCCAAGGCGCTGTTAGCTCGGTCCGTGCGTCCAGCGAGCGCCGAAAAGTAGGCGTTCAGCGCAGTCGTGTGGTTGGAAGGCAGATTGCTGGCGTCAATGCACTGGAGGACACCGTTCGTGATGTTCTCGTAGTTGTAGCCACTCTCCAGAAGCATGACGCCAATGGCCGCGGTGAAGTCGGGGGCTGTCTGGGGGCCGAAGGGTGACGAGCCGGGCTGGTTCTCAACGAAACAACCGGTGGCAGCGTTCCAGTGCTGCAGGACATAGCGCACGCTAGTTTCGATGTCCGCATCGACGCTGGTGCCCAGGTACTCCTTGGCGGCGCTCAGGGCTTTCACGGCGAAGGCGGTGAGGAACACGCTTCTCGGGAACTCGCTAGAGCCAAACTGAGCGTATGAGCCGTCGGAGGACCGGAAGCTGTTTTGAGCCTGGGACGCTTGGCGAATGCGGGATCGGAGGCCGGAGTTCACCGTGTCAGTCAGAGTCCCCGTCTGCTCCAGGTACTTGTGGAGGTAGACGCTGGAGGCAAGCACAGCCAGTGTTCCCTCGGCGTTGGAATAGGTGATGGTGGGAACNNNNNNNNNNNNNNNNNNNNNNNNNNNNNNNNNNNNNNNNNNNNNNNNNNNNNNNNNNNNNNNNNNNNNNNNNNAGGTTGACTTGCAGGGTGTCCGGAGCGCTGTCTGCATCACCTGAAGCACACAGGAGGAACGTGTTGATTTCCCTGATGGGGAAGCCTTCAGGCCTAACATCGATGGTCTGGATGACAGTATCGCTTGAGTTGACGTTGGACAGTTTGACGTCTGGATACTGTGCCTGGGCCTCTGGTCTCGTCTGGACGCGGGCTTCCAGCCGGGATTCCTCAAGCGTGGTAGCGCGCACGTCAAACGGGAATGACGCGGATGTGCTGGAGTTGGGGCAGAGTAGTGCGTCGGTGCTGTTGGGACCACCGACCACCTCGAGATTCTCCAAAGACCTCAGACTGACCCGGACCGCGACGCACTCTTCGCCGTAGTTGAATGCCGTGAGAATGACCGTGGCCACTTCGTTGCGCTGCATGTACGCCGGCAACGTGAGCGATACGAATAATGGCTGGAATCCGGTGACGTTCGCCACTTGGGAGACGCCGAGGCCGTTTCGCGGGTGCAGGCATACGGCAGTGCCCTGCCATGTGGTGATTGTGTCCGGGAGCGTCTCGGAGTACACCAAGGAACCATCAGGGCTCACTCTCTTGATCTGCCAGAGCCATGTTTCAGGGAAGAGTGTGCGGACGGAGTTTGACCCCTGGACGACGGGCGAAAAATCGGCTTGCGCAGACTCTGCGATAGGTGCCGGTCCAGCAAATCCGGGGGAGGCACTCGGAGCAAATGCGACTTTGTTTACAGCTCCGAAACCGCCGAAACTGGGATAGTAGCGATTTGTGGATCCCAGGACTTGATTGTTGGGATAGCCAGGGAAAGCACGGTTGGGACACGGCCTCGTCTGGATTGGCAGGTTGGTGAAGACGACAAGACCAGTGTTCTCGAACGAGCTCAGGGAGTCGTAGCTGAAGGAGACTGAAGGGGCCACTGTCTGCCTCCTCCTCCGCACCTTGAGCTGGGAGTCACCCGACTTCTCGTAGGGTTCGTTGTAGAGATAACCGTCAACATAGCAGTAGCTCTGGTTGACGAGAGAAGTGCGGGTGTACCTGTAGTTGAAGTAGCTGATCTGGTTGAGGAGGGCGTCCCTAGAATTGCGCTGGTTGTAACCATCCAGAAGNNNNNNNNNNNNNNNNNNNNNNNNNNNNNNNNNNNNNNNNNNNNNNNNNNNNNNNNNNNNNNNNNNNNNNNNNNNNNNNNNNNNNNNNNNNNNNNNNNNNNTGTTCTCCAGGCACTTTTGGACACTGAACTCCGCCCAGTCCGAGACGACTTCGATGGGCCTTTCATTGGAGTCACGGTGCACGTAGTACACAAGGAGTTGCACCTTGGGGCTGGCTTCCGCGGGTAGCCNNNNNNNNNNNNNNNNNNNNNNNNNNNNNNNNNNNNNNNNNNNNNNNNNNNNNNNNNNNNNNTCGTTGCCGTTGGACTGGTCCATGGTGAGCTCGGCTTCGGTGAAGTGCTTTGTGAAATTCTGCGTCTCGAGGATGTGGCCGCCCGAAGTGAGCGTGAGGAAGATGTGGTAGTCGACGTTGGCGTCGGCAGTCATCACGATGGGGCGCGTGAACTGGGCCTGGCACGTGTACACACTGCCGTCTCCTCGGTCGATGCGGATAGAGCTGGCCGTGGGCGAGTAGAAGGGGACAAGGCTCAGTTGCGCCGAGGGCTTCTCCAGGGTGGGGCCATTCTTGACTACGATGCGCGGGAAATTGACAG

>MG4838340

GCCCCATTCTGTGACTAAGTGGAGAGCAGGCGCTGTGTGCGTACACACATCTCACGGCCTGGGTGTCGCCACAGATTCTGTGGATGCTATGCAGCCCTTCTTCATTCAGCTGTCGCTTCCCAACATCACCAGACGTGGAGAGATTATTCCTGTCACTGCTTCGCTGTTTAGTTACCTGAAGTCGTGCATACCTGTCAAGTTTGGTGTGACTGCTGATGCCGACTGTTTTAAGGTGACTGGCAGGGTGCAAAAACGCTTCTGCCTGTGCAGTGATGACACGGCTTCATTGATGTTTTATGCAAGGGCAGAATGTATAGGCCAAATCAACATCACGGCTCATGCCCTGAGCCTTCCTCGAGATGATTCCATTTGTGATCCTGGCAGTGTTCTCGAAACAAGAAGAGCTCGAGATGCTGTGCTCAAAAGCCTGCATGTTTTGCCAGAGGGGATACCCAGGACAAAG

>MG4838633

ATGAGCCCTGACGGATCCTCTGTTTACTCCGAAACTCTCCCGGACACCATCACCACATGGCAGGGAACTGCCGTATGCCTGCACCCACAAAATGGCCTCGGCATCTCACAAGTGGCGAACGTCACCGGGTTCCAGCCGTTATTCGTGTCGCTCACGTTGCCGGCATACCTGCAGCGCAACGAAGTCGCCACGGTCATTCTCTCCGCGTTCAACTACGGCGATGAGTGCGCAGCGGTCCGGGTCGGTCTGAG

>MG48453

CGCAAAGCGAAGGCGTTCAAAAAGCCCCCGGAGCTGTTCGCTTTTGTTCAAGCGCGAACTGTCCAGTCGCGACGAGCACGGCGCGTGCGTACTCGAGCACGCGCGGATTCACCTGACGGCGAGGTCGGAACCCGGTACAGCCAACAACTCCCTGTCTGTTTGCGCTTCGTGGTTTGGCCCCAGTCAATCACACGCCCGGCAAAAAAAGGTGCCATAAGTCCCCACCATGGCGTCTGGTTGGACGCACCAGCTGCTACTGCTGGCAGCTGTGGTCGCGCTGGGAACAACGCCCACCGCCGCCTCTATCGAGTACCTTGTGACTGCACCCGGATTCTTGACTCCCAATACCGACATCAAGATCACCGCTGTGGTGACCAACCCGGCGTCGGCGGGACAACTGAAGGTAGATCTGTGGGGCACTCGGGTGGGAATCAACGAGAACAGCACAGTGCTGGCAAGTCGGACGTATGACATCGGCGCAGATGGCATCGACGCCGAGCTACTCTTCCACGTTCCCGACCTGAGCCCCAGCGTCTTCTACAACCTGTACCTCGATGTGACCGGACAATTCGGCAGCGACCAATTCCAAAACAGATCGTCTGTTCAACTGGAATACGTTACCAATGTCAACGTCGTTGTTCAGACGGATAAGCCCATGTACCGTCAAGGAAGCACAGTGAACTACCGAATACTGCTGCTTGACAACAACCTCCTCCCCGTGACGAATCAGCTGGCCAACATTACCATCACGAACCCGTATGGCCAGCTCTTGTTCCAGCAGCAGTACGTCAACTTCACCGACGGCCTCCTTCAAGGCTCCTACGACCTCCTTGCCATTACCGACGAAGGCACGTGGACGATCAGCGTGTCGGCAGGCACAAACTCTGGTTCGGCAACGTTCCAAGTTCAGGACTACGTGCTGCCAAAGTTTTCCGTTACCATCACTCCTGACACCAATGACGTCGTCACGAACCCTACTGTGCTGTACACTATCTGCGCCAAGTATACCTATGGCGAAGATGTCAAGGGCACTGTGCAAATCTACACATCTCCCTTCAACTACTACTACCCAGTGCGCCAGAAGCCAGTCATCCTTCGCGTCGCTGAGATCAACGGCTGCTACGACTACGTCTTCAACGTGTCGCTGCTCAACACCGTGAACTACACATACCCCACATACCCTTCAATCAACGTCACTGCCAAGGTCATCGAAGACGGCACAGGTGTCTCTCAAACTGCCACCGAGCTGCACAGCCGTGGTACCACACGACTGAGGCTCACCTTCGGACAGAAGTACAATTCCAACAACCAATACACTAGCACAGACAACACCTTCAAGCTTAACCTCCTCTACAAGGGACAGCTTTACGTGCAAGAGTTGGATGGAACTGCGCGCCCGAATGAGCTAGTGCAGCTCTGCATGTTCGTGGAGCGCAACACGTTCAAGTGGCGCTCATGGGAGACGGACAACGTGCTCGCCTGCCGAAACTACACCTCCGATGCCGACGGTCTCGTTGGCTTCAGCCTGCCACCTTTCAATCAGAGGGTCACTGGCGTCATTGTTCAGGCCATCGCTGTCAATTTCCCGCGCATCGT

>MG489185

CTGGGCGGTGTGAATGGACCGGTTCCACTGACCGCGTACACCCTTCTCACCCTGCAAGAATGCGATCGAGAAGGCGTTCAGGTGGCCGGCCTGACAGAGTCCTTCGCAAAAGCAACAGGGTTCATCGAGGGACAGCTGAATCCAAACACTTCGCCCTACGTGCTCTCGCTGGCAGCGTACGCGCTCTCCTTGGGCAAAAGCCCCGCAAAGAACGACACCCTGCGCCGGCTCGGCGGCATTTTCCAGAGAGGGAATGAAGGAGGGCTGTATGTGTCCGCCGGTTCCGAGCCACTATCGGTGCAGGCCACGTCATACGCGCTCATGGCACTGCTCAATGCCGGCGAGAGTAGGGACGACATCACGGCCATGGTGCAATGGCTTAACCTGCGAATGAACCCCAGCGGGTCGCTGCGGTCCAGCCAGGATACCGTGGTGGCGCTTCAAGCCCTGTCCAAGTATGCCCTCTACGCACGAGACGCAGACATTGACCTGACCTGTGAAGTGACGTTGAGCGGGGACCGTAACTTTAACCGCACCCTTCGAATCAAGCGAGACAACGCCCAACAGCGCAACAGGATCGAGATTCCAGACAGCAATGACAAGATATTTGTCAATGTGAAAGGAACTGGAACGGCGACTATGTACGTTGTCACAAGCTACGAGTCCCCTGTCAGTGTGGACCTTTTGTGCAAGTTTGATCTGAACATAAACTTCACACAGCACAAGGTCAACGTGTCGAAAGCGTTGGAAGGTAAAGGGAGTTTCAAAGAGATTTACTCGATGGAAGTCTGTGCGAGGTCTCTTGAAAAAAAATTGAAAGGCATGGCCATCCTGGACGTAGGTCTCCTAACCGGCTTCAATCCCGTGTTGGCCGATTTGGACAAGATGGTGGCCGACAAACGTGTGGACTCGTACGAGTTGAGCCAGCGTAGCGTGGTATTCTACCTGTCGACCATTCCATCGAACGCCAGCGTGTGCGTCGAGTTTGGCCTGCAGCAGGCATTCGCCGTAGGCAAGTTGCAGTCGGGCTCCGTGAAGGCGTACGCCTACTACGACCCCGATATATCGTGCAGGAAATTCTACTCGCCAGACAGCACCAGTCCCATGCTCAAGTCAAACTGCAGCGATGACAATGAGGGTCACTCTGAAGTCTGCAGATGCTTGGAAGGTGGTTGCCCGCCCCAAGAAGTCGAGGAGATGTTCACGAAAGAAGAGGGTGCTGTTCTGAAAACCTCAGATTGCCGAGAATACATGAGATACCACGCCTGCGACAATGTTGATTTTGTATGGCTTGGCACGCCCCTTAATGGAACCCACAAAGATGGATTCATCACTGTTCCTTTCTTTATCAACAAAATCCTCAAGCCAGGCATTGAGTCACAGCAGGACCTTCTCAACAAGACGCGAGTGGTCAAGGCTCGCGAGAATTGCGAATCCTTCAANNNNNNNNNNNNNNNNNNNNCTTCGGCGATGAACAATACGTGTACGTGATAGACAGCGACTCCGTTGTCATCCTAGCGGAAGCAGCCAAAAAACCAAGTCGGACAGAGGGAGGAGGCAGAAACCGGCCCGGGACGAAACCGAGCGGAAAGAGGCCACGTCAAAAGCCAGAAGACCAGCTGTGCGACTTCGTAAAACTCGTCGGCTGGTTCATCAACGAGTTTTCCGACGAGTCGAAGCGTTGCAACACATGAGCACAGTGTCTATGGGCTTTTCTTTTGCTAAAGTTTCTTGAAGTCAATAAATAATAAAACATGCAGGAAGCCCAT

>MG4812515

AACTCCAGGAACAGTCCGGTTCGGCGAGGAATTCAAGTTAACGTGTCCACGACATCGGGTCCAGACTGCAGCGCCCAGCTGGACATATGTGCACGGTACACCGAAGGTTTCCTACGCAGCGTTGCGATCGTCCAGATCACTCTGCTTTCTGGATACTCCGCGGAAGACCAGTCTCTCAAAAACCTGGTGAGCAGCGGCGTCGTAAAGCGGTATGTCGCCAGTGACAACCAGATTCAGCTCATCTTTCAGACGCTGACTGTGGAGCCCACTTGCTTCAGCGTCACAGAAAGCAGGAACCTGCCAGTGGCGAACCTTCAGGATTCGATCGTGGAGGTGTACGACTACTACC

>MG4813720

CTACAGTCCAGATGGGTGCTTAGGTGACTCCGAAAACAGTAGCAAAATCGCAGACAAAGTGCATAATAAAAAGAGCTAGTCTTTCTTTGGCTTTTCTCTACAATTTTTTTTATACCTGGCTTCAGTACAATCCCAATACGCATTACAGCAGTTTTTCACAGCTAAACTGGAACCTGAACTGGAAGGCATTACTGCACAGTAGTATTGTATGCTACAGAAAAGAAATATTGGACAGCTTGCATTTTAAGCTAATTAGTTCATGGTCAGTAGATATAAAGAATTGATGCACGGCATGACTCCAAATAGACTCCACATTCATGCAGCGTTGCTACAGTGGTGCTGCTTTTAACAAGAATCTGGCGATGATATGAAGTTTCAGGTTTGAATGAGGCTTTTTATGGAGGGAATATTGCCAACTATAATGTGCTGAAAACAGGTGGCTTTCAGTACAGTGATGACAGACTATAAAACTTGGGCCAAGCAAGGTCACCCAACTTCACTCCACAAGAAAGATATGGGCACCTGTATTTAACATGTTGCTCAGTTGCATTTCCAAGTTACAAATGAGGAAAGTTCGCATGCTCAATATGTTATGCACACATTTTGGTTCTCAGGTGTAAACTCTGAACAAGTGTATTTTTTTGCTTACCACAAAAGCGAATAAAGCTTCAACTTAATAATTATATGCTTCTTTCAAAAGACAGTTCTGGCTCTTCTATGTTGTTCCTTATAGTGTTCTTTTTATAAAAACTGGAGTGCATTGAGGTTCAACTTGGAGCACATCCCAGCAACTTGCAAGTTCTGTCAGTATGAAAGTTTTCTGCACGTTTATGTCCTTTTGCCGGTCAGGTCTGAGGAGCAGGCACTGTAACAGGCATGTTTGATTCAGGGCCATCAGGGAAGTCCAAGTCTTGTTCGATATTTCGGAAGTTTCCAAGATGAGTCCTGGCTGAACGAATATCTTCTTGCATTATTTCTGACTGCAGGGGCATTTGTGAGTCACTAAGGTCTTGTTGATCCCTCTCACAACTTGGAATTGTGTAGTTCCTTAGTACAATGACACCAGGATCATAGTAGTCCTGCAATACAACAGCTGCAGGAGCACTGTCTTGCACTTCAAACTCCTGGGTCAACATTAAGGTGATGCACATTCTTCGATTACTTAGCTCATCAAAGTACAAGTTTATTTTGTCAGGCTCTTCCTCAGTCTTCCTAACTTCCAGGCCATCAACAACCTTGCTCAAACGGAACCCAGTTTGCAGATGGACTTGTATGATCACCATTCCAGACATGCCCCAGGAG

>MG4820646

TTTTGACCGGCTTCAGCGCCATTGAGGATGACCTCAAGAGGGTTATCATCGACACCCCAGAAGTCAAGAAGTACGAGATAAATGAGAAGAGTGTCATCTTTTACCTGGCCACGATAAAGTCAGCTATTGAGACGTGTGTGGCCTTCCGAATCAAAAAAGATTTTGCGGTAACAAACCTCCAAACTGCAANNGTGAAAGTTTACGACTACTACAAGCCAGAGGATTCTTGCAGTCAGTTCTACAGACCCGCTGGTTCCTTGGTGGGCTTCTTTTGCGAAGGCAGCCAATGCAAATGTACCGCAGCGGAGTGCCCCATTGAAGAAACATTCCAACTTCTTTTAAACAAAACAACACAGAAGCAGCGGGCCATCATAGAAAAAATAGCATGTGACAAGCATGACTTCGTGTGGAAAGGAAAAGTTGTTTCCAAGTACGTGACCACGGGCTACAGGCACCTAGTGTTCAACATCAGCCTCGTTATAAAGGAAGGAGAAGAGAACAAAACGACTCTGACCGGTAACGTGAGGGTATTCCAAGGTCGAGAGCTTTGCAACGCTACAGACCTTGATGTGAACAGGGAGTACGTTATATTTGGCAAAGACAGTGACCAGCACGTCACAGAGGAAGCCACCAGTCTTAGGTACCCG

>MG4829405

TTGAAGAGGTGCCTCCACAGCAATACCAGAAGCACAGTCAGGGGCAACAGTGCTTGCCGTTGGTTCAGGTGCAAAAAAGAGGGTGCTGTGTTGAACACTGGGCAGAATGGGCACTGGTAGGAGCCACACACGTGACAGATGCTGAGTGCGTACAGGTTGTACACTTCAAACATTGACTCGTTGAACCGCTCCGGTGCATAATAGTCGTAGACACGAATTGAAATAAACCGTGTCATGTTGGCAACAGGATACCAACGCTGGGCAGTAAAGTTGACACAGATGGGTGATGTATCGAGATAGTCAAAGTAGATTTCAGCCTTCTTCTCCTCATAACGAGCTTCCCGAAGGTTTCGCACAGCTCCAGATTGCACGTATGCATCCAGAGTCTGCTGCTGAATGTAGTAACCTGATGGCAAGTTCACTTCCAGCACTGCCATGCCACTACGGGGACTTTCATCAGTACGGATCCAGCTTTGGCACGACCTAAATGAAACATGAGAGCTGTTCCTTCCATATGAATACTGTCGTATGTTGAGTGCAAATGCAGGCACTGGCGGAGGTGTGACCAAATGCTTCCATGTGTCAACATTGTATTCCATGTGCAGCTGGACAATGGCCAGTCCTGTTCCTTGAGCTTTCACAATGATCACACCCCATGCATTGGGAATAGACAGTGTCTGCAGCTTGGACAAGTTTTCAGGCCCAATGTGAAGCTCCCTGGTGAAACCCGGTGTTGAAGGAGCCTCCACAGTGACTTTGATGTCAGTGACATCGCGTGAGCGAGAGGTGATCGAGAACTCTAGCAGTG

>MG488951

CGGCGCTCCGCTGCATCAGCGCCCAGCGAGACCCCAGTGCCCACAACTTGGCCCTGTCCGCCTACGCCGCGGCCCTGGCGGGACATGAGTCTGCCAAAGATTACCTCGAGAAATTGGAGTCCATCGCAGTTCACAAAGGTGCCCTGACCTACTGGAGCAACGCGGGCAAGAAGGGTCCATCAGCGTCGGCCGACGTGGAAACGGCGGCGTATGCAGTGCTCGCCTACCTCAAGCTGAACGCCCAAGAGAACCTGAGCAAGGCGCAGCCCATTGTCCGCTGGATGGCGACCAAGAGAAACAGCCGCGGAGGCTTCCCATCCACACAGGATACAGTTCTCGGTCTCCAGGCTCTGTCGGCTTTCGCTACTTACGTTAGCAAAGATCCCGTTGACATCTCAGTTAAGGTCGACGGTACTGACGTCAGTGAGTCGTATAACCTTAAGGAGGACACCAAACTGGTCGTACAGGAGAAGAAGGTTGTCAGCTTGCCCAACAAGCTCACTTCAGAAGCAACAGGTCCTGGATGTGCCCTTATATCGGCGACTCTGAAGTACAACGTTCACACTGCACCGAAGAGCGAGGGTTTCGAGCTCACAGCCACTCCATCTCAAGAGGCGTCTGACTGCAATGACCACAAGCTCAAAATCTGTCTGAGGTTCGACGGTGAGCAACCATCCAACATGGCAGTGGTGGAGCTCAAGCTGGTATCGGGCTACACTCCAGATGAGGATCACATCTTTGGACTCTACCGTGAGAAAGACGTCAAGCTGAAGAGGCATGAAGTTGAAAAGAACCAGGTTAACTTTTACTTTGAAGAAATAACGTCCGAAAACAAGTGCTTCGACGTTCGCGTTCACCGCGAGTTCGCTATTGAAGATGCCAAACCTGCCACAGTTAAAGTCTACGACTATTATGAGCAAGAAAACAGCAACTCCGTGCCCTACTCTCTTGTGGCGTCCTGCTAGTGAACATCGGCCATCAGTTTTTCTTACTCTCCCTTTTTTTTATGTCTCTTTTTTTCGAACGAACGCGCCAAGACAATAAGAGTCACAACATACCATTCATTCAATGCGCATTTATCCTTTGGTTGCCAATTTTCTTTTTACCAGAATCATGCTTTCTCGCTCCCTCGGTAGTGTTCAACACCAAAGGCTTTTTATACCGGTCACACTTCTTGAGAAGGACATACGTGCCATGTCGCGAACGAAAAAAAAAAGATTAATGGCTTCCACAGCGCACATCGCGAAGAAATCAAGACAATGGTCACATGATGTTTTATCTTACAGCCTCGATATTGGGTGCGGACGACTGAGGGAGCGCTGCGCAGTGTGTGCTTTTGTGTGCGTGAACAGAGCCGTGAACTTTTGACGTCGCCATGTTGTTTCCCGTAGCGCGTCTTATCCAGCTGAGTGGAAGAGTAGCTTTATATATTTTCTACTTGCGAGAAAAGCGCGTCATCGTCTGCGAGGCTTCACCGTTAT

>MG4817911

GAGGTTCACCATCGCCGGCACTGTAGGGATGACCACCTCCTGAAGCAGCATTGCATTGCTTGGTTCCACAAAAAGCTCTTTGGCCTCTGTGTGTTGGGACGACACTCTCATTGACAAGTGTGTGCTTTGCAGAACAGTTGCTTTGGCAAATGCAGCCAGGGCTTGCAAGGCTACAATAGTGTCCTGTGTAGACGCGAATCCTCCATGGTCACTCCTCTGCTTGACCAACCACTGGACTGCGGGCAAGGCTTTAGGTACACCTGCTACCTTGAGCAGTGTCATGCAGGAGAGAATTGCATAGCCGCTTGTCTCTACAGCCACTCCCAAAGACACGTTGTGGCTCCAATGTAACGTGCCATCAACGTCTGATTCAGCCCTGCTCCAAAGCAAGTCAAAAGTTTGCTGTGCATCATGATGGTTTGAAAGAGCCAAGGCATAAGCCTGAAGTGCCA

>MG4827761

CGATCCTAATATTCTTCTTGAAAATTTTGCTGTTGCTCAAGGTCACCTCGCATGCCAAGTCGAGGTTGTTTTCTTTGGCGTATGCGGCAAACTTGGTGAGCGCTTGCAACACCATCACAGTGTCCTGCGTAGATTGAACAGACCCAGTGGGGCCGGTCCTTTTGCTCAGCCAACGCTGAAAACTTCTTANNNNNNNNNNNNNNNNNNNNNNNNTGATGAGCGCCATGAGTGCATATGCGGTTGCTTGTGCAGACAGCAGTTCGCTGCTCGCCGGAACGTGTCGGTCGTCTGTGACTTGGTCATAGCGCACGCT

>MG4842057

CCGACCGGCTGAAGTCGATGGATGTCTTGAAAAGCCATCTGCTGGAAGACACTGAGATGAACACATTGAGCACGGGAGACGACGCCACTGGCGTGGACGTNGAGGGCACTAGCTACGCATTGCTCGCACACCTGAGNNNNNNNNNNNNNNNNNNNNGCAAGAAATTCGTGAACTGGCTCCAGCGGCACCGGTCAGCGTCTGNNNCGTTTGTGTCCACCCAGGACACTGTAGTCGCACTGCAAGCCCTTTCCGAGTACTCCATGCAAGCAACGAAAGCTGCTCCAAATATCTCCGTCTCAATCTT

>MG962181

GCACAAAGTTCTCACCACGAATGCTGTCAGCCATAGACTGGAGTTGTAATTCGACCAAACCGCGAAGGACCCGTCAGATTTCCGGTAGTTGAGGTTCATCTGATAGCCACGGCGGATGTAGCGCAGCGCTATGCGTTCGTCCTCGGGCGATATGCGGCTGGCGGTCTTCAGATACTCGTAAGCGTACAGCGTTGGCGCCAGGTATATCATGATCTGTTCACCGCAGTTTTTTTGGCATCCTTTTCAAAAGATCCCCCGGGTTCTTTACCGACGCCTCCAAAGCAGCTCCCATTTCATCACCAACGATGTTGATCTCACAGCGTTCCGAGCCAGGAATCACTTCCTTGGGGGATCGGTCAGGCCGCTTTATCACGGCGAACTGCTTGCCCCCAGGCGCGAAACTCTCTCTGTAGCCGTGCGTGGGGCTTGCGGCATTCCG

>MG9612212

CGCATATCGCCCGTGGACGAGGACAGAGCGCTGGGCTTCATCAGCAGTGGCTACCAGCGGATCCTCAATTTCCGGAAACCTGACGGGTCCTTCGCGGTTTGGAATCATTACAGGTCCAGTCTATGGCTAACAGCATTCGTGGTGAGAACTTTGTGCGAAGCCCGGAAGTCTATCCTCATCGACGAGAAGGTCGTCACAAGTGGGCTCCGTTACATTTTGACCCAGCAGAAGCAGGACGGCAGTTTCCACGACATATCCAACCTAATCCACGGTGACCTCCTGGGCGGTGTGAATGGACCGGTTCCACTGACCGCGTACACCCTTCTCACCCTGCAAGAATGCGATCGAGAAGGCGTTCAGGTGGCCGGCCTGACAGAGTCCTTCGCAAAAGCAACAGGGTTCATCGAGGGACAGCTGAATCCAAACACTTCGCCCTACGTGCTCTCGCTGGCAGCGTACGCGCTCTCCTTGGGCAAAAGCCCCGCAAAGAACGACACCCTGCGCCGGCTCGGCGGCATTTTCCAGAGAGGGAATGAAGGAGGGCTGTATGTGTCCGCCGGTTCCGAGCCACTATCGGTGCAGGCCACGTCATACGCGCTCATGGCACTGCTCAA

>MG9642852

CTTTGATGTATCGGTAACCCTTCTCCTCTAGTGCTTCGTCGATGAGCTTGTTTTGCTTGAGGTACTCGAGAGCGTACAGCGTCGGTGCCATAAGCATCATGGTCTGCTCTCCGCAGCCCGTGGGCATTGTGATGAGCGACTCGATGTTCTCAAGCGTCATTTGAACGGATAGTCCCAGCTGATTTCCTATGACCATGAGAGAGCAGGTCTTTGNNNNNNNNNNNNNNNNNNNGGGCATTCTCGTGTTTACAGAGATCACTTGTAAGTTTGNNNNNNNNNNNNNNNNNNNNNNNNAGAGCTCTCCTTTGATGCTTCTGGTTCTTCGTCTACGAGCATTCGTTGGGTCAATGGGCACAGAGGCACTCTTCTCT

>MG969585

CCAGCGATGCGGGTATCCTACCATTCCTATGAAGCCGCGGCACCAGCGATCCCTCTAGCGAGACCACTCGCACTGTCGGGTCCTCCACCTCCGGCTGGGGTGGCCTTTTCCAACCGCATTGTAGGTGGCCTGCCCGGCGTGCCCGTCGCCCTGGACTCTGTGGCGCAGAGCGCACCATCGGCCAACATCCCGGCCAAGTCGGCCGTTGAAGTGCGCACCTACTTCCCCGAGACGTGGCTGTGGGACCTGAAGGAGCTGGATGAGCACGGCGAACTGAACTTCAAAGAAAAGATTCCGCACACCATCACCGAGTGGGTGGGAAGCACTGTGTGCATCAACTCAGAGGACGGAATCGGCATCTCAGATCCGGCAAAGATCAAGGCCTTCCAGCCGTTCTTCGCCTCGTTCAACCTGCCTTACTCTGTGGTGCGCGGAGAACTGGTGCCCGTCAAGGTGTCCGTGTTCAACTACCTCGAAAAGTGCCTGCCGGTCGATCTGAAGCTGGCTGAATCCGCGGACTTCCACATTGAGGGCGAGTCTGAGGCGACGCTTTGCGTGTGCGGCAGCAAGAGCCAAGTGCACAAGTTCCAGATCCGGCCACAAACTATCGGAGAGGTGAACCTGACCGTGTCGGCGGCCGGATCGCAGAGCGATGCCGCATGTGGAGAGCAGCCAACTGAGAAGCTGGTGGCCAGGGACGCAGTCACACGACCGCTCATCATCGAGGCGGAAGGCTTCCCTAAGGAAGAGACGCAGAGTGTCTTTGTTTGTCCTAAAGACGCTGGAGAAGGCGGCGCAAAGAACGAATTTGACTTGGTGCTTCCTGAAGACTTGGTGGAAGGATCGGCCCGTGCCTACGTTCAAGTCACAGGAGATATTATGGGCCCAGCAATCAAGAACCTCGACTCACTGGTGCAAGTTCCAACGGGATGTGGTGAGCAGAACATGGTAAAGTTTACGCCAAACGTCTATGTCCTGGACTACCTTAAGGCTACGGGCAAGAACCAGGAAGACATCGAAAGGAAGGCTGTGCAGAATCTCAAGACAGGCTACCAGCGCCAGCAGAAGTACAAGCACTACGATGGTTCGTACAGCGCCTTTGGAAACCGCGACAACTCGGGCAGCATGTTCCTCACGGCGTTTGTAGTCAAGTCCTTCAAGCAGGCTGAGAAATACATTCCTATTGACGCCGGAAACCTCAACGAGAGCATAAAGTGGATCATCACGAAGCAGAAAACCAACGGCTGCTTCCAGAACATCGGCACTGTTCTTAGCTCCGGCTTGAAGGGCAAGGTTAACTCCACTGCCCCGGGAGCGCTGACGGCGTACG

>MG9636271

CTGTAGTTGAGCTGCTGTTGGTAACCTGTGATGATGCTTCTCCTGAGCTTGGCCACCAAGGGATGATTGGATTGCCCCACAGAGAAGAGGTAGTCCAGCACCACCACACGGGTGGCCAACATAGCCAAGTTCTGTTCGCCACAGCCAGTTGGCAGTTGAACGAGGGACTCCAGGCCTGATGCTACAACTGGGCTGAGTATGTCACCTGAAACCATAAACAGTCCTCTAGAGGAGCCGGGAATGGCATTGCTGGGAAGCTTTAGTACAAAGCTGCGAGTTGCTGTGCCATTTTCTGTGGTATTAATGCATATGTATTCAGAGACTGACTTTGTCCTGGGTATCCCCTCTGGCAAAACATGCAGGCTTTTGAGCACAGCATCTCGAGCTCTTCTTGTTTCGAGAACACTGCCAGGATCACAAATGGAATCATCTCGAGGAAGGCTCAGGGCATGAGCCGTGATGTTGATTTGGCCCATACACTCTGCCCTTGCATAAAACCTCAACGAAGCCGTGTCATCACTGCACAGGCAGAAGCGTTTTTG

>MG9638302

GTTGTTTCCCATCAGTAGGCCGTGTGCTGAACACCCAGCTCAAGGGCGGCGTACAGGGGACATCACTCTCACCACTAACATCCTATGTGTTAATCGCACTGCTTGAAGCAAATGTGACTCTCTCCAGATCTGTACTAAATGCAGCCATCCAATGCCTCATAGAAGAGACTCCTCACGAGCAGGACAGCTACTCACTGGCACTTCAGGCTTATGCCTTGGCTCTTTCAAACCATCATGATGCACAGCAAACTTTTGACTTGCTTTGGAGCAGGGCTGAATCAGACGTTGATGGCACGTTACATTGGAGCCACAACGTGTCTTTGGGAGTGGCTGTAGAGACAAGCGGCTATGCAATTCTCTCCTGCATGACACTGCTCAAGGTAGCAGGTGTACCTAAAGCCTTGCCCGCAGTCCAGTGGTTGGTCAAGCAGAGGAGTGACCATGGAGGATTCGCGTCTACACAGGACACTATTGTAGCCTTGCAAGCCCTGGCTGCATTTGCCAAAGCAACTGTTCTGCAAAGCACACACTTGTCAATGAGAGTGTCGTCCCAACACACAGAGGCCAAAGAGCTTTTTG

>MG966305

CGGTGTACCGTGTGCATATTTCCAGTTCAAGGGTGCTGCAATCTGGTCCAGATGTCGTGGACACGTTGACTTGTATTCCTCGCTGAACCGGACTGTTCCTGGAGTTGTAGTAGTACTTAACCTGTAAAGCGGCGCACCCTGCGCTGCCGTCAGCAAGAGATGCCTTGAAGCTGTACGAATCGCTTCTGTTCAAAAGACGCTCCTGGTATAGCAGTTTGTTCTGCTCAGAGATCGGGAAAGAGACCGTTTCGGGGGATGAACTGTCGACAGCAACGTTCATAGTCAAGTTTGTGTTCTTCGAGAATGCCACGCTCGAGTACTGGGTCAGGGCTTGGATGGCGACGGTGTAGACCTCGGAATAGGAGAAAGTGTATCGGGCGTAGGTCTGCTGCATGAGCCAACGAACGATTGGCAGCGCTTCTCCCAGGTTTCGGTCGAGCAACTTCAAAGTGAGAACTGCGTAACCAGCGGTGTCTGCCGAGCCGTAAGTCAGGCCATCGCCGCTCCATGACGTCAAGCCACTGCTTTTATCAGCTTCGCTCAGAAGCGTGTCCAAGGCGCTGTTAGCTCGGTCCGTGCGTCCAGCGAGCGCCGAAAAGTAGGCGTTCAGCGCAGTCGTGTGGTTGGAAGGCAGATTGCTGGCGTCAATGCACTGGAGGACACCGTTCGTGATGTTCTCGTAGTTGTAGCCACTCTCCAGAAGCATGACGCCAATGGCCGCGGTGAAGTCGGGGGCTGTCTGGGGGCCGAAGGGTGACGAGCCGGGCTGGTTCTCAACGAAACAACCGGTGGCAGCGTTCCAGTGCTGCAGGACGTAGCGCACGCTAGTTTCGATGTCGGCTTCGACGCTGGTGCCCAGGTACTCCTTGGCGGCGCTCAGGGCTTTCACGGCGAAGGCGGTGAGGAACACGCTTCTCGGGAACTCGCTAGAGCCAAACTGAGCGTATGAGCCGTCGGAGGACCGGAAGCTGTTTTGAGCCTGGGACGCTTGGCGAATGCGGGATCGGAGGCCGGAGTTCACCGTGTCAGTCAGAGTCCCCGTCTGCTCCAGGTACTTGTGGAGGTAGACGCTGGAGGCAAGCACAGCCAGTGTTCCCTCGGCGTTGGAATAGGTGATGGTGGGAACCGATAGGTCGTTCAAGCTGAGAGCGAGGATGTCTCCTGTGCCAACTAGTACAACCTGCTGAGAGCCTTCAACAAGAGCGACAATAGTCGGGAGGTTGACTTGCAGGGTGTCCGGAGCGCTGTCTGCATCACCTGAAGCACACAGGAGGAACGTGTTGATTTCTCTGATGGGGAAGCCTTCAGGCCTAACATCGATGGTCTGGATGACAGTATCGCTTGAGTTGACGTTGGACAGTTTGACGTCCGGATACTGTGCCTGGGCTTCGGGTCTCGTCTGGACGCGGGCTTCCAGCCGGGATTCCTCAAGCGTGGTAGCGCGCACGTCAAACGGGAATGACGCGGATGTGCTGGAGTTGGGGCAGAGTAGTGCGTCGGTGCTGTTGGGACCACCGACCACCTCGAGATTCTCCAAAGACCTCAGACTGACCCGGACCGCGACGCACTCTTCGCCGTAGTTGAATGCCGTGAGAATGACCGTGGCCACTTCGTTGCGCTGCATGTACGCCGGCAACGTGAGCGATACGAATAATGGCTGGAATCCGGTGACGTTCGCCACTTGGGAGACGCCGAGGCCGTTTCGCGGGTGCAGGCATACGGCAGTGCCCTGCCATGTGGTGATTGTGTCCGGCAGCGTCTCGGAGTACACCAAGGAACCATCAGGGCTCACTCTCTTGATCTGCCAGAGCCATGTTTCAGGGAAGAGTGTGCGGACGGAGTTTGACCCCTGGACGACGGGCGAAAAATCGGCTTGCGCAGACTCTGCGATGGGTGCCGGTCCAGCAAATCCGGGGGAGGCACTCGGAGCAAATGCCACTTTGTTCACAGCTCCGAAACCGCCGAAACTGGGATAGTAGCGATTTGTCGATCCCAGGACTTGATTGTTGGGATAGCCAGGGAAAGCACGGTTGGGACATGGCCTCGTCTGGATTGGCAGGTTGGTGAAGACGACAAGACCAGTGTTCTCGAACGAGCTCAGGGAGTCGTAGCTGAAGGAGACTGAAGGGGCCACTGTCTGCCTCCTCCTCCGCACCTTGAGCTGGGAGTCACCCGACTTCTCGTAGGGTTCATTGTAGAGATAACCATCAACGTAGCAGTAGCTCTGGTTGACGAGAGAAGTGCGGGTGTACCTATAGTTGAAGTAGCTTATCTGGTTGAGGAGGGCGTCCCTAGAATTGCGCTGGTTGTAACCATCCAGAAGGGTGACGCTGCTGTCAACAGCTCCGACACCACACAGGGAGTCTCCGGCAGCAGCAAGAGACAGCGACACGTTCGAGCCGGGCAGAGCTTTCTTGGGATCAAACTCCAGCGACACGTTGTTCTCCAGGCACTTTTGGACGCTGAACTCCGCCCAGTCCGAGA

>MG9616496

TGTAGTTGAAGACTGTGACACGTATACCAAGCTGCTCTCCAAGACGAATCTTTGTTGGCATTTCTACGTTCATGTAGAACGGCTTTGCACTTGAAAATGAGATGCTGTTTGACAAAATCCCAAATCCACTGGACTCGTCCATTCCAAATGCACTGATCATCCATTCTGTTGGCACATCAGGAACTGGGATGTTGAAGATGTAGTAACCCAATGGCCCAATGTTGATGTCTTTCCAAAGCCATGAATTGGTATATAGCCGCTGAT

>MG9642660

GCACGGTCAGCGTGCGCACCGATGAAGGCTTGCCTTCTTTGACACCCGAGCACACGTCCTTCGTCCCGAGCATCGCCACCCTAACCTTGAGATCCTTGTGGTCGTAGTTGTACACCGTCGCCGGTATCTCGATCTGTTCCTCCTTGATCACGGAGTAAGGGAGGTTGACTTCGACGAAGAGCTTCTTGAAGACCGGAATCTGGAGAGGATCAAGTACACATATCCCGCCAGATGGGGCCACGCCCACAGCGCTGACCTCCCACGTGGTGATCGAGTCTGGGACGGTCACCTCAAGGCTAGCCTCTCCATGTTCCCGTAGGGTTAGTTGATTGAATATCCACGTCTCTCTAAAGTTTCGCCTTTCTTCACCCTCTAAATCTTTGAAGTTGCTCATGCCCAACACGTCGGCTTCCTCTGCAGATGACCTTCCGACAGCCTGGTTGTCTTCGACATGGATGCAGCACTTGCGAAAGGCTGCGACACATTCTATCGTGTAGAGGGCCACGCCGGCATCCAGATAGCTGAGCAGAGCCTCCTCGCGTTCCGAGCAACTGCGTAGGAGCTGGTCAGGCTGCATCCCCCGCGAGCAGCAGTCTCGGAGCGTCTCGTTTTCGTACTCTTTCACTATGTCCAGCTTGATTTCACGTTTTGCGCGCCGTCTCTCTACCGCTGGTGCGCCGTAGCTGGCCTGTGATGTGAGCAGCACAACGCCCGCATTCGCAAG

>MG9626644

ATCGGCGCAGATGGCATCGACGCCGAGCTGCTCTTCCACGTTCCCGACCTAAGCCCCAGCGTCTTCTACAACCTGTACCTCGATGTGACCGGACAATTCGGCAGCGACCAATTCCAAAACAGATCGTCTGTTCAACTGGAATACGTTACCAATGTCAACGTCGTTGTACAGACGGATAAGCCCATGTACCGTCAAGGAAGCACAGTGAACTACCGCATACTGCTGCTTGACAACAACCTCCTCCCCGTGACGAATCAGCTGGCCAACATTACCATCACGAACCCGTATGGCCAGCTCTTGTTCCAGCAGCAGTACGTCAACTTCACCGACGGCCTCCTTCAAGGCTCCTACGACCTCCTTGCCATTACCGACGAAGGCACGTGGACGATCAGCGTGTCGGCAGGCACAAACTCTGGTTCGGCAACGTTCCAAGTTCAGGACTACGTGCTGCCAAAGTTTTCCGTTACCATCACTCCTGACACCAATGACGTCGTCACGAACCCTACTGTGCTGTACACTATCTGCGCCAAGTATACCTATGGCGAAG

>MG9613521

CATCACTGAAGTTTATTGCTGCGTTTTAAAGCTGGGGCAAGTCGTTCGCATCGAGAAGTTTCGAACACAGTTCGTACTCGCCAATGTTCGGTGCATGAATGGTTGTTAAACGAACGCACGCCATCTGTGTGTTATGTTAATGACAATACCGGTATTCTGCTGGCGAAAAATCACATTTACATTACCGTAATTGCCATAGCCATGAAAAGGTTCGGGCCACTCATAAGAAACAGCGAGTTCTCTCATCGGAAAACTCATTGATGAACCACGCCGCAAGTCTATTCTGTTGTTTATTCTTGGGCTTCGCGGGGAAAACCATCGAGCTGCTGTCGATCAGATACAGGAACTGCTTGGTCTCGAAGAGGTCTTTCTCGATATACGTGGAGTCTTTTCCCATGACAATATACTCCTCGCCATTGGGAATATTGAATGTATTGCAGGTATCACGAGCCTTGATGCGACGTATCTTTCCTTTCAAGTCGTCTTCTTGGCCTGGCTTGAGAACTTGAGTGATGAGGAAAGCCACTTCTATGAAGCCATCCGTCGATACATTGGCTGTCGAATTGCCTCTCCATACGTAGTGAACTCCGTCACAGGCGAATTCTCTCAGAAGCTCACGCTGTTCTTCGTCTTCAAAGTATTCGTTGTCCCGTGTTTTTATGAACCTGTCGAGTGGCTTCTCCGGCGGGCAACCACCTTCCGCGCAAACGCAGACATCGGAGTCGTCACACTTATCATCAACCTTTAGTAGGGGGCTTGTCTTGTCGGGCGAGTAGAATCGTGTGCAAGAAAAGTCTGGTTTGTAGTAAGAGTATGCCTTCACGTAGCTTGACTGCAGTTTACCTGCATTGAATTCTTGTTCGAGAGAGAAATTAACGCAGTGCGTCATGTTTGCAGCAATGAAGGGTACGTAAAAGTCAACATGGCGACTTGTGATAGTATAAAGCTGAATCTTTCTTTCTTTCACGAGCTCATCCAACTCGGTAGCATTGGGCTTGAATCCAGTCAGAAGACCCACTTCCAACATGAACATGCCATCTGGAGCATCCTCGAGTGGCCTTGCACAGGCCTCCATGCGGTAGTTTGGCTTCAGGTCAGATGGTGGACGTGAGGAGGTTTTTTCGACACTTCGAGAAACTCGTGTGAGTATGAGTTCTATGTCCGGTTTCGTTTCCACAAAGTTTGCTGTGATGTTAAACTTGCAAAGCTCACCGTCAAGAACTTTAGCGTCGTATGTGTAGTTAAAGTACAGGATGCCGGTACCGGACCCTTTGACTCTGACGAAGATTTTCTCTCCCGGTCGATCAATCTCGATCTTATTCAGGATGGTGGCGTTATCTCGCTTGATCCTAATATTCTTCTTGAAAATTTTGCTGTTGCTCAAGGTGACCTCGCATGCCAAGTCGAGGTTGTTTTCTTTGGCGTATGCGGCAAACTTGGTGAGCGCTTGCAACACCATCACAGTGTCCTGCGTAGATTGAACAGACCCAGTGGGGCCGGTCCTTTTGCTCAGCCAACGCTGAAAACTTCTTATGATCTCGCTTTGTGCCG

>MG9616283

GCATTTTCCAGAGAGGGAATGAAGGAGGGCTGTATGTGTCCGCCGGTTCCGAGCCACTATCGGTGCAGGCCACGTCATACGCGCTCATGGCACTGCTCAATGCCGGCGAGAGTAGGGACGACATCACGGCCATGGTGCAATGGCTTAACCTGCGAATGAACCCCAGCGGGTCGCTGCGGTCCAGCCAGGATACCGTGGTGGCGCTTCAAGCCCTGTCCAAGTATGCCCTCTACGCACGAGACGCAGACATTGACCTGACCTGTGAAGTGACGTTGAGCGGGGACCGTAACTTTAACCGCACCCTTCGAATCAAGCGAGACAACGCCCAACAGCGCAACAGGATCGAGATTCCAGATAGCAATGACAAGATATTTGTCAACGTCAAAGGGACTGGAAGGGCGACTATGTATTTTGTCACAAGCTACGAGTCCCCTGTCGGTGCGGACCTTTTGTGCAAGTTTGATCTGAAGATAAACTTCACACAGCACAAGGTCAACGTGTCGAAAGCGTTGGAAGGTAAAGGGAGTTTCAAAGAGATTTACTCGATGGAAGTCTGTGCGAGGTCTCTTGAAAAAAAATTGAAAGGCATGGCCATCCTGGACGTAGGTCTCCTAACCGGCTTCAATCCCGTGTTGGCCGATTTGGACAAGATGGTGGCCGACAAACGTGTGGACTCGTACGAGTTGAGCCAGCGTAGCGTGGTATTCTACCTGTCGACCATTCCATCGAACGCCAGCGTGTGCGTCGAGTTTGGCCTGCAGCAGGCATTCGCCGTAGGCAAGTTGCAGTCGGGCTCCGTGAAGGCGTACGCCTACTACGACCCCGATATATCGTG

>MG9616296

GCGGGAAGTGAACTTGCCATCAGGTTACTACATTCAGCAGCAGACTTTGGATGCATACATGCAATCTGGAGCTGTGCGAAACCTTCGGGAAGCTCGTTATGAGGAGAAGAAGGCTGAAATCTACTTTGACTATCTCGATACATCACCCATCTGTGTCAACTTTACTGCCCAGCGTTGGTATCCTGTTGCCAACATGACACGGTTTATTTCAATTCGTGTCTACGACTATTATGCACCGGAGCGGTTCAACGAGTCAATGTTTGAAGTGTACAACCTGTACGCACTCAGCATCTGTCACGTGTGTGGCTCCTACCAGTGCCCATTCTGCCCAGTGTTCAAC

>MG9625519

AAAGTTCACATGCTCAATGGGTTAAGCACACATTTTGGTTTTCAGGTGTAAACTCTGAACAAGTGTATTTTTTTGCTTACCACAAAAGCGAATAAAGCTTCAACTTAATAATTATACGCTTCTTTCAAAAGACAGTTCTGGCTCTTCTATGTTGTTCCTTAGTGTTCGTTTTATAAAAACTGGAGTGCATCGAGATTCAACTTGGAGCACATCCCAGCAACTTGCAAGTTCCGTCAGTATGAAAGTTTTCTGCACGTTTATGTCCTTTTGCCAGTCAGGTCTGAGGAGCAGGCACTGTAACAGGCATGTTTGATTCAGGGCCATCTGGGAAGTCCAAGTCTTGTTCAATATTTCGGAAGTTTCCAAGATGAGTTCTGGCTGAACGAATATCTTCTTGCATTATTTTTGACTGCAGGGGCATTTGTGAGTCACTAAGGTCTTGTTGATCCCTCTCACAACTTGGAATTGTGTAGTTCCTTAGTACAATGACACCAGGATCATAGTAGTCCTGCAATACAACAGCTGCAGGAGCACTGTCTTGCACTTCAAACTCCTGGGTCAACATTAAGGTGATGCACATCCTTCGATTACTTAGCTCATCAAAGTACAAGTTTATTTTGTCAGGCTCTTCTTCAGTCTTCCTAACTTCCAGGCCATCAACAACCTTGCTCAAACGGAACCCGGTTTGCAGATGGACTTGAATGATCACCATTCCAGACATGCCCCAGG

>MG964726

TGACGGCGTACGTGCTCACCGCTCTTCTGGAGGGCGGCCTTGCGGACGAGAAGGTCGTTGAGTCGGCGCTCCGCTGCATCAGCGCCCAGCGAGACCCCAGTGCCCACAACTTGGCCCTGTCCGCCTACGCCGCGGCCCTGGCGGGACATGAGTCTGCCAAAGATTACCTCGAGAAATTGGAGTCCATCGCAGTTCACAAAGGTGCCCTGACCTACTGGAGCAACGCGGGCAAGAAGGGTCCATCAGCGTCGGCCGACGTGGAAACGGCGGCGTATGCAGTGCTCGCCTACCTCAAGCTGAACGCCCAAGAGAACCTGAGCAAGGCGCAGCCCATTGTCCGCTGGATGGCGACCAAGAGAAACAGCCGCGGAGGCTTCCCATCCACACAGGATACAGTTCTCGGTCTCCAGGCTCTGTCGGCTTTCGCTACTTACGTTAGCAAAGATCCCGTTGACATCTCAGTTAAGGTCGACGGTACTGACGTCAGTGAGTCGTATAACCTTAAGGAGGACACCAAACTGGTCGTACAGGAGAAGAAGGTTGTCAGCTTGCCCAACAAGCTCACTTCAGAAGCAACAGGTCCTGGATGTGCCCTTATATCGGCGACTCTGAAGTACAACGTTCACACTGCACCGAAGAGCGAGGGTTTCGAGCTCACAGCCACTCCATCTCAAGAGGCGTCTGACTGCAATGACCACAAGCTCAAAATCTGTCTGAGGTTCGACGGTGAGCAACCATCCAACATGGCAGTGGTGGAGCTCAAGCTGGTATCGGGCTACACTCCAGATGAGGATCACATCTTTGGACTCTACCGTGAGAAAGACGTCAAGCTGAAGAGGCATGAAGTTGAAAAGAACCAGGTTAACTTTTACTTTGAAGAAATAACGTCCGAAAACAAGTGCTTCGACGTTCGCGTTCACCGCGAGTTCGCTATTGAAGATGCCAAACCTGCCACAGTTAAAGTCTACGACTATTATGAGCAAGAAAACAGCAACTCCGTGCCCTACTCTCTTGTGGCGTCCTGCTAGTGAACATCGGCCATCAGTTTTTCTTACTCTCCCTTTTTTTTATGTCTCTTTTTTTCGAACGAACGCGCCAAGACAATAAGAGTCACAACATACCATTCATTCAATGCGCATTTATCCTTTGGTTGCCAATTTTCTTTTTACCAGAATCATGCTTTCTCGCTCCCTCGGTAGTGTTCAACACCAAAGGCTTTTTATACCGGTCACACTTCTTGAGAAGGACATACGTGCCATGTCGCGAACGAAAAAAAAAAGATTAATGGCTTCCACAGCGCACATCGCGAAGAAATCAAGACAATGGTCACATGATGTTTTATCTTACAGCCTCGATATTGGGTGCGGACGACTGAGGGAGCGCTGCGCAGTGTGTGCTTTTGTGTGCGTGAACAGAGCCGTGAACTTTTGACGTCGCCATGTTGTTTCCCGTAGCGCGTCTTATCCAGCTGAGTGGAAGAGTAGCTTTATATATTTTCTACTTGCGAGAAAAGCGCGTCATCGTCTGCGAGGCTTCACCGTTATAAATAGTGTCATAAAGAGTGAATAAAACTAATTTTTTTCAAAAAAAA

>MG9621638

CCGTGCTTTAAACCGCGCTCCGCCGCTCCGGAGGGATGCCCTCGCTGGCGCCGCGAGAGCGTAGGGTGGTCTGTCGTAGATGTTCTTCCGGCATGGCCTGGTTTCAAGCGTCAAGTCTGACATGACCACCACCCCTGATTCGTCGAAGGCGGTGATCGAATCGACGTATTCAACATTTGAAGATCTCGGTCCATTCCAGATGTGGCGCTTGAAATGTTGCGGATTCTTCGCCAACTGCTTGCGACAGTAGTCGTACGACGCCTGCTTCGGCCACGTGTAGCGGGTGATATCCAGACGCTTCAAAATGTCATAAACTTTGTCCTTTGTCAGTTGGTTATCCTGCTTCAGGAGGTGAACACTCTANNNNNNNNNNNNNACACCACAGAAGGACCGAGGAGAACCATTCACATGGATGGCAGCAGATGTGGCAGGCTGTACAGTTTCAGATCCGAATCTCATGGTTACGTTGTTCTGAAGGCACTTCTCCACTTCGAACTGCTCGGAGTCAGCGATGACCTCGCCGTCGGGGCGGACGTAGAAAGCGAGCACTTTGACGCGTGGCACGTGGCTGAAATCTGGCTCGAGCTCGAACTCGAAGCTCCCTGNCGACACGCTGCCCTCGGCCATGTTGGTCGGAAGCGTCTCATTCAGGTCCTCCTCCACCAGGAAGCTGTCGTCAACAGAGAGCACCTCTTCCGGCTTGAAG

>SG961768

AACAGATCCACGTGAAGGCACGCAGCACCAGCGGCGAAGGGGACGAGGTCAAAGTGGAGCTGAATGTCCGGCCTCCGGGAGTTCAGAGAACACGATCGTTTGCCGTCATCTTGGATCCCGAAAACCCTAAAAAGCGAGGCACGAGGAACATCCAACAGGAATACACAGAGACCTTTGGTACAAATGGAACGCAGGTGNNNNNNNNNNNNNNNNNNNNNNGTGATTTTGCTTTGCCAAACACAGAGCGCTGCGAAATCGACATCGTCGGAGACGGAGTGACTGCCGTTCTTCAATCTATAATCAAGAAGCCGGACCAAGCGTTCATTTATCCCTCTGATTGTGGAGAACAGACCACGGCCAAGCTGATGCCAGTACTGTACGCCTACGAGTTCTTCAAGACCACCAACCGCATCAGCATTGCTGAAGAGAACGACGCTCTTGACTATATTCGAAGAGCCTACAACCAAATACTCAAGTACAGGAAACCGGATGGCTCTTTCAGTGTTTTCGAATGGAGTAGTGCTAGCCCATGGCTGACGGCTTTCGTCATTCGCAACTTGTGCGAAGCTACGAAATCCGTCATGATTGATGAAAACGTCATCCGAAGNNNNNNNNNNTATATAACTAGCAGGCAGCAAGGAAATGGGGGCTTCCATGAATATAGCCTCAGCGAACTAGTGCTAAATTTCGGGCACCCATCAGCGCTGACTGCCTTCATTCTCATCACCTTCGAGGAGTGCGCTGAAGGAGGATACTCGGTTTCGCAAACCTCGAGGGCGAGAGCAGCGGCGTTTCTGGAGCGGAATTTGCACCGCGGAGACTCACCGGGAGCGTTGGCGCTGGCTGCCTATGCCCTGTCCTTGGCCAACAACACGGGGAAAGATGGACTGATCCAGTGGCTGATGGAAAGCGTGCGCTATGACCAAGTCACAGACGACCGACACGTTCCGGCGAGCAGCGAACTGCTGTCTGCACAAGCAACCGCATATGCACTCATGGCGCTCATCAGGGAGAACGCACAAAGCGAGATCATAAGAAGTTTTCAGCGTTGGCTGAGCAAAAGGACCGGCCCCACTGGGTCTGTTCAATCTACGCAGGACACTGTGATGGTGTTGCAAGCGCTCACCAAGTTTGCCGCATACGCCAAAGAAAACAACCACGACTTGGCATGCGAGGTGACCTTGAGCAACAGCAAAATTTTCAAGAAGAATATTAGGATCAAGCGAGATAACGCCACCATCCTGAATAAGATCGAGATTGATCGACCGGGAGAGAAAATCTTCGTCAGAGTCAAAGGGTCCGGTACCGGCATCCTGTACTTTAACTACACATACGACGCTAAAGTTCTTGACGGTGAGCTTTGCAAGTTTAACATCACAGCAAACTTTGTGGAAACGAAACCGGACATAGAACTCATACTCACACGAGTTTCTCGAAGTGTCGAAAAAACCTCCTCACGTCCACCATCTGACCTGAAGCCAAACTACCGCATGGAGGCCTGTGCAAGGCCACTCGAGGATGCTCCAGATGGCATGTTCATGTTGGAAGTGGGTCTTCTGACTGGATTCAAGCCCAATGCTACCGAGTTGGATGAGCTCGTGAAAGAAAGAAAGATTCAGCTTTATACTATCACAAGTCGCCATGTTGACTTTTACGTACCCTTCATTGCTGCAAACATGACGCACTGCGTTAATTTCTCTCTCGAACAAGAATTCAATGCAGGTAAACTGCAGTCAAGCTACGTGAAGGCATACTCTTACTACAAACCAGACTTTTCTTGCACACGATTCTACTCGCCCGACAAGACAAGCCCCCTACTAAAGGTTGATGATAAGTGTGACGACTCCGATGTCTGCGTTTGCGCGGAAGGTGGTTGCCCGCCGGAGAAGCCACTCGACAGGTTCATAAAAACACGGGACAACGAATACTTTGAAGACGAAGAACAGCGTGAGCTTCTGAGAGAATTCGCCTGTGACGGAGTTCACTACGTATGGAGAGGCAATTCGACAGCCAATGTATCGACGGATGGCTTCATAGAAGTGGCTTTCCTCATCACTCAAGTTCTCAAGCCAGGCCAAGAAGACGACTTGAAAGGAAAGATACGTCGCATCAAGGCTCGTGATACCTGCAATACATTCAATATTCCCAATGGCGAGGAGTATATTGTCATGGGAAAAGACTCCACGTATATCGAGAAAGACCTCTTCGAGACCAAGCAGTTCCTGTATCTGATCGACAGCAGCTCGATGGTTTTCCCCGCGAAGCCCAAGAATAAACAACAGAATAGACTTGCGGCGTGGTTCATCAATGAGTTTTCCGATGAGAGAACTCGCTGTTTCTTATGAGTGGCCCGAACCTTTTCATGGCTATGGCAATTACGGTAATGTAAATGTGATTTTTCGCCAGCAGAATACCGGTATTGTCATTAACATAACACACAGATGGCGTGCGTTCGTTTAACAACCATTCATGCACCGAACATTGGCGAGTACGAACTGTGTTCGAAACTTCTCGATGCGAACGACTTGCCCCAGCTTTAAAACGCAGCAATAAACTTCAGTGATGGATCACCGAAT

>SG962732

CCTGCTGCAGGCCAAACTCGACGCACACGCTGGCGTTCGATGGAATGGTCGACAGGTAGAATACCACGCTACGCTGGCTCAACTCGTACGAGTCCACACGTTTGTCGGCCACCATCTTGTCCAAATCGGCCAACACGGGATTGAAGCCGGTTAGGAGACCTACGTCCAGGATGGCCATGCCTTTCAATTTTTTTTCAAGAGACCTCGCACAGACTTCCATCGAGTAAATCTCTTTGAAACTCCCTTTACCTTCCAACGCTTTCGACACGTTGACCTTGTGCTGTGTGAAGTTTATCTTCAGATCAAACTTGCACAAAAGGTCCACACTGACAGGGGACTCGTAGCTTGTGACAACGTACATAGTCGCCGTTCCAGTTCCTTTCACATTGACAAATATCTTGTCATTGCTGTCTGGAATCTCGATCCTGTTGCGCTGTTGGGCGTTGTCTCGCTTGATTCGAAGGGTGCGGTTAAAGTTACGGTCCCCGCTCAACGTCACTTCACAGGTCAGGTCAATGTCTGCGTCTCGTGCGTAGAGGGCATACTTGGACAGGGCTTGAAGCGCCACCACGGTATCCTGGCTGGACCGCAGCGACCCGCTGGGGTTCATTTGCAGGTTAAGCCATTGCACCATGGCCGTGATGTCGTCCCTACTCTCGCCGGCATTGAGCAGTGCCATGAGCGCGTATGACGTGGCCTGCACCGATAGTGGCTCGGAACCGGCGGACACATACAGCCCTCCTTCATTCCCTCTCTGGAAAATGCCGCCGAGCCGGCGCAGGGTGTCGTTCTTTGCGGGGCTTTTGCCCAAGGAGAGCGCGTACGCTGCCAGCGAGAGCACGTAGGGCGAAGTGTTTGGATTCAGCTGTCCCTCGATGAACCCTGTTGCTTTTGCGAAGGACTCTGTCAGGCCGGCCACCTGAACGCCTTCTCGATCGCATTCTTGCAGGGTGAGAAGGGTGTACGCGGTCAGTGGAACCGGTCCATTCACACCGCCCAGGAGGTCACCGTGGATTAGGTTGGATATGTCGTGGAAACTGCCGTCCTGCTTCTGCTGGGTCAAAATGTAACGGAGCCCACTTGTGACGACCTTCTCGTCGATGAGGATAGACTTCCGGGCTTCGCACAAAGTTCTCACCACGAATGCTGTTAGCCATAGACTGGACCTGTAATGATTCCAAACCGCGAAGGACCCGTCAGGTTTCCGGAAATTGAGGATCCGCTGGTAGCCACTGCTGATGAAGCCCAGCGCTCTGTCCTCGTCCACGGGCGATATGCGGCTGGCGGTCTTCAGATACTCGTAGGCGTACAGCGTTGGTGCCAGGCCGATCATGGTCTGTTCACCGCAGCCATGTGGCATCCTCAGAAGCGTTCCCGGGTTCTTTACGGACGCCTCCAAAGCAGCGCCCATGTCATCACCAACGATGTCGATCTCACAGCGTTCGGAGCCAGGAATCACTTCCTTAGGGGATCG

>SG9638685

TGGCGGTCTTCAGATACTCGTAAGCGTACAGCGTTGGCGCCAGGTATATCATGATCTGTTCACCGCAGTTTTTTGGCATCCTTTTCAAAAGATCCCCCGGGTTCTTTACCGACGCCTCCAAAGCAGCTCCCATTTCATCACCAACGATGTTGATCTCACAGCGTTCCGAGCCAGGAATCACTTCCTTGGGGGATCGGTCAGGCCGCTTTATCACGACGAACTGCTTGCCCCCAGGCGCGAAACTCTCTCTGTAGCCGTGCGTGGGGCTTGCGGCATTCCGCGCTGGTCGTTTCTGTGTGTTTTGAGGGTCGATAATGAGATTGAAGCTCTCATTCTTGCTGACACCAGGTGGCTCTACCCTGAGCATCACTTTGACAGAG

>SG961018

TTTTTTTTGAAAAAAATTAGTTTTATTCACTCTTTATGACACTATTTATAACGGTGAAGCCTCGCAGACGATGACGCGCTTTTCTCGCAAGTAGAAAATATATAAAGCTACTCTTCCACTCAGCTGGATAAGACGCGCTACGGGAAACAACATGGCGACGTCAAAAGTTCACGGCTCTGTTCACGCACACAAAAGCACACACTGCGCAGCGCTCCCTCAGTCGTCCGCACCCAATATCGAGGCTGTAAGATAAAACATCATGTGACCATTGTCTTGATTTCTTCGCGATGTGCGCTGTGGAAGCCATTAATCTTTTTTTTNNNNNNNNNNNNNNNNNNNAAGCCATTAATCTTTTTTTTTTCGTTCGCGACATGGCACGTATGTCCTTCTCAAGAAGTGTGACCGGTATAAAAAGCCTTTGGTGTTGAACACTACCGAGGGAGCGAGAAAGCATGATTCTGGTAAAAAGAAAATTGGCAACCAAAGGATAAATGCGCATTGAATGAATGGTATGTTGTGACTCTTATTGTCTTGGCGCGTTCGTTCGAAAAAAAGAGACATAAAAAAAAGGGAGAGTAAGAAAAACTGATGGCCGATGTTCACTAGCAGGACGCCACAAGAGAGTAGGGCACGGAGTTGCTGTTTTCTTGCTCATAATAGTCGTAGACTTTAACTGTGGCAGGTTTGGCATCTTCAATAGCGAACTCGCGGTGAACGCGAACGTCGAAGCACTTGTTTTCGGACGTTATTTCTTCAAAGTAAAAGTTAACCTGGTTCTTTTCAACTTCATGCCTCTTCAGCTTGACGTCTTTCTCACGGTAGAGTCCAAAGATGTGATCCTCATCTGGAGTGTAGCCCGATACCAGCTTGAGCTCCACCACTGCCATGTTGGATGGTTGCTCACCGTCGAACCTCAGACAGATTTTGAGCTTGTGGTCATTGCAGTCAGACGCCTCTTGAGATGGAGTGGCTGTGAGCTCGAAACCCTCGCTCTTCGGTGCAGTGTGAACGTTGTACTTCAGAGTCGCCGATATAAGGGCACATCCAGGACCTGTTGCTTCTGAAGTGAGCTTGTTGGGCAAGCTGACAACCTTCTTCTCCTGTACGACCAGTTTGGTGTCCTCCTTAAGGTTATACGACTCACTGACGTCAGTACCGTCGACCTTAACTGAGATGTCAACGGGATCTTTGCTAACGTAAGTAGCGAAAGCCGACAGAGCCTGGAGACCGAGAACTGTATCCTGTGTGGATGGGAAGCCTCCGCGGCTGTTTCTCTTGGTCGCCATCCAGCGGACAATGGGCTGCGCCTTGCTCAGGTTCTCTTGGGCGTTCAGCTTGAGGTAGGCGAGCACTGCATACGCCGCCGTTTCCACGTCGGCCGACGCTGATGGACCCTTCTTGCCCGCGTTGCTCCAGTAGGTCAGGGCACCTTTGTGAACAGCGATGGACTCCAATTTCTCGAGGTAATCTTTGGCAGACTCATGTCCCGCCAGGGCCGCGGCGTAGGCGGACAGGGCCAAGTTGTGGGCACTGGGGTCACGCTGGGCGCTGATGCAGCGGAGCGCAGACTCAACGACCTTCTCGTCCGCAAGGCCGCCCTCCAGAAGAGCGGTGAGCACGTACGCCGTCAGCGCTCCCGGGGCAGTGGAGTTAACCTTGCCCTTCAAGCCGGAGCTAAGAACAGTGCCGATGTTCTGGAAGCAGCCGTTGGTTTTCTGCTTCGTGATGATCCACTTTATGCTCTCGTTGAGGTTTCCGGCGTCAATAGGAATGTATTTCTCAGCCTGCTTGAAGGACTTGACTACAAACGCCGTGAGGAACATGCTGCCCGAGTTGTCGCGGTTTCCAAAGGCGCTGTACGAACCATCGTAGTGCTTGTACTTCTGCTGGCGCTGGTAGCCTGTCTTGAGATTCTGCACAGCCTTCCTTTCGATGTCTTCCTGGTTCTTGCCCGTAGCCTTAAGGTAGTCCAGGACATAGACGTTTGGCGTAAACTTTACCATGTTCTGCTCACCACATCCCGTTGGAACTTGCACCAGTGAGTCGAGGTTCTTGATTGCTGGGCCCATAATATCTCCTGTGACTTGAACGTAGGCACGGGCCGATCCTTCCACCAAGTCTTCAGGAAGCACCAAGTCAAATTCGTTCTTTGCGCCGCCTTCTCCAGCGTCTTTAGGACAAACAAAGACACTCTGCGTCTCTTCCTTAGGGAAGCCTTCCGCCTCGATGATGAGCGGTCGTGTGACTGCGTCCCTGGCCACCACCTTCTCAGTTGGCTGCTCTCCACATGCGGCATCGCTCTGCGATCCGGCCGCCGACACGGTCAGGTTCACCTCTCCGATAGTTTGTGGCCGGATCTGGAACTTGTGCACTTGGCTCTTGCTGCCGCACACGCAAAGCGTCGTCTCAGACTCGCCCTCAATGTGGAAGTCCGCGGATTCAGCCAGCTTCAGGTCGACCGGCAGGCACTTTTCGAGGTAGTTGAACACGGACACCTTGACGGGCACCAGTTCTCCGCGCACCACAGAGTAAGGCAGGTTGAACGAGGCGAAGAACGGCTGGAAGGCCTTGATCTTTGCCGGATCTGAGATGCCGATTCCGTCCTCTGAGTTGATGCACACAGTGCTTCCCACCCACTCGGTGATGGTGTGCGGAATCTTTTCTTTGAAGTTCAGTTCGCCGTGCTCATCCAGCTCCTTCAGGTCCCACAGCCACGTCTCGGGGAAGTAGGTGCGCACTTCAACGGCCGACTTGGCCGGGATGTTGGCCGATGGTGCGCTCTGCGCCACAGAGTCCAGGGCGACGGGCACGCCGGGCAGGCCACCNNNNNNNNNNNNNNNNNNNNNNNNNNNNNNNNNNNNNNNNNNNNNNNNNNNNNNNNNNNNNNNNNNNNNNNNNNNNNNNNNNNNNNNNNNNNNNNNNNNNNNNNNNNNNNNNNNNNNNNNNNGTTTCAAGCGTCAAGTCTGACATGACCACCACCCCTGATTCGTCGAAGGCGGTGATCGAATCGACGTATTCAACATTTGAAGATCTCGGTCCATTCCAGATGTGGCGCTTGAACTGTTGCGGATTCTTCGCCAACTGCTTGCGACAGTAGTCGTACGACGCCTGCTTCGGCCACGTGTAGCGGGTGATATCCAGACGCTTCAAAATGTCATAAACTTTGTCCTTTGTCAGTTGGTTATCCTGCTTCAGGAGGTGAACACTCTTGTCTACGACGCCAACACCACAGAAGGACCGAGGAGAACCATTCACATGGATGGCAGCAGATGTTGCAGGCTGTACAGTTTCAGATCCAAATCTCATGGTTACGTTGTTCTGGAGGCACTTCTCCACTTCGAACTGCTCGGAGTCAGCGATGACCTCGCCGTCGGGGCGGACGTAGAAAGCGAGCACTTTGACGCGTGGCACGTGGCTGAAATCTGGCTCGAGCTCGAACTCGAAGCTCCCTGTCGACACGCTGCCCTCGGCCATGTTGGTCGGAAGCGTCTCATTCAGGTCCTCCTCCACCAGGAAGCTGTCGTCAACAGAGAGCGCCTCTTCCGGCTTGAAGGTCACATCCAGAACCTTGTCCTTGAGGATCTTGCCGCGAGCTATCACCTGTAAATGGAACTGCTTCTCTGAGTCGGGCTGACCCGTGTAGCGGAGGCGCACGGGGTGCTTGCCCGAGCAGCGCAAGGGTCCCTTGGACGGCTCGATCTGGATGAAGTTGTTGCTGGCCGAGTACCAAGCCTGGAGGTACAGAGTGCTCTTGGGCTGGTTGATCTTCACGCCGTAGGTGTCGTACTTCACAGTCTCGTAGTTCATCGCCACCGCCTCGACGCTGATGGTGACGACAGTTGTCTTCAGGGGAGGGATGGTGAACTTGATGATGCCTGACTCGTCCGAAGTGTAGTTCTTGCAGCTGAGACGGCGGTCGGTGCGCCACCACCGTGGCTTGATCTCCTCAGCCTGTGACAGCAGACAAAGTTGCACCAGCTCGCCGCCCACAGGAACGCCGTCAGGCTTCTTCACCAGGAGCCTGCCGTAGAAAGGCATGGTGGGCTTGAAGTAGTTCTTTCCGCGGTCTCCTTCCAAAAAGTTGAGCTCAAGCGGGTTGAAGCTGCGGCTGATGTAGTTCGTCTTGTTCATCGTGATGCCCGTGCCGGTCTCGTTCACTTGTGCGACGAGGTAGATGCGCTTGTAGATCTCGTAGTTCTCGTTGAAGCGCAGCAGGCTCGTGTTCACCGTCATATCGAAGCAGCCGTTGATGGGTCCTGTGTGGTTGATCTTTGGGTAGTCGTCCTTCTCCCAACTGTAACGCTCGTATGTCACGTTCACCGTCAGGGTGCCGTCCACTGGCTGTCCAAAAGTATAGTGGGCACAAATCTTCCAGGTGATCTCCTTTGCATCAGCCAAGACGTAGGATGGAGGCTTGATGGTCACCTCAAATTTGGGAAGTACGTATTCGTTCACCTCAAAATGCTGCCTCACGGTTTGCGTGGGAAGTTCGACGACTATCTGCCACAGACCCAACTCAGGCTCTTCTGTAAGCTGAAAGTCTCTCTGGACAATGCCTTTTTCAAAAGACACGTCGTTCCACTGAGCTATTCTTACATCGCTGGGACTTGTCACGTAAATAGTTGCCTTCACATCGGTGACTGGTTTCAGCTCATTGTTGATAGGGAGGACGCGGAATTGAACCCTCTGTCCAGGCTTATAAAGTGCCTTATCGGACTGAACCAGGATCGTGTTCTTGGACTTCTGGAAGTCGATCTCTTTCCGATCGCCGAAGACATAGTCTCCAAAGGTTCCGTTCACTTCGATCTTGGCTTGGCTGTCGAGGTGCTCGGGCACTCGGAACGGCAGAAAGGTGCTTTCACCGTTCTTGATGTCGTACTCTTGTTCTGCCAAGACGATGCTGTCGTTGTTGTACTTGAGCAGGCGGACGGTGACCTTGCCATCCTCCTTGACGTCTGTGAGCGTTAGCCGAAACTGTTCGTCCGTTTCGCTGCGCAGGATCTTCGGCGCCGTGAAGATGTACCCACTTTCAGCAAGGCGCGATGCCAAGGCACCAAGGACTAAAATGAAGGTGCCCCAATGCATTCTAGCTCTGGCCACCGGCACCTCTCGTCACCGTCTGTGCTAGTTTTCTTGCGTCTAAGCCAGCCTTAGACACCTTTCCGTAGACTGTACAGCACAGCTTTCTCTGCTGCGCGCGCTAAGGCCGGTTCCGGGAGCTTTTTCTTGCTACCCTTCTTGTCTCGCTCCTGGCTTGACGTCGCGTGGCGAAGCAAAACACAACACACGCGCTCTAAGGCGGCGGCGTTGGTGCGCAGAGAGTGGTGCGAGACGAGGCCGACAAGACCCTCCGACAGCCCAAGCCCGAACTGATCCGGCGGC

>SG9633055

GCTCCCATTCCTGAAATGTCGCCTTGTGGAACACTCGTGCACAAAATGCCGTCAGCCAAACACTCGGCTGACTGTGCCATCGAAACACCTGGAAGCTTCCATCCTCATTCTGATAAGTCAGCTGCCTTTGGTATCCCAAGTTCAGATATTTGAATGCCTGTCTTTCCAAATGCATGTCACGTTGGTTTGTCTGTCGTAGATAGTGCACCGTGAACAAATTTGCCCCAAAGCTGAACATGTTTTGTTCCCCACAGTAAAACGGCTTTGACAACAGGCTGCTGGAATTGACAGGCATAGTTGGGAATGCTGGACCAAACACATCACCAACCACAGATAAAGTGGCCTTGTTACTGCCGAACACGTACAAGCGATCTTGTCGGAAAGGCAAGATGGGTGACTCAGTAATGTTTGTGTCGAGATACTTTATCAAGTAGGCGCCCTGAGTGAGGTCCAGAACCATGGATGTGTGACGGTATTGAGGAATGCCATCAGGCTCGACAAGAAGAGAACGCGTCACCATGTCCTTTGCAATCTGCGTCTTGGCAAGAACAGTGACTTCAATTGTTC

>SG9638439

TGCTGCGCATGTTTCCGTCATTGGAGACATCGTCGGTACGCCCCTGGAAGGAGACGTGGAACCAGAAGAGTTCGGGTTCTCAAGCGCCACAAAATCCGGGGAGCATGCCATGTTCAGCTTCGCCTATCAAGTTATACGGCTCACCTATCTGCGCCTCACAGACCAGCTGACCCGCGACATTGCGAAGCCCATCTTTGAGAAGTTGAACAAAGCCTACGTTTATCAGAGCTCTTACTTCAAGAATGGGGCATTCACCATGTTCAAGAAGGAGCCTAGTGTTTGGCTGACTGCATTCAGCCTACGGATGTACATGCTGACGGTGTTTCCGGACTGGGAG

>SG9641852

ATCAAAACATAAGCTGTCAGTGCAGGCCCACTTCCAGCGCCACTCTGCATTGGCTTGTAAATGATCTCTCCAGGTTCATTGAATGAGCCATCACTCTGCTGACGTGCTACCAGCCATGCAGTAGCATTTTCAAGTACTTTGGGGTCAATGACAGTGTACGGAGCAGCTTGGGCAAAGCTCTTGAGCACAAATGCTGTCAACCATGTACTGCCACTCTTGTCTGTATTGCCAAAAGCAC

>SG966037

CCAGCGCAATTCTAGGGACGCCACGGTTATCTCTACAACGAACCCTACGAGAAGTCGGGTGACTCCCAGCTCAAGGTGCGGAGGAGGAGGCAGACAGTGGCCCCTTCAGTCTCCTTCAGCTACGACTCCCTGAGCTCGTTCGAGAACACTGGTCTTGTCGTCTTCACCAACCTGCCAATCCAGACGAGGCCGTGCCCCAACCGTGCTTTCCCTGGCTATCCCAACAATCAAGTCCTGGGATCGACAAATCGCTACTATCCCAGTTTCGGCGGTTTCGGAGCTGTAAACAAAGTCGCATTTGCTCCGAGTGCCTCCCCCGGATTTGCTGGACCGGCACCTATCGCAGAGTCTGCGCAAGCCGATTTTTCGCCCGTCGTCCAGGGGTCAAACTCCGTCCGCACACTCTTCCCTGAAACATGGCTCTGGCAGATCAAGAGAGTGAGCCCTGATGGTTCCTTGGTGTACTCCGAGACGCTGCCGGACACAATCACCACATGGCAGGGCACTGCCGTATGCCTGCACCCGCGAAACGGCCTCGGCGTCTCCCAAGTGGCGAACGTCACCGGATTCCAGCCATTATTCGTATCGCTCACGTTGCCGGCGTACATGCAGCGCAACGAAGTGGCCACGGTCATTCTCACGGCATTCAACTACGGCGAAGAGTGCGTCGCGGTCCGGGTCAGTCTGAGGTCTTTGGAGAATCTCGAGGTGGTCGGTGGTCCCAACAGCACCGACGCACTACTCTGCCCCAACTCCAGCACATCCGCGTCATTCCCGTTTGACGTGCGCGCTACCACGCTTGAGGAATCCCGGCTGGAAGCCCGCGTCCAGACGAGACCCGAAGCCCAGGCACAGTATCCGGACGTCAAACTGTCCAACGTCAACTCAAGCGATACTGTCATCCAGACCATCGATGTTAGGCCTGAAGGCTTCCCCATCAGGGAAATCAACACGTTCCTCCTGTGTGCTTCAGGTGATGCAGACAGCGCTCCGGACACCCTGCAAGTCAACCTNNNNNNNNNNNNNNNNNNNNNNNNNNNNNNNNNNNNNNNNNNNNNNNGACATCCTCGCTCTCAGCTTGAACGACCTATCCGTTCCCACCATCACCTATTCCAACGCCGAGGGAACACTGGCTGTGCTTGCCTCCAGCGTCTACCTCCACAAGTACCTGGAGCAGACGGGGACTCTGACTGACACGGTGAACTCCGGCCTCCGATCCCGCATTCGCCAAGCGTCCCAGGCTCAAAACAGCTTCCGGTCCTCCGACGGCTCATACGCTCAGTTTGGCTCTAGCGAGTTCCCGAGAAGCGTGTTCCTCACCGCCTTCGCCGTGAAAGCCCTGAGCGCCGCCAAGGAGTACCTGGGCACCAGCGTCGATGCCGACATCGAAACTAGCGTGCGCTATGTCCTGCAGCACTGGAACGCTGCCACCGGTTGTTTCGTTGAGAACCAGCCCGGCTCGTCACCCTTCGGCCCCCAGACAGCCCCCGACTTCACCGCGGCCATTGGCGTCATGCTTCTGGAGAGTGGCTACAACTACGAGAACATCACGAACGGTGTCCTCCAGTGCATTGACGCCAGCAATCTGCCTTCCAACCACACGACTGCGCTGAACGCCTACTTTTCGGCGCTCGCTGGACGCACGGACCGAGCTAACAGCGCCTTGGACACGCTTCTGAGCGAAGCTGATAAAAGCAGTGGCTTGACGTCATGGAGCGGCGATGGCCTGACTTACGGCTCGGCAGACACCGCTGGTTACGCAGTTCTCACTTTGAAGTTGCTCGACCGAAACCTGGGAGAAGCGCTGCCAATCGTTCGTTGGCTCATGCAGCAGACCTACGCTCGATACACTTTCTCCTATTCCGAGGTCTACACCGTCGCCATCCAAGCCCTGACCCAGTACTCGAGCGTGGCATTCTCGAAGAACACAAACTTGACTATGAACGTTGCTGTCGACAGTTCATCCCCCGAAACGGTCTCTTTCCCGATCTCTGAGCAGAACAAACTGCTATACCAGGAGCGTCTTTTGAACAGAAGCGATTCGTACAGCTTCAAGGCATCTCTTGCTGACGGCAGCGCAGGGTGCGCCGCTTT

>SG9612694

CGCTCCGAGACTGCTGCTCGCGGGGGATGCAGCCTGACCAGCTCCTACGCAGTTGCTCGGAACGCGAGGAGGCTCTGCTCAGCTATCTGGATGCCGGCGTGGCCCTCTACACGATAGAATGTGTCGCAGCCTTTCGCAAGTGCTGCATCCATGTCGAAGACAACCAGGCTGTCGGAAGGTCATCTGCAGAGGAAGCCGACGTGTTGGGCATGAGCAACTTCAAAGATTTAGAGGGTGAAGAAAGGCGAAACTTTAGAGAGACGTGGATATTCAATCAACTAACCCTACGGGGACATGGAGAGGCTAGCCTTGAGGTGACCGTCCCAGACTCGATCACCACGTGGGAGGTCAGCGCTGTGGGCGTGGCCCCATCTGGCGGGATATGTGTACTTGATCCTCTCGAGATTCCGGTCTTCAAGAAGCTCTTCGTCGAAGTCAACCTCCCTTACTCCGTGATCAAG

>SG9614003

TACAACAGAGGAGAACCGTACATTGGTGAGTTGTTGGTATACCATGGCAACAGCAGCACCACAAGACAATGGCACAGGATCAGCAGAGCCCCCTAGTTCTGTCAATATCTTTTGGTGCTTCTTCTACCACAGATTGAATATTCTATATTCAGTACTTCTCATGATAACTTGGTATCTTGCAAATACTTTAAAAAATCTATATTTAGAATGAACTTAAACACTTCTACCAATTCTTGAATTACTCTTAACATGCTTCAAGTTTTTTTTTTTTTTGGGAA

>SG9634559

GGCCAGACAGGTTCCTTCGCTCATGCTCGAAGAGGGCGGGCATCCTGAGGAAGTACATGGAGCAGGGTAGCGCCAACATCACGCAGGATTGCGTGGAAGCCTTCGAGCGGTGCTGCGACATTGCTGAACACGACGCCGCTCAAAGATCCTCATCCGCTGGAGTCGATGAACTCTCAGACGAGTATCTTGGAGTACACAACCAAATTCGAGACGACTTTCGTGAGACGTGGATATTCCATAGCATGACAATTAGGGACGACGGCACCGCCGAATTCTCGGCCACTTTGCCGGCGTCGGTCACCACATGGGAGGTGAACGCTGTGAGCGTGTCGCCCAGCGGAGGAGTATGCGCGGTGGAACCTCTCGAAATCGTGGCGACGAAGAAGTTCTTCGTTGAGGTCAACGTTCCCTACTCGGTCGTCAAGAAAGAGCAAGTCGAGATCCCGGCCACCGTCTACAATTACGGCACGAAACAAATCACGGCTAAGGTGGTGCTGCTTGGGACTAACGACATCTGCTCTGGGGCCAAACTAGGAAAACCATCCGCGGTCCGGGTGCTCGAGATTCCACCCGGACATGGCCGCACCGCCATATTCC

>SG9641520

CAAGACGAATCTTTGTTGGCATTTCTACGTTCATGTAGAATGGCTTTGCACTTGAAAATGAGATGCTGTTTGACAAAATCCCAAATCCACTGGACTCGTCCATTCCAAATGCACTGATCATCCATTCTGTTGGCACATCAGGAACTGGGATGTTGAAGATGTAGTAACCCAATGGCCCAATGTTGATGTCTTTCCAAAGCCATGAATTGGTATATAGCCGNNNNNNNNNNNNNNNNNGATACATTTCAAACCTAGGGATGCTGAACCGCTGTTCGTCCTCACATCTTGTTTCGTCAGAGCCGTCCTCACAGTCAAAGTAGCCATCACATTGTCGCTCAAGGCGGT

>SG9611795

TGTGGGGTATGTGTAGTTCACGGTGTTGAGCAGCGACACGTTGAAGACGTAGTCGTAGCAGCCGTTGATCTCAGCGACGCGAAGGATGACTGGCTTCTGGCGCACTGGGTAGTAGTAGTTGAAGGGAGATGTGTAGATTTGCACAGTGCCCTTGACATCTTCGCCATAGGTATACTTGGCGCAGATAGTGTACAGCACAGTAGGGTTCGTGACGACGTCATTGGTGTCAGGAGTGATGGTAACGGAAAACTTTGGCAGCACGTAGTCCTGAACTTGGAACGTTGCCGAACCAGAGTTTGTGCCTGCCGACACGCTGATCGTCCACGTGCCTTCGTCGGTAATGGCAAGGAGGTCGTAGGAGCCTTGAAGGAGGCCGTCGGTGAAGTTGACGTACTGCTGCTGGAACAAGAGCTGGCCATACGGGTTCGTGATGGTAATGTTGGCCAGCTGATTCGTCACGGGGAGGAGGTTGTTGTCAAGCAGCAGTATGCGGTAGTTCACTGTGCTTCCTTGACGGTACATGGGCTTATCCGTCTGTACAACGACGTTGACATTGGTAACGTATTCCAGTTGAACAGACGATCTGTTTTGGAATTGGTCGCTGCCGAATTGTCCGGTCACATCGAGGTACAGGTTGTAGAAGACGCTGGGGCTTAGGTCGGGAACGTGGAAGAGCAGCTCGGCGTCGATGCCATCTGCGCCGATGTCATACGTCCGACTTGCCAGCACTGTGCTGTTCTCGTTGATTCCCACCCGAGTGCCCCACAGATCTAC

>SG965693

GCTGAAATCTACTTTGACTATCTCGATACATCACCCATCTGTGTCAACTTTACTGCCCAGCGTTGGTATCCTGTTGCCAACATGACACGGTTTATTTCAATTCGTGTCTACGACTATTATGCACCGGAGCGGTTCAACGAGTCAATGTTTGAAGTGTACAACCTGTACGCACTCAGCATCTGTCACGTGTGTGGCTCCTACCAGTGCCCATTCTGCCCAGTGTTCAACACAGCACCCTCTTTTTTGCACCTGAACCAACGGCAAGCACTGTTGCCCCTGACTGTGCTTCTGGTATTGCTGTGGAGGCACCTCTTCAAAGGCTAGCAGCANNNNNNNNNNNNNNNNNNTTATTTTTCTCAAGCAACAAGAACAAACTTTTTCAGAAGACATTGCCACAGCAGCTGTGCAGTCTGCTTCAGTCAAGACTCATTTGTGCAGCACGTTACGTGTTCCCTGTTTGGGCTGGCTCTGCAGAGCTGCAGCCTCCAATTTTTCTTTCAGTATCTGCCTGGATCATTCCGTTTGGAGGCAGGTTCAATGCTTGCTTTCGCCACTGAATTGTGGTGTGGAGCTGTTCCATTTTGCTATTGCGAGAGAGGACACGGCATTACTGCCTGGCTCAGCATAGCTGCCCTGGGAAGAAGGCACCTGCCAGTCAACAGAGTTTAGGCGCCTTTCCTTTTTTGTGCATTTTTCTAGAGTACTGGTTTTAGTATGCACCTTTCATTTGGCCTTGTGCAGGAGACGTGCACTTTATTTTTGGTGTTATAAATTGAGAGTTCATTTTCAGCTTAGCTACAGCAGTTTTCTCTTTGGATCATCAGAGCTGATTCTTAGTTTGGCATTCATCTGTTATGAACATCTAGTAGTAGTCATCTAGAGTTTGACATATCTAAGCAAGAGTTGTGTGTATGTGTTGAGTGCTTGTTTACTTTTGCATTCTTTCATATGCTGCATTTTGTATGATCATGTGCTATCAGCAGTGTTAATTTTTGAACATCCCATCTGAAAGAAGAGATGATGAATACTGTTTATTGCGAGCTTTGTTCATCTATTCCATTTTTTTTTTACTGAAAATCTGAAAGCAATAGTCCTCATTCTTCATGTGAAAACAGTTGAACATCACAGGAAGGCATTGTGATTAAAGAGGTAAAATGTTCACTGGAGGCAAGTCAGATGTACTGCCCCCAATTTGTATGACGTTTTATGTGAAGTAAATTATTGCTACACACAGTTTTTCATTTGCTAAAATATTCTGGAAGGCCTGCTCATAAGTCGTAAAGGTATGTATGTGGTCAAAAATTACTGTGAGAGCTTACCAGAAGGAATTGTAATTTTTGGTTCAAGTTAGTAGCAATTCATTGCAAATATTAGTGTATGGCTGATTCACTGCTGTTTGAAAGCAGTTGTTATGAAGTCCTTCAGCAAAAATGAAACAAATGGCATACTGAGAAGACTACTTAAATAATCATAACCCTTTGCGATCTTTATGAGGCTTTTTCAGGTTTTTATTCTGCTTTCATTGTAAGAGACAGTCTTTCAATGTCCCTAGCTTTTTCACAGTTGCAAGCTCCCAAACTTAAAAACTCTGCCAACTTGCTCACCTGTTATTGACGTCAGATAAATGCATCTTGTTGAAAAAGAAGTTGAATGACATGCTGTATTGCAGTGAAAGCAAATGTTGCATCGCTGCCGTACTGTTGCTAACATTGTATTCGGCACCTGGAACAAGGTACATATATTGAGTTCACATTTTTATGTCTCGTGCAACGTTCAAGTGCTGCAAGGACGAGCAATAATATAGAGTGGTTTTTTACCAAGGCCAAAACACATTGCTCTGGACTATTTATCTTGCTGTCTTGCCAAAAAACTGTAAATACTTAGACATAAAATGTTCTTGTAAATGCCTCTGGGTGAAACTTCAGGTCTCGCAGTTGTGTGAGTTGCTTCACAAGGAATTGGTGTGCATTCCAGTATTGAAGCAGCCATGCTGTGACTCTGAAAGTAGGTTGTGAGTAATGTGTGTAGTTTCCCAATCTATTCATGATACATTTGAAGCAGTTGAACGTTTTTATTAACTATTCAATAAAACAAAAGTCAAG

>SG9630383

CCAACAACTCCCTTTTGGCCAAGTATGAAATGACTGAGAAAAATGTGATCCTTTATTTCGACAAGATACCTTGGGAATCCCCGACGTGCGTCAAATTCAGAATAGAGCGTCAGCATGTGGTCTACAATGTTCAGTCTGCCGTCGTGAAGGTGTATGACTATTACAACCCAATGCACTCGTGTTCACAATTCTACGGGCCCGGATCCACGAGTCCCCTTCTGAAGCTGATCTGCGAAAACAATCAGTGCCAGTGCGCCGAAGCGGCATGCCCAAGGAAAGAGCCCTTCATTGACGTCGAGAGATCAGGAACGACAGTGAAGAAAAGGCAGAAGCTAGTCGAACTCACGTGCCAGGAGCATGATTTCGTTTGGATCGGAATGGTGTCAGCCAACAGGATCGTCAACGGTTACCGTTACATAGACTTCCGTGTGGACAGTGTNGTGAAAGAAGGCGCTGAGAACAGCACCTCCTNNNNNNNNNNNNTGAAAGTATTCGTGGCGAGGGAGTTTTGCAACACGGCCGACCTCACCAAGGGACGTAGCTACTTCATATTCGGCCGAGACAGCGAACCCCACGAGAAGGA

>SG9638795

CTCCTTGTAGTTCTCTGGAGAGTTGTATTCATACACCTTGGCTTTCAGGTACCTCGTTGAGTTGGCAACGGGATACCACCGCTGTATGAGGAAGTTGACGCAGGTCCATTCGCTTGTGAGGTATTCAAACGTGAACACGGCTGTCCTGGGGAGGACCTTAGCCATGCTGAGTCGAGGAACTACTCCTGACTTCACGTAGTTGTCCAGGNNNGGCCGGTAGTTTGCGTAACCAGTAGGGATGGCCAGTTCAAGAACAGCCGCCCCACTGGCTGCGCTCATATTGGTCAATGTCCACCGA

>SG9622509

CTACCGCACCCAGAGGACTCGTCCGCTGTGGAGGCCACTGCGTACGTCCTGCAGGTCTACCTCAAACTTGGCGGCCTCTTCCAGGACCAGATCGTGCGCTGGCTGAACCGCATGCGCTCCACAGACTATGGCTTCATCGGCACACAGGACACACTTGCAGCCCTCGAAGCGCTTACACAGTATTCCTTCCGAACGCACGTCCGAGGCATAACAGAAATGAAGGTCACTGTGGAGTCATCATCAAACCCCGGCTTTCCCAGCACCTTGCGCATATCCAAGGATGACCTTGCACGCAGAAAAGTCTTTGNNNNNCAACCAAACGTTTGGGGTCACTGCGACGTGCTGCCTCAAGGGTCCGGCCTGTCTCTTATCCAGTTGGATGTCACCTACAACGTCGATCGTGACTTCTTGCTTGTACCTCCTGCATATGAGGCGTTTGATCTCACCTTGATGCCGCAGTTCTCTGGGAGGAACAGGTCACACATCAACATCCGCAGCTGTGTTCGGTGGACATTGA

>SG9637488

CGTCACCTGGCCCAGCTGTGTCTCCAGGTACCGTACGCCGTCGCGCTTGGCAGTGTTGGCATGCTCTCGAATGCTTCCTGTAAGATCGGTGACCTTGGAAAGCGTGAGAACAACTTGCGCAGTCAGCGTGATGTTTTTGTCCTTGTTGTTAGCCTCCGATCTCATTCTGCGATTCGATGGGTTGCGCGTGGTCTCGTAGAAAGCGCCCGTGGTGTCCTGATGCTTGAGCAGGAACTGGACCCCTTGAGTGATGAGCCGAGGATCGATGTATATGTAGTCCTCCC

>SG9620656

ACATTGTTTTTATAGGAAATTGTACTGGCTGGATCGTACAAGCAAGCAGATGTATGTGGCCGAGTTGGATGGTACCAGCAGAAAAACGCTGCTGCCAAAGGGAATTTCAGATCCTCGAGCCGTTGCTGTGCACCCAGGCATTGGCTACTTGTTCTTTACTGACTGGGGCCATCATGCATTCATTGGCCGCCTTGGTTTGGATGGAACCAATTTCACTCGTATTGTCCTGTATGAAAACAAACTTGTCTGGCCAAATGCTCTTACAATCGACTACTTCAGTGACAAGCCTTTTTTGGGCTGATGCTCACCTTGACTACATTGAGTTTTCAGATTTGGATGGCAAAAACAGACACACAGTGCTGCAGGGGCGCACCGTTCCACATGTGTTTGCCCTGACTGTATTTGATGACTGGCTTTTCTGGACGGATTGGAACATGAAGGGAGTCTACAGGGCCCACAAGTTCACTGGGGAGAAGCTACAAGTTCTACGCAACACGTCGCACCGCCCATATGACATTCATGTTTACCACCCACTGCGCCAGTTGCCTTATCCCAATCCATGTGGCACCAACAATGGTGGCTGTTCACACCTGTGCCTGATCTCTCCTGGAGCCAGCAGCTTTCGATGTGCTTGTCCAGACAATTTTGTGCTGCTGCCTGATGAGAAAACATGCATTGCCAACTGCACCGAAGGCCAGTTCCGATGTGGTGGCTCTGATGACCGCTGCATCTCTGTATTCTGGAAATGTGATGGAGAGAAAGACTGTCGTGATGGCTCGGATGAAGTGGGCTGTCCTGCTTTCTCCTGTAAGAAGGGTCAATTCCAGTGCCATAACAATGCCACTTGTGTGCCAAGGATTAAGATTTGCGACGGCCATAATGATTGTGGAGACCAGAGTGATGAGAAGTTCTGTGGTTATCCATGTGGTGAACATAACTTTAAATGCCAGAATACTGGCCGATGCGTTCCTGTTTCTTGGCAGTGTGATGGTGACAATGACTGCACTGATGGCTCGGATGAAGATCCTGCTATTTGCCACCACCAAGAGTGCAACACAGACACACAGTACAAGTGCAACAATGGGAAATGCATACCAAAACTGTGGTATTGTGACTTTGATGATGACTGTGGTGATAACTCGGATGAGCCAGCACACAAGTGTCGAAACCGCAACTGCACTACAGGCTGGCAAAAATGTCCGTCCGTTAACAACTACCGGTGTATACCAACCTGGCTCTTCTGTGATGGTAAAGATGACTGCAGAGACAATTCTGATGAGACAAGTCCTGATTACTGTCCCAAATGTCATGAAACTGGTGATTTCAAGTGTAGAAACAACCGCTGTATACCACTGCGTTGGAGGTGCGACTTTGAAGATGATTGTGGAGACAATTCTGATGAGGACAGCACGATGTGTGCTGAACTCTACAGAGACTGCTCAGAAAGCGAATTCCAGTGCGCAAACAAAAAGTGTATCCCATCAAGATGGCGTTGTGACCACGACAACGATTGTGACGATGGAAGTGATGAAAAAGAC

>SG9633311

GGCCAGTGCATTCCTTACGAGCGTGTGTGCAACAAGCAGCAGGATTGCTCAGATGGGTCTGATGAACCTGCGCACTGCAATGTAGATGAGTGTGCCAAAGTAGAGCTCAATCAGTGTGAACACAAGTGTGTCAATACACTGACCAGCTTCTACTGCGAGTGCAATGATGGCTATGAATTGCTGAAGGACGGCAAGGCCTGTGCCGACATCAATGAGTGCCAGGTGAAGCCCGCTGTATGCAGCCAGTACTGCGTTAACAGTCCAGGTTCCTACTTCTGCA

| >AAFF14173  TAACAGCAGCATACCAACGGCAACTGTCCTTTCGCAACGAGAATGGCTCCTTCTCTTCATTCAAGGATGCCCTGGGGAGCGTCTGGCTGACGGCGTTCGCTACACGCATTATGTGCCAAGCGACACGCTACGCAAGCATCGACTATGACGTCATCAAGACAGGCTTGGCGTGGCTGATACGCAAACGGCAGGAGAGTGGAAACCTGCACTTCGAGTGGCCCTTGGTGGACAAGAGCATCCCGGGACCTGGTGATAGAGCCGTTTCTACGACAGCCTTCGTTGGCCGCACTTTCAGGGAGTGCTGGCACGTGGTGAAGCCTGAAGGCAGCGACGTCAATACCTGGCAGGCGCTAGCACAGCCTTTCAGTCCCGGTGGTGCTTACACGAACATGGCCGACTTGGAAAGAGTCAACCCTCATGGTGCAGCGCTTATGGCATACATATTCTTGATGACGGAGCATCAACAGCCTCACAACATAACATCGATACTCAAGCAGAAGCTCATCTACGACCCGGTACATAACACGCGAAGTGCGGGAAGTGATGCCAGCCCCTTGGTGGTCGAAAGCACAGCCTACGTTCTGCTGGTTCTACTGGCCTCCAACGACAGCGACACCGAGATGATCCAAACGTTGGCAAACTGGCTGGAGTTGCACCGCTCGTCGTCTGATATCCCCACGCACCACTCTGTGGTAGCCCTGCAGGCGTTGACGGAGTTCGCACTCAAGTACCAGCAGTCCGACATGGACCTGACGTGCAACGTGACTCACAGTGGCAAGCGCGCCTTCCAGAAAAGCATCCGGATCAAGCGGGACAACGCGGCGGTTCTGCAGCAGCTCGACGTTCACGACGTGACGGGTAAGTTGCTCGTCACAGCGAACGGAACCGGAAGTGGACTGCTCTCCGTGAGACTCAAGTACAACGTGCTCCTCCCACCTGAGCTCGTGTGCATGTTCAACATTTCAGTTCGAGCTGACATTCATACCCCGAAGGAGAAAAGCGCTGCAGTGTCGGATTTCCCGGAAGAGCTTCTGCTCGAGCTACTGGGAGAAAACAGGGCACGTCGATCCGACCGTGCCGGCTGGTCCTTGCCAGGAATCACTGGTACTACGGCCTCCCAAAGCGGCTCGTCACAGCAGAGCAAGCTCATTTACGACGTCGAAGTGTGTGCACAGTACCTTAGTGAGTCGGACTCTAACATGACGGTCATAGAAGTTGGACTCCTGAGTGGCTTTGTTCCCATTGCAGAGGACCTGAAAGCGGTCAAAATTAAAAATCAGCTCCTGGCTAACTACGTCATAACTGAGAAGAATGTAATGCTATATTTTCATTGGATACCATGGCGAAATGCAACCTGCCTCAAGTTCAGGACAGAGCGTCAGCACGTCGTCTACAACGTTCAATCGGCTCTCGTGAAGGTGTACGACTACTATGACCCGACGCGTTCGTGCACGCAATTCTACGGTCCTGATTCGACAAGCCCGCTCCTGAAGCTTACCTGTGAACGAGACGAGTGCCGATGCGTGGAAGATGAATGCCCACCCAGAGAACCCTTCAGAACAATTGACAACGTGGGAGCGACTTCTGAAAAGAGGCGCAAGCTGCTCGACCTAGCTTGCAAAGAGCACGACTTCGTGTGGGTTGGAACCGTGTCTCGGAACCATGTCACCAATGGTTATCGCCAAATCGAACTTCGTGTTGACACGGTGATCAAACAAGGCGCTGAACAGAGGGAGGCCGCCTTGACGGGCACTAAGCTGTTCGTAGCTCGCAAACACTGCAACACGGCTGACATGAGTGAAGGCTCACGCTACTTTGTCTTCGGCCGAGACGGCGAGCTCTTCGAGAAGGACGGCGTAGTTGTGTCTCGCTACATTCTGGATAAGCATGTTCGTCTCTTCGACACAAAAACGGCTGGGACATCACAAAGCGCCAGCAGGATAAACTCCGTCCTACAGTGGCTTACAGCAAGCATGCAAAGATCGGGAGGATGCCAAAGTAGTTGAGAGCCGTCCTTCTCAACACCAGCTGTCATATTGCGGATATCGTGAACATGGGCTATATAACGGTCATTTTGTGGGATGCAAGCTTCACTGCATGACGGCTGCGTCGACAACTGAAGGTGGCAAAATATTCTGTGAGAGCATTTCCGGTCAACGTCAATACGATACCATGCCACCGAGTGCTGCGACACGGGTACAAGATCGATATGCAGCCTTCCGGGAGATTTTTTCTGTCTGTCAAGCAGCGGTCTTTCATTAAGAAAAGAANN |
| --- |
| >AAFF26232  CTCTTCCGTCTGAGAGGCCAGAAGACCCGGTGCGGTTTCGCAATGTGTCTCTAACCGCGCACGTGCTCATCACATTGGCCGAAGTTAGGGATATCAGAGGTGAGATCGGATCGCGTGCAGCCACAGCTCGTGCTGCTGCTGCTCGGTACCTGGAGCGCATGCTGCACCTCATTCAGAAGTTGGAGGACCCCTACGAATTGGCCATTGTGGCATATGCACTCACACTGGTCAACAGCGCTGACGGGGAGGTGGCCTTCAACCTCCTTGATGAAAAGATGAGGGAGACAAGTGGCATGAGGTACTGGTCTCGCACTGATCTGCCAGCCCCAACAGTAGTGATTGAAAACAACAAACCTTACCTGCACCCACGACTTCCTTACGTTTATGATGCATCCAATGTGGAGACTACGGCGTATGGGCTGTTGGTGCATGTAAAACGCCAGGCAGTTGTTCAGAAGGAAATTGTAGAATGGCTGAACACACAGCGCCTCTCATATGGTGGATGGGCATCAACTCAGGACACGCTGCTGGCGATGGAAGCACTGCTAGAGTTCTCGATCACCTCTCGCTCACGCGATGTCACTGACATCAAAGTCACTGTGGAGGCTCCTTCAACACCGGGTTTCACCAGGGAGCTTCACATTGGACCTGAAAACTTGTCCAAGCTGCAGACACTGTCTATTCCCAATGCATGGGGTGTGATCATTGTGAAAGCTCAAGGAACAGGACTGGCCATTGTCCAGCTGCACATGGAATACAATGTTGACACATGGAAGCATTTGGTCACACCTCCGCCAGTGCCTGCATTTGCACTCAACATAC |
| >AAFF42733  TTATGCCTTGGCTCTTTCAAACCATCATGATGCACAGCAAACTTTTGACTTGCTTTGGAGCAGGGCTGAATCAGACGTTGATGGCACGTTACATTGGAGCCACAACGTGTCTTTGGGAGTGGCTGTAGAGACAAGCGGCTATGCAATTCTCTCCTGCATGACACTGTTCAAGGTAGCAGGTGTACCTAAAGCCTTGCCCGCCGTCCAGTGGTTGGTCAAGCAGAGGAGTGACCATGGAGGATTCGCGTCTACACAGGACACTATTGTAGCCTTGCAAGCCCTGGCTGCATTTGCCAAAGCAACTGTTCTGCAAAGCACACACTTGTCAATGAGAGTGTCGTCCCAACACACAGAGGCCAAAGAGCTTTTTGTGGAACCAAGCAATGCAATGCTGCTTCAGGAGGTGGTCATCCCTACAGTGCCGGCGATGGTGAACCTCAACGTCACAGGGAAGGGGTGTGCCTTTGTGCAGGCC |
| >AAFF46126  TCCACAGTGACCTTCATTTCTGTTATGCCTCGGACGTGCGTTCGGAAGGAATACTGTGTAAGCGCTTCGAGGGCTGCAAGTGTGTCCTGTGTGCCGATGAAGCCATAGTCTGTGGAGCGCATGCGGTTCAGCCAGCGCACGATCTGGTCCTGGAAGAGGCCGCCAAGTTTGAGGTAGACCTGCAGGGCGTACGCAGTGGCCTCCACAGCGGACGAGTCCTCTGGGTGCGGTAGTCGGGGCATCAGGAACGGCCGCTGGCTCTGGGACACGATCACGGGCGGCGGAATGTGCACGCTGCTCCAGTAGACAGCCCCCTCGGCATCTTTCTTCATGGATTCCAGCAGGTTGTAGCCGAATTGGGCTTCGCTGGAGCCAGCCTCAAGGAGTGCGTAAGTCGTCAGGGCTACGGTGTATGGGTCCGTCACCTGGCCCAACTGTGTCTCCAGGTACCGTACGCCGTCGCGCTTGGCAGTGTTGGCATGCTCTCGAATGCTTCCTGTAAGATCAGTGACCTTGGAAAGCGTGAGAACAACTTGCGCAGTCAGCGTGATGTTTTTGTCCTTGTTGTTAGCCTCCGATCTCATTCTGCGATTCGATGGGTTGCGCGTTGTCTCGTAGAAAGCGCCCGTGGTGTCCTGATGCTTGAGCAGGAACTGAACTCCTTGAGTGATGAGCCGAGGATCGATGTATATGTAGTCCTCCCAGTCCGGAAACACCGTCAGCATGTACATCCGTAGGCTGAATGCAGTCAGCCAAACACTAGGCTCCTTCTTGAACATGGTGAATGCCCCATTCTTGAAGTAAGAGCTCTGATAAACGTAGGCTTTGTTCAACTTCTCAAAGATGGGCTTCGCAATGTCGCGGGTCAGCTGGTCTGTGAGGCGCAGATAGGTGAGCCGTA |
| >AAFF13015  ATCTTTTTCATTCACTGCAGGCTTGCATGGTTATGAAGCAATACAATTTTTTTCTCACAAAGGCCTTGACTTTTGTTTTATTGAATAGTTAATAAAAACGTTCAACTGCTTCAAATGTATCATGAATAGATTGGGAAACTACACACATTACTCACAACCTACTTTCAGAGTCACAGCATGGCTGCTTCAATACTGGAATGCACACCAATTCCTTGTGAAGCAACTCACACAACTGCGAGACCTGAAGTTTCACCCAGAGGCATTTACAAGAACATTTTATGTCTAAGTATTTACAGTTTTTTGGCAAGACAGCAAGATAAATAGTCCAGAGCAATGTGTTTTGGCCTTGGTAAAAAACCACTCTATATTATTGCTCGTCCTTCTTGCAGCTCTTGAACGTTGCACGAGACATAAAAATGTGAACTCAATATATGTACCTTGTTCCAGGTGCCGAATACAATGTTAGCAACAGTACGGCAGCGATGCAACATTTGCTTTCACTGCAATACAGCATGTCATTCAACTTCTTTTTCAACAAGATGCACTTATCTGACGTCAATAACAGGTGAGCAAGTTGGCAGAGTTTTTAAGTTTGGGAGCTTGCAACTGTGAAAAAGCTAGGGGCATTGAAAGACTGTCTCTTACAATGAAAGCAGAATAAAAACCTGAAAAAGCCTCATAAAGATCGCAAAGGGTTATGATTATTTAAGTAGTCTTCTTAGTATGCCATTTGTTTTATTTTTGCTGAAGGACTTCATAACAACTGCTTTCAAACAGCAGTGAATCAGCCATACACTAATATTTGCAATGAATTGCTACTAACTTGAACCAAAAATTACAATTCCTTCTGGTAAGCTCTCACGGTAATTTTTGACCACATACATACCTTTACGACTTATGAGCAGGCCTTCCAGAATATTTTAGCAAATGAAAAACTGTGTGTAGCAATAATTTACTTCACATAAAACGTCATACAAATTGGGGGCAGTACATCTGACTTGCCTCCAGTGAACATTTTACCTCTTTAATCACAATGCCTTCCTGTGATGTTCAACTGTTTTCACATGAAGAATGAGGACTATTGCTTTCAGATTTTCAGTAAAAAAAAATGAAATAGATGAACAAAGCTCGCAATAAACAGTATTCATCATCTTTTCTTTCAGATGGGATGTTCAAAAATTAACACTGCTGATAGCACATGATCATACAAAATGCAGCATATGAAAGAATGCAAAAGTAAACAAGCACTCAACACATACACACAACACAACTCTTGCTTAGATATGTCAAACTCTAGATGACTACTACTAGATGTTCATAACAGATGAATGCCAAACTAAGAATCAGCTCTGATGATCCAGTGAGAAAACTGCTGTAGCTAAGCTGAAAATGAACTCTCAATTTATAAACCCAAAAATAATGTGCACGTCTCCTGCACAAGGCCAAATGAAAGGTGCATACTAAAACCAGTACTCTAGAAAAATGCACAAAAAAGGAAAGGCGCCTAAACTCTGTTGACTGGCAGGTGCCTTCTTCCCAGGGCAGCTATGCTGAGCCAGGCAGTAATGCCGTGCCCTCCCTCGCAATAGCAAAATGGAACAGCTCCACACCACAATTCAGTGGCGAAAGCAAGCATTGAACCTGCCTCCAAACGGAATGATCCAGGCAGATACTGAAAGAAAAATTGGAGGCTGCAGCTCTGCAGAGCCAGCCCAAACAGGGAACACGTAACGTGCTGCACAAATGAGTCTTGACTGAAGCAGACTGCACAGCTGCTGTGGCAATGTCCTCTGAAAAGTTTGTTCTTGTTGCTTGAGAAAAATAACTGTCACAGCACCACAACTACTGCTAGCCTTTGAAGAGGTGCCTCCACAGCAATACCAGAAGCACAGTCAGGGGCAACAGTGCTTGCCGTTGGTTCAGGTGCAAAAAAGAGGGTGCTGTGTTGAACACTGGGCAGAATGGGCACTGGTAGGAGCCACACACGTGACAGATGCTGAGTGCGTACAGGTTGTACACTTCAAACATTGACTCGTTGAACCGCTCCGGTGCATAATAGTCGTAGACACGAATTGAAATAAACCGTGTCATGTTGGCAACAGGATACCAACGCTGGGCAGTAAAGTTGACACAGATGGGTGATGTATCGAGATAGTCAAAGTAGATTTCAGCCTTCTTCTCCTCATAACGAGCTTCCCGAAGGTTTCGCACAGCTCCAGATTGCACGTATGCATCCAGAGTCTGCTGCTGAATGTAGTAACCTGATGGCAAGTTCACTTCCAGCACTGCCATGCCACTACGGGGACTTTCATCAGTACGGATCCAGCTTTGGCACGACCTAAATGAAACATGAGAGCTGTTCCTTCCATATGAATACTGTC |
| >AAFF13202  AGCTTTGTGCTAGTTACCTCCTCTCCTGGGGCATATCTGGAATGGTGATCATACAAGTCCATCTGCAAACCGGGTTCCGTTTGAGCAAGGTTGTTGATGGCCTGGAAGTTAGGAAGACTGAGGAAGAGCCTGACAAAATAAACTTGTACTTTGATGAGCTAAGTAATCGAAGAATGTGCATCACCTTAATGCTGACCCAGGAGTTTGAAGTGCAAGACAGTGCTCCTGCAGCTGTTGTATTGCAGGACTACTATGATCCTGGTGTCATTGTACTAAGGAACTACACAATTCCAAGTTGTGAGAGGGATCAACAAGACCTTAGTGACTCACAAATGCCCCTGCAGTCAGAAACAATGCAAGAAGATATTCGTTCAGCCAGGACTCATCTTGGAAACTTCCGAAATATCGAACAAGACTTGGACTTCCCAGATGGCCCTGAATCAAACATGCCTGTTACAGTGCCTGCTCCTCAGACCTGACCGGCAAAAGGACATAAACGTGCAGAAAACTTTCATACTGACAGAACTTGCAAGTTGCTGGGATGTGCTCCAAGTTGAACCTCAATGCACTCCAGTTTTTATAAAAAGAACACTATAAGGAACAACATAGAAGAGCCAGAACTGTCTTTTGAAAGAAGCATATAATTATTAAGTTGAAGCTTTATTCGCTTTTGTGGTAAGCAAAAAAATACACTTGTTCAGAGTTTACACCTGAGAACCAAAATGTGTGCTTAACGTATTGAGCATGCGAACTTTCCTCATTTGTAACTTGGAAGTGCAACTGAGCAACATGTTAAATACAGGTGCCCATATCTTTCTTGTGGAGTGAAGTTGGGTGACCTTGCTTGGCCCAAGTTTTACAGTCTGCGTCATCACTGTACTGAAAGCCACCTGTTTT |
| >AAFF23147  TTTTTATTCATTGCAGCCACCTCGCCTTTCAAAGGGTCCAGCTAGCCAGTCGAGGGTCTTTTTGATAGGGCGTTTGCGATCACCTGTTCTGCGGTCGTAGATGCGAACGTACTGGTTGAGCGGGTACCTAAGACTGGTGGCTTCCTCTGTGACGTGCTGGTCACTGTCTTTGCCAAATATAATGTACTCCCTGTTCACATCAAGGTCTGTAGCGTTGCAAAGCTCTCGACCTTGGAATACCCTCACGTTACCGGTCAGAGTCGTTTTGTTCTCTTCTCCTTCCTTTATAACGAGGCTGATGTTGAACACTAGGTGCCTGTAGCCCGTGGTCACGTACTTGGAAACAACTTTTCCTTTCCACACGAAGTCATGCTTGTCACATGCTATTTTTTCTATGATGGCCCGCTGCTTCTGTGTTGTTTTGTTTAAAAGAAGTTGGAATGTTTCTTCAATGGGGCACTCCGCTGCGGTACATTTGCATTGGCTGCCTTCGCAAAAGAAGCCCACCAAGGAACCAGCGGGCCTGTAGAACTGACTGCAAGAATCCTCTGGCTTGTAGTAGTCGTAAACTTTCACCGTTGCAGTTTGGAGGTTTGTTACCGCAAAATCTTTTTTGATTCGGAAGGCCACACACGTCTCAATAGCTGACTTTATCGTGGCCAGGTAAAAGATGACGCTCTTCTCATTTATCTCGTACTTCTTGACTTCTGGGGTGTCGATGATAACCCTCTTGAGGTCATCCTCAATGGCGCTGAAGCCGGTCAAAATTCCAACATCTATGATAGCCATGTTGGACTCCTGCCCACCTGAGTACCTTGAGCAGACCTTGACATTGTAGGTGCTCTTGTCTGCAGGCGTCTTTGAGTTCACTCCAGCTGATCTTCCCACACGGTTTCTTGATAGCAGTTCTTTATCGACGCGTAACTTATCCAGAAGTGCAGCAGGAAATTCTTCGCGTAGTTTGTTGCCTCCTTTACCAGTACCAGCTTCAACGGAAAGTGAGAACTTGCACACTTTTTCAGGAGGCTTCAGGACGTTGTACCTGAGCCTGACATTCAGCGCAGCCACTCCCATTCCGGTTGCGTTGATGACTATTGGTCGTCTGGCGTCAAAGATATCAAACTCCTTCAACACACCAGCGTTGTCACGGTTGATGTCATAATAATACTTCGTATTATCGGCGAAGATTGAGACGGA |
| >AAFF43607  TCGGCATCTACTTTGACAGGATTGGAAGACAGGAGCTGTGCGTCACTGTACCAGCCCATAGGGAGCACAAGGTTGCCAACCAAAAACCAGTCCCAGTCAAGGTTTACGACTACTACAACTTGGCTCGCAGTGCTAGAATGTTCTACAGCCCTCACAAGGCAACATTATGCGACATCTGTGAGGGTGTTGAGTGTGGCAATGACTGCAATGCTGTGAAGCAGACAAAGTCAGATGCTACGCAGCTGGAAAGAGAATCTGAGCCGGACGGTGCTGCTGGTATCCATGCATCCCATCTTTCTGTAGCAGTGCTTGCAATTACTGTGGCACTGTTTTGGAGATAGTGGCATAGTGAGGCTGACTACCAAGCAGGACTGCTTGGTATACGTAGCGCTGCATTTCATGTTAATGCAGTTCTGTGACTTGTTCTCATCACAAAGGCGTGCTCAGATGTGTTAAGCTTTATTTTGGCATTCATACTGCGGCATCTGTAGTTTTGCTTGTGTACTACTCTGTGTCAACTATGTAGTGCATTTTTGCACAGTGTGAAGCTCGTCACTGTTTTAGTGTTTCTTGTGACCCTGGCAGTGATAAGTTGCTGCTTTATGTTTGTTGCTATCATATCTCTTTTTTCTATGCTTGCACAGACAAGTACCCTCGTCATTTGAGGTACAGTCAATATGACAGGGAACACATTACTTGCATGCAGCCAGCGGTTTCCACCAAGGTGCAAATGAGAACCTTGCATTTTATTTTCTGTGGAAGTTTTCCTGTTGACAAGTGGTCCTTATATTTCTTTCTAGTTTGATACAGGTTTAAAGAAAAAAAAGTTGAGATTTGATTGTGAATGAAGTGTTTTCTTTTGTATTGCATGTACCTGTACATTGTAAAATTACTCTGGGGTTAACTGGTCATTGGAGATGCCTTGTAGATGTTGACATTGCTAGATGCTGTTATTTGTGTAGGTGAATTGTGAAAGCATTAATCTA |
| >AAFF14703  GCGATGGGTGCCGGTCCAGCAAATCCGGGGGAGGCACTCGGAGCAAATGCCACTTTGTTCACAGCTCCGAAACCGCCGAAACTGGGATAGTAGCGATTTGTCGATCCCAGGACTTGATTGTTGGGATAGCCAGGGAAAGCACGGTTGGGACACGGCCGCGTCTGGATTGGCAGGTTGGTGAAGACGACAAGACCAGTGTTCTCGAACGAGCTCAGGGAGTCGTAGCTGAAGGAGACTGAAGGGGCCACTGTCTGCCTCCTCCTCCGCACCTTGAGCTGGGAGTCACCCGACTTCTCGTAGGGTTCGTTGTAGAGATAACCGTCAACGTAGCAGTAGCTCTGGTTGACGAGAGAAGTGCGGGTGTACCTGTAGTTGAAGTAGCTGATCTGGTTGAGGAGGGCGTCCCTAGAATTGCGCTGGTTGTAACCATCCAGAAGGGTGACGCTGCTGTCAACAGCTCCGACACCACACAGGGAGTCTCCGGCAGCAGCAAGAGACAGCGACACGTTCGAGCCGGGCAGAGCTTTCTTGGGATCAAACTCCAGCGACACGTTGTTCTCCAGGCACTTTTGGACGCTGAACTCCGCCCAGTCCGAGACGACTTCGATGGGCCTTTCATTGGAGTCACGGTGCACGTAGTACACAAGGAGTTGCACCTTGGGGCTGGCTTCCGCGGGTAGCCTTGACTGGAACTCGAAGCTGCCCACCGACCGCACGGTCTCTGGATCTACCACCTGGGGCTCGGAGCTTCCAGCTGGAGGGTCGCCGTTTTTGATCTCGTTGCCGTTGGACTGGTCCATGGTGAGCTCGGCTTCGGTGAAGTGTTTTGTGAAATTCTGCGTCTCGAGGATGTGGCCGCCCGAAGTGAGCGTGAGGAAGATGTGGTAGTCAACGTTGGCGTCGGCAGTCATCACGATGGGGCGCGTGAACTGGGCCTGGCACGTGTACACACTTCCGTCTCCTCGGTCGATGCGGATAGAGCTGGCCGTGGGAGAGTAGAAGGGGACAAGGCTCAGCTGCGCCGAGGGCTTCTCCAGGGTGGGGCCATTCTTGACCACGATGCGCGGGAAATTGACAGCGATGGCCTGAACAATGACGCCAGTGACCCTCTGATTGAAAGGTGGCAGGCTGAAGCCAACGAGACCGTCGGCATCGGAGGTGTAGTTTCGGCAGGCGAGCACGTTGTCCGTCTCCCATGAGCGCCACTTGAACGTGTTGCGCTCCACGAACATGCAGAGCTGCACTAGCTCATTCGGGCGCGCAGTTCCATCCAACTCTTGCACGTAAAGCTGTCCCTTGTAGAGGAGGTTAAGCTTGAAGGTGTTGTCTGTGCTAGTGTATTGGTTGTTGGAATTGTACTTCTGTCCGAAGGTGAGCCTCAGTCGTGTGGTACCACGGCTGTGCAGCTCGGTGGCAGTTTGAGAGACACCTGTGCCGTCTTCGATGACCTTGGCAGTGACATTGATTGAAGGATATGTGGGGTACGTGTAGTTCACGGTGTTGAGCAGCGACACGTTGAAGACGTAGTCGTAGCAGCCGTTGATCTCAGCGACGCGAAGGATGACTGGCTTCTGGCGCACTGGGTAGTAGTAGTTGAAGGGAGATGTGTAGATTTGCACAGTGCCCTTGACATCTTCGCCATAAGTATACTTGGCGCAGATAGTGTACAGCACAGTAGGGTTCGTGACGACGTCATTGGTGTCAGGAGTGATGGTAACGGAAAACTTTGGCAGCACGTAGTCCTGAACTTGGAACGTTGCCGAACCAGAGTTTGTGCCTGCCGACACGCTGATCGTCCACGTGCCTTCGTCGGTAATGGCAAGGAGGTCGTAGGAGCCTTGAAGGAGGCCGTCGGTGAAGTTGACGTACTGCTGCTGGAACAAGAGCTGGCCATACGGGTTCGTGATGGTAATGTTGGCCAGCTGATTCGTCACGGGGAGGAGGTTGTTGTCAAGCAGCAGTATGCGGTAGTTCACTGTGCTTCCTTGACGGTACATGGGCTTATCCGTCTGAACAACGACGTTGACATTGGTAACGTATTCCAGTTGAACAGACGATCTGTTTTGGAATTGGTCGCTGCCGAATTGTCCGGTCACATCGAGGTACAGGTTGTAGAAGACGCTGGGGCTCAGGTCGGGAACGTGGAACAGCAGCTCGGCGTCGATGCCATCTGCGCCGATGTCATACGTCCGACTTGCCAGCACTGTGCTGTTCTCGTTGATTCCCACCCGAGTGCCCCACAGATCTACTTTCAGTTGTCCCGCCGACGCCGGGTTGGTCACCACAGCGGTGATCTTGATGTCGGTATTGGGAGTCAAGAATCCGGGTGCAGTCACAAGGTACTCGATAGAGGCGGCGGTGGGCGTTGTTCCCAGCGCGACCACAGCTGCCAGCAGTAGCAGCTGGTGCGTCCAACCAGACGCCATGGTGGGGACTTATGGCACCGTTTCCACTAGCAGCGGTCGATCACCGTAGCAAAGCCAACGGCCTACGCAAGCACCGATGCCACCGGAGCGCGACGAGCACAGAGCCGCGCTACAATCCTCAGACGCGCG |
| >AAFF25300  TTCTTTCAGACGACTCTGGACGTGGCAGCAGGCTCTACGGTGGTTGGCGAAGTATTGGTCAGGCCCGAAGACGTTCCCGATATGCAGCTCACGAAAGGAGACGCGACGGTGAAATTGTCTGTGCAATGCGGAGCGTGGAGTCGTGACGTGCTGCTGCCTTTGAGCGGGGCATCCGCAGACCACCTCTTCCTGCAGACGGATAAGCCCATCTACCATCCCGGATCCACCGTGAACATCAGGTTCATTGCTCTGAATGGAACTCTGGAGCCATCCTCTATTCCGTTCAAGCTTGAAGTCAGGAATCCTCAAAACGTGGTCTTGGAGG |
| >AAFF32258  AATTACAGCGCAGGGCTCAGGCGGTCTCAACTTCCGCAATGAAACTGACCTCACCTACGAACACAAGAGCTATTCTGTTTTCACGCAGACTGACAAAGCTGTCTACAAGCCTGGCCAGAAAGTTCTGTTTCGGGTAATTGTGGTGGACCCATATCTCCTGCCCACTGTGACTGGCGCCATGGATGTGTATATCGCTGATGCCAAGGGAAACAGAATCCATCAATGGGATCGAGTGTTCACGCAGAGAGGCATCTATTCTTCTGAGCTCCAACTCTCTGATCAACCTGTTCTTGGAGACTGGACTATCAATGTGGATGTGCTGGTAAGTTGACCTTATTTGATGAAGCTGTAGCTTTGTGGAATGAGTTTTATTTTTAATGCATTACATTTTTATATACTACTTGTGCAAGGAGGC |
| >AAFF32911  TCGGCTGCCACGCTGTCCGGCCAACTAGTCACCCTCGAGCCAACATGGCGAGCACACTGGCACTCGTGTGTGCCCTCTTTGTGGCTTTGTGCACGCCGCGGGTGTCTGCCGAAGAGAACTGCTTTGTGGTGGCACCGAACGTCTTCCGGCTGGGCACCGACGAAACCTTCGCGGTGATGGTGGACGGTGTCAAGAAAAGGGTGACGGTCACACTTCAGAACTACCCGAGCAGTCGGGGTGCGTTTTTCCACTGGACGGGGGACGTGGCCAGTGGTACTCCACGAATAGTGGACATCGCCGTAAAGGAAACCGACTTACCTGAGCTGCTCTACGGGCGGGACCAAATCTACGTCACGCTGGAGGTCACTTGCGGCAGACTGTGGACGAGGAAAACGCAGGTCTTGGTCAGCCCTGCATCGGGAGAGCATTTCTTCCTCCAGACAGAAAAGCCGATCTACCACCCGGGAAGCACGGTTAACATCAGGTTCCTGGCCGTCGATGGAAAGCTGGAACCATCCGCGAGTTCCTTCAGGCTCGAAGTTCGGAACCCCCAAAACGTGATTGTGGAAAGGACCGATTTTCAGCCGAGCAAAGAGCTGATGCTCACGCACACCTACGAGCTGCCCGAGCGCACGCTGCTGGGAGAATGGGCGCTCGTCGTGAAGTACGGCTACAAGTTTCAGCAAAACACAACAGCCACATTTCTGGTTGATAAGTACGTGCTTCCCAGGTTCAAGGTTGATCTGAGCGTTCCGGATTACGTACTGAGCAATTTTTCCACAATACCCTGCAGAGTGAAAGCAAGCTTCGTGAACAAAATGCCCGTCCATGGAGTTGTGCTGCTCGAATTTGGGTTGAG |
| >AAFF2850  AATCCGGTCCTTCCGCCCGGTGGGCTCCCTGAAGCACCACCTCAGGTGCCTGGCCGCATTAGGCTGCGTCAGCAGTACCCCGAAACTTGGCTTTGGACCAATGCCACTGCCAGTCCTGATGGTAGGGCTGTTATCGCAAGCACAATCCCTGACACCATTACATCTTGGGTGATCAGTGCTTTTGCTCTGGATTCTCTGACTGGTCTTGGAATTGCTCCCTCGCAAGCAAAAGTGACAGTCTTCCGGCCATTTTTTGTGACTGCTAGTCTGCCGTATTCTGTTCTTCGGGGAGAGTCTGTGGCAATCCAGTGTGTTGTTTTCAACTACAACAAGAAGCCAGTTGAGGCGGAAGTGACACTGGAGAATAACCGCGGGGAATTCATCTTTAGCTCTCTGTCGAATGATGTCACGGGAGAAGAAAACAAGGAGAGACGCACAAAGAAGGTGATGGTGCCAGCTCAAGATGGCATCCCTGTGTCATTTCTTATTACTCCTCTCAAGCTGGGTTACATAGACATTCGTGTGTCGGCTACTTCAAGTCTGGCTGGTGATGCCATACTCAAGAAGCTTCTTGTCAAGCCTGAAGGCTCGAAGCAATACTTCAACAAGGCCATCCTAATTGATAGACGGAATCC |
| >AAFF11160  TTGCTGGACCGGCACCCATCGCAGAGTCTGCGCAAGCCGATTTTTCGCCCGTCGTCCAGGGGTCAAACTCCGTCCGCACACTCTTCCCTGAAACATGGCTCTGGCAGATCAAGAGAGTGAGCCCTGACGGTTCCTTGGTGTACTCCGAGACGCTCCCGGACACAATCACCACATGGCAGGGCACTGCTGTATGCCTGCACCCACGAAACGGCCTCGGCGTCTCCCAAGTGGCAAACGTCACCGGATTCCAGCCATTATTCGTGTCGCTCACGTTGCCGGCGTACATGCAGCGCAACGAAGTGGCCACGGTCATTCTCACGGCATTCAACTACGGCGAAGAGTGCGTCGCGGTCCGGGTCAGTCTGAAGTCTCTCGAGAATCTCGAGGTGGTCGGTGGTCCCAACAGCACCGACGCACTGCTCTGCCCCAACTCCAGCACATCCGCGTCATTCCCGTTTGACGTGCGCGCTACCACGCTTGAGGAATCCCGGCTGGAAGCCCGCGTCCAGACGAGACCCGAGGCCCAGGCACAGTATCCGGACGTCAAACTGTCCAACGTCAACTCAAGCGATACTGTCATCCAGACCATCGATGTTAGGCCTGAAGGCTTCCCCATCAGGGAAATCAACACGTTCCTCCTATGTGCTTCAGGTGATGCCGACAGCGCTCCGGACACCCTGCAAGTCAACCTTCCGACACCTGTCGCTCTTGTTGAAGGCTCCCAGCAGGTTGTACTAGTGGGCACAGGAGACATCCTCGCTCTCAGCTTGAACGACCTATCAGTTCCCACCATCACCTATTCCAACGCCGAGGGAACACTGGCTGTGCTTGCCTCCAGCGTCTACCTCCACAAGTACCTGGAGCAGACGGGTACTCTGACTGACACGGTGAACTCTGGCCTCCGATCCCGCATTCGCCAAGCGTCCCAGGCTCAAAACAGCTTCCGGTCCTCCGACGGCTCGTACGCTCAGTTTGGCTCTAGCGAGTTCCCGAGAAGCGTGTTCCTCACCGCCTTCGCCGTGAAAGCCCTGAGCGCCGCCAAGGAGTACCTGGGCACCAGCGTCGAAGCCGACATCGAAACTAGCGTGCGCTACGTCCTGCAGCACTGGAACGCTGCCACCGGTTGTTTCGTTGAGAACCAGCCCGGCTCGTCACCCTTCGGCCCCCAGACAGCCCCGGACTTCACCGCGGCCATTGGCGTCATGCTTCTGGAGAGTGGCTACAACTACGAGAACATCACGAACGGTGTCCTCCAGTGCATTGACGCCAGCAATCTGCCTTCCAACCACACGACTGCGCTGAACGCCTACTTTTCGGCGCTCGCTGGGCGCACGGACCGAGCTAACAGCGCCTTGGACACGCTCCTGAGCGAAGCTGATAAAAGCAGTGGCTTGACGTCGTGGAGCGGCGATGGCCTGACTTACGGCTCGGCAGACACCGCTGGTTACGCAGTTCTCACTTTGAAGTTGCTCGACCGAAACCTGGGAGAAGCGCTGCCAATCGTCCGTTGGCTCATGCAGCAGACCTACGCCCGATACACTTTCTCCTATTCCGAGGTCTACACCGTCGCCATCCAAGCCCTGACCCAGTACTCGAGCGTGGCATTCTCGAAGAACACTAACTTGACTATGAACGTTGCTGTCGACAGTTCATCCCCCGAAACGGTCTCTTTCCCGATCTCTGAGCAGAACAAACTGCTATACCAGGAGCGTCTTTTGAACAGAAGCGATTCGTACAGCTTCAAGGCATCTCTTGCTGACGGCAGCGCAGGGTGCGCCGCTTTGCAGGTTAAGTACTACTACAACTCCAGGAACAGTCCGGTTCAGCGAGGAATACAAGTCAACGTGTCCACGACATCTGGACCAGATTGCAGCACCCTTGAACTTGAAATATGCACACGGTACACCGAAGGCTTTCTGCGCAGCTCCGCGATCGTCCAGATCACCCTGCTTTCTGGATACTCGGCAGATGACCAGTCTCTCAAAAACCTGGTGAGCAGCGGCGTCGTTAAGCGGTATGTCGTCAACGGCAACCAAATCCAGCTCATCATTCAGACGTTGACCATCCAGCCGACATGCTTCAGCGTAACGGAAAGCAGGTACCTGCAAGTGGCGAATCTTCAGGATGCAGTGGTGGAGGTGTACGACTACTACCAGTACCAGTACAAGGCAACGGCTGGCTACAGAGTAGAGGGCAACTGCACGCCCGCTGAAGTCGCGCCACCCACGAGCCTCAACGAAGTGGACAGCGTGTTCTTCGTTTAGAGATCTTTTCTCACTTCCTTTCCATCCATCCGCACTTCTCTAAGCATCTGGACAAACGATCTCAAGTGCCTCCGAGTCATCTGCCTTGTGATACCTTGTGTTCGTCTGTATTTTAGCAATCGCTCCTCTCATCCTCAATAAATGTATCCTCTCAAGAAAAA |
| >AAFF22245  GTGAGAATGACCGTGGCCACTTCGTTGCGCTGCATGTACGCCGGCAACGTGAGCGATACGAATAATGGCTGGAAACCGGTGACGTTTGCCACTTGGGAGACGCCGAGGCCGTTTCGCGGGTGCAGGCATACGGCAGTGCCCTGCCATGAGGTGATGGTGTCTGGGAGCGTCTCTGAGTACACCAAGGACCCGTCAGGGCTCACTCTCTTGATCTGCCAGAGCCATGTTTCAGGGAAGAGTGTGCGGACGGAGTTCGACCCCTGGACGACGGGCGAAAAATCGGCTTGCGCAGACTCCGCGATGGGTGCCGGTCCAGCAA |
| >AAFF45887  AGGAATCGGAGATGGGGGTGATGGTGAGATTGCTGAGCTGGCCCCAGTAGCTACAGTTTCAAAGTCTGCAGTGGACATCCGTAGTCACTTCCCTGATACCTGGCTCTGGGAGCTCCATCACATTCGAGGGGATGTACCACTCAACCTGAGCCGAGAGTTGCCCCATTCTGTGACTAAGTGGAGAGCAGGCGCTGTGTGCGTACACACATCTCACGGCCTGGGTGTAGCCACAGATTCTGTGGATGCTATGCAGCCCTTCTTCATTCAGCTGTCGCTTCCCAACATCACCAGACGTGGAGAGATTATTCCTGTCACTGCGTCGCTGTTTAGTTACCTGAAGACGTGCATACCT |
| >AAFF6837  TTTTTTTGTGAAAAAAATTAGTTTTATTCACTCTTTATGACACTATTTATAACGGTGAAGCCTCGCAGACGATGACGCGCTTTTCTCGCAAGTAGAAAATATATAAAGCTACTCTTCCACTCAGCTGGATAAGACGCGCTACGGGAAACAACATGGCGACGTCAAAAGTTCACGGCTCTGTTCACGCACACAAAAGCACACACTGCGCAGCGCTCCCTCAGTCGTCCGCACCCAATATCGAGGCTGTAAGATAAAACATCATGTGACCATTGTCTTGATTTCTTCGCGATGTGCGCTGTGGAAGCCATTAATCTTTTTTTTCTTTCGCGACATGGCACGTATGTCCTTCTCAAGAAGTGTGACCGGTATAAAAAAGCCTTTGGTGTTGAACACTACCGAGGGAGCAAGAAAGCATGATTCTGGTAAAAAGAAAATTGGCAACCAAAGGATAAATGCGCATTGAATGAATGGTATGTTGTGACTCTTATTGTCTTGGCGCGTTCGTTCGAAAAAAAGAGACATAAAAAAAAGGGAGAGTAAGAAAAACTGATGGCCGATGTTCACTAGCAGGACGCCACAAGAGAGTAGGGCACGGAGTTGCTGTTTTCTTGCTCGTAATAGTCGTAGACTTTAACTGTGGCAGGTTTGGCATCTTCAATAGCGAACTCGCGGTGAACGCGAACGTCGAAGCACTTGTTTTCGGACGTTATTTCTTCAAAGTAAAAGTTAACCTGGTTCTTTTCAACTTCATGCCTCTTCAGCTTGACGTCTTTCTCACGGTAGAGTCCAAAGATGTGATCCTCATCTGGAGTGTAGCCCGATACCAGCTTGAGCTCCACCACTGCCATGTTGGATGGTTGCTCACCGTCGAACCTCAGACAGATTTTGAGCTTGTGGTCATTGCAGTCAGACGCCTCTTGAGATGGAGTGGCTGTGAGCTCGAAACCCTCGCTCTTCGGTGCAGTGTGAACGTTGTACTTCAGAGTCGCCGATATAAGGGCACATCCAGGACCTGTTGCTTCTGAAGTGAGCTTGTTGGGCAAGCTGACAACCTTCTTCTCCTGTACGACCAGTTTGGTGTCCTCCTTAAGGTTATACGACTCACTGACGTCAGTACCGTCGACCTTAACTGAGATGTCAACGGGATCTTTGCTAACGTAAGTAGCGAACGCCGACAGAGCCTGGAGACCGAGAACTGTATCCTGTGTGGATGGGAAGCCTCCGCGGCTGTTTCTCTTGGTCGCCATCCAGCGGACAATGGGCTGCGCCTTGCTCAGGTTCTCTTGGGCGTTCAGCTTGAGGTAGGCGAGCACTGCATACGCCGCCGTTTCCACGTCGGCCGACGCTGATGGACCCTTCTTGCCCGCGTTGCTCCAGTAGGTCAGGGCACCTTTGTGAACAGCGATGGACTCCAATTTCTCGAGGTAATCTTTGGCAGACTCATGTCCCGCCAGGGCCGCGGCGTAGGCGGACAGGGCCAAGTTGTGGGCACTGGGGTCACGCTGGGCGCTGATGCAGCGGAGCGCAGACTCAACGACCTTCTCGTCCGCAAGGCCGCCCTCCAGAAGAGCGGTGAGCACGTACGCCGTCAGCGCTCCCGGGGCAGTGGAGTTAACCTTGCCCTTCAAGCCGGAGCTAAGAACAGTGCCGATGTTCTGGAAGCAGCCGTTGGTTTTCTGCTTCGTGATGATCCACTTTATGCTCTCGTTGAGGTTTCCGGCGTCAATAGGAATGTATTTCTCAGCCTGCTTGAAGGACTTGACTACAAACGCCGTGAGGAACATGCTGCCCGAGTTGTCGCGGTTTCCAAAGGCGCTGTACGAACCGTCGTAGTGCTTGTACTTCTGCTGGCGCTGGTAGCCTGTCTTGAGATTCTGCACAGCCTTCCTTTCGATGTCTTCCTGGTTCTTGCCCGTAGCCTTAAGGTAGTCCAGGACATAGACGTTTGGCGTAAACTTTACCATGTTCTGCTCACCACATCCCGTTGGAACTTGCACCAGTGAGTCGAGGTTCTTGATTGCTGGGCCCATAATATCTCCTGTGACTTGAACGTAGGCACGGGCCGATCCTTCCACCAAGTCTTCAGGAAGCACCAAGTCAAATTCGTTCTTTGCGCCGCCTTCTGCAGCGTCTTTAGGACAAACAAAGACACTCTGCGTCTCTTCCTTAGGGAAGCCTTCCGCCTCGATGATGAGCGGTCGTGTGACTGCGTCCCTGGCCACCACCTTCTCAGTTGGCTGCTCTCCACATGCGGCATCGCTCTGCGATCCGGCCGCCGACACGGTCAGGTTCACCTCTCCGATAGTTTGTGGCCGGATCTGGAACTTGTGCACTTGGCTCTTGCTGCCGCACACGCAAAGCGTCGTCTCGGACTCGCCCTCAATGTGGAAGTCCGCGGATTCAGCCAGCTTCAGATCGACCGGCAGGCACTTTTCGAGGTAGTTGAACACGGACACCTTGACGGGCACCAGTTCTCCGCGCACCACAGAGTAAGGCAGGTTGAACGAGGCGAAGAACGGCTGGAAGGCCTTGATCTTTGCCGGATCTGAGATGCCGATTCCGTCCTCTGAGTTGATGCACACAGTGCTTCCCACCCACTCGGTGATGGTGTGCGGAATCTTTTCTTTGAAGTTCAGTTCGCCGTGCTCATCCAGCTCCTTCAGGTCCCACAGCCACGTCTCGGGGAAGTAGGTGCGCACTTCAACGGCCGACTTGGCCGGGATGTTGGCCGATGGTGCGCTCTGCGCCACAGAGTCCAGGGCGACGGGCACGCCGGGCAGGCCACCTACAATGCGGTTGGAAAAGGCCACCCCAGCCGGAGGTGGAGGACCCGACAGTGCGAGTGGTCTCGCTAGAGGGATCGCTGGCGCCGCGAGAGCGTAGGGTGGTCTGTCGTAGATGTTCTTCCGGCATGGCCTGGTTTCAAGCGTCAAGTCTGACATGACCACCACCCCTGATTCGTCGAAGGCGGTGATCGAATCGACGTATTCAACATTTGAAGATCTCGGTCCATTCCAGATGTGGCGCTTGAACTGTTGCGGATTCTTCGCCAACTGCTTGCGACAGTAGTCGTACGACGCCTGCTTCGGCCACGTGTAGCGGGTGATATCCAGACGCTTCAAAATGTCATAAACTTTGTCCTTTGTCAGTTGGTTATCCTGCTTCAGGAGGTGAACACTCTTGTCTACGACGCCAACACCACAGAAGGACCGAGGAGAACCATTCACATGGATGGCAGCAGATGTTGCAGGCTGTACAGTTTCAGATCCGAATCTCATGGTTACGTTGTTCTGGAGGCACTTCTCCACTTCGAACTGCTCGGAGTCAGCGATGACCTCGCCGTCGGGGCGGACGTAGAAAGCGAGCACTTTGACGCGTGGCACGTGGCTGAAATCTGGCTCGAGCTCGAACTCGAAGCTCCCTGTCGACACGCTGCCCTCGGCCATGTTGGTCGGAAGCGTCTCGTTCAGGTCCTCCTCCACCAGGAAGCTGTCGTCAACAGAGAGCGCCTCTTCCGGCTTGAAGGTCACATCCAGAACCTTGTCCTTGAGGATCTTGCCGCGAGCTATCACCTGTAAATGGAACTGCTTCTCTGAGTCGGGCTGACCCGTGTAGCGGAGGCGCACGGGGTGCTTGCCCGAGCAGCGCAAGGGTCCCTTGGACGGCTCGATCTGGATGAAGTTGTTGCTGGCCGAGTACCAAGCCTGGAGGTACAGAGTGCTCTTGGGCTGGTTGATCTTCACGCCGTAGGTGTCGTACTTCACAGTCTCGTAGTTCATCGCCACCGCCTCGACGCTGATGGTGACCACAGTTGTCTTCAGGGGAGGGATGGTGAATTTGATGATGCCTGACTCATCCGAAGTGTAGTTCTTGCAGCTGAGACGGCGGTCGGTGCGCCACCACCGTGGCTTGATCTCCTCAGCCTGTGACAGCAGACAAAGTTGCACCAGCTCGCCGCCCACAGGAACGCCGTCAGGCTTCTTCACCAGGAGCCTGCCGTAGAAAGGCATGGTGGGCTTGAAGTAGTTCTTTCCGCGGTCTCCTTCCAAAAAGTTGAGCTCAAGCGGGTTGAAGCTGCGGCTGATGTAGTTCGTCTTGTTCATCGTGATGCCCGTGCCGGTCTCGTTCACTTGTGCGACGAGGTAGATGCGCTTGTAGATCTCGTAGTTCTCGTTGAAGCGCAGCAGGCTCGTGTTCACCGTCATGTCGAAGCAGCCGTTGATGGGTCCTGTGTGGTTGATCTTTGGGTAGTCGTCCTTCTCCCAACTGTAACGCTCGTATGTCACGTTCACCGTCAGGGTGCCGTCCACTGGCTGTCCAAAAGTATAGTGGGCACAAATCTTCCAGGTGATCTCCTTTGCATCAGCCAAGACGTAGGATGGAGGCTTGATGGTCACCTCAAATTTGGGAAGTACGTATTCGTTCACCTCAAAATGCTGCCTCACGGTTTGCGTGGGAAGTTCGACGACTATCTGCCACAGACCCAACTCAGGCTCTTCTGTAAGCTGAAAGTCTCTCTGAACAATGCCTTTTTCAAAAGACACGTCGTTCCACTGAGCTATTCTTACATCGCTGGGACTTGTCACGTAAATAGTTGCCTTCACATCGGTGACTGGTTTCAGCTCATTGTTGATAGGGAGGACGCGGAATTGAACCCTCTGTCCAGGCTTATAAAGTGCCTTATCGGACTGAACCAGGATTGTGTTCTTGGACTTCTGGAAGTCGATCTCTTTCCGATCGCCGAAGACATAGTCTCCAAAGGTTCCGTTCACTTCGATCTTGGCTTGGCTGTCGAGGTGCTCGGGCACTCGGAACGGCAGAAAGGTGCTTTCACCGTTCTTGATGTCGTACTCTTGTTCCGCCAAGACGATGCTGTCGTTGTTGTACTTGAGCAGGCGGACGGTGACCTTGCCATCCTCCTTGACGTCTGTGAGCGTTAGCCGAAACTGTTCGTCCGTTTCGCTGCGCAGGATCTTCGGCGCCGTGAAGATGTACCCACTTTCAGCAAGGCGCGATGCCAAGGCACCAAGGACTAAAATGAAGGTGCCCCAATGCATTCTAGCTCTGGCCACCGGCACCTCTCGTCACCGTCTGTGCTAGTTTTCTTGCGTCTAAGCCAGCCTTAGACACCTTTCCGTAGACTGTACAGCACAGCTTTCTCTGCTGCGCGCGCTAAGGCCGGTTCCGGGAGCTTTTTCTTGCTACCCTTCTTGTCTCGCTCCTGGCTTGACGTCGCGTGGCGAAGCAAAACACAACACACGCGCTCTAAGGCGGCGGCGTTGGGGCGCAGAGAGTGGTGCGAGACGAGGCCGACAAGACCCTCCGACAGCCCAAGCCCGAACTGATCCGGCGGCTGCTCGCC |
| >AAFF40317  GGCGAACAGAACTTGGCTATGTTGGCCACCCGTGTGGTGGTGCTGGACTACCTCTTCTCTGTGGGGCAATCCAATCATCCCTTGGTGGCCAAGCTCAGGAGAAGCATCATCACAGGTTACCAACAGCAGCTCAACTACAGGCATCAGAACAATGGCTATAGTGCCTTTGGCACTGCAGATCCTGAACCTAGCCTGTGGCTGGCAGCATTTGTGGTGCGGACCTTTGGACAAGCCCG |
| >AAFF42976  CGTTCCTCGAAGGTGCCATTTGCTTGTTGTTTCGTGGTAAGCCACCACAGGCCGCTCTCTATGACTTCAGGGCTGATGTCGATTACCTTTCTGGCCTCGCAAAAGACGCGGAGCACGAATGCTGTTAACCACACGTTTCCCGGGCCGTTCTGGAAAGTAGAAAATGATCCGTTGTCATTCCTAAACGACAATTCTTGCTGATATCCTTTCCGCAGATAGGTTAAAGCCGTGTTGAACAACGTCTCGTTAAGCATGTTTTTCATCTTGAGATAGTTGTATGCATACAGCACTGGAGCCATTATCATCATGTTCTGCTCTCCGCATCCCATCGGCATCGATATCAGCGAGTCGACATTTGAAATAACTGCTTCTGTCAACATGGACGTTTCGCTACCAATGATTGACACACTGCACGATTCAGTGTGCGGAACCGTATCGGGAGGATACCATGGAATTATAGAGATCACTTGGCGCCGGTTTTCTTCGTCCAGTATATCTCTGCAGCCTCTCTTTAGGACAGCTCTCGTCTTTCGGCGTTGTTCATTGCTGGGGTCCAGTCGAACGGAGAAGGTTTCTTCAAATGGCATTCCTTCGGGCACAACCACGAGGGTCTTCTCCACGAGGTCC |
| >AAFF46667  TGGTTTGAAAGAGCCAAGGCATAAGCCTGAAGTGCCAGTGAGTAGCTGTCCTGCTCATGAGGAGTCTCTTCTATGAGGCATTGGATGGCTGCATTTAGTACAGATCTGGAGAGAGTCACATTTGCTTCAAGCAGTGCGATTAACACATAGGATGTTAGTGGTGAGAGTGATGTCCCCTGTACGCCGCCCTTGAGTTGGGTGTTCAGCACACGGCCTACTGATGGGAAACAACCAGTATCATATTGATGCCCGAGTAGCCACTGAGTGCTTAGGCTCAGCTCAGCATCATCAAAGAAAACATATTGGCGGGCTTGTCCAAA |
| >AAFF47627  CCTTTGGTATCCCAAGTTCAGATATTTGAATGCCTGTCTTTCCAAATGCATGTCACGTTGGTTTGTCTGTCGTAGATAGTGCACCGTGAACAAATTTGCCCCAAAGCTGAACATGTTTTGTTCCCCACAGTAAAACGGCTTTGACAACAGGCTGCTGGAATTGACAGGCATAGTTGGGAATGCTGGACCAAACACATCACCAACCACAGATAAAGTGGCCTTGTTACTGCCGAACACATACAAGCGATCTTGTCGGAAAGGCAAGATGGGTGACTCAGTAATGTTTGTGTCGAGATACTTGATCAA |
| >AAFF615  AGGGATCCGCACTTACCCACACCACCCACCTTTCCGCAACGCGTGCAAGATCGAGAGCGTTTCGGCACTGAGGTCACCATGGACGAGATCGATGGTGACGAATCGCCTGGCACCATGGTACGGAATGACTTTCGAGAGACATGGCTCTTCGACGAACAAGTGATTGGGCCTGACGGAGTAGCCGACTTCGCTGTGAGCCTGCCGCACAGCATCACCACGTGGTCGGTGCAGGCGGTGAGCGTCTCGCCAACCGGGGGAGTGTGCGTGCCCAAGGCCGAAGAGGTGCGCGTCTTCCAGCCCATCTTCCTTCAGGTGGCGCTGCCTTACAAGGTGGTGCGCAACGAGCAGATTGAGGTGCTCGTCACCGTCTACAACTACGGCAGCGAGTCCATCAGGGGCAACGTCTACATCTACGGCGTCGAAGGCCTGTGCACCGGGGCCCTTCCGGGAGAGCGCTCGGAGCGCCGCCCTGTGGTGGTGGAGGCCAACTCGGCGTCCAGCGTCACTTTTCCCGTGATACCACTCAAGGAAGGCGTGTTCGTCATCAAGGTCTTCGTGAAAAGCTCAATGGGCGAGGATGTCGTCGAGAAGGAACTGAACGTCGTGCCAGAAGGGGTGACAGTAGAGAAGAGTGCCTCTGTGCCAATTGACCCAACGAACGCTCGTAGACGAAGAACCAGAAGCATCAAAGGAGAGCTCTACCAAGATTCACTGGACCCTGGCGCAAACTTACAAGTGATCTCTGTAAACACGAGAATGCCACCTGACGCAATCCCGGATACAAAGACCTGCTCTCTCATGGTCATAGGAAATCAGCTGGGACCATCCGTTCAAATGACGCTTGAGAACATCGAGTCGCTCATCACAATGCCCACGGGCTGCGGAGAGCAGACCATGATGCTTATGGCACCGACGCTGTACGCTCTCGAGTACCTCAAGCAAAACAAGCTCATCGACGAAGCACTAGAGGAGAAGGGTTACCGATACATCAAAGAAGGCTATCAAAAGGAACTATCGTTTCGAAAAGACGACGGCTCGTTTTCTGCATTCACGCACAGGAAGAGCAGCGTATGGTTGACGGCCTTCGTGCTGCGCATCTTTTGCAAGTCGAGGCGCTACACGCACATCGACCCCACTGTGATTGAGAGCGGCATGCTGTGGCTCGCGGCTGCCCAAAAGTACGATGGAAGCTATGTGGAAAAGAAGCCCATCATGCACAAGATCATGCTGGGAGGAGTCCGAGGGTCTGTGCCCATGACAGCCTTTGTCCTGCTAACTTTCTTGGAGTGCATGAAGCCTCCGAGCATGACGAATGGTCCACACGACCCTTTTGCAACTACCAACAAAGCGATACTGCGTCGGACGGTGGAGCTGTCTCAGAGGTACCTGCGGGAGAACGCGTTCGACAGCGACGAGCCTTACGTCGCGGCCCTAGTTGCTTACGCGCTCAGCCAAGCCAACGACACGGAGAAGTACGCCACCCTGTCCGCACTCAAGGAAAAGCTCATCTATGACCACATGCTCAGCACGAGAAGCACGGGAATGGAGGCCAGTCCTCTGGTCGTCGAAGGCACCGGTTATGCGCTTCTAGCGCTACTTGCCCACAACGACATCGAAACGAGCAAGACAGTGGTCAACTGGCTCAATACGCATCGCTCGGCGTCCGGCGCTTTCGCATCGACACAGGACACCGTGGTGGCCCTTGAAGCGTTGACGGAATTCGCTCTGAAATCTCGAGAGCCAAATGTGGACCTCACGTGTAATGTCACCCTTAGCAGTAAACGGGGCTTCCAGAAAAGCATTCGGCTTAAGCGTGACAACGCTGCCATCCTTCAGCAAGTGGACATTCACGACATTAAAGGCAAAATGTTCGTCAAAGCCAGCGGCACTGGTAGTGGGCTGCTCTCGGTCAAGTTGAAGTACAATGTGGTCGTGCCACCAGAAGTTCTGTGCAAGTTCAACATCACGGTTCGGGCTGACATCCACAAACCGGCAGCAAAGACGGCCAAAAAATTCGACGATTTCCCTCCAGAACTTCTCGAAGATTTGTTCGGCGGAGGCAGAAGACGTCGTTCAGTTGGCTCATGGTTTCGAAGTTTTAGAAGGCAATCACCCACAACGACACCATACTACCTTGAGGCGAAGCCAGTGAGGTCTCCATGGGCGGCTGAATCACGAAGAAGGGACTCGCCAGCGGCCCCCTTCTTCCCTGATAGGGACACATTCTCAAGCGGTAACAGTGACACCGCCGCCGTCTCAGTGGAAGGACCCCGACAGAGCAAGCTTACTTACGACATCGAAGTGTGCTCACGCTACATTGGCCGGGAGGATTCCAACATGGCCGTTATAGAGGTTGGCCTGTTCAGTGGATTCAAACCCATGGAAAAAGATCTGGAAGCGGCAAGAGAAGCCAACAACTCCCTTTTGGCCAAGTATGAAATGACTGAGAAAAATGTGATCCTTTATTTCGACAAGATACCTTGGGAATCCCCGACGTGCGTCAAATTCAGAATAGAGCGTCAGCATGTGGTCTACAATGTTCAGTCTGCCGTTGTGAAGGTGTATGACTATTACAACCCAATGCACTCGTGTTCGCAGTTCTACGGGCCCGGATCCACGAGTCCCCTTCTGAAGCTGATCTGTGAAAATGATCAGTGCCAGTGCGCCGAAGCGGCATGCCCAAGGAAAGAGCCCTTCATTGACGTCGAGAGATCAGGAACGACAGTGAAGAAAAGGCAGAAGCTAGTCGAACTCACGTGCCAGGAGCATGATTTCGTTTGGATCGGAATGGTGTCTGCCAACAGGATCGTCAACGGTTACCGTTACATCGACTTCCGTGTGGACAGCGTTGTGAAAGAAGGCGCTGAGAACAGCACCTCCGCAATGACGGGTGTGAAAGTATTCGTGGCGAGGGAGTTTTGCAACACGGCCGACCTCACCAAGGGACGTAGCTACTTCATATTCGGCCGAGACAGCGAACCCCACGAGAAGGACGGCGAAGTTGTCATGAGGTACGTTTTGGACAAGAACGTGCGACTCTTTAACACCGAAGATGGCAGAGCATCCTCGAGAGGAAGTAGACTGTACTCGGTGCTCATGTGGCTCACGTCGGGATTAGCCAGGCAAGGCGGCTGCCAGGGCCAGTGACGTAGCCGCCACTCACCAATCACACGGCTCGCATTTTCCTGGAGGAAATTCCGGTGCCAGCGTGAACGCGCCCACATTTTGCGGCGCTCCAGCCAAGCATCCACCATACGTCATGAGCGCGTCCGCTTGGCAATGGGAAATTCCGCGAATACTCAGCGCCATGCATCATGGGAACTGTGGGAGCTTAAGGTCCTGAAGCCCTAGAGAGGGCACTGCCTCCAAGCTCCAAGACGTAGACTCACGGGGATGTCATCGCCACTGAATTTTGGGCATTCAAATGCATGTCAGTTTACTATTTTGTTTTCATTTCAGAAGTGCCGTTTACTGCATTTTTTGTAAGAGCAAACTATCGATGTGGACGACTTTTCTGTATATGATGCCCTTACCTAAATTCTCCGTATTGAATACTCATTAGAGGCTCTTAATTTGCTTTCCTCCAGCTTTGTTGGACACGCACCACTCTCGATAGCAGCGGTGTTAAAACAGTAATAATATACTGCTTCGGATGAGAAAAGAAAACTAAGCAAACCTACAATCTAGAGGCTGTTCTTTTGAGGCCCCCATTCTTCCATGCAGCGAATTTTATATTTGCACTTTGGTAGAGAAAAAGTCTCCAACAACTTAAATGAGTTGGATACATTCAGTGGTAGCCTCTATCAACCTCCTGCCGATAACACGTTCTGTTTTCACAGAAAAACAATTTGATCAGCCTCTGATAGGCCCTGATTCACTTCAGAGCATCTATCCTATAGTCAAATAACGGATAAAAACTGCCTGTGGTATAAACTTGTCATATCGGCCCTGGGTGTGGGCAGCGCAAAATAT |
| >AAFF3475  NNTTGAAGGATTCGCAATTCTCGCGAGCCTTGACCACTCGCGTCTTGTTGAGAAGGTCCTGCTGTGACTCAATGCCTGGCTTGAGGATTTTGTTGATAAAGAAAGGAACAGTGATGAATCCATCTTTGTGGGCTCCATTAAGGGGCGTGCCAAGCCATACAAAATCAACATTGTCGCAGGCGTGGTATCTCATGTATTCTCGGCAATCTGAGGTTTTCAGAACAGCACCCTCTTCTTTCGTGAACATCTCCTCGACTTCTTGGGGCGGGCAACCACCTTCCAAGCATCTGCAGACTTCAGAGTGACCCTCATTGGCATCGCTGCAGTTTGACTCGAGCATGGGACTGGTGCTGTCTGGCGAGTAGAATTTCCTGCACGATATATCGGGGTCGTAGTAGGCGTACGCCTTCACGGAGCCCGACTGCAACTTGCCTACGGCGAATGCCTGCTGCAGGCCAAACTCGACGCACACGCTGGCGTTCGATGGAATGGTCGACAGGTAGAATACCACGCTACGCTGGCTCAACTCGTACGAGTCCACACGTTTGTCGGCCACCATCTTGTCCAAATCGGCCAACACGGGATTGAAGCCGGTTAGGAGACCTACGTCCAGGATGGCCATGCCTTTCAATTTTTTTTCAAGAGACCTCGCACAGACTTCCATCGAGTAAATCTCTTTGAAACTCCCTTTACCTTCCAACGCTTTCGACACGTTGACCTTGTGCTGTGTGAAGTTTATCTTCAGATCAAACTTGCACAAAAGGTCCACACCGACAGGGGACTCGTAGCTTGTGACAACGTACATAGTCGCCGTTCCAGTTCCTTTCACATTGACAAATATCTTGTCATTGCTGTCTGGAATCTCGATCCTGTTGCGCTGTTGGGCGTTGTCTCGCTTGATTCGAAGGGTGCGGTTAAAGTTACGGTCCCCGCTCAACGTCACTTCACAGGTCAGGTCAATGTCTGCGTCTCGTGCGTAGAGGGCATACTTGGACAGGGCTTGAAGCGCCACCACGGTATCCTGGCTGGACCGCAGCGACCCGCTGGGGTTCATTCGCAGGTTAAGCCATTGCACCATGGCCGTGATGTCGTCCCTACTCTCGCCGGCATTGAGCAGTGCCATGAGCGCGTATGACGTGGCCTGCACCGACAGTGGCTCGGAACCGGCGGACACATACAGCCCTCCTTCATTCCCTCTCTGGAAAATGCCGCCGAGCCGGCGCAGGGTGTCGTTCTTTGCGGGGCTTTTGCCCAAGGAGAGCGCGTACGCTGCCAGCGAGAGCACGTAGGGCGAAGTGTTTGGATTCAGCTGTCCCTCGATGAACCCTGTTGCTTTTGCGAAGGACTCTGTCAGGCCGGCCACCTGAACGCCTTCTCGATCGCATTCTTGCAGGGTGAGAAGGGTGTACGCGGTCAGTGGAACCGGTCCATTCACACCGCCCAGGAGGTCACCGTGGATTAGGTTGGATATGTCGTGGAAACTGCCGTCCTGCTTCTGCTGGGTCAAAATGTAACGGAGCCCACTTGTGACGACCTTCTCGTCGATGAGGATAGACTTCCGGGCTTCGCACAAAGTTCTCACCACGAATGCTGTTAGCCATAGACTGGACCTGTAATGATTCCAAACCGCGAAGGACCCGTCAGGTTTCCGGAAATTGAGGATCCGCTGGTAGCCACTGCTGATGAAGCCCAGCGCTCTGTCCTCGTCCACGGGCGATATGCGGCTGGCGGTCTTCAGATACTCGTAGGCGTACAGCGTTGGTGCCAGGCCGATCATGGTCTGTTCACCGCAGCCATGTGGCATCCTCAGAAGCGTTCCCGGGTTCTTTACGGACGCCTCCAAAGCAGCGCCCATGTCATCACCAACGATGTCGATCTCACAGCGTTCGGAGCCAGGAATCACTTCCTTAGGGGATCGGCCAGGCCGCTTCATCTGGATGAGTTGCTTGCCTCCAAGCCCGAACTTTTCTTCGTAGCTGTACGTGGGGCTCGCGACGTTCCGCGCTGGCCGTTTCTGTGTGTTTTGAGGGTCGAGAATGAGATTGAAGCTCTCATTCTTGCTGACACCAGGTGGCTCTACCCTGAGCATCACTTTGACAGAGTCCCTAGCTGACCCATCGCTAAGGGCGGCCACGCGGATCTCCCTCTCGCCAGCAGCCAACGGCACCACGGGGAAGACCACCGTGCGGCCCTGTCCCGCCGGCACGGTCAGCGTGCGCACCGATGAAGGCTTGCCTTCTTTGACACCCGAGCACACGTCCTTCGTCCCGAGCATCGCCACCCTAACCTTGAGATCCTTGTGGTCGTAGTTGTACACCGTCGCCGGTATCTCGATCTGTTCCTCCTTGATCACGGAGTAAGGGAGGTTGACTTCGACGAAGAGCTTCTTGAAGACCGGAATCTGGAGAGGATCAAGTACACATATCCCGCCAGATGGGGCCACGCCCACAGCGCTGACCTCCCACGTGGTGATCGAGTCTGGGACGGTCACCTCAAGGCTAGCCTCTCCATGTTCCCGTAGGGTTAGTTGATTGAATATCCACGTCTCTCTAAAGTTTCGCCTTTCTTCACCCTCTAAATCTTTGAAGTTGCTCATGCCCAACACGTCGGCTTCCTCTGCAGATGACCTTCCGACAGCCTGGTTGTCTTCGACATGGATGCAGCACTTGCGAAAGGCTGCGACACATTCTATCGTGTAGAGGGCCACGCCGGCATCCAGATAGCTGAGCAGAGCCTCCTCGCGTTCCGAGCAACTGCGTAGGAGCTGGTCAGGCTGCATCCCCCGCGAGCAGCAGTCTCGGAGCGTCTCGTTTTCGTACTCTTTCACTATGTCCAGCTTGATTTCACGTTTTGCGCGCCGTCTCTCTACCGCTGGTGCGCCGTAGCTGGCCTGGGATGTGAGCAGCACAACGCCCGCATTCGCAAGGGTTTCCACCGCTTTCCTGCCAGCGTCCTGTCCGTTGCCCATGTCCTTCGCTTCCATGGACTGGAACACCTTCTTTCGCGTTAGGAGGTCCTTGCGTCGCAGAAGGTATACTGCCTGGTCCACGCCCAGCAAGCCAACTCGTGTGCCTTCTCTACCCTTCAGCAAGAGCTTCTCAATAGAACCGGGTTCCAGCACTGCTCCAGGGGTTCTCCGGGTCAAAGTGAAGTGCGAGTCCGTCGTGCACGTAGGCACAGCGTTGACGTAGACGGCGTCTGTGACCAGATCGTTGTCCACAAAGGCGACCACCAGGAGTCGGAAGCTGGGCGTCATCTCCGGAGTCACGACGAAGGCCACCTTCGGGTCGAGGACACCACCTTCCGTGGCGATCTTCGCTGCCTCCATTACCCGGCCGCGGGAGAGGACCACGTAATACGCTGATGTCAGCTGGGAGTTCTGAGGTTTGAAGATCGAGGCCTCATACGTGGAGCCCGCCGCAACTAAAGCCCTGGGATCCTTCCTCTCGATGGCGATGTATTTTGTTGATCCGTCTTTGTATGCCTGCAGCATGACAAAAGCTTTGGCCTGCTGATCCTCCGGAAAGTGCTGGTCGGCGGTTTCCACCGTAAACTTGAATTCACCGTCACCAGACGTGGAAATTATGAGGAAGGTCACCACGCCGTTAACGTCCGTCCTGTGACTCATAGTTTGCACTCGACCGTTTTCACTCGTGAGTCTTGTCGGCACGCGCGCGGCTGGCTCACCGTTGGTGTACGTCACTTCCGCCACCACGTAGAACTTGGTGCCCGGCTTGAAGCTTTTCTCGCTTCGTGCTGTCGATATCAGGTAAGGCGAGGTGGTGAAAACGGCCTGCTCATTACGCCCGGTCTCCTGGATGCCCGTGGCCTCC |
| >AAFF8830  TTTTTTTCGGTGATCCATCACTGAAGTTTATTGCTGCGTTTTAAAGCTGGGGCAAGTCGTTCGCATCGAGAAGTTTCGAACACAGTTCGTACTCGCCAATGTTCGGTGCATGAATGGTTGTTAAACGAACGCACGCCATCTGTGTGTTATGTTAATGACAATACCGGTATTCTGCTGGCGAAAAATCACATTTACATTACCGTAATTGCCATAGCCATGAAAAGGTTCGGGCCACTCATAAGAAACAGCGAGTTCTCTCATCGGAAAACTCATTGATGAACCACGCCGCAAGTCTATTCTGTTGTTTATTCTTGGGCTTCGCGGGGAAAACCATCGAGCTGCTGTCGATCAGATACAGGAACTGCTTGGTCTCGAAGAAGTCTTTCTCGATATACGTGGAGTCTTTTCCCATGACAATATACTCCTCGCCATTGGGAATATTGAATGTATTGCAGGTATCACGAGCCTTGATGCGACGTATCTTTCCTTTCAAGTCGTCTTCTTGGCCTGGCTTGAGAACTTGAGTGATGAGGAAAGCCACTTCTATGAAGCCATCCGTCGATACATTGGCTGTCGAATTGCCTCTCCATACGTAGTGAACTCCGTCACAGGCGAATTCTCTCAGAAGCTCACGCTGTTCTTCGTCTTCAAAGTATTCGTTGTCCCGTGTTTTTATGAACCTGTCGAGTGGCTTCTCCGGCGGGCAACCACCTTCCGCGCAAACGCAGACATCGGAGTCGTCACACTTATCATCAACCTTTAGTAGGGGGCTTGTCTTGTCGGGCGAGTAGAATCGTGTGCAAGAAAAGTCTGGTTTGTAGTAAGAGTATGCCTTCACGTAGCTTGACTGCAGTTTACCTGCATTGAATTCTTGTTCGAGAGAGAAATTAACGCAGTGCGTCATGTTTGCAGCAATGAAGGGTACGTAAAAGTCAACATGGCGACTTGTGATAGTATAAAGCTGAATCTTTCTTTCTTTCACGAGCTCATCCAACTCGGTAGCATTGGGCTTGAATCCAGTCAGAAGACCCACTTCCAACATGACCATGCCATCTGGAGCATCCTCGAGTGGACTTGCACAGGCCTCCATGCGGTAGTTTGGCTTCAGGTCAGATGGTGGACGTGAGGAGGTTTTTTCGACACTTCGAGAAACTCGTGTGAGTATGAGTTCTATGTCCGGTTTCGTTTCCACAAAGTTTGCTGTGATGTTAAACTTGCAAAGCTCACCGTCAAGAACTTTAGCGTCGTATGTGTAGTTAAAGTACAGGATGCCGGTACCGGACCCTTTGACTCTGACGAAGATTTTCTCTCCCGGTCGATCAATCTCGATCTTATTCAGGATGGTGGCGTTATCTCGCTTGATCCTAATATTCTTCTTGAAAATTTTGCTGTTGCTCAAGGTCACCTCGCATGCCAAGTCGAGGTTGTTTTCTTTGGCGTATGCGGCAAACTTGGTGAGCGCTTGCAACACCATCACAGTGTCCTGCGTAGATTGAACAGACCCAGTGGGGCCGGTCCTTTTGCTCAGCCAACGCTGAAAACTTCTTATGATCTCGCTTTGTGCGTTCTCCCTGATGAGCGCCATGAGTGCATATGCGGTTGCTTGTGCAGACAGCAGTTCGCTGCTCGCCGGAACGTGTCGGTCGTCTGTGACTTGGTCATAGCGCACGCTTTCCATCAGCCACTGGATCAGTCCATCTTTCCCCGTGTTGTTGGCCAAGGACAGGGCATAGGCAGCCAGCGCCAACGCTCCCGGTGAGTCTCCGCGGTGCAAATTCCGCTCCAGAAACGCCGCTGCTCTCGCCCTCGAGGTTTGCGAAACCGAGTATCCTCCTTCAGCGCACTCCTCGAAGGTGATGAGAATGAAGGCAGTCAGCGCTGATGGGTGCCCGAAATTTAGCACTAGTTCGCTGAGGCTATATTCATGGAAGCCCCCATTTCCTTGCTGCCTGCTAGTTATATAGCGCAGTCCGCTTCGGATGACGTTTTCATCAATCATGACGGATTTCGTAGCTTCGCACAAGTTGCGAATGACGAAAGCCGTCAGCCATGGGCTAGCACTACTCCATTCGAAAACACTGAAAGAGCCATCCGGTTTCCTGTACTTGAGTATTTGGTTGTAGGCTCTTCGAATATAGTCAAGAGCGTCGTTCTCTTCAGCAATGCTGATGCGGTTGGTGGTCTTGAAGAACTCGTAGGCGTACAGTACTGGCATCAGCTTGGCCGTGGTCTGTTCTCCACAATCAGAGGGATAAATGAACGCTTGGTCCGGCTTCTTGATTATAGATTGAAGAACGGCAGTCACTCCGTCTCCGACGATGTCGATTTCGCAGCGCTCTGTGTTTGGCAAAGCAAAATCAGGACGTGGCGACCGAATGTGTACCACCTGCGTTCCATTTGTACCAAAGGTCTCTGTGTATTCCTCTTGGATGTTCCTCGTGCCTCGCTTTTTAGGGTTTTCGGGATCCAAGATGACGGCAAACGATCGTGTTCTCTGAACTCCCGGAGGCCGGACATTCAGCTCCACTTTGACCTCGTCCCCTTCGCCGCTGGTGCTGCGTGCCTTCACGTGGATCTGTTTTTTTCCTGCGGCAAGGGGCACCACTGGGAATATGGCGGTGCGGCCATGTCCGGGTGGAATCTCGAGCACCCGGACCGCGGATGGTTTTCCTAGTTTGGCCCCAGAGCAGATGTCGTTAGTCCCAAGCAGCACCACCTTAGCCGTGATTTGTTTCGTGCCGTAATTGTAGACGGTGGCCGGGATCTCGACTTGCTCTTTCTTGACGACCGAGTAGGGAACGTTGACCTCAACGAAGAACTTCTTCGTCGCCACGATTTCGAGAGGTTCCACCGCGCAGACTCCTCCGCTGGGCGACACGCTCACAGCGTTCACCTCCCATGTGGTGACCGACGCCGGCAAAGTGGCCGAGAATTCGGCGGTGCCGTCGTCCCTAATTGTC |

| >SG481232  TTTTTTTTTGAAAAAAATTAGTTTTATTCACTCTTTATGACACTATTTATAACGGTGAAGCCTCGCAGACGATGACGCGCTTTTCTCGCAAGTAGAAAATATATAAAGCTACTCTTCCACTCAGCTGGATAAGACGCGCTACGGGAAACAACATGGCGACGTCAAAAGTTCACGGCTCTGTTCACGCACACAAAAGCACACACTGCGCAGCGCTCCCTCAGTCGTCCGCACCCAATATCGAGGCTGTAAGATAAAACATCATGTGACCATTGTCTTGATTTCTTCGCGATGTGCGCTGTGGAAGCCATTAATCTTTTTTTTCTTTCGCGACATGGNNNNNNNNNNNNNNNNNNNNNNNNNNNNNNNNNNNNNNNNNNNNAGAAAGCATGATTCTGGTAAAAAGAAAATTGGCAACCAAAGGATAAATGCGCATTGAATGAATGGTATGTTGTGACTCTTATTGTCTTGGCGCGTTCGTTCGAAAAAAAGAGACATAAAAAAAAGGGAGAGTAAGAAAAACTGATGGCCGATGTTCACTAGCAGGACGCCACAAGAGAGTAGGGCACGGAGTTGCTGTTTTCTTGCTCATAATAGTCGTAGACTTTAACTGTGGCAGGTTTGGCATCTTCAATAGCGAACTCGCGGTGAACGCGAACGTCGAAGCACTTGTTTTCGGACGTTATTTCTTCAAAGTAAAAGTTAACCTGGTTCTTTTCAACTTCATGCCTCTTCAGCTTGACGTCTTTCTCACGGTAGAGTCCAAAGATGTGATCCTCATCTGGAGTGTAGCCCGATACCAGCTTGAGCTCCACCACTGCCATGTTGGATGGTTGCTCACCGTCGAACCTCAGACAGATTTTGAGCTTGTGGTCATTGCAGTCAGACGCCTCTTGAGATGGAGTGGCTGTGAGCTCGAAACCCTCGCTCTTCGGTGCAGTGTGAACGTTGTACTTCAGAGTCGCCGATATAAGGGCACATCCAGGACCTGTTGCTTCTGAAGTGAGCTTGTTGGGCAAGCTGACAACCTTCTTCTCCTGTACGACCAGTTTGGTGTCCTCCTTAAGGTTATACGACTCACTGACGTCAGTACCGTCGACCTTAACTGAGATGTCAACGGGATCTTTGCTAACGTAAGTAGCGAAAGCCGACAGAGCCTGGAGACCGAGAACTGTATCCTGTGTGGATGGGAAGCCTCCGCGGCTGTTTCTCTTGGTCGCCATCCAGCGGACAATGGGCTGCGCCTTGCTCAGGTTCTCTTGGGCGTTCAGCTTGAGGTAGGCGAGCACTGCATACGCCGCCGTTTCCACGTCGGCCGACGCTGATGGACCCTTCTTGCCCGCGTTGCTCCAGTAGGTCAGGGCACCTTTGTGAACTGCGATGGACTCCAATTTCTCGAGGTAATCTTTGGCAGACTCATGTCCCGCCAGGGCCGCGGCGTAGGCGGACAGGGCCAAGTTGTGGGCACTGGGGTCACGCTGGGCGCTGATGCAGCGGAGCGCAGACTCAACGACCTTCTCGTCCGCAAGGCCGCCCTCCAGAAGAGCGGTGAGCACGTACGCCGTCAGCGCTCCCGGGGCAGTGGAGTTAACCTTGCCCTTCAAGCCGGAGCTAAGAACAGTGCCGATGTTCTGGAAGCAGCCGTTGGTTTTCTGCTTCGTGATGATCCACTTTATGCTCTCGTTGAGGTTTCCGGCGTCAATAGGAATGTATTTCTCAGCCTGCTTGAAGGACTTGACTACAAACGCCGTGAGGAACATGCTGCCCGAGTTGTCGCGGTTTCCAAAGGCGCTGTACGAACCATCGTAGTGCTTGTACTTCTGCTGGCGCTGGTAGCCTGTCTTGAGATTCTGCACAGCCTTCCTTTCGATGTCTTCCTGGTTCTTGCCCGTAGCCTTAAGGTAGTCGAGGACATAGACGTTTGGCGTAAACTTTACCATGTTCTGCTCACCACATCCCGTTGGAACTTGCACCAGTGAGTCGAGGTTCTTGATTGCTGGGCCCATAATATCTCCTGTGACTTGAACGTAGGCACGGGCCGATCCTTCCACCAAGTCTTCAGGAAGCACCAAGTCAAATTCGTTCTTTGCGCCGCCTTCTCCAGCGTCTTTAGGACAAACAAAGACACTCTGCGTCTCTTCCTTAGGGAAGCCTTCCGCCTCGATGATGAGCGGTCGTGTGACTGCGTCCCTGGCCACCAGCTTCTCAGTTGGCTGCTCTCCACATGCGGCATCGCTCTGCGATCCGGCCGCCGACACGGTCAGGTTCACCTCTCCGATAGTTTGTGGCCGGATCTGGAACTTGTGCACTTGGCTCTTGCTGCCGCACACGCAAAGCGTCGTCTCAGACTCGCCCTCAATGTGGAAGTCCGCGGATTCAGCCAGCTTCAGATCGACCGGCAGGCACTTTTCGAGGTAGTTGAACACGGACACCTTGACGGGCACCAGTTCTCCGCGCACCACAGAGTAAGGCAGGTTGAACGAGGCGAAGAACGGCTGGAAGGCCTTGATCTTTGCCGGATCTGAGATGCCGATTCCGTCCTCTGAGTTGATGCACACAGTGCTTCCCACCCACTCGGTGATGGTGTGCGGAATCTTTTCTTTGAAGTTCAGTTCGCCGTGCTCATCCAGCTCCTTCAGGTCCCACAGCCACGTCTCGGGGAAGTAGGTGCGCACTTCAACGGCCGACTTGGCCGGGATGTTGGCCGATGGTGCGCTCTGCGCCACAGAGTCCAGGGCGACGGGCACGCCGGGCAGGCCACCTACAATGCGGTTGGAAAAGGCCACCCCAGCCGGAGGTGGAGGACCCGACAGTGCGAGTGGTCTCGCTAGAGGGATCGCTGGCGCCGCGAGAGCGTAGGGTGGTCTGTCGTAGATGTTCTTCCGGCATGGCCTGGTTTCAAGCGTCAAGTCTGACATGACCACCACCCCTGATTCGTCGAAGGCGGTGATCGAATCGACGTATTCAACATTTGAAGATCTCGGTCCATTCCAGATGTGGCGCTTGAAATGTTGCGGATTCTTCGCCAACTGCTTGCGACAGTAGTCGTACGACGCCTGCTTCGGCCACGTGTAGCGGGTGATATCCAGACGCTTCAAAATGTCATAAACTTTGTCCTTTGTCAGTTGGTTATCCTGCTTCAGGAGGTGAACACTCTTGTCTACGACGCCAACACCACAGAAGGACCGAGGAGAACCATTCACATGGATGGCAGCAGATGTTGCAGGCTGTACAGTTTCAGATCCGAATCTCATGGTTACGTTGTTCTGAAGGCACTTCTCCACTTCGAACTGCTCGGAGTCAGCGATGACCTCGCCGTCGGGGCGGACGTAGAAAGCGAGCACTTTGACGCGTGGCACGTGGCTGAAATCTGGCTCGAGCTCGAACTCGAAGCTCCCTGTCGACACGCTGCCCTCGGCCATGTTGGTCGGAAGCGTCTCATTCAGGTCCTCCTCCACCAGGAAGCTGTCGTCAACAGAGAGCGCCTCTTCCGGCTTGAAGGTCACATCCAGAACCTTGTCCTTGAGGATCTTGCCGCGAGCTATCACCTGTAAATGGAACTGCTTCTCTGAGTCGGGCTGACCCGTGTACCGGAGGCGCACGGGGTGCTTGCCCGAGCAGCGCAAGGGTCCCTTGGACGGCTCGATCTGGATGAAGTTGTTGCTGGCCGAGTACCAAGCCTGGAGGTACAGAGTGCTCTTGGGCTGGTTGATCTTCACGCCGTAGGTGTCGTACTTCACAGTCTCGTAGTTCATCGCCACCGCCTCGACACTGATGGTGACCACAGTTGTCTTCAGGGGAGGGATGGTGAACTTGATGATGCCTGACTCGTCCGAAGTGTAGTTCTTGCAGCTGAGACGGCGGTCGGTGCGCCACCACCGTGGCTTGATCTCCTCAGCCTGTGACAGCAGACAAAGTTGCACCAGCTCGCCGCCCACAGGAACGCCGTCAGGCTTCTTCACCAGGAGCCTGCCGTAGAAAGGCATGGTGGGCTTGAAGTAGTTCTTTCCGCGGTCTCCTTCCAAAAAGTTGAGCTCAAGCGGGTTGAAGCTGCGGCTGATGTAGTTCGTCTTGTTCATCGTGATGCCCGTGCCGGTCTCGTTCACTTGTGCGACGAGGTAGATGCGCTTGTAGATCTCGTAGTTCTCGTTGAAGCGCAGCAGGCTCGTGTTCACCGTCATGTCGAAGCAGCCGTTGATGGGTCCTGTGTGGTTGATCTTTGGGTAGTCGTCCTTCTCCCAACTGTATCGCTCGTATGTCACGTTCACCGTCAGGGTGCCGTCCACTGGCTGTCCAAAAGTATAGTGGGCACAAATCTTCCAGGTGATCTCCTTTGCATCAGCCAAGACGTAGGATGGAGGCTTGATGGTCACCTCAAATTTGGGAAGTACGTATTCGTTCACCTCAAAATGCTGCCTCACGGTTTGCGTGGGAAGTTCGACGACTATCTGCCACAGACCCAACTCAGGCTCTTCTGTAAGCTGAAAGTCTCTCTGGACAATGCCTTTTTCAAAAGACACGTCGTTCCACTGAGCTATTCTTACATCGCTGGGACTTGTCACGTAAATAGTTGCCTTCACATCGGTGACTGGTTTCAGCTCATTGTTGATAGGGAGGACGCGGAATTGAACCCTCTGTCCAGGCTTATAAAGTGCCTTATCGGACTGAACCAGGATCGTGTTCTTGGACTTCTGGAAGTCGATCTCTTTCCGATCGCCGAAGACATAGTCTCCAAAGGTTCCGTTCACTTCGATCTTGGCTTGGCTGTCGAGGTGCTCGGGCACTCGGAACGGCAGAAAGGTGCTTTCACCGTTCTTGATGTCGTACTCTTGTTCCGCCAAGACGATGCTGTCGTTGTTGTACTTGAGCAGGCGGACGATGACCTTGCCATCCTCCTTGACGTCTGTGAGCGTTAGCCGAAACTGTTCGTCCGTTTCGCTGCGCAGGATCTTCGGCGCCGTGAAGATGTACCCACTTTCAGCAAGGCGCGATGCCAAGGCACCAAGGACTAAAATGAAGGTGCCCCAATGCATTCTAGCTCTGGCCACCGGCACCTCTCGTCACCGTCTGTGCTAGTTTTCTTGCGTCTAAGCCAGCCTTAGACACCTTTCCGTAGACTGTACAGCACAGCTTTCTCTGCTGCGCGCGCTAAGGCCGGTTCCGGGAGCTTTTTCTTGCTACCCTTCTTGTCTCGCTCCTGGCTTGACGTCGCGTGGCGAAGCAAAACACAACACACGCGCTCTAAGGCGGCGGCGTTGGTGCGCAGAGAGTGCTGCGAGACGAGGCCGACAAGACCCTCCGACAGCCCAAGCCCGAACTGATCCGGCGGCTGCTCGCCG |
| --- |
| >SG4844567  GTAATGCCACATGGGTGCGGAGAGCAAAACATGCTGGATTTTGTACCTAACGTTGTTGTTCTTGACTACCTGAAACGGGCCAACCGCCTCTCGCCACCTGTCCAAAACAAAGCTATACGGAATCTTGAAGATGGCTATCAGCGAGAACTGACATACAAACGCGATGATAATTCATTTAGTGCTTTTGGCAATACAGACAAGAGTGGCT |
| >SG482943  TTTTTTTCTTGAGAGGATACATTTATTGAGGATGAGAGGAGCGATTGCTAAAATACAGACGAACACAAGGTATCACAAGGCAGATGACTCGGAGGCACTTGAGATCGTTTGTCCAGATGCTTAGAGAAGTGCGGATGGATGGAAAGGAAGTGAGAAAAGATCTCTAAACGAAGAACACGCTGTCCACTTCGTTGAGGCTCGTGGGTGGCGCGACTTCAGCGGGCGTGCAGTTGCCCTCTACTCTGTAGCCAGCCGTTGCCTTGTACTGGTACTGGTAGTAGTCGTACACCTCCACCACTGCATCCTGAAGGTTCGCCACTTGCAGGTACCTGCTTTCCGTTACGCTGAAGCACGTCGGCTGGATGGTCAGCGTCTGAATGATGAGCTGGATTTGGTTGCCGTTGACGACATACCGCTTAACGACGCCACTGCTCACGAGATTTTTGAGAGACTGGTCATCTGCCGAGTATCCAGAAAGCAGGGTGATCTGGACGATCGCGGAGCTGCGCAGAAAGCCTTCGGTGTACCGTGTGCATATTTCCAGTTCAAGGGTGCTGCAATCTGGTCCAGATGTCGTGGACACGTTGACTTGTATTCCTCGCTGAACCGGACTGTTCCTGGAGTTGTAGTAGTACTTAACCTGTAAAGCGGCGCACCCTGCGCTGCCGTCAGCAAGAGATGCCTTGAAGCTGTACGAATCGCTTCTGTTCAAAAGACGCTCCTGGTATAGCAGTTTGTTCTGCTCAGAGATCGGGAAAGAGACCGTTTCGGGGGATGAACTGTCGACAGCAACGTTCATAGTCAAGTTTGTGTTCTTCGAGAATGCCACGCTCGAGTACTGGGTCAGGGCTTGGATGGCGACGGTGTAGACCTCGGAATAGGAGAAAGTGTATCGGGCGTAGGTCTGCTGCATGAGCCAACGGACGATTGGCAGCGCTTCTCCCAGGTTTCGGTCGAGCAACTTCAAAGTGAGAACTGCGTAACCAGCGGTGTCTGCCGAGCCGTAAGTCAGGCCATCGCCGCTCCACGACGTCAAGCCACTGCTTTTATCAGCTTCGCTCAGAAGCGTGTCCAAGGCGCTGTTAGCTCGGTCCGTGCGTCCAGCGAGCGCCGAAAAGTAGGCGTTCAGCGCAGTCGTGTGGTTGGAAGGCAGATTGCTGGCGTCAATGCACTGGAGGACACCGTTCGTGATGTTCTCGTAGTTGTAGCCACTCTCCAGAAGCATGACGCCAATGGCCGCGGTGAAGTCGGGGGCTGTCTGGGGGCCGAAGGGTGACGAGCCGGGCTGGTTCTCAACGAAACAACCGGTGGCAGCGTTCCAGTGCTGCAGGACATAGCGCACGCTAGTTTCGATGTCCGCATCGACGCTGGTGCCCAGGTACTCCTTGGCGGCGCTCAGGGCTTTCACGGCGAAGGCGGTGAGGAACACGCTTCTCGGGAACTCGCTAGAGCCAAACTGAGCGTATGAGCCGTCGGAGGACCGGAAGCTGTTTTGAGCCTGGGACGCTTGGCGAATGCGGGATCGGAGGCCGGAGTTCACCGTGTCAGTCAGAGTCCCCGTCTGCTCCAGGTACTTGTGGAGGTAGACGCTGGAGGCAAGCACAGCCAGTGTTCCCTCGGCGTTGGAATAGGTGATGGTGGGAACGGATAGGTCGTTCAAGCTGAGAGCGAGGATGTCGCCTGTGCCCACTAGTACAACCTGCTGGGAGCCTTCAACAAGAGCGACAGGTGTCGGAAGGTTGACTTGCAGGGTGTCCGGAGCGCTGTCTGCATCACCTGAAGCACACAGGAGGAACGTGTTGATTTCCCTGATGGGGAAGCCTTCAGGCCTAACATCGATGGTCTGGATGACAGTATCGCTTGAGTTGACGTTGGACAGTTTGACGTCTGGATACTGTGCCTGGGCCTCTGGTCTCGTCTGGACGCGGGCTTCCAGCCGGGATTCCTCAAGCGTGGTAGCGCGCACGTCAAACGGGAATGACGCGGATGTGCTGGAGTTGGGGCAGAGTAGTGCGTCGGTGCTGTTGGGACCACCGACCACCTCGAGATTCTCCAAAGACCTCAGACTGACCCGGACCGCGACGCACTCTTCGCCGTAGTTGAATGCCGTGAGAATGACCGTGGCCACTTCGTTGCGCTGCATGTACGCCGGCAACGTGAGCGATACGAATAATGGCTGGAATCCGGTGACGTTCGCCACTTGGGAGACGCCGAGGCCGTTTCGCGGGTGCAGGCATACGGCAGTGCCCTGCCATGTGGTGATTGTGTCCGGGAGCGTCTCGGAGTACACCAAGGAACCATCAGGGCTCACTCTCTTGATCTGCCAGAGCCATGTTTCAGGGAAGAGTGTGCGGACGGAGTTTGACCCCTGGACGACGGGCGAAAAATCGGCTTGCGCAGACTCTGCGATAGGTGCCGGTCCAGCAAATCCGGGGGAGGCACTCGGAGCAAATGCGACTTTGTTTACAGCTCCGAAACCGCCGAAACTGGGATAGTAGCGATTTGTCGATCCCAGGACTTGATTGTTGGGATAGCCAGGGAAAGCACGGTTGGGACATGGCCTCGTCTGGATTGGCAGGTTGGTGAAGACGACAAGACCAGTGTTCTCGAACGAGCTCAGGGAGTCGTAGCTGAAGGAGACTGAAGGGGCCACTGTCTGCCTCCTCCTCCGCACCTTGAGCTGGGAGTCACCCGACTTCTCGTAGGGTTCGTTGTAGAGATAACCGTCAACATAGCAGTAGCTCTGGTTGACGAGAGAAGTGCGGGTGTACCTGTAGTTGAAGTAGCTGATCTGGTTGAGGAGGGCGTCCCTAGAATTGCGCTGGTTGTAACCATCCAGAAGGGTGACGCTGCTGTCAACAGCTCCGACACCACACAGGGAGTCTCCGGCAGCAGCAAGAGACAGCGACACGTTCGAGCCGGGCAGAGCTTTCTTGGGATCAAACTCCAGCGACACGTTGTTCTCCAGGCACTTTTGGACACTGAACTCCGCCCAGTCCGAGACGACTTCGATGGGCCTTTCATTGGAGTCACGGTGCACGTAGTACACAAGGAGTTGCACCTTGGGGCTGGCTTCCGCGGGTAGCCTTGACTGGAACTCGAAGCTGCCCACCGACCGCACGGTCTCTGGATCTACCACCTGGGGCTCGGAGCTTCCAGCTGGAGGGTCGCCGTTTTTGATCTCGTTGCCGTTGGACTGGTCCATGGTGAGCTCGGCTTCGGTGAAGTGCTTTGTGAAATTCTGCGTCTCGAGGATGTGGCCGCCCGAAGTGAGCGTGAGGAAGATGTGGTAGTCGACGTTGGCGTCGGCAGTCATCACGATGGGGCGCGTGAACTGGGCCTGGCACGTGTACACACTGCCGTCTCCTCGGTCGATGCGGATAGAGCTGGCCGTGGG |
| >SG4826398  ACGAACAGCGGTTCAGCATCCCTAGGTTTGAAATGTATCAAACAAACAGATATCAGCGGCTATATACCAATTCATGGCTTTGGAAAGACATCAACATTGGNNNNNNNNNNNNNNACATCTTCAACATCCCAGTTCCTGATGTGCCAACAGAATGGATGATCAGTGCATTTGGAATGGACGAGTCCAGTGGATTTGGGATTTTGTCAAACAGCATCTCATTTTCAAGTGCAAAGCCGTTCTACATGAACGTAGAAATGCCAACAAAGATTCGTCTTGGAGAGCAGCTTGGTATACGTGTCACAGTCTTCAACTACAGGCTCTATGAAACTGAGGTACTTATCACACTGGCTAGCTCCCCAGATTACAGATTTGTCTTGGTGGGACCTCTAGGCCGGG |
| >SG4822289  CGATGTGACCGGACAATTCGGCAGCGACCAATTCCAAAACAGATCGTCTGTTCAACTGGAATACGTTACCAATGTCAACGTCGTTGTTCAGACGGATAAGCCCATGTACCGTCAAGGAAGCACAGTGAACTACCGAATACTGCTGCTTGACAACAACCTCCTCCCCGTGACGAATCAGCTGGCCAACATTACCATCACGAACCCGTATGGCCAGCTCTTGTTCCAGCAGCAGTACGTCAACTTCACCGACGGCCTCCTTCAAGGCTCCTACGACCTCCTTGCCATTACCGACGAAGGCACGTGGACGATCAGCGTGTCGGCAGGCACAAACTCTGGTTCGGCAACGTTCCAAGTTCAGGACTACGTGCTGCCAAAGTTTTCCGTTACCATCACTCCTGACACCAATGACGTCGTCACGAACCCTACTGTGCTGTACACTATCTGCGCCAAGTATACCTATGGCGAAGATGTCAAGGGCACTGTGCAAATCTACACATCTCCCTTCAACTACTACTACCCAGTGCGCCAGAAGCCAGTCATCCTTCGCGTCGCTGAGATCAACGGCTGCTACGACTACGTCTTCAACGTGTCGCTGCTCAACACCGTGAACTACACATACCCCACATACCCTTCAATCAACGTCACTGCCAAGGTCATCGAAGACGGCACAGGTGTCTCTCAAACTGCCACCGAGCTGCACAGCCGTGGTACCACACGACTGAGGCTCACCTTCGGACAGAAGTACAATTCCAACAACCAATACACTAGCACAGACAACACCTTCAAGCTTAACCTCCTCTACAAGGGACAGCTTTACGTGCAAGAGTTGGATGGAACTGCGCGCCCGAATGAGCTAGTGCAGCTCTGCATGTTCGTGGAGCGCAACACGTTCAAGTGGCGCTCATGGGAGACGGACAACGTGCTCGCCTGCCGAAACTACACCTCCGATGCCGACGGTCTCGTTGGCTTCAGCCTGCCACCTTTCAATCAGAGGGTCACTGGCGTCATTGTTCAGGCCATCGCTGTCAATTTCCCGCGCATCGTAGTCAAGAATGGCCCCACCCTGGAGAAGCCCTCGGCGCAACTGAGCCTTGTCCCCTTCTACTCGCCCACGGCCAGCTCTATC |
| >SG485689  TCGACAGCGACGAGCCTTACGTCGCGGCCCTAGTTGCTTACGCGCTTAGCCAAGCCAACGACACGGAGAAGTACGCCACCCTGTCCGCACTCAAGGAAAAGCTCATCTATGACCACATGCTCAGCACGAGAAGCACGGGAATGGAGGCCAGTCCCCTGGTCGTCGAAGGCACCGGTTATGCGCTTCTAGCGCTACTTGCCCACAACGACATCGAAACGAGCAAGACAGTGGTCAACTGGCTCAATACGCATCGCTCGGCGTCCGGCGCTTTCGCATCGACACAGGACACCGTGGTGGCCCTTGAAGCGTTGACGGAATTCGCTCTGAAATCTCGAGAGCCAAATGTGGACCTGACGTGTAATGTCACCCTTAGCAGTAAACGGGGCTTCCAGAAAAGCATTCGGCTTAAGCGTGACAACGCTGCCATCCTTCAGCAAGTGGACATTCACGACATTAAAGGCAAAATGTTCGTCAAAGCCAGCGGCACTGGTAGTGGGCTGCTCTCGGTCAAGTTGAAGTACAATGTGGTCGTGCCACCAGAAGTTCTGTGCAAGTTCAACATCACGGTTCGGGCTGACATCCACAAACCGGCAGCAAAGACGGCCAAAAAATTCGACGATTTCCCTCCAGAACTTCTCGAAGATTTGTTCGGCGGAGGCAGAAGACGTCGTTCAGTTGGCTCATGGTTTCGAAGTTTTAGAAGACAATCACCCACAACGACACCATACTACCNNNNNNNNNNNNNNGTGAGGTCTCCATGGGCGGCTGAATCACGAAGAAGAGACTCGCCAGCGGCCCCCTTCTTCCCTGATAGGGACACATTCTCAAGCGGTAACAGTGACACCGCCGCCGTGTCAGTGGAAGGACCCCGACAGAGCAAGCTTACTTACGACATCGAAGTGTGCTCACGCTACATTGGCCGGGAGGATTCCAACATGGCCGTTATAGAGGTTGGCCTGTTCAGTGGATTCAAACCCATGGAAAAAGATCTGGAAGCGGCAAGAGAAGCCAACAACTCCCTTTTGGCCAAGTATGAAATGACTGAGAAAAATGTGATCCTTTATTTCGACAAGATACCTTGGGAATCCCCGACGTGCGTCAAATTCAGAATAGAGCGTCAGCATGTGGTCTACAATGTTCAGTCTGCCGTTGTGAAGGTGTATGACTATTACAACCCAATGCACTCATGTTCGCAATTCTACGGGCCCGGATCCACGAGTCCCCTTCTGAAGCTGATCTGTGAAAATGATCAGTGCCAGTGCGCCGAAGCGGCATGCCCAAGGAAAGAGCCCTTCATTGACGTCGAGAGATCAGGAACGACAGTGAAGAAAAGGCAGAAGCTAGTCGAACTCACGTGCCAGGAGCATGATTTCGTTTGGATCGGAATGGTGTCTGCCAACAGGATCGTCAATGGTTACCGTTACATCGACTTCCGTGTGGACAGCGTTGTGAAAGAAGGCGCTGAGAACAGCACCTCCGCAATGACGGGTCTGAAAGTGTTCGTGGCGAGGGAGTTTTGCAACACGGCCGACCTCACCAAGGAACGTAGCTACTTCATATTCGGCCGAGACAGCGAACCCCACGAGAAGGACGGCGAAGTTGTCATGAGGTACGTTTTGGACAAGAACGTGCGACTCTTTAACACCGAAGATGGCAGAGCATCCTCGAGAGGAAGTAGACTGTACTCGGTGCTCATGTGGCTCACGTCGGGATTAGCCAGGCAAGGCGGCTGCCAGGGCCAGTGACGTAGCCGCCACTCACCAATCACACGGCTCGCATTTTCCTGGAGGAAATTCCGGTGCCAGCGTGAACGCGCCCACATTTTGCGGCGCTCCAGCCAAGCATCCACCATACGTCATGAGCGCGTCCGCTTGGCAATGGGAAATTCCGCGAATACTCAGCGCCATGCATCATGGGAACTGTGGGAGCTTGAGGTCCTGAAGTCCAAGAGAGGGCACTACCTCCAAGCTCCAAGACGTAGACTCACGGGGATGTCATCGCCACTGAACTTTGGGCATTCAGATGCATGTCAGTTTACTATTGTTTTCATTTCAGAAGTGCCGTTTATTGTATTTTTTGTAGGAGCAAACTATCCGTGCGGACGACTTTTTTGTATATGATGCCCTTACTTAAATTATCCATATTGAATACTCATTAGAGGCTCTCAATTTGCTTTCCTCCAGGTTTGTTGAACACGCATCACTCTCGATAGCAGCGGCGTTAAAACAGTAATATACTGCGTCGGATGAAAAAAGAAAGCTAAGCAAACCTACAATCTAGAGGCTGTTCTCTC |
| >SG4816897  CCTGCATGTTTTATTATTTATTGACTTCAAGAAACTTTAGCAAAAGAAAAGCCCATAGACACTGTGCTCATGTGTTGCAACGCTTCGACTCGTCGGAAAACTCGTTGATGAACCAGCCGACGAGTTTTACGAAGTCGCACAGCTGGTCTTCTGGCTTTTGACGTGGCCTCTTTCCGCTCGGTTTCGTCCCGGGCCGGTTTCTGCCTCCTCCCTCTGTCCGACTTGGTTTTTTGGCTGCTTCCGCTAGGATGACAACGGAGTCGCTGTCTATCACGTACACGTATTGTTCATCGCCGAAGAGGGGGTCCTTCTCCTTGTACTTGGCGTCCTTGCCCATGATGATGTATCGGCTGCCTTCAGTCAGTTTGAAGGATTCGCAATTCTCGCGAGCCTTGACCACTCGCGTCTTGTTGAGAAGGTCCTGCTGTGACTCAATGCCTGGCTTGAGGATTTTGTTGATAAAGAAAGGAACAGTGATGAATCCATCTTTGTGGGCTCCATTAAGGGGCGTGCCAAGCCATACAAAATCAACATTGTCGCAGGCGTGGTATCTCATGTATTCTCGGCAATCTGAGGTTTTCAGAACAGCACCCTCTTCTTTCGTGAACATCTCCTCGACTTCTTGGGGCGGGCAACCACCTTCCAAGCATCTGCAGACTTCAGAGTGACCCTCATTGTCATCGCTGCAGTTTGACTTGAGCATGGGACTGGTGCTGTCTGGCGAGTAGAATTTCCTGCACGATATATCGGGGTCGTAGTAGGCGTACGCCTTCACGGAGCCCGACTGCAACTTGCCTACGGCGAATGCCTGCTGCAGGCCAAACTCGACGCACACGTTGGCGTCCGAAGGAATGGTCGGCAGGTAAAAGACCACGCTACGC |
| >SG4824254  GCCAAATGCGTTCATGAAACCCCACATGCTTACAAGCCGTTATTCCGGGTTGTGAATGTACTTGCTTTTCGTTTTTTTTCTTTTGTGACGCTGTTTCACANNNNNNNNNNAGTTTGATCTGAAGATAAACTTCACACAGCACAAGGTCAACGTGTCGAAAGCGTTGGAAGGTAAAGGGAGTTTCAAAGAGATTTACTCGATGGAAGTCTGTGCGAGGTCTCTTGAAAAAAAATTGAAAGGCATGGCCATCCTGGACGTAGGTCTCCTAACCGGCTTCAATCCCGTGTTGGCCGATTTGGACAAGATGGTGGCCGACAAACGTGTGGACTCGTACGAGTTGAGCCAGCGTAGCGTGGTATTCTACCTGTCGACCATTCCATCGAACGCCAGCGTGTGCGTCGAGTTTGGCCTGC |
| >SG4829934  AAACTTTTGACTTGCTTTGGAGCAGGGCTGAATCAGACGTTGATGGCACGTTACATTGGAGCCACAACGTGTCTTTGGGAGTGGCTGTAGAGACAAGCGGCTATGCAATTCTCTCCTGCATGACACTGCTCAAGGTAGCAGGTGTACCTAAAGCCTTGCCCGCAATCCAGTGGTTGGTCAAGCAGAGGAGTGACCATGGAGGATTCGCGTCTACACAGGACACTATTGTAGCCTTGCAAGCCCTGGCTGCATTTGCCAAAGCAACTGTTCTGCAAAGCACACACTTGTCAATGAGAGTGTCGTCCCAACACACAGAGGCCAAAGAGCTTTTTGTGGAACCAAGCAATGCAATGCTGCTTCAGGAGGTGGTCATCCCTACAGTGCCGGCGATGGTGAACCTCAACGTCACAGGGAAGGGGTGTGCCTTTGTGCAGGCCACTGCAAAGTTCAACATTCCTGCAGTGGCTGATACCAACAAGTTTGATCTCATGGTACTGGCATCCCATGCTGACTGTAAGCCAAAGATACAGCTTTGTGCTAGTTACCTCCTCTCCTGGGGCATATCTGGAATGGTGATCATACAAGTCCATCTGCAAACTGGGTTCCGTTTGAGCAAGGTTGTTGATGGCCTGGAAGTTAGGAAGACTGAGGAAGAGCCTGACAAAATAAACTTGTACTTTGATGAGCTAAGTAATCGAAGAATGTGCATCACCTTAATGTTGACCCAGGAGTTTGAAGTGCAAGACAGTGCTCCTGCAGCTGTTGTATTGCAGGACTACTATGATCCTGGTGTCATTGTACTAAGGAACTACACAATTCCAAGTTGTGAGAGGGATCAACAAGACCTTAGTGACTCACAAATGCCCCTGCAGTCAGAAATAATGCAAGAAGATATTCGTTCAGCCAGGACTCATCTTGGAAACTTCCGAAATATCGAACAAGACTTGGACTTCCCTGATGGCCCTGAATCAAACATGCCTGTTACAGTGCCTGCTCCTCAGACCTGACTGGCAAAAGGACATAAACGTGCAGAAAACTTTCATACTGACGGAACTTGCAAGTTGCTGGAATGTGCTCCAAGTTGAACCTCA |
| >SG4830773  CTCGAAGTCGTCTTTCTCGATATACGTGGAGTCTTTTCCCATGACAATATACTCCTCGCCATTGGGAATATTGAATGTATTGCAGGTATCACGAGCCTTGATGCGACGTATCTTTCCTTTCAAGTCGTCTTCTTGGCCTGGCTTGAGAACTTGAGTGATGAGGAAAGCCACTTCTATGAAGCCATCCGTCGATACATTGGCTGTCGAATTGCCTCTCCATACGTAGTGAACTCCGTCACAGGCGAATTCTCTCAGAAGCTCACGCTGTTCTTCGTCTTCAAAGTATTCGTTGTCCCGTGTTTTTATGAACCTGTCGAGTGGCTTCTCCGGCGGGCAACCACCTTCCGCGCAAACGCAGACATCGGAGTCGTCACACTTATCATCAACCTTTAGTAGGGGGCTTGTCTTGTCGGGCGAGTAGAATCGTGTGCAAGAAAAGTCTGGTTTGTAGTAAGAGTATGCCTTCACGTAGCTTGACTGCAGTTTACCTGCATTGAATTCTTGTTCGAGAGAGAAATTAACGCAGTGCGTCATGTTTGCAGCAATGAAGGGTACGTAAAAGTCAACATGGCGACTTGTGATAGTATAAAGCTGAATCTTTCTTTCTTTCACGAGCTCATCCAACTCGGTAGCATTGGGCTTGAATCCAGTCAGAAGACCCACTTCGAACATGACCATGCCATCTGGAGC |
| >SG4842226  CTACAACGTTCAATCGGCTCTCGTGAAGGTGTACGACTACTATGACCCGACGCGTTCGTGCACGCAATTCTACGGTCCTGATTCGACAAGCCCGCTCCTGAAGCTTACCTGTGAACGAGACGAGTGCCGATGCGTGGAAGATGAATGCCCACCCAGAGAACCCTTCAGAACAATTGACAACGTGGGAGCGACTTCTGAAAATAGGCGCAAGCTGCTCGACNNNNNNNNNNNNNNNNNNNNCTTCGTGTGGGTTGGAACCGTGTCTCGGAACCATGTCACCAATGGTTATCGCCAAATCGAACTTCGTGTTGACACGGTGATCAAACAAGGCGCTGAACAGA |
| >SG4844595  CTCTGGAGCGTTGTATTCATACACCTTGGCTTTCAGGTACCTCGTTGAGTTGGCAACGGGATACCACCGCTGTATGAGGAAGTTGACGCAGGTCCATTCGCTTGTGAGGTATTCAAACGTGAACACGGCTGTCCTGGGGAGGACCTTAGCCATGCTGAGTCGAGGAACTACTCCTGACTTCACGTAGTTGTCCAGGTGCGGCCGGTAGTTTGCGTAACCAGTAGGGATGGCCAGTTCAAGAACAGCCGCCCCACTGGCTGCGCTCATATTGGTCAATGTCCACCGAACACAGCTGCG |
| >SG4822516  GCGGCCGGTAGTTTGCGTAACCAGTAGGGATGGCCAGTTCAAGAACAGCCGCCCCACTGGCTGCGCTCATATTGGTCAATGTCCACCGAACACAGCTGCGGATGTTGATGTGTGACCTGTTCCTCCCAGAGAACTGCGGCATCAAGGTGAGATCAAACGCCTCATATGCAGGAGGTACAAGCAAGAAGTCACGATCGACGTTGTAGGTGACATCCAACTGGATAAGAGACAGGCCGGACCCTTGAGCCAGCACGTCGCAGTGACCCCAAACGTTTGGTTGGACATCAAAGACTTTTCTGCGTGCAAGGTCATCCTTGGATATGCGCAAGGTGCTGGGAAAGCCGGGGTTTGATGATGACTCCACAGTGACCTTCATTTCTGTTATGCCTCGGACGTGCGTTCGGAAGGAATACTGTGTAAGCGCTTCGAGGGCTGCAAGTGTGTCCTGTGTGCCGATGAAGCCATAGTCTGTGGAGCGCATGCGGTTCAGCCAGCGCACGATCTGGTCCTGGAAGAGGCCGCCAAGTTTGAGGTAGACCTGCAGGGCGTACGCAGTGGCCTCCACAGCGGACGAGTCCTCTGGGTGCGGTAGTCGGGGCATCAGGAACGGCCGCTGGCTCTGGGACACGATCACGGGCGGCGGAATGTGCACGCTGCTCCAGTAGACAGCCCCCTCGGCATCTTTCTTCATGGATTCCAGCAGGTTGTAGCCGAATTGGGCTTCACTGGAGCCAGCCTCAAGGAGTGCGTA |
| >SG4836493  TGCTGGGAGGAGTCCGAGGGTCTGTGCCCATGACAGCCTTTGTCCTGCTAACTTTCTTGGAGTGCATGAAGCCTCCGAGCATGACGAATGGTCCACACGACCCTTTTGCAACTACCAACAAAGCGATACTGCGTCGNNNNNNNGAGCTGTCTCAGAGGTACCTGCGGGAGAACGCGTTCGACAGCGACGAGCCTTACGTCGCGGCCCTAGTTGCTTACGCGCTCAGCCAAGCCAACGACACGGAGAAGTACGCCAC |
| >SG4837538  CGGACTGGGAGGACTACATATACATCGATCCTCGGCTCATCACTCAAGGGGTCCAGTTCCTGCTCAAGCATCAGGACACCACGGGCGCTTTCTACGAGACAACGCGCAACNNNNNNNNNNNNNNNNNNNNNNNNNAGGCTAACAACAAGGACAAAAACATCACGCTGACTGCGCAAGTTGTTCTCACGCTTTCCAAGGTCACCGATCTTACAGGAAGCATTCGAGAGCATGCCAACACTGCCAAGCGCGACGGCGTACGGTACCTGGAGACACAGCTGGGCCAGGTGACGGACCCATAC |

| >SG12019417  CAGCGCTGTTGACCAGTGTGAGTGCATATGCCACAATGGCCAATTCGTAGGGGTCCTCCAACTTCTGAATGAGGTGCAGCATGCGCTCCAGGTACCGAGCAGCAGCAGCACGAGCTGTGGCTGCACGCGATCCGATCTCACCTCTGATATCCCTAACTTCGGCCAATGTGATGAGCACGTGCGCGGTTAGAGACACATTGCGAAACCGCACCGGGTCTTCTGGCCTCTCAGACGGAAGAGACATTTTGCGGTCATAAGCATAGAAAGATGTCTCGTGAAAGGAGCCTTCGGGAGACTGCCTGTCTAGCAACCATGAAATGGCCTTTTGTATGACAGTGGGATCGATGTATAAG |
| --- |
| >SG1203023  TCCCTCTAGCGAGACCACTCGCACTGTCGGGTCCTCCACCTCCGGCTGGGGTGGCCTTTTCCAACCGCATTGTAGGTGGCCTGCCCGGCGTGCCCGTCGCCCTGGACTCTGTGGCGCAGAGCGCACCATCGGCCAACATCCCGGCCAAGTCGGCCGTTGAAGTGCGCACCTACTTCCCCGAGACGTGGCTGTGGGACCTGAAGGAGCTGGATGAGCACGGCGAACTGAACTTCAAAGAAAAGATTCCGCACACCATCACCGAGTGGGTGGGAAGCACTGTGTGCATCAACTCAGAGGACGGAATCGGCATCTCAGATCCGGCAAAGATCAAGGCCTTCCAGCCGTTCTTCGCCTCGTTCAACCTGCCTTACTCTGTGGTGCGCGGAGAACTGGTGCCCGTCAAGGTGTCCGTGTTCAACTACCTCGAAAAGTGCCTGCCGGTCGATCTGAAGCTGGCTGAATCCGCGGACTTCCACATTGAGGGCGAGTCTGAGACGACGCTTTGCGTGTGCGGCAGCAAGAGCCAAGTGCACAAGTTCCAGATCCGGCCACAAACTATCGGAGAGGTGAACCTGACCGTGTCGGCGGCCGGATCGCAGAGCGATGCCGCATGTGGAGAGCAGCCAACTGAGAAGGTGGTGGCCAGGGACGCAGTCACACGACCGCTCATCATCGAGGCGGAAGGCTTCCCTAAGGAAGAGACGCAGAGTGTCTTTGTTTGTCCTAAAGACGCTGCAGAAGGCGGCGCAAAGAACGAATTTGACTTGGTGCTTCCTGAAGACTTGGTGGAAGGATCGGCCCGTGCCTACGTTCAAGTCACAGGAGATATTATGGGCCCAGCAATCAAGAACCTCGACTCACTGGTGCAAGTTCCAACGGGATGTGGTGAGCAGAACATGGTAAAGTTTACGCCAAACGTCTATGTCCTGGACTACCTTAAGGCTACGGGCAAGAACCAGGAAGACATCGAAAGGAAGGCTGTGCAGAATCTCAAGACAGGCTACCAGCGCCAGCAGAAGTACAAGCACTACGATGGTTCGTACAGCGCCTTTGGAAACCGCGACAACTCGGGCAGCATGTTCCTCACGGCGTTTGTAGTCAAGTCCTTCAAGCAGGCTGAGAAATACATTCCTATTGACGCCGGAAACCTCAACGAGAGCATTAAGTGGATCATCACGAAGCAGAAAACCAACGGCTGCTTCCAGAACATCGGCACTGTTCTTAGCTCCGGCTTGAAGGGCAAGGTTAACTCCACTGCCCCGGGAGCGCTGACGGCGTACGTGCTCACCGCTCTTCTGGAGGGCGGCCTTGCGGACGAGAAGGTCGTTGAGTCTGCGCTCCGCTGCATCAGCGCCCAGCGTGACCCCAGTGCCCACAACTTGGCCCTGTCCGCCTACGCCGCGGCCCTGGCGGGACATGAGTCTGCCAAAGATTACCTCGAGAAATTGGAGTCCATCGCTGTTCACAAAGGTGTCCTGACCTACTGGAGCAACGCGGGCAAGAAGGGTCCATCAGCGTCGGCCGACGTGGAAACGGCGGCGTATGCAGTGCTCACCTACCTCAAGCTGAACGCCCAAGAGAACCTGAGCAAGGCGCAGCCCATTGTCCGCTGGATGGCGACCAAGAGAAACAGCCGCGGAGGCTTCCCATCCACACAGGATACAGTTCTCGGTCTCCAGGCTCTGTCAGCGTTCGCTACTTACGTTAGCAAAGATCCCGTTGACATCTCAGTTAAGGTCGACAGTACTGACGTCAGTGAGTCGTATAACCTTAAGGAGGACACCAAACTAGTCGTACAGGAGAAGAAGGTTGTCAGCTTGCCCAACAAGCTCACTTCAGAAGCAACAGGTCCTGGATGTGCCCTTATATCGGCGACTCTGAAGTACAACGTTCATACTGCACCGAAGAGCGAGGGTTTCGAGCTCACAGCCACTCCATCTCAAGAGGCATCTGACTGCAATGACCACAAGCTCAAAATCTGTCTGAGGTTCGACGGTGAGCAACCATCTAACATGGCAGTGGTGGAGCTCAAGCTGGTATCGGGCTACATTCCAGATGAGGATCACATCTTTGGACTCTACCGTGAAAAAGACGTCAAGCTGAAGAGGCATGAAGTTGAAAAGAACCAGGTTAACTTTTACTTTGAAGAAATAACGTCCGAAAACAAGTGCTTCGACGTTCGCGTTCACCGCGAGTTCGCTATTGAAGATGCCAAACCTGCCACAGTTAAAGTCTACGACTATTACGAGCAAGAAAACAGCAACTCCGTGCCCTACTCTCTTGTGGC |
| >SG12014421  ATGAAATATTTATTGTCTTTATTTTTACAGTGACTAAACATTTCAGGACCAAAGTGCGAGCAAGGAAAGACCACACATTCATAATTTTATTTTCATCCTTTAGCTGCACTTCACTGACTTAGTGACCACGAGCCGGCACTCTTCGCTGCATAGCTTAGGCAGAAATTGTGCGATCCCGGGAGACGCCACATGATTAAGTAGTTTTGCCTCTTTGATGTTCCTACGAATGTGAAGTCTATGACCTGTGAGAAGACAAACCTTTCGCGATAAGCTAGAGGCATATTTTGCGCTGCACACACCCACGGCCGAGATGACAAGTTTATACCGCAGGCAGATTTTGTCCCCTATATGACTATAGGATAGGTGCTCTGAAGTGGATCAGGGCCTATCAGAAGCTGATCAAATTGTTGTTCTGTGAAAACAGAACGTGTTATCGGCAGGAGATTGATAGAGGCTACCACTGAATGTATGCAACTCACTTAAGTTGTTGGAGACTTTCTCTACCAAAGTGCAAATGTAAAATTCGCTGCATGGAAGAATGGAGGCCTTGAGAGAACAGCCTCTAGATTGTAGGTTTGCTTAGCTTTCTTTTTTCATCCGACGCAGTATATTACTGTTTTAACGCCGCTGCTATCGAGAGTGATGCGTGTTCAACAAACCTGGAGGAAAGCAAATTGAGAGCCTCTAATGAGTATTCAATATGGATAATTTAAGTAAGGGCATCATATACAAAAAAGTCGTCCGCACGGATAGTTTGCTCCTACAAAAAATACAATAAACGGCACTTCTGAAATGAAAACAATAGTAAACTGACATGCATCTGAATGCCCAAAGTTCAGTGGCGATGACATCCCCGTGAGTCTACGTCTTGGAGCTTGGAGGCAGTGCCCTCTCTGGGGCTTCAGGACCTTAAGCTCCCACAGTTCCCATGATGCATGGCGCTGAGTATTCGCGGAATTTCCCATTGCCAAGCGGACGCGCTCATGACGTATGGTGGATGCTTGGCTGGAGCGCCGCAAAATGTGGGCGCGTTCACGCTGGCACCGGAATTTCCTCCAGGAAAATGCGAGCCGTGTGATTGGTGAGTGGCGGCTACGTCACTGGCCCTGGCAGCCGCCTTGCCTGGCTAATCCCGACGTGAGCCACATGAGCACCGAGTACAGTCTACTTCCTCTCGAGGATGCTCTGCCATCTTCGGTGTTAAAGAGTCGCACGTTCTTGTCCAAAACGTACCTCATGACAACTTCGCCGTCCTTCTCGTGGGGTTCGCTGTCTCGGCCGAATATGAAGTAGCTACGTCCCTTGGTGAGGTCGGCCGTGTTGCAAAACTCCCTCGCCACGAACACTTTCAGACCCGTCATTGCGGAGGTGCTGTTCTCAGCGCCTTCTTTCACAACGCTGTCCACACGGAAGTCGATGTAACGGTAACCGTTGACGATCCTGTTGGCAGACACCATTCCGATCCAAACGAAATCATGCTCCTGGCACGTGAGTTCGACTAGCTTCTGCCTTTTCTTCACTGTCGTTCCTGATCTCTCGACGTCGATGAAGGGCTCTTTCCTTGGGCATGCCGCTTCGGCGCACTGGCACTGATTGTTTTCGCAGATCAGCTTCAGAAGGGGACTCGTGGATCCGGGCCCGTAGAACTGCGAACACGAGTGCATTGGGTTGTAATAGTCATACACCTTCACAACGGCAGACTGAACATTGTAGACCACATGCTGACGCTCTATTCTGAATTTGACGCACGTCGGGG |
| >SG12019001  TTCTCTCGTATACCAGAGCTACTGCTACGTTGATGGTTATCTCCACAATGAACCCTACGAGAAGTCAGGTGACTCCCAGNNNNNNNNNNNNNNNNNNNNNNNNNNNNNNNNNNCTTCAGTCTCCTTCAGCTACGACTCCCTGAGCTCGTTCGAGAACACTGGTCTTGTCGTCTTCACCAACCTGCCAATCCAGACGAGGCCGTGCCCCAACCGTGCTTTCCCTGGCTATCCCAACAATCAAGTCCTGGGATCGACAAATCGCTACTATCCCAGTTTCGGCGGTTTCGGAGCTGTAAACAAAGTCGCATTTGCTCCGAGTGCCTCCCCCGGATTTGCTGGACCGGCACCTATCGCAGAGTCTGCGCAAGCCGATTTTTCGCCCGTCGTCCAGGGGTCAAACTCCGTCCGCACACTCTTCCCTGAAACATGGCTCTGGCAGATCAAGAGAGTGAGCCCTGATGGTTCCTTGGTGTACTCCGAGACGCTCCCGGACACAATCACCACATGGCAGGGCACTGCCGTATGCCTGCACCCGCGAAACGGCCTCGGCGTCTCCCAAGTGGCGAACGTCACCGGATTCCAGCCATTATTCGTATCGCTCACGTTGCCGGCGTACATGCAGCGCAACGAAGTGGCCACGGTCATTCTCACGGCATTCAACTACGGCGAAGAGTGCGTCGCGGTCCGGGTCAGTCTGAGGTCTTTGGAGAATCTCGAGGTGGTCGGTGGTCCCAACAGCACCGACGCACTACTCTGCCCCAACTCCAGCACATCCGCGTCATTCCCGTTTGACGTGCGCGCTACCACGCTTGAGGAATCCCGGCTGGAAGCCCGCGTCCAGACGAGACCCGAAGCCCAGGCACAGTATCCGGACGTCAAACTGTCCAACGTCAACTCAAGCGATACTGTCATCCAGACCATCGATGTTAGGCCTGAAGGCTTCCCCATCAGGGAAATCAACACGTTCCTCCTGTGTGCTTCAGGTGATGCAGACAGCGCTCCGGACACCCTGCAAGTCAACCTTCCGACACCTGTCGCTCTTGTTGAAGGCTCCCAGCAGGTTGTACTAGTGGGCACAGGCGACATCCTCGCTCTCAGCTTGAACGACCTATCCGTTCCCACCATCACCTATTCCAACGCCGAGGGAACACTGGCTGTGCTTGCCTCCAGCGTCTACCTCCACAAGTACCTGGAGCAGACGGGGACTCTGACTGACACGGTGAACTCCGGCCTCCGATCCCGCATTCGCCAAGCGTCCCAGGCTCAAAACAGCTTCCGGTCCTCCGACGGCTCATACGCTCAGTTTGGCTCTAGCGAGTTCCCGAGAAGCGTGTTCCTCACCGCCTTCGCCGTGAAAGCCCTGAGCGCCGCCAAGGAGTACCTGGGCACCAGCGTCGATGCGGACATCGAAACTAGCGTGCGCTATGTCCTGCAGCACTGGAACGCTGCCACCGGTTGTTTCGTTGAGAACCAGCCCGGCTCGTCACCCTTCGGCCCCCAGACAGCCCCCGACTTCACCGCGGCCATTGGCGTCATGCTTCTGGAGAGTGGCTACAACTACGAGAACATCACGAACGGTGTCCTCCAGTGCATTGACGCCAGCAATCTGCCTTCCAACCACACGACTGCGCTGAACGCCTACTTTTCGGCGCTCGCTGGACGCACGGACCGAGCTAACAGCGCCTTGGACACGCTTCTGAGCGAAGCTGATAAAAGCAGTGGCTTGACGTCGTGGAGCGGCGATGGCCTGACTTACGGCTCGGCAGACACCGCTGGTTACGCAGTTCTCACTTTGAAGTTGCTCGACCGAAACCTGGGAGAAGCGCTGCCAATCGTCCGTTGGCTCATGCAGCAGACCTACGCCCGATACACTTTCTCCTATTCCGAGGTCTACACCGTCGCCATCCAAGCCCTGACCCAGTACTCGAGCGTGGCATTCTCGAAGAACACAAACTTGACTATGAACGTTGCTGTCGACAGTTCATCCCCCGAAACGGTCTCTTTCCCGATCTCTGAGCAGAACAAACTGCTATACCAGGAGCGTCTTTTGAACAGAAGCGATTCGTACAGCTTCAAGGCATCTCTTGCTGACGGCAGCGCAGGGTGCGCCGCTTT |
| >SG120169  CCAACGCCGCCGCCTTAGAGCGCGTGTGTTGTGTTTTGCTTCGCCACGCGACGTCAAGCCAGGAGCGAGACAAGAAGGGTAGCAAGAAAAAGCTCCCGGAACCGGCCTTAGCGCGCGCAGCAGAGAAAGCTGTGCTGTACAGTCTACGGAAAGGTGTCTAAGGCTGGCTTAGACGCAAGAAAACTAGCACAGACGGTGACGAGAGGTGCCGGTGGCCTGAGCTAGAATGCATTGGGGCACCTTCATTTTAGTCCTTGGTGCCTTGGCATCGCGCCTTGCTGAAAGTGGGTACATCTTCACGGCGCCGAAGATCCTGCGCAGCGAAACGGACGAACAGTTTCGGCTAACGCTCACAGACGTCAAGGAGGATGGCAAGGTCATCGTCCGCCTGCTCAAGTACAACAACGACAGCATCGTCTTGGCAGAACAAGAGTACGACATCAAGAACGGTGAAAGCACCTTTCTGCCGTTCCGAGTGCCCGAGCACCTCGACAGCCAAGCCAAGATCGAAGTGAACGGAACCTTTGGAGACTATGTCTTCGGCGATCGGAAAGAGATCGACTTCCAGAAGTCCAAGAACACGATCCTGGTTCAGTCCGATAAGGCACTTTATAAGCCTGGACAGAGGGTTCAATTCCGCGTCCTCCCTATCAACAATGAGCTGAAACCAGTCACCGATGTGAAGGCAACTATTTACGTGACAAGTCCCAGCGATGTAAGAATAGCTCAGTGGAACGACGTGTCTTTTGAAAAAGGCATTGTCCAGAGAGACTTTCAGCTTACAGAAGAGCCTGAGTTGGGTCTGTGGCAGATAGTCGTCGAACTTCCCACGCAAACCGTGAGGCAGCATTTTGAGGTGAACGAATACGTACTTCCCAAATTTGAGGTGACCATCAAGCCTCCATCCTACGTCTTGGCTGATGCAAAGGAGATCACCTGGAAGATTTGTGCCCACTATACTTTTGGACAGCCAGTGGACGGCACCCTGACGGTGAACGTGACATACGAGCGTTACAGTTGGGAGAAGGACGACTACCCAAAGATCAACCACACAGGACCCATCAACGGCTGCTTCGATATGACGGTGAACACGAGCCTGCTGCGCTTCAACGAGAACTACGAGATCTACAAGCGCATCTACCTCGTCGCACAAGTGAACGAGACCGGCACGGGCATCACGATGAACAAGACGAACTACATCAGCCGCAGCTTCAACCCGCTTGAGCTCAACTTTTTGGAAGGAGACCGCGGAAAGAACTACTTCAAGCCCACCATGCCTTTCTACGGCAGGCTCCTGGTGAAGAAGCCTGACGGCGTTCCTGTGGGCGGCGAGCTGGTGCAACTCTGTCTGCTGTCACAGGCTGAGGAGATCAAGCCACGGTGGTGGCGCACCGACCGCCGTCTCAGCTGTAAGAACTACACTTCGGATGAGTCAGGCATCATCAAATTCACCATCCCTCCCCTGAAGACAACTGTCGTCACCATCAGCGTCGAGGCGGTGGCGATGAACTACGAGACCGTTAAGTACGACACCTACGGCGTGAAGATCAACCAGCCCAAGAGCACTCTGTACCTCCAGGCTTGGTACTCGGCCAGCAACAACTTCATCCAGATCGAGCCGTCCAAGGGACCCTTGCGCTGCTCGGGCAAGCACCCCGTGAGCCTCCGGTACACGGGTCAGCCCGACTCAGAGAAGCAGTTTCATTTACAGGTGATAGCTCGCGGCAAGATCCTCAAGGACAAGGTTCTGGATGTGACCTTCAAGCCGGAAGAGGTGCTCTCTGTTGACGACAGCTTCCTGGTGGAGGAGGACCTGAATGAGACGCTTCCGACCAACATGGCCGAGGGCAGCGTGTCGACAGGGAGCTTTGAGTTCGAGCTCGAGCCAGATTTCAGCCACGTGCCACGCGTCAAAGTGCTCGCTTTCTACGTCCGCCCCGACGGCGAGGTCATCGCTGACTCCGAGCAGTTCGAAGTGGAGAAGTGCCTCCAGAACAACGTAACCATGAGATTCGGATCTGAAACTGTACAGCCTGCAACATCTGCTGCCATCCATGTGAATGGTTCTCCTCGGTCCTTCTGTGGTGTTGGCGTCGTAGACAAGAGTGTTCACCTCCTGAAGCAGGATAACCAACTGACAAAGGACAAAGTTTATGACATTTTGAAGCGTCTGGATATCACCCGCTACACGTGGCCGAAGCAGGCGTCGTACGACTACTGTCGCAAGCAGTTGGCGAAGAATCCGCAACAGTTCAAGCGCCACATCTGGAATGGACCGAGATCTTCAAATGTTGAATACGTCGATTCGATCACCGCCTTCGACGAATCAGGGGTGGTGGTCATGTCAGACTTGACGCTTGAAACGAGGCCATGCCGGAAGAACATCTACGACAGACCACCCTACGCTCTCGCGGCACCAGCGATCCCTCTAGCGAGAC |
| >SG12035473  TCGGTAGTTCACTGTGCTTCCTTGACGGTACATGGGCTTATCCGTCTGTACAACGACGTTGACATTGGTAACGTATTCCAGTTGAACAGACGATCTGTTTTGGAATTGGTCGCTGCCGAATTGTCCGGTCACATCGAGGTACAGGTTGTAGAAGACGCTGGGGCTCAGGTCGGGAACGTGGAAGAGTAGCTCGGCGTCGATGCCACCTGCGCCGA |
| >SG12010492  GAAAGGAACTGGAACGGCGACTATGTACGTTGTCACAAGCTACGAGTCCCCTGTCAGTGTGGACCTTTTGTGCAAGTTTGATCTGAAGATAAACTTCACACAGCACAAGGTCAACGTGTCGAAAGCGTTGGAAGGTAAAGGGAGTTTCAAAGAGATTTACTCGATGGAAGTCTGTGCGAGGTCTCTTGAAAAAAAATTGAAAGGCATGGCCATCCTGGACGTAGGTCTCCTAACCGGCTTCAATCCCGTGTTGGCCGATTTGGACAAGATGGTGGCCGACAAACGTGTGGACTCGTACGAGTTGAGCCAGCGTAGCGTGGTATTCTACCTGTCGACCATTCCATCGAACGCCAGCGTGTGCGTCGAGTTTGGCCTGCAGCAGGCATTCGCCGTAGGCAAGTTGCAGTCGGGCTCCGTGAAGGCGTACGCCTACTACGACCCCGATATATCGTGCAGGAAATTCTACTCGCCAGACAGCACCAGTCCCTTGCTCAAGTCAAACTGCAGCGATGCCAATGGGGGTCACTCTGAAGTCTGCAGATGCTTGGAAGGTGGTTGCCCGCCCCAAGAAGTCGAGGAGATGTTCACGAAAGAAGAGGGTGCTGTTCTGAAAACCTCAGATTGCCGAGAATACATGAGATACCACGCCTGCGACAATGTTGATTTTGTATGGCTTGGCACGCCCCTTAATGGAGCCCACAAAGATGGATTCATCACTGTTCCTTTCTTTATCAACAAAATCCTCAAGCCAGGCATTGAGTCACAGCAGGACCTTCTCAACAAGACGCGAGTGGTCAAGGCTCGCGAGAATTGCGAATCCTTCAAACTGACTGAAGGCAGCCGATACATCATCATGGGCAAGGACGCCAAGTACAAGGAGAAGGACCCCCACTTCGGCGATGAACAATACGTGTACGTGATAGACAGCGACTCCGTTGTCATCCTAGCGGAAGCAGCCAAAAAACCAAGTCGGACAGAGGGAGGAGGCAGAAANNNNNNNNNNNNNNNNNNNCGGCCCGGGACGAAACCGAGCGGAAAGAGGCCACGTCAAAAGCCAGAAGACCAGCTGTGCGACTTCGTAAAACTCGTCGGCTGGTTCATCAACGAGTTTTCCGACGAGTCGAAGCGTTGCAACACATGAGCACAGTGTCTATGGGCTTTTCTTTTGCTAAAGTTTCTTGAAGTCAATAAATAATAAAACATGCTCAACGAGTTTTCCGACGAGTCGAAGCGTTG |
| >SG12011552  TCTGACTGGATTCAAGCCCAATGCTACCGAGTTGGATGAGCTCGTGAAAGAAAGAAAGATTCAGCTTTATACTATCACAAGTCGCCATGTTGACTTTTACGTACCCTTCATTGCTGCAAACATGACGCACTGCGTTAATTTCTCTCTCGAACAAGAATTCAATGCAGGTAAACTGCAGTCAAGCTACGTGAAGGCATACTCTTACTACAAACCAGACTTTTCTTGCACACGATTCTACTCGCCCGACAAGACAAGCCCCCTACTAAAGGTTGATGATAAGTGTGACGACTCCGATGTCTGCGTTTGCGCGGAAGGTGGTTGCCCGCCGGAGAAGCCACTCGACAGGTTCATAAAAACACGGGACAACGAATACTTTGAAGACGAAGAACAGCGTGAGCTTCTGAGAGAATTCGCCTGTGACGGAGTTCACTACGTATGGAGAGGCAATTCGACAGCCAATGTATCGACGGATGGCTTCATAGAAGTGGCTTTCCTCATCACTCAAGTTCTCAAGCCAGGCCAAGAAGACGACTTGAAAGGAAAGATACGTCGCATCAAGGCTCGTGATACCTG |
| >SG12024337  CCGCCGTGTCAGTGGAAGGACCCCGACAGAGCAAGCTTACTTACGACATCGAAGTGTGCTCACGCTACATTGGCCGGGAGGATTCCAACATGGCCGTTATAGAGGTTGGCCTGTTCAGTGGATTCAAACCCATGGAAAAAGATCTGGAAGCGGCAAGAGAAGCCAACAACTCCCTTTTGGCCAAGTATGAAATGACTGAGAAAAATGTGATCCTTTATTTCGACAAGATACCTTGGGAATCCCCGACGTGCGTCAAATTCAGAATAGAGCGTCAGCATGTGGTCTACAATGTTCAGTCTGCCGTTGTGAAGGTGTATGACTATTACAACCCAATGCACTC |
| >SG12026731  ATCGCATCATGAAGGTTCGCCACTGGCAGGTTCCTGCTTTCTGTCACGCTGAAGCAAGTGGGCTCCACAGTCAGCGTCTGAAAGATGAGCTGAATCTGGTTGTCACTGACGACATACCGCTTTACGACGCCGCTGCTCACCAGGTTTTTGAGAGACTGGTCTTCCGCGGAGTATCCAGAAAGCAGAGTGATCTGGACGATCGCAACGCTGCGTAGGAAACCTTCGGTGTACCGTGCACATATGTCCAGCTGGGCGCTGCAGTCTGGACCCGATGTCGTGGACACGTTAACTTGAATTCCTCGCCGAACCGGACTGTTCCTGGAGTT |
| >SG12012594  TAATAACGGTTCATATTTTTTTCTTTTTTTTTTTTTTTTTGAGAAGTATAAAACTCTTGTATGTTATATTGCTTCTGTACACCATACATTCTTGTGCAAGGCTCTACATGTGAATCCAGTTTACATTTGACAGATGAAGAAAATGCAGAACCACAACTCCACATGCCTGTAGGTCCATTACATCACACTTTCTTAGTGACTGACAGTCTCAAGAATGCAAACACACACAGACACACACAACAACAACGAAGAATGTGCAAAAAAAATGAAAACTTGAGTCCTACTCTTCAGACACATCTTATAGCAAAGCAACACAATCATTTCTTCGTGTAGATGTGGCAGTGACATGTTTTGATGATTAGATGTCTATAACAGACACAAATGAGATTCTGGGCTACATTTACCACGCAGCATTGGAAACGCACTTACTAATCTCAGTTTGTGTATCTTGACTATAAGCCCATGTACCAAGATAATGAGCAGATAAAATTAAAAACACTAGTGAGGCAGCATTCTTTGCACAGCTCGCCACCACAAACCATAGCATCTCCAAAGTGGTGAGCACGGTTGTCGACAGTGGCACCACTAGTCGGAGCAGCTATCTGACCAACATTACTAGTCCTCTAAGAAGACACCTAATTGTGCAGTGTTTCACAAAAATGTTCGGACATAAACACGTGTTTAAGAATTTGCAGAGCCCCTTTTGTGTGGGTTTAGGCAATAGGTTGACAACCCTTTAGCAACAATTTTTGCTGTACAGAAAGATTTGCTTATACTGGGTCTGTTCGAAGAAGGCTGCCTTCATATATCAAGGATTTGACCTGTCCAGAAGATATTGTTTCAAAATTCTATGATGTCTTTTAGCCACCGCACATTACCACTCACTTCCTTCATACACGTGATGGCTTTCACTCCCAACAACAATGATATGATGAAATGCGTTGCACGACGCAATGAGTGTGCTGTCTCTGTCAAACATTAATTATTTTGAAAACCAAGTTGTTCACAGGTGATAGTCACAAAATATAGGAAAGAAAACACCATACACCAACAAACATTCCTCTGTACAGTGCATGGCCTCTGCACAAGTACAAACATAAAATACTAGAGTGTGCAAATATTTCAATGCTTCCATGCAATGAAGGGCTGTCGTGCAAATATGTCGTCTATTTATTAAAATCTTCCTTTCAGTTTGGGCAAGGGGAGATAGCTAAACATTAAAAGTTACCCCAAGACCAAAAAATGCATCTTGGGCAGCAAGCACATGGCCACTTTCCATGCTCACATACACCACTACTTTTCATCGCACTTTGTCATATCTTTCATGGGTTCGTTGTAAAAGGTCTCAATTAATATGGTGATTTTTTAAGATGTTTTGCAATGAAATTATGATGTTTAGTACAGAGGCTGGGTTTTTGTAGTGCTACCATTGAGTCACTAGGAAAACTCATGCCAATATATGTACACACACATACTCATGCTGAAAAAGCAGAGTTGCCTGTATTTTAATTATAATTCAAGTTATGATTCCCTCCTAAAACATCATACTCCAGCTTAGAGAGCCCAGACACCAGCGGTTGAGAGAGTGGCAGGTGATGGATATGCCTGTGCAAACCAGAGCAGAGTCAATAAAATTCATATTAGGTCTATCAGACCAGAATGTGCTTTTTTCCTCCCACAGAAATAAATAACACTCCTGGATTACAAACAGAATGTTCTCTTTATGGCACAGAAGGACTTACAGCTGCAACAATGATTTCAAACTGAAAGAATAGTAGCTACACAGGCTAAAACAATTTCAGTGAAAGTATGCAGAAAGTATTTCTACTGCTTGTACAGGAGAAATATTTTCGTCAGGTTCTGGCTGCACCGCTCCATCAATTACAAAAGCCTCTGTTGAAATGAACTCCTTGAATTCGGAGTCCTTTTTCAGGAAAGCCCGCCGCATTTGATGTGGGTTTTTCGGCAATGCACTCAGCATTCATTGCAAACAACGACAGGATATATTTGCACTCTTTTGCTTTATAAACTAACTGATTTTACATGAAAAATGTAACAGATTATTCATTAAAAAAAGTCACCGGACCAGTAACAACAACAAAAATATTAACTTCACTACAAGGCACAGTAATTGCACAAACTTCTTCAGAATTCCCAAGTGCCCGCCAGTATGAACTGCGATAATTTGGGCTAACAATGTTACTCGCAAGTGACCTTTAGCAAACTTGCACTTACCATACGCTCATTAGCAACCATACCTTCCATACCTTGAACTATTACAGAACTAAAGGAGTAAGTGTGACAAAAAATTGGCACCAGCAAGGCAGCACTACAACTTCTCTCTCTTTTTGCACTATATTAAAGCTCCACTAGGCCTTTTGGATGATGCCTTTTAACTGTGCAGGTCATCGAGATGTGCAAGAAAAATCTGCCTGCATTTATTCCTCATTTCGGTACAATGCAAGAATGAAAAAAAAAATCTGCTTTACAAAACTTGGCTTCGAAAACAAATTACGTTCACACTTGGGGCTATTGAAAGAATAGCCAAATATTTTCCACAGCTTTTAGTAAACAACTTTGAGAGTAATGAAAAACCTGGCAGTGATACTTCAAGGTAGCAATTTTAAAATTTTAAATGAAATCCTGCTGTAATGTTTCAGAATCTCATCCTGATACGTATTCTTCTAAATTTTGCATATGTGCGTACCTCTTCACAAGTTAACTTTTTATCAGGAATTTGGGATAACACTTAGCTAGCGTCACTAAGCCTTGTGGCCTAAGCTGCTTCAATGGGAGATGCCATTTTTTGGTAAAACCAATCCTACAGAAAATGGACTATGCTTTTCTACAGAATTTAGATTTTAAGTGATCCGTGCACATTAAAGGCAGTAACGTTTTGATGCCTTGTCCAACGTTTGTTTCAAAACCATTCAAAGTTTAAGCATTGCATTATGGTGCCACAAGTATGAAAGGATATGTCTACAACACTGTGCACCACCAGCATGGAGTAAGCGAAAAAATATGCTCATGATTACCTGCTTTGCAAGCAGCTAGAGCGACATGAATGAGCAAACTTGCATATCGACGCCTAGTGGCAAAACCAACGTACACAGGTATAAAAAAAATACTCAACACCCATGTTACTTTTTTTTCTTCTTATATAAATAATATATTCCAGCTTCTGCTGTTAGCCTCATAGAAAATTCTGATTCCCTTACTCTTCTTCGTCACATGGGATCGTGACGTTGACCTGGAAAACCAATCATTACCAAACCTCGCACCATGCTGATTCAGCACGCAGGTTACACTGTTCTCGATGGCAGATGCTCTGAGGACCTAAAGTACTATCTTCCTTCTATGGCGCACTTTGTGTGACTTTTTTAAAACCAGTGCATGTTCCACAAAAGCAGTCAATCATCACCAGTACATCATCCATATTTTTCACAAACGCTGGGCTTTCCACTGGATCTTTTATCGTAGTCATGATATGCAAGCATTCACAAGCCTTAAGGACACAGCATACAACATCAAACATTTGTAAAAACAAGAATTAAGAAGTCCTACTGGTGCTGCAGTAATCTCTTCACATTCCATTAACACTTTTAGGTTTCTTCTTAAGTAGCAAGCTTTTTCTACTTTAATTAAAACTAGGGTAAAAATGAGTACAGTGATGCTTATTCTTCACCACAAATATTATCGCACAACTGATTTTTGTCAAGACTAATAAATCGTGGAATTTTTAAATGATGACCACCGTGGATTGAAAATCATAACCTTGCAACACATTGTCGATGGCTACAGTGTATATAAAAAAAATTTACACTAACTCCAGCATGGTGAAAAATTTTATCTCTGAGTACACTTTTCCAAAAACAAAACGCAACAGCTTTTCTGCGCCAATTACACTTATCTTCAACATGAGATGGCACTGCTTTTCCTTTCAACTTTAAGGATAACTTATTCCACAACCTGTCAACTTACGACTTCTTGCAAACATCGCAGCTACCTTCACTGAAGAAATTATGCAAGCAAACCATAAGCAAAGCCACAAGAAAGGCTAAACAATGAGCCTAAAAGCAGTACAGTGTTTCTCACACAGGCAGTGCGCAAAAATTTTGATACCTCAGCAGTTATCAATTGGCCTTGAGGATCATATCAAACATGCCCATAACTGCGAGCGTCATCTACCGCACCTGGCCAAACCACACAAACCAAAGTTCAGAAAACCTCGTGACAACACATTAAACATGCACTGAGTCCAGGACTTAAAAAGGTGCTACCATCACTCACATATTACTTGCACACATTGCAAAGGATTCACACACAAAACATCAGCAGTTCCAATCAGAGCAAAGTTCCTGGCCGAGGAGAACTGCCACCACTAACAGGAGAGTCCAACTCAGGGCAGGAATGCCAGCAGCGCTGAAGTATGGGCAGTATGGGCACTGGTAGGAACCACACACTTCACAGATGCTTAGCACATAAAGGTCATAGTTTTCCCATATCTTCTCCTTGTAGTTCTCTGGAGAGTTGTATTCATACACCTTGGCTTTCAGGTACCTCGTTGAGTTGGCAACGGGATACCACCGCTGTATGAGGAAGTTGACGCAGGTCCATTCGCTTGTGAGGTATTCAAACGTGAACACAGCTGTCCTGGGGAGGACCTTAGCCATGCTGAGTCGAGGAACTACTCCTGACTTCACGTAGTTGTCCAGGTGCGGCCGGTAGTTTGCGTAACCAGTAGGGATGGCCAGTTCAAGAACAGCCGCCCCACTGGCTGCGCTCATATTGGTCAATGTCCACCGAACACAGCTGCGGATGTTGATGTGTGACCTGTTCCTCCCAGAGAACTGCGGCATCAAGGTGAGATCAAACGCCTCATATGCAGGAGGTACAAGCAAGAAGTCACGATCGACGTTGTAGGTGACATCCAACTGGATAAGAGACAGGCCGGACCCTTGAGCCAGCACGTCGCAGTGACCCCAAACGTTTGGTTGGACATCAAAGACTTTTCTGCGTGCAAGGTCATCCTTGGATATGCGCAAGGTGCTGGGAAAGCCGGGGTTTGATGATGACTCCACAGTGACCTTCATTTCTGTTATGCCTCGGACGTGCGTTCGGAAGGAATACTGTGTAAGCGCTTCGAGGGCTGCAAGTGTGTCCTGTGTGCCGATGAAGCCATAGTCTGTGGAGCGCATGCGGTTCAGCCAGCGCACGATCTGGTCCTGGAAGAGGCCGCCAAGTTTGAGGTAGACCTGCAGGGCATACGCAGTGGCCTCCACAGCGGACGAGTCCTCTGGGTGCGGTAGTCGGGGCATCAGGAACGGCCGCTGGCTCTGGGACACGATCACGGGCGGCGGAATGTGCACGCTGCTCCAGTAGACAGCCCCCTCGGCATCTTTCTTCATGGATTCCAGCAGGTTGTAGCCGAATTGGGCTTCGCTGGAGCCAGCCTCAAGGAGTGCGTAAGTCGTCAGGGCTACGGTGTATGGGTCCGTCACCTGGCCCAGCTGTGTCTCCAGGTACCGTACGCCGTCGCGCTTGGCAGTGTTGGCATGCTCTCGAATGCTTCCTGTAAGATCGGTGACCTTGGAAAGCGTGAGAACAACTTGCGCAGTCAGCGCGATGTTTTTGTCCTTGTTGTTAGCCTCCGATCTCATTCTGCGATTCGATGGGTTGCGCGTGGTCTCGTAGAAAGCGCCCGTGGTGTCCTGATGCTTGAGCAGGAACTGAACTCCTTGAGTGATGAGCCGAGGATCGATGTATATGTAGTCCTCCCAGTCCGGAAACACCGTCAGCATGTACATCCGTAGGCTGAATGCAGTCAGCCAAACACTAGGCTCCTTCTTGAACATGGTGAATGCCCCATTCTTGAAGTAAGAGCTCTGATAAACGTAGGCTTTGTTCAACTTCTCAAAGATGGGCTTCGCAATGTCGCGGGTCAGCTGGTCTGTGAGGCGCAGATAGGTGAGCCGTATAACCTGATAGGCGAAGCTGAACATGGCATGCTCCCCGGATTTTGTGGCGCTTGAGAACCCGAACTCTTCTGG |
| >SG12013193  TTGACTTTTGTTTTATTGAATAGTTAATAAAAACGTTCAACTGCTTCAAATGTATCATGAATAGATTGGGAAACTACACACATTACTCACAACCTACTTTCAGAGTCACAGCATGGCTGCTTCAATACTGGAATGCACACCAATTCCTTGTGAAGCAACTCACACAACTGCGAGACCTGAAGTTTCACCCAGAGGCATTTACAAGAACATTTTATGTCTAAGTATTTACAGTTTTTTGGCAAGACAGCAAGATAAATAGTCCAGAGCAATGTGTTTTGGCCTTGGTAAAAAACCACTCTATATTATTGCTCGTCCTTCTTGCAGCACTTGAACGTTGCACGAGACATAAAAATGTGAACTCAATATATGTACCTTGTTCCAGGTGCCGAATACAATGTTAGCAACAGTACGGCAGCGATGCAACATTTGCTTTCACTGCAATACAGCATGTCATTCAACTTCTTTTTCAACAAGATGCATTTATCTGACGTCAATAACAGGTGAGCAAGTTGGCAGAGTTTTTAAGTTTGGGAGCTTGCAACTGTGAAAAAGCTAGGGGCATTGAAAGACTGTCTCTTACAATGAAAGCAGAATGAAAACCTGAAAAAGCCTCATAAAGATTGCAAAGGGTTATGATTATTTAAGTAGTCTTCTCAGTATGCCATTTGTTTCATTTTTGCTGAAGGACTTCATAACAACTGCTTTCAAACAGCAGTGAATCAGCCATACACTAATATTTGCAATGAATTGCTACTAACTTGAACCAAAAATTACAATTCCTTCTGGTAAGCTCTCACGGTAATTTTTGACCACATACATACCTTTACGACTTATGAGCAGGCCTTCCAGAATATTTTAGCAAATGAAAAACTGTGTGTAGCAATAATTTACTTCACATAAAACGTCATACAAATTGGGGGCAGTACATCTGACTTACCTCCAGTGAACATTTTACCTCTTTAATCACAATGCCTTCCTGTGATGTTCAACTGTTTTCACATGAAGAATGAGGACTATTGCTTTCAGATTTTCAGTAAAAAAAAAATGAAATAGATGAACAAAGCTCGCAATAAACAGTATTCATCATCTCTTCTTTCAGATGGGATGTTCAAAAATTAACACTGCTGATAGCACATGATCATACAAAATGCAGCATATGAAAGAATGCAAAAGTAAACAAGCACTCAACACATACACACAACTCTTGCTTAGATATGTCAAACTCTAGATGACTACTACTAGATGTTCATAACAGATGAATGCCAAACTAAGAATCAGCTCTGATGATCCAAAGAGAAAACTGCTGTAGCTAAGCTGAAAATGAACTCTCAATTTATAAACCCAAAAATAATGTGCACGTCTCCTGCACAAGGCCAAATGAAAGGTGCATACTAAAACCAGTACTCTAGAAAAATGCACAAAAAAGGAAAGGCGCCTAAACTCTGTTGACTGGCAGGTGCCTTCTTCCCAGGGCAGCTATGCTGAGCCAGGCAGTAATGCCGTGTCCTCTCTCGCAATAGCAAAATGGAACAGCTCCACACCACAATTCAGTGGCGAAAGCAAGCATTGAACCTGCCTCCAAACGGAATGATCCAGGCAGATACTGAAAGAAAAATTGGAGGCTGCAGCTCTGCAGAGCCAGCCCAAACAGGGAACACGTAACGTGCTGCACAAATGAGTCTTGACTGAAGCAGACTGCACAGCTGCTGTGGCAATGTCCTCTGAAAAGTTTGTTCTTGTTGCTTGAGAAAAATAACTGTCACAGCACCACAACTACTGCTAGCCTTTGAAGAGGTGCCTCCACAGCAATACCAGAAGCACAGTCAGGGGCAACAGTGCTTGCCGTTGGTTCAGGTGCAAAAAAGAGGGTGCTGTGTTGAACACTGGGCAGAATGGGCACTGGTAGGAGCCACACACGTGACAGATGCTGAGTGCGTACAGGTTGTACACTTCAAACATTGACTCGTTGAACCGCTCCGGTGCATAATAGTCGTAGACACGAATTGAAATAAACCGTGTCATGTTGGCAACAGGATACCAACGCTGGGCAGTAAAGTTGACACAGATGGGTGATGTATCGAGATAGTCAAAGTAGATTTCAGCCTTCTTCTCCTCATAACGAGCTTCCCGAAGGTTTCGCACAGCTCCAGATTGCACGTATGCATCCAGAGTCTGCTGCTGAATGTAGTAACCTGATGGCAAGTTCACTTCCAGCACTGCCATGCCACTACGGGGACTTTCATCAGTACGGATCCAGCTTTGGCACGACCTAAATGAAACATGAGAGCTGTTCCTTCCATATGAATACTGTCGTATGTTGAGTGCAAATGCAGGCACTGGCGGAGGTGTGACCAAATGCTTCCATGTGTCAACATTGTATTCCATGTGCAGCTGGACAATGGCCAGTCCTGTTCCTTGAGCTTTCACAATGATCACACCCCATGCATTGGGAATAGACAGTGTCTGCAGCTTGGACAAGTTTTCAGGTCCAATGTGAAGCTCCCTGGTGAAACCCGGTGTTGAAGGAGCCTCCACAGTGACTTTGATGTCAGTGACATCGCGTGAGCGAGAGGTGATCGAGAACTCTAGCAGTGCTTCCATCGCCAGCAGTGTGTCCTGAGTTGATGCCCATCCACCATATGAGAGGCGCTGTGTGTTCAGCCATTCTACAATTTCCTTCTGAACAACTGCCTGGCGTTTTACATGCACCAACAGCCCATACGCCGTAGTCTCCACATTGGATGCATCATAAACGTAAGGAAGTCGTGGGTGCAGGTAAGGTTTGTTGTTTTCAATCACTACTGTTGGGGCTGGCAGATCAGTGCGAGACCAGTACCTCATGCCACTTGTCTCCCTCATCTTTTCATCAAGGAGGTTGAAG |
| >SG12031630  TGACAAATATCTTGTCATTGCTGTCTGGAATCTCGATCCTGTTGCGCTGTTGGGCGTTGTCTCGCTTGATTCGAAGGGTGCGGTTAAAGTTACGGTCCCCGCTCAACGTCACTTCACAGGTCAGGTCAATGTCTGCGTCTCGTGCGTAGAGGGCATACTTGGACAGGGCTTGAAGCGCCACCACGGTATCCTGGCTGGACCGCAGCGACCCGCTGGGGTTCATTCGCAGGTTAAGCCATTGCACCATGGCCGTGATGTCGTCCCTACTCTCGCCGGCATTGAGCAGTGCCATGAGCGCGT |
| >SG12040320  CGACATCGAAACGAGCAAGACAGTGGTCAACTGGCTCAATACGCATCGCTCGGCGTCCGGCGCTTTCGCATCGACACAGGACACCGTGGTGGCCCTTGAAGCGTTGACGGAATTCGCTCTGAAATCTCGAGAGCCAAATGTGGACCTCACGTGTAATGTCACCCTTAGCAGTAAACGGGGCTTCCAGAAAAGCATTCGGCTTAAGCGTGACAACGCTGCCATCCTTCAGCAAGTGGACATTCACGACATTAAAGGCAAAATGTTCGTCAAAGCCAGCGGCACTGGTAGTGGGCTGCTCTCGGTCAAGTTGAAATACAATGTGGTCGTGCCACCAGAGATTCTGTGCAAGTTCAATATCACGGTTCGGGCTGACATCCACAAACCATCAGCAAAG |
| >SG12038200  GTCCCTGCCGAGTCGTTTACAGACGCCATTGGAAGGGCGGCCTTCCTCGTGCAGACTGAGCGACGTCACGCTAAAATCAAGGCAAAGGTTCAGACGCGTGATCCGAAGTACCACTCCCACCAGCAGTCCGAAGGAAGCATTACCATCGATGCGTTTGACTCTCCTGTGCAAGCGTTCGTTGCTTTGGAACGCATGGACACAAGGAGATCATTCATGGTCGGAGAGTACTTCGACAGCCAGGTGATTACTGAACCAGCAGACATTTCGCCAGTCTACTATATGGTGACACATCGTGGCCAAACGAAAGTTCATGGCTCTGTGGAAGGCAGTGGGAACAACCCACACAGATCCGTGCACTTCGCTGTGACGAGGGACATGAGTCCAAGAATACGCGTACTTGTGTACGCCTTTCATCG |

| >MG120 46839  CGAGAATGGCTCCTTCTCTTCATTCAAGGATGCCCCAGGGAGCGTCTGGCTGACGGCGTTCGCTACACGCATTATGTGCCAAGCGACACGCTACGCAAGCATCGACTATGACGTCATCAAGACAGGCTTGGCGTGGCTGATACGCAAACGGCAGGAGAGTGGAAACCTGCACTTCGAGTGGCCTTTGGTGGACAAGAGCATCCCGGGACCTGGTGATAGAGCCGTTTCTACGACAGCCTTCATTGGCCGCACTTTCAGGGAGTGCTGGCACGTGGTGAAGCCTGAAGGCAGCGACGTCAATACCTGGCAG |
| --- |
| >MG120 20515  CTGTGAGATCGACATCGTTGGTGATGACATGGGCGCTGCTTTGGAGGCGTCCGTAAAGAACCCGGGAACGCTTCTGAGGATGCCACATGGCTGCGGTGAACAGACCATGATCGGCCTGGCACCAACGCTGTACGCCTACGAGTATCTGAAGACCGCCAGCCGCATATCGCCCGTGGACGAGGACAGAGCGCTGGGCTTCATCAGCAGTGGCTACCAGCGGATCCTCAATTTCCGGAAACCTGACGGGTCCTTCGCGGTTTGGAATCATTACAGGTCCAGTCTATGGCTAACAGCATTCGTGGTGAGAACTTTGTGCGAAGCCCGGAAGTCTATCCTCATCGACGAGAAGGTCGTCACAAGTGGGCTCCGTTACATTTTGACCCAGCAGAAGCAGGACGGCA |
| >MG120 1594  GGAAGCACTGTGTGCATCAACTCAGAGGACGGAATCGGCATCTCAGATCCGGCAAAGATCAAGGCCTTCCAGCCGTTCTTCGCCTCGTTCAACCTGCCTTACTCTGTGGTGCGCGGAGAACTGGTGCCCGTCAAGGTGTCCGTGTTCAACTACCTCGAAAAGTGCCTGCCGGTCGACCTGAAGCTGGCTGAATCCGCGGACTTCCACATTGAGGGCGAGTCCGAGACGACGCTTTGCGTGTGCGGCAGCAAGAGCCAAGTGCACAAGTTCCAGATCCGGCCACAAACTATCGGAGAGGTGAACCTGACCGTGTCGGCGGCCGGATCGCAGAGCGATGCCGCATGTGGAGAGCAGCCAACTGAGAAGGTGGTGGCCAGGGACGCAGTCACACGACCGCTCATCATCGAGGCGGAAGGCTTCCCTAAGGAAGAGACGCAGAGTGTCTTTGTTTGTCCTAAAGACGCTGCAGAAGGCGGCGCAAAGAACGAATTTGACTTGGTGCTTCCTGAAGACTTGGTGGAAGGATCGGCCCGTGCCTACGTTCAAGTCACAGGAGATATTATGGGCCCAGCAATCAAGAACCTCGACTCACTGGTGCAAGTTCCAACGGGATGTGGTGAGCAGAACATGGTAAAGTTTACGCCAAACGTCTATGTCCTGGACTACCTTAAGGCTACGGGCAAGAACCAGGAAGACATCGAAAGGAAGGCTGTGCAGAATCTCAAGACAGGCTACCAGCGCCAGCAGAAGTACAAGCACTACGATGGTTCGTACAGCGCCTTTGGAAACCGCGACAACTCGGGCAGCATGTTCCTCACGGCGTTTGTAGTCAAGTCCTTCAAGCAGGCTGAGAAATACATTCCTATTGACGCCGGAAACCTCAACGAGAGCATAAAGTGGATCATCACGAAGCAGAAAACCAACGGCTGCTTCCAGAACATCGGCACTGTTCTTAGCTCCGGCTTGAAGGGCAAGGTTAACTCCACTGCCCCGGGAGCGCTGACGGCGTACGTGCTCACCGCTCTTCTGGAGGGCGGCCTTGCGGACGAGAAGGTCGTTGAGTCTGCGCTGCGCTGCATCAGCGCCCAGCGTGACCCCAGTGCCCACAACTTGGCCCTGTCCGCCTACGCCGCGGCCCTGGCGGGACATGAGTCTGCCAAAGATTACCTCGAGAAATTGGAGTCCATCGCAGTTCACAAAGGTGCCCTGACCTACTGGAGCAACGCGGGCAAGAAGGGTCCATCAGCGTCGGCCGACGTGGAAACGGCGGCGTATGCAGTGCTCGCCTACCTCAAGCTGAACGCCCAAGAGAACCTGAGCAAGGCGCAGCCCATTGTCCGCTGGATGGCGACCAAGAGAAACAGCCGCGGAGGCTTCCCATCCACACAGGATACAGTTCTCGGTCTCCAGGCTCTGTCGGCGTTCGCTACTTACGTTAGCAAAGATCCCGTTGACATCTCAGTTAAGGTCGACGGTACTGACGTCAGTGAGTCGTATAACCTTAAGGAGGACACCAAACTGGTCGTACAGGAGAAGAAGGTTGTCAGCTTGCCCAACAAGCTCACTTCAGAAGCAACAGGTCCTGGATGTGCCCTTATATCGGCGACTCTGAAGTACAACGTTCACACTGCACCGAAGAGCGAGGGTTTCGAGCTCACAGCCACTCCATCTCAAGAGGCGTCTGACTGCAATGACCACAAGCTCAAAATCTGTCTGAGGTTCGACGGTGAGCAACCATCCAACATGGCAGTGGTGGAGCTCAAGCTGGTATCGGGCTACACTCCAGATGAGGATCACATCTTTGGACTCTACCGTGAGAAAGACGTCAAGCTGAAGAGGCATGAAGTTGAAAAGAACCAGGTTAACTTTTACTTTGAAGAAATAACGTCCGAAAACAAGTGCTTCGACGTTCGCGTTCACCGCGAGTTCGCTATTGAAGATGCCAAACCTGCCACAGTTAAAGTCTACGACTATTACGAGCAAGAAAACAGCAACTCCGTGCCCTACTCTCTTGTGGCGTCCTGCTAGTGAACATCGGCCATCAGTTTTTCTTACTCTCCCTTTTTTTTATGTCTCTTTTTTTCAAACGAACGCGCCAAGACAATAAGAGTCACAACATACCATTCATTCAATGCGCATTTATCCTTTGGTTGCCAATTTTCTTTTTACCAGAATCATGCTTTCTTGCTCCCTCGGTAGTGTTCAACACCAAAGGCTTTTTATACCGGTCACACTTCTTGAGAAGGACATACGTGCCATGTCGCGAACGAAAAAAAAAAGATTAATGGCTTCCACAGCGCACATCGCGAAGAAATCAAGACAATGGTCACATGATGTTTTATCTTACAGCCTCGATATTGGGTGCGGACGACTGAGGGAGCGCTGCGCAGTGTGTGCTTTTGTGTGCGTGAACAGAGCCGTGAACTTTTGACGTCGCCATGTTGTTTCCCGTAGCGCGTCTTATCCAGCTGAGTGGAAGAGTAGCTTTATATATTTTCTACTTGCGAGAAAAGCGCGTCATCGTCTGCGAGGCTTCACCGTTATAAATAGTGTCATAAAGAGTGAATAAAACTAATTTTTTTCACAAAAAAAA |
| >MG120 22300  ATTGAGGATGAGAGGAGCGATTGCTAAAATACAGACGAACACAAGGTATCACAAGGCAGATGACTCGGAGGCACTTGAGATCGTTTGTCCAGATGCTTAGAGAAGTGCGGATGGATGGAAAGGAAGTGAGAAAAGATCTCTAAACGAAGAACACGCTGTCCACTTCGTTGAGGCTCGTGGGTGGCGCGACTTCAGCGGGCGTGCAGTTGCCCTCTACTCTGTAGCCAGCCGTTGCCTTGTACTGGTACTGGTAGTAGTCGTACACCTCCACCACTGCATCCTGAAGGTTCGCCACTTGCAGGTACCTGCTTTCCGTTACGCTGAAGCACGTCGGCTGGATGGTCAGCGTCTGAATGATGAGCTGGATTTGGTTGCCGTTGACGACATACCGCTTAACGACGCCACTGCTCACGAGATTTTTGAGAGACTGGTCATCTGCCGAGTATCCAGAAAGCAGGGTGATCTGGACGATCGCGGAGCTGCGCAGAAAGCCTTCGGTGTACCGTGTGCATATTTCCAGTTCAAGGGTGCTGCAATCTGGTCCAGATGTCGTGGACACGTTGACTTGTATTCCTCGCTGAACCGGACTGTTCCTGGAGTTGTAGTAGTACTTAACCTGTAAAGCGGCGCACCCTGCGCTGCCGTCAGCAAGAGATGCCTTGAAGCTGTACGAATCGCTTCTGTTCAAAAGACGCTCCTGGTATAGCAGTTTGTTCTGCTCAGAGATCGGGAAAGAGACCGTTTCGGGGGATGAACTGTCGACAGCAACGTTCATAGTCAAGTTTGTGTTCTTCGAGAATGCCACGCTCGAGTACTGGGTCAGGGCTTGGATGGCGACGGTGTAGACCTCGGAATAGGAGAAAGTGTATCGGGCGTAGGTCTGCTGCATGAGCCAACGGACGATTGGCAGCGCTTCTCCCAGGTTTCGGTCGAGCAACTTCAAAGTGAGAACTGCGTAACCAGCGGTGTCTGCCGAGCCGTAAGTCAGGCCATCGCCGCTCCACGACGTCAAGCCACTGCTTTTATCAGCTTCGCTCAGAAGCGTGTCCAAGGCGCTGTTAGCTCGGTCCGTGCGTCCAGCGAGCGCCGAAAAGTAGGCGTTCAGCGCAGTCGTGTGGTTGGAAGGCAGATTGCTGGCGTCAATGCACTGGAGGACACCGTTCGTGATGTTCTCGTAGTTGTAGCCACTCTCCAGAAGCATGACGCCAATGGCCGCGGTGAAGTCGGGGGCTGTCTGGGGGCCGAAGGGTGACGAGCCGGGCTGGTTCTCAACGAAACAACCGGTGGCAGCGTTCCAGTGCTGCAGGACATAGCGCACGCTAGTTTCGATGTCCGCATCGACGCTGGTGCCCAGGTACTCCTTGGCGGCGCTCAGGGCTTTCACGGCGAAGGCGGTGAGGAACACGCTTCTCGGGAACTCGCTAGAGCCAAACTGAGCGTATGAGCCGTCGGAGGACCGGAAGCTGTTTTGAGCCTGGGACGCTTGGCGAATGCGGGATCGGAGGCCGGAGTTCACCGTGTCAGTCAGAGTCCCCGTCTGCTCCAGGTACTTGTGGAGGTAGACGCTGGAGGCAAGCACAGCCAGTGTTCCCTCGGCGTTGGAATAGGTGATGGTGGGAACGGATAGGTCGTTCAAGCTGAGAGCGAGGATGTCGCCTGTGCCCACTAGTACAACCTGCTGGGAGCCTTCAACAAGAGCGACAGGTGTCGGAAGGTTGACTTGCAGGGTGTCCGGAGCGCTGTCTGCATCACCTGAAGCACACAGGAGGAACGTGTTGATTTCCCTGATGGGGAAGCCTTCAGGCCTAACATCGATGGTCTGGATGACAGTATCGCTTGAGTTGACGTTGGACAGTTTGACGTCTGGATACTGTGCCTGGGCCTCTGGTCTCGTCTGGACGCGGGCTTCCAGCCGGGATTCCTCAAGCGTGGTAGCGCGCACGTCAAACGGGAATGACGCGGATGTGCTGGAGTTGGGGCAGAGTAGTGCGTCGGTGCTGTTGGGACCACCGACCACCTCGAGATTCTCCAAAGACCTCAGACTGACCCGGACCGCGACGCACTCTTCGCCGTAGTTGAATGCCGTGAGAATGACCGTGGCCACTTCGTTGCGCTGCATGTACGCCGGCAACGTGAGCGATACGAATAATGGCTG |
| >MG120 34566  CTGAAGCAGCATTGCATTGCTTGGTTCCACAAAAAGCTCTTTGGCCTCTGTGTGTTGGGATGACACTCTCATTGACAAGTGTGTGCTTTGCAGAACAGTTGCTTTGGCAAATGCAGCCAGGGCTTGCAAGGCTACAATAGTGTCCTGTGTAGACGCGAATCCTCCATGGTCACTCCTCTGCTTGACCAACCACTGGACTGCGGGCAAGGCTTTAGGTACACCTGCTACCTTGAGCAGTGTCATGCAGGAGAGAATTGCATAGCCGCTTGTCTCTACAGCCACTCCCAAAGACACGTTGTGGCTCCAATGTAATGTGCCATCAACGTCTGATTCAGCCCTGCTCCAAAGCAAGTCAAAAGTTTGCTGTGCATCATGATGGTTTGAAAGGGCCAAGGCATAAGCCTGAAGTGCCAGTGAGTAGCTGTCCTGCTCGTGAGGAGTCTCTTCTATGAGGCATTGGATGGCTGCATTTAGTACAGATCTGGAGAGAGTCACATTTGCTTCAAGCAGTGCGATTAACACATAGGATTCGCGTCT |
| >MG120 26873  CTGAAGTTTATTGCTGCGTTTTAAAGCTGGGGCAAGTCGTTCGCATCGAGAAGTTTCGAACACAGTTCGTACTCGCCAATGTTCGGTGCATGAATGGTTGTTAAACGAACGCACGCCATCTGTGTGTTATGTTAATGACAATACCGGTATTCTGCTGGCGAAAAATCACATTTACATTACCGTAATTGCCATAGCCATGAAAAGGTTCGGGCCACTCATAAGAAACAGCGAGTTCTCTCATCGGAAAACTCATTGATGAACCACGCCGCAAGTCTATTCTGTTGTTTATTCTTGGGCTTCGCGGGGAAAACCATCGAGCTGCTGTCGATCAGATACAGGAACTGCTTGGTCTCGAAGAGGTCTTTCTCGATATACGTGGAGTCTTTTCCCATGACAATATACTCCTCGCCATTGGGAATATTGAATGTATTGCAGGTATCACGAGCCTTGATGCGACGTATCTTTCCTTTCAAGTCGTCTTCTTGGCCTGGCTTGAGAACTTGAGTGATGAGGAAAGCCACTTCTATGAAGCCATCCGTCGATACATTGGCTGTCGAATTGCCTCTCCATACGTAGTGAACTCCGTCACAGGCGAATTCTCTCAGAAGCTCACGCTGTTCTTCGTCTTCAAAGTATTCGTTGTCCCGTGTTTTTATGAACCTGTCGAGTGGCTTCTCCGGCGGGCAACCACCTTCCGCGCAAACGCAGACATCGGAGTCGTCACACTTATCATCAACCTTTAGTAGGGGGCTTGTCTTGTCGGGCGAGTAGAATCGTGTGCAAGAAAAGTCTGGTTTGTAGTAAGAGTATGCCTTCACGTAGCTTGACTGCAGTTTACCTGCATTGAATTCTTGTTCGAGAGAGAAATTAACGCAGTGCGTCATGTTTGCAGCAATGAAGGGTACGTAAAAGTCAACATGGCGACTTGTGATAGTATAAAGCTGAATCTTTCTTTCTTTCACGAGCTCATCCAACTCGGTAGCATTGGGC |
| >MG120 28446  CGAATAATGGCTGGAATCCGGTGACGTTCGCCACTTGGGAGACGCCGAGGCCGTTTCGCGGGTGCAGGCATACGGCAGTGCCCTGCCATGTGGTGATTGTGTCCGGGAGCGTCTCGGAGTACACCAAGGAACCATCAGGGCTCACTCTCTTGATCTGCCAGAGCCATGTTTCAGGGAAGAGTGTGCGGACGGAGTTTGACCCCTGGACGACGGGCGAAAAATCGGCTTGCGCAGACTCTGCGATAGGTGCCGGTCCAGCAAATCCGGGGGAGGCACTCGGAGCAAATGCCACTTTGTTCACAGCTCCGAAACCGCCGAAACTGGGATAGTAGCGATTTGTGGATCCCAGGACTTGATTGTTGGGATAGCCAGGGAAAGCACGGTTGGGACACGGCCTCGTCTGGATTGGCAGGTTGGTGAAGACGACAAGACCAGTGTTCTCGAACGAGCTCAGGGAGTCGTAGCTGAAGGAGACTGAAGGGGCCACTGTCTGCCTCCTCCTCCGCACCTTGAGCTGGGAGTCACCCGACTTCTCGTAGGGTTCGTTGTAGAGATAACCGTCAACATAGCAGTAGCTCTGGTTGACGAGAGAAGTGCGGGTGTACCTGTAGTTGAAGTAGCTGATCTGGTTGAGGAGGGC |
| >MG120 46668  CCGGCGTGGCCCTCTACACGAGAGAATGTGTCGCAGCCTTTGGCAAGTGCTGCATCCATGTCGAAGACAACCAGGCTGTCGGAAGGTCATCTGCAGAGGAAGCCGACGTGTTGGGCATGAGCAACTTCAAAGATTTAGAGGGTGAAGAAAGGCGAAACTTTAGAGAGACGTGGATATTCAATCAACTAACCCTACGGGAACATGGAGAGGCTAGCCTTGAGGTGACCGTCCCAGACTCGATCACCACGTGGGAGGTCAGCGCTGTGGGCGTGGCCCCATCTGGCGGGATATGTGTACTTGATCCTCTCCAGATTCCGGTCTTCAAGAAGCTCTTCGNCGAAGTCAACCTCCCTTACTCCGTGATCAAGGAGGAACAGATCGAGATACCGGCGACGGTGTACAACTACGACCACAAGGATCTCAAGGTTAGGGTGGCG |
| >MG120 39707  TCAAAGTGCTGCCTCACGGTTTGCGTGGGAAGTTCGACAACTATCTGCCACAGACCCAACTCAGGCTCTTCTGTAAGCTGAAAGTCTCTCTGAACAATGCCTTTTTCAAAAGACACGTCGTTCCACTGAGCTATTCTTACATCGCTGGGACTTGTCACGTAAATAGTTGCCTTCACATCGGTGACTGGTTTCAGCTCATTGTTGATAGGGAGGACGCGGAATTGAACCCTCTGTCCAGGCTTATAAAGTGCCTTATCGGACTGAAC |
| >MG120 10721  ATGAAAGTCCCCGTAGTGGCATGGCAGTGCTGGAAGTGAACTTGCCATCAGGTTACTACATTCAGCAGCAGACTCTGGATGCATACGTGCAATCTGGAGCTGTGCGAAACCTTCGGGAAGCTCGTTATGAGGAGAAGAAGGCTGAAATCTACTTTGACTATCTCGATACATCACCCATCTGTGTCAACTTTACTGCCCAGCGTTGGTATCCTGTTGCCAACATGACACGGTTTATTTCAATTCGTGTCTACGACTATTATGCACCGGAGCGGTTCAACGAGTCAATGTTTGAAGTGTACAACCTGTACGCACTCAGCATCTGTCACGTGTGTGGCTCCTACCAGTGCCCATTCTGCCCAGTGTTCAACACAGCACCCTCTTTTTTGCACCTGAACCAACGGCAAGCACTGTTGCCCCTGACTGTGCTTCTGGTATTGCTGTGGAGGCACCTCTTCAAAGGCTAGCAGTAGTTGTGGTGCTGTGACAGTTATTTTTCTCAAGCAACAAGAACAAACTTTTC |
| >MG120 22574  GGACCTTTTGTGCAAGTTTGATCTGAAGATAAACTTCACACAGCACAAGGTCAACGTGTCGAAAGCGTTGGAAGGTAAAGGGAGTTTCAAAGAGATTTACTCGATGGAAGTCTGTGCGAGGTCTCTTGAAAAAAAATTGAAAGGCATGGCCATCCTGGACGTAGGTCTCCTAACCGGCTTCAATCCCGTGTTGGCCGATTTGGACAAGATGGTGGCCGACAAACGTGTGGACTCGTACGAGTTGAGCCAGCGTAGCGTGGTATTCTACCTGTCGACCATTCCATCGAACGCCAGCGTGTGCGTCGAGTTTGGCCTGCAGCAGGCATTCGCCGTAGGCAAGTTGCAGTCGGGCTC |
| >MG120 25317  AGTTGCACTTCCAAGTTACAAATGAGGAAAGTTCGCATACTCAATATGCTAAGCACACATTTTGGTTCTCAGGTGTAAACTCTGAACAAGTGTATTTTTTTTGCTTACCACAAAAGCGAATAAAGCTTCAACTTAATAATTATATGCTTCTTTCAAAAGACAGTTCTGGCTCTTCTATGTTGTTCCTTATAGTGTTCTTTTTATGAAAACTGGAGTGCATCGAGGTTCAACTTGGAGCACATCCCAGCAACTTGCAAGTTCCGTCAGTATGAAAGTTTTCTGCACGTTTATGTCCTTTTGCCAGTCAGGTCTGAGGAGCAGGCACTGTAACAGGCATGTTTGATTCAGGGCCATCAGGGAAGTCCAAGTCTTGTTCGATATTTCGGAAGTTTCCAAGATGAGTCCTGGCTGAACGAATATCTTCTTGCATTATTTCTGACTGCAGGGGCATTTGTGAGTCACTAAGGTCTTGTTGATCCCTCTCACAACTTGGAATTGTGTAGTTCCTTAGTACAATGACACCAGGATCATAGTAGTCCTGCAATACAACAGCTGCAGGAGCACTGTCTTGCACTTCAAACTCCTGGGTCAACATTAAGGTGATGCACATTCTTCGATTACTTAGCTCATCAAAGTACAAGTTTATTTTGTCAGGCTCTTCTTCAGTCTTCCTAACTTCCAGGCCATCAACAACCTTGCTCAAACGGAACCCGGTTTGCAGATGGACTTGAATGATCACCATTCCAGACATGCCCCAGGAGAGGAGGTAACTAGCACAAAGCTGTATCTTTGGCTTACAGTCAGCATGGGATGCCAGTACCATGAGATCAAACTTGTTGGTATCAGCCACTGCAGGAATGTTGAACTTTGCAGAGGCCTGCACAAAGGCACACCCCTTCCCTGTGACGTTGAGGTTCACCATCGCCGGCACTGTAGGGATGACCAC |
| >MG120 35422  CCAAGCTCCGAAGTGCCAGAAAGGGCTCTCTAGGAGGGCATTCATCTTGCTCGCATCGACACTCGTCTTCCTGGCACGACAGCTTCATAAGCGGGCTTGTGGAGTCAGGACCGTAGAATTGCGTGCACGAACGCGTCGGATCGTAGAAGTCGTACACCTTCACTACAGCTGACTGAATGTTGTGGACGACCTCCTTGCGTTCTGTCCTGAACTTGAGGCATGTTGGAGCTTCATGCGGTACGTGAGGAAAGTAAAGGATAACACTCTTCTCCGTCACGACGTAGTTCATCAGAAAGGGGTTTTCGATTCGAACCGCCTTTAAAT |
| >MG120 20509  TCTGGAATCTCGATCCTGTTGCGCTGTTGGGCGTTGTCTCGCTTGATTCGAAGGGTGCGGTTAAAGTTACGGTCCCCGCTCAACGTCACTTCACAGGTCAGGTCAATGTCTGCGTCTCGTGCGTAGAGGGCATACTTGGACAGGGCTTGAAGCGCCACCACGGTATCCTGGCTGGACCGCAGCGACCCGCTGGGGTTCATTCGCAGGTTAAGCCATTGCACCATGGCCGTGATGTCGTCCCTACTCTCGCCGGCATTTAGCAGTGCCATGAGCGCGTATGACGTGGCCTCCACCGACAGTGGCTCGGAACCGGCGGACACATACAGCCCTCCTTCATTCCCTCTCTGGAAAATGCCGCCGAGCCGGCGCAGGGTGTCGTTCTTTGCGGGGCTTTTGCCCAAGGAGAGCGCGTACGCTGCCAGCGAGAGCACGTAGGGCGAAGTGTTTGGATTCAGCTGTCCCTCGATGAACCCTGTTGCTTTTGCGAAGGACTCTGTCAGGCCGGCCACCTGAACGCCTTCTCGATCGCATTCTTGCAGGGTGAGAAGGGTGTACGCGGTCAGTGGAACCGGTCCATTCACACCGCCCAGGAGGTCACCGTGGATTAGGTTGGATATGTCGTGGAAACTGCCGTCCTGCTTCTGCTGGGTCAAAATGTAACGGAGCCCACTTGTGACGACCTTCTCGTCGATGAGGATAGACTTCCGGGCTTCGCACAAAGTTCTCAC |
| >MG120 30236  CTGCCATGCCACTACGGGGACTTTCATCAGTACGGATCCAGCTTTGGCACGACCTAAATGAAACATGAGAGCTGTTCCTTCCATATGAATACTGTCGTATGTTGAGTGCAAATGCAGGCACTGGCGGAGGTGTGACCAAATGCTTCCATGTGTCAACATTGTATTCCATGTGCAGCTGGACAATGGCCAGTCCTGTTCCTTGAGCTTTCACAATGATCACACCCCATGCATTGGGAATAGACAGTGTCTGCAGCTTGGACAAGTTTTCAGGTCCAATGTGAAGCTCCCTGGTGAAACCCGGTGTTGAAGGAGCCTCCACAGTGACTTTGATGTCAGTGACATCGCGTGAGCGAGAGGTGATCGAGAACTCTAGCAGTGCTTCCATCGCCAGCAGCGTGTCCTGAGTTGATGCCCATCCACCATATGAGAGGCGCTGTGTGTTCAGCCATTCTACAATTTCCTTCTGAACAACTGCCTGGCGTTTTACATGCACCAACAGCCCATACGCCGTAGTCTCCACATTGGATGCATCATAAACGTAAGGAAGTCGTGGGTGCAGGTAAGGTTTGTTGTTTTCAATCACTACTGTTGGGGCTGGCAGATCAGTGCGAGACCAGTACCTCATGCCACTTGTCTCCCTCATCTTTTCATCAAGGAGGTTGAAG |
| >MG120 34137  TCATGGTTACGTTGTTCTGGAGGCACTTCTCCACTTCGAACTGCTCGGAGTCAGCGATGACCTCGCCGTCGGGGCGGACGTAGAAAGCGAGCACTTTGACGCGTGGCACGTGGCTGAAATCTGGCTCGAGCTCGAACTCAAAGCTCCCTGTCGACACGCTGCCCTCGGCCATGTTGGTCGGAAGCGTCTCATTCAGGTCCTCCTCCACCAGGAAGCTGTCGTCAACAGAGAGCACCTCTTCCGGCTTGAAGGTCACATCCAGAACCTTGTCCTTGAGGATCTTGCCGCGAGCTATCACCTGTAAATGGAACTGCTTCTCTGAGTCGGGCTGACCCGTG |
