# Supplementary material for: A repertoire of protease inhibitor families in Amblyomma americanum and other tick species: inter-species comparative analyses
Source: Parasit Vectors. 2017 Mar 22;10:152. doi: 10.1186/s13071-017-2080-1 (PMC5361777; doi:10.1186/s13071-017-2080-1)
Supplement: Supplementary file 3 — FASTA sequences for Amblyomma americanum contigs from Illumina sequencing, by PI family. (ZIP 638 kb) [file 13071_2017_2080_MOESM3_ESM.zip › A. americanum I43.docx]

>MG4827183

GCAGGTTTTAACACAGCATCTTTCTGTGCAATTTGGCCAAACTGCCAACCTCACTTGCATTGCTACTGGTGATCACCCAATTACGGTAACGTGGCTTAAAGGTGAAAAGTCAGTAGCAACCAACAGCCAACTTTATGACAGAGTTATTGTGTCCAACGACACTCAGAAAGAACGACTGGTGTCTTCATTGATACTACAGCAGGTGACCGCTGGCGATGCAGGTCGTTATACCTGCAGAGTGAAAAATGCTTATGCAGAAGACACCAAGGCTATTAGGCTGAATGTCCAACAGCCTCCCTCTAATCCCACTGAGGTTGAAGTGTCGGATGTATGGAGTCGTAGTGCCAGAATCCGCTGGAAAAGCCCTACAAGTTCAACTGTTTTGTCATACCAAGTGCGCTTTTGGTCGCACCGAGAGGATGAAATGTTGAACCACACTGTCCGTGGTGGTGTCACCACGGCTCTTGTCCGAGACCTCCATCCAGCAACTGAGTATAGAGTCGCCATTGTAGCAGTAAATTCCGCAGGCGCAAGTGAATCCTCTCCCATAATTCGATTTACAACGACGCAAGAAGAGCCATCTGCTGCACCTGTCAACGTTCGTATTGAAAAATCTGGGGCAACTTTTGTACTAGTCACGTGGAGTCCCCCACCAGAAAG

>MG4846872

CGAGAACCGTGTGGGGGCACCACTGCAGCTCGACTGCTACGTGACTGGTGCCCCTGAACCACGAGTATCCTGGTTGCACGATGGGGAGCGGGTTGAGACAGATGGCCATCGTGTGCTCATGGAGAACTACACGCTGTTGATACCAGCGGCTGCCATGTCAGACGGAGGCAAGTACTCCTGCCTGGCGGACAACGGCCACAGCAACGAGTCCGTCTCGGTGCATATCATCATGGATGAT

>MG482087

TTTTTGCATAACTTGGCTTTATTGAAATTTTTCTCCACAAGCAATCGCTACAGGTTTCGGCCACTTGGAGGTCTAGGACGGAGCGCGTTCGCCTACACATATCGCCTGGATAAGCTACAGCCGGGGTCGCGCGCACGCCTTTGCGGCGTCGACGGCGTCAGCTGCTGTAGTGACTAGCGTGGCGAATTGCGAACGGCTCACCGCGAGGAACAAAACAAAACGCAAACAAGGGGCGCCCGAGAGAGTTTAAAAACAAGCAGCTAGCGTTATCACATATCAAACGCGGCTGGCAGTGTGAGGTCGCCTTCTCGACCGGATGGTTAAATGTGTTAATCGCCTAAACCTGTCTAACTCGTCTTGTGGCAGTCAGTGCCGATTATTTCGCGGTCCTAACGCCATGATGAAACCCGCGATGGTGGCCAATCGGTTCGAATAGGAGTAATAAGCGATGGTGTTAATAGCCCCAATATGCAATAAATTGTAAGGCTCCTCACGAATGCACATCTATAGTGGCGCCCTACGGTGGCGTTGTGCGCGCTTTGTTCGAGCGCTTCAGTGCCGCTAACTGACCGAGATGTGCAGTCCTGCAACGCTTGTTGCAAACGAAGGAAAAAAAAAACTCGTGAGTAAATATAGAAATGTCAGTAATATACAAAACTTCACCCTTCTGTTAGGCAGAACTTGGTTAATGAAACGAATAAACCTGTTAGGCATGAATGTCGCGATTTCATCGTATAAAAAAGGGGGCGAAATGAACATTCCCTGTACGTACATTAGTTGTTTTTCTTTACAAAATGTTTTAAACCAAAACAGGAACAAGGAGTAAACGAAAGCGAACGCAATGTAAAGAACGGACTCCGATTCGAAATTTTTTGCTCTCAACAAAAAGAGGGAAGGTTAAGTGACTGTAAACCAAAAATATGCAAAAGAGACTCTCTCCAGAAATAAATAAATTAATAAATAAATTAATTGAAAATAGATTCTATGCGTCACCGCTAAGACGCAAGATACATTATATCTCGTTATTACTGCGCTGTAGTTAGAGAACGCGAAAGTGAGGAGCCAAACTTTTTTTGTTGCTGTTTTTGTTCTCTCTCTCTCTCGGTTTGAGCAGCTTGTACGCGCAGCTTTTCGAATGGAGCGCTATAAGCCGCAGCGAGCAAGTAGGCCAGGGCGCGACCTCTACTTTTTGGCCTTACGTTGCACGGTAAGCCGGGCAGAGGTCTCGGCTTGGCCCGAGGCGTTGGTGGCACGACAGACGTAGACGCCTTCGTCTTCGGGGTACGTGTGCCTGATGACCAGAGACGCTGAGCCTTGGTCTTGGTTCATCTGAAAGTCACGGGACTGCTTGATGATGTGGCCCTCGCGCACCCAGATGACCTTGGGCGGTGGCACGCCTCCCAGGGATGTGACGAAACGGGCGGGCGAGCCTTCGGGCACGGTCAGGTCGGCCAGAGGAGTGACCGTCGGCGGCACCTCGTTCAGTTCGGGCACAATCACTTTAAGGTTGGCGCTGGTAGAGACAGTGCCCGCCGGGTTTGTGGCGACGCACTTGTAGACACCTTCGTCCTCCGGGAACGCTTCGGAGATCTTGAGCGTGAAGAGGTTATTCTCTTGGGACATGCGGAAGTACCGCGACTGCTTCACTTGCTGGTCTCCTCGGAACCATTTTACTTGCGCCCCCGGGACGGCGGGTATTCGGCAGCGGAAGACGGCGCTCTGGCCTTCCTTGACGGCGAGTGGCTTGAGGGGCTCTGTCACCGTGGGTGGCTTCTGGTCTCCGGGCGCCTCCTTGGGGCTCGTGGGCGGCGTCTTGGGCTTGGCGCCTTCGATCACCACGTGAGCCTGGCAGCGGGCTTCTCCCGCCGAGTTGATGGCGACACACTCGTAGCTTCCGGAGTCGGAGTCCGCCTGGGCCTCCAAAATGAGCAGCGTGTACTGGTCGTCCTCCTCCACGATTTTGTGCGACACGTCGGGCGTCACTTTCTTGCCGTTCTTGAGCCAGTAGACATCCAACGGCTTCGATCCTGAGACCTTTGCGTCGAGGCGGACCAGCTTTCCGGCTTTCGACGAGACGTCCTGGATGGTCCGGGTGAAGTTTGGCGGCACGACACCGCTGCGTTGCTCTCGCCTCTCTTCAACAACCAAGTTGGCGGAGGACTTGGCTGAGCCGCCACGGTTTTCAGCCTTCACTGAGAAGACGCCGGAATCTTCCACATACACTTCGCGGATGATCAGCGTCGACGTGCTGTCAGTTGTCACGATCTGGAAATCCTGCGAGGGCTGGATGGGGAAGTTCTCGCGGTACCAGGTGATCTTGGGCGCTGGCTGTCCGGTGAAGGTGCACTGGAACTTGGCGCCTCGGCCTTCGTCCACGCGGCACGGCTGCAGCTTCTTGGTGAACACGGGAGCCTTCGCCGGCTCTTGCGCGGGCCCCTGGACGCGCTGCTCCTTGGTGACGCCCTTGTGCTCTTGCTCGAGCGTCTCGGTGAGCGTCTTCTTGCGGGTGATCTCGAGGTCGCCCTGCTGCTCCTTCTGAGTCTGCCGCTGGGTGGTGGTGTGAACCTCGCGGCCCTGCACCACGGACTCGGGCGAAATGCCGGTAGCGCCGCGCTGCTGCTTCTCGGCCTCGGTCTCGAAGTACTTGGCCATGTACTGCGCCTGGGAGCCCGTCTCCACCTTCTTCTCGCCCGTGCGCGCATCGTAATAAATCGAGTCGGTCTTGAAGCGCTCCACTTTCGGCTTGAGCTTCTCCAGCTCTTCTTCTTCGATCCTCTTCTTGATCTTTTGGTGTTCACCTTGCTGGCCCTGTTCAGTCTTCCAGACATCGATACGAGTGCCTCCAGGTGTAAGCTTCTCCTCAAACACTCGTTGCAGTTCAGTCTCATGTCGGTACTTTTGATAAGACTTGACGTCTTGGTCGTACCATGGCTTAGGAGAATGCTTGAGAACGGCTCTGTAGTCATCGAACTTGGGCCGGACTTCAAGAGTTGTGAAGCAGTAGGCTTCGCCGCAGAAGTTCCTTGCAAAAACTTCTACTTTTCCTTGGTCATACTGGCGGGTCTTAGGGATGTCAAGATGGTAGATACCGTCGTAAGTCAGCTTGTACCTTGAGCCGGCGATGACCGTGTGGCCGTTGAGAACCCAGATGAGCCTTGGCTTGGGGTGGCCAATAACTCGGCACTGGAACTTTGCCCATTCGCCCTCGATGACCACTTGAGGCTCTGGCTTCATAATGAACATGGGAGCGCATGGTTCCTCTTCCACTTCTTCCTGGGTCTCAGCGCGCTGCCAGGAGGCTTCCATTTCAGCGATCTTCTTAACACCATCCTTCATGTCCTTTGGAAGTTGGGTCCTGTAGTCCAGCTTTGGCTTCGCTGTGCACCTGAGGGTGGCCTGAGTTTGGTCCTCGCCAACGTCGTTGACGGCCCGTGCAACGTATGTGCCCGAGTCCTCAGGGTACACGTATAGCACGTCCAATGCAACGAAGCCGAAATCGTGCGTTGTCTTGAACCGGTGACCTGATCTCAAGGGTTGTCCGTTGTGGTACCATTCCACCCTCAGCTTTGGATCGTTGATGGGCTCCAGGCGGCATTCGAAGTGAGCCGAGTCGCCCTCATTAAGGTTTTGCTGAGGCTGTATCTGGGTGATGAACCTGGGCTTGACCGGTTCCTGCTCCTGAATTGTCTCTTCTTGTGGCGTCGGGTACTCCAGGTCGCGCAGCTTGTCAACGTTCATGCCCTCTGGCAGCTGGCTGTCCATGATGATGTCTTTCTTGGCTTTAATCTTTAGGGTTGCCTTGGTCGTGTCGGAGCCCCAACGGTTCGTGGCACGACACATATACTCTCCAGAGTCGCGGGGGAAGAGCCAGTCGATGTCCAGCACCACGAAGCCGAAGTCATCGATGGTGTGAACACGCGTACCCGTAACAAGGGGACGTCCGTTCAAGAACCATTCCACTTTCAGGTTGGGATCTCCGACTGGGATAAGCTTGCATTCGAAGTGAGCGCTGTCGCCTTCCACCTTATCGAGGAGCGATTGGATCTGCGTGACAAACTTCGGTGGTTCCTTCTTATCATCGTCATAGATCTTCTCTTCAGTTCGGTACATCGACTCTTCCAACTTCTGAATGCTTTCATACCCAGATCGGAATTCTTGAGGTAGCTGTGGTTCCATCACGACTGACTGCTTGCCCTTTACGGCGAGCTTGCAGGAGACCGACGCTTCGCCAACCTTGTTGACGGCTCTGCATGTGTAAACACCCGAATCTCGTGGGTAAACACCGGCCACCTCCAGAATGACGTATCCAAAGTCGCTGATTGTCTTGACTCGAGAGCCCGTGACAAGTGGCTTGCCATTGTGATACCAGTCAACTCTCAGCGTAGGGTCACCGACTGGCGTCAACCTGCACTCGAAATGAGCCAGAGAGTTCTCTGCCAGCGTCAGGTCCTGCGGCGTGGTGATGAACTTGGGCGGCTGAGAGGTGTCCTTGTCCGTCCACTTCTCGTCGATCCTAGCCGAGGAGACTTCTTCGAACTTGGCAATCTTCTCGATGCCTGTGGTCATTGACTCTGGCAGCTGTGTCTCGAGAATGATGCTACGCTTTCCTTGACACTTGAGTGTGCATGTGGTGACGGCCTCGCCGAAACGGTTTCTAGCACGGCAGGAGTAGACGCCGGAGTCCTCCGGGTACACAGGGCTCATTTCGAGCACCACGAAGCCGAAGTCGTTGATGGTTCGGATTCTTGTTCCTGACATCAGCGGCATGTTGTTCTTGAACCATTCCACGNNNNNNNNNGGATCATCTGTAGGCGTCAGCCGGGCTTCCAGATGTGCGTTGTCTCCCTCCCTCAGATCCTCAACGTTAGTGAGCGGTACGGTGAAACGAGGCGCCTCAGCGGGTCCCTCGGGCTCAATGACCGCCGCTGTGCGGTACAGGCTTTCTTCGAGAGCGATGATGTTCTGTGTCGCCGAGGCCATTTCAGGGGGCACTTGAGGAGTCAGGATCAAGGAGCTCTTGGACTTGCACTGTAGTGTCGCGCGGGTCACATCTGAGCCGAGCTTGTTCGTGGCCTTGCAGACCCATTCGCCGGAGTCCTCTCCGTAGCAGTACAGGATGTCCAGGATAACGATGCCGAAATCGTGGAACGTCCGGAAACGATGGCCTGAGCGCAGCAGCTGGCCATTGAAGTACCACTCAACCGTGAGGTCTGGGTCGTTGACCGGCACCAGCTGGCACTCGAAGTGTGCGCTCTGGCCTTCGACCAGGTTTGTGATGTTGTTCAGCTGTGACAGGAACTTGGGCGGCTGGAGCCGGGTCGCCTCCATCACGGCCGATGTCCTGGTGAGAGCCGAAGCACCTTCCAGTTCAGCGATCCTGTCAAGGGATTGCGGCTGTAGCGAGTCCCTGAATATCTTGCCAGTGCCTGCGCACTGAATGGTGCACTTAGTGGCGTCGGAGCCGTATTTGTTCGTGGCCACGCACACGTAGTCTCCAGAGTCTTCGGCGTGCACGAATGAGATGTCCATCACCACGTAGCCGAAGTCGCTGAGTGTCTTGATGCGAGAACTGTCGCGAAGTGGCTGCGAGTTTTTGTACCACTCGACCTTCATGTCTGNNNCGCCAACAGGCACCAGGTGGCATTCGAGGTGAGCGCTGTTTCCTTCCTTGAGGTCCATGAGGTTCTGCAGCTGCGTGGTGAATTGCGGCCGCTGTCCCTTGGTCTTGTCGGTGCACTCGAGGTCGACGGTGACCTCGGCCTTGCCCCACTTGTTGGTCGCACGGCAAGTGTAGCGGCCAGAGTCCTCCAGCACAGTCCCCAAGATCTCGAGCACCACCATGCCGAACGCGTGGACCGTCCGGATGCGGTGCCCTGCTTTGAGGGGTTTGCCGCGGAAGAACCATTCCACCTGCATGGTCTGGTCGCCGACTGGCGTCAGCGTGGCCTCGAAGTGGGCGATCTCGCCCTCGTTCAGGTCCTTGAGGTTGACGAACTGAGATGTGAAGACTGGCGGCTGGCCAGCGGCTTCCTCCTGGATCGCTTCGGGCACGCGCGTCAGCGACTCCTCCAGGTGCTGGATGGACTCGAGGCCCTTGCGGCCCTCCGGGTGCAGCGTGTC

>MG4814399

CCAAGCTGAAGGTCGAGTGGTTCAAGAACGGCCAGCCGGTGCCAGCAGGATCTCGTTTCGTCGAGATGTGCAACTTCGGTTTCGTGTCTCTGGACATCCTCAACACCTACGCTGAAGACTCCGGCACCTACACCTGCAAAGCCACGAACCAGCTGGGAGAAGCTGTCGTGTCTGCTCAACTCAAGTGCCACGCCGAAAAGTCGTTGATACTGGACACGCAAAACCAGGAAGCGTACGAAAAGATCCAGCAACTCGAGGACTACGGACGCCAAGCAAGGCCCGCCTATGTCGTGGAAGAGATGACCACGCAAGCGCCCGTGTTTACTCAAGCTATGAAGAACCTGAGCCTCAACGAAAACCAGAGTGCTCACTTTGAGTGTAAGCTCATACCAGTGGGCGACCCCAACCTCAAAGTGGAATGGTTCCACAACGGACTCCCCATACAGAAAGCCAACCGGGTCAACACCATTCATGACTTCGGATTCGTCGCTCTTGACCTGAGCTACGTCAAAGCACAGGACTCGGGCACTTACACTTGCAAGGCAACCAACTCCCTGGGATCTGCCGTTTGTTCTGCCACTCTCAACGTCCAAGATTCCAAGTCGCTGGTGTTCGACACTCAACACCCAGAAGGACTTCAGAAGATTCAACAGCTCGAGGAACTAGGACGTTACAAGCCAGAAGTGACGCAGGAAGCCCCGTGCCCGGGCCCACCGATGTTCGTCACCCAGCTCCAGGGCCCGAGCCGTATG

>MG4822957

TTCCTGGATGGCTCTGATACTGTCCTCCTGGATTGCTTTGCGTTCGATTGCCGGCCGAGCTGTGCATCGAAGTGTGGCCGTGGTCGTCGCCTCTCCGGCTTCGTTGGCTGCACGACAGACGTATACGCCAGAGTCCTCAGGATAGACGTGCAGCAGCGTCAGCGCCACGTAGCCGAAGCGGTACGTGGTCATAACTCGTGAGCTCGCTTCGATGGGGCGTCCGTTACAGAACCATTCGATGGTCATGGTTTCGTCTCCGATGGGAATCAGCCTAGCGTCGAGGTGGCCACGCTCGCCCTCGTGCAGGACCAGGTCCTGAAGGTGCGTCGTGAAGGCCGGCTTCTGGGGCGCCCTCTCGTCATCTTGGCCACGCACCGGCTGGTAGGCGCGCTCCACATGTCGCAGCCAAATGGTCTCACCGGGCGGCGACTTTTCGGGTGAAATAACCTTGGTCGGCTTAGGCAGATGAAGCGCCCACGGCTCGGTGTGCTTCTTGGGTTCAGCTATCACGTGCAGACGCGCCCTCGTCGCTGTCGAACCAGCTTGGTTTTGCGCGGTGCACTGGTACCACGCGGAGTCGGCCACGCTAGCACGGTTCAGGTACAGGGCGGAGCTCCCGTCTTGCGTCTCAATGATGAGGTTGGGTGGCTGCGAGTGGACCTGGCGGCCGTCCTTCTGCCAGGTGATGCGCGGTGCCGGCGTGGCCACGGCGCGGCAGTGGAGGGTCACGGACTCGCCCTCNNNNNNNNNNNNNNNNNNNNNNNNNNNNNNGAACTTGGGTGCCACCACCTGCTCTTTCTCAATCACCACGAGGTGCACCTCGAAGCGGACCTCGCCGCTCTTGTTGTTGGCGATGCACGTCCACGTGCCG

>MG4823707

CCCTCCGGGTGCAGCGTGTCCTCCTGCACGCCCTTGCGGGTCAGGCACGTGATTGTGGTTGTGGTAAAGGCTTCGCCTGCGGCGTTGCTGGCGCGGCAGGTGTAAACGCCGGCGTCTCGTCCCCAGAAGTCGGTCATGGCCAGCACAACGAAGCCGAAGTCGTGTGTGACGGTGTGCCGGGATCCTGGCGCAAGTGGTTTGCCGTTCAGGAACCACTCGACCCTGAGCTTCGGGTCGCTTGCTGGTTCCACCTTGCACTCGAGGGTGACAGGGCTACCCTCTTGGATGGAGAAGTTTGGCTCAAGGGGCACCACGAACACCGGGCGCGGGAACACCTTCTCAGCCTCTTCGACGGGAGCCTGGTATCTGGTCAAGTAGGAAGTCTCTAGCTCCTGGACCTTGGACAGTCCCTGTGCGCCCATTGGGTGAAGAGTGTCGGACAGCACGCCGCTTTTTCCTTGGACTTTGAGAGAGCCGGTAGTGACCGCTTCGCCCTTCGCGTTGCGGGCTCGGCACGTGTAGATGCCGGCGTCCTCCGGGTATGCGTTGCTGACATCCAGTGTGACGAAGCCAAAGTCGTTGTTGAAGTTGTACTTTGAACCAGCAGGCACTGGCTTGTTGTTCTTGAGGAATTCCACCTTGAGCGTGGGATCCTTGGATGGCTCCACTCGGCACTCGAAGTGCGCAGTCGCACCCTCGCGGATCTCCAGGTTGTTGAGGTGCGTGACGAACACGGGCCCTTCGTAATCTGGTTCAGGCACGTACTTCTCAGGCGGCACATACTCTTCCAGCTGGCGGATCTTGGGCAACGAGTCGGGGTGCTGGGAAGCCAGGATGATGTTCTCGCGGCCTTCAACCTTGATTGTGCACGTCGATATAGCTTCGCCAACATCGTTGGTGGCCTTACAGGTGTAAATTCCAGAATCGTCCGGCCTAGCGCTGGAAATGTCCAACGAAACCAAGCCGAAATCGTCCGTAGGCTTGATACGAGTACCCATGACCAGAGGCTTGCCGTTCTTGAACCATTTGACTTTGAGCTTGTCGTCGTTGGTAGGTTCAATGCGCCCTTCGATGCGCAGGTAGTGGCCTTCAACAGCGCCATCGATGTTGTTCAAGTGCTGCACAAACACTGGCGCCGCCTTGGGCTTCTCGTCGGACCACTTCTCAGGAATGCGGCTGGAGTCGTACTCGAACTTCTGCGTCTGCCTGTACGCCTCGGGATGGTGGGAGTCCAGCAGCACACCAGCTCGGCCTTTCACTCGAAGTGAAGTTGAAGTGACGGCTTCTCCTGCCTTGTTCACCGCTTTGCACATGTAGACTCCAGAGTCTTCCGCGATGCCCGACAGGATGTCCAGGGTCACAAATCCAAAGTCGTTGTTGGGAATCAACCTCGAACCTTTTGGAAGCTCGATGCCGTTGACGTACCAGTAGAACTTGAGGTCAGGGTCTCCGACAGGGACACAGCGGCACTCCAGACGTGCAGGCTGTCCCTCGTTTATCTCGCCAGGACCAACCAAGTGGCTGGTGAACACTGGCTTTTCGTACACTGCGTCTGGTTTCTCCGGAGGCTTCTGGCCCTTCAAGTCTTCCAGCTCACGGATCTTCTCGTAGCCTTGAGGTTGCAGGGTGTCCAGAAGAATGGACGCCTTGGGTTTAACCTTGAGCATGCAGGTGGTCACTGCCTGGCCCAGGGAATTGGTGGCCTTGCACGTGTAGACGCCAGTGTCCTCCGGGCGCACGTAGTCCATGTCGAGTGCAACGTAGCCAAAGTCGTGAACTGGATGGAACCTCGTGCCTTCCATCAGTGGCGTGTCGTTGAAGAACCACTGGACATTGAGGGTAGGATCTCCGACTGGGATCAGCCTGCACTCCAGGTGGACGTTTCTCTGGTCTTCAACGAGGTTCTCCAGGTTCTGCAGAGGAACCGTGAACACGGGCGTCTGGTAGGTGACTGGCTCCTGGATGACTTCACGCTTGAAACGAGACTCGTCTTCCATCTCCCTGATCTTAGCCAGTCCTTCTGGGTGCTGGGTGTCGG

>MG4834077

GCTGGTTCCACGCGACACTCCAAGTGGGCGCTTTCACCCTCCGAGATCTCCTGGGTTCCCCTCAGTTCAGTGATGAACACAGGCTTTGAGATCGGCTTCTCCTGAATTTCGGCAGACTGGTACTTCACTTGCGCTTCGAGCTCCCGGATTTTCTCAAGACCCTCCGGATGATGCGTATCATAGAAGATCTTTTGCTTGGGTAGGGCACGCAGGTTACACGTTGTAACCGCTTCTCCGAGAGAGTTGGTTGCCTTGCACATGTAGGTTCCGCTATCTTCGGCATACACATACATAATGTCGAGGGCCACATATCCAAAGTCGTGCGTCGTCCTGAACCGATGTCCTGGCCGGATTTCCACGCCGTTGACGTACCACTGAACTTTGAGNNNNNNNNNNNNNNNNGGCTCGAGTCTGCACTCCAAGTGAGCGCTCTGTCCTTCCACAAGACCGTCCAGCGAGTTCAGAGGCACCGTGAACACGGGCCTTTGCAGCTGGATCACCTGTTCAGGCTTCTCCGGCGCCTGGTACTCTTCAAGTTGCCTGATTTTCTCCAGGCCCTTTTCGTGGTAAGTGTCGGTTACAATGGCGGACTTTCCGAGAACGTTGATTGAGCATGTGGTAACGGCTTCGCCGAGCTGATTGGTTGCCTTGCACATGTATGTGCCAGAATCCTCCGGGTAGGCGTAGAGGATGTTGAGAGCCACGTAGCCAAAGTCAGAAACAGTCTTGAACCTGTGGCCAAGCGGAATTTCTACGCCGTTGTGGAACCACTGAATCTTCATGTTAGCGTCGTTGATTGGTGTCAGCGTAGCCTCAAGGTGAGCGCTTTGGCCTTCATGCAGAGTTTCCAGGTTGTAAAGACCCCGAACAAAGTTAGGCTTGTCCTTGATCACCACTTCTTCCTGCGTTGGCCTGATGTAGCCGCATGATTCCTCGAGCTGCTTGATCTTCGGGAGGGCGTCTGGCTGCTGGCTTTCGAGGATGACGCCACCCTTGGCATCAACATGAATGCGCGCCGTGGACCTCGCGGTGCCAAGCTCGTTCGTGGCCTGGACCACGTACTCGCCGGAGTGCTCAGCATTCGCTGACAGAATGTCCAAGGCGCAGAAGCCGAAGTCGTAC

>MG4836507

CTCGGGCACATACACCGCCGTGGCCAAAAACAAAGCCGGAGAGACGGCCTGCTCCTGCCAAGTGAAAGTGGCCGAGGACGCCGCTCCGCCCGAGCCTCCGCGTGTGCTCAAGGCCCTGGAGGATCTGGAAGTAAAGCCTGGACCGGACCCTATCACGCTGGAATGCATCATCGTCGGCCGGCCCGAACCAGAGGTCATCTGGTACCACAACACCCAACCCATCAAGGAGTCTGAGCGGGTACGGCTGCTGTTCCGCGGGGACAAGTGCTCTCTTGTCCTGAACGGCGTCAGCGCCCAGAACGCCGGCACCTACCGATGCTCGGCGGTCAACCCCATGGGCAGCTGCTACACGGAGTGCAACATGCGCGTGCCGCTTTCTGCTCCCGTGTTTCTGGAGCCGCTCCGAGACGTGACCACGGATGAGGGCTGCCGCGTGGTGCTCACTGCAAAGCTATGGGCACCCGAGCCACCTTTCGTCCGCTGGTTTAAGGATGGAAGAGAGGTGCTTCCAAGCCCAGACTTCCAGGTCAGCCACGATCCGGACGGAACTGTGAAGCTACTGATCCCGAAGGCGGCGGCCAGCAACAGCGGCCATTACGAAGTCGAGGCCAGCAACCCCGGCGGTCGCACGCGCACGGGCTGCAAGGTGCACGTTCGTGAAGCTCAGAAGACGGTCCAGGCGTCCAGCCAGCTTGCG

>MG4837467

CAACGACGCTGGAGAGGCTGCTTCAGACGCCGATGTTAAGACCAAACCCGAAGCGCGCGTCCCCGGCGAAAAGCCTGAATTTGTCATCGAGCTCAAGAACGCGGATCTTCTAGAGGGGCAACCCCTGCATCTCGTGGGAAAAGTCAAGAGCGACAGCCCTTTCACGACAGAATGGCTCAAAGACGGCAAAAAAAGTGGAACCTTCTCTAAGACTGGCGCTGTCTCAAGNNNNAGACGGCACAGTTCTTTTGTCGATAGAGCACGCAACGCCAGACGACGCCGGGAAGTACGTCTGCGTCGCCAGGAACCCCGAGGGCAAGGCAGAAAGTTCGAGCACGGTGAAAGTGGCAGAGCTCCCGAAATATGAGCCTGAAATTGTAGAGGAGCTTAAGCCGGCAGTCTTCACTGAAGGTGAACCTGGAAAATTGGAGGCCAAGGTATCTGGGGACCCGATGCCAGATGTCAAATGGATCAAAGATGGCAAAGAGCTTCCCGAAGACAGCCGCATTCGTTCGACCACTTCACCTGGCGGAGACGTGGCGCTCACGATTGATCCTGTGAAGCCCGAAGACGCCGGAAAGTACGACCTTGTTGCAGCCAATGACGAGGGCGAATGCAGAACCTCTGCTCCCGTTACAGTGAACCATCCGCCGAAATTCGTGAAGCCGCTCGAGCCTGTTGAAGTTGTGGAAGGCTACCCGGCCAGGCTTGAGACGACACTCGCTGGTC

>MG4845526

GCTGCCAAAACCCATCCACTCTGGAGGTCACTGCTGTAGTACTCAATCGTGTAGCCCTTCAGTGATGACGCGCCCACTTTTTCACTGCGTCTCCAAGTCAGGGTTATCGATGTTTCGGTGGTGTTCACTACCACAGGCTGCGATGGAGGTCCAGGAAACGTGGATGGGTCAGGAGAACGGTGAAAATTGACGTTAGGGTTGTGTGGTGACTCGACAGTGAGCGACGCCGTCCAAGATGTCTCGCCACTCTCACTTGACGCTTTACAGGTGTATGCGCCACTGTCCATCATTTGGAGATCTTGTATCTGAAGTGTGCCGGACTCGAGCAGCGTGAACCGGGGCCGG

>MG4816344

GGCGCTGCAGCTGACCTGCTCGGCCACGTTGCCTTCGTAGACAAGGAGGTGTCCGTGTCACACCTCCTTGTCTACGAAGGCAACGTGGCCGAGCAGGTCAGCTGCAGCGCCAACTCCAAGCCGTCCAGCGAATACATGTGGATGTACAACGACCAAGTCATCTCCGACAGCCCACTGCTGTTCATGAACTACAGCCTGGGCAGGGAGCGCACTGGAAACTATACGTGCGTCGCCAGCAACAGGCACGGCTCGGCATCGGTCAACACCTATATTGACGTCATCTACCCACCGTCGTGCGTGCTGTACACGAGCAAGAACTCGGAGGGCCAGGTGACGCTCATATGCGAGGTGGACGCCAACCCGCCCCAGGTGAACATCAGCTGGACGCTGGGCAACGAGACGCTCGAGCGGAACGTCTACTCGGAAGGTCTGCGCAGCATCTACACAGTGCCGGACTCGACGGCGCCCGAGTACTACGGTCTCTACCTCTGCCAGCCGAACAACTCTCTGGGAGCCGCCGAATGCGAATACCGTNNNNNNNNNCCCGGCGATGCCTACAATCATGTCTACCTAGACGATGAGCAAGTCATGATGATTACGGGCATCGCGGGACTCATCGTTTTAATTTTTATCATTATCATAG

>MG487314

GGGCGCTCCGCTGCGAGGCGGTGGGGGCGCCTGCCCCACGCCTGCAGCTGCTCAAGGACGGGGAGCCCCTGACCAACTGGACCGCACAGCGCCTGGTGCACGTGCTGAGCAGTATTAGCCACTCCCAAGCAGGGTCCTACCAGTGCCTGGTCGAGAATGCAGCTGGCGCGCTGCTCAGCGCCAAGGCCCGGCTACGGGTGGCACACCTAACACGAGCCGAAGAAGTCCCTGAAACAGTTCCTGTGAACGCTCGCAAGGGTGGCGACGTGATCTTGGCACCGCCGCTTGTTGACAGTGTTCCGCCAGCGACTGCGGTCTGGACACGGCTGGACGGCAGAAACTTGGACAGCAGAAACTTTGCACAAACACAGGATAACCGCCTGGTCATTCTTGACGTCAGCCCCAAGGATGCCGGCCAGTACCGGGTGGAACTGACCAATCCGCACACGGGAGACAACCTCTCGGGACCAGTGGTGGAGCTGACCGTCGATGACAATGAAGAAGACCAGGCAGAACTCTCCATTGTTGTTCCGCCATCAGATCGAGAGTTCAACAACCTTGGCAATGGCTATGACAGCACTCTTGAGTGCATTGCCTCTGGCAGGCCTCTGGACCAGGTGCAGATTGAGTGGCTGAAGGATGGTAGGGCCCTGGGTGAGCTGCCACACGTTCTCACCCACTGGAACCGCACCCTGACCCTGCTGCGCCTGGGGCCCGGGCACACTGGCCGCTACTCCTGCCAGGTCACCCTTCGACATGCCCCTGAGGACTCCTCTGCTGTCGTGGCCCATGCCAACGTCACTGTGTCAGTGCTGCCCTCCTTGAGCCAGAAGGTGAATGAGGAGACGGCAGTGGAACTGGGGCAGCAGGTGCGGCTTCCCTGCGTGGCTGAGGGCCACCCAGAGCCCCAGGTGCACTGGCTCCTGGATGCGCGGCCTGTGGACCAGAGCACAGGGCACTTCCACGTTGGTGACGGCGGCAGCCTCGAGATTGGAGCCCTGGCTATGGAAGATGCCGGGGTGTACCAGTGTGTGGCGGAGAACCCGCTGGGTGAAGCTCGCGCTTCTACCTGGCTTCATGTAAAAAGAACTTGCCATTTGCCATCTAC

>MG9637567

CGCCTGCCTGACAAGGTGTAAGCTGAAACCAACACGTGGACTGTTCTGTTGAACACTCGGTTGACGGAGGGATCGTGGTGACGGGCAACACATGTGTACCAGCCGGAATCAGGCTCTTCCAGGTGATAGATGGTCATNGTCCTTTCGTTGAAGTGTACCTCCTCGATCTTGATTCGGTGCGCTTGGCGCACCTTGCCTTGAGTGATCACCCATGTTATGCGGTACCTTTCTTTAGAAGCGACTCTACAGGTGAGGTTGAGGTCATCCTCGACTCGCCGCGTTACTTTGCCAGACGGGACGGTTATGACAGGCTCTTTATGTTCATACTCGGAGGACCCGTGTACTGCACCAAAAAGAACCACAACTGCAAGCAATTGGGGAAAAGTAGGGAGGATATCCATTTATATAAACGCATTANNNNNNNNNNNNNNGGGACGCTCGGATAGGCGACTACTCGAATTCCGAGCGCGTAAAAGAACCTGCGTTCCGCTCTTTGGCATCCGGCAAAACGGCGTAAAGATGTTAATCTTC

>MG963474

GCCAGCTGCTGCGCTCAGGCCATCGTTTCCGGACGTTCCACGATTACGGCATCGTTATCCTGGACATCCTGTACTGCTACGGAGAGGACTCCGGCGAATGGGTCTGCAAGGCCACGAATAAGCTCGGCTCAGATGTGACCCGCGCGACACTACAGTGCAAGTCCAAGAGCTCCTTGATCCTGACTCCTCAAGTGCCCCCTGAAATGGCCTCGGCGACACAGAACATCATCGCTCTCGAAGAAAGCCTGTACCGCACAGCGGCGGTCATTGAGCCCGAGGGACCCGCTGAGGCGCCTCGTTTCACCGTACCGCTCACTAACGTTGAGGATCTGAGGGAGGGAGACAACGCACATCTGGAAGCCCGGCTGACGCCTACAGATGATCCAGACCTGACTGTGGAATGGTTCAAGAACAACATGCCGCTGATGTCAGGAACAAGAATCCGAACCATCAACGACTTCGGCTTCGTGGTGCTCGAAATGAGCCCTGTGTACCCGGAGGACTCCGGCGTCTACTCCTGCCGTGCTAGAAACCGTTTCGGCGAGGCCGTCACCACATGCACACTCAAGTGTCAAGGAAAGCGTAGCATCATTCTCGAGACACAGCTGCCAGAGTCAATGACCACAGGCATCGAGAAGATTGCCAAGTTCGAAGAAGTCTCCTCGGCTAGGATCGACGAGAAGTGGACGGACAAGGACACCTCTCAGCCGCCCAAGTCTGCCGGCACGCCGCAGGACCTGACGCTGGCAGAGAACTCTCTGGCTCATTTCGAGTGCAGGTTGACGCCAGTCGGTGACCCTACGCTGAGAGTTGACTGGTATCACAATGGCAAGCCACTTGTTACGGGCTCTCGAGTCAAGACAATCAGCGACTTTGGATACGTCATTCTGGAGGTGGCCGGTGTTTACCCACGAGATTCGGGTGTTTACACATGCAGAGCCGTCAACAAGGTTGGCGAAGCGTCGGTCTCCTGCAAGCTCGCCGTAAAGGGCAAGCAGTCAGTCGTGATGGAACCACAGCTACCTCAAGAATTCCGATCTGGGTATGAAAGCATTCAGAAGTTGGAAGAGTCGATGTACCGAACTGAAGAGAAGATCTATGACGATGATAAGAAGGAACCACCGAAGTTTGTCACGCAGATCCAATCGCTCCTCGATAAGGTGGAAGGCGACAGCGCTCACTTCGAATGCAAGCTTATCCCAGTCGGAGATCCCAACCTGAAAGTGGAATGGTTCTTGAACGGACGTCCCCTTGTTACGGGTACGCGTGTTCACACCATCGATGACTTCGGCTTCGTGGTGCTGGACATCGACTGGCTCTTCCCCCGCGACTCTGGAGAGTATATGTGTCGTGCCACGAACCGTTGGGGCTCCGACACGACCAAGGCAACCCTAAAGATTAAAGCCAAGAAAGACATCATCATGGACAGCCAGCTGCCAGAGGGCATGAACGTTGACAAGCTGCGCGACCTGGAGTACCCGACGCCACAAGAAGAGACAATTCAGGAGCAGGAACCGGTCAAGCCCAGGTTCATCACCCAGATACAGCCTCAGCAAAACCTTAATGAGGGCGACTCGGCTCACTTCGAATGCCGCCTGGAGCCCATCAACGATCCAAAGCTGAGGGTGGAATGGTACCACAACGGACAACCCTTGAGATCAGGTCACCGGTTCAAGACAACGCACGATTTCGGCTTCGTTGCATTGGACGTGCTATACGTGTACCCTGAGGACTCGGGCACATACGTTGCACGGGCCGTCAACGACGTTGGCGAGGACCAAACTCAGGCCACCCTCAGGTGCACAGCGAAGCCAAAGCTGGACTACAGGACCCAACTTCCAAAGGACATGAAGGATGGTGTTAAGAAGATCGCTGAAATGGAAGCCTCCTGGCAGCGCGCTGAGACCCAGGAAGAAGTGGAAGAGGAACCATGCGCTCCCATGTTCATTATGAAGCCAGAGCCTCAAGTGGTCATCGAGGGCGAATGGGCAAAGTTCCAGTGCCGAGTTATTGGCCACCCCAAGCCAAGGCTCATCTGGGTTCTCAACGGCCACACGGTCATCGCCGGCTCAAGGTACAAGCTGACTTACGACGGTATCTACCATCTTGACATCCCTAAGACCCGCCAGTATGACCAAGGAAAAGTAGAAGTTTTTGCAAGGAACTTCTGCGGCGAAGCCTACTGCTTCACAACTCTTGAAGTCCGGCCCAAGTTCGATGACTACAGAGCCGTTCTCAAGCATTCTCCTAAGCCATGGTACGACCAAGACGTCAAGTCTTATCAAAAGTACCGACATGAGACTGAACTGCAACGAGTGTTTGAGGAGAAGCTTACACCTGGAGGCACTCGTATCGATGTCTGGAAGACTGAACAGGGCCAGCAAGGTGAACACCAAAAGATCAAGAAGAGGATCGAAGAAGAAGAGCTGGAGAAGCTCAAGCCGAAAGTGGAGCGCTTCAAGACCGACTCGATTTATTACGATGCGCGCACGGGCGAGAAGAAGGTGGAGACGGGCTCCCAGGCGCAGTACATGGCCAAGTACTTCGAGACCGAGGCCGAGAAGCAGCAGCGCGGCGCTACCGGCATTTCGCCCGAGTCCGTGGTGCAGGGCCGCGAGGTTCACACCACCACCCAGCGGCAGACTCAGAAGGAGCAGCAGGGCGACCTCGAGATCACCCGCAAGAAGACGCTCACCGAGACGCTCGAGCAAGAGCACAAGGGCGTCACCAAGGAGCAGCGCGTCCAGGGGCCCGCGCAAGAGCCGGCGAAGGCTCCCGTGTTCACCAAGAAGCTGCAGCCGTGCCGCGTGGACGAAGGCCGAGGCGCCAAGTTCCAGTGCACCTTCACCGGACAGCCAGCGCCCAAGATCACCTGGTACCGCGAGAACTTCCCCATCCAGCCCTCGCAGGATTTCCAGATCGTGACAACTGACAGCACGTCGACGCTGATCATCCGCGAAGTGTATGTGGAAGATTCCGGCGTCTTCTCAGTGAAGGCTGAAAACCGTGGCGGCTCAGCCAAGTCCTCCGCCAACTTGGTTGTTGAAGAGAGGCGAGAGCAACGCAGCGGTGTCGTGCCGCCAAACTTCACCCGGACCATCCAGGACGTCTCGTCGAAAGCCGGAAAGCTGGTCCGCCTCGACGCAAAGGTCTCAGGATCGAAGCCGTTGGATGTCTACTGGCTCAAGAACGGCAAGAAAGTGACGCCCGACGTGTCGCACAAAATCGTGGAGGAGGACGACCAGTACACGCTGCTCATTTTGGAGGCCCAGGCGGACTCCGACTCCGGAAGCTACGAGTGTGTCGCCATCAACTCGGCGGGAGAAGCCCGCTGCCAGGCTCACGTGGTGATCGAAGGCGCCAAGCCCAAGACGCCGCCCACGAGCCCCAAGGAGGCGCCCGGAGACCAGAAGCCACCCACGGTGACAGAGCCCCTCAAGCCACTCGCCGTCAAGGAAGGCCAGAGCGCCGTCTTCCGCTGCCGAATACCCGCCGTCCCGGGGGCGCAAGTAAAATGGTTCCGAGGAGACCAGCAAGTGAAGCAGTCGCGGTACTTCCGCATGTCCCAAGAGAATAACCTCTTCACGCTCAAGATCTCCGAAGCGTTCCCGGAGGACGAAGGTGTCTACAAGTGCGTCGCCACAAACCCGGCGGGCACTGTCTCTACCAGCGCCAACCTTAAAGTGATTGTGCCCGAACTGAACGAGGTGCCGCCGACGGTCACTCCTCTGGCCGACCTGACCGTGCCCGAAGGCTCGCCCGCCCGTTTCGTCACATCCCTGGGAGGCGTGCCACCGCCCAAGGTCATCTGGGTGCGCGAGGGCCACATCATCAAGCAGTCCCGTGACTTTCAGATGAACCAAGACCAAGGCTCAGCGTCTCTGGTCATCAGGCACACGTACCCCGAAGACGAAGGCGTCTACGTCTGTCGTGCCACCAACGCCTCGGGCCAAGCCGAGACCTCTGCCCGGCTTACCGTGCAACGTAAGGCCAAAAAGTAGAGGTCGCGCCCTGGCCTACTTGCTCGCTGCGGCTTATAGCGCTCCATTCGAAAAGCTGCGCGTACAAGCTGCTCAAACCGAGAGAGAGAGAGAACAAAAACAGCAACAAAAAAAGTTTGGCTCCTCACTTTCGCGTTCTCTAACTACAGCGCAGTAATAACGAGATATAATGTATCTTGCGTCTTAGCGGTGACGCATAGAATCTATTTTCAATTAATTTATTTATTAATTTATTTATTTCTGGAGAGAGTCTCTTTTGCATATTTTTGGTTTACAGTCACTTAACCTTCCCTCTTTTTGTTGAGAGCAAAAAATTTCGAATCGGAGTCCGTTCTTTACATTGCGTTCGCTTTCGTTTACTCCTTGTTCCTGTTTTGGTTTAAAACATTTTGTAAAGAAAAACAACTAATGTACGTACAGGGAATGTTCATTTCGCCCCCTTTTTTATACGATGAAATCGCGACATTCATGCCTAACAGGTTTATTCGTTTCATTAACCAAGTTCTGCCTAACAGAAGGGTGAAGTTTTGTATATTACTGACATTTCTATATTTACTCACGAGTTTTTTTTTTCCTTCGTTTGCAACAAGCGTTGCAGGACTGCACATCTCGGTCAGTTAGCGGCACTGAAGCGCTCGAACAAAGCGCGCACAACGCCACCGTAGGGCGCCACTATAGATGTGCATTCGTGAGGAGCCTTACAATTTATTGCATATTGGGGCTATTAACACCATCGCTTATTACTCCTATTCGAACCGATTGGCCACCATCGCGGGTTTCATCATGGCGTTAGGACCGCGAAATAATCGGCACTGACTGCCACAAGACGAGTTAGACAGGTTTAGGCGATTAACACATTTAACCATCCGGTCGAGAAGGCGACCTCACACTGCCAGCCGCGTTTGATATGTGATAACGCTAGCTGCTTGTTTTTAAACTCTCTCGGGCGCCCCTTGTTTGCGTTTTGTTTTGTTCCTCGCGGTGAGCCGTTCGCAATTCGCCACGCTAGTCACTACAGCAGCTGACGCCGTCGACGCCGCAAAGGCGTGCGCGCGACCCCGGCTGTAGCTTATCCAGGCGATAT

>MG9631466

CTCCAGGCCCTTTTCGTGGTAGGTGTCGGTTACAATGGCGGACTTTCCGAGAACGTTGATTGAGCATGTGGTAACGGCTTCGCCGAGCTGATTGGTTGCCTTGCACATGTATGTGCCAGAATCCTCCGGGTAGGCGTAGAGGATGTTGAGAGCCACGTAGCCAAAGTCAGAAACAGTCTTGAACCTGTGGCCAAGCGGAATTTCTACGCCGTTGTGGAACCACTGAATCTTCATGTTAGCGTCGTTGATTGGTGTCAGCGTAGCCTCAAGGTGAGCACTTTGGCCTTCATGCAGAGTTTCCAGGTTGTAAAGACCCCGAACAAAGTTAGGCTTGTCCTTGATCACCACTTCTTCCTGCGTTGGCCTGATGTAGCCGCATGATTCCTCGAGCTGCTTGATCTTCGGGAGGGCGTCTGGCTGCTGGCTTTCGAGGATGACGCCACCCTTGGCATCAACATGAATGCGCGCCGTGGACCTCGCGGTGCCAAGCTCGTTCGTGG

>MG9639977

ATGTTGCACTCCGTGTAGCAGCTGCCCATGGGGTTGACCGCCGAGCATCGGTAGGTGCCGGCGTTCTGGGCGCTGACGCCGTTCAGGACAAGAGAGCACTTGTCCCCGCGGAACAGCAGCCGTACCCGCTCAGACTCCTTGATGGGTTGGGTGTTGTGGTACCAGATGACCTCTGGTTCGGGCCGGCCGACGATGATGCATTCCAGCGTGATAGGGTCCGGTCCAGGCTTTACTTCCAGATCCTCCAGGGCCTTGAGCACACGCGGAGGCTCGGGCGGAGCGGCGTCCTCGGCCACTTTCACTTGGCAGGAGCAGGCCGTCTCTCCGGCTTTGTTTTTG

>MG961893

GGCGGGCGCTCCGCTGCGAGGCGGTGGGGGCGCCTGCCCCACGCCTGCAGCTGCTCAAGGACGGGGAGCCCCTGACCAACTGGACCGCACAGCGCCTGGTGCACGTGCTGAGCAGTATTAGCCACTCCCAAGCAGGGTCCTACCAGTGCCTGGCCGAGAATGCAGCTGGCGCGCTGCTCAGCGCCAAGGCCCGGCTACGGGTGGCACACCTAACACGAGCCGAAGAAGTCCCTGAAACAGTTCCTGTGAACGCTCGCAAGGGTGGCGACGTGATCTTGGCACCGCCGCTTGTTGACAGTGTTCCGCCAGCGACTGCGGTCTGGACACGGCTGGACGGCAGAAACTTGGACAGCAGAAACTTTGCACAAACACAGGATAACCGCCTGGTCATTCTTGACGTCAGCCCCAAGGATGCCGGCCAGTACCGGGTGGAACTGACCAATCCGCACACGGGAGACAACCTCTCGGGACCAGTGGTGGAGCTGACCGTCGATGACAATGAAGAAGACCAGGCAGAACTCTCCATTGTTGTTCCGCCATCAGATCGAGAGTTCAACAACCTTGGCAATGGCTATGACAGCACTCTCGAGTGCATTGCCTCTGGCAGGCCTCTGGACCAGGTGCAGATTGAGTGGCTGAAGGATGGTAGGGCCCTGGGTGAGCTGCCACACGTTCTCACCCACTGGAACCGCACCCTGACACTGCTGCGCCTGGGGCCCGGGCACACTGGCCGCTACTCCTGCCAGGTCACCCTTCGACATGCCCCTGAGGACTCCTCTGCTGTCGTGGCCCATGCCAACGTCACTGTGTCAGTGCTGCCCTCCTTGAGCCAGAAGGTGAATGAGGAGACGGCAGTGGAACTGGGGCAGCAGGTGCGGCTTCCCTGCGTGGCTGAGGGCCACCCAGAGCCCCAGGTGCACTGGCTCCTGGATGCGCGGCCTGTGGACCAGAGCACAGGGCACTTCCACGTTGGTGACGGCGGCAGCCTCGAGATTGGAGCCCTGGCTATGGAAGATGCCGGGGTGTACCAGTGTGTGGCGGAGAACCCGCTGGGTGAAGCTCGCGCTTCTACCTGGCTTCATGTAAAAAGAACTTGCCATTTGCCATCTACATTTCATTACCCATGGTGACAATCATCTATCTTCTTGCCAATATTTCATACTTTGTGGTCCTCACTGCTGACGAAGTTCAGTCGGCAAATGCAGTGGCTGTGCCTATTTTTTGTTGGCGCAAGACAGGGACACCTGCCTAGTTGTCTCGCCATGATCAATGTGACTCATTTTACACCAGCTCCCAGCCTAGTCATTTTGTGCCTTCTCAGCCTGCTGTACCTGACAAACACTGATGTGTTTGTTCTCATCACGTACACTGCCTTTAGTGAGGCCATGTTCATCATGCTGTCTGTGGGTGGGATTCTCTGGCTTCGGATAAAGCAGCCTAATACGAAACGACCAATTAAGGTGAACATTATTCTGCCGGTGGTGTTCTTCCTCATCTCGCTGTTCCTCGTGGTGCTGCCTTTCTTCAGCCAGCCCCTAGAAACATCCATTGGTGCAGGCATAATGCTTTCTGGAATTCCAGTTTACTTTCTCACCATCTACTGGAAGGATAAACCTCTTGCATACAGGAAATCAATTTATGTGGTGACAGAGTATGTGCAGAGAATATTGTACAGTGGACCCCAAGAAGACAAGGTGGGATAAATGAATAACAGCACACATTCTACAGAGCAAGAATTTCCAGGAAAGATGCATTCATCACATGGAACATGTTCTCATCTTACTTGAAATTACCATCTTTATGAGGCACATGTTATGCTAGTCTTGAAGAACATAACTCTTTGCATGAACACTTTTTACACATATTTTATATTCTACGATATATTCAATGGTTTGTCAGAATGTGGTTCAGTGTAGAAGAAAAAAAAATGCCAGTGGAATGAACTTCAAATAGTGCTTTTTATTTCTTATAATCATTACCTTGACTTGTATTTGGTTGTGTGCAAAATGTGAGTGATGATGTAAGAGCTTTTTTCTTTTATTTGTTTATTTTGTCTCTGACTTTGTATGCAGCATTTCGGAGTTCTAGCGCTTCAGTTAATATAGTATACATTTGTTATTGCTTGTTGACAGCATGGAAGGGTGCTGGAAATGTTGCATCTGAGGTGAAGAAGATTTGTAAAGAACATTTTTTGTGTCATAGTCTATTGTACTTAAGTTGGTCTGTTTGAAACAAGCTGAGGTGTCCAAAAGACATTTTTGCAAGTTTCACATTATTGTAATGTGAAAATATTAAGTTCAAGACAGAGCGCTTCTGATGTTAGTGTTTACAT

>MG9621233

CTTCTTGGTGCTTGGTCATTTTCCTTATGTGAATTCCCATTTCTGAAATGGTGATGTGGGATGTGTGATGAACTCTTTTGCCATTATGAAACCAGTGGACGGTTGGCACTGGGTTACCCTGTACGGAGCACTGCAACTGCAGCTCTCCTCCATGCAATACGATATGGTTTTGCAACGGTGTCACTATGTGAGGCGGCTCTTGAACAACCAACTGCGTCTTGTGAGCAAAAATCATTCCAAGTCGGTTGGCAGCTTGGCAAACGTATATGCCATTGTCTCTCGAGTGCACACTAAAAATTTCTAGGTTGCCAAGCACCATCCTGTGGCGTGTTCTGGGCAAATGGCCCCCTTCCTTGTTCCAGCTGATGACAGGGGCAGGATTCCCATTAGCAGCACACTCCATAGTTACATTACTTCCAGTGACCACATTCATAACTTCTGGTGGCACGGACGTAAAAGAAGGCACTTCTTCAGCTGCTGGCTCATGCACTGTCAAGTAGATTTTGTGCAATGCCGTCAGCCTCTTTCTGATTAGTGGATTGTGAGCTGTACAAACATACACACTAGAGTCCCAGATTGTGCTCGATTGAATCTGCAGGTCCCCAGAAGGCATTATGTGGTGTCGATCAACTGTTCTATTTACAGATGTTTTGTTGAAACGAAACTCTGCAACCGCTGGAGGGTTGCTGTGAGGCAGCCTGCAAGGAACCACAGCAGTGTTGCCAGCAGTAACGGAAACGTGAACATCCTCTTGAGGCACAAAGGGTTCCAAAGCAGCAACTATGGCAGTGGC

>MG1203248

GCAGGCCACGAACGAGCTTGGCACCGCGAGGTCCACGGCGCGCATTCATGTTGATGCCAAGGGTGGCGTCATCCTCGAAAGCCAGCAGCCAGACGCCCTCCCGAAGATCAAGCAGCTCGAGGAATCATGCGGCTACATCAGGCCAACGCAGGAAGAAGTGGTGATCAAGGACAAGCCTAACTTTGTTCGGGGTCTTTACAACCTGGAAACTCTGCATGAAGGCCAAAGCGCTCACCTTGAGGCTACGCTGACACCAATCAACGACACTAACATGAAGATTCAGTGGTTCCACAACGGCGTAGAAATTCCGCTTGGCCACAGGTTCAAGACTGTTTCTGACTTTGGCTACGTGGCTCTCAACATCCTCTACGCCTACCCGGAGGATTCTGGCACATACATGTGCAAGGCAACCAATCAGCTCGGCGAAGCCGTTACCACATGCTCAATCAACGTTCTCGGAAAGTCCGCCATTGTAACCGACACCTACCACGAAAAGGGCCTGGAGAAAATCAGGCAACTTGAAGAGTACCAGGCGCCGGAGAAGCCTGAACAGGTGATCCAGCTGCAAAGGCCCGTGTTCACGGTGCCTCTGAACTCGCTGGACGGTCTTGTGGAAGGACAGAGCGCTCACTTGGAGTGCAGACTCGAGCCCATCAATGACGCTGATCTCAAAGTTCAGTGGTACGTCAACGGCGTGGAAATCCGGCCAGGACATCGGTTCAGGACGACGCACGACTTTGGATATGTGGCCCTCGACATTATGTATGTATATGCCGAAGATAGCGGAACCTACATGTGCAAGGCAACCAACTCTCTCGGAGAAGCGGTTACAACGTGTAACCTGCGTGCCCTACCCAAGCAAAAGATCTTCTATGATACGCATCATCCGGAGGGTCTTGAGAAAATCCGGGAGCTCGAAGCGCAAGTGAAGTACCAGTCTGCCGAAATTCAGGAGAAGCCGATCTCAAAGCCTGTGTTCATCACTGAACTGAGGGGAACCCAGGAGATCTCGGAGGGTGAAAGCGCCCACTTGGAGTGTCGCGTGGAACCAGCCCACGACGCTAAGTTGAAGATTGATATCCTTCACAACGGACGGCCGCTTACAGCTGCGACGCGCGTTCACATCACCAGCGACTTCGGCTACGTGGCGATAGACGCGACAAGCGCCATTCCAGAGGATTCCGGCACTTACACGGTGCGCGCCACTAACGACCTAGGAACTGCCGAGACAACAGCCACGCTGCGGGTCCTGCCAAAATCCAGCATCATCTCCGACACCCAGCACCCAGAAGGACTGGCTAAGATCAGGGAGATGGAAGACGAGTCTCGTTTCAAGCGTGAAGTCATCCAGGAGCCAGTCACCTACCAGACGCCCGTGTTCACGGTTCCTCTGCAGAACCTGGAGAACCTCGTTGAAGACCAGAGAAACGTCCACCTGGAGTGCAGGCTGATCCCAGTCGGAGATCCTACCCTCAATGTCCAGTGGTTCTTCAACGACACGCCACTGATGGAAGGCACGAGGTTCCATCCAGTTCACGACTTTGGCTACGTTGCACTCGACATGGACTACGTGCGCCCGGAGGACACTGGCGTCTACACGTGCAAGGCCACCAATTCCCTGGGCCAGGCAGTGACCACCTGCATGCTCAAGGTTAAACCCAAGGCGTCCATTCTTCTGGACACCCTGCAACCTCAAGGCTACGAGAAGATCCGTGAGCTGGAAGACTTGAAGGGCCAGAAGCCTCCGGAGAAACCAGACGCAGTGTACGAAAAGCCAGTGTTCACCAGCCACTTGGTTGGTCCTGGCGAGATAAACGAGGGACAGCCTGCACGTCTGGAGTGCCGCTGTGTCCCTGTCGGAGACCCTGACCTCAAGTTCTACTGGTACGTCAACGGCATCGAGCTTCCAAAAGGTTCGAGGTTGATTCCCAACAACGACTTTGGATTTGTGACCCTGGACATCCTGTCGGGCATCGCGGAAGACTCTGGAGTCTACATGTGCAAAGCGGTGAACAAGGCAGGAGAAGCCGTCACTTCAACTTCGCTGCGAGTGAAAGGCCGAGCTGGTGTGCTGCTGGACTCCCACCATCCCGAGGCGTACAGGCAGACGCAGAAGTTCGAGTACGACTCCAGCCGCATTCCTGAGAAGTGGTCCGACGAGAAGCCCAAGGCGGCGCCAGTGTTTGTGCAGCACTTGAACAACATCGATGGCGCTGTTGAAGGCCACTACCTGCGCATCGAAGGGCGCATTGAACCTACCAACGACGACAAGCTCAAAGTCAAATGGTTCAAGAACGGCAAGCCTCTGGTCATGGGTACTCGTATCAAGCCTACGGACGATTTCGGCTTGGTTTCGTTGGACATTTCCAGCGCTAGACCAGACGATTCTGGAATTTACACCTGTAAGGCCACCAACGATGTTGGCGAAGCTATATCGACGTGCACAATCAAGGTTGAAGGCCGCGAGAACATCATCCTGGCTTCCCAGCACCCCGACTCGTTGCCCAAGATCCGCCAGCTGGAAGAGTATGTGCCGCCTGAGAAGTACGTGCCTGAACCAGATTACGAAGGGCCCGTGTTCGTCACGCACCTCAACAACCTGGAGATCCGCGAGGGTGCGACTGCGCACTTCGAGTGCCGAGTGGAGCCATCCAAGGATCCCACGCTCAAGGTGGAATTCCTCAAGAACAACAAGCCAGTTCCTGCTGGTTCAAAGTACAACTTCAACAACGACTTTGGCTTCGTCACACTGGATGTCAGCAATGCATACCCGGAGGACGCCGGCATCTACACGTGCCGAGCCCGCAACGCGAAGGGCGAAGCGGTCACTACCGGCTCTCTCAAAGTCCAAGGCAAAAGCGGCGTGCTGTCCGACACTCTTCACCCAATGGGCGCACAGGGACTGTCCAAGGTCCAGGAGCTAGAGACTTCCTACTTGACCAGATACCAGGCTCCCGTCGAAGAGGCTGAGAAGGTGTTCCCGCGCCCGGTGTTCGTGGTGCCCCTTGAGCCAAACTTCTCCATCCAAGAGGGTAGCCCTGTCACCCTCGAGTGCAAGGTGGAACCAGCAAGCGACCCGAAGCTCAGGGTCGAGTGGTTCCTGAACGGCAAACCACTTGCGCCAGGATCCCGGCACACCGTCACACACGACTTCGGCTTCGTTGTGCTGGCCATGACCGACTTCTGGGGACGAGACGCCGGCGTTTACACCTGCCGCGCCAGCAACGCCGCAGGCGAAGCCTTTACCACAACCACAATCACGTGCCTGACCCGCAAGGGCGTGCAGGAGGACACGCTGCACCCGGAGGGCCGCAAGGGCCTCGAGTCCATCCAGCACCTGGAGGAGTCGCTGACGCGCGTGCCCGAAGCGATCCAGGAGGAAGCCGCTGGCCAGCCGCCAGTCTTCACATCTCAGTTCGTCAACCTCAAGGACCTGAACGAGGGCGAGATCGCCCACTTCGAGGCCACGCTGACGCCAGTCGGCGACCAGACCATGCAGGTGGAATGGTTCTTCCGCGGCAAACCCCTCAAAGCAGGGCACCGCATCCGGACGGTCCACGCGTTCGGCATGGTGGTGCTCGAGATCTTGGGGACTGTGCTGGAGGACTCTGGCCGCTACACTTGCCGTGCGACCAACAAGTGGGGCAAGGCCGAGGTCACCGTCGACCTCGAGTGCACCGACAAGACCAAGGGACAGCGGCCGCAATTCACCACGCAGCTGCAGAACCTCATGGACCTCAAGGAAGGAAACAGCGCTCACCTCGAATGCCACCTGGTGCCTGTTGGCGACCCAGACATGAAGGTCGAGTGGTACAAAAACTCGCAGCCACTTCGCGACAGTTCTCGCATCAAGACACTCAGCGACTTCGGCTACGTGGTGATGGACATCTCATTCGTGCACGCCGAAGACTCTGGAGACTACGTGTGCGTGGCCACCAACAAATACGGCTCCGACGCCACTAAGTGCACCATTCAGTGCGCAGGCACTGGCAAGATATTCAGGGACTCGCTACAGCCGCAATCCCTTGACAGGATCGCTGAACTGGAAGGTGCTTCGGCTCTCACCAGGACATCGGCCGTGATGGAGGCGACCCGGCTCCAGCCGCCCAAGTTCCTGTCACAGCTGAACAACATCACAAACCTGGTCGAAGGCCAGAGCGCACACTTCGAGTGCCAGCTGGTGCCGGTCAACGACCCAGACCTCACGGTTGAGTGGTACTTCAATGGCCAGCTGCTGCGCTCAGGCCATCGTTTCCGGACGTTCCACGATTTCGGCATCGTTATCCTGGACATCCTGTACTGCTACGGAGAGGACTCCGGCGAATGGGTCTGCAAGGCCACGAACAAGCTCGGCTCAGATGTGACCCGCGCGACACTACAGTGCAAGTCCAAGAGCTCCTTGATCCTGACTCCTCAAGTGCCCCCTGAAATGGCCTCGGCGACACAGAACATCATCGCTCTCGAAGAAAGCCTGTACCGCACAGCGGCGGTCATTGAGCCCGAGGGACCCGCTGAGGCGCCTCGTTTCACCGTACCGCTCACTAACGTTGAGGATCTGAGGGAGGGAGACAACGCACATCTGGAAGCCCGGCTGACGCCTACAGATGATCCAGACCTGACTGTGGAATGGTTCAAGAACAACATGCCGCTGATGTCAGGAACAAGAATCCGAACCATCAACGACTTCGGCTTCGTGGTGCTCGAAATGAGCCCTGTGTACCCGGAGGACTCCGGCGTCTACTCCTGCCGTGCTAGAAACCGTTTCGGCGAGGCCGTCACCACATGCACACTCAAGTGTCAAGGAAAGCGTAGCATCATTCTCGAGACACAGCTGCCAGAGTCAATGACCACAGGCATCGAGAAGATTGCCAAGTTCGAAGAAGTCTCCTCGGCTAGGATCGACGAGAAGTGGACGGACAAGGACACCTCTCAGCCGCCCAAGTTCATCACCACGCCGCAGGACCTGACGCTGGCAGAGAACTCTCTGGCTCATTTCGAGTGCAGGTTGACGCCAGTCGGTGACCCTACGCTGAGAGTTGACTGGTATCACAATGGCAAGCCACTTGTTACGGGCTCTCGAGTCAAGACAATCAGCGACTTTGGATACGTCATTCTGGAGGTGGCCGGTGTTTACCCACGAGATTCGGGTGTTTACACATGCAGAGCCGTCAACAAGGTTGGCGAAGCGTCGGTCTCCTGCAAGCTCGCCGTAAAGGGCAAGCAGTCAGTCGTGATGGAACCACAGCTACCTCAAGAATTCCGATCTGGGTATGAAAGCATTCAGAAGTTGGAAGAGTCGATGTACCGAACTGAAGAGAAGATCTATGACGATGATAAGAAGGAACCACCGAAGTTTGTCACGCAGATCCAATCGCTCCTCGATAAGGTGGAAGGCGACAGCGCTCACTTCGAATGCAAGCTTATCCCAGTCGGAGATCCCAACCTGAAAGTGGAATGGTTCTTGAACGGACGTCCCCTTGTTACGGGTACGCGTGTTCACACCATCGATGACTTCGGCTTCGTGGTGCTGGACATCGACTGGCTCTTCCCCCGCGACTCTGGAGAGTATATGTGTCGTGCCACGAACCGTTGGGGCTCCGACACGACCAAGGCAACCCTAAAGATTAAAGCCAAGAAAGACATCATCATGGACAGCCAGCTGCCAGAGGGCATGAACGTTGACAAGCTGCGCGACCTGGAGTACCCGACGCCACAAGAAGAGACAATTCAGGAGCAGGAACCGGTCAAGCCCAGGTTCATCACCCAGATACAGCCTCAGCAAAACCTTAATGAGGGCGACTCGGCTCACTTCGAATGCCGCCTGGAGCCCATCAACGATCCAAAGCTGAGGGTGGAATGGTACCACAACGGACAACCCTTGAGATCAGGTCACCGGTTCAAGACAACGCACGATTTCGGCTTCGTTGCATTGGACGTGCTATACGTGTACCCTGAGGACTCGGGCACATACGTTGCACGGGCCGTCAACGACGTTGGCGAGGACCAAACTCAGGCCACCCTCAGGTGCACAGCGAAGCCAAAGCTGGACTACAGGACCCAACTTCCAAAGGACATGAAGGATGGTGTTAAGAAGATCGCTGAAATGGAAGCCTCCTGGCAGCGCGCTGAGACCCAGGAAGAAGTGGAAGAGGAACCATGCGCTCCCATGTTCATTATGAAGCCAGAGCCTCAAGTGGTCATCGAGGGCGAATGGGCAAAGTTCCAGTGCCGAGTTATTGGCCACCCCAAGCCAAGGCTCATCTGGGTTCTCAACGGCCACACGGTCATCGCCGGCTCAAGGTACAAGCTGACTTACGACGGTATCTACCATCTTGACATCCCTAAGACCCGCCAGTATGACCAAGGAAAAGTAGAAGTTTTTGCAAGGAACTTCTGCGGCGAAGCCTACTGCTTCACAACTCTTGAAGTCCGGCCCAAGTTCGATGACTACAGAGCCGTTCTCAAGCATTCTCCTAAGCCATGGTACGACCAAGACGTCAAGTCTTATCAAAAGTACCGACATGAGACTGAACTGCAACGAGTGTTTGAGGAGAAGCTTACACCTGGAGGCACTCGTATCGATGTCTGGAAGACTGAACAGGGCCAGCAAGGTGAACACCAAAAGATCAAGAAGAGGATCGAAGAAGAAGAGCTGGAGAAGCTCAAGCCGAAAGTGGAGCGCTTCAAGACCGACTCGATTTATTACGATGCGCGCACGGGCGAGAAGAAGGTGGAGACGGGCTCCCAGGCGCAGTACATGGCCAAGTACTTCGAGACCGAGGCCGAGAAGCAGCAGCGCGGCGCTACCGGCATTTCGCCCGAGTCCGTGGTGCAGGGCCGCGAGGTTCACACCACCACCCAGCGGCAGACTCAGAAGGAGCAGCAGGGCGACCTCGAGATCACCCGCAAGAAGACGCTCACCGAGACGCTCGAGCAAGAGCACAAGGGCGTCACCAAGGAGCAGCGCGTCCAGGGGCCCGCGCAAGAGCCGGCGAAGGCTCCCGTGTTCACCAAGAAGCTGCAGCCGTGCCGCGTGGACGAAGGCCGAGGCGCCAAGTTCCAGTGCACCTTCACCGGACAGCCAGCGCCCAAGATCACCTGGTACCGCGAGAACTTCCCCATCCAGCCCTCGCAGGATTTCCAGATCGTGACAACTGACAGCACGTCGACGCTGATCATCCGCGAAGTGTATGTGGAAGATTCCGGCGTCTTCTCAGTGAAGGCTGAAAACCGTGGCGGCTCAGCCAAGTCCTCCGCCAACTTGGTTGTTGAAGAGAGGCGAGAGCAACGCAGCGGTGTCGTGCCGCCAAACTTCACCCGGACCATCCAGGACGTCTCGTCGAAAGCCGGAAAGCTGGTCCGCCTCGACGCAAAGGTCTCAGGATCGAAGCCGTTGGATGTCTACTGGCTCAAGAACGGCAAGAAAGTGACGCCCGACGTGTCGCACAAAATCGTGGAGGAGGACGACCAGTACACGCTGCTCATTTTGGAGGCCCAGGCGGACTCCGACTCCGGAAGCTACGAGTGTGTCGCCATCAACTCGGCGGGAGAAGCCCGCTGCCAGGCTCACGTGGTGATCGAAGGCGCCAAGCCCAAGACGCCGCCCACGAGCCCCAAGGAGGCGCCCGGAGACCAGAAGCCACCCACGGTGACAGAGCCCCTCAAGCCACTCGCCGTCAAGGAAGGCCAGAGCGCCGTCTTCCGCTGCCGAATACCCGCCGTCCCGGGGGCGCAAGTAAAATGGTTCCGAGGAGACCAGCAAGTGAAGCAGTCGCGGTACTTCCGCATGTCCCAAGAGAATAACCTCTTCACGCTCAAGATCTCCGAAGCGTTCCCGGAGGACGAAGGTGTCTACAAGTGCGTCGCCACAAACCCGGCGGGCACTGTCTCTACCAGCGCCAACCTTAAAGTGATTGTGCCCGAACTGAACGAGGTGCCGCCGACGGTCACTCCTCTGGCCGACCTGACCGTGCCCGAAGGCTCGCCCGCCCGTTTCGTCACATCCCTGGGAGGCGTGCCACCGCCCAAGGTCATCTGGGTGCGCGAGGGCCACATCATCAAGCAGTCCCGTGACTTTCAGATGAACCAAGACCAAGGCTCAGCGTCTCTGGTCATCAGGCACACGTACCCCGAAGACGAAGGCGTCTACGTCTGTCGTGCCACCAACGCCTCGGGCCAAGCCGAGACCTCTGCCCGGCTTACCGTGCAACGTAAGGCCAAAAAGTAGAGGTCGCGCCCTGGCCTACTTGCTCGCTGCGGCTTATAGCGCTCCATTCGAAAAGCTGCGCGTACAAGCTGCTCAAACCGAGAGAGAGAGAGAACAAAAACAGCAACAAAAAAAGTTTGGCTCCTCACTTTCGCGTTCTCTAACTACAGCGCAGTAATAACGAGATATAATGTATCTTGCGTCTTAGCGGTGACGCATAGAATCTATTTTCAATTAATTTATTTATTAATTTATTTATTTCTGGAGAGAGTCTCTTTTGCATATTTTTGGTTTACAGTCACTTAACCTTCCCTCTTTTTGTTGAGAGCAAAAAATTTCGAATCGGAGTCCGTTCTTTACATTGCGTTCGCTTTCGTTTACTCCTTGTTCCTGTTTTGGTTTAAAACATTTTGTAAAGAAAAACAACTAATGTACGTACAGGGAATGTTCATTTCGCCCCCTTTTTTATACGATGAAATCGCGACATTCATGCCTAACAGGTTTATTCGTTTCATTAACCAAGTTCTGCCTAACAGAAGGGTGAAGTTTTGTATATTACTGACATTTCTATATTTACTCACGAGTTTTTTTTTTCCTTCGTTTGCAACAAGCGTTGCAGGACTGCACATCTCGGTCAGTTAGCGGCACTGAAGCGCTCGAACAAAGCGCGCACAACGCCACCGTAGGGCGCCACTATAGATGTGCATTCGTGAGGAGCCTTACAATTTATTGCATATTGGGGCTATTAACACCATCGCTTATTACTCCTATTCGAACCGATTGGCCACCATCGCGGGTTTCATCATGGCGTTAGGACCGCGAAATAATCGGCACTGACTGCCACAAGACGAGTTAGACAGGTTTAGGCGATTAACACATTTAACCATCCGGTCGAGAAGGCGACCTCACACTGCCAGCCGCGTTTGATATGTGATAACGCTAGCTGCTTGTTTTTAAACTCTCTCGGGCGCCCCTTGTTTGCGTTTTGTTTTGTTCCTCGCGGTGAGCCGTTCGCAATTCGCCACGCTAGTCACTACAGCAGCTGACGCCGTCGACGCCGCAAAGGCGTGCGCGCGACCCCGGCTGTAGCTTATCCAGGCGATATGTGTAGGCGAACGCGCTCCGTCCTAGACCTCCAAGTGGCCGAAACCTGTAGCGATTGCTTGTGGAGAAAAATTTCAATAAAGCCAAGTTATGCAAAACAAAAAAAAATTTCAATAAAGCCAAGTTATG

>MG120822

GTGGGGGCGCCTGCCCCACGCCTGCAGCTGCTCAAGGACGGGGAGCCCCTGACCAACTGGACCACACAGCGCCTGGTGCACGTGCTGAGCAGTATTAGCCACTCCCAAGCAGGGTCCTACCAGTGCCTGGCCGAGAATGCAGCTGGCGCGCTGCTCAGCGCCAAGGCCCGGCTACGGGTGGCACACCTAACACGAGCCGAAGAAGTCCCTGAAACAGTTCCTGTGAACGCTCGCAAGGGTGGCGACGTGATCTTGGCACCGCCGCTTGTTGACAGTGTTCCGCCAGCGACTGCGGTCTGGACACGGCTGGACGGCAGAAACTTGGACAGCAGAAACTTTGCACAAACACAGGATAACCGCCTGGTCATTCTTGACGTCAGCCCCAAGGATGCCGGCCAGTACCGGGTGGAACTGACCAATCCGCACACGGGAGACAACCTCTCGGGACCAGTGGTGGAGCTGACCGTCGATGACAATGAAGAAGACCAGGCAGAACTCTCCATTGTTGTTCCGCCATCAGATCGAGAGTTCAACAACCTTGGCAATGGCTATGACAGCACTCTTGAGTGCATTGCCTCTGGCAGGCCTCTGGACCAGGTGCAGATTGAGTGGCTGAAGGATGGTAGGGCCCTGGGTGAGCTGCCACACGTTCTCACCCACTGGAACCGCACCCTGACCCTGCTGCGCCTGGGGCCCGGGCACACTGGCCGCTACTCCTGCCAGGTCACCCTTCGACATGCCCCTGAGGACTCCTCTGCTGTCGTGGCCCATGCCAACGTCACTGTGTCAGTGCTGCCCTCCTTGAGCCAGAAGGTGAATGAGGAGACGGCAGTGGAACTGGGGCAGCAGGTGCGGCTTCCCTGCGTGGCTGAGGGCCACCCAGAGCCCCAGGTGCACTGGCTCCTGGATGCGCGGCCTGTGGACCAGAGCACAGGGCACTTCCACGTTGGTGACGGCGGCAGCCTCGAGATTGGAGCCCTGGCTATGGAAGATGCCGGGGTGTACCAGTGTGTGGCGGAGAACCCGCTGGGTGAAGCTCGCGCTTCTACCTGGCTTCATGTAAAAAGAACTTGCCATTTGCCATCTACATTTCATTACCCATGGTGACAATCATCTATCTTCTTGCCAATATTTCATACTTTGTGGTCCTCACTGCTGACGAAGTTCAGTCGGCAAATGCAGTGGCTGTGCCTATTTTTTGTTGGCGCAAGACAGGGACACCTGCCTAGTAGTCTTGCCATGATCAATGTGACTCATTTTACACCAGCTCCCAGCCTAGTCATTTTGTGCCTTCTCAGCCTGCTGTACCTGACAAACACTGATGTGTTTGTTCTCATCACGTACACTGCCTTTAGTGAGGCCATGTTCATCATGCTGTCTGTGGGTGGGATTCTCTGGCTTCGGATAAAGCAGCCTAATACGAAACGACCAATTAAGGTGAACATTATTCTGCCGGTGGTGTTCTTCCTCATCTCGCTGTTCCTCGTGGTGCTGCCTTTCTTCAGCCAGCCCCTAGAAACATCCATTGGTGCAGGCATAATGCTTTCTGGAATTCCAGTTTACTTTCTCACCATCTACTGGAAGGATAAACCTCTTGCATACAGGAAATCAATTTATGTGGTGACAGAGTATGTGCAGAGAATATTGTACAGTGGACCCCAAGAAGACAAGGTGGGATAAATGAATAACAGCACACATTCTACAGAGCAAGAATTTCCAGGAAAGATGCATTCATCACATGGAACATGTTCTCATCTTACTTGAAATTACCATCTTTATGAGGCACATGTTATGCTAGTCTTGAAGAACATAACTCTTTGCATGAACACTTTTTACACATATTTTATATTCTACGATATATTCAATGGTTTGTCAGAATGTGGTTCAGTGTAGAAGAAAAAAAAATGCCAGTGGAATGAACTTCAAATAGTGCTTTTTATTTCTTATAATCATTACCTTGACTTGTATTTGGTTGTGTGCAAAATGTGAGTGATGATGTAAGAGCTTTTTTCTTTTATTTGTTTATTTTGTCTCTGACTTTGTATGCAGCATTTCGGAGTTCTAGCGCTTCAGTTAATATAGTATACATTTGTTATTGCTTGTTGACAGCATGGAAGGGTGCTGGAAATGTTGCATCTGAGGTGAAGAAGATTTGTAAAGAACATTTTTTGTGTCATAGTCTATTGTACTTAAGTTGGTCTGTTTGAAACAAGCTGAGGTGTCCAAAAGACATTTTTGCAAGTTTCACATTATTGTAATGTGAAAATATTAAGTTCAAGACAGAGCGCTTCTGATGTTAGTGTTTACATAGTGGTACTTCGTTGTGTGCTTGTATATATGACTAGATTATGAGATTTCATTTGAAGGAAATGGGGGAAAGATAGGGCCCTATGCTTGTGATTTCACCTTGATTCTATTTAGACCGTCTGGTGACATCCGCGAGGTGTTTTTTTCTTAACTATAATAATTCTTTCGCGTTGCTCCCTATGAGGCCATGAATTTTTTCATAGGTGCCAGAGCTGTTGTTATATCTAGGTGCATAATGTAATGGTACTGTTAATAATTTGTGGCCGCAAATGCAATTAGGCTAATATGCAATCCTGTCACACATATTTCACTGTGTTTATATAGTTCATAGGAATAAATTAAGTGGCAACTACGTGTGTACCGGATAGTTTTGCACACATTTCCACATACTTTTCAGCCTTTTTTTTCTTGGCTTAAGATAAATAGCAGGTACAGTAATAATAATGTCACCAGAGGCAGTTAATTTTTCTGCGCAAATGTTGCAGCAAATTGTCCAGATAACAGCAGTTACTTAAGCTAGCTCTTCTGAACTGAGTTATGCCATAGTGTGTGTTTTGTTGCTGGGCATTAAAATCAGACATAACGTTGGTTTTAATGTGATCGAAAGGTCGCAATGATTGTTCCCTCGTAACTGTTACCTTGAAATGTATGTGTGGGCTGAGGGGCATTTGTGAAATAGATGCACCTGTTGATGCTTCCAGCCACTGCACAGTGTTCAGTACTGAGAATTTACACGTTGCCATGGATGAGAATCTAGCGTGTGTGTGTGGGTAGATGTGCTAAATCATCTGTACCGAGCCAAATCTACTCGTGGATGACTTTTTGTGGTTATGCAATGGAAGCCATGAGCATGATGTGATGGCTTTGACATGCTCACACACCAAATGAAGTCCTACAAGCTGGTAGCTCGCACTCATAGCCTCCATTGCATAGCCAAAGAAAATTGTTCACAAGTTTAGCACAGGTATGTGGTGTAGTTCTTACTTTCCCGTTCTTTATTTCTGAAATGGAGGAATGGTTTTCATGTTTTGCAGAAGACTTAAAAGGGCTGTTGTGCGAGTTTTGGCAGTTATAATTTCAGGTTTATTAAATACTTCGCCAAACGTTGCATTGTGCAGTGGTTCAAAACTGGGGAAGCTCACTGTTGCAAATTTCAAGTCTAGCAGCCTGTGGCGCCATCTCCTGTGGGTAAATGAAGGACAAGGCATTGGTCTTGACATGCATAAAAAGTTTCAGGGTGCTGCAGGCAGCAAATATCATAGCTGCTAATCTCTGGAGTATGACCACTGGGTATGCGCGGATATTGGACTGCAGTTTTTCAATAGGTATTGTTAAGAGCCTCTAGTTCATTGTGAGTGCAGTGGTGCATGTGGACTGATTGCTGTAGCATGGTCAGTCGCGCTTCTTGTTGGGAACACTATGTTGTGGCTGTTCCTGTATTATAAGAATCCTTGCATTGAGAGTGTGTTGGCTGTCAACTGCAACATCTGCCCACATATTGAGAACTGAGAGGCCACATTTCAGAACTTTTTATTTAGACATGTTTGTGTAAACTTGTGGTGATGTATGTACTTTTAAATATTTGGATAGTGTTTGCCACGAGCAGTTATTGACTTGCAGATGCGAAAAATTTTGTTTTGATGTAGTTGGATTGTCAACGTGTTTGCGTTATGCTGCTGTATTTTGACATAGTTTTTTTTTTCTAGTTGTCAGTGATATTTACTATAAATGAAAGGTTGGTGTAAATTTCCATCTATGCTGGAAGTTATGAATGCGTATGTCATCTGTGCTGTCAAATATTCAGATTTAGTGTGCACAAGATACATGAAATCCAAAGGGACCATGCGAATGTGAATTTATTTTGATTTTTGCTTCCTGAGCTCTGTTGCCAAAGTGAAATGTGTGGCTTGTGAAAACAAGGTTGTGTTGGTTTTGTATTGCTTTTTATAATTTCAAGTGGTCCTTGCCTTATGGGTAAGAGTAACTGTTCATACGAATTGAATTGAGTTTACATTGCTCGAGTGTCCAGCATTCTTTACATGCTCGCTGAAAATGTGTGAGCATGGCTATCACTGGAACCTGAAAAGTATCACAATCAGTAGACAGCTCCAGGCTATGTTGGACTCTTGTTGTTGTTGCCAGGTTTGTCTCAGCACAGCAAATGAAGGCAAGCTTGACACATTGGAAGCACAAAAACATCCACACAGAAACTCTAGTTTTCTATTGTGTGGTTATAGTGGAGAATAGTACCACTGATGAATGACAAGGCAGGCAAAAGGATGAGTTCCTTTTAAGTCCTATTGTGTACTATGCAGCGAATTGCTTTTATTAGACATCTAAAGTGACATCTTACTTCAAATCTGTACTTGTTGAACCAGTAGCATATCTGATAATGCCCCCAGCACGCATGGAAATAAAATACTTCAATTTGAAATAATTACTGTCATGGACTGGCATGTACTTCAGGGAGTAAAACATTCTAACGATTTATGACTTATTTCACCAACATGAACTGGACTTGATGTTCCTGCTTAACTGGAACATGGGATTTTACTCCTGTGCTTTTCTGCCACATTACCTATTGCAGATAAAATGGCACTTGCAGACTGTACCTTGCAGTTTAGTTGACCTGCTTAAAGATAACTGCTTTTAAACTGATGGCAAGGGTGCACAGCATCTTGCATGAGAATGACATTCTATGAATAAAGCAGACTTGAGTGGTACCTGTTTTAGAAATACAAAAAGGGGCTATGTCAGTGTTTCAACAGCCACTTGGCACATTGTTCGATTCAGAGGAAGGTCGAATTTTCCGCTAGTTAGTAACACATTGAGAGGCACAGCTTGCATAGAGGCAAAGGTGAAACACGGGATGCCTGTCCTTCCTTTTTTCTCATCCTGTTCTTCCTTCCTCTCGTTTGCATTTCACCGCTGTGCCTTTCTGCCGGTATCTTCGATGCTGGTTTGGCACTTCATGTTACTTAATTTGTTTTGACACTTGCTTTCACCTCCTCAAATTTTATGGTGCATTAGGGCATTTTTAAAACCTTAACTATTCCCACTTCTGCAGTGCAAATGTTAATGTACAAGAAAACATAATGGAAACGGGGAAGTTTTCCGATTGCATCGTGACAGCCACAGGCTCAAGCAAGTCGTAGCTGGCTGAAGTGCAATAATTGTGCCATTCCTCAGGCATTGCTTGTTATTTCTGAAAGATAATCTCTTGGTGCTACAAAGAATAATTGACAGCTCTTGATAAAAAAAGGTAGTCTGCATTTCTTTGGGAGTAAACTCGTGAATTACTAGAAACAGACCATGCCTTTAAAGAAACAGCAGTGAGTAGTTTATTTTCCTAACATTTGATGCTGATTACCTCCACTAAAATTCATTAGGGATTGCATGCGACCGTGTTCTGAATAAATGTATGCTTTTAGCAGCATCCCATTTGGGCACATGAAAACGTGAAATGGGCTGCCCGTCTTGGTTGCTTGTGCAAGACGGTGGGGTGGGAGTAGCGGTCATAGGATGATATAAGTGGCCTGTGCAAATTTGCCTCATATAGCCACGTATCGCTGGTAGTAACAAATTTATTACGACGAACCTGGGGCAGCCAGATGGCATGGTACGTCACTTTTGGCTCTATGTGGCGAAAATGGTAATCCCCGATTCCCGCTGCTACATCTGGCCCGAAAACAGTAATGTGTTTGGTAACTTTAAAACACGCCACTCAAACATGAGAAATGAACCTGNNNNNNNNNNNNNNNNNNNNNNNNNNNNNNNNNNNNNNNNNNNNNNNNNNNNNNNNNNNNNNNNNNNNNNCTGGAAACGCACTCTGCCGGGATATGGCCACGTGAGAAGAACTGGCCAATGCGTGGCGCAGCGGCGAATACAAAATAGTTGCGGTGCTTTCCACGTTCCAGTTACTTGCTTTTAGCAGCCACCCACACCTCCATGCACACCTCGCATGACCCTGGTGCGGATTCTGAATACTGCTGCGATGGCATCCAGTGGGACAGTACTTCACGTGTGTTTTGTTGCGCGCTAA

>MG12048954

CGCCGGGAAGTACGGACAAAAAAAGTGGAACCTTCTCTAAGACTGGCGCTGTCTCAAGAACCAGACGGCACAGTTCTTTTGTCGATAGAGCACGCAACGCCAGACGACGCCGGGAAGTACGTCTGCGTCGCCAGGAACCCCGAGGGCAAGGCAGAAAGTTCGAGCACGGTGAAAGTGGCAGAGCTCCCGAAATATGAGCCTGAAATTGTAGAGGAGCTTAAGCCGGCAGTCTTCACTGAAGGTGAACCTGGAAAATTGGAGGCCAAGGTATCTGGGGACCCGATGCCAGATGTCAAATGGAT

>MG12034984

CGGGCCTGTAAGGCCCCTTGGAGTTCCTCTGGGGATGGAAAGGCATGTTCTCGTAGGTGGCCCTGTTTTCGGGCTCGCTGCTGCCGAGCGGCGGAGGCCCGTTCTCCGTCTTGCCTTCGACGTTCTGGTACACCGGCTCGGCTCCGGCCTTCGACTTGGACTTCACCGGACTGTTGAGATCTCTCCGCCGCCTCCTTTTCATTATGAGGACGAGAACAACTATGATAATGATAAAAATTAAAACGATGAGTCCCGCGATGCCCGTAATCATCATGACTTGCTCATCGTCTAGGTAGACATGATTGTAGGCATCGCCGGGCCCATTCACGCGGTATTCGCATTCGGCGGCTCCCAGAGAGTTGTTCGGCTGGCAGAGGTAGAGACCGTAGTACTCGGGCGCCGTCGAGTCCGGCACTGTGTAGATGCTGCGCAGACCTTCCGAGTAGACGTTCCGCTCGAGCGTCTCGTTGCCCAGCGTCCAGCTGATGTTCACCTGGGGCGGGTTGGCGTCCACCTCGCATATGAGCGTCACCTGGCCCTCCGAGTTCTTGCTCGTGTACAGCACGCACGACGGTGGGTAGATGACGTCAATATAGGTGTTGACCGATGCCGAGCCGTGCCTGTTGCTGGCGACGCACGTATAGTTTCCAGGGCGCTCCCTGCCCAGGCTGTAGTTCATGAACAGCAGTGGGCTGTCGGAGATGACTTGGTCGTTGTACATCCACATGTATTCGCTGGACGGCTTGGAGTTGGCGCTGCAGCTGACCTGCTCGGCCACGTTGCCTTCGTAGACAAGGAGGTGTGACACGGACACCTCGATGTCCTCCGGAG

>MG12017795

GTGCCACCAGAAGTTATGAATGTGGTCACTGGAAGTAATGTAACTATGGAGTGTGCTGCTAATGGGAATCCTGCCCCTGTCATCAGCTGGAACAAGGAAGGGGGCCATTTGCCCAGAACACGCCACAGGATGGTGCTTGGCAACCTAGAAATTTTTAGTGTGCACTCGAGAGACAATGGCATATACGTTTGCCAAGCTGCCAACCGACTTGGAATGATTTTTGCTCACAAGACGCAGTTGGTTGTTCAAGAGCCGCCTCACATAGTGACACCGTTGCAAAACCATATCGTATTGCATGGAGGAGAGCTGCAGTTGCAGTGCTCCGTACAGGGTAACCCAGTGCCAACCGTCCACTGGTTTCATAATGGCAAAAGAGTTCATCACACATCCCACATCACCATTTCAGAAATGGGAATTCACATAAGGAAAATGACCAAGCACCAAGGAGGCATGTATCAGTGCTTTGCAAACAACTTCTTGGGCACTGTCTACTCAACGGCCAGAGTCTCTGTCCTGCCGCTGAATGAATCTCATATTCCAGACAAGAGCGGTGATGAGGAAAATGAACCTGACAGCATCAACGGTGCTGCGGAGAATGCTGGGAAAAGAAAAGATGGCCACAAAAGAAAAAAGAGCAAAGGAGTGAAACTGGTGTTGCCATCCCGGCCAGAAATATCCCGACTGTCTGATGACTCGGTGATGGTCCGCTGGAGCGTGCCTCACAACGATGGGCTACCCATCTCGTTCTTCAAGGTCCAGTACCGGGATGTCTCCTCAGCACACTCTCACTGGAAGACAGTAGAGGAAGACGTGCCTCCGCACATCCATTCGCATGCGATCACTGGCCTCAAAGCAGGAGGAAGATATCGATTTCGCATTGTGGCTGTTTATTCCAACAACGACAACAAAAATGGGCCGAACTCAATAAAGTTTCTTCTGCACAAAGACCCTCCAAAGAGGAAACCCACACAGGGGCCCATTATTCGCCGTGTTAAGGCCGAGAGCTCGTCTGCAATCACCCTCTTCTGGGAGTACTCGGACCTAGACGCGGTGGATGTCGAAGGTTTCTACATCTACTACCGTCTCACCCAAAGTGCGGGCGACTACCTTAAGATCACCGTGGCTGGCTCACACGCACGCTCATACACTGTCAGTTACCTGTTGCCTGATACTTCATATGACATCAAGATGCAGTGCTTCAATGTGGCTGGAGCCAGTGAATTCTCCAACATATGCACCCACAAAACATTGGCACTGCCTTCCATGGGGGAGAAAAAGGTGACTCCTGTTCCAGACCTGTTGGAGTCTTCGGTGTCTGGAGACAAGGACCTCCTCTACATTGTCCTTGGAGTGGTGGGAGGGGTGCTGGTGCTTGTCGTGGCAGTGTTCGTGGTGCTGTTCATCCTGCGACACAGGCACAAGAGGAGAAGATGCGTGCAGGGCACGAGTGACCACAACAGCAATCTGCAACAGAATGGCCATATGGGGAATGGGGATTATTTCACCACTCAAAGTAGGATCACCATCAATCCTCTGGACTCAATGGAGCTTTCAGAGCTGTCCAGGGAGAAGTATGCAGCTGCCATAGTGACACAGATCTCAAATGGCCACAGGATCAGTCCAATGAATTTGAGTGCCGAAGACATCATAGTGAAAGCAGAGGACGTCTAGGAGAATCTCATCTGTATTTATGCTTGAGAAGTTGGAGTTGAGCATGGCTATGCAATTTCTGCTGGACATACTGCATTGTACAATGAATTTTATATACATGTTTATTAAATATTCG

>SG4829403

AGTTTACAGGACACACCTTTAGAACCAGTGCTTGCTGTCAAAGGAGATGAAGCACTGATAGACTGTGTGGTGAAAGATCAAGCAAACTACACAGTGCTATGGAGGAGGGTGTCTGATCGCGACAAGGGGGCCGTTCTGACGGCAGGCAATGTGCGTGTCATTGGTGATCCTAGGATATCAGTCCTACATAACCCTGAGCACAACAACTGGGTCCTCAGGATTAGCAATGTGCAACCTCTGGACTCTGGTCGATACAGCTGTGAGCTGAACACATCACCAAACCAAAGGATAACAAGATTACTCACAGTGCTGGAAGATGCAAGGCGGTCGGCACCTTTCCTCCTGACACACAACTACACTGATTGCTGTAATGAGAGGGGCATTCCACCTGAGTGCTTTGGCTACTGCACACTTCAAGGCATCGTCACAGGGCGACACCACAGCCCTAGGACATGTCTTGAGTACATCGGCATCCTCACTCAGTGCCTTACAGATGGCCGGAAG

>SG4820803

CAAAAATGGCGAGTCTCTGAAGTCCTCTGAAATCATCAGCTTGAAGTACAAGAATCGGGAAGCCTCTCTCAGCATTGGTGAAGTGTATCCCGAGGATGAAGGAGAATACAAGTGTGTCGCGACCAATGCTGAAGGCAAGGCTGAGACACGTGGAAAGCTGACCGTCCTGCCTATGGAGAAAGAGGAGGTAGAGCCCAAAGGCTATGACGGCAAGTCGCCAAAGTTTGCGGAACACCTCAAGAGCCACGTTGTCAAGGACGGCGACGCTGTGACGCTGCAGTGCACGATCAGAGGATCCAGCAAGTTTGATGCGGTATGGCTGCACAACGAAAAGGAAATCAAGTCAAGCAAGGACTTCCAGTACGTCACCGAGGGCGACGTTTACAAGCTTGTGATTGCTGAAGTGTACCCCGAGGATAGCGGCACTTACACCTGTGAAGCTTTCAACGATGTAGGGGAAGCGTTCAGCACCTGCACTCTGGACGTCCTAGTTCCAGGTGAACCATTCGTGGGACCTGGATTCTCCATATATCCAAGGTCGACGACATCTGGAGAAGGCCAACCAGTCGTCTTCAAGTGTGTAACAGACAAGGAAGCTCTTGGAGTTCGGTGGCTGAAAGATGGCCAGACCCTGGAGGAGAGCGCCCACTACAAGCTCGCCCAAGACGGCCGCACCTGCACGCTGACCATCACGCAGGCCACGGTGACGGACGTGGGCCAGTACCAGGTCGTCGCAAAGGACGCCAGTGGCGAGAGCACTGCCAGCTTTGCCCTCAACGTGGTCTCTGAGGCTGACCAGCTATGAGAACCTCAAT

>SG4829522

TTCAGCCTAATAGCCTTGGTGTCTTCTGCATAAGCATTTTTCACTCTGCAGGTATAACGACCTGCATCGCCAGCGGTCACCTGCTGTAGTATCAATGAAGACACCAGTCGTTCTTTCTGAGTGTCGTTGGACACAATAACTCTGTCATAAAGTTGGCTGTTGGTTGCAACTGACTTTTCACCTTTAAGCCACGTTACCGTAATTGGGTGATCACCAGTAGCAATGCAAGTGAGGTTGGCAGTTTGGCCAAATTGCACAGAAAGATGCTGTGTTAAAACCTGCGCTTGGGCGACTGTGTACACAGTAACAGCGACAGCCTTTTTCAGCGCCGAACCAATACCATTGTCTGCACTACACGTGTACATGCCAGAGTCAGATTTCTGAACGTCTCGTATCAGAAGCGTGCCATTAGAGAGCAGCTTGTGCCGATCAGAATCCTCATTGCCTGACATGGATCGCAACTGAGCGACTCTTCCTCCATTGTCATGCGCCCATGCTACTTGTGGAACGGGCGTGCCTTTCACGTTGCAATGAACTGTGACCGCGCTCCCTTCTGCAGCCTTTACATCTGATGGTTCGACTACCCATCGAGGCGCAGCATTCACTACCAGCTCCACTGACGCGCTATCCACACCCGCAGCGTTTCTG

>SG4821540

AGCCCCTGACCAACTGGACCACACAGCGCCTGGTGCACGTGCTGAGCAGTATTAGCCACTCCCAAGCAGGGTCCTACCAGTGCCTGGCCGAGAATGCAGCTGGCGCGCTGCTCAGCGCCAAGGCCCGGCTACGGGTGGCACACCTAACACGAGCCGAAGAAGTCCCTGAAACAGTTCCTGTGAACGCTCGCAAGGGTGGCGACGTGATCTTGGCACCGCCGCTTGTTGACAGTGTTCCGCCAGCGACTGCGGTCTGGACACGGCTGGACGGCAGAAACTTGGACAGCAGAAACTTTGCACAAACACAGGATAACCGCCTGGTCATTCTTGACGTCAGCCCCAAGGATGCCGGCCAGTACCGGGTGGAACTGACCAATCCGCACACGGGAGACAACCTCTCGGGACCAGTGGTGGAGCTGACCGTCGATGACAATGAAGAAGACCAGGCAGAACTCTCCATTGTTGTTCCGCCATCAGATCGAGAGTTCAACAACCTTGGCAATGGCTATGACAGCACTCTCGAGTGCATTGCCTCTGGCAGGCCTCTGGACCAGGTGCAGATTGAGTGGCTGAAGGATGGTAGGGCCCTGGGTGAGCTGCCACACGTTCTCACCCACTGGAACCGCACCCTGACACTGCTGCGCCTGGGGCCCGGGCACACTGGCCGCTACTCCTGCCAGGTCACCCTTCGACATGCCCCTGAGGACTCCTCTGCTGTCGTGGCCCATGCCAACGTCACTGTGTCAGTGCTGCCCTCCTTGAGCCAGAAGGTGAATGAGGAGACGGCAGTGGAACTGGGGCAGCAGGTGCGGCTTCCCTGCGTGGCTGAGGGCCACCCAGAGCCCCAGGTGCACTGGCTCCTGGATGCGCGGCCTGTGGACCAGAGCACAGGGCACTTCCACGTTGGTGACGGCGGCAGCCTCGAGATTGGAGCCCTGGCTATGGAAGATGCCGGGGTGTACCAGTGTGTGGCGGAGAACCCGCTGGGTGAAGCGCGCGCTTCTACCTGGCTTCATGTAAAAAGAACTTGCCATTTGCCATCTAC

>SG966167

TCGATGACAATGAAGAAGACCAGGCAGAACTCTCCATTGTTGTTCCGCCATCAGATCGAGAGTTCAACAACCTTGGCAATGGCTATGACAGCACTCTCGAGTGCATTGCCTCTGGCAGGCCTCTGGACCAGGTGCAGATTGAGTGGCTGAAGGATGGTAGGGCCCTGGGTGAGCTGCCACACGTTCTCACCCACTGGAACCGCACCCTGACCCTGCTGCGCCTGGGGCCCGGGCACACTGGCCGCTACTCCTGCCAGGTCACCCTTCGACATGCCCCTGAGGACTCCTCTGCTGTCGTGGCCCATGCCAACGTCACTGTGTCAGTGCTGCCCTCCTTGAGCCAGAAGGTGAATGAGGAGACGGCAGTGGAACTGGGGCAGCAGGTGCGGCTTCCCTGCGTGGCTGAGGGCCACCCAGAGCCCCAGGTGCACTGGCTCCTGGATGCGCGGCCTGTGGACCAGAGCACAGGGCACTTCCACGTTGGTGACGGCGGCAGCCTCGAGATTGGAGCCCTGGCTATGGAAGATGCCGGGGTGTACCAGTGTGTGGCGGAGAACCCGCTGGGTGAAGCTCGCGCTTCTACCTGGCTTCATGTAAAAAGAACTTGCCATTTGCCATCTACATTTCATTACCCATGGTGACAATCATCTATCTTCTTGCCAATATTTCATACTTTGTGGTCCTCACTGCTGACGAAGTTCAGTCGGCAAATGCAGTGGCTGTGCCTATTTTTTGTTGGCGCAAGACAGGGACACCTGCCTAGTTGTCTCGCCATGATCAATGTGACTCATTTTACACCAGCTCCCAGCCTAGTCATTTTGTGCCTTCTCAGCCTGCTGTACCTGACAAACACTGATGTGTTTGTTCTCATCACGTACACTGCCTTTAGTGAGGCCATGTTCATCATGCTGTCTGTGGGTGGGCTTCTCTGGCTTCGGATAAAGCAGCCTAATACGAAACGACCAATTAAGGTGAACATTATTCTGCCGGTGGTGTTCTTCCTCATCTCGCTGTTCCTCGTGGTGCTGCCTTTCTTCAGCCAGCCCCTAGAAACATCCATTGGTGCAGGCATAATGCTTTCTGGAATTCCAGTTTACTTTCTCACCATCTACTGGAAGGATAAACCTCTTGCATACAGGAAATCAATTTATGTGGTGACAGAGTATGTGCAGAGAATATTGTACAGTGGACCCCAAGAAGACAAGGTGGGATAAATGAATAACAGCACACATTCTACAGAGCAAGAATTTCCAGGAAAGATGCATTCATCACATGGAACATGTTCTCATCTTACTTGAAATTACCATCTTTATGAGGCACATGTTATGCTAGTCTTGAAGAACATAACTCTTTGCATGAACACTTTTTACACATATTTTATATTCTACGATATATTCAATGGTTTGTCAGAATGTGGTTCAGTGTAGAAGAAAAAAAAATGCCAGTGGAATGAACTTCAAATAGTGCTTTTTATTTCTTATAATCATTACCTTGACTTGTATTTGGTTGTGTGCAAAATGTGAGTGATGATGTAAGAGCTTTTTTCTTTTATTTGTTTATTTTGTCTCTGACTTTGTATGCAGCATTTCGGAGTTCTAGCGCTTCAGTTAATATAGTATACATTTGTTATTGCTTGTTGACAGCATGGAAGGGTGCTGGAAATGTTGCATCTGAGGTGAAGAAGATTTGTAAAGAACATTTTTTGTGTCATAGTCTATTGTACTTAAGTTGGTCTGTTTGAAACAAGCTGAGGTGTCCAAAAGACATTTTTGCAAGTTTCACATTATTGTAATGTGAAAATATTAAGTTCAAGACAGAGCGCTTCTGATGTTAGTGTTTACAT

>SG9633649

CCACATTGTCGCCGCTTGTGGGACGTGGGAAAAAAAGCGCCGGCCGCGTCGTCGTTAACGGTGGCCTCTGCTGCCGTCGAAAACACAGACGCGGCAGCGAGTGGCCCGTGGTAACAATCGGAACGAGGAGACCGCACGAGCGCGGTCTTGCGTTGCGCGGAAGCCGTGTTTATCGACATTGTCAGCGAGTGGCTGACGGATCCCAGACCGTTGTGGGCCCTGCACGTGTACTTCCCGGCGTCCTCTGGTGTGACTGCTTCG

>SG9636313

CACGCCGCAGGACCTGACGCTGGCAGAGAACTCTCTGGCTCATTTCGAGTGCAGGTTGACGCCAGTCGGTGACCCTACGCTGAGAGTTGACTGGTATCACAATGGCAAGCCACTTGTTACGGGCTCTCGAGTCAAGACAATCAGCGACTTTGGATACGTCATTCTGGAGGTGGCCGGTGTTTACCCACGAGATTCGGGTGTTTACACATGCAGAGCCGTCAACAAGGTTGGCGAAGCGTCGGTCTCCTGCAAGCTCGCCGTAAAGGGCAAGCAGTCAGTCGTGATGGAACCACAGCTACCTCAAGAATTCCGATCTGGGNNNNNNNNNNNNNNNNNGTTGGAAGAGTCGATGTACCGAACTGAAGAGAAGATCTATGACGATGATAAGAAGGAACCACCGAAGTTTGTCACGCAGATCCAATCGCTCCTCGATAAG

>SG9629673

CGGCGATTGTGTCAAATGAACGAGTGATGAACAGGCCGTAGACTCAAGTTCAGTGTTGTGCACCGATCTTGCAAAATTTCTCATTTGGCGTGGTCTTCACTGCAGAACCTCCAAGGGTCAACCCGTTCTCCTTCTTGAAACGCTGGCGGGCCGGCGAGAAAACGACGGTGACATGCATGGTCGCGTCCGGCACGGCGCCCATGAAGTTCATTTGGATGAAAGACGGGAAGGAGCTGACGGAGACTAGCAGCGTCCGCTTCAAGCACGAACAGGGCTACTCGATGCTTTTCATCGAGCCCGTCGAAGTGAACAGCGGAGGCAACTACACCTGACAATGTCGATAA

>SG9638655

CTAGCTTTTCCGTGAGCTCCTCCGTTTCGTCGATGGAGGGCACGGTGACTGCGCTTGCGTTCCTTGTGTGCAGGATGTTGTCTGGGCCCCTCGCTTGGTCTCCAGAAACAACCACTAGCACGGTGAAGTTCTTCCAGGCCACGCCATCGTCGTTGGAAGCCTTGCAGAAGTAGCGTCCGGCATCGCGCGGCTGCGCGTTCTGGATGACCAGCGCGTGCCGAG

>SG12018304

GTGGATCGTTCACAAGGGCTTCCAAGCCAGCGTTCAAGTGCGCTGCGGCCACCCACCACCATTGGAGGGCCTGCCTGTGCTCGACATTGCTGCCGAGAACTTCACATGCAATGATTTTCCCAAGCCTCAGATCTTGCTGCAAAGTCCCGAGAACCAGGTTGCACTCAAGGGCCAGAACATCACGCTCGTCTGCAAGGCAGCAACTGCAAGTGCTTCAAGACTGGAGTTTCAATGGAGAAAAGAACTAAAATTCCTGTCCGATGTTGAGACAGAAGTCTCTGAAAAAGTAGAGCCGAACGATGTCGTGGTCTTCACAAGCTACCTGCACCTGAGGAATATCCAAAACAAGGATGAGGGGCGGTACCAGTGTGTGATTCGAAATCAGTTTGGCTCTGTCTACTCCAACCAGTCCAACATTAGTGTGTATGTCCTGCCTACGTTTGTCAAGACTCCATCCAACTTGACGGTGCGTGCGGGAGGCACAGCGCGGCTTGAGTGCGGAGCCACGGGCCAGCCAACACCGACGGTGTCTTGGCAGAAGGACGGTGGGGACGACTTCCCAGCTGCCAGAGAGAGGCGAATGCATGTCATGCCCACTGACGACGTCTTCTTTGTTGTGAGTCTCAAGGCAGCCGACTCGGGAGTCTACACCTGCACTGCCCGCAGCCGAGCCGGTGTGGTGCGGGCCAACGCCACGCTCACCGTGCTTGAAACACCAGCATTTGTGCGCCCCATGCGGAGCAAGCAGGTGGCGGCCGGGGACACGGC

>SG12019296

CCAGTCGATGTCCAGCACCACGAAGCCGAAGTCATCGATGGTGTGAACACGCGTACCCGTAACAAGGGGACGTCCGTTCAAGAACCATTCCACTTTCAGGTTGGGATCTCCGACTGGGATAAGCTTGCATTCGAAGTGAGCGCTGTCGCCTTCCACCTTATCGAGGAGCGATTGGATCTGCGTGACAAACTTCGGTGGTTCCTTCTTATCATCGTCATAGATCTTCTCTTCAGTTCGGTACATCGACTCTTCCAACTTCTGAATGCTTTCATACCCAGATCGGAATTCTTGAGGTAGCTGTGGTTCCATCACGACTGACTGCTTGCCCTTTACGGCGAGCTTGCAGGAGACCGACGCTTCGCCAACCTTGTTGACGGCTCTGCATGTGTAAACACCCGAATCTCGTGGGTAAACACCGGCCACCTCCAGAATGACGTATCCAAAGTCGCTGATTGTCTTGACTCGAGAGCCCGTAACAAGTGGCTTG

>SG12027103

TCGCTCTCGAAGAAAGCCTGTACCGCACAGCGGCGGTCATTGAGCCCGAGGGACCCGCTGAGGCGCCTCGTTTCACCGTACCGCTCACTAACGTTGAGGATCTGAGGGAGGGAGACAACGCACATCTGGAAGCCCGGCTGACGCCTACAGATGATCCAGACCTGACTGTGGAATGGTTCAAGAACAACATGCCGCTGATGTCAGGAACAAGAATCCGAACCATCAACGACTTCGGCTTCGTGGTGCTCGAAATGAGCCCTGTGTACCCGGAGGACTCCGGCGTCTACTCCTGCCGTGCTAGAAACCGTTTCGGCGAGGCCGTCACCACATGCACACTCAAGTGTCAAGGAAAGCG

>SG12039217

CATGCGACTGTGGGCTTTGGAAATCCATGAATTGTGCAGGGAACTTTGAATTCATCTTGCTTGTTGATACGGACATTGCGTAGTTTTTCATCAAATTCTGCTCGTGGTGGATCTTGGACAGACAGTGTGCATGTTGTTTCTGTGGATCCATGTGGGTTGGTTGCCTTGCAAGTGTATTTTCCTCCTGACTTCGTGGATGCCTCCTTGATACGAAGTACAGCTTTTTGGTCTTTAAATTCAGCACTGAAGTTGCTGATCATGACAAGATCTTTGCCATTTTTAAGCCATTTGACTGTAGGTGTAGGGTTTCCAGAAATTCTGCAGGTTAATTCAGCAGGTTTCTTGATGCCAACAACCNNNNNNNNNNNNNNCTCCAAGATAACTGGTGGTTCACCAGCTTTTGGTTGAATGCGGATGTATTTGGTTCCAGGAGATGGATCGCCTTTTCCAGCTTCATTTTCAGCTGTGACGCGAAACATGTACTCTTCATCGGGTGATAGGCCTTCCACTTTAGTGAAAGTGTCCTGGACTGTGTCAGAATAAAG

>SG1209233

GTAGATGGCAAATGGCAAGTTCTTTTTACATGAAGCCAGGTAGAAGCGCGAGCTTCACCCAGCGGGTTCTCCGCCACACACTGGTACACCCCGGCATCTTCCATAGCCAGGGCTCCAATCTCGAGGCTGCCGCCGTCACCAACGTGGAAGTGCCCTGTGCTCTGGTCCACAGGCCGCGCATCCAGGAGCCAGTGCACCTGGGGCTCTGGGTGGCCCTCAGCCACGCAGGGAAGCCGCACCTGCTGCCCCAGTTCCACTGCCGTCTCCTCATTCACCTTCTGGCTCAAGGAGGGCAGCACTGACACAGTGACGTTGGCATGGGCCACGACAGCAGAGGAGTCCTCAGGGGCATGTCGAAGGGTGACCTGGCAGGAGTAGCGGCCAGTGTGCCCGGGCCCCAGGCGCAGCAGGGTCAGGGTGCGGTTCCAGTGGGTGAGAACGTGTGGCAGCTCACCCAGGGCCCTACCATCCTTCAGCCACTCAATCTGCACCTGGTCCAGAGGCCTGCCAGAGGCAATGCACTCAAGAGTGCTGTCATAGCCATTGCCAAGGTTGTTGAACTCTCGATCTGATGGCGGAACAACAATGGAGAGTTCTGCCTGGTCTTCTTCATTGTCATCGACGGTCAGCTCCACCACTGGTCCCGAGAGGTTGTCTCCCGTGTGCGGATTGGTCAGTTCCACCCGGTACTGGCCGGCATCCTTGGGGCTGACGTCAAGAATGACCAGGCGGTTATCCTGTGTTTGTGCAAAGTTTCTGCTGTCCAAGTTTCTGCCGTCCAGCCGTGTCCAGACCGCAGTCGCTGGCGGAACACTGTCAACAAGCGGCGGTGCCAAGATCACGTCGCCACCCTTGCGAGCGTTCACAGGAACTGTTTCAGGGACTTCTTCGGCTCGTGTTAGGTGTGCCACCCGTAGCCGGGCCTTGGCGCTGAGCAGCGCGCCAGCTGCATTCTCGGCCAGGCACTGGTAGGACCCTGCTTGGGAGTGGCTAATACTGCTCAGCACGTGCACCAAGCGCTGTGTGGTCCAGTTGGTCAGGGGCTCCCCGTCCTTGAGCAGCTGCAGGCGTGGGGCAGGCGCCCCCACCGCCTCGCAG

>SG12023064

CAGTACAGTTCGAGTTCGTCAACATCGCTGTGCGAGACATGCGTCCTCCTGAGTTCAACATGACCACCCTGCGGAACGACGTGGTGAGCGACGTGTCGTACCTCGAACTGTACTGCTCAGCGAACGGCCTGCCGGAGCCCAGCATCTCGTGGTTCAAGGATGGCAAGCCGCTCAACATGACTGGCCGCTGGCTCATCAAGCGCAAGGTGACGCCCCAAGACAGCGGCTTCTACCAGTGCCGGGCCGAGAACAGGGCCGGGGCCATCTATGCCAACACCACCATCTATGCCACCAGTGGAGTACTGGCACATGGCATGAGTAGCACCCAAATAACTTGGATAAGCGTCGGGTTCACGGCATTCGGCGTTTGCATCCTCGTCACCTTGTTCTTTTTCTGCAAGAAATACCGCGCAGTTATGAAAGAAGAGAAGGAGATGGAGTTATTGAACAAAACCCTCTTTGACAAGGGTCAG

>AAFM2010

CACAGAAGCCCACCTTCAGCCGGCCGCTGCACAACGTGGAGACCGTGGAGGGAGCCAACGTGCACCTCGAGTGCCGGCTGCAGCCTGTCGGGGACCCCACCATGCGGGTCGAGTGGTTCCGCAACAGTGTGCCCATCAAAGTCGGCCATCGATTCAGGCCAGCGCACGAGTTTGACTACGTCGCCTTGGACATCCTCAGCTTCTATCCCGAGGACTCTGGCATCTACACGTGCAAGGCCACCAGCTCTCTCGGTGAAAATGTGACGTCCTGCAACGTGAACTGCTTTGCCAAGTCGCAGCTCATTCTGGAGTCTCAGCACCCTGAAGGCCTTCAGAAAATACAGCAGTTGGAAGACCAATCTCGCTACCGCCGCGAGATAATTGAGGAGACAACCGTGAAGACCAAGCCCTCCTTCACATCTAACATGACGGCTCTGAGCCTCCGCGAAGGACAGAACGCCCACCTGGAGTGCCGACTTGAGCCTGTCAACGACGCTGACCTCAGGGTTGAATGGTTCCGCAACGGCGTCTCTCTCCCCATCGGTCATCGGTACAGGCCATTCCATGACTTTGGCTACGTCGCACTGAACATTCTGTCCCTTGTTCCTGAGGACTCGGGAACCTACACCGTTCGTGCCACCAATTCTCTTGGCAAGGCCGAGCTGTCCACGACAATTAATGTCGAAGGAAAGTCTTCCATTGACACGGACACTCAACACCCTGAGGGACTGCAAAAGATCCAGGCCCTCGAAGGCCACCACTATGAGAGGGACACTGACGATCTGGACCAGTCAGTTACGACAGCGCCCGTATTCACATCAGCTCCCAAATCCATCGTGGTCCAAGAGGGACAGAAGGCGCATCTGGAGTGCCGCCTGATCCCCGTGGGAGATACCAAGCTGAAGGTCGAGTGGTTCAAGAACGGCCAGCCGGTGCCAGCAGGATCTCGTTTCGTCGAGATGTGCAACTTCGGTTTCGTGTCTCTGGACATCCTCAACACCTACGCTGAAGACTCCGGCACCTACACCTGCAAAGCCACGAACCAGCTGGGAGAAGCTGTCGTGTCTGCTCAACTCAAGTGCCACGCCGAAAAGTCGTTGATACTGGACACGCAAAACCAGGAAGCGTACGAAAAGATCCAGCAACTCGAGGACTACGGACGCCAAGCAAGGCCCGCCTATGTCGTGGAAGAGATGACCACGCAAGCGCCCGTGTTTACTCAAGCTATGAAGAACCTGAGCCTCAACGAAAACCAGAGTGCTCACTTTGAGTGTAAGCTCATACCAGTGGGCGACCCCAACCTCAAAGTGGAATGGTTCCACAACGGACTCCCCATACAGAAAGCCAACCGGGTCAACACCATTCATGACTTCGGATTCGTCGCTCTTGACCTGAGCTACGTCAAAGCACAGGACTCGGGCACTTACACTTGCAAGGCAACCAACTCCCTGGGATCTGCCGTTTGTTCTGCCACTCTCAACGTCCAAGATTCCAAGTCGCTGGTGTTCGACACTCAACACCCAGAGGGACTTCAGAAGATTCAACAGCTCGAGGAACTAGGACGTTACAAGCCAGAAGTGACGCAGGAAGCCCCGTGCCCGGGCCCACCGATGTTCGTCACCCAGCTCCAGGGCCCGAGCCGTCTGACAGAAGGCGAAAGTGCGCACCTGGAATGTCGTATTGCGCCCTACCCCGATGCCACTATGAAAGTGCAGTGGTTCCACAATGGCGTCGAGCTGCAGTCCGGTCATCGTTACCGGACGATGTACGACTTCGGCTTCTGCGCCTTGGACATTCTGTCAGCGAATGCTGAGCACTCCGGCGAGTACGTGGTCCAGGCCACGAACGAGCTTGGCACCGCGAGGTCCACGGCGCGCATTCATGTTGACGCCAAGGGTGGCGTCATCCTCGAAAGCCAGCAGCCAGACGCCCTCCCGAAGATCAAGCAGCTCGAGGAATCATGCGGCTACATCAGGCCAACGCAGGAAGAAGTGGTGATCAAGGACAAGCCTAACTTTGTTCGGGGTCTTTACAACCTGGAAACTCTGCATGAAGGCCAAAGCGCTCACCTTGAGGCTACGCTGACACCAATCAACGACGCTAACATGAAGATTCAGTGGTTCCACAACGGCGTAGAAATTCCGCTTGGCCACAGGTTCAAGACTGTTTCTGACTTTGGCTACGTGGCTCTCAACATCCTCTACGCCTACCCGGAGGATTCTGGCACATACATGTGCAAGGCAACCAATCAGCTCGGCGAAGCCGTTACCACGTGCTCAATCAACGTTCTCGGAAAGTCCGCCATTGTAACCGACACCTACCACGAAAAGGGCCTGGAGAAAATCAGGCAACTTGAAGAGTACCAGGCGCCGGAGAAGCCTGAACAGGTGATCCAGCTGCAAAGGCCCGTGTTCACGGTGCCTCTGAACTCGCTGGACGGTCTTGTGGAAGGACAGAGCGCTCACTTGGAGTGCAGACTCGAGCCCATCAATGACGCTGATCTCAAAGTTCAGTGGTACGTCAACGGCGTGGAAATCCGGCCAGGACATCGGTTCAGGACGACGCACGACTTTGGATATGTGGCCCTCGACATTATGTATGTGTATGCCGAAGATAGCGGAACCTACATGTGCAAGGCAACCAACTCTCTCGGAGAAGCGGTTACAACGTGTAACCTGCGTGCCCTACCCAAGCAAAAGATCTTCTATGATACGCATCATCCGGAGGGTCTTGAGAAAATCCGGGAGCTCGAAGCGCAAGTGAAGTACCAGTCTGCCGAAATTCAGGAGAAGCCGATCTCAAAGCCTGTGTTCATCACTGAACTGAGGGGAACCCAGGAGATCTCGGAGGGTGAAAGCGCCCACTTGGAGTGTCGCGTGGAACCAGCCCACGACGCTAAGTTGAAGATTGATATCCTTCACAACGGACGGCCGCTTACAGCTGCGACGCGCGTTCACATCACCAGCGACTTCGGCTACGTGGCGATAGACGCGACAAGCGCCATTCCAGAGGATTCCGGCACTTACACGGTGCGCGCCACTAACGACCTAGGAACTGCCGAGACAACAGCCACGCTGCGGGTCCTGCCAAAATCCAGCATCATCTCCGACACCCAGCACCCAGAAGGACTGGCTAAGATCAGGGAGATGGAAGACGAGTCTCGTTTCAAGCGTGAAGTCATCCAGGAGCCAGTCACCTACCAGACGCCCGTGTTCACGGTTCCTCTGCAGAACCTGGAGAACCTCGTTGAAGACCAGAGAAACGTCCACCTGGAGTGCAGGCTGATCCCAGTCGGAGATCCTACCCTCAATGTCCAGTGGTTCTTCAACGACACGCCACTGATGGAAGGCACGAGGTTCCATCCAGTTCACGACTTTGGCTACGTTGCACTCGACATGGACTACGTGCGCCCGGAGGACACTGGCGTCTACACGTGCAAGGCCACCAATTCCCTGGGCCAGGCAGTGACCACCTGCATGCTCAAGGTTAAACCCAAGGCGTCCATTCTTCTGGACACCCTGCAACCTCAAGGCTACGAGAAGATCCGTGAGCTGGAAGACTTGAAGGGCCAGAAGCCTCCGGAGAAACCAGACGCAGTGTACGAAAAGCCAGTGTTCACCAGCCACTTGGTTGGTCCTGGCGAGATAAACGAGGGACAGCCTGCACGTCTGGAGTGCCGCTGTGTCCCTGTCGGAGACCCTGACCTCAAGTTCTACTGGTACGTCAACGGCATCGAGCTTCCAAAAGGTTCGAGGTTGATTCCCAACAACGACTTTGGATTTGTGACCCTGGACATCCTGTCGGGCATCGCGGAAGACTCTGGAGTCTACATGTGCAAAGCGGTGAACAAGGCAGGAGAAGCCGTCACTTCAACTTCGCTGCGAGTGAAAGGCCGAGCTGGTGTGCTGCTGGACTCCCACCATCCCGAGGCGTACAGGCAGACGCAGAAGTTCGAGTACGACTCCAGCCGCATTCCTGAGAAGTGGTCCGACGAGAAGCCCAAGGCGGCGCCAGTGTTTGTGCAGCACTTGAACAACATCGATGGCGCTGTTGAAGGCCACTACCTGCGCATCGAAGGGCGCATTGAACCTACCAACGACGACAAGCTCAAAGTCAAATGGTTCAAGAACGGCAAGCCTCTGGTCATGGGTACTCGTATCAAGCCTACGGACGATTTCGGCTTGGTTTCGTTGGACATTTCCAGCGCTAGACCGGACGATTCTGGAATTTACACCTGTAAGGCCACCAACGATGTTGGCGAAGCTATATCGACGTGCACAATCAAGGTTGAAGGCCGCGAGAACATCATCCTAGCTTCCCAGCACCCCGACTCGTTGCCCAAGATCCGCCAGCTGGAAGAGTATGTGCCGCCTGAGAAGTACGTGCCTGAACCAGATTACGAAGGGCCCGTGTTCGTCACGCACCTCAACAACCTGGAGATCCGCGAGGGTGCGACTGCGCACTTCGAGTGCCGAGTGGAGCCATCCAAGGATCCCACGCTCAAGGTGGAATTCCTCAAGAACAACAAGCCAGTGCCTGCTGGTTCAAAGTACAACTTCAACAACGACTTTGGCTTCGTCACACTGGATGTCAGCAACGCATACCCGGAGGACGCCGGCATCTACACGTGCCGAGCCCGCAACGCGAAGGGCGAAGCGGTCACTACCGGCTCTCTCAAAGTCCAAGGAAAAAGCGGCGTGCTGTCCGACACTCTTCACCCAATGGGCGCACAGGGACTGTCCAAGGTCCAGGAGCTAGAGACTTCCTACTTGACCAGATACCAGGCTCCCGTCGAAGAGGCTGAGAAGGTGTTCCCGCGCCCGGTGTTCGTGGTGCCCCTTGAGCCAAACTTCTCCATCCAAGAGGGTAGCCCTGTCACCCTCGAGTGCAAGGTGGAACCAGCAAGCGACCCGAAGCTCAGGGTCGAGTGGTTCCTGAACGGCAAACCACTTGCGCCAGGATCCCGGCACACCGTCACACACGACTTCGGCTTCGTTGTGCTGGCCATGACCGACTTCTGGGGACGAGACGCCGGCGTTTACACCTGCCGCGCCAGCAACGCCGCAGGCGAAGCCTTTACCACAACCACAATCACGTGCCTGACCCGCAAGGGCGTGCAGGAGGACACGCTGCACCCGGAGGGCCGCAAGGGCCTCGAGTCCATCCAGCACCTGGAGGAGTCGCTGACGCGCGTGCCCGAAGCGATCCAGGAGGAAGCCGCTGGCCAGCCGCCAGTCTTCACATCTCAGTTCGTCAACCTCAAGGACCTGAACGAGGGCGAGATCGCCCACTTCGAGGCCACGCTGACGCCAGTCGGCGACCAGACCATGCAGGTGGAATGGTTCTTCCGCGGCAAACCCCTCAAAGCAGGGCACCGCATCCGGACGGTCCACGCGTTCGGCATGGTGGTGCTCGAGATCTTGGGGACTGTGCTGGAGGACTCTGGCCGCTACACTTGCCGTGCGACCAACAAGTGGGGCAAGGCCGAGGTCACCGTCGACCTCGAGTGCACCGACAAGACCAAGGGACAGCGGCCGCAATTCACCACGCAGCTGCAGAACCTCATGGACCTCAAGGAAGGAAACAGCGCTCACCTCGAATGCCACCTGGTGCCTGTTGGCGACCCAGACATGAAGGTCGAGTGGTACAAAAACTCGCAGCCACTTCGCGACAGTTCTCGCATCAAGACACTCAGCGACTTCGGCTACGTGGTGATGGACATCTCATTCGTGCACGCCGAAGACTCTGGAGACTACGTGTGCGTGGCCACCAACAAATACGGCTCCGACGCCACTAAGTGCACCATTCAGTGCGCAGGCACTGGCAAGATATTCAGGGACTCGCTACAGCCGCAATCCCTTGACAGGATCGCTGAACTGGAAGGTGCTTCGGCTCTCACCAGGACATCGGCCGTGATGGAGGCGACCCGGCTTCAGCCGCCCAAGTTCCTGTCACAGCTGAACAACATCACAAACCTGGTCGAAGGCCAGAGCGCACACTTCGAGTGCCAGCTGGTGCCGGTCAACGACCCAGACCTCACGGTTGAGTGGTACTTCAATGGCCAGCTGCTGCGCTCAGGACATCGTTTCCGGACGTTCCACGATTTCGGCATCGTTATCCTGGACATCCTGTACTGCTACGGAGAGGACTCCGGCGAATGGGTCTGCAAGGCCACGAACAAGCTCGGCTCAGATGTGACCCGCGCGACACTACAGTGCAAGTCCAAGAGCTCCTTGATCCTGACTCCTCAAGTGCCCCCTGAAATGGCCTCGGCGACACAGAACATCATCGCTCTCGAAGAAAGCCTGTACCGCACTGCGGCGGTCATTGAGCCCGAGGGACCCGCTGAGGCGCCTCGTTTCACCGTACCGCTCACTAACGTTGAGGATCTGAGGGAGGGAGACAACGCACATCTGGAAGCCCGGCTGACGCCTACAGATGATCCAGACCTGACTGTGGAATGGTTCAAGAACAACATGCCGCTGATGTCAGGAACAAGAATCCGAACCATCAACGACTTCGGCTTCGTGGTGCTCGAAATGAGCCCTGTGTACCCGGAGGACTCCGGCGTCTACTCCTGCCGTGCTAGAAACCGTTTCGGCGAGGCCGTCACCACATGCACACTCAAGTGTCAAGGAAAGCGTAGCATCATTCTCGAGACACAGCTGCCAGAGTCAATGACCACAGGCATCGAGAAGATTGCCAAGTTCGAAGAAGTCTCCTCGGCTAGGATCGACGAGAAGTGGACGGACAAGGACACCTCTCAGCCGCCCAAGTTCATCACCACGCCGCAGGACCTGACGCTGGCAGAGAACTCTCTGGCTCATTTCGAGTGCAGGTTGACGCCAGTCGGTGACCCTACGCTGAGAGTTGACTGGTATCACAATGGCAAGCCACTTGTCACGGGCTCTCGAGTCAAGACAATCAGCGACTTTGGATACGTCATTCTGGAGGTGGCCGGTGTTTACCCACGAGATTCGGGTGTTTACACATGCAGAGCCGTCAACAAGGTTGGCGAAGCGTCGGTCTCCTGCAAGCTCGCCGTAAAGGGCAAGCAGTCAGTCGTGATGGAACCACAGCTACCTCAAGAATTCCGATCTGGGTATGAAAGCATTCAGAAGTTGGAAGAGTCGATGTACCGAACTGAAGAGAAGATCTATGACGATGATAAGAAGGAACCACCGAAGTTTGTCACGCAGATCCAATCGCTCCTCGATAAGGTGGAAGGCGACAGCGCTCACTTCGAATGCAAGCTTATCCCAGTCGGAGATCCCAACCTGAAAGTGGAATGGTTCTTGAACGGACGTCCCCTTGTTACGGGTACGCGTGTTCACACCATCGATGACTTCGGCTTCGTGGTGCTGGACATCGACTGGCTCTTCCCCCGCGACTCTGGAGAGTATATGTGTCGTGCCACGAACCGTTGGGGCTCCGACACGACCAAGGCAACCCTAAAGATTAAAGCCAAGAAAGACATCATCATGGACAGCCAGCTGCCAGAGGGCATGAACGTTGACAAGCTGCGCGACCTGGAGTACCCGACGCCACAAGAAGAGACAATTCAGGAGCAGGAACCGGTCAAGCCCAGGTTCATCACCCAGATACAGCCTCAGCAAAACCTTAATGAGGGCGACTCGGCTCACTTCGAATGCCGCCTGGAGCCCATCAACGATCCAAAGCTGAGGGTGGAATGGTACCACAACGGACAACCCTTGAGATCAGGTCACCGGTTCAAGACAACGCACGATTTCGGCTTCGTTGCATTGGACGTGCTATACGTGTACCCTGAGGACTCGGGCACATACGTTGCACGGGCCGTCAACGACGTTGGCGAGGACCAAACTCAGGCCACCCTCAGGTGCACAGCGAAGCCAAAGCTGGACTACAGGACCCAACTTCCAAAGGACATGAAGGATGGTGTTAAGAAGATCGCTGAAATGGAAGCCTCCTGGCAGCGCGCTGAGACCCAGGAAGAAGTGGAAGAGGAACCATGCGCTCCCATGTTCATTATGAAGCCAGAGCCTCAAGTGGTCATCGAGGGCGAATGGGCAAAGTTCCAGTGCCGAGTTATTGGCCACCCCAAGCCAAGGCTCATCTGGGTTCTCAACGGCCACACGGTCATCGCCGGCTCAAGGTACAAGCTGACTTACGACGGTATCTACCATCTTGACATCCCTAAGACCCGCCAGTATGACCAAGGAAAAGTAGAAGTTTTTGCAAGGAACTTCTGCGGCGAAGCCTACTGCTTCACAACTCTTGAAGTCCGGCCCAAGTTCGATGACTACAGAGCCGTGCTCAAGCATTCTCCTAAGCCATGGTACGACCAAGACGTCAAGTCTTATCAAAAGTACCGACATGAGACTGAACTGCAACGAGTGTTTGAGGAGAAGCTTACACCTGGAGGCACTCGTATCGATGTCTGGAAGACTGAACAGGGCCAGCAAGGTGAACACCAAAAGATCAAGAAGAGGATCGAAGAAGAAGAGCTGGAGAAGCTCAAGCCGAAAGTGGAGCGCTTCAAGACCGACTCGATTTATTACGATGCGCGCACGGGCGAGAAGAAGGTGGAGACGGGCTCCCAGGCGCAGTACATGGCCAAGTACTTCGAGACCGAGGCCGAGAAGCAGCAGCGCGGCGCTACCGGCATTTCGCCCGAGTCCGTGGTGCAGGGCCGCGAGGTTCACACCACCACCCAGCGGCAGACTCAGAAGGAGCAGCAGGGCGACCTCGAGATCACCCGCAAGAAGACGCTCACCGAGACGCTCGAGCAAGAGCACAAGGGCGTCACCAAGGAGCAGCGCGTCCAGGGGCCCGCGCAAGAGCCGGCGAAGGCTCCCGTGTTCACCAAGAAGCTGCAGCCGTGCCGCGTGGACGAAGGCCGAGGCGCCAAGTTCCAGTGCACCTTCACCGGACAGCCAGCGCCCAAGATCACCTGGTACCGCGAGAACTTCCCCATCCAGCCCTCGCAGGATTTCCAGATCGTGACAACTGACAGCACGTCGACGCTGATCATCCGCGAAGTGTATGTGGAAGATTCCGGCGTCTTCTCAGTGAAGGCTGAAAACCGTGGCGGCTCAGCCAAGTCCTCCGCCAACTTGGTTGTTGAAGAGAGGCGAGAGCAACGCAGCGGTGTCGTGCCGCCAAACTTCACCCGGACCATCCAGGACGTCTCGTCGAAAGCCGGAAAGCTGGTCCGCCTCGACGCAAAGGTCTCAGGATCGAAGCCGTTGGATGTCTACTGGCTCAAGAACGGCAAGAAAGTGACGCCCGACGTGTCGCACAAAATCGTGGAGGAGGACGACCAGTACACGCTGCTCATTTTGGAGGCCCAGGCGGACTCCGACTCCGGAAGCTACGAGTGTGTCGCCATCAACTCGGCGGGAGAAGCCCGCTGCCAGGCTCACGTGGTGATCGAAGGCGCCAAGCCCAAGACGCCGCCCACGAGCCCCAAGGAGGCGCCCGGAGACCAGAAGCCACCCACGGTGACAGAGCCCCTCAAGCCACTCGCCGTCAAGGAAGGCCAGAGCGCCGTCTTCCGCTGCCGAATACCCGCCGTCCCGGGGGCGCAAGTAAAATGGTTCCGAGGAGACCAGCAAGTGAAGCAGTCGCGGTACTTCCGCATGTCCCAAGAGAATAACCTCTTCACGCTCAAGATCTCCGAAGCGTTCCCGGAGGACGAAGGTGTCTACAAGTGCGTCGCCACAAACCCGGCGGGCACTGTCTCTACCAGCGCCAACCTTAAAGTGATTGTGCCCGAACTGAACGAGGTGCCGCCGACGGTCACTCCTCTGGCCGACCTGACCGTGCCCGAAGGCTCGCCCGCCCGTTTCGTCACATCCCTGGGAGGCGTGCCACCGCCCAAGGTCATCTGGGTGCGCGAGGGCCACATCATCAAGCAGTCCCGTGACTTTCAGATGAACCAAGACCAAGGCTCAGCGTCTCTGGTCATCAGGCACACGTACCCCGAAGACGAAGGCGTCTACGTCTGTCGTGCCACCAACGCCTCGGGCCAAGCCGAGACCTCTGCCCGGCTTACCGTGCAACGTAAGGCCAAAAAGTAGAGGTCGCGCCCTGGCCTACTTGCTCGCTGCGGCTTATAGCGCTCCATTCGAAAAGCTGCGCGTACAAGCTGCTCAAACCGAGAGAGAGAGAGAACAAAAACAGCAACAAAAAAAGTTTGGCTCCTCACTTTCGCGTTCTCTAACTACAGCGCAGTAATAACGAGATATAATGTATCTTGCGTCTTAGCGGTGACGCATAGAATCTATTTTCAATTAATTTATTTATTAATTTATTTATTTCTGGAGAGAGTCTCTTTTGCATATTTTTGGTTTACAGTCACTTAACCTTCCCTCTTTTTGTTGAGAGCAAAAAATTTCGAATCGGAGTCCGTTCTTTACATTGCGTTCGCTTTCGTTTACTCCTTGTTCCTGTTTTGGTTTAAAACATTTTGTAAAGAAAAACAACTAATGTACGTACAGGGAATGTTCATTTCGCCCCCTTTTTTATACGATGAAATCGCGACATTCATGCCTAACAGGTTTATTCGTTTCATTAACCAAGTTCTGCCTAACAGAAGGGTGAAGTTTTGTATATTACTGACATTTCTATATTTACTCACGAGTTTCTTTTTTTTCCTTCGTTTGCAACAAGCGTTGCAGGACTGCACATCTCGGTCAGTTAGCGGCACTGAAGCGCTCGAACAAAGCGCGCACAACGCCACCGTAGGGCGCCACTATAGATGTGCATTCGTGAGGAGCCTTACAATTTATTGCATATTGGGGCTATTAACACCATCGCTTATTACTCCTATTCGAACCGATTGGCCACCATCGCGGGTTTCATCACGGCGTTAGGACCGCGAAATAATCGGCACTGACTGCCACAAGACGAGTTAGACAGGTTTAGGCGATTAACACATTTAACCATCCGGTCGAGAAGGCGACCTCACACTGCCAGCCGCGTTTGATATGTGATAACGCTAGCTGCTTGTTTTTAAACTCTCTCGGGCGCCCCTTGTTTGCGTTTTGTTTTGTTCCTCGCGGTGAGCCGTTCGCAATTCGCCACGCTAGTCACTACAGCAGCTGACGCCGTCGACGCCGCAAAGGCGTGCGCGCGACCCCGGCTGTAGCTTATCCAGGCGATATGTGTAGGCGAACGCGCTCCGTCCTAGACCTCCAAGTGGCCGAAACCTGTAGCGATTGCTTGTGGAGAAAAATTTCAATAAAGCCAAGTTATGCAAAACATGTTG

>AAFM6361

CAGGCGACCAACTCCATCGGCTCAGCTACTACACAGGCCCGCCTCGTCTGCGTGTCTCAAGCGTCAATTGTGAAGGAGACTCAGCACCCCGGCGGCCTCCAGAAGATTCAGCATCTGGAAGATTCCGCGCACTACCAACGATCCGAACTCGAAGAGGTCTCCATTAAGGAGAAACCCAAGTTCACGCAACCGCTCCAAGGGCCCTCGGCAATAAAGGAAGGCCAGAGTGTTCACCTCGAGACTCGCCTGGAGCCAATGGGTGACGCCAACATGAAGGTTGAGTGGTTCCACAACGGTCGTCCACTGGCCACCGGTCATCGCTTCAAGACGTACTTCGACTTCGGCTACGTCGCTTTGGACATTTTGTACACATTCCCTGAGGACACCGGCACTTTTGAAGTCCGTGCCACCAACAAACTTGGAACGGATGTACTAACCAAGGAGATAACAGTTCAAGCGAAAGCCTCTGTTGACACAAGCGCGATTCACGAAGTAGGGCTTGAGAAAATTGAGTACCTTGAGGGCTCTGGAGTTGATCACAGCAGCTACTTCCACATCGAAGAAGTCACAAGGACTAAGCCGTACTTCAAGATTCCCCTTAAAGACCCCAAGGGACCCCTAAAAGAGGGTGATTCCATTGTGTTAGAATGCGCCATCGAGCCTCTTCAGGACCCTACCATGAAGGTGGAGTGGTTCTTTAATGGTCGCCCGCTGCCGACAGGTCATCGGTTCATTACCAAGTATGACTTTGGACGAGCATTTCTTCAAATCTTGAACCTGGTGCCCGAAGATACCGGGCAGTACACTCTGCGAGCGACCAATCATCTTGGATCGGCGCATTCAACGTCTTGCATCAAGGTTATCGGCAAGTCCGGCGTGATCACCGAGTCGCAGTACCCCGAAGGCTGGGAGAAGATCCAGCACCTGGAAGACCACTCACGTTACGCCCGCCAGGAGCACGTGGAAAGTGTCATCACACAGAAGCCCACCTTCAGCCGGCCGCTGCACAACGTGGAGACCGTGGAGGGAGCCAACGTGCACCTCGAGTGCCGGCTGCAGCCTGTCGGGGACCCCAC

>AAFM27572

TCTCACCTCTCCTCGGTCAAGTCCAAGGTGGTGACGCTGCAAGTCAACTATCCTGCTGAGTGGAAGCAGCCCATCCAGGACACGGCCTACTCAAGCCCTGGCGCCATGGCCGTGCTCCACTGCGAGGCCGCAGGCATGCCCGAGCCTAGCGTGCGCTGGTTCCGCAACGACCATCTCCTGGAGCCCGACGAGAGCCGCAATATTGTTGGGGATGTGGGCACCAGCACGCTCAAGATCAAAGTGGCTGACGTATCTGACTTTGGGGAGTACATGTGCCGAGCTCGCAATGACCTTGGAGTCCTGGAGCATGTCATCACTCTCAAAGAAGGAGAGGCGCCCAAGGCTCCACGAGTGGCCGTGATCGACTCCAAGCCGAACAGGCTGGTGTTGAAGATCGAGCACCTGCGCAATGAACCTCTCGAGGTGGTGGGCTTTCGTGTCGAGTACAAGACAGACAAGGATTCCAGCTGGGACAGTGCAGAGCACCAGGAATATGGTACCGAAAATGGCATGCAGTATGTGCTGGGGAACCTGAACCACGACACCAGCTACGCCATTCGTGTTTCAGCGAAAAATGCGGCGGGCTATGGTGACTTCTCAGAAGAGATCTACCACCGGACCAAGGACCCGCAACACTACACGCAACACAACACCAACGCCGCTGTCGGCGTAGCCCAAATTGCGCCCGTCCTTGCCTCCTTAAACTGCCTGCTCCTCCTCAGGCTTTAACGAGCCCCGCGGTACTCACAGTGGGAGGGGGGGGGGAAGAAAAAATATTGGAGGACAGGCTTAAGAAAGAAATGGAGCCACATTTCCTCTTTTCCAACACTTTCTTTTTGCTGCTCCCGGAGGACTGTCTTCGGGCAGGTGACT

>AAFM18350

GCGCAACCGAGAGCGACTCGGGCACATACACCGCCGTGGCCAAAAACAAAGCCGGAGAGACGGCCTGCTCCTGCCAAGTGAAAGTGGCCGAGGACGCCGCTCCGCCCGAGCCTCCGCGTGTGCTCAAGGCCCTGGAGGATCTGGAAGTAAAGCCTGGACCGGACCCTATCACGCTGGAATGCATCATCGTCGGCCGGCCCGAACCAGAGGTCATCTGGTACCACAACACCCAACCCATCAAGGAGTCTGAGCGGGTACGGCTGCTGTTCCGCGGGGACAAGTGCTCTCTTGTCCTGAACGGCGTCAGCGCCCAGAACGCCGGCACCTACCGATGCTCGGCGGTCAACCCCATGGGCAGCTGCTACACGGAGTGCAACATGCGCGTGCCGCTTTCTGCTCCCGTGTTTCTGGAGCCGCTCCGAGACGTGACCACGGATGAGGGCTGCCGCGTGGTGCTCACTGCAAAGCTATGGGCACCCGAGCCACCTTTCGTCCGCTGGTTTAAGGATGGAAGAGAGGTGCTTCCAAGCCCAGACTTCCAGGTCAGCCACGATCCGGACGGAACTGTGAAGCTACTGATCCCGAAGGCGGCGGCCAGCAACAGCGGCCATTACGAAGTCGAGGCCAGCAACCCCGGCGGTCGCACGCGCACGGGCTGCAAGGTGCACGTTCGTGAAGCTCAGAAGGCGGTCCAGGCGTCCAGCCAGCTTGCGGTGTCGAGGACATCGCAGCAGACCGTGTCCGTTTCGAAGAGCGGCTCGGAGCTGCGGCTCGCCCGCTCGCTGCCCTCGGAGCTGGTGGTGCAGAGCGGAACCAAGGTGTGCCTCAGCGTGGCCTGTTCGCAACCAGCCGGCGGCCAGCTGACCGCCTCGTGGTTCCGGTCGGGCCAGCCCATCGCCGACTCTCCGGACTTCCGGCTCACCCGTTCTCTGGAGAACGTGGGTGGCGCATCGCTGTCAGTGTTCAGCCTAACCATCTCGGAGGCCTTTCCCGAGGACTCCGGAGACCTTGAAGTGCGCGTGCAAGGCCCCAGCGGCGTTGTGACGGCGCGCACAAAGCTCGTGGTGCTCGACGAAGACGACGACGTCACTGACAGCGTGCAGAGCCGCGACGAGAGCATCCAGCAAGCGCGCGAGGTGAAGGTGTACGAGACGAAGCTGGCTCGACCGGGCCCGGTGGACTCTGCCGGCTTGGCTCTCACGGGAGACCTGCAGCTGCGCGTTACCGAGGTGACGCACGTGAACGAAATGCCGCCGGAGGTCTCGGAAGAGAAGACCACCAAAGTAGTGGACCAAGCTGGCGCCAAAGACAAAGTCACCGAAGAAGTAACTCGCCGAGTGACCAAGCGGGTCACTGAGATTACCAAGACTCACACTAAAGAGGTGAGGGGTCCCTTGGTCTACAAATGGGAGTTTCGCAAGTAGCAGGCCTTCTAGTTTCTTCCTTAGACTTGCTCGGCCGGCACGGAGACTGTCCCGGGAGCTCTTGTCCGCGCTTTCTGGTGGTGCTCTCCGTTGTGTCTGTCTGGCTGTCGCTCGGAACAGAGCGCTCTCCTTCCTTACCCTTGGCCCCTAAGTGTTCGGTGTAGCATGCTCGTCACAGTGTCTTGACCAAAGAAAATAATCTTGACAGTTGTAATGGTGATGTGAAATAAAGCCATGCTTTGTG

>AAFM11696

CTTGCGCCAACAAAAAATAGGCGGGATGATGCGAAAATTCCACCATTGAGGCCGCCAAATGTAGACAGTGCCACAGAGACCGGCATGATCCACGACACCACACCAAGAACCTTTTCTCCAAATGTCACAGCCACTGCATTTGCCGACTGAACTTCGTCAGCAGTGAGGACCACAAAGTATGAAATATTGGCAAGAAGATAGATGATTGTCACCATGGGTAATGAAATGTAGATGGCAAATGGCAAGTTCTTTTTACATGAAGCCAGGTAGAAGCGCGAGCTTCACCCAGCGGGTTCTCCGCCACACACTGGTACACCCCGGCATCTTCCATAGCCAGGGCTCCAATCTCGAGGCTGCCGCCGTCACCAACGTGGAAGTGCCCTGTGCTCTGGTCCACAGGCCGCGCATCCAGGAGCCAGTGCACCTGGGGCTCTGGGTGGCCCTCAGCCACGCAGGGAAGCCGCACCTGCTGCCCCAGTTCCACTGCCGTCTCCTCATTCACCTTCTGGCTCAAGGAGGGCAGCACTGACACAGTGACGTTGGCATGGGCCACGACAGCAGAGGAGTCCTCAGGGGCATGTCGAAGGGTGACCTGGCAGGAGTAGCGGCCAGTGTGCCCGGGCCCCAGGCGCAGCAGGGTCAGGGTGCGGTTCCAGTGGGTGAGAACGTGTGGCAGCTCACCCAGGGCCCTACCATCCTTCAGCCACTCAATCTGCACCTGGTCCAGAGGCCTGCCAGAGGCAATGCACTCAAGAGTGCTGTCATAGCCATTGCCAAGGTTGTTGAACTCTCGATCTGATGGCGGAACAACAATGGAGAGTTCTGCCTGGTCTTCTTCATTGTCATCGACGGTCAGCTCCACCACTGGTCCCGAGAGGTTGTCTCCCGTGTGCGGATTGGTCAGTTCCACCCGGTACTGGCCGGCATCCTTGGGGCTGACGTCAAGAATGACCAGGCGGTTATCCTGTGTTTGTGCAAAGTTTCTGCTGTCCAAGTTTCTGCCGTCCAGCCGTGTCCAGACCGCAGTCGCTGGCGGAACACTGTCAACAAGCGGCGGTGCCAAGATCACGTCGCCACCCTTGCGAGCGTTCACAGGAACTGTTTCAGGGACTTCTTCGGCTCGTGTTAGGTGTGCCACCCGTAGCCGGGCCTTGGCGCTGAGCAGCGCGCCAGCTGCATTCTCGGCCAGGCACTGGTAGGACCCTGCTTGGGAGTGGCTAATACTGCTCAGCACGTGCACCAGGCGCTGTGTGGTCCAGTTGGTCAGGGGCTCCCCGTCCTTGAGCAGCTGCAGGCGTGGGGCAGGCGCCCCCACCGCCTCGCAGCGGAGCGCCC

>AAFM58852

GGTGCTGGGATCGGCCTCCACGTCGCACGATAGAGACACCTCCTCGAAGGGAGCCGTGTACACAACCTTGGCGTGGTGCTGCTGCTGCGCGTTTTGGCACCGGGGCGAGTGCTGTATGCGTATGTGCAGCGTCTTGGAAGTAGTCACTCCCTCCGGGTTGGTGACGGTGCATGAGTAGTTTCCAGAGTCAGCCGACTGAACGTTACGTAGAACCAGGTAGCGCTGGTTGACAATCAGAGGTCCCCGTGCCATCAATGAGGAGGTGCTGTTGGCGGAGTGCTGCGGCTGCAGCCTGTGATCGTGCTGCTGCTGCTGGTCCTCTAATGGGACGCCATTCAACTTCC

>AAFM50441

CGGAACACCAAGCCAGCGGCGGACATCAGGTGGCTGCGGAACGACCTCCCGCTCGCCCAAGAGAAAGCTTTGACCAAGAAGAACGCCTCCGGGGAGAAGCTTTTCTCGGTGTACAGCTCTGTCACGCTCTACCCGAAGCTGGACGACAATCGCGCCGTGTACACCTGCGAGGCCACGCACCCGGCCCTCGAGAGCCCTCTGCAAGCGTCCGTCACTGTTAGCGTATTGTATCCTCCGGGCGCGCCCGAGATCGAGGGCTACCACGAGGGCGACATCGTGCAAGTGGGCGACACCCTGACGCTGGCGTGCATCTCTCGCGGCGGCAACCCGCCCGCTGCGCTCACCTGGAG

>AAUM27907

GCAGTTGGCCCGGGCGGCCGGCCTCCAGCGCGTGCGTCGCGCCCACGATGGACACGCTGGTCGGGCCCAGTATGAGGTCGACGAGGACCTGCGCGCTGAGTGGCTCGGAGATGTTGTTGTTGGAGGCACGGCAGACGAGCTGAGCGTGGAGGTCTCGGCGACCAAGAGGTCCCACGGCCAGTTGGCTGGCGACGTCGCTGGTGCCGTCACCACCTGGCACGAGCACGTCTTCCAGGCGTCGACCCTCGCGCCACCACGAAAGGCTGGCCGGCGGACGCCCTCCGGGTGCCGTGCAGAGAAGCGACAGAGACTGGCCGTCAGTGGCGGTGACACTTCCGTTGAGCGGCCTCCCTCGGCTGTCCCGGATGATGACCGCCCGGGGCGGCACGATCACGGTCAGCCGCACCAGCCGGTTGAGCGTTTGCGCCTGGCGGAAGTCGACGCGGCACACGTACACCCCTTGCTGGTCGGCTCGCAGCGACGGAAGCACCAGGCCCGCGGGACGAAGGGTGAAGCGCGCCTCCCAGGAGTCGTCCTTCCACACCTTGGCGTTGGCGAGTCCGCCTCCGCGGCGGGCGTCCGCGCTGAGTATGGGGGTAGCTGAGCCCTCACGGAACCACAGCACCAGGATAGGTTCATCATCGGGAGCCAGACCCGGGAACTGAGAGCTGCCGCATGGTAAGGACACCTCGCCTCCAGCAAGACCAAGAACTTCTTCTGGAGTCTCATCGTCTTGTAGATATCCGAGGACAGGTGGCGGTGAGGCGATCAACGCGCAGACCACGATCACCGCCGTCACGGCTGCGACGAACGCATCGGCAAACACCGGTGGAGGCCGGACTCGCAGCGTAGCCCGCGACGGCGCCATGGCGCGAAGCCCGAGCCGAGCGAGAAGTTGTTCTTCGAAGCGGCGCGCCACTCGTCG

>AAUM43201

CTTCCTTGTGCACATCAGACACCTCCAAAGGACCTTCAGGGTTGCCTGGAACGTCGAGGACATTGACCGTGACAGTAGCGGTGTCCTTGCCAGAGCTGTTTGTGGCGGTAATGGTGTACTTTCCAGAGTCACCGCGCTCAGCTCGGCGCACAGCCAACTTGGTGTTGTAATCTTCGGTTTCGATGTTGCGGTGAGGAGTAGATTTGATGGTCTCATCGTCTAGAGCCCAAACGATAGTTGGAGGAGGCTCTCCCTGGACGTTAACGTACAACTTGATTGGCTGTCCAGCCTTAATGGTTAAGTCGTGGAGATTCTTGCGGTCGATGCGTGGTGCAAGGAATCTCGGCTTAGCCACGACAGGCTTGGAGGCCTCGCTCGGCTCTCCGGGGCCGGCCTTGTTGACGGCGATGACGCGGAACTCGTACTCCTTTCCTTCGTCCAAGAAAGGGGCGGTTCCCTTGCACTGGTCGCCAGGCACCTCGGCTGCCTNNNNNNNNNNNNNNNNNNNNNNNNCTTTCTTCTCGATCAAGTAGTGTGAGATGGGCGCACCTCCGTCCTTGTCTGGTGGCGTCCACTTGAGGTCGACGTGGTCCTTGTTCCAGTCGGTGGCCTCCGGCTTACCAGGGGCCGACGGGGCGTCGTACGGGTCCTTGGCAGTGATGGGAATGTCAGTCTCCAGAGGCTCTGACTCCCCTTCTTTGTTGACCGCCTTGACGCGGAACTTGTAGTCCTTGCCAGGAGTGAGCCCCGTGACTTGGAACTCGGGCGAGTGGCTCGCACCCACGGGCACCCAGGCACCTGTATCTGGGTCCTGCTTCTCC

>AAUM18537

CGGGTTGCCGCCGCGAGAGATGCACGCCAGCGTCAGGGTGTCGCCCACTTGCACGATGTCGCCCTCGTGGTAGCCCTCGATCTCGGGCGCGCCCGGAGGATACAATACGCTAACAGTGACGGACGCTTGCAGAGGGCTCTCGAGGGCCGGGTGCGTGGCCTCGCAGGTGTACACGGCGCGATTGTCGTCCAGCTTCAGGTACAGCGTGACAGAGCTGTACACCGAGAAAAGCTTCTCCCCGGAGGCGTTCTTCTTGGTCAAAGCTTTCTCTTGGGCGAGCGGGAGGTCGTTCCGCAGCCACCTGATGTCCGCCGCTGGCTTGGTGTTCCGCACCCAGCAGGCGATGACGAGGCTTTCGGCTTCCCGAACTTCCACCACACTGCCGTTTCCGCGGTGTCGGAGCTCGATTTCTTTCGTTGGCACAAGCACTGTCAGGCGCGCTGCAGTCCAAATGGCGTGATTCTTTGGGGCTGGTCCCACCTGGCACTGGTATTCCGCCTCGTCTTCCATCTGAACGTTGGTGATGCGCAGGTTATAGACGCCGCGTTGATTGTCTACGATCATGGTGTACCGCGGAAAACCGGGTATGGACGGGTCGAAACCGAGCAGGAATCCGTCCTTGCTCCACTGCACAGGTCCGGCCAGGTTGCCGACGTGGCACTGCAGCTCGGCCGTCTGTCCCTCGACCGCCTGGGCGTCGCGGGGCCGCACGCGGAAGTACTGCTGGCCGCCCGCCCACGCCACCGAGATGCCTTGCCACCCAAGGAGGGCGAAGGCCAGGGAGAGCCGCGCGCAACCTCCGCGCGCACCCATCACGTCGATGGCCGCAGCACTGCG

>AAUM3455

CTCGCGCGCTGTCCACTGCGCTCGCGGGCGTCCTCCTTTGCGCGGGTGCTCACCGTTCCTTCCACTGCTTGCCTTCCAGAAACCGGGATGGTCGTGCAGCCGCCCCACACGTGGGGGCCTCCGAATGCCAGCCCGCCTACGTTCGAGCCAATCACACAAGGCGGCGGGCTGCTGTACGAAGGATCTGATGCAAAGTTCCAAGTCAAAGTGCACGGGAACCCACCTCCCGAGGTGGTGTTCTCGCGGAGGGGCATGCCGCTGCGCAACGACCCCAGGCGCCAGGTGACCTACGACCCGACGACGGGCATCTGCTGCCTACACATCCGCAACCTGACAGCCGAGGATGATGGCGACTACAACTGCGTCGCCGTCAACTGCGCGGGCGAGGCCTCGCTCACGCTCACCATCCGCGCTGCAGCGGCAGCTGTTATGCGCGGCCAGATGCAACAGATGACGACTGTTCAGAGGACGCAAGTCGTCGACTCGAGGACACATGTGCAAGATGGCGTTTCGCCTATTCACGCCCAGCCTATTCACTACTTTGCTGGTCATGACCAGACTTTCAGAGTGGACACGTTCGAGTACCGGCTCCTGCGTGAAGTCGAGTTCCGGCAGTTCGTGATCGGTCGCGAGGCGGCTCCTCAGCCCGCCATTCCGGGAGCGCCCATTAGCGCTCCCCAGATTCAGGCGCGACCACGCAACTCGAAGCTTCTGGAAGGATCTGATGCCACGTTCACAGTGAAGATCGCTTCCAACCCACCAGCACAGGTGTACTGGTTCCGAAATGGCGAGCTCCTACAAGCATCGCAGAGGGTCATCATGGAGCAGCAGGGCTCGACGTACATCCTGCGCGTCCACATGGCTCTGCCGGAAGACGCCGGCTACTACACAGTCCTCGCAGAAAACTCGCAAGGCCGCGTCGCCTGCTCCGCACACTTGGTGATCGAATCCATGCCTAAGGCCGAGGAGCCACGACAGGCGGACTCGTCCAGTTTCGTGATGACAGAAACGTCCCGAACGCTCAAGCCCAGCTTCGTCAAGATACCGCAGGATCAGGAAGTGACCGAAGGCAAGATGGTGCGCTTCGACCTGCGCGTGTCGGGGCGGCCCTTCCCAGACATCACGTGGCTGCGCAACGGACAGCCCGTCAACGACGACGCCACGCACAAGCTGCTCGTCAACGAGGGCGGCCAGCACGCCCTCATGATCACGGCCGCATCCCGCGAGGACGCCGGCACGTGGACGTGCATCGCCAACAACAAGAGCGGCGAGGTCCGCTTCGAGGTGCACCTCGTGGTGATTGAGAAAGAGCAGGTGGTGGCACCCAAGTTCGTCGAGCGCTTCCAGTCGCTCAACGTGCGCGAGGGCGAGTCCGTGACCCTCCACTGCCGCGCCGTGGCCACGCCGGCACCGCGCATCACCTGGCAGAAGGACGGCCGCCAGGTCCACTCGCAGCCACCCAACCTCATCATTGAGACGCAAGACGGGAGCTCCGCCCTGTACCTGAACCGTGCTAGCGTGGCCGACTCCGCGTGGTACCAGTGCACCGCGCAAAACCAAGCTGGTTCGACAGCGACGAGGGCGCGTCTGCACGTGATAGCTGAACCCAAGAAGCACACCGAGCCGTGGGCGCTTCATCTGCCTAAGCCGACCAAGGTTATTTCACCCGAAAAGTCGCCGCCACGTGAAACCATTTGGCTGCGACATGTGGAGCGCGCCTACCAGCCGGTGCGTGGCCAAGATGACGAGAGGGCGCCCCAGAAGCCGGCCTTCACGACGCACCTTCAGGACCTGGTCCTGCACGAGGGCGAGCGTGGCCACCTCGACGCTAGGCTGATTCCCATCGGAGACGAAACCATGACCATCGAATGGTTCTGTAACGGACGCCCCATCGAAGCGAGCTCACGAGTTATGACCACGTACCGCTTCGGCTACGTGGCGCTGACGCTGCTGCACGTCTATCCTGAGGACTCTGGCGTATACGTCTGTCGTGCAGCCAACGAAGCCGGAGAGGCGACGACCACGGCCACACTTCGATGCACAGCTCGGCCGGCAATCGAACGCAAAGCAATCCAGGAGGACAGTATCAGAGCCATCCAGGAACTGGAAGACGCCGAGAAATACTCGCGACAACTGTCCATCGACGAGACCGCCAGCCTAACAAAGCCTGTCTTCATTCGACCGCTGAGCAACAAAGACAACCTTCTCGAAGGAGCAAACTGCCACTTCGAGGCCCAGCTGACTCCTGTAAACGATTCTACCATGAAGGTAGAATGGTTCCTCAATGGCCAGCCACTGACAACAGGTTCGCGTATCAACAGCACTTTCAGCTTTGGCTACGTCGCCTTGAACATCATGTCACTCCGGTGTGAGGATTCCGGGGTGTACATGTGCAAGGCCACTAACGCCAAGGGAGAGGCTGTTTCCACAGCGACCCTGAGAGTCAAAGCGCAAGGCCTTGTCACAGGAGACCTGGGCATTCCAGAACAGGAGGCCTACATAAGGAAGACGCAGGAGCTGGAAGCGTACCAGATGAGCCTGCACGCGCCAAAGGAAATCATCTTCGAACCGGTTTCCACTATGAAGCCGAACTTCAAGACAGGTCTGCGTGATCAGCTGAACGTTAAGGAGAACAGCACAGTGCACTTCGAGGCTCGCCTCGAGCCAACCGGGGACTCGTCGATGCGCGTCGAATGGTTCAAGGATGGACGTCCGCTTGAAGCAGGCTCTCGAACGACGACTTTCTTCAACTTCGGCTACGTGTCTCTGACGATCCGCGGAGTTGACTCCCGCGACTCTGGCGTGTACACCTGCCAGGCGACCAACTCCATCGGCTCAGCTACTACACAGGCCCGCCTCGTCTGCGTGTCTCAAGCGTCAATTGTGAAGGAGACTCAGCACCCCGGCGGCCTCCAGAAGATTCAGCATCTGGAAGATTCCGCGCACTACCAACGATCCGAACTCGAAGAGGTCTCCATTAAGGAGAAACCCAAGTTCACGCAACCGCTCCAAGGGCCCTCGGCAATAAAGGAAGGCCAGAGTGTTCACCTCGAGACTCGCCTGGAGCCAATGGGTGACGCCAACATGAAGGTTGAGTGGTTCCACAACGGTCGTCCACTGGCCACCGGTCATCGCTTCAAGACGTACTTCGACTTCGGCTACGTCGCTTTGGACATTTTGTACACATTCCCTGAGGACACCGGCACTTTTGAAGTCCGTGCCACCAACAAACTTGGAACGGATGTACTAACCAAGGAGATAACAGTTCAAGCGAAAGCCTCTGTTGACACAAGCACGATTCACGAAGTAGGGCTTGAGAAAATTGAGTACCTTGAGGGCTCTGGAGTTGATCACAGCAGCTACTTCCACATCGAAGAAGTCACAAGGACTAAGCCGTACTTCAAGATTCCCCTTAAAGACCCCAAGGGACCCCTAAAAGAGGGTGATTCCATTGTGTTAGAATGCGCCATCGAGCCTCTTCAGGACCCTACCATGAAGGTGGAGTGGTTCTTTAATGGTCGCCCGCTGCCGACAGGTCATCGGTTCATTACCAAGTATGACTTTGGACGAGCATTTCTTCAAATCTTGAACCTGGTGCCCGAAGATACCGGGCAGTACACTCTGCGAGCGACCAATCATCTTGGATCGGCGCATTCAACGTCTTGCATCAAGGTTATCGGCAAGTCCGGCGTGATCACCGAGTCGCAGTACCCCGAAGGCTGGGAGAAGATCCAGCACCTGGAAGACCACTCGCGTTACGCCCGCCAGGAGCACGTGGAAAGTGTCATCACACAGAAGCCCACCTTCAGCCGGCCGCTGCACAACGTGGAGACCGTGGAGGGAGCCAACGTGCACCTCGAGTGCCGGCTGCAGCCTGTCGGGGACCCCACCATGCGGGTCGAGTGGTTCCGCAACAGTGTGCCCATCAAAGTCGGCCATCGATTCAGGCCAGCGCACGAGTTTGACTACGTCGCCTTGGACATCCTCAGCTTCTATCCCGAGGACTCTGGCATCTACACGTGCAAGGCCACCAGCTCTCTCGGTGAAAATGTGACGTCCTGCAACGTGAACTGCTTTGCCAAGTCGCAGCTCATTCTGGAGTCTCAGCACCCTGAAGGCCTTCAGAAAATACAGCAGTTGGAAGACCAATCTCGCTACCGCCGCGAGATAATTGAGGAGACAACCGTGAAGACCAAGCCCTCCTTCACATCTAACATGACGGCTCTGAGCCTCCGCGAAGGACAGAACGCCCACCTGGAGTGCCGACTTGAGCCTGTCAACGACGCTGACCTCAGGGTTGAATGGTTCCGCAACGGCGTCTCTCTCCCCATCGGTCATCGGTACAGGCCATTCCATGACTTTGGCTACGTCGCACTGAACATTCTGTCCCTTGTTCCTGAGGACTCGGGAACCTACACCGTGCGTGCCACCAATTCTCTTGGCAAGGCCGAGCTGTCCACGACAATTAATGTCGAAGGAAAGTCTTCCATTGACACGGACACTCAACACCCTGAGGGACTGCAAAAGATCCAGGCCCTCGAAGGCCACCACTATGAGAGGGACACTGACGATCTGGACCAGTCAGTTACGACAGCGCCCGTATTCACATCAGCTCCCAAATCCATCGTGGTCCAAGAGGGACAGAAGGCGCATCTGGAGTGCCGCCTGATCCCCGTGGGAGATACCAAGCTGAAGGTCGAGTGGTTCAAGAACGGCCAGCCGGTGCCAGCAGGATCTCGTTTCGTCGAGATGTGCAACTTCGGTTTCGTGTCTCTGGACATCCTCAACACCTACGCTGAAGACTCCGGCACCTACACCTGCAAAGCCACGAACCAGCTGGGAGAAGCTGTCGTGTCTGCTCAACTCAAGTGCCACGCCGAAAAGTCGTTGATACTGGACACGCAAAACCAGGAAGCGTACGAAAAGATCCAGCAACTCGAGGACTACGGACGCCAAGCAAGGCCCGCCTATGTCGTGGAAGAGATCACCACGCAAGCGCCCGTGTTTACTCAAGCTATGAAGAACCTGAGCCTCAACGAAAACCAGAGTGCTCACTTTGAGTGTAAGCTCATACCAGTGGGCGACCCCAACCTCAAAGTGGAATGGTTCCACAACGGACTCCCCATACAGAAAGCCAACCGGGTCAACACCATTCATGACTTCGGATTCGTCGCTCTTGACCTGAGCTACGTCAAAGCACAGGACTCGGGCACTTACACTTGCAAGGCAACCAACTCCCTGGGATCTGCCGTTTGTTCTGCCACTCTCAACGTCCAAGATTCCAAGTCGCTGGTGTTCGACACTCAACACCCAGAGGGACTTCAGAAGATTCAACAGCTCGAGGAACTAGGACGTTACAAGCCAGAAGTGACGCAGGAAGCCCCGTGCCCGGGCCCACCGATGTTCGTCACCCAGCTCCAGGGCCCGAGCCGTCTGACAGAAGGCGAAAGTGCGCACCTGGAATGTCGCATTGCGCCCTACCCCGATGCCACTATGAAAGTGCAGTGGTTCCACAATGGCGTCGAGCTGCAGTCCGGTCATCGTTACCGGACGATGTACGACTTCGGCTTCTGCGCCTTGGACATTCTGTCAGCGAATGCTGAGCACTCCGGCGAGTACGTGGTCCAGGCCACGAATGAGCTTGGCACCGCGAGGTCCACGGCGCGCATTCATGTTGACGCCAAGGGTGGCGTCATCCTCGAAAGCCAGCAGCCAGACGCCCTCCCGAAGATCAAGCAGCTCGAGGAATCATGCGGCTACATCAGGCCAACGCAGGAAGAAGTGGTGATCAAGGACAAGCCTAACTTTGTTCGGGGTCTTTACAACCTGGAAACTCTGCATGAAGGCCAAAGCGCTCACCTTGAGGCTACGCTGACACCAATCAACGACGCTAACATGAAGATTCAGTGGTTCCACAACGGCGTAGAAATTCCGCTTGGCCACAGGTTCAAGACTGTTTCTGACTTTGGCTACGTGGCTCTCAACATCCTCTACGCCTACCCGGAGGATTCTGGCACATACATGTGCAAGGCAACCAATCAGCTCGGCGAAGCCGTTACCACGTGCTCAATCAACGTTCTCGGAAAGTCCGCCATTGTAACCGACACCTACCACGAAAAGGGCCTGGAGAAAATCAGGCAACTTGAAGAGTACCAGGCGCCGGAGAAGCCTGAACAGGTGATCCAGCTGCAAAGGCCCGTGTTCACGGTGCCTCTGAACTCGCTGGACGGTCTTGTGGAAGGACAGAGCGCTCACTTGGAGTGCAGACTCGAGCCCATCAATGACGCTGATCTCAAAGTTCAGTGGTACGTCAACGGCGTGGAAATCCGGCCAGGACATCGGTTCAGGACGACGCACGACTTTGGATATGTGGCCCTCGACATTATGTATGTGTATGCCGAAGATAGCGGAACCTACATGTGCAAGGCAACCAACTCTCTCGGAGAAGCGGTTACAACGTGTAACCTGCGTGCCCTACCCAAGCAAAAGATCTTCTATGATACGCATCATCCGGAGGGTCTTGAGAAAATCCGGGAGCTCGAAGCGCAAGTGAAGTACCAGTCTGCCGAAATTCAGGAGAAGCCGATCTCAAAGCCTGTGTTCATCACTGAACTGAGGGGAACCCAGGAGATCTCGGAGGGTGAAAGCGCCCACTTGGAGTGTCGCGTGGAACCAGCCCACGACGCTAAGTTGAAGATTGATATCCTTCACAACGGACGGCCGCTTACAGCTGCGACGCGCGTTCACATCACCAGCGACTTCGGCTACGTGGCGATAGACGCGACAAGCGCCATTCCAGAGGATTCCGGCACTTACACGGTGCGCGCCACTAACGACCTAGGAACTGCCGAGACAACAGCCACGCTGCGGGTCCTGCCAAAATCCAGCATCATCTCCGACACCCAGCACCCAGAAGGACTGGCTAAGATCAGGGAGATGGAAGACGAGTCTCGTTTCAAGCGTGAAGTCATCCAGGAGCCAGTCACCTACCAGACGCCCGTGTTCACGGTTCCTCTGCAGAACCTGGAGAACCTCGTTGAAGACCAGAGAAACGTCCACCTGGAGTGCAGGCTGATCCCAGTCGGAGATCCTACCCTCAATGTCCAGTGGTTCTTCAACGACACGCCACTGATGGAAGGCACGAGGTTCCATCCCGTTCACGACTTTGGCTACGTTGCACTCGACATGGACTACGTGCGCCCGGAGGACACTGGCGTCTACACGTGCAAGGCCACCAATTCCCTGGGCCAGGCAGTGACCACCTGCATGCTCAAGGTTAAACCCAAGGCGTCCATTCTTCTGGACACCCTGCAACCTCAAGGCTACGAGAAGATCCGTGAGCTGGAAGACTTGAAGGGCCAGAAGCCTCCGGAGAAACCAGACGCAGTGTACGAAAAGCCAGTGTTCACCAGCCACTTGGTTGGTCCTGGCGAGATAAACGAGGGACAGCCTGCACGTCTGGAGTGCCGCTGTGTCCCTGTCGGAGACCCTGACCTCAAGTTCTACTGGTACGTCAACGGCATCGAGCTTCCAAAAGGTTCGAGGTTGATTCCCAACAACGACTTTGGATTTGTGACCCTGGACATCCTGTCGGGCATCGCGGAAGACTCTGGAGTCTACATGTGCAAAGCGGTGAACAAGGCAGGAGAAGCCGTCACTTCAACTTCGCTGCGAGTGAAAGGCCGAGCTGGTGTGCTGCTGGACTCCCACCATCCCGAGGCGTACAGGCAGACGCAGAAGTTCGAGTACGACTCCAGCCGCATTCCTGAGAAGTGGTCCGACGAGAAGCCCAAGGCGGCGCCAGTGTTTGTGCAGCACTTGAACAACATCGATGGCGCTGTTGAAGGCCACTACCTGCGCATCGAAGGGCGCATTGAACCTACCAACGACGACAAGCTCAAAGTCAAATGGTTCAAGAACGGCAAGCCTCTGGTCATGGGTACTCGTATCAAGCCTACGGACGATTTCGGCTTGGTTTCGTTGGACATTTCCAGCGCTAGACCGGACGATTCTGGAATTTACACCTGTAAGGCCACCAACGATGTTGGCGAAGCTATATCGACGTGCACAATCAAGGTTGAAGGCCGCGAGAACATCATCCTGGCTTCCCAGCACCCCGACTCGTTGCCCAAGATCCGCCAGCTGGAAGAGTATGTGCCGCCTGAGAAGTACGTGCCTGAACCAGATTACGAAGGGCCCGTGTTCGTCACGCACCTCAACAACCTGGAGATCCGCGAGGGTGCGACTGCGCACTTCGAGTGCCGAGTGGAGCCATCCAAGGATCCCACGCTCAAGGTGGAATTCCTCAAGAACAACAAGCCAGTGCCTGCTGGTTCAAAGTACAACTTCAACAACGACTTTGGCTTCGTCACACTGGATGTCAGCAACGCATACCCGGAGGACGCCGGCATCTACACGTGCCGAGCCCGCAACGCGAAGGGCGAAGCGGTCACTACCGGCTCTCTCAAAGTCCAAGGAAAAAGCGGCGTGCTGTCCGACACTCTTCACCCAATGGGCGCACAGGGACTGTCCAAGGTCCAGGAGCTAGAGACTTCCTACTTGACCAGATACCAGGCTCCCGTCGAAGAGGCTGAGAAGGTGTTCCCGCGCCCGGTGTTCGTGGTGCCCCTTGAGCCAAACTTCTCCATCCAAGAGGGTAGCCCTGTCACCCTCGAGTGCAAGGTGGAACCAGCAAGCGACCCGAAGCTCAGGGTCGAGTGGTTCCTGAACGGCAAACCACTTGCGCCAGGATCCCGGCACACCGTCACACACGACTTCGGCTTCGTTGTGCTGGCCATGACCGACTTCTGGGGACGAGACGCCGGCGTTTACACCTGCCGCGCCAGCAACGCCGCAGGCGAAGCCTTTACCACAACCACAATCACGTGCCTGACCCGCAAGGGCGTGCAGGAGGACACGCTGCACCCGGAGGGCCGCAAGGGCCTCGAGTCCATCCAGCACCTGGAGGAGTCGCTGACGCGCGTGCCCGAAGCGATCCAGGAGGAAGCCGCTGGCCAGCCGCCAGTCTTCACATCTCAGTTCGTCAACCTCAAGGACCTGAACGAGGGCGAGATCGCCCACTTCGAGGCCACGCTGACGCCAGTCGGCGACCAGACCATGCAGGTGGAATGGTTCTTCCGCGGCAAACCCCTCAAAGCAGGGCACCGCATCCGGACGGTCCACGCGTTCGGCATGGTGGTGCTCGAGATCTTGGGGACTGTGCTGGAGGACTCTGGCCGCTACACTTGCCGTGCGACCAACAAGTGGGGCAAGGCCGAGGTCACCGTCGACCTCGAGTGCACCGACAAGACCAAGGGACAGCGGCCGCAATTCACCACGCAGCTGCAGAACCTCATGGACCTCAAGGAAGGAAACAGCGCTCACCTCGAATGCCACCTGGTGCCTGTTGGCGACCCAGACATGAAGGTCGAGTGGTACAAAAACTCGCAGCCACTTCGCGACAGTTCTCGCATCAAGACACTCAGCGACTTCGGCTACGTGGTGATGGACATCTCATTCGTGCACGCCGAAGACTCTGGAGACTACGTGTGCGTGGCCACCAACAAATACGGCTCCGACGCCACTAAGTGCACCATTCAGTGCGCAGGCACTGGCAAGATATTCAGGGACTCGCTACAGCCGCAATCCCTTGACAGGATCGCTGAACTGGAAGGTGCTTCGGCTCTCACCAGGACATCGGCCGTGATGGAGGCGACCCGGCTTCAGCCGCCCAAGTTCCTGTCACAGCTGAACAACATCACAAACCTGGTCGAAGGCCAGAGCGCACACTTCGAGTGCCAGCTGGTGCCGGTCAACGACCCAGACCTCACGGTTGAGTGGTACTTCAATGGCCAGCTGCTGCGCTCAGGACATCGTTTCCGGACGTTCCACGATTTCGGCATCGTTATCCTGGACATCCTGTACTGCTACGGAGAGGACTCCGGCGAATGGGTCTGCAAGGCCACGAACAAGCTCGGCTCAGATGTGACCCGCGCGACACTACAGTGCAAGTCCAAGAGCTCCTTGATCCTGACTCCTCAAGTGCCCCCTGAAATGGCCTCGGCGACACAGAACATCATCGCTCTCGAAGAAAGCCTGTACCGCACTGCGGCGGTCATTGAGCCCGAGGGACCCGCTGAGGCGCCTCGTTTCACCGTACCGCTCACTAACGTTGAGGATCTGAGGGAGGGAGACAACGCACATCTGGAAGCCCGGCTGACGCCTACAGATGATCCAGACCTGACTGTGGAATGGTTCAAGAACAACATGCCGCTGATGTCAGGAACAAGAATCCGAACCATCAACGACTTCGGCTTCGTGGTGCTCGAAATGAGCCCTGTGTACCCGGAGGACTCCGGCGTCTACTCCTGCCGTGCTAGAAACCGTTTCGGCGAGGCCGTCACCACATGCACACTCAAGTGTCAAGGAAAGCGTAGCATCATTCTCGAGACACAGCTGCCAGAGTCAATGACCACAGGCATCGAGAAGATTGCCAAGTTCGAAGAAGTCTCCTCGGCTAGGATCGACGAGAAGTGGACGGACAAGGACACCTCTCAGCCGCCCAAGTTCATCACCACGCCGCAGGACCTGACGCTGGCAGAGAACTCTCTGGCTCATTTCGAGTGCAGGTTGACGCCAGTCGGTGACCCTACGCTGAGAGTTGACTGGTATCACAATGGCAAGCCACTTGTCACGGGCTCTCGAGTCAAGACAATCAGCGACTTTGGATACGTCATTCTGGAGGTGGCCGGTGTTTACCCACGAGATTCGGGTGTTTACACATGCAGAGCCGTCAACAAGGTTGGCGAAGCGTCGGTCTCCTGCAAGCTCGCCGTAAAGGGCAAGCAGTCAGTCGTGATGGAACCACAGCTACCTCAAGAATTCCGATCTGGGTATGAAAGCATTCAGAAGTTGGAAGAGTCGATGTACCGAACTGAAGAGAAGATCTATGACGATGATAAGAAGGAACCACCGAAGTTTGTCACGCAGATCCAATCGCTCCTCGATAAGGTGGAAGGCGACAGCGCTCACTTCGAATGCAAGCTTATCCCAGTCGGAGATCCCAACCTGAAAGTGGAATGGTTCTTGAACGGACGTCCCCTTGTTACGGGTACGCGTGTTCACACCATCGATGACTTCGGCTTCGTGGTGCTGGACATCGACTGGCTCTTCCCCCGCGACTCTGGAGAGTATATGTGTCGTGCCACGAACCGTTGGGGCTCCGACACGACCAAGGCAACCCTAAAGATTAAAGCCAAGAAAGACATCATCATGGACAGCCAGCTACCAGAGGGCATGAACGTTGACAAGCTGCGCGACCTGGAGTACCCGACGCCACAAGAAGAGACAATTCAGGAGCAGGAACCGGTCAAGCCCAGGTTCATCACCCAGATACAGCCTCAGCAAAACCTTAATGAGGGCGACTCGGCTCACTTCGAATGCCGCCTGGAGCCCATCAACGATCCAAAGCTGAGGGTGGAATGGTACCACAACGGACAACCCTTGAGATCAGGTCACCGGTTCAAGACAACGCACGATTTCGGCTTCGTTGCATTGGACGTGCTATACGTGTACCCTGAGGACTCGGGCACATACGTTGCACGGGCCGTCAACGACGTTGGCGAGGACCAAACTCAGGCCACCCTCAGGTGCACAGCGAAGCCAAAGCTGGACTACAGGACCCAACTTCCAAAGGACATGAAGGATGGTGTTAAGAAGATCGCTGAAATGGAAGCCTCCTGGCAGCGCGCTGAGACCCAGGAAGAAGTGGAAGAGGAACCATGCGCTCCCATGTTCATTATGAAGCCAGAGCCTCAAGTGGTCATCGAGGGCGAATGGGCAAAGTTCCAGTGCCGAGTTATTGGCCACCCCAAGCCAAGGCTCATCTGGGTTCTCAACGGCCACACGGTCATCGCCGGCTCAAGGTACAAGCTGACTTACGACGGTATCTACCATCTTGACATCCCTAAGACCCGCCAGTATGACCAAGGAAAAGTAGAAGTTTTTGCAAGGAACTTCTGCGGCGAAGCCTACTGCTTCACAACTCTTGAAGTCCGGCCCAAGTTCGATGACTACAGAGCCGTGCTCAAGCATTCTCCTAAGCCATGGTACGACCAAGACGTCAAGTCTTATCAAAAGTACCGACATGAGACTGAACTGCAACGAGTGTTTGAGGAGAAGCTTACACCTGGAGGCACTCGTATCGATGTCTGGAAGACTGAACAGGGCCAGCAAGGTGAACACCAAAAGATCAAGAAGAGGATCGAAGAAGAAGAGCTGGAGAAGCTCAAGCCGAAAGTGGAGCGCTTCAAGACCGACTCGATTTATTACGATGCGCGCACGGGCGAGAAGAAGGTGGAGACGGGCTCCCAGGCGCAGTACATGGCCAAGTACTTCGAGACCGAGGCCGAGAAGCAGCAGCGCGGCGCTACCGGCATTTCGCCCGAGTCCGTGGTGCAGGGCCGCGAGGTTCACACCACCACCCAGCGGCAGACTCAGAAGGAGCAGCAGGGCGACCTCGAGATCACCCGCAAGAAGACGCTCACCGAGACGCTCGAGCAAGAGCACAAGGGCGTCACCAAGGAGCAGCGCGTCCAGGGGCCCGCGCAAGAGCCGGCGAAGGCTCCCGTGTTCACCAAGAAGCTGCAGCCGTGCCGCGTGGACGAAGGCCGAGGCGCCAAGTTCCAGTGCACCTTCACCGGACAGCCAGCGCCCAAGATCACCTGGTACCGCGAGAACTTCCCCATCCAGCCCTCGCAGGATTTCCAGATCGTGACAACTGACAGCACGTCGACGCTGATCATCCGCGAAGTGTATGTGGAAGATTCCGGCGTCTTCTCAGTGAAGGCTGAAAACCGTGGCGGCTCAGCCAAGTCCTCCGCCAACTTGGTTGTTGAAGAGAGGCGAGAGCAACGCAGCGGTGTCGTGCCGCCAAACTTCACCCGGACCATCCAGGACGTCTCGTCGAAAGCCGGAAAGCTGGTCCGCCTCGACGCAAAGGTCTCAGGATCGAAGCCGTTGGATGTCTACTGGCTCAAGAACGGCAAGAAAGTGACGCCCGACGTGTCGCACAAAATCGTGGAGGAGGACGACCAGTACACGCTGCTCATTTTGGAGGCCCAGGCGGACTCCGACTCCGGAAGCTACGAGTGTGTCGCCATCAACTCGGCGGGAGAAGCCCGCTGCCAGGCTCACGTGGTGATCGAAGGCGCCAAGCCCAAGACGCCGCCCACGAGCCCCAAGGAGGCGCCCGGAGACCAGAAGCCACCCACGGTGACAGAGCCCCTCAAGCCACTCGCCGTCAAGGAAGGCCAGAGCGCCGTCTTCCGCTGCCGAATACCCGCCGTCCCGGGGGCGCAAGTAAAATGGTTCCGAGGAGACCAGCAAGTGAAGCAGTCGCGGTACTTCCGCATGTCCCAAGAGAATAACCTCTTCACGCTCAAGATCTCCGAAGCGTTCCCGGAGGACGAAGGTGTCTACAAGTGCGTCGCCACAAACCCGGCGGGCACTGTCTCTACCAGCGCCAACCTTAAAGTGATTGTGCCCGAACTGAACGAGGTGCCGCCGACGGTCACTCCTCTGGCCGACCTGACCGTGCCCGAAGGCTCGCCCGCCCGTTTCGTCACATCCCTGGGAGGCGTGCCACCGCCCAAGGTCATCTGGGTGCGCGAGGGCCACATCATCAAGCAGTCCCGTGACTTTCAGATGAACCAAGACCAAGGCTCAGCGTCTCTGGTCATCAGGCACACGTACCCCGAAGACGAAGGCGTCTACGTCTGTCGTGCCACCAACGCCTCGGGCCAAGCCGAGACCTCTGCCCGGCTTACCGTGCAACGTAAGGCCAAAAAGTAGAGGTCGCGCCCTGGCCTACTTGCTCGCTGCGGCTTATAGCGCTCCATTCGAAAAGCTGCGCGTACAAGCTGCTCAAACCGAGAGAGAGAGAGAACAAAAACAGCAACAAAAAAAGTTTGGCTCCTCACTTTCGCGTTCTCTAACTACAGCGCAGTAATAACGAGATATAATGTATCTTGCGTCTTAGCGGTGACGCATAGAATCTATTTTCAATTAATTTATTTATTAATTTATTTATTTCTGGAGAGAGTCTCTTTTGCATATTTTTGGTTTACAGTCACTTAACCTTCCCTCTTTTTGTTGAGAGCAAAAAATTTCGAATCGGAGTCCGTTCTTTACATTGCGTTCGCTTTCGTTTACTCCTTGTTCCTGTTTTGGTTTAAAACATTTTGTAAAGAAAAACAACTAATGTACGTACAGGGAATGTTCATTTCGCCCCCTTTTTTATACGATGAAATCGCGACATTCATGCCTAACAGGTTTATTCGTTTCATTAACCAAGTTCTGCCTAACAGAAGGGTGAAGTTTTGTATATTACTGACATTTCTATATTTACTCACGAGTTTTTTTTTTCCTTCGTTTGCAACAAGCGTTGCAGGACTGCACATCTCGGTCAGTTAGCGGCACTGAAGCGCTCGAACAAAGCGCGCACAACGCCACCGTAGGGCGCCACTATAGATGTGCATTCGTGAGGAGCCTTACAATTTATTGCATATTGGGGCTATTAACACCATCGCTTATTACTCCTATTCGAACCGATTGGCCACCATCGCGGGTTTCATCATGGCGTTAGGACCGCGAAATAATCGGCACTGACTGCCACAAGACGAGTTAGACAGGTTTAGGCGATTAACACATTTAACCATCCGGTCGAGAAGGCGACCTCACACTGCCAGCCGCGTTTGATATGTGATAACGCTAGCTGCTTGTTTTTAAACTCTCTCGGGCGCCCCTTGTTTGCGTTTTGTTTTGTTCCTCGCGGTGAGCCGTTCGCAATTCGCCACGCTAGTCACTACAGCAGCTGACGCCGTCGACGCCGCAAAGGCGTGCGCGCGACCCCGGCTGTAGCTTATCCAGGCGATATGTGTAGGCGAACGCGCTCCGTCCTAGACCTCCAAGTGGCCGAAACCTGTAGCGATTGCTTGTGGAGAAAAATTTCAATAAAGCCAAGTTAT

>AAUM26361

GCGATCTTGAGAGGCGTCCTGTTCCCTTCGTTGTATATTTGGAAATCTCTAGATGACTTGATAACTTTCCCATTCAGGTACCAAGTGAATGAGACAACTTCGGGGAAAGATGTAACCTCTCCAACGAACGTGACTGGCGATCCTTCAACGACTGTTTGTGAGACCAAGTTTCTAGTGAAGACCGGCGGCTTTAGTTGGGTGAGGCTTTCTGGCGGCGAAGTTGGTCTTTCTTCCGTCGGCTCAGCGCCAGGTTGTACTTTACTCAACGGTGATGTTGGAAGCGACTGTTCCTGTATAACGCGGAGGTTAGCGGACGTTGTTGCCCGTCCAGCAGGGTTCTGCGCCCTGCACTCGTAGGGCCCAGCGTCTTCGGGGAACAATTCGGCAATCACAAGAGAGCTCTTGAGGCCTTCAGAGGTCACTAGGAAGTCCCTTGATGACTTCAGCATCTTGCCCTCAAAGAACCAGTGAAAGTCGGGGTTGGGCACGGCGTGAACTTCGAC

>AAUM30547

GCTGTTGCTGGCCGCCGCCTTCGGGATCAGTAGCTTCACAGTTCCGTCCGGATCGTGGCTGACCTGGAAGTCTGGGCTTGGAAGCACCTCTCTTCCATCCTTAAACCAGCGGACGAAAGGTGGCTCGGGTGCCCATAGCTTTGCAGTGAGCACCACGCGGCAGCCCTCATCCGTGGTCACGTCTCGGAGCGGCTCCAGAAACACGGGAGCAGAAAGCGGCACGCGCATGTTGCACTCCGTGTAGCAGCTGCCCATGGGGTTGACCGCCGAGCATCGGTAGGTGCCGGCGTTCTGGGCGCTGACGCCGTTCAGGACAAGAGAGCACTTGTCCCCGCGGAACAGCAGCCGTACCCGCTCAGACTCCTTGATGGGTTGGGTGTTGTGGTACCAGATGACCTCTGGTTCGGGCCGGCCGACGATGATGCATTCCAGCGTGATAGGGTCCGGTCCAGGCTTTACTTCCAGATCCTCCAGGGCCTTGAGCACACGCGGAGGCTCGGGCGGAGCGGCGTCCTCGGCCACTTTCACTTGGCAGGAGCAGGCCGTCTCTCCGGCTTTGTTTTTGGCCACGGCGGTGTATGTGCCCGAGTCGCTCTCGGTTGCG

>AAUM16482

AAATGTAGATGGCAAATGGCAAGTTCTTTTTACATGAAGCCAGGTAGAAGCGCGCGCTTCACCCAGCGGGTTCTCCGCCACACACTGGTACACCCCGGCATCTTCCATAGCCAGGGCTCCAATCTCGAGGCTGCCGCCGTCACCAACGTGGAAGTGCCCTGTGCTCTGGTCCACAGGCCGCGCATCCAGGAGCCAGTGCACCTGGGGCTCTGGGTGGCCCTCAGCCACGCAGGGAAGCCGCACCTGCTGCCCCAGTTCCACTGCCGTCTCCTCATTCACCTTCTGGCTCAAGGAGGGCAGCACTGACACAGTGACGTTGGCATGGGCCACGACAGCAGAGGAGTCCTCAGGGGCATGTCGAAGGGTGACCTGGCAGGAGTAGCGGCCAGTGTGCCCGGGCCCCAGGCGCAGCAGTGTCAGGGTGCGGTTCCAGTGGGTGAGAACGTGTGGCAGCTCACCCAGGGCCCTACCATCCTTCAGCCACTCAATCTGCACCTGGTCCAGAGGCCTGCCAGAGGCAATGCACTCGAGAGTGCTGTCATAGCCATTGCCAAGGTTGTTGAACTCTCGATCTGATGGCGGAACAACAATGGAGAGTTCTGCCTGGTCTTCTTCATTGTCATCGACGGTCAGCTCCACCACTGGTCCCGAGAGGTTGTCTCCCGTGTGCGGATTGGTCAGTTCCACCCGGTACTGGCCGGCATCCTTGGGGCTGACGTCAAGAATGACCAGGCGGTTATCCTGTGTTTGTGCAAAGTTTCTGCTGTCCAAGTTTCTGCCGTCCAGCCGTGTCCAGACCGCAGTCGCTGGCGGAACACTGTCAACAAGCGGCGGTGCCAAGATCACGTCGCCACCCTTGCGAGCGTTCACAGGAACTGTTTCAGGGACTTCTTCGGCTCGTGTTAGGTGTGCCACCCGTAGCCGGGCCTTGGCGCTGAGCAGCGCGCCAGCTGCATTCTCGGCCAGGCACTGGTAGGACCCTGCTTGGGAGTGGCTAATACTGCTCAGCACGTGCACCAGGCGCTGTGTGGTCCAGTTGGTCAGGGGCTCCCCGTCCTTGAGCAGCTGCAGGCGTGGG

>AAUM37690

CTGGCCCTCACGCCACCAGCGGTACTCAGGACGGGGCCACCCAGGTGGCCGAGCACCACAAGTCAGCACAAAAGGACGACCAGCCACTGCTGTGGGGCCCTGAGGAGTAATCTCTGCTTCTCCTGGTTTGTGTCGCACGTGCAACACAACAGAGTCGTTTCCAACAACTTCGGTGTGGATGGCAGTGGCAGAAGGTCGCATGATGACTGTGGCCTGACAAACGTAGGTTCCGGCATCCTCAGCAGTGACACGATCAAGGCGCAGAGTGTCTCCTTGAAGTGCAAACTGGGGGTCACCTTCCTTGGTCCACAATACAGAATGTGGCTCAGGGTTAGAAGCAACATTGCATTTTATGGCTATATTTTCTCCTTGAGAGGATTCCTGCTCTGGAAGCACGTTCACACGAGGCCCATACAGCACACTAAGACGCAGGTCGGCTTGAGTGGGCT

>AAUM64929

GAGCGCTCCCGTGCTGCTGGCAGCCCCGTGCTGGTTCTTCACGTAGCAGGTGTAGTTGCCTTCATCCTCCCGCCGCACCGGCTGAATCCGCAGGAACCCATTGCTCAGCAGCTGCACGCGACCGCCCATAGGAACACTGGAGCCGTCTCGTCGCCACGTGAACTCAGGAAATGGTGCAGCTTCGGGGCGACACGGTATGGTGACATTGCCTTCCTCAGCGGCATACGTTTCGGAATCCAGGGGATACTTCGCAAAGTTGGGAGCCATGGTGACAACACGCAGCTGCGCTGTGGAAAAGCTGGTGCCCAGGGAGTTGGTGGCTGAGCACTGGTACATGCCTTCATCAGCT

>AAUM52236

CTCGGCATGTATAGAGGCCCTGGTCGTCTGGATAAATCTCCGGGATGGTAAGCGAGACTAAGCCAGTCAGCGGATCGTAGTGCATCTGGAAGTCTTTCGTATCCTTGACTTCTCGTCTGTTCTGAAACCATTTGATATGTATAGGCTGGCTGCCGACTACTGTGCAGCGGAGAATCGCAGGAGAACCATCTGTAGCGGCCACAGGCTCCAACTGTTCGATGAATCTCGGGGGCTCTTTGCCTCCTTTGACCTCCAAGGTAACTTGGATGCTCTCGGCGCGACCCCAGCAGTTCGTAGCAACGCACGTATATTTTCCCGCGTCGTCAACGGTGACGTTGTTGATGGTGAGCGTAGTGGTGACCTCTTCTTGAGACGTCGGCGTCGTG

>AAUM55603

CGGTGTTCTTTTTTCCATCGAATGACCGGCACCGGGAAACCATCCGCCTGGCAGTCAAAGCTCACTGCTTGACCCATGATGGCCGCCTTGTCCACTGGCTCGTGACTCCATGAGGGAGCAACGCGAACAACCATGGGCGCGGTGAAGTTGGCCGAGGCTGCCGGGTTGGAGGCGACGCAGGTGTAGTTGCCAGAGTGCATCCGGCGCACGCCGGTGAAGGAGAGGAAAGACGTGTAGTCGTTGGCCTTGGCCACCGAGGCCTCCAGGGCGGGGTCCAGCTCCCGGCCGTCCTTGAACCACTCGATGGTGATGGGCAGGTCGCCGTCCGAGATGATGCAGGCGGCGCCCGCACGCTTCCCTTCCGTTAGGTCGTCCGGGAAACTGAACGGGCTCACCACTGGTGGAG

>AAUM59262

CAACTATCCTGCCGAGTGGAAGCAGCCCATCCAGGACACGGCCTACTCAAGCCCTGGCGCCATGGCCGTGCTCCACTGCGAGGCCGCAGGCATGCCCGAGCCTAGCGTGCGCTGGTTCCGCAACGACCATCTCCTGGAGCCCGACGAGAGCCGCAATATTGTTGGGGACGTGGGCACCAGCACGCTCAAGATCAAAGTGGCTGACGTATCTGACTTTGGGGAGTACATGTGCCGAGCTCGCAATGACCTTGGAGTCCTGGAGCATGTCATCACTCTCAAAGAAGGAGAGGCGCCCAAGGCTCCACGAGTGGCCGTGATCGACTCCAAGCCGAACAGGCTGGTGTGGAAGATCGAGCACCTGCGCAATGAACCTCTCGAGGTGGTGGGCTTTCGTGTCGAGTACAAGACAGACAAGGATTCCAGCTGGGACAGTGCAGAGCACCAGGAATATGGTACCGAAAATGGCATGCAGTATGTGCTGGGGAACCTGAACCACGACACCAGCTACGCCATTCGTGTTTCAGCGAAAAATGCGGCGGGCTATGGTGACTTCACAGAAGAGATCTACCACCGGACCAAGGTGCCGCAACACTACACGCAACACAACACCAACGCCGCTGTCGGCGTAGCCCAAATTGCGCCCGTCCTTGCCTCCTTAAACTGCCTGCTCCTCCTCAGGCTTTAACGAGCCCAGCGGTACTCACAGTGGGACGG

>AAUM61335

GTTTGATTGCTGCGTCGACGTAGCCTCCCAGTGCTTCTTGACTTGTAACTCCCTGGAAGACGCCACTGTCGAAGACGTCGGCCGCGGTATCTCCTGGAGCTTGATGTTGGCCTCGGTCTCTCCGAGGGGGTTCTTGGAGACGCACTTGTAGGTGCCGAAGTCTTCGGGCTTGAGGTCGCGCACGGTGAGTCGCAGCAGCAGGCGGTAGGGCCCCGTCTCGAGCACCAGCGAGTCATGCTTGGCGTTCGAGATGAGCATAGTGCCGTCGTCGCGCTCCCAGAAAGTCATGCCGCGCGGGTGCGTCTCCAGGTTGCAC

>AAUM60898

CGGCTGACAACGTCTCATACGAAGGCGAAGAGCTGAGCATCACTAAGGTGAGCCGTCTTCACATGGGGCCCTACCTGTGCATTGCCTCCAACGGCGTCCCATCACCCGTCAGCCGGAGGATTCTGCTCCAAGTCCACTTTCCGCCTATGATCTGGATACCGAACCAGCTCATCGGAGCTCCTCTTGGCGGTGAAGTGGTGATGGTGTGCAACACAGAGGCCTATCCGATATCGATCAACTACTGGACACTGGAGAGCGGCGATCTCATCGCCGAGTCGGCCAAGTACTCTCTGAACCGCACCGAGAACGTCTACAAGGTCCACATGAGGCTGCGGATAAGGCGCATTGGTCCCGAAGACTTCGGCGCTTATCGATGCTTCGCCAAGAACTCCCTGGGCTCCACAGAGGGATCCATACGACTATACGAGATTCACGTG

>AAUM53366

GCCCAACGTCCCAACACACCGGCTCCACGTGACTGCAGGCGGGGGCCGGCCCGCTGCCGAACACCTGAGATGCACCTCGGAGCCTTCGGCAGCCGAGACATCTTGGGGTTCGTCAATCCATTGTGAGGGCTGTTTCACTTCCAGCGTCGACGTGTACCGGTCCGTCCCCGCTGCGTTGCTCACCACGCAGGTGTAGTTTCCGGCGCTCTCGGCCGTCACCGGGTCAATGCTGAGCGCCGAGAAGTCGTCCTGCGACGATATCCCTTTGCGCACAGGGGCGCCGTCCTTGATCCAGGCGAACGTCAGAGGCCGCGTTCCG

>AAFF40516

CTCAACCTCACCTGTAGAGTCGCTTCTAAAGAAAGGTACCGCATAACATGGGTGATCCCTCAAGACAAGGTGCGCCAAGCGCACCGAATCAAGATCGAGGAGGTACACTTCAACGAAAGGACGGTGACCATCTATCACCTGGAAGAGCCTGATTCCGGCTGGTACACATGTGTTGCCCGTCACCACGATCCCTCCGTCAACCGAGTGTTCAACAGAACAGTCCACGTGTTGGTTTCAGCTTACACCTTGTCTGGCAGGCGGCC

>AAFF2494

CTGAGCCTCCGCGAAGGACAGAACGCCCACCTGGAGTGCCGACTTGAGCCTGTCAACGACGCTGACCTCAGGGTTGAATGGTTCCGCAACGGCGTCTCTCTCCCCATCGGTCATCGGTACAGGCCATTCCATGACTTTGGCTACGTAGCACTGAACATTCTGTCCCTTGTTCCTGAGGACTCGGGAACCTACACCGTGCGTGCCACCAATTCTCTTGGCAAGGCCGAGCTGTCCACGACAATTAATGTCGAAGGAAAGTCTTCCATTGACACGGACACTCAACACCCTGAGGGACTGCAAAAGATCCAGGCCCTCGAAGGCCACCACTATGAGAGGGACACTGACGATCTGGACCAGTCAGTTACGACAGCGCCCGTATTCACATCAGCTCCCAAATCCATCGTGGTCCAAGAGGGACAGAAGGCGCATCTGGAGTGCCGCCTGATCCCCGTGGGAGATACCAAGCTGAAGGTCGAGTGGTTCAAGAACGGCCAGCCGGTGCCAGCAGGATCTCGTTTCGTCGAGATGTGCAACTTCGGTTTCGTGTCTCTGGACATCCTCAACACCTACGCTGAAGACTCCGGCACCTACACCTGCAAAGCCACGAACCAGCTGGGAGAAGCTGTCGTGTCTGCTCAACTCAAGTGCCACGCCGAAAAGTCGTTGATACTGGACACGCAAAACCAGGAAGCGTACGAAAAGATCCAGCAACTCGAGGACTACGGACGCCAAGCAAGGCCCGCCTATGTCGTGGAAGAGATGACCACGCAAGCGCCCGTGTTTACTCAAGCTATGAAGAACCTGAGCCTCAACGAAAACCAGAGTGCTCACTTTGAGTGTAAGCTCATACCAGTGGGCGACCCCAACCTCAAAGTGGAATGGTTCCACAACGGACTCCCCATACAGAAAGCCAACCGGGTCAACACCATTCATGACTTCGGATTCGTCGCTCTTGACCTGAGCTACGTCAAAGCACAGGACTCGGGCACTTACACTTGCAAGGCAACCAACTCCCTGGGATCTGCCGTTTGTTCTGCCACTCTCAACGTCCAAGATTCCAAGTCGCTGGTGTTCGACACTCAACACCCAGAGGGACTTCAGAAGATTCAACAGCTCGAGGAACTAGGACGTTACAAGCCAGAAGTGACGCAGGAAGCCCCGTGCCCGGGCCCACCGATGTTCGTCACCCAGCTCCAGGGCCCGAGCCGTCTGACAGAAGGCGAAAGTGCGCACCTGGAATGTCGTATTGCGCCCTACCCCGATGCCACTATGAAAGTGCAGTGGTTCCACAATGGCGTCGAGCTGCAGTCCGGTCATCGTTACCGGACGATGTACGACTTCGGCTTCTGCGCCTTGGACATTCTGTCAGCGAATGCTGAGCACTCCGGCGAGTACGTGGTCCAGGCCACGAACGAGCTTGGCACCGCGAGGTCCACGGCGCGCATTCATGTTGACGCCAAGGGTGGCGTCATCCTCGAAAGCCAGCAGCCAGACGCCCTCCCGAAGATCAAGCAGCTCGAGGAATCATGCGGCTACATCAGGCCAACGCAGGAAGAAGTGGTGATCAAGGACAAGCCTAACTTTGTTCGGGGTCTTTACAACCTGGAAACTCTGCATGAAGGCCAAAGCGCTCACCTTGAGGCTACGCTGACACCAATCAACGACGCTAACATGAAGATTCAGTGGTTCCACAACGGCGTAGAAATTCCGCTTGGCCACAGGTTCAAGACTGTTTCTGACTTTGGCTACGTGGCTCTCAACATCCTCTACGCCTACCCGGAGGATTCTGGCACATACATGTGCAAGGCAACCAATCAGCTCGGCGAAGCCGTTACCACGTGCTCAATCAACGTTCTCGGAAAGTCCGCCATTGTAACCGACACTTACCACGAAAAGGGCCTGGAGAAAATCAGGCAACTTGAAGAGTACCAGGCGCCGGAGAAGCCTGAACAGGTGATCCAGCTGCAAAGGCCCGTGTTCACGGTGCCTCTGAACTCGCTGGACGGTCTTGTGGAAGGACAGAGCGCTCACTTGGAGTGCAGACTCGAGCCCATCAATGACGCTGATCTCAAAGTTCAGTGGTACGTCAACGGCGTGGAAATCCGGCCAGGACATCGGTTCAGGACGACGCACGACTTTGGATATGTGGCCCTCGACATTATGTATGTGTATGCCGAAGATAGCGGAACCTACATGTGCAAGGCAACCAACTCTCTCGGAGAAGCGGTTACAACGTGTAACCTGCGTGCCCTACCCAAGCAAAAGATCTTCTATGATACGCATCATCCGGAGGGTCTTGAGAAAATCCGGGAGCTCGAAGCGCAAGTGAAGTACCAGTCTGCCGAAATTCAGGAGAAGCCGATCTCAAAGCCTGTGTTCATCACTGAACTGAGGGGAACCCAGGAGATCTCGGAGGGTGAAAGCGCCCACTTGGAGTGTCGCGTGGAACCAGCCCACGACGCTAAGTTGAAGATTGATATCCTTCACAACGGACGGCCGCTTACAGCTGCGACGCGCGTTCACATCACCAGCGACTTCGGCTACGTGGCGATAGACGCGACAAGCGCCATTCCAGAGGATTCCGGCACTTACACGGTGCGCGCCACTAACGACCTAGGAACTGCCGAGACAACAGCCACGCTGCGGGTCCTGCCAAAATCCAGCATCATCTCCGACACCCAGCACCCAGAAGGACTGGCTAAGATCAGGGAGATGGAAGACGAGTCTCGTTTCAAGCGTGAAGTCATCCAGGAGCCAGTCACCTACCAGACGCCCGTGTTCACGGTTCCTCTGCAGAACCTGGAGAACCTCGTTGAAGACCAGAGAAACGTCCACCTGGAGTGCAGGCTGATCCCAGTCGGAGATCCTACCCTCAATGTCCAGTGGTTCTTCAACGACACGCCACTGATGGAAGGCACGAGGTTCCATCCAGTTCACGACTTTGGCTACGTTGCACTCGACATGGACTACGTGCGCCCGGAGGACACTGGCGTCTACACGTGCAAGGCCACCAATTCCCTGGGCCAGGCAGTGACCACCTGCATGCTCAAGGTTAAACCCAAGGCGTCCATTCTTCTGGACACCCTGCAACCTCAAGGCTACGAGAAGATCCGTGAGCTGGAAGACTTGAAGGGCCAGAAGCCTCCGGAGAAACCAGACGCAGTGTACGAAAAGCCAGTGTTCACCAGCCACTTGGTTGGTCCTGGCGAGATAAACGAGGGACAGCCTGCACGTCTGGAGTGCCGCTGTGTCCCTGTCGGAGACCCTGACCTCAAGTTCTACTGGTACGTCAACGGCATCGAGCTTCCAAAAGGTTCGAGGTTGATTCCCAACAACGACTTTGGATTTGTGACCCTGGACATCCTGTCGGGCATCGCGGAAGACTCTGGAGTCTACATGTGCAAAGCGGTGAACAAGGCAGGAGAAGCCGTCACTTCAACTTCGCTGCGAGTGAAAGGCCGAGCTGGTGTGCTGCTGGACTCCCACCATCCCGAGGCGTACAGGCAGACGCAGAAGTTCGAGTACGACTCCAGCCGCATTCCTGAGAAGTGGTCCGACGAGAAGCCCAAGGCGGCGCCAGTGTTTGTGCAGCACTTGAACAACATCGATGGCGCTGTTGAAGGCCACTACCTGCGCATCGAAGGGCGCATTGAACCTACCAACGACGACAAGCTCAAAGTCAAATGGTTCAAGAACGGCAAGCCTCTGGTCATGGGTACTCGTATCAAGCCTACGGACGATTTCGGCTTGGTTTCGTTGGACATTTCCAGCGCTAGACCGGACGATTCTGGAATTTACACCTGTAAGGCCACCAACGATGTTGGCGAAGCTATATCGACGTGCACAATCAAGGTTGAAGGCCGCGAGAACATCATCCTAGCTTCCCAGCACCCCGACTCGTTGCCCAAGATCCGCCAGCTGGAAGAGTATGTGCCGCCTGAGAAGTACGTGCCTGAACCAGATTACGAAGGGCCCGTGTTCGTCACGCACCTCAACAACCTGGAGATCCGCGAGGGTGCGACTGCGCACTTCGAGTGCCGAGTGGAGCCATCCAAGGATCCCACGCTCAAGGTGGAATTCCTCAAGAACAACAAGCCAGTGCCTGCTGGTTCAAAGTACAACTTCAACAACGACTTTGGCTTCGTCACACTGGATGTCAGCAACGCATACCCGGAGGACGCCGGCATCTACACGTGCCGAGCCCGCAACGCGAAGGGCGAAGCGGTCACTACCGGCTCTCTCAAAGTCCAAGGAAAAAGCGGCGTGCTGTCCGACACTCTTCACCCAATGGGCGCACAGGGACTGTCCAAGGTCCAGGAGCTAGAGACTTCCTACTTGACCAGATACCAGGCTCCCGTCGAAGAGGCTGAGAAGGTGTTCCCGCGCCCGGTGTTCGTGGTGCCCCTTGAGCCAAACTTCTCCATCCAAGAGGGTAGCCCTGTCACCCTCGAGTGCAAGGTGGAACCAGCAAGCGACCCGAAGCTCAGGGTCGAGTGGTTCCTGAACGGCAAACCACTTGCGCCAGGATCCCGGCACACCGTCACACACGACTTCGGCTTCGTTGTGCTGGCCATGACCGACTTCTGGGGACGAGACGCCGGCGTTTACACCTGCCGCGCCAGCAACGCCGCAGGCGAAGCCTTTACCACAACCACAATCACGTGCCTGACCCGCAAGGGCGTGCAGGAGGACACGCTGCACCCGGAGGGCCGCAAGGGCCTCGAGTCCATCCAGCACCTGGAGGAGTCGCTGACGCGCGTGCCCGAAGCGATCCAGGAGGAAGCCGCTGGCCAGCCGCCAGTCTTCACATCTCAGTTCGTCAACCTCAAGGACCTGAACGAGGGCGAGATCGCCCACTTCGAGGCCACGCTGACGCCAGTCGGCGACCAGACCATGCAGGTGGAATGGTTCTTCCGCGGCAAACCCCTCAAAGCAGGGCACCGCATCCGGACGGTCCACGCGTTCGGCATGGTGGTGCTCGAGATCTTGGGGACTGTGCTGGAGGACTCTGGCCGCTACACTTGCCGTGCGACCAACAAGTGGGGCAAGGCCGAGGTCACCGTCGACCTCGAGTGCACCGACAAGACCAAGGGACAGCGGCCGCAATTCACCACGCAGCTGCAGAACCTCATGGACCTCAAGGAAGGAAACAGCGCTCACCTCGAATGCCACCTGGTGCCTGTTGGCGACCCAGACATGAAGGTCGAGTGGTACAAAAACTCGCAGCCACTTCGCGACAGTTCTCGCATCAAGACACTCAGCGACTTCGGCTACGTGGTGATGGACATCTCATTCGTGCACGCCGAAGACTCTGGAGACTACGTGTGCGTGGCCACCAACAAATACGGCTCCGACGCCACTAAGTGCACCATTCAGTGCGCAGGCACTGGCAAGATATTCAGGGACTCGCTACAGCCGCAATCCCTTGACAGGATCGCTGAACTGGAAGGTGCTTCGGCTCTCACCAGGACATCGGCCGTGATGGAGGCGACCCGGCTTCAGCCGCCCAAGTTCCTGTCACAGCTGAACAACATCACAAACCTGGTCGAAGGCCAGAGCGCACACTTCGAGTGCCAGCTGGTGCCGGTCAACGACCCAGACCTCACGGTTGAGTGGTACTTCAATGGCCAGCTGCTGCGCTCAGGACATCGTTTCCGGACGTTCCACGATTTCGGCATCGTTATCCTGGACATCCTGTACTGCTACGGAGAGGACTCCGGCGAATGGGTCTGCAAGGCCACGAACAAGCTCGGCTCAGATGTGACCCGCGCGACACTACAGTGCAAGTCCAAGAGCTCCTTGATCCTGACTCCTCAAGTGCCCCCTGAAATGGCCTCGGCGACACAGAACATCATCGCTCTCGAAGAAAGCCTGTACCGCACTGCGGCGGTCATTGAGCCCGAGGGACCCGCTGAGGCGCCTCGTTTCACCGTACCGCTCACTAACGTTGAGGATCTGAGGGAGGGAGACAACGCACATCTGGAAGCCCGGCTGACGCCTACAGATGATCCAGACCTGACTGTGGAATGGTTCAAGAACAACATGCCGCTGATGTCAGGAACAAGAATCCGAACCATCAACGACTTCGGCTTCGTGGTGCTCGAAATGAGCCCTGTGTACCCGGAGGACTCCGGCGTCTACTCCTGCCGTGCTAGAAACCGTTTCGGCGAGGCCGTCACCACATGCACACTCAAGTGTCAAGGAAAGCGTAGCATCATTCTCGAGACACAGCTGCCAGAGTCAATGACCACAGGCATCGAGAAGATTGCCAAGTTCGAAGAAGTCTCCTCGGCTAGGATCGACGAGAAGTGGACGGACAAGGACACCTCTCAGCCGCCCAAGTTCATCACCACGCCGCAGGACCTGACGCTGGCAGAGAACTCTCTGGCTCATTTCGAGTGCAGGTTGACGCCAGTCGGTGACCCTACGCTGAGAGTTGACTGGTATCACAATGGCAAGCCACTTGTCACGGGCTCTCGAGTCAAGACAATCAGCGACTTTGGATACGTCATTCTGGAGGTGGCCGGTGTTTACCCACGAGATTCGGGTGTTTACACATGCAGAGCCGTCAACAAGGTTGGCGAAGCGTCGGTCTCCTGCAAGCTCGCCGTAAAGGGCAAGCAGTCAGTCGTGATGGAACCACAGCTACCTCAAGAATTCCGATCTGGGTATGAAAGCATTCAGAAGTTGGAAGAGTCGATGTACCGAACTGAAGAGAAGATCTATGACGATGATAAGAAGGAACCACCGAAGTTTGTCACGCAGATCCAATCGCTCCTCGATAAGGTGGAAGGCGACAGCGCTCACTTCGAATGCAAGCTTATCCCAGTCGGAGATCCCAACCTGAAAGTGGAATGGTTCTTGAACGGACGTCCCCTTGTTACGGGTACGCGTGTTCACACCATCGATGACTTCGGCTTCGTGGTGCTGGACATCGACTGGCTCTTCCCCCGCGACTCTGGAGAGTATATGTGTCGTGCCACGAACCGTTGGGGCTCCGACACGACCAAGGCAACCCTAAAGATTAAAGCCAAGAAAGACATCATCATGGACAGCCAGCTGCCAGAGGGCATGAACGTTGACAAGCTGCGCGACCTGGAGTACCCGACGCCACAAGAAGAGACAATTCAGGAGCAGGAACCGGTCAAGCCCAGGTTCATCACCCAGATACAGCCTCAGCAAAACCTTAATGAGGGCGACTCGGCTCACTTCGAATGCCGCCTGGAGCCCATCAACGATCCAAAGCTGAGGGTGGAATGGTACCACAACGGACAACCCTTGAGATCAGGTCACCGGTTCAAGACAACGCACGATTTCGGCTTCGTTGCATTGGACGTGCTATACGTGTACCCTGAGGACTCGGGCACATACGTTGCACGGGCCGTCAACGACGTTGGCGAGGACCAAACTCAGGCCACCCTCAGGTGCACAGCGAAGCCAAAGCTGGACTACAGGACCCAACTTCCAAAGGACATGAAGGATGGTGTTAAGAAGATCGCTGAAATGGAAGCCTCCTGGCAGCGCGCTGAGACCCAGGAAGAAGTGGAAGAGGAACCATGCGCTCCCATGTTCATTATGAAGCCAGAGCCTCAAGTGGTCATCGAGGGCGAATGGGCAAAGTTCCAGTGCCGAGTTATTGGCCACCCCAAGCCAAGGCTCATCTGGGTTCTCAACGGCCACACGGTCATCGCCGGCTCAAGGTACAAGCTGACTTACGACGGTATCTACCATCTTGACATCCCTAAGACCCGCCAGTATGACCAAGGAAAAGTAGAAGTTTTTGCAAGGAACTTCTGCGGCGAAGCCTACTGCTTCACAACTCTTGAAGTCCGGCCCAAGTTCGATGACTACAGAGCCGTGCTCAAGCATTCTCCTAAGCCATGGTACGACCAAGACGTCAAGTCTTATCAAAAGTACCGACATGAGACTGAACTGCAACGAGTGTTTGAGGAGAAGCTTACACCTGGAGGCACTCGTATCGATGTCTGGAAGACTGAACAGGGCCAGCAAGGTGAACACCAAAAGATCAAGAAGAGGATCGAAGAAGAAGAGCTGGAGAAGCTCAAGCCGAAAGTGGAGCGCTTCAAGACCGACTCGATTTATTACGATGCGCGCACGGGCGAGAAGAAGGTGGAGACGGGCTCCCAGGCGCAGTACATGGCCAAGTACTTCGAGACCGAGGCCGAGAAGCAGCAGCGCGGCGCTACCGGCATTTCGCCCGAGTCCGTGGTGCAGGGCCGCGAGGTTCACACCACCACCCAGCGGCAGACTCAGAAGGAGCAGCAGGGCGACCTCGAGATCACCCGCAAGAAGACGCTCACCGAGACGCTCGAGCAAGAGCACAAGGGCGTCACCAAGGAGCAGCGCGTCCAGGGGCCCGCGCAAGAGCCGGCGAAGGCTCCCGTGTTCACCAAGAAGCTGCAGCCGTGCCGCGTGGACGAAGGCCGAGGCGCCAAGTTCCAGTGCACCTTCACCGGACAGCCAGCGCCCAAGATCACCTGGTACCGCGAGAACTTCCCCATCCAGCCCTCGCAGGATTTCCAGATCGTGACAACTGACAGCACGTCGACGCTGATCATCCGCGAAGTGTATGTGGAAGATTCCGGCGTCTTCTCAGTGAAGGCTGAAAACCGTGGCGGCTCAGCCAAGTCCTCCGCCAACTTGGTTGTTGAAGAGAGGCGAGAGCAACGCAGCGGTGTCGTGCCGCCAAACTTCACCCGGACCATCCAGGACGTCTCGTCGAAAGCCGGAAAGCTGGTCCGCCTCGACGCAAAGGTCTCAGGATCGAAGCCGTTGGATGTCTACTGGCTCAAGAACGGCAAGAAAGTGACGCCCGACGTGTCGCACAAAATCGTGGAGGAGGACGACCAGTACACGCTGCTCATTTTGGAGGCCCAGGCGGACTCCGACTCCGGAAGCTACGAGTGTGTCGCCATCAACTCGGCGGGAGAAGCCCGCTGCCAGGCTCACGTGGTGATCGAAGGCGCCAAGCCCAAGACGCCGCCCACGAGCCCCAAGGAGGCGCCCGGAGACCAGAAGCCACCCACGGTGACAGAGCCCCTCAAGCCACTCGCCGTCAAGGAAGGCCAGAGCGCCGTCTTCCGCTGCCGAATACCCGCCGTCCCGGGGGCGCAAGTAAAATGGTTCCGAGGAGACCAGCAAGTGAAGCAGTCGCGGTACTTCCGCATGTCCCAAGAGAATAACCTCTTCACGCTCAAGATCTCCGAAGCGTTCCCGGAGGACGAAGGTGTCTACAAGTGCGTCGCCACAAACCCGGCGGGCACTGTCTCTACCAGCGCCAACCTTAAAGTGATTGTGCCCGAACTGAACGAGGTGCCGCCGACGGTCACTCCTCTGGCCGACCTGACCGTGCCCGAAGGCTCGCCCGCCCGTTTCGTCACATCCCTGGGAGGCGTGCCACCGCCCAAGGTCATCTGGGTGCGCGAGGGCCACATCATCAAGCAGTCCCGTGACTTTCAGATGAACCAAGACCAAGGCTCAGCGTCTCTGGTCATCAGGCACACGTACCCCGAAGACGAAGGCGTCTACGTCTGTCGTGCCACCAACGCCTCGGGCCAAGCCGAGACCTCTGCCCGGCTTACCGTGCAACGTAAGGCCAAAAAGTAGAGGTCGCGCCCTGGCCTACTTGCTCGCTGCGGCTTATAGCGCTCCATTCGAAAAGCTGCGCGTACAAGCTGCTCAAACCGAGAGAGAGAGAGAACAAAAACAGCAACAAAAAAAGTTTGGCTCCTCACTTTCGCGTTCTCTAACTACAGCGCAGTAATAACGAGATATAATGTATCTTGCGTCTTAGCGGTGACGCATAGAATCTATTTTCAATTAATTTATTTATTAATTTATTTATTTCTGGAGAGAGTCTCTTTTGCATATTTTTGGTTTACAGTCACTTAACCTTCCCTCTTTTTGTTGAGAGCAAAAAATTTCGAATCGGAGTCCGTTCTTTACATTGCGTTCGCTTTCGTTTACTCCTTGTTCCTGTTTTGGTTTAAAACATTTTGTAAAGAAAAACAACTAATGTACGTACAGGGAATGTTCATTTCGCCCCCTTTTTTATACGATGAAATCGCGACATTCATGCCTAACAGGTTTATTCGTTTCATTAACCAAGTTCTGCCTAACAGAAGGGTGAAGTTTTGTATATTACTGACATTTCTATATTTACTCACGAGTTTTGTTTTTTTTTCCTTCGTTTGCAACAAGCGTTGCAGGACTGCACATCTCGGTCAGTTAGCGGCACTGAAGCGCTCGAACAAAGCGCGCACAACGCCACCGTAGGGCGCCACTATAGATGTGCATTCGTGAGGAGCCTTACAATTTATTGCATATTGGGGCTATTAACACCATCGCTTATTACTCCTATTCGAACCGATTGGCCACCATCGCGGGTTTCATCACGGCGTTAGGACCGCGAAATAATCGGCACTGACTGCCACAAGACGAGTTAGACAGGTTTAGGCGATTAACACATTTAACCATCCGGTCGAGAAGGCGACCTCACACTGCCAGCCGCGTTTGATATGTGATAACGCTAGCTGCTTGTTTTTAAACTCTCTCGGGCGCCCCTTGTTTGCGTTTTGTTTTGTTCCTCGCGGTGAGCCGTTCGCAATTCGCCACGCTAGTCACTACAGCAGCTGACGCCGTCGACGCCGCAAAGGCGTGCGCGCGACCCCGGCTGTAGCTTATCCAGGCGATATGTGTAGGCGAACGCGCTCCGTCCTAGACCTCCAAGTGGCCGAAACCTGTAGCGATTGCTTGTGGAGAAAAATTTCAATAAAGCCAAGTTATGCAAAACATGTTGTGTCTGATTTAGGCTTGCTCTCTCAGACTCACACTTTGTCGGAACTTCGACATTTGCATGCTTTTTTCTTTTATTCTTTTAATTATTGCTCTCGACTAATTTCTTTTTTATTCTGGCGTTATGCAACTCAATAACCTTTTAATCGATCGCAACAAAAATCTCTTTTTATTTTGATTTCTGCG

>AAFF12979

TCCAGGGCCGGCCCGAGCCACGCGTCTCCTGGTCCAAGGACCTGCTCCCCGTCCGCGACGCCGCCAAGCCCTCGCAGGAGCAGGGAAGGGCGCGGCTGGTGCTCAGTGGCGCAACCGAGAGCGACTCGGGCACATACACCGCCGTGGCCAAAAACAAAGCCGGAGAGACGGCCTGCTCCTGCCAAGTGAAAGTGGCCGAGGACGCCGCTCCGCCCGAGCCTCCGCGTGTGCTCAAGGCCCTGGAGGATCTGGAAGTAAAGCCTGGACCGGACCCTATCACGCTGGAATGCATCATCGTCGGCCGGCCCGAACCAGAGGTCATCTGGTACCACAACACCCAACCCATCAAGGAGTCTGAGCGGGTACGGCTGCTGTTCCGCGGGGACAAGTGCTCTCTTGTCCTGAACGGCGTCAGCGCCCAGAACGCCGGCACCTACCGATGCTCGGCGGTCAACCCCATGGGCAGCTGCTACACGGAGTGCAACATGCGCGTGCCGCTTTCTGCTCCCGTGTTTCTGGAGCCGCTCCGAGACGTGACCACGGATGAGGGCTGCCGCGTGGTGCTCACTGCAAAGCTATGGGCACCCGAGCCACCTTTCGTCCGCTGGTTTAAGGATGGAAGAGAGGTGCTTCCAAGCCCAGACTTCCAGGTCAGCCACGATCCGGACGGAACTGTGAAGCTACTGATCCCGAAGGCGGCGGCCAGCAACAGCGGCCATTACGAAGTCGAGGCCAGCAACCCCGGCGGTCGCACGCGCACGGGCTGCAAGGTGCACGTTCGTGAAGCTCAGAAGGCGGTCCAGGCGTCCAGCCAGCTTGCGGTGTCGAGGACATCGCAGCAGACCGTGTCCGTTTCGAAGAGCGGCTCGGAGCTGCGGCTCGCCCGCTCGCTGCCCTCGGAGCTGGTGGTGCAGAGCGGAACCAAGGTGTGCCTCAGCGTGGCCTGTTCGCAACCAGCCGGCGGCCAGCTGACCGCCTCGTGGTTCCGGTCGGGCCAGCCCATCGCCGACTCTCCGGACTTCCGGCTCACCCGTTCTCTGGAGAACGTGGGTGGCGCATCGCTGTCAGTGTTCAGCCTAACCATCTCGGAGGCCTTTCCCGAGGACTCCGGAGACCTTGAAGTGCGCGTGCAAGGCCCCAGCGGCGTTGTGACGGCGCGCACAAAGCTCGTGGTGCTCGACGAAGACGACGACGTCACTGACAGCGTGCAGAGCCGCGACGAGAGCATCCAGCAAGCGCGCGAGGTGAAGGTGTACGAGACGAAGCTGGCTCGACCGGGCCCGGTGGACTCTGCCGGCTTGGCTCTCACGGGAGACCTGCAGCTGCGCGTTACCGAGGTGACGCACGTGAACGAAATGCCGCCGGAGGTCTCGGAAGAGAAGACCACCAAAGTAGTGGACCAAGCTGGCGCCAAAGACAAAGTCACCGAAGAAGTAACTCGCCGAGTGACCAAGCGGGTCACTGAGATTACCAAGACTCACACTAAAGAGGTGAGGGGTCCCTTGGTCTACAAATGGGAGTTTCGCAAGTAGCAGGCCTTCTAGTTTCTTCCTTAGACTTGCTCGGCCGGCACGGAGACTGTCCCGGGAGCTCTTGTCCGCGCTTTCTGGTGGTGCTCTCCGTTGTGTCTGTCTGGCTGTCGCTCGGAACAGAGCGCTCTCCTTCCTTACCCTTGGCCCCTAAGTGTTCGGTGTAGCATGCTCGTCACAGTGTCTTGACCAAAGAAAATAATCTTGACAGTTGTAATGGTGATGTGAAATAAAGCCATGCTTTGTGTTCGATATGTGTTGCATGTTGTCTCCTTCCCAACCAAAACCAGACGTGAGCACCTTGCACACACGTGGCTGCCGCTTTCTTGCGCTGCACATAGATGGGCCTGCTGCTTGCGGCACACTGTGCGCAAGCTCGATGCACAAACAACTGCGGTGTCGGGGCTCGCCCCACTGAGCTTTGCATGGACGCGATGGGGTCCCGTTTCCGTTGCAGCTGAGCACCAGTCGGGCCAGCCGCGC

>AAFF29296

CTGGAGTTTCAATGGAGAAAAGAACAAAAATTCCTGTCCGATGTTGAGACAGAAGTCTCTGAAAAAGTAGAGCCGAACGATGTCGTGGTCTTCACAAGCTACCTGCACCTGAGGAATATCCAAAACAAGGATGAGGGGCGGTACCAGTGTGTGATTCGAAATCAGTTTGGCTCTGTCTACTCCAACCAGTCCAACATTAGTGTGTATGTCCTGCCTACGTTTGTCAAGACTCCATCCAACTTGACGGTGCGTGCGGGAGGCACAGCGCGGCTTGAGTGCGGAGCCACGGGCCAGCCAACACCGACGGTGTCTTGGCAGAAGGACGGTGGGGACGACTTCCCAGCTGCCAGAGAGAGGCGAATGCATGTCATGCCCACTGACGACGTCTTCTTTGTTGTGAGTCTCAAGGCAGCCGACTCAGGAGTCTACACCTGCACTGCCCGCAGCCGAGCCGGTGTGGTGCGGGCCAACGCCACGCTCACCGTGCTTGAAGCACCAGCATTTGTGCGCCCCATGCGGAGCAAGCAGGTGGCGGCCGGGGACACGGCAGTGTTGGAATGTCTGTCGTCAGGCAGCCCCAAGCCCCGGCTGACGTGGCTCAAGGATGGAGCACCCTTGGTGACCACCGAGCGCCACTTCCTGGTGGCCGAGGCACAGCTGTTGGTCATCACCGACAGCCGGTCCTCGGACTCGGGCCAGTATGCGTGCGAGATGACCAACACCCTGGGCATCGAGA

>AAFF47956

GCATGATGACTGTGGCCTGACAAACGTAGGTTCCGGCATCCTCAGCAGTGACACGATCAAGGCGCAGAGTGTCTCCTTGAAGTGCAAACTGGGGGTCACCTTCCTTGGTCCACAATACAGAATGTGGCTCAGGGTTAGAAGCAACATTGCATTTGATGGCTATATTTTCTCCTTGAGAGGATTCCTGCTCTGGAAGCACGTTCACACGAGGCCCATACAGCACACTAAGACGCAGGTCGGCTTGAGCGGGCTGCATAACTCCATTATCAGCAACACAGGCATAGTTTCCACTGTCTTCAGGAGTCACAGATGGGATGGTGTGGTTGTACGTGTTGGACAGCAGCTGGCCTCGCTTCATCCATCGGACGGAACGCACAGGAGGATTGGCCAGCACAGAGCATGTCAGTGTGGCATCAGACCCCAC

>AAFF17185

GCTTCACCCAGCGGGTTCTCCGCCACACACTGGTACACCCCGGCATCTTCCATAGCCAGGGCTCCAATCTCGAGGCTGCCGCCGTCACCAACGTGGAAGTGCCCTGTGCTCTGGTCCACAGGCCGCGCATCCAGGAGCCAGTGCACCTGGGGCTCTGGGTGGCCCTCAGCCACGCAGGGAAGCCGCACCTGCTGCCCCAGTTCCACTGCCGTCTCCTCATTCACCTTCTGGCTCAAGGAGGGCAGCACTGACACAGTGACGTTGGCATGGGCCACGACAGCAGAGGAGTCCTCAGGGGCATGTCGAAGGGTGACCTGGCAGGAGTAGCGGCCAGTGTGCCCGGGCCCCAGGCGCAGCAGGGTCAGGGTGCGGTTCCAGTGGGTGAGAACGTGTGGCAGCTCACCCAGGGCCCTACCATCCTTCAGCCACTCAATCTGCACCTGGTCCAGAGGCCTGCCAGAGGCAATGCACTCNN

>AAUF27591

GCACAAGCTGCTCTCTAATGGCACGCTTCTGATACGAGACGTTCAGAAGTCTGACTCTGGCATGTACACGTGTAGTGCAGACAATGGTATTGGTTCGGCGCTGAAAAAGGCTGTCGCTGTTACTGTGTACACAGTCGCCCAAGCGCAGGTTTTAACACAGCATCTTTCTGTGCAATTTGGCCAAACTGCCAACCTCACTTGCATTGCTACTGGTGATCACCCAATTACGGTAACGTGGCTTAAAGGTGAAAAGTCAGTAGCAACCAACAGCCAACTTTATGACAGAGTTATTGTGTCCAACGACACTCAGAAAGAACGACTGGTGTCTTCATTGATACTACAGCAGGTGACCGCTGGCGATGCAGGTCGTTATACCTGCAGAGTGAAAAATGCTTATGCAGAAGACACCAAGGCTATTAGGCTGAATGTCCAACAGCCTCCCTCTAATCCCACTGAGGTTGAAGTGTCGGATGTATGGAGTCGTAGTGCCAGAATCCGCTGGAAAAGCCCTACAAGTTCAACTGTTTTGTCATACCAAGTGCGCTTTTGGTCGCACCGAGAGGATGAAATGTTGAA

>AAUF45409

AAGTGGTGATGGTGTGTAACACAGAGGCCTATCCGATATCGATCAACTACTGGACACTGGAGAGCGGCGATCTCATCGCCGAGTCGGCCAAGTACTCGCTCAACCGCACCGAGAACGTGTACAAGGNNNNNNTGAGGCTGCGGATAAGGCGCATTGGTCCCGAAGACTTCGGCGCGTACCGTTGCTNNGCCAAGAACTCCTTGGGCTCCACGGAGGGATCCATACGATTGTACGAAATTCATGTCCCGCCAACAGCAAAGGTGAAAGAGCCTTCAACTGCAAGAATTCAGAGCGTCGAGGAAGGCAAACTCGAGGCATCCGACGCAGCTATCACCAAACATCAAAGCTTCCCGTTAAGGCCGCCCCCCTCCGGGTCTCTGCCGGAAGGCGAAGGCTCTGNNNNNNNNNNNNNNNNNNNNNNNNNNNNNNNNNNNGTCGTCTGGGCAATGTTGCTGGTGGTTTCTGC

>AAUF27677

CAGGAATCCGTCCTTGCTCCACTGCACAGGTCCGGACAGACGGCCGAGCTGCAGTGCCACGTCGGCAACCTGGCCGGACCTGTGCAGTGGAGCAAGGACGGATTCCTGCTCGGTTTCGACCCGTCCATACCCGGTTTTCCGCGGTACACCATGATCGTAGACAATCAACGCGGCGTCTATAACCTGCGCATCACCAACGTTCAGATGGAAGACGAGGCGGAATACCAGTGCCAGGTGGGACCAGCCCCAAAGAATCACGCCATTTGGACTGCAGCGCGCCTGACAGTGCTTGTGCCAACGAAAGAAATCGAGCTCCGACACCGCGGAAATGGCAGCGTGGTGGAAGTTCGGGAAGCCGAAAGCCTCGTCATCGCCTGCTGGGTGCGGAACACCAAGCCAGCGGCGGACATCAGGTGGCTGCGGAACGACCTCCCGCTCGCCCAAGAGAAAGCTTTGACCAAGAAGAACGCCTCCGGGGAGAAGCTTTTCTCGGTGTACAGCTCTGTCACGCTGTACCCGAAGCTGGACGACAATCGCGCCGTGTACACCTGCGAGGCCACGCACCCGGCCCTCGAGAGCCCTCTGCAAGCGTCCGTCACTGTTAGCGTATTGTATCCTCCGGGCGCGCCCGAGATCGAGGGCTACCATGAGGGCGACATCGTGCAAGTGGGCGACACCCTGACGCTGGCGTGCATCTCTCGCGG

>AAUF4212

CTTTTCTTTTTTTTTTTGCACCGAGCTGTGAGAGCAGCACATCTGTGCCAGATGTAGGCCTGTCTCCCTCACACTTTTAGGCCGCCAATGCTTTTCGGTCTTTGCATGCTTCGCAGAAATCAAAATAAAAAGAAATTTTTGTTGCGATCGATTAAAAGGTTATTGAGTTGCATAACGCCAGAATAAAAAAGAAATTAGTCGAGAGCAATAATTAAAAGAATAAAAGAAAAAAGCATGCAAATGTCGAAGTTCCGACAAAGTGTGAGTCTGAGAGAGCAAGCCTAAATCAGNNNNNNNNNNTTTTGCATAACTTGGCTTTATTGAAATTTTTCTCCACAAGCAATCGCTACAGGTTTCGGCCACTTGGAGGTCTAGGACGGAGCGCGTTCGCCTACACATATCGCCTGGATAAGCTACAGCCGGGGTCGCGCGCACGCCTTTGCGGCGTCGACGGCGTCAGCTGCTGTAGTGACTAGCGTGGCGAATTGCGAACGGCTCACCGCGAGGAACAAAACAAAACGCAAACAAGGGGCGCCCGAGAGAGTTTAAAAACAAGCAGCTAGCGTTATCACATATCAAACGCGGCTGGCAGTGTGAGGTCGCCTTCTCGACCGGATGGTTAAATGTGTTAATCGCCTAAACCTGTCTAACTCGTCTTGTGGCAGTCAGTGCCGATTATTTCGCGGTCCTAACGCCGTGATGAAACCCGCGATGGTGGCCAATCGGTTCGAATAGGAGTAATAAGCGATGGTGTTAATAGCCCCAATATGCAATAAATTGTAAGGCTCCTCACGAATGCACATCTATAGTGGCGCCCTACGGTGGCGTTGTGCGCGCTTTGTTCGAGCGCTTCAGTGCCGCTAACTGACCGAGATGTGCAGTCCTGCAACGCTTGTTGCAAACGAAGGAAAAAAAAAACTCGTGAGTAAATATAGAAATGTCAGTAATATACAAAACTTCACCCTTCTGTTAGGCAGAACTTGGTTAATGAAACGAATAAACCTGTTAGGCATGAATGTCGCGATTTCATCGTATAAAAAAGGGGGCGAAATGAACATTCCCTGTACGTACATTAGTTGTTTTTCTTTACAAAATGTTTTAAACCAAAACAGGAACAAGGAGTAAACGAAAGCGAACGCAATGTAAAGAACGGACTCCGATTCGAAATTTTTTGCTCTCAACAAAAAGAGGGAAGGTTAAGTGACTGTAAACCAAAAATATGCAAAAGAGACTCTCTCCAGAAATAAATAAATTAATAAATAAATTAATTGAAAATAGATTCTATGCGTCACCGCTAAGACGCAAGATACATTATATCTCGTTATTACTGCGCTGTAGTTAGAGAACGCGAAAGTGAGGAGCCAAACTTTTTTTGTTGCTGTTTTTGTTCTCTCTCTCTCTCGGTTTGAGCAGCTTGTACGCGCAGCTTTTCGAATGGAGCGCTATAAGCCGCAGCGAGCAAGTAGGCCAGGGCGCGACCTCTACTTTTTGGCCTTACGTTGCACGGTAAGCCGGGCAGAGGTCTCGGCTTGGCCCGAGGCGTTGGTGGCACGACAGACGTAGACGCCTTCGTCTTCGGGGTACGTGTGCCTGATGACCAGAGACGCTGAGCCTTGGTCTTGGTTCATCTGAAAGTCACGGGACTGCTTGATGATGTGGCCCTCGCGCACCCAGATGACCTTGGGCGGTGGCACGCCTCCCAGGGATGTGACGAAACGGGCGGGCGAGCCTTCGGGCACGGTCAGGTCGGCCAGAGGAGTGACCGTCGGCGGCACCTCGTTCAGTTCGGGCACAATCACTTTAAGGTTGGCGCTGGTAGAGACAGTGCCCGCCGGGTTTGTGGCGACGCACTTGTAGACACCTTCGTCCTCCGGGAACGCTTCGGAGATCTTGAGCGTGAAGAGGTTATTCTCTTGGGACATGCGGAAGTACCGCGACTGCTTCACTTGCTGGTCTCCTCGGAACCATTTTACTTGCGCCCCCGGGACGGCGGGTATTCGGCAGCGGAAGACGGCGCTCTGGCCTTCCTTGACGGCGAGTGGCTTGAGGGGCTCTGTCACCGTGGGTGGCTTCTGGTCTCCGGGCGCCTCCTTGGGGCTCGTGGGCGGCGTCTTGGGCTTGGCGCCTTCGATCACCACGTGAGCCTGGCAGCGGGCTTCTCCCGCCGAGTTGATGGCGACACACTCGTAGCTTCCGGAGTCGGAGTCCGCCTGGGCCTCCAAAATGAGCAGCGTGTACTGGTCGTCCTCCTCCACGATTTTGTGCGACACGTCGGGCGTCACTTTCTTGCCGTTCTTGAGCCAGTAGACATCCAACGGCTTCGATCCTGAGACCTTTGCGTCGAGGCGGACCAGCTTTCCGGCTTTCGACGAGACGTCCTGGATGGTCCGGGTGAAGTTTGGCGGCACGACACCGCTGCGTTGCTCTCGCCTCTCTTCAACAACCAAGTTGGCGGAGGACTTGGCTGAGCCGCCACGGTTTTCAGCCTTCACTGAGAAGACGCCGGAATCTTCCACATACACTTCGCGGATGATCAGCGTCGACGTGCTGTCAGTTGTCACGATCTGGAAATCCTGCGAGGGCTGGATGGGGAAGTTCTCGCGGTACCAGGTGATCTTGGGCGCTGGCTGTCCGGTGAAGGTGCACTGGAACTTGGCGCCTCGGCCTTCGTCCACGCGGCACGGCTGCAGCTTCTTGGTGAACACGGGAGCCTTCGCCGGCTCTTGCGCGGGCCCCTGGACGCGCTGCTCCTTGGTGACGCCCTTGTGCTCTTGCTCGAGCGTCTCGGTGAGCGTCTTCTTGCGGGTGATCTCGAGGTCGCCCTGCTGCTCCTTCTGAGTCTGCCGCTGGGTGGTGGTGTGAACCTCGCGGCCCTGCACCACGGACTCGGGCGAAATGCCGGTAGCGCCGCGCTGCTGCTTCTCGGCCTCGGTCTCGAAGTACTTGGCCATGTACTGCGCCTGGGAGCCCGTCTCCACCTTCTTCTCGCCCGTGCGCGCATCGTAATAAATCGAGTCGGTCTTGAAGCGCTCCACTTTCGGCTTGAGCTTCTCCAGCTCTTCTTCTTCGATCCTCTTCTTGATCTTTTGGTGTTCACCTTGCTGGCCCTGTTCAGTCTTCCAGACATCGATACGAGTGCCTCCAGGTGTAAGCTTCTCCTCAAACACTCGTTGCAGTTCAGTCTCATGTCGGTACTTTTGATAAGACTTGACGTCTTGGTCGTACCATGGCTTAGGAGAATGCTTGAGCACGGCTCTGTAGTCATCGAACTTGGGCCGGACTTCAAGAGTTGTGAAGCAGTAGGCTTCGCCGCAGAAGTTCCTTGCAAAAACTTCTACTTTTCCTTGGTCATACTGGCGGGTCTTAGGGATGTCAAGATGGTAGATACCGTCGTAAGTCAGCTTGTACCTTGAGCCGGCGATGACCGTGTGGCCGTTGAGAACCCAGATGAGCCTTGGCTTGGGGTGGCCAATAACTCGGCACTGGAACTTTGCCCATTCGCCCTCGATGACCACTTGAGGCTCTGGCTTCATAATGAACATGGGAGCGCATGGTTCCTCTTCCACTTCTTCCTGGGTCTCAGCGCGCTGCCAGGAGGCTTCCATTTCAGCGATCTTCTTAACACCATCCTTCATGTCCTTTGGAAGTTGGGTCCTGTAGTCCAGCTTTGGCTTCGCTGTGCACCTGAGGGTGGCCTGAGTTTGGTCCTCGCCAACGTCGTTGACGGCCCGTGCAACGTATGTGCCCGAGTCCTCAGGGTACACGTATAGCACGTCCAATGCAACGAAGCCGAAATCGTGCGTTGTCTTGAACCGGTGACCTGATCTCAAGGGTTGTCCGTTGTGGTACCATTCCACCCTCAGCTTTGGATCGTTGATGGGCTCCAGGCGGCATTCGAAGTGAGCCGAGTCGCCCTCATTAAGGTTTTGCTGAGGCTGTATCTGGGTGATGAACCTGGGCTTGACCGGTTCCTGCTCCTGAATTGTCTCTTCTTGTGGCGTCGGGTACTCCAGGTCGCGCAGCTTGTCAACGTTCATGCCCTCTGGTAGCTGGCTGTCCATGATGATGTCTTTCTTGGCTTTAATCTTTAGGGTTGCCTTGGTCGTGTCGGAGCCCCAACGGTTCGTGGCACGACACATATACTCTCCAGAGTCGCGGGGGAAGAGCCAGTCGATGTCCAGCACCACGAAGCCGAAGTCATCGATGGTGTGAACACGCGTACCCGTAACAAGGGGACGTCCGTTCAAGAACCATTCCACTTTCAGGTTGGGATCTCCGACTGGGATAAGCTTGCATTCGAAGTGAGCGCTGTCGCCTTCCACCTTATCGAGGAGCGATTGGATCTGCGTGACAAACTTCGGTGGTTCCTTCTTATCATCGTCATAGATCTTCTCTTCAGTTCGGTACATCGACTCTTCCAACTTCTGAATGCTTTCATACCCAGATCGGAATTCTTGAGGTAGCTGTGGTTCCATCACGACTGACTGCTTGCCCTTTACGGCGAGCTTGCAGGAGACCGACGCTTCGCCAACCTTGTTGACGGCTCTGCATGTGTAAACACCCGAATCTCGTGGGTAAACACCGGCCACCTCCAGAATGACGTATCCAAAGTCGCTGATTGTCTTGACTCGAGAGCCCGTGACAAGTGGCTTGCCATTGTGATACCAGTCAACTCTCAGCGTAGGGTCACCGACTGGCGTCAACCTGCACTCGAAATGAGCCAGAGAGTTCTCTGCCAGCGTCAGGTCCTGCGGCGTGGTGATGAACTTGGGCGGCTGAGAGGTGTCCTTGTCCGTCCACTTCTCGTCGATCCTAGCCGAGGAGACTTCTTCGAACTTGGCAATCTTCTCGATGCCTGTGGTCATTGACTCTGGCAGCTGTGTCTCGAGAATGATGCTACGCTTTCCTTGACACTTGAGTGTGCATGTGGTGACGGCCTCGCCGAAACGGTTTCTAGCACGGCAGGAGTAGACGCCGGAGTCCTCCGGGTACACAGGGCTCATTTCGAGCACCACGAAGCCGAAGTCGTTGATGGTTCGGATTCTTGTTCCTGACATCAGCGGCATGTTGTTCTTGAACCATTCCACAGTCAGGTCTGGATCATCTGTAGGCGTCAGCCGGGCTTCCAGATGTGCGTTGTCTCCCTCCCTCAGATCCTCAACGTTAGTGAGCGGTACGGTGAAACGAGGCGCCTCAGCGGGTCCCTCGGGCTCAATGACCGCCGCAGTGCGGTACAGGCTTTCTTCGAGAGCGATGATGTTCTGTGTCGCCGAGGCCATTTCAGGGGGCACTTGAGGAGTCAGGATCAAGGAGCTCTTGGACTTGCACTGTAGTGTCGCGCGGGTCACATCTGAGCCGAGCTTGTTCGTGGCCTTGCAGACCCATTCGCCGGAGTCCTCTCCGTAGCAGTACAGGATGTCCAGGATAACGATGCCGAAATCGTGGAACGTCCGGAAACGATGTCCTGAGCGCAGCAGCTGGCCATTGAAGTACCACTCAACCGTGAGGTCTGGGTCGTTGACCGGCACCAGCTGGCACTCGAAGTGTGCGCTCTGGCCTTCGACCAGGTTTGTGATGTTGTTCAGCTGTGACAGGAACTTGGGCGGCTGAAGCCGGGTCGCCTCCATCACGGCCGATGTCCTGGTGAGAGCCGAAGCACCTTCCAGTTCAGCGATCCTGTCAAGGGATTGCGGCTGTAGCGAGTCCCTGAATATCTTGCCAGTGCCTGCGCACTGAATGGTGCACTTAGTGGCGTCGGAGCCGTATTTGTTGGTGGCCACGCACACGTAGTCTCCAGAGTCTTCGGCGTGCACGAATGAGATGTCCATCACCACGTAGCCGAAGTCGCTGAGTGTCTTGATGCGAGAACTGTCGCGAAGTGGCTGCGAGTTTTTGTACCACTCGACCTTCATGTCTGGGTCGCCAACAGGCACCAGGTGGCATTCGAGGTGAGCGCTGTTTCCTTCCTTGAGGTCCATGAGGTTCTGCAGCTGCGTGGTGAATTGCGGCCGCTGTCCCTTGGTCTTGTCGGTGCACTCGAGGTCGACGGTGACCTCGGCCTTGCCCCACTTGTTGGTCGCACGGCAAGTGTAGCGGCCAGAGTCCTCCAGCACAGTCCCCAAGATCTCGAGCACCACCATGCCGAACGCGTGGACCGTCCGGATGCGGTGCCCTGCTTTGAGGGGTTTGCCGCGGAAGAACCATTCCACCTGCATGGTCTGGTCGCCGACTGGCGTCAGCGTGGCCTCGAAGTGGGCGATCTCGCCCTCGTTCAGGTCCTTGAGGTTGACGAACTGAGATGTGAAGACTGGCGGCTGGCCAGCGGCTTCCTCCTGGATCGCTTCGGGCACGCGCGTCAGCGACTCCTCCAGGTGCTGGATGGACTCGAGGCCCTTGCGGCCCTCCGGGTGCAGCGTGTCCTCCTGCACGCCCTTGCGGGTCAGGCACGTGATTGTGGTTGTGGTAAAGGCTTCGCCTGCGGCGTTGCTGGCGCGGCAGGTGTAAACGCCGGCGTCTCGTCCCCAGAAGTCGGTCATGGCCAGCACAACGAAGCCGAAGTCGTGTGTGACGGTGTGCCGGGATCCTGGCGCAAGTGGTTTGCCGTTCAGGAACCACTCGACCCTGAGCTTCGGGTCGCTTGCTGGTTCCACCTTGCACTCGAGGGTGACAGGGCTACCCTCTTGGATGGAGAAGTTTGGCTCAAGGGGCACCACGAACACCGGGCGCGGGAACACCTTCTCAGCCTCTTCGACGGGAGCCTGGTATCTGGTCAAGTAGGAAGTCTCTAGCTCCTGGACCTTGGACAGTCCCTGTGCGCCCATTGGGTGAAGAGTGTCGGACAGCACGCCGCTTTTTCCTTGGACTTTGAGAGAGCCGGTAGTGACCGCTTCGCCCTTCGCGTTGCGGGCTCGGCACGTGTAGATGCCGGCGTCCTCCGGGTATGCGTTGCTGACATCCAGTGTGACGAAGCCAAAGTCGTTGTTGAAGTTGTACTTTGAACCAGCAGGCACTGGCTTGTTGTTCTTGAGGAATTCCACCTTGAGCGTGGGATCCTTGGATGGCTCCACTCGGCACTCGAAGTGCGCAGTCGCACCCTCGCGGATCTCCAGGTTGTTGAGGTGCGTGACGAACACGGGCCCTTCGTAATCTGGTTCAGGCACGTACTTCTCAGGCGGCACATACTCTTCCAGCTGGCGGATCTTGGGCAACGAGTCGGGGTGCTGGGAAGCCAGGATGATGTTCTCGCGGCCTTCAACCTTGATTGTGCACGTCGATATAGCTTCGCCAACATCGTTGGTGGCCTTACAGGTGTAAATTCCAGAATCGTCCGGTCTAGCGCTGGAAATGTCCAACGAAACCAAGCCGAAATCGTCCGTAGGCTTGATACGAGTACCCATGACCAGAGGCTTGCCGTTCTTGAACCATTTGACTTTGAGCTTGTCGTCGTTGGTAGGTTCAATGCGCCCTTCGATGCGCAGGTAGTGGCCTTCAACAGCGCCATCGATGTTGTTCAAGTGCTGCACAAACACTGGCGCCGCCTTGGGCTTCTCGTCGGACCACTTCTCAGGAATGCGGCTGGAGTCGTACTCGAACTTCTGCGTCTGCCTGTACGCCTCGGGATGGTGGGAGTCCAGCAGCACACCAGCTCGGCCTTTCACTCGCAGCGAAGTTGAAGTGACGGCTTCTCCTGCCTTGTTCACCGCTTTGCACATGTAGACTCCAGAGTCTTCCGCGATGCCCGACAGGATGTCCAGGGTCACAAATCCAAAGTCGTTGTTGGGAATCAACCTCGAACCTTTTGGAAGCTCGATGCCGTTGACGTACCAGTAGAACTTGAGGTCAGGGTCTCCGACAGGGACACAGCGGCACTCCAGACGTGCAGGCTGTCCCTCGTTTATCTCGCCAGGACCAACCAAGTGGCTGGTGAACACTGGCTTTTCGTACACTGCGTCTGGTTTCTCCGGAGGCTTCTGGCCCTTCAAGTCTTCCAGCTCACGGATCTTCTCGTAGCCTTGAGGTTGCAGGGTGTCCAGAAGAATGGACGCCTTGGGTTTAACCTTGAGCATGCAGGTGGTCACTGCCTGGCCCAGGGAATTGGTGGCCTTGCACGTGTAGACGCCAGTGTCCTCCGGGCGCACGTAGTCCATGTCGAGTGCAACGTAGCCAAAGTCGTGAACTGGATGGAACCTCGTGCCTTCCATCAGTGGCGTGTCGTTGAAGAACCACTGGACATTGAGGGTAGGATCTCCGACTGGGATCAGCCTGCACTCCAGGTGGACGTTTCTCTGGTCTTCAACGAGGTTCTCCAGGTTCTGCAGAGGAACCGTGAACACGGGCGTCTGGTAGGTGACTGGCTCCTGGATGACTTCACGCTTGAAACGAGACTCGTCTTCCATCTCCCTGATCTTAGCCAGTCCTTCTGGGTGCTGGGTGTCGGAGATGATGCTGGATTTTGGCAGGACCCGCAGCGTGGCTGTTGTCTCGGCAGTTCCTAGGTCGTTAGTGGCGCGCACCGTGTAAGTGCCGGAATCCTCTGGAATGGCGCTTGTCGCGTCTATCGCCACGTAGCCGAAGTCGCTGGTGATGTGAACGCGCGTCGCAGCTGTAAGCGGCCGTCCGTTGTGAAGGATATCAATCTTCAACTTAGCGTCGTGGGCTGGTTCCACGCGACACTCCAAGTGGGCGCTTTCACCCTCCGAGATCTCCTGGGTTCCCCTCAGTTCAGTGATGAACACAGGCTTTGAGATCGGCTTCTCCTGAATTTCGGCAGACTGGTACTTCACTTGCGCTTCGAGCTCCCGGATTTTCTCAAGACCCTCCGGATGATGCGTATCATAGAAGATCTTTTGCTTGGGTAGGGCACGCAGGTTACACGTTGTAACCGCTTCTCCGAGAGAGTTGGTTGCCTTGCACATGTAGGTTCCGCTATCTTCGGCATACACATACATAATGTCGAGGGCCACATATCCAAAGTCGTGCGTCGTCCTGAACCGATGTCCTGGCCGGATTTCCACGCCGTTGACGTACCACTGAACTTTGAGATCAGCGTCATTGATGGGCTCGAGTCTGCACTCCAAGTGAGCGCTCTGTCCTTCCACAAGACCGTCCAGCGAGTTCAGAGGCACCGTGAACACGGGCCTTTGCAGCTGGATCACCTGTTCAGGCTTCTCCGGCGCCTGGTACTCTTCAAGTTGCCTGATTTTCTCCAGGCCCTTTTCGTGGTAGGTGTCGGTTACAATGGCGGACTTTCCGAGAACGTTGATTGAGCACGTGGTAACGGCTTCGCCGAGCTGATTGGTTGCCTTGCACATGTATGTGCCAGAATCCTCCGGGTAGGCGTAGAGGATGTTGAGAGCCACGTAGCCAAAGTCAGAAACAGTCTTGAACCTGTGGCCAAGCGGAATTTCTACGCCGTTGTGGAACCACTGAATCTTCATGTTAGCGTCGTTGATTGGTGTCAGCGTAGCCTCAAGGTGAGCGCTTTGGCCTTCATGCAGAGTTTCCAGGTTGTAAAGACCCCGAACAAAGTTAGGCTTGTCCTTGATCACCACTTCTTCCTGCGTTGGCCTGATGTAGCCGCATGATTCCTCGAGCTGCTTGATCTTCGGGAGGGCGTCTGGCTGCTGGCTTTCGAGGATGACGCCACCCTTGGCGTCAACATGAATGCGCGCCGTGGACCTCGCGGTGCCAAGCTCGTTCGTGGCCTGGACCACGTACTCGCCGGAGTGCTCAGCATTCGCTGACAGAATGTCCAAGGCGCAGAAGCCGAAGTCGTACATCGTCCGGTAACGATGACCGGACTGCAGCTCGACGCCATTGTGGAACCACTGCACTTTCATAGTGGCATCGGGGTAGGGCGCAATGCGACATTCCAGGTGCGCACTTTCGCCTTCTGTCAGACGGCTCGGGCCCTGGAGCTGGGTGACGAACATCGGTGGGCCCGGGCACGGGGCTTCCTGCGTCACTTCTGGCTTGTAACGTCCTAGTTCCTCGAGCTGTTGAATCTTCTGAAGTCCCTCTGGGTGTTGAGTGTCGAACACCAGCGACTTGGAATCTTGGACGTTGAGAGTGGCAGAACAAACGGCAGATCCCAGGGAGTTGGTTGCCTTGCAAGTGTAAGTGCCCGAGTCCTGTGCTTTGACGTAGCTCAGGTCAAGAGCGACGAATCCGAAGTCATGAATGGTGTTGACCCGGTTGGCTTTCTGTATGGGGAGTCCGTTGTGGAACCATTCCACTTTGAGGTTGGGGTCGCCCACTGGTATGAGCTTACACTCAAAGTGAGCACTCTGGTTTTCGTTGAGGCTCAGGTTCTTCATAGCTTGAGTAAACACGGGCGCTTGCGTGGTGATCTCTTCCACGACATAGGCGGGCCTTGCTTGGCGTCCGTAGTCCTCGAGTTGCTGGATCTTTTCGTACGCTTCCTGGTTTTGCGTGTCCAGTATCAACGACTTTTCGGCGTGGCACTTGAGTTGAGCAGACACGACAGCTTCTCCCAGCTGGTTCGTGGCTTTGCAGGTGTAGGTGCCGGAGTCTTCAGCGTAGGTGTTGAGGATGTCCAGAGACACGAAACCGAAGTTGCACATCTCGACGAAACGAGATCCTGCTGGCACCGGCTGGCCGTTCTTGAACCACTCGACCTTCAGCTTGGTATCTCCCACGGGGATCAGGCGGCACTCCAGATGCGCCTTCTGTCCCTCTTGGACCACGATGGATTTGGGAGCTGATGTGAATACGGGCGCTGTCGTAACTGACTGGTCCAGATCGTCAGTGTCCCTCTCATAGTGGTGGCCTTCGAGGGCCTGGATCTTTTGCAGTCCCTCAGGGTGTTGAGTGTCCGTGTCAATGGAAGACTTTCCTTCGACATTAATTGTCGTGGACAGCTCGGCCTTGCCAAGAGAATTGGTGGCACGAACGGTGTAGGTTCCCGAGTCCTCAGGAACAAGGGACAGAATGTTCAGTGCGACGTAGCCAAAGTCATGGAATGGCCTGTACCGATGACCGATGGGGAGAGAGACGCCGTTGCGGAACCATTCAACCCTGAGGTCAGCGTCGTTGACAGGCTCAAGTCGGCACTCCAGGTGGGCGTTCTGTCCTTCGCGGAGGCTCAGAGCCGTCATGTTAGATGTGAAGGAGGGCTTGGTCTTCACGGTTGTCTCCTCAATTATCTCGCGGCGGTAGCGAGATTGGTCTTCCAACTGCTGTATTTTCTGAAGGCCTTCAGGGTGCTGAGACTCCAGAATGAGCTGCGACTTGGCAAAGCAGTTCACGTTGCAGGACGTCACATTTTCACCGAGAGAGCTGGTGGCCTTGCACGTGTAGATGCCAGAGTCCTCGGGATAGAAGCTGAGGATGTCCAAGGCGACGTAGTCAAACTCGTGCGCTGGCCTGAATCGATGGCCGACTTTGATGGGCACACTGTTGCGGAACCACTCGACCCGCATGGTGGGGTCCCCGACAGGCTGCAGCCGGCACTCGAGGTGCACGTTGGCTCCCTCCACGGTCTCCACGTTGTGCAGCGGCCGGCTGAAGGTGGGCTTCTGTGTGATGACACTTTCCACGTGCTCCTGGCGGGCGTAACGCGAGTGGTCTTCCAGGTGCTGGATCTTCTCCCAGCCTTCGGGGTACTGCGACTCGGTGATCACGCCGGACTTGCCGATAACCTTGATGCAAGACGTTGAATGCGCCGATCCAAGATGATTGGTCGCTCGCAGAGTGTACTGCCCGGTATCTTCGGGCACCAGGTTCAAGATTTGAAGAAATGCTCGTCCAAAGTCATACTTGGTAATGAACCGATGACCTGTCGGCAGCGGGCGACCATTAAAGAACCACTCCACCTTCATGGTAGGGTCCTGAAGAGGCTCGATGGCGCATTCTAACACAATGGAATCACCCTCTTTTAGGGGTCCCTTGGGGTCTTTAAGGGGAATCTTGAAGTACGGCTTAGTCCTTGTGACTTCTTCGATGTGGAAGTAGCTGCTGTGATCAACTCCAGAGCCCTCAAGGTACTCAATTTTCTCAAGCCCTACTTCGTGAATCGCGCTTGTGTCAACAGAGGCTTTCGCTTGAACTGTTATCTCCTTGGTTAGTACATCCGTTCCAAGTTTGTTGGTGGCACGGACTTCAAAAGTGCCGGTGTCCTCAGGGAATGTGTACAAAATGTCCAAAGCGACGTAGCCGAAGTCGAAGTACGTCTTGAAGCGATGACCGGTGGCCAGTGGACGACCGTTGTGGAACCACTCAACCTTCATGTTGGCGTCACCCATTGGCTCCAGGCGAGTCTCGAGGTGAACACTCTGGCCTTCCTTTATTGCCGAGGGCCCTTGGAGCGGTTGCGTGAACTTGGGTTTCTCCTTAATGGAGACCTCTTCGAGTTCGGATCGTTGGTAGTGCGCGGAATCTTCCAGATGCTGAATCTTCTGGAGGCCGCCGGGGTGCTGAGTCTCCTTCACAATTGACGCTTGAGACACGCAGACGAGGCGGGCCTGTGTAGTAGCTGAGCCGATGGAGTTGGTCGCCTGGCAGGTGTACACGCCAGAGTCGCGGGAGTCAACTCCGCGGATCGTCAGAGACACGTAGCCGAAGTTGAAGAAAGTCGTCGTTCGAGAGCCTGCTTCAAGCGGACGTCCATCCTTGAACCATTCGACGCGCATCGACGAGTCCCCGGTTGGCTCGAGGCGAGCCTCGAAGTGCACTGTGCTGTTCTCCTTAACGTTCAGCTGATCACGCAGACCTGTCTTGAAGTTCGGCTTCATAGTGGAAACCGGTTCGAAGATGATTTCCTTTGGCGCGTGGAGGCTCATCTGGTACGCTTCCAGCTCCTGCGTCTTCCTTATGTAGGCCTCCTGTTCTGGAATGCCCAGGTCTCCTGTGACAAGGCCTTGCGCTTTGACTCTCAGGGTCGCTGTGGAAACAGCCTCTCCCTTGGCGTTAGTGGCCTTGCACATGTACACCCCGGAATCCTCACACCGGAGTGACATGATGTTCAAGGCGACGTAGCCAAAGCTGAAAGTGCTGTTGATACGCGAAGCTGTTGTCAATGGCTGGCCATTGAGGAACCATTCTACCTTCATGGTAGAATCGTTTACAGGAGTCAGCTGGGCCTCGAAGTGGCAGTTTGCTCCTTCGAGAAGGTTGTCTTTGTTGCTCAGCGGTCGAATGAAGACAGGCTTTGTTAGGCTGGCGGTCACGTCGATGGACAGTTGTCGCGAGTATTTCTCGGCGTCNNNNNNNNNNNNNNNNNNNNNNNNNNNNNNNNNCTGGATTGCTTTGCGTTCGATTGCCGGCCGAGCTGTGCATCGAAGTGTGGCCGTGGTCGTCGCCTCTCCGGCTTCGTTGGCTGCACGACAGACGTATACGCCAGAGTCCTCAGGATAGACGTGCAGCAGCGTCAGCGCCACGTAGCCGAAGCGGTACGTGGTCATAACTCGTGAGCTCGCTTCGATGGGGCGTCCGTTACAGAACCATTCGATGGTCATGGTTTCGTCTCCGATGGGAATCAGCCTAGCGTCGAGGTGGCCACGCTCGCCCTCGTGCAGGACCAGGTCCTGAAGGTGCGTCGTGAAGGCCGGCTTCTGGGGCGCCCTCTCGTCATCTTGGCCACGCACCGGCTGGTAGGCGCGCTCCACATGTCGCAGCCAAATGGTTTCACGTGGCGGCGACTTTTCGGGTGAAAGAACCTTGGTCGGCTTAGGCAGATGAAGCGCCCACGGCTCGGTGTGCTTCTTGGGTTCAGCTATCACGTGCAGACGCGCCCTCGTCGCTGTCGAACCAGCTTGGTTTTGCGCGGTGCACTGGTACCACGCGGAGTCGGCCACGCTAGCACGGTTCAGGTACAGGGCGGAGCTCCCGTCTTGCGTCTCAATGATGAGGTTGGGTGGCTGCGAGTGGACCTGGCGGCCGTCCTTCTGCCAGCTGATGCGC

>AAUF27145

CGAGCCACGCGTCTCCTGGTCCAAGGACCTGCTCCCCGTCCGCGACGCCGCCAAGCCCTCGCAGGAGCAGGGAAGGGCGCGGCTGGTGCTCAGTGGCGCAACCGAGAGCGACTCGGGCACATACACCGCCGTGGCCAAAAACAAAGCCGGAGAGACGGCCTGCTCCTGCCAAGTGAAAGTGGCCGAGGACGCCGCTCCGCCCGAGCCTCCGCGTGTGCTCAAGGCCCTGGAGGATCTGGAAGTAAAGCCTGGACCGGACCCTATCACGCTGGAATGCATCATCGTCGGCCGGCCCGAACCAGAGGTCATCTGGTACCACAACACCCAACCCATCAAGGAGTCTGAGCGGGTACGGCTGCTGTTCCGCGGGGACAAGTGCTCCCTTGTCCTGAACGGCGTCAGCGCCCAGAACGCCGGCACCTACCGATGCTCGGCGGTCAACCCCATGGGCAGCTGCTACACGGAGTGCAACATGCGCGTGCCGCTTTCTGCTCCCGTGTTTCTGGAGCCGCTCCGAGACGTGACCACGGATGAGGGCTGCCGCGTGGTGCTCACTGCAAAGCTATGGGCACCCGAGCCACCTTTCGTCCGCTGGTTTAAGGATGGAAGAGAGGTGCTTCCAAGCCCAGACTTCCAGGTCAGCCACGATCCGGACGGAACTGTGAAGCTACTGATCCCGAAGGCGGCGGCCAGCAACAGCGGCCATTACGAAGTCGAGGCCAGCAACCCCGGCGGTCGCACGCGCACGGGCTGCAAGGTGCACGTTCGTGAAGCTCAGAAGGCGGTCCAGGCG

>AAUF46589

TCGGAAGCCGAAGATAACGGGACTCAAACCACACGTGAGAAGTCGTGGGTTCCCGTCCACAAGCTCATTATGGAGATTTCTGCTGACGTCACGGTAGCCGAAGGAGACGAGGAAACGCTCAAGTGCATCCCCAGCGTGTCTGGGGCCAGCGTACAGTGGTTAAAGGACGAGACGTTGCTGAACGGCGATATACCCCTGGATAACCTGCACGTGGTGTCCCCGGGGATGTTGTGGATCCGACGCATGCACTCTGACCTGGCAGGAAGCTATACCTGCGTAGTAGCCACGAAGGATAGGCAGGCCCGGGGTACTGCACAAGTCGCCATGACTGCACCGAAACAACGCGACCAGTGCGACGTCTACTTCCACAGGAGTCCCAAGGACGAGCATCTATTCCACGGTGAGACGGCCATCATGCAGTGCGCGGCTCAGCCCCGTCAGCGGCCTTCGTCCGACGTGGAGATAAGATGGCTGCGCAACGGCCTGCCGTTCCCGACCAGCAGCCGTTACCGCGACTTCGGCGACGGCCTGGTCTACATCAGCGACGCCGTGCCCAAGGACTCGGCCGTGTATACGTGTGTGGCCA

>AAUF40295

CGCTGCTCTCCGGGTTCGAGCTGCCGGACTCGCACGTCAGAACCACCGTGTCGCCTTCCTTCGGAGTTTGAGGAACCACCGTGATCGAGACAACTTTTGGAGGAAACAATACTGTTGTCTTCACGGACGTTGTGAGGGGCTCCGTGGTGGCCGAATTGGAGGCCTTGCAGTGAAACGTTGCCTGGTTGTCGTCGGGCTGGGCCATGATTTCCAACACGCTTGACACTCCACTTCCTGTGACCGTAGACAGCGATGAGTACTCCTTGGTGCCCTTGTACCACTTGACCGATGGCAGCGGGTTTCCCCCGATTGAGACGCACTTGATGCGCTGTATCGAGTTGGCCACAATAGGGGTGCCTTCGTCGTAGCCGACTATCACAGGCGCGCGGGGCGGATATATGACTTTGACTGTCACCGTTTGGACGACTGTTGCTTGCAACTTCTGGTTTTCTGCAATGCAGGTGAAAGACTTGACGTCGGGGTCCTGCCTTGTCAACGTGATGGTCAGGTTTGAAGACGTGATCCAGCCATTTTTGTTCTGCACCGTGGACTGATCGGAGGCCGGCTGCACGGTGCGGCCATCGAGCCTCCAGGACACCTCAGCCGCCGGGTTGCTGGGTGCCGTGGTGCATGAGACGGTCACCAGGTCCCCGGCCTTGGCCTCCTTAGGCGCCGTCATGAAGACGCGCGAGGGTGGGAAGAGAACGCTGAGCGTTACCGACGCCTGCGATGGCTGCAGGGTGACCAGGTTGCTG

>AAUF43082

CTGGCAGAAGTAACGTCCTTCATGACTGCTGTGCACTTGGAGGAAGCTGTGGTTGGCCCTGTGACCCAGGTCATTGTGTTCCTGGCCCTCACGCCACCAGCGGTACTCAGGACGGGGCCACCCAGGTGGCCGAGCACCACAAGTCAGCACAAAAGGACGACCAGCCACTGCTGTGGGGCCCTGAGGAGTAATCTCTGCTTCTCCTGGTTTGTGTCGCACGTGCAACACAACAGAGTCGTTTCCAACGACTTCGGTGTGGATGGCAGTGGCAGAGGGTCGCATGATGACTGTGGCCTGACAAACGTAGGTTCCGGCATCCTCAGCAGTGACACGATCAAGGCGCAGAGTGTCTCCTTGAAGTGCAAACTGGGGGTCACCTTCCTTGGTCCACAATACAGAATGTGGCTCAGGGTTAGAAGCAACATTGCATTTTATGGCTATATTTTCTCCTTGAGAGGATTCCTGCTCTGGAAGCACGTTCACACGAGGCCCATACAGCACACTAAGACGCAGGTCGGCTTGAGTGGGCTGCATAACTCCATTATCAGCAACACAGGCATAGTTTCCACTGTCTTCAGGAGTCACAGATGGGATGGTGTGGTTGTACGTGTTGGACAGCAGCTGGCCTCGCTTCATCCATCGGACGGAACGCACAGGAGGATTGGCCAGCACAGAGCATGTCAGTGTGGCATCAGACCCCACCATGACGTTCAGAGGGTTGTAGGGCCCGACAGTGACCTTGGGTGCATAGTGGACACGTATGGGGGTGCGCGTCTCGTACGCGCTGCCCGGCGTCATGGCGCGGTTGGTGACGCGGCAGCGGAACTCGG

>AAUF20277

CTGGACCACACAGCGCCTGGTGCACGTGCTGAGCAGTATTAGCCACTCCCAAGCAGGGTCCTACCAGTGCCTGGCCGAGAATGCAGCTGGCGCGCTGCTCAGCGCCAAGGCCCGGCTACGGGTGGCACACCTAACACGAGCCGAAGAAGTCCCTGAAACAGTTCCTGTGAACGCTCGCAAGGGTGGCGACGTGATCTTGGCACCGCCGCTTGTTGACAGTGTTCCGCCAGCGACTGCGGTCTGGACACGGCTGGACGGCAGAAACTTGGACAGCAGAAACTTTGCACAAACACAGGATAACCGCCTGGTCATTCTTGACGTCAGCCCCAAGGATGCCGGCCAGTACCGGGTGGAACTGACCAATCCGCACACGGGAGACAACCTCTCGGGACCAGTGGTGGAGCTGACCGTCGATGACAATGAAGAAGACCAGGCAGAACTCTCCATTGTTGTTCCGCCATCAGATCGAGAGTTCAACAACCTTGGCAATGGCTATGACAGCACTCTCGAGTGCATTGCCTCTGGCAGGCCTCTGGACCAGGTGCAGATTGAGTGGCTGAAGGATGGTAGGGCCCTGGGTGAGCTGCCACACGTTCTCACCCACTGGAACCGCACCCTGACACTGCTGCGCCTGGGGCCCGGGCACACTGGCCGCTACTCCTGCCAGGTCACCCTTCGACATGCCCCTGAGGACTCCTCTGCTGTCGTGGCCCATGCCAACGTCACTGTGTCAGTGCTGCCCTCCTTGAGCCAGAAGGTGAATGAGGAGACGGCAGTGGAACTGGGGCAGCAGGTGCGGCTTCCCTGCGTGGCTGAGGGCCACCCAGAGCCCCAGGTGCACTGGCTCCTGGATGCGCGGCCTGTGGACCAGAGCACAGGGCACTTCCACGTTGGTGACGGCGGCAGCCTCGAGATTGGAGCCCTGGCTATGGAAGATGCCGGGGTGTACCAGTGTGTGGCGGAGAACCCGCTGGGTGAAGCGCGCGCTTCTACCTGGCTTCATGTAAAAAGAACTTGCCATTTGCCATCTAC

>AAUF55753

CACGTCGCACGATAGAGAAACCTCCTCGAAGGGAGCCGTGTACACAACCTTGGCGTGGTGCTGCTGCTGCGGGTTTTGGCACCGGGGCGAGTGCTGTATGCGTATGTGCAGCGTCTTGGAAGTAGTCACTCCCTCCGGGTTGGTGACTGTGCATGAGTAGTTGCCAGAGTCAGCCGACTGAACGTTACGTAGAACCAGGTAGCGCTGGTTGACAATCAGGGGACCCCGTGTCATCAGTGAGGAGGTGCTGTTGGCGGTGTGCTGCGGCTGCAGCCTGTGATCGTGCTGCTGCTGCTGCTGGT

>AAUF55189

CAGAAGTCTCTGAAAAAGTAGAGCCGAACGATGTCGTGGTCTTCACAAGCTACCTGCACCTGAGGAATATCCAAAACAAGGATGAGGGGCGGTACCAGTGTGTGATTCGAAATCAGTTTGGCTCTGTCTACTCCAACCAGTCCTACATTAGTGTGTATGTCCTGCCTACGTTTGTCAAGACTCCATCCAACTTGACGGTGCGTGCGGGAGGCACAGCGCGGCTTGAGTGCGGAGCCACGGGCCAGCCAACACCGACGGTGTCTTGGCAGAAGGACGGTGGGGACGACTTCCCAGCTGCCAGAGAGAGGCGAATGCATGTCATGCCCACTGACGACGTCTTCTTTGTTGTGAGTCTCAAGGCAGCCGACTCGGGAGTCTACACCTGCACTGCCCGCAGCCGAGCCGGTGTGGTGCGGGCCAACGCCACACTCACCGTGCTTGAAGCACCAGCATTTGTGCGCCCCATGCGGAGCAAGCAGGTCGCGGCTGG
