# Supplementary material for: A repertoire of protease inhibitor families in Amblyomma americanum and other tick species: inter-species comparative analyses
Source: Parasit Vectors. 2017 Mar 22;10:152. doi: 10.1186/s13071-017-2080-1 (PMC5361777; doi:10.1186/s13071-017-2080-1)
Supplement: Supplementary file 3 — FASTA sequences for Amblyomma americanum contigs from Illumina sequencing, by PI family. (ZIP 638 kb) [file 13071_2017_2080_MOESM3_ESM.zip › A. americanum I51.docx]

>AAFM819

TCTGCTAGTGGGACGCCAGCCTTTTGACGGCAAGGCAGAGAGGCATTAGAAGCCAACGTAATGATCTGTTCAACTAAGACAGAACATTCTTTTTTTCTCTTACTGCCGAATAGCTGCCATGTTTGTTTTCAGTAANNNCCGACTCTACGCCACAGATGGCGTTAAAACGCTGACCTTGTTGCGAACAATCTATCCGCGTGACAAACGCTCAAGCTCTTTGTAGTTTTGCGTAAAAAAAAGCATACCAGCGATTCTTGCACTTAAAAATTAATGATGTTTCGAGCTGTCTGTTCGCGAGCAATCTTTGCGTTCGGCCCTCTCAGCGCTTCTTTGACCTCTTCCCAGAGAGCAGCATCTGCATCCGGGTTTTCATCGTCGTTTCGCCGTTTGCTTCAACAGAGCAGTGTGTACCTTCAGAAGGGCACCGGGATGACAAAGATGGAGGACAACCAAGTTGTTCCGGACGTGATCGACACGGTCCCGAAAGATACCGTTGAGGTGACCTACAACGGTGTCTCGGTTAACATGGGAAATACGCTGACGCCGACGCAAGTCCAGTGTCCACCAACTGTTTCTTACCCGACGGACGCTGGCGCCCTTTACACGCTATGCATGACTGACCCCGATGCTCCGAGCCGGCAAACACCCAAGTATCGGGAGTGGCATCACTGGCTTGTGGTAAACATTCCTGGCTGCAAAGTCTCAGATGGAGAGACTCTGTCTCAGTATGTTGGTTCAGGACCTCCAAAAGGAACAGGTCTCCATCGGTATGTCTTTGTGGTTTACAAGCAGCCTGGGAAGCTCTCCTGTGATGAAAAGCGCCTCACAAACCGCTCTGGAGATCACCGAGGTGGCTTCAAGATCCGAGACTTTGCCAAGAAGTATCAGCTGGGAGAGCCAGTTGCTGCAAACTTCTACCAGGCAGAATGGGATGACTATGTGCCCAAGCTCTATGAGCAGCTGAGCGGCAACTGAGATCAAAGTGCACTGATTGGCCTATGGGGTTTGCCCTTTCCTGTCTGTCCTTACTGCGCTGTTCTTTCCTTGTTCTTTGCTTTCTGTTGCATAGCCCCCTCTTTGAGCTCAAGCACTTTACTGTGCTCTGGCATGCTCTGAATAAAGCAGAATTTATGTTGGTCTTAAACTCCTTCCTTGATTATTCCCAAGCCTCTTTCTCTTTTCTACATACACTACCCTTATTAATACCCTGAGTATGGTGCATGTTTACAGTGGTGGAAACATGTCTAAATTTTTCGCTTAAGTGCAGAAGATTTTTGTGCACATCTATTAGCAACTGCATAGTGCAGAATTCTGCTGTGATAGAGCAGGACACATGAATGCATGCCCAACATTTATTGTCTACACTGCATTGTGCACAATTCTGCTGTGAACATGCCCAAATCACCAGATTTGTCTGGAGGTGTCTGGTACTTTCGTCTTGCTATGCTTGCACTATGGGTCTCCTTCCTAGTAGAAAATGCTGAAATCGGCATGATAGCACAGCTGTAAGTGCATGGCCTATTGCAGTCATATGTGTGGGCTGGTGCACTTTGACAGTGTTGCAGAGGTACAGGCTTGTGTTGATGGTCACATTGTGCTAAGCTGACATGACATTGATGAATGCATGCCTGAACCAATTGCGACAAATCCGTGCAGTTATTGTCAAAAATAAGTGTGCACTATGCAAGTTTGCTCAGGGGCATGCATTCAGAGGGTCAGTCAAATGGCAACTTCCTTTTGTGTACCGCAGCATTTTATTTTTTTTTGTGCTCGCCTTGGGCTAAGGAATGCCCTTTGGCATTGCCTAATAAACACAGTTTGTGCTGGAAAAAAA

>AAFM2248

CTCGAAGTGACTTACGCGGGCGATTTGAAAGTGACGCTGGGCAACACGCTGACGCCTCAGCAAGCCAGCACTGCGCCTACGGTGAGCCTGAATGTTCCGGTTGACTGCGAAGGTCCGTACGCGCTGCTCATGGTGGACCCGGACGCGACCAGTCGCAAGAACCCGGTGTACCGCAGTGTGATGCATTGGATGGTGTTGAACATCAACAGCACGGAGAAGCTCCAGGAAGGGGACGCCGCGTTGCCTTACAACGGGCCTGCTCCGCCCAAGGGATCGGGACTGCACCGATACGTGTTCCTGGTGTACTGTCAGCGCGGAATGCGCCTGCAAGGCGAAGATCTGGCGCCGAAGGAAAGGAAGAACTTCAACCTGGCCGAATTCGTCAACAGGACGAGCCTGGGCACGCCGTTGGCTGGAAACTTCTTCGTCGCTGAGAACCCCGCAGCTGTGTAGTT

>AAFM3137

GAAGCCAGCAAAATCGCACCGGACTTGATCGAGGCGATACCGAAGGCCGTCTTCGAGGCGACCTTCCCGAGAGGACAGGTCTCCATGGGCAACTTCTTCACCCTCGACCAGGCGTCCAAAAATCCCTCCAACATCGCCTTTCCGCGGACCCCAGGCGCCAAGTACACGATCGCCATGCTGGACCCGGACGTGCCCACTATGAAGGACCACAAGTTCTCGCCGATATTGCACTGGCTTGTGGTCAACGTGGAGTCGGGGGACGTCAAGGCGCCGGTAGACTTCAAGACCGGATTCGAGCTCTACAAGTACCGCGGCCCCAAACCACCCATGGGGGCGGGGCCACACAGGTACGTCTTCCTGGCCTACAAGCAGTCCAAGGCCATTGACTCGCCTCAGTCGCTGATCGTCCCGTTCGAGAAGAGGAAGAACTACAACCTGGCGAAGTTCGCCAACGACCACGGCCTGGGGAAGCCCATCGCCGTGAACTACTTCATCTCCGAGAACTGCCACACCGCCGGATACACGCCATTCGTGGTCTGCACTACNNNNNNNNNNNNNNNNNNNNNNNNNNNNNNNNNNNNNNNNNNNNTTGCTATGGCTCTTACGGGACTTGTCGGCCGGTTAGTTTTACTGCAAGTTTACATGTTGTGAGTGGACGGGACCTGTCTTGACTCGCCTAGTCTCGCAACGCCAGGCGCGTAGACGCGCCACACATGGACGTGTTTTTAATTTTGTGTTTTGAGTGCTGCTTGTGATGCGCCGGACTACGACGTGTGACTGTGAGCTGTCATGAAACAGTGCAGTGTGCTTTGTGGACACTTGGTAACCAGGATTACGATGACTGCAATGGAGGAGCGGGCAATAATGCGGTGTTCGTGAAGTATTTGAACCGCACCTGTTGCAGTAATGAAGCAAATTAAAAATTCAGCGCGGCCTACACTTACGAGGAACAAGAAGAAAAAAATTAGCCGCCGCTGTATCACCGCCGTGCTGCTGCCGAGCCGTGCAAAATTAACACCTAGAATCGATAAAGTTAATAAAACCGCTTGCGTTAATGCGTCGGACTCACCAATGCTTGCCGGTATTTTTGGAATTGCCCTGAATGCGCCAGCCTGGCCTATGCAT

>AAFM4147

CAACGTTTTCAGGCGTTTTCCGCGTAAAAGAAGGTGCCTGCAGCGGGTATGCTCGAGGGCACCTTCTGAGCGATACTTGTCACCTGGAAGTTGTTCCTTTGCTGGGGAGCTACGTCTGCGCTATTCAGTCGCTTGCCGTCCTGACAGTAGACCAGGAGGGCGTAGCGGTGAGGTCCAGAACCTTTGGGCGGGGTGGGTCCGTTGTACGGCATCGCGACCTCGCCATCTTGGAAGCGGGACTTGTGCTCGGCGTTGATCACCATCCAGTGGAGCCAGCTTCGGGCCGTGGGATTTCCCTTGTTGGGCGCGTCCGGGTCGACCATGACGAGTGTATACGGCGGGCCGCAGTGTAGGAAGCCGTCGAGGCTCACCATAGGGGCACTGGCTGCCTGTCCAGGCGCAAGCGTTTCGCCCAACTTCATCACTGTGGTGCTTCCGAACTTCACGTCGATCAGGGCGTGCGGGGCGTCTGCCAGAGACAGGTCATCCACGAGGCCGGACGTCCTCCACATGTTGATGTCCTCCGCCGACAGTTCCGTCAGTTTCGGAACTTCGTCAGTCTCGGAGGGACGTTCCTCTGCTGGGCTGTGGCTGCATAGAGTCAGCAGAAGCAACGTCGTA

>AAFM5014

CGGACGTATGGACATCCGCAGGCATAGTGAGCGACCTCTCGCTTCCAGACACGCTGCGTGCCGATCTGGAAGTGAGGTACGGGGATCATCTCTCTGTGGTCAAGAACGGCACTCTGACGCCCGCGCAGACTGCCGAGGCCCCGACGCTGGTCAGACTGAGGGGCGCCATCAACTGCATTCCGCCCTTCGCACTCGTCATGTTGGACCCGGACGCACCGAGCCGTGAGAACCCCACAGCGCGCAGTAGGCTGCATTGGATGGTGCTGAATGCGGACAGCACGAC

>AAFM5015

TGACGATGACACTCATCGGTCTTGCGACCATACTCCTGTTGATTCTGTTCGGCAACAACCTGGGAGAGGAGAGCTCCGCTGCCAGAAAGGCCGTCCCGGAATCGGCGGGCCTTTCAGAGGCCGACCTCAACATATGGAGGACCTCTGGCCTGGTGGACGACCTCACGCTGGACGGCGCTCCGCGCCTTCTTCTCGAGGTGAAGTACGACAGCAAGACCCTAACGAAACCGGGCGAAACGTTGACGCCTAAGGAGGCCGCGAAGGCCCCGTCGTTGACGTTGAAGGGCACCCTCCACTGCAAGTCGCCGTACGCCCTGCTCATGGTCGACCCGGACGCCCGCAGCAGAGCGTGTCCAATGTACCGCAGCTGGCTTCACTGGATGGTGGTCAACATCGGCAACACAGCCACCTTCCAAGACGGCCAGGAGGCGTCGCCCTACTTGGGACCCGGACCACCCAACGGAACGGGACCCCACCGCTACGCGTTTCTCGTCTACTGTCAGGGCGGCACGAGGCTTAACGCCGCTGAAGTGGCACCCAAGGATAAGAAGAACTTCGACGTCGACAAGTTTGCTCTGACAATGGCCTCAACAGTTCCCGCTGCGGGCAATTTCTTTTACAGCGAATTTACCGCGCGACGTGCCTAGGACAGTTCTTTCGAGCTTCTTCAGCTGGAAGGTCTGCAGGTTCGCTACGAAAAGATAGATCGACATCTAATCGAAGCCTGACCAACCACTGATCTACAGCTGACCGGCGACGGACCGACACGTGAGAACTACAAGCGATCGTATGTGGGCGGCCCAGCAGCATTCGTGTCATAGCGTCGCCTTCCTGGGCATATCACGTCCAATGCCGTTGTAGACGGTATGAGCATGCGCACTTTGATACATTCGACGTGACGTATACTACGCAATGCTTCTCNNACGTAGTTTAAACTGTCAAAGTACTGAGACTTATACGAAGTTAAT

>AAFM6224

GCTGCGCACGGCATGGATGGCATGGTTTTGCCAGTGGTTTAGCGCTTGCTACTGCTGCTTCTGCCTGATCGAGGGTTGTGTCTTATGAATGTGTTACTTTGCAGCAGAGTTCGTAAAATACCTGGACGCAACTCTTTAAAATTTCAGCAATGGCTACACTGGCCGCATTTTTGAAAAACTTTCGGGCTTTGAGGCGCCTTGAGCCTGCAATGGCTCGTTGTGTTCGGCTTCCGCCAATGGATACCTTGAAAAGTTTGGAAGAGAAGATGCAAGCTCTAAAAGCAAAAGAGCCAGTGTTTGAAGAACGAGTCAATATTGGTTTCAAGGTCGCACCTCACAGTGTCAATAAACAAGAATGGAAAAGACAACGAAAGATAATCCAAGAGAACAAGGACAATTATGACCTTGCTGAGAAGTCACGTAATGGAACCTTGAAGATACAATGTGAAAAAGCCAGGCAAGAATGGAGAAAAACCTATGCACCAATGCACGTGAAAGAAGTGGCAGACCACTACGGTGTCTTCAGAGACCTTTATGAGTTTGGATTCTTTCACCCTGTTATTCCAATGGATGTTTTGTACGAGTATGATGCAGAGTATTTCACACCTGTTCATTATGGTAACATAATTCTTCCTTCTGAGGCTGCAAAAGCACCAACAGTTTTATTTGATTCCGAGCCTAACATGCTTTGGACACTTGTCTTGACAAGCTTGGATTCTCATCTCCTTGAAAATGACAAAGAGTATCTTCATTGGTTCATTGGCAACATAAAAGGAAACCAGGTTCCAAGTGGTGAAGTGGTGTGCGACTACATGCAGCCTTTTCTTCCTCGTGGTACTGGCTATCATCGGTTTGTGTTTGTTTTGTACAAACAAGAAGGATTAATTGACTACTCTAACCAGAAATTGTCTGCAAACAGTACCAGCCTGAAGGAGAGGACATTTAAAACCTATGACTTCTACAAGGAATTCGAAAATGTGCTCACACCTGCTGGCCTAGCATTCTTTCAGTGCACCTGGGAGGACAGCCTGGTGGACTTCTTTCACAAAACTTTGAAAATGCGAGTCCCCACTTTTGA

>AAFM6412

GGTACAGACAACGTCGCACATATCACAATACGCGTTTTCCAGTTCGACAAATGCCTTGAAGGTGATGAGCTCTGTGTCGAATCGATCATAGGAGAAATGCCAAGGCGGCTGTGTACGCGGGCGCCTTCAGTTCATCACTGACGGTGACCGAGGTTGTCTGCTCGCCGGCCTATTCATGACTAAGCCATGGAGGACGTTCTTGTGGAACATGCTCGAAGGCAACGTTTTCAGGCGTTTTCCGCGTAAAAGAAGGTGCCTGCAGCGGGTATGCTCGAGGGCACCTTCTGAGCGACACTTGTCACCTGGAAGTTGTTCCTGTGCTGGGGAGCTACGTCTGCGCTATTCAGTCGCTTGCCGTCCTGACAGTAGACCAGGAGGGCGTAGCGGTGAGGTCCAGAACCTTTGGGTGGGGTGGGTCCGTTGTACGGCA

>AAFM12786

TTCACCGCCACCAGTTGTGTCAGGTTCGCCGCTTGGACAAACTTCTTGAGGTCGAACTTGGCTCGCTCCTTGCCGAAGTCCTTCACAGCTTGTCGGGTGAGGACGTTCTTCTGTTCGTACACCAGAAAGACATAGCGGTGCTTGCCCGTGCCCCGAGGAGGCGTTGGTCCCGCGTACTTGGTCAACGTCGTCGTGCTGTTGTCCGGCACGTTCACCACGAGCCAGTGAAGCCAGAAGCGCATCTTNNNNNNNNNNNNNNNNNNNNNNNNNNNNNNNNNNNNNTTTTCTCCACCGAAGTTAACGTTGGGCAGTTTGGCGGCCTGTCCTTGTGTGAGTGTGTTGCCCAGCTGCACAGCGGCGCCTCCCGGGTAGGCGACTTCGGCCGCGCGGCTGGGCACCGTCGCTATCACGTCCGGCACGACGCCCGCCTTCTTCAGGTTCAGGTGCAGCTCATCGGTCGTCACGCACGAGCCGAACGGAGCCCACAGCACGGCTAGAACCGCGGCTAGGGTAGCAAGCGCGGCGGACCTCGCGTGCATGGTGCTCGATGGAACACGTCCAGCGGTATGCGATTCCGTTGATCCCGTTCTCTGACACGCGCAGACCTAGATGGAG

>AAFM14357

CGAAGAGGAAGACGAAATCTCTGACGACGAAAGCGTTTGGTTCAAAGCGCGAATCGCGACAGGTGCCACGCGTCGACAAAGCACCCGGGTCGGCCATGCCGTATCCGTCACTCATTTTGAGCGTGGCTCTGGCGGCCAGCTTCGTTCCACTCTCCAACCTCAGCCGCACCGGTTCCCGCGTCCGAAGGGGGGAGGACCCACCCGAGCTCTTCGCCAACACGCTTACCGAGCTCAAAAAACTGATGGTGACCAAGCTCCACGAGGACCAAGTGATCCCAGAGGTCATTACAGAGACACCCGAGCAGCCTGTGGCCGTATTCTACCCGTACGCCAAAGTCATGATGGGCAATCACATCGACCCACCAACACTGCGCTTCGTTCCACAGTTCTTTTTCAAGTCGAAGCCCGACAGGCTCTACACGCTGATGCTGCTCGGGCCCGACTACCCGACTCGTGAGGCCCACACCGAGAGGAACCGTTTGATGTGGTGCGTGACCAACTTCGCGCGCTTCAACGCCACGTACACGCTTCGACCCATTGGACTCGTCTCCTACGAGCAGCCGAAGCCAGCGAGCAACAGCGGCAAGATGCGCTTCGTGTTCCTGGTCTACGAGCAACCGACGGACAAGGACTGGCGCAAGATGCTCATCGAAACGCCGAGGCCGCCAACAAACTTCATGCTCGATTGGTATGTGGACAGGAATGGTCTAACCTTGGCGGCGGTCAACTACTTCATTATCGACACGGGACAAGAGGGGACGCAGACCAACTCGTCGTTCGGAAGGGTCACGGGTGNNNNNNNNNNNNNNNNNNNNNNNNNNNNNNNNNNNNNNNNNNNNNNNNNNNNNNNNNNNNNNNNNNNNNNNNNNNNNNNNNNNNNNNNNNNNNNNNNNNNNNNNNAAAAAACGAAGCCGGGATTGGTGAGAAGACGACTGACGCTGGCGCTAAAGTGGCTCGAGCTGGTGAAAAGACAGACGGACCTGGTGAAAAGGAAGCTCGGAGTGATGGAAAGTCAACCGGCGCTGGCGCGATTGAGGCTGGCACTGGGGAAAAGGCGACTAGAGCTGGCGGAATCGAGGCTAGGGCTGGTGAAAAAATGATCAGCACTGTCGAAAAGGGAGCTTCGGGCGGTGCGAAGGCGACTGGAATTGGTTCAAAGAAACCTGGGACTGAGGAAAAGGCGACTGGTG

>AAFM16255

CTCAAGCCGTCGCCACCCGGGCGGAGGACAAGCAACATGGCCGCCACCGAACCCGTTCAGAAGCTCAAGCACCCCGTGCCGCCTGCGGCCAAGGCCACGGGCCCCGCAGCCACCGCAGGCGCCGGGGCCATCGCTGGCATGGAGGCGCTGGTGCCCGACGTCATCCCCAAGATGGCCAAGCAGGCGCTGGGGGTCACCTTCCAGCGGGGCGTCGAAGTGCGCATGGGCAACACCCTGACCTGCGACCAGACGGCCACGGCGCCCGAGAGCATCTCGTTCAAGGGCCCGTCGAGCGCGTACTACACCGTGATGATGGTGGACCCCGACGCGCCGAGCCGCGTCTCGGCGCGTCTTCGCCACTGGCGCCACTGGCTCGTCATCAACGTGCCCAACAGCTGCGACGTCAAGGCGGGCGACACGATCACAGAGTACACGGGTCCCAGTCCGCCCAAGGGTTCGGGACTGCACCGCTACGCGTTCCTGGTCTACTCGCAGGGGACCAACAGGATAGCCGAGAAGGACGCGCCGGTGCCGGACGCCCGCGGACAGTTCAACGTGGCCCGCTTCGTGAACGACATGAAGCT

>AAFM16908

TCAACTGCATGCCACCGTTCGCGCTGGTCATGGTTGACCCTGACGTGCCGAGTCGCGAGCAGCCCACGGAACGCAGCAGGGTACATTGGATGGTGCTCAACGCCAACAGCACGGAGAGGCTGCACGAAGGCGAAGAGGCGGTGCCTTACAGCGGTCCGAGCCCGCCAGAGGGAACGGGACCCCATCGCTACGTGTTTTTGGCGTACTGCCAAGGTGGCAAGTGGGTGTCGAGCGACGACATTGCGCCGAAGCGAAGGAACAAATTCGAGTTGGGAGATTTTGCAGCGAATTTGAACGCTGGTAACGCATTTGGAGGCACGTTCTTTTACGCAGAGAACGCCTGAAAGGTAACCTTCGAACCAGTACGGTGGGACTTTATGAATATGGGATGAAAGCTCCGAAAGTTTACTTAGTTGAAGACACCTGTAGGCCCATTGGTGAACATTTGTGAACGCAGGAAAATGCAGCCAGTAATGTGAATGACGATAAATAAATTTGATGAAAACACAAAAAAA

>AAFM23294

CACAGCCTTTATTACTAACGCGATGCGCATCACATAGGAAGTAGATATTTGTCATTAACCGTTTGAGCCTGGTGCCAGAAAGCATGCTCTGAAGAAACGTTATAATGTAACGGCTAATGTATTGTGCTCACGTTAAGGTCTTTGTTCTTATTTTTTTTTCAATGATGAGGAAGTAGAAGGCGAGGTATACTGTCAGCATGAGACACGCATTGTCGATGCAATCATCGCCATGACGACGAGCAGAAGGGCACGCTGCCGGTTGGCGACGCACTGGCATGGCGCCGCAGACAGCCCGGCTCGTGGTCTCGCCGAACGCGCCTCGACGAGAAAGTAGTTGATGCCCACCGGATCGCCGAGGTCGTTTGTGGCCGCGAAGCCCCGCACGTCGAACTTCGACCGTACGCCGTCCACCGCCATGTTTCGCGCACTCAGGCGCCGCATTCCCTGCGAATAGACGAGGAAAACTAAGCGGTGGGGTCCCGTGTCGTGCGCCGGTCTCGGCCCGATGTACTCCGTGACGACCGTGCCGTCCAGCAGGTTCATGGTCGACGGAACGTTGACGACGAGCCACTGGCGGTAGCTGCGCTGGCTCGGGTGCGAGCGGCTGGGCGCGTCCAGGTCCACGAGCGTCAGCGTGTAGTAGTTGTCGGGCGGCGCGTTGAAGCTCACATCCTCGGGCTGCCGCGTCGTGTTGGAGACGGACAGCGTGTTGCCCAGCGCGACGCTGACGCCCCGCTTGTAGTAGACGTAGAGCACTTCGACGGGCGCGAAGTGGGTGAGGTCTTCGACGACGCCGTCGTACACGAGATTCGCCAGCCGCTCCTTGATGTCCGACTCCTGGCCTTCCACCAGACACCGGCGATGGGGCAGGACCAGGGACAAGGACAGCGTGAGGAAGACTCTCGCTGCTGGCCGCCACAGAGACCCAGCCATCGGGGACATTG

>AAFM33422

CTAACTTAAAAATCCTGTTTTCAAAAATTGTGTTGCGTTAGGTGTAACAGCCCGTATATTTCGCGCCAAAAATCAATGGGAACATCTGGAAGCAAAAATCCGTCGTTAGGTTTACTTGCGGTCAGCGCTCATCGATGTACCGGTAGAAACTGTGGTTTAGTGATGCGGAAAAAAAATCTAGTTGAGCAGCCCAGCTCGTGCTCAATCACTGGCTAATGGTCGCTGCACTGTCACCAGCAGGCAGATGTTTAAAGTATGCTTCTGTATTCCGTGTCGATCCGGAAGAAGTTGAAGGCAGCGATCTTGCTAATTTCGTTGTCCGCCATGAAGGCGCTCACGTTGAAGGCCGCGCGCAATGGTATGAAGGTGAAGTTGCCCTGCTTGAACTCGTAGCGTTGCTCGAACTGGATGAACGCAAACCGGTGCGAACCAGTGCTCGTCGCCGGCGTCGGAGGGCTGTACGCCGCCAGCACTTTCCCCGCCTGCACCTTGCTTGCCGGTATGTTGACGACGAGCCAGTGGAGCCAGGAACGGTACCTGGGCTTGGCTGCCGAGGGCGCATCCACATCGAGCANNCACAGCGAGGAGAACTTCCCGCTCCTGACGTCGAGCTGGACGCGTGGGGCGAATGCCACATCGCTCGGCTCCAGTTCGTTGCCCATGTCCACCTT

>AAFM34116

GATGTGGTCCTGATCTCCTGGGTGGGGTCGGACCGTTGTAGCGCTTCACTGTTTTGCCTTCATGCAGCCTCCTCGTGCTTTTCGCATTCACCACGATCCAGTGCAACCAGCTGCGTGCCGTGGGTCTCCTGCGACTCGGCGCATCCGGGTCGATCATCACCAGCGCGAACGGAGGCATGCAGTTGACGGCTCCTCTCAGTCTGACCATCGTCGGAGGCTTGGCAGTCTGTGCGGGCGTCA

>AAUF1717

CGCAGCTCGGGCATTACTTGAGAGATTGCACACTGACGAAGCACTCATAACTCGGCAGACTTCACGATGAGCATATCACAGTGGCTGATGCGCCCCAGCAGCTATAGATGTCTTCAACTTCTCGAGCAACGGGACGGAGCTTCTAGACTGGATTCCGAGGCGACGGTGGTCTACCACTTCGTCATCGGCGGTCCGGTCTTCCATCGGTTCGTTGCCGTACTTTGTAGAAAGAGGCTGTAAAGTTCAAAGACTAAACTACACAGCTGCGNNNNNNNNNNNNNNNNNNNNNNNNNNNNCGGCCAGGTTGAAGTTCTTCCTCTCCTTCGGCGCCAGATCTTCGCCTTGCAGGCGCATTCCGCGCTGACAGTACACCAGGAACACGTATCGGTGCAGTCCAGATCCCTGGGGTGGAGCGGGCCCTCTGTAGGGCAACGCGGCGTCCCCTTCGTGGAGTTTCTCCGTGCTGTTGATGTTCAACACCATCCAGTGCATCACACTGCGGTACACCGGGTTCTTTCGACTGGTCGCGTCCGGGTCCA

>AAUF1718

CGACGGTGGTCTACCACTCCGTCATCGGCGGTCCGGTCTTCCATCGATTCGCTGCCGTACTTTGTAGAAAGAGGCTGTGAAGCGTCAAGACTAAACTACACAGCTGCGGGGTTCTCAGCGACGAAGAAGTTCCCAGCCAACGGCGTGCCCAGGCTCGTCCTGTTGACGAATTCGGCCAGGTTGAAGTTCTTCCTTTCCTTCGGCGCCAGATCTTCGCCTTGCAGGCGCATTCCGCGCTGACAGTACACCAGGAACACGTATCGGTGCAGTCCAGATCCCTGGGGAGGAGCGGGCCCTCTGTAGGGCAACGCGGCGTCCCCTTCGTGGAGCTTCTCCGTGCTGTTGA

>AAUF1853

CTAGGAAGGAGACCCATAGTGCAAGCATAGCAAGACGAAAGTACCAGACACCTCCAGACAAATCTGGTGATTTGGGCATGTTCACAGCAGAATTGTGCACAATGCAGTGTAGACAATAAATGTTGGGCATGCATTCATGTGTCCTGCTCTATCACAGCAGAATTCTGCACTATGCAGTTGCTAATAGATGTGCACAAAAATATTCTGCACTTAAGCGAAAAATTTAGACATGTTTCCACCACTGTAAACATGCACCATACTCAGGGTATTAATAAGGGTAGTGTATGTAGAAAAGAGAAAGAGGCTTGGGAATAATCAAGGAAGGAGTTTAAGACCAACATAAATTCTGCTTTATTCAGAGCATGCCAGAGCACAGTAAAGTGCTTGAGCTCAAAGAGGGGGCTATGCAACAGAAAGCAAAGAACAAGGAAAGAACAGCGCAGTAAGGACAGACAGGAAAGGGCAAACCCCATAGGCCAATCAGTGCACTTTGATCTCAGTTGCCGCTCAGCTGCTCATAGAGCTTGGGCACATAGTCATCCCATTCTGCCTGGTAGAAGTTTGCAGCAACTGGCTCTCCCAGCTGATACTTCTTGGCAAAGTCTCGGATCTTGAAGCCACCTCGGTGATCTCCAGAGCGGTTTGTGAGGCGCTTTTCATCACAGGAGAGCTTCCCAGGCTGCTTGTAAACCACAAAGACATACCGATGGAGACCTGTTCCTTTTGGAGGTCCTGAACCAACATACTGAGACAGAGTCTCTCCATCTGAGACTTTGCAGCCAGGAATGTTTACCACAAGCCAGTGATGCCACTCCCGATACTTGGGTGTTTGCCGGCTCGGAGCATCGGGGTCAGTCATGCATAGCGTGTAAAGGGCGCCAGCGTCCGTCGGGTAAGAAACAGTTGGTGGACACTGGACTTGCGTCGGCGTCAGCGTATTTCCCATGTTAACCGAGACACCGTTGTAGGTCACCTCAACGGTATCTTTCGGGACCGTGTCGATCACGTCCGGAACAACTTGGTTGTCCTCCATCTTTGTCATCCCGGTGCCCTTCTGAAGGTACACACTGCTCTGTTGAAGCAAACGGCGAAACGACGATGAAAACCCGGATGCAGATGCTGCTCTCTGGGAAGAGGTCAAAGAAGCGCTGAGNNNNNNNNNNNNNNNNNNNNNNNNNNNNNNNNNNNNNNNNNNNNNNNNNNNNNNNNNNNNNNNNNNNNNNNNNNNNNNNNNNNNNNNNNNNNNNNNNNNTTTTAAGTGCAAGAATCGCTGGTATGCTTTTTTTTACGCAAAACTACAAAGAGCTTGAGCGTTTGTCACGCGGATAGATTGTTCGCAACAAGGTCAGCGTTTTAACGCCATCTGTGGCGTAGAGTCGGAGCTTACTGAAAACAAACATGGCAGCTATTCGGCAGTAGTAA

>AAUF4747

TCAACAGCACGGAGAAGCTCCAGGAAGGGGACGCCGCGTTGCCTTACAACGGGCCCGCTCCACCCCAAGGATCTGGACTGCACCGATACGTGTTCCTGGTGTACTGTCAGCGCGGAATGCGCTTGCAAGCCAAAGATCTGGCGCCAAAGGAAAGGAAGAACTTCAACCTGGCCGAATTCGTCAACAGGACGAGCCTGGGCACGCCGTTGGCTGGAAACTTCTTCGTCGCTGAGAACCC

>AAUF6698

TTTTTTTCCCTTGTCAAGTTTTTTATTTCTCTGTACTCTTTTAAATAGCTATGCATGAATGAAAATGAAAACTGCACATATACAGACACACTCATGATGGCGATGATTTGGCAGCCTTTTGCTCAGCCCACTTGCGCTCAAACTCTTTCTGCTCCCTCCACGCTTTTACTTTGGGGTCCTCATACTCCTCCTCTGGGTAAATGCTGAAAGGCCTCAGGTCCTTGTATTTTCCAATGCGAAAGCGTTCCTTCCTCATCTCATCCACCAGCCAGCTTGGTGTGCCTTGTGGAATCTTGTATATATTGGGGTACTTTGGCATGGGTGGCTCATCTTCCAAAGGATTGAGAGATTTCAGGCGCTTAAGGTAAACTTCTTCACGGATGTCCCTTGGGTCCCGGTACATGTCAAGGTACGTGTTAAAAGGCTGCTTGTGAGGGTACAGAACTTGCTTTGGGACGTATTCTGGAGGGTGGACATACTCAAAAGTGGGGACTCGCATTTTCAAAGTTTTGTGAAAGAAGTCCACCAGGCTGTCCTCCCAGGTGCACTGAAAGAATGCTAGGCCAGCAGGTGTGAGCACATTTTCGAATTCCTTGTAGAAGTCATAGGTTTTAAATGTCCTCTCCTTCAGGCTGGTACTGTTTGCAGACAATTTCTGGTTAGAGTAGTCAATTAATCCTTCTTGTTTGTACAAAACAAACACAAACCGATGATAGCCAGTACCACGAGGAAGAAAAGGCTGCATGTAGTCGCACACCACTTCACCACTTGGAACCTGGTTTCCTTTTATGTTGCCAATGAACCAATGAAGATACTCTTTGTCATTTTCAAGGAGATGAGAATCCAAGCTTGTCAAGACAAGTGTCCAAAGCATGTTAGGCTCGGAATCAAATAAAACTGTTGGTGCTTTTGCAGCCTCAGAAGGAAGAATTATGTTACCATAATGAACAGGTGTGAAATACTCTGCATCATACTCGTACAAAACATCCATTGGAATAACAGGGTGAAAGAATCCAAACTCATAAAGGTCTCTGAAGACACCGTAGTGGTCTGCCACTTCTTTCACGTGCATTGGTGCATAGGTTTTTCTCCATTCTTGCCTGGCTTTTTCACATTGTATCTTCAAGGTTCCATTACGTGACTTCTCAGCAAGGTCATAATTGTCCTTGTTCTCTTGGATTATCTTTCGTTGTCTTTTCCATTCTTGTTTATTGACACTGTGAGGTGCGACCTTGAAACCAATATTGACTCGTTCTTCAAACACTGGCTCTTTTGCTTTTAGAGCTTGCATCTTCTCTTCCAAACTTTTCAAGGTATCCATTGGCGGAAGCCGAACACAACGAGCCATTGCAGGCTCAAGGCGCCTCAAAGCCCGAAAGTTTTTCAAAAATGCGGCCAGTGTAGCCATTGCTGAAATTTTAAAGAGTTGCGTCCAGGTATTTTACGAACTCTGCTGCAAAGTAACACATTCATAAGACACAACCCTCGATCAGGCAGAAGCAGCAGTAGCAAGCGCTAAACCACTGGCAAAACCATGCCATCCATGCCGTGCGCAGCACCCTGGCAGCACTGGATCAAGTTGG

>AAUF9610

TCTCACGGCTCGGTGCGTCCGGGTCCAACATGACGAGTGCGAAGGGCGGAATGCAGTTGATGGCGCCCCTCAGTCTGACCAGCGTCGGGGCCTCCGCAGTCTGCGCGGGCGTCAGAGTGCCGTTCTTGACCACAGAGAGATGATCCCCGTACCTCACTTCCAGATCGGCACGCAGCGTGTCTGGAAGCGAGAGGTCGCTCACTATGCCTGCGGATGTCCATACGTCCGGCTCAGACTGCGACGATGCCGCAGGTTCCTCCGCGTGGCAGCTGCTCCATAGGGCCAAGAGAAGCAGGGACGAAGGCAAAACAAGCCTCATATTTGTGCCTTCGTGTGGCGAAGCACACCTGAACGACTGGGCAGAGTTGTCTCTCACACGGGTGCCGTCAAC

>AAUF13199

TTGAAAAGCACTCAAGGACTTCGGCATCCGGCGGAGCAGCAGGAGTGTCGGCGGCTTCCGCGTCAGATGACTCTGCTTGGTCACTCTAGTAGTGAGCGCCGTTTGGCGGAAGACGAGAAGATGAGGCTCCTTCCGACGTCAGCCTTGCTTATCTTGGCCCTATGGAGTGGCAGCCAGGTGAAAGAGGCTACCGCACCGTGGGCTGCGACGAAATCGTCGTCCCAGTGGAGGCAGCAGGCAGAGTCTGATCTGGATTTATGGACGTCCTCGGGACTTGTGAGGGACCTCTCGCTACCTGACACGCTGCATGCCGAGCTCGAAGTGAGGTACGGGAGGGTGTCGGTGGTCATGAACGGAACACTGACGCCCGCACAGACTGCCAAGCCCCCGACGATGGTCAGACTGAGAGGAGCCGTCAACTGCATGCCTCCGTTCGCGCTGGTGATGATCGACCCGGATGCACCGAGTCGCAGGAGACCCACGGCACGCAGCTGGTTGCACTGGATGGTGGTGAACGCGAAAAGCACGAGGAGGCTGCATGAAGGCAAAACAGTGAAGCGGTACAACGGTCCGACCCCGCCCAGGAGATCAGGACCACATCGGTACGTGTTTCTGGCCTTCTGTCAGGGCCGCAAGCGCGTGCGGGGCAGGAAGATTGCGCCGAGGCGGAGGAACAACTTCAACCTGGCTAAATTCATCAGAAAACTGAAGGCGGGAAACCCGTTCGGAGGCAACTTCTTTTACGCCGAGAACGCATGATGTGTGTTAGTGGGACTACGTGATGAGTGGTTCGATGAAGGTTGACATTTACTGACTTGCTCAGAAATTGCGGCTGCATGTGTGCTGTCACTGAATAAGAATGAAATTAATGTAAAAATTCCTTTTCGTCCTCTGCAGCATCGTAATATTGATATTCCTTCCTGGGTATTACGCAGTGAGTGGTGATGGCTGTG

>AAUM837

TCAACAGCACGGAGAAGCTCCAGGAAGGGGACGCCGCGTTGCCTTACAACGGGCCCGCTCCACCCCAAGGATCTGGACTGCACCGATACGTGTTCCTGGTGTACTGTCAGCGCGGAATGCGCTTGCAAGCCAAAGATCTGGCGCCAAAGGAAAGGAAGAACTTCAACCTGGCCGAATTCGTCAACAGGACGAGCCTGGGCACGCCGTTGGCTGGAAACTTCTTCGTCGCTGAGAACCCCGCAGCTGTGTAGTTCAGTCTTGACGCTTCACAG

>AAUM2536

CCGGACAGTCTGTGTGGATTAAGGAGGAGCTGGTCGCCGACCTCTCGCTGCCCGGAGTGCCGAACGCAACGTTGGAAGTGACCTACGCGGGCGATTTGAAAGTGGCGCTGGGCAACTACACATTCACGCCTCAGCAAGCCAGCACTGCGCCTACGGTGAGCCTGAATGNNNNNNNNNNNNNNNNNNNNNNNNNNNNNNNTCCGGTTGACTGCGAAGGTCCGTACGCGCTGCTCATGGTGGACCCGGACGCGACCAGTCGAAAGAACCCGGTGTACCGCAGTGTGATGCACTGGATGGTGTTGAACATCAACAGCACGGAGAAACTCCACGAAGGGGACGCCGCGTTGCCCTACAGAGGGCCCGCTCCACCCCAGGGATCTGGACTGCACCGATACGTGTTCCTGGTGTACTGTCAGCGCGGAATGCGCCTGCAAGGCGAAGATCTGGCGCCGAAGGAGAGGAAGAACTTCAACCTGGCCG

>AAUM2537

CATCGATGGCTATGCCTGCCGCGCCGGCAATGGCTGGGCCTGCCGCGCCGGCAGCGGCGGGAAACCAGACAGCCGAAAACCAGACGACGGCAAACCAGACGGTGGTGGATTCTGGACAGTCTGTGTGGATTAAGGAGGAGCTGGTCGCCGACCTCTCGCTGCCCGGAGCGCCGAATGCCACCCTCGAAGTGACTTACGCGGGCGATTTGAAAGTGACGCTGGGCAACACGCTGACGCCTCAGCAAGCCAGCACTGCGCCTACGGTGAGCCTGAATGCTCCGGTTGACTGCGAAGGTCCGTACGCGCTGCTCATGGTGGATCCGGACGCGACAAGTCGCAAGAACCCGGTGTTCCGCAGCTGGATGCACTGGATGGTGTTGAACATCAACAGCACGGAGAA

>AAUM4413

TGCCTTGTCAAGTTTTTTATTTCTCTGTACTCTTTTAAATAGCTATGCATGAATGAAAATGAAAACTGCACATATACAGACACACTCATGATGGCGATGATTTGGCAGCCTTTTGCTCAGCCCACTTGCGCTCAAACTCTTTCTGCTCCCTCCACGCTTTTACTTTGGGGTCCTCATACTCCTCCTCTGGGTAAATGCTGAAAGGCCTCAGGTCCTTGTATTTTCCAATGCGAAAGCGTTCCTTCCTCATCTCATCCACCAGCCAGCTTGGTGTGCCTTGTGGAATCTTGTATATATTGGGGTACTTTGGCATGGGTGGCTCATCTTCCAAAGGATTGAGAGATTTCAGGCGCTTAAGGTAAACTTCTTCACGGATGTCCCTTGGGTCCCGGTACATGTCAAGGTACGTGTTAAAAGGCTGCTTGTGAGGGTACAGAACTTGCTTTGGGACGTATTCTGGAGGGTGGACATACTCAAAAGTGGGGACTCGCATTTTCAAAGTTTTGTGAAAGAAGTCCACCAGGCTGTCCTCCCAGGTGCACTGAAAGAATGCTAGGCCAGCAGGTGTGAGCACATTTTCGAATTCCTTGTAGAAGTCATAGGTTTTAAATGTCCTCTCCTTCAGGCTGGTACTGTTTGCAGACAATTTCTGGTTAGAGTAGTCAATTAATCCTTCTTGTTTGTACAAAACAAACACAAACCGATGATAGCCAGTACCACGAGGAAGAAAAGGCTGCATGTAGTCGCACACCACTTCACCACTTGGAACCTGGTTTCCTTTTATGTTGCCAATGAACCAATGAAGATACTCTTTGTCATTTTCAAGGAGATGAGAATCCAAGCTTGTCAAGACAAGTGTCCAAAGCATGTTAGGCTCGGAATCAAATAAAACTGTTGGTGCTTTTGCAGCCTCAGAAGGAAGAATTATGTTACCATAATGAACAGGTGTGAAATACTCTGCATCATACTCGTACAAAACATCCATTGGAATAACAGGGTGAAAGAATCCAAACTCATAAAGGTCTCTGAAGACACCGTAGTGGTCTGCCACTTCTTTCACGTGCATTGGTGCATAGGTTTTTCTCCATTCTTGCCTGGCTTTTTCACATTGTATCTTCAAGGTTCCATTACGTGACTTCTCAGCAAGGTCATAATTGTCCTTGTTCTCTTGGATTATCTTTCGTTGTCTTTTCCATTCTTGTTTATTGACACTGTGAGGTGCGACCTTGAAACCAATATTGACTCGTTCTTCAAACACTGGCTCTTTTGCTTTTAGAGCTTGCATCTTCTCTTCCAAACTTTTCAAGGTATCCATTGGCGGAAGCCGAACACAACGAGCCATTGCAGGCTCAAGGCGCCTCAAAGCCCGAAAGTTTTTCAAAAATGCGGCCAGTGTAGCCATTGCTGAAATTTTAAAGAGTTGCGTCCAGGTATTTTACGAACTCTGCTGCAAAGTAACACATTCATAAGACACAACCCTCGATCAGGCAGAAGCAGCAGTAGCAAGCGCTAAACCACTGGCAAAACCATGCCATCCATGCCGTGCGCAGCACCCTCCCCCGGCAGAGTAAGCTGCAAGTATCTG

>AAUM4909

ATTTGAGATGGCCGTTTTCAATAAATGACAACAAGCAGACAAGCAGCAAAACAAGACTTAGTATTACAACTACACCAAAGGCCAATTTACGAAGCCCAAAAGAAAATGACAGCAATGAACAGCAAATAAATCAGCTGTGCGTTAACTACCTAGTGTGCCCAATGGTCACAAAATGAAGTCAAAGAAACACTGCTGCAACTCGAAGCAATGAACACAACATAAGCAGCTGTAATTTGCCCGCTCCCTTACCCAGTAGTTATGATTTCAAGACCAGAAGACAGCACCACTGTAGCTCACAGCAATCAAACACTGACACACCCAGCTGCAGATCAAGTTGCTACAAAACTGCAATCAAGTATACAATGTATTCTATGCATTTAAAAAAATCACTAGCCCTTTTTAATAACTGGCCACATTTCAACATACATTGCAAATTTTTCTGTTACATTTTCAAACTAATTTACATGCTACTTGTCCATTTCTATGCACCTGCGGCCATGAACAGTTCCATGCAGGTTGTCCCCAGAATGTTTCAGATCTTTCAGCTCTGCAATGCTGAACACACTACTTCATATGCTTATAAAATTTGCACCATCTGAAATCACATCTCACCATGGTCCAAAGGGAGTGTGCTAGTACATCCTGAAGTCTCCTGAATTCTGGAACCCTCAAACAAGCCCTGCGTCTAGGCAAGCTACATAGCTTTGTCATCACAGAGGAACGTCTTGGACAAAGTGTTTCATCTGCAGAGTACTAAGGATCCTGATCAACCGCAACAGATTAAACTTCCCTTCAATGTATGCACAAAATTTTTTGTTTCTCAAAAGGCCCTGTTTGCAGTTTTGTCCGCTACACTCAATTCACTAAATGTGCACACCACAAAGACAATGCACCTACGGCCAAATTATAGCACTTTTCAACACAAGAGACCATGGACACACATCGCTGCTAACGCTCCAGATAACCCCTTTCGTAACTGCTGGCCACTGCTCCTCCAGTCTACACCGATCTTTGGATGAACAGTGGTGGCTTTGAGCAAGCTCCATGCTGGGTGGGCGAGCAGCAGTGCCAGCAGCTTCTGCTCAAATGCCCGAAGCGGACTCCAACACTGGACACGCGTGCTACTCCTGGTCAGAGTCGCCATCACTAGAGTAGCCACTCTGAGAGTCTGTGAGCCGATGATGTGGCGAAAGTTTACGATAGGCCTCCAGCCACTGGTCCACCATTTGTAGCACCAGGTGAGGACTGCGGGGCACCTTCTGCTGGGCTACAAGTAGGCCACCCTCTGTCACACAGCAGTCGAGCCATTGCTTGATTATCTCCTCTTCACGCCCAACCTCGCCTTGGGTGAGGAAGTGTAGGGGTAACAGCTGACGGTCAGCTGTCATCAGAGGAAGGAAGGACACCTGCAGGTCATCTCGTGGATTGGCCTCAGCGCCTCCTATCACTTCAGGTTCGGGCTCCATTGGCACCAGGGCTGAGAAGTGGCCACGTGTGTAGCCCAGAGCTATCGGGGACTTCCAGCAGAAACTGGGCTCCCATAGCAGAGGTATGTAGATGCCTTCAAAACGAGCATAGCCGAGGGCTTCCCCACGAAAGCTCTTGACGTACTTGACACCATACACGACAATGGGTCGTCGCAGAACGTGGGCCAGTGTGAACACATGCATCTGCTCGAGTGCTGAGCCAGGCTGTGCAGCCATGGAGACCAGTGTGGCCCAGTCCTCCTGCCACTGTGCCTCGTCCAGAGAAAAATGCAGCAGCCGTGCCTGCAATGCCTCCGCTTCTCGCCACCGAGGGTACAACACAGAAGCAGCTTCGCTCAGGCTGTCACCAAGAACTTGCCGTAGCGAGTTGTCACGGTCAAACACGCCCCAAGTTGCTTGCAGCGCAGAGTCTAGAAGACAGTTGCCAGCACTGCGATTCCACAGGGCATACAGCCTTGAACCTAAGGTCTCAGTCAGCTCCAGACTCCAGTTGATAATAGCAGAATCCTCTTCAAGCTCTTTCTGCACATCCTGATCCAGAAGCTCATCGAACAAAACCTTCTGCACAGATGAATGAAACTCCTTGATTTCTGTTGGTAGTGCGAAGGTGACAAATTCAGTGACAAAGTAGCATGAAAAAGGTCCCTTCCTCTGCTTGAAAGAAGCTGCAATGTGGCGGCGGATGTCAGCAGCAGCATCCCGGCCAACGTCGGATGGCACACGCTTGGCAACAGGGTGAGTCATGCCCGAATTGGACAGCAGCAAGGCCAGAATATCATCACGCTCTTGGCGAATAGCCATGTGTGCCAGGGTGAAGCCTACATCAAAGTTGGCAGTGCGTGCTAAGAGCCCTGCCTCAGCATTTGTCAGCTGCCTTGCAGGGTCTCCGCCAGAGTTCAGGTAACGACTGATGGGTTCAGGCTCTCCATCCACAACAGCTCGGCATGCGTTGAGCCACAGACGATCCATTTCACGTGCTTGACGCCGTAAACTGCACAGGTAGTTGTTGCTTGGTGGGTTTTCCTCTGCCGACATCTGAACCTCAATCTCAGTTGTTCCCGTGAGGCGATTGCTGCCACCAGTGCACACAGCAGGTACTCCAGTGGGGGTGTTTCGCAGGTCATCAGGATCAGGGGTCTGGTTTCCTACTGGTGAATCACCTTTAGAACGGGTAGCGTTGCACATGATGCACTTGCGGGACTTGGGCCAGTTCTCGTAAGTGCAGGCACCACACGTCCACTTGGACCGCAGGTCTGCACTGGTGTGCTCCACTTCGGTAGATGTGGTAGCCTGCTGTGGCTGACTGGAAGGTGGGCTGGCTTGAGCATTGCACGGTGATCCCCGTGGATGCAAACACTGGGTGCATTTGCTTGACCGCGGCCAGTTCATGTATGTGCACCGGCCACATGCCCATTTCTGGTATGAGCTGGTCAGATCAGGGGACACCAGAGGTGAGATCTTGTAAATGTCGCGTTCCTCACTAGACGCTTCCTCTTCAATCAGAGTGGGTCTAGGGTTTTGGCAGATGACACACCGCAATGCCTTTGGCCAGTTCTCATACGTGCAGTGCTGGCAAGTCCACTTGCAACCCTGCCGACTCATGACTCCACCGGGCCCAGCACGTCTCGCGTCCAGCTCTTCAACAGCATCTGCCGGCCGCCTCTGCTAGTGGGACGCCAGCCTTTTGACGGCAAGGCAGAGAGGCATTAGAAGCCAACGTAATGATCTGTTCAACTAAGACAGAACATTCTTTTTTTCTCTTACTACTGCCGAATAGCTGCCATGTTTGTTTTCAGTAAGCTCCGACTCTACGCCACAGATGGCGTTAAAACGCTGACCTTGTTGCGAACAATCTATCCGCGTGACAAACGCTCAAGCTCTTTGTAGTTTTGCGTAAAAAAAAGCATACCAGCGATTCTTGCACTTAAAAATTAATGATGTTTCGAGCTGTCTGTTCGCGAGCAATCTTTGCGTTCGGCCCCCTCAGCGCTTCTTTGACCTCTTCCCAGAGAGCAGCATCTGCATCCGGGTTTTCATCGTCGTTTCGCCGTTTGCTTCAACAGAGCAGTGTGTACCTTCAGAAGGGCACCGGGATGACAAAGATGGAGGACAACCAAGTTGTTCCGGACGTGATCGACACGGTCCCGAAAGATACCGTTGAGGTGACCTACAACGGTGTCTCGGTTAACATGGGAAATACGCTGACGCCGACGCAAGTCCAGTGTCCACCAACTGTTTCTTACCCGACGGACGCTGGCGCCCTTTACACGCTATGCATGACTGACCCCGATGCTCCGAGCCGGCAAACACCCAAGTATCGGGAGTGGCATCACTGGCTTGTGGTAAACATTCCTGGCTGCAAAGTCTCAGATGGAGAGACTCTGTCTCAGTATGTTGGTTCAGGACCTCCAAAAGGAACAGGTCTCCATCGGTATGTCTTTGTGGTTTACAAGCAGCCTGGGAAGCTCTCCTGTGATGAAAAGCGCCTCACAAACCGCTCTGGAGATCACCGAGGTGGCTTCAAGATCCGAGACTTTGCCAAGAAGTATCAGCTGGGAGAGCCAGTTGCTGCAAACTTCTACCAGGCAGAATGGGATGACTATGTGCCCAAGCTCTATGAGCAGCTGAGCGGCAACTGAGATCAAAGTGCACTGATTGGCCTATGGGGTTTGCCCTTTCCTGTCTGTCCTTACTGCGCTGTTCTTTCCTTGTTCTTTGCTTTCTGTTGCATAGCCCCCTCTTTGAGCTCAAGCACTTTACTGTGCTCTGGCATGCTCTGAATAAAGCAGAATTTATGTTGGTCTTAAACTCCTTCCTTGATTATTCCCAAGCCTCTTTCTCTTTTCTACATACACTACCCTTATTAATACCCTGAGTATGGTGCATGTTTACAGTGGTGGAAACATGTCTAAATTTTTCGCTTAAGTGCAGAATATTTTTGTGCACATCTATTAGCAACTGCATAGTGCAGAATTCTGCTGTGATAGAGCAGGACACATGAATGCATGCCCAACATTTATTGTCTACACTGCATTGTGCACAATTCTGCTGTGAACATGCCCAAATCACCAGATTTGTCTGGAGGTGTCTGGTACTTTCGTCTTGCTATGCTTGCACTATGGGTCTCCTTCCTAGTAGAAAATGCTGAAATCGGCATGATAGCACAGCTGTAAGTGCATGGCCTATTGCAGTCATATGTGTGGGCTGGTACACTTTGACAGTGTTGCAGAGGTACAGGCTTGTGTTGATGGTCACATTGTGCAAAGCTGACATGACATTGATGAATGCATGCTTGAACCAATTGCGACAAATCCG

>AAUM6216

CGAAAGCGAAAAGCTGCATCTCTCAGGCTAAGCAGCGACCACTTTTGTGTCGCTGATAACGCTACCTCTGCTCGAAGCGATTGCCGCGGCTGCTGCGAGGGGCGTATCGACCAATGAGATAAACATACAAGGACGGCGAAAGCCGAGTCAGCGCAGCAGTCTGTTGACGGCACCCGTGTGAGAGACAACTCTGCCCAGTCGCCCAAGTGTGCGTCGCCCCACGAAGGCACAAATATGAGGCTTGTTTTGCCTTCGTCCCTGCTTCTCTTGGCCCTATGGAGCAGCTGCCACGCGGAGGAACCTGCGGCATCGTCGCAGTCTGAGCCGGACGTATGGACATCCGCAGGCATAGTGAGCGACCTCTCGCTTCCAGACACGCTGCGTGCCGATCTGGAAGTGAGGTACGGGGATCATCTCTCTGTGGTCAAGAACGGCACTCTGACGCCCGCGCAGACTGCCGAGGCCCCGACGTTGGTCAGACTGAGGGGCGCCATCAACTGCATTCCGCCCTTCGCACTCGTCATGTTGGACCCGGACGCACCGAGCCGTGAGA

>AAUM26901

GCCAGAAACACGTACCGATGTGGTCCTGATCTCCTGGGCGGGGTCGGACCGTTGTACCGCTTCACTGTTTTGCCTTCATGCAGCCTCCTCGTGCTTTTCGCGTTCACCACCATCCAGTGCAACCAGCTGCGTGCCGTGGGTCTCCTGCGACTCGGTGCATCCGGGTCGATCATCACCAGCGCGAACGGAGGCATGCAGTTGACGGCTCCTCTCAGTCTGACCATCGTCGGGGGCTTGGCAGTCTGTGCGGGCGTCAGTGTTCCGTTCATGACCACCGACACCCTCCCGTACCTCACTTCGAGCTCGGCATGCAGCGTGTCAGGTAGCGAGAGGTCCCTCACAAGTCCCGAGGACGTCCATAAATCCAGATCAGACTCTGCCTGCTGCCTCCACTGGGACGACGATTTCGTCGCAGCCCACGGTGCGGTAGCCTCTTTCACCTGGCTGCCACTCCATAGGGCCAAGATAAGCAAGGCTGACGTCGGAAGGAGCCTCATCTTCTCGTCTTCCGCCAAACGGCGCTCACTACTAGAGTGACCAAGCAGAGTCATCTGACGCGGAAGACGCGGACACTCCTGCTGCGCCGCCGACTGCCG

>AAUM28094

CTCGAAGGCAACGTTTTCAGGCGTTTTCAGCGTAAAAGAAGGTGCCTGCAGCGGGCATGCTCGAGGGCACCTTCTGAGCGACACTTGTCACCTGGAAGTTGTTCCTTTGCTGGGGAGCTACGTCTGCGCTATTCAGTCGCTTGCCGTCCTGACAGTAGACCAGGAGGGCGTAGCGGTGAGGTTGAGAACCTTTGGGCGGGGTGGGTCCGTTGTACGGCATCGCGACCTCGCCATCTTGGAAGCGGGACTTGTGCTCGGCGTTGATCACCATCCAGTGGAGCCAGCTTCGGGCCGTGGGATTTCCCTTGTTGGGCGCGTCCGGGTCGACCATGACGAGTGTATACGGCGGGCCGCAGTGTAGGAAGCCGT

>SG1202291

TTTCTCCAGCACAAACTGTGTTTATTAGGCAATGCCAAAGGGCATTCCTTAGCCCAAGGCGAGCACAAAAAAAAAATAAAATGCTGCGGTACACAAAAGGAAGTTGCCATTTGACTGACCCTCTGAATGCATGCCCCTGAGCAAACTTGCATAGTGCACACTTATTTTTGACAATAACTGCACGGATTTGTCGCAATTGGTTCAAGCATGCATTCATCAATGTCATGTCAGCTTTGCACAATGTGACCATCAACACAAGCCTGTACCTCTGCAACACTGTCAAAGTGTACCAGCCCACACATATGACTGCAATAGGCCATGCACTTACAGCTGTGCTATCATGCCGATTTCAGCATTTTCTACTAGGAAGGAGACCCATAGTGCAAGCATAGCAAGACGAAAGTACCAGACACCTCCAGACAAATCTGGTGATTTGGGCATGTTCACAGCAGAATTGTGCACAATGCAGTGTAGACAATAAATGTTGGGCATGCATTCATGTGTCCTGCTCTATCACAGCAGAATTCTGCACTATGCAGTTGCTAATAGATGTGCACAAAAATATTCTGCACTTAAGCGAAAAATTTAGACATGTTTCCACCACTGTAAACATGCACCATACTCAGGGTATTAATAAGGGTAGTGTATGTAGAAAAGAGAAAGAGGCTTGGGAATAATCAAGGAAGGAGTTTAAGACCAACATAAATTCTGCTTTATTCAGAGCATGCCAGAGCACAGTAAAGTGCTTGAGCTCAAAGAGGGGGCTATGCAACAGAAAGCAAAGAACAAGGAAAGAACAGCGCAGTAAGGACAGACAGGAAAGGGCAAACCCCATAGGCCAATCAGTGCACTTTGATCTCAGTTGCCGCTCAGCTGCTCATAGAGCTTGGGCACATAGTCATCCCATTCTGCCTGGTAGAAGTTTGCAGCAACTGGCTCTCCCAGCTGATACTTCTTGGCAAAGTCTCGGATCTTGAAGCCACCTCGGTGATCTCCAGAGCGGTTTGTGAGGCGCTTTTCATCACAGGAGAGCTTCCCAGGCTGCTTGTAAACCACAAAGACATACCGATGGAGACCTGTTCCTTTTGGAGGTCCTGAACCAACATACTGAGACAGAGTCTCTCCATCTGAGACTTTGCAGCCAGGAATGTTTACCACAAGCCAGTGATGCCACTCCCGATACTTGGGTGTTTGCCGGCTCGGAGCATCGGGGTCAGTCATGCATAGCGTGTAAAGGGCGCCAGCGTCCGTCGGGTAAGAAACAGTTGGTGGACACTGGACTTGCGTCGGCGTCAGCGTATTTCCCATGTTAACCGAGACACCGTTGTAGGTCACCTCAACGGTATCTTTCGGGACCGTGTCGATCACGTCCGGAACAACTTGGTTGTCCTCCATCTTTGTCATCCCGGTGCCCTTCTGAAGGTACACACTGCTCTGTTGAAGCAAACGGCGAAACGACGATGAAAACCCGGATGCAGATGCTGCTCTCTGGGAAGAGGTCAAAGAAGCGCTGAGGGGGCCGAACGCAAAGATTGCTCGCGAACAGACAGCTCGAAACATCATTAATTTTTAAGTGCAAGAATCGCTGGTATGCTTTTTTTTACGCAAAACTACAAAGAGCTTGAGCGTTTGTCACGCGGATAGATTGTTCGCAACAAGGTCAGCGTTTTAACGCCATCTGTGG

>SG1205099

CTTGTCACGTTTTTTATTTCTCTGTACTCTTTTAAATAGCTATGCATGAATGAAAATGAAAACTGCACATATACAGACACACTCATGATGGCGATGATTTGGCAGCCTTTTGCTCAGCCCACTTGCGCTCAAACTCTTTCTGCTCCCTCCACGCTTTTACTTTGGGGTCCTCATACTCCTCCTCTGGGTAAATGCTGAAAGGCCTCAGGTCCTTGTATTTTCCAATGCGAAAGCGTTCCTTCCTCATCTCATCCACCAGCCAGCTTGGTGTGCCTTGTGGAATCTTGTATATATTGGGGTACTTTGGCATGGGTGGCTCATCTTCCAAAGGATTGAGAGATTTCAGGCGCTTAAGGTAAACTTCTTCACGGATGTCCCTTGGGTCCCGGTACATGTCAAGGTACGTGTTAAAAGGCTGCTTGTGAGGGTACAGAACTTGCTTTGGGACGTATTCTGGAGGGTGGACATACTCAAAAGTGGGGACTCGCATTTTCAAAGTTTTGTGAAAGAAGTCCACCAGGCTGTCCTCCCAGGTGCACTGAAAGAATGCTAGGCCAGCAGGTGTGAGCACATTTTCGAATTCCTTGTAGAAGTCATAGGTTTTAAATGTCCTCTCCTTCAGGCTGGTACTGTTTGCAGACAATTTCTGGTTAGAGTAGTCAATTAATCCTTCTTGTTTGTACAAAACAAACACAAACCGATGATAGCCAGTACCACGAGGAAGAAAAGGCTGCATGTAGTCGCACACCACTTCACCACTTGGAACCTGGTTTCCTTTTATGTTGCCAATGAACCAATGAAGATACTCTTTGTCATTTTCAAGGAGATGAGAATCCAAGCTTGTCAAGACAAGTGTCCAAAGCATGTTAGGCTCGGAATCAAATAAAACTGTTGGTGCTTTTGCAGCCTCAGAAGGAAGAATTATGTTACCATAATGAACAGGTGTGAAATACTCTGCATCATACTCGTACAAAACATCCATTGGAATAACAGGGTGAAAGAATCCAAACTCATAAAGGTCTCTGAAGACACCGTAGTGGTCTGCCACTTCTTTCACGTGCATTGGTGCATAGGTTTTTCTCCATTCTTGCCTGGCTTTTTCACATTGTATCTTCAAGGTTCCATTACGTGACTTCTCAGCAAGGTCATAATTGTCCTTGTTCTCTTGGATTATCTTTCGTTGTCTTTTCCATTCTTGTTTATTGACACTGTGAGGTGCGACCTTGAAACCAATATTGACTCGTTCTTCAAACACTGGCTCTTTTGCTTTTAGAGCTTGCATCTTCTCTTCCAAACTTTTCAAGGTATCCATTGGCGGAAGCCGAACACAACGAGCCATTGCAGGCTCAAGGCGCCTCAAAGCCCGAAAGTTTTTCAAAAATGCGGCCAGTGTAGCCATTGCTGAAATTTTAAAGAGTTGCGTCCAGGTATTTTACGAACTCTGCTGCAAAGTAACACATTCATAAGACACAACCCTCGATCAGGCAGAAGCAGCAGTAGCAAGCGCTAAACCACTGGCAAAACCATGCCATCCATGCCGTGCGCAGCACC

>SG1208158

CGGCACCCGTGTGAGGGACAACTCTGCCCAGTCGCCCAAGTGTGCGTCACCCCACGAAGGCACAAATATGAGGCTTGTTTTGCCTTCGTCCCTGGTTCTCTTGGCCCTATGGAGCAGCTGCCACGCGGAGGAACCTGCGGACGTATGGACATCCGCAGGCATAGTGAGCGACCTCTCGCTTCCAGACACGCTGCGTGCCGATCTGGAAGTGAGGTACGGGGATCATCTCTCTGTGGTCAAGAACGGCACTCTGACGCCAGCGCAGACTGCCGAGGCCCCGACGCTGGTCAGACTGAGGGGCGCCATCAACTGCATTCCGCCCTTCGCACTCGTCATGTTGGACCCGGACGCACCGAGCCGTGAGAACCCCACAGCGCGCAGTAGGCTGCATTGGATGGTGCTGAATGCGGACAGCACGAC

>SG12013536

CACGTATCGGTGCAGTCCAGATCCTTGGGGTGGAGCGGGCCCGTTGTAAGGCAACGCGGCGTCCCCTTCCTGGAGCTTCTCCGTGCTGTTGATGTTGAACACCATCCAGTGCATCCAGCTGCGGAACACCGGGTTCTTGCGACTGGTCGCGTCCGGGTCCACCATGAGCAGCGCGTACGGACCTTCGCAGTCAACCGGAACATTCAGGCTCACCGTAGGCGCAGTGCTGGCTTGCTGAGGCGT

>SG12018365

CCACGAAGGGGACGCCGCGTTGCCCTACAGAGGGCCCGCTCCACCCCAGGGATCTGGACTGCACCGATACGTGTTCCTGGTGTACTGTCAGCGCGGAATGCGCTTGCAAGCCAAAGATCTGGCGCCAAAGGAAAGGAAGAACTTCAACCTGGCCGAATTCGTCAACAGGACGAGCCTGGGCACGCCGTTGGCTGGAAACTTCTTCGTCGCTGAGAACCCCGCAGCTGTGTAGTTCAGTCTTGACGCTTCACAGTCTCTTTCCTGAAAGCGCCGCCGCGGTAAGGACGGACTGTCGATGATGGAGTAGACCGCGGTCGCCTAGGAAACCAGTAAGGAAGCTCCGTCCCGTTCTAGAAATTTGTCAAGACGCCTTTAGCTGTCAGTCCTCTGTGACACGTTCACCTTAAGCCCAGCCGAATGATATGTGCTTCGTCAGTGTCCAATCCCTCGAGAAATGCCTGCGCTTCGCCACGCATCGTTCCTTGCAACAGAACTGTCGCGATTGGCAAGTGAGTGTCGTCGTGTTGGAGGCTATCAGATGTACACAAGCCAAC

>SG96128

AAAACGCTGACCTTGTTGCGAACAATCTATCCGCGTGACAAACGCTCAAGCTCTTTGTAGTTTTGCGTAAAAAAAAGCATACCAGCGATTCTTGCACTTAAAAATTAATGATGTTTCGAGCTGTCTGTTCGCGAGCAATCTTTGCGTTCGGCCCTCTCAGCGCTTCTTTGACCTCTTCCCAGAGAGCAGCATCTGCATCCGGGTTTTCATCGTCGTTTCGCCGTTTGCTTCAACAGAGCAGTGTGTACCTTCAGAAGGGCACCGGGATGACAAAGATGGAGGACAACCAAGTTGTTCCGGACGTGATCGACACGGTCCCGAAAGATACCGTTGAGGTGACCTACAACGGTGTCTCGGTTAACATGGGAAATACGCTGACGCCGACGCAAGTCCAGTGTCCACCAACTGTTTCTTACCCGACGGACGCTGGCGCCCTTTACACGCTATGCATGACTGACCCCGATGCTCCGAGCCGGCAAACACCCAAGTATCGGGAGTGGCATCACTGGCTTGTGGTAAACATTCCTGGCTGCAAAGTCTCAGATGGAGAGACTCTGTCTCAGTATGTTGGTTCAGGACCTCCAAAAGGAACAGGTCTCCATCGGTATGTCTTTGTGGTTTACAAGCAGCCTGGGAAGCTCTCCTGTGATGAAAAGCGCCTCACAAACCGCTCTGGAGATCACCGAGGTGGCTTCAAGATCCGAGACTTTGCCAAGAAGTATCAGCTGGGAGAGCCAGTTGCTGCAAACTTCTACCAGGCAGAATGGGATGACTATGTGCCCAAGCTCTATGAGCAGCTGAGCGGCAACTGAGATCAAAGTGCACTGATTGGCCTATGGGGTTTGCCCTTTCCTGTCTGTCCTTACTGCGCTGTTCTTTCCTTGTTCTTTGCTTTCTGTTGCATAGCCCCCTCTTTGAGCTCAAGCACTTTACTGTGCTCTGGCATGCTCTGAATAAAGCAGAATTTATGTTGGTCTTAAACTCCTTCCTTGATTATTCCCAAGCCTCTTTCTCTTTTCTACATACACTACCCTTATTAGTACCCTGAGTATGGTGCATGTTTACAGTGGTGGAAACATGTCTAAATTTTTCGCTTAAGTGCAGAATATTTTTGTGCACATCTATTAGCAACTGCATAGTGCAGAATTCTGCTGTGATAGAGCAGGACACATGAATGCATGCCCAACATTTATTGTCTACACTGCATTGTGCACAATTCTGCTGTGAACATGCCCAAATCACCAGATTTGTCTGGAGGTGTCTGGTACTTTCGTCTTGCTATGCTTGCACTATGGGTCTCCTTCCTAGTAGAAAATGCTGAAATCGGCATGATAGCACAGCTGTAAGTGCATGGCCTATTGCAGTCATATGTGTGGGCTGGTACACTTTGACAGTGTTGCAGAGGTACAGGCTTGTGTTGATGGTCACATTGTGCTAAGCTGACATGACATTGATGAATGCATGCCTGAACCAATTGCGACAAATCCGTGCAGTTATTGTCAAAAATAAGTGTGCACTATGCAAGTTTGCTCAGGGGCATGCATTCAGAGGGTCAGTCAAATGGCAACTTCCTTTTGTGTACCGCAGCATTTTATTTTTTTTTTGTGCTCGCCTTGGGCTAAGGAATGCCCTTTGGCATTGCCTAATAAACACAGTTTGTGCTGGAAAAAAAGAA

>SG962995

TTCCTTGTCACGTTTTTTATTTCTCTGTACTCTTTTAAATAGCTATGCATGAATGAAAATGAAAACTGCACATATACAGACACACTCATGATGGCGATGATTTGGCAGCCTTTTGCTCAGCCCACTTGCGCTCAAACTCTTTCTGCTCCCTCCACGCTTTTACTTTGGGGTCCTCATACTCCTCCTCTGGGTAAATGCTGAAAGGCCTCAGGTCCTTGTATTTTCCAATGCGAAAGCGTTCCTTCCTCATCTCATCCACCAGCCAGCTTGGTGTGCCTTGTGGAATCTTGTATATATTGGGGTACTTTGGCATGGGTGGCTCATCTTCCAAAGGATTGAGAGATTTCAGGCGCTTAAGGTAAACTTCTTCACGGATGTCCCTTGGGTCCCGGTACATGTCAAGGTACGTGTTAAAAGGCTGCTTGTGAGGGTACAGAACTTGCTTTGGGACGTATTCTGGAGGGTGGACATACTCAAAAGTGGGGACTCGCATTTTCAAAGTTTTGTGAAAGAAGTCCACCAGGCTGTCCTCCCAGGTGCACTGAAAGAATGCTAGGCCAGCAGGTGTGAGCACATTTTCGAATTCCTTGTAGAAGTCATAGGTTTTAAATGTCCTCTCCTTCAGGCTGGTACTGTTTGCAGACAATTTCTGGTTAGAGTAGTCAATTAATCCTTCTTGTTTGTACAAAACAAACACAAACCGATGATAGCCAGTACCACGAGGAAGAAAAGGCTGCATGTAGTCGCACACCACTTCACCACTTGGAACCTGGTTTCCTTTTATGTTGCCAATGAACCAATGAAGATACTCTTTGTCATTTTCAAGGAGATGAGAATCCAAGCTTGTCAAGACAAGTGTCCAAAGCATGTTAGGCTCGGAATCAAATAAAACTGTTGGTGCTTTTGCAGCCTCAGAAGGAAGAATTATGTTACCATAATGAACAGGTGTGAAATACTCTGCATCATACTCGTACAAAACATCCATTGGAATAACAGGGTGAAAGAATCCAAACTCATAAAGGTCTCTGAAGACACCGTAGTGGTCTGCCACTTCTTTCACGTGCATTGGTGCATAGGTTTTTCTCCATTCTTGCCTGGCTTTTTCACATTGTATCTTCAAGGTTCCATTACGTGACTTCTCAGCAAGGTCATAATTGTCCTTGTTCTCTTGGATTATCTTTCGTTGTCTTTTCCATTCTTGTTTATTGACACTGTGAGGTGCGACCTTGAAACCAATATTGACTCGTTCTTCAAACACTGGCTCTTTTGCTTTTAGAGCTTGCATCTTCTCTTCCAAACTTTTCAAGGTATCCATTGGCGGAAGCCGAACACAACGAGCCATTGCAGGCTCAAGGCGCCTCAAAGCCCGAAAGTTTTTCAAAAATGCGGCCAGTGTAGCCATTGCTGAAATTTTAAAGAGTTGCGTCCAGGTATTTTACGAACTCTGCTGCAAAGTAACACATTCATAAGACACAACCCTCGATCAGGCAGAAGCAGCAGTAGCAAGCGCTAAACCACTGGCAAAACCATGCCATCCATGCCGTGCG

>SG9610797

CGTGCAAAAAACAGCTGGCTTCCGAGAAGTCTTTGCCTCCCGCATTAGAACTCTTTCACTCTTATAGTCGCCATCCTCACAAGCATCATCAGCAGACCACAGCCATCACAACTCACTGCGTAATACCCAGAAAGGAAAATCAATATTACGATGCTGCAGAGGACTAAAAGGAATTATTACATTAATTTAATTCTTATTCAGTGACAGCACACATGCAGCCGCAAATTCTGAGCAAGTCAGTAAATGTCGGCCTTCATCGAACCACTCATCACGTAGTTCTACTAACACACATCATGCGTTCTCGGCGTAAAAGAAGTTGCCTCCGAACGGGTTTCCCGCCTTCAGTTTTCTGATGAATTTAGCCAGGTTGAAGTTGTTCCTCCGCCTCGGCGCAATCTTCCTGCCCCGCACGCGCTTGCGGCCCTGACAGAAGGCCAGAAACACGTAACGATGTGGTCCTGATCTCCTGGGTGGGGTCGGACCGTTGTACCGCTTAACTGTTTTGCCTTCATGCAGCCTCCTCGTGCTTTTCGCGTTCACCACCATCCAGTGCAACCAGCTGCGTGCCGTAGGTCTCCTGCGACTCGGTGCATCCGGGTCGATCATCACCAGCGCGAACGGAGGCATGCAGTTGACGGCTCCTCTCAGTCTGACCATCGTCGGAGGCTTGGCAGTCTGTGCGGGCGTCAGTGTTCCGTTCATGACCACCGACACCCTCCCGTACCTCACTTCGAGCTCGGCATGCAGCGTGTCAGGTAGCGAGAGGTCCCTCACAAGTCCCGAGGACGTCCATAAATCCAGATCAGACTCTGCATGCTGCCTCCACTGTGACGACGATTTCGTCGCAGCCCAGGGCGCGGTAGCCTCTTTAACCTGGCTGCCACTCCATAGGGCCAAGATAAGCAAGGCTGACGTCGGAAGGAGCCTCATCTTCTCGTCTTCCGCCAAACGGCGCTCACTACTAGAGTGACCATGCAGAGTCATCTGACGCGGAAGCCGCGGACACTCCTGCTGCGCCGCCGGCTGCCGAAGTCCTTGAGTGTTTTTCTCACTAGTCGCCCCGCCCCTTGCAGCAG

>SG9620367

CTGTTGGCTGTGTCTACATCTGATAGCTTCGAACACGACGACGCTCACTTGCCAATCGCGGCAGTTTTGCTGCAAGAAACGATGTGTGGCGAAGCGCAGGCATTTCTCGAGGGATTGGACACTGACGAAGCACATATCAATCGGCTGGGCTTACGGTGAATGTGTCACAGAGGACTTGTGCGGCCGGACAGCTAAAGGCGTCTTGACAAATTTCTAGAACGGGACGGAGCTTCCTTACTGGTTTCCTAGGCGACCGCGGTCTACTCCATCATCGACAGTCCGTCCTTACCGCGGCGGCGCTTTCAGGAAAGAGACTGTGAAGCGTCAAGACTGAACTACACAGCTGCAGGGTTCTCAGCGACGAAGAAGTTTCCAGCCAACGGCGTGCCCAGGCTCGTCCTGTTGACGAATTCGGCCAGGTTGAAGTTCTTCCTTTCCTTTGGCGCCAGATCTTTGGCTTGCAAGCGCATTCCGCGCTGACAGTACACCAGGAACACGTATCGGTGCAGTCCAGATCCTTGGGGTGGAGCGGGCCCGTTGTAAGGCAACGCGGCGTCCCCTTCCTGGAGCTTCTCCGTGCTGTTGATGTTCAACACCATCCAGTGCATCCAGCTGCGGAACACCGGGTTCTTGCGACTTGTCGCGTCCGGATCCACCATGAGCAGCGCGTACGGACCTTCGCAGTCAACCGGAGCATTCAGGCTCACCGTAGGCGCAGTGCTGGCTTGCTGAGGCGTCAGCGTGTTGCCCAGCGTCACTTTCAAATCGCCCGCGTAAGTCACTTCGAGCGTGGCATTCGGCGCTCCGGGCAGAGAGAGGTCGGAGACCAGTTCCTCCTTAATCCACACAGACTGTCCAGGATCCACCACCGTCTGGTTTGCCGTCGTCTGGTTTTCCG

>SG9621335

CTCTGCCCAGTCGCCCAAGTGTGCGTCACCCCACGAAGGCACAAATATGAGGCTTGTTTTGCCTTCGTCCCTGGTTCTCTTGGCCCTATGGAGCAGCTGCCACGCGGAGGAACCTGCGGACGTATGGACATCCGCAGGCATAGTGAGCGACCTCTCGCTTCCAGACACGCTGCGTGCCGATCTGGAAGTGAGGTACGGGGATCATCTCTCTGTGGTCAAGAACGGCACTCTGACGCCAGCGCAGACTGCCGAGGCCCCGACGCTGGTCAGACTGAGGGGCGCCATCAACTGCATTCCGCCCTTCGCACTCGTCATGTTGGACCCGGACGCACCGAGCCGTGAGAACCCCACAGGGCGCAGTAGGCTGCATTGGATGGTGCTGAATGCGGACAGCACGACGAGGCTGCATGAAGGCGAAGTAGCGGTGCCGTACACGGGTCCCAACCCGCCCAGGGGATCGGGAGCACATCGGTACGTGTTTCTGGCCTACTGTCAAGGCGCCCAGCGTGCGTTGACCAGCGAATTTGCCCCACAGCAAAGGAACAACTTCAACCTGACAGCTTTCGTCGAGAGATTGAATGCAG

>SG481744

GTTTTCAGTAAGCTCCGACTCTACGCCACAGATGGCGTTAAAACGCTGACCTTGTTGCGAACAATCTATCCGCGTGACAAACGCTCAAGCTCTTTGTAGTTTTGCGTAAAAAAAAGCATACCAGCGATTCTTGCACTTAAAAATTAATGATGTTTCGAGCTGTCTGTTCGCGAGCAATCTTTGCGTTCGGCCCCCTCAGCGCTTCTTTGACCTCTTCCCAGAGAGCAGCATCTGCATCCGGGTTTTCATCGTCGTTTCGCCGTTTGCTTCAACAGAGCAGTGTGTACCTTCAGAAGGGCACCGGGATGACAAAGATGGAGGACAACCAAGTTGTTCCGGACGTGATCGACACGGTCCCGAAAGATACCGTTGAGGTGACCTACAACGGTGTCTCGGTTAACATGGGAAATACGCTGACGCCGACGCAAGTCCAGTGTCCACCAACTGTTTCTTACCCGACGGACGCTGGCGCCCTTTACACGCTATGCATGACTGACCCCGATGCTCCGAGCCGGCAAACACCCAAGTATCGGGAGTGGCATCACTGGCTTGTGGTAAACATTCCTGGCTGCAAAGTCTCAGATGGAGAGACTCTGTCTCAGTATGTTGGTTCAGGACCTCCAAAAGGAACAGGTCTCCATCGGTATGTCTTTGTGGTTTACAAGCAGCCTGGGAAGCTCTCCTGTGATGAAAAGCGCCTCACAAACCGCTCTGGAGATCACCGAGGTGGCTTCAAGATCCGAGACTTTGCCAAGAAGTATCAGCTGGGAGAGCCAGTTGCTGCAAACTTCTACCAGGCAGAATGGGATGACTATGTGCCCAAGCTCTATGAGCAGCTGAGCGGCAACTGAGATCAAAGTGCACTGATTGGCCTATGGGGTTTGCCCTTTCCTGTCTGTCCTTACTGCGCTGTTCTTTCCTTGTTCTTTGCTTTCTGTTGCATAGCCCCCTCTTTGAGCTCAAGCACTTTACTGTGCTCTGGCATGCTCTGAATAAAGCAGAATTTATGTTGGTCTTAAACTCCTTCCTTGATTATTCCCAAGCCTCTTTCTCTTTTCTACATACACTACCCTTATTAATACCCTGAGTATGGTGCATGTTTACAGTGGTGGAAACATGTCTAAATTTTTCGCTTAAGTGCAGAATATTTTTGTGCACATCTATTAGCAACTGCATAGTGCAGAATTCTGCTGTGATAGAGCAGGACACATGAATGCATGCCCAACATTTATTGTCTACACTGCATTGTGCACAATTCTGCTGTGAACATGCCCAAATCACCAGATTTGTCTGGAGGTGTCTGGTACTTTCGTCTTGCTATGCTTGCACTATGGGTCTCCTTCCTAGTAGAAAATGCTGAAATCGGCATGATAGCACAGCTGTAAGTGCATGGCCTATTGCAGTCATATGTGTGGGCTGGT

>SG4810946

GTCCTTGTCAAGTTTTTTATTTCTCTGTACTCTTTTAAATAGCTATGCATGAATGAAAATGAAAACTGCACATATACAGACACACTCATGATGGCGATGATTTGGCAGCCTTTTGCTCAGCCCACTTGCGCTCAAACTCTTTCTGCTCCCTCCACGCTTTTACTTTGGGGTCCTCATACTCCTCCTCTGGGTAAATGCTGAAAGGCCTCAGGTCCTTGTATTTTCCAATGCGAAAGCGTTCCTTCCTCATCTCATCCACCAGCCAGCTTGGTGTGCCTTGTGGAATCTTGTATATATTGGGGTACTTTGGCATGGGTGGCTCATCTTCCAAAGGATTGAGAGATTTCAGGCGCTTAAGGTAAACTTCTTCACGGATGTCCCTTGGGTCCCGGTACATGTCAAGGTACGTGTTAAAAGGCTGCTTGTGAGGGTACAGAACTTGCTTTGGGACGTATTCTGGAGGGTGGACATACTCAAAAGTGGGGACTCGCATTTTCAAAGTTTTGTGAAAGAAGTCCACCAGGCTGTCCTCCCAGGTGCACTGAAAGAATGCTAGGCCAGCAGGTGTGAGCACATTTTCGAATTCCTTGTAGAAGTCATAGGTTTTAAATGTCCTCTCCTTCAGGCTGGTACTGTTTGCAGACAATTTCTGGTTAGAGTAGTCAATTAATCCTTCTTGTTTGTACAAAACAAACACAAACCGATGATAGCCAGTACCACGAGGAAGAAAAGGCTGCATGTAGTCGCACACCACTTCACCACTTGGAACCTGGTTTCCTTTTATGTTGCCAATGAACCAATGAAGATACTCTTTGTCATTTTCAAGGAGATGAGAATCCAAGCTTGTCAAGACAAGTGTCCAAAGCATGTTAGGCTCGGAATCAAATAAAACTGTTGGTGCTTTTGCAGCCTCAGAAGGAAGAATTATGTTACCATAATGAACAGGTGTGAAATACTCTGCATCATACTCGTACAAAACATCCATTGGAATAACAGGGTGAAAGAATCCAAACTCATAAAGGTCTCTGAAGACACCGTAGTGGTCTGCCACTTCTTTCACGTGCATTGGTGCATAGGTTTTTCTCCATTCTTGCCTGGCTTTTTCACATTGTATCTTCAAGGTTCCATTACGTGACTTCTCAGCAAGGTCATAATTGTCCTTGTTCTCTTGGATTATCTTTCGTTGTCTTTTCCATTCTTGTTTATTGACACTGTGAGGTGCGACCTTGAAACCAATATTGACTCGTTCTTCAAACACTGGCTCTTTTGCTTTTAGAGCTTGCATCTTCTCTTCCAAACTTTTCAAGGTATCCATTGGCGGAAGCCGAACACAACGAGCCATTGCAGGCTCAAGGCGCCTCAAAGCCCGAAAGTTTTTCAAAAATGCGGCCAGTGTAGCCATTGCTGAAATTTTAAAGAGTTGCGTCCAGGTATTTTACGAACTCTGCTGCAAAGTAACACATTCATAAGACACAACCCTCGATCAGGCAGAAGCAGCAGTAGCAAGCGCTAAACCACTGGCAAAACCATGCCATCCATGCCGTGCGCAGCACCCTGGCAGCACTGGATCAAGTTGG

>SG4816750

TTTTTGAAGTGCAAGTTTTTATTGACTCACTGCTGGCTGTGTACATCTGATACCTTCGAACACGACGACACCCACTTGCCAATCGCGGCAGTTCTGTTGCAAGAAACGATGTGTGGCGAAGCGCAGGCATTTCTCGAGGGATTGGACACTGACGAAGCACATATCATTCGGCTGGGCTTACGNNNNNNGGACTTGTGCAACCTGACAGCTAAAGGCGTCTTGACAAATTTCTAGAACGGGACGGAGCTTCCTTACTGGTTTCCTAGGCGACCGCGGTCTACTCCATCATCGACGGTCCGTCCTTACCGCGGCGGCGCTTTCAGGAAAGAGACTGTGAAGCGTCAAGACTGAACTACACAGCTGCGGGGTTCTCAGCGACGAAGAAGTTTCCAGCCAACGGCGTGCCCAGGCTCGTCCTGTTGACGAATTCGGCCAGGTTGAAGTTCTTCCTCTCCTTCGGCGCCAGATCTTCGTCTTGCAGGCGCATTCCGCGCTGACAGTACACCAGGAACACGTATCGGTGCAGTCCAGATCCTTGGGGTGGAGCGGGCCCGTTGTAAGGCAACGCGGCGTCCCCTTCCTGGAGCTTCTCCGTGCTGTTGATGTTCAACACCATCCAGTGCATCCAGCTGCGGAACACCGGGTTCTTGCGACTGGTCGCGTCCGGGTCCACCATGAGCGGCGCGTACGGACCTTCGCAGTCAACCGGAGCATTCAGGCTCACCGTAGGCGCAGTGCTGGCTTGCTGAGGCGTCAGCGTGTTGCCCAGCGTCACTTTCAAATCGCCCGCGTAAGTCACTTCGAGGGTGGCATTCGGCGCTCCGGGCAGCGAGAGGTCGGCGACCAGTTCCTCCTTAATCCACACAGACTGTCCAGAATCCACCACCGTCTGGTTTGCCG

>SG4819364

ACTAACACACATCATGCGTTCTCGGCGTAAAAGAAGTTGCCTCCGAAAGGATACCCTGCATTCAATCTCTCGACGAAATCTGTCAGGTTGAAGTTGTTCCTTTGCTGTGGGGCAAATTCGCTGGTCAACGCACGCTGGGCGCCTTGACAGTAGGCCAGAAACACGTACCGATGTGCTCCCGATCCCCTGGGCGGGTTGGGACCCGTGTACGGCACCGCTACTTCGCCTTCATGCAGCCTCGTCGTGCTGTCCGCATTCAGCACCATCCAATGCAGCCTACTGCGCCCTGTGGGGTTCTCACGGCTCGGTGCGTCCGGGTCCAACATGACGAGTGCGAAGGGCGGAATGCAGTTGATGGCGCCCCTCAGTCTGACCAGCGTCGGGGCCTCGGCAGTCTGCGCTGGCGTCAGAGTGCCGTTCTTGACCACAGAGAGATGATCCCCGTACCTCACTTCCAGATCGGCACGCAGCGTGTCTGGAAGCGAGAGGTCGCTCACTATGCCTGCGGATGTCCATACGTCCGCAGGTTCCTCCGCGTGGCAGCTGCTCCATAGGGCCAAGAGAAGCAGGGACGAAGGCAAAACAAGCCCCATATTTGTGTTTTCGTGTGGCTAAGCACACTTGCACGACTGGGCAGAGCAGTCTGTTGACGGCACCCGTGTGAGAGACAAC

>SG4822164

GGCAGCAACTGCCGCAGCTGCTGCGAGGGGCGAGAAAAACACTCAAGGACTTCGGCAGCCGGCGCAGCAGGAGTGTCCGCAGTTTCCGCGTCAGATGACTCTGCTTGGTCACTCTAGTAGTGAGCGCCGTTTGGCGGAAGACGAGAAGATGAGGCTCCTTCCGACGTCAACCTTGCTTATCTTGGCCCTATGGAGTGGCAGCCAGGTGAAAGAGGCAACCGCACCGTGGGCTCCGACGAAATCGTCCTCCCAGTGGAGGCAGCATGCAGAGTCTGATCTGGATTTATGGACGTCCTCGGGACTTGTGAGGGACCTCTCACTACTTGACACGCTGCATGCCGAGCTCGAAGTGAGGTACAGGAGGGTGTCGGTGGTCATGAACGGAACACTGACGCCCGCACAGACTGCCAAGCCTCCGACGATGGTCAGACTGAGAGGAGCCGTCAACTGCATGCCTCCGTTCGCGCTGGTGATGATCGACCCGGATGCACCGAGTCGCAGGAGACCTACGGCACGCAGCTGGTTGCACTGGATGGTGGTGAACGTGAAAAGCACGAGGAGGCTGCATGAAGGCAAAACAGTTAAGCGGTACAACGGTCCGACCCCACCCAGGAGATCAGGACCACATCGTTACGTGTTTCTGGCCTTCTGTCAGGGCCGCAAGCGCGTGCGGGGCAGGAAGATTGCGCCGAGGCGGAGGAACAACTTCAACCTGGCTAAATTCATCAGAAAACTGAAGGCGGGAAACCCGTTCGGAGGCAACTTCTTTTACGCCGAGAACGCATGATGTGTGTTAGTGGGACTACGTGATGAGTGGTTCGATGAAGGTCGACATTTACTGACTTGCTCAGAAATTGCGGCTGCATGTGTGCTGTCACTGAATAAGAATGAAATTAATGTAATAATTCCTTTCCGTCCTCTGCAGCATTGTAATATTGATTTTCCTTTCTGGGTATTACGCAGTGAGTTGTGATGGCTGTGGTCTGCTGATGATGCTTACGAGGATGGCGACTATAAGAGTGAAAGAGTTCTAATGCGGGAGGCAAAGACTTCTCGAAAGCCAGCTGTTTTTTGCACGTCATGAAGAAAGTGCATAGAAAAACATAGCGAGAACTGACGTGAAGTAAATAAAGTGGTGCAAAATTTTAATTAAAGGAGCGTCTGTCAAATTAAATATATAGATAACAGCTTCCATTGTATTGTCATTAAATTTTCAATTCATATCACTTGATCAAGTTTTAGCGCTGCTATCAGGGAAAAGTTTCCTCTGTGGCGCAGTGTTGTTTGTGAATAAAGAACGCAGACATAAAATTAAGTACAGCAGATGCTCCACTAAACATTTACGCTGCGTTAGGAGTGATGGATGTATTCCGGGTGAGTGCTAGTGTTTATGTTCTGCAGAACAATTCATACGTGTCAAAACGAGTAAGGCCTTGGGCCAGTGTGAAATGAGATCGAACAATAAAT

>MG120427

CCACAGATGGCGTTAAAACGCTGACCTTGTTGCGAACAATCTATCCGCGTGACAAACGCTCAAGCTCTTTGTAGTTTTGCGTAAAAAAAAGCATACCAGCGATTCTTGCACTTAAAAATTAATGATGTTTCGAGCTGTCTGTTCGCGAGCAATCTTTGCGTTCGGCCCTCTCAGCGCTTCTTTGACCTCTTCCCAGAGAGCAGCATCTGCATCCGGGTTTTCATCGTCGTTTCGCCGTTTGCTTCAACAGAGCAGTGTGTACCTTCAGAAGGGCACCGGGATGACAAAGATGGAGGACAACCAAGTTGTTCCGGACGTGATCGACACGGTCCCGAAAGATACCGTTGAGGTGACCTACAACGGTGTCTCGGTTAACATGGGAAATACGCTGACGCCGACGCAAGTCCAGTGTCCACCAACTGTTTCTTACCCGACGGACGCTGGCGCCCTTTACACGCTATGCATGACTGACCCCGATGCTCCGAGCCGGCAAACACCCAAGTATCGGGAGTGGCATCACTGGCTTGTGGTAAACATTCCTGGCTGCAAAGTCTCAGATGGAGAGACTCTGTCTCAGTATGTTGGTTCAGGACCTCCAAAAGGAACAGGTCTCCATCGGTATGTCTTTGTGGTTTACAAGCAGCCTGGGAAGCTCTCCTGTGATGAAAAGCGCCTCACAAACCGCTCTGGAGATCACCGAGGTGGCTTCAAGATCCGAGACTTTGCCAAGAAGTATCAGCTGGGAGAGCCAGTTGCTGCAAACTTCTACCAGGCAGAATGGGATGACTATGTGCCCAAGCTCTATGAGCAGCTGAGCGGCAACTGAGATCAAAGTGCACTGATTGGCCTATGGGGTTTGCCCTTTCCTGTCTGTCCTTACTGCGCTGTTCTTTCCTTGTTCTTTGCTTTCTGTTGCATAGCCCCCTCTTTGAGCTCAAGCACTTTACTGTGCTCTGGCATGCTCTGAATAAAGCAGAATTTATGTTGGTCTTAAACTCCTTCCTTGATTATTCCCAAGCCTCTTTCTCTTTTCTACATACACTACCCTTATTAATACCCTGAGTATGGTGCATGTTTACAGTGGTGGAAACATGTCTAAATTTTTCGCTTAAGTGCAGAATATTTTTGTGCACATCTATTAGCAACTGCATAGTGCAGAATTCTGCTGTGATAGAGCAGGACACATGAATGCATGCCCAACATTTATTGTCTACACTGCATTGTGCACAATTCTGCTGTGAACATGCCCAAATCACCAGATTTGTCTGGAGGTGTCTGGTACTTTCGTCTTGCTATGCTTGCACTATGGGTCTCCTTCCTAGTAGAAAATGCTGAAATCGGCATGATAGCACAGCTGTAAGTGCATGGCCTATTGCAGTCATATGTGTGGGCTGGT

>MG120674

TAAATGCAACATTTCAGGCGTTCTCGGCGTAAAAGAAGTTGCCTCCGAAAGGGTACCCTGCATTCAATCTCTCGACGAAATCTGTCAGGTTGAAGTTGTTCCTTTGCTGTGGGGCAAATTCGCTGGTCAACACACGCTGGGCGCCTTGACAGTAGGCCAGAAACACGTACCGATGTGCTCCCGATCCCTTCGGTGGGTTGGGACCCGTGTACGGCACCGCTACTTCGCCTTCATGCAGCCTTGTCGTGCTGTCCGCATTCAGCACCATCCAATGCAGCCTACTGCGCGCTGTGGGGTTCTCACGGCTCGGTGCGTCCGGGTCCAACATGACGAGTGCGAAGGGCGGAATGCAGTTGATGGCGCCCCTCAGTCTGACCAACGTCGGGGCCTCGGCAGTCTGCGCGGGCGTCAGAGTGCCGTTCTTGANNNNNNNNNNNNNNNNNNNCCTGCGGATGTCCATACGTCCGGCTCAGACTGCGACGATGCCGCAGGTTCCTCCGCGTGGCAGCTGCTCCATAGGGCCAAGAGAAGCAGGGACGAAGGCAAAACAAGCCCCATATTTGTGTCTTCGTGTGGCGACGCACACTTGGGCGACTGGGCAGAGTTGTCTCTCACACGGGTGCCGTCAACAG

>MG1201292

ACGGTCGGGTTCGAACCCGGGACCAAACGCGGGTATCTAATCGCGCAGAATGTGTTATCGCGTTTGCAGCGGCCCAAGCGGCTGACAAAAGCAGTTTTGAGTCACCGAATGGAACAACTGCAGTGCCACCTTGGGAGCGCAGGTTCCCCATCGCTACAAAATTGCTGGTAGTNNNNNNNNAGCGCAGGTTCCCCATCGCTACAAAATTGCTGGTAGTCTAGCTCTAGTTAACCCTGCTAGAAAGCGAAAAGCTGCATCTGTCAGGCTAAAGAGCGACCACTTTTGTGTCGCTGATAACGCTACCTCTGCTCGAAGCGATTGCCGCGGCTGCTGCGAGGGGCGTATCGACCAATGAGATAAACATACAAGGACGGCGAAAGCCGAGTCAGCGCAGCAGTCTGTTGACGGCACCCGTGTGAGGGACAACTCTGCCCAGTCGCCCAAGTGTGCGTCACCCCACGAAGGCACAAATATGAGGCTTGTTTTGCCTTCGTCCCTGGTTCTCTTGGCCCTATGGAGCAGCTGCCACGCGGAGGAACCTGCGGACGTATGGACATCCGCAGGCATAGTGAGCGACCTCTCGCTTCCAGACACGCTGCGTGCCGATCTGGAAGTGAGGTACGGGGATCATCTCTCTGTGGTCAAGAACGGCACTCTGACGCCAGCGCAGACTGCCGAGGCCCCGACGCTGGTCAGACTGAGGGGCGCCATCAACTGCATTCCGCCCTTCGCACTCGTCATGTTGGACCCGGACGCACCGAGCCGTGAGAACCCCACAGGGCGCAGTAGGCTGCATTGGATGGTGCTGAATGCGGACAGCACGACGAGGCTGCATGAAGGCGAAGTAGCGGTGCCGTACACGGGTCCCAACCCGCCCAGGGGATCGGGAGCACATCGGTACGTGTTTCTGGCCTACTGTCAAGGCGCCCAGCGTGCGTTGACCAGCGAATTTGCCCCACAGCAAAGGAACAACTTCAACCTGACAGATTTCGTCGAGAGATTGAATGCAGGGTATCCTTTCGGAGGCAACTTCTTTTACTCCGAAAACGCCTGAAATGTTGCATTTAGATCAACTTTATTTTTTAAAGTGTGGCTAGAGTTGGACTGACCTATGATGAGAGCCCTGCAATTGACGAGATGCGGCGATGTGCTAATTAATAGCCCACGCCAATATGCATGCCGACTGAATAAAAATAGTATTAGTTGCAAAAAAAAAA

>MG12011686

CTGCCAGGGTGCTGCGCACGGCATGGATGGCATGGTTTTGCCAGTGGTTTAGCGCTTGCTACTGCTGCTTCTGCCTGATCGAGGGTTGTGTCTTATGAATGTGTTACTTTGCAGCAGAGTTCGTAAAATACCTGGACGCAACTCTTTAAAATTTCAGCAATGGCTACACTGGCCGCATTTTTGAAAAACTTTCGGGCTTTGAGGCGCCTTGAGCCTGCAATGGCTCGTTGTGTTCGGCTTCCGCCAATGGATACCTTGAAAAGTTTGGAAGAGAAGATGCAAGCTCTAAAAGCAAAAGAGCCAGTGTTTGAAGAACGAGTCAATATTGGTTTCAAGGTCGCACCTCACAGTGTCAATAAACAAGAATGGAAAAGACAACGAAAGATAATCCAAGAGAACAAGGACAATTATGACCTTGCTGAGAAGTCACGTAATGGAACCTTGAAGATACAATGTGAAAAAGCCAGGCAAGAATGGAGAAAAACCTATGCACCAATGCACGTGAAAGAAGTGGCAGACCACTACGGTGTCTTCAGAGACCTTTATGAGTTTGGATTCTTTCACCCTGTTATTCCAATGGATGTTTTGTACGAGTATGATGCAGAGTATTTCACACCTGTTCATTATGGTAACATAATTCTTCCTTCTGAGGCTGCAAAAGCACCAACAGTTTTATTTGATTCCGAGCCTAACATGCTTTGGACACTTGTCTTGACAAGCTTGGATTCTCATCTCCTTGAAAATGACAAAGAGTATCTTCATTGGTTCATTGGCAACATAAAAGGAAACCAGGTTCCAAGTGGTGAAGTGGTGTGCGACTACATGCAGCCTTTTCTTCCTCGTGGTACTGGCTATCATCGGTTTGTGTTTGTTTTGTACAAACAAGAAGGATTAATTGACTACTCTAACCAGAAATTGTCTGCAAACAGTACCAGCCTGAAGGAGAGGACATTTAAAACCTATGACTTCTACAAGGAATTCGAAAATGTGCTCACACCTGCTGGCCTAGCATTCTTTCAGTGCACCTGGGAGGACAGCCTGGTGGACTTCTTTCACAAAACTTTGAAAATGCGAGTCCCCACTTTTGAGTATGTCCACCCTCCAGAATACGTCCCAAAGCAAGTTCTGTACCCTCACAAGCAGCCTTTTAACACGTACCTTGACATGTACCGGGACCCAAGGGACATCCGTGAAGAAGTTTACCTTAAGCGCCTGAAATCTCTCAATCCTTTGGAAGATGAGCCACCCATGCCAAAGTACCCCAATATATACAAGATTCCACAAGGCACACCAAGCTGGCTGGTGGATGAGATGAGGAAGGAACGCTTTCGCATTGGAAAATACAAGGACCTGAGGCCTTTCAGCATTTACCCAGAGGAGGAGTATGAGGACCCCAAAGTAAAAGCGTGGAGGGAGCAGAAAGAGTTTGAGCGCAAGTGGGCTGAGCAAAAGGCTGCCAAATCATCGCCATCATGAGTGTGTCTGTATATGTGCAGTTTTCATTTTCATTCATGCATAGCTATTTAAAAGAGTACAGAGAAATAAAAAACGTGACAAGGAATTAAAA

>MG12039353

GTCTTGACAAATTTCTAGAACGGGACGGAGCTTCCTTACTGGTTTCCTAGGCGACCGCGGTCTACTCCATCATCGACAGTCCGTCCTTACCGCGGCGGCGCTTTCAGGAAAGAGACTGTGAAGCGTCAAGACTGAACTACACAGCTGCGGGGTTCTCAGCGACGAAGAAGTTTCCAGCCAACGGCGTGCCCAGGCTCGTCCTGTTGACGAATTCGGCCAGGTTGAAGTTCTTCCTTTCCTTTGGCGCCAGATCTTTGGCTTGCAAGCGCATTCCGCGCTGACAGTACACCAGGAACACGTATCGGTGCAGTCCAGATCCTTGGGGTGGAGCGGGCCCGTTGTAAGGCAACGCGGCGTCCCCTTCCTGGAGCTTCTCCGTGCTGTTGATATTCAACACCATCCAATGCATCCAGCTGCGGAACACCGGGTTCTTGCGACTGGTCGCGTCCGGGTCCACCATGAGCAGCGCGTACGGACCTTCGCAGTCAACCGGAACATTCAGGCTCACCGTAGGCGCAGTGCTGGCTTGCTGAGGCGTGAATGTGTAGTTGCCCAGCGCCACTTTCAAATCTCCCGCGTAGGTCACTTCCAACGTTGCGTTCG

>MG96250

CACCCGATTTGTTCTAGTTATCATCCTCACGATAATGAACATGTCTCAACCATCACCACTCGAAGTGTGATATGAAGCTAGAAAACTAACAATCCAAGCTTGTCAAGGAATCAACACACAGTATGCAACTAATACTATTTTTATTCAGTCGGCATGCATATTGGCGTGGGCTATTAATTAGCACATCGCCGCATCTCGTCAATTGCAGGGCTCTCATCATAGGTCAGTCCAACTCTAGCCACACTTTAAAAAATAAAGTTGATCTAAATGCAACATTTCAGGCGTTTTCGGAGTAAAAGAAGTTGCCTCCGAAAGGATACCCTGCATTCAATCTCTCGACGAAATCTGTCAGGTTGAAGTTGTTCCTTTGCTGTGGGGCAAATTCGCTGGTCAACGCACGCTGGGCGCCTTGACAGTAGGCCAGAAACACGTACCGATGTGCTCCCGATCCCCTGGGCGGGTTGGGACCCGTGTACGGCACCGCTACTTCGCCTTCATGCAGCCTCGTCGTGCTGTCCGCATTCAGCACCATCCAATGCAGCCTACTGCGCCCTGTGGGGTTCTCACGGCTCGGTGCGTCCGGGTCCAACATGACGAGTGCGAAGGGCGGAATGCAGTTGATGGCGCCCCTCAGTCTGACCAGCGTCGGGGCCTCGGCAGTCTGCGCTGGCGTCAGAGTGCCGTTCTTGACCACAGAGAGATGATCCCCGTACCTCACTTCCAGATCGGCACGCAGCGTGTCTGGAAGCGAGAGGTCGCTCACTATGCCTGCGGATGTCCATACGTCCGCAGGTTCCTCCGCGTGGCAGCTGCTCCATAGGGCCAAGAGAACCAGGGACGAAGGCAAAACAAGCCTCATATTTGTGCCTTCGTGGGGTGACGCACACTTGGGCGACTGGGCAGAGTTGTCCCTCACACGGGTGCCGTCAACAGACTGCTGCGCTGACTCGGCTTTCGCCGTCCTTGTATGTTTATCTCATTGGTCGATACGCCCCTCGCAGCAGCCGCGGCAATCGCTTCGAGCAGAGGTAGCGTTATCAGCGACACAAAAGTGGTCGCTGCTTAGCCTGAGAGATGCAGCTTCTCGCTTTCCAGCAGGGTTAACCAGAGCTACACTACCAGCAATTTTGTAGCGATGGGGAACCTGCGCTCCCAAGGTGGCACTGCAGTTGTTCCATTCGGTGACTCAAA

>MG962745

CGTAATGATCTGTTCAACTAAGACAGAACATTCTTTTTTCTCTTACTACTGCCGAATAGCTGCCATGTTTGTTTTCAGTAAGCTCCGACTCTACGCCACAGATGGCGTTAAAACGCTGACCTTGTTGCGAACAATCTATCCGCGTGACAAACGCTCAAGCTCTTTGTAGTTTTGCGTAAAAAAAAGCATACCAGCGATTCTTGCACTTAAAAATTAATGATGTTTCGAGCTGTCTGTTCGCGAGCAATCTTTGCGTTCGGCCCTCTCAGCGCTTCTTTGACCTCTTCCCAGAGAGCAGCATCTGCATCCGGGTTTTCATCGTCGTTTCGCCGTTTGCTTCAACAGAGCAGTGTGTACCTTCAGAAGGGCACCGGGATGACAAAGATGGAGGACAACCAAGTTGTTCCGGACGTGATCGACACGGTCCCGAAAGATACCGTTGAGGTGACCTACAACGGTGTCTCGGTTAACATGGGAAATACGCTGACGCCGACGCAAGTCCAGTGTCCACCAACTGTTTCTTACCCGACGGACGCTGGCGCCCTTTACACGCTATGCATGACTGACCCCGATGCTCCGAGCCGGCAAACACCCAAGTATCGGGAGTGGCATCACTGGCTTGTGGTAAACATTCCTGGCTGCAAAGTCTCAGATGGAGAGACTCTGTCTCAGTATGTTGGTTCAGGACCTCCAAAAGGAACAGGTCTCCATCGGTATGTCTTTGTGGTTTACAAGCAGCCTGGGAAGCTCTCCTGTGATGAAAAGCGCCTCACAAACCGCTCTGGAGATCACCGAGGTGGCTTCAAGATCCGAGACTTTGCCAAGAAGTATCAGCTGGGAGAGCCAGTTGCTGCAAACTTCTACCAGGCAGAATGGGATGACTATGTGCCCAAGCTCTATGAGCAGCTGAGCGGCAACTGAGATCAAAGTGCACTGATTGGCCTATGGGGTTTGCCCTTTCCTGTCTGTCCTTACTGCGCTGTTCTTTCCTTGTTCTTTGCTTTCTGTTGCATAGCCCCCTCTTTGAGCTCAAGCACTTTACTGTGCTCTGGCATGCTCTGAATAAAGCAGAATTTATGTTGGTCTTAAACTCCTTCCTTGATTATTCCCAAGCCTCTTTCTCTTTTCTACATACACTACCCTTATTAATACCCTGAGTATGGTGCATGTTTACAGTGGTGGAAACATGTCTAAATTTTTCGCTTAAGTGCAGAATATTTTTGTGCACATCTATTAGCAACTGCATAGTGCAGAATTCTGCTGTGATAGAGCAGGACACATGAATGCATGCCCAACATTTATTGTCTACACTGCATTGTGCACAATTCTGCTGTGAACATGCCCAAATCACCAGATTTGTCTGGAGGTGTCTGGTACTTTCGTCTTGCTATGCTTGCACTATGGGTCTCCTTCCTAGTAGAAAATGCTGAAATCGGCATGATAGCACAGCTGTAAGTGCATGGCCTATTGCAGTCATATGTGTGGGCTGGTACACTTTGACAGTGTTGCAGAGGTACAGGCTTGTGTTGATGGTCACATTGTGCAAAGCTGACATGACATTGATGAATGCATGCCTGAACCAATTGCGACAAATCCGTGCAGTTATTGTCAAAAATAAGTGTGCACTATGCAAGTTTGCTCAGGGGCATGCATTCAGAGGGTCAGTCAAATGGCAACTTCCTTTTGTGTACCGCAGCATTTTATTTTTTTTTGTGCTCGCCTTGGGCTAAGGAATGCCCTTTGGCATTGCCTAATAAACACAGTTTGTGCTGGAAAAAAAA

>MG966864

GCTCTTCCGATCTCACGTTTTTTATTTCTCTGTACTCTTTTAAATAGCTATGCATGAATGAAAATGAAAACTGCACATATACAGACACACTCATGATGGCGATGATTTGGCAGCCTTTTGCTCAGCCCACTTGCGCTCAAACTCTTTCTGCTCCCTCCACGCTTTTACTTTGGGGTCCTCATACTCCTCCTCTGGGTAAATGCTGAAAGGCCTCAGGTCCTTGTATTTTCCAATGCGAAAGCGTTCCTTCCTCATCTCATCCACCAGCCAGCTTGGTGTGCCTTGTGGAATCTTGTATATATTGGGGTACTTTGGCATGGGTGGCTCATCTTCCAAAGGATTGAGAGATTTCAGGCGCTTAAGGTAAACTTCTTCACGGATGTCCCTTGGGTCCCGGTACATGTCAAGGTACGTGTTAAAAGGCTGCTTGTGAGGGTACAGAACTTGCTTTGGGACGTATTCTGGAGGGTGGACATACTCAAAAGTGGGGACTCGCATTTTCAAAGTTTTGTGAAAGAAGTCCACCAGGCTGTCCTCCCAGGTGCACTGAAAGAATGCTAGGCCAGCAGGTGTGAGCACATTTTCGAATTCCTTGTAGAAGTCATAGGTTTTAAATGTCCTCTCCTTCAGGCTGGTACTGTTTGCAGACAATTTCTGGTTAGAGTAGTCAATTAATCCTTCTTGTTTGTACAAAACAAACACAAACCGATGATAGCCAGTACCACGAGGAAGAAAAGGCTGCATGTAGTCGCACACCACTTCACCACTTGGAACCTGGTTTCCTTTTATGTTGCCAATGAACCAATGAAGATACTCTTTGTCATTTTCAAGGAGATGAGAATCCAAGCTTGTCAAGACAAGTGTCCAAAGCATGTTAGGCTCGGAATCAAATAAAACTGTTGGTGCTTTTGCAGCCTCAGAAGGAAGAATTATGTTACCATAATGAACAGGTGTGAAATACTCTGCATCATACTCGTACAAAACATCCATTGGAATAACAGGGTGAAAGAATCCAAACTCATAAAGGTCTCTGAAGACACCGTAGTGGTCTGCCACTTCTTTCACGTGCATTGGTGCATAGGTTTTTCTCCATTCTTGCCTGGCTTTTTCACATTGTATCTTCAAGGTTCCATTACGTGACTTCTCAGCAAGGTCATAATTGTCCTTGTTCTCTTGGATTATCTTTCGTTGTCTTTTCCATTCTTGTTTATTGACACTGTGAGGTGCGACCTTGAAACCAATATTGACTCGTTCTTCAAACACTGGCTCTTTTGCTTTTAGAGCTTGCATCTTCTCTTCCAAACTTTTCAAGGTATCCATTGGCGGAAGCCGAACACAACGAGCCATTGCAGGCTCAAGGCGCCTCAAAGCCCGAAAGTTTTTCAAAAATGCGGCCAGTGTAGCCATTGCTGAAATTTTAAAGAGTTGCGTCCAGGTATTTTACGAACTCTGCTGCAAAGTAACACATTCATAAGACACAACCCTCGATCAGGCAGAAGCAGCAGTAGCAAGCGCTAAACCACTGGCAAAACCATGCCATCCATGCCGTGCGCAGCACCCTGGCAGCACTGGATCAAGTTGGACTGATTTACGGGTT

>MG481288

CGGATTTGTCGCAATTGGTTCAGGCATGCATTCATCAATGTTTTACTCTAGTCATGTCAGCTTAGCACAATGTGACCATCAACACAAGCCTGTACCTCTGCAACACTGTCAAAGTGCACCAGCCCACACATATGACTGCAATAGGCCATGCACTTACAGCTGTGCTATCATGCCGATTTCAGCATTTTCTACTAGGAAGGAGACCCATAGTGCAAGCATAGCAAGACGAAAGTACCAGACACCTCCAGACAAATCTGGTGATTTGGGCATGTTCACAGCAGAATTGTGCACAATGCAGTGTAGACAATAAATGTTGGGCATGCATTCATGTGTCCTGCTCTATCACAGCAGAATTCTGCACTATGCAGTTGCTAATAGATGTGCACAAAAATATTCTGCACTTAAGCGAAAAATTTAGACATGTTTCCACCACTGTAAACATGCACCATACTCAGGGTATTAATAAGGGTAGTGTATGTAGAAAAGAGAAAGAGGCTTGGGAATAATCAAGGAAGGAGTTTAAGACCAACATAAATTCTGCTTTATTCAGAGCATGCCAGAGCACAGTAAAGTGCTTGAGCTCAAAGAGGGGGCTATGCAACAGAAAGCAAAGAACAAGGAAAGAACAGCGCAGTAAGGACAGACAGGAAAGGGCAAACCCCATAGGCCAATCAGTGCACTTTGATCTCAGTTGCCGCTCAGCTGCTCATAGAGCTTGGGCACATAGTCATCCCATTCTGCCTGGTAGAAGTTTGCAGCAACTGGCTCTCCCAGCTGATACTTCTTGGCAAAGTCTCGGATCTTGAAGCCACCTCGGTGATCTCCAGAGCGGTTTGTGAGGCGCTTTTCATCACAGGAGAGCTTCCCAGGCTGCTTGTAAACCACAAAGACATACCGATGGAGACCTGTTCCTTTTGGAGGTCCTGAACCAACATACTGAGACAGAGTCTCTCCATCTGAGACTTTGCAGCCAGGAATGTTTACCACAAGCCAGTGATGCCACTCCCGATACTTGGGTGTTTGCCGGCTCGGAGCATCGGGGTCAGTCATGCATAGCGTGTAAAGGGCGCCAGCGTCCGTCGGGTAAGAAACAGTTGGTGGACACTGGACTTGCGTCGGCGTCAGCGTATTTCCCATGTTAACCGAGACACCGTTGTAGGTCACCTCAACGGTATCTTTCGGGACCGTGTCGATCACGTCCGGAACAACTTGGTTGTCCTCCATCTTTGTCATCCCGGTGCCCTTCTGAAGGTACACACTGCTCTGTTGAAGCAAACGGCGAAACGACGATGAAAACCCGGATGCAGATGCTGCTCTCTGGGAAGAGGTCAAAGAAGCGCTGAGAGGGCCGAACGCAGAGCAATCTTTGCGTTCGGCCCTCTCAGCGCTTCTTTGACCTCTT

>MG484405

CGCGAGGGTGCTGCGCACGGCATGGATGGCATGGTTTTGCCAGTGGTTTAGCGCTTGCTACTGCTGCTTCTGCCTGATCGAGGGTTGTGTCTTATGAATGTGTTACTTTGCAGCAGAGTTCGTAAAATACCTGGACGCAACTCTTTAAAATTTCAGCAATGGCTACACTGGCCGCATTTTTGAAAAACTTTCGGGCTTTGAGGCGCCTTGAGCCTGCAATGGCTCGTTGTGTTCGGCTTCCGCCAATGGATACCTTGAAAAGTTTGGAAGAGAAGATGCAAGCTCTAAAAGCAAAAGAGCCAGTGTTTGAAGAACGAGTCAATATTGGTTTCAAGGTCGCACCTCACAGTGTCAATAAACAAGAATGGAAAAGACAACGAAAGATAATCCAAGAGAACAAGGACAATTATGACCTTGCTGAGAAGTCACGTAATGGAACCTTGAAGATACAATGTGAAAAAGCCAGGCAAGAATGGAGAAAAACCTATGCACCAATGCACGTGAAAGAAGTGGCAGACCACTACGGTGTCTTCAGAGACCTTTATGAGTTTGGATTCTTTCACCCTGTTATTCCAATGGATGTTTTGTACGAGTATGATGCAGAGTATTTCACACCTGTTCATTATGGTAACATAATTCTTCCTTCTGAGGCTGCAAAAGCACCAACAGTNNNNNNNNNNNNNNNNAGGCTGCAAAAGCACCAACAGTTTTATTTGATTCCGAGCCTAACATGCTTTGGACACTTGTCTTGACAAGCTTGGATTCTCATCTCCTTGAAAATGACAAAGAGTATCTTCATTGGTTCATTGGCAACATAAAAGGAAACCAGGTTCCAAGTGGTGAAGTGGTGTGCGACTACATGCAGCCTTTTCTTCCTCGTGGTACTGGCTATCATCGGTTTGTGTTTGTTTTGTACAAACAAGAAGGATTAATTGACTACTCTAACCAGAAATTGTCTGCAAACAGTACCAGCCTGAAGGAGAGGACATTTAAAACCTATGACTTCTACAAGGAATTCGAAAATGTGCTCACACCTGCTGGCCTAGCATTCTTTCAGTGCACCTGGGAGGACAGCCTGGTGGACTTCTTTCACAAAACTTTGAAAATGCGAGTCCCCACTTTTGAGTATGTCCACCCTCCAGAATACGTCCCAAAGCAAGTTCTGTACCCTCACAAGCAGCCTTTTAACACGTACCTTGACATGTACCGGGACCCAAGGGACATCCGTGAAGAAGTTTACCTTAAGCGCCTGAAATCTCTCAATCCTTTGGAAGATGAGCCACCCATGCCAAAGTACCCCAATATATACAAGATTCCACAAGGCACACCAAGCTGGCTGGTGGATGAGATGAGGAAGGAACGCTTTCGCATTGGAAAATACAAGGACCTGAGGCCTTTCAGCATTTACCCAGAGGAGGAGTATGAGGACCCCAAAGTAAAAGCGTGGAGGGAGCAGAAAGAGTTTGAGCGCAAGTGGGCTGAGCAAAAGGCTGCCAAATCATCGCCATCATGAGTGTGTCTGTATATGTGCAGTTTTCATTTTCATTCATGCATAGCTATTTAAAAGAGTACAGAGAAATAAAAAACTTGACAAAAA

>MG4834191

AAGAACCCGGTGTTCCGCAGCTGGATGCACTGGATGGTGTTGAACATCAACAGCACGGAGAAGCTCCAGGAAGGGGACGCCGCGTTGCCTTACAACGGGCCCGCTCCACCCCAAGGATCTGGACTGCACCGATACGTGTTCCTGGTGTACTGTCAGCGCGGAATGCGCTTGCAAGCCAAAGATCTGGCGCCAAAGGAAAGGAAGAACTTCAACCTGGCCGAATTCGTCAACAGGACGAGCCTGGGCACGCCGTTGGCTGGAAACTTCTTCGTCGCTGAGAACCCCGCAGCTGTGTAGTTTAGTCTTGACGCTTCACAGCCTCTTTGCTGAAAGTGCCGCAGCGGTAAGGACGGACCGTCGATGATGGAGTAGACCGCGGTCGCCTAGGAAACCAGTAAGGAAGCTCCGTCCCGTTCTAGAAATTTGTCAAGACGCCTTTAGCTGTCAGTCCTCTGTGACACGTTCACCTTAAGCCCAGCCGAATGATATGTGCTTCGTCAGTGTCCAATCCCTCGAGAAATGCCTGCGCTTCGCCACGCATCGTTCCTTGCAACAGAACTGTCGCGATTGGCAAGTGAGTGTCGTCG
