# Supplementary material for: A repertoire of protease inhibitor families in Amblyomma americanum and other tick species: inter-species comparative analyses
Source: Parasit Vectors. 2017 Mar 22;10:152. doi: 10.1186/s13071-017-2080-1 (PMC5361777; doi:10.1186/s13071-017-2080-1)
Supplement: Supplementary file 3 — FASTA sequences for Amblyomma americanum contigs from Illumina sequencing, by PI family. (ZIP 638 kb) [file 13071_2017_2080_MOESM3_ESM.zip › A. americanum I63.docx]

>MG12017411

AGAATCTCGAGACCATGGTGACAAGTACTTCTCCATTGATCGTGAGACTGGAGACATCTACACCAAGGTTGAGTTTGACAGAGAAGAGAAGATGGCCTACGCAATCCTGGTTCGGGCAGAGGATGGTGCTCCATCGGCACGGCCCCACATGACAGACAACCGGCCCAACTCAGTGACCAAGTACATCCGCATTGGGATCGGCGACAAGAACGACAACCCGCCGTACTTCGGCCAGGCGCTCTACGAGGCTGAGGTCAACGAAGACGAGGATGTGCAGCACACTGTCATCACTGTCACCGCCAAGGACAAGGACGAATCCTCGAGGATACGCTACGAGATCACCCAGGGGAACATAGGAGGAGCGTTTGCAGTGAAAAACGAGACTGGCGCCATCTACGTTGCCGGCCCGCTCGACTACGAGACCAGGAAAGAGTTTCGACTGCGGCTGGTGGCATCGGATAACCTCAACGAGAACCATACAACAGTACTCATCAAGGTCAAGGACGTCAATGACAATGCCCCCATCTTTGACCGGCCTACCTATGAGGCTCAGATCACTGAGGAGAACGACCGGAACCTTCCCCAGAAGATACTTCAGGTGACAGCAAC

>MG12017842

ACTCGAAGAGTGTAGTTCTGACGTGCTTCCCTATCCAGCGGTCTTTGTACGAACACATCACCACTACTGCGCACCGCAAACTGCTGACCAGGATCACCGGAGAGCACGTAATAGCCAACAGGAGCCGGCTCCTTGTCAGCATCTTCCACGCGCAGGCTGGCAACTACAGTGCCAGCAACAGCATCTTCAGAGACTTGCACTTGGTATAGGGTCTGCTCGAACACGGGAGGATTGTCATTTACATCCAGGACCTCGACGTACACCCAAGCTGAGGAAGACAGCAGACCGTCATCACGGGCCCTCACTGTAAAGTTGTAAAATGGCACGGCTTCTCGATCGAGAGCTACCAGAGTTGTAATAACCCCACTCTGCGGGTCAATCCGGAAGATGTCAGCAGCCGGATCAGTCTCTTCGTAAAATTCGTACGTCACCCTTCG

>MG12019823

CTCGGAATTAGAGGTTACCTCCGTTCCTGCAGCCTGCGAATCTGCAGGGGGAATGTCAAGCCACTCCTTGCAGCGATGCAGGCAGGAGAGCACATCAGGAGACTCTGTACGTCCCCTCAGCACAGACAGGCCCGTCAGGTAGCCACGGAAGTGGAATGCCATCTTGTTGTCCTTGCCTTGCCAGCAGGCTCCTACAACAAAGGTGGTGTTGATGTTCTTGGTCTGGTGAAGAGGCCAGTCATCTACAATTTCCGGGTTGTTAGCCGTCACCTTAAAGGGCCGTCCATCGACATAGAGTGATGCCTCGGGGAAGTTGACAGAGACTGCGTAGTGGTGCCACCTGTCGTCGCACACCTCGGGTGTCTTCCAGCGCCACTCAGCTGGTGTGAATTTGTTGGCCTGCTCCTGGGTGGGCTCCCGGCGGAGCAGCAGGATAAGCCGGCAGTTGCGAATGAAGAGCGCTGTGTGATGCCGGCTCATGCGGTGGTCATCAGAGCTGCACAGGACATGCTCCTTGAGGTGGCTGGCGTTGTGGGCTGTGGGTGCGCGGTGACGCATCCAGAAGCCCACAGTGAAAACGTTGGTTAAGTTGTGCGACACAGTAGACTCGGGAATGACTACAGCATTTGTGGCACCATCAAACTCGTAGATCTGGTCACTCTCACGACCTTCATCCGTTGGCAGACCTTGCGTCCACTCGGCCCCGACGCCGGGGCTTGGAAGCAGGTCAACACTGTCTGAGCTGGCCCCGCAGAGCTTCCTTTGAGAATCGACTGAGTAGGTGTCCCGGTCACAACCCTTCCCCACGTGGCGGGTGGCCAGAGTCAGACGAGCCGACAGCTGTTCGGGTTCACAGGCTCCTTCGCACAGCTCCAGCTGGGCATCCGGGAACAGGGCCCGGCGACCAGAGCCAGGTGTGTACTCCACATGCTCCTCCATACCTTTCCATCCAACCTTGCAGACCCTGTTGACCTTGATAGTGACAGTGACCGGATGACTCTGTTTCATGCTACAGTCGAAGGCCACCACCTGGAATATGTGGTTGCTGCTTGCCTCCCAATCCAGGGGCTCCGTGGTCCAGATGGTGCCTTCAGAGTCTATGGAGAAGGGCACATGTGCGTCCAGGATGTCATACTTGCAGATCTCACTGTTCTTGGGCGTGCAGTCAGCATCAAGAGCACGCACGCGGAGCACACGCTCGTGTGGCGGCCGGCCCTCATCCACGCTGCCTTGGTAAGACTCCTCCTCCCAGCGGGGCGCGAACTCATTGACGTCATCCACTGTCAGGTGCACAGTAACGTTTTCGGAGACGAGCCCGTTGCATCCCACGGCGGCGATGTCGAACTTGTAGTTTCGGTGCTTCTCGCAGTTGAGCTCCTTCTTGGCGAACAGCTCGGCCTTGCCCGTCGCCTCGTCCGTGATGCGCACCTCGAAGGGCGCCTCACCGTGACGCTTGTTGGTCACCAAGAAGCGGCACACCTTGGCGTCCAGCACCCGGATCCTGGGCATGATCTCCACATTGCGGCTGTTTTCCTTGACGATGGCATGGTAGCCGACCTCTGTGTTCACGTGCTCCAGGCGAGGTGCTTTGAGCGCGGAGGCGAGTCCGACGAAGAAAAGCGCGAGCACCGGGGCACACTTCATGATCGCATCCAGCTGTAGTGGACGACTGTTTGGATCACAAGCAGTGGCTCACCATAGGCGCACGCACAAAGTCAGAGAAACGCTTCGGGAAGCGGGGGCACGCCGTGTAAAATTCCCGTGCCAAGAAGTCTCGGGCGTTTCCGCTCGCCGAAGGGACGACTGCGCGCGCTCCG

>MG12023274

CTGCAAATCTTCATAATCTAAAGGCTTGGCAATTTTTAAAGAACCAGTTCCATCAGAATTGGTGACCATAGTGAATTTATCAGAGCCAAACGTGTTGTCAATAACTTTGTAGCTAAACCGGTTAGTTTCTAGTAGATCTCCATCATTGACTGATACCACTAAGATGGGGTTTTCAGGAATATTGTCTGCCTCAGTTTCTTCAACATCAACAAACCAATCCTTTTTGGTAAACTCTGGAGGCATGTCATTGATATCCTTGATCTTGATTGTGGCGGTTCCAGTTCCTTTTAAACCTCCACCGTCGACAGCCACCACTTTGATGGTGTACTCTGGGTTTGTCTCACGGTCTAAACAGCAAACAGCTGTCGAAATGACGCCAGTTTCTTCATCAATGGTGAAAATCAGCTCTCCATTTTCGTTAACCTGGTTCTGCTCAATAGAGTAGGCGAGTCGTGCGTTGTTGCCCTCATTGGGATCGTCATAGTCAGTGGCTGTCATTGTCATCACAGTCATACCAGCTGTTCCATTTTCTGTGACATTGCCTGTGTAAATGGCTGAAGGGAAGAAGGGGGCATTATCATTGACGTCCTTGAGGTTGACGAGAACGTCCGCAAAGCCCACCAAACCATTGCCCCCTTCGTCCTCCGCAAAAAC

>MG12031119

CGTACGCGTTTATTAATTGTCTCCGCTCTTGTCGTTATATGCCACGAATGGGTCACTCAACTAGCCGCCCTTTTTTTGGGGGGCGTTACTCTTGCAGTGGCCAAGAAGGGCAAAAAGTCTGAGGAAGAGTCGGCGCATGTCGCTTTCGACGAGGCCCTCGTCGTGGTGAACGTGGGGGACGAAAACGACAACTCGCCCGTCTTCGACCACCAGGGCAAGCCCATCGTGGCCGCCGTGCCTCTCGAGGCCTCTTTCGGCTACCAGGTCACCAAGCTACACGCCCGCGATGCTGACACGGGCTTCAACGGCGCCATCCGCTACGAGATCATCCAGAAGCCCGAAGACGCGTCGTCAAAGTTCCAGATCGACCCGGTGACCGGCGTGGTGCGCTCCGTCGTCACCTTCTCCCTGGACGGCGGCAAGATGTATGGATTCGACGTCAAGGC

>MG12037347

CAGCACGAGATGATGGTGCCTGCTGTCGAAATGGGGCTCTGACTCCCCACACAAGCACAGCCCTAGTGGTTGTGTTCATAACGGATGTCAATGACAACAAGCCCGTATTTGAGGAATGCCAGACCTATACGCCTAAGGTAGAAGAGGGTGCTCAAAGTGGCACCTCAGTCATCAAGGTGAAAGCAAGAGATCTTGACAAAGGACACAATGGTCAAGTGCGCTACTCCATCGTCCAGCAGCCTAATCAAAAGGGCACAAAGTTCAGTGTCGATGAACTCACCGGAGAAATCAGGACAAACAAGGTTTTTGACCGTGAAGGGGATGATGGTCGTTTTGTGAGTGTCACTGTAAAAGCAACTGACCGTGGCAGCCCACCACTGGAAGGAGTCTGTTCCTTCAAAGTAGAAATAACTGATATCAATGACAACCCACCCCTATTTGACAGGCAGGAGTATAGGGAAAATGTGAAGCAAGACACCCAGGTTGGGATTCATATCTTGAGGGTGTCTGCTTCTGATGAAGACGCAGATAACAATGGTGCCATTGTCTACAATTTGACTGCACCATATGACCCTGAGCACTTGGCATATTTCTCCATCAATCCAGACTCCGGGTGGATTAGCCTGCAAAAGGCCCTTGACCGTGACCAGTATCAGCTGCGAGCCATAGCGCTGGACAAAGGTGTTCCCCAGCATCAGGCAACGGTGGAAGTGATCATTGATGTTGTTGACAGAGCCAATAATCCACCGATATGGG

>MG12038124

TGGTGTGAGGGCTACCAGCCGAGATGCAGGCGTCAATGCTCAGATCACATACTCCCTGGTTGCAGGCAACGACATGGGACACTTCAGCATTGAGCCAAAGACAGGTGTGCTGCGGGTGGCCCGACCCTTGGACTTTGAGAGCGCGCGCAGCTACCAACTGGCAGTGGAGGCTCGCGATGGGGGCGAGCCACCCCTCAGTGCCCGCGCCTGGCTGAATGTGTCCATACTGGACGCAAATGACAACGCGCCTGTGTTCGGAGGCCCCTACAGTGGCACTGTCGCGGAGGATGCAACCCCTGGGCAGCTTGTGCTCCAGGTGCAGGCCAGTGATGCCGACAGTGGCGGAGAGCTGCGATATGCTCTGCTGCAGCCGGGCCCTTTTACTCTGGACCCGGCCACTGGCCAGCTAAGTGTGGCTGGGCCGCTGGACCATGAGACGGTGAGCCGGTACGCACTGGAGGTGGAGTGCTGGGATGGTGGCACACCGCCACTGTCGGCTCGGGCACTGGTGCACCTGGAGGTGCTGGACGTGAATGACCATCCACCGCGCTTTGACCAGAGCAACTACACGGCCGTGGTTCATGAGGGTCGGCCAGTCGGCTGGACCGTG

>MG12039237

CGTCAATGACAATGAGCCCACTTTTGGTGCTTCTGAATATGAGGCTAGCATCTGGACAAACACTAGCGTGGGCACTACACTTGTCAAGGTTCGTGCTTTTGACCGTGACCAAGGAACATGGCCTCGTTACAGCCTTCATGAAGCCCCAACCAATGTCAGTGCATTGTTCAATGTCACTCATGACTCGGGAGAGCTTTACCTGAAGGCGCCCCTCAGAGGAGCTGGCGTGTTCCAGTTCTTCGTGCACGTCAGTGACCAGGGTAATCCTAGACCTCTCTGGGCTGTGGTGCCTGTTACGG

>MG12041709

CTATCAAAGCCAGATCCGGAATCCCACAGAATCCTGATGTGTTCTACACGTTGATGAAAGGAAGTACAGAGCAGACCAACAAGAAGGACACTTTCTACTTGAATCAAAAGCTTGAGAATCGCCAAACAGTGGCAGAGCTGGTGGTCAATTATCCTCTGGACTATGAACGTATCCAGCAGTACAACCTTACTGTTCGTGTCGAGAACAACGGCATCCAGCAGTTGGCATCAGAGGCAACTGTCTACATAGTCCTTGAGGACGTCAATGATGAG

>MG12042944

CAGGGTGGCCAGCATCCGATGCCACCACCTGTAGCCGGTGCAGGGCTTGTGTCTCCCGATCAAGAGGGGCCCGAAGCCGCAGGACACCGGAACCTTCCAGGTGGAAAGTGGCACTGTCGTCGTCAGCCAGTCTGAGCTCCAGCATTGGTCCATCACGGTCACTGGCGCGCAACTCCAGAAGCACGCTGGCCACAGGCGTGGATTCTGACACGCTCACATTGTACCAGGGCTGCTCAAAGATGGGGGCACAGTCATTGATGTCCTCCACGTTGATGGTGACGGGCACATCAGAGTGTGCACCAGTCACGGAGTCTGTGGCTCGGACCATCAGCTCATGCCGTG

>MG12043607

GACAGTACCAGTCAACGCGTGAGGAGCGCCTCTCAGTGGTTGGTGGCAAGCGCCAGCCTCAGTTTTATGTGCCCTTTTATGAAGCGGTCATTCCAGAGAACCAGAAGAAAGACTCCGATATCATCGAGGTGAAGGCCAAATCTTTTGCCGATCGGGAGATCCGGTACACTCTGCGGGCCCAGGGCAAGGGAGCTGGCACCTTCAACATCGGACCTACCAGCGGAGTAGTGAAGTTGGCCAAAGAGCTGGACTATGAAGACCTACGGCAGCCAAAATCCTACTCTCTTATTGTCACTGCAACTGAAGACTCCGGTGGCTTTTCAACCTCTGTTGAGCTAACAATTAAAGTAACTGATGTGAACGACAATGCGCCTCGATTTGAGCTTCCAGACTATCAAGCCCATAATGTTGATGAGGACATAGCAGTGGGAACATCCATCCTTCAAGTGTCTGCAACGGATATGGACACTGGCCGCAATGCTGAGCTTACTTACTCGCTTGATAAAGAAGATTTTACCATTGACAGCCGGGGTGTGGTGTACTCCAATCGGCG

>MG12047897

CGGAACAGCCTGCGGGCCCAGGCCTCTACAATGGAAAATGACAGCTGTGCATTGAGGCCGGTGTCTTCATCCTCAGCCACCACCACCAGAGGCTGGCTGTACCTGAGAACTGCACTACCCGCGGGGGCCGCTTCACTCACNNNNCCATGATACAGTAGCTGCCGGAAGCGAGGCGCATGGTCATTACTGTCGAGCACACGTACTGTCACATGACCCTGGGCCATGGCACCGGCCAGATTGATGGCACGCACTGTCAGGTTATGCACAACCGCCTGCTCATAGTCTAGGACTTCATCGCCTGTGGAAAGCACGCCTGTCACTGGATCCAACCGGAAGTGGCTGCCAGCCCCTTGCTGCAGC

>MG9613779

TGGTGCTCTGGCCAAACACAGGCGGGTTATCATTGACATCACTCAGCAAGAGCTCCACGCTGCTGTTGCATTACCACTCCCAACGAGCCACGTCACGCACATGTGCCACCAGTGAGTAGTGCGGCCGCTGCTCTCGGTCTAGAGGCCGGGTCGTGTGCAGCACCCCACTGCTGGCATCCAGAACAAAGTCCTCAGATCCTTGGCCTGACAGGTAGAAGTGCAGCTGAGCATGGTGGGCATCGTCCGCATCCGTGGCTCCAACC

>MG9621172

TTTAAAAGGAACTGGAACCGCCACAATCAAGATCAAGGATATCAATGACATGCCTCCAGAGTTTACCAAAAAGGATTGGTTTGTTGATGTTGAAGAAACTGAGGCAGACAATATTCCTGAAAACCCCATCTTAGTGGTATCAGTCAATGATGGAGATCTACTAGAAACTAACCGGTTTAGCTACAAAGTTATTGACAACACGTTTGGCTCTGATAAATTCACTATGGTCACCAATTCTGATGGAACTGGTTCTTTAAAAATTGCCAAGCCTTTAGATTATGAAGATTTGCAGCAGCGTTTTGGGTTCAACATCACCATTCAAGTGAGCGACAATGGTGGAGAATCAACAGATCCCTATCATGCGGACTACGCAAAGGTCAAAGTTAGGGTTAAAGACATAAATGACAACAAACCTGAATTTGAGAGACCCAACATTGAAGTGTCTGTTCCTGAGAATTCCACCGTTGGTTCAAGTTTGGCAACATTTAAGGCCACTGATGCCGACCAAGGTGGAAAGTCACGTGTCAGCTACATGATTGACCGATCATCTGATAAAAAGAGGCAGTTCAAGATTAATCCCAATGGTGTTGTTGAAATTCAGCGTACACTGGACAGAGAAGACATCCCAAGGCACCAAGTCAAGATCTTGGCTATTGACGATGGTGTACCATCACGGACAGCTACTGCAACGCTTACTGTTGTTGTTAGTGACATCAATGACAACCCACCAAGGTTCCAGTACGACTACAGGCCAGTCATACCGGAGCACACCCCTCCACAAAAAGTACAAGAGATTCTGGCCACAGATGATGACGATCGTTCCAAAAACAATGGTCCTCCGTTTACATTCCGCATGGATCCTAATGCACCGGAGCTTATTCAGCAGTTCTTCCGTGTGGAACATGACCCCACTGGTGCCAATGGCGACGGTATGGCTGTGGTGCGCTCTCGAGAAACTTTCGACAGAGAAACTCAGAAGGAATACTTGGTTCCCATCCTTATCAAAGACAACGGCAACCCATCTCTCACGGGGACTTCGACACTCACTGTCATTATCGGGGATGTGAATGACAACCGCATGCACCCTGGCAGCAAGAGCA

>MG9623999

CTCCGTGTCGCCGTGTGCGGAGCGCGCGCAGTCGTCCCTTCGGCGAGCGGAAACGCCCGAGACTTCTTGGCACGGGAATTTTACACGGCGTGCCCCCGCTGCCCGAAGCGTTTCTCTGACTTTGTGCGTGCGCCTATGGTGAGCCACTGCTTGTGATCCAAACAGTCGTCCACTACAGCTGGATGCGATCATGAAGTGTGCCCCGGTGCTCGCGCTTTTCTTCGTCGGACTCGCCTCCGCGCTCAAAGCCCCCCGACTGGAGCACGTGAACACAGAGGTCGGCTACCATGCCATCGTCAAGGAAAACAGCCGCAATGTGGAGATCATGCCCAGGATCCGGGTGCTGGACGCCAAGGTGTGCCGCTTCTTGGTGACCAACAAGCGTCACGGTGAGGCGCCCTTCGAGGTGCGCATCACGGACGAGGCGACGGGCAAGGCCGAGCTGTTCGCCAAGAAGGAGCTCAACTGCGAGAAGCACCGAAACTACAAGTTCGACATCGCCGCCGTGGGATGCAACGGGCTCGTCTCCGAAAACGTTACTGTGCACCTGACAGTGGATGACGTCAATGAGTTCGCGCCCCGCTGGGAGGAGGAGTCTTACCAAGGCAGCGTGGATGAGGGCCGGCCGCCACACGAGCGTGTGCTCCGCGTGCGTGCTCTTGATGCTGACTGCACGCCCAAGAACAGTGAGATCTGCAAGTATGATATCCTGGACGCACATGTGCCCTTCTCCATAGACTCTGAAGGCACCATCTGGACCACAGAGCCCCTGGATTGGGAGGCAAGCAGCAACCACATATTCCAGGTGGTGGCCTTCGACTGTAGCATGAAACAGAGTCATCCGGTCACTGTCACTATCAAGGTCAACAGGGTCTGCAAGGTTGGATGGAAAGGTATGGAGGAGCATGTGGAATACACACCAGGCTCCGGTCGCCGGGCCCTGTTCCCGGATGCCCAGCTGGAGCTGTGCGAAGGAGCCTGTGAACCCGAACAGCTGTCGGCTCGTCTGACTCTGGCCACCCGCCACGTGGGGAAGGGTTGTGACCGGGACACCTACTCAGTCGATTCTCAAAGGAAGCTCTGCGGGGCCAGCTCAGACAGTGTTGACCTGCTTCCAAGCCCCGGAGTCGGGGCCGAGTGGACACAAGGTCTGCCAACGGATGAAGGTCGTGAGAGTGACCAGATCTACGAATTTGATGGTGCCACAAATGCTGTAGTCATTCCCGAGTCTACTGTGTCGCACAACTTAACCAACGTTTTCACTGTGGGCTTCTGGATGCGTCACCGCGCACCCACAGCCCACAACGCCAGCCACCTCAAGGAGCATGTCCTGTGCAGCTCTGATGACCACCGCATGAGCCGGCATCACACAGCGCTCTTCATTCGCAACTGCCGGCTTATCCTGCTGCTCCGCCGGGAGCCCACCCAGGAGCAGGCCAACAAATTCACACCAGCTGAGTGGCGCTGGAAGACACCCGAGGTGTGCGACGACAGGTGGCACCACTACGCAGTCTCTGTCAACTTCCCCGAGGCATCACTCTATGTCGATGGACGGCCCTTTAAGGTGACGGCTAACAACCCGGAAATTGTAGATGACTGGCCTCTTCACCAGACCAAGAACATCAACACCACCTTTGTTGTAGGAGCCTGCTGGCAAGGCAAGGACAACAAGATGGCATTCCACTTCCGTGGCTAC

>MG9627106

GCCAGCCTCAGTTTTATGTGCCCTTTTATGAAGCGGTCATTCCAGAGAACCAGAAGAAAGACTCCGATATCATCGAGGTGAAGGCCAAATCTTTTGCCGATCGGGAGATCCGGTACACTCTGCGGGCCCAGGGCAAGGGAGCTGGCACCTTCAACATCGGACCTACCAGCGGAGTAGTGAAGTTGGCCAAAGAGCTGGACTATGAAGACCTACGGCAGCCAAAATCCTACTCGCTTATTGTCACTGCAACTGAAGACTCCGGTGGCTTTTCAACCTCTGTTGAGCTAACAATTAAAGTAACTGATGTGAACGACAATGCGCCTCGATTTGAGCTTCCAGACTATCAAGCCCATAATGTTGATGAGGACATAGCAGTGGGAACATCCATCCTTCAAGTGTCTGCAACGGATATGGACACTGGCCGCAATGCTGAGCTTACTTACTCGCTTGATAAAGAAGATTTTACCATTGACAGCCGGGGTGTGGTGTACTCCAATCGGCGGCTTGATGCTGATGTGAACAACACGTATGTTCTGACAGTGCGGGCTACAGATCGTGGGGAGCCGCCACTGACAGGCACGGCCACAATTCGCATATACACGGG

>MG9629606

CTGCACTGTTGGTGACACTAACTTCCAAAGAGTAATTAGCCCTGGCTTCATGGTCAAGCCAGTGGGCTATACTGAGCCTTCCAGTGGCTGGGTTCAGTGCAAAGTGACCATCTTCATTGCCTGACTCAATATGGTAAACAAGGGGCACTGATCCAGCCACACCAGCCTTGACCTGTACNNNNNNNGTGCCTGGTGGGGCTTCCTCAGATATCTCTGCCTCATAGTGAGAGCGCTCCAACTGAGGGACCACATTATTCAGCTCAGAAAGATTGATGGAAACCTTTTCATCAGTGAACTGAGAAGGC

>MG9631642

GAAAACAAGAACGATGAGGCGCCGAAGTTCAGCCAGGACGTTTACACGCCAAACGTGGATGAAAATGCTGGGCCCAGCACTCTCGTGACCACTGTGGTAGACAGCGACAAGGATGGAGATAACATCCTCTTTGGATTTGTCAACAGTGGCACCACCTCTGGCATGTTCCAGATTGAAGAAAGGACAGGTGTCATCCGACTCATCAATGGCCCCATCCACCTGGACAAGGACAAGTACGAGTTGAATGTGACAGCACGAGATGATGGTGCCTGCTGTCGAAATGGGGCTCTG

>MG9637108

AACAAGAGCAAACGCCAGGCGGCCGCCACCATCAGTGGCAGAGATGGGAACCTCGTAAAGATGTTGATGTTCGCGGTCCAAAGCTTCCCGAGTCACCAGCTCTCCTGTGGTGCTGTTGATGTTGAAGTGCAGTGCACAGTCTTGACTGCGGATGGCATATGTCAGCGTGCCCAGCGCCCCCTGGTCGGGATCCGTGGCAGACACGCGGGTCACATGGGCACCTGGCGGCAAGTTCTC

>MG9639702

CCCACCAAACCATTGCCCCCTTCGTCCTCCGCAAAAACGGTGAATCGCCACTGGGAACGTCCATGTGGAAGGTCTCTGTCTAATGGCTTTAACACATATATTTCTCCTGTAGTTGTGTTGATTGCAAACTTGCTATTCCCAGGGTCTTCATCAACACCTTGACCCGTCAAAAAGTAAACTATCAAGGACTCCCTGTCCTTATCACCATCTGTTGCTGTCACCTGAAGTATCTTCTGGGGAAGGTTCCGGTCGTTC

>MG9644906

GCGGAGAACATCAGTTCTGGTGGTGATCAAAATCCAGGATGAGAATGACAATGCTCCAGAGTTCACCCAGTCGGGCTACACGGCTGTTGTGCCAGAAAACGTTCCGACAGGCTTCAGTATTCTGACTGTGAGGNNNNNNNNNNNNNNNNNNNNNNNNNNNNCGGAAATCAGCTACTCTTTTGTGGATGAACCTGAAATGGGAGCCATCACACTTTTTGCAATCAACGACAAGACTGGCGTCATTACGGTGATTCAACCACTGTCTGGGAGGGGGCG

>MG9646973

TCCGGATTGACCCGCAGAGTGGGGTTATTACAACTCTGGTAGCTCTCGATCGAGAAGCCGTGCCATTTTACAACTTTACAGTGAGGGCCCGTGATGACGGTCTGCTGTCTTCCTCAGCTTGGGTGTACGTCGAGGTCCTGGATGTAAATGACAATCCTCCCGTGTTCGAGCAGACCCTATACCAAGTGCAAGTCTCTGAAGATGCTGTTGCTGGCACTGTAGTTGCCAGCCTGCGCGTGGAAGATGCTGACAAGGAGCCGGCTCCTGTTGGC

>MG9647110

AAGGACACTTTCTACTTGAATCAAAAGCTTGAGAATCGCCAAACAGTGGCAGAGCTGGTGGTCAATTATCCTCTGGACTATGAACGTATCCAGCAGTACAACCTTACTGTTCGTGTCGAGAACAACGGCATCCAGCAGTTGGCATCAGAGGCAACTGTCTACATAGTCCTTGAGGACGTCAATGATGAGATCCCTCTTTTCATAGAGCGGGAGCAAGAAACAGTCTTGGAAGGTTTACCACCAGGCACCAAAGTGACTCAGGTCCAGGCTGTTGACAAAGACGGCACTTACCCGAATAACAAGGTTTATTATGCCATAGAATCTCGAGACCATGGTGACAAGGACTTCTCCATTGATCGTGAGACTGGAGACATCTACAC

>MG488469

ACCGACCCTTGGATTTTGAGAGCGCGCGCAGCTACCAACTGGCAGTGGAGGCTCGCGATGGGGGCGAGCCACCCCTCAGTGCCCGCGCCTGGCTGAATGTGTCCGTACTGGACGCAAATGACAACGCGCCTGTGTTCGGAGGCCCCTACAGTGGCACTGTCGCGGAGGATGCAACCCCTGGGCAGCTTGTGCTCCAGGTGCAGGCCAGTGATGCCGACAGTGGCGGAGAGCTGCGATATGCTCTGCTGCAGCCGGGCCCTTTTACTCTGGACCCGGCCACTGGCCAGCTAAGTGTGGCTGGGCCGCTGGACCATGAGACGGTGAGCCGGTACGCACTGGAGGTGGAGTGCTGGGATGGTGGCACACCGCCACTGTCGGCTCGGGCACTGGTGCACCTGGAGGTGCTGGACGTGAATGACCATCCACCGCGCTTTGACCAGAGCAACTACACGGCCGTGGTTCATGAGGGTCGGCCAGTCGGCTGGACCGTGCTGCGCTTCTCGCTCAGTGATGCTGACTCTGCTGCTCATGGACCTCCTTTCCGGCTGGAGCTGCTGGATGGAGCTCAAGCTGGGCAGGAGGAGGAGGAACCCTTTTTCCAAGTTGAGGGCCACGAGCTGCGGTTAGCACGACCACTTCCTCCGCGCTCGCGGCATGAGCTGCGTGTGCAGGCTCACGACTCGGGCAGCCCACCTCTGGCGACAGAGGCGCATGTGACTGTGCTGGCTGTTGAGAAGAGCCGCTTCAGGCCACAGGTGCAGCCCTTGACTGTGGTTGTCTCTTCTTACTTGGACGACTTCCCGGGTGGGCTGCTAGGCCGTGTGCATGCCACGGATGAGGACCCGTACGACCGCCTCAGCTTGTCCCTCTCTGGGCCACACGCATCGCTGTTTGTCCTCGACAGGGATGATGGGACTTTGCGTGCTTTGCCAGGCCTTGATGCTGGCAGCTATGTCCTTAATGTGAGCGCTTCAGATGGCAGCACAACGCCAGCACATGCATCAGTGAATGTGCATGTTGTCGGGGTGACCGAGGAGGCTCTCAAGGCAGCTGTGGCTCTGCGCTTGGCTGGAACCACGACTGAGCGCTTCTTATCTACGGACCGGCGGCCATTGCTGCGAGCTTTGCGAACAGCTCTCAGCGTGCGTTTGCGGGACCTGTTGCTGGTGAGCATACAGCCAGCACCAGCGGAGGCCTCAGGAGGGAGCGAGCAGCTGGACTTGTTGTTGGCAGTGCAGCCTGAAGGAGATCCAGTGCCAGCTAGCACAGTGGCTTCCCGGCTTCACGAGCGCCATGTAGCCCTCGAGGCAGCTACTGGTCTACGTGTTCACATATTGCCCCAGGGGGACCGCTGCCCTTCCCTGCACTGTGTGCACGGCGAGTGTCAGGACCGGCTGGTGCTGGACACCAGCGACGCTGTTGTCCTTGCTGGTGGTGGCCACAGTTTCGTATCACCACGACACAGCCGCCGGACGGCGTGTGTCTGCAACCCTGGTTTTGGCGGTGATGCATGCGAAACAGCAGTGGATGAATGTGCCCAGCAGCCCTGCCCTGCAGGGCGCCTCTGCGTGCCAGACGCGTCTCCACTCGGCCACAGTTGCCAATGTCCATCTGGTCGCACTGGTTCTGAATGCGACACCCCTTGCCAGGAACCCAGCTGCTATGAAGAAAAGCGGCCCATCTCGTTTGGTGGCCAGAGCTATGCGCTCTACGTGCTGTCACAGCCCTTGGACCGACGCCTCTCGTTCTCTGTGATGCTGCGCACAATCCATCCCAGTGGCACTCTGCTGCACACATCGGGACCCCGAGACTACGCCATCCTTGAGGTGTCGGACGGGCACGTGCAGTACCGCTTCGACTGTGGCAGCGGTGAGGGTTTGGTGCGTGTGACTGGCCGTCGGGTGGATGACGGTGTCTGGCATGCACTGCGTCTCGAGCGTCGTGGCAGCAATGCGCGGCTCGCTGTCGACGTGCATTACCAAGCTTCTGGTGCCGCGCCTGGGCCACACGATGTTCTCAACCTGGAGGGCCGTGAGCTGCACCTGGGCGGGGCACCTGCTGTGGCAGGCTTGGTCGGCTGTCTGGATGATGCGCAGGTGGGCGGCCAGCCACTACCGCTGCATCTGCGACCTGCTGGCCACGCGCAGCTGCGCCGGCTGGCCAACGTGCAGTTTTCTTGCCACCTCGACCCATGTGGCAGCCAGCCCTGCCTCAATGGTGCAACCTGCCGGCCGCTTCTCACCACCGCTGGATACTCGTGCACTTGCCCGGCGCACTTTCAGGGTCCCCAGTGCAGAGAGGCAGTAGCTGAGAGCCGATGCACTGACGAAGCATGTGAGCCAGCCAGCTGCCAGCCCAACTTGTGCCTCCATGGGGGCCTGTGCCAGCCAGGTGGACACTGCCACTGCCCAACTCCTTATCAGGGCAACCGATGTGAGCTGGTGGAGGCATGTACGGCGTGCGAGAACGGGGAGAGCTGTGTGCGGCTGCCAGATGGCTTCCAGTGTGGCTGTCTGGGGGATGCATCGTGTGATGCCACTTTCGAGGGCCTGCCCCTCGCTTGGCCTCTGGGAGGTGCTGCTGCTGCTGTGCTGCTTCTGCTCCTGCTTCTGGCCATTGTTTGCTGCTGCCGCCAATGCTGTCGTTGCCGCCACCGCCACGAACATGTTGACAGCCATCAGTGCAGCAATCCTGTCACTGCCAAGAACTGTGTCCTGGCCACCACAAACATTAGGCCAAAGATAAGCAACCTGGAGCAGCGGCCAGCTTCGTACACGACGACTGGCGTGAGAGAGATCACACTGAACAACTTTGACACGGTGCGCAGCTATGGAAGTGCAGCTGATGACCTTGAGTCACGCTTTCAGCCCAATGACCTGCGCTGCCACAATCTGGCTCGCAGTCCATCTGGTAGTGCCACAGGGCCCCCACAGGGAGGCCACAAAGGCCTGTACCTGGACAAGATCCCCAACGACCTGAAGGCAGCTCTGTCTCCGCCCTTGGCACCACCAAGCTCGGCAACCAGCATTGCATCAGATCTCCCAGGCTATTGCTGGGATTACTCGGACCTAGCAGCGCATGCAGAGGAAGACGTTTCGAGTGATGAAGCGCCTGGCAGCTCCCGAAGCTCACACTGCTCCGAAGGCACTCCTCTGCAGGGAGGTGAAGAACGGTACACCTGCCACCCCGATCAGTACCTGCCTCGGCACTGCCCCAGTGAACCAGCAGTGTGTGCCATTGAGGACAGTGATGAAGAGCTGGCCTGACATCTGTGCAAGATGCTGGTGCTAAAGCCAGCAGTGGTGCCATACAAGGAGTGCCAGCGTGATGGTTTCTCCTCGCTGGCCTGCATTCGTGACTGCGCCCGTGGGCAGAGAGCATGACCTCTCTGAGGGAATGCTGTGTTGTCTATCTAGGGACTGCTTGCCTATGACGTTGCCTGAAAACTGTGCCTTGTGTTACCGGCCTCGCAAGCTGTGTGCATTGTCCTCATGTTTAGAGTAAAATTGAACTTGCAGAAATAAAATACAAATGCCAAATC

>MG489189

CGGCACCTATAAGGGTGCAGAGTGCGTATGTGACCCTGGCTGGCATGGTCCAAACTGTGACAGAGAAACACAGTCCAAGATGTTCCAACAAAGCAGCTACATCAAGTATGCGCTGCCATTTGATCCACATCCTTACAAAACGGACATTCAGCTACAGTTCCGTACACGTCAGAAGCATGGAGAGCTTTTCCGTATCACGAGCAAGCATGGGCGGGAGTACTGCATTCTTGAGATTCGGGACAAAAAGCTGCGCTTCCGTTTCAATTTGGACCGAAGCCGAGCCTCTGAGGAGCGTGAACTGTGGCTTCCATGGCTGTGGGTGAATGACGGCCAGTGGCACACGGTGCGTGCCCTGCGGTATGGGGCCTCTGCTAGCCTGGCTTTGGATGGGGGCAGTGGGCGACGCTTCAACGAGCTGCTGGATTGGCACAGCCCCCACCAGCATATGTGGGTGGAGAAACAGAATGTCATTGCTGGTGGAGATGTCCAGTATGTTGGCCCAGGTGTGACAGTCGTTGACAACGACTACCAAGAAGGGTGCATGAATGACATCCGGCTTGACCAGCACTACTTGCCAATGGAAAGTGGTTCTGAATATGCGGCTGTAGTAGAATGGCGCAACCTTATTGATGGATGTCCATCCAATAATCCTTGCCATGGAATCAGTTGCCCACGCCCCTTCGTTTGTGTCGACCTTTGGATGCTCCATGAGTGCAGGTGCTCCCGTGGCTTTGCTGTAACGGAGAATGGCAAGAACTGCACGGATGCGGATGAGTGCCTGACGGAGCCATGCCTCAACGGAGGCACCTGCATGAACCGGCCACACGGCGAGGGCTTCTACTGCCTATGCCCAGATGGTTTTGGGGGCGACCTTTGTGGCGCCTTGCGACAGGAGAAGATAATGCGACTCAGTATGGCTGCCTTGGCTGCCATCCTGGTCTGTCTGCTCAACATCCTCATTTTGGTCCTGGTGATTGTGGCATACACCCGGAGCCGACGGTCAGACCAGAAGTTTGGCCATGGTGGAGTGGACGACGATGTGCGAGAGAACATCATCAGCTATGATGATGAAGGCGGCGGAGAAGATGACATGAATGCCTATGACATAACACCACTACGAATACCAGTTGATGCATCTGGCATGCAGATTGGCGCAAAGCCTGGCCCAGAAAAAGCCCCACCTTTGCAGAAAGAAGTTCGTGTCCGGGAACCACCCACAGGGGCCCACCCAGATGTGGGTGACTTCATTCGGGACCACCTGGACAAGGCAGACCAAGACCCTGGAGCTCCTCCAGTGGATGATGTGCGCAACTATGCCTATGAGGGTGGTGGCTCCACTGCAGGCTCACTCAGTTCCCTGGCCTCAGAGACGGATGACCATGAGCAGCGCTTCGACTACCTCAATGGCTGGGGGCCCCGCTTTCAGAAGCTGGCCGACATGTACGGACAGGGCGAGAGTGAGGAAGACTAGGCGCCATTTGCACATCTGCCTCCTTGTCCCCCATTTCATTGACCCTTTAGTCATTGATTTTGTTCACTTTGGCCTGCTCTTTTGTCCACTCACATTCGCCACTCACTCACACACTCAAGTCCCGCAGCTCACGATGGATAGGGGCACGTTGCTTTTTTTATGGGCAAGTGTTTGCACCAAGTGAAGATCAAAAACATCTGAGAAGCTTAGGCAGCTAAGGCAATGCTCATTCTTGATGTTGCAGGCTTACATCTTGGTACTTTGCTGTTGTTGCCACCTCTGCTTGATGAACACAGCAGCTGAACTAGTGGCACACTTCTGACTTCAGTAGATAATACAAATCTTGATGGTTGACTTAGCCAGCAAGACTGTACAGCCGACCGATGCTGTTTCTAGCACTCTAGGAATACTACTGCGATACGTACATAAATGCCATACTGACTTAATATTAGTGTGCCATCTGGATATTCTTCTGTGCAGGTTGGGTTTGTCTGTCCTGAACATATAAAGCTGCCCCCTACATCGTGGTCTGCGTTTGTTGACTTGAATGCCCAAATTTGCCTCGTGCCTTCCTTGAGGCTGGTCATTGAATCATCCCAAGTGTTTCCTGCATCATTTGTGTATAGTTTTGTTCGTTTTTAAAGCTGTTTCAACATTCATCATATTTACCAACATAAAACACAGTTCTTGTTTTCTGTGGTGTTTCTGTAGCAGCACGGTGCAATCCAAAGGGACAGTGTACAAGTGAGCTTGGTTGCTCCGGTCTTCCGCAACGTTTTCTTCTCCAGAAATGAGCAGTATTTTTTCTTCCATCAGCATTGTGAACTTTCTTTTCACTGTGTCCCTGATGCACATCAAAGTGGCCATATAGAGCAATCTTTACCACTCTCATTTGTTAATGATGAAATCATGCATGCTTAAGGTTGTGTTTTTTATTATGTTGCCTAGTGTCATCACAAACATGCACATGGCTTGAAGAAATTCCCACTGCCTTTGATTTATATCTTCATGACGTTAGTGTGTACTGTAATTGCTCTGTGGGTATGTTCTTGCCATATTAATGTTTACAACTGAAGGTCTTTTTCAAGCTGTGTGCTTGTTGCTGATGCCAGTTTTATGTCTTGTGATGGAGACAGTGTTTTTATCTGTCTTAAGGCTCGAGTGAATTTCAAAACTGCCCAAATTTTTGTGCAGATTTTTTTCTTTTGGTGAAATTGTTTGTTGTAATGTAGCTTGTGAAAAATATTGTGTAGTATAGTATGTTTTATCAGTTCACGTGTATGGCTGTAAATTATGTTTTGATATGGCAGCTATGAATTTAAGGTCTCAGTTTTAGGCAGTTCCTTTTGTAACACACCACACATGGAGTTTTTCTCTGCATGCAGGTGTGGCTCATATGTGCCCTTTTATAGACAACCCACCTCCCACCCATTTATATGTCCTGAACTGATCCTCGCGAATGTCGTGGCACACTCGTGATCGTCCCTCGACGTTCTGCCGAACACTTCTCATGGCGCTGTTGATGCAGGTTTGCGCAGAGTGAGGTGGCTAGAGAAGAAGTCATGAGCGGTGAACTTTTGCTGCACAAGTGCCAGGCAGCCTTTGGTCTGTGCGCATACTCCAAGGTGCATTCCATTTTTTAGGCGGCAGTGCTGCTTCATGCCTCAAATATTGCTGCTCCAGTCGAGACAGCACAAGAGAGAAGCTGTGTTTCCAGGGGTCATCGTGCTGCCCCTCTTACCATCCAGAAACTCTGCTGCTTCCACTGGGACAAAGTGAGCAACAGGTTCCTTTGTTGCGCCGGAAGCAAGACAAAGGGGGTTATTTATTATTATGTCAGAGTTGTATACACACAGGAGGGAGAGCATACACCCATTGCAGTCCCCTGCCTTTTGTGTGTGGCAAACAGTGGAAGGTGAAGCGTTTTTCTACACGTTACTGTATACAACATGCACATTATACTTGAAGCGTGTTCACCCAGTGCAAGTGTGTATGGCTGTGTGGGTGTATTTAAAGAAAAAAAAAGAAAAGCATGCATCTTAGTTTGTATGTGTCGCATTTGTCAGGAGCAGCTCGCATTTTGTTTTGAGAAAGCCAAGATGCTCAAGTCTAAGCAAAGTTTGATTTATTATTGTGTAAACATGCAACTAAAATGCTCACTGTGACAGTGCTGCAACTATACCACAGTATGTAGAAAGCGCCTGCATGAAGACAAGGTTGTGTTTTGATCCTAATGAGAGGATGAACTTAACATGTTGACGGGGTGTGTTATTTCTTTTGTGAACTTCAGTATATTAGTTTGAATGGATGAACAGAAAAATTGTTCTTGAATTGGTGCTTGTGTATAGCAAAGTGCATTTCAGGGACCTCCCCAAAAATTTGTTTAGACCCAAGTTTCCAAGGCACTGTTGTCCTGTTTGGCATAGTTTGCTGGTATTTTTCTGTCTGTAGCTTAGCGAGCATATCATAATGGGCTGTGAGCTAGACTTAACATGCCATTCATTCCGGGTGTGTATTGGCACATTATGCTGTATTTTTAGCGAAAACCAATGCTTGTATTGTGTGGCATGTGTCATTGCAAGTTTCATCTTTGTCTTTTTTATTATTTTTGGTTTTAATCATCACATTGTGCTAGTCACACCTTTAATGGAATACATTTAATGGAATGCACTTGTGGTTATTGCAAACACATCTGTTTGCTGACATGGTGACATGTGCACCCAACGCAATGTGCATTTTCCTTCTGTGCGTATACTCTTCGGGTCCCATTCAAAGTTGTTGCCCTTCTGCCCTCAGTACTGTGGTGTGTGTTGGCTGCAGCTGCACCAGTGAGCAGCAGTCATGCTTTTATTTCTCCCCCCTGCTTTGAGTGAGTTAGCAGCAGCTGTGAAAGTTGAACAAAGCTGTGCGCATACTCTTGAAGCAACTACCAGCAGAAAAGCAAAGCCATCAAGCATGTAGTACCACCACAACTTGTAGTGGAATCATATATATATATATATATTATGTATATTATATATGAAAGAGAGAAAGAATGCAGGTAGGGATTATATAATGTTGGTGTGTGTG

>MG4813710

GTCAAAGATGGGGGCATTGTCATTGACGTCCTTGACCTTGATGAGTACTGTTGTATGGTTCTCGTTGAGGTTATCCGATAACACACCAACTGTGCAAACCTCTTTCCTGGTCTCGTAGTCGAGCGGGCCGGCAACGTAGATGGCGCCAGTCTCGTTTTTCACTGCAAACGCTCCTCCTATGTTCCCCTGGGTGATCTCGTAGCGTATCCTCGAGGATTCGTCCTTGTCCTTGGCGGTGACAGTGATGACAGTGTGCTGCACATCCTCGTCTTCGTTGACCTCAGCCTCGTAGAGCGCCTGGCCGAAGTACGGCGGGTTGTCGTTCTTGTCGCCGATCCCAATGCGGATGTACTTGGTCACTGAGTTGGGCCGGTTGTCTGTCATGTGGGGCCGTGCCGATGGAGCACCATCCTCTGCCCGAACCAGGATTGCGTAGGCCATCTTCTCTTCTCTGTCAAACTCAACCTTGGTGTAGATGTCTCCAGTCTCACGATCAATGGAGAAGTACTTGTCACCATGGTCTCGAGATTCTATGGCATAATAAACCTTGTTATTCGGGTAAGTGCCGTCTTTGTCAACAGCCTGGACCTGAGTCACTTTGGTGCCTGGTGGTAAACCTTCCAGGACTGTTTCTTGCTCCCGCTCTATGAAAAGAGGGATCTCATCATTGACGTCCTCAAGGAC

>MG4818639

CTGACCCTGATGGGGATCACCTGTGGTACAGCCTTACAGGTGGGAATGATGAAGGAAAGTTTATGATTGACTGTGAGCTTGGCTTCATCAAGTTGGCAAGACCCCTGGATCGAGAAGCTTGCTCCAACTACAATCTGACTGTCGAAGTCACTGATGGCACTGCCAAATCCACAGCCACGGTACATGTGGAAGTCCTAGACGCCAATGACAATTGGCCCATTTTCTCGGAGAGCTTGTACCAGGTGGAAGTCTCCGAAAGCACAGCTGCTGGCACAGAGATTCTGCAGCTGACAGCTGCAGACGCTGATGAGGACCAACGTCTCTTCTACAGCATTCACAACAGTGGCCAAGTGGCAAGTGCAATCCACTTTCGGCTCGACTCAAACACGGGCTTGCTGTCGTTAGCAGAGCAGCTTGACCACGAG

>MG4820640

CTGGCCAAACACAGGCGGTTTATCATTGACATCACTCAGCAAGAGCTCCACGCTGCTGTTGCATTGCCACTCCCAACGAGCCACGTCACGCACATGTGCCACCAGTGAGTAGTGCGGCCGCTGCTCTCGGTCTAGAGGCCGGGTCGTGTGCAGCACCCCACTGCTGGCATCCAGAACAAAGTCCTCAGATCCTTGGCCTGACAGGTAGAAGTGCAGCTGAGCATGGTGGGCATCATCCGCATCTGTGGCTCCAACCGTCAAGATGGCAGTACCCACAGCAATGCTCTCCGAGACAAGCTCCGTGTACTTGGACTTGAGGCAGATTGGTGGATTATCGTTGCTGTCCAGGATGGTTATATGAAGCTGAGTCATGGCCACATGCAGACCGTCTGTGGCCACCACTCGAAGAGTGTAGTTCTGACGTGCTTCCCTATCCAGCGGTCTTTGTACGAACACATCACCACTACTGCGCACCGCAAACTGCTGACCAGGATCACCGGAGAGCACGTAATAGCCAACAGGAGCCGGCTCCTTGTCAGCATCTTCCACGCGCAGGCTGGCAACTACAGTGCCAGCAACAGCATCTTCAGAGACTTGCACTTGGTATAGGGTCTGCTCGAACACGGGAGGATTGTCATTTACATCCAGGACCTCGACGTACACCCAAGCTGAGGAAGACAGCAGACCGTCATCACGGGCCCTCACTGTAAAGTTGTAAAATGGCACGGCTTCTCGATCGAGAGCTACCAGAGTTGTAATAACCCCACTCTGCGGGTCAATCCGGAAGATGTCAGCAGCCGGATCAGTCTCTTCGTAAAATTCGTACGTCACCCTTCGCACTGAGACATCAGGGTCGTGTGCCACCAACCTGGCCACTACACAACCTTCCTCAGCATTCTCAGCAACTGCAGTCTGGTATGACATCACATCGAAGACAGNNNNNNNNNNNNNNNNNNNNATGACATGCATGGTCACATCAAGTGCGGAAGCCAGACCACGGGGTGTCTCTGCGCGCAAGGTGAGCTGATGTGATGCCTGGTGTTCGCGGTCAAGTGGCGCAGCAATGACCAAGTTGTTCCCTGCATCAATGCTGAACTCCGAGCTGCCTAGAAGTGTGGTGCCTGGTGCCAGCACCAGCAGTGGACTGCCGAGAGCTGCACTCTCACGAACATGCAGGTCACGGGTGGACACACCCA

>MG4825678

AGACTATGAGCAGGCGGTTGTGCATAACCTGACAGTGCGTGCCATCAATCTGGCCGGTGCCATGGCCCAGGGTCATGTGACAGTACGTGTGCTCGACAGTAATGACCATGCGCCTCGCTTCCGGCAGCTACTGTATCATGGTACCGTGAGTGAAGCGGCCCCCGCGGGTAGTGCAGTTCTCAGGTACAGCCAGCCTCTGGTGGTGGTGGCTGAGGATGAAGACACCGGCCTCAATGCACAGCTGTCATTTTCCATTGTAGAGGCCTGGGCCCGCAGGCTGTTCCGCATCGACGCCAACACAGGGGCCCTAAGCCTGGTGCAGCCCCTCGATCGGGAGTTGCAGGCCGAGTACAACTTCACTGTGGAGGTGTCGGACTGCGGGCAGCCACGACTGTCAGCTCAGCAGCCCGCCATGGTGAGCATTCGCGTGTCGGATGTCAACGACAGCCCACCCCGCTTCGAGCATGAGCAGTACAATGCCAGCCTGCTGCTGCCAACGTACGCCGGCGTGCGCGTGGTGCAAGTAGCCGCCCATGATCCGGACCTGGAGGGGCCCAGCTTACGGTACACCTTGATGGCTGGGGACCACGAG

>MG4825824

AAACCATTGCCCCCTTCGTCCTCCGCAAAAACGGTGAATCGCCACTGGGAACGTCCATGTGGAAGGTCTCTGTCTAAAGGCTTTAACACATATATTTCTCCTGTAGTTGTGTTGATTGCAAACTTGCTATTCCCAGGGTCTTCATCAACACCTTGACCCGTCAAAAAGTAAACTATCAAGGACTCCCTGTCCTTATCACCATCTGTTGCTGTCACCTGAAGTATCTTCTGGGGAAGGTTCCGGTCGTTCTCCTCAGTGATCTGAGCCTCATAGGTAGGCCGGTCAAAGATGGGGGCA

>MG4828426

CTTCTCTGTCCAGTGTACGCTGAATTTCAACAACACCATTGGGATTAATCTTGAACTGCCTTTTTATCAGATGATCGGTCAATCATGTAGCTGACACGTGACTTTCCACCTTGGTCGGCATCAGTGGCCTTAAATGTTGCCAAACTTGAACCAACGGTGGAATTCTCAGGAACAGACACTTCAATGTTGGGTCTCTCAAATTCAGGTTTGTTGTCATTTATGTCTTTAACCCTAACTTTGACCTTTGCGTAGTCCGCATGATAGGGATCTGTTGATTCCCCACCATTGTCGCTCACTTGAATGGTGATGTTGAACCCAAAACGCTGCTGCAAATCTTCATAATCTAAAGGCTTGGCAATTTTTAAAGAACCAGTTCCATCAGAATTGGTGACCATAGTGAATTTATCAGAGCCAAACGTGTTGTCAATA

>MG4829618

CTCAGCTGCGGCTGATGGTGGTGCCGAGCAATGAACACTCGCCCCGGTTCAGCCAGTCTGTGTATGAAGCAGTGGTGGCAGAGAACTTGCCGCCAGGTGCCCATGTGACCCGCGTTTCTGCCACGGATCCCGACCAGGGGGCGCTGGGCACGCTGACATATGCCATCCGCAGTCAAGACTGTGCACTGCACTTCAACATCAACAGCACCACAGGAGAGCTGGTGACTCGGGAAGCTTTGGACCGCGAACATCAACATCTTTACGAGGTTCCCATCTCTGCCACTGATGGTGGCGGCCGCCTGGCGTTTGCTCTTGTTCGTGTGGCTGTCACTGACGTCAATGACAATGAGCCCACTTTTGGTGCTTCTGAATATGAGGCTAGCATCTGGACAAACACTAGCGTGGGCACTACACTTGTCAAGGTTCGTGCTTTTGACCGTGACCAAGGAACATGGCCTCGTTACAGCCTTCATGAAGCCCCAACCAATGTCAGTGCATTGTTCAATGTCACTCATGACTCGGGAGAGCTTTACCTGAAGGCGCCCCTCAGAGGAG

>MG4832866

CAAGCCCGTATTTGAGGAATGCCAGACCTATACGCCTAAGGTAGAAGAGGGTGCTCAAAGTGGCACCTCAGTCATCAAGGTGAAAGCAAGAGATCTTGACAAAGGACACAATGGTCAAGTGCGCTACTCCATTGTCCAGCAGCCTAATCAAAAGGGCACAAAGTTCAGTGTCGATGAACTCACCGGAGAAATCAGGACAAACAAGGTTTTTGACCGTGAAGGGGATGATGGTCGTTTTGTGAGTGTCACTGTAAAAGCAACTGACCGTGGCAGCCCACCACTGGAAGGAGTCTGTTCCTTCAAAGTA

>MG4834784

GTTCGACATCGCCGCCGTGGGATGCAACGGGCTCGTCTCCGAAAACGTTACTGTGCACCTGACAGTGGATGACGTCAATGAGTTCGCGCCCCGCTGGGAGGAGGAGTCTTACCAAGGCAGCGTGGATGAGGGCCGGCCGCCACACGAGCGTGTGCTCCGCGTGCGTGCTCTTGATGCTGACTGCACGCCCAAGAACAGTGAGATCTGCAAGTATGATATCCTGGACGCACATGTGCCCTTCTCCATAGACTCTGAAGGCACCATCTGGACCACAGAGCCCCTGGATTGGGAGGCGAGCAGCAACCACATATTCCAGGTGGTGGCCTTCGACTGTAGCATGAAACAGAGTCATCCGGTCACTGTCACTATCAAGGTCAACAGGGTCTGCAAGGTTGGATGGAAAG

>MG4835166

CGGGCCACCCGCAGCACACCTGTCTTTGGCTCAATGCTGAAGTGTCCCATGTCGTTGCCTGCAACCAGGGAGTATGTGATCTGAGCATTGACGCCTGCATCTCGGCTGGTAGCCCTCACACCAGCGCTGACGGGCGAGCCCACGGGGGCAGCTTCAGAGACGCTGGCAGAGTACGCTTGCTGTGTGAACTCGGGCGGGCTGTCATTAATGTCCTGCACAATGACTGTAAGAGGCACGCGGGCAGCCCGGGGAGGCTGACCATGGTCCTGGGCCTGCACCGTCAGGTTGAACCTGGCGC

>MG4836783

CCATGACAAGCACCTACTTATTGTGACTGTGGCAGACAAGGCTCCTCAGGATCCCCTCTCCTCCACAGCCACTGTCAGCATTGTGCTTAGGGATGTTAATGACAATGCACCACGCTTTGCCCTTGCTCCTTACGTGGTCAGGATCCGTGAGGACTTGCCTGTGGGCACCCTGGTGACATCTGTGCATGCTGAAGATGCCGACTTTGGAGAGAATGGCAGGGTCCAATACTCTCTGGATGGCCAGCATGCTGGTGTCTTCGACATCGATCCTGACACTGGCCTTATTCGGCTGGCTGCATCGCTAGACTTTGAAACACTACGGCTGTACAACGTGTCTGTGCAGGCTCAGGATTGTGGTGACCCACCGTTCAGTAGTGTGGCGAGCTTAATCGTCGAAGTACAGGATGTCAACGAGAATCTGCATTCACCAGTCTTTCCTGAACAGGTGTCCAGTGTCAGCGTGGATGAGAACCAGCCTCCAGGAACATTTGTTACCACCCTGGCAGCTACAGATGAAGACGGTGTCACATACGCCATCACAAGTGGCAACGGCCTGGGCCTTTTCAGCGTTGATGATAATGGTACCATTCGGAGCCGGGTGTCACTGGATCGCGAGACCTGCGCTCACTACTGGCTCACTGTGGTGGCTCGTGACCTCAGCCCTGTGCCCCTAGAGGCTCGCCTTGACCTTCATGTGGCTGTGAATGATGTTAACGATCAGGTTCCCCTCACGGAAAAGCCAGCCTACA

>MG4837943

CAAACACTGGCGGGTTATCATTCATGTCTATCACGGTGACCCAGACACGGGCTGTAGAGCTCAGAGGTACAGGGTGGCCAGCATCCGATGCCACCACCTGTAGCCGGTGCAGGGCTTGTGTCTCCCGATCAAGAGGGGCCCGAAGCCGCAGGACACCGGAACCTTCCAGGTGGAAAGTGGCACTGTCGTCGTCAGCCAGTCTGAGCTCCAGCATTGGTCCATCACGGTCACTGGCGCGCAACTCCAGAAGCACGCTGGCCACAGGCGTGGATTCTGACACGCTCACATTGTACCAGGGCT

>MG4840520

CTTATTCAGCAGTTCTTCCGTGTGGAACATGACCCCACTGGTGCCAATGGCGACGGTATGGCTGTGGTGCGCTCTCGAGAAACTTTCGACAGAGAAACTCAGAAGGAATACTTGGTTCCCATCCTTATCAAAGACAACGGCAACCCATCTCTCACGGGGACTTCGACACTCACTGTCATTATCGGGGATGTGAATGACAACCGCATGCACCCTGGCAGCAAGAGCATCTTCGTGTACAATTTCAAAGGGGAGTCTCCACCAACAAAAATTGGGCGCATTCATGTTGAGGACTTGGATGACTGGGACCTGCCTGATAAGACTTTCTTTTGGGAGAATAATAATGCTCATCCCAACTTTGACATGGACGGCCACACAGGCATGATCACAATGCTGAACTCAACAGGGGCAGGCACCTACCATTTGCGTTTTTTGGTTTATGACCGTATGCACACAATGGATGCTCATGCCAATGTGACAGTAACAGTCAAAGAAATTCCCGAAGAGGCTATCTACAATTCTGGATCTATTCGTGTTCATGGTATTACTGATGAAGACTTCATAAGAGTTTGGGATTGGAAAAAGCAGCGGCAAGTGAAGAGCAAATATGAAATATTTCGAGACTTGATTGCTGAACGCATCAAGACAAAGCCATACAATGTGGACATCTTCAGCGTCATCTTGAAG

>MG4841453

CAAGTACCATCATCACATCAGCAAAGTGGTGGCCATCTGTTGCTGTCACATTCACACGGGCAGGCTTGCGGAGTTGACACGCTAGGCTGAGCACACCTGTGCTGGTGTTAAGGTGGAAGCAAGTGCTATCACTGCTTGCCTCCATCTTGTAGTGCCCCCTGCCAGTACCACCATCATCAAAATCCACAGCCGAAAGCGTCAAGACTTGGCTTCCANNNNNNNNNNNNNNNGACACAGAAGCAATACAGTCAACCTTCTCGAATTGGGGCCTGTGGTCATTGACATCTCTCAGTGTCACCATGAGAGCCATCTCTGCCTGCCGCTGGTAAGGTGCACCCCAGTCAGTGGCACGAATGCGTAGCGTGTAGCTGCGGCGCATTGTCTCATAGTCCAACACCTCAGTCGTAGACACCTCACCGC

>MG4842170

CGGGGTCAATCGAGAACTGCCCAGGACTGGGCATGGAGTAGCTGAGATGGCCTTGCACATCATCAGGGTCCAGAGCCAACACCTGGCACAGAGGTGCTGGTTCCTGCTGGCCATTTGCTGGAAGGACACGGAAGCGGTACAAAGGNNNNNNNNNNNNNNGCTCATGATCGTTGACATCGGTGAGTGTGATGAGCACCTGGGTGGTGGACGAGAGCGGTGGCTGGCCGCCATCACTGATCACCACCTCAAGCACGTGCCG

>MG4844492

GGAGGAGCGTGCTTTCCACGAGCTGGCTCTGGAGGCACGCTCTGCCCGTGCGGTGGCCCATGCTCTTCTGCGTGTGCGCGTGCTGGATGAGAATGATAAAGCGCCAATCTTTGTCGACCGGCCTTACCACTCGGCCATCACTGTGGATGCCCAGCCTGGAGACCTCGTGCTCAAAGTGAAAGCCATTGACTTGGATGAAGGCCCNNNNNNNNNNNNNNNNNNGTTTTTGCATGAGGGTGATGGTCAGACGTTTGGTGTGGATGAGCAGTCAGGTGAAATCCGGCTGCTCCGAGCTCCTAGTCGTCGGGACGAACTTGCCCTGGTGCTTGTGGCTCGTGACCATGGGAGTCCTCCTCTCGAGAGCCATGCTAGTGTGGCTGTGAAGGTGACAGATCGAGC

>MG4846772

CCACTATGAGGCAGTCGTCTCAGAGGCAGCGCCACTGGGCCACACACTGCTCCGCCTCGGACCTTCATCTCCGGGTCTTCGCTACACCATCAGCTCGGGAAACCGGCTTGGCTGCTTCCGGCTTGACGTTGCTGGTGACTTGGTTGTGGCCTCAGCACTGGATTATGATGCTGTTCCGGAGATGCGTCTCGTGGTGCGTGCTACAGACAGTCGCCGGGCAGCACTAGCCAGTGTACTTGTGCGTCTGCAAGATGAGAACGACAATGCACCACGGTTCCCATTGCC

>MG4846903

CTGGAAGCTCAAATCGAGGCGCATTGTCGTTCACATCAGTTACTTTAATTGTTAGCTCAACAGAGGTTGAAAAGCCACCGGAGTCTTCAGTTGCAGTGACAATAAGAGAGTAGGATTTTGGCTGCCGTAGGTCTTCATAGTCCAGCTCTTTGGCCAACTTCACTACTCCGCTGGTAGGTCCGATGTTGAAGGTGCCAGCTCCCTTGCCCTGGGCCCGCAGAGTGTACCGGATCTCCCGATCGGCAAAAGATTTGGCCTTCACCTCGATGATATCGGAGTCTTT

>SG12034410

CCGAAGTACGGCGGGTTGTCGTTCTTGTCGCCGATCCCAATGCGGATGTACTTGGTCACTGAGTTGGGCCGGTTGTCTGTCATGTGGGGCCGTGCCGATGGAGCACCATCCTCTGCCCGAACCAGGATTGCGTNNNNNNNNNNNNNNNNNNCGTCAAACTCAACCTTGGTGTAGATGTCTCCAGTCTCACGATCAATGGAGAAGTACTTGTCACCATGGTCTCGAGATTCTATGGCATAATAAACCTTGTTATTCGGGTAAGTGCCGTCTTTGTCAACAGCCTGGACCTGAGTCACTTTGGTGCCTGGTGGTAAACCTTCCAGGACTGTTTCTTGCTCCCGCTCTATGAAAAGAGGGATCTCATCATTGACGTCCTCAAGGACTATGTAGACAGTTGCCTCTGATGCCAACTGCTGGATGCCGTTGTTCTCGACACGAACAGTAAGGTTGTACTGCTGGATACGTTCATAGTCCAGAGGATAATTGACCACCAGCTCTGCCACTGTTTGGCGATTCTCAAGCTTTTGATTCAAGTAGAAAGTGTCCTTCTTGTTGGTCTGCTCTGTACTTCCTTTCATCAACGTGTAGAACACATCAGGATTCTGTGGGATTCCGGATCTGGCTTTGATAGCAATGACACGAGTGCCCACTTCAAGGTTCTCCTTGATAAATATTGGACCATACACAGGTTGGTCCCATATCGGTGGAT

>SG12035984

CGGCGACCGGAGCCTGGTGTGTACTCCACATGCTCCTCCATACCTTTCCATCCAACCTTGCAGACCCTGTTGACCTTGATAGTGACAGTGACCGGATGACTCTGTTTCATGCTACAGTCGAAGGCCACCACCTGGAATATGTGGTTGCTGCTTGCCTCCCAATCCAGGGGCTCCGTGGTCCAGATGGTGCCTTCAGAGTCTATGGAGAAGGGCACATGTGCGTCCAGGATGTCATACTTGCAGATCTCACTGTTCTTGGGCGTGCAGTCAGCATCAAGAGCACGCACGCGGAGCACACGCTCGTGTGGCGGCCGGCCCTCATCCACGCTGCCTTGGTAAGACTCCTCCTCCCAGCGGGGCGCGAACTCATTGACGTCATCCACTGTCAGGTGCACAGTAACGTTTTCGGAGACGAGCCCGTTGCATCCCACGGCGGCGATGTCGAACTTGTAG

>SG12036366

CTTGGGATGTCTTCTCTGTCCAGTGTACGCTGAATTTCAACAACACCATTGGGATTAATCTTGAACTGCCTCTTTTTATCAGATGATCGGTCAATCATGTAGCTGACACGTGACTTTCCACCTTGGTCGGCATCAGTGGCCTTAAATGTTGCCAAACTTGAACCAACGGTGGAATTCTCAGGAACAGACACTTCAATGTTGGGTCTCTCAAATTCAGGTTTGTTGTCATTTATGTCTTTAACCCTAACTTTGACCTTTGCGTAGTCCGCATGATAGGGATCTGTTGATTCCCCACCATTGTCGCTCACTTGAATGGTGATGTTGAACCCAAAACGCTGCTGCAAATCTTCATAATCT

>SG12038717

GGTCGTTTTGTGAGTGTCACTGTAAAAGCAACTGACCGTGGCAGCCCACCACTGGAAGGAGTCTGTTCCTTCAAAGTAGAAATAACTGATATCAATGACAACCCACCCCTATTTGACAGGCAGGAGTATAGGGAAAATGTGAAGCAAGACACCCAGGTTGGGATTCATATCTTGAGGGTGTCTGCTTCTGATGAAGACGCAGATAACAATGGTGCCATTGTCTACAATTTGACTGCACCATATGACCCTGAGCACTTGGCATATTTCTCCATCAATCCAGACTCCGGGTGGATTAGCCTGCAAAAGGCCCTTGACCGTGACCAGTATCAGTGTCGAGCCATAGCGCTGGACAAAGGTGTTCCCCAGCATCAGGCAACGGTGGAAGTGATCATTGATGTTGTTGACAGAGCCAATAATCCACCGGTATGGGA

>SG12041201

GCCACCAGTGAGTAGTGCGGCCGCTGCTCTCGGTCTAGAGGCCGGGTCGTGTGCAGCACCCCACTGCTGGCATCCAGAACAAAGTCCTCAGATCCTTGGCCTGACAGGTAGAAGTGCAGCTGAGCATGGTGGGCATCATCCGCATCTGTGGCTCCAACCGTCAAGATGGCAGTACCCACAGCAATGCTCTCCGAGACAAGCTCCGTGTACTTGGACTTGAGGCAGATTGGTGGATTATCGTTGCTGTCCAGGATGGTTATATGAAGCTGAGTCATGGCCACATGCAGACCGTCTGTGGCCACCACTCGAAGAGTGTAGTTCTGACGTGCTTCCCTATCCAGCGGTCTTTGTACGAACACATCACCACTACTGCGCACCGCAAACTGCTGACCAGGATCACCGGAG

>SG12044093

GGCCAGGCGCTCTACGAGGCTGAGGTCAACGAAGACGAGGATGTGCAGCACACTGTCATCACTGTCANCGCCAAGGACAAGGACGAATCCTCGAGGATACGCTACGAGATCACCCAGGGGAACATAGGAGGAGCGTTTGCAGTGAAAAACGAGACTGGCGCCATCTACGTTGCCGGCCCGCTCGACTACGAGACCAGGAAAGAGGTAGGGTACGCCGTCCTGGCTGGGAGCTCCGATTCCTCGCCTCTTCGAATGTTCCTCAGG

>SG12044707

GTCAAAGTTGGGATGAGCATTATTATTCTCCCAAAAGAAAGTCTTATCAGGCAGGTCCCAGTCATCCAAGTCCTCAACATGAATGCGCCCAATTTTTGTTGGTGGAGACTCCCCTTTGAAATTGTACACGAAGATGCTCTTGCTGCCAGGGTGCATGCGGTTGTCATTCACATCCCCGATAATGACAGTGAGTGTCGAAGTCCCCGTGAGAGATGGGTTGCCGTTGTCTTTGATAAGGATGGGAACCAAGTATTCCTTCTGAGTTTCACTGTCGAAAGTTTC

>SG968858

CGACCCTTGGACTTTGAGAGCGCGCGCAGCTACCAACTGGCAGTGGAGGCTCGCGATGGGGGCGAGCCACCCCTCAGTGCCCGCGCCTGGCTGAATGTGTCCGTACTGGACGCAAATGACAACGCGCCTGTGTTCGGAGGCCCCTACAGTGGCACTGTCGCGGAGGATGCAACCCCTGGGCAGCTTGTGCTCCAGGTGCAGGCCAGTGATGCCGACAGTGGCGGAGAGCTGCGATATGCTCTGCTGCAGCCGGGCCCTTTTACTCTGGACCCGGCCACTGGCCAGCTAAGTGTGGCTGGGCCGCTGGACCATGAGACGGTGAGCCGGTACGCACTGGAGGTGGAGTGCTG

>SG9625689

GTGAACACAGAGGTCGGCTACCATGCCATCGTCAAGGAAAACAGCCGCAATGTGGAGATCATGCCCAGGATCCGGGTGCTGGACGCCAAGGTGTGCCGCTTCTTGGTGACCAACAAGCGTCACGGTGAGGCGCCCTTCGAGGTGCGCATCACGGACGAGGCGACGGGCAAGGCCGAGCTGTTCGCCAAGAAGGAGCTCAACTGCGAGAAGCACCGAAACTACAAGTTCGACATCGCCGCCGTGGGATGCAACGGGCTCGTCTCCGAAAACGTTACTGTGCACCTGACAGTGGATGACGTCAATGAGTTCGCGCCCCGCTGGGAGGAGGAGTCTTACCAAGGCAGCGTGGATGAGGGCCGGCCGCCACACGAGCGTGTGCTGCGCGTGCGTGCTCTTGATGCTGACTGCACGCCCAAGAACAGTGAGATCTGCAAGTATGATATCCTGGACGCACATGTGCCCTTCTCCATAGACTCTGAAGGCACCATCTGGACCACAGAGCCCCTGGATTGGGAGGC

>SG9633166

TGAAGAGCTGCCTGTGCTGGGTGTGTCCACCCGTGACCTGCATGTTCGTGAGAGTGCAGCTCTCGGNNNNNNNNNNNNNNNNNNNNNNNNNNNNNNCACACTTCTAGGCAGCTCGGAGTTCAGCATTGATGCAGGGAACAACTTGGTCATTGCTGCGCCACTTGACCGCGAACACCAGGCATCACATCAGCTCACCTTGCGCTCAGAGACACCCCGTGGTCTGGCTTCCGCACTTGATGTGACCGTGCATGTCATGGATGTCAATGACTGTGAGCCTGTCTTCGATGTGATGTCATACCAGACTGCAGTTGCTGAGAA

>SG9642036

CCTTGTTATTCGGGTAAGTGCCGTCTTTGTCAACAGCCTGGACCTGAGTCACTTTGGTGCCTGGTGGTAAACCTTCCAGGACTGTTTCTTGCTCCCGCTCTATGAAAAGAGGGATCTCATCATTGACGTCCTCAAGGACTATGTAGACAGTTGCCTCTGATGCCAACTGCTGGATGCCGTTGTTCTCGACACGAACAGTAAGGTTGTACTGCTGGATACGTTCATAGTCCAGAGGATAATTGACCACCAGCTCTGCCACTGTTTGGCGATTCTCAAGCTTTTGATTCAAGTAGAAAGTGTCCT

>SG4812566

CAGGTGGATGGGGCCATTGATGAGTCGGATGACACCTGTCCTTTCTTCAATCTGGAACATGCCAGAGGTGGTGCCACTGTTGACAAATCCAAAGAGGATGTTATCTCCATCCTTGTCGCTGGCTACCACAGTGGTCACGAGAGTGCTGGGCCCAGCATTTTCATCCACGTTTGGCGTGTAAACGTCCTGGCTGAACTTCGGCGCCTCATCGTTCTCATTTTCCGTGTATATGCGAATTGTAGCCGTGCCTGTCAGTGGCGNNNNNNNNNGATCTGTAGCCCGCACTGTCAGAACATACGTGTTGTTCACATCAGCATCAAGCCGCCGATTGGAGTACACCACACCCCGGCTGTCAATGGTAAAATCTTC

>SG4818739

CGGTAAGCCACTGTGCTAGCTGGCACTGGATCTCCTTCAGGCTGCACTGCCAACAACAAGTCCAGCTGCTCGCTCCCTCCTGAGGCCTCCGCTGGTGCTGGCTGTATGCTCACCAGCAACAGGTCCCGCAAACGCACGCTGAGAGCTGTTCGCAAAGCTCGCAGCAATGGCCGCCGGTCCGTAGATAAGAAGCGCTCAGTCGTGGTTCCAGCCAAGCGCAGAGCCACAGCTGCCTTGAGAGCCTCCTCGGTCACCCCGACAACATGCACATTCACTGATGCATGTGCTGGCGTTGTGCTGCCATCTGAAGCGCTCACATTAAGGACATAGCTGCCAGCATCAAGGCCTGGCAAAGCACGCAAAGTCCCATCATCCCTGTCGAGGACAAACAGCGATGCGTGTGGCCCAGAGAGGGACAAGCTGAGGCGGTCGTACGGGTCCTCATCCGTGGCATGCACACGGCCTAGCAGCCCACCCGGGAAGTCGTCCAAGTAAGAAGAGACAACCACAGTCAAGGGCTGCACCTGTGGCCTGAAGCGGCTCTTCTCAACAGCCAGCACAGTCACATGCGCCTCTGTCGCCAGAGGTGGGCTGTCCGAGTCGTGAGCCTGCACACGCAGCTCATGCCGCGAGCGCGGAGGAAGTGGTCGTGCTAACCGCAGCTCGTGGCCCTCAACTTGGAAAAAGGGTTCCTCCTCCTCCTGCCCAGCTTGAGCTCCATCCAGCAGCTCCAGCCGGAAAGGAGGTCCATGAGCAGCAGAGTCAGCATCACTGAGCGAGAAGCGCAGCACGGTCCAGCCGACTGGCCGACCCTCATGAACCACGGCCGTGTAGTTGCTCTGGTCAAAGCGCGGTGGATGGTCATTCACGTCCAGCACCTCCAGGTGCACCAGTGCCCGAGCCGACAGTGGCGGTGTGCCACCATCCCAGCACTCCACCTCCAGTGCGTACCGGCTCACCGTCTCATGGTCCAGCGGCCCAGCCACACTTAGCTGGCCAGTGGCCGGGTCCAGAGTAAAAGGGCCCGGCTGCAGCAGAGCATATCGCAGCTCTCCGCCACTGTCGGCATCACTGGCCTGCACCTGGAGCACAAGCTGCCCAGGGGTTGCATCCTCCGCGACAGTGCCACTGTAGGGGCCTCCGAACACAGGCGCGTTGTCATTTGCGTCCAGTACGGACACATTCAGCCAGGCGCGGGCACTGAGGGGTGGCTCGCCCCCATCGCGAGACTCCACTGCCAGTTGGTAGCTGCGCGNNNNNNNNNNNNCCAAGGGTCGGGCCACCCGCAGCACACCTGTCTTTGGCTCAATGCTGAAGTGTCCCATGTCGTTGCCTGCAACCAGGGAGTATGTGATCTGAGCATTGAC

>SG4825502

AATGTCATTGCTGGTGGAGATGTCCAGTATGTTGGCCCAGGTGTGACAGTCGTTGACAACGACTACCAAGAAGGGTGCATGAATGACATCCGGCTTGACCAGCACTACTTGCCAATGGAAAGTGGTTCTGAATATGCGGCTGTAGTAGAATGGCGCAACCTTATTGATGGATGTCCATCCAATAATCCTTGCCATGGAATCAGTTGCCCACGCCCCTTCGTTTGTGTCGACCTTTGGATGCTCCATGAGTGCAGGTGCTCCCCTGGCTTTGCTGTAACGGAGAATGGCAAGAACTGCACGGATGCGGATGAGTGCCTGACGGAGCCATGCCTCAACGGAGGCACCTGCATGAACCGGCCACACGGCGAGGGCTTCTACTGCCTATGCCCAGATGGTTTTGGGGGCGACCTTTGTGGCGCCTTGCGACAGGAGAAGATAATGCGACTCAGTATGGCTGCCTTGGCTGCCATCCTGGTCTGTCTGCTCAACATCCTCATTTTGGTCCTGGTGATTGTGGCATACACCCGGAGCCGACGGTCAGACCAGAAGTTTGGCCATGGTGGAGTGGACGACGATGTGCGAGAGAACATCATCAGCTATGATGATGAAGGCGGCGGAGAAGATGACATGAATGCCTATGACATAACACCACTACGAATACCAGTTGATGCATCTGGCATGCAGATTGGCGCAAAGCCTGGCCCAGAAAAAGCCCCACCTTTGCAGAAAGAAGTTCGTGTCCGGGAACCACCCACAGGGGCCCACCCAGATGTGGGTGACTTCATTCGGGACCACCTGGACAAGGCAGACCAAGACCCTGGAGCTCCTCCAGTGGATGATGTGCGCAACTATGCCTATGAGGGTGGTGGCTCCACTGCAGGCTCACTCAGTTCCCTGGC

>SG4826366

CTGCTCTCGGTCTAGAGGCCGGGTCGTGTGCAGCACCCCACTGCTGGCATCCAGAACAAAGTCCTCAGATCCTTGGCCTGACAGGTAGAAGTGCAGCTGAGCATGGTGGGCATCATCCGCATCTGTGGCTCCAACCGTCAAGATGGCAGTACCCACAGCAATGCTCTCCGAGACAAGCTCCGTGTACTTGGACTTGAGNCAGATTGGTGGATTATCGTTGCTGTCCAGGATGGTTATATGAAGCTGAGTCATGGCCACATGCAGACCGTCTGTGGCCACCACTCGAAGAGTGTAGTTCTGACGTGCTTCCCTATCCAGCGGTCTTTGTACGAACACATCACCACTACTGCGCACCGCAAACTGCTGACCAGGATCACCGGAGAGCACGTAATAGCCAACAGGAGCCGGCTCCTTG

>SG4827944

TGTTGCTGTCACCGTCATAATTTTCATTGGCAAATTGTGGGACACCTCCTCCTCGATGGTGGTCATGTATGAAGGTTGGCCAAAAACAGGCGGAAGATCGTTGACATCTTTGACGTGGATAAGAACTGTGGTGTTGCCTTCATGAAAGCCATCCGATGCCACCAGCGTCAAGTTAAACTCTTTCCTGGTCTCGTAGTCGAGCGGGCCGGCAACGTAGATGGCGCCAGTCTCGTTTTTCACTGCAAACGCTCCTCCTATGTTCCCCTGGGTGATCTCGTAGCGTATCCTCGAGGATTCGTCCTTGTCCTTGGCGGTGACAGTGATGACAGTGTGCTGCACATCCTCGTCTTCGTTGACCTCAGCCTCGTAGAGCGCCTGGCCGAAGTACGGCGGGTTGTCGTTCTTGTCGCCGATCCCAATGCGGATGTACTTGGTCACTGAGTTGGGCCGGTTGTCTGTCATGTGGGGCCGTGCCGATGGAGCGCCATCCTCTGCCCGAACCAGGATTGCGTAGGCCATCTTCTC

>SG4831166

GTACGAGTTGAATGTGACAGCACGAGATGATGGTGCCTGCTGTCGAAATGGGGCTCTGACTCCCCACACAAGCACAGCCCTAGTGGTTGTGTTCATAACGGATGTCAATGACAACAAGCCCGTATTTGAGGAATGCCAGACCTATACGCCTAAGGTAGAAGAGGGTGCTCAAAGTGGCACCTCAGTCATCAAGGTGAAAGCAAGAGATCTTGACAAAGGACACAATGGTCAAGTGCGCTACTCCATCGTCCAGCAGCCTAATCAAAAGGGCACAAAGTTCAGTGTCGATGAACTCACCGGAGAAATCAGGACAAACAAGGTTTTTGACCGTGAAGGGGATGATGGTCGTTTTGTGAGTGTCACTGTAAAAGCAACTGACCGTGGCAGCCCACCACTGGAAGGAGTCTGTTCCTTCAAAGTAGAAATAACTGATATCAATGACAACCCACCCCTATTTGACAGGCAGGAGTATAGGGAAAATGTGAAGCAAGACACACAGGTTGGGATTCATATCTTGAGGGTGTCTGCTTCTGATGAAGACGCAGATAACAATGGTGCCATTGTNTACAATTTGACTGCACCATATGACCCTGAGCACTTGGCATATTTCTCCATCAATCCAGACTCCGGATGGATTAGCCTGCAAAAGGCCCTTGACCGTGACCAGTATCAGCTGCGAGCCATAGCGCTGGACAAAGGTGTTCCCCAGCATCAGGCAACGGTGGAAGTGATCATTGATGTTGTTGACAGAGCCAATAATCCACCGATATGGGACCAACCTGTGTATGGTCCAATATTTATCAAGGAGAACCTTGAAGTGGGCACTCGTGTCATTGCTATCAAAGCCAGATCCGGAATCCCACAGAATCCTGATGTGTTCTACACGTTGATGAAAGGAAGTACAGAGCAGACCAACAAGAAGGACACTTTCTACTTGAATCAAAAGCTTGAGAATCGCCAAACAGTGGCAGAGCTGGTGGTCAATTATCCTCTGGACTATGAACGTATCCAGCAGTACAACCTTACTGTTCGTGTCGAGAACAACGGCATCCAGCAGTTGGCATCAGAGGCAACTGTCTACATAGTCCTTGAGGACGTCAATGATGAGATCCCTCTTTTCATAGAGCGGGAGCAAGAAACAGTCTTGGAAGGTTTACCACCAGGCACCAAAGTGACTCAGGTCCAGGCTGTTGACAAAGACGGCACTTACCCGAATAACAAGGTTTATTATGCCATAGAATCTCGAGACCATGGTGACAAGTACTTCTCCATTGATCGTGAGACTGGAGACATCTACACCAAGGTTGAGTTTGAGA

>SG4837050

CGCATGCACCCTGACGGTATGGCTGTGGTGCGCTCTCGAGAAACTTTCGACAGAGAAACTCAGAAGGAATACTTGGTTCCCATCCTTATCAAAGACAACGGCAACCCATCTCTCACGGGGACTTCGACACTCACTGTCATTATCGGGGATGTGAATGACAACCGCATGCACCCTGGCAGCAAGAGCATCTTCGTGTACAATTTCAAAGGGGAGTCTCCACCAACAAAAATTGGGCGCATTCATGTTGAGGACTTGGATGACTGGGACCTGCCTGATAAGACTTTCTTTTGGGAGAATAATAATGCTCATCCCAACTTTGACATGGACGGCCACACAGGCATGATCACAATGCTGAACTCAACAGGGGCAGGCACCTACCATTTGCGTTTTTTGGTTTATGACCGTATGCACACAATGGATGCTCATGCCAATGTGACAGTAACAGTCAAAGAAATTCCCGAAGAGGCTATCTACAATTCTGGATCTATTCGTGTTCATGG

>SG4842298

GAACTTGCCGCCAGGTGCCCATGTGACCCGCGTTTCTGCCACGGATCCCGACCAGGGGGCGCTGGGCACGCTGACATATGCCATCCGCAGTCAAGACTGTGCACTGCACTTCAACATCAACAGCACCACAGGAGAGCTGGTGACTCGGGAAGCTTTGGACCGCGAACATCAACATCTTTACGAGGTTCCCATCTCTGCCACTGATGGTGGCGGCCGCCTGGCGTTTGCTCTTGTTCGTGTGGCTGTCACTGACGTCAATGACAATGAGCCCAC

>SG4845085

GTGCTTAGGGATGTTAATGACAATGCACCACGCTTTGCCCTTGCTCCTTACGTGGTCAGGATCCGTGAGGACTTGCCTGTGGGCACCCTGGTGACATCTGTGCATGCTGAAGATGCCGACTTTGGAGAGAATGGCAGGGTCCAATACTCTCTGGATGGCCAGCATGCTGGTGTCTTCGACATCGATCCTGACACTGGCCTTATTCGGCTGGCTGCATCGCTAGACTTTGAAACACTACGGCTGTACAACGTGTCTGTGCAGGCTCAGGATTGTGGTGACCCACCGTTCAGTAGTGTGGCGAGCTTAATCGTCGAAGTACAGGATGTCAACGAGAATCTGCATTCACCAGTCTTTCCTGAACAGGTGTCCAGTGTCAGCGTGGATGAGAACCAG

>AAUF10035

CAGGTTCAACCTGACGGTGCAGGCCCAGGACCATGGTCAGCCTCCCCGGGCTGCCCGCGTGCCTCTTACAGTCATTGTGCAGGACATTAATGACAGCCCGCCCGAGTTCACACAGCAAGCGTACTCTGCCAGCGTCTCTGAAGCTGCCCCCGTGGGCTCGCCCGTCAGCGCTGGTGTGAGGGCTACCAGCCGAGATGCAGGCGTCAATGCTCAGATCACATACTCCCTGGTTGCAGGCAACGACATGGGACACTTCAGCATTGAGCCAAAGACAGGTGTGCTGCGGGTGGCCCGACCCTTGGACTTTGAGAGCGCGCGCAGCTACCAACTGGCAGTGGAGGCTCGCGATGGGGGCGAGCCACCCCTCAGTGCCCGCGCCTGGCTGAATGTGTCCGTACTGGACGCAAATGACAACGCGCCTGTGTTCGGAGGCCCCTACAGTGGCACTGTTGCGGAGGATGCAACCCCTGGGCAGCTTGTGCTCCAGGTGCAGGCCAGTGATGCCGACAGTGGCGGAGAGCTGCGATATGCTCTGCTGCAGCCGGGCCCTTTTACTCTGGACCCGGCCACTGGCCAGCTAAGTGTGGCTGGGCCGCTGGACCATGAGACGGTGAGCCGGTACGCACTGGAGGTGGAGTGCTGGGATGGTGGCACACCGCCACTGTCGGCTCGGGCACTGGTGCACCTGGAGGTGCTGGACGTGAATGACCATCCACCGCGCTTTGACCAGAGCAACTACACGGCCGTGGTTCATGAGGGTCGGCCAGTCGGCTGGACCGTGCTGCGCTTCTCGCTCAGTGATGCTGACTCTGCTGCTCATGGACCTCCTTTCCGGCTGGAGCTGCTGGATGGAGCTCAAGCTGGGCAGGAGGAGGAGGAACCCTTTTTCCAAGTTGAGGGCCACGAGCTGCGGTTAGCACGACCACTTCCTCCGCGCTCGCGGCATGAGCTGCGTGTGCAGGCTCACGACTCGGGCAGCCCACCTCTGGCGACAGAGGCGCATGTGACTGTGCTGGCTGTTGAGAAGAGCCGCTTCAGGCCACAGGTGCAGCCCTTGACTGTGGTTGTCTCTTCTTACTTGGACGACTTCCCGGGTGGGCTGCTAGGCCGGGTGCATGCCACGGATGAGGACCCGTACGACCGCCTCAGCTTGTCCCTCTCTGGGCCACACGCATCGCTGTTTGTCCTCGACAGGGATGATGGGACTTTGCGTGCTTTGCCAGGCCTTGATGCTGGCAGCTATGTGCTTAATGTGAGCGCTTCAGATGGCAGCACAACGCCAGCACATGCATCAGTGAATGTGCATGTTGTCGGGGTGACCGAGGAGGCTCTCAAGGCAGCTGTGGCTCTGCGCTTGGCTGGAACCACGACTGAGCGCTTCTTATCTACGGACCGGCGGCCATTGCTGCGAGCTTTGCGAACAGCTCTCAGCGTGCGCTTGCGGGACCTGTTGCTGGTGAGCATACAGCCAGCACCAGCGGAGGCCTCAGGAGGGAGCGAGCAGCTGGACTTGTTGTTGGCAGTGCAGCCTGAAGGAGATCCAGTGCCAGCTAGCACAGTGGCTTCCCGGCTTCACGAGCGCCATGTAGCCCTCGAGGCAGCTACTGGTCTACGTGTTCACATATTGCCCCAGGGGGACCGCTGCCCTTCCCTGCACTGTGTGCACGGCGAGTGTCAGGACCGGCTGGTGCTGGACACCAGCGACGCTGTTGTCCTTGCTGGTGGTGGCCACAGTTTCGTATCACCACGACACAGCCGCCGGACGGCGTGTGTCTGCAACCCTGGTTTTGGCGGTGATGCATGCGAAACAGCAGTGGATGAATGTGCCCAGCAGCCCTGCCCTGCAGGGCGCCTCTGCGTGCCAGACGCGTCTCCACTCGGCCACAGTTGCCAATGTCCATCTGGTCGCACTGGTTCTGAATGCGACACCCCTTGCCAGGAACCCAGCTGCTATGAAGAAAAGCGGCCCATCTCGTTTGGTGGCCAGAGCTATGCGCTCTACGTGCTGTCACAGCCCTTGGACCGACGCCTCTCGTTCTCTGTGATGCTGCGCACAATCCATCCCAGTGGCACTCTGCTGCACACATCGGGACCCCGAGACTACGCCATCCTTGAGGTGTCGGACGGGCACGTGCAGTACCGCTTCGACTGTGGCAGCGGTGAGGGTTTGGTGCGTGTGACTGGCCGTCGGGTGGATGACGGTGTCTGGCATGCACTGCGTCTCGAGCGTCGTGGCAGCAATGCGCGGCTCGCTGTCGACGTGCATTACCAAGCTTCTGGTGCCGCGCCTGGGCCACACGATGTTCTCAACCTGGAGGGCCGTGAGCTGCACCTGGGCGGGGCACCTGCTGTGGCAGGCTTGGTCGGCTGTCTGGATGATGCGCAGGTGGGCGGCCAGCCACTACCGCTGCATCTGCGACCTGCTGGCCACGCGCAGCTGCGCCGGCTGGCCAACGTCCAGTTTTCTTGCCACCTCGACCCATGTGGCAGCCAGCCCTGCCTCAATGGTGCAACCTGCCGGCCGCTTCTCACCACCGCTGGATACTCGTGCACTTGCCCGGCGCACTTTCAGGGTCCCCAGTGCAGAGAGGCAGTAGCTGAGAGCCGATGCACTGACGAAGCATGTGAGCCAGCCAGCTGCCAGCCCAACCTGTGCCTCCATGGGGGCCTGTGCCAGCCAGGTGGACACTGCCACTGCCCAACTCCTTATCAGGGCAACCGATGTGAGCTGGTGGAGGCATGTACGGCGTGCGAGAACGGGGAGAGCTGTGTGCGGCTGCCAGATGGCTTCCAGTGTGGCTGTCTGGGGGATGCATCGTGTGATGCCACTTTCGAGGGCCTGCCCCTCGCTTGGCCTCTGGGAGGTGCTGCTGCTGCTGTGCTGCTTCTGCTCCTGCTTCTGGCCATTGTTTGCTGCTGCCGCCAATGCTGTCGTTGCCGCCACCGCCACGAACATGTTGACAGCCATCAGTGCAGCAATCCTGTCACTGCCAAGAACTGTGTCCTGGCCACCACAAACATTAGGCCAAAGATAAGCAACCTGGAGCAGCGGCCAGCTTCGTACACGACGACTGGCGTGAGAGAGATCACACTGAACAACTTTGACACGGTGCGCAGCTATGGAAGTGCAGCTGATGACCTTGAGTCACGCTTTCAGCCCAATGACCTGCGCTGCCACAATCTGGCTCGCAGTCCATCTGGTAGTGCCACAGGGCCCCCACAGGGAGGCCACAAAGGCCTGTACCTGGACAAGATCCCCAACGACCTGAAGGCAGCTCTGTCTCCGCCCTTGGCACCACCAAGCTCGGCAACCAGCATTGCATCAGATCTCCCAGGCTATTGCTGGGATTACTCGGACCTAGCAGCGCATGCAGAGGAAGACGTTTCGAGTGATGAAGCGCCTGGCAGCTCCCGAAGCTCACACTGCTCCGAAGGCACTCCTCTGCAGGGAGGTGAAGAACGGTACACCTGCCACCCCGATCAGTACCTGCCTCGGCACTGCCCCAGTGAACCAGCAGTGTGTGCCATTGAGGACAGTGATGAAGAGCTGGCCTGACATCTGTGCAAGATGCTGGTGCTAAAGCCAGCAGTGGTGCCATACAAGGAGTGCCAGCGTGATGGTTTCTCCTCGCTGGCCTGCATTCGTGACTGCGCCCGTGGGCAGAGAGCATGACCTCTCTGAGGGAATGCTGTGTTGTCTATCTAGGGACTGCTTGCCTATGACGTTGCCTGAAAACTGTGCCTTGTGTTACCGGCCTCGCAAGCTGTGTGCATTGTCCTCATGTTTAGAGTAAAATTGAACTTGCAGAAATAAAATACAAATGCCAAATCATCGCTTTCATGTTTCATCATCCCTGCAAAAGTCTGTGAAACCCATTGGTAGTGTGAGACTACACAGAG

>AAUF21782

ATGATGGTCGTTTTGTGAGTGTCACTGTAAAAGCAACTGACCGTGGCAGCCCACCACTGGAAGGAGTCTGTTCCTTCAAAGTAGAAATAACTGATATCAATGACAACCCACCCCTATTTGACAGGCAGGAGTATAGGGAAAATGTGAAGCAAGACACCCAGGTTGGGATTCATATCTTGAGGGTGTCTGCTTCTGATGAAGACGCAGATAACAATGGTGCCATTGTCTACAATTTGACTGCACCATATGACCCTGAGCACTTGGCATATTTCTCCATCAATCCAGACTCCGGGTGGATTAGCCTGCAAAAGGCCCTTGACCGTGACCAGTATCAGCTGCGAGCCATAGCGCTGGACAAAGGTGTTCCCCAGCATCAGGCAACGGTGGAAGTGATCATTGATGTTGTTGACAGAGCCAATAATCCACCGATATGGGACCAACCTGTGTATGGTCCAATATTTATCAAGGAGAACCTTGAAGTGGGCACTCGTGTCATTGCTATCAAAGCCAGATCCGGAATCCCACAGAATCCTGATGTGTTCTACACGTTGATGAAAGGAAGTACAGAGCAGACCAACAAGAAGGACACTTTCTACTTGAATCAAAAGCTTGAGAATCGCCAAACAGTGGCAGAGCTGGTGGTCAATTATCCTCTGGACTATGAACGTATCCAGCAGTACAACCTTACTGTTCGTGTCGAGAACAACGGCATCCAGCAGTTGGCATCAGAGGCAACTGTCTACATAGTCCTTGAGGACGTCAATGATGAGATCCCTCTTTTCATAGAGCGGGAGCAAGAAACAGTCCTGGAAGGTTTACCACCAGGCACCAAAGTGACTCAGGTCCAGGCTGTTGACAAAGACGGCACTTACCCGAATAACAAGGTTTATTATGCCATAGAATCTCGAGACCATGGTGACAAGTACTTCTCCATTGATCGTGAGACTGGAGACATCTACACCAAGGTTGAGTTTGACAGAGAAGAGAAGATGGCCTACGCAATCCTGGTTCGGGCAGAGGATGGTGCTCCATCGGCACGGCCCCACATGACAGACAACCGGCCCAACTCAGTGACCAAGTACATCCGCATTGGGATCGGCGACAAGAACGACAACCCGCCGTACTTCGGCCAGGCGCTCTACGAGGCTGAGGTCAACGAGGACGAGGATGTGCAGCACACTGTCATCACTGTCACCGCCAAGGACAAGGACGAATCCTCGAGGATACGCTACGAGATCACCCAGGGGAACATAGGAGGAGCGTTTGCAGTGAAAAACGAGACTGGCGCCATCTACGTTGCCGGCCCGCTCGACTACGAGACCAGGAAAGAGTTTCGACTGCGT

>AAUF22204

CACACGCTCGTCATCGGCACGCTGGAGAACGAAGCCAACGACGCCATGGCCACATGCACCGTCCGAGTCGCCGTCGAGGACCGGAACGATGTTGCCCCACGCTTCACGAGCGTCCCGTTGCCGATCAGGCTGCAGGACACTGTGCCGCTGGGAACAATTGTGACAACAGTGGTGGCGTCCGACATGGACGGATCGGCACCCGGGAACGTCGTGCGTTACGAAATCAGCGGCCAAGGTCGGGCCCCATTCTACTTTCTCATCGACAGCACCAGTGGCGTGATAACTGTCAAGGACGACCTCAGGAAAGAGCCCGACTCCGAGTACAGGATCGAAGTTCAAGCGAACGACCTCGGCGATCCCTCGTTAGCAGCCACGGCTGTCGTCACGGTCTACGTGGAGCACATCGCCACCGTGCCCCCGGACTCCGGGCTCGGTTTCGCCGACGGATTCTACACGGTTGAAGTTCCAGAGAACGCGCTGGCGAACACCCTCGTCAAATCGTTGCCGATTATCAACAAGCCGCGAGGAAATTTCCCCGTTCAGTGCCAGATCGTCACTGGAAACGAGAAAGGGCATTTCTACATATTGGACAATGAACAGCGCGACTGTGAAGTGCGTGTCCAGAACCAGAACCTCGACTACGAAGAACAGAACAAATATCTCTTGACTGTCCACTTGAACACAATTGGCGGAGTGTTCGGTTCTTCGAGGCTGACCACCCAGGTGGCTGTGCACTTGATTGACCAGAACGACAACCGGCCGCGCTTCGTAGTGCCCCCCATGTACAGCCAGCTGACGCAGAACCGCTACCTGGCAGCGCTGTCGTCGGACGCCCCGGCCGGAACCCGCTTCATCCAAGTGATGGCTGAAGATCTCGACTCCGGTTCGAATGGCAAGATTGTGTACGACCTGTCCCCTGACAGCGACCCCGAAGGCAAGTTCAGCATTGACCCCCAGACCGGCTACGTGAGCAACGTGAAGACGTTCGAGGATCTGCAAAACGTGGAGCTGCCCCTGAAGCTCAAGGTCACAGCCAGAGACAGCCCCGACCTGACCGCCAGGGCCCTCACGGAATCCGCATACGTTTATGTAAACCTTATCCGCTACGAAAACCGGCTGGTCTTGGCAATCGGTGATGTCCTGCCGGACAAAGTACTGCAGCTCAAGGATCACATAATGAGCATCACACAGCAACAGACGCGTCTCATCGCAAGCGTCGAGAAGGTGGTCGCCTTGAAGGTGTTGAGAAATAACACCATTGCGACAGACTTCTCCGGGAGCGATGTCTGGATGTACCTAGTGGAGCCGGAAAGCCTACGAATTCTCACAACAAACGATCCTCGAGTACAAATGCTGATGAGCACCGGCAGTGCGCAGTCTTCGTTCCTGGACCACCTGTCGCGCAGCCTGGGCATCACGGCCCAGCAGCTGCGGGCCCCGTACCTGACGCCAACAGCTCCGCCCAACCTGGTGCGGCCCGTCAAGATCGCCGGCGGAGATTCGAGCGATCTGGGCGCCGCTCTCATCGTGCTGGCCTGCATCATCGCCGTGCTGGGCTTCGTCGGCATCGTCTACCACTGCTGCATGTGGTCTAGGTACGTGGCCAACAAAGAACGGATCAAGCGGATGTGCGTGGCTCCCAGATACGAACCAGTGTACGCCGATTCAAGTTTAAAAGAATACGAGACGCAGGTGCTGCAGATGAGCGTGCCGGTGGACGAGGACGGCAT

>AAUF24440

GTCACGCTTCTCTCGATCCAGGACGGCTGTGGTGCTCACAAGACCAGATCCAGGGTCTATCTCAAACTCCAGGTCTTCTCCAACAGCACTGTGTTTATCAAGAAATTTATATGCTACCTGGCCGTTTGAAGACAGAGCACTATCTGGGTCGACAGCCTGGACTTGGAACACTGAAGCTCCAATGGTGACGTTCTCATGCACGTAAGCTACCTCACCCATTCTGGGCCGAATGAAGCGCGGTATGCCATCATTTGAAGACACGTCCCCGATAATGATGAGTATCTTG

>AAUF29304

CGGAAACGCCCGAGACTTCTTGGCACGGGAATTTTACACGGCGTGCCCCCGCTGCCCGAAGCGTTTCTCTGACTTTGTGCGTGCGCCTATGGTGAGCCACTGCTTGTGATCCAAACAGTCGTCCACTACAGCTGGATGCGATCATGAAGTGTGCCCCGGTGCTCGCGCTTTTCTTCGTCGGACTCGCCTCCGCGCTCAAAGCCCCCCGACTGGAGCACGTGAACACAGAGGTCGGCTACCATGCCATCGTCAAGGAAAACAGCCGCAATGTGGAGATCATGCCCAGGATCCGGGTGCTGGACGCCAAGGTGTGCCGCTTCTTGGTGACCAACAAGCGTCACGGTGAGGCGCCCTTCGAGGTGCGCATCACGGACGAGGCGACGGGCAAGGCCGAGCTGTTCGCCAAGAAGGAGCTCAACTGCGAGAAGCACCGAAACTACAAGTTCGACATCGCCGCCGTGGGATGCAACGGGCTCGTCTCCGAAAACGTTACTGTGCACCTGACAGTGGATGACGTCAATGAGTTCGCGCCCCGCTGGGAGGAGGAGTCTTACCAAGGCAGCGTGGATGAGGGCCGGCCACCACACGAGCGTGTGCTCCGCGTGCGTGCTCTTGATGCTGACTGCACGCCCAAGAACAGTGAGATCTGCAAGTATGATATCCTGGACGCACATGTGCCCTTCTCCATAGACTCTGAAGGCACCATCTGGACCACAGAGCCCCTGGATTGGGAGGCAAGCAGCAACCACATATTCCAGGTGGTGGCGTTCGATTGTAGCATGAAACAGAGTCATCCGGTCACTGTCACTATCAAGGTCAACAGGGTCTGCAAGGTTGGATGGAAAGGTATGGAGGAGCATGTGGAGTACACACCAGGCTCTGGTCGCCGGGCCCTGTTCCCGGATGCCCAGCTGGAGCTGTGCGAAGGAGCCTGTGAACCCGAACAGCTGTCGGCTCGTCTGACTCTGGCCACCCGCCACGTGGGGAAGGGTTGTGACCGGGACACCTACTCAGTCGATTCTCAAAGGAAGCTCTGCGGGGCCAGCTCAGACAGTGTTGACCTGCTTCCAAGCCCCGGAGTCGGGGCCGAGTGGACACAAGGTCTGCCAACGGATGAAGGTCGTGAGAGTGACCAGATCTACGAATTTGATGGTGCCACAAATGCTGTAGTCATTCCCGAGTCTACTGTGTCGCACAACTTAACCAACGTTTTCACTGTGGGCTTCTGGATGCGTCACCGCGCACCCACAGCCCACAACGCCAGCCACCTCAAGGAGCATGTCCTGTGCAGCTCTGATGACCACCGCATGAGCCGGCATCACACAGCGCTCTTCATTCGCAACTGCCGGCTTATCCTGCTGCTCCGCCGGGAACCCACCCAGGAGCAGGCCAACAAATTCACACCAGCTGAGTGGCGCTGGAAGACACCCGAGGTGTGCGACGACAGGTGGCACCACTACGCAGTCTCTGTCAACTTCCCCGAGGCATCACTCTATGTCGATGGACGGCCCTTTAAGGTGACGGCTAACAACCCGGAAATTGTAGATGACTGGCCTCTTCACCAGACCAAGAACATCAACACCACCTTTGTTGTAGGAGCCTGCTGGCAAGGCAAGGACAACAAGATGGCATTCCACTTCCGTGGCTACCTGACGGGCCTGTCTGTGCTGAGGGGACGTACAGAGTCTCCTGATGTGCTCTCCTGCCTGCATCGCTGCAAGGAGTGGCTTGACATTCCCCCTGCAGATTCGCAGGCTGCAGGAACGGAGGTGACCTCTAATTCCGAGCGCAGCGAGGTCACAGTGACAGCTCGTGACCAGGACACTCTCGAAGATCTCGTGTCCCGTGTGGCCTATGTCAATTCACGTGACTTCCCCACACCTGGTCGTCGTACTGTTCATGTGGCCACTACTGTCATGTGCAGCAATGGAAAAGCACAGAAGGTACCACCTGTGGAGTCGTGGGTGACCATCTTGGCTGCTGAACAGCCAAGCATCACCATTAATGGCACACCCAACCTGGCGCGCGAATACGAGCCTTTCAAGCAGGGCATCGAGTTATTTGCCTCGGTCTTCATCTCGGTTGGCCGCGAGCATCCCACCACAACACAGGCCAGCACGGCTTCATCCTCCTCACAGGAGGATGAGGACGACGATGATGAAGAGGACAACCAGGTGTCACCGCCCGCTGCCTCCCAGCGGCCGGGACGCCTTGACTCCTGCAGCGTGCAGGTCTACCCTCCACTGAACCCAGACCACGAACACTTCCAGCTGCCCTCATACATGATGGCACATTTGGGCATCCATCATCGAGAGAGCAAAGATGGGCTCGTCATCTATGGAGCTGACAAGGTGCAGCACTATGAAAATGTGCTGCGACAGATCCTGTATTTCAACAAGAAGCCTGCCTATTACCTAAACCGAGCCTTCAAACTTGTGTGCTCCGAGCTGAATGGTCGTTTTGTCAGCAACGAATACATTCAGACGCTCACTGTCATCCACCCACGGCCTGAGTCCCCCAGCCAGCACGAAACGGCTGCACCCGCGACTGGGGCCATGCGCACCACCCCGGCCTCGGCG

>AAUF33243

GTCAGTGAAAAAGAACTGTATTCCTATATTTTGTCCAGCCTAAAGCGATTTCCTGAGATATGCACCATAAGCTGTGCCCACCTGTTGACCTGTTGATGGCAAAAAGCTGTGCCTCGTTGCCAGCAACGATGGCATATGACAGCTGGTCAGAGATATCGGGGTCCAGTGCAACAAGCCGAGCAATAAACTGGCCTCGATGAGCCCGCTCGTCGACCACAGCTGTGTACGTCGGCTGTCCAAACACTGGTGGGTTATCATTCATGTCTATCACGGTGACCCAGACACGGGCTGTAGAGCTCAGAGGTACAGGGTGGCCAGCATCCGATGCCACCACCTGTAGCCGGTGCAGGGCTTGTGTCTCCCGATCAAGAGGGGCCCGAAG

>AAUF41964

ACACTCACAAAACGACCATCATCCCCTTCACGGTCAAAAACCTTGTTTGTCCTGATTTCTCCGGTGAGTTCATCGACACTGAACTTTGTGCCCTTTTGATTAGGCTGCTGGACAATGGAGTAGCGCACTTGACCATTGTGTCCTTTGTCAAGATCTCTTGCTTTCACCTTGATGACTGAGGTGCCACTTTGAGCACCCTCTTCTACCTTAGGCGTATAGGTCTGGCATTCCTCAAATACGGGCTTGTTGTCATTGACATCCGTTATGAACACAACCACTAGGGCTGTGCTTGTGTGGGGAGTCAGAGCCCCATTTCGACAGCAGGCACCATCATCTCGTGCTGTCACATTCAACTCGTACTTGTCCTTGTCCAGGTGGATGGGGCCATTGATGAGTCGGATGACACCTGTCCTTTCTTCAATCTGGAACATGCCAGAGGTGGTGCCACTGTTGACAAATCCAAAGAGGATGTTATCTCCATCCTTGTCGCTGGCTACCACAGTGGTCACGAGAGTGCTGGGCCCAGCATTTTCATCCACGTTTGGCGTGTAAACGTCCTGGCTGAACTTCGGCGCCTCATCGTTCTTGTTTTCCGTGTATATGCGAATTGTAGCCGTGCCTGTCAGTGGTGGCTCCCCACGATCTGTAGCCCGCACTGTCAGAACATACGTGTTGTTCACATCAGCATCAAGCCGCCGATTGGAGTACACCACACCCCGGCTGTCAATGGTAAAATCTTCTTTATCAAGCGAGTAAGTAAGCTCAGCATTGCGGCCAGTGTCCATATCCGTTGCAGACACTTGAAGGATGGATGTTCCCACTGCTATGTCCTCATCAACATTATGGGCTTGATAGTCTGGCAGCTCAAATCGAGGCGCATTGTCGTTCACATCAGTTACTTTAATTGTTAGCTCAACAGAGGTTGAAAAGCCACCGGAGTCTTCAGTTGCAGTGACAATAAGAGAGTAGGATTTTGGCTGCCGTAGGTCTTCATAGTCCAGCTCTTTGGCCAACTTCACTACTCCGCTGGTGGGTCCGATGTTGNNNNNNNNNNNNNNNNGGCCCTGGGCCCGCAGAGTGTACCGGATCTCCCGATCGGCAAAAGATTTGGCCTTCACCTCGATGATATCGGAGTCTTTCTTCTGGTTCTCTGGAATGACCGCTTCATAAAAG

>AAUF42678

CTGATCCAGAAGGGATAGTGATACCGGGTCCAAACACTGGAGGTCCCGTCTTTTCGAAAGAGAAGTATGAAGCTCAAGTCAGCGAAGGAGCTTCTGTCGGCTCTGCTGTCCTAACGCTGAAGGCCACTGATCCAGAAGGGGACGANNTGTCGTACTCAATAGCGGAGGGCAACGACAAAGGCGCCTTCACCGTTGATCCTTCGAGTGGGACCATTTCTGTGGCCAAGCCTCTCAACCGCGAGGAAGTGGCCTCGTACACACTGCAAGCGAAAGCGGAAGACAAGAACAACTTGTTCAACCTGGCAACAGTGCAGATTGCTGTGACGGACATAAATGACCAGAACCCCGTGTTTGTGCAAGAGAGCTACAACTTCACCGTCGAAGAAGGAAAAGCGGGGGCCGTTGTCGGGACCGTGCAGGCCCGAGACGAAGACGTCGGCG

>AAUF46068

CTGCTCGAATACTGGTGCAAACTCGTCACGGCTGAGCACGTGCACGTGCACACGGGCTGTGGCCCAACGGCCACCAGCATCAGTGGCACGGATAGTGAGCTGGAAGTGAGGTTGTGCCTCGTAGTCAAACACAGNNNNNNNNNNNNNNNNNCCTGTGGCAGTGTCCACAACAGCCAACTGTGTGCCTTCAACGAGTGTGTAGTTAAGCCGGCCAAATGGTCCCCGGTCAGCATCCACAGCTCGTGCCACAAGAAGTGCACTGCCCATGGGCGCACCTTCCTCTACTGAAGCCACATACTCAGGCAATGGGAACCGTGGTGCATTGTCGTTCTCATCTTGCAGACGCACAAGTACACTGGCTAGTGCTGCCCGGCGACTGTCTGTAGCACGCACCACGAGACGCATCTCCGGAACAGCATCATAATCCAGTGCTGAG

>AAUF46223

GCACGTCAGAACTACACTCTTCGAGTGGTGGCCACAGACGGTCTGCATGTGGCCATGACTCAGCTTCATATAACCATCCTGGACAGCAACGATAATCCACCAATCTGCCTCAAGTCCAAGTACACGGAGCTTGTCTCGGAGAGCATTGCTGTGGGTACTGCCATCTTGACGGTTGGAGCCACAGATGCGGATGATGCCCACCATGCTCAGCTGCACTTCTACCTGTCAGGCCAAGGATCTGAGGACTTTGTTCTGGATGCCAGCAGTGGGGTGCTGCACACGACCCGGCCTCTAGACCGAGAGCAGCGGCCGCACTACTCACTGGTGGCACATGTGCGTGACGTGGCTCGTTGGGAGTGGCAATGCAACAGCAGCGTGGAGCTCTTGCTGAGTGATGTCAATGATAACCCGCCTGTGTTTGGCCAGAGCACCTACGAGGTGGCGCTACCCGAGGACACGCCTGCCGGCCGCCTGGTTGCCCAAGTGCATGCCTCTGACCGTGATTTGGGTCCAAATCGACGCCTCAGCTATTCGCTGGTTGAAGCATCAGCCAACAGCCACTTCAGCGTGGATGCACTGTCTGGTTTGGTGCGGTTGGAGCGACCACTAGACCGAGAGGAATGCGCCAGGTTCAACCTGACGGTGCAGGC

>AAUF47321

GCCCGACTGGGTGAACTCTGGAGCATTGTCATTCTCATCCTGGATTTTGATCACCACCAAAACTGATGTTCTCCGCTGGTTTGAGGGTGCACCTGGGTTGTCAACTGCAGTCACCTGCAGGTTGTAGAGTGGCTGTTTTTCCCTGTCAAGAATTGCACCTTCCTGGATACCCAGGGCTCCCGTTGTTTCATTGATGCTGAACACATCTGACCCTTCTCCGGAGACGTAATATCTCACGATACCATAACCTTGCTGTGTGTCCTTGTCAGTTGCTGTTATAAGCAGGATAGTGTGAGGGTACTGAGTAGCCTCCGACACCATTGTTACATATGAATTCTGTGAGAAAACTGGACTGTTGTCATTTATATCTTGCACATTAATGATCACTGTAGCAATTGATGATTTCTGGTTGTTTTTATCTTGACCAATGATGGCTTTCACATCGAATGCCAACACCTTTTCTGGCAGTTCTTCATAATCAAGCCTCTGGTTAAG

>AAUF49964

AAACGCTTCGATGGCATGCTGACTTGCACAGTAAGAAGGTAGCGATCCTTTTGCTCCCGGTCAACTCTCCTTTTGGTTAATATCTCGCCCTCATTGTTGCCAGTCCGCTGAATAGTGAAAACATTGTCCATGTTGCCACCCACAATGTAGTAATCAATAAAGGCATTCGTTCCAATGTCCGCATCAAAGGCTCTCACTTTGCCAACGATAGTACCAATGGGAATTTCCTCTTTTATCTTCATCACAACAGGCTCAGCATCAGTTGACCTATCAAACTCAGGTTCATTGTCGTCAACATCAAGCACTACAACTCTCAAGATCTGGTGTGCTTCTTGCGGGGGAACTCCCATGTCA

>AAUF50855

GCCTCCTTGGAGTCCCGACCATCCGGAGCTGTCCATCAGTGTTATGGAAGAGCAGCCCGTCGGCAGCGTCGTCGCGTCGTTCGTTGCCACGGACCCGGACTCAAACATTGCCAGTTATTCTATCGAACCCGAAAATCCTTACTTCGCCATTGACAAACTTTCCGGTGTGGTCACAATACACAAAAGAGTCGATTATGAGCAAGTGCAAGAATTGCGGTTCTCTGTCGTTGTGAGAGATACGGGCATTCCTCAGTTGTCAGCGGTCGCAATGGTGACGGCAACTGTGACCAACATAAACGACAACGACCCGACGTTTTCGCAGAAAGCTTACCAGGCTTCAGTCCAGGAGAACGCCCCTCAAGGCACGTTCGTGACGAAAGTTGAGGCGAAAGACATCGATGCCGGAGAATTCGGCGTCGTTTCGTACTCCTTGCTGGGAGAGAAAAGCGGAGACTTTCAAGTCAACAAAAGGGGCGAGATAAGTGTCGCGGGAATGGCCAATTTAGACAGAGAG

>AAUF51052

CGTCGTTTCGTACTCCTTGCTGGGAGAGAAAAGCGGAGACTTTCAAGTCAACAAAAGGGGCGAGATAAGTGTCGCGGGAATGGCCAATTTAGACAGAGAGATCACTCCTGCAATCACCCTTCAAGTGGTAGCCACTGACATGGGCCACGATGCAACGACGAGGAGGTCGGTCTCCGTTCCGGTGTACCTTACCCTGCTCGACGACAATGACAGTCCTCCCGTGTTCACGAAAAAGACTTACGAGGCTTCGTTCGTCGCCAACAGCCCGCTTGAGTCTGCGCAGAGCATCGTTCAGGTTTCAGCAACGGATGCGGATGAAGGGATCAACGCCGAAGTCCGCTACTCCATCGTCGCTGGAAACGAAAACGGAGTTCTCGCCGTCAACCCCAAGACTGGAATCGTGTACCCCGTGAAGAAGCTGGAGAGCAGTCGCAAGGAGTACCGCATCGGGTTGGAGGCACGCGACGGCGCCGTNNNNNNNNCGAACACGGACAACTGCGTGGTGCTCATCAGGCTGATCGAAATAAATCTGGACAAGCCACATTTCGTGACACCCTCCCTACCCAACGCCACCGTGGAAGTGCTCGAGAACCAGACCCTCTCGAGCCAAATCATAATGACTGTCGAGGCCATGGACAAGGACCACGGCGACAACGGGCGCATCTCTTACTACTTCAAAGTTGGAGACAGGAACGTCGCAGAAACGGACGAGTTTCGCATCGACGAAGTTACCGGAGAAATCCATGCCAAGGCCGTCCTCGACAGGGAAGTCAGGCCGCGCTACGAGCTGGTGCTCGTCGCTAAAGACCACGGTACTCCCGCCCCGTTTGAAACGCTACGGTTCCTCACGGTCGTCCTGAAGGATATCGACGACAATTCGCCTTTGTTTCCTCGCACACGGTCGACGAACCCTTACGTGTTTCACATCAAGGAAAACTTAGACCCCGGCTTTCCCGTTGGAAGGG

>AAUF51433

CGTCAGAAAGTATGGCTCTGACCGCCCCCTCCCAGACAGTGGTTGAATCACCGTAATGACGCCAGTCTTGTTGTTGATTGCAAAAAGTGTGATGGCTCCCATTTCAGGTTCATCCACAAAAGAGTAGCTGATTTCCGAGTTAGAACCGGCATCTTTGTCATTGGCCCTCACAGTCAGAATACTGAAGCCTGTCGGAACGTTTTCTGGCACAACAGCCGTGTAGCCCGACTGGGTGAACTCTGGAG

>AAUF52392

CGCACACGACCGGGACCGTGGCCCTGATGGCAGAGTTCCTTATCAGATCCGTGGTGGAAGTGCCCATTTCCGTGTCAATGCATCTTCTGGCACATTATCCGTGCGTACCCCGTGGCCCGGGGGAAGTGGCAACCGCCTAGAGCTGGTAGCAAGCTCAGGGCGCCGTGGCTCCCTGCTGGCCACTGCTGGAGTCAGTCTGGAGGAAGCACCAGCAACACCTGCTGCTGCATCGGCATCTCGTGGTCTTCCTGCCTGGGCTCTGGCGCTGCTGTTGCTGGCTCTGGCAGCAGTGGCTGTACTGGCTGCAGCAGTGGCACTGCTTCGTGGGCGACTGCAATCGAAGCGACCTGCTGAGCCCAGTTTGGACACATCATTT

>AAUF53075

CCACGATCACGGCCAATGATGTGGACACACACCCTGTTCTGCGTTACTCTTTGGCCGAGCCGGGTGGTACCTTTGTCGTGGACCACTTCTCCGGCCGCCTGCTTCTGGCTGACAGACTTGACTATGAGCAGCAGCGCGAGTACCGGCTACGACTGCACGTGTCGGNNNNNNNNNNNNNNNNNAAGACTCTCGTCACTGTGCGTGTTTTGGATACCAANGACCACGCACCCCGCTTCCTGCAGCCATCCTACAATGTGCTTGTTTCTGAGTCGGCTACGGTCG

>AAUF57449

GCTGGAATGACCGCTTCATAAAAGGGCACATAAAACTGAGGCTGGCGCTTGCCACCAACCACTGAGAGGCGCTCCTCACGCGTTGACTGGTACTGTCGATCTCCTACTTGGCCATTGAGGTCTTCAGCCTTGACATACAGCACATACTCCTTGTCTAGCATGAATGGTGCTGAACCCAACGTCACCACCTCACCCGAGTGCGCGTCCACGTCAAACCTGTTGCCACTCCTGTCGCGCACAATAGAGTAGCGAATATTGTGGTCCGTGTCCGGATCCCTGGCCTGCAACTTGAAGACAGGAGTGCCGGCCGGGGCATTCAACTGTACAACAGCCTGCATCGGAATCGGCCGGTTGATGAAGTAGGGGGGCTCGTCGTTCACGTCCGCTATCCGGATGATGATTCGTTGCCTCTCCTTCTCAGCTCTTCCAGGGGACCTGGCCAGCACCCAAAAGTCGATAGACTTCTCCTTATCCAGCTGCTCATAGTCCCATCTCTCCTTGACGCGCACGTCGCCGTTGGGGCTCACGTAGACCCAGCGATCGGGCTTTTCGAGCTCGAAGGTCTCGTCGACCGCCTTCTTGTCGAGCGTGAAGACAGTCTTGTTGACGTCGCGGCCCTTGGTCTCCTCGAAACGGTACGTCTTGGTAGGCCG

>AAUF7293

ACGCCTCGTGGTCGCTGCGAAGGAGCGTTAAGTGTCTTGTGCTGGCGTGATGGGTCCGCGTGGCGTGTAAGTAATGCGACCGCTAACACGGCCCCTCCAAAATATGCGTCTGTCCTCAGGCGCACGTTGTAGATGTCAGTTCCCCGGTGTCCAAGGTGCGTTCCTTGTACCTTCTTAGTTGTTCGATGTTCACAGTTTTCTCGTTTAACTTTCCAGGAATGTGAGGGATCCTGCTTATGACAGCCATGTTGGGACCAGTCAGTGCAGTTATTTCGAACGGGCCTTCGTATCTTGCATCCAATGTAGATCTTGCGCCGATTTCGAGAAGAACGAGATCTCCACACTGGAAATGCGGCGTCTGACGTCTTTCGTTGTAGCGTTTCGTCGATTCCTCCTGGCTCTTCTCAAGAGAGTTTTTCATGTCGCGGCGCCCCTTCGCCATTTCGTGTAGTCTTTGTGCGGCGTCTATTTCCTCCCCGATGGTTCCAATGTTCAGTT

>AAUF57971

CGGGACTCCAAGGAGGCGGGAACGAAGGCGGAAAATCGTTGAGGTCCACTATAGTGATCACAAGGGTGCCTAATCCAGTCTGGAGTGGCACAGCGCTGACGTCAGTCACCACGGTGGTTAGCATGACGACGGCTGCAGCTTCGCGGTCGAGCCTGGATGCCACTCTGATCTCGCCAGTGTCCGCGTCCACATCGAAGAAGGCTTTGTAAGCTTGAACTGACTCATTCACGTTTCTCCCATCTTTAGCAATGGCTGTCACAGGTTCCGCAATGCCGTACTTCAAAAATTCCGGGGAGCTCGCATCCGGATCTTTGGCCACAAGTTTGTAGAATCTGTAGCCAACTGGCGCCCCTTCGGACACTTGCGCTCTTTGTGTCGTCGGCTGAAAATATGGCATCTTGTCGTTGCTGTTCAAAATGTTCACGCTAAGCGTCG

>AAUF58805

GTGAACTGTCACTAACTGGAAGCCTGAATGTCACTGCAGCTCCTTATGGTGCCACCCGATTTGAAGTGATAATACAGGCCACTGATTTCGGGGAGCCAAGGCTTGCATCAGAAATTCCATTCATCATCCGGCTTAAACGTAAAAATGAAGGTGCACCTTATTTCTTGAATGCACCATATAAGGCAAGTGTCAACGAAAATGCCAAACCAGGAACTACGGTTCTTCAGTTAGTGGCCACTGACCCAGACGGCCCAGATGGCAACATCAGCTACTACATTGATTCTGGCGCCCGGGACAATTTTGTTCTGAACAAGCTGACAGGAATCCTCAGCACGTCGTCAAATGCAAATTTGGACAGGGAGCTGAATGGAGACTCGTACCACATTTTGGTGTATGCTGTGGATGGGGGTGTAGTACCACAGAAGGCCTTTACCACAGTGACAGTCTCTGTTCAAG

>AAUF62981

CAAAGTCTTCAAGGGTCACTGAGAATTTTAGTGTGTCTGTTACCTCCCGATCAATGTTGTTTATCACAGTGACGTCGCCGGTGCTCCGATCTACAGCAAGGAGGTCCGTTCCTTCAAGTCCGTAGTAAACTGGCGAATTTTCAGGATCCGATCCTTCCAGGCGAAATACAGATGTACCGACGGGAGTGTTTTCAGGTATCACAGCAAGGTCAATGGTTTTGGTAAACTTTGGCGGCAAATTGGCACTGGTTAGATGGACGGCCGCAGAGAGGAGCAAAAGAGCGAGCGCCATCTTTTTCTCTGTCCGGTGCGCTGCGAGTCCGACCGCTTTCGCAG

>AAUF63151

ATCCACACCAAACGTCTGACCATCACCCTCATGCAAAAAGTAGGACACCTTGCCATTGGGGCCTTCATCCAAGTCAATGGCTTTCACTTTGAGCACGAGGTCTCCAGGCTGGGCATCCACAGTGATGGCCGAGTGGTAAGGCCGGTCGACAAAGATTGGTGCTTTATCATTCTCATCCAGCACGCGCACACGCAGAAGAGCATGGGCCACCGCACGGGCAGAGCGTGCCTCCAGAGCCAGCTCGTGGAAAGCACGCTCCTCCCGATCCAGGGACGCCAGGCAACGCACAAGGCCCGAG

>AAUF63918

TTCGAGCTTAACATTTCGGTTACGGACGGCGTGTACCAGAGCTATACACAGCTGCGGCTGATGGTGGTGCCGAGCAATGAACACTCGCCCCGGTTCAGCCAGTCTGTGTATGAAGCAGTGGTGGCAGAGAACTTGCCGCCAGGTGCCCATGTGACCCGCGTGTCTGCCACGGATCCCGACCAGGGGGCGCTGGGCACGCTGACATATGCCATCCGCAGTCAAGACTGTGCACTGCACTTCAACATCAACAGCACCACAGGAGAGCTGGTGACTCGGGAAGCTTTGGACCGCGAACATCAACATCTTTACGAGGTTCCCATCTCTGCCACTGATGCGGGCGGCCGCCTGGCGTTTGCTCTTGTTCGTGTGGCTGTCACTGACATCAATGACAATGAGCCCACTTTTGGTGCTTCTGAATATGAGGCTAGCATCTGG

>AAUF57566

CGGCTCCTTGTCAGCATCTTCCACGCGCAGGCTGGCAACTACAGTGCCAGCAACAGCATCTTCAGAGACTTGCACTTGGTATAGGGTCTGCTCGAACACGGGAGGATTGTCATTTACATCCAGGACCTCCACGTACACCCAAGCTGAGGAAGACAGCAGACCGTCATCACGGGCCCTCACTGTAAAGTTGTAAAATGGCACGGCTTCTCGATCGAGAGCTACCAGAGTTGTAATAACCCCACTCTGCGGGTCAATCCGGAAGATGTCAGCAGCCGGATCAGTCTCTTCGTAAAATTCGTACGTCACCCTTCGCACTGAG

>AAUM7591

CTTGGGTGTACGTGGAGGTCCTGGATGTAAATGACAATCCTCCCGTGTTCGAGCAGACCCTATACCAAGTGCAAGTCTCTGAAGATGCTGTTGCTGGCACTGTAGTTGCCAGCCTGCGCGTGGAAGATGCTGACAAGGAGCCGGCTCCTGTTGGCTATTACGTGCTCTCCGGTGATCCTGGTCAGCAGTTTGCGGTGCGCAGTAGTGGTGATGTGTTCGTACAAAGACCGCTGGATAGGGAAGCACGTCAGAACTACACTCTTCGAGTGGTGGCCACAGACGGTCTGCATGTGGCCATGACTCAGCTTGATATAACCATCCTGGACAGCAACGATAATCCACCAATCTGCCTCAAGTCCAAGTACACGGAGCTTGTCTCGGAGAGCATTGCTGTGGGTACTGCCATCTTGACGGTTGGAGCCACGGATGCGGATGATGCCCACCATGCTCAGCTGCACTTCTACCTGTCAGGCCAAGGATCTGAGGACTTTGTTCTGGATGCCAGCAGTGGGGTGCTGCACACGACCCGGCCTCTAGACCGAGAGCAGCGGCCGCACTACTCACTGGTGGCACATGTGCGTGACGTGGCTCGTTGGGAGTGGCAATGCAACAGCAGCGTGGAGCTCTTGCTGAGTGATGTCAATGATAACCCGCCTGTGTTTGGCCAGAGCACCTACGAGGTGGCGCTACCCGAGGACACGCCTGCCGGCCGCCTGGTTGCCCAAGTGCATGCCTCTGACCGTGATTTGGGTCCAAATCGACGCCTCAGCTATTCGCTGGTTGAAGCATCAGCCAACAGCCACTTCAGCGTGGATGCACTGTCTGGTTTGGTGCGGTTGGAGCGACCACTAGACCGAGAGGAATGCGCCAGGTTCAACCTGACGGTGCAGGCCCAGGACCATGGTCAGCCTCCCCGGGCTGCCCGCGTGCCTCTTACAGTCATTGTGCAGGACATTAATGACAGCCCGCCCGAGTTCACACAGCAAGCGTACTCTGCCAGCGTCTCTGAAGCTGCCCCCGTGGGCTCGCCCGTCAGCGCTGGTGTGAGGGCTACCAGCCGAGATGCAGGCGTCAATGCTCAGATCACATACTCCCTGGTTGCAGGCAACGACATGGGACACTTCAGCATTGAGCCAAAGACAGGTGTGCTGCGGGTGGCCCGACCCTTGGACTTTGAGAGCGCGCGCAGCTACCAACTGGCAGTGGAGGCTCGCGATGGGGGCGAGCCACCCCTCAGTGCCCGCGCCTGGCTGAATGTGTCCGTACTGGACGCAAATGACAACGCGCCTGTGTTCGGAGGCCCCTACAGTGGCACTGTCGCGGAGGATGCAACCCCTGGGCAGCTTGTGCTCCAGGTGCAGGCCAGTGATGCCGACAGTGGCGGAGAGCTGCGATATGCTCTGCTGCAGCCGGGCCCTTTTACTCTGGACCCGGCCACTGGCCAGCTAAGTGTGGCTGGGCCGCTGGACCATGAGACGGTGAGCCGGTACGCACTGGAGGTGGAGTGCTGGGATGGTGGCACACCGCCACTGTCGGCTCGGGCACTGGTGCACCTGGAGGTGCTGGACGTGAATGACCATCCACCGCGCTTTGACCAGAGCAACTACACGGCCGTGGTTCATGAGGGTCGGCCAGTCGGCTGGACCGTGCTGCGCTTCTCGCTCAGTGATGCTGACTCTGCTGCTCATGGACCTCCTTTCCGGCTGGAGCTGCTGGATGGAGCTCAAGCTGGGCAGGAGGAGGAGGAACCCTTTTTCCAAGTTGAGGGCCACGAGCTGCGGTTAGCACGACCACTTCCTCCGCGCTCGCGGCATGAGCTGCGTGTGCAGGCTCACGACTCGGGCAGCCCACCTCTGGCGACAGAGGCGCATGTGACTGTGCTGGCTGTTGAGAAGAGCCGCTTCAGGCCACAGGTGCAGCCCTTGACTGTGGTTGTCTCTTCTTACTTGGACGACTTCCCGGGTGGGCTGCTAGGCCGGGTGCATGCCACGGATGAGGACCCGTACGACCGCCTCAGCTTGTCCCTCTCTGGGCCACACGCATCGCTGTTTGTCCTCGACAGGGATGATGGGACTTTGCGTGCTTTGCCAGGCCTTGATGCTGGCAGCTATGTCCTTAATGTGAGCGCTTCAGATGGCAGCACAACGCCAGCACATGCATCAGTGAATGTGCATGTTGTCGGGGTGACCGAGGAGGCTCTCAAGGCAGCTGTGGCTCTGCGCTTGGCTGGAACCACGACTGAGCGCTTCTTATCTACGGACCGGCGGCCATTGCTGCGAGCTTTGCGAACAGCTCTCAGCGTGCGTTTGCGGGACCTGTTGCTGGTGAGCATACAGCCAGCACCAGCGGAGGCCTCAGGAGGGAGCGAGCAGCTGGACTTGTTGTTGGCAGTGCAGCCTGAAGGAGATCCAGTGCCAGCTAGCACAGTGGCTTCCCGGCTTCACGAGCGCCATGTAGCCCTCGAGGCAGCTACTGGTCTACGTGTTCACATATTGCCCCAGGGGGACCGCTGCCCTTCCCTGCACTGTGTGCACGGCGAGTGTCAGGACCGGCTGGTGCTGGACACCAGCGACGCTGTTGTCCTTGCTGGTGGTGGCCACAGTTTCGTATCACCACGACACAGCCGCCGGACGGCATGTGTCTGCAACCCTGGTTTTGGCGGTGATGCATGCGAAACAGCAGTGGATGAATGTGCCCAGCAGCCCTGCCCTGCAGGGCGCCTCTGCGTGCCAGACGCGTCTCCACTCGGCCACAGTTGCCAATGTCCATCTGGTCGCACTGGTTCTGAATGCGACACCCCTTGCCAGGAACCCAGCTGCTATGAAGAAAAGCGGCCCATCTCGTTTGGTGGCCAGAGCTATGCGCTCTACGTGCTGTCACAGCCCTTGGACCGACGCCTCTCGTTCTCTGTGATGCTGCGCACAATCCATCCCAGTGGCACTCTGCTGCACACATCGGGACCCCGAGACTACGCCATCCTTGAGGTGTCGGACGGGCACGTGCAGTACCGCTTCGACTGTGGCAGCGGTGAGGGTTTGGTGCGTGTGACTGGCCGTCGGGTGGATGACGGTGTCTGGCATGCACTGCGTCTCGAGCGTCGTGGCAGCAATGCGCGGCTCGCTGTCGACGTGCATTACCAAGCTTCTGGTGCCGCGCCTGGGCCACACGATGTTCTCAACCTGGAGGGCCGTGAGCTGCACCTGGGCGGGGCACCTGCTGTGGCAGGCTTGGTCGGCTGTCTGGATGATGCGCAGGTGGGCGGCCAGCCACTACCGCTGCATCTGCGACCTGCTGGCCACGCGCAGCTGCGCCGGCTGGCCAACGTCCAGTTTTCTTGCCACCTCGACCCATGTGGCAGCCAGCCCTGCCTCAATGGTGCAACCTGCCGGCCGCTTCTCACCACCGCTGGATACTCGTGCACTTGCCCGGCGCACTTTCAGGGTCCCCAGTGCAGAGAGGCAGTAGCTGAGAGCCGATGCACTGACGAAGCATGCGAGCCAGCCAGCTGCCAGCCCAACCTGTGCCTCCATGGGGGCCTGTGCCAGCCAGGTGGACACTGCCACTGCCCAACTCCTTATCAGGGCAACCGATGTGAGCTGGTGGAGGCATGTACGGCGTGCGAGAACGGGGAGAGCTGTGTGCGGCTGCCAGATGGCTTCCAGTGTGGCTGTTTGGGGGATGCATCGTGTGATGCCACTTTCGAGGGCCTGCCCCTCGCTTGGCCTCTGGGAGGTGCTGCTGCTGCTGTGCTGCTTCTGCTCCTGCTTCTGGCCATTGTTTGCTGCTGCCGCCAATGCTGTCGTTGCCGCCACCGCCACGAACATGTTGACAGCCATCAGTGCAGCAATCCTGTCACTGCCAAGAACTGTGTCCTGGCCACCACAAACATTAGGCCAAAGATAAGCAACCTGGAGCAGCGGCCAGCTTCGTACACGACGACTGGTGTGAGAGAGATCACACTGAACAACTTTGACACGGTGCGCAGCTATGGAAGTGCAGCTGATGACCTTGAGTCACGCTTTCAGCCCAATGACCTGCGCTGCCACAATCTGGCTCGCAGTCCATCTGGTAGTGCCACAGGGCCCCCACAGGGAGGCCACAAAGGCCTGTACCTGGACAAGATCCCCAACGACCTGAAGGCAGCTCTGTCTCCGCCCTTGGCACCACCAAGCTCGGCAACCAGCATTGCATCAGATCTCCCAGGCTATTGCTGGGATTACTCGGACCTAGCAGCGCATGCAGAGGAAGACGTTTCGAGTGATGAAGCGCCTGGCAGCTCCCGAAGCTCACACTGCTCCGAAGGCACTCCTCTGCAGGGAGGTGAAGAACGGTACACCTGCCACCCCGATCAGTACCTGCCTCGGCACTGCCCCAGTGAACCAGCAGTGTGTGCCATTGAGGACAGTGATGAAGAGCTGGCCTGACATCTGTGCAAGATGCTGGTGCTAAAGCCAGCAGTGGTGCCATACAAGGAGTGCCAGCGTGATGGTTTCTCCTCGCTGGCCTGCATTCGTGACTGCGCCCGTGGGCAGAGAGCATGACCTCTCTGAGGGAATGCTGTGTTGTCTATCTAGGGACTGCTTGCCTATGACGTTGCCTGAAAACTGTGCCTTGTGTTACCGGCCTCGCAAGCTGTGTGCATTGTCCTCATGTTTAGAGTAAAATTGAACTTGCAGAAATAAAATACAAATGCCAAATCATCGCTTTCATGTTTCATCATCTCTGCAAAAGTCTGTGAAACCCATTGGTAGTATGAGACTACACAGAGCTGGCGCTTCTTTGTCCACTATTTGTAATAAAAGAAGCAAAAGCTGTCATGCCCATGGATCAGAGTGTTGTGACAACTTTGTAGAGCTTCTCATCAG

>AAUM7835

GTGTCTGTGCAGGCTCAGGATTGTGGTGACCCACCGTTCAGTAGTGTGGCGAGCTTAATCGTCGAAGTACAGGATGTCAACGAGAATCTGCATTCACCAGTCTTTCCTGAACAGGTGTCCAGTGTCAGCGTGGATGAGAACCAGCCTCCAGGAACATTTGTTACCACCCTGGCAGCTACAGATGAAGACGGTGTCACATACGCCATCACAAGTGGCAACGGCCTGGGCCTTTTCAGCGTTGATGATAATGGTACCATTCGGAGCCGGGTGTCACTGGATCGCGAGACCTGCGCTCACTACTGGCTCACTGTGGTGGCTCGTGACCTCAGCCCTGTGCCCCTAGAGGCTCGCCTTGACCTTCATGTGGCTGTGAATGATGTTAACGATCAGGTTCCCCTCACGGAACAGCCAGCCTACAATGTGACGGTACCAGAAGACGCCCCGCCAGGCACGTCTGTGCTTACAATGGCTGCCCAGGATGAAGACTCCCCAACAGTGCACTTCAGGCTCGCAGATGATGCAGCCGGTCGCTTTGCCATCGATAGTCGCTCGGGTGTCATCACTACTGCCAAAGTGTTAGACCGTGAAGTGAACGAGCGGCACGTGCTTGAGGTGGTGATCAGTGATGGCGGCCAGCCACCGCTCTCGTCCACCACCCAGGTGCTCATCACACTCACCGATGTCAACGATCATGAGCCACAGTTCACTCAGCCTTTGTACCGCTTCCGTGTCCTTCCAGCAAATGGCCAGCAGGAACCAGCACCTCTGTGCCAGGTGTTGGCTCTGGACCCTGATGATGTGCAAGGCCATCTCAGCTACTCCATGCCCAGTCCTGGGCAGTTCTCGATTGACCCTGTCACTGGCCTGCTCCATGCACATGTGTCTCTGCTTGATGGGGACACCCACGAGCTCACAGTACAGGCATCTGACTCTGGAAGCTGGCCAGCCAGTGCACGTGTGCAGCTACATGTGGTGTCGCGGCCGGTTGCATCACAAAATGCGCCAGTCGTGCACGAGGTGGAGGTGTCACCCACACTGGAGACGGACCCTGTGGGCCACCTGGTAGCCTTTGTGCGTGCATCTGACCCTGATGGGGATCACCTGTGGTACAGCCTTATAGGTGGGAATGATGAAGGAAAGTTTATGATTGACTGTGAGCTTG

>AAUM9856

CTGGAACCTTGGTGGGTTGTCATTGATGTCACTAACAACAACAGTAAGTGTTGCAGTAGCTGTCCGTGATGGTACACCATCGTCAATAGCCAAGATCTTGACTTGGTGCCTTGGGATGTCTTCTCTGTCCAGTGTACGCTGAATTTCAACAACACCATTGGGATTAATCTTGAACTGCCTCTTTTTATCAGATGATCGGTCAATCATGTAGCTGACACGTGACTTTCCACCTTGGTCGGCATCAGTGGCCTTAAATGTTGCCAAACTTGAACCAACGGTGGAATTCTCAGGAACAGACACTTCAATGTTGGGTCTCTCAAAATCAGGTTTGTTGTCATTTATGTCTTTAACCCTAACTTTGACCTTTGCGTAGTCCGCATGATAGGGATCTGTTGATTCCCCACCATTGTCGCTCACTTGAATGGTGATGTTGAACCCAAAACGCTGCTGCAAATCTTCATAATCTAAAGGCTTGGCAATTTTTAAAGAACCAGTTCCATCAGAATTGGTGACCATAGTGAATTTATCAGAGCCAAACGTGTTGTCAATAACTTTGTAGCTAAACCGGTTAGTTTCTAGTAGATCTCCATCATTGACTGATACCACTAAGATGGGGTTTTCAG

>AAUM11558

CTTGTCGCTGGCTACCACAGTGGTCACGAGAGTGCTGGGCCCAGCATTTTCATCCACGTTTGGCGTGTAAACGTCCTGGCTGAACTTCGGCGCCTCATCGTTCTTGTTTTCCGTGTATATGCGAATTGTAGCCGTGCCTGTCAGTGGTGGCTCCCCACGATCTGTAGCCCGCACTGTCAGAACATACGTGTTGTTCACATCAGCATCAAGCCGCCGATTGGAGTACACCACACCCCGGCTGTCAATGGTAAAATCTTCTTTATCAAGCGAGTAAGTAAGCTCAGCATTGCGGCCAGTGTCCATATCCGTTGCAGACACTTGAAGGATGGATGTTCCCACTGCTATGTCCTCATCAACATTATGGGCTTGATAGTCTGGCAGCTCAAATCGAGGCGCATTGTCGTTCACATCAGTTACTTTAATTGTTAGCTCAACAGAGGTTGAAAAGCCACCGGAGTCTTCAGTTGCAGTGACAATAAGAGAGTAGGATTTTGGCTGCCGTAGGTCTTCATAGTCCAGCTCTTTGGCCAACTTCACTACTCCGCTGGTGGGTCCGATGTTGAAGGTGCCAGCTCCCTTGCCCTGGGCCCGCAGAGTGTACCGGATCTCCCGATCGGCAAAAGATTTGGCCTTCACCTCGATGATATCGGAGTCTTTCTTCTGGTTCTCTGGAATGACCGCTTCATAAAAGGGCACATAAAACTGAGGCTGGCGCTTGCCACCAACCACTGAGAGGCGCTCCTCACGCGTTGACTGGTACTGTCGATCTCCTACTTGGCCATTGAGGTCTTCAGCCTTGACATACAGCACATACTCCTTGTCTAGCATGAATGGTGCTGAACCCAACGTCAC

>AAUM24752

CGATGACGAGGGCGAGTATGCTCGTATCAGCTACTCCGTCTACCACGTGTCCAACAACGGTCGTGACAGGTTCCGCATCGACCCCAACACTGGCGTGGTGCAAGTGATCGGAAGGGTCTCCTCCGGGGAGCAGTACAGCATTACTGTGCAGGCGACGGACTCCGGCGGAAGGTTCAGTCAAGGCATTTTGGATGTGATAGTGATACCGGGTCCGAACACTGGAGGTCCCGTCTTTTCGAAAGAGAAGTATGAAGCTCAAGTCAGCGAAGG

>AAUM27336

CTCGATGGTGGTCATGTATGAAGGTTGGCCAAAAACAGGCGGAAGATCGTTGACATCTTTGACGTGGATAAGAACTGTGGTGTTGCCTTCATGAAAGCCATCCGATGCCACCAGCGTCAAGTAAAACTCTTTCCTGGTCTCGTAGTCGAGCGGGCCGGCAACGTAGATGGCGCCAGTCTCGTTTTTCACTGCAAACGCTCCTCCTATGTTCCCCTGGGTGATCTCGTAGCGTATCCTCGAGGATTCGTCCTTGTCCTTGGCGGTGACAGTGATGACAGTGTGCTGCACATCCTCGTCCTCGTTGACCTCAGCCTCGTAGAGCGCCTGGCCGAAGTACGGCGGGTTGTCGTTCTTGTCGCCGATCCCAATGCGGATGTACTTGGTCACTGAGTTGGGCCGGTTGTCTGTCATGTGGGGCCGTGCCGATGGAGCACCATCCTCTGCCCGAACCAGGATTGCGTAGGCCATCTTCTCTTCTCTGTCAAACTCAACCTTGGTGTAGATGTCTCCAGTCTCACGATCAATGGAGAAGTACTTGTCACCATGGTCTCGAGATTCTATGGCATAATAAACCTTGTTATTCGGGTAAGTGCCGTCTTTGTCAACAGCCTGGACCTGAG

>AAUM27310

CGACAAGGATGGAGATAACATCCTCTTTGGATTTGTCAACAGTGGCACCACCTCTGGCATGTTCCAGATTGAAGAAAGGACAGGTGTCATCCGACTCATCAATGGCCCCATCCACCTGGACAAGGACAAGTACGAGTTGAATGTGACAGCACGAGATGATGGTGCCTGCTGTCGAAATGGGGCTCTGACTCCCCACACAAGCACAGCCCTAGTTGTTGTGTTCATAACGGATGTCAATGACAACAAGCCCGTATTTGAGGAATGCCAGACCTATACGCCTAAGGTAGAAGAGGGTGCTCAAAGTGGCACCTCAGTCATCAAGGTGAAAGCAAGAGACCTTGACAAAGGACACAATGGTCAAGTGCGCTACTCCATTGTCCAGCAGCCTAATCAAAAGGGCACAAAGTTCAGTGTCGATGAACTCACCGGAGAAATCAGGACAAACAAGGTTTTTGACCGTGAAGGGGATGATGGTCGTTTTGTGAGTGTCACTGTAAAAGCAACTGACCGTGGCAGCCCACCACTGGAAGGAGTCTGTTCCTTCAAAGTAGAAATAACTGATATCAATGACAACCCACCCCTATTTGACAGGCAGGAGTATAGGGAAAATGTGAAGCAAGACACCCAGGTTGGGATTCATATCTTGAGGGTGTCTGCTTCTGATGAAGACGCAGATAACAATGGTGCCATTGTCTACAATTTGACTGCACCATATGACCCTGAGCACTTGGCATATTTCTCCATCAATCCAGACTCCGGGTGGATTAGCCTGCAAAAGGCCCTTGACCGTGACCAGTATCAGCTGCGAGCCATAGCGCTGGACAAAGGTGTTCCCCAGCATCAGGCAACGGTGGAAGTGATCATTGATGTTGTTGACAGAGCCAATAATCCACCGATATGGGACCAACCTGTGTATGGTCCAATATTTATCAAGGAGAACCTTGAAGTGGGCACTCGTGTCATTGCTATCAAAGCCAGATCCGGAATCCCACAGAATCCTGATGTGTTCTACACATTGATGAAAGGAAGTACAGAGCAGACCAACAAGAAGGACACTTTCTACTTGAATCAAAAGCTTGAGAATCGCCAAACAGTGGCAGAGCTGGTGGTCAATTATCCTCTGGACTATGAACGTATCCAGCAGTACAACCTTACTGTTCGTGTCGAGAACAACGGCATCCAGCAGTTGGCATCAGAGGCAACTGTCTACATAGTCCTTGAGGACGTCAATGATGAGATCCCACTTTTCATAGAGCGGGAGCAAGAAACAGTCCTGGAAGGTTTACCACCAGGCACCAAAGTGACTCAGGTCCAGGCTGTTGACAAAGACGGCACTTACCCGAATAACAAGGTTTATTATGCCATAGAATCTCGAGACCATGGTGACAAGTACTTCTCCATTGA

>AAUM28079

CTCGTAGTCAAACACAGTGGCTGATGTGAGGCCACCTGTGGCAGTGTCCACAACAGCCAACTGTGTGCCTTCAACGAGTGTGTAGTTAAGCCGGCCAAATGGTCCCCGGTCAGCATCCACAGCTCGTGCCACAAGAAGTGCACTGCCCACGGGCGCACCTTCCTCTACTGAAGCCACATACTCAGGCAATGGGAACCGTGGTGCATTGTCGTTCTCATCTTGCAGACGCACAAGTACACTGGCTAGTGCTGCCCGGCGACTGTCTGTAGCACGCACCACGAGACGCATCTCCGGAACAGCATCATAATCCAGTGCTGAGGCCACAACCAAGTCACCAGCAACGTCAAGCCGGAAGCAGCCAAGCCGG

>AAUM29386

GCTGAACCCAACGTCACCACCTCACCCGAGTGCGCGTCCACGTCAAACCTGTTGCCACTCCTGTCGCGCACAATAGAGTAGCGAATATTGTGGTCCGTGTCCGGATCCCTGGCCTGCAACTTGAAGACAGGAGTGCCGGCCGGGGCATTCAACTGTACAACAGCCTGCATCGGAATCGGCCGGTTGATGAAGTAGGGGGGCTCGTCGTTCACGTCCGCTATCCGGATGATGATTCGTTGCCGCTCCTTCTCAGCTCTTCCAGGGGACCTGGCCAGCACCCAAAAGTCGATAGACTTCTCCTTATCCAGCTGCTCATAGTCCCATCTCTCCTTGACGCGCACGTCGCCGTTGGGGCTCACGTAGACCCAGCGATCGGGCTTTTCGAGCTCGAAGGTCTCGTCGACCGCCTTCTTGTCGAGCGTGAAGACAGTCTTG

>AAUM31759

CAGATGTCAACGATAATGCTCCCACATTCCCGACTGCTGGTTACACAAGCACAGTCAGTGAAGACGCCCTCATTGGAACAAGCGTTGTGCACATTTCAGCAACAGACAGCGATCTTGGCCTAAACGGTCAGATTCGTTACACTTTCCTTGGAGGGAATGATGGAAGCGGTGCCTTTGGAGTTGATCCAACATCAGGAATTATTCGGACAAACAGGGTTCTCGATCGTGAGACATTGGCTGTGTATCACCTTGTTGCCTTTGCTGTCGACCGTGGGTCACCATCACTTTCAGCTTCCGTGCCTGTGGTCGTTTATATCGAAGACGTCAACGATAGTCCCCCACGTTTTTCGGCAGATCGAATTCGACTGTTTGTCCCAGAAAATAGCCCGATTGGTTCTATTGCAGGAGAAATAGAAGCACATGACCCAGATGAAGGACCAAATGCTATCATACAGTATGCCATCGTTGGCGGCCCCGATGCCGATGCTTTTTCACTTGTAGCAAGGCCAGGAGAACCTGCTGAGATTGTTACAAGGACAGACCTGGATTATGAATCTCCCCACAAGAAGTACACACTGATTGTGCGTGCTTCTTCACCCCCGCTCCGAAATGATGTTGAAGTTGAGGTGTGGGTCACAGACGTCAACGACAATGCTCCTGTTCTCAAGGATTTTAGCATCGTGTTCAACAACTACCAGCATCATTTCCCCGTGGGTTCAATTGGACGTGTGCCTGCTTGGGATGCTGATGTCGCTGATCAGCTGCGCTATCGATTTGTCTCTGGCAATAATGCGAACCTTCTCATGCTCAACGAAACATCTGGTGACATCCGTCTAAGCCCAAGCCTTAACTCTAACGTTCACATCAATGCTGACATGGAAGTCAGCGTATTTGATGGTATCAACGAGGTGTCAGCCATGTGCCACCTCTCAGTGAGGCTTGTATCGGAGGCTATGTTGTTCAACAGTGTCACTGTCCGTCTGGGAGGCATCACCAAGGAACAGTTCCTGTCACCGCTTTATGACCAATTTGTCAGCGGTCTTGCAGCCATCGTGCCGTGTGCGAAGGAAAGTGTCTTCATATTTAGCATCCAGGACGACACCGACGTTGACAGCACAGTTCTCAACATCAGCTTTTCAGCACTAAAAGGAAGCGAGGATGACTTCTTTCGACCGCAGTACCTCCAGGAAAGGGTCTACCTAAATAGAGCCATACTCGCCAAACTCACTAATGTGCATGTACTGCCCTTTGATGACAACCTGTGTGTCCGTGAGCCCTGCATCAACTTTGAGGAATGCCTGTCGGTGCTCAAGTTTGGAAACGCGTCCTCGTTCGTCGCATCAGACACAGTTCTTTTCCGACCCATCTACCCTGTCAACACATTTGCCTGCCGCTGCCCAGTTGGCTTTACGGGTATGAAAAGGAAGTATGACTGCGACATTGAGATAAACCTGTGTTATTCAAATCCTTGTGGCCAGAACGGGACCTGTGTTCGACAAGAGTCGGGATACACTTGCATCTGCCAGGAGGGCTTTACAGGGACAAACTGTGAAGTCAAAGTCAAGAC

>AAUM32407

GCTTTCGTTGGTTCGGATTCTCGTAGTCCAGTGGTCCTTTGACAATGCGAATGCTCACAGATGTTGAACCGGTTGCGATGGTGGGCTCCACGGAAAACATCCCCGATGAATCGACCAGATTTATATTAAAAACGGAGTTTGAACCCGTATCCGTGTCCTGGACGAACATGTCCAGGTTGGGCAGCGGGCTTCCGGGTGGGATGTTCTCATTGACGCTGACGCTGTAACTGGGCCGGCTGAATGTTGGTGCCTCGTCGTTCACGTCCTTCCCCGTGATGGACAGCGCGGCGGTCGTCG

>AAUM32580

CCTTGCAGACCCTGTTGACCTTGATAGTGACAGTGACCGGATGACTCTGTTTCATGCTACAATCGAACGCCACCACCTGGAATATGTGGTTGCTGCTTGCCTCCCAATCCAGGGGCTCTGTGGTCCAGATGGTGCCTTCAGAGTCTATGGAGAAGGGCACATGTGCGTCCAGGATGTCATACTTGCAGATCTCACTGTTCTTGGGCGTGCAGTCAGCATCAAGAGCACGCACGCGGAGCACACGCTCGTGTGGCGGCCGGCCCTCATCCACGCTGCCTTGGTAAGACTCCTCCTCCCAGCGGGGCGCAAACTCATTGACGTCATCCACTGTGAGGTGCACAGTAACGTTTTCGGAGACAAGCCCGTTGCATCCCA

>AAUM34621

ATTATCATTATCATCAAGGATGGTAATTTCAACTGTTGTTGATGCTGATTTCCGGTCAGTGACTGGACCTGAGTCGCTTGCCTGGACAATAAGCGAGTAGAAGGAAATGATCTCTCTGTCGAGAGATGTTTGCGTTGTAATGATGCCACTTTTTGAGTCTATTCCAAAAGCACTGTTTGTACCAACGGGATTAAGAATTGAGTAGTCAATGTTGGCATTCGAACCAGAGTCCTGATCAGTTGCCCTGACTGTGACAACAGTGTAACCAATTGGCACAGACTCTCTGATCGAGGTTTCATAAGTTGACTGCTCAAACATGGGAGCATGGTCATTTTCATCAAGTACATTCACCTGCAAAGTGCTGCTAGCAGTTACGGGTGGATGTGCGCTACCATCGATGGCAATAACTCTT

>AAUM35474

AGCTGCCTGCTGCGACACAGTAGCAATACAGTCAACCTTCTCAAATTGGGGCCTGTGGTCATTGACATCTCTCAATGTCACCATGAGAGCCATCTCTGCCTGCCGCTGGTAAGGTGCACCCCAGTCAGTGGCACGAATGCGTAGCGTGTAGCTGCGGCGCATTGTCTCATAGTCCAACACCTCAGTCGTAGACACCTCACCGCTAAACGGATCAATGCTAAAAGGCACTGTGTTCAGGTTGACCAAGCTGTAGCTTACATAGCCATTTTCTGCTTCATCCTCATCGTGGGCATGAACAGTGAACACCTTGGTCCCCACTGGTTTGTTTTCCTCGAGTAAAATCTCTGCGATGCTGGGATCAAAGACTGGGGCGTGGTCATTGAAGTCAAGTACTCGGATGGTGACTATGGCGACCGCCTTGTGACGCGCACCTCTGGCTGCACTGTTGGTGACACTAACTTCCAAAGAGTAATTAGCCCTGGCTTCATGGTCAAGCCAGTGGGCTATACTGAGCCTTCCAG

>AAUM35920

CTCGTGTAGACCAGGTAGTCGGGGTTGCTCGTGGCTTTCACCACTAGTTCAAACAGGTCCTTGGATTCGCGGTCGAACGAGGCGTTCGAGTAGATGGTACCCTGCATCTTGTCTATCGTGAACTGTGTGTCCCAATTTCCATCGATAATGTAGTAATAGACCATGGCATTTTCTCCGACGTCTTGGTCAATCGCCGTTACCCTTCCAACGGGAAAGCCGGGGTCTAAGTTTTCCTTGATGTGAAACACGTAAGGGTTCGTCGACCGTGTGCGAGGAAACAAAGGCGAATTGTCGTCGATATCCTTCAGGACGACCGTGAGGAACCGTAGCGTTTCAAACGGGGCGGGAGTACCGTGGTCTTTAGCGACGAGCACCAGCTCGTAGCGCGGCCTGACTTCCCTGTCGAGGACGGCCTTGGCATGGATTTCTCCGGTAACTTCGTCGATGCGAAACTCGTCCGNNNNNNNNNNNNNNNNNATTCCAACTTTGAAGTAGTAAGAGATGCGCCCGTTGTCGCCGTGGTCCTTGTCCATGGCCTCGACAGTCATTATGATTTGGCTCGAGAGGGTCTGGTTCTCGAGCACTTCCACGGTGGCGTTGGGTAGGGAGGGTGTCACGAAATGTGGCTTGTCCAGATTTATTTCGATCAGCCTGATGAGCACCACGCAGTTGTCCGTGTTCGGTCCAGTGCCGGCGCCGTCGCGTGCCTCCAACCCGATGCGGTACTCCTTGCGACTGCTCTCCAGCTTCTTCACGGGGTACACGATTCCAGTCTTGGGGTTGACGGCGAGAACTCCGTTTTCGTTTCCAGCGACGATGGAGTAGCGGACTTCGGCGTTGATCCCTTCATCCGCATCCGTTGCCGAAACCTGAACGATGCTCTGCGCAGACTCCAGCGGGCTGTTGGCGACGAACGAAGCCTCGTAAGTCTTTTTCGTGAACACGGGAGGACTGTCATTGTCGTCGAGCAGGGTAAGGTACACCGGAACGGAGACCGACCTCCTCGTCGTTGCATCGTGGCCCATGTCAGTGGCTACCACTTGAAGGGTGATTGCAGGAGTGATCTCTCTGTCTAAATTGGCCATTCCCGCGACACTTATCTCGCCCCTTTTGTTGACTTGAAAGTCTCCGCTTTTCTCTCCCAGCAAGGAGTACGAAACGACGCCGAATTCTCCGGCATCGATGTCTTTCGCCTCAACTTTCGTCACGAACGTGCCTTGAGGGGCGTTCTCCTGGACTGAAGCCTGGTAAGCTTTCTGCGAAAACGTCGGGTCGTTGTCGTTTATGTTGGTCACAGTTGCCGTCACCATTGCGACCGCTGACAACTGAGGAATGCCCGTATCTCTCACAACGACAGAGAACCGCAATTCTTGCACTTGCTCATAATCGACTCTTTTGTGTATTGTGACCAGACCGGAAAGTTTGTCAATGGCGAAGTAAGGATTTTCGGGTTCGATAGAATAACTGGCAATGTTTGAGTCCGGGTCCGTGGCAACGAACGACGCGACGACGCTG

>AAUM37237

CATGGTACCGTGAGCGAAGCGGCCCCCGCGGGTAGTGCAGTTCTCAGGTACAGCCAGCCTCTGGTGGTGGTGGCTGAGGATGAAGACACCGGCCTCAATGCACAGCTGTCATTTTCCATCGTAGAGGCCTGGGCCCGCAGGCTGTTCCGCATCGACGCCAACACAGGGGCCCTGAGCCTGGTGCAGCCCCTCGACCGGGAGTTGCAGGCCGAGTACAACTTCACTGTGGAGGTGTCGGACTGCGGGCAGCCACGACTGTCAGCTCAGCAGCCCGCCATGGTGAGCATTCGCGTGTCGGATGTCAACGACAGCCCACCCCGCTTCGAGCATGAGCAGTACAATGCCAGCCTGCTGCTGCCAACGTACGCCGGCGTGCGCGTGGTGCAAGTAGCCGCCCATGATCCGGACCTGGAGGGGCCCAGCTTACGGTACACCTTGATGGCTGGGGACCACGAGGGGCATTTTGAGGTGCGTCCGGACACCGGTGAAGTGGTCGTGAGGGAACCAAGTGGCCTGCAAGGGCTCTATCGGCTGCTAATTTCAGCCAGTGACGGCCGGTCTGAGACAGCCACCTTGGTGCTGGTGACTGTGGGCCGGGCCAACACAGGCCACCTACGCTTTGTGCGACCACTATGGGAGGCAGCCGTGCGCGAGAACGAGCCGGCAGAGCAGCG

>AAUM37991

CGCGAGAACGAGCCGGCAGAGCAGCGGGTGACTTTGCTGTCCGTGCTTGGAACGGCGGTCGGGGAGCCCGTGCGGTTCTCCTTGCTGACTCCGAGTGACCACTTTGCAGTGAGCACCTACTCAGGCCTTGTGCGTTGCCTGGCGCCCCTGGATCGGGAGGAGCGTGCCTTCCACGAGCTGGCTCTGGAGGCACGCTCTGCCCGTGCGGTGGCCCATGCTCTTCTGCGTGTGCGCGTGCTGGATGAGAATGATAAAGCACCAATCTTTGTCGACCGGCCTTACCACTCGGCCATCACTGTGGATGCCCAGCCTGGAGACCTCGTGCTCAAAGTGAAAGCCATTGACTTGGATGAAGGCCCCAATGGCAAGGTGTCCTACTTTTTGCATGAGGGTGATGGTCAGACGTTTGGTGTGGATGAGCAGTCAGGTGAAATCCGGCTGCTCCGAGCTCCTAGTCGTCGGGACGAACTTGCCCTGGTGCTAGTGGCTCGTGACCATGGGAGTCCTCCTCTCGAGAGCCATGCTAGTGTGGCTGTGAAGGTGACAGATCGAGCTATGCCCACTTTTGGGCAGCATTTCTACACTGGATCTGTGGCCGAGGATGCTGCACCAGGGACTGCACTGCTAACAGTGCAAGCCGAGGGTGCCCACCCACTCGTCTTCAGCTTGGCCAGTGACCAGTTTGCCATTGACTGCGCTACCGGGGTGCTGTCCCTCGTCGAGTCGCTGGACTACGAG

>AAUM38288

GTTGACGTCCAGCACGCTGATCTCGACCTGAAGAAGCGTGTTGTCTTTGGGGTCGAAGCGGCTCACGGGCCGTGGCTCGTGGAAACAGTCGTCCGAGGCGCGCACCACCAGCGTGTAGTTGGCCTGCTCCTCGCGATCCAGGGGCTCGGTGGCTGACAGCTGGTGCGTGAAGATGTCCAACACGAAGCGGCCCTTCTCGTTGCCCCCGACTATGTAGTAGCAAGGTATTGGCTTCAGGATGCTGAGGTCGTCATCCCGGTCGATCGTGCTTATCAGCTT

>AAUM38549

CACTGTTGTCTTATGGGGCTGAAGAGCCCCATTCCGATCAACAGCAGCCACATTGAATGTAATGACTGAAGCTGCATTGTAATCCAAAGGGCTGTTGACTGTTATCTGACCTGTAACTCCATTTATGCGAAAAGCATTCTTATAGTTATACGTGATAGTTGAAGTGACCAAGGATCCAGTCTTGTCCCTTGCTATGATAGGCTCCTCAATGTCGTACCGCACCTTTGCCCCAACATCAGGATCAACAGCAGTTACGTTCAATACCTCACGTCCAACTGGTAGGCTCTCAGTGACCCAGTGTACATAACTGTCCTTTGCAAAAACAGGTGGCTTGTTGTTGACATCTTGAACAGTGACTGTCACTGTGGTAAAGGCCTTCTGTGGTACTACACCCCCATCCACAGCATACACCAAAATGTGGTACGAGTCTCCATTCAGCTCCCTGTCCAAATTTGCATTTGACGACGTGCTGAGGATTCCTGTCAGCTTGTTCAGAACAAAATTGTCCCGGGCGCCAGAATCAATGTAGTAGCTGATGTTGC

>AAUM41639

CAGCCCTGTTTCGGATAGACAGTGACACTGGCGTCCTCACCACTACGGGGCTGTTTGATCGTGAAAAGCGTGCCAGCTATGCCTTCGAAGTGCGGGCTTCAGACAGTGGCCGCTACCAGGCGCACTGGGCCCATGCTCGAGTGCAAGTGACGGTGGTGGACGTCAATGACAACTGCCCCCGTTTCCTGGAGTTCCCGTACGTGGCCCATGTCAGCCCCCATGCACCCCTTGGTTCTCAGGTCGCTTTGGTACAGGCCCATGACGGAGATGACGGCCCCAACGCGGATGTATTTTACACAACACCAAGTGGAGGCAAGCTGCACCTTGATGCTGACACGGGCCTGGTGACAGTGGCTGCCAGCCTGGTGGCTGACAGTGGTCGGCTGCTGCGCCTCGACCTGGTGGCTCATGACCGTGGCCGACCC

>AAUM45841

TGTACAGCCGTAGTGTTTCAAAGTCTAGCGATGCAGCCAGCCGAATAAGGCCAGTGTCAGGATCGATGTCGAAGACACCAGCATGCTGGCCATCCAGAGAGTATTGGACCCTGCCATTCTCTCCAAAGTCGGCATCTTCAGCATGCACAGATGTCACTNNNNNNNNNNCAGGCAAGTCCTCACGGATCCTGACCACGTAAGGAGCAAGGGCAAAGCGTGGTGCATTGTCATTAACATCCCTAAGCACAATGCTGACAGTGGCTGTGGAGGAGAGGGGATCCTGAGGAGCCTTGTCTGCCACAGTCACAATAAGTAGGTGCTTGTCATGGCGCTCCCGGTCAAGCGGGCCATGTACGGTCAGCAGACCTGTCTGGTCATCTAAGTGGAATTGCTCAGAGTCTAGCTGAAAATGCAACTCTGCATTAGGCCCTTGATCCCTATCAGATGCCCTAAGCCGAGCTACAGATGTACCATTAGGTGCGTCTTCGTTGATGACAAAGTTGTAGGCAGACTTCTCAAACAGCGGAGCATTGTCGTTGACATCAAGCACCCGAATAAACAATGTCTGTGAACTAGATCTAGGTGGCTGGCCCTGATCATAGGCCGTTACATTAAGTAAGTGGGAAGAGGCCAGTTCGTAGTCGAGAGGGGCAGTCAGGAGAAGTGCCCCAGTCTGCATGTCCACACGAAAGCAAGAATGATGGTCACCTCTTGATATGGCATACACCAGGCGGCCATTGTAGCCATGATCTTGATCTTTGGCTTTGACATAGCCCAGCACTGAGCCAGGGAGAAGGCTCTCATTCACAGAGAGATCATCAGGCCAACTCAGTTGCGGACTGTGGCGGTTTTCCAGAAAACTGGCCGCAGTTGG

>AAUM46064

CGATCGGTAACTGTGACCCACAGCTTGGCCGTGGCACTCATGCTGGGCTTGCCCTGGTCGTACGCCACCACGGTCATGTTGTAGAACTCCTGCTTCTCGAAGTCCAGCTTGCTCAGAGTCCATACGGTACCGTCTTTGCTGTCAATGTAGAACTTGTTGTCATGGCCACCTCCAAGTTTGTAGAAGACTAGGCCGTTTTCACCGGTGTCAGGATCAAAAGCGCGAATCTGGGTGACCTTTTGTCCAGGGTCCAAATTCTCGGGGATCTGAGCCAAGTACGGATCAGTAAAGAACCGTGGTTTCTCGTCGTTCGCGTCTTCAACGGTGACTGTTATATTGACTGTCGAGTTTTGAGGAGGGTCTCCGTTATCCAAAGCAAGAATGTG

>AAUM46120

TCAAGTTGGCAAGACCCCTGGATCGAGAAGCTTGCTCCAACTACAATCTGACTGTCGAAGTCACTGATGGCACTGCCAAATCCACAGCCACGGTACATGTGGAAGTCCTAGATGCCAATGACAATTGGCCCATTTTTTCGGAGAGCTTGTACCAGGTGGAAGTCTCCGAAAGCACAGCTGCTGGCACAGAGATTCTGCAGCTGACAGCTGCAGACGCTGATGAGGACCAACGTCTCTTCTACAGCATTCACAACAGTGGCCAAGTGGCAAGTGCAATCCACTTTCGGCTCGACTCAAACACGGGCTTGCTGTCGTTAGCAGAGCAGCTTGACCACGAGGCGTCACAGCGGCACATGCTGACGGTAGCAGTGCGGGACGCGGGCGGGTCTGTAACGCGACGTGGCTTTGCCCGTGTTCTGGTGCTGGTTGCTGACCACAACGACCATGCACCCGAGTTCCTGCAAGCACGGTACGAGGTGCGCGTCTCAGAGACCGCCACTCCGG

>AAUM46453

AATCCAATTTTTGCTCCTCCGTGGACTCCAGCAAACCCAAAGATTGAGATAACTGTCCCCGAGGAGTCTACAGTTGGTTCCACTATATTCACGGTCTCAGCTCGCGATCCACTGACACACGCTGCTGTCACCAACTTCGCAAAGATTCCTGAGTCAGACCACGGAAGCTTCTTTTCTGTTAGCCCCATTTCTGGTGCTGTAACACTTAACCAGAGGCTTGATTATGAAGAACTGCCAGAAAAGGTGTTGGCATTCGATGTGAAAGCCATCATTGGTCAAGATAAAAACAACCAGAAATCTTCAATTGCTACAGTGATCATTAATGTGCAAGATATAAACTAGAACAGTCCAGTTTTCTCACAGAANNNNNNNNNNNNNNNNGTGTCGGAGGCTACTCAGTACCCTCACACTATCCTGCTTATAACAGCAACTGACAAGGACACACAGCAAGGTTATGGTGTCGTGAGATATTACGTCTCCGGAGAAGGGTCAGATGTGTTCAGCATCAATGAAACAACGGGAGCCCTGGGTATCCAGGAAGGTGCAATTCTTGACAGGGAAAAACAGCCACTCTACAACCTGCAGGTGACTGCAGTTGACAACCCAGGTGCACCCTCAAACCAGCGGAGAACATCAGTTCTGGTGGTGATCAAAATCCAGGATGAGAATGACAATGCTCCAGAGTTCACCCAGTCGGGCTACACGGCTGTTGTGCCAGAAAACGTTCCGACAGGCTTCAGTATTCTGACTGTGAGGGCCAATGACAAAGATGCCGGTTCTAACTCGGAAATCAGCTACTCTTTTGTGGATGAACCTGAAATGGGAGCCATCACACTTTTTGCAATCAACAACAAGACTGGCGTCATTACGGTGATTCAACCACTGTCTGGGAGGGGGCGGTCAGAGCCATACTTTCTG

>AAUM47016

CTGGGCTATAGACACCACTGCCTGCCTATTATGGCTCACGATGCTGAAGCGATCCCTTATATTCCCGCTTGTGATATGATAAACAAGGCGAGAATTTTCGTCTCGGTCTGATGCTGTAACACTCATCACAGGAGTTCCAGGAGGATCAGCCTCAGATACTGTGCCTTCGTAGATCTTGGGATCAAATACAGGGTCANNNNNNNNCACATCCTGAACTTTGAGAAGAATCGTGGCTGTGGCAGATCGTGCTGGAGAACCATGGTCCCTAGCAATCACTTGAAAGTTATAGTTTGCATTTTCTTCTCGATCAAGCTCTCGAG

>AAUM48645

CTTGAACCGTGGTTATGTTCAGCCAAGCGAGAAGATGGGTGTGCACGTGGACAGCTCTCATCTGCAGGTCCGCTACAAGATTGTCGCTGGCGACAAGGAGCGGCAGTTCAAGGCTGAAAGCCGACTAGTGGGTGACTTCTGGCTCCTCCTGATACGGACCCGCTCTGAGAGCACAAGCACAGTGGCCTTGAACCGTGAATATGAGGATAGTTACCGTGTGCGAGTGAAAGGCTCAGTACATGACACGTTGGGACATAGGCGCCTACCTGATGTCCACTGTGAGGTGCTGATACAGGTGACCGACAAAAACGATCTCAGCCCACTGTTTTTCCCCACCGTGTACAATGCCACGGTGCCTGAAGACACACCTCTGCATCATGTCCTGGTAAAGTTGAATGCCTATGATCCAGACCTTGGTGTCAATGGGGAGATCTACTACCGTCTGCTGGAACCTTCACGGCAGTTTGCTGTCCATCCGACGATGGGAACAATCTTGTTGACACGGCCGCTTGACTTCCAAAGAAAACCAATTCACCAGCTAACTGTGGTGGCTGAAGACAGGGGCCCCAAGCCCAAGGCTGGCAGCATACTCAAGTCAAGTACAGCACAAGTAGTGGTACGTGTGGAGCCAGTCAACAGACACAGGCCAGCCATATTTGTGCGCCATGTCCATGCACTTGTGGAGGCTCCTGAGGTATGGGCTGTGGTTGTGGTGACAGATGATGACCCTGGTATTCATGGCACGATTGGTGGGCTGGACATTGTGGATGGGGATGCTGATGACCACTTTACAGTAGTCGCTGGCTCGCAGCCTGGTGAATACAGCCTTCGTATTGACCCACGGCGCCCACTGAACCCTAGGGGCTACACACTGACACTCAGAGCATGGGACCAAG

>AAUM48972

GCCAAGGACATAGGAAGGCTTCTCAAATGAAGGCGAGTTATCATCAATGTCCTCAATCTTTATGTCAACCAGCAGCTGCGTCTTGTCATCGGGATTGTAGGTGGCATCAACCTGCTCCAAACGCTTCGATGGCATGCTGACTTGCACAGTAAGAAGGTAGCGATCCTTTTGCTCCCGGTCAACTCTCCTTTTGGTTAATATCTCGCCCTCATTGTTGCCAGTCCGCTGAATAGTGAAAACATTGTCCATGTTGCCACCCACAATGTAGTAATCAATAAAGGCATTCGTTCCAATGTCCGCATCAAAGGCTCTCACTTTGCCAACGATAGTACCAATGGGAATTTCCTCTTTTATCTTCATCACAACAGGCTCAGCATCAGTTGACCTATCAAACTCAGGTTCATTGTCGTCAACATCAAGCACTACAACTCTCAAGATCTGGTGTGCTTCTTGCGGGGGAACTCCCATGTCATATGCCACGACCACAAGCGTGTAGTTGTCACGCTTCTCTCGATCCAGGACGGCTGTGGTGCTCACAAGACCAGATCCAGGGTCTATCTCAAACTCCAGGTCTTC

>AAUM50393

CACCTCTACACGGCGCTCATCGACGAAGGGGACGTGCGCTTCGACCCCCCGCTGAGGGTCCAGGCGCGGGATCCTGACGTGACGTCGTTCGTGAAGTACAGCATCGTCTCCGGAAACAGCTACAACCTCTTTACCATCAACTCACAGACTGGTGACATTACAGTCACGAGTCGCCAAGGGCTGGATGTTTCCCTTCTCAGAACGGACACGATAACGCTAACCGTACAAGCCTCTGACGGTGGCTCTGGCATCGACACTGCAATAGT

>AAUM51536

CGCTCGCTCTTTTGCTCCTCTCTGCGGCCGTCCATCTAACCAGTGCCAATTTGCCGCCAAAGTTTACCAAAACCATTGACCTTGCTGTGATACCTGAAAACACTCCCGTCGGTACGTCTGTATTTCGCCTGGAAGGATCGGATCCTGAAAATTCGCCAGTTTACTACGGACTTGAAGGAACGGACCTCCTTGCGGTAGATCGGAGCACCGGCGACGTCACTGTGATAAACAACATTGATCGGGAGGTAACAGACACACTAAAATTCTCAGTGACCCTTGAAGACTTTGTTGGAGGACTTGATCAAAATAACATCGTCAAGGTTCCAGTCAGCGTCATTGTACTGGACGTCAACGATAACATCCCAAGGTTTCAAAATGTTCCGTACGAGGCAAAGATATCAGAGGATGCGCCAGTGGGACACACGGTCCTGAACAACATCAGAGTGACCGACTTAGATTCTGCAGGGAACGTGCTGCAGGTTCAGTGCTTCCCGAACAACAATGTCCCGGAAGCCTGCTCCACGTTCTCTGTGGTGGTCACAATGTCTAGTCCCCAGGAGCTGAACGCATCTTTGGTACTGCGGAAGCTGTTGGACCACGCTGTCCGTCATGTGTACGA

>AAUM53168

CTCCGCCAATTGTGTTCAAGTGGACAGTCAAGAGATATTTGTTCTGTTCTTCGTAGTCGAGGTTCTGGTTCTGGACACGCACTTCACAGTCGCGCTGTTCATTGTCCAATATGTAGAAATGCCCTTTCTCGTTTCCAGTGACGATCTGGCACTGAACGGGGAAATTTCCTCGCGGCTTGTTGATAATCGGCAACGATTTGACGAGGGTGTTCGCCAGCGCGTTCTCTGGAACTTCAACCGTGTAGAATCCGTCGGCGAAACCGAGCCCGGAGTCCGGGGGCACGGTGGCGATGTGCTCCACGTAGACCGTGACGACAGCCGTGGCTGCTAACGAGGGATCGCCCAGGTCGTTCGCTTGAACTTCGATCCTGTACTCGGAGTCGGGCTCTTTCCTGAGGTCGTCCTTGACAGTTATCACGCCACTGGTGCTGTCGATGAGAAAGTAGAATGGGGCCCGACCTTGGCCGCTGATTTCGTAACGCACGACGTTC

>AAUM54204

TATCTCTTGACCGTCCACTTGAACACAATTGGCGGAGTGTTCGGTTCTTCGAGGCTGACCACCCAGGTGGCTGTGCACTTGATTGACCAGAACGACAACCGGCCGCGCTTCGTAGTGCCCCCCATGTACAGCCAGCTGACGCAGAACCGCTACCTGGCAGCACTGTCGTCGGATGCCCCGGCCGGAACCCGCTTCATCCAAGTGATGGCTGAAGATCTCGACTCCGGTTCGAATGGCAAGATTGTGTACGACCTATCCCCTGACAGCGACCCCGAAGGCAAATTCAGCATTGACCCCCAGACCGGCTACGTGAGCAACGTGAAGACGTTCGAGGATCTGCAAAACGTGGAGCTGCCCCTGAAGCTCAAGGTCACAGCCAGAGACAGCCCCGACCTGAC

>AAUM55388

CCTCGACGTACACCCAAGCTGAGGAAGACAGCAGACCGTCATCACGGGCCCTCACTGTAAAGTTGTAAAATGGCACGGCTTCTCGATCGAGAGCTACCAGAGTTGTAATAACCCCACTCTGCGGGTCAATCCGGAAGATGTCAGCAGCTGGATCAGTCTCTTCGTAAAATTCGTACGTCACCCTTCGCACTGAGACATCAGGGTCGTGTGCCACCAACCTGGCCACTACACAACCTTCCTCAGCATTCTCAGCAACTGCAGTCTGGTATGACATCACATCGAAGACAGGCTCACAGTCATTGACATCCATGACATGCACGGTCACATCAAGTGCGGAAGCCAGACCACGGGGTGTCTCTGCGCGCAAGGTGAGTTGATGTGATGCCTGG

>AAUM55512

GCCATGGCACCGGCCAGATTGATGGCACGCACTGTCAGGTTATGCACAACTGCCTGCTCATAGTCTAGGACTTCATCGCCTGTGGAAAGCACGCCTGTCACTGGATCCAACCGGAAGTGGCTGCCAGCCCCTTGCTGCAGCTGGAAGAAGACAGACGAGCTGCTCCGCACAGTGAACGCCAGCAGAAATGCTCCGGCTGGCTGGTTCTCGGCCACCTCAATGGTCTGCTCAGCAGGCTCAAAGTTGGGCGGTGCATTGTCTGCCAGTGTG

>AAUM58273

CGGTGTTGGTCCTGCCGGATGACGTCTACCCCGGCCTGAGCGTGAAGCAGCTTCCAAGCGTAGGCCAGTCATTCGCCCTGACCGGAGAGCCGCGAGTGACCCGTTGTTTCAGCCTGCTCAGCGACGGCCTGCTCATGGTCGCCTCGAACGTGTCTCACCTGGTCGGGAAGCCGGGGAAGCTTGTCCTCACAGAGACTAACTTCCCGAGTTGGCCTCTGGAGCCGCAGGACCGCGTCCAGGAGCTGCTCGTGCATGTCATCCCCAAGAACAAACTCCTCAAGTTCCCAGAGACCGAGTTCCGGGGCACCGTGCTAGAAAACGAGTGCGCCGGGACGACGGTGCTCGAGCTGGACGGTGCTACGAAGGATGCCAGCCGCAGCGTCAAGTACGAGCTGCCGCCAGACGAGGATGGCGGCCACGAGGAGTTCACCGTGTCTCGGNNNNNNGACAACTCCACGCGGCTGGCGGTTCGCACTCGCCGGCCGCTAGACCGCGAACACCGCCGCCGCTACGAGCTCAAGCTGAGGGCCTCGGACGGCGTCGACTCGGCGCTGACACGCCTGCTGGTCAACGTGCTGGACGTGAACGACATAGACCCCGTGGCGGACCGCGATTCGTACGTGTTCCGCGTGGCCCGCGACGCCGAACCGTACTCGATCGTGGGCAACGTGAGCGCTTCGGACGCAGATGGCGACAGCGTGAGCTTTCGCCTCGCCCAGCGTCATCCCTTCTTCACCGTGGTGCCCAAGACGGGAGAGCTCATGGTCACCCGCGGCCTCGAGCCCAAATCGTACCTGATGGCCGTTCGCGTTGGCGACCGCGGC

>AAUM60851

GCCGGCTTGATTATGAAGATGAAAATGCCCGCACGTTTGTATTTCAAGTTGTGGCATCTCGGCATGGATTTGAAGTTGCGAGCGCCACTGTTACTGTGTTTNNNNNNNNNNNNNNNNNNNNNNNNCCGATGTTTGAGAATGAGCAATACCTAATACAGGTACCTGAAAGNNNNNNNNNNNNNNNNNNNATTTTTTCGCTTGAGGCCTATGATGCAGACAGTGGACCATTTGGGAAATTGTCATATTCAATGAAGGGCTATGGATCTGAAAAGTTCGTGGTGGAAAAGGATACTGGGCGCATAAAGATTGCCACCTGCAAAAATGGCTGCCTCGACTATGA

>AAUM65145

CGGGTCCTGGTCGTCCCCGTCCTCGACGAACACCGTCAACGTGGTCGTGCTGGACAGCCGCTCCGACGGGTTCGACGCCGTGTCGGTTGCCTTGATGAGCAGGTGGTAGNNNNNNNNNNNTTCGTAGTCCAGGGGCCTCGCTATCGTCANNNNNNNNNNNNNNNNNNNNNNNNNNNNNNNNNNNNNNNNNNNNNNGTTGTTGCCGCCGTCCCCGGGAGCCGTGGTGTACTCGACGAGCCCGTTGGTCCCCGTGTCCATGTCCATGGTGGCCAAGTCACGGAAGATGGTCGTGCCCACTGGCGCCAACTCTGAGACTTTTGTTTCGTAGTGGGGGCGCCTGAAGAGTGGGGGGTTGTCGTTGATGTC

>AAUM31278

CACGGTCACATCAAGTGCGGAAGCCAGACCACGGGGTGTCTCTGTGCGCAAAGTGAGCTGATGTGATGCCTGGTGTTCGCGGTCAAGTGGCGCAGCAATGACCAAGTTGTTTCCTGCATCAATGCTGAACTCCGAGCTGCCTAGAAGTGTGGTGCCTGGCGCCAGCGCCAGCAGTGGACTGCCGAGAGCTGCACTCTCACGAACATGCAGGTCACGGGTGGACACACCCAGCACAGGCAGCTCTTCAGGTGGCTGCACCACAGCCACCGTAACAGGCACCACAGCCCAGAGAGGTCTAGGATTACCCTGGTCACTGACGTGCACGAAGAACTGGAACACGCCAGCTCCTCTGAGGGGTGCCTTCAGGTAAAGCTCTCCTGAGTCGTGAGTGACATTGAACAATGCACTGACATTGGTTGGGGCTTCATGAAGGCTGTAACGAGGCCATGTTCCTTGGTCACGGTCAAAAGCACGAACCTTGACAAGTGTAGTGCCCACGCTAGTGTTTGTCCAGATGCTAGCCTCATATTCAGAAGCACCAAAAGTGGGCTCATTGTCATTGACGTCAGTGACAGCCACACGAACAAGAGCAAACGCCAGGCGGCCGCCACCATCAGTGGCAGAGATGGGAACCTCGTAAAGATGTTGATGTTCGCGGTCCAAAGCTTCCCGAGTCACCAGCTCTCCTGTGGTGCTGTTGATGTTGAAGTGCAGTGCACAGTCTTGACTGCGGATGGCATATGTCAGCGTGCCCAGCGCCCCCTGGTCGGGATCCGTGGCAGACACGCGGGTCACATGGGCACCTGGCGGCAAGTTCTCTGCCACCACTGCTTCATACACAGACTGGCTGAACCGGGGCGAGTGTTCATTGCTCGGCACCACCATCAGCCGCAGCTGTGTATAGCTCTGGTACACGCCGTCCGTAACCGAAATGTTAAGCTCGAAGCCCCCAGGTGGCCACGCAGAGGGCTGGTTGGACAGTGTCACCACTCCTGTTGACCTGTTGATGGCAAAAAGCTGTGCCTCGTTGCCAGCAACGATGGCATATGACAGCTGGTCAGAGATATCGGGGTCCAGTGCAACAAGCCGAGCAATAAACTGGCCTCGATGAGCCCGCTCGTCGACCACAGCTGTGTACGTCGGCTGTCCAAACACTGGCGGGTTATCATTCATGTCTATTACGGTGACCCAGACACGGGCTGTAGAGCTCAGAGGTACAGGGTGGCCAGCATCCGATGCCACCACCTGTAGCCGGTGCAAGGCTTGTGTCTCCCGATCAAGAGGGGCCCGAAGCCGCAGGACACCGGAACCTTCCAGGTGGAAAGTGGCACTGTCGTCGTCAGCCAGTCTGAGCTCCAGCATTGGTCCATCACGGTCACTGGCGCGCAACTCCAGAAGCACGCTGGCCACAGGCGTGGATTCTGACACGCTCACATTGTACCAGGGCTGCTCAAAGATGGGGGCACAGTCATTGATGTCCTCCACGTTGATGGTGACGGGCACATCAGAGTGTGCACCAGTCACGGAGTCTGTTGCTCGGACCATCAGCTCATGCCGTGGTTGGC

>AAFF7007

GAGCCGACAGCTGATCGGGTTCACAGGCTCCTTCGCACAGCTCCAGCTGGGCATCCGGGAACAGGGCCCGGCGACCGGAGCCTGGTGTGTACTCCACATGCTCCTCCATACCTTTCCATCCAACCTTGCAGACCCTGTTGACCTTGATAGTGACAGTGACCGGATGACTCTGTTTCATGCTACAGTCAAAGGCCACCACCTGGAATATGTGGTTGCTGCTTGCCTCCCAATCCAGGGGCTCTGTGGTCCAGATGGTGCCTTCAGAGTCTATGGAGAAGGGCACATGTGCGTCCAGGATGTCATACTTGCAGATCTCACTGTTCTTGGGCGTGCAGTCAGCATCAAGAGCACGCACACGGAGCACACGCTCATGTGGCGGCCGGCCCTCATCCACGCTGCCTTGGTAAGACTCCTCCTCCCAGCGGGGCGCGAACTCATTGACGTCATCCACTGTCAGGTGCACAGTAACGTTTTCGGAGACGAGCCCGTTGCATCCCACGGCGGCGATGTCGAACTTGTAGTTTCGGTGCTTCTCGCAGTTGAGCTCCTTCTTGGCGAACAGCTCGGCCTTGCCCGTCGCCTCGTCCGTGATGCGCACCTCGAAGGGCGCCTCACCGTGACGCTTGTTGGTCACCAAGAAGCGGCACACCTTGGCGTCCAGCACCCGGATCCTGGGCATGATCTCCACATTGCGGCTGTTCTCCTTGACGATGGCATGGTAGCCGACCTCTGTGTTCACGTGCTCCAGTCGGGGGGCTTTGAGCGCGGAGGCGAGTCCGACGAAGAAAAGCGCGAGCACCGGGGCACACTTCATGATCGCATCCAGCTGTAGTGGACGACTGTTTGGATCACAAGCAGTGGCTCACCATAGGCGCACGCACAAAGTCAGAGAAACGCTTCGGGCAGCGGGGGCACGCCGTGTAAAATTCCCGTGCCAAGAAGTCTCGGGCGTTTC

>AAFF10805

TCTGATCCATGGGCATGACAGCTTTTGCTTCTTTTATTACAAATAGTGGACAAAGAAGCGCCAGCTCTGTGTAGTCTCATACTACCAATGGGTTTCACAGACTTTTGCAGAGATGATGAAACATGAAAGCGATGATTTGGCATTTGTATTTTATTTCTGCAAGTTCAATTTTACTCTAAACATGAGGACAATGCACACAGCTTGCGAGGCCGGTAACACAAGGCACAGTTTTCAGGCAACGTCATAGGCAAGCAGTCCCTAGATAGACAACACAGCATTCCCTCAGAGAGGTCATGCTCTCTGCCCACGGGCGCAGTCACGAATGCAGGCCAGCGAGGAGAAACCATCACGCTGGCACTCCTTGTATGGCACCACTGCTGGCTTTAGCACCAGCATCTTGCACAGATGTCAGGCCAGCTCTTCATCACTGTCCTCAATGGCACACACTGCTGGTTCACTGGGGCAGTGCCGAGGCAGGTACTGATCGGGGTGGCAGGTGTACCGTTCTTCACCTCCCTGCAGAGGAGTGCCTTCGGAGCAGTGTGAGCTTCGGGAGCTGCCAGGCGCTTCATCACTCGAAACGTCTTCCTCTGCATGCGCTGCTAGGTCCGAGTAATCCCAGCAATAGCCTGGGAGATCTGATGCAATGCTGGTTGCCGAGCTTGGTGGTGCCAAGGGCGGAGACAGAGCTGCCTTCAGGTCGTTGGGGATCTTGTCCAGGTACAGGCCTTTGTGGCCTCCCTGTGGGGGCCCTGTGGCACTACCAGATGGACTGCGAGCCAGATTGTGGCAGCGCAGGTCATTGGGCTGAAAGCGTGACTCAAGGTCATCAGCTGCACTTCCATAGCTGCGCACCGTGTCAAAGTTGTTCAGTGTGATCTCTCTCACGCCAGTCGTCGTGTACGAAGCTGGCCGCTGCTCCAGGTTGCTTATCTTTGGCCTAATGTTTGTGGTGGCCAGGACACAGTTCTTGGCAGTGACAGGATTGCTGCACTGATGGCTGTCAACATGTTCGTGGCGGTGGCGGCAACGACAGCATTGGCGGCAGCAGCAAACAATGGCCAGAAGCAGGAGCAGAAGCAGCACAGCAGCAGCAGCACCTCCCAGAGGCCAAGCGAGGGGCAGGCCCTCGAAAGTGGCATCACACGATGCATCCCCCAAACAGCCACACTGGAAGCCATCTGGCAGCCGCACACAGCTCTCCCCGTTCTCGCACGCCGTACATGCCTCCACCAGCTCACATCGGTTGCCCTGATAAGGAGTTGGGCAGTGGCAGTGTCCACCTGGCTGGCACAGGCCCCCATGGAGGCACAGGTTGGGCTGGCAGCTGGCTGGCTCACATGCTTCGTCAGTGCATCGGCTCTCAGCTACTGCCTCTCTGCACTGGGGACCCTGAAAGTGCGCCGGGCAAGTGCACGAGTATCCAGTGGTGGTGAGAAGAGGCCGGCAGGTTGCCCCATTGTGGCAGGGCTGGCTGCCACATGGGTCGAGGTGGCAAGAAAACTGGACGTTGGCCAGCCGGCGCAGCTGCGCGTGGCCAGCAGGTCGCAGATGCAGCGGTAGTGGCTGGCCGCCCACCTGCGCATCATCCAGACAGCCGACCAAGCCTGCCACAGCAGGTGCCCCGCCCAGGTGCAGCTCACGGCCCTCCAGGTTGAGAACATCGTGTGGCCCAGGCGCGGCACCAGAAGCTTGGTAATGCACGTCGACAGCGAGCCGCGCATTGCTGCCACGACGCTCAAGACGCAGTGCATGCCAGACACCGTCATCCACCCGACGGCCAGTCACACGCACCAAACCCTCACCGCTGCCACAGTCGAAGCGGTACTGCACGTGCCCGTCCGACACCTCAAGGATGGCGTAGTCTCGGGGTCCCGATGTGTGCAGCAGAGTGCCACTGGGGTGGATTGTGCGCAGCATCACAGAGAACGAGAGGCGTCGGTCCAAGGGCTGTGACAGCACGTAGAGCGCATAGCTCTGGCCACCAAACGAGATGGGCCGCTTTTCTTCATAGCAGCTGGGTTCCTGGCAAGGGGTGTCGCATTCAGAACCAGTGCGACCAGATGGACATTGGCAACTGTGGCCGAGTGGAGACGCGTCTGGCACGCAGAGGCGCCCTGCAGGGCAGGGCTGCTGGGCACATTCATCCACTGCTGTTTCGCATGCATCACCGCCAAAACCAGGGTTGCAGACACACGCCGTCCGGCGGCTGTGTCGTGGTGATACGAAACTGTGGCCACCACCAGCAAGGACAACAGCGTCGCTGGTGTCCAGCACCAGCCGGTCCTGACACTCGCCGTGCACACAGTGCAGGGAAGGGCAGCGGTCCCCCTGGGGCAATATGTGAACACGTAGACCAGTAGCTGCCTCGAGGGCTACATGGCGCTCGTGAAGCCGGGAAGCCACTGTGCTAGCTGGCACTGGATCTCCTTCAGGCTGCACTGCCAACAACAAGTCCAGCTGCTCGCTCCCTCCTGAGGCCTCCGCTGGTGCTGGCTGTATGCTCACCAGCAACAGGTCCCGCAAGCGCACGCTGAGAGCTGTTCGCAAAGCTCGCAGCAATGGCCGCCGGTCCGTAGATAAGAAGCGCTCAGTCGTGGTTCCAGCCAAGCGCAGAGCCACAGCTGCCTTGAGAGCCTCCTCGGTCACCCCGACAACATGCACATTCACTGATGCATGTGCTGGCGTTGTGCTGCCATCTGAAGCGCTCACATTAAGGACATAGCTGCCAGCATCAAGGCCTGGCAAAGCACGCAAAGTCCCATCATCCCTGTCGAGGACAAACAGCGATGCGTGTGGCCCAGAGAGGGACAAGCTGAGGCGGTCGTACGGGTCCTCATCCGTGGCATGCACCCGGCCTAGCAGCCCACCCGGGAAGTCGTCCAAGTAAGAAGAGACAACCACAGTCAAGGGCTGCACCTGTGGCCTGAAGCGGCTCTTCTCAACAGCCAGCACAGTCACATGCGCCTCTGTCGCCAGAGGTGGGCTGCCCGAGTCGTGAGCCTGCACACGCAGCTCATGCCGCGAGCGCGGAGGAAGTGGTCGTGCTAACCGCAGCTCGTGGCCCTCAACTTGGAAAAAGGGTTCCTCCTCCTCCTGCCCAGCTTGAGCTCCATCCAGCAGCTCCAGCCGGAAAGGAGGTCCATGAGCAGCAGAGTCAGCATCACTGAGCGAGAAGCGCAGCACGGTCCAGCCGACTGGCCGACCCTCATGAACCACGGCCGTGTAGTTGCTCTGGTCAAAGCGCGGTGGATGGTCATTCACGTCCAGCACCTCCAGGTGCACCAGTGCCCGAGCCGACAGTGGCGGTGTGCCACCATCCCAGCACTCCACCTCCAGTGCGTACCGGCTCACCGTCTCATGGTCCAGCGGCCCAGCCACACTTAGCTGGCCAGTGGCCGGGTCCAGAGTAAAAGGGCCCGGCTGCAGCAGAGCATATCGCAGCTCTCCGCCACTGTCGGCATCACTGGCCTGCACCTGGAGCACAAGCTGCCCAGGGGTTGCATCCTCCGCAACAGTGCCACTGTAGGGGCCTCCGAACACAGGCGCGTTGTCATTTGCGTCCAGTACGGACACATTCAGCCAGGCGCGGGCACTGAGGGGTGGCTCGCCCCCATCGCGAGCCTCCACTGCCAGTTGGTAGCTGCGCGCGCTCTCAAAGTCCAAGGGTCGGGCCACCCGCAGCACACCTGTCTTTGGCTCAATGCTGAAGTGTCCCATGTCGTTGCCTGCAACCAGGGAGTATGTGATCTGAGCATTGACGCCTGCATCTCGGCTGGTAGCCCTCACACCAGCGCTGACGGGCGAGCCCACGGGGGCAGCTTCAGAGACGCTGGCAGAGTACGCTTGCTGTGTGAACTCGGGCGGGCTGTCATTAATGTCCTGCACAATGACTGTAAGAGGCACGCGGGCAGCCCGGGGAGGCTGACCATGGTCCTGGGCCTGCACCGTCAGGTTGAACCTGGCGCATTCCTCTCGGTCTAGTGGTCGCTCCAACCGCACCAAACCAGACAGTGCATCCACGCTGAAGTGGCTG

>AAFF14128

CGGACACTGGCGAGATCAGAGTGGCATCCAGGCTCGACCGCGAAGCTGCAGCCGTCGTCATGCTAACCACCGTGGTGACTGACGTCAGCGCTGTGCCACTCCAGACTGGATTAGGCACCCTTGTGATCACTATAGTGGACCTCAACGATTTTCCGCCTTCGTTCCCGCCTCCTTGGAGTCCCGACCATCCGGAGCTGTCCATCAGTGTTATGGAAGAGCAGCCCGTCGGCAGCGTCGTCGCGTCGTTCGTTGCCACGGACCCGGACTCAAACATTGCCAGTTATTCTATCGAACCCGAAAATCCTTACTTCGCCATTGACAAACTTTCCGGTGTGGTCACAATACACAAAAGAGTCGATTATGAGCAAGTGCAAGAATTGCGGTTCTCTGTCGTTGTGAGAGATACGGGCATTCCTCAGTTGTCAGCGGTCGCAATGGTGACGGCAACTGTGACCAACATAAACGACAACGACCCGACGTTTTCGCAGAAAGCTTACCAGGCTTCAGTCCAGGAGAACGCCCCTCAAGGCACGTTCGTGACGAAAGTTGAGGCGAAAGACATCGATGCCGGAGAATTCGGCGTCGTTTCGTACTCCTTGCTGGGAGAGAAAAGCGGAGACTTTCAAGTCAACAAAAGGGGCGAGATAAGTGTCGCGGGAATGGCCAATTTAGACAGAGAGATCACTCCTGCAATCACCCTTCAAGTGGTAGCCACTGACATGGGCCACGATGCAACGACGAGGAGGTCGGTCTCCGTTCCGGTGTACCTTACCCTGCTCGACGACAATGACAGTCCTCCCGTGTTCACGAAAAAGACTTACGAGGCTTCGTTCGTCGCCAACAGCCCGCTCGAGTCTGCGCAGAGCATCGTTCAGGTTTCTGCAACGGATGCGGATGAAGGGATCAACGCCGAAGTCCGCTACTCCATCGTCGCTGGAAACGAAAACGGAGTTCTCGCCGTCAACCCCAAGACTGGAATCGTGTACCCCGTGAAGAAGCTGGAGAGCAGTCGCAAGGAGTACCGCATCGGGTTGGAGGCACGCGACGGCGCCGGCACTGGACCGAACACGGACAACTGCGTGGTGCTCATCAGGCTGATCGAAATAAATCTGGACAAGCCACATTTCGTGACACCCTCCCTACCCAACGCCACCGTGGAAGTGCTCGAGAACCAGACCCTCTCGAGCCAAATCATAATGACTGTCGAGGCCATGGACAAGGACCACGGAGACAACGGGCGCATCTCTTACTACTTCAAAGTTGGAGACAGGAACGTCGCAGAAACGGACGAGTTTCGCATCGACGAAGTTACCGGAGAAATCCATGCCAAGGCCGTCCTCGACAGGGAAGTCAG

>AAFF19547

AGGGAAGTCAGGCCGCGCTACGAGCTGGTGCTCGTCGCTAAAGACCACGGTACTCCCGCCCCGTTTGAAACGCTACGGTTTCTCACGGTCGTCCTGAAGGATATCGACGACAATGCGCCTTTGTTCCCTCGCACACGGTCGACGAACCCTTACGTGTTTCACATCAAGGAAAACTTAGACCCTGGCTTTCCCGTTGGAAGGGTAACGGCGATTGACCAAGACGTCGGAGAAAATGCCATGGTCTATTACTACATTATCGATGGAAATTGGGACACACAGTTCACGATAGACAAGATGCAGGGTACCATCTACTCGAACGCCTCGTTCGACCGCGAATCCAAGGACCTGTTTGAACTAGTGGTGAAAGCCACGAGCAACCCAGACTACCTGGTCTACACGAGGCAGGCTGATGGCCTTCCGCCCAGCATACGGAGCTACAGCGAGTCTGACCGCACTGTGGCCCTGGTCAATGTTCACATCGACGACGTAAACGACAACGCCCCTGTCTTCATAAATGCCCCGTACTATGCAGGGCTGCGGTTCACTGCCGTGGAAGGGGACACGGTGTTCACGGTGCGGGCCCGGGACCCGGACGAAGAGACGGCGGACGGCGCGGCCATTGCGTACCGAATCGAACACGTGACGCTTTTTCTGCCGGGCTCAACGGGAGGCATCCGGCCCATTCCGGCCTCGTTCAACGTTTCAGCAGACGGCCGCGTGTGCGCCACGCATCCCATGGCCCAGTACAGCCAG

>AAFF20658

CTGGAGGCTGGTTCTCATCCACGCTGACACTGGACACCTCTTCAGGAAAGACTGGTGAATGCAGATTCTCGTTGACATCCTGTACTTCGACGATTAAGCTCGCCACACTACTGAACGGTGGGTCACCACAATCCTGAGCCTGCACAGACACGTTGTACAGCCGTAGTGTTTCAAAGTCTAGCGATGCAGCCAGCCGAATAAGGCCAGTGTCAGGATCGATGTCGAAGACACCAGCATGCTGGCCATCCAGAGAGTATTGGACCCTGCCATTCTCTCCAAAGTCGGCATCTTCAGCATGCACAGATGTCACCAGGGTGCCCACAGGCAA

>AAFF20702

TTGCATTGCCACTCCCAACGAGCCACGTCACGCACATGTGCCACCAGTGAGTAGTGCGGCCGCTGCTCTCGGTCTAGAGGCCGGGTCGTGTGCAGCACCCCACTGCTGGCATCCAGAACAAAGTCCTCAGATCCTTGGCCTGACAGGTAGAAGTGCAGCTGAGCATGGTGGGCATCATCCGCATCCGTGGCTCCAACCGTCAAGATGGCAGTACCCACAGCAATGCTCTCCGAGACAAGCTCCGTGTACTTGGACTTGAGGCAGATTGGTGGATTATCGTTGCTGTCCAGGATGGTTATATCAAGCTGAGTCATGGCCACATGCAGACCGTCTGTGGCCACCACTCGAAGAGTGTAGTTCTGACGTGCTTCCCTATCCAGCGGTCTTTGTACGAACACATCACCACTACTGCGCACCGCAAACTGCT

>AAFF24933

TTTTGGTGGTGATCAAAATCCAGGATGAGAATGACAATGCTCCAGAGTTCACCCAGTCGGGCTACACGGCTGTTGTGCCAGAAAACGTTCCGACAGGCTTCAGTATTCTGACTGTGAGGGCCAATGACAAAGATGCCGGTTCTAACTCGGAAATCAGCTACTCTTTTGTGGATGAACCTGAAATGGGAGCCATCACACTTTTTGCAATCAACGACAAGACCGGCGTCATTACGGTGATTCAACCACTGTCTGGGAGGGGGCGGTCAGAGCCATACTTTCTGACGGTGCGAGCCACAGATGGTGGCTCTCCATCACTCCACACAGATGTCAAGATACTCATCATTATCGGGGACGTGTCTTCAA

>AAFF28122

GTGCTGTAACACTTAACCAGAGGCTTGATTATGAAGAACTGCCAGAAAAGGTGTTGGCATTCGATGTGAAAGCCATCATTGGTCAAGATAAAAACAACCAGAAATCATCAATTGCTACAGTGATCATTAATGTGCAAGATATAAATGACAACAGTCCAGTTTTCTCACAGAATTCATATGTAACAATGGTGTCGGAGGCTACTCAGTACCCTCACACTATCCTGCTTATAACAGCAACTGACAAGGACACACAGCAAGGTTATGGTGTCGTGAGATATTACGTCTCCGGAGAAGGGTCAGATGTGTTCAGCATCAATGAAACAACGGGAGCCCTGGGTATCCAGGAAGGTGCAATTCTTGACAGGGAAAAACAGCCACTCTACAACCTGCAGGTGACTGCAGTTGACAACCCAGGTGCACCCTCAAACCAGCGGAGAAC

>AAFF31491

GTGTCGGTTGCCTTGATGAGCAGGTGGTAGTACTTGGCCCTTTCGTAGTCCAGGGGCCTCGCTATCGTCACTAGGCCTTGATATGGCAGGTCAATAGCGAAGTATTCATACCCGTCGTTGTTGCCGCCGTCCCCGGGAGCCGTGGTGTACTCGACGAGCCCGTTGGTCCCCGTGTCCATGTCCATGGTGGCCAAGTCACGGAAGATGGTCGTGCCCACTGGCGCCAACTCTGAGACTCTGGTTTCGTAGTGGGGGCGCCTGAAGAGTGGAGGGTTGTCGTTGATGTCGTTGACACGCACCAACACCTGGATGGCTCGCTGCGAGAAAGGCTGCACTCTGCCGAGCCTTGCTTGGCAAAGAACTTGAAGCCTAATGGACGCCAAATCATCGGCGTCTCTGTCCAGGGGCCTCTTGAGGATGA

>AAFF33263

TTCTCGACGCTTGCGATGAGACGCGTCTGTTGCTGTATGATGCTCATTATGTGATCCTTGAGCTGCAGTACTTTGTCCGGCAGGACATCACCGATTGCCAAGACCAGCCGGTTTTCGTAGCGGATAAGGTTTACATAAACGTATGCGGATTCCGTGAGGGCCCTGGCGGTCAGGTCGGGGCTGTCTCTGGCTGTGACCTTGAGCTTCAGGGGCAGCTCCACGTTTTGCAGATCCTCGAACGTCTTCACGTTGCTCACGTAGCCGGTCTGGGGGTCAATGCTGAACTTGCCTTCGGGGTCGCTGTCAGGGGATAGGTCGTACACAATCTTGCCATTCGAACCGGAGTCGAGATCTTCAGCCATCACTTGGATGAAGCGGGTTCCGGCCGGGGCGTCCGACGACAGCGCTGCCAGGTAGCGGTTCTGCGTCAGCTGGCTGTACATGGGGGGCACTACGAAGCGCGGCCGGTTGTCGTTCTGGTCAATCAAGTGCACAGCCACCTGGGTGGTCAGCCTCGAAGAACCGAACACTCCGCCAATTGTGTTCAAGTGGACAGTCAAGAGATATTTGTTCTGTTCTTCGTAGTCGAGGTTCTGGTTCTGGACACGCACTTCACAGTCGCGCTGTTCGTTGTCCAATATGTAGAAATGCCCTTTCTCGTTTCCAGTGACGATCTGGCACTGAACGGGGAAATTTCCTCGCGGCTTGTTGATAATCGGCAACGATTTGACGAGGGTGTTCGCCAGCGCGTTCTCTGGAACTTCAACCGTGTAGAATCCGTCGGCGAAACCGAGCCCGGAGTCCGGGGGCACGGTGGCGATGTGCTCCACGTAGACCGTGACGACAGCCGTGGCTGCTAACGAGGGATCGCCCAGGTCGTTCGCTTGAACTTCGATCCTGTATTCGGAGTCGGGCTCTTTCCTGAG

>AAFF34347

GAGGGCCGTGCCTCGAAGTCCAGGCAGTTGCCACGCCCGGGCGTCTCGCACGGCGCCACCGTCACCACGCCCGAGCTGGGGTCCACGTGGAACTTGTCTGCGCCGTTTCCAAGAAGAGAGTACATTAAACCGGACTCTCCGTGAGGGCTTGTGTCTCTGTCGGTAGCCACTATAGTCGTTACCACGCTTCCAGGTGACGCGTCTTCAACGACTGTTGCCGTGTAGCTCTCTTCTTCAAACACTGGGGCGTTGTCATTGATGTCGAGCACGTTCACTGTTACCGTGGCTGTGCTCGACAGTTTGGGATTCGTGAACGCTTCTTCTGCAACCACCAGCAAGATGAACTTTCGTTGGTTTGGATTCTCGTAGTCCAGTGGTCCTTTGACAATGCGAATGCTCACAGATGTTGAACCGGTTGCGATGGTGGGCTCCACGGAAAACATCCCCGATGAA

>AAFF36175

TTGAAGTGGGCACTCGTGTCATTGCTATCAAAGCCAGATCCGGAATCCCACAGAATCCTGATGTGTTCTACACGTTGATGAAAGGAAGTACAGAGCAGACCAACAAGAAGGACACTTTCTACTTGAATCAAAAGCTTGAGAATCGCCAAACAGTGGCAGAGCTGGTGGTCAATTATCCTCTGGACTATGAACGTATCCAGCAGTACAACCTTACTGTTCGTGTCGAGAACAACGGCATCCAGCAGTTGGCATCAGAGGCAACTGTCTACATAGTCCTTGAGGACGTCAATGATGAGATCCCTCTTTTCATAGAGCGGGAGCAA

>AAFF36498

CCACACTCAGCCCCGTCACCATCCAGGCCGGCGATGGAGTACGGAACGTCACGCAGGTCCAGGCCCGAGACGATGACGAGGGCGAGTATGCTCGTATCAGCTACTCCGTCTACCACGTGTCCAACAACGGTCGTGACAGGTTCCGCATCGACCCCAACACTGGCGTGGTGCAAGTGATCGGAAGGGTCTCCTCCGGGGAGCAGTACAGCATTACTGTGCAGGCGACGGACTCAGGCGGAAGGTTCAGCCAAGGCATTTTGGATGTGATAGTGATACCGGGTCCGAACACTGGAGGTCCCGTCTTTTCGAAAGAG

>AAFF36499

TTGTAGCTCTCTTGCACAAACACGGGGTTCTGGTCATTTATGTCCGTCACAGCAATCTGCACTGTTGCCAGGTTGAACAAGTTGTTCTTGTCTTCCGCTTTCGCCTGCAGTGTGTACGAGGCCACCTCCTCGCGGTTGAGAGGCTTGGCCACAGAAATGGTCCCACTCGAAGGATCAACGGTGAAGGCGCCTTTGTCGTTGCCCTCCACTATTGAGTACGACACGTCGTCCCCTTCTGGATCAGTGGCCTTCAGCGTTAGGACAGCAGAGCCGACAGAAGCTCCTTCGCTGACTTGAGCTTCATACTTCTCTTTCGAAAAGACGGG

>AAFF37324

ACAGGTACCTGAAAGCGCTAGGAATGGAATGACTATTTTTTCGCTTGAGGCCTATGATGCAGACAGTGGACCATTTGGGAAATTGTCATATTCAATGAAGGGCTATGGATCTGAAAAGTTCGTGGTGGAAAAGGATACTGGGCGCATAAAGATTGCCACCTGCAAAAATGGCTGCCTCGACTATGAGAGTGTTCCGAATTACTCTTTCACATATGAGGCACAGGATGGAGG

>AAFF39151

NNGGTTATTACAACTCTAGTAGCTCTCGATCGAGAAGCCGTGCCATTTTACAACTTTACAGTGAGGGCCCGTGATGACGGTCTGCTGTCTTCCTCAGCTTGGGTGTACGTCGAGGTCCTGGATGTAAATGACAATCCTCCCGTGTTCGAGCAGACCCTATACCAAGTGCAAGTCTCTGAAGATGCTGTTGCTGGCACTGTAGTTGCCAGCCTGCGCGTGGAAGATGCTGACAAGGAGCCGGCTCCTGTTGGCTATTACGTGCTCTCCGGTGATCCTGGTCAGCAGTTTGCAGTGCGCA

>AAFF39959

CTCCCTGTCCAGGCTGGAGGTCACCGACAGTCGGCCAGTGTCGGGCTCGATAGCAAAGAGTCCGTTGTAGTTGTTGCCAACGATTGCGTACTGAACGGGTCTGTGGGCGATTTCTTCCGTTGCATTCAGATCGAGAATAACCTGAGGCGCCAGCTGGTTTTCAAATACAAGCACCTCGTACATTCGAAGTGGGAACAGGCGCACGCCTTGTCCGCTGCCGATGATGATGTTGACCTCGGCGTGGTCAGACTTTGGCGGGTCTCCGTCGTCGGTGGCCGACACAACGAGACGGTAGTGGCTCTGGTGGATGCTGTCCAGTGCCTTGGCAAGTCTGATTTCACCCGTGTTTGGCTCCACCGAGAACGTGTCCACCTCGTTTGGATTGTCCAGCGTGTAGAGGAAGTTGCCAGGTCCGGCGTCCAGTGTGTAGACAACGTCTCCGACGCGCGTGTTTTCGGCCACCTCGAGAGTGTAGACGGACTTGGGGAACTCGGGCACCACGTCGTTGCGATCGGTTACTGTGACCCACAGCTTGGCCGTGGCGCTCATGCTGGGCTTGCCCTGGTCGTACGCCACCACGGTCATGTTGTAGAACTCCTGCTTCTCGAAGTCCAGCTTGCTC

>AAFF14128

CGGACACTGGCGAGATCAGAGTGGCATCCAGGCTCGACCGCGAAGCTGCAGCCGTCGTCATGCTAACCACCGTGGTGACTGACGTCAGCGCTGTGCCACTCCAGACTGGATTAGGCACCCTTGTGATCACTATAGTGGACCTCAACGATTTTCCGCCTTCGTTCCCGCCTCCTTGGAGTCCCGACCATCCGGAGCTGTCCATCAGTGTTATGGAAGAGCAGCCCGTCGGCAGCGTCGTCGCGTCGTTCGTTGCCACGGACCCGGACTCAAACATTGCCAGTTATTCTATCGAACCCGAAAATCCTTACTTCGCCATTGACAAACTTTCCGGTGTGGTCACAATACACAAAAGAGTCGATTATGAGCAAGTGCAAGAATTGCGGTTCTCTGTCGTTGTGAGAGATACGGGCATTCCTCAGTTGTCAGCGGTCGCAATGGTGACGGCAACTGTGACCAACATAAACGACAACGACCCGACGTTTTCGCAGAAAGCTTACCAGGCTTCAGTCCAGGAGAACGCCCCTCAAGGCACGTTCGTGACGAAAGTTGAGGCGAAAGACATCGATGCCGGAGAATTCGGCGTCGTTTCGTACTCCTTGCTGGGAGAGAAAAGCGGAGACTTTCAAGTCAACAAAAGGGGCGAGATAAGTGTCGCGGGAATGGCCAATTTAGACAGAGAGATCACTCCTGCAATCACCCTTCAAGTGGTAGCCACTGACATGGGCCACGATGCAACGACGAGGAGGTCGGTCTCCGTTCCGGTGTACCTTACCCTGCTCGACGACAATGACAGTCCTCCCGTGTTCACGAAAAAGACTTACGAGGCTTCGTTCGTCGCCAACAGCCCGCTCGAGTCTGCGCAGAGCATCGTTCAGGTTTCTGCAACGGATGCGGATGAAGGGATCAACGCCGAAGTCCGCTACTCCATCGTCGCTGGAAACGAAAACGGAGTTCTCGCCGTCAACCCCAAGACTGGAATCGTGTACCCCGTGAAGAAGCTGGAGAGCAGTCGCAAGGAGTACCGCATCGGGTTGGAGGCACGCGACGGCGCCGGCACTGGACCGAACACGGACAACTGCGTGGTGCTCATCAGGCTGATCGAAATAAATCTGGACAAGCCACATTTCGTGACACCCTCCCTACCCAACGCCACCGTGGAAGTGCTCGAGAACCAGACCCTCTCGAGCCAAATCATAATGACTGTCGAGGCCATGGACAAGGACCACGGAGACAACGGGCGCATCTCTTACTACTTCAAAGTTGGAGACAGGAACGTCGCAGAAACGGACGAGTTTCGCATCGACGAAGTTACCGGAGAAATCCATGCCAAGGCCGTCCTCGACAGGGAAGTCAG

>AAFF40409

GATAGAGCCCTTGCAGGCCACTTGGTTCCCTCACGACCACCTCACCGGTGTCCGGACGCACCTCAAAATGCCCCTCGTGGTCCCCAGCCATCAAGGTGTACCGTAAGCTGGGCCCCTCCAGGTCCGGATCATGGGCAGCTACTTGCACCACGCGCACGCCGGCGTACGTTGGCAGCAGCAGGCTGGCATTGTACTGCTCATGCTCGAAGCGGGGTGGGCTGTCGTTGACATCCGACACACGAATGCTGACCATGGCGGGCTGCTGAGCTGACAGTCGTGGCTGCCCGCAGTCCGACACCTCCACAGTGAAGTTGTACTCGGCCTGCAACTCCCGGTCGAGGGGCTGCACCAGGCTCAGG

>AAFF41411

AAACAGTCCTGGAAGGTTTACCACCAGGCACCAAAGTGACTCAGGTCCAGGCTGTTGACAAAGACGGCACTTACCCGAATAACAAGGTTTATTATGCCATAGAATCTCGAGACCATGGTGACAAGTACTTCTCCATTGATCGTGAGACTGGAGACATCTACACCAAGGTTGAGTTTGACAGAGAAGAGAAGATGGCCTACGCAATCCTGGTTCGGGCAGAGGATGGTGCTCCATCGGCACGGCCCCACATGACAGA

>AAFF42137

TCAAAGTTAGGGTTAAAGACATAAATGACAACAAACCTGAATTTGAGAGACCCAACATTGAAGTGTCTGTTCCTGAGAATTCCACCGTTGGTTCAAGTTTGGCAACATTTAAGGCCACTGATGCCGACCAAGGTGGAAAGTCACGTGTCAGCTACATGATTGACCGATCATCTGATAAAAAGAGGCAGTTCAAGATTAATCCCAATGGTGTTGTTGAAATTCAGCGTACACTGGACAGAGAAGACATCCCAAGGCACCAAGTCAAGATCTTGGCTATTGACGATGGTGTACCATCACGGACAGCTACTGCAACACTTACTGTTGTTGTTAGTGACATCAATGACAACCCACCAAGGTTCCAGTACGACTACAGGCCAGTCATACCG

>AAFF10755

CCATTCTTGTACCACGAGTAGCAGTAGTCGCGCCGGGAGTCGACACAAGATCTGCCACCCGGCCCAAGGCTATAGCCGTCGTCGCAAAGGCAGCGGAAACTGCCCACCGTGTTTTCGCACCGTCCATTGCCACACACGTTGTGAAGCTCGCGACACTCGTCAATATCGACGCAGTGATGGTGGTCAGTAGACTCCTTGAATCCCGCGTTACAGCGGCATTTGACGCCACCTTCCGTGTTCACGCATATGCCGTTGCGGCACATGTCTGTAGTCTCGCACTCGTTGATGTCTACACAGAACTGGCCGTCCTGCGACCAGACGTAGCCGTTTTTGCAGGTGCATCGGTAACTCCCAGGTGTGTTCTCGCATACGCCACCTCGTAGGCATATCTGGCTGTTCTCGGCGCATTCGTTGATGTCGATGCAGGCGTGGCCACTGGGTGACAGCGTGAATCCAGGGCTGCACACACACTTGTACGAGCCTGCCATGTTGACGCAGCGGCCGTTCCCGCACATGCCACTCTCCGCGCACTCGTCACGATCGCTGCAGTCCGTGCCATCAGGAGTGATGTCGAATCCGACCGGNN

>AAFF22142

TTCTTATAAAAATCTCAATACTTGTTACCAAACGTCCTCAAACATCAATATGAGCATTGCTGCTTCCCTAATAAAAACAATAAAAATCAGGAATCGGTGTTCACACGCCAACTTAATGTATGTTTGAAGATAGAAACCACTAATAGGGCTAGCTACCAATCGGCAATCTGCACGATCCCACAATATGCCAGTGAAACAACACAAGAAAATCAAATATTGTACAAATGAGTCATGAAGAAAGATTGAAAAGAGCTATGCACACACGCAGTCAACGCTGCATTCAAGATCGGTTCATGCGGGAACAACAAATTAGAAATCCAGAAATTGTGAATGCACAACTGCAATGCAGTCCATGCACACCGTTTACAGTGTGCACACGGAACGCACAGCAAACACTCATTTCCCAAAGTACAATACAATACGGATCAAATAATATTAAGCGCAGGCTCTGA

>AAFF29141

TGCTTTCAGCCCGCCCCACTGCGGCGAAACGAAGTCATCGTTCGGCGAGTCTTGCACCCTGCACTGCGCCGACGGCTTCCGTGTGTCTGGACCCGAGACACGCCATTGCCTCAGCACGGGAACCTGGAGTGAGCCCGACAACTCCGCTGCCTGCGTTGACGCCGTGCCTCCCACCATCATGAACTGTCCCGGAGACATCGAGGTCGGCTCGGAGCCGGGC

>AAFF35264

NNTTTTTCTTTCTTTTGAACCTAACTATGGGCTCTGCAGAAGTATTTAGCGCTTGTGGACGAATTCTGCACTGCAGTAAATGTGTACGACAACACGCTGTATGTTGGGCAGCGTGATTGCTCTGGCCAGTACATAACAGCAATGGTGCAGATGGAATTATTAAACTTTTCACTTGTCGAAGACACGTGTCGGTGTGTTTTTGCGGAAAGCTTCGCGTGCATGTTTTTTTTTCTTAAAACGGCACGAAGACAACACCGATGTCAGGGTACATCTGTATATGCCATCTTTGTATCAAGGCTTATATATGTTGGTATGTACAG

>AAFF42553

TCAAGGCCACGGATCCGGACAGCACGCCCAGCATAACGTACACCATCAGGGAGGGCGACCAGTCGCTGTTCGCCGTGGACCCCGTGACGGGCGTCGTGAGGACAATCGCGGGCCTCGACTACGAGAAGAAGACCAGCCACACGCTCGTCATCGGCACGCTGGAGAACGAAGCCAACGACGCCATGGCCACGTGCACCGTGCGAGTCGCCGTCGAGGACCGGAACGATGTTGCCCCACGCTTCACGAGCGTCCCGTTGCCGATCAGGCTGCAGGACACTGTGCCGCTGGGAACAATTGTGACAACAGTGGTGGCGTCCGACATGGACGGATCGGCACCTGGGAACGTCGTGCGTTACGAAATCAGCGGCCAAGGTCGGGCCCCATTCTACTTTCTCATCGACAGCACCAGTGGCGTGATAACTGTCAAGGACGACCTCAGGAAAGAGCCCGACTCCGAGTACAGGATCGAAGTTCA

>AAFF42689

AAGGATCCAGTCTTGTCCCTTGCTATGATAGGCTCCTCAATGTCGTACCGCACCTTTGCCCCAACATCAGGATCAACAGCAGTTACGTTCAATACCTCACGTCCAACTGGTAGGCTCTCAGTGACCCAGTGTACATAACTGTCCTTTGCAAAAACAGGTGGCTTGTTGTTGACATCTTGAACAGAGACTGTCACTGTGGTAAAGGCCTTCTGTGGTACTACACCCCCATCCACAGCATACACCAAAATGTGGTACGA

>AAFF43584

CTGATACAGGTGACTGACAAAAACGATCTCAGCCCACTGTTTTTCCCCACCGTGTACAATGCCACGGTGCCTGAAGACACACCTCTGCATCATGTCCTGGTAAAGTTGAATGCCTATGATCCAGACCTTGGTGTCAATGGGGAGATCTACTACCGTCTGCTGGAACCTTCACGGCAGTTTGCTGTCCATCCGACGATGGGAACAATCTTGTTGACACGGCCGCTTGACTTCCAAAGAAAACCAATTCACCAGCTAACTGTGGTGGCTGAA

>AAFF44301

TCATCAATGGCCCCATCCACCTGGACAAGGACAAGTACGAGTTGAATGTGACAGCACGAGATGATGGTGCCTGCTGTCGAAATGGGGCTCTGACTCCCCACACAAGCACAGCCCTAGTGGTTGTGTTCATAACGGATGTCAATGACAACAAGCCCGTATTTGAGGAATGCCAGACCTATACGCCTAAGGTAGAAGAGGGTGCTCAAAGTGGCACCTCAGTCATCAAGGTGAAAGCAAGAGATCTTGACAAAGGACACAATGGTCAAGTGCGCTACTCCATCGTCCAGCAGCCTAATCAAAAGGGCACAAAGTTCAGTGTCGATGAACTCACCGGAGAAATCAGGACAAACAAGGTTTTTGACCGTGAAGGGGAT

>AAFF45647

TTGACTTTGACAGTGACCACACCGTTAGATGAGCCCAGTTTTTCCTTGTCAAGCAGGTTCGCAGTCATTATAACTCCTGTGTTTTTGTTTACCGCAAAGAAATTTTCGGGATTTGAAACGAGAGAGTAGCGGATGCCCCTAGGAACTCCTCGGTCGCCGTCGATTGCTTTTACTGTCATCACGTACGTCCCTACGGGGACATCTTCACTGACAATGGCCGACATCGAGCCAAGAAACACCGGGGGTCGATTCTGAACGTCCGTCACTTTCACAACGGCTGTCGTGGTGGCGTTGAAGGAAC

>AAFF46759

CGTGGTGGCGTTGAAGGAACCATCATCCGCTACGAGATTCACTTCGTACACATGACGGACAGCGTGGTCCAACGGCTTCCGCAGTACCAAAGATGCGTTCAGCTCCTGGGGACTAGACATTGTGACCACCACAGAGAACGTGGAGCAGGCTTCCGGGACATTGTTGTTCGGGAAGCACTGAACCTGGAGCACGTTCCCTGCAGAATCTAAGTCGGTCACTCTGATGTTGTTCAGGACCGTGTGTCCCACTGGCGCATCCTCTGATATCTTCGCCTCGTACGGAACATTTTGAAACCTTGGGATGTTATCGTTGACGTCCAGTACAATGACGCTGACTGGAACCTTGACGATGTTATTTTGATCAAGTCCTCCAACAAAGTCTTCAAGGGTCACTGAGAATTTTAGTGTGTCTGTTACCTCCCGATCAATGTTGTTTATCACAGTGACGTCGCCGGTGCTCCGATCTACAGCAAGGAGGTCCGTTCCTTCAAGTCCGTAGTAAACTGGCGAATTTTCAGGATCCGATCCTTCCAGGCGAAATACAGACGTACCGACGGGAGTGTTTTCAGGTATCACAGCAAGGTCAATGGTTTTGGTAAACTTTGGCGGCAAATTGGCACTGGTTAGATGGACGGCCGCAGAGAGGAGCAAAAGAGCGA

>AAFF48335

AGCTCTTCAGGTGGCTGCACCACAGCCACCGTAACAGGCACCACAGCCCAGAGAGGTCTAGGATTACCCTGGTCACTGACGTGCACGAAGAACTGGAACACGCCAGCTCCTCTGAGGGGCGCCTTCAGGTAAAGCTCTCCCGAGTCATGAGTGACATTGAACAATGCACTGACATTGGTTGGGGCTTCATGAAGGCTGTAACGAGGCCATGTTCCTTGGTCACGGTCAAAAGCACGAACCTTGACAAGTGTAGTGCCCACGCTAGTGTTTGTCCAGATGCTAGCCTCATATTCAGAAGCACCAAAAGTGGGCTCATTGTCATTGACGTCAGTGACAGCCACACGAACAAGAGCAAA

>AAFF48696

TTGAGGGTGTCTGCTTCTGATGAAGACGCAGATAACAATGGTGCCATTGTCTACAATTTGACTGCACCATATGACCCTGAGCACTTGGCATATTTCTCCATCAATCCAGACTCCGGGTGGATTAGCCTGCAAAAGGCCCTTGACCGTGACCAGTATCAGCTGCGAGCCATAGCACTGGACAAAGGTGTTCCCCAGCATCAGGCAACGGTGGAAGTGATCATTGATGTTGTT

>AAFF51259

GGCGCTCATCGACGAAGGGGACGTGCGCTTCGACCCCCCGCTGAGGGTCCAGGCGCGGGATCCTGACGTGACGTCGTTCGTGAAGTACAGCATCGTCTCCGGAAACAGCTACAACCTCTTTACCATCAACTCACAGACGGGTGACATTACAGTTACGAGTCGCCAAGGGCTGGATGTTTCCCTTCTCAGAACGGACACGATAACGCTAACCGTACAAGCCTCTGACGGTGGCTCTGGCATCGACACTGCAATAGTTAAGATCACCGTGAGGGACGCCAACAACAACAGTCCCGTTTTCCAGAAAGATCGCTATGTCGCTTCAGTGCCGGAAGCGTCGCCACCAGGAACCCTTGT

>AAFF42899

TCGGCACGGCCCCACATGACAGACAACCGGCCCAACTCAGTGACCAAGTACATCCGCATTGGGATCGGCGACAAGAACGACAACCCGCCGTACTTCGGCCAGGCGCTCTACGAGGCTGAGGTCAACGAGGACGAGGATGTGCAGCACACTGTCATCACTGTCACCGCCAAGGACAAGGACGAATCCTCGAGGATACGCTACGAGATCACCCAGGGGAACATAGGAGGAGCGTTTGCAGTGAAAAACGAGACTGGCGCCATCTACGTTGCCGGCCCGCTCGACTACGAGACCAGGAAAGAGTTTAACTTGACGCTGGTGGCATCGGATGGCTTTCATGAAGGCAACACCACAGTTCTTATCCACGTCAAAGATGTCAACGATCTTCCGCCTGTTTTTGGCCAACCTTCATACATGACCACCATCGAGGAGGAGGTGTCCCACAATTTGCCAATGAAAATTATGACGGTGACAGCAACAGATGGTGATAAGGACAGGGAGTCCTTGATAGTTTACTTTTTGACGGGTCAAGGTGTTGATGAAGACCCTGGGAATAGCAAGTTTGCAATCAA

>AAFM12236

CACGACCCTGATGTCTCAGTGCGAAGGGTGACGTACGAATTTTACGAAGAGACTGATCCAGCTGCTGACATCTTCCGGATTGACCCGCAGAGTGGGGTTATTACAACTCTAGTAGCTCTCGATCGAGAAGCCGTGCCATTTTACAACTTTACAGTGAGGGCCCGTGATGACGGTCTGCTGTCTTCCTCAGCTTGGGTGTACGTCGAGGTCCTGGATGTAAATGACAATCCTCCCGTGTTCGAGCAGACCCTATACCAAGTGCAAGTCTCTGAAGATGCTGTTGCTGGCACTGTAGTTGCCAGCCTGCGCGTGGAAGATGCTGACAAGGAGCCGGCTCCTGTTGGCTATTACGTGCTCTCCGGTGATCCTGGTCAGCAGTTTGCGGTGCGCAGTAGTGGTGATGTGTTCGTACAAAGACCGCTGGATAGGGAAGCACGTCAGAACTACACTCTTCGAGTGGTGGCCACAGACGGTCTGCATGTGGCCATGACTCAGCTTGATATAACCATCCTGGACAGCAACGATAATCCACCAATCTGCCTCAAGTCCAAGTACACGGAGCTTGTCTCGGAGAGCATTGCTGTGGGTACTGCCATCTTGACGGTTGGAGCCACGGATGCGGATGATGCCCACCATGCTCAGCTGCACTTCTACCTGTCAGGCCAAGGATCTGAGGACTTTGTTCTGGATGCCAGCAGTGGGGTGCTGCACACGACCCGGCCTCTAGACCGAGAGCAGCGGCCGCACTACTCACTGGTGGCACATGTGCGTGACGTGGCTCGTTGGGAGTGGCAATGCAACAGCAGCGTGGAGCTCTTGCTGAGTGATGTCAATGATAACCCGCCTGTGTTTGGCCAGAGCACCTACGAGGTGGCGCTACCCGAGGACACGCCTNNCCACCGCCTGGTTGCCCAAGTGCATGCCTCTGACCGTGATTTGGGTCCAAATCGACGCCTCAGCTATTCGCTGGTTGAAGCATCAGCCAACAGCCACTTCAGCGTGGATGCACTGTCTGGTTTGGTGCGGTTGGAGCGACCACTAGACCGAGAGGAATGCGCCAGGTTCAACCTGACGGTGCAGGCCCAGGACCATGGTCAGCCTCCCCGGGCTGCCCGCGTGCCTCTTACAGTCATTGTGCAGGACATTAATGACAGCCCGCCCGAGTTCACACAGCAAGCGTACTCTGCCAGCGTCTCTGAAGCTGCCCCCGTGGGCTCGCCCGTCAGCGCTGGTGTGAGGGCTACCAGCCGAGATGCAGGCGTCAATGCTCAGATCACATACTCCCTGGTTGCAGGCAACGACATGGGACACTTCAGCATTGAGCCAAAGACAGGTGTGCTGCGGGTGGCCCGACCCTTGGATTTTGAGAGCGCGCGCAGCTACCAACTGGCAGTGGAGGCTCGCGATGGGGGCGAGCCACCCCTCAGTGCCCGCGCCTGGCTGAATGTGTCCGTACTGGACGCAAATGACAACGCGCCTGTGTTCGGAGGCCCCTACAGTGGCACTGTTG

>AAFM25502

AGGCAACAATCAGGACACATATTTCAGTCCATTTCATCTTGTAGGGGTTTCTTCCAGGTGTACACTTCTCAATGCCTACAATGCTGTAGTTGTGGTACAGTGCCTGAACCTTATGCGATGATGCCATATCCAGCATTTTGGCTACAACGTCGGGGCTCACAACCAGCCCATTCTCAAGCAGCTGAAAGCAAGAGCTGGCACTTTCAAAGTCAAGGCTTCCANNNNNNNNNGTCGAATACTGGGTGTCATAAATGTTGGCAGTCACAGCACTGGCAAGAGCTTGGTTCAAGTCTTTTTCAAACTGGGGGATGACTTTCCTAATTTCTTGCGGCTGTTTGTAGAAGATGAATTTCATGAGGTCTGAATCATGAAGTATGTGAACCCTCACATGTGTAACAGCAGTGTTCTGGGGACCAGGAGCACTCTGGCATTCAACTGTTATGTCAAAATACCCATCTCGGTATTTTCCCAAGCTTCGAACATTTCTCAGGCTGCCTTTGTGAGCGTCTAGCTCGAACTGGCCCATAACAGGCATTGATTCTTTTGATGATGGTCTGTAGTATGTTACATTTCGGATGGAGTACATGATCATGTTTGATGAGCTGTCAGGGTCCCTTGCCTTTAGTGTAATTAGTTCACTGTTGATTTCAGAATTAATTCGTATGCCAAGGACATAGGAAGGCTGCTCAAATGAAGACGAGTTATCATCAATGTCCTCAACCTTTATGTCAACCAGCAGCTGCGTCTTGTCATCGGGATTGTAGGTGGCATCAACCTGCTCCAAACGCTTCGATGGCATGCTGACTTGCACAGTAAGAAGGTAGCGATCCTTTTGCTCCCGGTCAACTCTCCTTTTGGTTAATATCTCGCACTCATTGTTGCCAGTCCGCTGAATAGTGAAAACATTGTCCATGTTGCCACCCACAATGTAGTAATCAATAAAGGCATTCGTTCCAATGTCCGCATCAAAGGCTCTCACTTTGCCAACGATAGTACCAATGGGAATTTCCTCTTTTATCTTCATCACAACAGGCTCAGCATCAG

>AAFM28719

CGTTTGAAGACAGAGCACTATCTGGGTCGACAGCCTGGACTTGGAACACTGAAGCTCCAATGGTGACGTTCTCATGCACGTAAGCTACCTCACCCATTCTGGGCCGAATGAAGCGCGGTATGCCATCATTTGAAGACACGTCCCCGATAATGATGAGTATCTTGACATCTGTGTGGAGTGATGGAGAGCCACCATCTGTGGCTCGCACCGTCAGAAAGTATGGCTCTGACCGCCCCCTCCCAGACAGTGGTTGAA

>AAFM33704

ATGCTCACCAGCAACAGGTCCCGCAAGCGCACGCTGAGAGCTGTTCGCAAAGCTCGCAGCAATGGCCGGCGGTCCGTAGATAAGAAGCGCTCAGTCGTGGTTCCAGCCAAGCGCAGAGCCACAGCTGCCTTGAGAGCCTCCTCGGTCACCCCGACAACATGCACATTCACTGATGCATGTGCTGGCGTTGTGCTGCCATCTGAAGCGCTCACATTAAGCACATAGCTGCCAGCATCAAGGCCTGGCAAAGCACGCAAAGTCCCATCATCCCTGTCGAGGACAAACAGCGATGCGTGTGGCCCAGAGAGGGACAAGCTGAGGCGGTCGTACGGGTCCTCATCCGTGGCATGCACACGGCCTAGCAGCCCACCCGGGAAGTCGTCCAAGTAAGAAGAGACAACCACAGTCAAGGGCTGCACCTGTGGCCTGAAGCGGCTCTTCTCAACAGCCAGCACAGTCACATGCGCCTCTGTCGCCAGAGGTGGGCTGCCCGAGTCGTGAGCCTGCACACGCAGCTCATGCCGCGAGCGCGGAGGAAGTGTTCGTGCTAACCGCAGCTCGTGGCCCTCAACTTGGAAAAAGGGTTCCTCCTCCTCCTGCCCAGCTTGAGCTCCATCCAGCAGCTCCAGCCGGAAAGGAGGTCCATGAGCAGCAGAGTCAGCATCACTGAGCGAGAAGCGCAGCACGGTCCAGCCGACTGGCCGACCCTCATGAACCACGGCCGTGTAGTTGCTCTGGTCGAAGCGCGGTGGATGGTCATTTACGTCCAGCACCTCCAGGTGCACCAG

>AAFM39974

CGATGAGACGCGTCTGTTGCTGTATGATGCTCATTATGTGATCCTTGAGCTGCAGTACTTTGTCCGGCAGGACATCACCGATTGCCAAGACCAGCCGGATTTCGTAGCGGATAAGGTTTACATAAACGTATGCGGATTCCGTGAGGGCCCTGGCGGTCAGGTCGGGGCTGTCTCTGGCTGTGACCTTGAGCTTCAGGGGCAGCTCCACGTTTTGCAGATCCTCGAACGTCTTCACGTTGCTCACGTAGCCGGTCTGGGGGTCAATGCTGAATTTGCCTTCGGGGTCGCTGTCAGGGGATAGGTCGTACACAATCTTGCCATTCGAACCGGAGTCCAGATCTTCAGCCATCACTTGGATGAAGCGGGTTCCGGCCGGGGCATCCGACGACAGTGCTGCCAGGTAGCGGTTCTGCGTCAGCTGGCTGTACATGGGGGGCACTACGAAGCGCGGCCGGTTGTCGTTCTGGTTAATCAAGTGCACAGCCACCTGGGTGGTCAGCCTCGAAGAACCGAACACTCCGCCAATTGTGTTCAAGTGGACGGTCAAGAGATATTTGTTCTGTTCTTCGTAGTCGAGGTTCTGGTTCTGGACACGCACTTCACAGTCGCGCTGTTCGTTGTCCAGTATGTAGAAATGCCCTTTCTCGTTTCCAGTGACGATCTGGCACTGAACGGGGAAATTTCCTCGCGGCTTGTTGATAATCGGCAACGATTTGACGAGGGTGTTCGCCAGCGCGTTCTCTGGAACTTCAACCGTGTAGAATCCGTCGGCGAAACCGAGCCCGGAGTCCGGGGGCACGGTGGCGATGTGCTCCACGTAGACCGTGACGACAGCCGTGGCTGCTAACGAGGGATCGCCGAGGTCGTTCGCTTGAACTTCGATCCTGTACTCGGAGTCGGGCTCTTTCCTGAG

>AAFM42947

CAACTTATGTTATGAGTCTATTTGCAAATGATCTTGCAATGGAAAATCGCATGAATTCTNCAGCTGATGTTATTATCAAAGTGAATGATGTTCAAGACCAGCCACCTGTATTTGTCGCTGACTCATACTCTGTGACTGTTGCTGAAAACAGCCCTGAGGGTACATCAGTTCTCTTGGCCTCAGCCCACGATGCAGATGCTGGCTTCAGGAGGTTGTTGTCATTCAAGTTGGTTAATGACACCAAGGGATACTTCAGGTTGGGAAAAGTGGATGTTGATGACAATGGGATTCACCATGTTGTCGTGGAAACTTCTNNNNNNNNNNNNNNNNGAGAAGATGAAGACATTGAGAAGAGTGGCGGG

>AAFM47326

CGAGTATGCTCGTATCAGCTACTCCGTCTACCACGTGTCCAACAACGGTCGTGACAGGTTCCGCATCGACCCCAACACTGGCGTGGTGCAAGTGATCGGAAGGGTCTCCTCCGGGGAGCAGTACAGCATTACTGTGCAGGCGACGGACTCAGGCGGAAGGTTCAGCCAAGGCATTTTGGATGTGATAGTGATACCGGGTCCGAACACTGGAGGTCCCGTCTTTTCGAAAGAGAAGTATGAAGCTCAAGTCAGCGAAGGAGCTTCTGTCGGCTCTGCTGTCCTAACGCTGAAGGCCACTGATCCAGAAGGGGACGACGTGTCGTACTCAATAGTGGAGGGCAA

>AAFM50368

GTCGAATCCATACATCTTGCCGCCGTCCAGGGAGAAGGTGACGACGGAGCGCACCACGCCGGTCACCGGGTCGATCTGGAACTTTGACGACGCGTCTTCGGGCTTCTGGATGATCTCGTAGCGGATGGCGCCGTTGAAGCCCGTGTCAGCATCGCGGGCGTGTAGCTTGGTGACCTGGTAGCCGAAAGAGGCCTCGAGAGGCACGGCGGCCACGATGGGCTTGCCCTGGTGGTCGAAGACGGGCGAGTTGTCGTTTTCGTCACCCACGTTCACCACGACGAGGGCCTCGTCGAAAGCGACATGCGCCGACTCTTCATCAGACTTTTTGCCCTTCTTGGCCACTGCACTCTCCCGAACAGCTCGGCCCACACGCTTCTTGTTCATGGCGCGCACTTTGACCACGTACTTGGGTATGGCCTCCCTGTCCAGGCTGGAGGTCACCGACAGTCGGCCAGTGTCGGGCTCGATAGCAAAGAGTCCGTTGTAGTTGTTGCCAACGATTGCGTACTGAACGGGTCTGTGGGCGATTTCTTCCGTTGCATTCAGATCGAGAATAACCTGAG

>AAFM51945

CCATTCCACGTGGATGTGCACACAGGAGAACTCTTCGCCACTGGCATCCTAGACCGGGAAACGAAGTTCAAGTACTACTTCCACATTCTTGCTTTGGATAACGGAGAGCCTCCTCAAAACTCGACAGTCAATATAACTGTCACCGTTGAAGACGCGAATNNCGAGAAACCACGGTTCTTTACTGATCCGTACTTGGCTCAGATCCCCGAGAACCTGGACCCTGGNCAAAAGGTCACCCAGATTCGCGCTTTTGATCCTGACACCGGTGAAAAC

>AAFM59627

GTCTTGGTCTGCCTTGTCCAGGTGGTCCCGAATGAAGTCACCCACATCTGGGTGGGCCCCTGTGGGTGGTTCCCGGACACGAACTTCTTTCTGCAAAGGTGGGGCTTTTTCTGGGCCAGCCTTTGCGCCAATCTGCATGCCAGATGCATCAACTGGTATTCGTAGTGGTGTTATGTCATAGGCATTCATGTCATCTTCTCCGCCGCCTTCATCATCATAGCTGATGATGTTCTCTCGCACATCGTCGTCCACTCCACCATGGCCAAACTTCTGGTCTGACCGTCGGCTCCGGGTGTATGCCACAATCACCAGGACCAAAANNNNNNNNNNNNNNAGACAGACCAGGATGGCAGCCAAGGCAGNNNNNNNNNNNNNNNNNNNNNNNNNCTGTCGCAAGGCGCCACAAAGGTCGCCCCCAAAACCATCTGGGCATAGGCAGTAGAAGCCCTCGCCGTGTGGCCGGTTCATGCAGGTGCCTCCGTTGAGG

>AAFM57968

TCTCGGGGACCAACGGGCTCGTCGAGTACACCACGGCTCCCGGGGACGGCGGCAACAACGACGGGTATGAATACTTCGCTATTGACCTGCCATATCAAGGCCTAGTGACGATAGCGAGGCCCCTGGACTACGAAAGGGCCAAGTACTACCACCTGCTCATCAAGGCAACCGACACGGCGTCGAACCCGTCGGAGCGGCTATCCAGCACGACCACGTTGACGGTGTTCGTCGAGGACGGGGACGACCAGGACCCGGCGTTCGTGCACGAAGGCTGCACTGTGGTGCACGGGGCGTGCGCCGACGTTGAGTACAGCGCCGAAGTCACCAGCGGCCTCATCTCGGGAATCCTCAA

>AAFM60604

CAGGACGTCAACGACAGTCCTCCGCAGTTTGAGAAGGAGGCGTATTACGAGTTCGTTTCGGAGAGTGCATCTGTGGGCACCGTTATCGATACCGTCAAGGCTACTGATCCTGACTCGCCAATGAACACCAAAATGACCTACTCTTTCGCCCGGGGCACCAGCAAGAGAGTGCCATTCATGATCGACCCTGTCCTGGGAACGGTGAACATCACCAGGGCACTCGACATATCGG
